# High-dimensional profiling uncovers heterogeneity, lineage-specific precursors and inflammation-induced changes in the mononuclear phagocyte compartment of the human intestine

**DOI:** 10.1126/sciimmunol.adz8650

**Published:** 2025-12-12

**Authors:** Thomas M. Fenton, Line Wulff, Venla Väänänen, Gareth-Rhys Jones, Camilla Koldbæk Lemvigh, Lene B. Riis, Mads Damsgaard Wewer, Julien Vandamme, Peter B. Jørgensen, Calum C. Bain, Julie Lee, Kirstine G. Belling, Gwo-Tzer Ho, Tune H. Pers, Anja Poulsen, Gorm R. Madsen, Ole H. Nielsen, Henrik L. Jakobsen, Jose MG. Izarzugaza, Flemming Bendtsen, Søren Brunak, Allan M. Mowat, Lars R. Olsen, Urs Mörbe, William W. Agace

**Affiliations:** 1 Mucosal Immunology group, Department of Health Technology, Technical University of Denmark, Kemitorvet, 2800 Kgs. Lyngby, Denmark; 2 School of Infection and Immunity, College of Medicine, Veterinary Medicine and Life Sciences, University of Glasgow, Glasgow, UK; 3 LEO Foundation Skin Immunology Research Center, Department of Immunology & Microbiology, University of Copenhagen, Copenhagen, Denmark; 4 Centre for Inflammation Research, Institute for Regeneration and Repair, University of Edinburgh, 4-5 Little France Drive, Edinburgh, UK; 5 Department of Pathology, Herlev Hospital, Copenhagen University Hospital, Herlev, Denmark; 6 Gastro Unit, Medical Section, Copenhagen University Hospital - Amager and Hvidovre, Hvidovre, Denmark; 7 Copenhagen Center for Inflammatory Bowel Disease in Children, Adolescents and Adults, Copenhagen University Hospital - Amager and Hvidovre, Hvidovre, Denmark; 8 Novo Nordisk Foundation Centre for Stem Cell Biology, DanStem, University of Copenhagen, Copenhagen N, Denmark; 9 Novo Nordisk Foundation Centre for Basic Metabolic Research, University of Copenhagen, Copenhagen N, Denmark; 10 Translational Disease Systems Biology, Novo Nordisk Foundation Centre for Protein Research, Faculty of Health and Medical Sciences, University of Copenhagen, Copenhagen, Denmark; 11 Department of Digestive Diseases, Transplantation and General Surgery, Section for IBD, University Hospital of Copenhagen, Rigshospitalet, Copenhagen, Denmark; 12 Department of Clinical Medicine, Faculty of Health and Medical Sciences, University of Copenhagen, Copenhagen, Denmark; 13 Department of Gastroenterology, Medical Section, Herlev Hospital, University of Copenhagen, Herlev, Denmark; 14 Department of Surgery, Herlev Hospital, Copenhagen University Hospital, Herlev, Denmark; 15 Center for Biological Sequence Analysis, Technical University of Denmark, 2800 Lyngby, Denmark; 16 Immunology Section, Lund University, Lund, Sweden

## Abstract

Understanding mononuclear phagocyte (MNP) diversity in the human intestinal lamina propria (LP) has proved difficult due to the expression of overlapping phenotypic markers and the inability to isolate these cells without contamination from gut-associated lymphoid tissues (GALT). Here, we exploit our novel method for isolation of human GALT-free LP in combination with single-cell (sc)RNA-seq, CITE-seq, flow cytometry and imaging to explore MNP heterogeneity in the human ileal and colonic LP in health and Crohn’s disease (CD). As well as monocytes, macrophage subsets, cDC1s and cDC2s, we find a CD1c^+^ cDC subset with transcriptional features of cDC3. Using computational tools, we identify monocyte–to–macrophage trajectories as well as putative subset-specific cDC precursors, including a population of *RORC*^+^*PRDM16*^+^ cells that appear to give rise to cDC2. We further show that LP *CCR7*^+^ cDC are increased in CD and provide evidence that these cells arise from cDC2/cDC3 but not cDC1. Collectively, these findings extend our current understanding of intestinal MNP diversity and development, highlighting both tissue-specific and inflammation-induced changes in MNP composition and function.

## Introduction

The mononuclear phagocyte (MNP) family consists of conventional dendritic cells (cDC), classical monocytes, non-classical monocytes, and macrophages, each of which play specific roles in immune responses, tissue homeostasis and inflammation(1–3). Whereas cDC are the principal cells involved in the induction and shaping of adaptive immune responses(4), tissue-resident macrophages are primarily involved in maintaining local tissue homeostasis, defense against infection, and tissue repair(5, 6). Recent studies have highlighted considerable heterogeneity amongst MNP and it is now evident that these cells develop unique functions depending on the niches in which they reside(7–11). Despite this, much remains to be understood regarding MNP diversity and function in human tissues in health and disease.

The intestine is continually exposed to food and microbial products that are essential for our health(12, 13). The intestinal immune system must respond appropriately to these products to maintain tissue homeostasis while retaining the ability to mount effective immunity to intestinal pathogens. Given this continual exposure to foreign material, it is unsurprising that the intestine contains the largest and most diverse immune compartments in the body. MNP are found in all anatomical layers of the human intestine, including the intestinal lamina propria (LP), the muscularis mucosa and the organized gut-associated lymphoid tissues (GALT); comprising the multi-follicular Peyer’s Patches (PP) of the ileum and the mucosal- and submucosal- isolated lymphoid follicles (ILFs) that are distributed along the length of the intestine(14). Much of our understanding of the roles of intestinal MNP diversity and function however comes from studies in mice. These studies have not only highlighted the different roles that MNP subsets play in intestinal homeostasis but also show that MNP composition and function is highly dependent on anatomical location in which they reside(1, 2, 11, 15, 16). Consistent with this, recent single-cell transcriptomic analysis suggests considerable heterogeneity between the macrophage compartment of the human colonic mucosa and muscularis mucosa(17–19). While these findings highlight the importance of assessing MNP diversity in different intestinal niches, this has not been possible in the human LP due to a lack of protocols to isolate LP tissue free from contaminating submucosa and GALT.

Further complications arise from the fact that the human gastrointestinal tract is not just a homogenous tube but consists of several anatomically and functionally distinct segments. For example, the small intestine, whose surface is characterized by finger-like projections termed villi, is the major site of food digestion and absorption. Conversely, the colonic surface consists of a flattened mucosa and it is home to the largest number and variety of microbes(20, 21). As a result, the concentration of dietary and microbial products and metabolites, many of which have direct impacts on local immune cell development and function, varies greatly along the length of the intestine. How variations in intestinal anatomy, function, luminal contents as well as inflammation impact local MNP diversity in humans thus remains incompletely understood.

Here we used our recently developed techniques to isolate intestinal LP free from contaminating GALT and submucosa (SM)(22, 23) to assess the phenotypic, transcriptional and developmental diversity of MNP in the human ileal and colonic LP. Our results provide novel insights into intestinal LP MNP diversity and reveal site-specific alterations within this compartment in inflammatory bowel disease (IBD), providing a framework for designing targeted approaches for modulating intestinal immune responses.

## Results

### MNP populations of the human ileal and colonic LP are highly heterogeneous

**Fig. 1:**
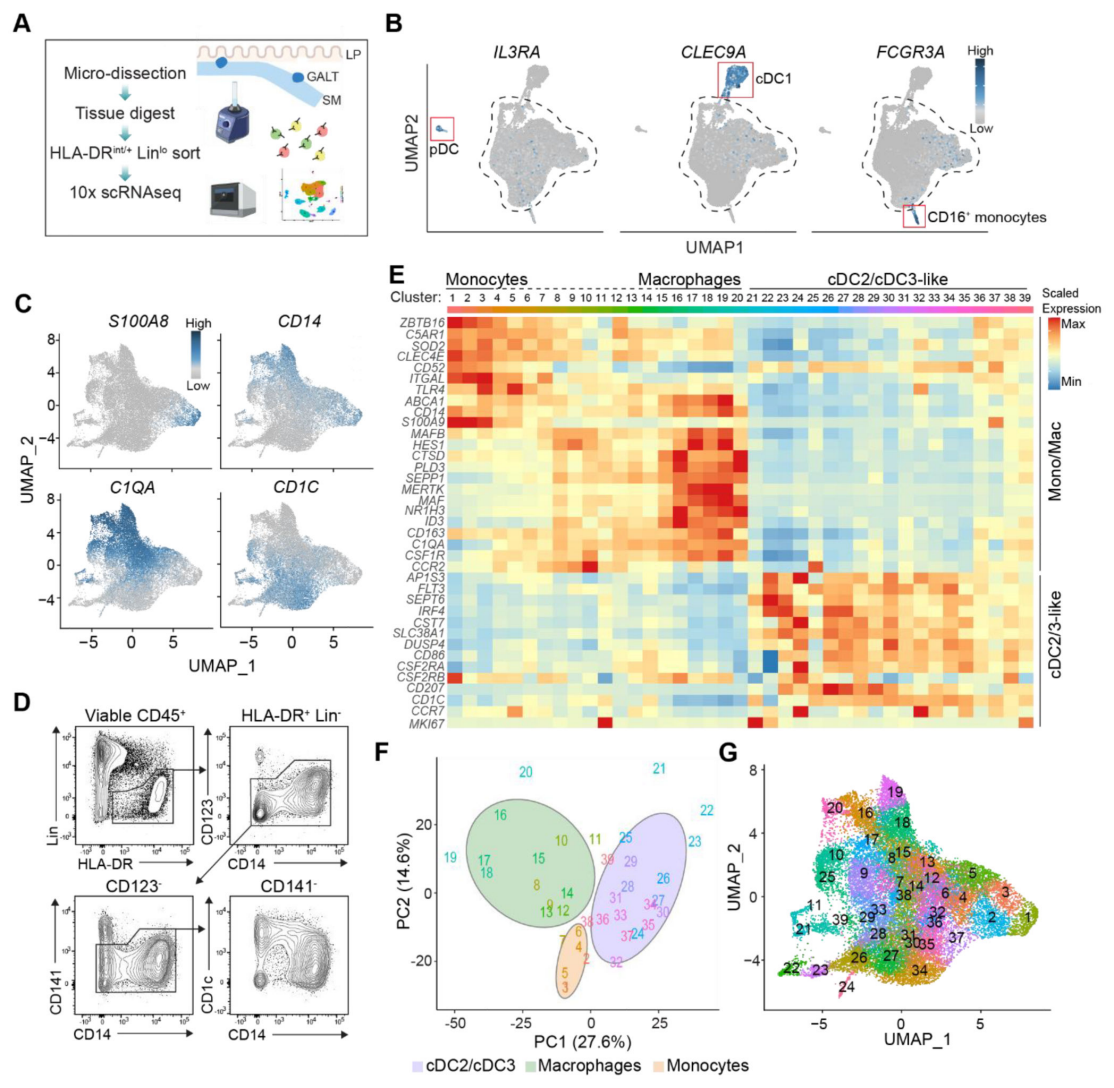
High-resolution clustering to disentangle MNP subsets of the human ileal and colonic LP. **A**) Experimental pipeline for the generation of single-cell transcriptional data of intestinal LP MNP. (**B**) UMAP of pooled ileal (n=4) and colonic LP (n=6) MNPs (28,758 cells), showing normalized gene expression of signature genes for pDC1 (*IL3RA*), cDC1 (*CLEC9A*) and non-classical monocytes (*FCGR3A*) signature gene. Dashed line encompasses MNP not identified as pDC, cDC1, or non-classical monocytes. (**C**) UMAP of *S100A8, CD14, C1QA* and *CD1C* expression by intestinal LP MNP after exclusion of pDC, cDC1 and non-classical monocytes. (**D**) Flow cytometry analysis showing CD1c and CD14 expression on colonic LP CD141^-^CD123^-^ MNP, representative staining of 10 resections analysed. (**E**) Curated pseudo-bulk heatmap (using averaged gene expression per cluster) of clusters within the dashed line of the UMAP in (**B**), showing expression of known monocyte, macrophage and cDC2/3 associated genes for clusters 1-39. (**F**) Pseudo-bulk principal component analysis of clusters from **E** using DEG gene lists for cDC2/cDC3 monocytes and macrophages from Mulder *et al* (35). (**G**) UMAP depicting the location of clusters 1-39 within the cDC2/cDC3/mono/mac supercluster.

To assess MNP diversity within the intestinal LP, healthy mucosa from ileum and colon was obtained from surgical resections from patients with colorectal cancer (CRC) taken >10 cm from the tumour margin. They were then processed to remove contaminating GALT and submucosa (SM), as described previously(22, 23). Following LP digestion, single-cell RNA sequencing (scRNA-seq) was performed on CD45^+^CD3^-^CD19^-^HLADR^int/+^ cells from LP cell suspensions, purified by fluorescence-activated cell sorting, using the 10x Chromium technology (Fig. 1A). Transcriptomes were obtained from six colonic LP and four paired ileal LP samples (Table S1). Distinct clusters of *CD3E*^+^ T cells, *CD79A*^+^ B cells, *VWF*^+^ endothelial cells, *MS4A2*^+^ mast cells, *COL3A1*^+^ stromal cells, and *NRXN1*^+^ glia were identified and excluded from further analysis (Fig. S1A). MHCII genes were expressed by one ‘supercluster’ and two peripheral clusters (Fig. S1B), which were computationally isolated and re-clustered to dissect their transcriptional identity. These 28,758 MHCII^+^ cells comprised distinct clusters of *IL3RA*^+^ plasmacytoid DC (pDC), *CLEC9A*^+^ cDC1, and *FCGR3A*^*+*^ non-classical monocytes, as well as a central supercluster (Fig. 1B). Cells within distinct regions of this supercluster expressed either the monocyte associated marker *S100A8*, the macrophage associated marker *C1Q*, or the cDC2 associated marker *CD1C* (Fig. 1C). As expected, the myeloid marker *CD14* was expressed by the *S100A8* and *C1Q* expressing cells (Fig. 1C), but it was also present on some *CD1C* expressing cells (Fig. 1C). Consistent with this, flow cytometric analysis of colon LP CD45^+^ HLA-DR^+^ cells showed the presence of cells expressing CD1c or CD14 alone, as well as cells expressing variable levels of both markers (Fig. 1D). To further determine the identity of cells within this supercluster, we first re-clustered them at high resolution into 39 subclusters. All clusters were present in the four ileal and six colonic LP samples, albeit in slightly different proportions (Fig S1C). Manual annotation based analysis of known monocyte-, macrophage-, and cDC2-associated genes(24–28) was used to assign each of the 39 clusters as either monocyte/macrophages (mono/mac) or cDC2/cDC3 (Fig. 1E). Cluster numbers were assigned to each of the mono/mac clusters based on manual annotation of transcriptionally related clusters, comprising monocytes (clusters 1-3), monocyte-macrophage intermediates (clusters 4-14) and differentiated macrophages (clusters 15-20) (Fig. 1E). Clusters 1-3 expressed the classical monocyte transcription factor *ZBTB16*(29), clusters 4-14 expressed intermediate levels of signature genes associated with both monocytes and macrophages and clusters 15-20 expressed high levels of genes associated with mature macrophages, such as *SEPP1, MERTK* and *MAF*, together with TFs involved in tissue-resident macrophage development such as *ID3*(30) (Fig. 1E). A second large group of clusters (clusters 21-39) could be identified based on their expression of *CD1C*. Of these, clusters 21-35 expressed low levels of monocyte and macrophage associated genes and high levels of the cDC2-associated genes *AP1S3, FLT3, SEPT6* and *IRF4*(31, 32) (Fig. 1E), while clusters 36-39 expressed both cDC2-associated genes, including *FLT3, IRF4* and *CD1C*, as well as monocyte and macrophage-associated genes, including *CD14, CD163, MERTK, S100A9* and *C5AR1* (CD88), albeit at lower levels than observed in mono/mac clusters (Fig. 1E). As cDC3 have been reported to co-express cDC2 and monocyte markers (33, 34), we putatively designated clusters 21-39 as cDC2/cDC3.

Pseudo-bulk PCA analysis on each subcluster using transcriptional input from a pan-tissue dataset of human cDC2, cDC3, monocytes and macrophages (35) confirmed our designation of the 39 subclusters as either mono/macs or cDC2/cDC3 (Fig. 1F). Consistent with this, the mono/mac and cDC2/cDC3 subclusters mapped to distinct sides of the supercluster UMAP, except for the proliferating mono/mac subcluster 11 which clustered together with the proliferating cDC2/cDC3 subclusters 21 and 39 (Fig. 1E and G).

### LP macrophage subsets arise from distinct monocyte derived intermediates

**Fig. 2:**
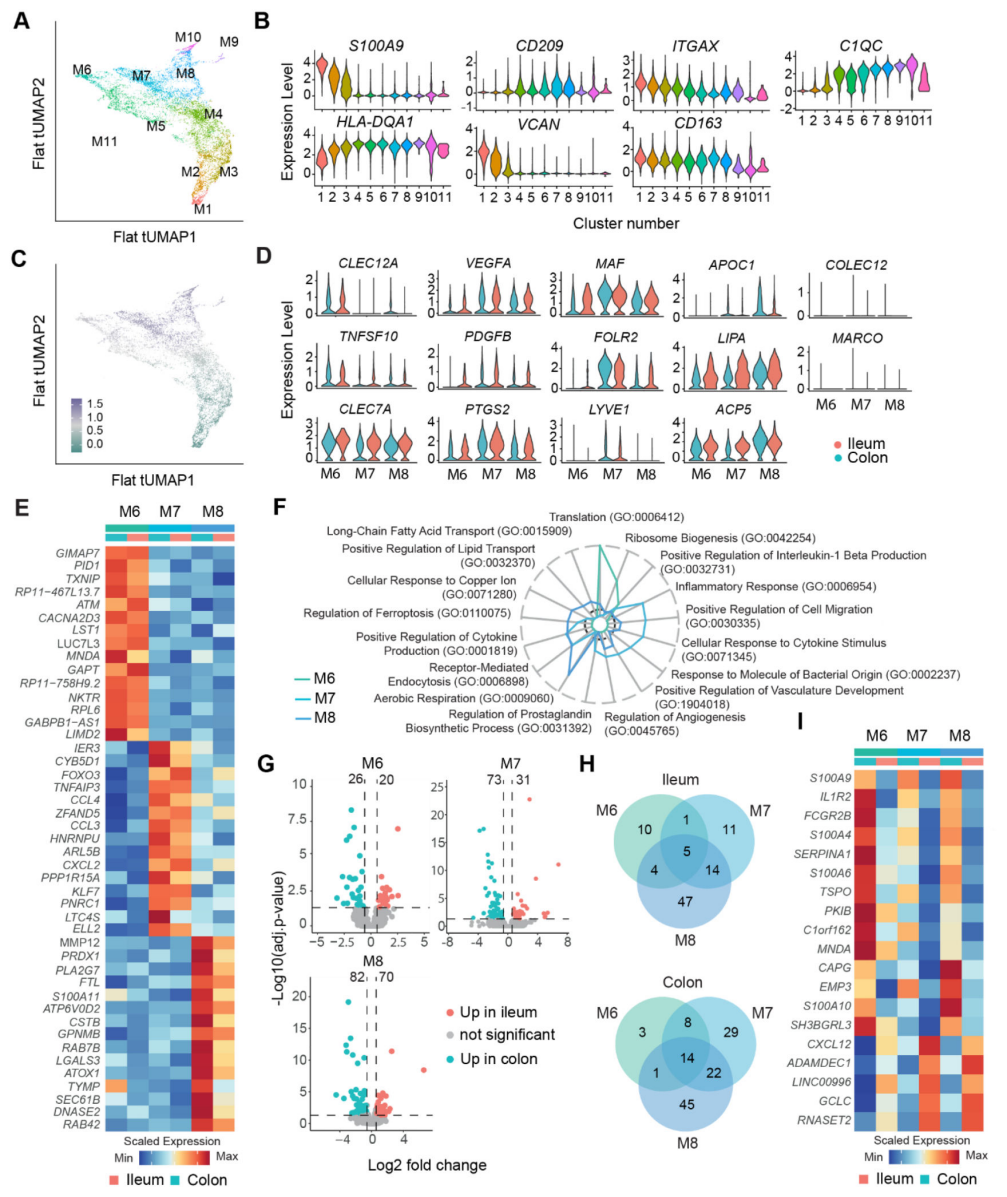
Characterization of intestinal LP macrophage populations. **A**) Two-dimensional representation of a 3-dimensional tSpace UMAP (Flat tUMAP) with Louvain clustering of ileal and colonic LP MNP identified as belonging to the monocyte-macrophage lineage. (**B**) Violin plots of normalized gene expression of indicated maturation-associated genes in M1-M11 clusters. (**C**) Pseudotime of cells calculated by averaging all tSpace trajectories starting from M1. (**D)** Violin plots of indicated genes for colonic and ileal LP M6-M8 clusters. (**E**) Pseudo-bulk heatmap of scaled gene expression of top 15 DEG (ordered by avg. logFC) between macrophage clusters M6-M8. (**F**) Radar plot displaying GO pathways enriched in cluster M7 and M8 macrophages. Y-axis = sqrt(-log(adjusted P-value)). Dashed line indicates significance threshold of adjusted P-value = 0.05. (**G**) Volcano plots demonstrating DEGs between ileal and colonic LP in macrophage clusters M6-M8. Dashed lines indicating significance cut-offs. Adjusted P-values < 0.05 and |avg. logFC| > 0.2. (**H**) Venn diagrams showing overlap of ileum- and colon-specific DEGs between differentiated macrophage subsets M6-M8. (**I**) Pseudo-bulk heatmap of scaled gene expression of common genes upregulated in M6-M8 clusters in the ileum or colon.

Studies of tissue macrophages have highlighted significant niche-specific phenotypic, functional and ontogenic diversity(8, 11, 30, 36, 37). While human ileal and colonic macrophage data feature in various cell atlases(18, 38, 39), direct comparison of these cells at high resolution is lacking. Furthermore, while most murine intestinal LP macrophages derive from monocytes via transitional intermediates(40–43), whether similar transitional states are present in the human intestinal LP remains to be determined. To address this directly, the ileal and colonic LP MNP clusters we had identified as mono/macs (Fig. 1E and F) were re-clustered separately and analysed by trajectory space (tSPACE), an algorithm that maps cells along nearest neighbour pathways to all other cells in a population, allowing unsupervised visualisation of developmental sequences(44). Two-dimensional representation of a 3-dimensional trajectory Space (tSPACE) UMAP (Flat tUMAP) (Fig. 2A and see Video S1 for 3D representation) identified eleven clusters (M1-M11) (Fig. 2A), all of which were present at varying proportions in paired ileal and colonic LP samples (Fig. S2A). The smallest cluster M11, consisting of only 16 cells, expressed high levels of cell-cycle associated genes including *MKI67* and *KIAA0101* (Fig. S2B), and we did not analyse these cells further due to their sparsity in the dataset. Cluster M1, that was located at one end of the trajectory space (Fig. 2A, Video S1), expressed the highest levels of the monocyte-associated genes, *S100A8, S100A9, FCN1* and *VCAN*(25, 45), the lowest level of *HLA* genes, and did not express the mature macrophage genes *CD209* and *C1QC* (Fig. 2B and Fig. S2B), indicating that they represent recently recruited monocytes. tSPACE and pseudo time analysis using cluster M1 as a trajectory starting point demonstrated that clusters M2 and M3 lay directly downstream of cluster M1 in the trajectory, while clusters M4 and M5 lay directly downstream of clusters M2 and M3 (Fig. 2A and C, Video S1), suggesting that M2-M3 represented early, and M4-M5 late intermediate clusters. Consistent with this idea, M2 and M3 expressed intermediate levels of *S100A9, VCAN* and *ITGAX* and low levels of *C1QC*, while M4-5 lacked expression of *S100A9* or *VCAN*, but expressed intermediate levels of *ITGAX* and *C1QC* (Fig. 2B). Clusters M6-M8 represented three distinct branches at the opposite end of the trajectory, with minor clusters M9 and M10 branching off cluster M8 (Fig. 2A and C, Video S1), suggesting that these clusters represented distinct populations of differentiated macrophages. Consistent with this, M6-M8 expressed the mature macrophage markers *CD209, C1QC* and high levels of *HLA* genes (Fig. 2B and Fig. S2B). Clusters M9 and M10 also had features of mature macrophages, including high expression of MHCII and *C1Q* genes, but expressed low levels of *CD209* and *CD163* (Fig. 2B and Fig. S2B). Trajectory analysis also indicated that clusters M6 and M7-M10 derived from distinct intermediates, with M6 appearing to arise from the late intermediate cluster M5, and clusters M7-M10 from late intermediate cluster M4 (Fig. 2A and C, Video S1). Collectively, these results suggest that there are different subsets of differentiated macrophages in the human intestinal LP that derive from monocytes and differentiate *in situ* via distinct intermediates.

### The intestinal LP contains functionally distinct macrophage subsets that display limited transcriptional differences between the ileum and colon

Given the limited number of cells within clusters M9 and M10, we focused our subsequent analysis of differentiated macrophages on clusters M6-M8. Cluster M6 differentially expressed 1482 genes compared to cluster M7 or M8 (Fig. S2C, Table S2), including genes encoding the C-type lectins CLEC7A and CLEC12A, as well as *TNFSF10* that encodes TNF-Related Apoptosis-Inducing Ligand (TRAIL) (Fig. 2D and E, Table S2). GO analysis demonstrated that cluster M6 was enriched in genes involved in positive regulation of interleukin 1 production, as well as in translation and ribosome biogenesis indicating high translational activity (Fig. 2F). Cluster M7 differentially expressed 1034 genes compared to either cluster M6 or M8 (Fig. S2C, Table S2), including several chemokines; the tissue repair and angiogenesis associated genes *VEGFA, PDGFB* (Fig. 2D, Table S2), and *PTGS2* encoding cyclooxygenase-2 a key enzyme in prostaglandin production (Fig. 2D and E, Table S2). Cluster M7 also expressed *FOLR2, MAF* and *LYVE 1* (Fig. 2D), which are associated with perivascular macrophages(46, 47). Consistent with this, GO analysis demonstrated that M7 macrophages were enriched in pathways associated with inflammatory response, responsiveness to cytokines and molecules of bacterial origin, positive regulation of cell migration, vascular development and regulation of angiogenesis (Fig. 2F). FOLR2 expressing macrophage are found in the middle and crypt region of the colonic LP and in the submucosa(19), however, the latter also express *COLEC12* (18) and *MARCO* (19) which were barely expressed by cluster M7 (Fig. 2D), consistent with efficient removal of submucosa in our LP isolation protocol. Cluster M8 expressed 1140 genes at significantly higher levels than cluster M6 or M7 (Fig. S2C, Table S2). These included high levels of the metalloprotease *MMP12; LGALS3* that encodes galectin-3, and the lipid metabolism-associated genes *PLA2G7, LIPA*, and *APOC1* (Fig. 2D and E, Table S2). Cluster M8 also expressed high levels of *ACP5* (Fig. 2D and Table S2), a marker of differentiated macrophages that locate at the tip of colonic crypts(18, 19). GO analysis demonstrated that M8 macrophages were enriched in pathways associated with aerobic respiration, receptor mediated endocytosis, cellular response to ions, regulation of ferroptosis and lipid transport (Fig. 2F). There were very few differentially expressed genes (DEG) between the ileum and colon for any of the clusters (Fig. 2G, Table S3), and while several genes encoding immune modulatory molecules were differentially expressed by all three clusters in either the ileum or colon (Fig. 2H and I), the relevance of these in regulating local immune homeostasis remains to be determined. Finally, to determine how clusters M6-M8 related to recently described colonic macrophage populations(18), we embedded the signature score (top 50 DEG) for each of our clusters onto a UMAP of the colonic dataset generated by Domanska *et al*(18). (Fig. S2D). Consistent with our findings, the signature score for each cluster overlapped primarily with clusters suggested previously to represent tissue resident macrophage subsets (18), located towards the top of the UMAP (Fig. S2D). While the signature score of M7 located primarily to the top left of the UMAP, corresponding with cluster 12 and half of cluster 10 in the Domanska *et al*. dataset(18), the signature scores of clusters M6 and M8 largely overlapped with one another. Why M6 and M8 could not be distinguished in the Domanska *et al*. dataset remains unclear.

### The proportion of monocytes, monocyte intermediates and differentiated macrophages differs between the ileal and colonic LP and in IBD

**Fig. 3:**
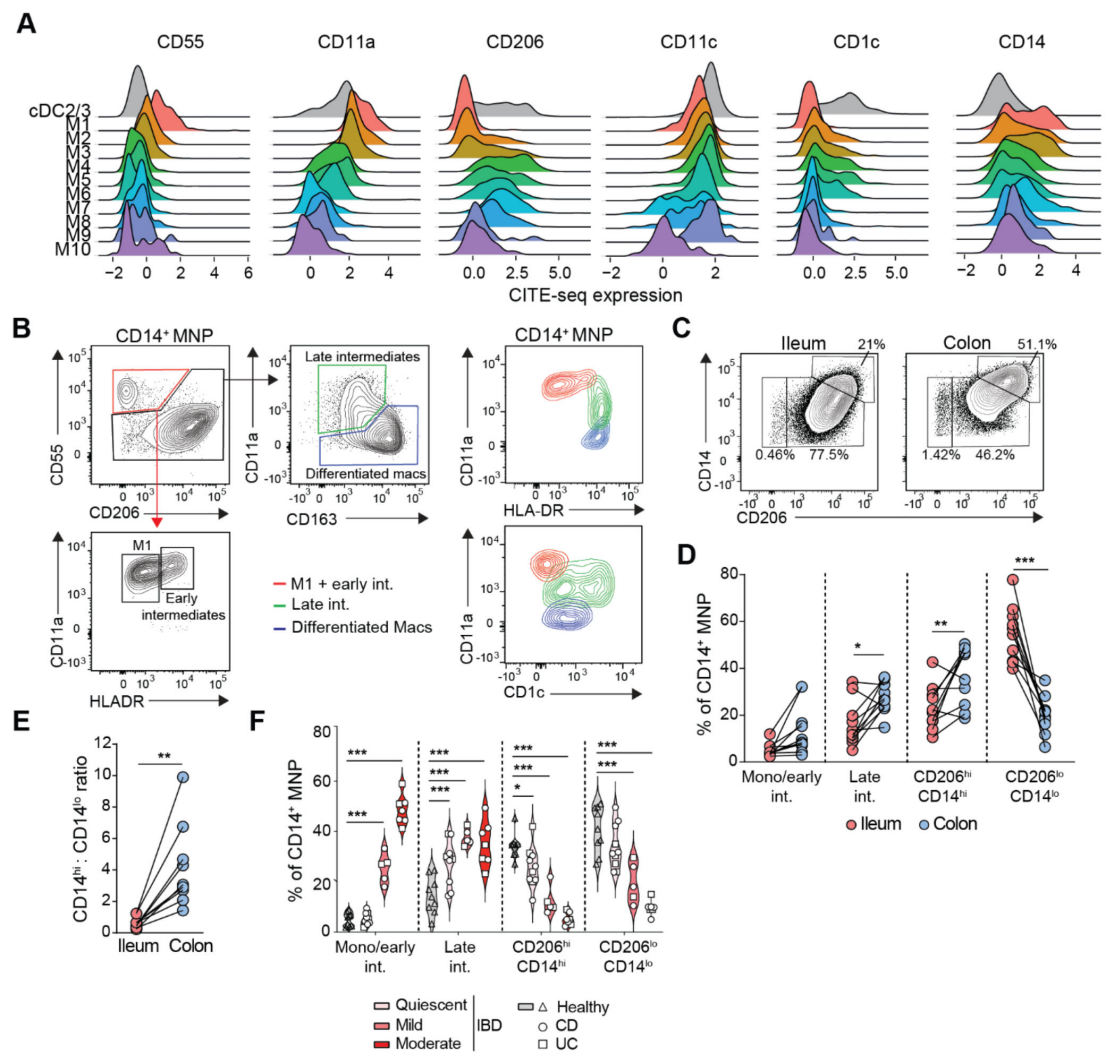
Flow cytometry analysis of mono/mac subsets. **A**) DSB-normalized CITE-seq expression of indicated surface markers on pooled colonic LP macrophage clusters after exclusion of the minor proliferating M11 cluster, as well as total cDC2/3 clusters as control. Data are integrated from three independent colon samples. (**B-E**) Flow cytometry analysis of LP CD14^+^ mono/mac subsets obtained from digested CRC patient resection samples. (**B**) Colonic LP CD14^+^ MNP, showing gating strategy to identify putative mono/mac subsets, and surface expression of CD11a, HLA-DR and CD1c on each identified subset. CD14^+^ MNP were pre-gated as viable CD3^-^ CD19^-^ CD38^-^ CD123^-^ HLADR^+^ CD14^+^ singlets. Data is a representative stain from 1 of 10 patients analyzed. Int, intermediate. (**C**) Surface expression of CD14 vs. CD206 on ileal and colonic LP CD14^+^CD55^-^CD11a^int/low^ macrophages. Data is concatenated from 10 individual patients. (**D**) Proportion of each mono/mac subset in paired ileal and colonic LP. Each symbol represents a paired ileal/colonic sample. Statistical significance was determined using 2-way ANOVA with Sidak’s multiple comparisons, *p<0.05, **p<0.01, ***p<0.001 (**E**) Ratio of CD14^hi^CD206^hi^ to CD14^lo^CD206^lo^ macrophage subsets within the ileal and colonic LP. Each symbol represents a paired ileal or colonic sample. Statistical significance was determined using Wilcoxon matched pairs signed rank test. **p<0.01. (**F**) Proportion of mono/mac subsets in IBD based on flow cytometry analysis of digested colonic biopsies. Extent of inflammation was scored at the time of biopsy by the clinician as quiescent, mild, or moderate. Each symbol represents an individual sample. CD, Crohn’s disease. UC, ulcerative colitis. Int, intermediate. Statistical significance was determined using 2-way ANOVA with Dunnett’s multiple comparison test. ***p<0.001, * p<0.05.

To identify surface antigens that may help identify the stages of intestinal monocyte development by flow cytometry, a LEGENDScreen™ assay was used to screen for surface marker expression on colonic CD14^+^CD1c^lo^ MNP (Fig. S3A and B). CD11c, CD11a, CD206, and CD55 showed heterogenous expression levels on CD14^+^CD1c^lo^ cells (Fig. S3B) and we thus used antibodies recognising these surface markers, together with CD14 and CD1c, to carry out CITE-seq analysis of MNP from three colonic (Fig. 3A) and one ileal LP sample (Fig. S3C). Based on these results and the transcriptional analysis depicted in Fig. 2B, we generated an antibody panel designed to distinguish monocyte-derived early intermediates, late intermediates and differentiated macrophages by flow cytometry (Fig. 3B). Differentiated macrophages could be further divided based on differential expression of CD14 and CD206 into CD14^hi^CD206^hi^ cells, likely to be enriched in cluster M6 and M7 macrophages, CD14^lo^CD206^int^ cells, likely enriched in cluster M8 macrophages, and a minor population of CD14^lo^CD206^lo^ cells, likely enriched in cluster M9 and M10 macrophages (Fig. 3A and C). Flow cytometry analysis from 10 matched ileal and colonic resection samples suggested the proportion of intermediate cells among total mono/mac to be higher in the colon compared with the ileal LP and that the colonic LP contained a higher proportion of CD14^hi^CD206^hi^ and lower proportion of CD14^lo^CD206^lo^ differentiated macrophages compared within the ileal LP (Fig. 3D and E). To assess whether the proportions of these populations changed during inflammation, similar analysis was performed from colonic biopsies from healthy individuals (where an endoscopic examination was scheduled, however, without any pathological signs), and patients with CD and ulcerative colitis (UC) (Fig. 3F, for patient information see Table S4). This revealed a clear correlation between the presence of inflammation and increased proportions of early and late intermediates, together with concomitant decreases in the proportions of both mature CD14^hi^CD206^hi^ and CD14^lo^CD206^lo^ macrophage populations (Fig. 3F). Thus, multiple stages of monocyte-macrophage differentiation can be identified in the human intestine by flow cytometry, and these differ between the between the ileum and colon as well as in the setting of IBD.

### Identification of cDC1, cDC2 and cDC3 in the human intestine

To explore intestinal LP cDC diversity, we computationally isolated and reclustered the cDC1 and cDC2/cDC3 cells identified in Figure 1. Cells were clustered at high resolution and visualized by tSPACE based clustering by Flat tUMAP as in Fig. 2A. All subclusters were present in both ileal and colonic LP datasets (Fig. S4A). Despite regressing out cell cycle effects (see Materials and Methods), there were several clusters located together at the top of the tUMAP that were enriched in cells expressing high levels of mitotic G2M/S genes (Fig. S4B) and relatively low levels of MHCII genes (Fig. S4C), both of which are characteristics of cDC precursors (see below). To assess the identity of the remaining clusters, we first analysed expression of the canonical cDC1 signature genes *CLEC9A, CADM1, XCR1, BATF3* and *IRF8*, identifying 7 clusters with a clear cDC1 signature score (Fig. S4D). We then ranked the remaining clusters based on their average module expression of the cDC2- or cDC3-associated signature genes published by Bourdely *et al*(48) (Fig. S4E). This allowed us to tentatively identify cDC2 and cDC3, as well as clusters that could not be identified definitively based on their cDC2/DC3 signature score, which we termed ambiguous clusters (Fig. S4E). We also identified a minor population of *LAMP3*^+^ cDC, which expressed high levels of the maturation markers *CCR7* and *CD40* (Fig. S4F), likely representing mature cDC with the capacity to migrate towards lymph nodes(49, 50).

**Fig. 4:**
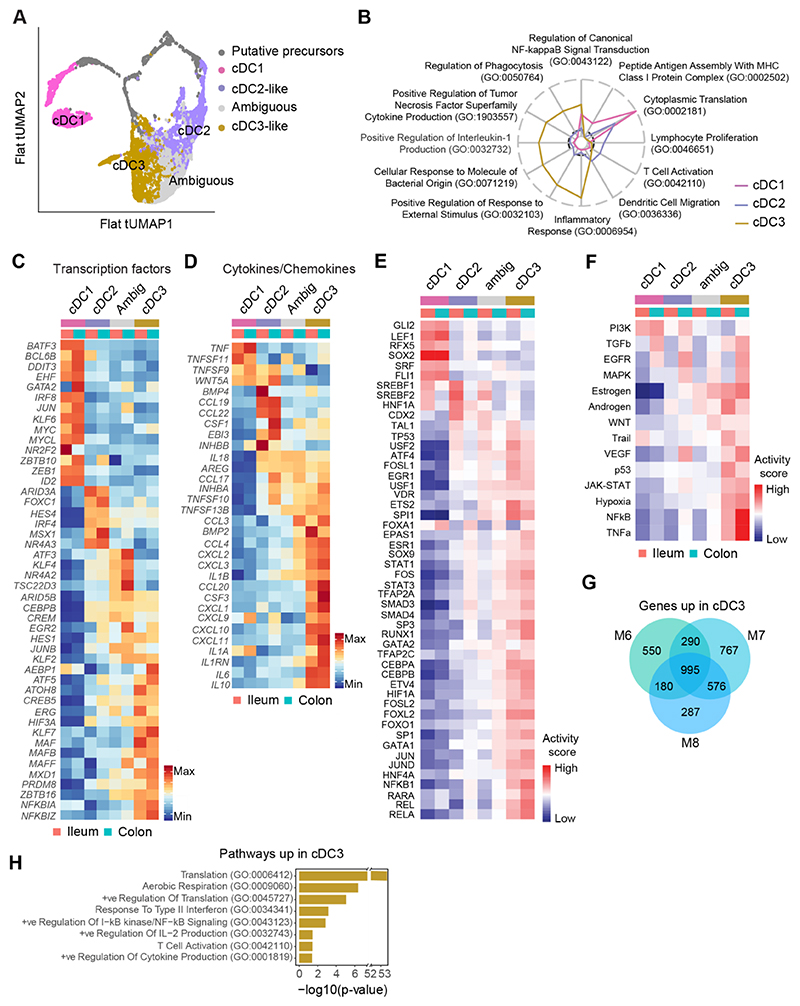
Transcriptional characterization of intestinal LP cDC subsets. **A**) 2-dimensional representation of a 3-dimensional tSpace UMAP (tUMAP) plot of ileal and colonic LP cDC clusters grouped into indicated populations based on high-definition clustering and analysis in Fig. S2. (**B**) Radar plot showing selected GO terms differentially enriched in each cDC subsets based on DEGs for each cDC subset (Table S5). Y-axis = sqrt(-log(adjusted P-value)). Dashed line indicates significance threshold of adjusted P-value = 0.05. (**C** and **D**) Manually curated pseudo-bulk heatmaps of differentially expressed (**C**) transcription factors and (**D**) cytokines and chemokines, between indicated cDC subsets. (**E**) DoRothEA based inferred transcription factor activity and (**F**) PROGENy based inferred signaling pathway activity in indicated cDC subsets and tissue. (**G**) Venn diagram displaying the number of genes expressed at higher levels by cDC3 compared with each of the indicated macrophage subsets. (**H**) Selected GO terms enriched in cDC3. Analysis was performed on the shared 995 genes upregulated in cDC3 compared with M6-M8.

The cDC1, cDC2 and cDC3 clusters were largely separated from each other in the tUMAP projection, with the ambiguous clusters positioned between the cDC2 and cDC3 (Fig. 4A). Each cDC subset expressed a large number of DEG, including genes supporting their designation as cDC1, cDC2 and cDC3 (Fig. S4G and H and Table S5). Specifically, the top DEG for the cDC1 cluster included *CLEC9A, CADM1* and *ID2*, the cDC2 cluster expressed high levels of *IRF4, PLAC8* and *CCL22*, the cDC3 cluster expressed high levels of *C1QA, S100A9* and *CD163*, while the ambiguous cluster expressed genes associated with both cDC2 and cDC3 (Fig. S4H). As has been observed previously for cDC2 and cDC3 in blood(29, 48), *CD1C, CLEC10A* and *FCER1A* were expressed at comparable levels by the ambiguous, cDC2 and cDC3 clusters in both ileal and colonic LP (Fig. S4H). GO terms enriched in the cDC subsets included ‘Cytoplasmic translation’, ‘Antigen processing and presentation of peptide antigen via MHC class I’ for cDC1, ‘T cell activation and lymphocyte proliferation for cDC2 and cDC3, and ‘cellular response to molecule of bacterial origin’ and ‘inflammatory response’ for cDC3 (Fig. 4B).

To gain further insights into potential differences between intestinal cDC subsets, we manually curated a list of DEG between the cDC subsets, focusing on GO terms associated with TFs involved in controlling cDC development and function, and the cytokines/chemokines (51) that mediate cDC function (Fig. 4C and D). In addition to classical cDC1-associated TFs such as *IRF8, ID2* and *BATF3*, ileal and colonic cDC1 differentially expressed several other TFs including *ZEB1* (Fig. 4C), implicated in cDC1-mediated Th1 responses(52), as well as *JUN, MYC* and *MYCL* implicated in cDC1 development and function (53, 54). As well as the cDC2 associated TF *IRF4, cDC2* selectively expressed the Notch pathway gene *HES4*, as well as *NR4A3*, implicated in cDC activation(55). As expected, cDC3 expressed the highest levels of macrophage-associated TFs including *MAF, MAFB, MAFF* and *ZBTB16*, and also expressed the inhibitory TFs *NFKBIA* and *NFKBIZ*, while cells within the ambiguous cluster expressed high levels of several TFs associated with activation, including *ATF3* and *JUNB* (Fig. 4C). Ambiguous cells also expressed the highest levels of *KLF4* (Fig. 4C), a transcription factor implicated in cDC2-mediated Th2 responses(56). Cytokines and chemokines were also expressed in a cDC subset-specific manner (Fig 4D). Ileal and colonic LP cDC1 expressed high levels of the TNF family members *TNF* and *TNFSF11* (RANK-L), cDC2 expressed high levels of *CCL19, CCL22* and *EBI3* and cDC3 expressed a wide range of cytokines and chemokines, including *IL10, IL1B* and *IL6* and the interferon-inducible chemokines *CXCL9, CXCL10* and *CXCL11* (Fig 4D).

To assess potential differences in TF and signalling pathway activity between cDC subsets, we used the Discriminant Regulon Expression Analysis package (DoRothEA), which infers transcription factor activity from expression of downstream target genes(57) (Fig. 4E), and the Pathway RespOnsive GENes package (PROGENy), which infers pathway activity in cells based on expression levels of pathway response genes(58) (Fig. 4F). DoRothEA analysis suggested selective SOX2, FLI1, LEF1, and FOXA1 activity in cDC1 (Fig. 4E), while cDC3 showed enhanced activity of a broad range of TF associated with different activation pathways, including JUN, JUND, NFKB1, REL, RELA, STAT1 and STAT3 (Fig. 4F), consistent with their TF and cytokine/chemokine gene expression profiles (Fig. 4C and D). PROGENy analysis suggested that the PI3K pathway was particularly active in cDC1, while cDC3 displayed a broad activation of the estrogen, androgen, WNT, TRAIL, VEGF, p53, JAK-STAT, hypoxia, NFκB, and TNFα pathways relative to the other cDC subsets (Fig. 4F), consistent with gene expression and DoRothEA analysis (Fig. 4C-E). Collectively, these results highlight the distinct transcriptional activities of human intestinal cDC subsets and suggest cDC3 have a more activated, pro-inflammatory phenotype compared with other LP cDC.

As cDC3 co-expressed several genes associated with the mono/mac lineage, we asked how they might compare with the three major differentiated LP macrophage subsets M6-M8. As well as differing from each of the macrophage subsets individually, cDC3 expressed a shared set of 995 genes at higher levels than all M6-M8 clusters (Fig. 4G and Table S6). GO analysis on this common gene set showed enrichment for pathways of translation and aerobic respiration (Fig. 4H), indicating high metabolic activity, together with genes associated with NF-kB signalling, cytokine production and T cell activation (Fig. 4H). Collectively these results indicate that cDC3 are likely to have unique functions in the intestine when compared with differentiated macrophages, cDC1 or cDC2.

### The transcriptional profile and proportion of cDC subsets differs between the ileum and colon lamina propria

**Fig. 5:**
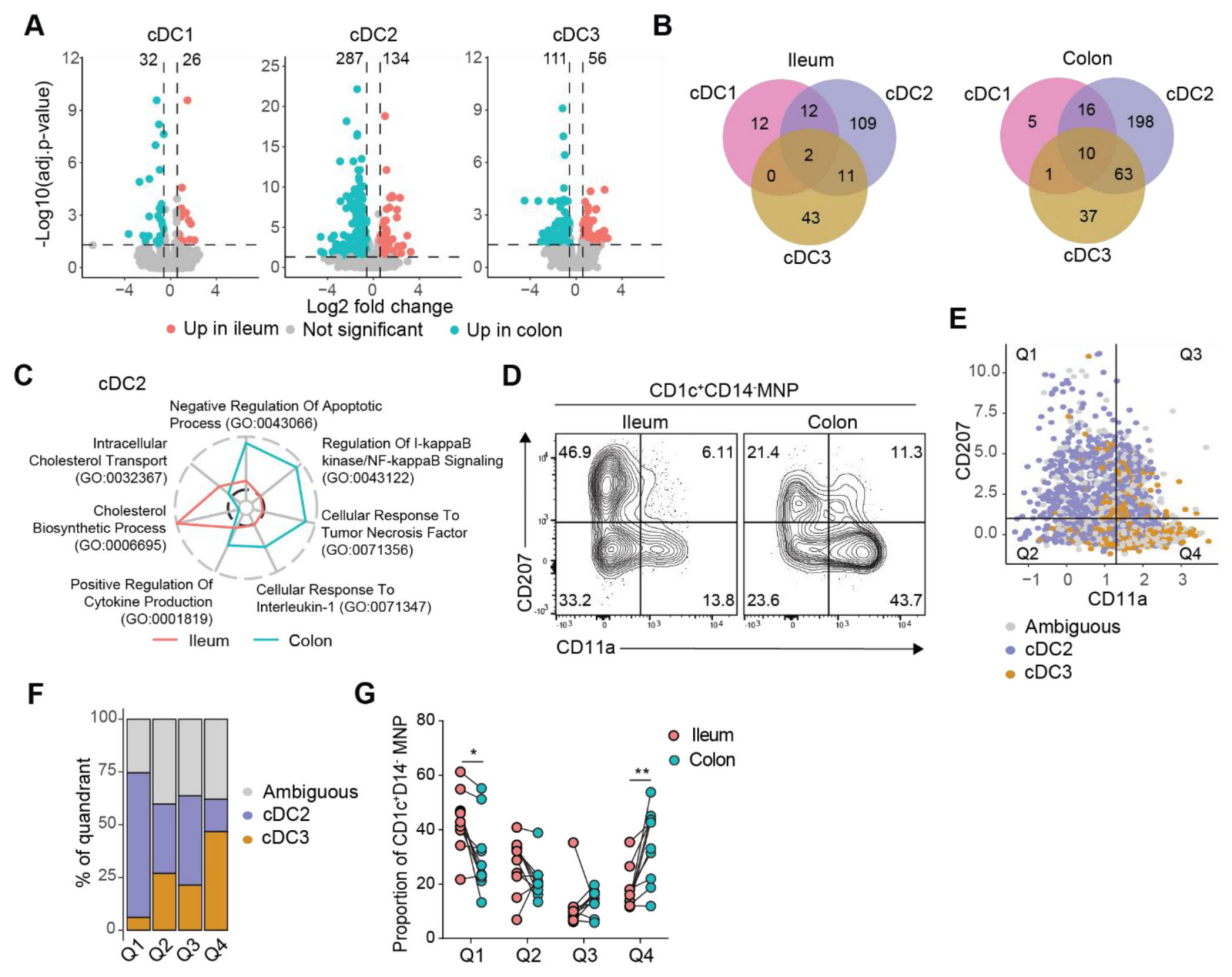
cDC2 and cDC3 are found in different proportions in the ileal and colonic LP. **A**) Volcano plots of DEGs of indicated cDC subsets between pooled ileum and colon LP samples. Dashed lines indicate significance cut-offs. Adjusted P-values < 0.05 and |avg. logFC| > 0.2. (**B**) Venn diagrams displaying number of genes upregulated in indicated cDC subset in ileum and colon as well as genes commonly upregulated within these subsets. (**C**) Selected GO terms enriched in ileal or colonic cDC2. Y-axis = sqrt(-log(adjusted P-value)). Dashed line indicates significance threshold of adjusted P-value = 0.05. (**D**) CD207 and CD11a surface expression on CD1c^+^CD14^-^ MNP from indicated tissues using flow cytometry. Results are representative of 10 ileal and colonic LP samples. (**E** and **F**) Pooled colonic LP samples from three CRC resection patients showing (**E**) CD207 and CD11a expression on colonic cDC2, cDC3 and ambiguous cDC clusters using DSB-normalized ADT portion of CITE-seq, and (**F**) proportion of each cDC cluster within each of the 4 quadrants (Q1-4) depicted in (**E**). (**G**) Proportion of CD207^+^CD11a^-^ (Q1), CD207^-^CD11a^-^ (Q2), CD207^+^CD11a^+^ (Q3) and CD207^-^CD11a^+^ (Q4) cells amongst CD1c^+^CD14^-^ MNP from paired ileal and colonic LP from CRC resection patients (n=10).

We next assessed whether the cDC subsets showed transcriptional differences between ileal and colonic LP. DEG analysis showed that the transcriptional profile of cDC1 differed little between the ileum and colon LP (Fig. 5A, Table S7), indicating that these distinct environments had little impact on cDC1 activity. In contrast, cDC2 differed in their expression of 421 genes, while cDC3 differed in their expression of 167 genes between the ileum and colon and a proportion of these genes was shared between the subsets (Fig. 5A and B, Table S7). Given the limited number of DEG in cDC3, we focused GO analysis on cDC2 and found ileal cDC2 to be enriched in pathways associated with cholesterol transport and cholesterol biosynthetic processes, whereas colonic cDC2 were enriched in cellular responses to cytokines, cytokine production and negative regulation of apoptotic process (Fig. 5C).

CITE-seq analysis demonstrated that the cDC2, cDC3 and ambiguous cDC populations could be distinguished from monocytes and macrophages based on their high expression of CD1c and low expression of CD14 (Fig. S5A). To assess whether cDC2 and cDC3 proportions differed between the ileum and colon, we first performed LEGENDScreen™ to identify surface markers of potential use for identifying cDC2 and cDC3 amongst CD1c^+^CD14^-^ MNP (data not shown). Of the antibodies screened, CD11a and CD207 were found to separate colonic and ileal LP CD1c^+^CD14^-^ MNP into 4 populations (Fig. 5D, for pre-gating see Fig. S5B) and CITE-seq analysis with these antibodies demonstrated that CD207^-^CD11a^-^, CD207^+^CD11a^-^, CD207^+^CD11a^+^ or CD207^-^CD11a^+^ cells could be identified within the cDC2/3 supercluster (Fig. 5E). While the ambiguous cDC population distributed evenly between all 4 quadrants (Fig. 5E and F), cDC2 were enriched in the CD207^+^ CD11a^-^ (Q1) gate, while cDC3 were enriched in the CD207^-^CD11a^+^ (Q4) gate (Fig. 5F). CITE-seq analysis of paired ileal and colonic LP samples from one patient showed similar enrichment of cDC2 in Q1 cells and cDC3 in Q4 cells in the ileum (Fig. S5C and D). To assess whether ileal and colon LP contained different proportions of these populations, flow cytometry analysis was performed on CD1c^+^CD14^-^ MNP from uninvolved paired ileal and colonic resection samples from CRC patients (Fig. 5G). Ileal LP CD1c^+^CD14^-^ MNP were significantly enriched in CD207^+^CD11a^-^ (Q1) cells compared with the colonic LP, while colonic LP CD11c^+^CD14^-^MNP were enriched in CD207^-^CD11a^+^ (Q4) cells (Fig. 5G). Collectively these results suggest that the transcriptional profile and proportions of cDC2 and cDC3 differs between the ileum and colon.

### The human intestinal LP contains putative cDC1, cDC2 and cDC3 precursors

**Fig. 6:**
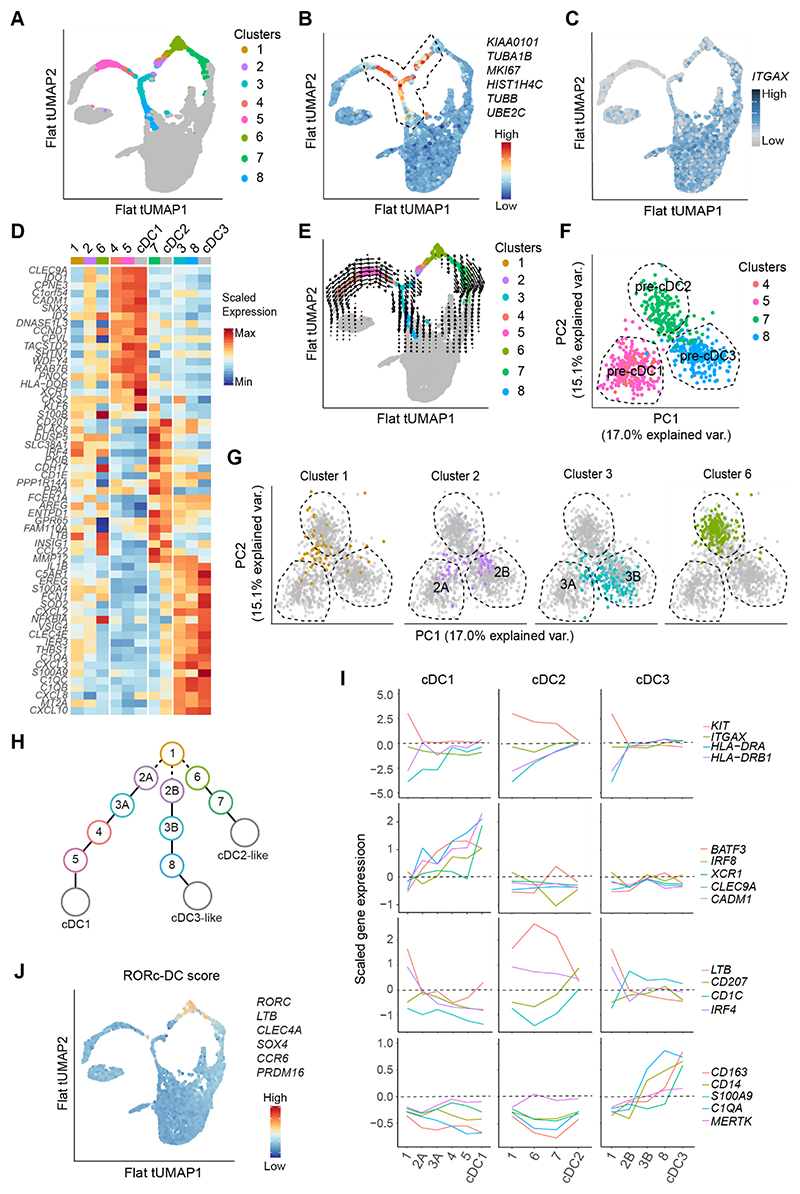
Identification of cDC committed precursors in the human intestine. **A-C**) Two-dimensional representation of a 3-dimensional tSpace UMAP (Flat tUMAP) of ileal and colonic LP cDC clusters. (**A**) HLA-DR^low^ cDC clusters (cluster 1-8) overlaid onto cDC tUMAP, (**B**) proliferation score of indicated cell-cycle-associated genes and (**C**) *ITGAX* expression levels overlaid onto cDC tUMAP. (**D**) Heat map of top 20 DEG (calculated using p.adj. < 0.05) between cDC1, cDC2 and cDC3, showing expression levels in HLA-DR^low^ cDC clusters. (**E**) RNA velocities (arrows) of HLA-DR^low^ cDC clusters 3-5 and 7-8 calculated with Velocyto package embedded onto the cDC tUMAP. (**F**) PCA plot of cells from clusters identified by shared DEG (from **D**) as either pre-cDC1 (clusters 4 and 5), pre-cDC2 (cluster 7) or pre-cDC3 (cluster 8) and **(G)** location of clusters not identifiable in (D) (clusters 1-3 and 6) overlaid on the PCA plot in (**F**). **(H)** Model of precursor cluster trajectories towards mature cDC subsets based on tSpace, velocity and transcriptional analysis. (**I**) Expression of indicated genes across proposed cDC1-, cDC2-, and cDC3- trajectories. (**J**) Score of indicated RORc-DC associated genes from Antonova *et al*.(67) embedded onto the cDC tUMAP.

Recent studies have identified putative committed precursors of cDC1 (pre-cDC1), cDC2 (pre-cDC2), and more recently, cDC3 (pre-cDC3), as well as uncommitted pre-cDC precursors, in human bone marrow, blood and tonsils(25, 29, 48, 59–61). To explore whether cDC precursors might also be present in human intestine, we focused on the HLA-DR^low^ cDC (Fig. S4C), which, using high-resolution tSpace based clustering, consisted of 8 clusters (Fig. 6A). These cells were highly proliferative compared with the mature cDC (Fig. 6B) and expressed low levels of *ITGAX* (encoding CD11c) (Fig. 6C), features consistent with previous studies of pre-cDCs in mice(62, 63) and humans(59, 60). Given that these proliferating clusters formed three distinct branches that aligned with cDC1, cDC2 and cDC3, we hypothesized that each branch potentially represented cDC subset-specific precursors.

To assess this possibility, we generated signatures composed of the top 50 DEGs which distinguished the mature cDC subsets from each other and examined how these were expressed by the various clusters of HLA^low^ putative pre-cDC. This analysis showed that cluster 4 and 5 shared a gene expression profile with cDC1, while cluster 7 expressed DEGs associated with cDC2 and cluster 3 and 8 had a similar gene expression pattern to cDC3 (Fig. 6D), suggesting the possibility that these clusters represented distinct cDC lineage specific precursors. RNA velocity analysis of mRNA splicing patterns(64) further supported this idea, with cluster 4 appearing to be at the beginning of a trajectory with directionality into cluster 5, and thereafter into the mature cDC1 clusters (Fig. 6E). Similarly, cluster 7 showed a trajectory into the mature cDC2 clusters, while cluster 3 showed a trajectory towards cluster 8 and then into mature cDC3 (Fig 6E); similar patterns were observed in the ileum and colon LP (Fig. S6A). Collectively, these gene expression and splicing patterns suggest that clusters 4 and 5 represent pre-cDC1, while cluster 7 represents pre-cDC2 and clusters 3 and 8 represent pre-cDC3.

Three adjacent clusters (clusters 1, 2, and 6) did not express DEG specific to the mature cDC subsets (Fig. 6D) and we hypothesized that they may be earlier, less-committed precursors. To determine whether clusters 1, 2, and 6 showed evidence of commitment to any of the cDC lineages, we used the top 50 DEGs from each of the committed precursor clusters 5 (putative pre-cDC1), 7 (putative pre-cDC2), and 8 (putative pre-cDC3) as input for a PCA of all the HLA^low^ clusters. A total of 150 DEGs were found between the putative precursor clusters, of which 79 were also DEGs between the relevant mature cDC populations. cDC1 precursor cluster 5, cDC2 precursor cluster 7 and cDC3 precursor cluster 8 split into 3 distinct areas in PC1-2 (Fig. 6F). Within this PCA, the putative early precursor cluster 6 aligned clearly with pre-cDC2 and most of those in cluster 3 aligned, as expected, with pre-cDC3 (3B). There were a few cells in the pre-cDC1 area (3A), while one subset of cluster 2 (2A) aligned with pre-cDC1 and another subset of cluster 2 (2B) aligned with pre-cDC3 (2B) (Fig. 6G and Fig. S6B)). In contrast, cluster 1 did not overlap clearly with any of the pre-cDC groups (Fig. 6G).

To investigate the identity of the cells in cluster 1, we compared their gene expression profile with that of a published human bone marrow hematopoietic single-cell dataset (65). Cluster 1 showed greatest correlation with hematopoietic stem cells (HSC), multipotent progenitors (MPP), lympho-myeloid precursors and early promyelocytes, together with some overlap with mature BM cDC. However, they showed no overlap with late promyelocytes, myelocytes and classical monocytes (Fig. S6C). Thus cluster 1 appears to represent early lympho-myeloid progenitors with a potential bias towards the cDC lineage.

To further assess the relationship between mature cDC and their putative precursors, we aligned clusters along the three putative cDC1, cDC2 and cDC3 developmental trajectories (Fig. 6H), and examined the expression of DC precursor and cDC subset associated genes across these trajectories (Fig. 6I). Compared with other clusters, cluster 1 expressed the highest levels of *KIT*, similar levels of *ITGAX* and the lowest levels of MHCII genes (Fig. 6I), consistent with the suggestion that cells within this cluster represent early progenitors(25, 29, 59, 61). In agreement with the proposed trajectories, pre-cDC1 clusters progressively increased their expression of cDC1 related genes *BATF3, IRF8, CLEC9A* and *CADM1* as they transitioned through clusters 2A, 3A, 4 and 5 to mature cDC1. *XCR1* expression increased during the final transition from cluster 5 to mature cDC1 (Fig. 6I), consistent with recent studies in mice suggesting this marker is expressed relatively late in cDC1 differentiation(66). As expected, expression of these cDC1 genes remained low throughout the putative cDC2 and cDC3 trajectories. Conversely, expression of the cDC2 associated genes *IRF4* and *LTB* remained high across the cDC2 trajectory but was downregulated along the cDC1 and cDC3 trajectories (Fig. 6I). *CD207* expression selectively increased along the cDC2 trajectory, while *CD1C* expression increased along both cDC2 and cDC3 trajectories and decreased along the cDC1 trajectory (Fig. 6I). Finally, the putative pre-cDC3 clusters displayed a progressive increase in expression of the myeloid and cDC3 associated genes *CD163, CD14, S100A9, C1QA* and *MERTK* as they transitioned through clusters 2B, 3B and 8 to mature cDC3 (Fig. 6I), albeit these genes were expressed at far lower levels than observed in the mono/mac clusters (Fig. 1E). A RORgt expressing antigen presenting cell population, termed RORgt-DC, has recently been identified in human tonsil and found to be capable of giving rise to cDC2 *in vitro*(67). Strikingly, the gene signature score of these RORgt-DC, including expression of *RORC* and the histone methyltranferase *PRDM16*, overlapped with our putative early cDC2 progenitor cluster 6 (Fig. 6J, Fig. S6D and E), but there was no overlap with progenitors of RORgt^-^ILC3. Together these findings indicate that intestinal cDC2 may originate from a *RORC*-expressing precursor.

### Intestinal cDC subset composition is altered in inflammatory bowel disease

**Fig. 7:**
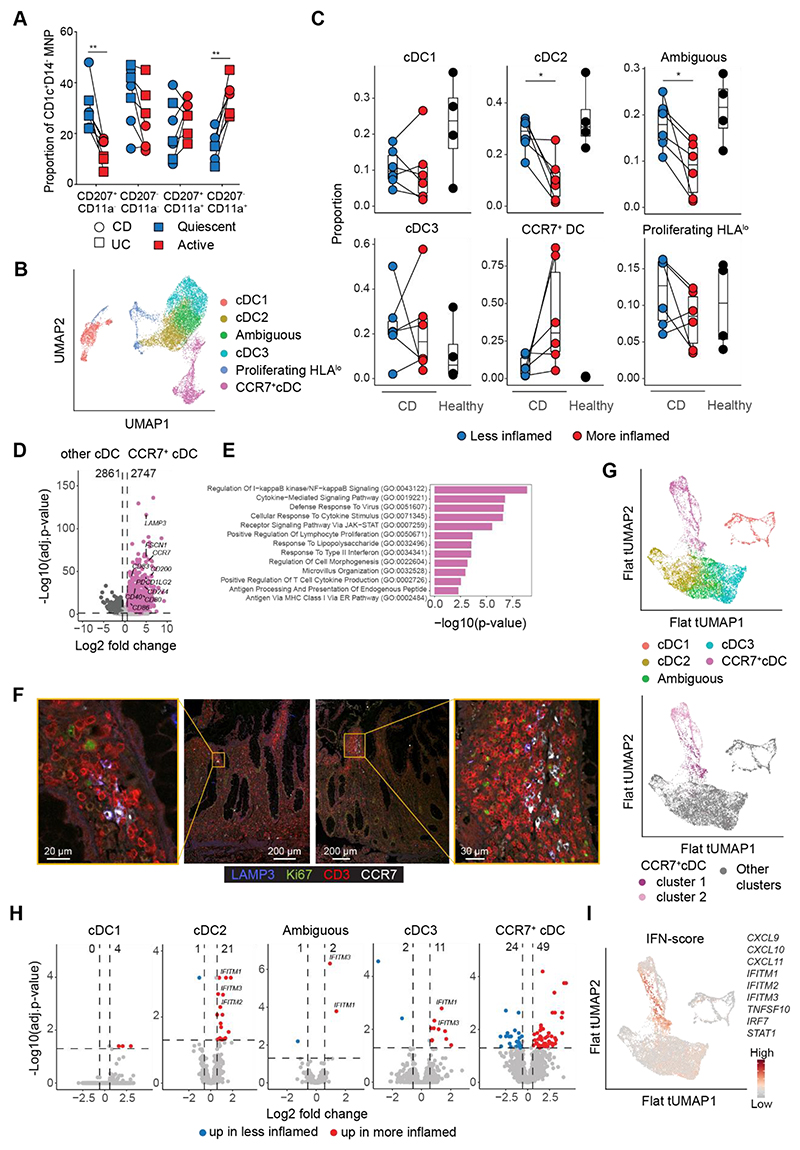
Intestinal cDC subset characterization in IBD. **A**) Paired colonic biopsies taken from areas of quiescent or active inflammation from IBD patients (n=7) as assessed by flow cytometry. Each symbol is from 3-5 pooled biopsies per site and the inflammatory activity of each site was scored as quiescent or active by the endoscopist at time of removal. CD, Crohn’s disease. UC, ulcerative colitis. Statistical significance was determined using 2-way ANOVA with Sidak’s multiple comparisons, **p<0.01. (**B**) UMAP showing cDC subset cluster designation in pooled ileal LP scRNA-seq datasets from surgical resections of 6 CD patients with paired more and less inflamed regions and **4** CRC samples. (**C**) Proportions of indicated cluster amongst total cDC comparing paired more and less inflamed ileal regions from Crohn’s disease (CD) patients. Healthy, proportions of indicated cluster observed in ‘healthy’ ileal regions from CRC patients. Each symbol represents scRNA-seq data from one ileal sample. Statistical significance was determined using paired t-test, *p<0.05. (**D**) Volcano plot showing number of DEG between CCR7^+^ cDC, with selected named genes upregulated in CCR7^+^cDC compared with other cDC subsets from the pooled dataset in (**B**). Dashed lines indicate significance cut-offs. Adjusted P-values < 0.05 and |avg. logFC| > 0.2. (**E**) GO analysis showing selected pathways upregulated in CCR7^+^ cDC compared with other cDC. (**F**) Inflamed ileal LP of CD patient stained for the indicated antigens. Results are epresentative of 1 of 3 patients analysed. (**G**) Two-dimensional representation of a 3-dimensional tSpace UMAP (Flat tUMAP) showing indicated cDC clusters (left) and CCR7^+^ cDC subclusters (right). (**H**) Volcano plot showing number of DEG between more and less inflamed ileum regions from CD patients for each of the cDC subsets. Dashed lines indicate significance cut-offs. Adjusted P-values < 0.05 and |avg. logFC| > 0.2. (**I**) Expression of interferon response module score embedded onto a Flat tUMAP of cDC.

To investigate whether intestinal cDC proportions are altered in IBD, colonic biopsies from treatment-naïve patients, undergoing endoscopic screening for inflammatory IBD diagnosis and subsequently diagnosed with CD or UC, were digested and analysed by flow cytometry (for patient information see Table S4). This demonstrated that areas of active inflammation contained significantly reduced proportions of CD1c^+^CD207^+^CD11a^-^ cDC and significantly increased proportions of CD1c^+^CD207^+^CD11a^-^ cDC (Fig. 7A). To gain a broader understanding of potential changes in cDC subset proportions and transcription in IBD, we performed scRNA-seq on flow cytometry-sorted CD45^+^CD3^-^CD19^-^HLADR^int/+^ cells from ileal LP surgical samples from CD patients with more and less inflamed regions taken from each patient (Table S8). After bioinformatic removal of mono/macs, the CD and CRC datasets were integrated with one another, visualized by UMAP and the identity of each cluster determined using the cDC labels from the CRC samples (Fig. S7A), together with cDC subset-specific signatures scores (Fig. S7A and B). As well as confirming the presence of all the major cDC subsets, this analysis also revealed a prominent population of *CCR7*^+^ cDC within the CD samples (Fig. 7B). Paired analysis demonstrated that the proportions of cDC2 and ambiguous cells were significantly reduced in the more inflamed areas of CD LP compared with the inflamed areas. The proportion of *CCR7*^+^ cDC also appeared to increase with inflammation (Fig. 7C), although this did not reach statistical significance (Fig. 7C).CITE-seq analysis indicated that all cDC subsets (except cDC1), including *CCR7*^+^ cDC, expressed similar levels of CD1c in more and less inflamed LP regions (Fig. S7C). Given that *CCR7*^+^ cDC expressed intermediate levels of CD11a and did not express *CD207* mRNA (Fig. S7C), it seems likely that the increased numbers of *CCR7*^+^ cDC contributed to the increased proportion of CD1c^+^CD207^-^CD11a^+^ cDC observed in inflamed biopsies of CD patients by flow cytometry (Fig. 7A).

Given the large fraction of *CCR7*^+^ cDC present in the LP of CD patients, we compared their transcriptional profile with other intestinal cDC subsets. *CCR7*^+^ cDC differed in their expression of >5000 genes compared with all other *CCR7*^-^ cDC (Fig. 7D). These included increased expression of the lysosomal gene *LAMP3*, the maturation markers *CD40, CD80, CD83, CD86* and *CD83*, aldehyde dehydrogenase *ALDH1A* involved in the metabolism of vitamin A, and the inhibitory molecules *CD273* (encoding PDL2) and *CD274* (encoding PDL1) (Fig. 7D, Fig. S7D, Table S9); there were also reduced levels of TLR and associated adaptor genes (Fig. S7D, Table S9). This profile is similar what is previously described for *CCR7*^+^ cDC in by Maier *et al*(50). Consistent with this, *CCR7*^+^ cDC were enriched in GO terms associated with NF-kB and cytokine signalling, response to Type II interferons, microvillus organisation, antigen processing and presentation and regulation of lymphocyte proliferation (Fig. 7E). Consistent with previous findings(38, 68), CCR7^+^LAMP^+^ cDC were readily detected in inflamed LP of CD patients (Fig. 7F), and in intimate association with T cell aggregates (Fig. 7F), some of which were proliferating, supporting a potential role in orchestrating local T cell responses(69, 70).

To examine *CCR7*^+^ cDC in more detail, we bioinformatically isolated them and found they split into two main subclusters that differed in expression of 4924 genes (Fig. S7E and Table S10). Of these, subcluster 1 was enriched in GO terms such as aerobic respiration, defense against virus, phagocytosis and antigen processing, while subcluster 2 was enriched in GO terms associated with NF-kB and Wnt signalling, T cell activation and proliferation (Fig. S7G, Table S10). There was a trend towards relative expansion of *CCR7*^+^ cDC subcluster 2 in more inflamed LP regions of CD patients, although this did not reach statistical significance(Fig. S7F) To assess more closely the relationship of the two *CCR7*^+^ cDC subclusters with one another and with other cDC, we performed tSpace on the cDC clusters (Fig. 7G, and Video S2). Consistent with their expression of CD1c (Fig. S7C), *CCR7*^+^ cDC connected directly with the cDC2/ambiguous/cDC3 supercluster, but not with cDC1 (Fig. 7G, and Video S2), suggesting that most *CCR7*^+^ cDC in the LP of CD patients do not derive from cDC1. While cDC3 numbers were not altered by the presence of inflammation (Fig. 7C) and *CCR7*^+^ cDC connected primarily to the cDC2 and ambiguous clusters (Fig. 7G, *upper panel* and Video S2), our findings do not exclude the possibility that some cDC3 may differentiate into *CCR7*^+^ cDC. Notably, *CCR7*^+^ subcluster 1 directly connected with the cDC2 and ambiguous clusters, while *CCR7*^+^ subcluster 2 connected only with subcluster 1 (Fig. 7C, *lower panel* and Video S2), suggesting that cells within subcluster 2 arose from subcluster 1. These results are consistent with recent findings that *CCR7*^+^cDC in the synovial tissue of rheumatoid arthritis patients consist of two subsets, *MIR155*^+^cDC and *LAMP3*^+^cDC, that originate from cDC2, with the *LAMP3*^+^cDC appearing to arise from *MIR155*^+^cDC intermediates(71). Indeed, we could show that the signature score of synovial derived *MIR155*^+^cDC correlated most closely with subcluster 1, while that of *LAMP3*^+^ cDC correlated most closely with subcluster 2 (Fig. S7H). Each cDC subset exhibited few DEG between more and less inflamed LP regions, with *IFITM1*-*IFITM3*, that encode interferon induced transmembrane proteins, being upregulated in cDC2, ambiguous and cDC3 in more inflamed LP (Fig. 7H, for full list of DEG see Table S11). Moreover, *CCR7*^+^ subcluster 1 was highly enriched in interferon response genes (Fig. 7I). Collectively, these results suggest that inflammation in the LP of CD patients drives cDC2 (and potentially cDC3) into *CCR7*^+^cDC and that this transition is associated with a transient increase in interferon signalling.

## Discussion

MNP play critical roles in tolerance, immunity and inflammation, but they are highly heterogeneous, and their subsets also acquire distinct functions depending on the niche in which they reside. Characterizing MNP diversity in distinct human tissues is thus essential for our understanding of their roles in homeostasis and disease. Here, we extend previous studies of human intestinal MNP (18, 38, 45, 72, 73), demonstrating that the human ileal and colonic LP contain numerous transcriptionally distinct MNP, including monocytes, monocyte-macrophage intermediates, differentiated macrophage subsets, cDC1, cDC2, cDC3 and mature CCR7^+^ cDC, as well as putative lineage-specific cDC precursors. We further show that the relative proportions of many of these populations’changes across sites and in the setting of inflammation. Collectively, our results provide an important roadmap of the human intestinal MNP compartment and a framework for future studies aimed at modulating this compartment for therapeutic purposes.

Prior scRNA-seq analyses of human intestinal macrophages have focused primarily on the colon and demonstrated macrophage diversity within both the mucosa and underlying mucosa muscularis(18, 19, 39). Here, we confirm and extend these findings, by showing that the ileal and colonic LP contain similar populations of monocytes, intermediates and differentiated macrophage subsets, albeit in different proportions. Our trajectory analysis of intestinal mono/mac indicated that all major differentiated macrophage subsets were derived from blood monocytes. However, our analysis was based on a relatively aged cohort of patients and as intestinal myeloid populations change with time (74), it remains possible that macrophage populations of embryonic origin exist in the intestinal LP of younger individuals and that the rate of replenishment may vary across subsets and life course. Our findings further suggest that the three major differentiated macrophage subsets develop *in situ* via distinct monocyte-derived intermediates. Such results are in line with murine studies demonstrating that monocyte-derived macrophage subsets acquire specialized functions in response to local niche-specific signals as they differentiate towards tissue residency(75).

While the mechanisms driving differences in the relative proportions of mono/mac subsets between the ileum and colon remain unclear, we speculate that the high proportions of intermediate monocytes in the colonic LP may reflect higher turnover of this compartment. Given that different populations of mature macrophages occupy distinct anatomical niches within tissues, including around blood vessels and neurons(76), differences in the proportion of macrophage subsets between the ileum and colon may reflect variation in the size of these niches in the two tissues. This may also help explain why we observed limited differences in the transcriptional profile of differentiated macrophage subsets between the ileum and colon, as the local factors specifying the final fate of each macrophage subset may be shared between these sites.

Our flow cytometry analysis also confirmed and extended prior studies(38, 72, 77), showing that the proportions of both early and late intermediate cells increased, while the proportions of differentiated macrophages decreased in IBD and these alterations correlated with disease severity. Whether intermediate cells accumulating in IBD are transcriptionally similar to those present in the healthy intestine or, as suggested in mice(42), acquire a distinct transcriptional profile because of local inflammatory cues, awaits further study.

The intestinal LP compartment contained cDC1 and cDC2 as well as cDC that expressed both cDC2 and mono/mac-associated genes which, in line with previous studies(33, 78), we termed cDC3. Our trajectory analysis suggested phenotypic convergence between cDC2 and cDC3 within the intestinal LP, and we were unable to distinguish some of these cells transcriptionally and using surface markers. However, our CITE-seq analysis demonstrated that CD207^+^CD11a^-^ CD1c^+^ cells were highly enriched in cDC2, while CD207^-^CD11a^+^CD1c^+^ cells were enriched in cDC3. Using these markers, we found that cDC3 were present in higher proportions in the colon compared with the ileal LP, while cDC2 showed the opposite pattern. Notably, intestinal cDC3 displayed a unique proinflammatory transcriptional profile compared both to other cDC and to differentiated macrophage subsets, suggesting they play distinct roles in intestinal immunity.

Previous studies in mice have demonstrated some functional differences between small intestinal and colonic cDC(79–81), including expression of the alcohol dehydrogenase *aldh1a2* and associated enhanced ability to generate retinoic acid by small intestinal cDC(80, 81). While this difference was not observed among human cDC, some site-specific differences were observed, particularly within the cDC2 compartment. Thus, colonic cDC2 showed evidence of enhanced NF-kappa B signalling, cytokine production, and responsiveness to cytokines. Conversely, ileal cDC2 were enriched for cholesterol transport and biosynthesis. Thus, cDC2 seem particularly sensitive to fine-tuning by local environmental signals.

Lineage-restricted cDC precursors have been identified in human blood, bone marrow and lymphoid tissues(25, 29, 48, 59–61), but it has been unclear whether such precursors exist in human non-lymphoid peripheral tissues, including the intestine. Here, our combined bioinformatic analyses provide evidence that both the human ileal and colonic LP contain cDC precursors that appear committed to either the cDC1, cDC2 or cDC3 lineage. Thus, we found that each mature cDC subset was directly connected in trajectory space to a distinct population of proliferating *HLA*^low^*ITGAX*^low^ cells. Secondly, these distinct proliferating populations displayed a unidirectional velocity-based developmental trajectory into either mature cDC1, cDC2 or cDC3. Finally, these putative lineage-restricted precursors displayed a progressive acquisition or loss of cDC lineage-associated marker genes and TFs as they transitioned towards each mature cDC subset. As expected, the number of these putative lineage-restricted cDC precursors was low and our ability to capture such cells was only made possible by our sorting strategy and use of surgical resections as opposed to biopsies. Our evidence of lineage-restricted cDC precursors in the human intestinal LP is consistent with recent studies indicating the presence of cDC1 and cDC2 restricted cDC precursors in the murine small intestine(62), and that cDC3 derive from distinct precursors to those of cDC1 and cDC2(27, 29, 48, 82).

Antonova *et al*. recently identified a *PRDM1*^+^RORgt DC-like cell which gave rise to cDC2-like cells *in vitro* and developed the ability to prime T cells(67). Remarkably, we found that the signature score of these RORgt DC-like cells selectively overlapped with cDC precursor cluster 6, located early in our putative cDC2 trajectory, suggesting that similar cells act as cDC2 precursors in the intestine. In mice, RORgt^+^ antigen presenting cells play an important role in the peripherally Treg induction to microbial and food antigens(83–88). While the origin of these cells in mice has been the subject of some debate, recent studies suggest they are potentially related to cDC2(86), although this remains to be confirmed.

In addition to putative lineage-restricted cDC precursors, we also identified a minor population of proliferating *ITGAX* expressing *HLA*^low^ cells that did not show transcriptional bias towards any particular cDC lineage. The transcriptional profile of these cells instead correlated best with early bone marrow precursors, indicating that these cells may lie upstream of lineage-committed cDC precursors. While such findings are consistent with the observation that haematopoietic stem cells and/or downstream myeloid precursors are present in the human intestine(89), the lineage potential and role these cells play in maintaining the intestinal MNP compartment awaits further study.

We found that the proportion of cDC2 was significantly reduced in the inflamed ileal LP of CD patients while that of *CCR7*^+^ cDC was increased. These *CCR7*^+^ cDC bore a similar transcriptional profile to migratory and mREG cDC, a population present in diverse contexts including tumors and rheumatoid synovium(26, 50, 71, 90). Further, while *CCR7* induction is usually associated with cDC migration to draining lymph nodes, clusters of LAMP3^+^CCR7^+^ cDC were readily detected in in the inflamed LP of CD patients in intimate association with T cell aggregates, some of which were proliferating, indicating a potential role in regulating local T cell responses. Such findings support previous studies showing *CCR7*^+^ cDC in the inflamed LP of a subset of IBD patients, associated with resistance to anti-TNF therapy(68).

While tumor-associated *CCR7*^+^ cDC derive from cDC1 and cDC2, our trajectory and CITE-seq analysis suggested that *CCR7*^+^ cDC originated from cDC2 and potentially cDC3, but not from cDC1. Furthermore, we found two developmentally related *CCR7*^+^ cDC subclusters, with more-inflammatory *CCR7*^+^ cDC cluster giving rise to *CCR7*^+^ cDC enriched in T cell activation pathways. Similar findings have been described recently in the synovial tissue of RA patients, where cDC2-derived *CCR7*^+^ cDC were also found associated with lymphoid-like clusters(71), indicating a common immunological niche that develops in both diseases.

In summary, our single-cell data highlight marked heterogeneity in the MNP compartment of the human intestinal LP, varying along the length of the human intestine and in the setting of disease. Additionally, by identifying novel transcriptomic and phenotypic markers, our work provides a road map for the study of MNP subsets, and their contribution to intestinal immune responses in health and disease.

## Materials and Methods

### Study Design

The main objectives of this study were to determine (1) MNP heterogeneity within the human intestinal LP, (2) whether precursors to mature MNP subsets were present in the intestine, (3) whether the proportions and transcriptional profiles of mature MNP subsets differed between human ileum and colon, and (4) inflammation induced changes in the intestinal MNP compartment. Our hypothesis was that multi-modal single-cell methods (scRNA-seq, CITE-seq, flow cytometry) combined with bioinformatic analysis would allow for the unambiguous identification of MNP subsets within the intestine. Patient material included tissue from surgical specimens from (a) colorectal cancer patients (>10 cm from tumour site), (b) CD patients undergoing surgery for disease relief and (c) biopsies from treatment-naïve patients undergoing endoscopy for suspected IBD. Patients below 18 or above 85 years of age were excluded from the study. Surgical samples were only used when it was possible to readily dissect mucosa from submucosa. Each experiment was replicated in at least three patients unless otherwise specified. No sampling replication was performed within an individual patient due to limited tissue availability.

### Methods

#### Human Subjects

Resection samples were obtained from patients undergoing surgery for colorectal cancer and from patients with CD without co-morbidities such as infectious or neoplastic diseases undergoing surgery for disease relief (see Table S1 and S8 for further patient information), after informed consent with ethical approval from the Scientific Ethics Committee of the Copenhagen Capital Region, Denmark (H-3-2013-118, H-20054066). Biopsy samples were obtained from adult patients at the time of their first colonoscopy, during which the diagnosis of IBD was established (both CD and UC), or for ongoing disease assessment (see Table S4 for further patient information) at the Western General Hospital, Edinburgh, UK, after informed consent under existing approvals (REC:19/ES/0087). All patients were part of the Lothian IBD registry(91) and a diagnosis of IBD was made using the Lennard-Jones criteria(92). Endoscopic assessment of disease severity at each biopsy site was made at the time of endoscopy (Simple Endoscopic Score for CD, SES-CD(93), or Mayo endoscopic sub-score(94)) and biopsy sites were classified as quiescent, mild, moderate or severe based on the above scoring (See Table S4 for more information). Two to four biopsies were taken per site and pooled for analysis. Further clinical information for biopsy samples were collected under the above approvals (REC:19/ES/0087), and included blood parameters (CRP, Haemoglobin, Albumin), stool biomarkers (faecal calprotectin) and drug treatment that were obtained +/- 2weeks of the date of endoscopy (see Table S4).

### Method Details

#### Tissue processing

Surgical samples were processed as described previously (23). Muscularis externa was removed using curved surgical scissors and the remaining tissue was incubated in RPMI-5 (RPMI/5% FCS/1% penicillin and streptomycin) containing 4 mM DTT for 2 × 10 min at 37°C on a shaking incubator (370 rpm) to remove mucus. Macroscopically visible submucosa (SM) was trimmed away using scissors and mucosa separated from SM under a stereo microscope using forceps.

Epithelial cells were removed by incubating the mucosa in Ca2^+^ and Mg2^+^ - free HBSS containing 1% penicillin and streptomycin and 5 mM EDTA at 37°C for 10 min in a shaking incubator, and this procedure was repeated four times. Isolated lymphoid follicles were dissected from the mucosa using a scalpel under a stereo microscope with a transmitted light source, and remaining GALT-free LP was cut into 2-4 mm^2^ pieces in preparation for digestion. LP was incubated in RPMI-5 containing DNase1 (30 μg/ml) and collagenase D (5 mg/ml) or Liberase TM (2.5 mg/ml) for 45 min at 37°C under gentle shaking (370 rpm). The resulting LP cell suspension was passed through a 100 μm filter and washed twice in fresh RPMI for downstream analysis. Biopsy samples were processed using the same protocol without peeling away the submucosa but with any visible follicles removed.

#### Flow cytometry, MNP enrichment and cell sorting

Cell suspensions were stained with indicated antibodies in Brilliant stain buffer (BD Biosciences) containing 4% normal mouse serum, according to standard procedures, with dead cells identified by 7-AAD staining and excluded from analysis. Samples were analyzed on a LSR Fortessa 2 (BD Biosciences) using Flowjo software (BD). The Legendscreen assay (Biolegend) was performed as per the manufacturer’s instructions. For scRNA-seq experiments, LP cell suspensions were either enriched for HLA-DR^+^ cells using anti-HLA-DR microbeads (Miltenyi Biotec) and LS MACS columns according to manufacturer’s instructions or processed further as total single cell suspensions without enrichment. Resulting cells were stained with the indicated antibodies (Table S12) and 7-AAD or LIVE/DEAD Fixable Far Red Dead Cell Stain Kit (Thermofisher) was used to exclude dead cells. Cells were sorted on a FACSMelody or FACSAria Fusion (BD) for subsequent scRNA-seq. For CITE-seq analysis, cells were stained with barcode-labelled TotalSeq-A antibodies (Table S12) or TotalSeq-C Human Universal Cocktail V1.0 (Biolegend) according to manufacturer’s instructions prior to sorting.

#### Immunohistochemical analysis

Formalin-fixed paraffin-embedded tissues were sectioned (4 μm) and deparaffinized. Antigen retrieval was performed by incubating slides in IHC-Tek Epitope Retrieval Solution (IHC World) for 30 min in a steamer. Sections were incubated with primary antibodies (Table S12) and DAPI (Thermo Fisher) in staining buffer (PBS containing FCS (2%), Tween 20 (0.1%) and ProClin 200 (0.05%)) overnight, washed in PBS containing Tween 20 (0.2%) for >4 h, and then with secondary antibodies (Table S12) overnight in staining buffer. After washing, slides were imaged using LSM900 confocal laser microscope with a Plan-Apochromat 20x/0.8 objective and the Zen Blue 3 software (Zeiss). Images were processed with Imaris Viewer v10.1.1 (Oxford Instruments).

#### 10x Chromium and sequencing

Freshly isolated sorted single cells were subjected to droplet-based massively parallel scRNA-seq using the Chromium Single Cell 3’ Reagent Kits v2, the Chromium Single Cell 3′ Reagent Kit v3.1 with Feature Barcoding technology for Cell Surface Protein or the Chromium Next GEM Single Cell 5’ Reagent Kits v2 (Dual Index) with Feature Barcode technology for Cell Surface Protein & Immune Receptor Mapping (10x Genomics) following the manufacturer’s instructions. For CITE-seq analysis, 5 μl of the purified smaller cDNA product was used as template for antibody-derived tag (ADT) sequencing and ADT sequencing libraries were constructed and indexed following the manufacturer’s instructions. Quality and quantity of the final libraries were measured using the Agilent 2100 Bioanalyzer equipped with High Sensitivity DNA chip (Agilent). Illumina sequencing was carried out at the Genomics Core Unit, Center of Excellence for Fluorescent Bioanalytics (University of Regensburg, Germany), at the SNP&SEQ Technology Platform, Sweden or at the Core Facility for Flow Cytometry and Single Cell Analysis, Faculty of Health and Medical Sciences, University of Copenhagen. Libraries were sequenced using HiSeq, NextSeq and NovaSeq systems (300 cycles), aiming for a minimum of 30,000 read pairs/cell for sc-RNA and 3000 read pairs/cell for ADTs.

#### Bioinformatic analysis

##### Data processing

Sequencing data was pre-processed and aligned with 10x Genomics Cell Ranger (version 2.2.0, 3.1.0, and 8.0.0)(95, 96). Sequencing data from samples stained with TotalSeq antibodies was processed with CITE-seq count(97). Each sample was read into a Seurat (version 3.1.5/5.0.1/5.2.1)(98) object in R (versions 3.5.1/4.0.1/4.3.0/4.3.1)(99) and processed by removing cells with exceptionally low or high UMI, gene counts (< 500-1000 and > 3000-6000 genes/cell) and mitochondrial gene content (>10%) according to current best practice(100) and likely representing debris and doublets. For CD samples, we predicted and removed doublets using scDblFinder(101) (version 1.16.0). To normalize CITE-seq data by denoising and scaling protein levels against background (DSB-normalization(102), the debris removed from each sample was used as empty droplet information (free floating CITE-seq antibody), while isotype controls were used to normalize for non-specific binding(102). The normalized protein data were incorporated with the RNA data for the individual samples by adding to it to the corresponding Seurat objects.

After log-normalization of RNA levels for individual samples, cell cycle gene modules were calculated using the Seurat CellCycleScoring function and variable genes were identified per sample. After initial data processing, all the samples were integrated either with Seurat anchor integration or harmony(103). Gene expression was subsequently scaled, regressing out the effect of cell cycle, UMI counts, and mitochondrial gene content based on their scoring on the individual samples except when combining data in Figure 7.

##### Identification of MNP subsets

To detect MNP subsets, a high-resolution clustering was initially performed which based on known monocyte-, macrophage-, and cDC2-associated genes split the clusters into three main groups. To validate these, we performed a PCA on the average expression of marker genes for subsets (“Macro”, “cMo”, “DC2/3” and “mReg”) published by Mulder *et al*(35).

##### Differential gene expression and gene ontology

Cluster markers were computed using Seurat’s FindAllMarkers function. Differential gene expression tests between specific cell subsets and across conditions were performed by pseudobulk analysis with DESeq2(104) (version 1.38.3/1.42.1) using Seurat’s AggregateExpression function. Gene ontology analysis was performed based DEG lists of above (padj < 0.05) and run on EnrichR’s web interface(105). The output tables based on GO Biological Processes 2023/2025 were downloaded and plotted in R using the package ggradar (version 0.1).

##### Comparisons to public datasets

Module scores were calculated with Seurat’s AddModuleScore function on indicated selected gene sets or gene sets from indicated literature. For analysis of the datasets from Triana *et al*(65), the processed Seurat object was downloaded and subsetted based on the clusters labelled “HSCs & MPPs”, “Lymphomyeloid prog”, “Early promyelocytes”, “Conventional dendritic cell 1”, “Conventional dendritic cell 2”, “Late promyelocytes”, “Myelocytes”, “Classical Monocytes”. Gene expression data was averaged, and Pearson correlations calculated based on variable genes from our data also present in the bone marrow dataset.

##### Signaling pathway analysis

DoRothEA was run using confidence levels A+B and referring to the human DoRothEA transcription factor interaction database(57). Progeny was run using organism = human and top = 500 genes(58).

##### Trajectory inference

Trajectory inference was performed with tSpace on PC spaces of indicated populations(44). The outputs were dimensionality reduced with UMAP(106, 107) from tPCs 1-15 (for both cDCs and macrophages), to 2 and 3 dimensional tUMAPs with distance metric set to Pearson. 3D tUMAP for the tSpace trajectories were angled and embedded into 2D. For CRC samples, clustering was performed with Louvain clustering for Seurat on the tPCs as input. Pseudotime (arbitrary time scale unit) was calculated by taking all trajectories from the tSpace output from M1 and averaging these per cell. Splicing patterns were first determined on individual samples with the advanced run setting for velocyto(64) with a repeated annotation file(108). Genes were filtered by 0.2 for spliced data and 0.05 for unspliced data. RNA velocity estimates were then calculated for T=1 and only included genes with splicing information also present in the variable genes and only on cells of interest (e.g. precursors)(109). The information was embedded on top of 2D tUMAP using n=400, scale=sqrt, grid.n=50 and arrow.scale=2.

#### Quantification and statistical analysis

Statistical analysis of flow cytometry data was performed using Prism software (GraphPad). Statistical analysis of sequencing data was performed in R. Statistical tests used for experimental data are outlined in the figure legends.

## Supplementary Material

Supplementary tables

Video S1

Video S2

Supplementary Figures

## Data Availability

scRNA-seq count data is available through CZ CELLxGENE: https://cellxgene.cziscience.com/e/bcdec5fa-a7fa-4806-92bc-0cd02f40242f.cxg/. Code is available: https://github.com/LineWulff/FentonWulff_LP_MNP. All other data will be made available upon reasonable request to the authors.

## One sentence summary

Fenton, Wulff and Väänänen *et al*. use single-cell methods to explore the complexity of the mononuclear phagocyte compartment of the human intestinal lamina propria in health and IBD, identifying distinct dendritic cell and macrophage subsets, lineage-specific precursors and site and state specific transcriptional signatures.
