# Supplementary material for: High-dimensional profiling uncovers heterogeneity, lineage-specific precursors and inflammation-induced changes in the mononuclear phagocyte compartment of the human intestine
Source: Sci Immunol. Author manuscript; Available in PMC 2026 Jul 10. (PMC7619228; doi:10.1126/sciimmunol.adz8650)

List of Supplementary Materials

Supplementary materials include seven supplementary figures, two supplementary videos and twelve supplementary tables.

Supplementary Figures

Figure S1. Identification of intestinal LP cell types.

Figure S2. Bioinformatic analysis of intestinal LP macrophage populations.

Figure S3. Flow cytometry and CITE-seq analysis of monocyte/macrophage surface markers

Figure S4. Transcriptional characterization of intestinal LP cDC subsets.

Figure S5. Identification of surface markers that help differentiate intestinal cDC2 and cDC3.

Figure S6. Identification and trajectories of ileum and colon cDC precursor clusters.

Figure S7. Analysis of cDC subsets isolated from the ileal LP of CD patients.

Supplementary Videos

Video S1. Three-dimensional trajectory (t) UMAP of mono/mac clusters M1-M11

Video S2. Three-dimensional trajectory (t) UMAP of cDC clusters identified in **Fig. 7G.**

Supplementary Tables

Table S1. Characteristics of CRC patient samples used for single-cell RNA-seq.

Table S2. Complete list of DEG between differentiated macrophage clusters M6-8 using combined data from the ileum and colon.

Table S3. Complete list of DEG between the ileum and colon for clusters M6-M8.

Table S4. Characteristics of IBD patients biopsied for flow cytometry analysis.

Table S5. Complete list of DEG between cDC1, cDC2, and cDC3 using combined data from the ileum and colon.

Table S6. Complete list of DEG between cDC3 and macrophage clusters M6-M8 using combined data from ileum and colon.

Table S7. Complete list of DEG between the ileum and colon for cDC1, cDC2 and cDC3.

Table S8. Characteristics of CD patients used for scRNA-seq.

Table S9. List of genes and associated GO terms expressed at a significantly higher level in $CCR7^+$ cDC compared with other cDC subsets.

Table S10. List of DEG between the two subclusters of $CCR7^+$ cDC.

Table S11. List of DEG between more and less inflamed regions of ileum from CD patients for each of the cDC subsets.

Table S12. List of antibodies used for flow cytometry, CITEseq and immunohistochemical analysis.

Supplementary Materials

Figure S1. Identification of intestinal LP cell types. (**A** and **B**) UMAP depicting scRNA-seq data of enriched ileal and colonic LP HLA-DR$^+$ cells (42,506 cells) isolated using the pipeline depicted in Fig. 1A. (**A**) Examples of signature gene expression used to identify contaminating T cells, B cells, endothelia, mast cells, stromal cells, and glial cells and (**B**) HLA score based on indicated HLA genes. (**C**) Relative abundance of high resolution MNP clusters in ileal and colonic LP samples. Related to Figure 1.

Figure S2. Bioinformatic analysis of intestinal LP macrophage populations. (**A**) Proportions of indicated clusters in paired ileal and colonic LP samples. **(B)** Pseudo-bulk heatmap of scaled gene expression of top 10 DEG (ordered by avg. logFC) between M1-M11 clusters. (**C**) Venn diagram displaying number of DEG between M6 and M8. (**D**) Signature score (top 50 DEG) of indicated cluster embedded onto a UMAP of a colonic mucosal macrophage dataset from Domanska *et al.*(*18*). Related to Figure 2.

Figure S3. Flow cytometry and CITE-seq analysis of monocyte/macrophage surface markers. (**A**) Representative flow cytometry analysis showing pre-gating for CD14$^+$ MNP. (**B**) Flow cytometry-based expression of indicated markers on CD14$^+$ MNP using Legendscreen. Grey fill, FMO. Red line, specific antibody stain. (**C**) DSB-normalized CITE-seq expression of indicated surface markers on ileal LP macrophage clusters after exclusion of the minor proliferating M11 cluster based on n=1. Related to Figure 2.

Figure S4. Transcriptional characterization of intestinal LP cDC subsets. (A) 2-dimensional representation of a 3-dimensional tSpace UMAP (tUMAP) of bioinformatically isolated and re-clustered cDC with high resolution Louvain clustering (52 clusters) and split into ileal and colonic LP. (**B**) Cell cycle profile (**C**) HLA score based on indicated HLA genes. (**B** and **C**) Dashed line represents clusters enriched in cells in G2M/S phase and with low HLA score. (**D**) cDC1 score based on cDC1 signature genes (*CLEC9A, CADM1, XCR1, BATF3,* and *IRF8*) by pseudo-bulk cDC clusters (44 clusters) after removal of the proliferating and HLA^{low} clusters in **B** and **C**. Dashed line indicating cDC1 identity, threshold cDC1 score > 0.4 for cDC1 identity. (**E**) Ranked expression of cDC2 and cDC3 scores by remaining cDC clusters (37 clusters) using signature genes identified by Bourdely et al(*48*). Dashed lines indicating cDC2, cDC3 and ambiguous identities. Clusters were classified as cDC2 when cDC2 score > 0.4 & cDC3 score < 0.3 and as cDC3 when cDC3 score > 0.4. (**F**) tUMAP plots colored by expression of indicated genes associated with cDC maturation and migration. (**G**) Venn diagram displaying number of DEG between cDC subsets. (**H**) Pseudo-bulk heatmap of top 25 DEG expressed by indicated cDC subsets from ileal and colonic LP. Related to Figure 4.

Figure S5. Identification of surface markers that help differentiate intestinal cDC2 and cDC3. (A) Violin plots of CD14 and CD1c surface expression by colonic MNP subsets after denoising and scaling to background (DSB)-normalization of CITE-seq. Results are pooled from three colonic and one ileal LP samples. **(B**) Pre-gating strategy to identify colonic-LP CD1c^{+}CD14^{-} cDC2, ambiguous and cDC3. (**C)** CD207 and CD11a expression as assessed by DSB-normalized

CITE-seq, Q, quadrant and (**D**) relative proportions of cDC2, cDC3 and ambiguous cDCs within the four CD207 and CD11a CITE-seq quadrants (**C**) in indicated tissue from a single CRC resection patient. Related to Figure 5.

Figure S6. Identification and trajectories of ileal and colonic cDC precursor clusters. (**A**) RNA velocities (arrows) of HLA-DR^{low} cDC clusters 3-5 and 7-8 split into ileum and colon derived cDC clusters and calculated with Velocyto package and embedded on Fig. 4A. (**B**) Location of indicated clusters not identifiable using DEG for mature cDC split into ileum and colon (Fig. 4D) on a PCA plot of clusters identified by shared DEG as either pre-cDC1, pre-cDC2 or pre-cDC3 (see Fig. 4F). (**C**) Heat map depicting Pearson correlation of each intestinal putative pre-cDC cluster and *CD14*$^{+}$ monocytes with indicated progenitor populations from BM described in Triana *et al*. (*65*). (**D**) Expression of individual genes or (**E**) score of indicated ILC3 progenitor associated genes from Antonova *et al*(*67*) embedded on intestinal cDC tUMAP. Related to Figure 6.

Figure S7. Analysis of cDC subsets isolated from the ileal LP of CD patients. (**A**) UMAP of intestinal cDC scRNA-seq datasets from combined CRC and CD samples, depicting the cDC labels derived from the CRC samples. (**B**) cDC subset score comprising of the indicated genes embedded on cDC UMAP. (**C**) scRNA-seq or CITE-seq expression of the indicated markers on each cDC subsets isolated from more (I) inflamed or (U) less inflamed regions of ileum from CD patients. Data are pooled from 6 (scRNA-seq) or 1 (CITE -seq) individual patients per sample. (**D**) Heat map displaying expression of selected genes previously shown by Maier *et al.*(*50*) to be

differentially expressed in CCR7$^+$ cDC. (**E**) UMAP of bioinformatically isolated CCR7$^+$ cDC cluster divided into two main subclusters. (**F**) Proportion of CCR7$^+$ subcluster 1 and 2 amongst total CCR7$^+$ cDC in indicated tissue sample. CD, Crohn's disease. (**G**) GO analysis showing selected pathways differentially upregulated in the two CCR7$^+$ cDC subclusters. (**H**) *MIR155HG*$^+$ cDC (*IL1B*, *CXCL8*, *CCL3*, *CCL4L2*, *MIR155HG*, *TNFAIP3*, *NINJ1*, *NLRP3*, *GADD45B*, *IL1R2*, *CD1C*, *ICAM1*, *EHD1*, *NFKBIZ*, *CXCL3*) and *LAMP3*$^+$ cDC (*TXN*, *MARCKSL1*, *IDO1*, *FSCN1*, *LAMP3*, *EBI3*, *NUB1*, *RAB9A*, *RPS27L*, *POGLUT1*, *GPR157*, *IL32*, *PSME2*, *GPX4*, *MGLL*) module score from MacDonald *et al.*(*71*) embedded onto a Flat tUMAP of cDC. Related to Figure 7.

Supplementary Figure 1

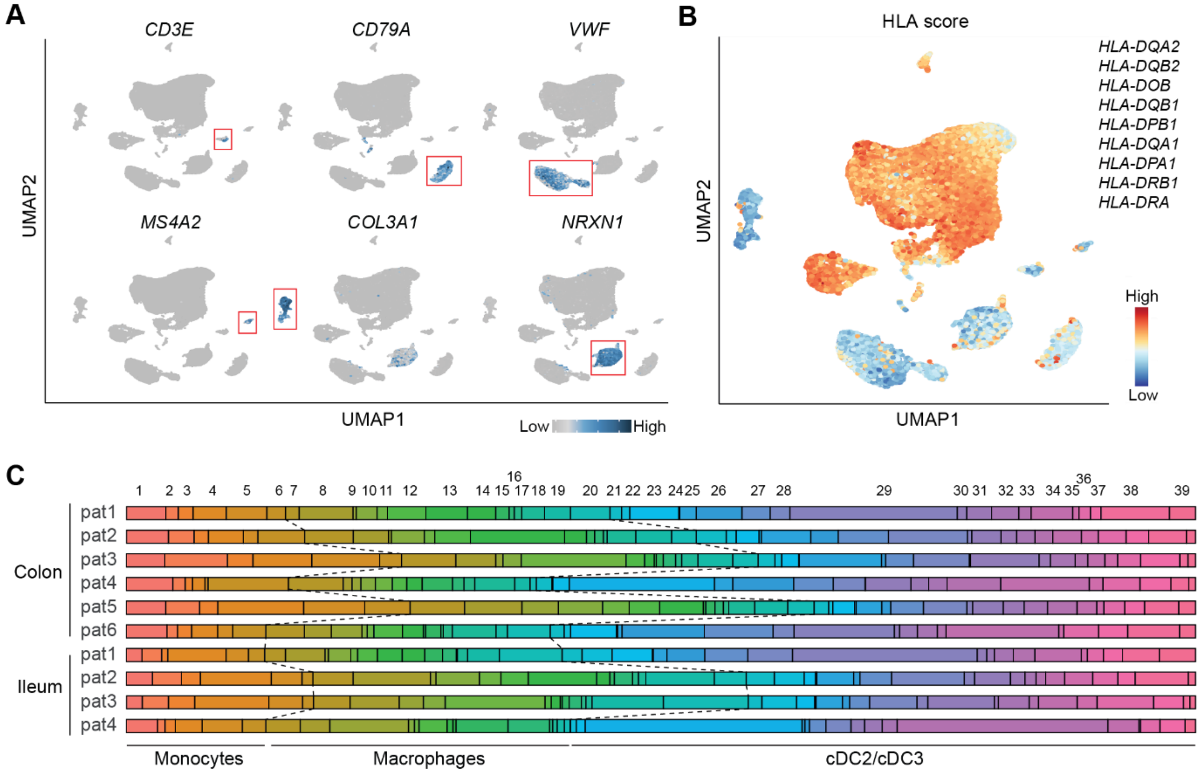

Supplementary Figure 2

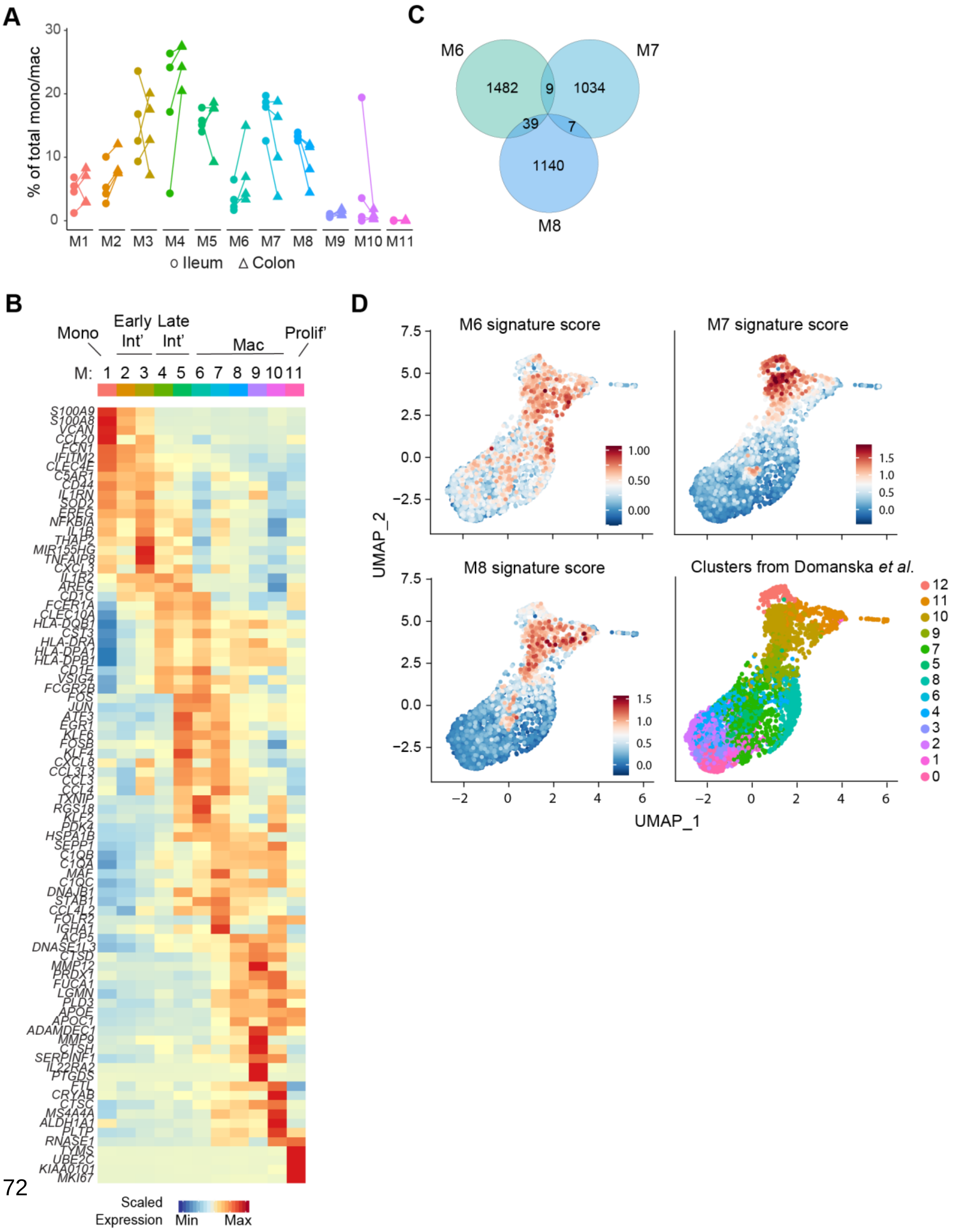

Supplementary Figure 3

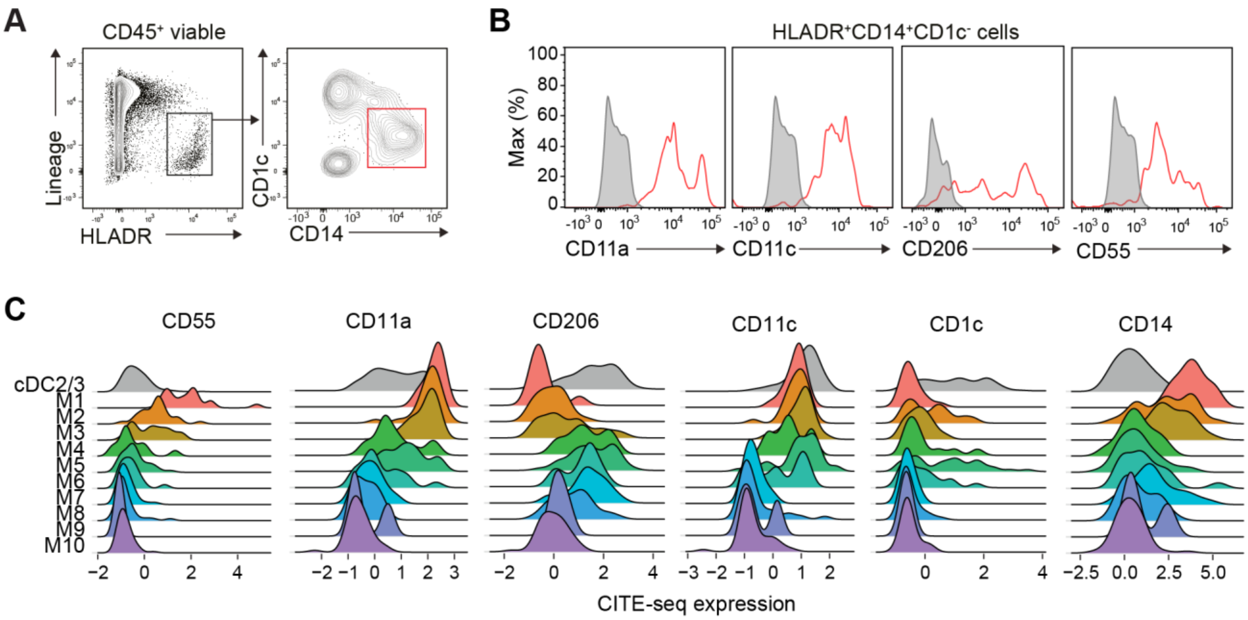

Supplementary Figure 4

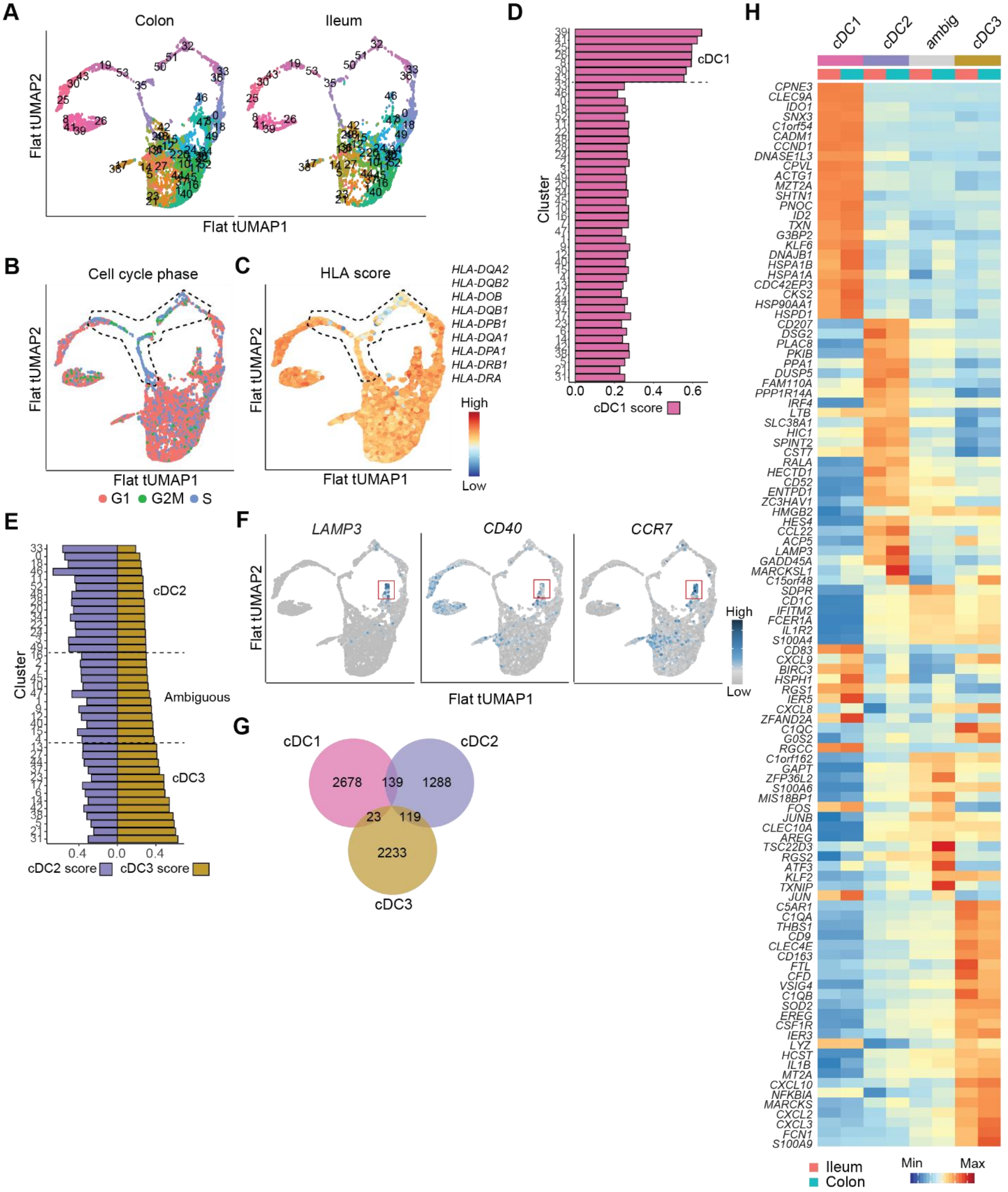

Supplementary Figure 5

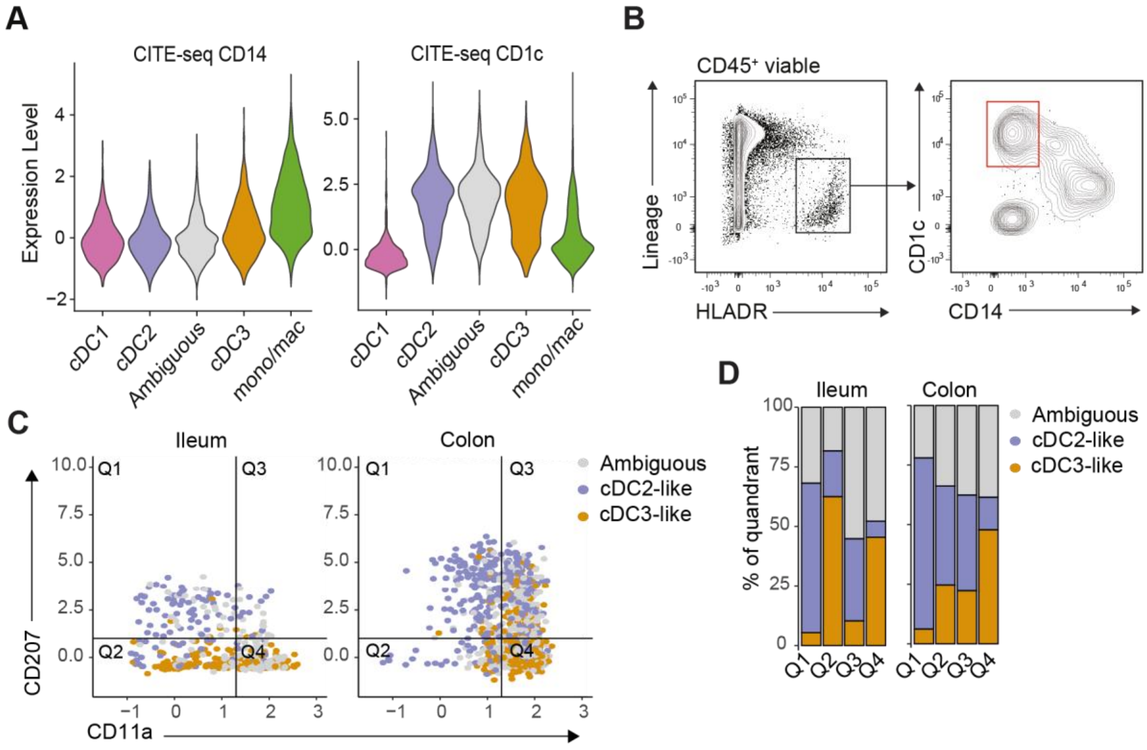

Supplementary Figure 6

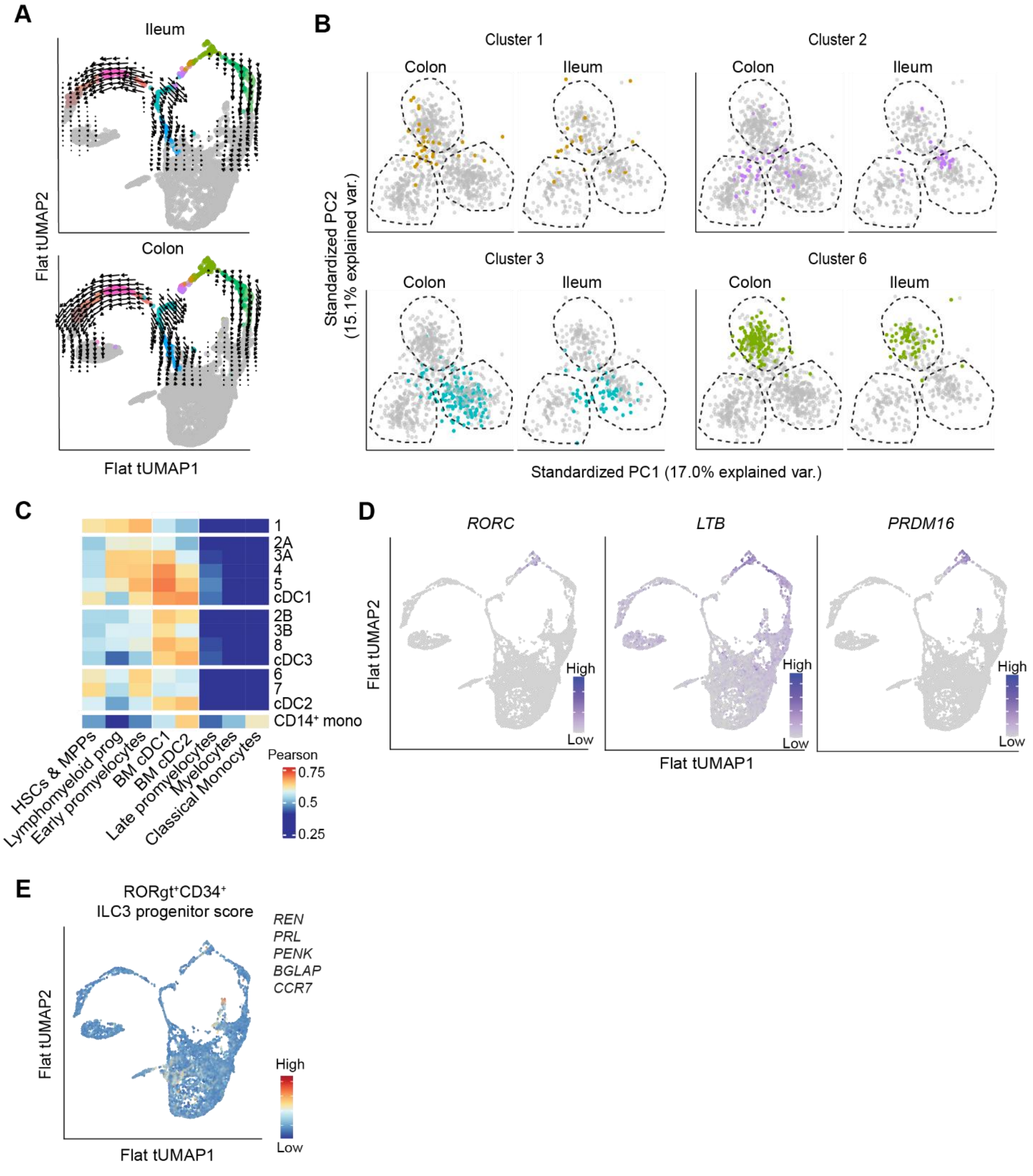

Supplementary Figure 7

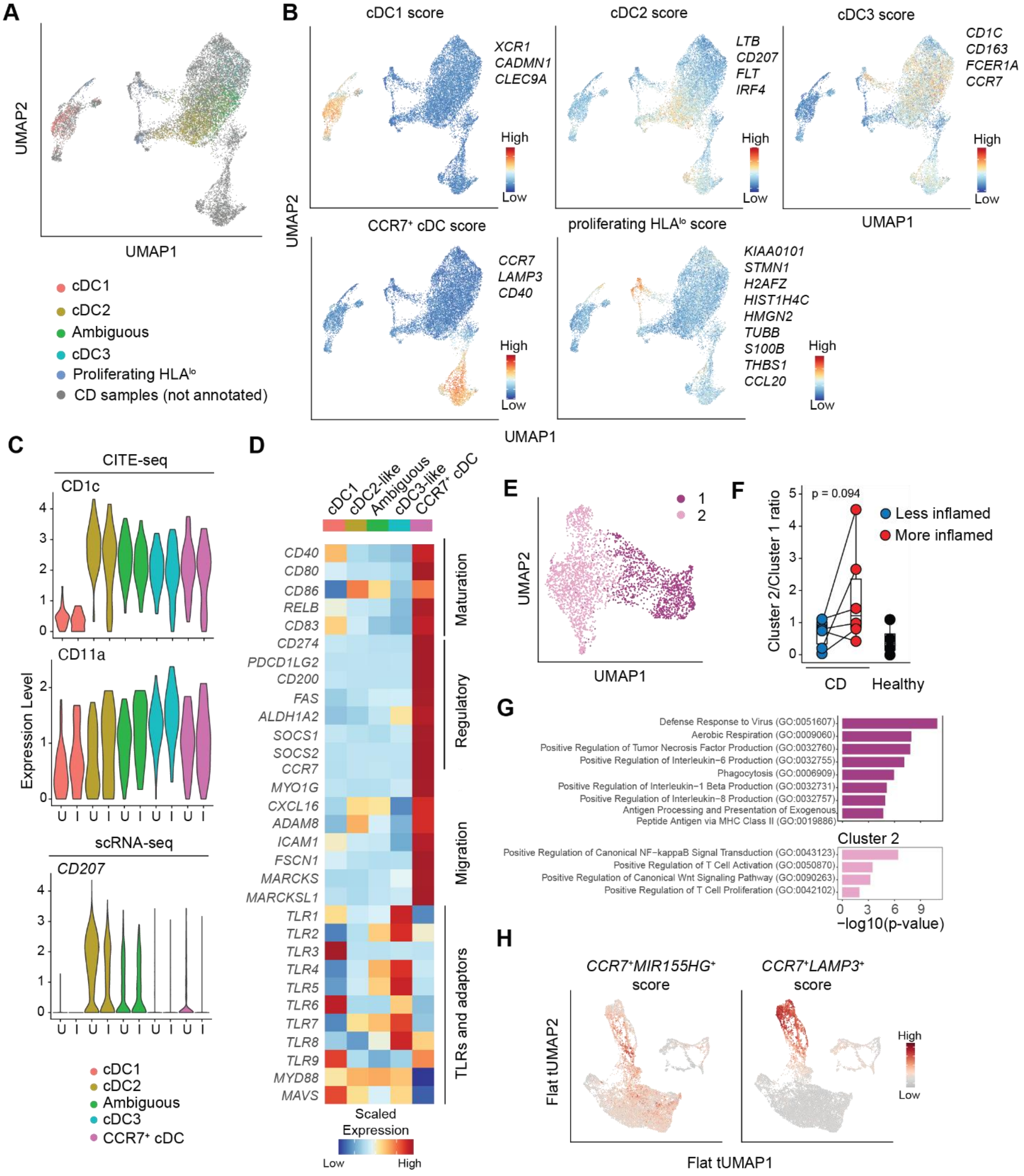

Supplement: Supplementary Figures [file EMS214277-supplement-Supplementary_Figures.pdf]
