# Supplementary material for: High-dimensional profiling uncovers heterogeneity, lineage-specific precursors and inflammation-induced changes in the mononuclear phagocyte compartment of the human intestine
Source: Sci Immunol. Author manuscript; Available in PMC 2026 Jul 10. (PMC7619228; doi:10.1126/sciimmunol.adz8650)
Supplement: Supplementary tables [file EMS214277-supplement-Supplementary_tables.pdf]

Table S1

Patient	Age	Sex	Diagnosis	Cancer stage	Site of colon resection	Site of SI resection	CITEseq
SC1	65-69	F	Adenocarcinoma	1	n.d.	Ileum	-
SC2	70-74	M	Adenocarcinoma	3b	Transverse	Ileum	-
SC3	60-64	F	Polyp	n.a.	Ascending	Ileum	-
SC4	75-80		Adenocarcinoma	2	Transverse	Ileum	Colon+Ileum
SC5	60-64	F	Polyp	n.a.	Ascending	-	Colon
SC6	80-84	M	Adenocarcinoma	3	Cecum	-	Colon

n.a = not applicable, n.d. = no data.

Table S2

gene	M6 vs. M7 + M8 log2FoldChange	padj
CCNL2	0.635913647	0.00182355
RP5-832C2.5	1.671802368	4.4449E-06
WRAP73	0.709662569	0.02937588
RPL22	0.149032623	0.01646671
TNFRSF8	1.54595326	0.02671469
RP3-467K16.2	3.308460996	0.00784836
DDI2	0.523482392	0.02248537
FLJ37453	0.984314849	0.00025741
RP4-798A10.7	4.234830017	5.6658E-09
RP11-108M9.4	0.693030205	0.00032106
RP11-108M9.6	3.405483407	9.8722E-07
KIF17	0.77989412	0.02195582
USP48	0.453019333	0.00518111
RPL11	0.268683771	0.00150635
TCEB3-AS1	1.742359176	0.00227417
HMGCL	0.760906417	0.000084318
RSRP1	0.584589723	1.9094E-06
PDIK1L	1.610466113	0.00160985
SH3BGRL3	0.532544987	0.00022163
UBXN11	0.703270767	0.00825968
CD52	1.266095781	0.0064121
HMGN2	0.244986051	0.0280362
RPS6KA1	0.722521314	3.8686E-06
SYTL1	2.697171034	0.0006392
FGR	0.957509839	0.00185172
RPA2	0.634576912	0.000051197
RP5-1092A3.4	1.624434395	0.02503649
EPB41	0.923364586	0.00192198
SNRNP40	0.638656825	0.00054446
RBBP4	0.383738968	0.00564778
PHC2	0.277514512	0.0480568
ZMYM4	0.504438657	0.04534293
AGO4	0.731988281	0.00228118
AGO1	0.892851801	0.00066716
ADPRHL2	0.642120285	0.00493776
LSM10	0.384347666	0.00680053
CSF3R	0.695245716	0.000054278
C1orf109	1.488082651	0.00035353
FHL3	0.873006641	0.00354936
MYCBP	0.881986881	0.00062414
PPIE	0.348670148	0.04968739
TRIT1	0.902275668	0.00008962
PPCS	0.311028861	0.00973071
CCDC30	1.397336432	0.04080482
ELOVL1	0.366000712	0.02724457
MED8	0.434615848	0.01360345
DPH2	1.109580503	0.01725675

gene	M7 vs. M6 + M8 log2FoldChange
HES4	0.454692518
SKI	0.84128086
TNFRSF25	2.523474476
PLEKHG5	3.04427521
RERE	0.594237472
RBP7	0.975282984
KIF1B	0.804711337
DHRS3	0.288485221
IGSF21	1.187516205
NBL1	0.930795649
C1QA	0.412376756
PNRC2	0.301566315
CLIC4	0.509808796
MAN1C1	1.3391431
PAQR7	0.841961185
ARID1A	0.535003924
WASF2	0.320988087
AHDC1	1.121270127
PHACTR4	0.31528269
SDC3	0.69532013
PUM1	0.394770216
SFPQ	0.353870464
ZC3H12A	0.740534839
PABPC4	0.519828626
RLF	0.358360215
SMAP2	0.385705361
PLK3	0.57626575
BTBD19	0.818536639
PIK3R3	0.763262608
NSUN4	0.782670714
MOB3C	0.621035249
EPS15	0.561177532
ZCCHC11	0.716444679
USP24	0.699140339
JUN	0.918045757
NFIA	1.140826037
LEPROT	0.220721545
WLS	1.285328083
DNAJB4	1.009987086
PRKACB	0.446488542
GTF2B	0.745895779
ZNF644	0.372003294
VCAM1	1.107451093
TAF13	0.4184197
GNAI3	0.29325733
GSTM3	0.833286142
DRAM2	0.453731939

RPS8	0.241416263	0.00529303	SLC16A1	0.588593202
RP11-117D22.2	2.568375356	0.00897268	HIPK1-AS1	1.428510703
TCEANC2	1.235649027	0.00288487	HIPK1	0.385640606
OMA1	0.817198248	0.0072	BCAS2	0.375433515
USP1	0.462116336	0.03921288	CSDE1	0.322381419
PTGER3	2.925975472	0.00025198	ATP1A1	0.285936278
ZRANB2	0.431936835	0.00500595	NOTCH2	0.516761844
USP33	0.508776094	0.00406917	NBPF26	0.788484839
ZNHIT6	0.640030924	0.02820825	FCGR1B	0.715509345
PKN2	0.498258603	0.00151268	SRGAP2C	0.515759844
LRRC8D	0.525126107	0.03742436	SRGAP2B	0.44492594
RPAP2	1.056870062	0.000087348	NBPF10	0.852839626
EVI5	0.404386314	0.01212669	NBPF14	0.559855426
RPL5	0.322629755	0.00024099	NBPF19	0.555375141
MTF2	0.849846685	7.3586E-08	MCL1	0.610325269
DR1	0.790959566	0.000015864	CDC42SE1	0.410332
PTBP2	0.870906537	0.03733568	MLLT11	0.936371766
DPYD	0.398333152	0.02720882	POGZ	0.381510591
TRMT13	0.778236635	0.00936557	UBAP2L	0.358120163
RNPC3	0.617497403	0.00303464	SHE	0.713753546
HENMT1	0.972660737	0.0050974	LMNA	0.888877956
PRPF38B	0.769292902	6.0561E-11	SEMA4A	1.315292162
RP5-1065J22.8	1.57237662	0.000056688	SLC25A44	1.643862299
PSMA5	0.217187412	0.0469496	MEF2D	0.50152003
CD53	0.301403206	0.000089682	ETV3	0.623502269
WDR77	0.689109921	0.00462157	TAGLN2	0.267172029
C1orf162	0.797379828	1.7533E-07	CD84	0.334447503
DDX20	1.10891107	0.00865136	FCGR2A	0.507378888
RHOC	0.705971043	0.00103985	ATF6	0.86035191
SLC16A1-AS1	0.753116185	0.04720429	OLFML2B	1.096136982
SLC22A15	1.500241773	0.02820825	RCSD1	0.334125115
CD101	0.662117645	0.03173876	SLC19A2	1.016519114
GDAP2	0.535383	0.04309565	ABL2	0.944647417
WDR3	0.762411822	0.02594376	GLUL	0.32549248
RP11-289H16.1	3.117367356	6.435E-07	RGL1	0.922125101
TXNIP	1.658790604	1.1132E-20	IVNS1ABP	0.495023334
PRKAB2	1.27600161	0.00652612	PTGS2	1.151121771
LINC01138	1.772724349	0.02405047	RGS1	0.557608895
CH17-189H20.1	0.572694228	0.00533227	BTG2	0.881094768
RP11-196G18.23	2.730050254	0.00526449	ZC3H11A	0.499410802
PLEKHO1	0.561501978	0.000054948	PPP1R15B	1.001962233
GABPB2	0.597321915	0.04259561	MDM4	0.238357666
TNFAIP8L2	1.187081924	2.0019E-08	SLC41A1	1.2434394
SCNM1	0.655311055	1.5427E-06	SRGAP2	0.402894417
VPS72	0.595995103	0.00973071	RP11-343H5.6	1.807572295
RP11-126K1.6	0.972874471	0.00125601	DYRK3	1.02104895
S100A6	0.92227716	0.00014252	MAPKAPK2	0.575910477
S100A4	0.920590766	0.000084112	IL10	0.764832372
INTS3	0.909919646	0.00122662	IL20	1.434620282
GATAD2B	0.748892762	0.00058228	CD55	0.703651527

RP11-422P24.12	1.400594059	0.02271301
RP11-422P24.11	2.276449712	0.0476649
RPS27	0.254937612	0.0009374
HAX1	0.964949251	1.4842E-07
PMVK	0.473507276	0.000070314
RP11-307C12.13	3.424952805	1.5634E-08
PYGO2	0.977098466	0.03275801
CKS1B	0.515291391	0.01772642
FLAD1	0.799094548	0.00558942
GON4L	0.535077322	0.00130337
CD1D	1.30731756	2.5938E-10
RP11-404O13.5	1.367937372	0.00063163
CD1C	1.810463776	1.6511E-06
CD1E	1.673055531	0.000015635
MNDA	1.268452337	1.2623E-15
IFI16	0.270406091	0.03037167
AIM2	1.164853321	0.00048784
FCER1A	1.581312704	5.5503E-07
PIGM	1.33378455	0.00056334
CD244	0.848254397	0.04986464
USF1	1.163839055	0.000077477
ARHGAP30	0.924690363	1.9287E-13
NIT1	0.888928242	0.000076729
NDUFS2	0.243665111	0.02516902
FCGR2B	0.80155769	0.00964128
RP11-474I16.8	1.503097255	0.00819211
UHMK1	0.403804909	0.00437139
RCSD1	0.404028737	0.00414482
SFT2D2	0.539802346	0.000058014
TBX19	1.263874516	0.02214799
RP1-206D15.6	2.255536637	0.00070607
SELL	0.922589719	0.01285174
SCYL3	1.23763597	0.0029504
CENPL	1.42696402	0.00673803
ZBTB37	1.687521384	5.3904E-06
KIAA0040	1.227827085	0.00022745
RP11-533E19.7	2.036898692	0.01627353
RP11-533E19.5	1.688466968	0.00844393
NCF2	0.407402203	0.00065591
EDEM3	0.648008328	0.00572854
FAM129A	0.68719894	0.02207578
RNF2	0.479033549	0.0393371
PACERR	1.472441576	0.0073795
RGS18	0.900309353	2.0284E-06
RGS2	0.352319976	0.04500251
PTPRC	0.235602002	0.00901486
RP11-134G8.5	1.830084365	0.00135208
RNPEP	0.349387344	0.00474764
SNRPE	0.226569759	0.03156772
MDM4	0.639902183	2.1803E-06

CD46	0.331310051
PLXNA2	1.592697211
ATF3	1.054469769
FAM71A	1.357316582
TATDN3	0.341026657
DUSP10	0.484117091
AIDA	0.325152816
FAM177B	1.563695327
TLR5	0.659160702
WDR26	0.421220781
RHOU	0.926421012
FAM89A	1.207469969
SIPA1L2	0.484671235
IRF2BP2	0.556968631
ERO1B	0.646659107
HNRNPU	0.615930553
NLRP3	1.220313054
ID2	0.638144314
ADAM17	0.748337588
YWHAQ	0.241781791
PUM2	0.552172832
RHOB	0.693102563
ITSN2	0.236867964
NCOA1	0.512703186
EMILIN1	0.814857142
SLC4A1AP	0.445768074
FOSL2	0.669004085
RP11-373D23.3	1.636923751
PPP1CB	0.420691478
SPDYA	1.149073014
FEZ2	0.392404482
SRSF7	0.337808502
EPAS1	1.053440897
RHOQ	0.424969881
CALM2	0.198457236
PSME4	0.555358952
REL	0.516900346
PELI1	0.847758862
AC074289.1	0.565712019
PPP3R1	0.414057914
CNRIP1	1.108051303
ANTXR1	1.917863281
MXD1	0.477970945
EXOC6B	0.907221638
TET3	0.520095589
MAT2A	0.360150288
EIF2AK3	0.4930151
ADRA2B	2.290493166
DUSP2	0.949200739
REV1	0.451782191

ELK4	0.839551184	0.000010295	TGFBRAP1	0.660953767
SRGAP2	0.596144439	0.0001475	RANBP2	0.395372133
TRAF3IP3	1.514314637	5.2699E-10	MERTK	1.099082534
RP11-61J19.5	0.846819341	0.02248537	IL1A	1.194984173
BATF3	0.688943231	0.0001018	IL1B	1.255889771
HLX	0.762987446	0.01642309	PAX8	1.034760831
TAF1A	1.429401611	0.00636415	DDX18	0.383257461
SRP9	0.345423025	0.00436959	AC009404.2	1.336524897
C1orf131	0.497052699	0.04738964	GYPC	0.757696862
RP5-827C21.4	2.17766491	1.891E-08	BIN1	0.718487765
TARBP1	0.879436209	0.00033016	MGAT5	0.797138666
ARID4B	0.297072733	0.02594023	DARS	0.329897242
GGPS1	0.49591922	0.03319753	CXCR4	0.54724992
LYST	1.385143757	5.3916E-11	HNMT	0.277217041
HNRNPU-AS1	0.890379759	0.000097756	NR4A2	1.243300611
EFCAB2	0.896113781	0.01994003	BAZ2B	0.427973986
PGBD2	2.129751862	2.3546E-07	MARCH7	0.297177316
AC142528.1	1.778984002	0.02583287	CD302	0.536817545
RPS7	0.284425536	0.000086924	RBMS1	0.655641398
CPSF3	0.477897993	0.04274744	TANK	0.590785488
RN7SL832P	2.724676535	0.000076646	CYBRD1	0.662341867
PQLC3	0.41641305	0.00250641	NFE2L2	0.703144379
ATAD2B	0.606648142	0.01071427	SESTD1	0.98523971
C2orf44	2.074554159	0.02039734	CWC22	0.449568923
PTRHD1	0.359111766	0.00383174	COL3A1	1.591014752
CAD	1.142422141	0.02693432	SLC40A1	0.81328842
SNX17	0.487899058	1.7476E-06	STK17B	0.267398157
ZNF512	1.016646998	0.02295046	SF3B1	0.219677451
CCDC121	1.817690578	0.0014994	CLK1	0.45653026
MRPL33	0.484416679	1.0702E-06	CFLAR	0.606802067
MEMO1	1.170218586	0.00065116	BMPR2	0.428596953
RP11-563N4.1	2.537832989	0.03214124	RAPH1	1.297997933
NLRC4	0.790245565	0.03846747	NRP2	0.927959926
YIPF4	0.544161605	0.000074635	KLF7	1.335469899
CEBPZOS	0.545496759	0.00229067	METTL21A	0.637013658
PRKD3	0.766220994	0.00144882	CCNYL1	0.453071574
QPCT	0.92395868	0.00166907	KANSL1L	0.788625934
GEMIN6	0.728221655	0.02086904	TNS1	1.450465421
SLC8A1	0.406398457	0.00210014	ARL4C	0.541911977
HAAO	0.932547622	0.01024372	NEU4	0.814807183
PRKCE	0.86011645	0.0334175	BHLHE40	0.686117741
ATP6V1E2	1.080431071	0.01291399	THUMPD3-AS1	0.581355801
FOXN2	0.730784365	8.4778E-06	IRAK2	0.898834697
RP11-191L17.1	2.135982916	0.00130083	HRH1	1.257370432
RPS27A	0.166215589	0.02860141	SH3BP5	0.502245369
AHSA2	0.896146303	3.9192E-06	PLCL2	0.673043289
XPO1	0.41285009	0.04850091	NR1D2	0.531565539
RP11-568N6.1	2.355947984	3.9273E-07	SLC4A7	0.45506545
RP11-474G23.3	1.926547593	0.000029582	TGFBR2	0.48112835
ARHGAP25	0.77223939	0.00031562	CMTM6	0.291550792

TIA1	0.638943	0.0050019	CRTAP	0.559991656
CLEC4F	1.636355029	0.000037161	CSRNP1	0.880209541
CD207	1.523449615	0.01851246	CTNNB1	0.537957436
NAGK	0.391796848	0.00156315	TRAK1	0.534827073
MPHOSPH10	0.439908963	0.0399578	CCRL2	0.51157567
ZNF638	0.605007183	0.00025331	C3orf62	0.494336172
TPRKB	0.598944222	4.8324E-06	HYAL2	0.572492607
STAMBP	0.398466055	0.04217469	NISCH	0.73731542
DGUOK-AS1	0.794718343	0.04736623	STAB1	0.895695258
RP11-287D1.2	1.910896165	0.04549144	DCP1A	0.458305825
DOK1	1.153608021	1.4752E-08	SELK	0.90899104
POLE4	0.266678743	0.02359263	FAM208A	0.383892913
TMSB10	0.296948633	0.00023604	FRMD4B	0.809613089
ELMOD3	0.909238803	0.02663555	FOXP1	0.536993846
MRPL35	0.474645466	0.01886751	LINC00877	0.828526809
KRCC1	0.563007061	0.00444545	RYBP	0.3708867
AC062029.1	2.001278782	0.00026817	FILIP1L	0.732486977
MRPS5	0.364168633	0.01376363	SENP7	0.564805132
ZNF514	1.013607353	0.02248537	NFKBIZ	0.94060514
MRPL30	0.597360672	0.01709612	ZBTB20	0.771033666
AFF3	0.982767607	0.00631052	GOLGB1	0.382450767
RPL31	0.332638763	0.00251231	CCDC14	0.399859494
RP11-332H14.2	2.365385021	4.6545E-10	ITGB5	0.803864069
GCC2	0.592467921	0.00066763	ZNF148	0.353852187
SLC20A1	0.334394505	0.04534625	TPRA1	0.748203486
RP11-480C16.1	3.334891524	0.03132579	PLXND1	0.729696847
RP11-141B14.1	1.052036817	0.04868962	STAG1	0.442444452
NIFK-AS1	1.427064186	0.00035385	ZBTB38	0.528985256
NIFK	0.593132404	0.000061519	ATP1B3	0.835432773
RP11-395A13.2	0.915569845	0.00140616	TFDP2	0.61740161
CCDC115	0.579073907	0.000087601	U2SURP	0.233840115
PTPN18	0.39276735	0.00492062	SLC9A9	0.846828779
SPOPL	0.705742893	0.000013751	TSC22D2	0.577101346
ZEB2-AS1	0.677473126	0.02848117	SIAH2	0.512397275
RP11-605B16.1	2.641873767	5.6563E-06	TIPARP	0.941227049
GCA	0.423629249	0.0036473	CCNL1	0.721674516
DHRS9	1.82873767	1.455E-09	MLF1	1.049627359
PPIG	0.285608299	0.00742209	MFSD1	0.258683234
AC013461.1	1.071494917	4.8324E-06	KPNA4	0.258477965
GPR155	0.545031167	0.03262711	GOLIM4	0.365995052
ITGA4	0.393349616	0.00134911	SKIL	0.523543343
DNAJC10	0.457056701	0.01451922	RPL22L1	0.278835773
OSGEPL1	1.229039247	0.00672807	FNDC3B	0.303359335
ORMDL1	0.52371945	0.00225926	FXR1	0.269420617
PMS1	0.765745692	0.00789445	B3GNT5	0.410315053
SLC39A10	0.652304893	0.00352843	KLHL24	0.371466544
RP11-347P5.1	2.074718886	2.1455E-11	TRA2B	0.510772634
ANKRD44	0.718284493	0.000014924	ETV5	0.429635403
CASP8	0.856469038	0.000074713	DNAJB11	0.501334812
EEF1B2	0.249208877	0.00529031	EIF4A2	0.407635544

RPL37A	0.248211508	0.00229719	ST6GAL1	0.447217444
SLC11A1	1.051700622	0.00926075	IL1RAP	0.606537332
ANKZF1	1.052494661	0.00034278	CCDC50	0.30591277
PID1	1.430658398	1.0137E-25	HES1	0.867788317
SP110	0.679181686	0.00192519	ZDHHC19	0.733000236
GPR55	2.278357151	0.00002219	CYTL1	2.592890465
COPS7B	0.735851225	0.03383633	STK32B	1.392911751
TIGD1	1.473420155	0.01109859	TBC1D14	0.498612773
CAPN10-AS1	1.304463571	0.03828754	SH3TC1	0.38750469
CAPN10	0.826210992	0.01416227	RBPJ	0.387522315
TRNT1	0.756054619	0.00883472	PDS5A	0.287647652
SUMF1	0.510539484	0.03026077	RBM47	0.38187559
ITPR1	0.628064025	0.03733764	NSUN7	0.643355969
OGG1	0.824609521	0.04108763	FRYL	0.39263899
ARPC4	0.472327151	5.7621E-08	CHIC2	0.34141753
GHRL	1.038162394	0.00021729	YTHDC1	0.384539953
TAMM41	1.201332479	0.00019364	JCHAIN	1.126475108
TSEN2	1.22594809	0.04644234	CXCL8	0.921044507
RPL32	0.291331685	0.000045795	CXCL3	1.34379396
NUP210	0.974697551	0.02872063	CXCL2	1.403748589
XPC	0.65847497	0.01973968	SEPT11	0.606025852
RPL15	0.204465844	0.00128602	BMP2K	0.56338807
OXSM	0.973102207	0.01749851	RASGEF1B	0.769930337
ACAA1	0.563783662	5.3554E-06	THAP9-AS1	0.478350068
MYD88	0.734652815	1.8405E-06	WDFY3	0.611405447
EXOG	0.912697404	0.01318928	ARHGAP24	1.117925259
CX3CR1	1.108486222	0.00025997	HSD17B11	0.323273595
RPSA	0.209032893	0.00563289	ABCG2	0.703578172
RPL14	0.217635407	0.00788384	FAM13A	0.807370105
NKTR	0.94429444	5.6273E-15	HPGDS	1.780434974
ZNF445	1.111278567	0.00019813	PPP3CA	0.474518988
TMEM42	0.696252164	0.01624715	NFKB1	0.791305845
CDCP1	0.592840065	0.04083464	UBE2D3	0.30926925
SACM1L	0.720926861	0.00195401	USP53	0.449511091
CCR2	2.781505355	7.0743E-14	KIAA1109	0.332801265
RP11-24F11.2	1.65590567	0.01630017	TBC1D9	0.694556182
RP11-708J19.1	1.759157625	0.00001479	TLR2	0.620243212
ELP6	0.570163956	0.03449255	PDGFC	0.734636105
ZNF589	1.267067778	0.0125804	FAM198B	0.439475964
NME6	0.648431352	0.04787963	FNIP2	0.486781438
TMA7	0.243597216	0.00035539	RAPGEF2	0.828352071
QARS	0.463647875	0.00199305	WWC2	0.848075901
GPX1	0.195080551	0.00180941	CDKN2AIP	0.419923026
RASSF1	0.466735125	0.01662784	MIR3945HG	0.93944244
CISH	1.013090409	0.02631782	FAM105A	0.239346056
MAPKAPK3	0.41623829	0.02090896	SLC1A3	0.727959963
RPL29	0.290176884	0.00079769	NIPBL	0.323918285
CACNA2D3	1.331058907	2.349E-17	DAB2	0.932637588
CCDC66	0.824077016	0.00149511	PTGER4	0.252613315
KCTD6	1.222312118	0.00080202	CCDC152	0.837721684

RP11-154H23.3	2.840766616	0.000011795	SEPP1	0.889122877
SHQ1	1.132341785	0.03723366	HMGCS1	0.605620191
ZNF717	1.01221396	0.0373716	EMB	0.853334993
MTRNR2L12	0.947127557	0.03863429	PARP8	0.544828902
CLDND1	0.398855493	0.03528988	IL6ST	0.876547379
ZBTB11	0.589396851	0.00618621	PLK2	0.47713436
ZBTB11-AS1	1.124343361	0.04696853	ZSWIM6	0.58393229
ABHD10	0.640987754	0.03536663	ERBB2IP	0.438059866
ZDHHC23	1.6212583	0.02110309	FCHO2	0.739176894
QTRTD1	0.702344084	0.00071956	JMY	0.793692929
GTF2E1	1.322264108	0.00315873	SERINC5	0.434565672
HCLS1	0.217048146	0.04819995	ZFYVE16	0.476402467
CSTA	0.66807545	2.7221E-07	SSBP2	0.561102288
WDR5B	2.370258821	0.00039806	RASA1	0.692411173
PARP14	0.271959358	0.02764986	CTC-428H11.2	0.767639131
SEC22A	0.931349115	0.04344494	CCNH	0.455081953
MYLK-AS1	3.297932497	0.00148963	ARRDC3	0.512739908
UMPS	0.84322225	0.00728206	RHOBTB3	0.696114396
CNBP	0.371780785	0.00026265	ELL2	1.588681621
MBD4	0.425221514	0.02248537	FER	0.629483715
NUDT16	0.407864203	0.00052004	PJA2	0.241426322
ACPP	1.655621802	0.00090088	WDR36	0.566056111
DZIP1L	0.991109849	0.01904682	EPB41L4A-AS1	0.394600498
DBR1	0.745552986	0.02313099	GRAMD3	0.829789696
GPR171	1.2735967	0.00038269	AC116366.6	1.138070283
P2RY14	0.934127307	0.000077464	IRF1	0.689901702
P2RY13	0.66312027	0.0010143	AFF4	0.736231005
MBNL1	0.334568633	0.00049666	CXCL14	1.411325565
RP11-305K5.1	2.229111233	0.03955141	TGFBI	0.455893849
IFT80	1.233515605	0.0073994	FAM53C	0.618176068
PHC3	0.768345536	0.01369819	EGR1	1.42002598
TNFSF10	0.982648143	1.478E-07	ETF1	0.468810385
KCNMB3	2.433559606	0.00253799	TMEM173	0.509128091
TTC14	0.791237083	1.1945E-06	HBEGF	0.682625942
MAGEF1	0.808498576	0.0164093	CD14	0.712979812
IGF2BP2	1.598716033	0.00530795	PCDH12	2.374665497
RP11-573D15.9	2.290492245	0.00024367	ARHGAP26	0.382172944
RTP4	0.957018436	0.03847863	NR3C1	0.432001094
LPP-AS2	2.608136084	0.01903098	ADRB2	0.545723585
LPP	0.39120063	0.00169852	CSF1R	0.386160799
UBXN7	0.786732168	0.00095708	TCOF1	0.695826977
NCBP2-AS2	0.506893597	0.0165293	RBM22	0.474941258
RPL35A	0.243516954	0.0022574	DCTN4	0.392020151
ZNF595	1.362312751	0.03182987	DOCK2	0.310347805
ZNF721	0.857968806	0.00011296	LCP2	0.641937482
CTBP1-AS	2.068374285	0.04802199	DUSP1	0.790648366
CTBP1	0.433935664	0.01622204	CREBRF	0.610167841
LYAR	0.441177593	0.02407737	CPEB4	0.60751829
BOD1L1	0.467109709	0.000060939	KIAA1191	0.458711381
ANAPC4	0.781670945	0.04822142	HNRNPH1	0.329618621

TLR10	2.06204085	0.00015044	MAML1	0.885239607
TLR6	1.040102181	0.00224431	SQSTM1	0.308137357
RFC1	0.550779526	0.00951877	MGAT1	0.38698235
LIAS	0.755173922	0.02762354	SERPINB9	0.797535326
SMIM14	0.639937385	3.0328E-06	PXDC1	0.478420972
NFXL1	1.211191351	0.0017946	F13A1	2.34766645
AASDH	0.811419187	0.0031959	PHACTR1	0.314646004
REST	0.448171555	0.00900846	CD83	1.067158274
POLR2B	0.630407069	0.00066763	MYLIP	0.821074713
UTP3	0.710587011	0.00010412	ATXN1	0.363068465
RUFY3	0.775010245	0.0003272	NUP153	0.470935346
GRSF1	0.360351637	0.02638785	RNF144B	0.58296033
COX18	0.953942107	0.01409816	C6orf62	0.271768049
PARM1	1.443233588	0.00901486	HFE	0.899460404
THAP6	1.054054254	0.00017225	BTN2A1	0.480303687
CNOT6L	0.424981515	0.01176042	HLA-E	0.299644958
RP11-109G23.3	1.797131334	0.000019795	PPP1R10	0.384110888
PLAC8	1.457101651	1.0382E-10	IER3	1.321137185
GPAT3	0.839215908	9.6849E-06	TNF	0.899416822
KLHL8	0.674638316	0.00508753	HSPA1A	0.744935111
PPM1K	0.689309556	0.00192207	HSPA1B	0.729788263
PYURF	0.2842863	0.03253527	NEU1	0.549246482
CCSER1	1.265188898	0.00158947	BRD2	0.451311634
METAP1	0.787313755	0.00104324	RGL2	0.548417648
DAPP1	1.236473199	1.1026E-13	PHF1	0.555444026
DNAJB14	0.424686216	0.02503649	ANKS1A	0.566349053
AC004069.2	2.184443445	0.00038333	PPARD	0.708800112
RP11-73K9.2	2.019172178	0.01204959	FKBP5	0.317897746
LARP7	0.281200628	0.02930605	SRSF3	0.552673866
C4orf3	0.205321329	0.0434218	CDKN1A	0.777910682
BBS12	1.729332257	0.02820825	VEGFA	1.062841632
SCLT1	0.743622332	0.00386202	HSP90AB1	0.510187622
MAML3	0.947869199	7.4449E-06	ENPP4	1.17400707
RP11-102N12.3	1.296443407	0.00174049	TNFRSF21	0.552572563
ANAPC10	0.612905183	0.03282713	OGFRL1	0.239282912
ABCE1	0.395714115	0.0221037	AKIRIN2	0.494184555
PRMT9	0.69134148	0.00121033	PNRC1	1.047016894
RPS3A	0.200909512	0.01065922	PRDM1	0.901719674
TMEM154	0.842308261	0.02566585	FOXO3	0.88890397
KIAA0922	1.095862833	0.00324345	MARCKS	0.452623278
PLRG1	0.735955028	0.00031151	DSE	0.260389502
GUCY1A3	0.806255849	0.00199672	MAN1A1	1.07128552
RP11-597D13.9	1.213716949	0.021183	SERINC1	0.475430862
TMA16	0.522906128	0.00230126	SMPDL3A	0.831615807
MARCH1	0.761604391	5.0661E-07	ARHGAP18	0.334307958
GALNT7	0.629070643	0.01662784	EPB41L2	0.429432711
RP11-798M19.6	1.015353702	0.00494552	SGK1	0.445681128
SCRG1	0.970961248	0.03935832	MAP3K5	0.46358709
RP11-326I11.3	2.055451209	7.8549E-08	TNFAIP3	1.419974061
RP11-701P16.2	2.244311555	0.00051651	NHSL1	0.645511337

LRP2BP	1.476841289	0.01785864	RP11-390P2.4	0.497837505
CTD-2256P15.2	1.419860278	0.04978766	REPS1	0.524931456
BRIX1	0.677791227	0.00467638	HECA	0.549901109
RICTOR	0.837380182	0.0002386	CITED2	0.656973813
RPL37	0.256628051	0.00011931	ADGRG6	1.356769168
CARD6	0.688732362	0.046517	STX11	0.670228287
GAPT	1.946420346	2.0795E-15	UTRN	0.253262004
KIF2A	0.470012797	0.02047105	TAB2	0.497827025
ADAMTS6	2.556818324	0.00317668	ZC3H12D	1.028064198
PPWD1	0.620127614	0.03567149	CCDC170	0.681787624
CD180	0.684869585	0.0015476	SCAF8	0.494010501
CENPH	0.887773277	0.03767101	ARID1B	0.563826508
MRPS27	0.667000685	0.01768447	SNX9	0.551222256
ZNF366	1.602183642	0.00015442	EZR	0.512317366
BTF3	0.169902742	0.01152796	TAGAP	0.489633559
POLK	0.620802519	0.00902082	SOD2	0.896937266
POC5	1.18145788	0.00172503	WTAP	0.514941992
S100Z	1.647634429	1.7724E-08	QKI	0.544009222
AGGF1	0.638914868	0.00020118	RNASET2	0.214489543
ZCCHC9	0.494721797	0.03262711	MAFK	0.665602361
RPS23	0.246830642	0.00218991	TNRC18	0.552255255
VCAN	1.288492942	0.00654852	FSCN1	1.137716326
TMEM161B-AS1	1.222409621	0.01023215	ZNF316	0.7060916
MEF2C	0.685246457	0.000020814	ICA1	0.787087252
MBLAC2	1.378769147	0.04558506	TMEM106B	0.441681163
FAM172A	0.677049476	0.0220247	ARL4A	0.495029131
SLF1	0.955821597	0.0004981	ETV1	1.743137527
LINC01554	1.474058633	0.03868924	AHR	0.42355016
GIN1	0.871198465	0.04749883	TWISTNB	0.655470505
NREP	1.353552455	0.00775343	SP4	0.973035321
DCP2	0.768738507	1.1285E-09	TRA2A	0.346609547
PGGT1B	0.621078509	0.00058977	CYCS	0.370544687
CTC-428G20.6	2.067025079	0.00413207	HNRNPA2B1	0.23245226
LMNB1	0.85973005	0.0071009	MTURN	0.723146615
LYRM7	0.678465859	0.00183249	PURB	0.311025465
C5orf56	0.863925999	0.00023818	TNS3	0.625498916
RAD50	0.501984403	0.00122751	RABGEF1	0.624170516
DDX46	0.419576853	0.000038644	SBDS	0.351338742
BRD8	0.674446558	0.0379377	WBSCR27	0.716525016
TAF7	0.436347998	0.01260272	DTX2	0.480648899
DIAPH1	0.506923473	0.00261286	CCDC146	0.750804955
HDAC3	0.388833834	0.0382514	FGL2	0.411062775
KIAA0141	0.527933072	0.02422252	PTPN12	0.257164097
TCERG1	0.365543467	0.03742436	HGF	1.244594029
PPARGC1B	0.848741655	0.00383174	AKAP9	0.200692587
RPS14	0.270483011	0.00572854	ANKIB1	0.475967729
MED7	0.816800494	0.00193179	PDK4	0.710946629
PWWP2A	0.592332507	0.04298615	GAL3ST4	0.915914564
KCNMB1	1.607192431	4.781E-12	MEPCE	0.666184052
HRH2	0.943964773	0.00008149	NAMPT	0.85223815

NSD1	0.479305007	0.00166775	RP11-22N19.2	0.526553637
RAB24	0.801731691	0.0009612	HBP1	0.426003569
PRELID1	0.371231548	0.00559183	IFRD1	0.450695447
RGS14	0.756126087	0.00945447	MDFIC	0.416349502
TMED9	0.249758823	0.01285174	CPED1	0.508489821
NHP2	0.395801775	0.0005843	RP11-138A9.1	0.572577147
CLK4	0.515150814	0.04590575	MKLN1	0.440197191
GNB2L1	0.233095221	0.00461841	CREB3L2	0.348549613
DUSP22	0.786227227	3.4487E-07	KIAA1147	0.740509121
RP11-532F6.3	1.693402246	0.00682806	EPHB6	2.225742075
GMDS-AS1	1.025253503	0.01500058	EZH2	0.7138285
WRNIP1	0.572980896	0.03645592	LINC00996	0.537460435
PRPF4B	0.642962667	0.000017711	GIMAP5	0.883438759
CAGE1	2.281144559	0.00552922	KMT2C	0.248902582
EEF1E1	0.464892247	0.04422928	DNAJB6	0.717479175
GCNT2	1.403918104	0.00029956	PLCXD1	0.707115509
TMEM170B	0.896709725	0.00086266	CD99	0.385854165
RP1-223E5.4	1.734138661	0.0373567	ARHGAP6	0.812329229
RP1-151F17.2	1.893004971	0.04778412	ASB9	1.289376416
SOX4	0.835248579	0.000033003	PIGA	0.802172277
MRS2	0.572403374	0.03662583	AP1S2	0.287464524
TRIM38	0.785366734	7.3325E-07	SYAP1	0.476230001
HIST1H3A	3.112331745	0.01991179	SCML1	0.563348841
HIST1H2BD	1.530480893	0.01208944	SAT1	0.268416165
HIST1H2BE	2.213113094	0.00106814	KLHL15	0.697685004
RP1-34B20.21	1.049721296	0.04573977	ZFX	0.312845071
HIST1H2BG	1.641233933	0.000018174	CYBB	0.420989539
BTN2A2	0.902026619	0.00019247	BCOR	0.604850366
BTN3A1	0.66463778	0.02932935	DDX3X	0.758007222
ZNF322	0.845698218	0.00445715	GPR34	0.713996928
HIST1H2AG	1.361382821	0.04069294	KDM6A	0.486509002
HIST1H2BN	2.691610074	2.0762E-09	RP6-99M1.3	1.891402195
RP1-313I6.12	0.982728062	0.00001565	CHST7	0.877042862
RP1-265C24.8	2.636373836	0.01321492	RP2	0.479297528
ZSCAN9	1.174835741	0.03947339	RRAGB	0.617026343
TRIM27	0.488816719	0.03179056	STARD8	0.621772879
GABBR1	0.920383709	0.02831579	EDA	1.468556755
ZNRD1-AS1	1.862790978	0.00034502	LINC00891	1.300674848
PPP1R10	0.345696965	0.04990175	XIST	0.274376619
PPP1R18	0.430298163	0.00158972	RLIM	0.482919134
LINC00243	2.020569011	0.00121614	CYSLTR1	0.392025564
XXbac-BPG299F1	1.350407944	0.0214117	ARMCX1	0.861528086
DDX39B	0.593422077	0.00377854	WBP5	0.660212132
LTB	0.851917101	0.00605471	MORF4L2	0.279230829
LST1	0.579068617	4.8422E-16	ALG13	0.673632542
C6orf47	0.678152303	0.03621885	DOCK11	0.47295026
GPANK1	0.788271275	0.00129212	IL13RA1	0.336127426
ATF6B	0.557979423	0.01944754	PGRMC1	0.410375342
GPSM3	0.521164394	2.8868E-08	STAG2	0.426488904
PSMB8	0.518745768	4.1207E-06	ARHGEF6	0.373887413

PSMB8-AS1	0.985144628	7.664E-09	CXorf40A	0.465629393
PSMB9	0.518570082	0.00012331	CXorf40B	0.612238392
HLA-DPB1	0.225264863	0.03831272	ARHGAP4	0.466136785
RPS18	0.308607969	0.00068613	NEIL2	0.60849779
PFDN6	0.378773476	0.03707399	CSGALNACT1	0.886119451
NUDT3	0.444708627	0.000014924	GFRA2	0.539906091
RPS10	0.228887322	0.00467191	SORBS3	0.647185691
RP11-140K17.3	1.099028544	0.00058592	EGR3	0.752749812
DEF6	0.864042538	0.00002057	TNFRSF10B	0.52158739
RPL10A	0.35825517	2.6169E-06	TNFRSF10D	0.84039596
MAPK14	0.634195478	0.00209279	BNIP3L	0.685263856
STK38	0.849010579	0.00130553	DPYSL2	0.404078736
CPNE5	2.432588961	0.02898786	ZNF395	0.548936444
MTCH1	0.280401966	0.01141907	KIF13B	0.480570265
FGD2	0.655130695	6.004E-07	FGFR1	0.817373915
RNF8	0.789252636	0.00392717	TACC1	0.298512349
GLO1	0.390299566	0.0042641	PLEKHA2	0.377950136
TFEB	0.749036635	0.000119	KAT6A	0.415628822
PRICKLE4	1.235459159	0.03442182	CEBPD	0.310130248
USP49	1.412419117	0.01389366	SDCBP	0.3152621
MED20	1.482311316	0.00434896	CHD7	0.549128033
CCND3	0.671316559	0.000017688	YTHDF3	0.321518654
MRPS10	0.427274128	0.02005465	RP11-79H23.3	1.605251092
UBR2	0.5648242	0.01381868	ZBTB10	1.677522257
CNPY3	0.476228028	9.9904E-08	RP11-48B3.4	1.890932382
KLHDC3	0.531187978	0.0053224	ZNF704	2.026498927
KIAA1586	0.712256229	0.04256209	PAG1	0.510975157
XXbac-BPGBPG55	2.866467825	2.8843E-08	WWP1	0.906924205
RP5-1148A21.3	2.950606506	0.0007564	PABPC1	0.463724577
B3GAT2	1.536039914	0.04512941	YWHAZ	0.364941817
MTO1	0.892994004	0.00235915	KLF10	0.893859825
RP11-553A21.3	3.412254044	0.01688337	AZIN1	0.631910581
PHIP	0.588288658	0.00021073	KB-1507C5.4	1.32392994
FAM46A	0.661127313	0.000034953	TMEM65	0.686561926
RP11-393I2.4	2.045464594	0.00012437	MTSS1	1.016956887
CASP8AP2	0.660107869	0.00117385	TRIB1	0.554484189
MANEA-AS1	1.722142364	0.00376489	PTK2	1.220943586
UFL1	0.37957496	0.02045459	SLC45A4	1.036416725
MMS22L	1.537966159	0.00286401	EEF1D	0.150479128
PNISR	0.784465252	8.7424E-07	SMARCA2	0.49281638
C6orf203	0.973709491	0.0017397	PLIN2	0.43574034
RP1-191J18.66	2.268545128	0.00199017	DENND4C	0.606275112
SESN1	0.769045453	0.000030657	C9orf72	0.318187811
FIG4	0.742283173	0.02309646	ACO1	0.509320586
CDC40	0.415794747	0.00665351	DNAJA1	0.637850236
CDK19	0.656301133	0.00613194	B4GALT1	0.749890184
KIAA1919	0.941742071	0.00715694	DCAF12	0.613928017
RP5-1112D6.8	1.690703575	0.04091248	UBAP1	0.404488857
TRAF3IP2-AS1	0.90069185	0.04654814	RP11-331F9.4	1.689631103
RP3-486I3.7	2.015949544	0.02615449	PIP5K1B	1.240266187

FAM26F	0.472566264	0.000044827	MAMDC2	3.398297354
KPNA5	1.280803254	0.00235241	KLF9	0.706551998
CEP85L	0.848207594	0.00068915	TMEM2	0.889591339
PKIB	0.80811983	0.00122662	ZFAND5	1.050734907
RNF146	0.591776307	0.036014	GCNT1	0.454038302
ECHDC1	0.627712968	0.00300209	PRUNE2	0.87716623
VNN1	1.319147196	0.00503868	GNAQ	0.390717346
VNN2	1.160821992	0.04740929	TLE4	0.380678661
RPS12	0.273642959	0.0019523	SYK	0.278787385
MYB	2.58320959	7.6274E-06	NINJ1	0.429778567
IFNGR1	0.473172347	0.000061519	NR4A3	0.751872983
RP11-356I2.4	0.847964704	0.00115842	ZNF189	0.60799036
SHPRH	1.124185139	1.0172E-07	ABCA1	0.522574002
RAB32	0.293682106	0.04597052	KLF4	1.200098328
IPCEF1	1.364978095	0.0000106	PTGS1	0.610148423
TFB1M	0.944207073	0.01130386	HSPA5	0.989760173
CAHM	1.315137067	0.00802753	MVB12B	0.491534284
WDR27	1.334905505	0.00224533	FNBP1	0.352906691
TCTE3	1.476034684	0.00016906	NUP214	0.586249792
AC093627.10	1.001797659	0.00158669	PRRC2B	0.411028227
C7orf50	0.36284813	0.01450159	RALGDS	0.549232037
FTSJ2	0.970046239	4.2307E-08	GBGT1	0.864082773
CHST12	0.738495099	0.00416479	EGFL7	1.652824375
ACTB	0.256475913	0.00064054	TUBB4B	0.456766549
PHF14	0.602295274	0.00015852	TSPAN4	0.31310711
HDAC9	0.726994411	0.01815171	AP2A2	0.696548625
NUPL2	0.668295368	0.00272469	CDKN1C	0.755654428
NFE2L3	0.66125565	0.000091733	RNF141	0.486058067
CBX3	0.404625444	0.00017397	LYVE1	2.947505957
SNX10	0.43102904	0.03120296	MRVI1	1.525005145
CTB-113D17.1	2.099987102	0.00022559	PIK3C2A	0.361413738
CPVL	1.011934231	7.2021E-12	SPTY2D1	0.321467689
CHN2	0.710078825	0.000057646	TTC17	0.413939815
SCRN1	0.625893377	0.0224723	TP53I11	0.809834322
GGCT	0.382831971	0.00561911	CELF1	0.333957368
RP9	0.706124017	0.01494631	PTPRJ	0.310904357
SEPT7	0.270802745	0.0080349	SERPING1	0.429711416
AOAH	0.277308043	0.0028462	FAM111A	0.371605391
YAE1D1	0.971824342	0.03258297	MPEG1	0.169148341
RP11-467D6.1	1.965139918	0.01012969	MS4A6A	0.366232552
MPLKIP	0.388134576	0.02663555	MS4A4A	0.927670079
INHBA-AS1	3.137505233	1.2788E-08	MS4A7	0.836733563
AC004951.6	1.058454511	0.01457596	RAB3IL1	0.963618853
TBRG4	0.576611973	0.02716574	FTH1	0.297909406
FIGNL1	1.244872992	0.00659144	AHNAK	0.583506761
ZNF680	1.184461583	0.00406949	CCDC88B	0.667997098
ZNF117	0.944805627	0.03916477	EHD1	1.1166924
ERV3-1	1.580342822	1.891E-08	CDC42EP2	0.79541116
GS1-124K5.3	2.271299417	0.00232387	NEAT1	0.798057808
NSUN5	0.622166876	0.00387299	SIPA1	0.3429481

LIMK1	0.630791446	0.01306286	CTSF	0.914413946
NCF1	0.305302581	0.01148772	CHKA	0.571875869
TRIM73	1.837877768	0.0017261	PPP6R3	0.364749558
MDH2	0.243720546	0.0379377	NUMA1	0.764583555
POMZP3	1.291325679	0.00507935	FOLR2	2.116196583
RSBN1L	0.817547583	4.3403E-09	FCHSD2	0.469869198
GNAI1	0.941751121	0.02281029	SLCO2B1	0.820658237
MTERF1	1.206879896	0.01668874	LRRC32	1.750941681
KRIT1	0.63639538	0.01001496	MYO7A	1.672660873
GATAD1	0.641506972	0.000016397	PCF11	0.531869443
SAMD9	0.820065495	0.00050839	PICALM	0.358751752
SAMD9L	0.89995957	0.000053871	CTSC	0.342457286
CASD1	0.670042769	0.04900927	MAML2	0.605315635
LAMTOR4	0.199955315	0.01420536	BIRC3	0.524369609
PPP1R35	1.245002391	2.8801E-06	BIRC2	0.422303769
TSC22D4	0.528698594	0.00326233	ZC3H12C	0.995835271
SLC12A9	0.490954834	0.01973018	IL18	0.41147894
TRIM56	1.074286662	6.5985E-10	IL10RA	0.44629357
ALKBH4	1.001301706	0.03121828	H2AFX	0.502266636
POLR2J3	0.948221352	1.7798E-11	FLI1	0.489903641
NAPEPLD	1.166443981	0.03249653	APLP2	0.397146896
ORC5	0.900496786	0.03831272	ZMYND11	0.44229644
KMT2E-AS1	0.65335348	0.03716202	DIP2C	2.298303055
THAP5	0.837492934	0.0005843	IDI1	0.589878155
LSMEM1	1.324954763	0.01106506	KLF6	0.836593289
TMEM168	0.792698557	0.04955038	IL2RA	0.79948764
TNPO3	0.594312106	0.03558246	PFKFB3	0.707812161
CNOT4	0.615557859	0.00037984	SFMBT2	0.584433556
C7orf55	0.44044613	0.04925791	CELF2	0.285275258
HIPK2	0.440998748	0.02152257	FRMD4A	0.548515113
CASP2	1.033830549	0.00039655	TMEM236	1.223375963
AC093673.5	1.008288888	0.01730428	MRC1	1.072026882
ZNF467	0.579337897	0.04999487	ARL5B	1.202527711
GIMAP8	0.650628734	0.00119179	MSRB2	0.405741178
GIMAP7	2.378732093	4.8885E-46	OTUD1	0.429882198
GIMAP4	0.910271787	4.6592E-07	ARHGAP21	0.597081856
GIMAP1	0.948131391	0.000014854	WAC	0.384663719
PRKAG2	0.583690628	0.00017462	RP11-472N13.3	0.46459575
PRKAG2-AS1	1.028290675	1.7701E-06	ARHGAP12	0.827506655
AC144652.1	1.387542643	0.00023604	NRP1	0.715931322
LINC01006	1.623202448	0.03590634	RASSF4	0.299433557
SLC25A6	0.158006191	0.02225997	FAM21C	0.464623557
HCCS	0.53817242	0.03182328	FAM21A	1.22663934
MSL3	0.705942743	2.7234E-06	SGMS1	1.057086686
TLR8	0.556696002	0.03175689	IPMK	0.409407369
OFD1	0.744994335	1.721E-08	UBE2D1	0.336101072
ZRSR2	0.701590914	0.00688124	RHOBTB1	0.840388757
CXorf21	1.392059538	1.0217E-10	JMJD1C	0.397866761
CFP	1.408850695	5.2631E-09	SIRT1	0.739287825
ZNF182	1.402749819	0.00672807	SRGN	0.838672339

TIMM17B	0.614547744	0.00043602	PALD1	0.468734167
PLP2	0.598139824	0.01785202	DDIT4	0.582960934
SYP	1.783738604	0.03750612	P4HA1	0.463681087
ARHGEF9	0.93964775	0.005632	PLAU	1.054092812
RP6-159A1.4	2.042279792	1.0081E-09	PPIF	0.527707225
TAF1	0.611554119	0.00770952	CH25H	0.849069205
PIN4	0.396519177	0.028392	CPEB3	0.539255295
RPS4X	0.215833517	0.02257167	FRA10AC1	0.468949693
BRWD3	0.571360783	0.04916	TBC1D12	0.498242818
RPL36A	0.359837143	0.000010409	BLNK	0.685275537
TSC22D3	0.583096551	0.00182205	RRP12	0.867032175
RNF113A	0.660764216	0.00537329	DNMBP-AS1	1.098633723
ZBTB33	1.265766171	0.000013842	NFKB2	0.719597416
RAB33A	1.231117249	0.00186306	SFR1	0.63725696
RPL10	0.207142214	0.00290232	ITPRIP	0.659987407
GAB3	0.810156037	0.00106814	MXI1	0.477606663
ERICH1	0.718126449	6.5626E-07	PDCD4	0.400986105
TNKS	0.764206609	0.00795573	TCF7L2	0.47849857
RP11-297N6.4	1.943348415	0.00056334	TDRD1	2.043780122
SLC25A37	0.828247175	2.0357E-06	GRK5	1.092091463
RP11-489E7.4	1.040365202	0.00012331	PLEKHA1	0.376960397
LEPROTL1	0.313805543	0.00842048	FAM53B	0.483144637
RP11-350N15.5	1.559775721	0.00697788	ZRANB1	0.814988522
LETM2	1.41590037	0.01946672	CTBP2	0.42138148
AP3M2	0.894362957	0.01955818	WNK1	0.303153201
RP11-231D20.2	2.450503838	0.00045253	ADIPOR2	0.450528175
IKBKB	0.581757305	0.02647296	TNFRSF1A	0.351713743
VDAC3	0.347411881	0.00500624	C12orf57	0.49085581
THAP1	0.592909834	0.03668635	CD163L1	0.826298953
SPIDR	0.632146093	0.03059358	CD163	0.899194211
LYPLA1	0.351650677	0.00618757	CLEC4E	0.848645254
PDE7A	0.853435189	0.00015852	A2M	0.629844665
RRS1	0.913682342	0.00842048	OLR1	0.773167852
VCPIP1	0.716548241	0.0003763	YBX3	0.955876423
C8orf44	1.626290405	0.0142175	CREBL2	0.381041464
RPL7	0.141897585	0.04386113	EPS8	0.895063951
UBE2W	0.539105302	0.007347	BCAT1	0.567884763
PEX2	0.466375217	0.01148772	ITPR2	0.69355654
RP11-48B3.5	2.306428013	7.312E-09	FAR2	1.063045399
C8orf59	0.36259385	0.00097547	YAF2	0.280770959
RP11-37B2.1	1.214707148	0.00083428	SLC38A2	0.790939341
OTUD6B-AS1	1.284206919	7.3723E-13	HDAC7	0.628301862
OTUD6B	0.721864721	0.01099009	RND1	0.9756377
LRRC69	2.565181763	0.0036624	FMNL3	0.494011995
CDH17	0.896263362	0.02322488	DIP2B	0.577025983
TP53INP1	0.751137614	0.02248537	METTL7A	0.559375799
MTERF3	0.829272684	0.04243319	LETMD1	0.385202963
RPL30	0.259174352	0.00018738	GRASP	0.873112379
EIF3E	0.2090928	0.03283864	NR4A1	0.889508877
NUDCD1	0.75062213	0.03493393	EIF4B	0.240857062

MRPL13	0.387091906	0.01463794	MMP19	0.871080475
RNF139-AS1	1.874824158	0.00036371	LRP1	0.520797695
PVT1	1.512495608	0.02418139	DDIT3	0.855982728
PHF20L1	0.411433245	0.00548488	SRGAP1	0.79168007
SLA	0.795324282	8.0983E-09	IRAK3	0.636463786
RP11-629O1.2	1.372929217	0.02219537	HELB	0.583158779
LY6E	0.308868007	0.00924731	CPM	0.919908429
TOP1MT	1.153060572	0.01622184	CNOT2	0.359043173
ZNF707	1.606561723	0.03009407	THAP2	1.023097519
ZNF34	1.63584577	0.00444545	RAB21	0.58012945
RPL8	0.253244438	0.00083428	TBC1D15	0.707511144
ZNF250	1.402037628	0.01529674	DUSP6	0.540422393
WASH1	0.580786916	0.01995144	DCN	1.945120621
PUM3	0.569116895	0.00634037	BTG1	0.516814234
JAK2	0.559172435	0.03471285	NUDT4	0.790294913
RP11-390F4.3	1.575756218	0.03133288	IGF1	0.60775126
RPS6	0.243341081	0.00010727	HSP90B1	0.624463196
RPP25L	0.656216022	0.00461841	KIAA1033	0.526704564
HINT2	0.483477978	0.01065135	SELPLG	0.514213864
TRMT10B	0.718285408	0.02175718	ATP2A2	0.515224154
LINC01410	1.904665157	0.00025741	SH2B3	0.630187053
ANKRD20A4	2.531423792	0.00146317	TPCN1	0.55511486
CBWD5	0.830003375	0.00176081	MED13L	0.557199129
ZNF658	2.188329443	0.00404255	P2RX7	0.707977505
CBWD3	1.169442614	0.00172094	RILPL2	0.779554452
FAM122A	0.59740516	0.04147898	ULK1	1.212572796
RFK	0.449973825	0.04614283	ZMYM2	0.464914569
FRMD3	1.499278947	0.03026077	NUP58	0.708716731
RMI1	1.320944576	0.000086648	USP12	0.60670125
ZCCHC6	0.740795114	0.000013324	UBL3	0.366407731
NOL8	0.574111072	0.00075486	HSPH1	0.719124072
BICD2	0.808411049	1.6117E-06	BRCA2	0.792282218
XPA	0.426391394	0.02018758	N4BP2L1	0.413299263
AKAP2	2.312727835	0.02583287	STARD13	1.009665681
INIP	0.791687174	0.0002313	LHFP	0.56360391
SNX30	0.80461148	0.00389332	FOXO1	0.615254872
SLC46A2	3.023089427	1.4123E-06	ELF1	0.731390536
CDC26	0.382993617	0.03523014	TPT1	0.274081922
POLE3	0.581027236	0.00054903	LRCH1	0.676846402
MEGF9	0.920642847	0.00195697	ITM2B	0.344444174
PSMD5-AS1	1.358849627	0.00037023	FNDC3A	0.363844004
CNTRL	0.525573121	0.00233895	NEK3	0.558123757
RAB14	0.31495254	0.02225997	KCTD12	0.486324166
MRRF	0.711613583	0.00550058	MYCBP2	0.281624484
RPL35	0.226470038	0.000079654	RBM26	0.369867737
URM1	0.601158995	0.00080094	MBNL2	0.577797981
C9orf114	0.715036055	0.02225997	GPR183	0.764377578
IER5L	0.689060628	0.01375299	IRS2	0.856323138
FAM78A	0.700808325	0.00617251	RAB20	0.517689961
RPL7A	0.225560074	0.00725505	LINC00346	1.007491445

REXO4	0.559670395	0.02764986	GAS6	1.065452393
BRD3	0.697987313	0.00147396	PNP	0.949271354
FCN1	3.030681768	3.1238E-06	RNASE1	1.25655672
PHPT1	0.325003816	0.0155857	HNRNPC	0.340551778
EDF1	0.197322305	0.01466095	NYNRIN	1.255511348
C9orf142	0.370729622	0.0112693	SNX6	0.509861876
SSNA1	0.323823297	0.03485623	BAZ1A	0.568425027
TMEM203	0.816897364	0.000034352	NFKBIA	0.996779964
DPH7	0.783587091	0.03283864	SAV1	1.079680808
ARRDC1	0.34664368	0.04832336	GCH1	1.049741033
SIGIRR	0.51815671	0.00897268	KTN1	0.219119487
TMEM80	0.966748466	0.0028014	DAAM1	0.895625733
RPLP2	0.154351292	0.04136637	HIF1A	0.366163421
LSP1	0.652509606	0.00346741	SNAPC1	0.76667283
ASCL2	0.662397092	0.00287157	ZBTB1	0.453513755
TSPAN32	3.264285227	0.000014854	ZFP36L1	0.615128364
NAP1L4	0.630349741	0.00005858	SUSD6	0.567475436
TRIM22	0.634710057	2.4117E-09	SRSF5	0.735664587
TIMM10B	0.514438915	0.0466357	PSEN1	0.516130542
RRP8	0.673856259	0.01326739	NPC2	0.213019163
ILK	0.313672963	0.03110464	LTBP2	2.230852029
EIF3F	0.284008101	0.02867817	FOS	0.868836415
ZNF143	0.59422811	0.04949067	JDP2	0.447339295
ZBED5	0.808300007	0.01086045	STON2	0.704373112
BTBD10	0.896706127	0.01211253	FOXN3	0.354706802
FAR1	0.557316075	0.0045783	RPS6KA5	0.642510683
HPS5	0.602223391	0.0001148	PPP4R3A	0.418685844
ARL14EP	0.571952424	0.01967236	LGMN	0.613261904
RCN1	0.610556242	0.00168613	DDX24	0.378960992
EIF3M	0.188307219	0.0379663	CCNK	0.306319731
LINC00294	1.233081973	0.01895488	TNFAIP2	0.879853638
CSTF3	0.562156044	0.04394718	EIF5	0.558762732
TRIM44	0.586325942	0.000027088	GPR132	0.719837436
COMMD9	0.512090381	0.00019302	IGHA1	0.949606301
PRR5L	1.206981133	0.01012969	IGHM	1.05627541
UBE2L6	0.491773307	0.00012256	MTMR10	0.516297787
FAM111A	0.442800161	0.04549144	FMN1	0.612481937
CCDC86	1.490419666	6.1767E-08	SPRED1	0.767114566
PRPF19	0.54485253	0.00571697	THBS1	1.313322224
RP11-881M11.2	2.597352807	0.00461823	GATM	0.736769082
TMEM109	0.57783874	0.00072198	BLOC1S6	0.268736671
METTL12	0.893886752	0.000033611	SECISBP2L	0.451934571
POLR2G	0.422951471	0.00144584	DMXL2	0.618121631
TMEM223	0.696843966	0.00191563	MYO5A	0.590661399
RP11-727F15.12	2.849566812	0.00034079	TCF12	0.74985229
RPS6KA4	0.59418381	0.00534882	BNIP2	0.216757768
AP003774.6	1.072950215	0.03109743	ICE2	0.413222599
RASGRP2	0.774699334	0.01335541	VPS13C	0.301719352
AP001462.6	2.308619856	0.00583065	TPM1	0.564622636
FAU	0.140565143	0.04325612	RAB8B	0.250392037

POLA2	0.820084047	0.03582105	APH1B	0.368037518
TIGD3	1.59537686	0.03981331	DENND4A	1.034938137
SLC25A45	0.529093912	0.03908224	ARIH1	0.565803086
MAP3K11	0.439892167	0.01749851	SIN3A	0.431943577
C11orf68	0.825150522	0.00455175	PEAK1	0.633801301
RAD9A	0.627204513	0.00781394	DNAJA4	0.865886689
AIP	0.488276321	0.00125466	BCL2A1	0.656543047
GSTP1	0.361414729	0.00043865	AKAP13	0.460611421
NDUFS8	0.212485637	0.03444942	IQGAP1	0.332091193
FADD	1.221237004	0.0062986	CHD2	0.420069769
NADSYN1	0.735184723	0.00049703	MEF2A	0.341830381
LRTOMT	2.264531114	0.00317759	SRRM2	0.342537825
LAMTOR1	0.406528561	0.000045619	CREBBP	0.370246534
ATG16L2	0.616602452	0.00230783	C16orf72	0.560736725
P2RY6	0.547240315	0.00164965	ATF7IP2	0.558520551
PAAF1	0.628613669	0.04386113	CTD-3088G3.8	1.686685832
RPS3	0.187223085	0.01452634	ZC3H7A	0.399007068
UVRAG	0.63522727	0.00130523	RRN3	0.68767411
PAK1	0.599011856	2.2215E-08	ARL6IP1	0.306021055
RP11-739B23.1	2.698567255	6.5198E-06	EEF2K	0.605065159
CLNS1A	0.403760434	0.00461066	RBBP6	0.507994298
ALG8	0.756552012	0.00248268	QPRT	0.616935603
PCF11	0.295511366	0.03008572	RP11-196G11.6	1.227797299
CREBZF	0.936887048	0.000012334	PRSS36	1.091241887
EED	0.523404305	0.04256508	FUS	0.301095099
SMCO4	0.703710877	0.00561911	ITGAM	1.030769149
MRE11A	0.651593026	0.00809182	ARMC5	0.798976336
ANKRD49	0.759641378	0.00381507	PHKB	0.344627995
SRSF8	0.750487185	0.000070026	LONP2	0.291748071
CARD16	0.515781569	4.9344E-06	CYLD	0.454474026
MSANTD4	0.865403428	0.04894291	HERPUD1	0.703557502
AASDHPPT	0.619585672	0.00033087	CNOT1	0.319427375
CWF19L2	0.452538754	0.02561592	DPEP2	0.470445305
ALKBH8	1.145997607	0.01700319	PLA2G15	0.630330269
CUL5	0.537897977	0.00090173	SNTB2	1.005912151
ATM	0.952143026	1.1733E-17	IST1	0.259947784
PCSK7	0.522158768	0.00620482	ZFHX3	0.711812309
JAML	0.723402595	3.2371E-09	MAF	1.143220081
MPZL3	1.513868907	0.01805275	CMIP	0.297044563
RPS25	0.196761549	0.00815331	PLCG2	0.567022873
CBL	0.904029111	1.4685E-07	DNAAF1	1.43590249
OAF	0.789023318	0.03949382	IRF8	0.820778612
SORL1	1.339436732	3.9454E-11	SLC7A5	0.761878261
NRGN	2.427654976	0.00100652	ZC3H18	0.433014756
ST3GAL4	1.376727924	0.01303043	MIR22HG	0.77383794
GDI2	0.258223252	0.00083971	WDR81	0.589797793
KIN	0.425201322	0.02615479	NLRP1	0.643080045
ECHDC3	0.697623061	0.02435473	POLR2A	0.431133986
UPF2	0.55330087	0.000084037	KDM6B	1.081431127
DCLRE1C	0.844419277	0.00081339	TMEM88	1.106793724

RPP38	0.718664453	0.01054499	CYB5D1	1.893056088
STAM-AS1	2.04270057	0.00280651	PER1	0.705152317
NSUN6	1.275875046	0.000048353	TMEM107	0.522862715
COMMD3	0.444045066	0.00583845	UBB	0.405678268
PIP4K2A	0.411506443	0.000048324	RP11-160E2.6	0.846532147
APBB1IP	0.558073002	3.9113E-06	ULK2	0.956991816
WAC-AS1	0.921691441	6.793E-09	MAP2K3	0.778628347
ZNF248	1.009000631	0.03008572	WSB1	0.369933752
RP11-162G10.5	1.874946384	0.01505591	NUFIP2	0.424263097
ZNF22	0.417694604	0.02528529	GOSR1	0.336720597
C10orf128	0.600445655	0.000013022	ADAP2	0.438385998
AGAP6	1.065405732	0.01796289	CCL2	1.142528287
CCDC6	0.427412732	0.0313822	CCL11	2.59125611
ADO	1.317105467	0.000022351	CCL13	2.493932758
EGR2	0.864282666	0.01282401	SLFN11	0.411960308
RP11-57G10.8	3.669814511	0.00055998	CCL18	0.841522798
HERC4	0.720682381	0.0154029	AC069363.1	1.363966008
TYSND1	0.785816492	0.02930913	CCL3	1.366128454
PPA1	0.442587868	0.0120562	CCL4	1.525094114
CDH23	1.347781308	0.00941686	CCL3L3	1.041528633
C10orf54	0.462414994	0.000024174	CCL4L2	1.077357089
RPS24	0.234939072	0.00220449	MLLT6	0.705510315
NUTM2B-AS1	0.503109384	0.02594376	CWC25	0.31373276
RP11-182L21.6	1.185909477	0.000096094	RPL23	0.212550472
RP11-338I21.1	4.255618392	0.000024464	PLXDC1	1.99193873
PTEN	0.241527536	0.0104339	FBXL20	0.5170815
ANKRD22	1.919690894	0.00902276	NR1D1	0.764492328
IFIT5	1.223995724	0.00566778	RARA	0.4872811
HHEX	0.94598953	0.000010763	IGFBP4	1.672481282
MYOF	0.712928515	0.01131933	EIF1	0.452687565
ENTPD1-AS1	1.124064784	0.018671	GHDC	0.90016225
PGAM1	0.488633074	0.00031164	TMEM106A	0.465363674
COX15	0.565543253	0.03008572	DUSP3	0.407776103
FBXL15	0.478852208	0.01071543	HEXIM1	0.359797639
C10orf95	1.893802915	0.00643438	ABCC3	0.596297192
CALHM2	0.731114319	0.04083464	SPAG9	0.397551399
SMC3	0.422606898	0.01149302	PCTP	0.494965635
NHLRC2	1.248905666	3.9206E-06	VEZF1	0.388800388
ADRB1	1.39860901	0.00042005	DYNLL2	0.363742418
SHTN1	0.552690773	8.8097E-07	TEX14	1.297605528
WDR11	0.494505508	0.01626534	MAP3K3	0.705426013
RP11-45A17.2	2.337793557	0.02752051	CCDC47	0.220132356
KDM5A	0.551502691	0.000011456	ERN1	0.376055103
CCDC77	1.322633379	0.00949115	PECAM1	0.507412359
ITFG2	0.795951804	0.00437139	DDX5	0.586845222
LTBR	0.425568956	0.00395088	FAM20A	1.284416257
MRPL51	0.283191496	0.01175207	H3F3B	0.33633079
ING4	0.754481319	0.00218881	UBALD2	0.561479158
TPI1	0.17738533	0.00546083	RHBDF2	0.368942839
SPSB2	1.067725777	0.01345009	TNRC6C	1.059683063

PTPN6	0.281759335	0.02172877	SOCS3	0.682895588
CLEC4A	0.464653937	0.00937122	USP36	0.520912624
CLEC4D	1.113953746	0.03334137	EIF4A3	1.050027716
CLEC2D	0.704011063	0.04303899	BAIAP2	0.521971399
RP11-290C10.1	2.716064291	0.00023521	RFNG	0.46885675
CLEC12A	1.470805036	5.6052E-10	WDR45B	1.029262433
CLEC9A	0.650692412	0.00552896	IL4I1	1.876036338
CLEC7A	0.49174379	1.3173E-07	TGIF1	0.483562618
PRR4	1.180694336	0.00553891	PTPRM	1.571005876
LOH12CR2	2.175770955	2.0915E-06	ANKRD12	0.246171666
ARHGDIB	0.28106722	0.00047195	RAB31	0.447474302
RP11-707G18.1	1.718861632	0.000005695	CHMP1B	0.581516573
FAM60A	0.404507314	0.03170531	FAM210A	0.641787727
RP11-467L13.7	3.192462202	2.7089E-18	RBBP8	1.198541369
KIAA1551	1.199072085	4.3121E-12	CABLES1	1.098544228
BICD1	1.27789442	0.00439381	DSC2	0.456144787
AC079630.4	1.418322104	0.04500251	TCF4	0.52053155
LRRK2	0.96868359	0.000029979	TNFRSF11A	1.688112045
PUS7L	0.71495285	0.00052004	KDSR	0.378693338
IRAK4	0.660341863	0.00217412	NFATC1	0.859902819
RP11-352M15.2	1.146294202	0.00037039	ADNP2	0.486575263
SCAF11	0.423363393	0.00032556	C20orf194	0.894508778
SLC38A1	0.987641088	0.00557267	SIGLEC1	0.883988712
ASB8	0.530327155	0.03144674	PRNP	0.792106817
RP11-370I10.12	2.076015529	0.00211235	BMP2	1.680625263
ZNF641	1.303660409	7.1816E-06	KIF16B	0.553574275
RP11-386G11.5	1.126162026	0.03593252	RIN2	0.522948265
PRKAG1	0.667579106	0.00078541	RALGAPA2	0.733792571
RP11-977B10.2	4.092571137	2.659E-11	CD93	0.749281368
BCDIN3D	1.472723772	0.00167413	TP53INP2	1.256886189
COX14	0.292262925	0.00168559	RBM39	0.356356028
TFCP2	0.681433488	0.00720218	LINC00657	0.333353896
SLC4A8	1.408734343	0.02916232	MAFB	0.529968783
PRR13	0.3181162	0.00093997	TOP1	0.19555626
RP11-793H13.11	2.073645322	0.000068624	SRSF6	0.337681622
ATP5G2	0.125293277	0.0372102	OSER1	0.436745585
CALCOCO1	0.642794518	0.04985657	SERINC3	0.477654071
CBX5	0.583655044	0.0071487	NEURL2	1.305703737
ZNF385A	0.33136127	0.02832017	PLTP	0.9070402
RPL41	0.225030979	0.00211298	ZNF335	0.708755554
RNF41	0.557365038	0.03370963	SLC12A5	0.843876989
STAC3	0.417054706	0.04922283	NCOA3	0.582232976
ARHGAP9	0.52642876	0.00118177	PREX1	0.411019835
DCTN2	0.337812903	0.04323793	STAU1	0.318348258
XRCC6BP1	0.940961771	0.01692257	DDX27	0.36681064
USP15	0.340183088	0.00536622	CEBPB	0.625999058
LEMD3	0.817655489	0.01429275	PCMTD2	0.579320815
NUP107	0.869568153	0.00232387	FSTL3	1.02470881
RP11-1143G9.5	2.593464423	0.00024081	PTBP1	0.387415982
LYZ	0.510350926	0.00046483	CFD	0.564598571

LINC01481	0.878793402	0.01319415	MIDN	0.549506084
GLIPR1	0.29341843	0.01172832	MKNK2	0.562244791
BBS10	1.177307254	0.01837721	GADD45B	0.606702882
ZDHHC17	0.596584888	0.0234794	GNA11	1.570245038
RP11-823E8.3	1.698238987	0.02060388	NFIC	0.447747491
LINC00936	1.15127072	0.00014892	LRG1	0.613646036
RP11-796E2.4	2.277651765	9.747E-14	SEMA6B	0.58865444
RP11-693J15.5	1.684426175	0.02972796	TICAM1	0.525970626
CEP83-AS1	2.367358886	0.04386113	MLLT1	0.643893887
HAL	3.164634229	0.0057609	VAV1	0.553847934
LTA4H	0.480267195	0.00889676	INSR	0.405126424
APAF1	0.873756196	2.3632E-06	ZNF358	0.585710688
ACTR6	0.657743127	0.00662014	CD209	0.44036148
C12orf42	2.217883853	0.00465089	ZNF812P	1.393411112
NT5DC3	1.651148439	0.029614	ZNF846	0.577453098
C12orf45	0.537377344	0.00753494	ICAM1	1.254023319
ALKBH2	1.041241159	0.00354936	ICAM4	1.37521163
TCHP	0.651668414	0.0349273	ICAM5	1.707846724
GIT2	0.50702817	0.02650554	LDLR	1.097432877
ANAPC7	0.645129808	0.03707399	KANK2	1.707297836
ATXN2	0.414165345	0.04968739	ZNF136	0.568530721
MAPKAPK5-AS1	0.874836061	3.9113E-06	JUNB	0.782152818
TMEM116	1.548805092	0.00036709	TRMT1	0.613609898
NAA25	0.821918875	0.00141124	IER2	0.926669969
TRAFD1	0.545798958	0.04519259	CTD-3252C9.4	0.681950312
RPL6	0.411606222	2.2529E-14	ADGRE5	0.429242923
RPLP0	0.209490568	0.00569899	DNAJB1	0.793167385
TRIAP1	0.608379428	0.00346741	BRD4	0.235208867
GATC	0.707542874	0.02122234	KLF2	1.132620433
POP5	0.57926201	0.00032929	SIN3B	0.613290301
UNC119B	2.038104025	0.00767787	F2RL3	1.634276682
C12orf65	1.380514967	9.1092E-07	CPAMD8	2.117529224
RP11-486O12.2	2.244458722	0.02932935	BST2	0.177903954
BRI3BP	0.524883477	0.00263322	COLGALT1	0.404829434
EP400NL	0.891942623	0.00571258	KIAA1683	1.958138413
RP11-46H11.12	3.095267879	0.00016491	JUND	0.894363128
ZNF605	1.873798995	8.5046E-06	HOMER3	0.893773025
ZNF84	0.784172091	0.00753494	TSHZ3	1.437823079
MPHOSPH8	0.593001244	1.7153E-07	LRP3	1.590946292
ZMYM5	0.684316332	0.00224065	KIAA0355	0.505264547
CENPJ	0.897954616	0.01212669	SCN1B	1.419361575
FLT3	1.611286223	1.6175E-06	NFKBID	0.838107249
PAN3	0.504132106	0.01046346	NFKBIB	0.604925152
HMGB1	0.204193122	0.00020121	MED29	0.276260702
USPL1	0.631403132	0.04614283	ZFP36	0.709207283
N4BP2L2	0.570165617	1.1388E-07	PLEKHG2	0.981233169
RFXAP	1.446630314	0.01000755	FCGBP	2.121242775
NHLRC3	0.619032583	0.00080048	SERTAD1	0.413076982
MRPS31	0.791341352	0.00049703	BLVRB	0.35478141
KBTBD7	1.038332989	0.01620001	HNRNPUL1	0.459343371

LCP1	0.203538982	0.00914251	TGFB1	0.325194965
ESD	0.243828322	0.01976596	IRGQ	0.726614366
LPAR6	0.445671008	0.000598	PLAUR	0.685383045
SETDB2	0.875782582	7.9265E-06	BCAM	1.040517719
ARL11	0.685953108	0.00335666	FOSB	1.241593089
TRIM13	0.550348778	0.04069294	C5AR1	0.817521801
DLEU1	0.900739492	0.04736623	C5AR2	0.638797512
INTS6-AS1	1.7325809	0.00253196	DHX34	0.900971944
WDFY2	0.804733615	3.4129E-06	PPP1R15A	0.889860996
COMMD6	0.407331436	3.6545E-08	RCN3	0.785154936
RNF219	1.018674357	0.00011709	SIGLEC8	1.156392185
DNAJC3-AS1	1.346335241	0.01326739	ZNF331	0.621386065
GPR18	1.632797581	0.0306931	MYADM	0.926084661
PCCA	0.767438971	0.03449255	LILRB5	1.079753912
ARGLU1	0.32852876	0.00392441	LAIR1	0.289734267
LIG4	1.148889036	0.00157812	IGLC3	2.375604232
COL4A2-AS2	2.314417761	0.00103743	TPST2	0.663778775
ANKRD10	0.504637042	0.00178844	XBP1	0.886411329
PCID2	0.571515647	0.02353935	CTA-292E10.6	0.900094446
UPF3A	0.452695768	0.00532782	AP1B1	0.325885057
CCNB1IP1	0.628671617	0.01163605	NCF4	0.30086515
RPPH1	1.216899699	0.00095737	CYTH4	0.397260045
APEX1	0.218395645	0.01667029	MAFF	1.220925755
NDRG2	0.999503656	3.2949E-06	DDX17	0.314555451
METTL3	0.933949975	0.02508581	PDGFB	1.014296957
PPP1R3E	1.401231396	0.00840351	ATF4	0.499677052
PSME1	0.302576147	0.00254157	EP300	0.451862913
PSME2	0.273764455	0.03203639	PRR5	1.971054644
LTB4R	0.809758955	0.01257979	PLXNB2	0.570726141
G2E3	0.640083859	0.01835917	NRIP1	0.529380916
SPTSSA	0.315889655	0.03350779	BTG3	0.584198301
PNN	0.524972945	0.00053395	MIR155HG	1.141871946
RP11-407N17.5	2.132099304	0.02940357	GABPA	0.439621773
PRPF39	0.906735098	0.00476537	USP16	0.378365276
MIS18BP1	0.500315293	0.006873	IL10RB-AS1	0.446503459
RPS29	0.262863374	0.00546083	SON	0.152983242
RP11-596C23.2	2.757189218	6.7655E-11	ITSN1	0.598715632
ATL1	2.305305001	0.01622671	RUNX1	0.613193921
TMEM260	0.820252514	0.02511813	DYRK1A	0.547760661
RTN1	0.597528363	0.0029697	ETS2	0.320643298
CTD-2302E22.4	2.595429448	0.02660995	ZBTB21	0.620746252
CTD-2302E22.6	2.679385429	0.00196147	SIK1	0.738301518
SYNJ2BP	0.779817192	0.00379212	ICOSLG	0.700438832
RP6-91H8.3	1.241038002	0.00028857	DIP2A	0.481864266
RBM25	0.289513289	0.00098619	MT-ND1	0.415782531
PTGR2	0.961363933	0.02282221	MT-ND2	0.319742506
ISCA2	0.488894277	0.03951971	MT-CO3	0.174354332
MLH3	1.04982157	0.0003436	MT-ND3	0.344765802
LINC01220	1.41104521	0.00937495	AC240274.1	0.804136047
VIPAS39	0.70214706	0.03820838	GARNL3	1.640846253

CTD-2506P8.6	1.571434237	0.01991619
C14orf159	0.648500778	0.00037199
ATXN3	0.472156716	0.01022788
RIN3	0.44625036	0.01895488
ASB2	1.520074772	1.6573E-09
SERPINA1	0.719841663	0.00652612
DICER1	0.336024518	0.04202674
RP11-872J21.3	1.739025069	0.00040309
YY1	0.271833147	0.01630254
RP11-1017G21.5	1.354567721	0.00010177
PLD4	0.787908936	5.6563E-06
HERC2	0.567569559	0.03288655
GOLGA8R	2.613525992	0.0025757
ARHGAP11B	2.112823443	0.000013095
RP11-1000B6.3	2.046911957	1.7816E-09
GOLGA8A	0.823522276	0.01291389
ZNF770	0.646476755	0.00605515
RP11-1008C21.2	1.558586692	0.03138973
RP11-624L4.2	1.608984376	0.000050663
RP11-37C7.3	2.744869257	0.00554273
SPINT1	0.64858679	0.005632
GANC	0.871699107	0.00103401
CAPN3	1.244444921	0.03512894
ZNF106	0.516805211	3.4753E-06
SNAP23	0.447034912	0.00595787
LRRC57	0.941153615	0.01457596
PPIP5K1	1.554441528	0.01170982
HYPK	1.857589931	6.4977E-08
RP11-96O20.5	1.888646787	0.00854878
EID1	0.285809248	0.00029355
DTWD1	0.668166058	0.01271606
ATP8B4	0.685333153	0.00012515
GABPB1-AS1	1.415847135	3.1584E-14
AP4E1	1.111181623	0.00115329
PIGBOS1	0.783266354	0.00010412
SLTM	0.304085866	0.01096913
MTFMT	0.578083608	0.01936172
PDCD7	0.75641192	0.000026772
CLPX	0.4739153	0.04082445
DPP8	0.488026128	0.02583287
RPL4	0.202599716	0.01709612
C15orf61	0.57816103	0.02672031
ANP32A	0.469814838	0.00026519
RPLP1	0.131675712	0.03675981
HEXA-AS1	2.763465966	3.7745E-07
UBL7-AS1	1.395093656	0.000027088
CSK	0.462704325	0.00822737
FBXO22	0.646668589	0.0320709
PSTPIP1	1.468457189	7.6054E-07
PSMA4	0.200617317	0.02481454

LTC4S	1.76409249
ZFY	1.243484282
DDX3Y	1.135230356

RP11-16K12.1	2.623410806	0.01487377
RPS17	0.183165938	0.00572538
WDR73	0.768770537	0.00226278
ARPIN	0.497641939	0.00643438
RP11-617F23.1	2.883918113	0.01275833
RP11-387D10.4	2.701386496	0.01977352
FES	0.372942049	0.03370963
CTD-2313J17.6	1.368546997	0.0073994
RHOT2	0.487080627	0.02804981
NME3	0.296752322	0.00973082
MRPS34	0.293458451	0.0237351
SPSB3	0.517146778	0.01602778
FAHD1	0.816386312	0.00048158
MSRB1	0.626879714	0.02398121
RPS2	0.267336314	0.00115329
SNHG9	0.591216967	0.01360345
PGP	0.414047735	0.00572854
RP11-473M20.16	2.820503385	0.00014892
ZNF75A	0.589479715	0.04405705
ZSCAN32	1.245124562	0.00701155
DNASE1	1.428704421	0.01210334
NUDT16L1	0.540501101	0.02638785
ANKS3	1.320349828	0.02588055
ZNF500	1.738059907	0.00043114
ROGDI	0.524957381	0.0479993
TMEM186	1.116519437	0.01727818
CIITA	0.894219587	3.6078E-07
RP11-490O6.2	1.372336809	0.00247772
RSL1D1	0.33320818	0.00014892
CPPED1	0.378929504	0.03620457
CTD-2135D7.5	3.044108113	0.000052668
KIAA0430	0.532761552	0.02664631
NDE1	0.738806303	0.02361615
RPS15A	0.231683081	0.00080084
COQ7	1.658985085	2.1762E-11
KNOP1	0.577749774	0.00897268
SDR42E2	1.516965746	0.00964233
PRKCB	0.459299116	0.02930513
FLJ21408	2.210446168	1.3687E-06
APOBR	0.574082241	0.0071487
RP11-1348G14.8	1.819761794	0.04925791
SH2B1	0.861221282	0.02275679
NFATC2IP	0.376449556	0.02915228
RP11-426C22.5	0.697434279	0.00219034
SPN	0.777911029	0.00125466
KIF22	0.678235229	0.02769641
CDIPT	0.503962199	0.01692257
CORO1A	0.535218017	2.3313E-10
SULT1A3	1.642906301	0.00486963
DCTPP1	0.566113691	0.00202651

ZNF785	0.980574503	0.04060958
PYCARD	0.341244902	0.00138973
RP11-452L6.5	1.368751756	0.01125279
GPT2	1.219390526	0.04274744
RP11-212I21.4	1.619139375	0.00092547
CKLF	0.556397302	4.1207E-06
CMTM4	2.348995101	0.04791945
KIAA0895L	1.25605095	0.03726035
RP11-297D21.4	1.971009333	0.000011565
PSKH1	1.292082852	0.02915228
PSMB10	0.323944384	0.03474998
SLC7A6	1.196105102	0.00987198
SLC7A6OS	0.520484956	0.0334175
RP11-252A24.3	1.294928795	0.02826132
GABARAPL2	0.29212122	0.04387583
MLYCD	1.028690262	0.00325821
TLDC1	1.429480764	0.00967298
COTL1	0.376584472	0.0042799
ZDHHC7	0.576854166	0.00180166
C16orf74	2.955226924	0.00136699
COX4I1	0.150081021	0.02067652
SNAI3	1.404012996	3.2406E-07
APRT	0.217451437	0.04498274
CBFA2T3	1.580088465	0.000086648
ACSF3	0.57474374	0.04242992
RP11-46C24.7	1.844882499	0.02000319
RPL13	0.275832929	0.00070128
CDK10	0.770820093	0.00328092
FANCA	1.260173725	0.00083428
CENPBD1	1.135198178	0.00574967
SERPINF2	1.427827818	2.2524E-06
NCBP3	0.568770273	0.02045459
P2RX1	1.582668695	0.02309646
ATP2A3	1.677436458	0.00213736
CYB5D2	0.785924737	0.0036671
SPNS3	1.124147343	0.0033701
MED11	0.63412185	0.00390434
RP11-333E1.1	1.848491994	0.00106814
SCIMP	0.670393629	4.2111E-07
NLRP1	0.691261808	0.00220158
KIAA0753	1.419610869	0.00624348
XAF1	0.649487429	0.00023086
CLEC10A	0.825939589	0.000001905
ASGR2	0.800593649	0.01994836
ASGR1	0.698220356	0.00125601
PHF23	1.10147499	0.0002486
EIF5A	0.272837512	0.03621406
NEURL4	1.224457774	0.03381087
ACAP1	0.939495579	0.00463524
CHRNB1	0.923704207	0.02378832

NAA38	0.254682247	0.02916232
BORCS6	0.911109276	0.00370325
LINC00324	1.115928389	0.00013402
RPL26	0.282386947	0.00043005
SCO1	0.587121762	0.02421074
ADPRM	0.864609981	0.04734902
TRIM16	1.373963574	0.02177907
ADORA2B	1.311844474	0.03200878
ZSWIM7	0.401760998	0.0146102
NCOR1	0.354868941	0.00507604
RP11-138I1.2	1.5835238	0.04512941
ZNF624	1.458809704	0.01460677
CCDC144A	1.059622754	0.00777975
COPS3	0.301406329	0.03842961
ATPAF2	0.522809872	0.01692862
SPECC1	0.542233994	0.00360147
IFT20	0.653320292	0.000032254
UNC119	0.459239443	0.04802199
PROCA1	1.331072213	0.01082006
RPL23A	0.282173898	0.000075612
NEK8	1.578342294	0.04838518
PHF12	0.630359137	0.03828754
SSH2	0.492962856	0.00924731
CRLF3	0.814393588	0.00082531
ATAD5	1.259485425	0.00047011
RNF135	0.575475398	0.00246606
EVI2A	0.268184775	0.04493442
RAB11FIP4	1.393331274	0.03758794
UTP6	0.527982821	0.00104653
SUZ12	0.39763288	0.01131119
SLFN12	0.902095531	0.00149029
TAF15	0.381827898	0.00613194
GGNBP2	0.431915086	0.00061256
MRM1	2.045649829	0.00025222
TADA2A	1.203793071	0.00146275
RPL19	0.216147373	0.0053224
STAT5A	0.517026319	0.03531787
RP11-400F19.6	1.238398981	0.01966741
EZH1	0.917561656	0.00020842
RUNDC1	0.937291557	0.01360057
RPL27	0.206624995	0.00470997
BRCA1	1.222012517	0.04172974
RP11-242D8.1	0.989890887	0.04569491
ARL4D	1.549586647	0.00104324
C17orf105	3.689746973	0.0281807
RP5-882C2.2	2.054012367	3.4165E-06
DCAKD	1.232620136	0.01354564
ACBD4	1.092375947	0.02930605
AC142472.6	1.375315825	0.04653758
ARL17A	1.049203917	0.01529601

NPEPPS	0.630268117	0.00059694
KPNB1	0.440632087	0.000031397
SNX11	0.88998157	0.000034005
ATP5G1	0.363726047	0.00203939
ABI3	0.280917015	0.04323793
ZNF652	1.02030384	6.3259E-06
SPOP	0.681582496	8.089E-07
LUC7L3	0.987692775	8.8839E-16
C17orf67	1.414103342	0.00117969
COIL	0.638684298	0.02307141
MRPS23	0.392501204	0.02973016
SRSF1	0.715844883	0.000029256
SUPT4H1	0.283627167	0.00714325
RNF43	2.591485364	0.04812416
YPEL2	0.432759006	0.03097832
RP11-758H9.2	2.246127724	2.5176E-15
VMP1	0.610555529	0.000083179
TUBD1	1.312198661	0.00988077
HEATR6	0.654809675	0.04512941
DCAF7	0.374129571	0.01858684
LIMD2	1.492892311	3.1875E-14
SMARCD2	0.574224097	0.01381868
POLG2	1.492337038	0.00000943
ABCA6	2.592876521	0.00795408
CD300LB	0.957429731	0.00242949
NAT9	0.933459512	0.0033701
ARMC7	1.006318035	0.01720162
MRPS7	0.400872672	0.00143198
RP11-318A15.2	1.756754975	0.03020709
RNF213	0.295833043	0.03096602
ACTG1	0.253145507	0.00243489
OXLD1	0.904686463	0.000057312
SIRT7	0.620181459	0.00280975
CCDC57	0.663717799	0.01442712
RP13-516M14.4	1.219892167	0.00379609
C17orf62	0.591246879	8.3261E-06
FN3KRP	0.651505731	0.02769179
RP13-270P17.3	1.727767767	0.00024727
MYL12A	0.186527259	0.01316666
IMPA2	0.624224261	0.00013866
RNF125	0.648683269	0.00412214
ZNF397	1.45274137	1.1977E-07
ZNF396	1.741573335	0.02244806
PSTPIP2	0.642100728	1.9041E-06
ATP5A1	0.226017123	0.00595787
MIR1539	2.905669361	0.0161649
POLI	0.978866475	0.03383633
RP11-35G9.3	1.131068234	0.00502208
ALPK2	1.631154131	0.00117208
TMX3	0.503884811	0.00583101

LINC00909	1.058397411	0.00090088
MBP	0.450758608	0.00078092
RP11-567M16.6	2.075428269	6.4775E-06
RBCK1	0.367242751	0.0186328
CSNK2A1	0.45345136	0.00564778
FKBP1A	0.25966664	0.00064573
SIRPB1	1.418058821	0.00106945
C20orf27	0.700861897	1.9991E-11
MAVS	0.912691814	0.000007981
RP11-119B16.2	1.648577896	0.00144611
TMEM230	0.280620935	0.01450159
ESF1	0.635527498	0.00054643
MGME1	0.80049927	0.000021029
NAPB	1.33027568	0.02093981
CST3	0.294591213	0.00873169
PDRG1	0.791999927	0.00334719
TRPC4AP	0.489352758	0.03531787
SAMHD1	0.938348495	2.9261E-13
L3MBTL1	2.227910615	0.00217529
DNTTIP1	0.449880638	0.03566614
ZNF217	0.614540639	0.01085067
RAE1	0.748124864	1.9709E-07
VAPB	0.790787407	0.03814684
STX16	0.593289804	0.03976264
NPEPL1	0.740011988	0.01130386
PPP1R3D	1.192167846	0.00301636
RPS21	0.250556836	0.00532782
RGS19	0.585026251	0.00012994
CNN2	0.41752091	0.0008606
POLR2E	0.269000387	0.03145979
RPS15	0.23583645	0.00034457
C19orf25	0.483022229	0.03576865
PLEKHJ1	0.595115571	0.00052478
C19orf35	1.334969177	0.00662364
LSM7	0.309352607	0.00285192
S1PR4	2.090978807	5.6618E-09
ARRDC5	1.442211766	0.01494912
RPL36	0.180200901	0.01983975
CTC-232P5.3	1.819929418	0.049408
INSR	0.59507779	0.00437139
RETN	1.572312514	0.02381529
PRAM1	1.148271004	0.000021248
MYO1F	0.397919941	0.00019617
ZNF559	1.39947878	0.00211298
ZNF121	0.692236986	0.03008572
ZNF562	0.99243798	3.6078E-07
MRPL4	0.34808545	0.03369867
ICAM3	1.009676085	0.00011243
TYK2	0.709554863	0.00058134
ILF3-AS1	1.724045147	1.4778E-13

C19orf38	0.60693288	0.00115329
RAB3D	1.596510316	0.03536146
SWSAP1	1.815158099	2.9414E-06
ZNF439	1.693631718	2.8348E-06
ZNF700	1.221849199	0.0008606
ZNF763	2.002809147	0.03304975
ZNF564	1.518045683	0.04208707
ZNF791	0.966538499	0.00000287
WDR83OS	0.250262918	0.00197698
NFIX	1.978902262	0.00620482
LYL1	0.96637963	6.3279E-08
STX10	0.804209941	1.772E-07
CCDC130	0.642859388	0.03097426
MRI1	0.696925477	0.04256508
C19orf53	0.246865759	0.01032317
DCAF15	0.8674542	0.00537329
RAB8A	0.376449123	0.01053309
HSH2D	1.883724982	0.000043838
FAM32A	0.516424256	0.00021044
CTD-2562J15.6	1.912960372	0.02519817
CTD-3222D19.8	1.242347247	0.00527385
SMIM7	0.348595499	0.00665351
BISPR	0.983922874	0.00046278
PGLS	0.299198017	0.00372266
FCHO1	1.30737626	0.000093996
JAK3	0.831953365	0.02949522
RPL18A	0.367984312	9.7257E-08
LSM4	0.316067332	0.01937213
LRRC25	0.378511872	0.00101944
FKBP8	0.212461707	0.0490333
AC005253.2	2.120008247	0.0002983
SUGP2	0.914687675	5.7184E-06
ZNF101	1.10191552	0.02234677
ZNF253	2.246746461	0.000047577
ZNF93	1.43323622	0.00523474
ZNF431	0.685665061	0.01541421
ZNF708	1.276671985	0.0050974
ZNF493	1.541239045	0.00022647
ZNF43	1.054853885	0.01548423
ZNF91	0.687477074	0.00702778
ZNF675	0.960534142	0.00055853
URI1	0.404904074	0.00491105
FAAP24	1.312166705	0.01282659
ZNF302	0.890306025	0.02128864
HCST	0.405125329	0.01555509
SDHAF1	0.814718709	0.00593296
ALKBH6	0.820026796	0.03522379
CAPNS1	0.312425815	0.00426455
ZNF146	0.769638416	0.00138704
LINC00665	1.371448443	0.04671909

ZNF567	1.266203486	0.00702778
ZNF573	1.413753655	0.01666133
SPINT2	0.480835967	0.00095708
KCNK6	1.040936749	1.4711E-11
MAP4K1	0.869874694	0.00082733
EIF3K	0.215854514	0.00019794
IFNL1	2.781811984	0.02318931
GMFG	0.262880285	0.021183
FBL	0.31706027	0.03892632
ZNF780B	0.95851891	0.00806104
SHKBP1	0.363397561	0.03016994
SNRPA	0.429630873	0.0161649
EXOSC5	0.622259561	0.00068915
ATP5SL	0.638139281	0.00924731
CEACAM4	1.70256373	1.4681E-09
RPS19	0.315265512	0.00047205
POU2F2	1.037434246	7.7592E-08
ZNF230	1.723425452	7.2649E-07
ZNF223	1.599967524	0.01904145
ZNF224	1.197861095	5.1109E-07
TRAPPC6A	0.347647112	0.04208707
BLOC1S3	1.072654423	0.02080981
CD3EAP	0.752485625	0.0334203
ERCC1	0.287638467	0.03203639
SNRPD2	0.385338975	2.3949E-06
PPP5D1	1.333136077	0.04467627
CARD8	0.585747429	0.000075612
RPL18	0.192625678	0.00729143
CD37	0.739011236	1.1831E-09
RPL13A	0.197127296	6.9162E-07
IRF3	0.6299834	0.00049746
PTOV1-AS1	1.825916239	0.04749821
NAPSA	2.896519919	0.00283021
EMC10	0.39194327	0.03240814
CD33	0.466110585	0.00053042
ZNF880	0.809679874	0.02978585
ZNF701	1.101180118	0.02588055
ZNF83	0.985492342	0.00023549
ZNF816-ZNF321P	1.773070312	0.02249123
ZNF525	1.143843124	0.03780664
NLRP12	2.800141654	0.00516081
OSCAR	1.149587416	0.000019534
LENG8-AS1	1.50169666	0.00417934
LENG9	1.250456422	0.00639842
RPL28	0.221606911	0.0003584
SSC5D	1.956644778	0.02623255
ZNF524	0.505098964	0.02789118
ZIK1	1.656042838	0.02623255
ZNF211	1.020564521	0.02829208
ZNF671	1.893052613	0.00334231

ZNF606	1.416738385	0.03383633
RPS5	0.219029508	0.01212669
ZNF324B	1.634810286	0.03054277
MZF1-AS1	2.785833695	2.5454E-11
CECR1	0.309694001	0.04298615
TANGO2	0.587278274	0.03181519
XXbac-B135H6.15	0.691220707	0.04191431
YDJC	0.618952651	0.00712991
PPIL2	0.738863436	0.00306395
LL22NC03-2H8.5	2.335536715	0.00021932
SNRPD3	0.221441993	0.02118635
HSCB	0.675188222	0.00025739
ZMAT5	0.563835615	0.00941588
PIK3IP1	0.361519544	0.04573168
PRR14L	0.671602533	0.0005568
APOL3	0.887914459	0.000037304
APOL4	0.884753571	0.00249097
APOL2	0.793622444	0.00792097
TXN2	0.567790288	0.000081284
RAC2	0.732281216	1.1625E-11
MFNG	0.776346205	2.2373E-07
LGALS2	1.33836963	0.00031151
EIF3L	0.314598107	0.00026174
CTA-228A9.4	1.502197152	0.01173204
RPL3	0.180960435	0.03324948
TNRC6B	0.502105207	0.0008863
MKL1	0.979396665	0.00541252
PMM1	0.502816956	0.0208416
C22orf46	1.086062142	0.00366045
RP5-821D11.7	1.919878004	0.000070804
SMDT1	0.284403233	0.00895185
TSPO	0.693169903	0.00464827
PARVG	0.663449647	5.732E-07
NUP50	0.623871365	0.00383174
KIAA0930	0.580538828	0.00055505
UPK3A	1.676935966	0.01130386
FAM118A	1.185606948	5.0056E-08
TRMU	1.122719586	0.00253083
CERK	0.979309444	0.00002189
CTA-29F11.1	0.891855367	0.00077892
TBC1D22A	0.449317059	0.01946672
RP1-29C18.10	2.916325391	0.00964114
DENND6B	0.458266928	0.01645213
CTA-384D8.35	1.568208633	0.02663555
CTA-384D8.34	1.255786425	4.5728E-06
PAXBP1-AS1	1.445194817	0.0017261
KCNE1	1.781628494	5.2958E-08
AATBC	2.391595528	0.00514808
ITGB2	0.368983122	0.00018119
ITGB2-AS1	1.334741452	6.5335E-08

LL21NC02-1C16.2	2.613542543	0.0215408
C21orf58	1.811073476	0.01429655
DIP2A	0.444176443	0.01867093
PRMT2	0.521216841	2.8303E-07
LINC01354	2.620457171	0.02820825
LGALS8-AS1	3.785557455	7.6834E-08
RP11-214N9.1	1.864964312	0.00862687
ZNF501	2.017146794	0.04534293
FAM53A	2.480501436	0.03520817
RP11-629B11.5	2.986194185	0.02254608
CTD-2165H16.4	2.057966644	0.00325622
RP1-168L15.5	2.44123935	0.00399862
AC004540.5	2.727233882	0.00535167
SOWAHD	1.109671442	0.00550058
SMC5-AS1	1.783998982	0.04197021
RP11-152N13.5	2.482603216	0.00185278
RP11-47A8.5	1.192611965	0.04069294
PRMT5-AS1	2.512981093	0.0044283
RP11-471B22.3	2.703576413	0.00120235
CEBPA-AS1	1.927126188	0.04448524
RP11-196G18.3	3.046682957	0.00065229
LINC01126	2.425468303	0.03049534
AC105760.2	2.813247174	0.00230126
CTD-2256P15.1	3.113168324	0.0017946
CTC-209H22.3	3.554402072	0.03203639
FRS3	1.697947845	0.01515302
HMGN3-AS1	1.920665551	0.03431075
VGF	3.317792005	0.01323984
GHET1	3.091662056	0.00388715
RP11-351D16.3	1.993642076	0.04753551
RP11-120K19.4	2.572769608	0.01268976
RP1-28H20.3	3.127167815	0.01389366
RP11-262H14.3	2.028736814	0.04076691

padj		gene	M8 vs. M6 + M7<br>log2FoldChange	padj
0.03255297		HES4	0.679322917	0.021775815
0.002282943		ISG15	0.561551176	0.00203369
0.000100484		TNFRSF18	1.726378448	0.000559572
3.61165E-07		SDF4	0.2628418	0.032060824
4.94973E-06		B3GALT6	0.465451566	0.049879637
0.003273801		AURKAIP1	0.31203845	0.001320376
0.00022984		MRPL20	0.347577474	1.01152E-05
0.032188292		NADK	0.475062044	0.000885943
0.013237275		RER1	0.343148241	0.003806799
0.012716122		RP3-395M	0.661792069	0.012672708
0.001114356		TNFRSF14	0.245684436	0.016141026
0.00473302		KLHL21	0.730608417	0.020057777
0.014581794		TNFRSF9	1.225865192	0.025137854
1.20496E-05		PARK7	0.415108745	1.07876E-08
0.01589645		ENO1	0.610831359	7.6143E-11
0.010497513		GPR157	0.925903403	0.016270717
0.002593341		SPSB1	1.270159594	0.004355735
0.007045974		PGD	0.717069177	4.83411E-08
0.03973724		SRM	0.411514833	0.012037284
0.002915367		EFHD2	0.493974606	1.03304E-06
0.0038377		SDHB	0.464140287	2.15986E-05
0.002151583		PADI2	1.417226544	0.029150084
0.001368679		CAPZB	0.27645682	2.12083E-06
2.30998E-05		MINOS1	0.292459756	0.003197793
0.012421526		DDOST	0.468626297	7.84807E-06
0.000371194		C1QB	0.349377259	0.022741364
0.001871768		LYPLA2	0.59226314	0.002999793
0.007629595		GALE	0.86643372	0.026297465
0.007420769		FUCA1	1.445473123	0.001657645
0.000378179		RUNX3	0.629123666	7.15419E-05
0.017743924		TMEM50A	0.310955552	0.001914717
0.012392673		DHDDS	0.45526839	0.04206327
0.001871768		ZDHHC18	0.576601162	0.009140369
0.000467597		NUDC	0.458771552	1.43454E-05
0.003132541		IFI6	0.566226791	0.000157599
2.08397E-06		DNAJC8	0.316720087	2.77437E-05
0.038879088		SESN2	0.683369787	3.5638E-05
1.55766E-09		RAB42	2.856257532	1.02336E-17
0.001152887		FABP3	1.550535225	0.002891684
5.90168E-05		PTP4A2	0.3448396	0.000879313
8.85314E-05		EIF3I	0.378346378	0.000162054
0.0075844		MARCKSL1	0.767587783	2.12617E-09
0.0003106		PSMB2	0.563303938	4.71812E-11
0.044706332		STK40	0.502745316	0.024009501
0.006129154		MRPS15	0.354355384	0.015234135
0.005921368		YRDC	0.598813616	0.014279581
2.97335E-05		C1orf122	0.514394451	0.001302249

0.006338349	NDUFS5	0.27094284	0.000808844
0.001991462	OXCT2	0.741993158	0.000243565
0.002269284	MFSD2A	0.77192355	0.045376112
0.0203243	CAP1	0.278762214	2.03456E-05
0.00057482	PPT1	0.33905425	1.25391E-08
0.026845682	YBX1	0.285715934	4.47727E-07
0.023491483	ATP6V0B	0.319145232	5.08677E-07
0.03415515	UROD	0.354636807	0.02912048
0.017177844	PRDX1	1.109905043	3.0287E-28
0.000696322	AKR1A1	0.533513821	1.14401E-11
1.95745E-05	UQCRH	0.182538431	0.003476029
0.001543989	ELAVL4	0.679238408	0.017781229
3.67744E-05	PDE4B	0.386449754	0.030991358
0.000103463	SERBP1	0.251803735	0.002179453
2.59455E-11	GADD45A	0.663419541	0.024796179
0.019121972	GNG5	0.349839568	7.17415E-09
0.002800079	MCOLN2	1.347178145	5.74213E-06
0.018185323	GBP2	0.538970351	0.000664682
0.020942605	PSMA5	0.379995842	0.000141234
0.036469944	GSTM4	0.82743832	1.47111E-06
3.37504E-07	LAMTOR5	0.420606594	2.05769E-07
3.0764E-10	ATP5F1	0.260865753	0.000995385
7.90828E-08	RAP1A	0.185529125	0.005644946
0.009476191	OLFML3	1.398421234	4.06658E-05
0.0004324	NRAS	0.422870848	0.029735193
0.002282943	RBM8A	0.25186599	0.027224397
0.029819109	PDE4DIP	0.590156755	0.000470087
2.21972E-05	C1orf54	0.297371337	0.006126687
1.0927E-07	ENSA	0.286456799	0.005415307
0.000366038	CTSS	0.171605402	0.000686095
0.007177515	PSMB4	0.414535295	8.19946E-06
1.16997E-08	S100A10	0.606237648	0.000926132
3.03303E-06	S100A11	0.678264651	2.49134E-24
0.004420499	ILF2	0.351774915	0.001104059
4.9576E-10	TPM3	0.243139579	0.00077643
0.000253268	MTX1	0.391176095	0.009229769
2.97301E-15	GBA	0.487692424	0.009329172
0.007372638	FDPS	0.358691468	0.003870422
1.49226E-08	SSR2	0.259409868	0.003668513
1.43908E-06	LAMTOR2	0.248505469	0.012640607
1.31307E-09	GLMP	0.887167515	5.54592E-05
0.047335187	CCT3	0.325457426	0.015934843
0.017869355	APOA1BP	0.358537636	0.03918817
0.024956099	SLAMF9	1.339722836	0.009604352
0.000240151	PEA15	0.451188882	3.20164E-05
0.000450912	COPA	0.26260402	0.023080257
9.10568E-05	CD48	0.484474341	4.71297E-09
0.006527888	SLAMF7	1.132078075	2.15101E-09
0.037316573	LY9	1.567999311	1.04776E-06
0.000446423	TSTD1	0.514357609	0.002941336

0.001981823	PFDN2	0.350857191	4.24927E-06
0.003764129	FCER1G	0.217466185	0.003578653
1.23345E-06	UAP1	0.641975757	0.004599152
0.048974238	MGST3	0.513298213	4.05899E-05
0.028922261	UCK2	0.618451307	0.043502151
0.004554342	MPC2	0.276572331	0.006901556
0.016793259	ATP1B1	0.765574301	6.60872E-08
0.010188595	PRDX6	0.360348382	0.001618845
0.002015989	CACYBP	0.444700788	0.003409639
0.003828191	TOR3A	0.339212033	0.021049431
0.021687665	NPL	0.800507286	0.001089316
0.038237394	ARPC5	0.250075678	0.00054632
0.031519611	C1orf21	1.459162103	0.003384213
1.14329E-06	MIR181A1	0.33467114	0.004804292
0.000148054	TIMM17A	0.345625991	0.002076922
5.39559E-23	RABIF	0.68097838	3.68754E-05
2.72053E-10	TMEM183	0.350921783	0.038180286
0.000342951	RAB7B	1.932944192	7.3052E-22
5.78544E-10	IKBKE	0.77167445	0.005903959
0.010791718	FCMR	0.523642352	0.035684034
0.001397376	G0S2	1.075616109	2.81184E-11
0.004577178	TRAF5	0.564410326	0.00468161
0.046613611	CNIH4	0.481381997	0.003119242
0.004262597	SDE2	0.419118247	0.00863943
0.042883415	H3F3A	0.330737436	3.64767E-05
0.032100208	PSEN2	0.825900067	0.014468686
3.09705E-05	ARF1	0.253923672	0.001216427
0.003973771	GUK1	0.345009411	0.000171084
0.005838719	GPR137B	0.948639195	3.182E-08
0.042092531	FH	0.395956044	0.020832442
3.42024E-06	DESI2	0.455641361	0.014907238
0.021896869	COX20	0.467695004	2.85326E-05
0.014517793	RNASEH1	0.545299907	0.00433598
0.000129226	ITGB1BP1	0.300335211	0.039900533
0.002850133	HPCAL1	0.468485352	0.010944448
0.002015989	ODC1	0.47036298	0.006309493
0.004797254	PDIA6	0.569469315	8.96225E-10
9.77547E-05	LPIN1	0.670660427	0.003176079
0.002767493	LAPTM4A	0.413034389	7.93116E-06
0.006548083	SF3B6	0.200285086	0.022905843
1.31473E-07	ADCY3	0.838056446	0.008648802
2.11203E-07	RAB10	0.263666849	0.008799068
0.007675228	SLC35F6	0.446493394	0.008562127
0.028874422	OST4	0.242284827	0.00042976
0.000223304	ATRAID	0.174354227	0.046601825
0.039633163	PPM1G	0.484971091	1.98094E-05
0.037578773	NRBP1	0.427216837	0.00362559
4.19422E-09	GPN1	0.612719207	0.003040767
2.4315E-11	RASGRP3	1.02792947	0.000959001
0.009332256	QPCT	0.992971556	6.41816E-05

0.009768656	CYP1B1	1.606412648	0.004199162
0.016743193	GALM	0.592433953	0.024366712
2.53069E-05	EML4	0.382640568	0.010651697
7.87522E-06	MCFD2	0.569856047	0.000683507
6.54539E-14	B3GNT2	0.566214302	0.027446348
0.040790731	MDH1	0.365633647	6.78429E-05
0.007226079	RAB1A	0.424240174	0.000238987
0.038489634	ANXA4	0.491278086	0.002209414
0.002943421	MXD1	0.343494312	0.0024752
4.64095E-08	PCBP1	0.173764579	0.015109758
0.003848589	SNRPG	0.454931222	1.95198E-07
0.01178873	PRADC1	0.540433387	0.008646946
6.47954E-05	CCT7	0.255280428	0.03687147
0.029381308	BOLA3	0.53627336	0.006269378
9.71664E-13	MOB1A	0.308674631	1.23591E-05
2.0713E-05	MTHFD2	0.31748141	0.016065888
0.01375705	AUP1	0.279127115	0.00765417
2.90633E-09	SUCLG1	0.222885121	0.048483734
1.74079E-06	TRABD2A	1.07805784	0.006432208
0.001460615	CAPG	1.239026453	1.14401E-11
0.001241491	LINC00152	0.64691545	3.47516E-10
1.47246E-06	ARID5A	0.479370561	0.000658772
0.007559168	ANKRD39	0.605130319	0.007901193
0.004443492	SEMA4C	0.417301547	0.049949121
0.049396807	FAM178B	1.780265609	0.024161774
6.4982E-07	COX5B	0.255900732	0.000894162
0.004044653	TXNDC9	0.482687378	0.000914823
0.002850133	PDCL3	0.331356338	0.030174192
1.24971E-05	RNF149	0.25829834	0.035521899
0.00155377	LIMS1	0.477520958	9.86343E-06
0.017107751	LINC00116	0.314420182	0.044950846
0.013226045	MIR4435-2	0.708185747	1.32591E-05
0.001732299	ACTR3	0.377339722	1.00828E-05
1.79208E-18	DBI	0.425720445	0.000117709
0.007675228	TMEM185	0.569215451	0.013040811
0.023990833	RALB	0.414133808	0.015140192
0.017558465	TFCP2L1	1.265128347	0.000214016
0.000131569	SFT2D3	0.523304656	0.021807781
0.006155696	PLEKHB2	0.387011838	0.007526824
0.036634845	TMEM163	2.258893968	8.19946E-06
0.004922003	CYTIP	0.273739793	0.0464135
3.06349E-05	PSMD14	0.423386899	0.00066878
0.007456563	SSB	0.21814579	0.023396604
5.50265E-05	ATP5G3	0.394993841	1.63199E-07
0.000249607	HNRNPA3	0.16012219	0.049949121
0.001939878	STAT1	0.589068085	0.000243323
0.001766728	STAT4	0.840265491	0.000560332
0.011832553	BZW1	0.193449967	0.036535135
0.000463225	NDUFB3	0.381721652	2.46511E-05
0.004044653	SUMO1	0.271987231	0.009282645

1.1062E-05	CTLA4	0.790382991	0.033610655
2.52804E-11	ATIC	0.553857297	0.002149386
0.005324643	MREG	2.178375757	0.026951476
0.004243529	ARPC2	0.34230093	2.3768E-10
0.009637509	CYP27A1	2.133606159	0.000423165
0.039036713	ITM2C	0.413825004	0.009087476
0.021008489	PSMD1	0.35094768	0.003350501
0.000137113	LRRFIP1	0.298236176	0.02833365
6.48074E-05	GPR35	1.115842241	8.91272E-09
0.009748613	ARL8B	0.425839164	0.001493155
1.32228E-09	EMC3	0.316371753	0.006537706
0.022427245	BRK1	0.24793334	0.001404196
5.24536E-10	SEC13	0.37127583	0.001279058
2.5864E-06	LSM3	0.258684211	0.003208404
0.008776791	LRRFIP2	0.419415665	0.017172128
0.007101116	SEC22C	0.557073439	0.006978965
2.26102E-14	EXOSC7	0.509537068	0.004948538
0.027972291	CCDC12	0.305516658	0.027847973
5.85654E-14	NDUFAF3	0.404682988	0.000120979
0.002097334	KLHDC8B	0.863503317	0.037430073
0.006288458	RHOA	0.312195257	1.58986E-07
0.047458111	GMPPB	1.192281593	4.94244E-07
2.00147E-07	TUSC2	0.422451096	0.007725427
0.027957786	MANF	0.507207309	0.000608733
0.00396004	TWF2	0.288159594	0.017710667
0.001969855	NT5DC2	0.706383331	0.002588684
0.004044653	SPCS1	0.244947081	0.00047681
7.36419E-05	TMEM110	1.21898453	0.012735856
4.92712E-06	ARF4	0.260928752	0.012807617
0.011663081	DNASE1L3	0.945642455	1.38406E-07
0.027086521	PSMD6	0.369030943	0.00069364
7.95204E-06	MITF	0.390309276	0.048196903
0.000248978	CHMP2B	0.435802823	0.00054103
0.016885496	NIT2	0.397114798	0.009198714
2.07797E-10	TFG	0.334227466	0.012704811
2.06904E-08	ALCAM	0.649249848	0.001011219
6.63588E-05	ATG3	0.213918714	0.047530147
0.044465897	NAA50	0.410328881	0.009198714
0.034316129	ATP6V1A	0.752249937	5.64927E-10
0.004072925	CD80	1.43033323	4.18289E-05
3.08784E-06	COX17	0.267744925	0.005061799
0.015948475	NDUFB4	0.175097221	0.025717351
0.03531463	CD86	0.357724966	0.00211805
0.027505019	FAM162A	0.253573006	0.025717058
0.048104977	MGLL	1.015481708	2.04682E-07
0.003413195	RPN1	0.363084787	0.000144147
0.039457009	COPG1	0.410155823	0.031947251
0.043068277	ATP2C1	0.454530617	0.03044879
0.001493768	CDV3	0.246140649	0.029620244
1.80138E-05	MRAS	0.59478305	0.016398049

0.007101116	MRPS22	0.431541346	0.029165969
0.000590083	RBP1	1.594978943	5.20594E-05
0.01819792	CHST2	1.336722748	9.65163E-05
0.002602227	SELT	0.35319095	1.10759E-05
0.020829357	SSR3	0.282814204	0.005825375
3.94501E-13	GPR160	0.649537516	4.45467E-10
0.036708722	MRPL47	0.501906436	9.92992E-05
0.007723892	LAMP3	1.981551954	1.9901E-08
0.044631327	AP2M1	0.285204444	3.95479E-05
0.004802482	ALG3	0.409888731	0.026768648
0.039036713	PSMD2	0.478596086	1.49427E-05
0.005978889	POLR2H	0.376896769	0.008103697
0.028394885	ATP13A3	0.535922816	0.002731514
0.01507471	TMEM44	1.208504621	0.026076842
0.042045019	TFRC	0.437070054	0.025600595
0.001067264	ATP5I	0.170909144	0.022915196
0.000268936	MFSD7	0.532259895	0.034633383
4.45163E-09	FGFRL1	1.676710857	0.031633411
1.41929E-15	MAEA	0.497295559	0.002553996
2.31924E-21	C4orf48	0.35498981	0.008338109
8.43631E-05	TNIP2	0.848205642	0.000112731
3.73191E-06	LRPAP1	0.594344117	8.37961E-08
1.31307E-09	GRPEL1	0.383057204	0.021565986
0.004044653	WDR1	0.305641862	0.006669524
0.000359985	HS3ST1	1.22958165	0.000516759
0.008698151	LAP3	0.327611587	8.90092E-05
0.000294859	RHOH	0.650618209	0.001154011
0.032188292	COMMD8	0.383817984	6.75038E-05
5.12695E-05	SRD5A3	0.494737581	0.028315808
0.001217944	EREG	0.605278015	0.004687229
0.003263709	CXCL9	2.587695508	4.33661E-10
0.000192621	CXCL11	1.836234978	0.026874194
0.004908325	PRDM8	0.915281406	0.04688503
0.026486331	GPAT3	0.468331448	0.0211534
0.035490519	NAP1L5	0.907097959	0.03918817
1.40928E-05	GPRIN3	0.370805713	0.038941979
0.000105622	SNCA	0.711035993	0.007901193
2.8833E-05	ADH5	0.313335251	0.035751313
0.017379138	CISD2	0.509427061	9.5214E-05
0.000383144	OSTC	0.362857748	9.42045E-05
0.010892876	TNIP3	1.201634086	0.002196629
0.002397492	ANXA5	0.472322095	2.04005E-05
0.046378128	SLC7A11	2.627647888	5.15816E-06
0.002544961	NDUFC1	0.416671869	0.002067529
0.022511564	SPCS3	0.395318788	8.14884E-07
0.003684025	MRPL36	0.34945151	0.03952131
0.007084562	MIR4458H	0.493309008	0.038373902
1.09838E-05	CCT5	0.369245672	0.016255848
0.025830336	ZNF622	0.488727281	0.000561571
0.049992799	BASP1	1.231933957	0.015747054

0.000696499	SUB1	0.371680803	4.49276E-08
0.021561073	IL7R	2.764150273	0.002267113
3.35705E-08	PLPP1	0.690292399	0.029165969
0.003282269	SGTB	0.631784836	0.017812891
1.31307E-09	HEXB	0.624324823	4.4745E-08
0.033093772	IQGAP2	0.321465068	0.006051588
0.000538386	TMEM167.	0.354689855	0.001608023
0.000253995	COX7C	0.149214597	0.00639821
0.000127947	GLRX	0.295624315	0.024458925
0.000197323	SRP19	0.296517978	0.036165468
0.014592202	REEP5	0.283593906	0.000337719
0.000385882	HSD17B4	0.457779041	7.82721E-05
0.034273473	SNX24	0.582328133	0.023634793
0.009900715	PPIC	1.227341449	0.005963763
0.031280576	HINT1	0.3143565	0.000166773
0.000486406	UQCRQ	0.266308778	5.09419E-05
5.08271E-05	HSPA4	0.345201057	0.013619259
0.005660471	VDAC1	0.446447548	5.12655E-06
2.09711E-16	UBE2B	0.35817952	0.022619439
0.013790493	H2AFY	0.321249917	0.001809546
0.04374512	SIL1	0.479492627	1.61114E-06
0.007187337	CXXC5	1.086063398	0.007023821
0.016589199	CYSTM1	0.923387302	5.74012E-11
0.028284819	PFDN1	0.301614793	0.014870029
0.00121538	SRA1	0.294851177	0.003702013
0.005358635	HARS	0.511580106	0.010960293
3.68175E-07	HDAC3	0.400869714	0.011504295
0.005978889	GNPDA1	0.480396227	0.011735002
1.96195E-07	CD74	0.158672675	0.039072004
0.003212799	SMIM3	0.556000543	0.001618845
6.43547E-09	ANXA6	0.990205346	1.12599E-09
0.000130094	GM2A	0.747943666	9.79682E-07
0.0037238	SLC36A1	0.731193052	0.012204853
0.022619857	ATOX1	0.96990713	2.90971E-20
0.000486565	HAVCR2	0.397160864	0.005813483
7.74634E-06	ADAM19	1.457650518	0.000142382
0.034605143	PTTG1	0.977324868	1.42341E-09
1.3712E-05	NPM1	0.216761418	0.005939417
0.000966869	ERGIC1	0.645235759	1.75174E-05
6.71601E-06	RPL26L1	0.277831322	0.040899963
0.015287198	ATP6V0E1	0.456988803	8.24166E-14
0.003142618	PRELID1	0.4594144	2.24009E-08
0.034771386	LMAN2	0.427282415	1.82472E-10
0.003932147	GRK6	0.402557059	0.023697363
8.87451E-05	PDLIM7	0.847079923	0.012092477
2.45724E-05	TMED9	0.380558682	7.25447E-07
9.5836E-05	HNRNPAB	0.258390309	0.005963696
0.000508536	RNF130	0.240885003	0.000190394
0.029819109	IRF4	0.716136937	0.014160003
0.003938374	TUBB2A	0.797720249	0.003024256

0.000672107	TPMT	0.291592811	0.008919289
0.002208583	ACOT13	0.509172364	0.00192889
0.000605596	HLA-A	0.236811552	0.001585906
8.5965E-06	ABCF1	0.31592543	0.033773817
0.037322242	TUBB	0.287869546	0.001866519
1.00517E-13	HLA-C	0.254775271	2.05233E-05
0.029793825	HLA-B	0.206744788	0.006265737
2.28289E-15	NCR3	2.110036527	0.027313644
2.67407E-05	CSNK2B	0.381884902	2.8757E-05
0.03409012	CLIC1	0.256524706	0.002920118
0.026155639	HLA-DQB1	0.257984658	0.025527647
4.38334E-06	HLA-DMA	0.185262017	0.012252128
0.032278682	PFDN6	0.356266883	0.015494728
0.016301062	BAK1	0.390723392	0.028887719
0.049141	UQCC2	0.546923318	1.78961E-05
0.006595992	C6orf1	0.40106172	0.040244567
0.040995993	SNRPC	0.312364275	0.006663476
3.82091E-37	PIM1	0.992626527	3.5779E-06
0.002010644	GLO1	0.372698659	0.002238188
0.015061571	TREM2	1.148881187	1.36019E-05
0.022148956	DNPH1	0.487285462	3.9091E-06
0.035208065	NFKBIE	0.522543451	0.008338109
0.000589205	PLA2G7	2.10154027	1.93892E-27
0.017177844	TMEM14A	1.010686167	0.008698027
0.030024027	GCLC	0.61803669	0.00049768
0.000746813	CD109	1.104141784	0.002076922
0.039267116	COX7A2	0.383203208	3.24497E-07
0.026869812	UBE2J1	0.435077505	6.42748E-05
2.68293E-08	RRAGD	0.916668457	8.57962E-07
2.69623E-10	SNX3	0.254047931	0.03266455
1.32847E-07	GTF3C6	0.62636518	1.63669E-08
0.007222213	RP11-557H	2.394700279	0.019213845
0.011162994	ABRACL	0.405501782	3.48089E-08
0.00381485	MTHFD1L	0.779194372	0.000101276
0.049049538	GTF2H5	0.237991951	0.042908346
0.000666853	DYNLT1	0.634257353	7.13106E-07
8.3187E-18	ACAT2	0.644171527	0.000633466
3.13956E-06	IGF2R	0.793169624	8.60539E-05
8.72803E-29	SFT2D1	0.287525224	0.001567965
0.003991197	MPC1	0.271941302	0.032784324
0.023016066	PSMB1	0.447423772	5.74012E-11
2.82712E-10	FAM20C	0.481112198	0.004303622
1.28499E-06	SNX8	0.586866359	0.001564578
0.008221574	EIF3B	0.323528565	0.015437259
0.015966119	GNA12	0.484687273	0.011652509
0.008279532	ACTB	0.283718165	0.000336311
0.011823602	RAC1	0.217039225	0.00014789
0.032832036	KDELR2	0.574482691	6.01466E-07
4.52622E-28	RPA3	0.274946713	0.039314639
0.03582472	NDUFA4	0.344747382	2.5594E-09

0.016042665	BZW2	0.487093038	0.00137794
0.038237394	TSPAN13	2.473792818	1.26404E-05
4.91854E-05	MACC1	1.887699673	1.70539E-08
0.000499401	ITGB8	1.47766207	0.04972708
3.01448E-07	IL6	1.143089574	0.002404479
0.000620258	GPNMB	1.682180895	7.21513E-22
0.007776659	GARS	0.417955904	0.009476411
0.007159046	EEPD1	0.727384165	0.028243069
0.000112425	STARD3NL	0.519575133	0.002108436
0.000318509	RALA	1.332550887	2.63965E-07
0.049791136	PSMA2	0.383172527	1.65098E-06
0.001378741	STK17A	0.494460689	0.009962168
0.028039213	BLVRA	0.367755171	0.000129721
0.010646156	DBNL	0.270343245	0.004928643
0.000941769	YKT6	0.526118549	0.014279581
1.37943E-06	PPIA	0.131296066	0.016772238
0.003648195	H2AFV	0.263575582	0.010558025
1.40424E-05	SEC61G	0.47875111	8.14884E-07
0.03636839	CCT6A	0.207313114	0.037701
0.009637509	CHCHD2	0.388528465	7.73187E-16
0.000996783	GUSB	0.379525238	0.001137232
0.013648228	WBSCR22	0.291056676	0.005806791
0.009629655	RHBDD2	0.398883346	0.003068677
0.007101116	MDH2	0.23935833	0.002209414
0.012414463	YWHAG	0.29685484	0.029165969
0.000679781	CD36	0.314153841	0.012601386
0.012347974	ABCB1	1.229267481	0.02045251
8.43208E-05	DBF4	0.580496958	0.00149956
0.002860645	SRI	0.262405434	0.016925696
0.013678412	SHFM1	0.265936198	0.001025766
0.017150141	BRI3	0.390744058	6.84659E-08
0.00236107	ARPC1B	0.273575007	0.002990993
0.01761659	BUD31	0.248604446	0.015934843
0.042651535	ATP5J2	0.285559961	4.17755E-05
0.028532809	AP1S1	0.477971443	0.018406089
0.00144805	FIS1	0.180840758	0.03266455
0.024956099	SH2B2	0.812890389	0.017035583
0.035490519	PSMC2	0.444325147	0.002123705
0.010989168	SND1	0.295358075	0.009606942
0.022885171	ATP6V1F	0.784500051	5.50609E-15
0.006618349	AKR1B1	0.455180344	7.82504E-06
2.97379E-10	ZC3HAV1	0.372961545	0.007245789
0.018244397	NDUFB2	0.28965359	0.00033797
0.017266554	SSBP1	0.245570036	0.008253554
0.045898802	CLEC5A	1.509761336	0.031810805
0.027362013	ZYX	0.228072519	0.01639987
0.009213703	TCAF2	1.716369523	0.005058289
0.001192421	PDIA4	0.510581042	0.0002536
0.001722878	TMEM176	0.572794865	0.005303565
3.60741E-09	TMEM176	0.529723028	0.005987518

0.036160667	RHEB	0.573657958	5.3835E-05
0.001512115	INSIG1	0.649304804	1.86816E-07
0.004467992	IL3RA	1.125436079	0.000234043
0.017138268	ASMTL	0.571080863	0.001099977
0.002168245	TMSB4X	0.216233623	0.000224156
0.002830979	RAB9A	0.905242124	2.15101E-09
0.015657572	PIR	1.488318508	0.00227886
0.040991223	PDHA1	0.338545905	0.015780676
0.019886196	SMS	0.51994272	0.002941336
0.006176126	PRDX4	0.447195913	0.000310236
0.012078783	ACOT9	0.417408416	0.001203434
0.007081328	APOO	0.714900558	0.000470087
2.97921E-05	DYNLT3	0.42949136	0.015072989
0.029628023	ATP6AP2	0.652558175	1.67083E-15
1.47246E-06	TIMP1	0.791629607	0.00181971
0.048726549	EBP	0.45997287	0.001126576
0.000146414	HSD17B10	0.31875235	0.022630239
0.045937858	MSN	0.201195266	0.038370504
3.7922E-05	IL2RG	0.86224079	0.001143256
0.001380519	PBDC1	0.445080209	0.004355735
0.0064395	COX7B	0.355079625	2.22994E-07
0.006320432	PGK1	0.488432174	8.85226E-10
8.07707E-05	P2RY10	0.803064506	0.026270674
0.009714667	TCEAL3	0.283145355	0.047736769
0.040837158	SLC25A5	0.303684245	8.48812E-07
0.031584749	UBE2A	0.29584102	0.012252128
0.001142357	SEPT6	0.513396658	0.006476772
0.035526123	NDUFA1	0.227908425	0.001314201
1.11726E-15	LAMP2	0.376209288	0.003384213
3.92483E-05	AIFM1	0.413058885	0.043875539
0.025621443	HPRT1	0.35267826	0.003454583
0.037083902	HMGB3	0.78262954	0.000849979
0.000769998	VMA21	0.247293762	0.028500252
0.000329727	BCAP31	0.404543309	2.77264E-07
0.010629073	SSR4	0.390545943	2.78159E-10
0.017206605	NAA10	0.335079671	0.01074925
0.005487196	FLNA	0.987038529	0.004006003
0.023678916	EMD	0.31233989	0.024009501
0.012421526	CH17-340I	0.338904447	0.026036657
0.017049141	ATP6AP1	0.354856381	0.000133481
0.02430406	FAM50A	0.446124527	0.000443472
5.67815E-07	LAGE3	0.620113561	1.68829E-08
0.016905799	G6PD	0.604932684	0.00108328
0.041571943	MPP1	0.631916783	3.45671E-06
0.000127366	CLN8	0.340046188	0.027706212
0.003732619	CTSB	0.422379671	0.000331066
0.002097334	ASAH1	0.573799273	6.87899E-06
0.009893218	ATP6V1B2	0.478297883	2.54743E-05
0.00010658	ADAMDEC	1.455368512	5.16718E-07
0.013748629	DUSP4	0.954715046	0.004973426

0.006297008	GTF2E2	0.433528441	0.028500056
0.015861296	NRG1	1.228504426	0.01386967
0.000388713	RAB11FIP1	0.544642353	0.000420621
0.036708722	HTRA4	2.515353721	0.003668513
0.001560463	ADAM9	0.346531589	0.012765397
4.07968E-06	ST18	1.305059138	0.024705468
9.34874E-05	ATP6V1H	0.684437054	4.22831E-07
0.03784967	SDCBP	0.258569716	0.024798387
0.001092197	RAB2A	0.28940357	0.004885789
0.012695807	DNAJC5B	0.986819842	0.001924194
1.9049E-05	TCEB1	0.358566687	0.001013612
5.73472E-07	TMEM70	0.356819789	0.019804134
0.021300515	FABP5	1.809780773	2.46659E-05
0.000610461	FABP4	1.922769795	0.013421694
0.039753208	CA2	1.16743624	0.003276768
0.000111336	ATP6V0D2	3.46491363	2.10049E-23
0.009900715	OSGIN2	0.462576407	0.026662184
0.002208583	DECR1	0.276654088	0.013808153
0.007523019	SDC2	1.130737521	1.48336E-05
0.012702802	MTDH	0.161614559	0.028753913
0.028139276	COX6C	0.361798273	1.86685E-09
0.024171612	POLR2K	0.296718715	0.039370797
0.034056226	ATP6V1C1	0.326127627	0.025562162
3.53932E-15	DCAF13	0.449847466	0.026360185
0.020774504	ENPP2	0.649735661	0.015627777
0.000917511	DERL1	0.463773697	0.001758463
0.000193539	NDUFB9	0.246900747	0.040399797
3.37473E-06	SQLE	0.824164906	0.007752997
8.4005E-07	FAM49B	0.288701888	0.000125813
0.002544961	AGO2	0.675833399	0.00205395
5.46288E-12	LY6E	0.441242283	5.92082E-07
2.15884E-06	TSTA3	0.451774692	0.031510369
2.63064E-05	PUF60	0.38037547	0.001257305
0.000100484	CYC1	0.343367848	0.000674243
2.01836E-06	COMMD5	0.400417065	0.005274504
0.001062463	CBWD1	0.468120066	0.003072802
0.049818253	PLGRKT	0.297435546	0.044122294
0.00021507	CD274	1.37924083	0.007933338
0.037296514	NDUFB6	0.332534144	0.016739675
0.02003506	BAG1	0.295804626	0.022166508
0.003371081	CCL19	1.138666352	0.009117057
0.018827348	VCP	0.433042333	9.50094E-05
0.036987422	STOML2	0.360289466	0.010944448
0.027963456	CCDC107	0.508730369	0.0019364
0.000590083	CREB3	0.669483022	0.000605215
6.30272E-08	GLIPR2	0.796880434	0.005663652
0.000493873	CLTA	0.342280781	0.002371665
0.047982296	SHB	0.767994418	0.011418876
0.004676092	TJP2	0.72379222	1.7751E-05
0.000127523	ANXA1	0.374444254	0.00022088

1.34859E-09	ISCA1	0.367247569	0.023400176
2.77296E-07	CTSL	0.83342218	0.000627847
0.000643369	CARD19	0.509241983	0.031510369
5.7173E-24	NINJ1	0.587335564	0.011553745
0.024956099	AAED1	0.380123985	0.009865521
0.048867414	NANS	0.307013986	0.003032931
0.00155377	CORO2A	0.98066487	0.00556341
0.005061384	TBC1D2	1.537053905	0.001025766
0.023281063	ALG2	0.588580483	8.52073E-05
0.044532956	SEC61B	0.551226881	1.36276E-18
2.12282E-05	ERP44	0.281959961	0.009232242
0.048606191	TMEM38B	0.649718517	0.007439246
0.001543989	TXN	0.705317584	0.002590986
1.64139E-07	SLC31A1	0.404403558	0.008728722
0.001264727	ATP6V1G1	0.589386428	1.76171E-12
4.33009E-09	GSN	0.39024419	0.004973426
0.015401083	NEK6	0.565507848	0.023425225
0.007657679	PSMB7	0.356752342	0.001814338
1.59405E-05	ARPC5L	0.392929913	0.015967336
0.023995043	ZBTB43	0.770622684	1.89168E-05
0.036108016	STXBP1	1.379276591	0.020847864
0.00016308	PTGES2	0.554847352	6.70535E-05
0.0139151	CERCAM	1.334962566	0.018380341
0.005074884	ZDHHC12	0.519000903	2.77437E-05
0.03594496	DOLK	1.310149262	0.002074338
1.88602E-05	NTMT1	0.37330078	0.022916507
0.042883415	RAPGEF1	0.462326962	0.036535135
0.003150301	SURF4	0.487871516	3.35015E-05
2.40688E-14	TMEM141	0.389001283	0.005608448
0.001656147	EDF1	0.1606988	0.019977864
0.006460527	PTGDS	1.843429206	0.038180286
0.047193274	DPP7	0.237192434	0.002742345
0.023487792	TUBB4B	0.375656032	0.040244567
0.048974238	NRARP	0.779102491	0.010591078
0.024301331	MRPL41	0.417534876	0.002758908
0.047999566	PSMD13	0.271339509	0.015672751
0.001348754	RNH1	0.470218036	1.87451E-05
0.03170943	TALDO1	0.769852473	2.08092E-15
0.02370903	POLR2L	0.258142722	0.00137794
4.04103E-08	TOLLIP	0.585659556	0.004191568
1.35513E-05	IFITM10	0.652435456	0.020481084
6.32048E-09	CTSD	1.190699098	2.31266E-13
0.019174536	CD81	0.401237825	0.002866398
0.042933294	PHLDA2	0.632626293	0.000992092
2.77788E-05	RHOG	0.173115079	0.048759739
0.024104015	TAF10	0.333042075	0.007287216
6.2438E-09	SWAP70	0.350208202	0.008141748
0.019519652	PSMA1	0.352021389	7.25447E-07
4.98117E-12	NUCB2	0.460970796	0.005534782
0.00789857	LDHA	0.628476269	3.14786E-07

8.33415E-07	CD59	0.694365646	0.002348693
4.13892E-05	FJX1	1.169109425	0.039370797
0.044850354	COMMD9	0.23139956	0.038128782
0.000350234	PSMC3	0.478627075	2.7409E-05
4.9462E-07	PTPMT1	0.321142483	0.010778802
0.007243079	MTCH2	0.630199943	4.68217E-07
1.63708E-06	SLC43A3	0.401252599	0.019808632
0.000197323	TIMM10	0.429078312	0.003509585
0.002098858	TMX2	0.333808795	0.031355973
5.91387E-06	C11orf31	0.283101135	0.002149096
0.00317396	LPXN	0.507910471	0.000157
0.009782347	SLC15A3	0.378016406	0.038281111
0.007467492	CYB561A3	0.666680025	0.003040767
0.000535022	TMEM138	0.911629141	3.45671E-06
0.005487196	TMEM258	0.201462953	0.014821276
0.000796634	FTH1	0.304900954	0.002076922
2.63254E-05	TMEM179	0.290322476	0.015560419
0.000128146	SLC3A2	0.712273088	2.03634E-08
0.003102127	COX8A	0.309114081	0.000350451
0.017107751	VEGFB	0.498269403	0.000735442
4.21081E-06	FKBP2	0.497700904	7.88575E-13
0.030982744	PPP1R14B	0.36920092	0.000910537
0.006911133	PRDX5	0.381731364	2.04682E-07
0.001361957	KAT5	0.410838492	0.036292368
4.96299E-06	RNASEH2C	0.377732969	0.000990227
0.039253018	AP5B1	0.574672804	0.04688503
5.44285E-06	CFL1	0.422023947	7.20613E-15
3.08784E-06	FIBP	0.385415441	0.000243323
0.000484893	DRAP1	0.331827759	6.75038E-05
0.014282021	BANF1	0.218019643	0.012222444
0.000108668	SF3B2	0.265863752	0.012491343
3.00886E-14	YIF1A	0.436309942	0.002180389
1.23816E-22	PC	1.270519197	0.041741956
0.009679469	PPP1CA	0.279485118	0.000649302
0.026761662	CDK2AP2	0.347915372	0.001304727
2.58423E-05	GSTP1	0.277638336	0.01815439
0.000285599	NDUFS8	0.323505421	1.21426E-05
0.032188292	MRPL21	0.27154068	0.026226756
0.012586337	DHCR7	1.342759554	0.03687147
0.045115766	IL18BP	0.825131452	9.86489E-14
0.010156418	ANAPC15	0.4289006	0.017281419
0.001130735	P2RY2	1.426603923	0.013895823
2.32817E-08	UCP2	0.557831253	8.22772E-08
0.00019223	SPCS2	0.414505879	1.99672E-06
0.034861836	NDUFC2	0.344557339	5.76089E-05
0.020348702	TMEM126A	0.286138169	0.013770955
0.000876673	TMEM123	0.244497749	0.026764543
4.93122E-06	MMP1	1.249054651	0.000400113
4.8061E-10	MMP12	3.436068121	2.73027E-38
3.70058E-13	ACAT1	0.440959688	0.008700601

0.01833024	FDX1	0.475902714	0.001341207
0.00024691	ARHGAP2(	0.663480203	0.019998229
0.006384976	TIMM8B	0.452376932	1.76577E-06
0.000654581	SDHD	0.222212281	0.008958579
0.002315509	PTS	0.492175382	0.004418499
1.52439E-05	RBM7	0.368376565	0.036788303
0.030081739	REXO2	0.460127647	0.004072972
0.016141824	CADM1	1.207680836	0.029923729
0.016350125	SIK3	0.424615767	0.03154455
0.001987678	DPAGT1	0.576702253	0.005710414
0.000247381	UBASH3B	0.820248347	6.19619E-05
0.01507471	SRPRA	0.286586026	0.032771666
0.003263709	ST14	0.517606295	0.005182289
0.008198538	PFKP	1.601401435	1.65841E-05
0.002622257	ATP5C1	0.263135578	0.001826567
0.045999956	NUDT5	0.387971591	0.003945984
0.001185408	CDC123	0.457386314	0.003535689
0.03244521	OPTN	1.308979474	0.034688179
0.001003936	FAM107B	0.443253972	0.002608716
0.004195067	VIM	0.422874417	0.014219262
0.0086637	MAP3K8	0.333508138	0.021049431
0.005900512	ALOX5	0.367437539	0.001877763
5.0881E-05	SYT15	2.089035063	0.000880199
0.020023874	RP11-556E	0.926530539	0.002710723
0.003318593	ARID5B	0.487700294	0.026923752
0.013858299	DNAJC12	1.681426782	0.019088725
0.002550643	HK1	0.411644693	0.001485904
0.001003936	PPA1	0.577010379	3.40878E-06
0.001415961	SGPL1	0.460733459	0.045357406
3.02697E-09	PCBD1	0.399763141	0.001314201
5.77012E-05	PSAP	0.499401586	4.32481E-09
0.000816252	MICU1	0.445113594	0.018221214
0.000505311	ANXA7	0.298862262	0.007228658
3.12548E-14	CHCHD1	0.338781795	0.002196364
0.003848977	VDAC2	0.271671871	0.031947251
0.000825488	KCNMA1	0.553117102	0.016757116
0.011213586	ANXA11	0.337263662	2.44386E-05
5.44285E-06	GHITM	0.412942161	9.30509E-08
0.009965969	LIPA	1.018513355	4.33661E-10
0.038762875	IFIT3	1.271659847	2.996E-09
9.23994E-08	PGAM1	0.467288997	4.43484E-07
0.049947013	AVPI1	1.273942394	8.23102E-14
0.00170449	BLOC1S2	0.824125857	1.63667E-08
0.024340431	PKD2L1	1.59095637	0.011504295
0.003021541	SCD	0.82832735	0.00541164
1.27774E-07	NDUFB8	0.352672574	9.36152E-08
0.047069669	NPM3	0.388843142	0.018737597
1.21483E-07	ACTR1A	0.398545064	0.008780176
1.35185E-05	USMG5	0.245506555	0.002394181
0.018072418	GSTO1	0.687626276	1.80299E-12

0.004855349	EIF3A	0.2051324	0.017100196
0.017049141	OAT	0.683014818	1.8959E-05
1.82369E-05	UROS	0.405363225	0.007228658
5.50886E-05	MKI67	1.173952357	0.016576203
1.53272E-11	GLRX3	0.395714935	0.012978478
0.025879094	FUOM	0.275586023	0.009300604
1.1555E-12	ECHS1	0.330905742	0.000237705
0.003340966	RP11-598F	1.40752879	0.031355973
0.000521469	TIGAR	0.461325142	0.00329188
0.000448816	NDUFA9	0.519304014	0.000273601
1.70192E-08	CD9	1.890671009	2.7466E-06
0.007241941	MRPL51	0.255352656	0.009229769
0.010080421	GAPDH	0.439866076	4.39006E-11
0.002010644	MLF2	0.300030433	0.003384213
8.48574E-09	PTMS	0.56291261	1.61544E-10
0.000353353	LAG3	1.125651083	0.005987518
2.23787E-07	P3H3	2.398660237	0.011039426
8.50829E-09	TPI1	0.296904791	3.84066E-07
0.003251022	M6PR	0.423100057	1.83748E-07
0.01640594	CLECL1	0.962718943	0.012451164
0.000215707	PLBD1	0.409251227	0.008098715
0.00119569	H2AFJ	0.369237883	0.012032488
2.98403E-06	STRAP	0.482937464	3.21658E-05
0.000876673	LDHB	0.337062965	0.000667245
1.89075E-05	PFKM	1.102086552	0.003870184
6.18094E-05	TUBA1B	0.274723801	0.001089316
0.000623722	TUBA1A	0.622401985	5.47084E-08
8.17561E-08	TUBA1C	0.631846529	8.07768E-07
0.025263432	TMBIM6	0.354907862	5.09388E-09
0.020964741	COX14	0.267224392	0.001211145
0.018547694	ITGB7	0.810106674	1.15116E-06
0.024148461	BLOC1S1	0.390479214	2.96006E-07
0.023678916	CD63	0.697796167	6.91703E-14
1.51012E-05	ORMDL2	0.656224782	5.41218E-09
0.048214275	MYL6	0.405185223	7.65914E-12
0.000263311	CNPY2	0.300180694	0.004389317
9.86789E-09	IL23A	0.79357544	0.018737597
0.000590083	ATP5B	0.401498508	3.21195E-06
0.005444498	CDK4	0.36577906	0.034741011
5.19758E-08	RASSF3	0.373558151	0.033610655
0.015401083	RAP1B	0.391187333	0.002895442
0.004347157	LYZ	0.650038654	1.20977E-06
8.61768E-05	RP11-1143	0.706571501	0.000248002
0.036208849	PHLDA1	0.417351591	0.032563919
0.007222213	PLXNC1	0.449890699	0.001618845
0.005177408	TMCC3	0.440269635	0.036411614
1.15005E-06	NDUFA12	0.200451902	0.043311982
1.20131E-09	SNRPF	0.290997587	0.003344258
0.004899548	ARL1	0.36665289	0.009387292
0.021333388	DRAM1	0.514801105	0.031826133

8.14689E-05	TXNRD1	0.728128314	1.95066E-07
2.41416E-06	CORO1C	0.533999151	0.000319495
0.033760255	SSH1	0.529447593	0.016170541
0.004238171	ARPC3	0.206462223	0.008485323
0.031519611	VPS29	0.210582447	0.010902206
1.9049E-05	ERP29	0.24685372	0.009310581
0.000647042	RASAL1	1.514655612	0.023391159
4.17785E-14	PLBD2	0.702477001	6.88319E-05
4.99642E-05	SDS	1.459648892	2.24287E-05
3.84625E-06	SDSL	0.99964624	3.97442E-13
0.001301428	C12orf49	0.633636682	0.011612537
0.009187672	PEBP1	0.232263681	0.007434855
0.049049538	COX6A1	0.426459969	2.62615E-12
0.006436402	SRSF9	0.353895871	0.002350034
0.013733728	MLEC	0.336847401	0.026297465
0.003772211	OASL	1.062129908	0.001537597
0.000126497	ORAI1	0.371779106	0.002990993
2.18511E-11	TMED2	0.396689362	8.53023E-05
0.019835926	UBC	0.309857182	0.001358664
0.008988494	SLC15A4	0.465626078	0.003208404
2.97335E-05	RAN	0.337907746	1.97012E-07
0.00064681	SAP18	0.194686657	0.020143376
0.001901412	MRPL57	0.266006826	0.003100418
0.004262597	POMP	0.316876379	1.05029E-06
0.00543886	ALOX5AP	0.849923963	0.002563297
0.002015684	ALG5	0.595231108	4.18289E-05
0.000633988	UFM1	0.239234345	0.046082674
0.012421526	RGCC	0.893405158	0.001787147
3.96421E-05	DNAJC15	0.253143927	0.007752997
0.039233627	TSC22D1	1.111210997	1.45991E-11
0.000103861	LCP1	0.319388995	3.67216E-06
7.32422E-09	NUDT15	0.511111783	0.042159786
0.005196719	UCHL3	0.718094863	2.13727E-06
0.006338349	DNAJC3	0.638155292	3.84066E-07
0.000462943	TEX30	0.70752679	0.013233882
0.028252201	DAD1	0.31300902	5.18932E-06
0.000491379	MRPL52	0.375954468	1.12912E-05
5.58017E-11	MMP14	0.529295161	0.030389362
7.35802E-10	PSMB5	0.361969346	0.001618845
2.85324E-07	PSME2	0.536770264	1.31095E-09
0.045771569	NEDD8	0.172470073	0.005138028
0.00928577	HECTD1	0.586266734	0.002838187
0.002965696	EGLN3	0.877882434	0.029955728
0.000281543	FAM177A1	0.408312674	6.41349E-05
0.000655152	FBXO33	0.552672999	0.002577684
0.043368294	RPL36AL	0.267695607	0.001081316
0.029798171	C14orf166	0.154521071	0.044449264
0.04221492	PTGER2	0.40443056	0.0421159
0.002018639	ERO1A	0.409835406	0.047609231
0.022427245	MAPK1IP1	0.216437967	0.049313219

0.038319297	LGALS3	2.041819662	2.64908E-20
2.08401E-06	FBXO34	0.596839889	0.000731934
0.002705595	PSMA3	0.633082783	2.19248E-06
0.024097115	ATP6V1D	0.347408619	0.039775202
0.009900715	EIF2S1	0.370157176	0.002965286
0.02002245	VTI1B	0.240394443	0.043311982
0.015209705	ACTN1	0.718538733	1.68926E-05
0.018608126	ERH	0.291030365	0.000486784
2.00679E-05	C14orf169	0.818384146	0.001253475
0.003596947	ACOT2	0.677436139	0.026492345
0.016049874	PNMA1	0.954663786	0.005987518
0.000263311	TMED10	0.312131263	1.96366E-06
0.021767676	SLIRP	0.447671345	1.83076E-07
7.329E-06	GPR65	0.412584384	0.032995568
0.015047806	PSMC1	0.305485943	0.023491485
0.029798171	CALM1	0.327018567	0.000166602
0.001871768	TMEM251	0.479205522	0.03441281
0.006843307	IFI27	0.693430678	0.039460352
0.001793934	WARS	0.747008631	1.38553E-08
0.003275176	TRAF3	0.860048601	0.00040444
0.0006637	C14orf2	0.180193623	0.032505419
0.000679781	NUDT14	0.589769263	0.005850068
0.006668476	CYFIP1	0.342392138	0.043967143
0.028325819	EMC7	0.381042794	0.001485904
3.20586E-05	EMC4	0.314010169	0.007901193
3.11746E-06	NOP10	0.437425687	1.42485E-05
0.01220499	SRP14	0.352888703	4.56795E-10
0.049570733	GCHFR	0.577501692	0.033773817
0.049780753	EHD4	0.39384557	0.044266716
0.001319988	PDIA3	0.407470117	0.000585837
5.88679E-06	SERF2	0.344021746	7.52849E-07
0.049818253	B2M	0.294868531	0.00180181
0.024284943	DUOX1	1.018581041	0.00488521
0.00198817	SHF	0.954078969	0.031643536
0.000571621	C15orf48	1.204340758	0.000148566
0.028111839	SQRDL	0.399107796	0.015954153
1.90174E-13	LYSMD2	0.395204902	0.031510369
2.50639E-08	MAPK6	0.672935741	0.000142409
0.028139276	GNB5	0.959601794	4.44137E-05
0.004883719	MYO1E	0.72470229	0.010415643
0.00399369	ANXA2	0.797698653	9.86489E-14
2.83717E-08	RPS27L	0.400786407	7.1885E-05
0.014853832	PPIB	0.497733304	1.20753E-10
0.047305962	OAZ2	0.28784809	0.004687229
0.003371081	SPG21	0.231351932	0.024850067
0.001921769	SNAPC5	0.417121805	0.030138051
0.002593341	PKM	0.588157765	4.01104E-07
0.005402811	HEXA	0.255825167	0.014029996
8.17093E-13	COX5A	0.473903744	4.62715E-12
1.32847E-07	C15orf39	0.504433869	0.024137796

1.26701E-36	COMMD4	0.645332114	6.16037E-05
1.30271E-06	ETFA	0.325793348	0.00077643
0.003595901	PSMA4	0.270757907	0.003917786
0.007401515	MORF4L1	0.167478732	0.013448339
0.020414395	CTSH	0.801598488	4.32763E-10
1.80138E-05	MESDC2	0.435736398	0.000359837
0.000830712	NMB	1.232061811	0.001578338
1.08932E-06	ISG20	0.459528083	0.021372515
0.000185626	ABHD2	0.475742512	0.028753913
0.019529997	RHCG	1.322025469	0.008091254
0.003205211	IDH2	0.318201036	0.00413459
0.002858597	CIB1	0.511279086	7.52849E-07
0.003205211	FURIN	0.593115942	0.005183807
0.000803148	LYSMD4	1.142340953	0.002747104
0.046511519	VIMP	0.357250251	0.047620657
0.033823254	TM2D3	0.303139384	0.020336725
0.015931161	SNRNP25	0.475060342	0.018035723
1.22122E-23	UBE2I	0.27764445	0.007752997
4.245E-27	MRPS34	0.245386434	0.033047376
5.29612E-09	PGP	0.284395035	0.042159786
6.9226E-13	AMDHD2	0.599074258	0.005318033
0.000556072	TCEB2	0.215876194	0.003990261
0.036708722	TNFRSF12/	1.487625493	0.000143574
0.000400116	HCFC1R1	0.35027411	0.040244567
4.96299E-06	MMP25	0.637372903	0.017061349
0.02081786	IL32	0.981154364	0.018274567
0.034303961	CARHSP1	0.603294025	0.004196422
0.013226045	EMP2	0.492887624	0.020798783
6.23798E-09	DEXI	0.97765838	0.011015519
1.65537E-10	LITAF	0.525043918	9.93379E-06
0.000228709	SNN	0.626980454	1.715E-05
0.002148209	GSPT1	0.446663041	0.000160051
0.041017653	CPPED1	0.348037652	0.007752997
0.001259294	NTAN1	0.419692438	0.008730364
0.026550319	SMG1	0.366516295	0.006903044
0.013080431	IGSF6	0.434706922	1.21615E-05
0.047279522	OTOA	2.581583845	2.39914E-13
0.030743633	UQCRC2	0.236820656	0.038259272
0.030191624	NDUFAB1	0.29036305	0.002149096
0.038567873	CLN3	0.386820795	0.035942892
8.33914E-06	NUPR1	1.65140838	0.003024256
0.047813387	SPNS1	0.510000086	0.001067676
0.024125026	MVP	0.38575914	0.002888786
3.52124E-05	ALDOA	0.57752242	6.92086E-15
2.46683E-12	PPP4C	0.350156734	0.012978478
0.026481406	DCTPP1	0.406039412	0.006905145
0.00052227	VKORC1	0.406608106	2.6694E-05
3.16153E-06	ITGAX	0.711646937	0.000770874
0.017478922	ZNF267	0.413219603	0.013949816
0.006063255	VPS35	0.203365884	0.041239614

9.87025E-05	DNAJA2	0.255090841	0.03918817
0.008824425	PAPD5	0.539009506	0.002076922
4.13247E-10	MT2A	1.090040168	0.000253521
0.017325691	MT1E	1.73716187	0.000409784
0.048066005	MT1F	1.210222509	0.001988148
1.01676E-07	MT1G	3.090000371	4.62715E-12
0.031534563	MT1X	1.621459549	2.23664E-08
0.000246948	KIFC3	1.11370163	0.006831896
0.002238992	FAM96B	0.370532861	1.73366E-05
0.002968673	TMEM208	0.537639366	1.65159E-06
1.47246E-06	ATP6V0D1	0.373126186	9.15945E-05
0.000129706	NQO1	1.492506206	0.000936308
0.004768058	EXOSC6	0.39592074	0.009722296
2.22372E-06	PSMD7	0.387039648	7.23411E-06
0.000445376	WDR59	0.552854078	0.013395464
0.007063929	TMEM170,	0.307030908	0.015513285
0.000638258	CENPN	0.907036503	0.001618845
0.000670863	HSBP1	0.388086247	0.001164331
0.003948617	CRISPLD2	0.842529698	0.020397592
4.09995E-07	EMC8	0.441054897	0.031947251
0.016054372	BANP	0.74373211	0.000176061
0.000112775	APRT	0.200343079	0.028887719
3.32884E-07	TRAPPC2L	0.522554016	0.000198215
6.09374E-12	SERPINF1	0.672461251	5.03567E-05
0.024352898	HIC1	0.745821196	0.000274076
0.006999716	EMC6	0.331228505	0.04618661
0.048606191	CXCL16	0.282416811	0.00495101
0.043476213	VMO1	0.607499656	2.19481E-05
5.44285E-06	PSMB6	0.59088535	6.0562E-12
3.61165E-07	PFN1	0.380792911	3.53738E-12
1.25783E-08	C1QBP	0.363846251	5.66425E-05
0.001460615	DERL2	0.433952978	0.000181251
0.000210123	TXNDC17	0.654066949	6.4159E-10
0.018547694	ACADVL	0.419139575	0.001426391
0.00353298	CLDN7	0.688304236	0.002279627
0.033483317	EIF5A	0.332450287	0.008768041
0.00169666	CD68	0.45652719	3.46308E-06
0.008423806	TRAPPC1	0.464981413	8.2296E-07
2.46122E-15	HS3ST3B1	0.68778454	0.005903959
0.017266554	PMP22	0.398395284	0.021702331
0.045854209	TMEM11	0.480454022	0.010944448
0.002193416	LGALS9	0.439829259	4.40226E-06
0.004120789	COPRS	0.533518933	0.002731711
0.007084562	PSMD11	0.476766986	6.25559E-05
0.001094636	PCGF2	1.246351681	0.042811227
0.00010676	PSMB3	0.417533456	2.98843E-11
0.020260006	PSMD3	0.488863496	0.001479242
0.007177515	CCR7	1.667250885	0.010136598
0.004402588	RAB5C	0.302245393	0.000426677
1.10053E-05	MLX	0.271032415	0.039541607

0.012421526	VAT1	1.461132611	6.46127E-06
3.09181E-05	SLC25A39	0.436485919	2.81969E-05
1.77302E-05	GRN	0.418905327	2.01806E-06
0.000785285	SNF8	0.370431895	0.008808852
0.003212799	GNGT2	0.835208426	9.59439E-08
0.028842253	PHB	0.289331023	0.00149956
0.000876673	SLC35B1	0.606682673	0.000324146
0.044996739	MRPL27	0.410914506	0.002563297
0.04377051	LRRC59	0.49451062	0.002292673
0.000484065	NME1	0.699797594	3.84066E-07
0.027672239	SCPEP1	0.637331985	3.91918E-17
0.003699983	PSMC5	0.463126629	1.60958E-07
0.0070519	GNA13	0.37550026	0.012777138
0.002329449	PSMD12	0.456931647	0.002780752
0.0354489	KPNA2	0.36172741	0.002773275
1.20131E-09	ARSG	1.054009821	0.000191225
5.66395E-08	SLC16A6	1.300725232	0.002773275
0.000842694	WIPI1	0.755885879	0.000164749
1.31473E-07	CD300A	0.403945113	0.017872989
0.004504583	CD300LF	0.807591959	5.99276E-05
0.023688059	ATP5H	0.27037266	1.95128E-05
3.12469E-07	HN1	0.821299434	2.49387E-13
0.004630387	SUMO2	0.153561628	0.049406077
0.000140373	SLC25A19	0.576768126	0.000681082
0.000742838	GRB2	0.215583326	0.025608839
0.007286728	SPHK1	0.664667323	0.024311674
0.013171165	SYNGR2	0.408868577	1.60958E-07
0.046844352	CYTH1	0.40586056	0.011713498
1.5087E-05	ACTG1	0.242091438	0.007062791
0.002726408	MRPL12	0.319973045	0.021082859
0.00587755	P4HB	0.401659512	3.48773E-07
0.003934964	ANAPC11	0.248629817	0.003570591
0.035559793	SLC16A3	0.729340279	0.000193843
0.000117516	USP14	0.305025644	0.016255848
0.000155266	EMILIN2	0.28045099	0.00556341
4.05263E-09	MYL12A	0.253893687	0.000600352
2.14971E-05	MYL12B	0.326633881	5.88595E-05
0.025748473	NDUFV2	0.557828101	1.49825E-10
0.003561602	SNRPD1	0.314660008	0.000666092
0.018890979	GALNT1	0.302203336	0.004704381
7.97171E-05	ATP5A1	0.180029572	0.027559133
2.8682E-09	C18orf32	0.312696116	0.033569953
8.38929E-05	ACAA2	0.565754805	0.000486393
0.023152219	TXNL1	0.213044542	0.026473523
2.77296E-07	MALT1	0.716393968	0.002909604
0.000459675	SEC11C	0.648941915	9.20751E-08
0.027594204	CD226	0.989683687	0.00144084
0.003797506	TSHZ1	0.523038763	0.013257865
2.73169E-05	TXNL4A	0.394239709	2.41513E-05
3.53326E-07	FAM110A	0.815947189	9.32881E-08

0.006041588	FKBP1A	0.22974828	0.000115015
0.002415402	NSFL1C	0.429953807	0.000235354
0.024352898	SNRPB	0.428085207	3.34364E-07
0.000112425	SMOX	0.953279615	0.000753521
1.80112E-12	TMEM230	0.212591026	0.011553334
9.50754E-06	CDS2	0.413461024	0.01801554
0.008721818	GPCPD1	0.275854415	0.043311982
0.006598653	DSTN	0.386684957	0.000721713
4.59387E-19	RRBP1	0.367192488	6.99573E-05
0.01367685	SEC23B	0.356990751	0.034662582
0.005418495	NAA20	0.380361693	0.001866519
0.000546276	CST7	1.703654446	5.47039E-09
1.68475E-10	ABHD12	0.622974971	6.97457E-05
5.33808E-05	HM13	0.516757425	1.21426E-05
3.22688E-05	HCK	0.306147207	0.015672751
0.000941057	CHMP4B	0.445728547	0.001567965
0.000276222	EIF2S2	0.295472413	0.002630663
1.10326E-11	AHCY	0.404374588	0.003935921
0.001272636	DYNLRB1	0.247839998	0.049933713
0.009675974	PROCR	0.851395787	0.031624906
0.041116065	EIF6	0.474790657	3.58625E-07
0.047341035	ROMO1	0.324913146	0.00161718
1.83818E-13	SCAND1	0.272851632	0.010259808
0.014810136	C20orf24	0.381678565	0.008149379
6.2527E-13	RPN2	0.392647084	1.60541E-05
4.35755E-05	SRC	0.45841078	0.011802682
0.001031243	CTNNBL1	0.465880708	8.52414E-06
4.08603E-06	SDC4	0.832803927	0.045248064
0.000537327	SYS1	0.38903043	0.01436295
2.58287E-05	CTSA	0.807132874	2.06082E-13
0.003845571	MMP9	0.838545655	0.017790814
0.000962677	CD40	0.853474462	0.000148566
0.038844561	TP53RK	0.575960176	0.028500056
0.001068979	SNAI1	1.024542839	8.32933E-07
0.040975387	PTPN1	0.594675049	0.001474088
0.034602702	DOK5	1.642906891	0.020694766
0.000100549	RBM38	0.367165055	0.035521899
6.03325E-06	PMEPA1	0.695162295	0.039968818
0.00159988	RAB22A	0.401304079	0.008820867
0.001259294	CTSZ	0.451406508	2.21113E-05
0.004605468	ATP5E	0.169488743	0.001582669
0.02002245	PSMA7	0.388211047	6.61403E-10
0.004505194	ADRM1	0.509790093	1.15497E-07
0.011137991	SLCO4A1	1.007223122	0.046923119
7.329E-06	PPDPF	0.577768754	0.000166083
0.001419511	DNAJC5	0.386632349	0.023055136
0.041159715	BSG	0.276257048	0.000523751
0.000351468	POLR2E	0.233488054	0.017051391
0.015389149	GPX4	0.498009084	4.73382E-05
0.01833024	NDUFS7	0.175200999	0.026662184

7.21877E-17	UQCR11	0.282930704	2.30679E-05
0.000852155	AP3D1	0.317392782	0.031355973
0.00011955	OAZ1	0.303758854	2.01482E-09
	TIMM13	0.191040506	0.02776377
	SLC39A3	0.550320866	0.020080996
	MFSD12	0.567850371	8.74073E-06
	MRPL54	0.226286222	0.027458274
	MYDGF	0.520623856	1.11509E-06
	C19orf70	0.268415086	0.017259612
	CLPP	0.323872082	0.048099277
	TNFSF14	1.204685719	0.001078973
	ADGRE1	0.62975708	0.047620657
	PEX11G	0.769634042	0.031864801
	NDUFA7	0.342850701	0.019051029
	HNRNPM	0.226866097	0.009387006
	UBL5	0.234235001	0.006268015
	OLFM2	2.631847275	0.011039426
	ANGPTL6	1.429471969	0.036602347
	S1PR2	0.642602161	0.022117413
	MRPL4	0.293657202	0.028572699
	CDC37	0.290234911	0.035521899
	PDE4A	0.544063279	0.011046445
	ACP5	1.699308566	5.82185E-11
	ASNA1	0.355450213	0.002076922
	PRDX2	0.40050806	0.001124724
	DNASE2	1.1857883	1.95332E-18
	FARSA	0.325710331	0.046923119
	CALR	0.424135678	0.007062791
	C19orf53	0.22782683	0.014418324
	DDX39A	0.406107316	0.004286126
	GIPC1	0.397956771	0.045469379
	NDUFB7	0.197384386	0.006476772
	TPM4	0.661516332	1.28519E-06
	IFI30	0.488722774	0.02777235
	LSM4	0.292266251	0.049933713
	GDF15	1.878393829	0.001750585
	COPE	0.280688267	0.004088011
	NDUFA13	0.245454933	2.81741E-05
	PDCD5	0.52696999	0.00012814
	PEPD	0.458866551	3.7897E-05
	FXYD5	0.183833554	0.040131374
	TMEM147	0.311928255	0.001618845
	COX6B1	0.184766645	0.000407802
	PSENEN	0.413922475	0.008839462
	PPP1R14A	0.858276239	0.006432208
	YIF1B	0.425619533	0.001877763
	PSMD8	0.403166574	0.000111461
	ECH1	0.282263159	0.0031004
	PSMC4	0.349061824	0.024886067
	PLD3	0.405329376	0.002077929

RABAC1	0.353929379	0.008126184
PLAUR	0.356240165	0.036292368
KCNN4	1.236819247	0.011612537
PVRL2	0.571890951	2.92541E-05
APOE	1.249563662	0.000914823
APOC1	1.873292753	4.03124E-16
RELB	0.539885196	0.022704659
CALM3	0.518834328	2.8512E-05
AP2S1	0.228276116	0.007901193
NAPA	0.485058776	0.000387114
EMP3	0.302506299	0.003236789
HSD17B14	1.038116482	0.001041946
BAX	0.417113369	2.82332E-08
FTL	0.504393861	1.08996E-24
PIH1D1	0.350555409	0.035684034
PRMT1	0.468217116	2.71378E-05
IL4I1	1.961203218	3.27877E-10
ATF5	0.680473757	0.00069364
JOSD2	0.389084612	0.005842624
ETFB	0.332165043	0.000233111
NKG7	1.581690733	2.3955E-06
SIGLEC5	0.759485678	0.036763003
FPR2	1.372957983	0.000681806
NDUFA3	0.16922997	0.017149844
TFPT	0.346240311	0.005993401
LILRA5	0.587548479	0.014279581
LILRA4	1.814397553	5.71121E-08
LILRB4	0.425053449	0.043365917
ISOC2	0.422199541	0.00077643
CHMP2A	0.424103757	0.000319268
UBE2M	0.393197224	0.007401554
ATP6V1E1	0.39456081	0.000435925
BID	0.729071424	1.8854E-07
MRPL40	0.509965148	2.88673E-06
UFD1L	0.314074451	0.046082674
RANBP1	0.334355178	0.005182289
SNAP29	0.633027937	1.0548E-07
UBE2L3	0.354469715	2.21113E-05
SDF2L1	0.687701649	3.47395E-07
CHCHD10	0.283661598	0.001618845
MIF	0.423491656	0.000154678
UQCR10	0.421788003	7.62468E-09
MTFP1	0.585340652	0.028315808
TCN2	0.618129023	0.000990836
YWHAH	0.528894995	9.91471E-11
FBXO7	0.273625454	0.026183373
HMOX1	0.579950191	0.00180181
MYH9	0.451689442	5.39694E-06
GGA1	0.410415956	0.00148503
LGALS1	0.827296069	3.76279E-16

POLR2F	0.267844905	0.048586363
RPS19BP1	0.352163335	0.001862323
ST13	0.195875572	0.004552834
RBX1	0.25470222	0.005415307
ACO2	0.275210235	0.035130193
DESI1	0.741383544	1.04776E-06
SNU13	0.230115153	0.006122009
NDUFA6	0.334677134	0.000248144
LINC01315	1.321655062	0.029605081
RRP7A	0.390496879	0.000169283
CYB5R3	0.474436358	0.000119863
A4GALT	2.418431034	2.72538E-08
ARFGAP3	0.473615021	0.005220913
CRELD2	0.73090859	1.52542E-06
PIM3	0.745207008	0.000672889
SCO2	0.377342353	0.004303622
TYMP	0.717029951	3.48961E-19
ARSA	0.423904597	0.006901556
SAMSN1	0.413941478	0.012777138
ATP5J	0.292121332	2.02673E-05
CCT8	0.258365358	0.012362804
SOD1	0.274198229	0.006605084
IL10RB	0.420684716	0.000578483
IFNGR2	0.275575717	0.029488609
ATP5O	0.185997665	0.019849171
CBR1	0.351169135	0.025829079
HMGN1	0.265237006	0.044939798
MX1	0.649437631	0.045119944
PDXK	0.471611265	0.001826567
CSTB	1.01617891	1.45051E-22
RRP1	0.462218312	0.049579627
PTTG1IP	0.290357187	0.011073865
FAM207A	0.439050618	0.022789501
S100B	0.583338582	0.005277644
MT-ND6	0.293374762	0.040450291
CST6	1.382648044	0.001567965
METTL7B	2.585856222	0.031215767
MT1H	3.111863039	0.004781116
DIRAS1	3.02140047	0.00263929

Table S3

			M6		
**Ileum DEGs**	**log2FC**	**adj. p-value**	**Colon DEGs**	**log2FC**	**adj. p-value**
CXCL12	2.55192	0.0067716	IGLC2	-3.17721	0.0101667
ADAMDEC1	2.530327	1.102E-07	S100A9	-2.64957	0.0002984
SGPL1	1.979376	0.0079748	IL10	-2.54832	0.0250758
LINC00996	1.75329	0.0025989	IL1R2	-2.27668	7.2E-07
CD163L1	1.582094	0.0380549	IGHA1	-2.24545	0.0016816
AP2A2	1.52891	0.0118696	JCHAIN	-2.18994	0.0238966
C5AR2	1.516605	0.0144283	CD9	-1.99285	0.0003403
LDLR	1.498921	0.0365905	FCGR2B	-1.97934	3.766E-07
LGMN	1.487087	0.0017643	S100A4	-1.83163	4.905E-09
SEPP1	1.400902	0.008138	SERPINA1	-1.75346	0.0052829
TMEM176B	1.355314	0.0256388	HSPA6	-1.69674	0.0004609
TMEM176A	1.345522	0.0043038	CD1C	-1.64781	0.0092775
GCLC	1.190185	0.0161852	S100A6	-1.56816	8.758E-08
IGF1	1.144755	0.0238966	TSPO	-1.45287	0.0004105
CCL4L2	1.100687	0.002028	HSPB1	-1.34857	0.0105637
SLCO2B1	1.064719	0.0079748	PKIB	-1.26938	0.0294577
C1QA	0.998779	0.0002984	C1orf162	-1.16582	0.0028313
PDK4	0.89357	0.0067716	LGALS3	-1.03624	0.0161852
SAMSN1	0.804436	0.0438171	FCER1A	-1.0318	9.516E-06
RNASET2	0.729495	0.0146587	MNDA	-1.03051	0.0072374
			CAPG	-1.01602	0.0365905
			EMP3	-0.97391	0.0288963
			S100A10	-0.92707	0.0016816
			SH3BGRL3	-0.82366	0.000129
			TXNIP	-0.73775	0.0365905
			CKLF	-0.73397	0.0416826

M7

Ileum DEGs	log2FC	adj. p-value	Colon DEGs	log2FC	adj. p-value
FABP6	6.829558	8.813E-12	NRG1	-4.66056	0.0316859
FABP2	5.370396	0.0038979	S100A9	-3.81388	7.47E-18
GSTA1	5.053252	0.0171852	EGFL7	-3.26931	0.005173
RBP2	4.963251	0.0235346	RNASE1	-3.26841	3.602E-18
ALDOB	4.842818	0.0051608	S100A8	-3.24437	0.0046689
C11orf96	3.77939	3.007E-09	FCGR2B	-3.02326	1.543E-07
ADAMDEC1	2.92534	1.781E-23	SERPINA1	-2.79511	3.363E-06
LAG3	2.59104	0.0006705	S100A4	-2.71094	1.483E-13
CDKN1C	2.351416	0.0002544	HSPA6	-2.70474	0.0336046
RP11-556E13	2.102597	0.0068546	TSPO	-2.63861	2.526E-12
CCDC186	1.971945	0.000204	IGLC2	-2.55601	3.031E-11
IL2RA	1.896137	0.0028704	CD9	-2.54068	1.105E-06
ITGB7	1.786543	2.71E-05	IGSF21	-2.53621	0.0371719
CXCL1	1.701377	0.0079016	CX3CR1	-2.47475	0.0215915
NET1	1.675645	0.004411	CCL2	-2.44569	0.0026227
IL6	1.632425	0.0019836	S100A6	-2.42948	0.0183991
CXCL12	1.632341	0.0024018	IGHA2	-2.30984	0.0003131
SCN4B	1.598664	0.0419419	PKIB	-2.27189	0.0060956
EPHX1	1.482763	0.0094776	FGR	-2.25667	0.0095446
CCL18	1.406527	0.0135273	FCGR3A	-2.23989	1.723E-09
FAM21A	1.389877	0.003154	CD1C	-2.20956	0.0177356
GCLC	1.326961	1.8E-06	ELK3	-2.18869	0.0336046
TSC22D1	1.312605	0.0051608	IGHA1	-2.14501	5.337E-12
EPB41L2	1.256445	0.0028704	ITGAM	-2.11004	6.447E-05
LINC00996	1.15774	0.0362523	MAP4K4	-2.0964	0.028487
ITM2C	1.030049	0.005173	IL1RN	-2.0518	0.009045
CTTNBP2	1.015478	0.025418	THBD	-1.96604	0.0023647
LGALS3BP	1.015017	0.0257843	IL1R2	-1.92441	0.0015983
TMEM176B	0.937185	0.0027388	MIR3945HG	-1.91819	0.00378
RNASET2	0.812034	0.0007067	ALOX5AP	-1.83493	6.929E-05
CTSC	0.709858	0.0068546	ZFAND2A	-1.81539	0.0001899
			DNAJA4	-1.81142	0.0028704
			HSPA1A	-1.80868	7.281E-09
			CAPN2	-1.75938	0.0139957
			HSPA1B	-1.75274	1.105E-06
			ITGAX	-1.73802	0.002648
			TESC	-1.69791	0.0320926
			BAG3	-1.66248	0.0208631
			CAPG	-1.65124	2.884E-06
			G0S2	-1.60866	0.0001076
			CCND2	-1.58445	0.0097298
			TREM2	-1.55779	0.0487314
			F13A1	-1.50789	0.0005583
			PNP	-1.45366	0.0079016
			JCHAIN	-1.42891	6.929E-05
			C1orf162	-1.42299	1.53E-05
			DNAJB1	-1.34058	0.0010428

APOE	-1.3051	0.0007355
MNDA	-1.29182	8.596E-05
GSN	-1.28078	2.862E-06
PID1	-1.2506	0.0073455
HSP90AA1	-1.24412	0.0100152
HSPE1	-1.24356	0.0135273
OLR1	-1.17232	0.01476
S100A10	-1.16236	0.0316859
GYPC	-1.14576	0.033915
HSPD1	-1.12518	0.028487
HSPB1	-1.12433	0.0395188
IL10	-1.12181	0.005173
EMP3	-1.02776	4.196E-06
IL1B	-1.01189	0.0060956
HCST	-1.00631	0.0114317
DNAJA1	-1.00456	0.003154
CD37	-0.95545	0.0120001
SH3BGRL3	-0.94707	0.0064653
HSP90AB1	-0.91948	0.0139957
LGALS1	-0.87703	0.0332827
FCGR2A	-0.86714	0.0042169
TLR2	-0.84517	0.0362523
FXYD5	-0.8126	0.0263067
IER3	-0.76941	0.0026132
CD14	-0.70974	0.0487314
ANXA1	-0.68752	0.0487314

M8

Ileum DEGs	log2FC	adj. p-value	Colon DEGs	log2FC	adj. p-value
FABP6	6.577636	3.719E-09	NRG1	-4.45772	3.054E-05
C11orf96	2.546696	4.009E-12	S100A8	-3.47169	0.0001116
LAG3	2.311866	0.0048472	RNASE1	-3.20117	4.482E-13
KCNMB4	2.157909	0.0120894	S100A9	-3.11843	4.928E-12
IL2RA	1.822674	4.697E-05	S100A4	-2.91156	7.033E-20
CXCL12	1.702399	0.0129776	TSPO	-2.64114	3.686E-14
STON2	1.679212	0.0442727	FCGR2B	-2.55395	9.564E-06
CXCL1	1.669693	0.0013893	FCN1	-2.47075	0.0223683
FAM101B	1.624848	0.0096366	SERPINA1	-2.37267	4.588E-05
LAMP5	1.52279	0.0120894	S100A6	-2.35975	1.561E-11
ID3	1.519245	0.0185545	CCR7	-2.24142	0.018486
FOLR2	1.517285	0.0120223	CCND2	-2.18511	0.0001136
TBC1D16	1.5139	0.0442727	APOE	-1.94807	4.647E-06
CDKN1C	1.513398	0.0375272	PNP	-1.9045	0.0001291
LILRB5	1.498052	0.0086476	IL1R2	-1.87454	0.0011636
CCDC186	1.491653	0.0065956	FLNA	-1.87414	0.0112523
SMPDL3A	1.473461	0.0246511	MIR3945HG	-1.81145	0.0222477
EPHX1	1.431793	0.018041	FGR	-1.7841	0.0013654
TSC22D1	1.423582	1.619E-05	G0S2	-1.74586	3.202E-10
IFITM10	1.418186	0.010826	BCAT1	-1.74542	0.018041
ADAMDEC1	1.381546	2.863E-05	NT5DC2	-1.66788	0.0120894
WLS	1.32357	0.0225979	IL3RA	-1.65943	0.042421
CDKN1B	1.320176	0.0216011	CAPN2	-1.6531	0.0460903
GPR34	1.307339	0.0074719	HLA-DQB2	-1.65037	0.0484414
SEPP1	1.294363	0.0092006	IL4I1	-1.62555	0.0051504
FCHO2	1.260573	0.018041	ZFAND2A	-1.59441	0.018041
CD163L1	1.24496	0.0127485	CAPG	-1.51657	0.0004754
ITGA9	1.244535	0.0246511	IL1RN	-1.51492	0.0233733
SIAH2	1.230205	0.0048472	FPR1	-1.50676	0.0197436
ITSN1	1.216292	0.0460903	ALOX5AP	-1.40123	0.0120894
PALLD	1.211384	0.0401737	HSPE1	-1.39949	0.0104187
QPRT	1.184548	0.0022396	S100A10	-1.39431	1.825E-05
TMEM37	1.180156	0.0442727	RHOC	-1.35711	0.0013654
EPB41L2	1.175423	0.0001116	LIMD2	-1.33502	0.0311046
LINC00996	1.175252	0.004882	UGCG	-1.32223	0.0454857
CLEC11A	1.14851	0.018041	C15orf48	-1.26898	0.0246511
SERPING1	1.090827	0.0241708	PKIB	-1.25508	0.0233733
CD209	1.058357	0.0030592	PPM1N	-1.25247	0.0499314
FRMD4A	1.049247	0.0484414	GBP1	-1.21504	0.0233733
LGALS3BP	1.028004	0.0022396	TNFAIP8	-1.19805	0.026798
THBS1	1.020895	0.0129776	MMP12	-1.17466	0.000149
ITM2C	0.975018	0.0083387	LSP1	-1.17378	0.0233733
GCLC	0.937312	0.0026482	HSPD1	-1.13686	0.0484414
EBI3	0.903389	0.0120894	C1orf162	-1.13312	0.0031864
C5AR2	0.896199	0.0225979	DRAM1	-1.12719	0.0442727
ZNF331	0.850239	0.0003907	ITGAX	-1.10881	0.0442727
RNASET2	0.84482	3.77E-05	SH3BGRL3	-1.07204	0.0134038

CPM	0.832364	0.004882	GNA15	-1.04528	0.045017
MRC1	0.789323	0.026798	ETHE1	-1.039	0.026798
CTSC	0.780649	0.0442727	MNDA	-1.02927	0.0017424
TIPARP	0.767769	0.0129776	HSP90AB1	-1.02458	0.0225979
JUNB	0.762651	0.0216028	GSN	-1.01511	0.0048472
C1QA	0.747374	0.0010554	EMP3	-1.00294	3.497E-05
SIGLEC10	0.742513	0.0225979	LGALS1	-0.92138	4.335E-11
ITGB7	0.726137	0.0233733	CD44	-0.91571	0.018041
FCGRT	0.716885	0.0024663	ANXA2	-0.91497	2.863E-05
SERPINF1	0.689008	0.0244005	LYZ	-0.89517	7.021E-06
HERPUD1	0.687721	0.0074719	NME1	-0.87794	0.0186724
LAIR1	0.684482	0.0257539	TUBB	-0.86167	0.0022396
VSIG4	0.642994	0.010826	CCT4	-0.85739	0.018041
RGS1	0.627188	0.0442727	CKLF	-0.83017	0.0440096
VEGFA	0.610902	0.0442727	CTSH	-0.80966	0.034385
AKAP9	0.610383	0.0442727	HCST	-0.80421	0.0484414
SMAP2	0.589033	0.0092006	PMAIP1	-0.75939	0.0120894
HLA-E	0.562938	0.018041	CD48	-0.73499	0.0015044
MCL1	0.542432	0.0061189	TYMP	-0.70851	0.0013893
CHMP1B	0.530555	0.0092006	CDKN1A	-0.70755	0.0434032
ATP1B3	0.482065	0.0382875	TCEB1	-0.6997	0.0335913
TYROBP	0.420233	0.02606	VIM	-0.69583	0.0442727
AIF1	0.41511	0.0434032	SSR3	-0.67758	0.018041
			ENO1	-0.65249	0.0102624
			LMNA	-0.64626	0.0300145
			ATOX1	-0.62354	0.0363043
			PSMA4	-0.62294	0.0236697
			PRDX1	-0.58491	0.0233733
			DNAJB6	-0.57223	0.0470819
			ANXA1	-0.56984	0.0013654
			FXYD5	-0.55603	0.0442727
			YWHAH	-0.55113	0.0447751
			COX7A2	-0.4957	0.0442727
			ACTG1	-0.47842	0.0442727
			PSAP	-0.38329	0.0434032

Table S4

	Healthy	Crohn's d
		Figure 3f
n	9	4
Female (%)	1 (18)	1 (25)
Age (Median, IQR)	50 (41)	26 (10)
Montreal classification (%)		
L1		0
L2		2 (50)
L3		2 (50)
L4+		0
B1		3 (75)
B2		0
B3		1 (25)
p+		0
E1		
E2		
E3		
IBD-Unclassified		
New diagnosis (%)		0
)isease duration (years, median IQR))		7 (15)
Bio-Naïve (%)		3 (75)
Biologic Treatment		
anti-TNF		0
anti-IL12/23		0
anti-α4β7		1 (25)
Steroids		1 (25)
C-reactive protein mg/L (median, IQR		7 (25)
missing (n, %)		
Haemoglobin g/L (median, IQR)		132 (55)
Albumin g/L (median, IQR)		38 (2)
missing (n, %)		1 (25)
Faecal calprotectin ug/g (median, IQR		540 (686)
missing (n, %)		

Endoscopic Assessment	SES-CD		Mayo	
	Median (IQR)	n (%)	Median (IQR)	n (%)
Quiescent	0 (1.0)	6 (46)	0 (1.0)	8 (50)
Mild	3.5 (1.0)	4 (31)	1.0 (0.8)	4 (25)
Moderate	6.0 (2.0)	3 (23)	2.5 (1.0)	4 (25)

disease	Ulcerative colitis (inc IBD-U)	
Figure 7a	Figure 3f	Figure 7a
5	3	9
4 (80)	2 (66)	3 (33)
37 (22)	51 (26)	54 (17)
0		
2 (40)		
3 (60)		
1 (20)		
5 (100)		
0		
0		
0		
	0	0
	1 (33)	4 (44)
	2(66)	3 (33)
	0	2 (22)
2 (40)	1 (33)	2 (22)
1 (6)	13 (19)	15 (17)
3 (60)	1 (33)	6 (66)
0	0	0
2 (40)	0	0
0	2 (66)	3 (33)
0	0	0
22 (108)	9 (1)	8 (5)
1 (20)	1 (33)	2 (22)
139 (44)	143 (14)	145 (13)
33 (7)	38 (5)	38 (8)
3 (60)	1 (33)	3 (33)
425 (723)	698 (988)	597 (1048)
2 (40)	1 (33)	2 (22)

Table S5

cDC1 vs. cDC2 + cDC3			cDC2 vs. cDC1 + cDC3		
gene	avg_logFC	adj. p-value	gene	avg_logFC	adj. p-value
SNX3	2.35120986	0	PLAC8	1.77468079	5.589E-64
CPNE3	3.62323473	0	RAPGEF4	4.5189123	3.917E-56
WDFY4	3.27999	0	DSG2	4.15853256	1.06E-53
CADM1	7.47699463	2.9127E-256	CD207	4.10219746	1.273E-50
TMEM14A	3.90430432	2.3794E-226	FAM110A	1.0261185	2.325E-50
VAC14	2.9508177	1.9926E-216	DUSP5	1.62792647	1.374E-46
CLNK	4.94218569	1.4533E-215	PAPD5	0.89302351	1.546E-40
IDO1	3.45042783	2.5352E-207	HIC1	1.06916074	9.477E-36
C1orf54	3.25839592	2.6984E-203	SNAP29	0.98585859	2.924E-34
CLEC9A	4.66200681	5.5634E-199	PRMT9	1.06072055	9.036E-33
SHTN1	2.068967	2.7118E-190	CFAP73	4.50070976	7.053E-32
PLCD1	2.97780225	9.5921E-169	CIB1	0.76670832	3.41E-31
ENPP1	6.65894242	5.8854E-161	IL2RG	0.83707635	6.837E-31
PLPP1	3.47400895	2.0543E-160	IRF4	1.92638601	1.328E-30
HLA-DOB	2.80830173	4.0386E-159	GHRL	1.51567576	9.32E-30
VCAM1	5.08588122	2.8213E-153	LYST	1.18892937	7.546E-29
CAMK2D	2.64779144	1.3566E-150	EXOC3	1.18580999	9.406E-29
TACSTD2	5.34641349	1.2963E-133	ABCB1	2.16913639	1.817E-28
KIF16B	2.10625145	8.1537E-131	TSEN54	1.84991687	1.657E-27
KIAA0226L	4.2280354	4.9991E-130	SPIB	1.75352495	1.92E-27
DBN1	7.31952504	3.8945E-124	SPINT2	0.55663504	5.473E-27
MZT2A	1.96864868	3.6898E-113	CEACAM1	4.47521763	6.985E-27
GCSAM	4.69809553	2.1541E-108	TBC1D9	0.88932367	7.099E-26
TMSB4X	0.80062346	2.1541E-108	PPP1R14A	1.66677557	2.245E-25
C1orf21	4.58549601	5.2931E-107	CDH17	1.50890267	4.122E-25
PARP1	1.67565291	4.3853E-106	TMEM131	0.76473008	5.532E-25
FKBP1B	3.64871997	4.945E-99	GABARAPL2	0.61911806	7.756E-25
CLCN4	5.58082203	3.14289E-98	SLC38A1	1.30810798	1.339E-24
BATF3	1.78251732	9.29873E-98	TRABD2A	1.70486085	4.415E-24
CYB5R3	1.40756239	2.14388E-97	FCMR	1.89626853	4.732E-24
PPT1	1.45937911	1.03322E-96	PAK1	0.66276977	5.225E-24
PDLIM7	2.90460546	4.27838E-96	GPR65	0.98090786	1.471E-23
ACTG1	1.04521506	1.27877E-95	BCL7A	1.34271809	4.796E-23
RAB7B	4.17138092	4.73148E-94	PSTPIP2	0.91955697	9.218E-23
RPLP1	0.55053833	1.88415E-91	IL22RA2	6.24475091	9.934E-23
AIM2	3.71107406	2.32045E-91	LAMP3	2.28702314	2.424E-22
ATP6V1H	1.7148255	2.5007E-91	DUSP22	0.88399133	2.596E-22
TSPAN33	1.58076673	7.4061E-91	PPP1R16A	1.69462965	5.468E-22
LRRCC1	2.08018042	7.07492E-89	CD1E	1.45043494	1.951E-21
BTLA	4.08082019	9.69038E-89	RNF2	1.00848619	3.519E-21
FNBP1	1.31515151	1.56863E-86	CLN8	0.8447397	5.047E-21
RGS10	0.96216141	4.72861E-86	HLA-DQB2	2.10165139	6.352E-21

EGLN3	4.62791882	1.00672E-85	PKIB	1.73100082	1.57E-20
CSRP1	1.78589024	4.75291E-85	SLC8A1	0.49492196	1.686E-20
CCND1	4.06028735	9.04362E-84	SFTPD	3.13002808	1.799E-20
DPP4	3.832066	9.86607E-84	GNB5	1.59633962	6.376E-20
LAG3	3.27362692	4.78517E-83	MTMR12	1.24977455	6.943E-20
UCP2	1.14804572	9.45441E-83	HECTD1	0.96315209	9.493E-20
SRI	0.97691492	1.06553E-80	GIPR	2.35006819	1.6E-19
PPM1J	3.24153673	3.30796E-80	CD1A	2.20978522	1.622E-18
ASB2	2.77276834	4.07162E-79	ITM2C	0.92042338	1.74E-18
PSMB9	1.08752495	4.43115E-78	MBOAT7	0.99309902	3.018E-18
MIR4435-2HG	1.72042119	4.43359E-78	UBE2E2	0.67490165	5.839E-18
CDK2AP2	1.31166792	4.85699E-78	AREG	0.71995349	6.091E-18
CPVL	1.64059502	3.37245E-77	TTC26	1.77858825	6.744E-18
CNFN	1.98126326	8.83499E-77	TSEN34	0.82013089	8.303E-18
XCR1	8.48141953	1.84482E-75	GNGT2	1.15497335	8.397E-18
ACTB	0.65269833	3.6046E-72	SMAP2	0.47782016	1.527E-17
PLPP3	5.76290033	1.99387E-71	TSHZ1	1.15726509	5.153E-17
DCTPP1	1.66622915	5.24798E-71	MLLT4	1.43955607	1.107E-16
CKB	2.63062375	6.74695E-71	SYT2	5.60131979	1.187E-16
LPP	1.46737522	1.29639E-70	RANBP2	0.57449837	1.76E-16
LSM6	1.21628242	8.87035E-70	SPATS2L	0.82496194	1.918E-16
CCDC74A	5.42165117	1.32296E-69	SLCO4C1	4.90689327	2.266E-16
FMNL2	3.56391198	9.27157E-69	ZDHHC14	1.68550661	2.916E-16
TMEM50B	1.28541266	5.23221E-68	NUDT1	0.60926718	2.916E-16
MZT2B	1.05676663	7.57893E-68	SCN4B	1.35868728	4.149E-16
SFT2D2	1.23924335	2.41511E-67	PALLD	1.1434997	4.412E-16
PAWR	5.19734437	2.70065E-67	ACAA1	0.58147571	9.996E-16
DEF8	1.97911264	5.17417E-65	CFP	0.74235368	1.044E-15
SNX22	4.99139359	7.38138E-65	JAML	0.59581106	1.822E-15
AC133644.2	3.43547169	1.04373E-64	CEBPZ	0.75216903	1.896E-15
PNOC	4.45677372	1.16819E-64	PPA1	0.61620666	1.986E-15
TSPAN2	4.79480778	1.35887E-64	ITGB1BP2	3.74358541	3.581E-15
NDUFC2	0.84361559	3.81428E-64	IL18	0.76144272	3.925E-15
TAP2	1.40192598	8.3954E-64	ARHGAP22	1.09777058	3.952E-15
MRC2	2.67480532	3.72482E-62	SEL1L3	0.69920632	4.801E-15
IQCE	2.57913754	3.38079E-61	ENTPD1	0.96836568	5.569E-15
NUBPL	1.89552677	1.17654E-60	ELMO1	0.63623224	7.2E-15
SLC46A3	2.01685095	3.32917E-60	NR4A3	0.52105066	8.004E-15
BIK	4.28285205	3.56608E-60	SCAF11	0.39390415	9.451E-15
DLGAP4	1.52770824	4.94303E-60	PLD4	0.70733426	1.495E-14
REPIN1	1.6194029	5.78588E-60	ZC3HAV1	0.51749531	1.589E-14
CCDC126	3.24049243	1.08231E-59	CACNA2D3	0.97925797	2.916E-14
EHD4	1.97105923	1.59378E-59	FNDC4	2.42665227	4.092E-14
CDH2	5.87026732	2.34074E-59	TMEM14C	0.43927829	5.077E-14
GPR31	5.22975126	9.10532E-59	ELAVL4	1.29238161	6.012E-14
CYP2E1	5.51183851	5.83821E-58	ASMTL	0.75509158	7.142E-14

PPY	6.08511311	6.71958E-58
LIMA1	1.84466418	6.87596E-58
PNMA1	3.11454088	3.62613E-57
TMED3	1.45519089	4.9765E-57
FBXO27	6.10101941	9.21647E-57
CD59	1.65032266	9.36737E-57
TSPAN15	2.12837301	4.38347E-56
SLC25A4	4.10329011	4.65034E-56
SAMD12	3.61979515	6.44166E-55
TSTD1	1.73699225	7.2411E-55
DYSF	5.55203718	1.58838E-54
EIF6	0.75889468	2.74741E-54
NAAA	1.4765605	2.52851E-53
ATP6V0E1	0.62104605	4.64967E-53
ATP1A1	1.26644986	1.13308E-52
SLC9A9	1.91521418	9.09375E-52
DNASE1L3	1.63122298	1.38118E-51
SERPINF2	2.40851074	1.88042E-51
CCSER1	1.91473164	5.99458E-51
ROGDI	1.41286659	2.93078E-50
PREX2	4.62780952	5.9055E-50
PPP1R3G	3.31121861	7.49926E-50
NME4	1.61769114	9.38221E-50
DSTN	1.16059639	1.95706E-49
TSPAN13	4.11739442	3.19855E-49
SERTAD3	1.43101537	9.98629E-49
PLEKHA5	1.64482911	1.18856E-48
CD74	0.60088792	4.00775E-48
CRTAM	2.2694565	4.90951E-48
CCNG2	1.71651662	7.01685E-48
GSTM4	1.56998307	7.52954E-48
OSBPL9	1.27891764	7.8474E-48
RUSC1	1.64905764	8.58335E-48
RGCC	1.1312421	9.11067E-48
RNASEH2C	1.5346953	9.57397E-48
G3BP2	1.42019767	1.24751E-47
RAB15	7.34125283	1.44952E-47
MYO9A	1.78126215	2.07325E-47
SEPT11	1.52684616	3.09942E-47
CLEC1A	4.21151954	3.9155E-47
FUT8	2.35178841	5.91121E-47
PTPN22	1.29583348	7.56922E-47
ENSA	0.91996627	1.46613E-46
SLC24A4	3.84619868	1.59385E-46
ATG3	0.76578523	4.64181E-46
MAP2K6	2.73042358	1.07895E-45
CCND2	0.61615948	8.294E-14
SMNDC1	0.57685516	8.539E-14
RBM38	0.81027465	9.872E-14
UBN2	0.77960191	1.573E-13
CLEC4A	0.76783674	1.594E-13
VPS41	0.59371295	1.634E-13
SMAGP	2.03031446	2.671E-13
YPEL5	0.58377675	3.319E-13
PFDN5	0.3311898	5.767E-13
PIP4K2A	0.61355171	7.54E-13
U2AF1L4	0.62895884	8.422E-13
IKBKB	0.96481665	1.041E-12
CXCR3	0.96428853	1.138E-12
PRELID1	0.47719531	1.157E-12
PRAM1	0.94650943	1.175E-12
AKNA	0.48545605	1.447E-12
SH3BP2	0.55676522	1.459E-12
SLC39A3	0.7317	1.681E-12
LST1	0.53571464	1.762E-12
SLC5A6	1.08410966	1.933E-12
TRIB2	2.00736353	2.107E-12
C11orf31	0.4761888	2.121E-12
RTFDC1	0.45012548	2.394E-12
MCCC1	0.82771376	2.427E-12
NUDT19	1.01936793	2.719E-12
FAM122C	1.40828186	3.075E-12
PLSCR1	0.60462749	3.437E-12
KIT	2.32772815	3.706E-12
LTB	1.16988444	3.717E-12
RGL4	1.90602842	3.717E-12
SLC4A3	2.0698617	4.946E-12
ELOVL6	5.01539489	6.019E-12
ANKRD55	4.08663807	7.128E-12
BDKRB2	4.85220871	8.23E-12
RP5-1171I10.5	0.57941612	8.626E-12
RASAL1	2.73207755	1.321E-11
PRR13	0.44313352	1.33E-11
CDC123	0.47386955	1.439E-11
UAP1	0.71823798	1.708E-11
FABP4	3.22059183	1.726E-11
PPP1R16B	1.59693867	1.992E-11
P2RY10	1.18013155	2.148E-11
GLYATL2	3.03839175	2.289E-11
ARL4C	0.45339344	2.622E-11
LPXN	0.83991831	2.764E-11
LBH	2.43459723	2.871E-11

CIB2	2.97416736	1.65198E-45	VPS35	0.324065	3.212E-11
BLOC1S2	0.774556	6.56781E-45	SNAI1	1.04986207	3.277E-11
FLOT2	1.4144726	7.10096E-45	RP11-863P13.	1.23316353	3.302E-11
SMS	0.94491476	8.59118E-45	PHTF1	1.03469161	3.502E-11
APOL3	1.82318988	1.47901E-44	RPS6KA1	0.68978729	4.251E-11
NCALD	4.83535532	2.81874E-44	HOPX	2.54037256	4.795E-11
GYPC	1.60690765	6.41624E-44	PIK3R6	1.06255075	5.212E-11
BASP1	0.98890655	7.20781E-44	HES4	0.59613092	5.302E-11
BCL2L11	1.55194824	8.53533E-44	GPRIN3	0.81626162	5.373E-11
MYEF2	2.02638617	3.70335E-43	ST20	1.17123468	5.638E-11
KIAA1958	2.87117717	6.85688E-43	UVRAG	0.52140021	6.3E-11
ST7	1.83235094	7.90687E-43	DERL1	0.46555544	6.353E-11
TOX	4.5790238	1.60637E-42	TRPC6	4.36474298	6.589E-11
CEP164	1.47509978	2.74843E-42	WBP11	0.43004965	7.402E-11
AC017002.1	4.11072299	4.22016E-42	ITSN1	0.95597493	7.436E-11
ST3GAL5	1.93382314	1.16543E-41	AHI1	0.77993351	8.057E-11
IFT20	0.89504328	1.76543E-41	DMTF1	1.00447184	1.145E-10
FLNB	2.94447492	1.78612E-41	COMMD6	0.37518953	1.223E-10
ATP6V1D	1.03429957	1.98704E-41	DDX24	0.40873983	1.407E-10
TLR3	3.38301696	3.12333E-41	SYTL3	0.77354061	1.463E-10
CASP2	2.19633771	3.17473E-41	ARL17A	0.96280259	1.496E-10
PPM1H	3.05825835	3.55106E-41	HNRNPUL1	0.30804885	1.522E-10
PTK2	4.53220099	1.0559E-40	FAM162A	0.38783986	1.636E-10
MYL6	0.69193084	2.14254E-40	ZNF263	1.08425011	1.926E-10
OXSR1	1.34188649	3.36946E-40	TNFSF14	1.33299218	2.015E-10
TSPAN6	7.76289047	3.75464E-40	SLC18A2	3.74263502	2.054E-10
SPATS2	1.56233848	3.9702E-40	DSC2	1.04507114	2.219E-10
ELOVL5	1.09923616	4.22879E-40	ABCC1	0.85574263	2.787E-10
PPM1M	1.36080387	5.79278E-40	SLFN13	1.49321536	2.794E-10
GSTP1	0.80197945	9.70225E-40	GATA1	5.42529062	2.897E-10
RCN2	1.12713664	9.70225E-40	SS18L2	0.50842053	2.961E-10
FAM102A	3.96450003	1.70403E-39	HPS5	0.50393989	3.09E-10
PSMC6	0.77461575	2.44662E-39	FRAT2	0.69702423	3.101E-10
ACOT7	2.28028669	2.70855E-39	KCNK17	2.86994129	3.273E-10
TLR10	2.22903886	4.05349E-39	ABLIM1	3.67630178	3.341E-10
AGPAT1	1.32218983	4.59444E-39	NCOA3	0.47883592	3.38E-10
HLA-B	0.90755351	4.93114E-39	FSCN1	1.07251824	3.678E-10
SHISA4	2.632573	5.0321E-39	RPS19	0.24796752	3.734E-10
CCDC186	1.39972345	1.06014E-38	TAF10	0.39379025	4.163E-10
TNNI2	1.1151756	1.35949E-38	TESPA1	1.70006539	4.387E-10
RAB32	1.30662379	2.49883E-38	SLC30A1	0.87756349	4.71E-10
SUSD3	1.9634858	4.72188E-38	GLDN	2.26856752	5.105E-10
ICOSLG	1.51266639	5.97836E-38	DBF4	0.64939455	6.047E-10
CCDC6	1.297243	7.05984E-38	PFKP	0.98016524	7.351E-10
C20orf27	1.12566677	9.76105E-38	LAD1	3.76836828	7.381E-10
SRP14	0.44945766	1.13682E-37	KDM2B	0.62522373	7.665E-10

C16orf45	3.4139984	1.88167E-37
RP3-522D1.1	4.22085218	2.29909E-37
HLA-A	0.74705156	2.39467E-37
GLTP	1.03205584	2.60093E-37
KCND3	5.70956413	4.84004E-37
CDC42EP3	2.03938208	1.15516E-36
EEF1B2	0.57969008	2.04465E-36
ASAP1	1.61818137	3.06253E-36
LYRM4	1.25893176	3.75543E-36
FRMD4A	2.72240488	3.9311E-36
TJP2	2.01357452	3.99699E-36
NAALADL1	2.46560513	4.52076E-36
MAGEF1	2.09301718	6.12285E-36
TRMT112	0.63129041	1.02086E-35
DGAT2	2.0198756	1.31087E-35
TRIP6	1.78977595	1.37281E-35
MACROD2	4.87348533	1.89346E-35
UPF2	1.24343858	2.36732E-35
LGALS1	0.93814461	4.94414E-35
ZYX	0.96271905	5.41332E-35
QPRT	1.99213867	5.50499E-35
FAM46B	2.82467422	6.17499E-35
ATG16L1	2.94180533	6.17499E-35
MAP4	1.00332418	7.64647E-35
APOL1	2.13805079	8.94919E-35
DAPK2	2.16442975	1.10084E-34
RAB30	2.29001292	1.91511E-34
PFDN2	0.82105979	2.24899E-34
CLCN1	4.17097985	2.66484E-34
DST	1.22366683	3.96381E-34
ERICH5	7.82270161	4.77885E-34
RP11-25K19.1	3.49966957	6.19436E-34
PPIC	3.92207796	1.1162E-33
IDO2	2.15315423	1.1839E-33
GCHFR	1.43765999	1.43939E-33
B3GNT7	1.89982102	1.67682E-33
ZEB1	1.98173607	1.99012E-33
C5orf24	1.51840118	2.30289E-33
DMD	3.43572978	2.65446E-33
GINM1	1.13935893	2.77535E-33
RP11-798K3.3	3.18170018	3.10421E-33
BOD1L1	0.70543322	4.14032E-33
SDHA	1.19062875	4.47557E-33
ROBO1	4.04586033	7.80005E-33
ATP6V0A2	1.64903563	1.14019E-32
RPS20	0.41136198	1.15299E-32

GADD45A	0.94797435	7.672E-10
RGS13	1.00544211	8.048E-10
AC013461.1	0.75581639	8.314E-10
ABHD15	0.93281872	8.751E-10
GALNT3	0.96276367	8.983E-10
FHL1	1.94731681	9.335E-10
TOP3A	0.64990549	9.687E-10
DAZAP2	0.31652341	9.73E-10
SLC41A2	0.82659705	1.08E-09
SESN2	0.83642317	1.131E-09
DDHD1	0.60748286	1.432E-09
NCR3	3.20771904	1.475E-09
FAM178B	4.29800167	1.779E-09
CLDND1	0.53871128	1.779E-09
TCTN3	0.70344803	1.845E-09
TMEM8B	1.05160349	1.859E-09
FDFT1	0.67784006	1.913E-09
RNF213	0.66397103	2.173E-09
FAM118A	0.9222398	2.222E-09
MTA3	1.57070627	2.596E-09
LAMTOR1	0.35594966	2.733E-09
GALNT1	0.46153494	3.255E-09
GPAT3	0.69633206	3.286E-09
TEX30	0.87159938	3.312E-09
MALT1	0.70779305	3.738E-09
RNF125	0.91974799	3.895E-09
RNF24	0.90807704	3.933E-09
CTNND1	0.7556431	4.136E-09
ZNF7	0.75596779	4.263E-09
YDJC	0.51175158	4.918E-09
FAM89B	0.62164985	4.932E-09
RP11-446N19	1.505993	5.092E-09
PRKAB1	0.97208786	5.321E-09
GDI2	0.32400373	5.524E-09
CD101	0.85368733	5.583E-09
TAX1BP1	0.28835769	5.671E-09
HIF1A	0.68642193	5.85E-09
CNOT6L	0.45308269	5.89E-09
PTPN6	0.41088249	5.943E-09
DCTN6	0.43044764	6.205E-09
PDE4A	0.74906576	6.217E-09
ANGPTL6	2.77904086	6.228E-09
OSBPL8	0.35897117	6.311E-09
CHKA	0.50540465	6.406E-09
TARS	0.47177857	6.931E-09
ARID3A	1.1429517	7.192E-09

EPS8L2	3.33830461	2.0024E-32	RAP2B	0.32548615	8.674E-09
FAM221A	2.43207531	2.20307E-32	FAM107B	0.538321	1.024E-08
GM2A	0.85253914	2.26885E-32	MTHFD2	0.51202565	1.068E-08
SLC25A29	1.38561866	4.23085E-32	STAM	1.18870886	1.068E-08
C19orf53	0.46301965	4.35606E-32	KCNMB1	1.0263824	1.218E-08
CBL	1.30009221	5.36748E-32	PTPRC	0.28833826	1.38E-08
PMM1	1.19179961	8.01901E-32	CD86	0.79379909	1.409E-08
HAVCR2	0.8352458	8.02468E-32	MEF2D	0.66287259	1.535E-08
AC096772.6	3.12688065	1.03075E-31	ATP10A	1.54302715	1.865E-08
FBXO17	5.85301031	1.21767E-31	SEC14L1	0.540705	1.876E-08
RPL8	0.44411072	1.54744E-31	IFI6	0.72496631	2.008E-08
METTL12	1.34699158	1.63882E-31	GBP4	0.58527271	2.318E-08
PPP1R14B	0.95337484	2.23387E-31	SEPT7	0.41222016	2.371E-08
ARHGAP20	2.17755371	2.24887E-31	CCL22	2.98188192	2.482E-08
CNN2	0.70975893	2.32035E-31	AFF3	0.82747845	2.989E-08
LINC01356	4.52279216	2.40922E-31	GPX7	1.66199154	3.732E-08
SPNS1	1.23187207	5.27625E-31	ERP29	0.34647799	3.802E-08
LY9	1.76705286	7.06403E-31	NDRG2	0.47351876	3.962E-08
TPMT	1.01386655	1.13951E-30	TIGAR	0.46502268	4.143E-08
TPM2	2.24454283	1.1797E-30	ZCCHC11	0.90201194	4.536E-08
ERC2	5.14696412	1.58691E-30	RPS6KA3	0.4307875	4.536E-08
CDKN2C	2.36795721	1.60174E-30	FAM159A	3.91873292	4.673E-08
CDS1	2.73275453	1.96701E-30	RP11-347P5.1	0.77537135	4.874E-08
TRPV2	1.16806667	2.08095E-30	NRARP	0.44131879	4.93E-08
THYN1	1.01074893	2.11266E-30	INSIG1	0.58374464	5.376E-08
MPEG1	1.01987783	2.31081E-30	C16orf91	0.59674957	5.55E-08
RAB11FIP1	1.22468459	3.18617E-30	UBE2L6	0.31679662	5.894E-08
LIMD2	1.14665631	3.77609E-30	FYTTD1	0.4270698	6.519E-08
CDV3	0.57424393	4.45738E-30	AAAS	0.83912453	6.519E-08
HLA-C	0.70570333	4.60608E-30	CHMP4A	0.29391376	6.581E-08
ZNF711	5.1701083	4.85067E-30	ADAM8	0.82765192	6.924E-08
TAP1	1.35846359	4.91085E-30	PP7080	1.29674713	7.719E-08
C1orf186	5.05322835	7.56922E-30	CD7	0.83941583	8.345E-08
FAR2	2.92159745	1.60552E-29	SEMA4D	0.59814357	8.636E-08
C1orf115	3.87753492	1.72316E-29	ARRB2	0.33556359	9.223E-08
EIF5B	0.60709564	2.21777E-29	STK17B	0.38041026	9.404E-08
HMOX1	1.42304029	2.65188E-29	A4GALT	2.09194792	1.061E-07
THRB	6.08173714	2.69663E-29	PLAGL1	0.76369367	1.123E-07
SLAMF7	1.5750461	3.1571E-29	MYL12B	0.29127401	1.16E-07
MCUR1	1.22927066	3.28122E-29	SYCP2L	1.10791095	1.17E-07
FGD6	2.34067411	3.60452E-29	NSF	0.68586099	1.17E-07
ZBED3	1.84331113	5.52613E-29	ADGRE3	1.1139467	1.17E-07
DHRS7	0.80482364	7.3208E-29	DOK5	2.54614123	1.177E-07
CFL1	0.46077855	8.10901E-29	IFITM2	0.54867239	1.203E-07
ENOX1	4.07997105	1.23108E-28	RUNX3	0.50371247	1.263E-07
SMCO4	1.00767424	1.25473E-28	GZMB	3.14508133	1.316E-07

ERRFI1	2.40021782	1.34766E-28	GPR162	1.1730231	1.412E-07
PARP3	2.09629677	1.65016E-28	DOK2	0.54575002	1.416E-07
SPINK2	4.10766617	2.05711E-28	IDI1	0.58308015	1.656E-07
MYLK	2.09618802	2.40002E-28	ATP6V1G1	0.33568207	1.659E-07
DCBLD1	2.04038304	2.66744E-28	NSUN7	1.16276532	1.89E-07
PCBP4	4.32889515	3.94609E-28	BCL7C	0.5231064	2.125E-07
LGALS2	1.1019979	4.00583E-28	COMMD5	0.57947723	2.167E-07
HYAL3	2.10135861	4.79394E-28	PON2	0.65086961	2.247E-07
FNIP2	0.98695929	5.07365E-28	RNF207	1.44941082	2.298E-07
CAMP	3.41389253	7.46395E-28	KRT8	0.99311588	2.32E-07
GRAP2	5.41093369	1.0118E-27	CXCR2	3.40525924	2.383E-07
TPD52	1.57304123	1.10839E-27	MB21D2	0.71825325	2.518E-07
KATNA1	1.37559445	1.12656E-27	AIM1	0.48817582	2.71E-07
LASP1	0.92367527	1.15144E-27	WAS	0.3205489	3.042E-07
PITPNC1	1.09688407	1.21044E-27	CEACAM4	0.82584583	3.052E-07
PIKFYVE	1.72793245	1.57672E-27	SH3YL1	0.9174317	3.29E-07
CCPG1	1.22527142	1.59038E-27	KLF13	0.5595874	3.358E-07
CARD11	2.66527189	1.65149E-27	TMEM156	0.78002454	3.809E-07
NAV1	1.29397382	1.669E-27	SMCO4	0.50526017	3.963E-07
REC8	1.39979846	2.0737E-27	NCOA4	0.31029893	4E-07
RP11-476B1.1	5.1486375	2.82987E-27	PIGR	1.75229877	4.191E-07
HTT	1.06688399	3.42527E-27	YWHAH	0.53178994	4.279E-07
PACS1	1.21070385	3.7785E-27	SPN	0.36819008	4.429E-07
SUB1	0.41764458	3.90724E-27	CD300A	0.71301179	4.516E-07
P3H2	2.94389177	5.19004E-27	RSBN1	0.53950546	4.682E-07
RAB8B	1.09732488	5.66876E-27	BORCS5	0.59471909	4.799E-07
ECE1	3.96473247	6.70746E-27	WSB2	0.65723149	5.195E-07
RPS8	0.3538091	7.32444E-27	BAK1	0.71809894	6.041E-07
INADL	4.22063703	1.1084E-26	HNRNPF	0.33333045	6.097E-07
HN1	0.98831166	1.2956E-26	POP5	0.39102864	6.303E-07
CAMLG	0.84457905	1.39782E-26	PHACTR2	0.51077713	6.982E-07
CAP1	0.43508668	1.54838E-26	MYO5C	1.83644625	7.06E-07
SPPL3	1.04695117	1.91996E-26	IL18R1	0.71074021	7.183E-07
ANXA4	0.8004216	2.04381E-26	GPT	3.35645142	7.265E-07
RPS21	0.55233472	2.75521E-26	MACF1	0.4662347	7.298E-07
SOD1	0.60382824	3.66739E-26	ZNF571	1.92258118	7.365E-07
TRERF1	1.46527532	3.98594E-26	RGS12	0.62668115	7.438E-07
CDKN2D	1.47987227	4.24297E-26	GSN	0.46166564	7.464E-07
NCOA7	1.47315693	5.91551E-26	IFI30	0.43476294	7.512E-07
APH1A	0.59554138	5.92799E-26	RP11-37B2.1	0.91347422	7.715E-07
TMEM136	2.75570671	6.70427E-26	PIK3CD	0.83411549	7.94E-07
ITPRIPL1	2.40181004	1.14859E-25	LMNB1	0.6067084	8.102E-07
GOLGA6L9	3.170322	1.52927E-25	TACC1	0.35597157	8.382E-07
ZNF366	2.46701395	2.13004E-25	RASSF5	0.51973241	8.423E-07
SAP18	0.45499915	2.38221E-25	ACTN4	0.31845181	8.54E-07
ANKRD20A3	2.67634606	2.47851E-25	RPL26	0.21317072	8.586E-07

FUCA1	2.41347415	2.88404E-25
CD226	1.94536986	3.99364E-25
KIAA0040	1.77333517	4.48949E-25
BRD2	0.83228157	4.74937E-25
KMT5A	1.10348151	5.35395E-25
ABRACL	0.7590841	6.40891E-25
STK32C	1.03328426	7.13092E-25
ZNF516	1.45396348	8.27419E-25
NAP1L1	0.70575479	1.00554E-24
COL4A4	4.27998164	1.03093E-24
CYFIP2	1.89387432	1.03517E-24
SNTB2	2.29583537	1.05875E-24
MSL3	1.12853657	1.39527E-24
FZD3	2.12785782	1.43138E-24
NDUFA9	0.6682304	1.56752E-24
SRPK1	1.1233055	1.82929E-24
ACTN1	1.07539431	1.86164E-24
CKS2	2.04219417	2.02086E-24
AC093673.5	1.74909014	2.22245E-24
SMARCA5	0.71235955	2.37349E-24
ITGB1	0.46694565	2.85176E-24
CTNNAL1	1.94407727	2.93862E-24
AP1G2	1.22701099	3.12119E-24
FAM46C	1.78120629	3.19163E-24
AUTS2	1.9757384	3.43999E-24
ABL1	1.5462585	3.85756E-24
MYO7B	4.26274156	4.03312E-24
ARPC2	0.4698379	4.04865E-24
IRF8	1.38473627	4.04865E-24
SNRNP25	0.80362257	4.24797E-24
HLA-DPB1	0.47253063	4.43069E-24
GNG7	1.18955275	5.35925E-24
FAM65A	1.25657775	6.64929E-24
MYCL	1.38312972	7.70979E-24
GAMT	1.75631165	8.46309E-24
LINC00152	1.31446708	8.59826E-24
DUSP4	1.57778722	9.06999E-24
SMO	3.73078317	1.0229E-23
ERN1	1.34846498	1.1328E-23
EHF	1.8447656	1.18825E-23
LDLRAD3	2.05622182	1.37933E-23
FLT3	1.09258621	1.37951E-23
ANPEP	1.68732114	1.48514E-23
NDUFS4	0.69409128	1.50427E-23
STX3	1.06835215	1.61399E-23
DENND1B	0.74341188	2.13029E-23

LY6E	0.39875678	8.626E-07
CST7	0.58164709	9.002E-07
N4BP2L2	0.28833469	9.074E-07
MTR	0.77056678	9.209E-07
CIRBP	0.27698741	9.318E-07
CCR6	0.60533062	9.462E-07
AOC1	4.44213891	9.648E-07
MIER2	0.81288554	1.03E-06
ST14	0.52702953	1.053E-06
CTD-2319I12.:	1.05039349	1.073E-06
NAA10	0.32749518	1.146E-06
LITAF	0.5265862	1.146E-06
DDX21	0.26188285	1.168E-06
ARPC5	0.25644524	1.194E-06
DOCK5	0.38251094	1.279E-06
FRMD4B	0.40486041	1.28E-06
RNF222	2.21469095	1.289E-06
SKIL	0.38140422	1.314E-06
ESYT1	0.64478791	1.322E-06
GPR183	0.24971059	1.346E-06
ASCC2	0.60015761	1.352E-06
SPINT1	0.59757286	1.392E-06
ABI3	0.39546841	1.405E-06
HGSNAT	0.33077457	1.439E-06
RPS27A	0.18563046	1.496E-06
KBTBD8	0.57892009	1.51E-06
RPL38	0.2143619	1.719E-06
LEPROTL1	0.38230573	1.73E-06
ZNF592	0.68012119	1.905E-06
OTULIN	0.59599267	1.972E-06
MED13L	0.35785064	1.997E-06
IGSF6	0.47030224	2.064E-06
IL13RA1	0.26098901	2.104E-06
AC104653.1	1.59415992	2.122E-06
GCA	0.46498068	2.167E-06
PPA2	0.30638719	2.17E-06
CSF2RA	0.32153809	2.402E-06
SGTA	0.47701984	2.405E-06
STK4	0.50384755	2.444E-06
ACTR2	0.22048277	2.445E-06
CHST11	0.57732223	2.464E-06
CCDC146	0.86584928	2.484E-06
RPL34	0.19609309	2.522E-06
ARHGAP5	0.54850179	2.549E-06
CTDP1	0.7699793	2.549E-06
C15orf39	0.6475751	2.56E-06

PRDX1	0.92775255	2.16036E-23	PENK	3.37613339	2.614E-06
SERPINB6	0.85428545	2.71613E-23	SEMA4C	0.9309227	2.647E-06
LRRC1	3.52256552	3.039E-23	CLECL1	0.93694214	2.684E-06
MTERF2	2.4008891	4.08916E-23	NR3C1	0.3676485	2.704E-06
CCDC122	2.13359539	4.91867E-23	LPIN1	0.61733101	2.841E-06
SLC1A5	1.10931996	5.77313E-23	CD180	0.81897041	2.894E-06
STK17A	1.23876306	6.79841E-23	RALA	0.83981663	2.952E-06
SMIM14	0.8026401	7.80099E-23	CA2	1.13197406	3.066E-06
ADAM19	1.72778783	1.20559E-22	NPFFR1	1.29026657	3.138E-06
EPCAM	2.86312943	1.34763E-22	DIRAS1	2.07718501	3.384E-06
RP11-527L4.2	5.3206152	1.76342E-22	TMEM163	1.87164678	3.418E-06
ATP5E	0.47506947	2.02397E-22	CEP83	0.54216107	3.444E-06
CGREF1	5.17585138	2.12518E-22	CHD1	0.36614153	3.483E-06
OAZ2	0.98178133	2.21128E-22	LRRC20	1.50243014	3.66E-06
HLA-F	0.86845574	2.41846E-22	TAOK3	0.26774719	3.739E-06
NT5C3B	1.41494738	3.07028E-22	C19orf24	0.29359893	3.786E-06
HIST1H2AE	2.26473016	4.29809E-22	ARHGAP9	0.55086971	3.872E-06
CALM3	0.7727882	4.83084E-22	COA1	0.51865217	4.029E-06
R3HDM4	1.04617113	5.10703E-22	ZBTB18	0.88137145	4.029E-06
RASAL2	1.85006367	5.36484E-22	SEC22C	0.5396094	4.238E-06
ROMO1	0.67522059	6.92242E-22	RP6-91H8.3	0.77832471	4.305E-06
NBEAL2	1.56669922	7.55155E-22	MRPL55	0.34554372	4.342E-06
ID2	2.23321441	7.57828E-22	ZC3H15	0.24523214	4.385E-06
VARS	0.94044293	8.80172E-22	PPCDC	0.6042413	4.419E-06
ZNF296	2.01291827	8.89925E-22	RP11-693J15.!	2.12428356	4.483E-06
TRAK1	1.04018276	9.25499E-22	CTA-384D8.34	0.59543778	4.599E-06
DAAM1	1.54904913	1.06887E-21	CDC42SE2	0.29210353	4.798E-06
WBSCR22	0.9043438	1.11597E-21	PI4KA	0.56312488	4.879E-06
SLCO5A1	3.43283921	1.11664E-21	ZFAND6	0.37683928	4.887E-06
OSBPL3	1.51901019	1.13978E-21	CHRNE	0.58798984	4.89E-06
CHD7	1.4792076	1.18579E-21	ARHGEF7	0.64963826	5.017E-06
PXN	1.86057122	1.33255E-21	PPHLN1	0.33074767	5.032E-06
NEDD4L	2.19030375	1.46482E-21	FAM134B	1.41861355	5.169E-06
AMD1	0.72807975	1.72551E-21	TRADD	0.53451093	5.243E-06
QPCT	1.11974388	2.20274E-21	EVA1B	0.37488973	5.87E-06
RPL31	0.4251926	2.28385E-21	NDUFS8	0.26813252	5.968E-06
TOR3A	0.90636956	3.57127E-21	NPM1	0.22504181	6.023E-06
ACSS2	1.23948296	3.6466E-21	STK11IP	0.90091868	6.134E-06
RPS27L	0.66439599	3.80655E-21	NAPA	0.38262235	6.289E-06
FAH	1.23305434	3.8265E-21	GNL3	0.3623077	6.337E-06
PDE6D	1.18963391	4.37571E-21	OPN3	0.49735411	6.567E-06
RAB11FIP4	1.2867205	4.42128E-21	LRR1	0.64922013	6.577E-06
BCL2L14	2.36376395	4.55279E-21	HSH2D	0.57552892	6.926E-06
LRMP	0.89541155	5.16397E-21	SLC1A4	0.48233292	7.085E-06
HSPB11	0.76578466	5.20336E-21	CEACAM3	1.26165465	7.18E-06
HSDL2	1.05181184	6.67689E-21	TMEM170A	0.34736887	7.283E-06

HSD17B14	2.90022716	6.69713E-21	ATXN7	0.48399208	7.426E-06
REEP3	0.82593822	7.00123E-21	RNF11	0.46002848	7.46E-06
SEPHS2	2.56293268	7.72177E-21	TCF25	0.2716578	7.517E-06
MPC2	0.67811961	7.85882E-21	EIF4E2	0.32139999	7.556E-06
FAM95B1	2.45391021	8.81908E-21	PRKCH	0.75462186	7.587E-06
GMPR	3.00130741	9.4854E-21	RUFY4	1.43281179	7.616E-06
GDPD5	2.29601861	9.89312E-21	ENPP2	0.7081033	8.127E-06
ZNF462	6.43188116	1.00632E-20	C11orf1	0.47701304	8.132E-06
SELPLG	1.66452295	1.22435E-20	RP11-430B1.2	2.14832351	8.357E-06
RFPL4A	6.27807386	1.22435E-20	EIF2AK4	0.39997138	8.6E-06
AL049794.1	3.40363955	1.27474E-20	STX6	0.41411036	9.865E-06
ACTR3	0.51507147	1.33812E-20	RNASE6	0.30908896	9.865E-06
HTR3A	4.39060732	1.59208E-20	LA16c-444G7.	1.66239462	9.865E-06
RP11-182L21.	1.50319156	1.66016E-20	IFITM3	0.60511214	1.012E-05
ODC1	1.07524304	2.33433E-20	TERF1	0.49761024	1.196E-05
CENPV	1.76124797	2.72087E-20	TRPM4	0.84281063	1.227E-05
LRRK2	1.2752574	2.88237E-20	UCHL3	0.36399439	1.302E-05
SLC2A1	1.680245	2.94465E-20	MAP3K8	0.40645048	1.35E-05
TXN	1.2947909	3.04839E-20	P2RY8	0.6168768	1.361E-05
FAM174B	3.19726059	3.24343E-20	FBXO6	0.53547215	1.456E-05
DSE	0.91414212	3.50035E-20	RP11-597D13	2.31451482	1.532E-05
SIGLEC10	0.75451415	5.34716E-20	TP53I13	0.40184969	1.548E-05
ASNS	1.89832466	6.36403E-20	CCDC125	0.64213133	1.612E-05
ANXA6	0.98085764	7.37922E-20	ENTPD7	0.79971782	1.615E-05
RGS3	3.89985413	7.44995E-20	NAA25	0.53831967	1.678E-05
MTMR10	1.20103593	7.63135E-20	CNOT10	0.59747468	1.722E-05
LCP1	0.43129874	9.25146E-20	C7orf50	0.4112399	1.786E-05
DUSP10	1.53076883	9.40277E-20	RGS2	0.35590392	1.794E-05
FARP2	1.02431038	9.40277E-20	GYPE	2.93753806	1.794E-05
CTNNBIP1	0.83335871	9.79809E-20	ALDOC	1.13876703	1.794E-05
REEP5	0.51307264	9.86972E-20	TRBVB	2.03103493	1.812E-05
TECR	0.73336612	1.05726E-19	SPTY2D1	0.38135924	1.893E-05
CDK2AP1	0.95244377	1.0627E-19	CLCN3	0.46270499	1.969E-05
AMFR	0.99442832	1.31985E-19	AGPAT5	0.71192237	1.994E-05
LDLRAD4	1.36372347	1.34656E-19	DNAJC5B	1.51780924	2.077E-05
MYO1C	1.45984408	1.4333E-19	SVIP	0.53721809	2.185E-05
SH3D21	1.25718989	1.45662E-19	TNNI2	0.56864832	2.2E-05
TRNP1	2.20597275	1.62167E-19	CYSLTR1	0.69610722	2.219E-05
WDR1	0.5831401	1.62585E-19	OBSL1	1.72219663	2.225E-05
PSMB8	0.54165029	1.68685E-19	PLXNC1	0.5921415	2.227E-05
TMEM238	1.48232705	1.77787E-19	ZCCHC2	0.57502168	2.231E-05
MYZAP	3.69818352	1.95267E-19	MAT2A	0.45381871	2.253E-05
C3orf58	1.42091149	2.6652E-19	GALNT10	0.93136404	2.253E-05
JAKMIP2	4.84752444	2.70177E-19	GPR82	0.7677204	2.268E-05
OXNAD1	2.47566669	2.89019E-19	ZNF200	0.84823401	2.292E-05
SRP19	0.70647225	3.25375E-19	DENND1B	0.41528624	2.339E-05

IFNL1	2.51886063	3.42557E-19	FOXN3	0.26804286	2.339E-05
PDPK1	0.81327529	3.61339E-19	RP1-34B20.21	0.8620644	2.365E-05
NAGS	2.88186466	3.85731E-19	AGRN	1.05938319	2.387E-05
INPP5F	1.35601569	4.81179E-19	GPR137B	0.48098725	2.451E-05
PFKFB3	0.72600297	5.03676E-19	NIN	0.38613567	2.538E-05
UNC119	0.75784714	5.16499E-19	PPM1G	0.30002796	2.557E-05
RAB38	2.53199106	6.1648E-19	TOMM34	0.49905306	2.561E-05
C10orf105	3.37197767	6.79741E-19	CELF2	0.28718519	2.671E-05
GDF7	6.36273665	6.9799E-19	TBC1D22A	0.33600271	2.777E-05
SEPT6	0.71088252	8.30854E-19	P2RY1	1.23149592	2.962E-05
PLCG2	1.33632926	9.73944E-19	TBC1D8	0.73750871	3.019E-05
RPS23	0.36068949	1.00778E-18	CTBP2	0.6023005	3.041E-05
PSENEN	0.63549988	1.20623E-18	LPIN2	0.3793883	3.273E-05
PSTPIP1	1.0638961	2.022E-18	RAB11FIP4	0.632166	3.294E-05
CAND1	0.79775103	2.10618E-18	PTP4A2	0.22371562	3.307E-05
ANKRD39	1.07545037	2.1327E-18	TRAPPC6A	0.26593504	3.341E-05
RAB10	0.48672675	2.2392E-18	SMOX	0.80510875	3.477E-05
CCNB1IP1	0.80678409	2.3604E-18	IGLON5	1.40790551	3.501E-05
DUSP18	1.4355265	2.62231E-18	LINC01480	1.07627134	3.584E-05
INPP5D	0.76834529	2.64796E-18	MYO1E	0.79467533	3.595E-05
PRELID2	2.55387153	4.10415E-18	ATHL1	0.85961235	3.679E-05
ISYNA1	1.60516531	4.4272E-18	BRD4	0.28168551	3.748E-05
WDR44	1.27304722	5.02928E-18	OGFRL1	0.40777786	3.866E-05
IDH3A	0.670276	5.03622E-18	CTSH	0.45565871	4.117E-05
ACE	2.98714723	5.0868E-18	EXOC3-AS1	0.89921273	4.356E-05
SLC25A20	1.39847594	5.36445E-18	PDE7A	0.53393872	4.43E-05
GPT2	2.82851304	5.62681E-18	CD1C	0.81649781	4.514E-05
RPL23A	0.34268781	5.62699E-18	TFEB	0.42203086	4.83E-05
CFAP97	1.09518164	5.84167E-18	ANAPC16	0.32259831	5.001E-05
PIK3CB	1.15211078	5.92673E-18	EEF1E1	0.63630372	5.036E-05
SHE	3.3909644	6.19159E-18	DDX6	0.272144	5.117E-05
PRDX2	0.7716195	6.46995E-18	ABHD6	0.81146289	5.148E-05
CHCHD6	1.35968318	6.59848E-18	TRPT1	0.50215405	5.288E-05
ANKDD1B	3.70204243	7.49628E-18	PTAFR	0.68086897	5.342E-05
VCP	0.50677131	7.56425E-18	GFOD1	0.73856268	5.688E-05
MPG	0.9739593	8.75524E-18	IFITM1	1.00954353	5.688E-05
STIM2	0.89983671	8.89914E-18	CHML	0.43799486	5.728E-05
ANKRD20A4	2.88106919	1.013E-17	FLT1	0.99716226	5.739E-05
TWF2	0.76713493	1.23964E-17	RGS19	0.41287575	5.739E-05
SLC25A33	0.78018004	1.28248E-17	MALAT1	0.19890296	5.739E-05
LBR	0.7820972	1.2835E-17	TMSB10	0.22223937	6.067E-05
IARS	0.88277169	1.43801E-17	ITGB2-AS1	0.45262845	6.067E-05
ARF6	0.68762921	1.50789E-17	MYL9	1.22708128	6.117E-05
PIGZ	2.04223976	1.63413E-17	NSMCE3	0.42268559	6.552E-05
RFTN1	0.96136122	1.7913E-17	RALGAPA2	0.99028092	6.574E-05
CLIC2	1.05787018	1.90097E-17	UTRN	0.33259864	6.671E-05

HSD17B12	0.83231721	1.94475E-17	NXT1	0.3954396	6.991E-05
SH3BP1	0.92406013	1.97901E-17	TCIRG1	0.36411879	7.082E-05
DESI1	0.79992685	1.97901E-17	PPM1F	0.45862103	7.082E-05
NMT1	0.6689562	2.07004E-17	ADRBK2	0.29799202	7.082E-05
RP3-414A15.1	3.63637573	2.09408E-17	KIAA1551	0.40997736	7.101E-05
VEGFB	0.89672252	2.37119E-17	ADGRG5	1.02738124	7.101E-05
LGALSL	2.06976217	2.53644E-17	COMMD8	0.42873582	7.136E-05
NPIPB6	2.78122421	2.53644E-17	CSNK2B	0.2293505	7.155E-05
GNAS	0.3921841	3.23607E-17	MTMR9	0.60920053	7.258E-05
RAMP1	2.05135327	3.3358E-17	C3	1.24337766	7.267E-05
TRAF4	1.59719777	3.46887E-17	RHOG	0.17789987	7.386E-05
PELO	1.32751196	3.56227E-17	DRAP1	0.25768152	7.387E-05
RNF8	1.29595241	4.96087E-17	GSTM3	0.77637922	7.455E-05
NSMCE1	0.68950718	4.98388E-17	LAGE3	0.36053199	7.468E-05
PPIB	0.4808509	5.11E-17	MMP12	2.02733568	7.672E-05
AIDA	0.95406446	5.27614E-17	MOB1A	0.33808293	7.752E-05
HIST1H3H	2.57295031	5.52647E-17	S100Z	0.70788473	7.773E-05
PAFAH1B3	0.77958784	5.55353E-17	B4GALT6	1.35446085	8.116E-05
MYL6B	1.05278847	5.73088E-17	UBE2W	0.44162516	8.265E-05
F11R	0.93558045	6.18152E-17	SMG1	0.33045617	8.382E-05
SP140	1.07323604	6.36841E-17	NKAP	0.45097424	8.669E-05
PPP1R12B	1.52308225	6.46648E-17	AVPI1	0.53117719	8.928E-05
CAMSAP1	1.61039012	7.07025E-17	IGHMBP2	0.88417859	9.158E-05
DCTN2	0.62258039	7.44029E-17	LINC00937	0.92456114	9.187E-05
LIPH	4.97696315	8.62794E-17	EBI3	1.10162153	9.244E-05
OST4	0.34070817	8.7778E-17	WDR91	0.55912556	9.247E-05
BCAT2	1.13475694	9.83436E-17	CTD-2336O2.1	0.47664258	9.281E-05
BEND5	1.91775101	1.05428E-16	C19orf66	0.50977222	9.802E-05
ITGB7	0.62578943	1.12105E-16	ZNF791	0.55704616	9.942E-05
DLG3	2.5335306	1.13653E-16	CCDC50	0.59017022	0.0001025
ZBTB46	1.19213264	1.15948E-16	HMGN1	0.23059847	0.0001025
THAP9-AS1	1.02283528	1.21756E-16	RP13-270P17.	0.85668539	0.000103
FKBP14	1.35203447	1.40211E-16	FAM124A	2.40665819	0.000104
DCP2	0.62816582	1.4525E-16	GMEB1	0.49677988	0.0001065
SLC25A17	1.43074737	1.50221E-16	RBBP7	0.32921299	0.0001065
RPL28	0.26317948	1.57242E-16	PRKCA	0.50944081	0.0001066
VMO1	1.42971258	1.68367E-16	SEPW1	0.32293373	0.000107
MIA3	0.62791342	1.85599E-16	ACAT2	0.66268028	0.000113
TAPBP	0.60566658	1.9994E-16	GAK	0.3738527	0.0001203
ICAM3	1.41449014	2.02291E-16	CD6	2.86711548	0.0001203
CBFA2T3	1.01388257	2.23121E-16	NIPSNAP3B	1.19814618	0.0001206
SBNO1	0.73952167	2.3666E-16	CCDC107	0.28043538	0.0001245
PTMS	1.10156014	2.41025E-16	FBXO2	2.88184719	0.0001252
CTTNBP2NL	1.07771951	2.57715E-16	OLFM2	2.66707594	0.0001264
RAB11A	0.80012707	3.11539E-16	CBWD3	0.6811871	0.0001289
PDE4DIP	0.95668605	3.42268E-16	FOXO3	0.31464895	0.0001308

RND1	1.81441383	3.55144E-16	BZRAP1	1.07339738	0.0001371
BPHL	1.39972909	4.16624E-16	CLIC3	1.37987004	0.0001371
DLG1	1.14407806	4.4422E-16	COPS3	0.26080743	0.0001373
TANC2	1.71194649	4.65707E-16	MTF2	0.44980585	0.0001404
SH3BP4	1.47752084	4.72938E-16	HAX1	0.27470026	0.0001415
SLC5A3	2.14956119	4.84384E-16	SACS	0.64180416	0.0001419
ITSN2	0.75205453	4.95101E-16	TRAF5	0.44826697	0.000144
RP11-160O5.1	1.63039279	5.70341E-16	ATP8B4	0.49867622	0.0001442
ENPP3	4.49467005	5.84907E-16	AMN	2.5530846	0.0001458
FAM129A	1.076595	6.11791E-16	MACC1	1.65979151	0.0001459
ALOX15	1.59606537	7.1418E-16	FAM196A	2.31245648	0.0001541
NCKAP5L	1.04620608	7.18775E-16	MYOF	0.48388901	0.0001553
ADSL	0.65231089	7.42969E-16	FBLN2	1.04806884	0.0001566
NDUFV2	0.46594919	7.49205E-16	RP11-532F12.	0.82824282	0.000157
ZNF165	1.39299572	7.75525E-16	FUZ	0.7624332	0.0001613
GMCL1	1.16861643	7.89596E-16	CDC37	0.3357638	0.000165
TULP4	1.03146616	8.01421E-16	INTS9	0.78887373	0.000167
MARCH3	1.99349932	8.38662E-16	TLN1	0.2429967	0.0001675
PLEKHG4B	6.4732563	9.11721E-16	IZUMO4	0.86094489	0.0001695
ARL4D	2.88469144	1.12868E-15	RPS3A	0.183274	0.0001728
MXD4	0.76607402	1.13995E-15	HPS1	0.34871015	0.0001817
RPL18A	0.31812663	1.20139E-15	PLEKHM1	0.52352052	0.0001823
NEDD8	0.32573535	1.31426E-15	GUSB	0.28309125	0.0001883
NEGR1	2.46824989	1.40881E-15	HMGCR	0.76677033	0.0001954
MRPS6	1.22916018	1.54742E-15	EPC1	0.23854631	0.0001962
SDK1	6.22828244	1.72143E-15	MYL12A	0.28229217	0.0001972
ANKRD20A1	3.64612202	1.8488E-15	LINC01215	1.44371389	0.0001975
SNRNP200	0.85994353	1.85769E-15	AUNIP	1.24205029	0.0001975
ZNF707	2.25067197	2.17178E-15	UFC1	0.25259688	0.0002024
FBXO30	1.94707703	2.59301E-15	NUP98	0.42693093	0.0002062
MAGED2	0.90187345	2.60723E-15	MYH9	0.30162	0.0002117
MPP3	2.79869326	2.82812E-15	GPR88	3.06436975	0.0002185
CLSTN2	6.11588938	2.82897E-15	CAPZA1	0.19402178	0.0002204
ANKRD65	1.54880414	2.90855E-15	CDIP1	0.57010552	0.0002219
SOX4	1.32205034	3.15446E-15	RP11-326K13.	1.44604109	0.0002266
ASPHD2	1.43904064	3.21028E-15	RPS6KA4	0.38951194	0.0002272
M6PR	0.3552488	3.2734E-15	CTB-83J4.1	1.89354095	0.0002282
HIST1H2AJ	2.50487057	3.32444E-15	C9orf142	0.28490363	0.0002312
USP46	1.32786038	3.44432E-15	RNMT	0.17940031	0.0002356
PSMD3	0.62486717	4.22985E-15	AMPD2	0.41935091	0.00024
AP003774.1	1.88013223	4.25251E-15	CNBD2	0.61468784	0.0002413
MRPS27	0.86861257	4.42566E-15	KRT18	0.90374377	0.0002414
ACER3	0.59710596	4.45934E-15	CD109	0.74221145	0.0002419
UQCRH	0.30777079	4.64359E-15	CLTB	0.32532805	0.0002422
EBPL	0.86244859	4.73988E-15	BZW1	0.23265922	0.000248
RPAP3	1.00662244	4.85783E-15	LINC01531	3.22847639	0.0002492

AR	3.64353229	5.0282E-15	PPIG	0.20500027	0.0002496
SCFD1	0.61510441	5.06643E-15	SLC26A11	0.80788533	0.0002566
KAT2B	1.14435167	5.21364E-15	HEXDC	0.51570168	0.0002566
SCARB1	1.48144951	5.41949E-15	PDCD6	0.20914932	0.0002606
MYO6	4.82364074	5.5392E-15	HGD	3.96772228	0.0002641
CACYBP	1.0405056	5.73188E-15	LINC01420	0.23496775	0.0002651
TRAPPC1	0.47758295	5.95937E-15	YPEL3	0.50295179	0.000266
PDLIM1	1.30563704	6.15924E-15	MXD3	0.91804287	0.0002697
CHD6	0.76512035	6.23346E-15	RNF168	0.44635707	0.0002732
ARPC5L	0.84790839	6.49382E-15	CCND3	0.31627875	0.0002762
P2RY14	0.75252357	6.6659E-15	PRC1	0.63850563	0.000277
QRSL1	1.16566087	7.38863E-15	EIF4G3	0.41084561	0.0002779
ZNF710	1.13728628	7.78309E-15	SUMO3	0.21178412	0.0002793
RP11-388P9.2	3.06827501	8.35805E-15	C12orf10	0.4272414	0.0002931
MVB12B	1.05099496	8.53663E-15	MX1	0.68177941	0.0002938
PARM1	1.46452559	8.95237E-15	CORO1A	0.34603418	0.0003119
PRDX5	0.36890338	9.54369E-15	RPS10	0.1857629	0.0003138
UBL7	0.64989688	9.71886E-15	SARAF	0.23395626	0.0003175
DAXX	1.08533267	1.19047E-14	LAMP5	0.76322039	0.000328
CCR6	1.34042242	1.29273E-14	IRF9	0.27908816	0.0003291
RAB29	1.55706145	1.31131E-14	SH2B2	0.61656421	0.0003351
C3orf14	1.37269368	1.37006E-14	CDYL	0.50922853	0.0003373
IGF2R	1.34539492	1.41029E-14	ZNF217	0.48228929	0.0003373
MAPK9	1.01628889	1.54364E-14	ANKRD11	0.34262537	0.0003439
STT3A	0.90036957	1.61042E-14	GCLC	0.467817	0.000347
STX12	0.71282045	1.65845E-14	PSD3	1.19365785	0.0003525
RHOC	0.70766746	1.66216E-14	TRAFD1	0.89100395	0.0003605
USP1	0.83218508	1.76914E-14	GPR18	0.70410424	0.0003716
CST3	0.84745841	1.80166E-14	RARRES3	0.34637231	0.0003747
CCDC69	0.87954408	1.80382E-14	INPP5B	0.61111304	0.0003836
NCKAP5	1.19987786	1.87064E-14	STRBP	0.64127031	0.0004017
NUDT17	2.72242892	1.92775E-14	ATG2A	0.3447508	0.0004018
LRBA	1.187798	2.31819E-14	NAP1L5	0.86660668	0.0004092
THBD	1.24012832	2.32909E-14	DBNL	0.20539534	0.0004265
THEM4	1.27420903	2.79318E-14	RPL3	0.14608437	0.0004331
SRSF7	0.91180632	3.08424E-14	RPL27	0.15636629	0.0004371
WDR41	0.69105001	3.1571E-14	PPM1B	0.36256156	0.0004402
ZNF662	3.43286359	3.8647E-14	AKT2	0.42335527	0.0004407
AP3M2	1.47487522	4.0424E-14	CARD16	0.35021523	0.000447
RPL13A	0.30690583	4.09874E-14	CCNT2	0.39345502	0.0004485
CENPBD1	1.85045508	4.26216E-14	SCD	0.8068195	0.0004492
SLC12A2	1.05942434	4.26707E-14	RPS9	0.15216523	0.0004568
CCDC26	1.5553413	4.80075E-14	POLB	0.69254936	0.0004669
PRKCZ	1.70670301	5.10046E-14	LYN	0.20839986	0.0004711
GOLGA8B	1.62273698	5.51914E-14	C12orf45	0.31491149	0.0004872
TMEM41A	1.14242534	5.76182E-14	ZNF652	0.37219417	0.0004898

NFE2L1	1.05955855	6.40267E-14	DRGX	1.46210761	0.0004946
CTSZ	0.43751841	6.55254E-14	CMTM8	0.89310333	0.0004969
GTF2E2	0.85811835	6.67031E-14	SEC23B	0.35520995	0.0004981
PLOD3	1.08500521	7.14372E-14	HOTAIRM1	0.44944906	0.0005044
PSEN2	1.27298659	7.79177E-14	RAB5C	0.20029742	0.0005068
GNL1	0.77380036	8.58384E-14	SAMD9L	0.70208444	0.0005204
SPCS1	0.31923921	9.83499E-14	RPS4X	0.17550679	0.0005262
PTPRE	0.57018009	1.07764E-13	TTF1	0.62145823	0.000533
RLTPR	3.37375242	1.07948E-13	ITPK1	0.33426877	0.000533
SLC22A23	1.53897543	1.12094E-13	WNT5A	0.81641168	0.0005341
MARCH9	1.77958781	1.14501E-13	LMBRD1	0.37658236	0.0005367
CLEC2B	0.61216943	1.19753E-13	CES2	0.51574801	0.0005449
TPM4	0.5558648	1.1999E-13	FXYD5	0.21146809	0.0005475
ETV6	0.97339474	1.40927E-13	SLC35E1	0.38323769	0.0005598
PTGES2	0.72140609	1.43791E-13	JADE1	0.59470351	0.0005633
LINC00882	5.95513198	1.47675E-13	NDUFAF7	0.61231007	0.0005636
PQLC2	1.51505514	1.67848E-13	MYCBP	0.40409215	0.0005666
SH3BGRL2	3.81883252	1.68916E-13	IPCEF1	0.76301491	0.0005704
MAP1LC3A	1.36922963	1.72E-13	CNOT2	0.22471906	0.0005803
ZNF500	2.03037239	1.97238E-13	BRWD1	0.32909364	0.000591
RPL22	0.27315628	1.98438E-13	GALC	0.6937081	0.0005915
SMIM20	0.57918823	2.09743E-13	DNAJC8	0.18546651	0.0005983
BACH1	0.83981264	2.3821E-13	FBXW11	0.41988891	0.0006068
ARID4A	0.6382227	2.51222E-13	CDK17	0.68084952	0.0006158
PLXNA1	1.50784846	2.81145E-13	SLC16A3	0.37972408	0.0006192
PLEKHO2	0.88374872	2.84217E-13	ETS2	0.35195802	0.0006253
PLEKHO1	0.64257248	3.00128E-13	HPRT1	0.30562858	0.0006266
GRAMD3	1.20909142	3.36302E-13	CAMKK1	0.70490101	0.0006266
PDIA3	0.37608265	3.37165E-13	RASSF3	0.31227558	0.0006283
ADD3	0.82784977	3.3873E-13	RP11-902B17.	2.44718037	0.0006376
SQSTM1	0.73148324	3.46474E-13	STXBP5	0.81143216	0.00064
KISS1R	2.51534133	3.77094E-13	IL18RAP	1.53301945	0.0006564
ZFP36L1	1.43243327	3.83293E-13	NEMF	0.27460902	0.0006588
FCRL6	2.54304386	3.9508E-13	RPA3	0.23527564	0.0006631
PRDX4	0.56348015	3.99274E-13	TNFRSF13C	1.23878718	0.0006654
TNNC2	3.4554458	4.02868E-13	OAZ1	0.18972988	0.0006804
HIST1H2BJ	2.46934635	4.30097E-13	DYNLT1	0.30592219	0.0006814
RAC3	3.31330397	4.56583E-13	LSP1	0.34216949	0.0006834
ST3GAL2	1.04620122	4.659E-13	NMRK1	0.57851689	0.0006899
HIP1R	2.82445687	4.70892E-13	MCM8	1.06807945	0.0006928
TPM3	0.3958653	4.73467E-13	CD52	0.80647219	0.0006982
GNAO1	2.67649575	5.1114E-13	ALDH4A1	1.04148297	0.0006995
GOLGA8A	1.21343068	5.57656E-13	ATOX1	0.26180194	0.0006995
OFD1	0.58246885	6.08004E-13	N4BP2L1	0.34472498	0.0006995
COX7C	0.39916166	6.57119E-13	USP34	0.26052071	0.0007032
PKP4	1.39365413	6.81473E-13	ZHX2	0.82956144	0.0007057

RPS18	0.25736446	6.86126E-13	CRNKL1	0.43818089	0.0007171
RPL35	0.26421341	7.12995E-13	FBXW7	0.39592871	0.000723
AC010226.4	1.61647123	7.8896E-13	DUSP23	0.37936774	0.0007244
CSNK1E	0.88357488	8.9195E-13	GAPT	0.62390119	0.0007244
TDRD10	4.41031409	9.98114E-13	ADPGK	0.22891486	0.0007244
AP1S3	0.93729014	1.06179E-12	PNISR	0.21283409	0.0007258
CDKL5	1.82596565	1.07251E-12	CBWD5	0.35193894	0.0007274
TMX1	0.47339603	1.09E-12	UBE2J1	0.3087423	0.0007334
SNX21	2.12614477	1.17798E-12	DHCR7	1.1022235	0.0007357
CYB561	1.28868306	1.23018E-12	CASP4	0.35384379	0.0007407
TMEM132E	6.27525013	1.2304E-12	HNRNPA1	0.22354124	0.0007575
KLRG1	1.92161158	1.3045E-12	SAMD9	0.6546487	0.0007597
SEMA7A	1.32877025	1.50407E-12	IFIT3	0.85497226	0.0007612
RPL14	0.29451389	1.52315E-12	STAC3	0.46736328	0.0007614
TUBB2A	1.44148968	1.64224E-12	FIS1	0.25535234	0.0007765
NET1	1.09233072	1.65092E-12	PRDX6	0.24708915	0.000787
TRA2A	0.49258321	1.76267E-12	HLA-DQA2	0.67267446	0.0007878
HIST1H2AC	1.16665219	1.81071E-12	DNAJC21	0.24259812	0.000805
STX18	0.76618821	1.84297E-12	TMEM184B	0.63980023	0.0008167
PGAP1	1.70997512	1.87997E-12	CNDP2	0.23013871	0.0008239
NIPA1	1.77405395	2.11748E-12	ARPP19	0.22622009	0.0008277
RPS15	0.25862087	2.37834E-12	RPL9	0.12840673	0.000829
PCGF5	0.68272035	2.38472E-12	NAP1L1	0.25737002	0.0008299
CYP2S1	0.93296057	2.43757E-12	YTHDF2	0.25446398	0.0008412
GNAI1	1.78395156	2.44064E-12	ARF6	0.36023818	0.0008512
GTSF1	1.34597566	2.45806E-12	RTN2	0.50960393	0.0008549
SMPD3	2.57008977	2.49192E-12	SYNGR2	0.30524571	0.0008642
ACP6	1.82782818	2.75942E-12	ZMYND15	0.40198743	0.0008726
FAM20B	1.02549207	2.90198E-12	MED28	0.23234256	0.0008794
RPSA	0.31941012	3.14136E-12	ZFP30	1.11070684	0.0008941
RASSF7	1.37146569	3.18104E-12	PXK	0.35296123	0.0009265
CTNNBL1	0.42682937	3.1907E-12	IL1R1	0.53040727	0.0009266
KLHL2	1.07965725	3.34479E-12	DHCR24	0.98606192	0.0009401
ATRX	0.34563768	3.63287E-12	RP13-516M14	0.70523356	0.0009456
EVL	0.53198237	3.69279E-12	ZNF503	0.35817234	0.0009511
SAT1	0.48117021	3.91641E-12	WDR55	0.58335577	0.0009624
FAM135A	1.39155072	3.93483E-12	TRIM21	0.92466846	0.0009745
FAM3B	2.08153412	4.28547E-12	MSMO1	0.86283881	0.0009762
MBD4	0.72886363	4.45267E-12	BCO2	1.06967736	0.0010133
ZSCAN16-AS1	0.68105995	4.6929E-12	SRRM1	0.14200339	0.0010172
OTUB1	0.58702746	4.6929E-12	AP2A1	0.37874032	0.0010181
LAT	1.43971106	4.71991E-12	GAS6	0.49316193	0.0010466
C1QBP	0.55609877	4.74786E-12	PIK3CG	0.53513444	0.0010662
RABGEF1	1.03959499	4.77283E-12	PARP14	0.28367916	0.0010802
DNAJB9	0.68018875	4.80904E-12	FCRLB	0.98593803	0.0010912
ATXN1	1.05143452	4.87194E-12	EZR	0.27515133	0.0011024

HOXA7	4.1594436	4.90258E-12	FGFR1OP	0.41979267	0.0011411
DEGS1	0.61442788	5.02653E-12	STK38L	0.3914674	0.0011525
RPS25	0.23668528	5.30462E-12	ARID5A	0.35430179	0.0011629
PDE6G	1.34856609	5.52685E-12	KDM5A	0.34937056	0.001169
MPST	0.61894732	5.6697E-12	ZNF672	0.37909742	0.0011769
ZDHHC17	0.93700987	5.85758E-12	KDM2A	0.273567	0.0012347
ZZEF1	0.91501855	5.97888E-12	RP11-382A20.	0.68663367	0.0012362
MAPK12	2.57309958	6.05511E-12	PHF20	0.26062261	0.0012382
RRAGC	0.72525907	6.15122E-12	FBXO34	0.26159604	0.0012513
ZDHHC18	1.19575504	7.05173E-12	RPIA	0.37379233	0.0012662
CNPPD1	0.67586417	7.15202E-12	CMC2	0.26268897	0.0012711
RPS28	0.32896691	7.3054E-12	RAC2	0.38002605	0.001289
PARVG	0.42997693	7.85644E-12	C12orf75	0.87396119	0.001338
DAPP1	0.91160295	8.35992E-12	IMPA2	0.47604714	0.001338
RALB	0.92000147	9.02453E-12	RPS14	0.1860054	0.001359
CFI	2.25612205	9.28342E-12	FAM46A	0.40219231	0.001359
LINC01374	2.38942789	9.96736E-12	RNF126	0.36299278	0.001359
PHLPP2	1.4835366	1.04954E-11	CTD-2538C1.2	0.95682328	0.001359
PLEKHB2	0.37168538	1.07027E-11	FCER1A	0.78426623	0.0013696
HDAC9	1.11182049	1.07027E-11	ETF1	0.27760504	0.0013696
PDP1	1.02811681	1.19708E-11	ITGAX	0.25159151	0.001379
SNX30	0.88859033	1.21289E-11	GTF2F1	0.36938223	0.0014008
LDOC1	3.63668034	1.23414E-11	PDE8B	1.56952631	0.0014059
COMTD1	1.12172685	1.33807E-11	CD300LB	0.50599097	0.001412
CCDC127	1.200815	1.34459E-11	NCCRP1	4.50322028	0.001412
GNB2	0.35569517	1.34459E-11	TIMM10	0.25284015	0.0014151
CRIP3	1.52905084	1.36192E-11	LNX1	1.56288178	0.0014221
CAMK2G	1.14419297	1.64988E-11	LHPP	0.43083644	0.001423
FARS2	0.97478998	1.88863E-11	ZNF250	0.66760242	0.0014379
HNRNPLL	0.86646477	1.96609E-11	MSL2	0.51994647	0.001442
RAB39B	1.15575928	2.00662E-11	HMGXB4	0.31682671	0.0014488
ZC3H12C	1.74214979	2.00662E-11	FOCAD	1.05512966	0.0014523
RAB5A	0.72225164	2.06567E-11	COL9A2	0.79714306	0.0014651
MIB2	1.25752914	2.0901E-11	CD3E	3.94248082	0.0014767
DDT	0.47281118	2.16414E-11	RAPGEF1	0.39820301	0.0014847
ADCY3	0.85911705	2.18513E-11	MYO1F	0.20491894	0.0014973
HSPA8	0.76589372	2.31469E-11	HMHA1	0.2487422	0.0015167
RAVER2	2.19277037	2.49923E-11	BEX2	0.81624221	0.0015203
LGMN	0.77292996	2.50225E-11	RP11-680A11.	0.94936279	0.0015517
RPL27A	0.32723077	2.52264E-11	NSMF	0.67837336	0.001553
TMEM243	0.59500721	2.58626E-11	SOCS4	0.39511554	0.001553
RP11-268J15.!	1.57240816	2.60092E-11	CUL3	0.30166596	0.0015572
MAP3K1	0.79873009	2.61892E-11	EIF2AK2	0.38754803	0.0015611
CUX1	0.48045896	2.62003E-11	USP44	2.77582116	0.0015678
BICD1	1.46094498	2.74317E-11	CD33	0.29486122	0.001616
MAP3K4	1.44758492	2.83997E-11	GUCY1B3	0.72135488	0.0016198

ROR1	3.46250132	2.84742E-11	TBC1D32	1.29426862	0.0016296
OMD	4.51100074	2.85092E-11	MTFMT	0.42225653	0.0016296
KDSR	0.63902279	2.91493E-11	MLEC	0.17313438	0.0016351
NBPF12	1.35681165	2.92999E-11	MASTL	0.50342286	0.0016497
ORAI2	0.74966098	3.04686E-11	BAHD1	0.80852316	0.0017105
SOGA1	1.01286133	3.09954E-11	PCNXL2	0.58949797	0.0017458
RPL23	0.23403303	3.29686E-11	CCDC189	0.90775047	0.0017508
GPR137B	0.78880204	3.40869E-11	STAT3	0.40189265	0.0017848
ERO1A	0.61951637	3.55279E-11	GAS6-AS1	0.70721971	0.0018146
SLC25A25-AS1	1.67866858	3.78911E-11	PIGG	0.58005441	0.0018498
ZNF827	3.91541917	3.89539E-11	ABCC4	0.5569915	0.0018652
PARP12	1.12913978	3.94639E-11	CCL19	3.00602929	0.0018809
CECR2	4.64488145	4.08655E-11	EPB41	0.4484604	0.0018998
B2M	0.38140811	4.15875E-11	ZNF667-AS1	0.77266259	0.0019086
CARM1	0.94299017	4.18367E-11	HEATR6	0.50321242	0.0019126
NSMAF	0.65859994	4.19702E-11	XPR1	0.46718435	0.0019773
PPP2R1B	1.35842021	4.19702E-11	MOB3B	0.42498005	0.001986
PHLDA3	2.66248571	4.33178E-11	NUMBL	1.02486586	0.001986
VPS26B	0.85736852	4.72061E-11	FBL	0.20702822	0.0020374
VKORC1L1	1.03610274	4.90899E-11	ALCAM	0.43650681	0.0020636
PDE1C	4.23783727	4.9124E-11	LAIR1	0.44304688	0.0020697
MTX2	0.81328237	4.92637E-11	TMUB1	0.34161083	0.0021297
TSHZ3	2.82990366	4.99223E-11	APEX2	0.53361013	0.0021386
C6orf48	0.42083459	5.0106E-11	C4orf33	0.410816	0.0021597
KIAA0101	1.0110669	5.0106E-11	TNFSF9	0.68176046	0.002163
CD48	0.53651114	5.06727E-11	LRRC8C	0.43973483	0.0021699
DYNC1LI2	0.71420971	5.08424E-11	ZBTB37	0.96407873	0.0021845
MYADM	0.66315674	5.14619E-11	SNRPD3	0.22702097	0.0021845
TIMM8B	0.40296998	5.18557E-11	PPCS	0.29437971	0.0022257
PPP1CC	0.46653576	5.19771E-11	RP3-460G2.2	0.68036444	0.00226
TRIM38	0.5495446	5.27439E-11	VCL	0.49989456	0.0022758
PLEKHM3	3.05430692	5.31558E-11	SSR1	0.22000259	0.002302
CCDC86	1.06149423	5.32667E-11	LINC00116	0.28686615	0.0023322
MBP	0.62504789	6.21189E-11	GPER1	0.48716365	0.0023392
ITPKB	1.46926396	6.68984E-11	N4BP2	0.24780527	0.0023742
MIIP	0.81669406	6.83401E-11	LRTM1	3.12361054	0.0023742
DDI2	0.86418295	6.93165E-11	ZCWPW1	0.88256993	0.0024669
WDR83OS	0.28240166	7.19415E-11	TFPT	0.33574618	0.0025145
MKI67	1.93996745	7.32805E-11	ACTR3	0.19798714	0.0025336
LSP1	0.53650032	7.83608E-11	NUB1	0.32015318	0.0025341
FNBP1L	1.92302295	7.95201E-11	KIF17	0.90376	0.0025514
C14orf2	0.40964156	8.09353E-11	USP47	0.31447729	0.0025861
TLE1	1.19783971	8.13763E-11	POLR3F	0.70110833	0.0025908
BTG2	0.7366924	8.17091E-11	SIGLEC6	2.86391439	0.0026012
VAV3	0.98231468	9.24033E-11	ZNF609	0.45260822	0.0026022
YBEY	0.85571659	9.83799E-11	ZNF44	0.48868538	0.0026117

LINC01588	1.24714077	9.94944E-11	INSM1	1.76651151	0.0026353
SLC9A7	1.00364415	1.0126E-10	APBB2	0.98126959	0.0026411
UQCC3	0.76458591	1.05437E-10	WDR59	0.4091154	0.0026817
MYL12A	0.28548446	1.08237E-10	SON	0.13627846	0.0027178
UBE2N	0.39795439	1.20432E-10	DDX54	0.37150684	0.0027179
CHN2	0.6149466	1.22935E-10	TUBA1A	0.33168704	0.0027283
SNX13	0.73743261	1.23317E-10	THADA	0.56216123	0.0027389
FAM149A	4.04025642	1.26207E-10	ZDHHC5	0.3897646	0.0027389
DHX36	0.47297054	1.36566E-10	RAB24	0.77098751	0.0027519
LDLRAD2	2.58587186	1.37399E-10	MMP1	1.33634039	0.0027624
CYYR1	3.55783572	1.42903E-10	ASF1A	0.45296457	0.0027667
OXCT1	0.81398905	1.50983E-10	SPNS3	0.54614964	0.002778
RP11-385D13.	1.43300243	1.51161E-10	TOR2A	0.35021651	0.0027784
PPP2R4	0.73552185	1.59327E-10	RIBC2	1.55388915	0.0028122
AP3D1	0.50121557	1.7011E-10	ZNF35	0.80613759	0.0028941
ACSS1	0.99744025	1.71293E-10	ZNF703	0.67768233	0.002912
PTP4A2	0.34739629	1.71614E-10	AVP	2.19841947	0.0029159
EIF3H	0.22791706	1.7242E-10	SNN	0.35305806	0.0029194
C2CD2	1.52677172	1.79479E-10	SEC61B	0.16018535	0.0029627
DNAJC15	0.3573032	1.87557E-10	ATPIF1	0.19759415	0.0030723
E2F5	2.49591428	1.90833E-10	LIG3	0.53569626	0.0030883
OSBP2	5.3690418	1.93771E-10	TP53INP1	0.84042461	0.0030904
NUP210	1.05123606	2.1166E-10	RAB3A	1.11770242	0.0031328
MCM5	0.68224406	2.14211E-10	ZBTB33	0.56473176	0.0031737
MYO1D	1.47834587	2.18355E-10	SPTAN1	0.29812808	0.0031872
KLF6	1.40526067	2.27073E-10	CERS5	0.52235394	0.0032186
CRYBG3	0.85509062	2.30209E-10	NRBP1	0.2892092	0.0032305
TPST2	0.78258771	2.32949E-10	ZNF720	0.57024082	0.0032305
PBXIP1	0.77597868	2.36504E-10	PPM1N	0.64737062	0.0032367
FZD5	1.68497646	2.39831E-10	BRCA2	0.40097196	0.003251
TP53TG1	0.77815297	2.91344E-10	PYDC1	3.07612347	0.0032522
TBX19	1.82207507	3.75385E-10	RP11-545E17.	0.5051361	0.0032632
DCXR	0.48590067	3.79009E-10	MSL1	0.33834546	0.0032934
C3orf67	3.28477775	3.7929E-10	P2RY13	0.3987452	0.0032941
GCC2	0.61160232	3.79907E-10	C7orf49	0.48512681	0.003308
SETBP1	1.34074706	3.82843E-10	ATP6V0D2	1.06687048	0.0033167
LINC01184	0.75646824	3.96607E-10	RNF41	0.3293798	0.003335
B3GNTL1	1.16248305	3.99318E-10	ELAVL1	0.23802096	0.0033561
BICD2	0.60906724	3.99461E-10	ARL14EP	0.28493647	0.0034076
ERBB2IP	0.60732127	4.04834E-10	NRIP1	0.37246058	0.0034936
ANO10	1.09836814	4.11269E-10	SETD2	0.2153698	0.0035183
DNAL1	1.36426758	4.3288E-10	ZDHHC12	0.31004174	0.003519
MESDC2	0.51162328	4.36077E-10	TBC1D1	0.2370239	0.003532
PDIA4	0.62300256	4.40918E-10	LAMTOR2	0.12365545	0.0035572
UGCG	1.03795843	4.78934E-10	FXYD1	1.27438132	0.0035987
CITED2	0.84447077	4.97979E-10	IQGAP2	0.35294563	0.0036104

EI24	0.65488756	5.41902E-10	NFATC2	0.33463908	0.0036712
KLF8	2.3272334	5.58099E-10	RNF138	0.29181973	0.0036983
TBL1XR1	0.47350079	5.66769E-10	ETV3	0.25452699	0.0037399
LAMA5	2.68402593	6.65757E-10	TREML1	4.82104403	0.0037525
CISD1	0.63495279	7.21312E-10	ZFYVE16	0.27410975	0.0037633
RPS11	0.30995636	7.36098E-10	MED10	0.22672287	0.0037677
LMF2	0.69595188	7.40136E-10	CBLB	0.60117676	0.0037743
SGK1	0.85176385	7.47666E-10	PPAN	0.4904166	0.0038127
LDHB	0.47952201	7.78464E-10	GRASP	0.26208194	0.0038233
EXOSC8	0.64952996	8.2194E-10	TXNRD1	0.326988	0.003829
ADGRL1	2.4646333	8.68685E-10	SCAND1	0.34203022	0.003835
AKR7A2	0.66267597	9.14236E-10	LETM2	1.06156414	0.0038547
MFN1	0.83758529	9.58232E-10	CCDC28B	0.60162126	0.0038728
PRCP	0.30205845	9.71159E-10	ALOX5	0.46321246	0.0039013
SPIDR	0.98100965	1.00073E-09	PIGS	0.38135797	0.0039013
CUEDC1	1.75152601	1.01053E-09	EXD3	0.69115856	0.0039084
AFTPH	0.63097242	1.02769E-09	RNF34	0.53645227	0.0039109
FAM160A2	1.17100114	1.07756E-09	PNPLA7	1.32839865	0.0039621
TBC1D10C	1.00060587	1.11652E-09	C6orf1	0.36545949	0.0039958
CKLF	0.84797854	1.12472E-09	NUTM2B-AS1	0.29749517	0.0040084
NCK2	1.17146797	1.24329E-09	B9D2	0.45605898	0.0040659
RPS16	0.24272191	1.32333E-09	BLOC1S5	0.70578306	0.0040733
CRACR2B	1.85699715	1.37783E-09	SERHL2	0.94327877	0.0041076
HIST1H1B	1.73319466	1.38495E-09	HINT1	0.22422085	0.0041112
YEATS2	1.2960726	1.43584E-09	EXTL3	0.58874061	0.0041453
NIP7	0.62206371	1.44838E-09	SCP2	0.19420719	0.0041912
SAMM50	0.50813438	1.46138E-09	TIGD3	0.94274358	0.0042388
RPS6	0.21036445	1.51028E-09	SATB1	0.53289387	0.0043062
MTUS2	2.73419051	1.51028E-09	RPS12	0.15965265	0.0043062
ZCCHC8	0.66519805	1.51786E-09	CTLA4	0.61324431	0.0043689
SCARF1	1.1441631	1.53656E-09	BRD3	0.41832655	0.0043906
ECI1	0.57693364	1.55382E-09	GPR146	0.68825712	0.0043966
H1F0	0.80617254	1.56715E-09	RP4-555D20.2	1.39236071	0.0044076
GPR160	0.71118676	1.58324E-09	RNF144A	1.58535843	0.0044327
EXOC5	0.64313458	1.62446E-09	C1orf174	0.37065008	0.0044814
LMTK2	2.15958292	1.71674E-09	MCOLN2	0.45875026	0.0044814
RSPH14	3.47753608	1.79009E-09	SLC2A3	0.27874115	0.0045441
TRIO	0.75185947	1.80128E-09	AKAP17A	0.28358875	0.0045478
RSRC2	0.52856268	1.80624E-09	PPIH	0.29103622	0.0045936
PLEKHJ1	0.73834891	1.82834E-09	ARHGAP25	0.42570611	0.0046121
KATNAL1	1.77472578	1.83062E-09	EEF1A1	0.12486886	0.0046831
POLA2	1.28515142	1.83388E-09	C9orf16	0.20423147	0.0046848
ADAP2	0.57421493	1.85484E-09	CYTH3	0.71895155	0.0047269
HMGA1	0.67630861	1.87634E-09	RASD1	0.99381129	0.0047321
NCKAP1L	0.44809927	1.89062E-09	CASP7	0.37434357	0.0048137
ESAM	3.14107609	1.92001E-09	LINC00467	0.47136245	0.0048631

DBNL	0.39148584	1.92113E-09	TRIM56	0.36686415	0.0048673
APOBR	0.72903976	1.96064E-09	TRIB1	0.35473215	0.0049007
CLSTN1	0.73535249	2.11674E-09	ADCK2	0.43372006	0.0049497
RPL36	0.27041626	2.28377E-09	ZNF350	0.3764851	0.0049674
C12orf57	0.58768004	2.29207E-09	CYSTM1	0.22716374	0.0049917
COX6A1	0.37377133	2.2966E-09	MS4A6E	1.56929582	0.0050038
DESI2	0.84363185	2.3802E-09	HOMER1	0.79618939	0.0050208
GSPT2	1.41431598	2.44592E-09	AC009950.2	1.68008443	0.0050384
RDH10	1.43751162	2.46088E-09	TTC14	0.30375131	0.0050384
HIST1H2BC	1.68215329	2.46277E-09	SPA17	1.08937908	0.0050384
COA6	0.67309393	2.54669E-09	COL18A1	0.74687685	0.0050955
REPS1	0.83900033	2.55131E-09	WHAMM	0.26918429	0.0051445
RHOD	4.25191851	2.55131E-09	RP11-620J15.:	0.72704498	0.0051671
RUNX1	1.10963932	2.58231E-09	RRP15	0.31687543	0.0051736
RP11-157E21.	2.50537275	2.60523E-09	NSFL1C	0.21159673	0.005217
H2AFJ	0.44509351	2.64895E-09	RPS29	0.18615256	0.0052307
ANO9	2.89410466	2.75483E-09	SPATA13	0.50870618	0.0052569
KLHL6	0.85428151	2.77206E-09	CD4	0.20631505	0.0052621
SNHG12	0.87465851	2.78997E-09	TPR	0.16945005	0.0052934
HLTF	0.85689706	2.93275E-09	ING3	0.29348229	0.0053268
TUBA4A	1.23845307	2.98488E-09	RPP25	0.6957626	0.0053306
HIST1H1C	1.11681332	3.10532E-09	CLCN5	0.61368592	0.0053624
RPL37A	0.29344999	3.40679E-09	PKIG	0.51699744	0.0053856
ZC3H12D	1.28943016	3.40679E-09	BACH2	1.52253185	0.0053882
VPS28	0.29021143	3.44232E-09	KHSRP	0.2880533	0.0054176
CHD9	0.62579358	3.56038E-09	ATP5S	0.29930111	0.0054512
AGPS	0.80204346	3.7336E-09	STK38	0.44404245	0.0054656
AC021188.4	2.23648635	3.76954E-09	NIPAL3	0.56221727	0.0054838
SLC25A3	0.34425921	4.08119E-09	LMBR1L	0.34836552	0.0055528
ST7-AS1	1.80128053	4.29851E-09	C11orf63	0.66512583	0.005555
HHEX	1.11289217	4.43236E-09	CEBPB	0.33071854	0.0055671
LACC1	1.02427412	4.64057E-09	RPS19BP1	0.20339979	0.0055841
HLA-DPA1	0.44486117	4.94339E-09	ADCY9	0.77652872	0.0055899
FBRS	0.67899108	4.94339E-09	PLAT	1.30740281	0.0056017
PELP1	0.77253926	5.07632E-09	RAB22A	0.25341435	0.0056266
NKTR	0.44170024	5.16627E-09	SMIM12	0.23585398	0.0056661
TNK1	4.99539877	5.17898E-09	SLC25A39	0.20608116	0.0056907
RNF4	0.64895846	5.34436E-09	DYX1C1	1.12252934	0.005727
RAB3IP	1.20148737	5.37304E-09	RP3-508I15.2:	0.65774526	0.005727
RAB33A	1.299234	5.38569E-09	MSN	0.22864816	0.0057296
AGGF1	0.7087779	5.81574E-09	SMARCC1	0.23506437	0.0057422
PRKD2	1.72635875	5.97395E-09	C5orf56	0.55394915	0.0058146
PANX2	2.71927465	6.05527E-09	ZFP91	0.3224742	0.0058164
RBM17	0.63504468	6.1211E-09	WDR66	1.11716404	0.0058465
BRF2	1.7656374	6.30308E-09	TTC19	0.25426413	0.0058846
DOPEY1	1.17794637	6.78176E-09	NET1	0.3760031	0.0059855

TSSC1	0.6212133	6.87263E-09	MPPED1	3.19770451	0.0060135
CNR2	4.69704321	6.99689E-09	DHX9	0.21276381	0.0062136
PIM2	0.75839666	7.03088E-09	NUS1	0.26672484	0.006272
AGFG2	1.89009715	7.59122E-09	CALCA	2.28967559	0.006365
BCL6	0.88848234	7.66647E-09	SERBP1	0.1780058	0.0064342
PDCD1	2.58212303	7.67914E-09	XPC	0.32402339	0.0064497
UBXN1	0.36220159	7.70321E-09	NPC1	0.61435469	0.0064609
AC011747.4	2.59502632	8.12326E-09	CLU	1.02934211	0.0064884
LINC00877	1.10540487	8.3258E-09	NKD1	1.3138595	0.0065251
TMEM237	1.53625189	8.62318E-09	MAP3K1	0.27794013	0.0066231
BST2	0.32249357	8.67809E-09	RPL35A	0.13764464	0.0066259
PAK3	5.07305314	8.81169E-09	CA5A	1.97567638	0.0066259
GRAMD2	5.33143773	9.04276E-09	CSNK1D	0.22298476	0.0066535
ELMSAN1	0.8091447	9.09353E-09	MFSD3	0.5267107	0.0066906
ZDHHC2	0.98801537	9.15671E-09	MGAM	1.98659038	0.0067331
AP4M1	1.04262923	9.33196E-09	EXOC6	0.34877966	0.0067426
ANKZF1	0.97407933	9.48601E-09	FOXP1	0.33471192	0.006778
MGST3	0.45897028	9.55519E-09	ZMIZ2	0.34782842	0.006817
AARD	3.83867637	9.59398E-09	TNFRSF18	1.02166914	0.0068462
RP11-803D5.4	2.1163018	9.87977E-09	ARPC3	0.16666185	0.0068824
TUFT1	1.77867085	1.02955E-08	RAB33A	0.64886743	0.0069159
CD40	1.35167638	1.03606E-08	SMARCA2	0.3603731	0.0069159
WHSC1L1	0.52067792	1.06714E-08	METRN	0.55365711	0.0069159
PSMA3	0.42156463	1.08433E-08	CHD1L	0.40196506	0.0069895
MFF	0.51169939	1.08611E-08	RASGRP3	0.46378933	0.0070212
PKN2	0.53223589	1.09404E-08	IDS	0.17353148	0.007082
NLRC5	1.08703294	1.19401E-08	ZNF24	0.25949439	0.007082
RPS29	0.28817581	1.2108E-08	DENND5B	0.62530066	0.007103
BOLA3	0.67468116	1.21731E-08	TMEM231	1.00807502	0.007103
RS1	5.16150031	1.2325E-08	TMEM154	0.38839719	0.0071163
S100B	2.25706007	1.25377E-08	MMAB	0.42742587	0.0071163
CASK	1.21041011	1.38028E-08	LINC00923	2.55241385	0.0071307
GOLGA6L10	2.81803082	1.3896E-08	PPP1R3B	0.59896077	0.0071688
AC240274.1	1.4104348	1.40799E-08	TPT1	0.1068955	0.0071701
SLC13A3	5.33072909	1.52107E-08	RP11-531A24.	1.59125462	0.0071886
CPNE1	0.41024562	1.54006E-08	C1RL	0.66610584	0.0071886
PRELID3B	0.43730461	1.57063E-08	ALYREF	0.20404446	0.0071886
COL9A3	1.99866064	1.61172E-08	MYB	0.85135673	0.0072697
PROC	2.59207442	1.66915E-08	CAPZB	0.14955438	0.0072831
PLEKHA2	0.64276642	1.85691E-08	RPS3	0.12055548	0.0072855
ATP5J2	0.33690139	1.90293E-08	RPS4Y1	0.39391397	0.0073102
PFN1	0.27055617	1.94328E-08	PPM1K	0.40574757	0.0073247
KCNK6	0.75162166	2.14831E-08	BIN3	0.39668874	0.0073892
AC013264.2	3.76933676	2.20407E-08	SQLE	0.36777589	0.0073981
TRAPPC2L	0.46962954	2.27385E-08	SIPA1L2	0.63465274	0.0074229
PHF6	1.09986069	2.29915E-08	TMA7	0.12778999	0.0074244

PPP6R1	0.57961943	2.30326E-08	ANKRD44	0.29449768	0.0074292
UBE2V2	0.40452664	2.30855E-08	GALNTL6	1.02052333	0.0074331
UBE2K	0.33886246	2.43143E-08	CLNS1A	0.17466343	0.0074506
MORC4	1.37400039	2.44946E-08	HMGN2	0.18885712	0.0074956
GRK6	0.67217552	2.45208E-08	CDCP1	0.54587207	0.0074957
TANC1	2.67878681	2.51996E-08	ATP6AP1	0.23104757	0.0075371
ZNF823	2.17999303	2.55543E-08	CCDC97	0.42595981	0.0075878
MLEC	0.44897778	2.5902E-08	WBP2	0.28951397	0.0076079
CLK1	0.82535882	2.66143E-08	ADAT1	0.42982708	0.0076277
TBC1D4	1.26858201	2.83688E-08	RP11-667K14.	1.14296726	0.0076546
IDH1	0.95383801	2.84642E-08	PSIP1	0.29783253	0.0077445
GNAZ	2.00378906	2.85862E-08	HNRNPH3	0.19201856	0.0077445
PRPSAP2	0.72566269	2.93484E-08	COX14	0.1444061	0.0079398
HCFC1R1	0.55945987	2.97832E-08	SSBP4	0.26087141	0.0079398
EVA1C	1.91303542	3.06841E-08	GRWD1	0.39229987	0.0079398
SLC41A1	2.81926513	3.1312E-08	WIPF1	0.18219338	0.0081036
RAD23B	0.4344375	3.1943E-08	ZNF680	0.88348399	0.0082733
TTLL7	2.67241905	3.35264E-08	RP11-46H11.1	1.56667402	0.0083402
REST	0.48545252	3.59626E-08	RBBP9	0.63678016	0.0083745
NRSN2	2.3132021	3.8064E-08	RELT	0.2919323	0.0084081
DHRS3	0.67196636	3.82984E-08	EYA3	0.52475374	0.0084248
PTK7	3.18908738	3.98078E-08	CYB5B	0.28708812	0.0084248
FAM98B	0.82029216	4.06963E-08	CEP95	0.42711138	0.0084387
NIPAL2	1.31144381	4.22286E-08	CH17-340M24	0.32566794	0.0085202
RND3	1.48646686	4.46096E-08	C2orf74	0.39660761	0.0085333
HIST2H2BF	1.63764003	4.53331E-08	SCHIP1	1.11424939	0.0086497
GAS2L3	0.74240853	4.53346E-08	SH2D2A	2.04737599	0.0086709
RPLP2	0.22376304	4.59633E-08	SIDT1	0.67587918	0.00868
APOL2	0.95521976	4.6155E-08	FOXA1	1.20896791	0.0086827
PRKRA	0.55738006	4.67096E-08	TAF5L	0.34140004	0.0087401
P2RY6	0.87541833	4.78233E-08	ARHGAP15	0.28940958	0.0087631
PUS10	1.26805741	4.83486E-08	PER1	0.2116091	0.0087755
LSM7	0.38971503	4.9029E-08	SIVA1	0.19596185	0.0087763
ERAP2	1.06824004	4.98149E-08	DNMT1	0.26462265	0.0088832
TMEM14B	0.33102193	5.07274E-08	RPS15A	0.13827234	0.0088886
TERF2	0.87772924	5.3579E-08	DEF6	0.27917732	0.0089005
ZBTB48	0.83911757	5.4359E-08	C9orf78	0.16851291	0.0090407
SUPT16H	0.38767586	5.63642E-08	GRIP1	0.55884959	0.0090915
UBL5	0.2813969	6.03401E-08	FIP1L1	0.28220628	0.0092051
STRBP	1.08429003	6.49458E-08	UQCR10	0.16843301	0.0092115
AUP1	0.35644634	6.65969E-08	RPS26	0.12083079	0.0092293
MYC	1.87927772	6.76683E-08	SVIL	0.51177014	0.0093044
SLC25A23	2.34846773	6.85231E-08	CD22	1.48650232	0.0093439
DNAJC4	0.46561454	6.97532E-08	CACUL1	0.28153793	0.0094122
NAA40	1.23610006	7.31165E-08	CEP78	0.66057577	0.0094263
CORO7	0.55004248	7.5876E-08	MYD88	0.2265282	0.0094656

TMEM97	0.88214288	7.6545E-08	MPV17L2	0.46799763	0.0095019
KIAA1671	3.90121784	7.65496E-08	HAAO	0.49310415	0.0095308
PSMD1	0.4573939	7.79261E-08	KCTD17	0.40599784	0.0095533
CSK	0.38574325	8.01958E-08	HIP1	0.41956364	0.0095541
TRIM32	3.05937199	8.17078E-08	OXCT1	0.37848452	0.009603
DSCAML1	5.0325847	8.31161E-08	ASGR2	0.41786969	0.0097554
INPP4A	0.74540612	8.93981E-08	DEGS2	0.82437173	0.0097902
ARPC4	0.31695308	9.03176E-08	SERTAD2	0.35192548	0.009829
RNASET2	0.32162054	9.14456E-08	CCR1	0.4535003	0.0098447
CSNK2A1	0.57243009	9.29317E-08	NDEL1	0.20277651	0.009889
PASK	2.09702566	9.42209E-08	WAPL	0.24942955	0.0098898
TSC22D4	0.58885521	9.45973E-08	ALG2	0.30912788	0.0099524
GSTK1	0.40795794	9.75561E-08	WDR7	0.50408433	0.0099681
EIF3D	0.34069098	9.79225E-08	GUCA2B	1.78037594	0.0100535
RPS17	0.21797042	1.00093E-07	FUT1	0.86049869	0.0100562
SEC24D	0.55646344	1.05292E-07	STAG2	0.16972299	0.0101319
TRAF5	0.52261017	1.11961E-07	SF1	0.18930679	0.0102006
RTKN	2.5723193	1.13148E-07	B3GNT6	2.22853802	0.0102296
LINC00582	4.06687395	1.16978E-07	CYTIP	0.19611448	0.0104077
SERTAD2	0.680746	1.20468E-07	TRIM44	0.20867279	0.0104077
ADGRG6	1.64766067	1.24234E-07	ANAPC15	0.24799169	0.0104396
C5orf45	1.68657158	1.2474E-07	CCR3	1.82707637	0.0105999
PSD	2.14428343	1.27653E-07	DNMT3A	0.6135642	0.0106924
RP1-313I6.12	1.22248398	1.29486E-07	SLA2	1.68337955	0.0107108
UCHL3	0.4360088	1.30883E-07	HOXA5	0.59538856	0.0108311
CLTB	0.39512357	1.30899E-07	CDK9	0.30234549	0.0108311
SNHG8	0.28750565	1.3344E-07	CTB-31O20.2	0.63536548	0.0108664
MARK3	0.75609794	1.33998E-07	LUC7L3	0.20271284	0.0108907
RCBTB1	1.30203801	1.34045E-07	ATF6B	0.24860912	0.0109131
MALAT1	0.24371834	1.3439E-07	ENHO	0.81868896	0.0109131
ABHD3	1.0085761	1.40155E-07	ZNF331	0.32408542	0.0109273
NFATC2	0.53091981	1.43731E-07	SC5D	0.38199864	0.0109543
CECR7	2.85847054	1.48138E-07	MAD2L2	0.26234666	0.0109673
PSME1	0.3488364	1.48962E-07	TMEM57	0.30686981	0.01098
CCT2	0.47395705	1.49264E-07	ACHE	1.58079263	0.01098
NCAPH	1.67057649	1.49357E-07	GOLGA8N	0.71713697	0.0109863
HSPBP1	0.74678697	1.49617E-07	SDCBP2	1.60484089	0.0109919
LMNA	0.74712615	1.51487E-07	TMEM204	0.88089002	0.0110046
MARCH1	0.49513003	1.51905E-07	DOCK1	0.66827911	0.0110066
MARVELD3	4.21738586	1.62958E-07	ADGRD1	1.40849414	0.0110165
CCNYL1	0.85995896	1.70967E-07	TRIM61	1.79588281	0.0110616
MRPL51	0.33743508	1.72836E-07	TCEAL5	1.36257542	0.0111251
CPNE2	1.33516801	1.78878E-07	RBM4	0.32207288	0.0111251
CARD8	0.58778913	1.8403E-07	ZNF667	1.46476143	0.0111517
IRAK2	1.38628711	1.8504E-07	RPSA	0.13932319	0.0111583
VASH1	0.75193156	1.87687E-07	MREG	1.39890048	0.0112702

VHL	0.58461624	1.88767E-07	POLD2	0.22436591	0.0113351
MTDH	0.25610588	1.88907E-07	CREBBP	0.3149829	0.011343
MRPL17	0.62640624	1.98491E-07	CHST2	0.44922991	0.0114254
ZFHX3	0.60075553	1.99656E-07	KLHL5	0.28852979	0.0114379
GGT7	1.44982433	1.99747E-07	FBXO11	0.29529515	0.0114415
PTCD2	1.57164765	2.0213E-07	TXLNA	0.34785723	0.01146
TMEM159	0.96272224	2.03603E-07	VPRBP	0.48602612	0.0115082
HEATR5A	0.90961748	2.07117E-07	PTGER4	0.27096186	0.011586
EIF4B	0.28512127	2.1933E-07	PWWP2B	0.48046108	0.0116701
PLK3	0.63038741	2.20127E-07	IL7R	1.14392542	0.0117527
ATP5A1	0.26641739	2.22486E-07	RIOK1	0.2750725	0.0117527
TIAM2	2.01531387	2.241E-07	NAA60	0.36338333	0.0118483
SEPT3	3.36216337	2.27549E-07	WDR54	0.37920734	0.0119304
TAGLN2	0.38621205	2.29248E-07	LCP1	0.15247399	0.0119304
HLA-DQB1	0.42914714	2.30497E-07	PARP4	0.37352603	0.0120477
AGPAT2	0.56286899	2.3285E-07	RUNX2	0.53773846	0.0120709
GALE	0.86087108	2.3492E-07	MOCOS	1.73086598	0.012076
TP53RK	0.81095063	2.38122E-07	APOC1	0.75594612	0.0121247
GPR89A	1.12262814	2.38225E-07	GPR160	0.30076807	0.0122698
LZTFL1	1.22508071	2.4671E-07	TDP1	0.4312518	0.0122888
MT-CYB	0.2712779	2.52311E-07	ATP2A3	0.62809798	0.0124327
RSBN1L	0.53552547	2.54546E-07	HPS3	0.32224477	0.0125146
SOX12	1.55468891	2.71103E-07	CTD-2186M1!	0.53701728	0.0125146
TRPS1	0.78141776	2.7612E-07	LOH12CR2	0.91417895	0.0125146
BTN2A2	0.62883642	2.78013E-07	LYRM9	0.53138902	0.0125664
MRPL40	0.46314377	2.79215E-07	FGF9	1.82533388	0.0126827
RPL10	0.18711946	2.80029E-07	IK	0.16824952	0.0127256
NMI	0.40987929	2.95558E-07	TMF1	0.22953157	0.0127398
SCNM1	0.37732989	3.03288E-07	FUNDC1	0.37861638	0.0127913
USP45	1.3424278	3.03528E-07	AQR	0.37329633	0.0128859
SNX25	1.67112669	3.13529E-07	NPIPB5	0.30852449	0.012889
RICTOR	0.63300348	3.21878E-07	ELK1	0.37307134	0.0129075
TKFC	0.71888569	3.29169E-07	ADAM28	0.2006004	0.013011
PELI1	0.86368276	3.29857E-07	EDARADD	1.70487336	0.0130316
PCNA	0.72491761	3.33383E-07	BCLAF1	0.16306544	0.0130488
COTL1	0.40665194	3.408E-07	UHMK1	0.21429612	0.0131121
FAM53A	3.27689497	3.42065E-07	RPA2	0.22299485	0.0133435
PRDM2	0.46354274	3.46996E-07	SMARCB1	0.30887857	0.0133448
SLC20A1	0.59503566	3.65226E-07	AKAP6	0.85342853	0.0133967
KLF3	0.4832011	3.6686E-07	PXDC1	0.280637	0.0135247
TUBG2	1.59940671	3.6686E-07	ZNF770	0.34794969	0.0135789
ANKDD1A	1.06398856	3.73637E-07	RP11-873E20.	1.82904625	0.0136971
MMP25	0.97960246	3.76091E-07	POMC	0.47485175	0.0137153
TBCA	0.23444233	3.80015E-07	ZCCHC7	0.29138071	0.0137153
FGFR1OP	0.74606524	3.96522E-07	DYNC2H1	1.10522814	0.013745
SMPDL3B	1.968438	3.97232E-07	DDX60L	0.48944911	0.0137948

NHP2	0.30100743	4.04265E-07	SMC5-AS1	1.06533944	0.0138515
UBN1	0.50996153	4.04265E-07	TSEN15	0.43755429	0.013856
TMEM241	1.92442781	4.07243E-07	DRAXIN	2.36581256	0.013915
RPS12	0.21958296	4.10208E-07	GPSM3	0.18983743	0.013915
SEL1L	0.49457909	4.17694E-07	CDK13	0.41977178	0.013915
IDNK	0.8386815	4.18772E-07	MCRS1	0.29891717	0.0139481
DCAKD	1.0963413	4.25116E-07	LPAR3	2.07532789	0.0139762
TRIM26	0.81587862	4.27759E-07	EIF4EBP2	0.21161913	0.014022
TUBB3	3.54438615	4.35755E-07	RP11-96H19.1	1.27840309	0.0140669
SEC62	0.26598913	4.38253E-07	MADD	0.48189711	0.0140786
RPL21	0.23601871	4.51498E-07	ZNF439	0.56675408	0.0141282
AKR1A1	0.3582576	4.57877E-07	SEPT6	0.24854914	0.0141693
CMB9-22P13.	0.96294473	4.58166E-07	MGAT2	0.2643285	0.0141779
TOB2	0.58661793	4.75135E-07	PPFIBP2	0.58768751	0.0142049
SSR2	0.22243772	4.75141E-07	AKAP13	0.30187296	0.0142452
SNRPD2	0.25510091	4.84141E-07	ATP1B1	0.22680056	0.0142779
TGDS	1.18727789	5.02545E-07	CD69	0.38112706	0.0143039
VEZT	0.53331953	5.13574E-07	DAGLB	0.48757796	0.0143378
ACYP1	1.09385549	5.23658E-07	AGBL3	1.63720585	0.0144853
DCAF7	0.41286812	5.3499E-07	MED4	0.23638849	0.0145275
RAB7A	0.23598739	5.38969E-07	SLC25A28	0.24499838	0.0145556
FUS	0.24638541	5.40198E-07	MRPS26	0.33901948	0.0146185
CLPX	0.611434	5.70611E-07	ZFPL1	0.4098534	0.014635
GPATCH2L	0.49576284	5.70843E-07	SGSM3	0.34931549	0.0146412
TSNARE1	1.66972896	5.75264E-07	RP1-244F24.1	0.80516659	0.0146928
NMB	0.98695324	5.83033E-07	ANXA3	3.42250537	0.0148483
LACE1	1.32822836	5.83579E-07	CMTM7	0.23519394	0.0148792
MARCH6	0.41417264	6.02339E-07	HEBP1	0.25354157	0.014888
PLEKHA3	0.58757088	6.13825E-07	BCL2L13	0.26419567	0.0149394
DCUN1D1	0.49649028	6.25885E-07	NCL	0.15625462	0.0150764
CDC42BPA	1.83191588	6.38769E-07	TMEM259	0.19871019	0.0151572
ATP5F1	0.25235134	6.55975E-07	CXCL16	0.24599333	0.0151974
KIF23	1.14523841	6.64674E-07	RNPS1	0.17845961	0.0153308
CHRNA5	3.0601297	6.8385E-07	ARMCX2	1.01305274	0.0154201
PKM	0.38675722	6.96884E-07	COTL1	0.25076443	0.0154507
THNSL1	2.51598301	7.01667E-07	KIAA0232	0.30725361	0.0155337
TCEB3	0.54868048	7.02139E-07	ZNF292	0.19401101	0.0156089
EDEM1	0.68126802	7.08548E-07	FAM126A	0.42464088	0.0157003
LPGAT1	0.62323571	7.31344E-07	MRPS33	0.18280324	0.0157389
DAPK1	0.44747634	7.31344E-07	ZNF571-AS1	1.95404874	0.0157389
SRGAP3	1.06119981	7.46183E-07	TMEM45A	1.65786346	0.0158028
C20orf24	0.42320752	7.63103E-07	AAMP	0.19828023	0.015907
MPRIP	0.67476602	7.69998E-07	LYSMD4	0.75098785	0.0159119
ACADVL	0.37048972	7.73127E-07	PYCR2	0.3254414	0.0159399
GSTM2	1.3082214	7.75803E-07	TAGAP	0.29402107	0.016003
RMND5A	0.61220936	7.77195E-07	RAB4B	0.21462602	0.0160697

RP11-1398P2.	0.94068108	7.8081E-07
SLCO3A1	0.72618652	7.84497E-07
UBC	0.36203398	7.92153E-07
RTN4	0.19970635	8.00204E-07
COX5A	0.26925668	8.49058E-07
ST8SIA4	0.67582069	8.49389E-07
FIBP	0.29631147	8.50743E-07
CLEC7A	0.4109116	8.86937E-07
ABHD14B	0.47742389	9.22439E-07
ARID1B	0.70818246	9.4609E-07
RNF5	0.36421218	9.62727E-07
MAP3K14	1.19705381	9.67737E-07
CD46	0.38135218	9.7143E-07
CD83	0.79367273	9.73754E-07
RMDN1	0.43529553	9.73754E-07
GTPBP8	0.52129142	9.89591E-07
AES	0.56981738	9.94003E-07
MMP24-AS1	0.46772439	9.9874E-07
DTX3	2.05816708	1.00002E-06
ACAP1	0.8226845	1.00002E-06
FBXO7	0.40291348	1.00472E-06
NRARP	0.51301331	1.01746E-06
RPS5	0.21978845	1.03173E-06
PACSIN2	0.42130624	1.07338E-06
CCDC64B	3.77261445	1.0775E-06
C7orf73	0.33289355	1.12991E-06
MED25	0.89102291	1.14386E-06
FHL2	2.23027626	1.18943E-06
GEMIN8	0.8807144	1.20997E-06
RP11-159H10	3.78140467	1.22101E-06
ZRSR2	0.68391117	1.2212E-06
CHPF2	0.79795571	1.25294E-06
CD200R1	1.75843977	1.25485E-06
CNIH1	0.4280336	1.25485E-06
FERMT3	0.38967415	1.28211E-06
C9orf135	4.58145435	1.3019E-06
MRPL22	0.50196352	1.31444E-06
PLP2	0.54264565	1.32247E-06
ACSL5	0.68220755	1.32247E-06
AP000439.1	4.35577076	1.34021E-06
RP11-395B7.4	1.88111775	1.35911E-06
HIST1H4H	1.42298577	1.3819E-06
RUFY3	0.47000334	1.38251E-06
TRIM47	2.4069071	1.38681E-06
DOCK7	0.93953671	1.39038E-06
FKBP8	0.38505898	1.40348E-06

DNAJB14	0.25015066	0.0161871
C8orf76	0.33744655	0.0161871
CD47	0.20205736	0.0162923
MRPS18C	0.31343622	0.0166475
SUOX	0.65280995	0.0166475
MYLPF	0.48550749	0.0168547
SSX2IP	0.52996187	0.0169515
GSC	1.73776106	0.0169612
NME3	0.1959733	0.016972
RELL2	0.56501785	0.0169783
FAM60A	0.17211454	0.0170112
IRF2BPL	0.59693928	0.0170491
RP11-147L13.	0.38759798	0.0170516
RP11-87C12.5	1.8079989	0.0171521
TMEM256	0.12989621	0.0171521
RP11-407N17	1.90939369	0.0171688
EID2B	0.65738775	0.0172064
MOB3A	0.27164645	0.017249
ALPK3	0.64077693	0.0172672
MAPK8IP1	1.29437352	0.0173428
SNX20	0.36000708	0.0174422
PFDN6	0.20414215	0.0175222
CEP250	0.60720477	0.0176047
RPL41	0.12696173	0.0176545
DHX58	0.53966186	0.0176675
MRTO4	0.24778485	0.0179009
CAPN12	0.65620741	0.0179363
FILIP1L	0.38117298	0.0180205
FAM221B	1.56807854	0.0180205
FAM26F	0.20031151	0.0180437
NONO	0.13831597	0.0180437
ZFYVE27	0.47967077	0.01805
RP11-489E7.4	0.39676578	0.0180576
NR1H2	0.27722539	0.018121
CTPS1	0.46424527	0.0181823
ATG16L2	0.24980392	0.0182344
SPNS2	1.6800918	0.0182344
ZNF230	0.66199269	0.0182344
RP11-834C11.	0.7622978	0.018237
ZNF529	0.53208676	0.0185022
CLSTN3	1.21017797	0.018608
HIPK1	0.25750175	0.0187209
RPS7	0.13379028	0.0187601
FRAT1	0.54439532	0.0187915
RPL37	0.1428741	0.0189506
ACTR3C	1.25633798	0.018986

ABI1	0.35443904	1.53667E-06	NKIRAS1	0.46176561	0.0190307
SLC25A25	1.15265201	1.55908E-06	RPL7A	0.11973895	0.0190321
KPNA4	0.37800254	1.56859E-06	ZNF626	0.51960892	0.0191673
PPP1R7	0.35766306	1.57505E-06	LIMS1	0.22579563	0.0191726
MBD2	0.36745874	1.59016E-06	LILRA1	0.52672169	0.0192405
TIMM17B	0.44352996	1.63315E-06	IKBIP	0.19015512	0.0192646
TERF2IP	0.30971727	1.66751E-06	NUDT3	0.17707543	0.0193131
TP53BP2	0.52895646	1.71745E-06	DHX8	0.35098715	0.0194498
CST7	0.72966307	1.71745E-06	SF3B5	0.13901169	0.019524
C2orf81	1.62636236	1.72632E-06	STIM2	0.20317723	0.0195443
DCPS	0.56495213	1.75981E-06	NUDT8	0.51104019	0.0195531
MICU1	0.5327525	1.7789E-06	DZIP3	0.55579229	0.0195961
VPS16	0.6447947	1.7789E-06	GPBP1	0.13747725	0.0195961
MND1	1.82499525	1.79337E-06	LGALS8	0.27344131	0.0197238
ZNF106	0.39129747	1.85227E-06	RNF130	0.17586325	0.0198247
HIST1H2BH	1.59816647	1.85299E-06	STT3B	0.23882706	0.0198756
ATF7IP	0.81593727	1.8911E-06	HNRNPD	0.18289847	0.0199074
DYRK2	0.86444688	1.90775E-06	GOLGA8R	1.43823073	0.0199164
BIRC3	0.65112265	1.98329E-06	CDC5L	0.19200527	0.020031
TMEM92	4.0177559	2.00277E-06	METRNL	0.23251186	0.0200874
DPM2	0.5608834	2.10241E-06	SSFA2	0.2548251	0.0203445
FLVCR1	0.77457863	2.16041E-06	ZNF16	0.79659129	0.0204483
RHOF	0.91295144	2.19357E-06	WDR46	0.26789337	0.0204899
SNRPN	0.56268267	2.2048E-06	RPL32	0.1132197	0.0205357
LMO4	0.57734174	2.22053E-06	LRRC14	0.70581265	0.0205357
CCT8	0.30342603	2.26997E-06	C12orf42	1.19469232	0.0206662
KIF2A	0.45929798	2.29236E-06	S1PR2	0.65580646	0.0206957
ALG14	0.84492537	2.29444E-06	FRMD8	0.30983094	0.0208907
PIF1	1.62966855	2.29962E-06	NPHP4	0.81402142	0.0210409
EBF4	2.04729813	2.35001E-06	CBWD2	0.29177106	0.0212138
NCAM1	2.62229134	2.36901E-06	ZNF830	0.26609514	0.0213692
LSM2	0.47963367	2.47636E-06	SESN1	0.56809293	0.0214983
STRA13	0.38936527	2.50346E-06	PIGW	0.60348117	0.0215405
MARC2	2.65841521	2.50478E-06	RCOR3	0.45972344	0.0215912
NABP1	0.59926125	2.51812E-06	GRIPAP1	0.21439931	0.0218232
CXCR3	0.83334401	2.53734E-06	RABIF	0.25687262	0.021978
KDM5B	0.87853687	2.60556E-06	PARVB	0.21910954	0.021978
MFSD14B	0.45285254	2.61103E-06	MARCKSL1	0.37089676	0.0219891
SNTA1	2.33433533	2.68132E-06	WDR27	0.71234119	0.0219891
FBXO31	1.34490908	2.73032E-06	ATG9B	1.33866075	0.0219891
PYCR1	1.29120664	2.82285E-06	ANTXR2	0.39910854	0.0220975
PHF23	1.04028271	2.87726E-06	RP11-49I11.1	1.21431525	0.0221303
PPP1CA	0.22900036	2.90486E-06	WDR37	0.42871496	0.0222993
CCDC88B	0.67185735	2.92602E-06	SF3A1	0.23535177	0.0223093
TVP23C	1.31193786	2.97131E-06	COQ7	0.35867557	0.0223689
ANK3	1.42053888	2.99695E-06	SYNGR3	1.80096971	0.0224697

ADCY7	0.5953178	2.99695E-06	SLC31A2	0.29814676	0.0225079
TMEM191B	0.89243316	3.21744E-06	URB2	0.74828073	0.0225559
TRIT1	0.63638298	3.25397E-06	CBARP	1.28578952	0.0226509
H2AFY	0.27023746	3.31899E-06	NOL8	0.29289276	0.0226775
DNAJC1	0.43541377	3.33344E-06	TMEM14B	0.1541502	0.0227859
CAMTA1	0.26570704	3.36717E-06	CASS4	0.35254978	0.0228126
FAM53C	0.42191426	3.38351E-06	DNM1L	0.17247762	0.0229006
C1orf50	0.62406475	3.4683E-06	PROSER3	0.64006232	0.0229714
UBQLN1	0.38756079	3.57112E-06	ADA	0.4991334	0.0229824
FKBP11	1.00833364	3.79798E-06	SPAG16	0.60550264	0.0230091
NDEL1	0.31763421	3.84696E-06	ITGB3BP	0.33489337	0.0230488
LRP10	0.50306411	3.84939E-06	AP4S1	0.54008325	0.0230488
RP11-326I11.3	1.26807612	3.90555E-06	RPP38	0.38177418	0.0232037
RPLP0	0.21646603	3.96554E-06	DTWD1	0.37404422	0.0232037
DHTKD1	0.71380805	4.01736E-06	RBX1	0.10371852	0.0232768
UBXN2A	0.64893732	4.05894E-06	ZNHIT3	0.2238512	0.0233085
CCDC12	0.43539178	4.12123E-06	INO80C	0.38846492	0.0233697
SNX1	0.4623435	4.21556E-06	SPECC1	0.27261494	0.0235035
LY86	0.33800283	4.22923E-06	SLC39A9	0.42152592	0.0236517
KDM5A	0.43615802	4.31069E-06	TM7SF2	0.59358009	0.023676
COMMD2	0.48995244	4.38012E-06	RPF1	0.30570703	0.023713
ERO1B	0.77620009	4.60681E-06	FOXC1	1.30007391	0.0239704
DUSP14	1.00346836	4.77915E-06	ATP5L	0.14838839	0.0239731
MAPK8	0.53803275	4.8253E-06	COQ10B	0.16685344	0.0241107
SIPA1L3	0.91052901	4.89906E-06	RASA2	0.39274648	0.0241107
NRBF2	0.66233022	4.90279E-06	SIAH3	1.86860622	0.0241107
LY75	0.8523438	5.11839E-06	RAD51C	0.3281417	0.024206
TMEM260	1.15833926	5.19815E-06	TOMM7	0.13311885	0.0242225
TXNL1	0.3024573	5.2502E-06	ATIC	0.2557834	0.0242787
VPS37D	3.94242623	5.25805E-06	KLF9	0.26907004	0.0242868
APBA1	1.4191315	5.31648E-06	ARHGAP5-AS1	0.50347952	0.0242868
COPZ1	0.29559096	5.62495E-06	RAB5B	0.29928886	0.0243878
ATP6V1A	0.4915888	5.67893E-06	RP11-467L13.	0.73615236	0.0246603
MTMR1	0.72755027	5.67893E-06	ALDH16A1	0.29455684	0.0249699
SAMD1	0.76756419	6.04014E-06	REV3L	0.31296209	0.02497
MKNK2	0.33868136	6.25331E-06	COPB1	0.20551603	0.0249895
NUTM2B-AS1	0.42188318	6.27033E-06	LSR	0.87730355	0.0251875
RASL11A	1.77358727	6.30978E-06	MAPK3	0.36509023	0.0252738
CLDN15	1.50886288	6.42049E-06	PTTG1	0.2636705	0.0253938
ARGLU1	0.24144988	6.54746E-06	TMEM230	0.16309202	0.0253951
CFDP1	0.35979851	6.55845E-06	ACTR3B	1.01435731	0.0254221
FBN1	2.89951351	6.69786E-06	NOL12	0.30776794	0.0256603
INF2	0.97037311	6.89064E-06	ZNF382	1.89520225	0.0256927
C16orf13	0.38285831	7.10826E-06	CEP135	0.34054748	0.0257899
AC009133.12	1.62923798	7.24425E-06	SLCO4A1	0.4057946	0.0258939
GXYLT1	0.72286231	7.25134E-06	FRYL	0.27758359	0.0259346

ECHDC1	0.39121653	7.32805E-06	MRPS12	0.18740779	0.0263428
OAT	0.43387898	7.40196E-06	AKIRIN1	0.23943081	0.0266728
EVI2A	0.38697572	7.43021E-06	RP11-191L17.	0.94304622	0.0267689
HIST1H2BF	1.93299241	7.46799E-06	DDX58	0.64297914	0.0267689
PPIA	0.18493204	7.56285E-06	CTD-2047H16	1.58694922	0.0268478
NDUFA7	0.44937523	7.56285E-06	HCFC1	0.27350353	0.0271978
KDM1B	0.77604827	7.78995E-06	MED19	0.34737334	0.0272172
DYNC1H1	0.40020946	8.12944E-06	FIGLA	1.25933935	0.0272629
PLK1	2.12250122	8.22727E-06	PPP5C	0.43994364	0.0272629
UBE2A	0.3285346	8.24547E-06	RP11-54O7.17	0.58428277	0.027263
RGS12	0.53702137	8.36038E-06	PPM1D	0.34903129	0.0273317
EPRS	0.31540194	8.42822E-06	PSMA5	0.16812313	0.0274225
NUCB1	0.32565713	8.45125E-06	TAL1	2.41030696	0.0275108
JMJD1C-AS1	2.57009762	8.61781E-06	SUPT5H	0.21741107	0.0275532
CEP152	0.69367814	8.66198E-06	RPL4	0.12523815	0.0276566
RP11-297C4.3	1.99350444	8.8166E-06	MLH1	0.35075416	0.0279051
NETO2	0.60988222	8.93123E-06	CABIN1	0.46456312	0.0280005
ATP2A3	0.85843873	9.29751E-06	ARPC4	0.13979515	0.0280774
HMOX2	0.54242978	9.31265E-06	CSNK1G2	0.35310145	0.0280774
COX4I1	0.24305168	9.50108E-06	ZDHHC23	0.73525586	0.0281234
RP11-1094M1	0.77680635	9.64018E-06	MAP3K2	0.13829611	0.0281952
TOPORS	0.33952386	9.65799E-06	RP11-296O14	0.99522063	0.0285484
SPI1	0.37328685	9.78014E-06	NTPCR	0.2793607	0.0285484
C5orf63	1.76585849	9.93028E-06	PHF3	0.20356182	0.0285583
SMCHD1	0.42000642	9.93387E-06	ECHS1	0.14266936	0.0285962
RNF169	0.75707068	1.00152E-05	LTV1	0.30295214	0.0287085
ABCA5	0.75432762	1.00459E-05	ZBTB6	0.68643575	0.0289686
RP11-841O20	0.64858705	1.0101E-05	STAM-AS1	1.24118926	0.0289686
MEA1	0.39902207	1.031E-05	SMG6	0.30538889	0.0290565
PACS2	0.63630752	1.03542E-05	RIPK2	0.33787465	0.0291995
LNPEP	0.40908978	1.04005E-05	SUFU	0.65441741	0.029209
RPS27	0.18871651	1.04282E-05	ATG101	0.22606278	0.0296559
NOP2	0.71621336	1.05138E-05	SP4	0.79766727	0.0296578
NPAS2	4.566999	1.05141E-05	KLHDC3	0.22537645	0.0298128
ARHGAP5	0.54374632	1.09354E-05	ABTB2	1.01669677	0.0299782
TMEM38B	0.64368799	1.09707E-05	TTC39A	2.09123448	0.0300619
ELOVL1	0.35860552	1.09968E-05	RP11-452L6.5	0.82454101	0.0301239
TTC37	0.48714936	1.10622E-05	PRPF4	0.27920857	0.0303848
DCTD	0.58525656	1.12682E-05	LRRC37A2	0.61542242	0.030438
BCAS4	2.11344534	1.12784E-05	SCML1	0.23341884	0.0305523
SMARCA1	2.99385784	1.15549E-05	GTDC1	0.46721779	0.0307127
EBLN3	0.46579722	1.17852E-05	TOE1	0.39819028	0.0308445
GFOD2	1.18134047	1.2103E-05	UBE2F	0.2694772	0.0308538
TOMM34	0.52042606	1.21143E-05	CHCHD5	0.16509785	0.0309353
CCDC167	0.47074599	1.21196E-05	RNF43	1.00166959	0.0310777
C18orf32	0.57393272	1.21506E-05	AKAP12	1.17997719	0.0311705

DDIT3	0.87368395	1.23139E-05	ATPAF2	0.23052928	0.0312129
MRPS21	0.25503757	1.24644E-05	ODF4	2.168232	0.0312358
RP11-17M16.:	1.50726356	1.26372E-05	BCCIP	0.21928963	0.0312875
NFAT5	0.81484106	1.29153E-05	LIMCH1	1.56645189	0.0315211
STMN1	0.65945232	1.31091E-05	HSPA13	0.34803639	0.0316158
GSTO2	1.54856462	1.36087E-05	C8orf59	0.17402897	0.0317224
ANKRD36C	0.58119951	1.3631E-05	MZF1-AS1	0.79172013	0.0318237
PRR7	0.72828779	1.37512E-05	RP11-458F8.4	1.10659455	0.0320192
CTA-390C10.9	2.79207803	1.41141E-05	BCL7B	0.28442994	0.0320192
SLC39A4	0.61996011	1.4253E-05	ITPR2	0.30455294	0.0321372
GTF2F2	0.46822117	1.44737E-05	SMC5	0.20988843	0.0321437
PPP1R35	0.66333191	1.45567E-05	USPL1	0.58426382	0.0322054
GPR27	2.82633966	1.4693E-05	CERS6	0.2458177	0.0324252
HCAR1	3.55902488	1.49246E-05	CD2BP2	0.23382585	0.0324495
DENND5B	0.9689515	1.49408E-05	RP11-386I14.4	0.42956195	0.0325094
CKAP2	0.81691136	1.51651E-05	C1GALT1	0.26988653	0.0327628
UBTF	0.45361152	1.53482E-05	TERF2IP	0.16728076	0.032814
RP4-737E23.2	2.22142179	1.59028E-05	SUPT4H1	0.15450151	0.0329173
EIF3F	0.21794203	1.62362E-05	DDX18	0.162664	0.0330396
FCHO1	0.87889188	1.62809E-05	INSIG2	0.33023844	0.0331087
EVC2	3.85665374	1.63058E-05	TRIM38	0.28300677	0.0332381
EPN2	1.88591141	1.63702E-05	SYTL1	0.55027549	0.0332835
FJX1	1.73334537	1.64134E-05	ENTPD1-AS1	0.5413103	0.0332835
FAM49B	0.21734926	1.72277E-05	C11orf58	0.12741397	0.0333302
RP11-511P7.5	1.57975696	1.72398E-05	SRRT	0.24208343	0.0333824
MRE11A	0.53833038	1.76123E-05	PSMC1	0.21750934	0.0333845
TLR6	1.17530736	1.76708E-05	FANCA	0.51114281	0.0334742
LINC00265	1.68658638	1.77843E-05	HOOK2	0.35738456	0.0335064
MCC	1.81038905	1.7837E-05	PHLPP1	0.37170631	0.033635
IDS	0.29295879	1.7891E-05	ZNF600	0.61019333	0.0337619
NALCN	4.60355911	1.80514E-05	PLGRKT	0.26530898	0.0338078
TM7SF2	0.99029708	1.83541E-05	GALNS	0.38481893	0.0340062
LCMT1	0.4506341	1.83705E-05	HYI	0.396236	0.0340229
CLEC14A	2.90060036	1.85851E-05	RELL1	0.41568823	0.0340229
RP9	0.77505571	1.86351E-05	ZNFX1	0.26841271	0.0341012
RP11-242D8.1	1.18632901	1.87752E-05	MAGOHB	0.25010229	0.0341128
BRK1	0.21425919	1.89608E-05	FAM131A	0.38189743	0.0341264
NEAT1	0.38407154	1.90869E-05	SAFB	0.20297839	0.0343019
FOS	0.96070364	1.91369E-05	HAUS5	0.62713351	0.0347288
EFHC1	0.76736227	1.92172E-05	C6orf62	0.14539778	0.0347327
SEMA4F	2.24603834	1.95017E-05	PARK7	0.11972973	0.0347456
CHAMP1	0.79155126	1.96351E-05	RIPK1	0.30667348	0.0348396
GLG1	0.50680082	2.00987E-05	OSGIN2	0.44468366	0.0349442
EIF4E3	0.99675957	2.0446E-05	HAS1	0.70022046	0.0349461
TM2D2	0.6788687	2.09808E-05	MARCH7	0.20530325	0.0349866
WDR47	0.82641418	2.10387E-05	HAVCR2	0.17773156	0.0349946

RP11-88H9.2	4.08309569	2.10464E-05	H2AFV	0.16033632	0.0350174
NUTF2	0.31024678	2.16695E-05	ZNF649	0.69423138	0.0351229
KLHL25	1.50475636	2.17734E-05	VSIG10	0.51967021	0.0353948
IWS1	0.3951031	2.20275E-05	CPD	0.49530272	0.0353948
RPL5	0.17774471	2.22181E-05	PGP	0.2150632	0.0354038
ZEB1-AS1	1.7185932	2.23602E-05	BRE	0.22758934	0.0356726
RUNDC1	0.93936129	2.25944E-05	MAML3	0.24478139	0.0357508
HIST1H2AG	1.56102482	2.28565E-05	ADIPOR2	0.26868228	0.0357767
ATR	0.6076734	2.31478E-05	FEN1	0.46504142	0.0360062
NDUFS3	0.33706705	2.41009E-05	ATP6V1E1	0.19674408	0.0360971
TWF1	0.60937276	2.41526E-05	DNAJC4	0.22903411	0.0362604
SLAIN1	2.15989075	2.43083E-05	CEPT1	0.36015462	0.0363664
TXN2	0.31301093	2.50537E-05	IGFBP6	1.0262037	0.0365647
LPIN3	3.40824172	2.57638E-05	MMP15	1.73724175	0.036747
NBPF11	1.01909997	2.58604E-05	VAMP2	0.21569496	0.0368527
CBX3	0.2600664	2.59389E-05	SMPD2	0.47884571	0.0369505
TMEM120B	1.03665814	2.60595E-05	ARAP2	0.55331939	0.0370492
CTB-113D17.1	1.10884145	2.6096E-05	EXOC3L1	0.92477082	0.0373671
CCDC106	0.79441448	2.6817E-05	SF3A3	0.19576697	0.0375859
CCDC85B	0.41115317	2.68386E-05	ZC3H13	0.18785506	0.0380127
FAAH	0.9740014	2.73505E-05	GTF2I	0.17492333	0.0380763
GPM6B	1.42083926	2.75888E-05	FAM122B	0.35015043	0.0380763
CTD-2020K17.	0.93002899	2.76291E-05	RP11-295P9.1	1.53956	0.0380763
CASP6	0.75275018	2.79304E-05	CERS4	0.59878877	0.0380896
SNRPE	0.2439936	2.86465E-05	CDC26	0.19111758	0.0381818
TRIM28	0.32006422	2.89642E-05	ANKRD36B	0.44359926	0.0385281
ARPC1B	0.31960453	2.91418E-05	CREB3L2	0.31613708	0.0386197
RASSF2	0.56381555	2.99313E-05	COX19	0.20627232	0.0388558
EMC8	0.4829505	3.0035E-05	FOXJ3	0.32276769	0.0389279
PLEKHH1	2.201256	3.06619E-05	TMEM158	0.49376828	0.0390036
MVP	0.39406618	3.1018E-05	MFSD12	0.2392785	0.0390086
SLC22A14	3.23837202	3.14048E-05	PFKFB2	0.93870089	0.0391358
NLGN2	2.6431559	3.14856E-05	CCDC59	0.16346856	0.0392031
DCAF13	0.41799645	3.14873E-05	SNAP47	0.45071507	0.0393901
PIK3CD-AS2	2.97380176	3.21164E-05	RASGRF1	0.6348987	0.0394639
TOMM20	0.21111267	3.25527E-05	LAPTM4B	0.79091865	0.0395839
HIST1H2BD	1.70420396	3.26231E-05	MON1B	0.376489	0.0397464
UHRF2	0.62261006	3.29587E-05	ALDH1B1	0.64609459	0.0400458
EIF1B	0.41696919	3.32217E-05	SUMO1	0.13694259	0.0400707
RP11-429J17.:	2.19196112	3.32217E-05	SHF	0.46269384	0.0402845
IPP	1.83230622	3.32996E-05	AC009005.2	0.50099549	0.0403167
ALDH7A1	1.24971222	3.4127E-05	SLC25A26	0.22207499	0.0406886
DUSP2	0.74473609	3.49325E-05	WDR64	3.34029654	0.0409938
KDM2A	0.35862303	3.50752E-05	RP4-781K5.6	1.62699431	0.0410835
TCF7	0.62128265	3.51769E-05	SKAP2	0.17508289	0.0411053
EVA1B	0.42732658	3.60187E-05	SP110	0.25820745	0.0412894

DOCK2	0.29837735	3.72221E-05
UBE2Z	0.3618517	3.7543E-05
CTC-548K16.1	3.68119775	3.78323E-05
MAP3K9	2.7979645	3.80169E-05
LRFN4	1.15890806	3.83414E-05
TMEM206	0.70761028	3.84837E-05
C16orf86	0.94098213	3.87569E-05
GNA13	0.44240718	3.9199E-05
HPS4	0.75619094	3.92917E-05
AP2B1	0.39265162	3.96635E-05
PRRG2	1.51329586	3.97437E-05
RASGRP3	0.61551796	3.97495E-05
PANK2	0.4765435	4.13124E-05
TMX3	0.46265352	4.13894E-05
C11orf80	1.31738277	4.15274E-05
MYRFL	4.36760446	4.21126E-05
NPAT	0.81302747	4.37632E-05
ISOC1	0.62381826	4.38339E-05
LCMT1-AS1	2.2450436	4.3989E-05
TMED9	0.30849215	4.5041E-05
C16orf87	0.651583	4.50941E-05
INIP	0.52321848	4.63568E-05
RP5-862P8.2	1.50853487	4.70309E-05
CAPN12	1.19874759	4.79901E-05
PRKRIP1	0.77227804	4.79907E-05
PRKAR2B	0.76047982	4.802E-05
OSER1	0.41161968	4.93007E-05
C19orf60	0.26573229	4.93172E-05
PPP1R13B	1.70237481	4.98952E-05
SBF1	0.84268205	4.99379E-05
ZNF768	0.85563488	5.01326E-05
NCBP3	0.42182592	5.02289E-05
ARMCX6	0.52085326	5.07841E-05
KTN1	0.27270603	5.09312E-05
SSRP1	0.44975098	5.10989E-05
CORO1B	0.33644351	5.1216E-05
CEP131	0.88600073	5.1256E-05
IDH2	0.38488058	5.13965E-05
PLBD2	0.47661016	5.15054E-05
RMND5B	0.99475159	5.21159E-05
BTN3A1	0.75561991	5.21338E-05
PHKB	0.4002005	5.23827E-05
EMC9	0.68633762	5.27012E-05
PPP3CC	0.6437327	5.27315E-05
BAD	0.51682047	5.29042E-05
MED13	0.4234352	5.37039E-05

AC020571.3	1.0494928	0.041665
TMEM51	0.56684135	0.0421354
MTHFR	0.29788177	0.0424159
MAPK1IP1L	0.14287099	0.042582
UBA6	0.23029451	0.0426
TARSL2	0.37471281	0.0426261
ST3GAL6	0.34881962	0.0428438
SBNO2	0.34323617	0.0429453
ARHGEF10	0.92473601	0.0429894
CLEC4C	0.86109094	0.0433105
SLC25A45	0.28520777	0.0434397
CCDC173	1.82963779	0.0436102
HMGN4	0.50443879	0.0436276
IQCJ-SCHIP1	0.94085701	0.0436314
CMTM6	0.16405817	0.0436997
RPL15	0.10139303	0.043989
METTL4	0.44460309	0.044005
PSMD13	0.135269	0.0440576
TAF11	0.23394278	0.0441847
FAM167A	1.33822351	0.0444001
KCTD9	0.4121757	0.0444206
PREX1	0.16791024	0.0444206
ELF4	0.42920432	0.0449265
ADAM12	2.03088677	0.0449265
MPZL3	0.57957408	0.0449686
CD1B	0.73335867	0.0449781
ADAMTS17	0.93852409	0.0449894
HSPBAP1	0.31822516	0.0450493
CASP9	0.25911757	0.0451549
UBE2I	0.10620249	0.0451549
TMEM248	0.16829837	0.0451597
RUBCN	0.27816545	0.0452768
MED8	0.23379793	0.0453775
C14orf169	0.39187512	0.0454363
DNAJC12	0.98883593	0.0454846
CEP83-AS1	1.15662475	0.0454932
OMA1	0.37583947	0.0455152
CHD3	0.26721299	0.0455152
GAB3	0.37573467	0.0455609
RP11-483F11.	1.79526562	0.0455609
SNAP23	0.1785491	0.045621
ACTN2	2.19832663	0.0457008
DKC1	0.21444425	0.045733
SH3BP5L	0.70280528	0.0458803
RP11-297N6.4	0.95421542	0.0458929
SSU72	0.17452123	0.0460025

RP11-568N6.1	0.96821013	5.41521E-05
SH3RF2	4.92373819	5.44948E-05
RP3-428L16.2	1.71662327	5.45906E-05
PSMB6	0.26734495	5.52287E-05
UBE2Q1	0.48497227	5.58105E-05
ERGIC1	0.46987855	5.65569E-05
RAD9A	0.66864426	5.69672E-05
PHF19	0.61908344	5.72833E-05
ST18	0.88707424	5.80013E-05
CHORDC1	0.97504952	5.82627E-05
PTPN7	0.48435689	5.83037E-05
TMEM219	0.20121892	5.83438E-05
CPQ	0.43216067	5.95211E-05
MAPRE2	0.63728799	5.9905E-05
TRNAU1AP	0.49860001	6.09517E-05
VOPP1	0.25373875	6.26768E-05
EXPH5	1.67875844	6.26768E-05
ATPAF2	0.44994821	6.28744E-05
PPIL3	0.42368169	6.3571E-05
FAM169A	2.82056165	6.3571E-05
N4BP2	0.35872037	6.3753E-05
GNB4	0.40290333	6.4044E-05
B4GALT3	0.60700811	6.4249E-05
PCBP2	0.27807219	6.44104E-05
RPS2	0.17743056	6.45207E-05
PHB2	0.21124886	6.56219E-05
LSM14A	0.34611242	6.56219E-05
CCDC148	1.79968255	6.59446E-05
SLC35A2	0.57122738	6.62012E-05
FAM195A	0.69037903	6.63071E-05
RTCA	0.45716511	6.69192E-05
BZRAP1-AS1	0.84537778	6.69798E-05
MYH9	0.29101206	6.70333E-05
RPL37	0.20773677	6.71335E-05
FAHD2B	1.09328658	6.7612E-05
FAM214B	0.56216394	6.82673E-05
NMNAT3	1.53095563	6.86439E-05
ISCA2	0.49385791	6.90858E-05
EED	0.56043786	6.93185E-05
LNX2	1.57925645	7.001E-05
PRR14L	0.49190461	7.04109E-05
FTSJ2	0.55734955	7.14643E-05
SARS	0.36184055	7.16175E-05
GLS2	1.8773187	7.19636E-05
MAZ	0.46096759	7.27638E-05
TMEM147	0.26963959	7.37777E-05
YARS2	0.62873249	7.56195E-05
TASP1	0.46449367	0.0461432
NXT2	0.41742188	0.0461727
EPG5	0.42842996	0.0462378
CCNT1	0.27717879	0.0463958
DMKN	2.71396635	0.0466974
PARP9	0.44742828	0.0467017
SMIM3	0.44638588	0.0467017
COA5	0.2434411	0.0470172
LINC00621	2.08674691	0.0472054
ZNF131	0.23814914	0.0474198
AHSA2	0.29906976	0.0474286
IQGAP3	1.47519582	0.0474444
SLC35C1	0.47116593	0.0475386
CREM	0.28590158	0.0476013
PPP3CB	0.27144657	0.047818
CD1D	0.1948809	0.0478308
RPS5	0.12196279	0.0478351
SCLT1	0.24212589	0.0481793
AMT	0.42617106	0.0486115
PFN1	0.12564916	0.0486762
KCNK6	0.35178031	0.0487341
HIST1H2BN	0.55495302	0.0487366
ACOT13	0.25151043	0.0487736
RP11-97C16.1	1.03250347	0.0488216
PTPN1	0.32540418	0.0489433
EFCAB11	0.44011861	0.0489719
SNRPG	0.1244154	0.0489827
PSMD5-AS1	0.35729451	0.0490804
ADCY3	0.27501387	0.0491812
LEO1	0.31018345	0.0491812
RP11-517P14.	0.66600626	0.0495694
AARSD1	0.5241964	0.049674

MOSPD2	0.56010261	7.59766E-05
UBE2G1	0.45703528	7.60439E-05
PIP4K2C	1.11970893	7.61105E-05
DTX4	1.33870116	7.73681E-05
TBCB	0.31834964	7.97031E-05
NBPF1	1.14067496	7.97507E-05
ARG2	1.26970565	7.97507E-05
CCDC91	0.46435709	8.0288E-05
RP1-34B20.21	0.92394254	8.05089E-05
RP11-350N15	1.31698532	8.13463E-05
KCNQ1OT1	1.10718677	8.33015E-05
LINC00649	1.66226847	8.3838E-05
ELP5	0.42334413	8.41336E-05
NAT6	0.92523688	8.42239E-05
MRPL9	0.38040898	8.47256E-05
DYM	0.43383796	8.95965E-05
PPAT	1.01366243	9.06008E-05
EDN1	1.54167637	9.13802E-05
MPPE1	0.37791837	9.19229E-05
PTRHD1	0.30052512	9.26419E-05
CRAT	0.95408208	9.27185E-05
BIN1	0.4602356	9.60812E-05
CSTB	0.32261578	9.61637E-05
FNTA	0.36333431	9.70636E-05
C11orf84	1.05173465	9.73631E-05
CARHSP1	0.37471866	9.73631E-05
CD79A	1.11153973	9.74535E-05
IL16	0.56093451	9.8159E-05
PNLDC1	2.69014885	9.84518E-05
TRMT13	0.76295057	9.86763E-05
VPS72	0.55838565	9.99502E-05
PTOV1	0.44174127	0.000101219
TRIB3	1.72318944	0.000101779
ABI2	0.61826795	0.000101983
LRSAM1	0.89283963	0.000103171
AKAP6	1.07556951	0.000103758
TSGA10	1.12461385	0.000104437
LIPE	1.88559957	0.000105764
ZNF232	1.41000304	0.000106832
SULF2	0.44356383	0.00010692
NR1D1	1.05341823	0.000107009
EVI2B	0.27532846	0.000107462
CXorf57	2.00431188	0.000108593
C8orf82	0.49606678	0.000109889
TBKBP1	1.79077193	0.000109889
LMAN2L	1.53176085	0.000110195
ARL5A	0.38036481	0.00011099
SEPT1	0.77388259	0.000114146
PDZD11	0.48656618	0.000114445
TMEM263	1.05938521	0.000114467

FAM234A	0.65453867	0.000114762
DMC1	2.91223451	0.00011608
CARD10	3.46003677	0.00011629
RP13-650J16.:	2.32732044	0.000118604
MFI2	1.06948248	0.000118749
CREBRF	0.40643111	0.000119101
SGPP1	0.79422486	0.000121235
SLC38A2	0.64869028	0.000121709
FYN	0.58597273	0.000122032
KLHL22	0.77766351	0.000122348
LTB	0.75491956	0.000124544
RPE	0.48031193	0.000125693
VDAC3	0.27644244	0.000126399
RNH1	0.29518148	0.000126399
ADAM15	0.58938192	0.00012799
STARD7	0.35987476	0.000128767
PHB	0.21954359	0.000129576
RPL24	0.14971275	0.00013114
DCLRE1C	0.60877067	0.00013154
C1orf35	0.50968164	0.000132861
RGS14	0.61825121	0.00013299
FXR2	0.85461948	0.000133109
ACAD8	0.70850482	0.0001335
TMEM229B	1.08381264	0.0001335
SLC7A11	1.20292339	0.000136107
STC1	3.11355945	0.000136245
CD82	0.98685754	0.000136245
LGALS9	0.26549605	0.000136642
FKBP15	0.38613656	0.00013847
MTERF4	0.50124459	0.000138742
FAM134A	0.48707533	0.000139381
PSMA2	0.1874039	0.00014003
C12orf10	0.3606458	0.00014003
RPL30	0.152636	0.000140319
RP11-452F19.	0.6498806	0.000143058
LRP5L	0.80462528	0.000144374
ROR2	3.15721335	0.000145771
ADRM1	0.26170076	0.000147814
MRPS25	0.3544846	0.000148167
NUPL2	0.40794323	0.000148167
PKIG	0.69126557	0.000148988
TLDC1	1.23807695	0.000149561
MAP3K2	0.29133664	0.000150175
RPL10A	0.1525404	0.000152301
PCBD2	0.71984488	0.000152994
GCOM1	3.24963002	0.000153819
C19orf43	0.19987923	0.000157643
CCDC92	0.67671711	0.000162023
CCNI	0.15135721	0.000162173
EMC7	0.31761511	0.000162226

HINT1	0.24638923	0.000164517
NKRF	1.04254244	0.000167121
LSM4	0.29942922	0.00016735
TNF	1.73859708	0.000167755
RBM25	0.23477751	0.000168568
FBXO33	0.3914228	0.000169183
JMJD1C	0.40913343	0.000173546
CFL2	0.93571058	0.000177316
MAP4K5	0.43937707	0.000177316
AIP	0.38121981	0.0001783
TXNDC17	0.32408695	0.000182435
RPS7	0.14215864	0.000186995
RFXAP	1.19077351	0.000187621
HHIPL1	3.22662297	0.000189535
DOCK10	0.35452288	0.0001898
SMAD3	1.53372805	0.000190017
NUFIP2	0.3021694	0.000190101
RANGRF	0.44811295	0.000190142
ENO1	0.24911676	0.000190663
SUCO	0.46054219	0.00019252
C14orf1	0.59893172	0.000193114
RAB42	1.91167133	0.000197491
FAM60A	0.47367871	0.000197904
HIST2H2BE	0.71868566	0.000199847
CLUAP1	0.809733	0.000199851
CLN3	0.40630602	0.000200014
CLASP2	0.56001299	0.000204662
MAP4K1	0.44432928	0.000206951
DCTN5	0.58461337	0.000209673
MICAL3	1.01658651	0.000210758
RPS24	0.16173174	0.000212487
SPG21	0.2843606	0.00021306
KCMF1	0.38133464	0.000216568
GYG1	0.36749197	0.000217006
HRAS	0.60348209	0.000217669
CCDC67	2.51461277	0.000218551
APOO	0.54504537	0.000222005
ISL2	2.25001545	0.000224346
ZNF664	0.77978072	0.000226139
FAM117B	0.88660243	0.000228399
KIAA1841	1.15245563	0.000231551
KPNA2	0.4472079	0.000232881
DHRS13	1.23234464	0.000234441
LMO7	1.43217453	0.00023796
FECH	0.72695372	0.000238152
GPR161	2.00771493	0.000242557
CAT	0.19686745	0.000249281
NUDT4	0.56467538	0.000250522
EHBP1L1	0.42690427	0.000257351
CCR9	3.67582025	0.000263551

RWDD1	0.24257104	0.0002636
KIAA0355	0.73579371	0.00026788
TUBGCP4	0.70138178	0.000268146
EPB41L5	2.17702553	0.00026924
ACOT8	0.5270872	0.000270516
SELL	0.82121367	0.000275597
TOMM7	0.18286859	0.000275643
L2HGDH	1.57961762	0.000276215
SNRNP27	0.34142427	0.000276257
ZBTB11	0.5871787	0.000279444
PPM1L	0.7470073	0.000279527
RP11-402D21.	1.99579175	0.000281405
AKAP11	0.52583802	0.000282709
MGAM2	2.4362854	0.000283334
TMEM208	0.26743516	0.000284045
TRANK1	0.82221346	0.000285588
GZF1	0.63145423	0.000287266
DEDD2	0.84815939	0.000287656
BMI1	0.58298751	0.000289824
RP11-303E16.	3.25589104	0.000290619
POM121	0.45410481	0.000290644
POLD4	0.44129858	0.000304206
USP14	0.31486308	0.000306945
SH3GLB2	0.47631445	0.000308229
PITPNM1	0.6336273	0.000311654
UBAP2	0.40327822	0.000313697
BLOC1S6	0.24047986	0.000313898
FAM89B	0.40376683	0.000315183
COPS7B	0.61487821	0.000319887
KAT6B	0.39947393	0.000322367
PSME2	0.24815554	0.000323349
PAK6	2.57676118	0.000323701
MAP3K11	0.42614937	0.000325051
RP11-379K17.	1.1008614	0.000328985
TAF4	0.99639981	0.000334596
ODF2L	0.75687011	0.000335043
NAT14	1.24293961	0.000342163
MIF	0.35809075	0.000343546
INCENP	1.25053392	0.000347491
TEX2	0.94594498	0.000347491
ZNF608	2.59414614	0.000348089
FAM131B	3.68696108	0.000350806
SSBP4	0.29795384	0.000353722
SYMPK	0.41511447	0.000354863
FBXO16	1.80501072	0.000358536
CPSF4	0.60764303	0.00035915
EIF5A2	1.1480584	0.000365562
MFNG	0.31407659	0.000365603
ZMYND8	0.4679388	0.000366466
CEP170	0.4472657	0.000375525

MGLL	0.55138286	0.000376575
GIPC1	0.58563056	0.000384933
CRAMP1	1.20387197	0.000385059
TAX1BP3	0.33424013	0.000387269
MMADHC	0.28099771	0.000389186
TSPAN3	0.39942365	0.000392516
LSM10	0.30872337	0.000393798
STYX	0.42556847	0.000407045
FAM229B	1.13479847	0.000409616
CBR3	1.4526137	0.00041033
COMMD10	0.30756192	0.000413783
MYH15	2.59303514	0.000416671
TMEM245	0.68089978	0.000423099
PNN	0.23109508	0.000427097
AQP3	2.03501452	0.000428795
TIFAB	0.69513599	0.00043872
CDH17	0.62807398	0.000439253
MDFIC	0.48908969	0.000444784
UBE2H	0.41692809	0.000450461
RPAIN	0.31494699	0.000450461
MRPS15	0.23217764	0.000457864
SRF	0.62252086	0.000459473
DCLK2	1.25318179	0.00046417
EP300	0.27343146	0.000465406
FASN	0.71937405	0.000468913
HEXIM1	0.39661199	0.000472712
GNA15	0.54861433	0.00048212
CARS2	0.41714031	0.000493433
GPR89B	0.92637986	0.000502666
HIST1H2BN	0.88054384	0.000506124
TTC1	0.32190135	0.000506649
SETX	0.3376773	0.00051071
UBA52	0.1450433	0.000515424
ASB1	0.72013756	0.000518396
SUDS3	0.39589893	0.000518396
HIST1H3J	2.93532845	0.000525047
PPP1R15A	0.56899347	0.000532021
RPP30	0.41954564	0.000543672
ATF4	0.29444784	0.000546922
KIFC1	0.83752161	0.00054731
CEP70	2.08359293	0.000554168
PRKX	0.53009381	0.000554724
ZCCHC24	0.85308483	0.00056297
ZNF853	2.72148221	0.000571054
GEM	0.70651965	0.000582135
GPC4	0.97585449	0.000584713
MRPS34	0.36517282	0.000585313
TUBA1A	0.37384603	0.000588805
SCUBE2	2.59512317	0.000596494
NME1	0.29928062	0.000596494

ZNF362	0.67600952	0.000606797
TRG-AS1	0.92646964	0.00061637
HAUS3	0.58653319	0.000621245
ZNF593	0.40520172	0.000623362
PIK3R3	1.28097082	0.000623711
SUPT5H	0.27460592	0.000626887
RP11-15E1.5	2.26395819	0.000633593
ZFYVE28	1.19516065	0.000639405
ZNF48	1.74792748	0.000639405
POU3F1	2.28810521	0.000639509
CAV1	1.35157933	0.00064313
TRIM22	0.2576207	0.00064313
AC009005.2	0.78364355	0.00064313
FAM69A	0.85118595	0.000652269
AC007620.3	1.94121581	0.000654337
LINC01095	2.48163509	0.000655537
ANKRD20A2	2.7802648	0.00065615
BZW2	0.2964481	0.000662775
AP1S2	0.41548071	0.000663667
ARHGAP30	0.33244477	0.000677809
SLAMF8	0.35145001	0.000682548
NME2	0.56301751	0.000684054
PAQR8	1.05999702	0.000689486
SLC38A5	1.88096414	0.000690473
PLXNB1	3.87084135	0.000705728
RUNDC3A-AS1	2.17603665	0.000707065
PPARA	0.66465652	0.000707899
HIST1H2BG	1.09742532	0.000713469
ZCCHC18	4.56575606	0.000715555
RPL4	0.17553361	0.000717562
CD81	0.42450371	0.000721961
CYTH2	0.38447024	0.000724066
TADA3	0.35154443	0.000725675
SETDB2	0.5457685	0.000725675
CLEC2L	3.98535097	0.000748024
FBXW5	0.34074882	0.000766142
BFSP2	1.1967357	0.000775351
FAF1	0.42077622	0.000779328
ETFA	0.2062081	0.000786673
ABCC11	2.21652039	0.000786673
STK39	0.7889775	0.000792495
ALKBH4	1.02513231	0.000797227
ARL10	1.46566409	0.000798317
TCOF1	0.64842597	0.000806636
TRIAP1	0.38172019	0.000808447
TULP3	1.17013568	0.000818481
FOXB1	4.02152702	0.000827857
TRIM9	3.23296324	0.000847877
RP11-563K23.	4.21743269	0.000852509
EIF4ENIF1	0.96835783	0.000855152

C1GALT1C1	0.50147166	0.000855187
H2AFY2	0.7644172	0.000856208
DNAJA4	1.22571975	0.000859196
ABCA3	1.77158067	0.000861381
ACAA2	0.36855905	0.000869868
WHAMM	0.35309851	0.000872375
ZNF789	0.65290724	0.000877455
MZF1	0.81573753	0.000878729
ZMYM5	0.4177162	0.000884147
PAPD7	0.58195898	0.000897876
EXOC6	0.38684761	0.000898989
SUSD6	0.4206877	0.000900673
FTSJ3	0.49928997	0.000912052
MRPL23	0.2941509	0.00091805
LRRC42	0.78611301	0.000919498
PRSS3	1.81650422	0.000920164
ZBTB39	1.50238618	0.000921256
RNF170	0.62573489	0.000926418
MAPRE3	0.77133622	0.00092767
BAZ1B	0.32228521	0.000942198
BLCAP	0.39839979	0.0009696
PRKD3	0.45454387	0.000971898
PPP1R2	0.19699242	0.000999376
TAF6	0.65891188	0.000999376
SETD7	0.40476084	0.001003131
EIF3C	0.46220303	0.001007116
MRPL18	0.56804588	0.001007385
ZNF467	0.4862643	0.001012402
RALY	0.20953509	0.001015015
CLEC4C	1.14113479	0.001020277
B4GALT4	0.48907009	0.001023151
KDELC1	1.75858825	0.001041655
HLA-DRB1	0.34815087	0.001046629
DACT1	3.7968608	0.001046851
DLAT	0.64705403	0.001047721
JAZF1	0.67620302	0.001059221
HLA-DQA1	0.25351793	0.001066578
STARD10	0.54522444	0.001074402
C17orf67	0.96727071	0.001075738
CYB5B	0.33894948	0.001084781
BTBD1	0.35307112	0.001101349
PTPRG	3.1345886	0.001104467
CSNK2A2	0.41632063	0.001109587
HMGB3	0.48630061	0.001114099
SLC12A7	0.59226611	0.001135086
CHID1	0.33490931	0.001145989
HDAC11	1.37715964	0.001152901
PEX13	0.59533442	0.001188753
COL4A3BP	0.33916176	0.001190291
TMEM185A	0.71518946	0.001200311

BAG2	0.65839768	0.001200584
PGLS	0.27136799	0.001213119
ERICH1	0.28508378	0.001217418
MICU3	1.85109775	0.001230042
KIFC2	0.6572686	0.001242132
RPS15A	0.11958781	0.00125645
RASSF8	1.62407202	0.001262009
SLC7A6	0.64004907	0.001271736
HMGN1	0.2015819	0.001273934
C12orf76	0.46964192	0.001285065
SCMH1	1.33905971	0.001287695
IFT57	0.42532117	0.001292952
ARHGAP5-AS1	0.8408196	0.001293666
NACA	0.11085529	0.001316609
SOBP	2.33979342	0.001318633
MED16	0.55249584	0.001329897
C3orf38	0.43038042	0.001354466
SGOL1	0.86574679	0.001355978
KIAA1324	2.10328616	0.001368242
EGR3	1.0980455	0.001397334
SREBF2	0.58659548	0.001407277
MAP7D1	0.30720332	0.001409514
SURF6	0.4779926	0.001419059
PPP1R12A	0.27346671	0.001446171
CA12	3.67639318	0.001447282
TNKS1BP1	1.1180703	0.001447651
PPM1G	0.24541593	0.001449502
SLC45A1	2.14667774	0.001466301
ITGB1BP1	0.29074959	0.001469707
GPR157	0.74704726	0.001470254
ZNF775	1.32313676	0.001479484
BEND4	2.56136186	0.001484898
HIST1H2AH	1.61979737	0.001492113
RTF1	0.25128185	0.001503447
PTTG1IP	0.30815201	0.001514804
MRPL37	0.3621824	0.00153324
P2RY10	0.60036081	0.001535551
NDUFB6	0.22808271	0.001536406
FBXO3	0.57457315	0.00153681
ZNF771	1.54263613	0.00153681
RPL32	0.10770044	0.001540214
NUB1	0.32527915	0.001548295
EGLN2	0.39187539	0.001550434
CLPP	0.31808077	0.001568159
SPRYD7	0.60859583	0.001569307
NXF1	0.41525937	0.001586066
RUVBL1	0.37449237	0.001593968
NENF	0.24269663	0.001600095
RPS14	0.15582175	0.001600484
PTOV1-AS1	1.95634119	0.001611509

PON2	0.35009787	0.001639468
ANKRD35	1.99072224	0.001658502
LRP6	2.24780205	0.001666735
LINC00493	0.16613337	0.001670494
NPEPPS	0.33501179	0.001672008
ACVR2B	1.47833888	0.001681386
LRIG2	0.8438085	0.001682117
GATC	0.57321469	0.00168672
VBP1	0.34021599	0.001689027
ERMP1	1.18413894	0.001692989
RP13-467H17.	2.57393325	0.001697084
KIF20B	0.4236475	0.001710183
ZFAS1	0.24372081	0.001713229
MAP3K13	0.26119369	0.001717292
PPP2R5C	0.26101047	0.001737073
MGME1	0.51436664	0.001748239
SCML1	0.38073192	0.001757092
HLA-G	3.49730319	0.001781295
SNAPC5	0.42326587	0.001790297
N4BP3	1.59701041	0.001820104
BBS4	0.87043147	0.00182223
GTF3A	0.21586997	0.001824915
PNKP	0.31006712	0.001855526
FGD2	0.35385298	0.001861354
SLC25A6	0.15290789	0.001895893
HLA-DMA	0.12720351	0.001896694
NQO1	1.19784087	0.001898612
FADS2	0.72804647	0.00190092
TRAM1	0.30152605	0.001915847
NELFCD	0.32578531	0.001917293
C3orf17	0.37553848	0.001937478
MICAL1	0.3608571	0.001946247
DCTN3	0.23364729	0.001950317
SLC35A5	0.57043457	0.001969603
APOBEC3B	2.19384235	0.00197492
CLMN	0.87056168	0.001980961
ZNF433	1.02305082	0.002001395
ARID4B	0.23819363	0.002006549
ZDHHC24	0.42515792	0.002007979
C19orf38	0.31051585	0.002007979
TEF	1.55670272	0.002010282
HDAC7	0.82946494	0.002011192
PILRB	0.51087594	0.002022326
RAN	0.17766555	0.002024745
MUC12	0.97196248	0.002027194
DNAJA1	0.61569644	0.002028551
CIC	0.51841138	0.002029225
SMURF2	0.42466101	0.002040315
CEP128	0.64732103	0.002054499
TIGAR	0.32810308	0.002061696

ZNF250	0.65304007	0.002084134
UBR2	0.41196296	0.00209396
HACD2	0.4587218	0.002094857
CABYR	2.02768138	0.002113524
HIST1H2AL	1.66981102	0.002117113
HS6ST1	0.74448848	0.002135807
VAC14-AS1	2.61034117	0.002138047
SLC25A36	0.27500202	0.002160999
MIB1	0.63562791	0.002174367
TUBA1C	0.26873826	0.002248014
HIST1H2BM	4.25560718	0.002252172
ATAD1	0.41934102	0.002252172
SFI1	0.57912223	0.002271592
GRPEL1	0.25898795	0.002283466
DYNC2LI1	0.7641448	0.002290057
AP1S1	0.36547199	0.00230354
FAM57A	0.84850582	0.002309897
SRRT	0.35517006	0.002312331
SLC25A22	0.74394611	0.002325239
YRDC	0.49286201	0.002335939
SRSF9	0.19599284	0.002343868
LA16c-321D4.	3.77570702	0.002343868
ACADSB	0.7440028	0.002360166
DIAPH1	0.24707179	0.002378682
RP11-244M2.1	4.04980562	0.002381034
TDRP	2.65081811	0.002388967
RPL12	0.11467505	0.002423585
PLIN3	0.265664	0.002436568
AE000662.93	2.49974707	0.002460311
MTMR14	0.26206088	0.002464164
FUT10	1.12928997	0.00247723
PLEKHN1	1.81623307	0.002481293
ASAP2	0.69644239	0.002481293
PTPRM	2.22255831	0.002507016
HIST1H4E	1.15557183	0.002557732
MTURN	0.51416197	0.002557732
ZFYVE26	0.88497454	0.002565805
LRFN1	1.31123967	0.002569129
CCT5	0.21043023	0.002579045
ARMC7	0.91897031	0.00258301
HIPK2	0.42770434	0.002583731
LMOD3	1.04559623	0.002600976
XRRA1	0.67333182	0.002630453
GSR	0.53120251	0.002652133
IFT43	0.47272297	0.002678221
CTB-50L17.10	0.45528316	0.002683245
ASXL1	0.36165348	0.002725219
SERPINH1	1.39256077	0.002725382
MCM3AP	0.46008623	0.002763669
FAAP20	0.24120714	0.002783898

BEND6	0.72415411	0.002833442
CTC-241N9.1	1.9325495	0.002887906
RP11-181E10.	1.74209727	0.002894375
SNRPF	0.23130808	0.002896989
SETD6	0.70933538	0.002896989
NOP16	0.35581411	0.002900805
WASL	0.44710881	0.0029161
C15orf57	0.33325823	0.002928388
RGP1	0.71646678	0.00292987
BACE1	0.69176947	0.00297299
PARD6B	1.30264513	0.002979553
TXNDC11	0.37923317	0.002992135
PRKAG1	0.2837201	0.00299377
SLC39A13	0.7797688	0.003003988
RP11-90L1.8	2.60145743	0.003031691
AC007325.4	1.00936447	0.003033553
NUDCD1	0.53601203	0.003038077
TNFRSF10B	0.35650798	0.003051268
IER5	0.69911397	0.003059082
MYO18A	0.73062565	0.003059082
GAN	0.53801276	0.00305935
ZBTB44	0.36389479	0.00306549
POMP	0.18559311	0.003070044
WWP2	0.44313764	0.003091449
KIAA0100	0.38771821	0.003101455
SLC9B2	1.03296893	0.003105774
DENR	0.22337496	0.003112999
OPHN1	0.94348875	0.003119178
ZDHHC13	0.80715989	0.003124907
RP1-193H18.2	2.32820658	0.003125833
NOP58	0.24052757	0.003179529
C9orf9	1.37075372	0.003179598
CCDC113	3.5291598	0.003202382
USP13	0.79555806	0.003203722
RPS3A	0.11577631	0.003205984
NANOS1	1.71499034	0.003235709
HS3ST1	1.24836047	0.003252152
ZNF114	2.83253118	0.003260379
AC023590.1	0.9313636	0.003263638
CPTP	0.72527787	0.003329273
FANCL	1.10035573	0.003338586
CBFB	0.37765038	0.003384172
NANOS3	2.25667664	0.003409963
GALNT2	0.32955478	0.003420286
UBE2D2	0.15382131	0.003420286
SMARCC1	0.24515191	0.003421793
ENDOD1	0.6658901	0.003427269
BYSL	0.65812733	0.003428343
SLC16A11	2.58979114	0.003445522
KEAP1	0.55699456	0.003470511

H2AFZ	0.27214735	0.003471401
SAP30L	0.64509227	0.003551175
ZCRB1	0.22032471	0.003578451
FBXW2	0.40598852	0.003603108
TCAF2	0.78035174	0.003656096
DCAF11	0.36082297	0.003697469
WDR3	0.65290847	0.003764984
IRF2	0.39756321	0.00377956
FYCO1	1.17184728	0.003807667
CHAC1	2.25883847	0.003807667
GEMIN7	0.56590124	0.003808899
EIF5	0.31026573	0.003810189
DPF2	0.44401711	0.003843945
RASSF8-AS1	1.44889004	0.003846868
ZUFSP	0.48118883	0.003908918
SIRT1	0.32753818	0.003910438
CTC-524C5.2	0.66852802	0.003952361
DYNC1LI1	0.2265728	0.003955594
TUBG1	0.68487959	0.003963251
DOHH	0.58165033	0.003964149
STRADB	0.57190707	0.003965178
RP11-48B3.4	1.17357276	0.004005967
VWA1	1.46533804	0.004018755
KRT18	0.70768955	0.004022392
HMGCS1	0.38692418	0.00402874
NEURL3	1.23851974	0.004059744
CHST15	0.5697707	0.004081092
RABL6	0.37946574	0.004170813
TCAF1	0.63500015	0.004195186
KRTCAP2	0.22147856	0.004209873
SLC12A9	0.36825791	0.004258542
SYNE2	1.44146586	0.004301972
TMA7	0.12937006	0.004309349
MAP1LC3B	0.27395501	0.004325955
MYD88	0.20849672	0.004384819
RPL41	0.14483983	0.004388971
PTGES3L	1.94438228	0.004398717
HSPA1B	1.5056432	0.004413851
COCH	2.67125064	0.004449321
HIST1H1D	0.41520304	0.004461127
MYBL1	1.00258497	0.004494245
CHD4	0.28072952	0.004500396
STARD9	1.53840638	0.004528819
PGM2L1	0.8788886	0.004557006
EGLN1	0.38138703	0.004566092
FAM111B	1.38689842	0.004574997
CBX6	0.35629605	0.00457953
TMEM101	0.323657	0.004582578
ELK4	0.30815409	0.00460148
FAM83G	1.36461037	0.00461269

DYNLL1	0.24333423	0.004632747
MESP1	1.24068669	0.004688629
MTRR	0.71083345	0.004728864
ALG1	0.53639711	0.004752751
SERTAD1	0.37310596	0.004770538
UROD	0.30147844	0.004774352
CTD-2165H16	1.73252413	0.004795546
PITPNA	0.2678124	0.004796509
TRIM6	2.57089574	0.004832051
FBXO34	0.33768041	0.004840037
VIM	0.32817877	0.00484484
SNHG19	0.50618364	0.004866362
MRPL36	0.31844573	0.004872524
C2orf80	1.95468372	0.004904008
COX7A2L	0.21414347	0.004917297
DAPL1	1.16854777	0.004993498
BTBD19	0.86697261	0.00499939
FAR1	0.36153789	0.005080991
RP11-489E7.4	0.62081414	0.005082308
NELFA	0.53353339	0.005084376
MAP4K2	0.593484	0.005109575
CNKSR1	2.56447185	0.005109896
GYS1	0.62016488	0.005112388
CCDC121	1.14250992	0.005133496
HPS5	0.28315852	0.005144279
PSMB4	0.19285727	0.005175042
LCORL	0.4795668	0.00519353
CPLX1	2.37963758	0.005204653
RPL29	0.0984789	0.005214051
FAM114A1	1.01802666	0.005227128
MSL1	0.32250511	0.005237641
RHOV	2.60670941	0.005262629
PSME3	0.35417924	0.005283587
AIFM2	1.05879071	0.005289397
ZNF215	2.17000091	0.005298313
KIF3B	0.58467384	0.005319347
FRY	0.9106232	0.005420483
NDUFAB1	0.16144493	0.005447318
KCTD11	1.12709306	0.005447318
CPSF3	0.30859994	0.005451679
DPP3	0.41443296	0.005460823
MAN1A1	0.24174361	0.005469384
TNRC6B	0.23907938	0.005543142
H2AFX	0.40752571	0.005562551
GABRE	3.33739775	0.005580412
NUP88	0.44271559	0.00567335
SERGEF	0.46694006	0.00568364
C3orf52	1.91487874	0.005734466
HSH2D	0.43512464	0.005799283
ZBTB38	0.37875888	0.005838593

ZNF451	0.27161334	0.005853139
OCIAD2	0.68534385	0.005854484
CTH	0.86571117	0.005858147
MTA1	0.40893028	0.005861027
RGS1	0.52511991	0.005861897
RP11-758H9.2	0.90379293	0.005872154
GAR1	0.29111318	0.005872561
MEGF8	0.94026589	0.005882598
CTD-2012K14.	1.4496736	0.005938891
RPL19	0.14746725	0.005969878
GRHL1	1.42668884	0.006013053
TEX264	0.24283661	0.006065211
BRI3BP	0.34145656	0.006065211
SKP1	0.14564069	0.006110532
TMC8	0.6723788	0.006123642
PLEK	0.47502139	0.006134529
OS9	0.22599377	0.00616887
APBB1IP	0.25239289	0.006173183
KLHL32	4.00912976	0.006206572
ALG5	0.23183159	0.006214369
MAN1A2	0.27321684	0.006260488
SNRPD1	0.23006466	0.00626376
SLC20A2	0.47178987	0.006283989
HSD11B2	2.67463873	0.006284437
SYCP2	1.56689503	0.006297287
MARVELD2	1.80050587	0.006372651
KIAA0895L	0.90199795	0.006380796
PARP16	1.10531469	0.006451744
NOSIP	0.25102855	0.006456692
LINC00853	2.90938891	0.006493519
MFSD10	0.28341183	0.006501596
CALR	0.26062247	0.006564501
LYRM1	0.38774422	0.006576575
PLEKHF2	0.38518416	0.006603514
RP11-484K9.4	2.6492863	0.006643873
CTPS2	0.66998104	0.006662727
METRN	0.57384367	0.006709503
SAMD12-AS1	3.6859298	0.006733587
LONRF1	0.35597756	0.006749563
GCSH	0.45670055	0.006763561
CNOT8	0.35212387	0.006809392
STK40	0.37770668	0.006890894
TMEM86B	0.98328204	0.006934749
SELM	0.36310409	0.006975731
PPP2R2A	0.29859467	0.006985101
HMGN3	0.16548089	0.007076257
TTC39C	0.45116519	0.007098686
DHRS1	0.43581005	0.0071101
SLC35E4	0.92373304	0.007186772
VARS2	0.77701387	0.007262882

CTB-119C2.1	1.32031893	0.00729247
FLJ13224	1.84786176	0.007372595
ATXN2	0.40143163	0.007390643
LINC00865	1.26728592	0.007423896
POLK	0.36993494	0.007498205
C10orf12	0.53971706	0.007514096
TNFSF18	2.56164994	0.007517741
NAP1L4	0.28484332	0.007562302
TTC32	0.50566236	0.007593505
HIST2H2AC	0.39587996	0.007618711
C16orf70	0.87578591	0.007725246
CHMP5	0.27469063	0.007795388
PSMC2	0.21910287	0.007804169
AIRN	2.04075081	0.00789658
CAMSAP2	0.76192988	0.008033982
FOXN2	0.36964392	0.008033982
CCDC71L	0.50951896	0.008167513
LSR	1.0022255	0.00822471
PRMT1	0.19602712	0.008226401
UHRF1BP1L	0.29437634	0.008278184
EIF3L	0.16893055	0.008291856
CNKSR3	2.21269402	0.008297752
RBBP6	0.22137256	0.008364375
NUSAP1	0.50061548	0.008396244
SRSF11	0.18133162	0.00852452
DLEU1	0.5475335	0.008566293
PCGF1	0.67303775	0.008615089
ACADM	0.33132147	0.008625092
NDUFA2	0.1709793	0.008625092
PAPSS1	0.296385	0.008628467
ATXN2L	0.2730458	0.008649529
COX8A	0.15666777	0.008656025
TNKS2	0.25002389	0.008708575
TDRD1	2.29647456	0.008735263
MTSS1L	2.76826295	0.008763887
SPIN3	1.56944709	0.00876506
TIFA	0.45227543	0.008773333
AACS	0.68503027	0.008816239
RP11-115C21.	0.66647915	0.008819745
TIMM22	0.4283444	0.008848756
SNRNP70	0.24910252	0.008913209
LINC00885	1.65710069	0.008989775
FAM114A2	0.45768493	0.009004265
TYW1B	0.86096988	0.009010192
AHCY	0.30728407	0.009027572
HIST2H2AA4	1.81598612	0.009067326
GART	0.42039465	0.009183493
MLF2	0.18414267	0.009214018
CPT1A	0.30491819	0.009215321
NADK2	0.63226998	0.009215321

CD320	0.639847	0.00925724
FBXO42	0.46732418	0.009367674
JADE3	1.32536284	0.009419328
MINPP1	0.66558421	0.009439793
FAM210A	0.37711032	0.009555646
LYL1	0.47382231	0.009580575
CTD-2555A7.2	0.88136494	0.009605126
NLRX1	0.9519522	0.009655134
FDX1L	0.51868495	0.009668382
PPP1R3C	3.50430901	0.009728047
LRRK1	0.46255102	0.009753262
AC137932.4	0.63786088	0.00977078
SFXN1	0.65090027	0.009795791
PPP4R1	0.43718555	0.009795791
C1orf122	0.31354293	0.009841848
CINP	0.25629949	0.009880859
SLC48A1	0.6890404	0.009887659
FAN1	0.71666931	0.009920891
ARHGAP17	0.55137418	0.009996381
MAP2K2	0.32306133	0.009996381
TRUB2	0.44846251	0.01007362
UGDH	0.49893991	0.010102913
TADA2B	0.66031798	0.010103194
PPME1	0.62737886	0.010105368
COX14	0.16334487	0.010138167
MRPL19	0.30166661	0.01016778
PCED1A	0.48964623	0.01020786
RPS6KB2	0.30217895	0.010261179
GCAT	0.77731043	0.010292746
TIAL1	0.23175539	0.010329768
AC012368.1	1.83073601	0.010381678
MBTD1	0.51143421	0.010386976
FAM104A	0.51196261	0.01045828
AHI1	0.37935617	0.010646244
TMEM214	0.39718432	0.010669173
TGM5	2.0319067	0.010766158
ZNF436	1.64930662	0.010864258
CASP8	0.29987715	0.010867912
RPL11	0.10041451	0.011003056
SEMA4G	1.09845767	0.011003056
BIVM	0.85730367	0.011040473
NDRG2	0.2615364	0.011042032
WASF3	1.74247013	0.011076462
PANO1	1.18408246	0.0111677
FLVCR1-AS1	0.67754983	0.011211195
PRRC2B	0.32405337	0.011268186
TMTC4	0.90645486	0.01131162
CRHBP	1.1494519	0.011531427
CPOX	0.61411074	0.011601643
FAM217B	0.52712885	0.01162977

AP3S2	0.5249094	0.011642731
NCAPG	1.39076755	0.011668904
BIRC6	0.21991516	0.011670365
ATP13A1	0.44463436	0.011745687
MLLT10	0.31617539	0.011773761
CACHD1	2.55010926	0.011828171
TIMELESS	0.85378345	0.011839569
UBE2M	0.23273342	0.011920286
GOLGA6L4	1.1521416	0.011925791
FBXO25	0.64036311	0.012079915
FAM134C	0.52194401	0.012091653
ANKS3	0.76513152	0.01209312
PTPRA	0.26412887	0.012147682
LPAR5	1.09578594	0.01217157
DTD2	0.54622257	0.012190937
HIST1H4J	0.69448844	0.01229225
TXNRD1	0.32020073	0.012301706
PLS1	1.82919079	0.012303784
IPMK	0.25630278	0.012332231
NAB1	0.4011011	0.012346393
RNF208	1.07441454	0.012390746
TRAPPC4	0.24215945	0.012431553
TPP1	0.14581842	0.012463457
CC2D1A	0.5774021	0.012476357
ECI2	0.30168273	0.012554428
ST3GAL4	0.58721069	0.012558086
VPS4B	0.21680033	0.01258991
RP11-395A13.	0.44211354	0.012622007
BTF3	0.11714348	0.012622007
EIF3M	0.12927035	0.012622007
KCTD13	0.63764825	0.012748203
LRRC7	2.22818569	0.01277139
MARCH8	0.38136171	0.012840511
ZNF334	1.70887748	0.012860202
ARHGDIB	0.16030965	0.012875516
NRIP3	0.4691194	0.012930467
TIAF1	2.21377188	0.012983542
RPL6	0.10228933	0.013191587
ABCB7	0.2970676	0.013192331
PDDC1	0.50947745	0.013248379
TOP2B	0.25356836	0.013261558
GNG5	0.1686831	0.013280972
ADGRB1	2.28311769	0.013350076
MTL5	1.39868463	0.013423855
TVP23A	0.63169808	0.013448284
ZNF747	0.69348129	0.013544376
HYPK	0.60028901	0.013774952
RP11-706O15	1.73758774	0.013823977
ZNF439	0.59723565	0.013830447
FBXO21	0.32459363	0.013939936

CAPRIN2	0.76953599	0.013991054
CTD-3035K23.	1.37867889	0.014068952
FRY-AS1	0.99286064	0.014112717
RAD50	0.26927976	0.014169719
IRF3	0.31224421	0.014278485
FAM212B-AS1	2.6319828	0.014302754
TAPBPL	0.29247037	0.014317856
ZNF124	0.36843484	0.01451033
RPL7A	0.10361144	0.01451033
PPP2R5D	0.65441609	0.014513432
THOC6	0.2836765	0.014607107
CHEK1	0.88436598	0.014613548
MIR222HG	0.95381114	0.014642447
WDR91	0.4437752	0.014714728
CDC40	0.2646377	0.014769385
AP5B1	0.49873038	0.014769385
RP11-293A21.	1.47263952	0.014874984
TUSC2	0.33245381	0.015166226
TSPYL5	0.68651352	0.015292967
H1FX	0.32571905	0.015304819
USP38	0.35368968	0.015326722
RABAC1	0.23440582	0.01547688
SUPV3L1	0.46121579	0.015555464
NPM3	0.25445783	0.01560485
LARP4B	0.31327019	0.015818646
TMEM79	1.35247857	0.015891629
HIST1H4C	0.24891534	0.015948264
MTHFD1L	0.40416912	0.015964154
PLXNA3	0.74942152	0.015964154
CCDC28A	0.29107864	0.016235487
SPTAN1	0.30477055	0.016276453
C19orf70	0.13131558	0.016484426
CDKL2	2.85821362	0.016499621
BPGM	0.53613905	0.016505377
REXO4	0.42901588	0.01651905
RFNG	0.39004166	0.016543568
RP11-20I23.8	1.0925223	0.016554132
DISC1	0.51857206	0.016595776
RP11-98D18.9	1.26347858	0.016637532
RP4-555D20.4	2.41057733	0.016801609
IAH1	0.19506327	0.01681519
KMT2B	0.3566024	0.016886755
CD300LG	1.33073259	0.01703185
CIART	2.2239771	0.017101319
RPL7	0.10987263	0.017151474
PPP1R3D	0.70207067	0.017357483
HIST2H4B	1.7666372	0.017444491
FAM136A	0.22982226	0.017520802
HIST1H2AI	1.73198954	0.017550681
ZNF623	1.13642021	0.017627506

NECAP1	0.51882423	0.017652096
SMG7	0.40086819	0.017677202
RNF113A	0.40935795	0.017684152
GNPNAT1	0.59029568	0.017684364
ZNF814	0.52309611	0.017777876
COMMD5	0.36064169	0.017787904
LRRC16A	0.73143921	0.01787626
WIPF2	0.29618435	0.017928004
MSMP	2.17337248	0.017953718
RMI1	0.58263267	0.01797799
CCNL1	0.30496751	0.017982716
SLC16A1-AS1	0.68381115	0.018082031
UST	0.58855983	0.018083724
TMEM121	1.16361001	0.018220038
ZNF143	0.56845513	0.018225296
PSD4	0.46813933	0.018284184
SCAPER	0.46546567	0.018347075
VSTM2L	2.76755202	0.018355267
MEN1	0.66776263	0.018416727
SH2D3C	0.78662466	0.018482153
HIVEP3	0.58637174	0.018659485
DNAJC19	0.23525075	0.018717805
MCOLN2	0.48077909	0.018949984
TMEM25	1.79428743	0.018959188
CALM1	0.1637795	0.01909521
C7orf55	0.28286629	0.019165795
TGS1	0.3416509	0.019165795
TNFSF8	0.62066615	0.019170756
DENND2D	0.64787735	0.019255289
NIPAL3	0.51407059	0.019344955
ARNT2	2.77427297	0.019356921
ACIN1	0.20951198	0.019387467
ATM	0.29498955	0.019537467
SCO2	0.19417263	0.019586827
GALNT12	0.84452372	0.019658876
RP5-1065J22.8	0.56009567	0.019762402
POLR2I	0.19875534	0.019778781
SPAG9	0.23080158	0.019784808
HDHD3	0.89593843	0.01982644
DNAJB1	1.10396402	0.019834286
CFAP45	1.0402556	0.019959073
ELMO3	1.50718274	0.020157141
RP11-290C10.	1.87966957	0.020262845
RP11-297C4.2	1.94356913	0.020571821
SDC2	0.82903852	0.020616319
ZNF641	0.53119041	0.020622149
PRRC2A	0.27588419	0.02065188
MRPS23	0.27458645	0.02065188
BACH1-AS1	1.19510233	0.020656412
DTNBP1	0.29775796	0.020663743

C14orf166	0.10852377	0.0207451
UBE2L3	0.14938715	0.020766142
STRN4	0.43850458	0.020868133
CRKL	0.45112661	0.02102842
LYPLA2	0.30540082	0.021080539
TMEM145	1.42510934	0.021118426
SULT1C4	1.35929584	0.021285215
RP11-307C12.	1.37506202	0.021362818
BAG3	1.22780723	0.021508445
RP11-532F12.	0.6865623	0.021514663
THOC5	0.38479319	0.021641908
RBBP5	0.44677327	0.021723565
LUC7L	0.29063611	0.021733861
HIST2H3D	2.06591462	0.021733861
MDK	1.16990831	0.021759897
NEU1	0.44688354	0.02185382
HOXA9	1.72476434	0.021904175
NGFR	2.45797832	0.021976579
MFSD14C	0.81184762	0.022019589
NFATC2IP	0.23664099	0.022077923
SNHG10	0.61855891	0.022108573
ASH1L	0.26235903	0.022186668
C9orf85	0.36947738	0.022310497
AFF1	0.29090155	0.022696108
SSB	0.18059013	0.022736855
ACLY	0.2870514	0.022885756
GARS	0.30839169	0.02291708
NOMO1	0.4754221	0.023058542
NDUFB11	0.12721985	0.023162157
NDUFB2	0.15165657	0.023174804
GSKIP	0.50542246	0.023200669
IGFBP5	1.87431257	0.023358776
ZBTB10	0.39059675	0.023368293
VASP	0.26661542	0.023466355
SASS6	0.78470304	0.0235438
TSPYL2	0.47180841	0.023696804
PSMB5	0.24638107	0.023700751
EDF1	0.11476944	0.023799233
PAM	1.02004636	0.023986778
WWC3	0.58264102	0.024010799
ACTL10	1.13062668	0.024044375
HIST1H3G	2.50460052	0.024123674
MFAP3L	1.68456425	0.024200702
CMTM8	0.68299988	0.02421876
ZC3H7B	0.5094513	0.024241554
CAPZB	0.14986317	0.024386006
SERPINB1	0.28016649	0.024436572
KLF5	0.69440039	0.024749004
ARRDC4	0.76129164	0.024792175
LBHD1	1.441074	0.024929624

LA16c-380H5.	2.17282849	0.025058234
TTI2	0.69475108	0.025105296
TMEM102	0.59802479	0.025122447
OTUD6B	0.40581533	0.025421102
HIGD1A	0.29578667	0.025437396
STRA6	3.19161708	0.025439796
UBR5	0.29913432	0.025635203
OGDH	0.29822049	0.025689449
IP6K2	0.28775534	0.025756525
SEMA4A	0.45860309	0.025756935
USP46-AS1	1.18103719	0.025880857
RP11-666O2.2	0.57175037	0.025881444
ZC3H7A	0.2597931	0.025933425
ITGAE	0.24084814	0.025949037
NFATC1	0.40076273	0.026119282
RP1-111D6.3	1.60503579	0.026156931
RP11-126K1.6	0.36561156	0.026169644
HINFP	0.48176157	0.026169644
SGTB	0.46923902	0.026184152
SRP9	0.13658608	0.026253976
SLC16A5	0.7144649	0.026319241
NAGA	0.20463425	0.026431029
TRPC4AP	0.28589635	0.026448231
AP001059.7	0.94635214	0.026452412
ZNF205	0.89712515	0.02647456
TSTD3	0.92700637	0.026477423
QARS	0.19318989	0.026489095
RHOXF1-AS1	1.50019513	0.026520592
ZNF764	0.84214649	0.026520592
SF3B6	0.11985339	0.026536061
PPP6R2	0.33058306	0.026616776
SFPQ	0.18570231	0.02665013
AC010976.2	1.10983396	0.026757826
NSMCE3	0.27666055	0.026846052
NAE1	0.34896165	0.026886165
CNP	0.4104435	0.026894482
CDS2	0.34089097	0.0269679
CCDC85C	1.05512246	0.027018765
CTD-2349P21.	1.0403219	0.027104918
PTPN4	0.8148836	0.027188971
SULT1A3	0.77575357	0.027498842
STXBP3	0.22149795	0.027520584
MRPL49	0.42605033	0.027595978
DGKE	0.4832038	0.027595978
FBXL20	0.49209408	0.027604849
UBE2I	0.1264263	0.027701222
C10orf35	1.60889932	0.027757269
SNRPA	0.2211154	0.027790683
MYPOP	0.50749742	0.027804056
PBX3	0.38042672	0.027865271

WHSC1	0.4183654	0.027908241
TBPL1	0.40401393	0.027908241
GPBP1L1	0.224343	0.027930241
KLF16	0.37809677	0.027975173
CLIP1	0.20988351	0.028297588
LPP-AS2	1.13286656	0.028371014
GABPB1-AS1	0.25542902	0.028419183
SLC41A3	0.45972621	0.028451765
ANAPC1	0.43493553	0.028455884
TTYH2	0.35754544	0.028492126
CCDC153	0.78925446	0.028513993
CMIP	0.20229858	0.028544731
DYNLRB1	0.144943	0.028593106
RAD52	0.92221876	0.028627582
RPH3AL	0.79342178	0.02864393
C12orf65	0.49723333	0.028670327
SUMO4	0.63732043	0.028825061
NEK8	1.12554629	0.028856368
DGAT1	0.28046021	0.02895024
YIF1B	0.19258797	0.028956134
TCP1	0.2685617	0.029119753
RP11-219B4.7	1.99372642	0.029552224
CARNMT1	0.53780982	0.029583625
ARMC6	0.39358532	0.029645531
DDX46	0.16285065	0.029647124
TTTY15	0.95166931	0.029752198
MAPKBP1	0.78349388	0.029803667
GFM2	0.54264581	0.029982932
HOOK1	1.91636135	0.030307852
CASP3	0.32506996	0.030357624
NOA1	0.30897389	0.030528534
CCNK	0.2105814	0.030795128
FBXW9	0.59366571	0.030797132
ARC	1.28200003	0.030883267
MAN1B1	0.35340484	0.030887195
COX6C	0.16140305	0.030934688
FAIM	0.57471582	0.031140392
KIAA1211	3.41307048	0.031154785
EIF4A2	0.22167109	0.031187138
RBX1	0.10918776	0.031190738
BACE1-AS	1.62732541	0.03122728
CEACAM3	0.69267862	0.031610175
PODXL2	0.91712674	0.031613529
CBFA2T2	0.57613637	0.031615838
TTLL3	0.48118572	0.031697395
MAP9	1.01406256	0.032053204
ZFYVE9	1.75383239	0.032319864
RUNDC3A	1.69781766	0.032334668
EBNA1BP2	0.22059855	0.032337596
MNAT1	0.4210281	0.032438481

ADORA2B	0.51897195	0.032503003
TMEM9B	0.15255533	0.032527246
LRWD1	0.65679293	0.03258073
TOPORS-AS1	0.29196775	0.032603172
LRRC8A	0.56240584	0.032624616
JUN	0.86391347	0.032643186
BIRC5	0.8851029	0.032738532
TGOLN2	0.13360762	0.032969818
HIST2H2AB	1.44460689	0.032969818
BCL2L12	0.35881472	0.032970552
TMEM231	0.73493154	0.033133792
POLR3K	0.30326639	0.033265144
AAGAB	0.41004196	0.033276281
RAB3GAP2	0.33880698	0.033381183
ASB8	0.34168335	0.033381183
ABCF3	0.37178343	0.033387734
MIPEP	0.71303381	0.033405953
TMEM216	0.45976565	0.033433781
AK9	0.74455111	0.033453306
NAPEPLD	0.88398502	0.033453306
C17orf89	0.20219068	0.033453306
TAF2	0.35262263	0.033810753
INPP5E	0.81007903	0.033949056
HIST1H2BL	1.55928987	0.034003089
JADE2	0.48176653	0.034020351
G6PC3	0.26837873	0.034051679
OTULIN	0.31775503	0.034056841
RP11-706O15	0.77214123	0.034061495
IMMP1L	0.37619549	0.034237377
PPTC7	0.2682138	0.034237377
PIGB	0.52365657	0.034319574
PTBP1	0.1790592	0.034434029
FLOT1	0.236932	0.0344806
VCPIP1	0.3288892	0.034559244
TOR1AIP2	0.21921141	0.034873892
COX17	0.17903753	0.034873892
BID	0.26467253	0.034928067
EVC	2.11633028	0.03494898
SYNRG	0.32121236	0.0350666
ST3GAL4-AS1	1.00202063	0.035109201
GSG2	1.54793268	0.035109608
POU2F1	0.4323526	0.035192796
FAU	0.08146244	0.035192796
ZFYVE1	0.64490049	0.035306213
DNAJA3	0.59073837	0.035440162
YPEL2	0.36108871	0.035445933
CCDC24	1.18233238	0.035450405
RAB11FIP3	0.87505758	0.03555358
CTD-2287O16	0.68323943	0.035616889
CNOT7	0.17607339	0.035619255

MAGI3	0.99697207	0.035647552
FBXO41	0.63091281	0.035648335
UBE3D	0.98440838	0.035818938
CTD-2544N14	0.57176185	0.03596765
RP11-430G17	2.27000465	0.036374836
USP31	0.80139594	0.036376404
POLL	0.42089225	0.036532662
WNT11	3.29128132	0.036536566
GNB2L1	0.09083907	0.03684203
RP11-48B3.3	1.60923396	0.037358069
DNAJC30	0.45355441	0.037424849
POLR2A	0.20216212	0.037661306
MCM4	0.40795733	0.03766751
MFHAS1	0.57780294	0.037676828
KLC1	0.33840317	0.037797158
CSTF3	0.2655984	0.037809127
ACAD10	0.70740621	0.037809127
DYRK3	0.62653701	0.037847856
MINA	0.42465048	0.037885842
ARID3B	0.62196186	0.037992568
ZMYND15	0.31393564	0.038090271
RP11-567M16	0.85996787	0.038363196
RP3-477O4.14	0.36486312	0.038424738
SMDT1	0.17177522	0.038755488
COMMD3	0.2225063	0.038817209
CORO1C	0.24896519	0.038897811
CENPN	0.36059806	0.038897938
ATXN7L3	0.391316	0.039278961
THEM6	0.73153088	0.039308868
BCAR3	0.62588443	0.039371273
ECH1	0.14071425	0.039417936
PTPRO	0.83041597	0.039507921
DHPS	0.20373213	0.039537766
LINC01006	0.8877975	0.039567999
TRAF6	0.45977573	0.039893969
FAM212B	0.61282731	0.039970987
IARS2	0.24774789	0.040008953
CPXM1	1.91513247	0.040016636
MRPS9	0.34477549	0.040020721
ATP5L	0.1142611	0.040047236
CCNJ	0.48917908	0.040141492
RP11-266L9.5	1.69613542	0.04060726
RRP7A	0.15022545	0.04062246
SULT1A1	0.50433974	0.040750866
POLR2J	0.14385753	0.040877307
ZFAND2A	0.81906405	0.040909463
VSTM4	1.99355116	0.040909463
ARHGEF12	0.43594573	0.040911237
EPB41	0.23004708	0.040977352
TMEM55B	0.30301507	0.040982459

PIK3R6	0.41490205	0.041047857
RTN2	0.3295419	0.041189175
CASC3	0.38904437	0.041263601
NFRKB	0.49197904	0.041361418
FBXL22	0.99383031	0.041434513
INO80E	0.26796278	0.041543397
TARS2	0.43475212	0.04157786
TMEM134	0.22693253	0.041594515
KLHL15	0.40896696	0.041670927
LINC00426	1.81204534	0.041670927
DNAL4	0.64634069	0.04168327
PYGM	1.46168767	0.04170871
LSM1	0.20217046	0.042089222
CCSER2	0.29076898	0.042251032
ALDH3A2	0.35875754	0.042251032
CEP85	0.73904207	0.042253516
SRSF2	0.14416033	0.042281661
KCTD1	1.75317393	0.042620543
UNC119B	0.91393172	0.042631609
ATAD3C	1.40767352	0.042795743
ITGA4	0.19341529	0.042825404
NT5C	0.19329991	0.042988908
ITPKB-AS1	1.26704747	0.043195971
CTU1	0.46241937	0.043272867
UBL7-AS1	0.46389416	0.043543338
DPY30	0.18937083	0.043548173
KIAA0930	0.19455509	0.043629855
PRPSAP1	0.26036062	0.04375859
LEMD3	0.41399639	0.043806868
CETP	0.96310649	0.043844927
FAM117A	0.54364092	0.043988564
CEP55	1.12032896	0.044028402
GATAD2B	0.25313657	0.044122078
ZNF776	0.87990182	0.044209974
HSPA2	0.71201458	0.044230813
ALDH1A1	0.44127872	0.044478231
TIMM17A	0.19085072	0.044780102
CTD-2260A17	0.95908189	0.04499464
SLC16A14	1.65702215	0.04514409
RP11-712L6.5	1.11983486	0.045665967
MRPL4	0.18824455	0.045680706
RP11-435I10.4	1.10799958	0.045816187
CECR1	0.2327966	0.046072333
NR2F6	0.75877644	0.046183107
LTBP3	0.38916248	0.046332193
SAAL1	0.36737507	0.046336019
MPP5	0.47267309	0.046336019
RP11-563J2.3	0.81713936	0.046390368
RP11-602N24	1.63829519	0.046453438
LINC00526	0.80105787	0.046593929

SSR4	0.10397093	0.04663047
TANK	0.23204601	0.046875685
NIPSNAP1	0.29716165	0.046958994
PMAIP1	0.47376022	0.047031053
GOLPH3L	0.53585317	0.047309839
CCDC137	0.32667041	0.047405302
TGFBI	0.22287659	0.04749923
TST	0.28990171	0.047568907
KNTC1	0.70641598	0.047621261
CAPNS1	0.18248531	0.047823038
POLR2G	0.16511233	0.047970271
AL161668.5	2.13420989	0.048311583
DDX26B	0.51430699	0.048320333
ACOT2	0.4225364	0.048603869
ZBED4	0.42626153	0.048637756
CYTH4	0.28193104	0.04864548
PIBF1	0.46719266	0.048721199
PTCD1	0.63775984	0.048810358
POLR1D	0.15992975	0.048854206
NUP37	0.30024483	0.048907711
RNF214	0.50560642	0.048984182
TRIB1	0.25464299	0.049104193
RP6-65G23.3	2.18145622	0.049416157
RP11-706O15	1.45053819	0.049491743
ZNF714	0.45243683	0.04976555
RP11-166P13.	0.6126474	0.049813451
RP4-569M23.:	2.65310111	0.049819936
RP11-218C14.	1.15222226	0.049872338
RP11-160H22.	1.45409484	0.049940099

	cDC3 vs. cDC1 + cDC2	
**gene**	**avg_logFC**	**adj. p-value**
CLEC4E	3.37649242	7.788E-168
HNMT	2.08756607	7.746E-141
CD302	1.39172897	1.656E-133
PID1	1.51750141	1.0798E-93
FCER1G	1.35219075	2.5957E-92
CD163	3.21895904	3.0929E-92
TLR2	2.32318739	2.9393E-84
C5AR1	3.77336555	2.0757E-82
APLP2	1.01790335	8.1713E-77
HCST	1.48124013	2.093E-74
TREM1	2.87026382	1.6774E-72
CD14	3.14767377	6.1219E-70
CSTA	1.96091798	2.4194E-69
OLR1	2.32023152	1.7809E-68
TNFSF13B	1.62460134	1.6971E-66
AGTRAP	2.52185247	3.0813E-64
CYBB	1.48599689	5.6501E-62
CD300E	3.28787275	1.0316E-59
EMP3	1.44419385	1.096E-59
CSF1R	1.89712663	8.5255E-59
ELL2	2.20060175	1.2292E-58
LAT2	1.26319122	3.0816E-58
FCN1	4.77708275	1.5575E-56
CPM	1.27093009	4.1205E-54
CTSS	1.12910142	3.1077E-53
FCGR1A	2.60161611	7.5482E-53
CAMK1	1.36310048	7.798E-53
VCAN	4.09374172	1.5753E-51
CDC42EP2	2.72940793	2.801E-50
THBS1	2.59783534	3.7599E-50
ITM2B	0.94129434	1.1144E-49
CXCL3	3.12168433	1.92E-49
VSIG4	2.13616683	1.92E-49
RNF13	0.99461021	5.302E-49
SLC7A7	2.01767957	8.526E-48
DMXL2	3.20385354	1.0925E-47
C3AR1	2.51930022	1.2739E-47
MMP19	3.29486518	3.7274E-47
ASAH1	0.88684101	5.915E-47
FCGR2A	1.86903092	7.4586E-47
LILRB2	2.1715572	7.4586E-47
PRNP	1.086602	5.957E-46

LILRB4	2.72580038	6.7001E-46
CD99	1.64248796	1.7262E-45
LYPD3	2.06866336	2.9587E-45
GFRA2	3.31189848	9.449E-45
CD209	2.9126422	1.4017E-44
ANXA2	1.00627661	3.4888E-44
IRAK3	1.70288299	2.2041E-43
TSPO	2.25283916	4.7569E-43
MS4A7	1.39443514	5.5246E-43
GLUL	1.48679463	7.0746E-43
CACNA2D4	2.28166974	7.5935E-42
CD44	1.45823743	5.3634E-41
CSF3R	1.18169424	2.3914E-40
ACSL1	1.65058176	2.7257E-39
IL1RN	3.15229411	4.7042E-39
NAIP	1.54367317	1.4704E-38
RNF130	1.00861246	1.4972E-38
SOD2	1.71744955	5.0818E-38
LILRB3	1.40805493	5.8728E-38
S100A9	4.38332291	6.528E-38
NUP214	1.63313178	2.3553E-37
PNRC1	0.90041782	3.1682E-37
CTSB	1.16054416	4.8056E-37
ASGR1	2.36127215	5.3474E-37
TMEM173	1.75951388	6.537E-37
RCSD1	1.16842628	1.0029E-36
NPC2	0.84838648	1.2913E-36
LMO2	0.97683693	3.3648E-36
HBEGF	1.87556848	4.7752E-36
SIRPA	1.44343398	5.9525E-36
EREG	2.49891197	1.8908E-35
LRP1	2.62555976	2.914E-35
PEPD	1.05167311	3.7529E-35
EPB41L3	1.68664998	4.0888E-35
STX11	0.86042588	4.3103E-35
TLR4	2.30645135	4.9724E-35
SAMSN1	1.15915475	4.9724E-35
SLC35E3	1.3674587	5.4686E-35
DPYD	1.52439779	8.0631E-35
PYGL	2.3002524	7.491E-34
YBX3	0.9917802	9.6623E-34
ZEB2	1.53607594	1.223E-33
SERPINA1	2.9348937	1.3246E-33
NUCB2	1.22710095	3.7245E-33
TYROBP	1.05736648	4.2494E-33
CLEC10A	1.1248505	8.2385E-33

CLEC12A	2.18322144	1.2745E-32
DPEP2	1.41124731	2.6105E-32
INSR	1.09366075	3.1367E-32
S100A11	0.71965513	8.7029E-32
ATP13A3	1.26229258	1.0903E-31
GIMAP4	2.24135171	1.1111E-31
MSRB2	1.36484381	7.9174E-31
UBXN11	1.90714445	9.3345E-31
GPR84	2.71261133	9.8369E-31
S100A4	2.03200738	1.1426E-30
PLXDC2	1.12193721	1.5984E-30
SCARB2	1.50963035	1.9164E-30
RP11-76E17.3	1.26405873	3.3544E-30
TFEC	2.52861846	3.7612E-30
S100A8	3.87307647	8.6539E-30
EMILIN2	0.60542485	2.0383E-29
LAMP1	1.5828509	2.388E-29
TMEM170B	1.48655007	4.6324E-29
KLF7	1.18503192	6.3016E-29
PLIN2	1.30880117	6.4842E-29
TMBIM4	0.52405729	6.8642E-29
AOAH	1.04085688	9.7353E-29
CXCL2	2.23301099	1.5615E-28
ZNF706	0.73430329	2.0403E-28
ARHGAP26	1.70040867	3.1117E-28
XBP1	0.88021794	4.0809E-28
RTN1	1.5439489	8.2916E-28
FAM198B	2.16582417	9.4306E-28
TNFRSF1B	0.88293949	9.5281E-28
CRTAP	1.12399782	1.5E-27
MARCKS	1.35305288	1.9516E-27
C5AR2	2.74803357	3.0298E-27
ADGRE2	1.29932726	3.445E-27
FTH1	0.67770159	3.6195E-27
SSH2	1.04389631	6.7423E-27
BLVRB	0.95073115	7.4877E-27
BNIP3L	0.83128661	1.2637E-26
PILRA	1.31914904	2.2252E-26
MDM2	1.09428745	3.4201E-26
B3GNT5	1.26644991	4.7167E-26
LCP2	1.12605516	6.3265E-26
FPR3	1.76158492	1.2034E-25
NCF4	0.92989982	1.8605E-25
DRAM2	0.70904534	1.9248E-25
GLIPR2	0.82691284	1.9998E-25
MAFB	2.14579421	2.084E-25

KCNE3	2.5218169	2.5935E-25
LDLR	1.65799202	3.916E-25
AP001055.6	1.61538909	5.3821E-25
MT2A	1.78183387	8.5926E-25
SQRDL	0.87885407	1.0729E-24
LACTB	0.71927763	1.1493E-24
HMGB2	1.0279895	1.1983E-24
AIF1	0.50132365	1.3915E-24
APOBEC3A	3.26098443	1.8873E-24
NFE2L2	0.57095747	2.403E-24
S100A6	1.34221097	2.5309E-24
TKT	0.65606166	4.839E-24
OSBPL1A	2.28652995	4.839E-24
PAG1	1.127886	6.1167E-24
IFI44	1.78331941	6.6603E-24
NBPF19	1.2106913	8.6034E-24
LILRB1	1.44993969	1.0369E-23
LHFP	2.50914681	1.0558E-23
STAB1	2.37731289	1.4519E-23
MTSS1	2.41723909	1.559E-23
FRMD4B	0.76987958	1.7117E-23
MPHOSPH6	1.22871089	1.8761E-23
RAB31	0.72917608	2.1977E-23
SAP30	1.99074821	2.9617E-23
NRG1	4.87618019	3.5564E-23
RHOU	2.62554263	3.5854E-23
TESC	1.63667575	3.9726E-23
ATF6	1.04173024	4.0112E-23
RIN2	1.49424938	4.0112E-23
FCGR1B	2.17066688	4.1837E-23
CATSPER1	2.62730239	4.1837E-23
RHBDF2	1.16462406	4.2711E-23
ARRDC3	1.77988687	5.2277E-23
ITGAM	2.07087081	5.4459E-23
GLRX	0.98415842	5.9023E-23
TMEM205	1.04964189	1.1257E-22
C1QA	2.56113398	1.2401E-22
RGL1	1.97835173	1.3344E-22
PRKAG2-AS1	1.47396947	1.3344E-22
AZI2	1.30736636	1.5898E-22
MS4A6A	0.83866975	1.802E-22
SRGN	0.68730093	1.802E-22
MTMR11	3.4349798	2.1135E-22
FGD4	1.05766246	2.6238E-22
GIMAP1	2.55281801	2.7363E-22
CAPG	1.37608756	2.7809E-22

PLAUR	1.30507085	3.1583E-22
ALDH3B1	2.03970705	3.3947E-22
CLEC4D	4.12697421	4.195E-22
QKI	0.71773623	4.2744E-22
MS4A4A	1.8281805	5.0701E-22
GNS	1.07307645	6.0302E-22
RBMS1	0.86808276	7.7002E-22
PIGA	1.22511329	8.4752E-22
VAPA	0.55009783	1.9736E-21
FYB	0.88823329	2.0745E-21
CCRL2	1.68968676	2.128E-21
BST1	2.92547302	2.4632E-21
NDUFA12	0.58826978	2.6049E-21
SIGLEC9	1.78473501	2.8681E-21
NCR3LG1	1.67325518	3.2854E-21
TMEM71	1.6165583	4.4711E-21
ADK	1.09072054	4.5614E-21
SLC25A37	1.04668645	4.7721E-21
SLC11A1	3.13570881	5.3648E-21
GBGT1	1.92354599	8.0434E-21
ZBTB16	2.14913311	9.819E-21
FAM102B	0.84756863	1.0833E-20
PSEN1	0.79531207	1.098E-20
GBAS	0.6155	1.1518E-20
SASH1	2.85922705	1.1943E-20
TNFAIP3	1.09239449	1.4037E-20
PABPC4	0.50776908	1.449E-20
PDE4D	1.61951906	1.6083E-20
LEPROT	0.75748004	1.63E-20
CREBL2	0.90216156	2.0069E-20
CAST	0.68401136	2.0761E-20
SIGLEC1	2.52704651	2.1236E-20
HEXB	0.5882987	2.1393E-20
ELF1	0.6511421	3.0458E-20
MIR3945HG	2.32513977	3.3511E-20
ATP6V1B2	0.94796495	5.1938E-20
ANXA1	0.98653194	5.5147E-20
KMO	1.64278896	5.9718E-20
CD36	0.84143125	6.2897E-20
SECTM1	1.09698297	1.1443E-19
GSTO1	0.54066412	1.1825E-19
ZFAND5	1.04585516	1.4693E-19
C1QB	2.90407072	1.474E-19
CASP1	1.15452179	2.1263E-19
VAMP3	0.65980448	2.3234E-19
SSR3	0.39557839	3.9964E-19

CALM2	0.42301645	4.0147E-19
SH3PXD2B	1.48733329	5.5365E-19
ATP6AP2	0.67559506	5.7038E-19
IL13RA1	0.88204885	6.6384E-19
CFD	3.23405456	6.8779E-19
CTSA	1.00614988	8.5893E-19
IGSF6	0.80028881	9.3644E-19
UBALD2	1.00425323	9.5E-19
P2RX7	1.69720553	1.1351E-18
SLC36A4	1.17901948	1.1506E-18
C1QC	2.97866777	1.5458E-18
SDCBP	0.83771181	1.7343E-18
RNASE2	4.47879175	1.7356E-18
SIGLEC14	1.79919127	1.7356E-18
TMCC3	1.29958409	1.8909E-18
IL10	3.04722732	2.0812E-18
GBP2	1.21064949	2.3296E-18
STXBP2	0.57189753	2.4405E-18
HCAR3	1.99223561	2.5468E-18
SLC43A3	1.47370455	2.7699E-18
FOXO3	0.77505646	4.3397E-18
SPTLC2	1.00047049	4.5657E-18
FCGRT	0.66772291	4.6949E-18
TGFBI	0.76913993	5.8948E-18
BTG1	0.79150798	6.4893E-18
RAB13	1.63094375	7.1167E-18
CPED1	1.61161099	8.0538E-18
ACSL3	0.73421027	8.2165E-18
PLK2	1.06778826	8.599E-18
NDFIP1	0.51565085	1.01E-17
VEGFA	0.92589011	1.01E-17
LILRA5	1.40216989	1.0195E-17
EMP2	2.04816413	1.8362E-17
IRS2	1.00609422	1.9966E-17
KIF13B	1.71817677	2.0466E-17
TWISTNB	0.92471515	2.1266E-17
AGTPBP1	0.89918026	2.1641E-17
SH3KBP1	0.62342511	2.2102E-17
CLEC4G	2.33579463	2.357E-17
GNAQ	0.62103463	2.4113E-17
CCL20	3.93831289	2.5126E-17
TSPAN4	1.4743903	2.6247E-17
HSP90B1	0.49180314	2.8403E-17
SLC15A3	0.99309976	3.0165E-17
TMX4	0.88984022	3.1932E-17
KCNN4	2.74959071	3.4403E-17

P2RX4	1.13445527	3.5221E-17
CXCL1	3.24930555	3.6723E-17
SOAT1	0.80235291	4.0489E-17
BCAT1	1.51919026	4.4117E-17
ARL8B	0.85068427	7.0052E-17
PMP22	1.47980504	7.2992E-17
PPP1CB	0.41015787	7.4629E-17
KCTD12	1.10124228	9.5788E-17
PRKAG2	0.9524521	9.7018E-17
S100A12	4.14710696	1.0162E-16
RAC1	0.34487119	1.1489E-16
IL1B	2.15660792	1.362E-16
DNAAF1	3.19655498	1.4359E-16
MRC1	1.2980785	1.4622E-16
GCH1	1.60158122	2.0599E-16
THAP2	1.43963075	2.2342E-16
LRPAP1	0.7172844	2.8447E-16
SDSL	0.97872897	2.8698E-16
ARMCX1	1.33107703	2.8713E-16
LY96	0.5854515	3.0127E-16
ZEB2-AS1	2.15625657	3.022E-16
SERP1	0.37230011	3.3266E-16
MARCO	5.24440984	3.3504E-16
SAMD4A	2.55117642	3.3631E-16
LILRA2	1.25524784	3.533E-16
SLC43A2	0.96020168	4.3076E-16
NANS	0.54198289	4.9329E-16
LIPA	1.03910007	6.1069E-16
LYSMD2	0.96024781	6.6039E-16
NFKBIA	1.39677721	6.667E-16
ZC3H12A	0.96013104	6.9715E-16
LINC00998	0.73352106	7.2015E-16
GLO1	0.63251632	7.2812E-16
S100A10	0.70347826	7.4941E-16
LIMS1	0.37614544	8.9152E-16
IER3	2.31009531	1.2316E-15
PCTP	1.32099669	1.2752E-15
MARVELD1	1.44451309	1.3293E-15
FES	0.69390374	1.3299E-15
GPBAR1	2.47340435	1.5386E-15
SH3BP5	0.73714615	1.6058E-15
FKBP5	0.9353788	1.8739E-15
FEZ2	0.62741522	2.0693E-15
SLC2A9	1.71701983	2.116E-15
GPX1	0.37936687	2.217E-15
ATF5	0.98781284	2.8218E-15

SLCO2B1	2.6691139	3.0042E-15
PTK2B	0.95149292	3.8036E-15
SEMA6B	1.03323288	4.6436E-15
AHR	0.70867711	5.0068E-15
LATS2	0.76613003	6.2322E-15
BLVRA	0.89509516	6.2388E-15
XAF1	1.24234501	6.4367E-15
ADAM17	0.56698849	6.5951E-15
YBX1	0.24577729	7.9898E-15
SLA	1.02305981	1.2025E-14
SNCA	1.52817833	1.2361E-14
SCPEP1	0.70818379	1.2368E-14
FAM111A	1.00392338	1.3062E-14
PPP2R3C	0.63025812	1.313E-14
A2M	1.3801086	1.3712E-14
ODF3B	0.67220578	1.3964E-14
SDHB	0.62478659	1.6719E-14
TOM1	1.13181846	1.7258E-14
DHRS4L2	0.65026956	1.8388E-14
SLC1A3	2.55618271	1.931E-14
TMEM236	2.07338326	1.939E-14
NRP1	1.69050213	1.9645E-14
RP11-472N13	1.64386554	1.966E-14
CD68	0.93452801	2.0452E-14
BRI3	0.40122507	2.178E-14
VAMP5	0.8979079	2.2482E-14
NBN	0.76552309	2.266E-14
HIPK3	0.7360637	2.3761E-14
TPPP3	1.85260461	2.438E-14
TCN2	1.84151066	2.438E-14
IL17RA	0.91255682	2.5841E-14
GPCPD1	0.81536906	2.6898E-14
SDC3	2.96995266	2.9343E-14
CKAP4	2.73129845	3.528E-14
CREG1	0.74658372	3.6412E-14
CD2	2.44953564	3.9212E-14
NCF2	0.88344465	4.0767E-14
NHSL1	1.47147287	4.3417E-14
GIMAP8	2.33230188	4.5361E-14
EIF1	0.25921979	5.123E-14
LTA4H	0.6493678	5.3521E-14
NRGN	2.14040409	5.7376E-14
WBP1L	1.20890506	5.907E-14
GIMAP2	1.39055065	6.4034E-14
SGMS2	1.4132773	6.4345E-14
CXCL11	4.37811672	6.9246E-14

CES1	3.93337409	7.4342E-14
TBC1D2B	1.37120938	8.3631E-14
MEGF9	1.42471589	8.5344E-14
TIAM1	1.21638197	1.0496E-13
FNDC3B	1.17043003	1.2052E-13
MT1F	2.25603233	1.3084E-13
RILPL2	0.55382972	1.3202E-13
CASP4	0.77670948	1.4227E-13
PPP1R15B	0.81870206	1.4399E-13
SESTD1	1.02824891	1.4674E-13
TNFRSF21	1.0351755	1.673E-13
B4GALT1	0.55807095	1.706E-13
PLTP	1.55476569	1.7568E-13
SOCS3	0.82368941	1.9116E-13
CAPN2	1.34623765	2.7713E-13
HES1	1.64380326	2.9854E-13
CCL4	1.90256297	3.2662E-13
TMEM106A	1.04415014	3.2701E-13
CD1D	0.62540808	3.4816E-13
CMKLR1	2.48732281	3.5318E-13
CH25H	1.80578775	3.7416E-13
MORF4L1	0.2941972	3.8127E-13
RAB20	0.81363743	4.0881E-13
DERA	0.73542812	4.1614E-13
MPP1	0.96280975	4.3398E-13
EAF2	0.88879781	5.0382E-13
SLC40A1	2.25378173	5.2718E-13
RP11-670E13.	1.74568192	5.4517E-13
SBF2	0.8529635	6.2773E-13
MITF	2.29220439	6.5208E-13
PDXK	0.83672915	6.7334E-13
NAMPT	0.61744935	6.8636E-13
EMP1	1.78585589	7.2303E-13
TLR5	1.80991668	7.2917E-13
PTGIR	2.31520392	7.8483E-13
IL3RA	1.31462612	7.9586E-13
RXRA	1.22873008	9.218E-13
MID1IP1	0.95531071	9.5451E-13
MEFV	1.40118719	1.0404E-12
MRPL15	0.55530068	1.0999E-12
NLRP3	1.18178753	1.1544E-12
ANKRD22	2.83239658	1.1717E-12
SPHK1	1.48851528	1.2539E-12
CCL4L2	1.61428581	1.2809E-12
GPRC5A	2.23882954	1.411E-12
CTB-61M7.2	4.72907442	1.5164E-12

CD9	1.66276153	1.5324E-12
CYBRD1	1.11877	1.5443E-12
TRMT1	0.79104429	1.7175E-12
ENOSF1	0.98111305	2.1832E-12
MAP2K3	0.88706629	2.6376E-12
C10orf11	1.00177085	2.7446E-12
ANKS1A	0.8763774	2.78E-12
RAPH1	1.8327123	2.818E-12
MXD1	0.86712658	3.1561E-12
C9orf72	0.63351632	3.5347E-12
FCGR3A	2.90345682	3.602E-12
TBXAS1	0.56774761	4.0097E-12
SELK	0.38429559	4.0196E-12
NFIL3	0.58293586	4.1311E-12
PLAU	1.4550968	4.1395E-12
FOSL2	0.6099209	4.1831E-12
TMEM120A	0.99659745	4.9035E-12
MGAT4A	0.80252327	5.051E-12
MAP3K3	1.09136675	5.1953E-12
LTC4S	1.40125087	5.3303E-12
SERPING1	1.6363122	5.7476E-12
AC109826.1	1.85396742	6.2876E-12
GATM	0.99674648	6.3046E-12
DTNA	2.98361818	6.4507E-12
GBP1	1.26251421	6.6896E-12
LUCAT1	2.71318913	6.9037E-12
PARP14	0.57812007	7.0011E-12
DGUOK-AS1	3.27031357	7.2285E-12
IQGAP1	0.35710916	8.4832E-12
SLFN11	1.06793257	8.5827E-12
FCER2	3.15529451	8.6001E-12
F13A1	2.39927242	8.7903E-12
BORCS7	0.63442614	8.8992E-12
ZNF267	0.6728351	8.9207E-12
RHOBTB3	2.1190264	9.1457E-12
FIS1	0.49399639	1.1292E-11
SDC4	2.23888557	1.1292E-11
RHOA	0.28416547	1.155E-11
SLC16A6	1.78267208	1.1631E-11
GABARAPL1	0.57154143	1.168E-11
PRKACA	0.68854752	1.2862E-11
RP1-239B22.5	1.78837314	1.5568E-11
NKG7	1.48265715	1.7567E-11
UBE2D1	0.61897711	1.7602E-11
IL10RB	0.56406268	2.3056E-11
GBP3	1.11274828	2.381E-11

CD300C	1.13954896	2.4311E-11
TNFAIP6	2.85350697	2.6339E-11
CXCL10	3.19665078	2.6339E-11
SRGAP1	1.11301907	2.7064E-11
HBP1	0.64310067	2.8039E-11
CTB-55O6.12	1.56146315	2.9391E-11
SORL1	0.84969924	3.1447E-11
KYNU	1.32003942	3.5392E-11
ARSG	1.01146004	3.5916E-11
ETHE1	0.61072703	3.6218E-11
AHNAK	0.76410214	4.1131E-11
LAPTM5	0.3641744	4.2802E-11
HSD17B11	0.50726597	4.3905E-11
ABHD12	0.59091343	4.7508E-11
APOBEC3C	0.75288519	4.8247E-11
CRYL1	0.76601015	5.8846E-11
SLC11A2	0.98400003	6.0596E-11
SLC22A18	0.86600661	6.7025E-11
ABL2	1.11184145	7.1237E-11
PRDM1	0.982084	7.3104E-11
USP3	0.67536005	7.914E-11
FAM45A	0.68025227	8.4287E-11
CCL5	1.99225473	9.618E-11
FLVCR2	1.44342903	9.892E-11
LGALS8	0.63117771	9.9517E-11
ADAMTSL4	1.17329107	1.0207E-10
TMIGD3	1.16094861	1.0381E-10
IL4I1	1.21856079	1.1883E-10
CEBPB	0.67432245	1.3861E-10
MGAT1	0.45628786	1.4533E-10
ANXA5	0.49852895	1.5351E-10
ELF2	0.54373256	1.5897E-10
CD33	0.70563953	1.6135E-10
FTL	0.67617563	1.7305E-10
RNASE4	2.07271989	1.7643E-10
CAPZA2	0.45791756	1.8839E-10
FXYD5	0.44468257	1.9013E-10
IL10RA	0.66322319	1.9177E-10
APH1B	0.76514271	1.953E-10
WBSCR27	2.23248218	2.1085E-10
HSBP1	0.38975919	2.1457E-10
SLC16A10	1.23322123	2.7526E-10
EVI5	0.91136868	2.7585E-10
EPSTI1	0.65972718	2.7782E-10
TSPAN14	0.77746585	2.7795E-10
ASB9	3.62031855	2.942E-10

SORT1	0.9690908	3.153E-10
LINC01451	1.8807654	3.1607E-10
ITGAX	0.44341183	3.1607E-10
ATP11A	0.72339713	3.1882E-10
CTSL	1.64201264	3.2453E-10
FMN1	0.97510366	3.2913E-10
MT1E	2.23600964	3.2925E-10
SMIM4	0.62087859	3.3688E-10
CD53	0.39582942	3.4039E-10
ATP6V1C1	0.64247477	3.4633E-10
PLD3	1.31754959	3.6679E-10
METTL7A	0.786654	3.7075E-10
NRP2	1.56112992	3.8633E-10
PDE3B	1.05098416	3.9809E-10
CTSC	0.99559414	4.0003E-10
CARD16	0.85766382	4.4117E-10
FOSL1	1.15678517	4.5733E-10
MAF	1.59868108	4.8978E-10
PELI2	1.35556615	4.9169E-10
TTC7A	0.81471383	4.9825E-10
ADAP1	0.93179749	5.0387E-10
LAMP2	0.56598172	5.1179E-10
IFNAR2	0.55299788	5.2009E-10
PECAM1	0.81751023	5.2756E-10
IFI44L	1.71320246	5.5393E-10
RHOQ	0.4779131	5.6192E-10
LYZ	0.92111491	5.6753E-10
TCHH	1.9727666	5.9064E-10
C1orf228	3.59066182	6.0556E-10
VPS37C	1.0250284	6.0763E-10
ADGRE5	0.50258818	6.1263E-10
CSGALNACT1	1.1167767	6.1315E-10
FOLR2	1.74267998	6.2617E-10
SGMS1	1.14915284	6.3137E-10
PSAP	0.74754701	6.3302E-10
CCDC170	1.66686667	6.4115E-10
TET3	0.92886401	6.5691E-10
SFXN3	0.85552924	6.8505E-10
CTSD	1.17230626	7.1059E-10
PRDM8	1.41424257	7.1808E-10
EAF1	0.98570945	7.2933E-10
ARHGAP24	2.13149583	7.3438E-10
LINC00996	2.180979	7.5383E-10
SLC22A4	2.06658385	7.5593E-10
OAF	1.5735239	7.6372E-10
LINC01272	1.42482946	8.1241E-10

SLC39A1	0.59435019	8.4196E-10
ALOX5	0.74186254	8.6895E-10
ARL6IP1	0.40243867	8.8427E-10
STAT4	1.14981257	8.8559E-10
DYNLT3	0.60567453	9.0729E-10
PHF20L1	0.45179283	9.7606E-10
HERPUD1	0.45871608	1.0342E-09
NUP58	0.60276055	1.0473E-09
MIR3142HG	2.24618484	1.0492E-09
SHMT1	0.87753544	1.0776E-09
NFAM1	0.9957481	1.0787E-09
OAS1	1.4469866	1.1161E-09
FCAR	3.56060213	1.1161E-09
SCN1B	2.09957614	1.124E-09
ATP1B3	0.40495117	1.1347E-09
SLC9A3R1	0.94280353	1.1799E-09
PDGFB	1.18772475	1.2092E-09
FUCA2	0.49590938	1.2489E-09
MT1X	0.83309258	1.2489E-09
IKBKE	2.38532031	1.2868E-09
FBP1	0.68588909	1.3369E-09
ATP13A2	2.76407593	1.3388E-09
TSPAN32	1.89260026	1.3742E-09
AKR1B1	0.6047587	1.4001E-09
CMTM3	0.59344024	1.4004E-09
TNNT1	4.47786107	1.5622E-09
XYLT1	1.04345731	1.715E-09
CHPT1	0.71640463	1.8172E-09
GBP5	1.56163168	1.8742E-09
C2	2.51596215	1.9635E-09
RP11-108M9.4	0.66049165	2.222E-09
TMEM109	0.50916253	2.3392E-09
DUSP6	1.36334428	2.3407E-09
PTGER2	1.05736667	2.3676E-09
CANX	0.34455667	2.4506E-09
LMAN1	0.63776861	2.6119E-09
AZIN1	0.43994088	2.8199E-09
DIRC2	0.92773265	2.8767E-09
LRRC25	0.72270191	2.9778E-09
IVD	0.70074111	3.0067E-09
BLM	1.96391145	3.0067E-09
APP	0.92087038	3.0166E-09
BLNK	2.78630913	3.0308E-09
ARHGEF40	1.31612061	3.1434E-09
PLA2G7	1.68890635	3.4234E-09
TP53I3	0.84707482	3.4732E-09

TAGAP	0.68217257	3.6749E-09
RAB27A	1.02022112	3.7107E-09
ARL4A	0.80672469	3.8005E-09
DENND3	0.83055371	3.8234E-09
CD300LF	0.95064025	4.1767E-09
NFKBIZ	0.95480517	4.2028E-09
ATG7	0.78295335	4.2845E-09
FGL2	0.51738566	4.3702E-09
DGKA	0.85546571	4.4525E-09
RNF19B	1.0465344	4.6404E-09
FOXN3	0.47269763	4.7248E-09
NINJ2	1.00601715	4.7877E-09
MT-ND1	0.31474989	4.8907E-09
DENND6B	0.57977044	4.8911E-09
ALOX5AP	1.13564367	5.0027E-09
PTX3	1.53132232	5.033E-09
TIMP2	0.70430189	5.1668E-09
IFI27L2	0.5098836	5.3669E-09
CCDC149	2.44085818	5.7964E-09
ANKRD28	1.02477069	5.8106E-09
SCRG1	2.38153113	6.0019E-09
HPSE	2.68936032	6.1649E-09
C1orf162	0.49577464	6.1763E-09
TNFSF10	0.75207242	6.1763E-09
FAM3C	0.56863243	6.3431E-09
SFR1	1.20805243	6.3431E-09
HOMER3	2.20633166	6.3682E-09
SHB	1.10103786	6.3737E-09
ZCCHC6	0.63485334	6.4019E-09
SCAMP2	0.46457185	6.4501E-09
TCEAL4	0.35992562	6.5216E-09
FPR1	1.26219406	6.534E-09
GAS2L1	0.92274387	6.7317E-09
GIMAP7	1.59584456	6.7391E-09
PNP	0.88084833	7.0149E-09
CTNNB1	0.39760175	7.0353E-09
TIMP1	0.69343073	7.1474E-09
MEI1	2.22469567	7.2205E-09
SERPINB8	0.8566401	7.7421E-09
CHRAC1	0.58215758	7.8681E-09
GAA	0.70410136	7.9294E-09
NCF1	0.66479575	8.1408E-09
GRB2	0.33385738	8.5129E-09
MAEA	0.72257142	8.792E-09
LAIR1	0.78348499	9.7782E-09
RENBP	1.49781187	1.0226E-08

HK1	0.55430387	1.1098E-08
ABCA1	1.25314341	1.1166E-08
SPR	2.37824017	1.1368E-08
CCR2	2.10808609	1.1663E-08
STX10	0.55882511	1.1863E-08
WDR45B	0.54844502	1.188E-08
CHCHD10	0.55484739	1.2253E-08
IQSEC2	1.22230488	1.2475E-08
ARRDC2	0.70221679	1.2475E-08
ABHD17C	2.02201379	1.257E-08
MSR1	1.30069749	1.2626E-08
CASP10	1.13102726	1.3829E-08
USP16	0.38426842	1.4026E-08
CECR1	0.43884001	1.4093E-08
CCL3	1.72483585	1.4645E-08
TPT1	0.20720358	1.5165E-08
ALOX15B	3.49825784	1.5381E-08
VPS37B	0.9149457	1.5688E-08
P2RX1	2.05761926	1.6421E-08
RETN	2.45895434	1.7211E-08
CRISPLD2	2.5835856	1.7829E-08
SMPDL3A	2.89246769	1.7851E-08
PARVB	0.57875393	1.7974E-08
IL1RAP	0.90119463	1.8338E-08
CEBPD	0.41246684	1.8541E-08
OSM	1.06513522	1.8731E-08
NINJ1	0.94738359	1.9672E-08
MMP9	1.2258572	1.9672E-08
TRPM2	1.18932402	1.9672E-08
ACKR3	2.42782906	2.1525E-08
BTBD7	0.6654604	2.2215E-08
KIAA1033	0.71970823	2.2965E-08
SKI	0.99141355	2.3268E-08
IL27RA	1.03370088	2.3423E-08
GJB2	1.69098107	2.3736E-08
SLITRK4	4.01548552	2.4379E-08
CPNE8	0.75352928	2.5259E-08
CEBPA	1.14546684	2.6545E-08
IQGAP2	0.49435411	2.6848E-08
IL2RA	2.24751845	2.7275E-08
ZGLP1	1.09507149	2.8716E-08
CREB5	1.07285075	2.9228E-08
STARD13	2.09543199	2.9228E-08
ZP3	1.47068847	3.0044E-08
TMEM106B	1.83972707	3.0656E-08
NSRP1	0.36477719	3.1245E-08

PHLDB1	1.13530729	3.2158E-08
PFDN1	0.58254442	3.2693E-08
NCOR2	0.49938407	3.2863E-08
SIGLEC7	0.69676863	3.4572E-08
PPP3CA	0.52451676	3.517E-08
ANG	1.44994349	3.632E-08
ARHGEF10L	2.99630049	3.6921E-08
DLG4	1.53416985	3.7485E-08
SDHD	0.35770781	3.7845E-08
ZG16B	2.10738697	3.8406E-08
LILRA6	2.31065809	3.8553E-08
PLPPR2	2.4564122	3.8847E-08
TAF9	0.45135965	4.0632E-08
SNX10	0.58299351	4.3016E-08
DAB2	1.36154975	4.4391E-08
ITGAL	1.31546948	4.5423E-08
DHRS9	1.53224721	4.5949E-08
RASSF4	0.71390535	4.6902E-08
SH2B3	0.33708911	4.7554E-08
KCNE1	2.95544022	5.0021E-08
SFMBT2	0.64736222	5.0542E-08
CTTNBP2	1.58315897	5.2715E-08
ZNF281	0.92101025	5.2779E-08
CTSW	1.94210449	5.2872E-08
UBE2E1	0.44268054	5.4146E-08
ITPRIP	0.79963991	5.4469E-08
DOCK4	1.27560026	5.4964E-08
TLN2	2.01914353	5.7255E-08
DRAM1	0.86858549	5.8841E-08
RNF141	0.69099193	5.8906E-08
MCEMP1	1.31123758	5.9036E-08
CD63	0.4645653	6.0171E-08
PNPLA8	0.54792092	6.0586E-08
UQCRC1	0.35671722	6.1235E-08
PLEKHA1	0.86800639	6.4497E-08
WIPI1	0.94432663	6.5757E-08
KCNQ1	0.59657167	6.6718E-08
KCNAB2	0.73371026	6.9063E-08
ITGB5	1.3222806	6.9179E-08
PCBP1	0.26290112	7.0416E-08
VPS26A	0.38585667	7.9167E-08
ARSD	0.97582993	8.0055E-08
TICAM1	0.77482169	8.0133E-08
EPS8	1.61522802	8.9009E-08
ADAM10	0.48859743	9.0365E-08
TMEM255B	1.84262317	9.0761E-08

MANBA	0.39114105	9.5978E-08
RP11-79H23.3	1.91254144	9.8038E-08
ISG20	0.76764022	9.8633E-08
RDH11	0.68015957	9.8714E-08
HCAR2	1.40014409	9.9192E-08
ISCA1	0.44331226	9.9379E-08
CSGALNACT2	0.4190156	1.0319E-07
PDE4B	0.41959354	1.0366E-07
ARFGAP3	0.44706242	1.0639E-07
ICAM2	0.58827334	1.0759E-07
CCS	0.48079407	1.1104E-07
SDPR	0.99669013	1.1197E-07
BIN2	0.59303564	1.1241E-07
KIF1B	0.63569805	1.2218E-07
CYFIP1	0.5173684	1.2352E-07
CD93	0.56001478	1.2471E-07
BCAS2	0.5041522	1.2503E-07
MCTP1	0.553209	1.2561E-07
C10orf54	0.29272746	1.3118E-07
TNFAIP2	0.89804747	1.3284E-07
LGALS3	0.80681735	1.3345E-07
ICAM1	0.89110929	1.3647E-07
ADGRE1	1.71484089	1.3858E-07
TMOD2	1.83521988	1.4901E-07
LINC01503	1.75479899	1.5088E-07
CCL3L3	1.90625733	1.5224E-07
RHCG	3.08916289	1.5466E-07
ACVRL1	1.01467561	1.5507E-07
IFITM3	0.64465057	1.5596E-07
SFXN4	0.62918006	1.5596E-07
CYBA	0.24735535	1.6241E-07
MAML3	0.62275045	1.6413E-07
TMEM220	1.12106054	1.6413E-07
SEPP1	2.30154252	1.6988E-07
RAB3D	2.60006401	1.7436E-07
CD163L1	1.92652592	1.7697E-07
NAPRT	0.50587216	1.8628E-07
GPR35	1.10471646	1.8736E-07
KIAA0513	0.8830288	1.8876E-07
KLF9	0.48194244	1.9548E-07
MN1	2.82725568	1.9903E-07
AFMID	0.56721916	1.9924E-07
RASGRP2	0.6778581	2.0135E-07
TMEM59	0.3344245	2.016E-07
CD37	0.45701529	2.0687E-07
SLC19A2	0.66994193	2.0924E-07

FABP5	0.2646782	2.1014E-07
PRDX3	0.36808565	2.2578E-07
GRINA	0.68757257	2.3166E-07
PRKCA	0.66713169	2.3166E-07
IFITM2	0.577476	2.4418E-07
CCR5	1.12751258	2.4828E-07
HK3	2.96644981	2.5516E-07
KRT10	0.4303262	2.5626E-07
NOD2	1.20672635	2.6201E-07
SETD3	0.50111263	2.6631E-07
LPCAT2	0.56382008	2.6862E-07
DOK2	0.60648983	2.7085E-07
PYM1	0.55526016	2.8124E-07
KCNMA1	1.82532248	2.8264E-07
CHST13	3.37288304	2.9101E-07
UACA	2.02847097	2.9374E-07
FAM195A	0.71309001	3.021E-07
STS	1.85595809	3.1196E-07
ITGAV	0.94900986	3.1587E-07
GIMAP5	2.60223275	3.1587E-07
UTRN	0.5527112	3.2285E-07
GPR132	0.64395104	3.3259E-07
RAB1A	0.35385222	3.3736E-07
RP11-11N9.4	0.67431259	3.4667E-07
ADM	0.9634755	3.475E-07
FER	1.0920382	3.5262E-07
PRKCD	0.64563567	3.5402E-07
FAM21C	0.83105345	3.8504E-07
MKKS	0.3550404	3.8504E-07
VENTX	1.34229775	3.9131E-07
EPHB2	2.82145761	4.0842E-07
PTGS2	1.06746999	4.2088E-07
MCF2L2	1.51666762	4.2589E-07
TRAF3IP3	1.00252081	4.2885E-07
ACOX2	3.90314072	4.3516E-07
RASGEF1B	0.66778185	4.3997E-07
DLGAP3	1.36678011	4.8063E-07
TNFSF12	0.68871795	4.9626E-07
NFKB2	0.58664342	5.1302E-07
CCDC109B	0.66807364	5.429E-07
MAGT1	0.50156954	5.6109E-07
ENG	0.77095167	6.246E-07
LILRB5	2.22983583	6.5191E-07
TREM2	2.33352935	6.668E-07
TMEM150B	2.85515207	6.8429E-07
SNRK	0.68137767	7.4638E-07

LHFPL2	1.67933873	7.4878E-07
MAML2	1.23651394	7.5171E-07
RAB35	0.64649667	7.5962E-07
HIBADH	0.73684203	7.6141E-07
SORBS3	0.82675817	7.766E-07
TOP1	0.34910076	8.0216E-07
HM13	0.28099619	8.0267E-07
RAP1B	0.44710424	8.1366E-07
JUP	1.18696314	8.3624E-07
AKIRIN2	0.57238658	8.5507E-07
LTB4R	0.73935973	8.7049E-07
HVCN1	0.65734825	8.7372E-07
TGIF1	0.38888336	8.9451E-07
KLF2	1.61640838	9.0458E-07
CNPY3	0.29073422	9.0708E-07
LMNA	0.41077308	9.3473E-07
MFSD1	0.43487045	9.6616E-07
TMEM167A	0.33549738	9.6779E-07
IGFBP7	0.94975192	1.0043E-06
TMEM53	1.26847806	1.0353E-06
SSBP2	0.89293151	1.044E-06
ETS2	0.5046062	1.0519E-06
SAT2	0.35878416	1.0601E-06
MXI1	1.08035457	1.086E-06
MIR22HG	0.59959921	1.0951E-06
THEMIS2	0.4328005	1.1011E-06
PRKCB	0.74163118	1.1031E-06
FGR	0.5427928	1.1621E-06
MED14OS	0.93150844	1.1621E-06
UBXN2B	0.78749035	1.1757E-06
AQP9	2.79420449	1.206E-06
MNDA	0.68616883	1.2065E-06
TPRA1	0.80300834	1.2085E-06
RASA3	1.35092856	1.3139E-06
CYCS	0.28484234	1.3213E-06
CDKN1A	0.52867174	1.3604E-06
CTNNA1	0.35622352	1.4181E-06
AGPAT4	0.75503533	1.4439E-06
PINK1	0.83932883	1.4466E-06
FUNDC2	0.30353492	1.4495E-06
CA11	0.75236467	1.462E-06
NISCH	0.73141159	1.4673E-06
BNIP2	0.31873558	1.4673E-06
CHD1	0.40219595	1.4811E-06
MERTK	2.0653806	1.5536E-06
WWC2	1.62827656	1.5838E-06

CLEC11A	0.78187571	1.5955E-06
FURIN	0.99681113	1.6208E-06
CD300LB	0.68499826	1.6461E-06
ADPRH	0.7990609	1.6558E-06
CD276	1.84084822	1.6687E-06
RNF213	0.49606389	1.7016E-06
HELZ2	1.18594534	1.7273E-06
NQO2	0.73979604	1.7342E-06
CST6	2.66301507	1.8014E-06
PLB1	0.76156757	1.8049E-06
SLC46A2	2.07132898	1.867E-06
TMED10	0.2888695	1.9186E-06
TGFB1	0.46516811	1.9406E-06
DEPTOR	1.34249919	1.945E-06
CCR1	0.76574175	1.9922E-06
FAS	1.22528847	2.0032E-06
APOL6	0.9506804	2.0074E-06
CCNH	0.48398098	2.0128E-06
MANF	0.35089439	2.022E-06
RP5-1136G13	1.34219357	2.1053E-06
ENPP4	1.35780902	2.1626E-06
USP36	0.35031166	2.1639E-06
SP3	0.42598621	2.1831E-06
CD4	0.34091124	2.2251E-06
SNX24	1.31704214	2.4234E-06
KDM6B	0.44338096	2.4278E-06
TOMM40L	0.97981053	2.4611E-06
DHX34	1.18428802	2.4948E-06
RRAGD	0.99775954	2.5258E-06
SLC30A7	0.63341966	2.5372E-06
TXNDC15	0.46118948	2.5956E-06
RCN1	0.56304829	2.5967E-06
GPNMB	1.86551384	2.6073E-06
IL15RA	1.3697954	2.6155E-06
HGF	3.05582478	2.6305E-06
NT5DC2	1.25000896	2.6337E-06
KCNJ2	1.8769112	2.6337E-06
CDH23	1.1295327	2.6381E-06
LYRM5	0.5918674	2.6509E-06
SNX27	0.54816792	2.6637E-06
IFNGR2	0.6193298	2.6692E-06
TMEM33	0.46765645	2.7053E-06
RP11-22N19.2	0.82211463	2.7157E-06
FAM13A	1.05302492	2.864E-06
TPCN1	0.87700499	2.902E-06
ITPR2	0.55928918	2.905E-06

IL4R	0.9226079	2.9967E-06
PDK4	1.30970196	3.0154E-06
ECHDC3	1.32562609	3.0798E-06
SIDT2	0.6826934	3.1249E-06
DIP2B	0.73839297	3.1249E-06
CTD-2313J17.	1.62692066	3.1723E-06
IFNAR1	0.34933182	3.2047E-06
STAT1	0.65046873	3.2386E-06
ELK3	0.91957685	3.3037E-06
SIGLEC11	2.46810778	3.3312E-06
C14orf119	0.47659832	3.4294E-06
USP15	0.40578339	3.4788E-06
MYO1G	0.73499595	3.511E-06
ARHGAP4	0.35628177	3.5168E-06
SH3BGRL	0.29193289	3.534E-06
CTSF	1.60405226	3.5468E-06
HELB	0.93193512	3.6303E-06
OASL	1.15747685	3.644E-06
ZDHHC7	0.66379245	3.6965E-06
STARD8	0.6332768	3.7597E-06
ZYG11B	0.90625584	3.783E-06
XKR8	0.71759024	3.8583E-06
KBTBD11	1.26686601	3.9302E-06
CCDC159	0.71331433	4.1429E-06
ARRB2	0.34396076	4.2418E-06
GIMAP6	1.61701259	4.2597E-06
RB1CC1	0.45549967	4.2992E-06
CD164	0.24644251	4.424E-06
TMEM217	1.39133237	4.7897E-06
ZDHHC1	1.00466332	4.8216E-06
RP11-404O13	0.96878225	4.8388E-06
RP11-473M20	1.001527	4.8583E-06
JAML	0.45602845	4.8884E-06
NDUFB9	0.3091234	4.9267E-06
PLCB1	2.62775774	4.9743E-06
GLRX2	0.43751394	5.0668E-06
MPZL2	0.94416281	5.1043E-06
SPPL2A	0.39665126	5.4677E-06
RHOB	1.08723684	5.5744E-06
ZBTB43	0.50474049	5.959E-06
IL1A	1.53689925	5.9948E-06
NOP10	0.31341335	6.0567E-06
ARHGEF6	0.37843407	6.1077E-06
SLC27A3	0.75134929	6.1305E-06
SLC45A3	3.76318015	6.154E-06
SDF2L1	0.31221728	6.2551E-06

ADRB2	1.32582646	6.2744E-06
CALU	0.50855444	6.2881E-06
LINC00884	1.21884311	6.3121E-06
ROBO3	0.98702282	6.4315E-06
DNMBP	0.74479393	6.575E-06
TMEM179B	0.27308523	6.6564E-06
TGFA	1.98057167	6.7424E-06
MCOLN1	0.75204935	7.0557E-06
MT-ND3	0.28369248	7.1896E-06
OAS2	0.85349837	7.2395E-06
WASF2	0.35032408	7.245E-06
SAR1B	0.39660002	8.0079E-06
RP11-325F22.	1.1388485	8.1896E-06
MTHFD2L	0.55557227	8.2414E-06
C11orf24	0.63553609	8.2414E-06
ARL5B	0.6400941	8.3719E-06
ARHGEF11	1.62712537	8.3854E-06
PVT1	1.66600194	8.5515E-06
PLD1	1.93539288	8.5801E-06
TTYH3	0.60334719	8.636E-06
RAE1	0.39905992	8.6584E-06
ARHGAP18	0.44966881	8.7053E-06
PDIA6	0.31380835	8.7973E-06
DCLRE1B	1.0012673	9.1594E-06
DNAJC14	1.02946232	9.1643E-06
TNFRSF8	2.0207739	9.2919E-06
RP11-796E2.4	1.10035636	9.2919E-06
LINC01315	1.32057577	9.3784E-06
DNMBP-AS1	2.0398988	9.4298E-06
MFSD7	0.89897157	9.5373E-06
SLIRP	0.31543436	9.6394E-06
SLC3A2	0.48243717	9.8786E-06
CRBN	0.53560683	1.0233E-05
PLCXD1	0.7456382	1.0284E-05
GADD45B	0.70922384	1.0643E-05
SLC2A4RG	0.97560669	1.0678E-05
TNFRSF1A	0.52858352	1.0742E-05
TFRC	1.29622258	1.1489E-05
SMAP2	0.38278465	1.167E-05
RNFT1	0.69236334	1.1954E-05
UFM1	0.3324207	1.2446E-05
TNFRSF9	1.70270771	1.2601E-05
TAF13	0.45885743	1.2887E-05
KIF13A	0.90121633	1.2887E-05
WBP4	0.5889579	1.3096E-05
RP11-798M1S	1.36534796	1.3313E-05

SEMA4A	0.65421763	1.333E-05
DDIT4	0.4113173	1.3358E-05
SIRPB1	1.01731311	1.3606E-05
COX7B	0.26139773	1.3856E-05
PEA15	0.57386947	1.3875E-05
SNTB1	2.07496693	1.3897E-05
GPX4	0.30038016	1.4058E-05
NR1H3	1.58972861	1.4406E-05
MAPK7	1.13877028	1.441E-05
ANKH	0.56466681	1.4487E-05
GNAI3	0.26769975	1.5335E-05
RB1	0.5263667	1.5428E-05
TCF7L2	0.86674841	1.5959E-05
TNFRSF10D	1.30656519	1.5977E-05
SRPK2	0.46451805	1.6054E-05
TNIP3	1.95244629	1.6703E-05
FAM120A	0.27084369	1.6804E-05
KDELR1	0.25698805	1.6987E-05
OSCAR	0.70464479	1.7297E-05
IL10RB-AS1	0.63195011	1.734E-05
SLFN5	1.18217491	1.7427E-05
HSPBAP1	0.54706396	1.7467E-05
FCGR2B	0.96239255	1.7507E-05
SLC7A8	1.70957608	1.7644E-05
SLC7A5	0.71050882	1.7896E-05
IL6	1.96650105	1.8221E-05
JDP2	0.74018689	1.8221E-05
ANO6	0.50429478	1.8243E-05
YWHAE	0.2256101	1.8638E-05
RAB18	0.36278957	1.9542E-05
CFH	2.03638615	1.9584E-05
NUDT16	0.44822664	1.9682E-05
TMEM39B	0.70392768	1.9748E-05
HSPA5	0.36386313	2.0417E-05
EPOR	1.14742302	2.0483E-05
PCNX	0.74645575	2.0573E-05
FXYD6	1.7804291	2.0717E-05
LINC00346	2.93442506	2.0735E-05
CYP20A1	0.41800064	2.089E-05
FAM129B	1.0263154	2.1136E-05
WTAP	0.3961462	2.1149E-05
UBAC2	0.43547674	2.1597E-05
FCHO2	1.34713488	2.2623E-05
PHYH	0.88896532	2.3251E-05
TMEM50A	0.28318874	2.3301E-05
LAMB2	1.91258052	2.3728E-05

SNX2	0.4537246	2.429E-05
CXCL8	1.42986004	2.5517E-05
IL18BP	0.57136212	2.6154E-05
RRN3	0.5778985	2.6447E-05
CTB-58E17.1	1.01709598	2.7251E-05
CNIH4	0.4177257	2.726E-05
C16orf74	2.08358491	2.7374E-05
CLEC4A	0.42816014	2.7553E-05
RAB12	0.76888942	2.8338E-05
RP11-69E11.4	1.06253976	2.8483E-05
RP5-858L17.1	1.44218795	2.8483E-05
RP11-443B7.1	1.03772922	2.8991E-05
NUP62	0.39519239	3.0192E-05
IL6ST	0.55027339	3.0353E-05
PCCA	0.90815791	3.2952E-05
UPP1	0.44844558	3.302E-05
GLMP	0.8901199	3.3125E-05
PLEC	0.46616993	3.3367E-05
OCIAD1	0.24091926	3.3952E-05
RP11-556E13.	1.74691455	3.4253E-05
DUSP3	0.48707258	3.4704E-05
TGFBR2	0.58445912	3.5314E-05
SLC35F6	0.67019767	3.5964E-05
RBP7	2.16737998	3.6224E-05
IFI16	0.31001683	3.6999E-05
LGALS3BP	0.70917954	3.7336E-05
DHRS7B	0.82100746	3.7442E-05
RCBTB2	0.66381472	3.803E-05
MBNL2	0.82014029	3.9885E-05
DIAPH2	0.66945415	4.0012E-05
IGF2BP2	0.86547403	4.0789E-05
FAM89A	2.18650221	4.1016E-05
EHD1	0.88381579	4.1841E-05
FMNL3	0.63453204	4.1923E-05
CDK16	0.48381054	4.2087E-05
SMC2	0.49073697	4.2779E-05
SERINC1	0.31370787	4.3048E-05
HEXA	0.25683289	4.3048E-05
AIG1	0.37603884	4.3394E-05
PNRC2	0.21880888	4.374E-05
METAP2	0.26896786	4.4159E-05
SPRED1	0.85207697	4.4488E-05
TLR8	0.91954566	4.5748E-05
CXCL5	3.34973032	4.6444E-05
MILR1	0.45188363	4.6684E-05
TMEM55A	0.54240341	4.684E-05

ZFY	0.96180967	4.6869E-05
C11orf73	0.29401925	4.7162E-05
MT-ND2	0.27266673	4.7912E-05
RAP2A	0.53869446	4.7936E-05
RIN1	0.8056952	4.8186E-05
RIT1	0.49887967	4.862E-05
NLN	1.26227707	4.8661E-05
FNDC3A	0.5685743	4.9334E-05
ZNF511	0.48469525	5.0114E-05
TMEM88	0.54966564	5.0359E-05
SELT	0.22188047	5.0799E-05
RPGR	0.48371277	5.1392E-05
CCDC88C	1.24368174	5.1481E-05
HPCAL1	0.58560631	5.2087E-05
RBM47	0.55258276	5.2697E-05
DPH6	0.75381773	5.3398E-05
SERINC3	0.31308221	5.382E-05
FFAR3	1.92873731	5.4474E-05
RGAG4	1.66058434	5.4933E-05
RARA	0.40336059	5.5348E-05
UBE2D3	0.26956196	5.5542E-05
FAM213A	0.86923367	5.6845E-05
RP1-266L20.2	2.08131106	5.7724E-05
PEAK1	1.01453845	5.7869E-05
CXCL9	2.01638991	5.8172E-05
SMARCD3	1.90876267	5.8184E-05
DCTN4	0.41906469	5.8242E-05
LYAR	0.44925664	5.8701E-05
LAMB3	0.72987867	5.8965E-05
C11orf21	2.17428423	5.9229E-05
MAP2K1	0.44032669	5.9287E-05
PPFIA1	0.50118979	5.9591E-05
DDX60L	0.74230592	6.0132E-05
CLIP2	0.99906493	6.0862E-05
TFDP1	0.52384259	6.0998E-05
TCEAL3	0.85005513	6.1412E-05
OXR1	0.49718854	6.2597E-05
SEPT8	1.45841508	6.3259E-05
FXN	0.55198574	6.7626E-05
PIK3C2A	0.41810685	6.8084E-05
PROCR	1.16552558	6.8212E-05
DENND1A	0.6018914	6.9987E-05
ARHGAP10	0.93172191	7.2874E-05
RP11-519G16	1.75382002	7.2939E-05
SLC37A2	0.97983117	7.4021E-05
LINC00243	2.15712474	7.6107E-05

PAIP2	0.23998987	7.6272E-05
CXCL12	1.69915371	7.6497E-05
RP11-84C10.2	3.73451443	7.9355E-05
LPCAT1	0.53874936	8.0319E-05
MMP14	1.70739174	8.1064E-05
ANKRD9	0.79571643	8.183E-05
WDFY1	0.59543345	8.3921E-05
CISD3	0.38631862	8.5188E-05
PCMT1	0.26301357	8.6091E-05
JMY	0.65389194	8.8056E-05
CUEDC2	0.29209408	8.8777E-05
SDS	1.33662402	8.9076E-05
SMYD4	0.93460648	9.2307E-05
UXS1	0.4753384	9.3188E-05
DDX1	0.49096456	9.5058E-05
RRAD	0.67017156	9.5411E-05
CCND2	0.5893303	9.5457E-05
VPS9D1	0.97368609	9.6823E-05
CD300A	0.53544289	9.8061E-05
ITGA5	0.83550691	9.9198E-05
SLC8B1	0.5652297	9.9774E-05
HDAC4	0.73801075	0.0001001
SH3BGRL3	0.36545291	0.00010165
GADD45GIP1	0.28551803	0.00010397
CTD-2006K23.	1.06347511	0.00010422
GSAP	0.4246424	0.00010454
RP11-61J19.5	0.68327303	0.00010463
LAP3	0.33798664	0.0001079
S100A13	0.8534486	0.00010838
BTBD11	2.36894083	0.00010838
CHD2	0.26648417	0.00010866
MB21D1	1.10639321	0.00010886
ABHD5	0.72615258	0.00010923
STX2	0.85055672	0.00010924
ZNF780A	0.63697407	0.00011109
CD79B	1.77892458	0.00011259
VIPR1	3.47328979	0.00011894
SNHG15	0.48265987	0.00011932
SLC46A1	1.55640774	0.00011949
STX8	0.27635986	0.00012095
QSOX1	0.4027417	0.00012145
RP11-489O18	2.93558061	0.00012228
RP11-542M13	1.58086296	0.00012228
FPR2	2.12342419	0.00012328
SYNGR1	0.8905966	0.00012329
PDCL3	0.39564397	0.00012813

DDO	2.05157681	0.00012824
RTCB	0.32413707	0.00012841
NDUFB8	0.23424986	0.0001296
SNX18	0.64001516	0.00012971
NPL	1.33723527	0.00012999
FASTKD1	0.68711722	0.00012999
SAV1	0.88507363	0.00013002
ZNF697	1.87905909	0.00013013
EPHX1	1.06446242	0.00013013
RTN3	0.3344628	0.00013043
HSDL1	1.2901257	0.0001308
DOC2A	1.47793905	0.00013667
NIPBL	0.27991763	0.00013725
SRBD1	0.75003009	0.00013737
ITGA9	1.40239645	0.00013743
IMPACT	0.39484857	0.00013781
CR1	1.02344809	0.00013845
OLFML2B	2.11948031	0.00014046
LRP4-AS1	2.87456514	0.00014046
SMIM3	0.76097106	0.00014286
PPIF	0.34387266	0.00014521
LINC01023	1.54678747	0.00014938
PDHA1	0.31814393	0.00015107
DGKD	0.71441503	0.00015146
UNC5B	1.77418792	0.00015424
CITED4	0.76780911	0.00015431
C11orf74	0.53757745	0.00015449
AP001046.5	2.07211659	0.00015524
WDR45	0.45232574	0.00015681
IQSEC1	0.61880282	0.0001593
AP2M1	0.20945206	0.00015968
NUDT14	0.80767351	0.00016143
CRY2	0.67067016	0.00016323
MFGE8	1.59760289	0.0001644
CCDC151	1.67054274	0.00016617
CHML	0.43127231	0.00016665
PRPF38B	0.26942589	0.00016858
QTRT1	0.41088083	0.00016969
PHLDA1	0.68180331	0.00017226
ITGB8	1.96144669	0.00017317
PTGS1	0.57262101	0.00017378
SLC4A7	0.44275025	0.00017411
SEPT2	0.18116372	0.00017497
HNRNPU	0.18867588	0.00017516
GNLY	2.73211781	0.00017915
RP11-661A12.	1.82701728	0.00018291

ABTB1	0.3752854	0.00018298
ASCL2	0.63292621	0.00018616
ZFP36L2	0.5732919	0.00018711
SLC38A6	1.11842943	0.00018752
RILP	0.50932558	0.00019109
BAZ2B	0.36679386	0.00019351
FICD	0.80507044	0.00019351
C16orf58	0.7554305	0.00019432
SH3TC1	0.32914969	0.00019665
DECR1	0.39370665	0.00019937
TOR4A	0.66469376	0.00020515
ACAT1	0.39977657	0.00020906
TNFRSF10A	1.41799399	0.00021262
STEAP4	2.31732331	0.0002128
USP11	0.50137202	0.00021468
RP11-254F7.2	0.81236179	0.00021699
ACOT11	2.2278403	0.00021764
DYNLT1	0.3226334	0.00021978
SIRPB2	0.93172091	0.00022126
DDX60	0.79831068	0.00022342
RYR1	0.52301971	0.00022489
RP5-899E9.1	1.66248894	0.00022946
GNPDA1	0.48578573	0.00023493
SLC2A3	0.30857243	0.00023543
PPP2CB	0.47966137	0.0002356
HSD17B4	0.36133483	0.00023563
LINC01504	0.97548277	0.00023635
RNF144B	0.3633939	0.00023723
SYPL1	0.37328992	0.00023821
MTHFD2	0.30978524	0.00024286
ROCK1	0.23560296	0.00025053
WWP1	0.69764947	0.00025088
PHLDB3	0.58537456	0.00025438
GGT5	1.38404442	0.00025608
CD84	0.7336411	0.00025742
TMEM138	0.50425931	0.00025749
THTPA	0.80232481	0.00025876
RP11-809O17	0.97560976	0.00026087
APEX1	0.20413113	0.00026186
KIFC3	1.87342856	0.00026628
FBXL5	0.40796613	0.00026761
LONRF3	1.31029474	0.00027827
OSGIN1	1.45483617	0.00027846
GBP4	0.56304096	0.00027848
USP53	0.503007	0.00027945
NSUN4	0.51129842	0.00028181

KLF11	0.83222091	0.00028246
RBBP8	0.74123642	0.00028246
CDIP1	0.54385819	0.00028533
BIRC2	0.39385748	0.00028718
PC	1.53461584	0.00028867
SRGAP2C	0.35984431	0.00028882
CTD-2350C19.	2.30966764	0.00029218
BTBD2	0.71743391	0.00029405
SIGLEC12	1.57247549	0.0002968
NRAS	0.37813199	0.00031265
RASA4	0.80966896	0.00031294
STOM	0.45306103	0.00031509
AC147651.4	1.15245039	0.00031524
CHST15	0.73643759	0.00031749
MFSD2A	1.02037072	0.00031874
COQ2	0.51888713	0.00032644
LINC01410	1.76561563	0.00033133
TMLHE	0.52007006	0.000333
PHF10	0.39494046	0.00033465
ABCC3	1.35846224	0.00033548
AP3S1	0.2536446	0.0003357
MLKL	1.00839391	0.00033602
CTD-2135D7.5	3.0548891	0.0003368
TMEM165	0.26192439	0.00034198
CYC1	0.3406933	0.00034198
PIP5K1B	1.67741478	0.00034245
NDUFAF2	0.34004118	0.00034521
PPFIBP1	1.18005312	0.00034738
SELL	0.66400538	0.00034817
PANX1	0.83747971	0.00035997
HRH2	0.71148655	0.00036919
CIDEB	0.65890497	0.00037097
MIR155HG	1.22317124	0.00037097
ITPR1	0.44962628	0.00037363
FADS1	0.63520936	0.00037445
SRD5A3	0.5439673	0.00037508
PCK2	1.11693405	0.00037748
MAFF	0.63370724	0.0003804
TPRKB	0.24271869	0.00038306
CLEC4F	0.86076405	0.00038588
SFT2D3	0.48955934	0.00039209
IDH3B	0.25404299	0.00039234
DDB2	0.67681967	0.0003989
SGPL1	0.64388195	0.00040555
IRG1	3.49931923	0.00040953
FAM210B	0.37896536	0.00041028

TMEM123	0.23456649	0.00042075
YIPF4	0.32096293	0.00043556
TMEM60	0.58249022	0.00044812
AMPD3	0.57637992	0.00044812
RNF115	0.30865184	0.00045125
GLB1	0.31547469	0.00045125
TCF4	0.46755249	0.00045218
NLRP1	0.71659051	0.0004552
FMR1	0.43710611	0.00045553
MFSD13A	1.02018877	0.00045864
CHMP1B	0.27171708	0.0004613
CTSH	0.37828841	0.00047308
WLS	1.82900362	0.00047607
RNF187	0.38488455	0.00048183
P4HB	0.23106737	0.00048747
TCTEX1D1	2.23608834	0.00048786
CDK18	1.74961736	0.00048978
UBXN10-AS1	2.92232907	0.00049148
JUNB	0.42248463	0.00049776
P2RY2	1.24152465	0.00049805
ANKRD37	0.58372472	0.00050983
TNFAIP8	0.63865125	0.00051508
IGFBP4	0.95892427	0.00051518
G6PD	0.38291493	0.00051664
ADAM15	0.44887476	0.00051879
CTD-2545M3.	2.48338781	0.00051879
NFE2L3	0.48315902	0.00052442
TMEM104	0.85807715	0.00053818
SH3GLB1	0.23025364	0.00054671
RRAS	0.64007427	0.00055088
LAYN	1.66150318	0.00055147
COX5B	0.21693733	0.00055289
SLC6A7	2.37984525	0.00055631
ADAR	0.24879399	0.00055931
AGAP3	0.47898251	0.00055931
ATRAID	0.26500357	0.00056082
MAD1L1	0.58318099	0.00056176
TMEM2	0.57784786	0.0005723
CNRIP1	1.4391468	0.00057564
SLC22A18AS	1.11872832	0.00058642
VIM-AS1	0.70717708	0.00058642
PIGX	0.43129383	0.00059138
NAT9	0.57514078	0.00059422
SURF4	0.2867362	0.00059652
IRF7	0.41423592	0.0006108
MGST1	0.95658211	0.0006112

POLE4	0.32732523	0.00061132
HSPA9	0.26583275	0.00061178
PVRL2	0.63157061	0.00061535
BMP2	2.30046082	0.00061825
P2RY12	1.84010207	0.00062473
NHS	2.82466018	0.00063016
GAS6	0.50905324	0.00063051
LRCH4	0.38592684	0.00063741
MRPS5	0.34591585	0.00064292
NBPF26	0.8096141	0.00064417
UQCRFS1	0.22284064	0.00064417
GINS4	0.72484683	0.00064488
CAPN1	0.36603894	0.00065546
TMEM19	0.440664	0.00065673
RP11-1143G9	0.67912292	0.00066123
ALG13	0.33463804	0.00067294
CDC42SE1	0.34230037	0.00067371
WAC-AS1	0.37991031	0.00067703
DENND4C	0.62810316	0.00069371
ARNTL2	1.53446184	0.00069722
CLCN7	0.46224267	0.0006991
RFFL	0.8584455	0.0006991
C6orf223	1.40923489	0.00070668
RBPJ	0.23520317	0.00071297
TMEM176B	1.51605774	0.00071297
LILRA1	0.69902453	0.00071426
JARID2	0.51768161	0.00071638
AP003774.6	0.82282131	0.00072439
ARHGAP6	2.1064989	0.00072715
MAP7	1.78221966	0.000729
DPYSL2	0.25172439	0.0007305
DAD1	0.15577119	0.00076074
COPA	0.27095665	0.00076244
NFKBIE	0.51388132	0.00076356
TMEM91	0.91215679	0.0007657
ZNF185	1.32263163	0.00076803
TLE4	0.47707643	0.00077146
SGIP1	3.28002365	0.00077246
TMOD3	0.31840407	0.0007737
PIK3CA	0.70134767	0.00077393
AC010883.5	1.69390278	0.00077399
RFX2	0.78968347	0.00077804
CLOCK	0.53152924	0.00078681
CSF3	3.40559612	0.00078995
PMVK	0.26239063	0.0007921
JAK3	0.60530977	0.00080058

SEC23A	0.45715147	0.00081131
CX3CR1	1.10758448	0.00081696
CD72	0.34680855	0.00082171
WIPI2	0.3958124	0.00082898
SRSF5	0.20810622	0.00084436
SDCCAG8	0.37471299	0.00084571
TRIM25	0.63362927	0.00084709
CLTA	0.23702169	0.00085307
PARL	0.273557	0.00087202
LIPN	1.47691077	0.00087348
BTK	0.31308127	0.0008849
MT-ND5	0.2346939	0.00089373
CCDC152	1.61274321	0.00090333
ACVR1B	1.0237561	0.00090406
NOTCH2NL	0.77096773	0.00090983
OSGEP	0.2859939	0.00091962
CTBP2	0.405179	0.00092071
YWHAB	0.16129125	0.00093292
ODF3L1	1.29050414	0.00093591
PTAFR	0.56154036	0.00093839
WDFY3	0.66765642	0.00094616
TFPI	1.59559018	0.00095407
G0S2	0.73142082	0.00097277
UTF1	0.68320414	0.00097747
UBE4A	0.37048638	0.00098315
CRTC3	0.50972818	0.00098651
RASGRP4	0.63540783	0.00100384
MMD	0.5195765	0.00100695
BBS2	0.80967418	0.0010116
C17orf58	0.77413587	0.00104049
COX7A2	0.20075541	0.00105278
FSTL3	1.25585778	0.00105545
HADHB	0.25666385	0.00106623
TTLL4	0.84293752	0.00107097
CYB5R1	0.39067451	0.0010849
AREG	0.43371571	0.00108523
VPS25	0.32751826	0.00109635
CHIC2	0.46673539	0.00110022
SERPINF1	1.21750441	0.00110315
BTN3A2	0.4940213	0.00110712
WWOX	0.76435858	0.00111212
TCF7L1	1.59764306	0.00111922
INHBA	1.04679244	0.00112863
MMGT1	0.41685726	0.00113614
FLI1	0.48873589	0.00114886
SULT1B1	1.41363076	0.00115914

CACUL1	0.3561544	0.00118686
ZNF189	0.99728241	0.0011877
TRIQK	0.50336211	0.00120095
AC002451.3	2.2323598	0.00120332
NDUFV3	0.22266303	0.00121279
OLFM1	3.96804819	0.00122396
ESD	0.22019011	0.00122683
MRAS	0.57018403	0.00123875
ZCCHC14	0.83158662	0.00124268
OGFRL1	0.3053127	0.00125421
RAB21	0.38062447	0.00126001
NDST2	0.65235113	0.00126033
CD101	0.41179823	0.00126058
FAM20C	0.59656452	0.00126081
KNSTRN	1.08915265	0.00126515
RP4-728D4.2	1.54041647	0.00127462
RP11-611O2.5	1.33513219	0.00127725
RP11-624L4.1	2.73283137	0.00128101
UBE2L6	0.22079574	0.00128197
YTHDF3	0.31553498	0.00128698
MCL1	0.1589448	0.00129416
METTL9	0.23196896	0.00129759
IST1	0.29241578	0.00130908
SEC63	0.2251504	0.00132914
OSBPL5	1.38802261	0.0013369
TPD52L2	0.35837454	0.001346
MRPL14	0.27838984	0.00135031
ZC3H11A	0.28549347	0.00135166
RNF175	1.54618071	0.00135516
PVR	0.92538236	0.0013629
PDGFC	0.81276578	0.0013631
RP11-1149M1	2.90486687	0.00137023
IFI6	0.42249357	0.0013768
AC009948.5	1.21205886	0.00138498
DHFR	0.65846756	0.00138626
PITPNB	0.39992922	0.00140494
RAB9A	0.47871081	0.00141089
IFITM10	1.27071073	0.00141129
PLA2G15	1.22102692	0.00142659
SRGAP2	0.35782681	0.00142679
AP2A2	0.5101308	0.00143337
BTD	0.59190673	0.00144645
ERCC1	0.25326466	0.00144645
ARRB1	0.42423643	0.00145857
CHMP3	0.28645748	0.00145943
RP11-295G20	0.58824013	0.00146196

INPP5A	1.34833491	0.001476
SLAMF1	1.8050027	0.00147871
GAB2	0.43507977	0.0014874
RP11-390P2.4	0.75497069	0.00150246
ING3	0.28381088	0.00150412
TBC1D14	0.5019463	0.00151136
B4GALT7	0.45161046	0.00151241
FXYD2	1.28388534	0.00152601
ZMYND11	0.54811893	0.00152616
RNH1	0.15504246	0.00153285
RIPK2	0.38999725	0.00153363
CLINT1	0.291416	0.00153787
LRP3	2.42203991	0.00153796
AGO4	0.47967414	0.0015497
AC002456.2	1.41337499	0.00155731
ZWINT	0.89870427	0.0015664
EZH1	0.58269207	0.00161578
MICAL2	0.61297301	0.00162932
NLRC4	0.82210563	0.00164585
AK2	0.28810871	0.00165425
RPN1	0.25234072	0.00165562
GAPT	0.65237002	0.00165562
C14orf159	0.43728487	0.00165796
C14orf80	0.64909326	0.00165796
CASP5	2.51182326	0.00166333
SP100	0.28278973	0.00166965
SIGLEC5	1.19875967	0.00167069
DNMT1	0.23707622	0.00167327
MT1G	1.31954454	0.00167419
SLC43A1	1.79716507	0.00172483
LINC01506	2.89091593	0.00173878
CTD-2588E21.	2.99393118	0.00174914
RP11-443B7.3	2.20060371	0.00176482
RP11-582J16.4	2.35357984	0.00176523
GATAD1	0.40142609	0.00180047
ABCG2	2.2418288	0.00180544
SRA1	0.23018094	0.00180544
IFIH1	0.37192748	0.00181624
PTPN12	0.42380179	0.00183064
LST1	0.31613677	0.00183447
TLR7	0.89789662	0.00184165
SLC26A2	0.50972771	0.00185734
ERCC8	0.67452938	0.001868
AAED1	0.25205137	0.00187505
MYO15B	0.65984947	0.00189296
STARD5	0.57883224	0.00189889
TFG	0.30443252	0.00191996

PCSK5	1.88894789	0.00192524
PLD4	0.41661624	0.00194087
RP11-1391J7.1	1.08791028	0.0019641
RAB37	2.11106552	0.00200332
TMCC1-AS1	1.25229413	0.00200616
RBM7	0.28979204	0.00201254
CALHM2	0.68032435	0.00203565
GALK1	0.29171401	0.00203943
IL1R2	0.71735211	0.00206928
HSBP1L1	0.69740805	0.00209032
GLS	0.22640212	0.0020986
ATP6V0A1	0.4667504	0.00210706
IL27	1.95284047	0.00211062
ADORA2A-AS1	1.81288275	0.00211678
CRY1	0.73454177	0.00212001
NBPF14	0.43880335	0.00215005
SLC19A1	0.76971438	0.00215416
CTA-292E10.6	0.47704838	0.00216826
PDE2A	0.74167603	0.00217174
SYNE1	0.8382971	0.0021834
MRPL33	0.19929033	0.00218532
GRIP1	0.70792145	0.00219007
ZNF581	0.32629152	0.00220121
CELA1	3.02883119	0.00220789
RAMP2	1.30688347	0.00221503
BAIAP2	0.35332479	0.0022205
DHRS4	0.46522853	0.0022351
NDUFS7	0.23398812	0.00224179
CENPE	0.80959159	0.00224449
MUC1	1.32453362	0.00225752
ATF7IP2	0.44838845	0.00228069
RNPC3	0.40096798	0.00228372
GPR34	0.71565989	0.00228588
PRKCE	0.68782958	0.00228882
RCAN1	0.46551058	0.00229847
NOTCH4	0.83041551	0.00230638
HEG1	1.17524045	0.00231036
IMMT	0.32091519	0.00232491
GAS7	0.37016947	0.00232988
TM6SF1	0.38226078	0.00233737
MEF2C	0.38376429	0.00233873
COA3	0.30430349	0.00234513
PTGES	2.45501713	0.00237514
MTRF1L	0.42590151	0.00238382
MDM4	0.28107589	0.0023954
CD58	0.45748211	0.00241422
NCOA2	0.45471799	0.00243944
CYB5D1	0.86425825	0.0024785
JAZF1	0.51913748	0.00248884
RSRC1	0.30374924	0.00251145

PIP4K2A	0.40363175	0.00252799
SEC24A	0.5132203	0.00252981
NDUFB7	0.16151888	0.00253405
FAM72A	0.5980486	0.00253994
SIAH2	0.39196408	0.00254054
XIST	0.22906828	0.0025855
LY6E	0.3005488	0.0025962
GABPB1	0.4099011	0.0025962
LRRC32	1.14985934	0.00260359
ABHD13	0.42989004	0.00261598
RP3-473L9.4	1.53557898	0.00268372
RP4-549L20.3	1.33741194	0.00277514
ERP44	0.20692195	0.00280293
MTMR6	0.3207012	0.00280293
RP11-598F7.3	0.72406531	0.0028305
CTDSP1	0.39154391	0.00284542
NFIX	1.16075889	0.00285492
UQCRC2	0.18117879	0.00287388
POLR3GL	0.27603645	0.00287471
YPEL2	0.5322213	0.00288507
AP1B1	0.24375795	0.00291359
YPEL4	0.65002994	0.00292335
MRPL32	0.27803966	0.00297257
BCL10	0.31959485	0.00301838
CECR5	0.42327496	0.00302499
FGD5-AS1	0.31069591	0.00307002
GPAT4	0.30611364	0.00311263
CEBPE	1.17833158	0.0031213
SERINC5	0.3806689	0.00312832
E2F3	0.41829931	0.00312832
TSG101	0.26979168	0.00313413
ZDHHC21	0.87005634	0.00316394
DHX32	0.67917468	0.00318493
NDUFA5	0.26393192	0.00319617
XRN2	0.16727329	0.00322806
HTATIP2	0.29200574	0.00324059
ITGB2	0.21043327	0.00325074
ZDHHC20	0.44697264	0.00326152
HACD3	0.48609426	0.00326484
GHDC	0.57519056	0.00326872
GRAMD1B	0.92518794	0.00328398
ADAMDEC1	1.13692772	0.00332064
GFM1	0.41004353	0.0033552
C1orf56	0.31401105	0.00336116
SAR1A	0.25347164	0.0033692
FAM26F	0.23547048	0.00340603
EPB41L2	0.31206937	0.00342057
DNPEP	0.30323285	0.00342782
SPCS2	0.16541529	0.00342981
CLPB	0.58745727	0.00343602

KAT8	0.3468549	0.00344418
CYSLTR1	0.50321422	0.00345357
VGLL4	0.50975079	0.00346256
MRPS28	0.30170941	0.00348512
CFLAR	0.34359298	0.00348692
COPB2	0.26001204	0.00349153
DTX3L	0.49903424	0.00349974
RASSF3	0.30517931	0.00350031
MORF4L2	0.21510955	0.00353078
DNAH17	1.19329915	0.00355
CYP27A1	2.52339079	0.00356385
MIS18A	0.58137825	0.00361438
XRCC4	0.47536634	0.00363579
PXK	0.32992791	0.00364777
OPN3	0.45587843	0.00365043
CUL4A	0.37281221	0.00367032
SYNE3	0.66286513	0.0036913
CDH26	2.34958792	0.00370298
RP11-378A13.	1.71793466	0.00374622
GRK5	1.06738444	0.00384892
FAM21A	0.51259166	0.00394197
NFKBIB	0.35391387	0.00398807
CCNE2	1.30945121	0.00403652
GTF2IRD2B	0.97931162	0.00405919
NTAN1	0.40216413	0.00413123
KLHL9	0.68101344	0.00413461
TEC	0.64029386	0.00416347
TMEM218	0.34579912	0.00417633
AGAP9	0.92796297	0.00422955
CLEC6A	2.33518849	0.00424717
CFAP46	2.79064002	0.00427126
CLIP4	0.97871037	0.00436807
NEU4	0.75021916	0.00437593
REL	0.35236762	0.00437665
FOXRED2	0.8464464	0.00441976
PIAS2	0.51409548	0.00450358
RCC2	0.25589789	0.00453833
DCAF10	0.41769301	0.00455915
SNX29	0.42957768	0.00457843
RASA1	0.88186468	0.00458375
TPCN2	0.65884537	0.00461929
CRIP1	0.28252667	0.00462305
CPEB4	0.38681014	0.00464051
DMXL1	0.30128162	0.00464736
CHD3	0.30627938	0.00470642
AK4	1.47937571	0.00476938
EEPD1	0.76471919	0.00482223
VMP1	0.27351201	0.00482582
MPO	2.08655979	0.00486278
AGO2	0.37555284	0.00486675

B3GALT6	0.33683397	0.00488356
EIF4EBP1	0.24963217	0.00489317
IRF1	0.42215816	0.00491989
C1orf53	1.13940367	0.00496185
NFKB1	0.41356126	0.00500115
DHRSX	0.30231529	0.00500115
WARS	0.41575267	0.00507415
MS4A14	0.97905891	0.0051146
ATP11C	0.44523474	0.00514507
LYSMD3	0.41240101	0.00520906
VAV1	0.3851282	0.00527056
TPM1	0.88371688	0.00530274
PLA2G16	0.42821778	0.005304
TMBIM1	0.52474348	0.00536972
OLFML3	1.79360454	0.00538713
ATP5C1	0.15040765	0.0054576
HAUS7	0.36666818	0.00546516
NFKBID	0.29578652	0.00547299
RP11-67L2.2	0.70097476	0.00549641
DCUN1D3	0.55898191	0.00560265
MEF2A	0.31385409	0.00567759
ADORA3	0.78205763	0.0057254
TMEM189	0.35848788	0.0057538
EDEM2	0.28188231	0.00577437
IL12RB1	0.49478348	0.00577472
CXCL14	1.11868204	0.00582894
TEKT5	2.20978602	0.00584882
PIK3C2B	1.49022383	0.00588457
MVB12A	0.35406562	0.00588753
IQCD	1.33794574	0.0059343
CPEB3	0.77470328	0.00599024
RP5-899B16.1	3.33221351	0.00604738
SLC9A8	0.61080589	0.00606529
FNIP1	0.27764819	0.00607996
ARAP1	0.336609	0.00609491
CISD2	0.35640439	0.00612025
ARHGAP21	0.3086916	0.00612025
TRMO	0.43009663	0.00615054
SEC11C	0.24143072	0.0061681
HGSNAT	0.25575668	0.006231
DAGLB	0.41471779	0.00628233
MPV17L	1.18243804	0.00629949
CARD19	0.39262212	0.00635181
LINC01127	3.41603641	0.00637272
BISPR	0.51736466	0.00639863
AKT1S1	0.45537251	0.00645098
RPS9	0.1442847	0.00647374
GNPTG	0.32256853	0.00650829
RP11-1055B8.	1.60310535	0.00650829
CECR6	2.78857164	0.00663501

ATP5B	0.17305608	0.0066663
SLC22A15	1.54942175	0.00667401
TUBB4B	0.22265389	0.00675122
SRGAP2B	0.30849265	0.0067815
ARHGEF2	0.48046848	0.00678509
ZNF524	0.30410927	0.00678509
CACNB4	1.48554096	0.00679056
CALCOCO2	0.28332249	0.00679493
DDX27	0.24206999	0.00681068
YIF1A	0.21941819	0.00683978
NT5DC4	3.0494119	0.0068683
AIMP1	0.19366432	0.00704014
CFB	1.83489196	0.00704095
ARID5B	0.59213078	0.00704676
RP11-573G6.1	2.70606956	0.00705518
HSF2BP	2.27905975	0.00713558
GPR108	0.34256354	0.00721837
HK2	0.52796756	0.00723181
MIF4GD	0.27839916	0.007305
AC061992.2	1.63357279	0.00730796
LOR	1.2935253	0.0073414
C19orf54	0.6675325	0.0073943
ANKLE2	0.36786415	0.00743423
RP1-261G23.7	2.13980738	0.00743443
MTF1	0.53975254	0.00744292
DAGLA	1.21568251	0.00745012
SMG9	0.63656166	0.00745012
SMIM15	0.40226535	0.00749332
KRAS	0.29406192	0.00755689
HLA-DMB	0.12318971	0.00765092
DGCR6L	0.26762891	0.00766775
AHCYL1	0.22335396	0.00767227
IFI27	0.77748346	0.0077445
MLLT11	0.61980576	0.00783015
CTSO	0.35985493	0.00783412
CCDC34	0.72593737	0.00786051
DCAF12	0.60697994	0.00794532
CASC11	2.43422525	0.00806021
NCOA1	0.30796127	0.00808673
VNN1	0.82041112	0.00809448
CTBS	0.28740062	0.0081283
TMEM52B	0.87065348	0.0081283
TMED2	0.1763306	0.00818463
ATP5H	0.16884513	0.00818869
RYK	0.50047233	0.00825937
RAMP2-AS1	1.73241025	0.00829472
ASPH	0.45679082	0.008369
HDGF	0.26835092	0.00836963
RP11-214O1.3	0.48926812	0.00839022
KCTD20	0.23171906	0.00843464

TBC1D16	1.21856634	0.00852401
SEMA4D	0.42607295	0.0085476
ETNK1	0.29482125	0.00857404
RP11-344B5.2	2.51893748	0.00867945
MTFP1	0.64538894	0.00870513
AP1S2	0.31436038	0.00874433
TSC22D1	0.65674464	0.00874433
NAA20	0.19837451	0.00874949
PROS1	1.58673103	0.008755
LRP11	2.9286164	0.008755
APPL2	0.72014762	0.0087664
LY6K	0.99961338	0.00877318
KLHDC2	0.41627338	0.0088039
CROCC	1.50122245	0.00882754
COL4A2	0.94186976	0.00882765
TMEM37	1.24747262	0.00882942
PLK3	0.2747655	0.00884168
RP11-536K7.3	2.09074475	0.00884168
LOXL3	0.73955363	0.0088924
LPAR6	0.47368382	0.00890741
MDH1	0.17341825	0.0089114
CCDC9	0.4312739	0.00899766
ADRB1	1.19130166	0.00902632
DARS	0.2220752	0.00905062
SCAMP1	0.38829823	0.0090569
C20orf196	0.54613684	0.00910236
RP11-356I2.4	0.46651725	0.00912983
TRMT6	0.49271045	0.00913692
TSNAX	0.29939778	0.00914301
PRPS2	0.35530339	0.00917178
NPTN	0.28951749	0.00918145
NCSTN	0.30810263	0.00919275
PLXNB2	0.31519252	0.0092988
IER3IP1	0.21105427	0.00930008
ALDH2	0.34743427	0.00933479
HPGD	0.92727412	0.0093417
IL7	1.07422948	0.00935005
DSTYK	0.64880268	0.00941104
LRRC8D	0.53264699	0.00941418
SLX4	0.86136151	0.00948561
RP11-468E2.4	0.38187728	0.00950222
CAMKK2	0.44668311	0.00952568
RBMS2	0.74263542	0.00954698
SLC31A2	0.30291933	0.00955725
DLEU7	1.1224548	0.00955906
CTB-41I6.1	1.07703003	0.00959546
TNFAIP8L2	0.37260776	0.00967429
VMA21	0.21089908	0.00967969
DNAJC10	0.25567424	0.0097229
CHSY1	0.4122574	0.00973585

TBC1D15	0.30242679	0.00979428
RP11-401F2.3	2.05070156	0.00984498
SIAE	1.33502797	0.00986884
AP2S1	0.1359549	0.00987086
CDC25B	0.74319279	0.00987547
SCCPDH	0.47989495	0.00987879
FSD1L	0.79212054	0.00993706
C2CD5	0.56311493	0.00993706
NLRP12	2.65643458	0.00994744
THUMPD3-AS	0.1835148	0.0099476
TCIRG1	0.28147788	0.00995845
CSF2	2.55313978	0.01003801
AC007950.2	1.2657349	0.01008927
ZFHX3	0.28172452	0.01012771
ZC3H3	0.45511121	0.01019422
HACD1	1.15848793	0.01022918
PLOD1	0.52413147	0.01026554
NSUN6	0.54515742	0.01027994
CEP57L1	0.86761061	0.01036057
PKD2L1	2.89673876	0.01036589
TRIOBP	0.33373185	0.01040694
N4BP2L2	0.19116324	0.01043257
SIL1	0.24932019	0.01043905
AZU1	2.27346745	0.01047127
XXYLT1	0.38062777	0.01065702
AF064858.6	1.49882941	0.01070685
IVNS1ABP	0.39457497	0.0108538
PER3	0.69218279	0.01106198
DENND6A	0.42151703	0.01112071
SOCS2	1.27010187	0.01112071
ORMDL1	0.22819954	0.01113591
ZNF358	0.51629299	0.01115205
RNF217	0.6462144	0.01117867
HERC5	0.86427508	0.01124735
POFUT2	0.56466758	0.01129381
GOLGA7	0.22523776	0.01142457
CYTIP	0.22404681	0.01147071
CDCP1	0.53818818	0.01152398
MMP25-AS1	1.2177982	0.01152713
RP11-284N8.3	2.10080726	0.01155545
OPA3	0.35518415	0.01163738
TCEAL1	0.40665954	0.01172908
SEC61A1	0.24034699	0.0117443
COL23A1	2.46623999	0.01176422
BCAP29	0.30629713	0.01180021
S1PR4	0.54822136	0.01182893
TMPO	0.2961579	0.01186324
KIDINS220	0.28070492	0.01193058
MT-ND4	0.16160631	0.0120686
KDM7A	0.29463374	0.01221188

USP2	1.26897673	0.01223322
PTGR2	0.75990608	0.01226193
HRH1	1.21560772	0.0122989
NFIC	0.36444159	0.01232023
GPRC5C	0.67165161	0.0124464
DTX2	0.74109261	0.01246185
CABLES1	1.68430189	0.01256284
LIMK2	0.73202916	0.0125761
HNRNPH2	0.1932174	0.01277137
MAPK6	0.42718622	0.01278167
UEVLD	0.60260422	0.01281017
MDM1	0.64080769	0.01294529
BCL2L2	0.78949762	0.01297091
SLAMF9	0.61996571	0.01309665
NDUFS6	0.17830607	0.01313119
BTG3	0.45371213	0.01314498
HMGXB4	0.24924629	0.01316223
TXNDC16	0.3848251	0.01328099
CHCHD7	0.2790788	0.01331509
AAK1	0.24484687	0.01332785
GCNT2	0.77975045	0.01332785
EFCAB13	1.29237511	0.01334278
CREB3L2	0.34064299	0.01335718
FAM109A	0.80264337	0.01344829
SLC25A11	0.20134061	0.01347081
RHBDD2	0.36492442	0.0134806
GK	0.35769327	0.01352588
GK5	0.5740373	0.01362881
CNBP	0.16942216	0.01368246
RPS4Y1	0.3831366	0.01368262
ANGPT1	1.47385043	0.01372379
MIS12	0.42736529	0.01372379
TRIM14	0.39441948	0.01377774
MPP7	0.78237592	0.01382459
AF064858.8	0.87530873	0.01382821
NBPF15	0.62051186	0.01384787
SIPA1L1	0.61264141	0.01384903
ADIPOR1	0.21130196	0.01394574
PPP4R3A	0.18180076	0.01394574
PFKFB4	1.47068143	0.01399034
SUPT4H1	0.18298387	0.01402191
FLNA	0.25946903	0.01408032
CFP	0.35541136	0.0140999
RP3-460G2.2	0.57866971	0.01420736
SFT2D1	0.14830137	0.01427209
YPEL3	0.36412207	0.01430494
SLC50A1	0.2716727	0.01431356
MRPS22	0.27179904	0.01435636
ALS2CL	2.07621094	0.01441566
NUMB	0.28864184	0.01445737

UTP11L	0.25975799	0.01454395
CAPN7	0.3451679	0.0147217
FIG4	0.44004605	0.01485779
RNF146	0.49147869	0.01496609
FAM101B	0.59874671	0.01509228
APBB3	0.57248927	0.01510073
ARF1	0.16575106	0.0151131
RP11-631N16	0.97137303	0.01512604
NEIL2	0.68087091	0.01525345
CD19	1.22299395	0.01527939
GHITM	0.16717406	0.01530847
LINC01537	1.68906984	0.01533743
CD38	0.34198675	0.01542896
RARRES3	0.28350659	0.01545465
MORN3	1.39993739	0.0154737
PDCD4	0.22770625	0.01547951
PKD2	0.76478044	0.015517
A2M-AS1	1.11490641	0.01555862
TRAF3IP2	0.77482414	0.01555931
MBTPS1	0.32518883	0.0156008
CMPK2	0.71363511	0.01570879
ATOH8	1.51128449	0.01572003
GGH	0.68491372	0.01573016
FAXDC2	0.89646155	0.0159355
ZDHHC9	1.06487285	0.01599743
ASGR2	0.43628085	0.01604918
GLT1D1	2.76384816	0.01625313
B3GALNT1	1.12152808	0.0162688
RHOG	0.1304505	0.0163977
CHST11	0.36297444	0.01640176
PICALM	0.19770633	0.01646111
IGF1	0.78085923	0.01652674
ARAP3	1.86544476	0.01674893
AHRR	0.87617564	0.0168178
MOB3C	0.61390344	0.01702218
KLF12	0.65556363	0.01713985
PTPN18	0.23074211	0.01724759
MGST2	0.148912	0.01740734
SHROOM1	1.33794556	0.01743709
SLC27A1	0.45229214	0.01746063
SYN1	0.81856007	0.01747466
RNF135	0.44187843	0.01752131
PGS1	0.3963325	0.01769774
TUBGCP2	0.2072482	0.01779749
MTX1	0.25062741	0.01781723
IKZF1	0.26516793	0.01783339
IRF5	0.44299576	0.01783886
EDEM3	0.38008067	0.0178476
CH507-42P11	2.54015307	0.01787604
ZNF704	1.24434402	0.01789975

MYBPC1	1.51961323	0.01789975
DNM3	1.98597172	0.0179089
SLC25A19	0.25646756	0.01799083
DNAJC18	0.74563144	0.01801366
ZNF557	0.41311703	0.01801892
NDRG1	0.34616321	0.01803766
USP24	0.42222907	0.01818515
CTC-428H11.2	0.60726875	0.01823073
TMCO4	0.49928367	0.01823606
CORO2A	0.98175581	0.01828569
KCNMB4	0.82252472	0.01835184
MRPL54	0.16082863	0.01856452
DUSP1	0.36743776	0.0186207
SLC25A40	0.44925295	0.01869775
FAM91A1	0.28428525	0.01881316
ULK2	0.63318459	0.01885186
RIN3	0.21921974	0.01899007
FBN2	2.38171847	0.01901086
NDUFV1	0.16566669	0.01907512
NID1	0.81284506	0.01910889
PRPF40A	0.14213298	0.01927383
NEDD9	0.30741736	0.01931012
ACRBP	0.55115365	0.01946766
SEPT10	1.37899786	0.01948544
R3HDM2	0.24041712	0.01951128
LIN7C	0.35654848	0.01953564
ARFRP1	0.31832719	0.01970875
DRAP1	0.16005837	0.01970961
RNF145	0.32590558	0.01971112
SPON2	0.55202088	0.01981452
PIPOX	1.09379243	0.01993548
SULT1A1	0.49443115	0.02001059
TMCO1	0.16471046	0.02002942
THAP6	0.54652852	0.02002942
TRAK2	0.47122085	0.02008561
MRVI1	2.69910507	0.02008561
CRK	0.29943186	0.0203418
KDELR2	0.19555315	0.02040955
ROBO4	1.77624848	0.02046248
CYSLTR2	0.3569942	0.02060601
RCHY1	0.34216072	0.02072553
CDIPT	0.31429976	0.02073567
NARS	0.22162649	0.02103106
ZNF277	0.26285115	0.02104084
HAPLN3	0.75356199	0.02115129
MMP17	2.10972107	0.02115129
SLC25A44	0.36514029	0.02127984
ALG2	0.29454685	0.02127984
QDPR	0.27717702	0.02135102
CDK1	1.00402366	0.02138767

LINC00657	0.15133475	0.02164351
DCP1A	0.2786047	0.02173914
SYAP1	0.17115479	0.02180525
VIMP	0.30129043	0.0218571
LYL1	0.37822878	0.021889
C1orf43	0.1423715	0.02193584
CCL23	2.40409235	0.02196695
TRGC1	2.54868966	0.0219974
BEX4	0.17750105	0.02204129
ADAMTS10	1.24191785	0.02212478
HAAO	0.45420463	0.02220527
CCDC47	0.17265675	0.02225637
MTFR1	0.58447073	0.02225875
DICER1	0.26876492	0.02227666
RRAGA	0.22382385	0.02227711
TP53I11	1.03197366	0.02227711
ATXN7L1	0.71637306	0.02230794
DENND4A	0.33420204	0.02233917
PRIMPOL	0.58101904	0.02248551
UBXN8	0.61120383	0.02250476
BTN3A3	0.5243835	0.02261459
CENPU	0.56155147	0.02263469
MORN2	0.70105835	0.02266968
CTD-2553L13.	0.78908615	0.02269066
ILVBL	0.30196306	0.02272742
TFDP2	0.2858752	0.02282682
UNC45A	0.32933886	0.02287752
WDTC1	0.45771414	0.02291174
BCKDHA	0.55918957	0.02303408
RIPK3	0.56148671	0.02321261
SIK3	0.30708633	0.0233347
TMEM176A	1.04673591	0.02333598
LENG8	0.24094335	0.02333598
RASAL3	0.38460408	0.02338414
CRLS1	0.30807962	0.02342662
RFC2	0.33941724	0.02344004
BMF	0.48151705	0.02349007
BAIAP2-AS1	0.77071906	0.02349007
NRDE2	0.36345471	0.02360551
FAM43A	0.46502322	0.02373054
DNAJC11	0.45253577	0.02378539
SEPT9	0.16868754	0.02379218
NUDT7	0.77113614	0.02411063
H6PD	0.44901539	0.02411454
HAMP	1.71155602	0.02411454
RPL22L1	0.17612183	0.02414138
RP11-156K23.	0.63648268	0.02414824
CLEC3B	2.29697541	0.02415064
ZFYVE16	0.22121963	0.02433197
MKNK1	0.35854343	0.02433754

FAM50A	0.19923113	0.02447768
TMEM126B	0.26271366	0.02462288
SUN1	0.62967935	0.02465684
WAC	0.17292143	0.02471177
NUDT6	0.72384784	0.02481985
PGRMC1	0.32117842	0.02481985
ACP2	0.43550582	0.02481985
FZD1	0.63107045	0.02490524
BMP2K	0.3501284	0.02496419
F3	0.9775437	0.02504216
UCHL5	0.27413896	0.02505092
NUBP1	0.20942834	0.02506926
TNIP1	0.38473112	0.02507667
CDC73	0.2526258	0.02510232
SLC12A6	0.41597264	0.0251794
SRP72	0.13857938	0.02519216
TMEM106C	0.76339585	0.02520189
KPTN	0.58376409	0.02522653
DDX3Y	0.42953039	0.02534497
AC005082.12	1.48588391	0.02543844
ZDHHC6	0.2971666	0.02552518
IFI35	0.21652134	0.02558528
GPR42	1.54764677	0.02558528
FHIT	0.40628849	0.02565819
GALNT6	0.39186208	0.02566148
CRADD	0.72135813	0.02579917
C21orf2	0.33449162	0.0261542
ELFN1-AS1	1.50815449	0.02620109
LINC00920	1.12008643	0.02625645
UBAC1	0.22305456	0.02648784
HADH	0.29821961	0.02651592
SSH1	0.47768906	0.02660911
ATP6V1F	0.16028337	0.02665484
CAMK1D	0.26671304	0.02666157
ATP2B1	0.24569078	0.0268038
MAT2B	0.20641248	0.02682824
ICA1	0.68094873	0.02682824
PTRH2	0.31559614	0.02689042
C2CD2L	0.45394353	0.02692589
UFSP2	0.35450469	0.02697117
SLC35A1	0.35912723	0.02697117
LARS	0.19113087	0.0269812
RNASEH2A	0.32616781	0.02725011
NAT1	0.61585676	0.02743837
DDX18	0.15910646	0.02749506
NOV	1.06955487	0.02749508
VEZF1	0.2625215	0.02752362
TMEM39A	0.32987941	0.02760223
MSRB3	1.89036138	0.02780051
NUF2	1.31782606	0.02780975

CCL8	1.93649378	0.02795656
ZNF219	0.82864047	0.02809303
LRRC59	0.22982379	0.02809303
AXL	0.51095639	0.02810221
NPEPL1	0.41665558	0.0282131
UBE2R2	0.15985395	0.02829045
KLF4	0.45417966	0.02829697
IFFO2	0.37079921	0.02831392
AC069363.1	1.67679552	0.02869545
SLC36A1	0.67459704	0.02871806
IDUA	0.52923894	0.02885743
SIDT1	0.53163395	0.02887949
RAB3IL1	1.28169234	0.02894708
PKNOX1	0.36206932	0.02894708
PTPN1	0.36193286	0.02897301
PSMA6	0.40953636	0.02900817
TPP2	0.22490345	0.02905382
TINF2	0.22795868	0.02927156
NIPAL4	2.37357287	0.02927156
SAMD9L	0.45584957	0.0292817
C9orf139	1.37572483	0.02935302
SWAP70	0.30667587	0.02945242
SPATA20	0.388529	0.02945242
TBL2	0.39079375	0.02963499
RP11-876N24	0.79676432	0.02971089
TMEM51	0.55126581	0.02978788
PRKAR2A	0.22456276	0.02978788
GALR2	0.57666167	0.02978788
TMCO3	0.40833491	0.029846
PIK3IP1	0.63119853	0.0298805
EMB	0.27340048	0.02997318
RP11-737O24	0.56497438	0.03037939
AKAP13	0.26640584	0.03045534
KLF10	0.39441103	0.03051768
SRFBP1	0.28227314	0.03056987
SLC38A10	0.34263472	0.03058491
FH	0.26680511	0.0306127
RP11-390E23.	0.53799464	0.03120325
LINC01094	1.53683792	0.03122048
PSTPIP2	0.39119409	0.03122048
ABCG1	0.55440914	0.03131285
ANKRD46	0.57833201	0.03142672
RELB	0.28199832	0.03146269
CRELD1	0.48981858	0.03153046
LMBRD1	0.2580398	0.03175816
NCEH1	0.43496227	0.03179011
CLEC7A	0.24827224	0.03179011
RAPGEF2	0.56483732	0.03194398
FAM110B	1.30175375	0.0319507
USE1	0.2209127	0.03210283

CD1B	0.59124681	0.03220034
FKBP9	1.46397596	0.03227375
GSDMD	0.17802242	0.0324395
STAT5B	0.44286981	0.0324395
CLCN5	0.41808231	0.03250364
PURB	0.30630998	0.03255349
MRPS18A	0.32266148	0.03257504
SLC25A51	0.42424813	0.03257504
STAC3	0.33521691	0.03258725
PUDP	0.48734033	0.03262375
PMEPA1	0.53590549	0.0328957
C10orf55	1.11754992	0.03296139
TACC3	0.3311561	0.03297122
BTBD19	0.64403924	0.0331611
LACC1	0.5103214	0.03322838
MRPL27	0.21020448	0.03330862
L3MBTL4-AS1	1.64394016	0.0333725
DPY19L1	0.53669796	0.03352726
RNASE1	0.71285448	0.03353026
NCOA4	0.16266407	0.03355244
PPDPF	0.26786578	0.03356104
CLEC5A	1.19615426	0.03356569
ZSWIM6	0.2708915	0.03427108
NETO2	0.30141641	0.03432088
GOLM1	1.76523882	0.0343416
SLC4A8	0.82442911	0.03449517
PHLDA2	0.27678862	0.03478716
WBP5	0.72882474	0.03489455
ATPAF1	0.37366419	0.03514455
BICC1	2.11644248	0.03518104
AFF4	0.20349746	0.03518995
ORAI1	0.28741377	0.03519432
MIEN1	0.1995574	0.03519432
DPP7	0.15604633	0.03522796
DNMT3A	0.52401264	0.03525357
TMEM80	0.37099113	0.03539102
RP11-356N1.2	1.60957733	0.03563601
VAV2	0.56206469	0.03570278
ADRA2B	0.70650135	0.0357033
NR1I3	1.52651141	0.03610017
INAFM1	0.34439501	0.03612026
RP11-597D13	1.27364385	0.03615104
RP11-4B16.4	0.99370442	0.03622001
E2F1	1.21831	0.03622001
CHST2	0.42159328	0.03628914
RP11-350G8.5	1.55656717	0.03634579
MOB4	0.25851026	0.03634579
BFAR	0.20890622	0.03635209
GLUD1	0.21337391	0.03688139
ZFC3H1	0.2299385	0.03688886

YTHDC1	0.16939261	0.03701972
PDCD1LG2	1.36763585	0.03723066
ID3	0.8546401	0.03736584
STK26	0.38742621	0.03737418
SETD9	0.26530214	0.03738062
FBXO38	0.43883036	0.03738122
FLCN	0.46390092	0.03770457
RP11-728F11.	2.24163514	0.03794107
TMED7	0.28809065	0.03804574
TYMS	0.97949514	0.03804574
TATDN3	0.42475926	0.03807406
PIK3R1	0.28628337	0.0382107
NANP	0.59383161	0.0382427
C9orf91	0.43462553	0.03860433
DUSP16	0.54387474	0.03860433
KCNK13	0.51277956	0.0388295
TAGLN	0.57433465	0.03885242
PRKCDBP	0.71376971	0.03888251
EIF2A	0.19132902	0.03894599
TPGS1	0.22184043	0.03905918
METTL1	0.38740008	0.0391392
MAP4K4	0.33218864	0.03916755
OSR2	0.95523193	0.0392275
EIF4A3	0.24102376	0.03927401
ACBD3	0.26702966	0.03953963
ASRGL1	0.56315751	0.03964083
GDE1	0.25969207	0.03978811
LINC00969	0.28232754	0.03988205
CCDC22	0.29652344	0.03989582
SENP7	0.38379723	0.03998374
STX5	0.24870831	0.04001817
SYT11	0.43167471	0.04017135
GAREM1	1.2818615	0.04017135
RP4-613B23.1	1.53171318	0.0403678
RP11-221J22.:	2.2913455	0.0403678
USP12	0.39724219	0.0403678
COQ10A	0.4911664	0.04044327
CAAP1	0.32370489	0.04055554
LRRFIP2	0.22235503	0.04062934
SPCS3	0.16284438	0.04082422
GOLGA4	0.20110826	0.04085736
RP11-561B11.	2.19238526	0.04087195
RP11-331F9.4	0.77573469	0.04095842
ATN1	0.40454618	0.04106729
METTL22	0.30234215	0.04120075
TNFAIP1	0.40362047	0.04122225
WWC3	0.58259282	0.04151156
ATP5O	0.15084503	0.04163861
C16orf54	0.59014169	0.04171753
FAM49A	0.2991812	0.04204245

SLC44A1	0.53456681	0.04232688
TIPRL	0.20311023	0.04235136
IL23A	0.98129648	0.04241628
TMEM87A	0.18877504	0.04269525
LYVE1	1.09786738	0.04278819
SGSH	0.40784595	0.04278819
CTD-2341M24	1.23800041	0.04279621
SLC45A4	0.74470199	0.04283428
RBMXL1	0.34498713	0.04287802
TRGV5	0.7917849	0.04304136
TRAF1	0.56989701	0.04305564
C19orf38	0.22845019	0.04326396
ARNT	0.41032731	0.04337382
LAPTM4A	0.17456232	0.0434211
UPK3A	0.51370694	0.0434211
GORAB	0.5882806	0.04376746
MTMR9	0.34719455	0.04377995
DLST	0.28459482	0.04393058
RNASEK	0.2213333	0.04412565
BEST1	0.5684849	0.04415956
KIAA0141	0.26869253	0.04432659
ZBTB7A	0.23361754	0.04444858
NAA15	0.21005357	0.04454798
TBC1D24	1.17305169	0.04454798
ABCC5	0.63791136	0.04465296
KIZ	0.60866417	0.04465296
NEAT1	0.20037123	0.04467772
COG5	0.33620062	0.0447186
AC005477.1	1.64786202	0.04476542
LAMP5	0.41099849	0.04476542
ATF7	0.28781501	0.04508442
HCLS1	0.15221272	0.04523168
GPI	0.22789231	0.04550351
FUOM	0.17208848	0.04551072
MACROD1	0.73292033	0.04586023
LINC00936	0.55959799	0.04596071
CCDC94	0.32110453	0.04637356
KLHDC8B	0.98585827	0.04664494
EPHB6	0.67937673	0.04664494
SLU7	0.23087738	0.04679217
FAM175A	0.29009421	0.04679464
BDH2	0.5380888	0.04679464
UBR5-AS1	0.60082515	0.04686093
SPATA5L1	0.40577228	0.0473079
CCL18	0.76363213	0.04737939
NAA38	0.17583853	0.04754101
SPRED2	0.46816977	0.04806932
MPC1	0.20047135	0.04822276
NDUFA11	0.09723257	0.04827681
DPH3	0.24047972	0.04853907

JAK2	0.32134792	0.04858025
MED29	0.24584333	0.04866912
MYO5A	0.30212893	0.04868343
C4orf27	0.36354543	0.04871348
GLA	0.26642167	0.04887506
SLC2A6	0.7842344	0.04899425
BRCA2	0.30149319	0.04947331
LMAN2	0.13206916	0.04948005
ZNF274	0.43187687	0.04949815
FAM127B	0.43605175	0.04952125
RP11-343H5.6	0.98083979	0.04974659
ITPKC	0.7041938	0.049861
DRG1	0.23372646	0.04992834
ZDHHC4	0.34793899	0.0499287

Table S6

			cDC3 vs M6		
**cDC3 DEGs**	**avg_logFC**	**adj. p-value**	**M6 DEGs**	**avg_logFC**	**adj. p-value**
CCR7	5.88666718	9.8813E-12	RP11-290C10.	-4.63237475	4.5111E-08
GPR157	4.35108912	1.6912E-17	LGALS8-AS1	-4.5589064	1.1954E-07
LAMB3	4.28095401	1.2811E-13	RP11-881M11	-4.3419963	2.4246E-05
CXCL11	4.24018998	4.2815E-09	RP11-307C12.	-4.25724938	3.1644E-10
CXCL9	4.13220403	5.737E-08	RP11-108M9.(	-4.23510781	4.455E-08
ABHD17C	4.12747198	4.2448E-08	C17orf105	-4.18171895	0.02619294
G0S2	3.97567633	1.1604E-17	CTC-209H22.3	-4.17871778	0.00629178
RAMP1	3.95312691	2.3847E-08	HEXA-AS1	-4.06278141	4.9449E-14
TFPI	3.94405826	7.8213E-08	GIMAP7	-4.02976172	8.647E-128
PDE4D	3.90269579	3.3721E-18	RP11-605B16.	-3.99526923	7.3233E-09
GJB2	3.85699584	2.89E-10	LINC01354	-3.94626007	0.01391305
IL7R	3.82557258	1.7735E-08	RP11-196G18.	-3.89970748	0.00010667
IRAK2	3.68701901	7.477E-10	PRMT5-AS1	-3.86113075	1.9281E-05
MIR155HG	3.59083014	1.4436E-09	RP11-473M20	-3.85103371	1.0666E-08
AQP9	3.57086543	0.00053926	RP4-798A10.7	-3.83106781	1.1956E-08
CCL20	3.56690294	7.4329E-07	RP11-77K12.9	-3.74509618	0.00335391
ENHO	3.55708752	2.5999E-07	RP11-387D10.	-3.711372	0.00765582
CXCL10	3.52705159	3.8661E-12	C2orf44	-3.70732871	0.00041797
TRAF1	3.47469431	6.8882E-12	RP11-1008C2:	-3.66196357	2.6021E-05
SLAMF9	3.44826757	7.2291E-12	SMTNL2	-3.63336004	0.00129764
TNIP3	3.34979994	9.4251E-06	RP11-338I21.1	-3.6260191	1.6E-06
SLC16A10	3.28864168	6.4513E-09	MORF4L2-AS1	-3.60198735	0.01057765
AC002456.2	3.22726283	0.00026469	RP3-486I3.7	-3.52177423	0.0005507
DUSP14	3.21204555	4.2128E-06	CPNE5	-3.50829555	0.01275654
ZC3H12C	3.12657836	2.513E-07	RP11-439A17.	-3.50788455	0.0479589
STAT4	3.11161928	6.907E-10	CTD-2165H16	-3.49254227	1.3748E-06
EMP1	3.10219516	2.0159E-10	ZKSCAN3	-3.48956005	4.356E-05
CRLF2	3.09426108	0.00020596	XXbac-BPGBP(	-3.48628002	6.919E-10
SLC7A5	3.09113411	5.2795E-21	TDRD10	-3.47951987	0.04634404
SDC4	3.04658636	4.6392E-12	RP11-277B15.	-3.45678577	0.03644805
APOBEC3A	3.03642386	3.8805E-08	CTD-2410N18	-3.44715908	0.04883165
THAP2	2.99498739	6.4668E-21	PARS2	-3.44530783	0.01288728
UBD	2.98724738	0.00072796	ZNF812P	-3.41912054	2.5837E-06
PLAUR	2.91169112	2.5455E-69	MIR1539	-3.40568065	0.02523815
FAS	2.89981303	1.1419E-10	RP11-46H11.1	-3.39192072	3.3244E-06
HAS1	2.8972579	0.03680177	CCDC80	-3.37261121	0.01901534
FLT1	2.88613754	1.1122E-07	RP11-390P24.	-3.34057603	0.00875671
PRDM8	2.86522151	1.1561E-06	RP11-701P16.	-3.32467507	1.6563E-07
RP11-519G16.	2.86005357	0.00054513	MRM1	-3.31733318	1.3827E-07
CXCR3	2.8433813	1.8527E-05	AC105760.2	-3.29347345	0.00031066
SH2D3A	2.84223423	1.9567E-05	CTD-2256P15.	-3.29089226	0.00049434
RP1-239B22.5	2.83358478	1.1561E-06	VGF	-3.28698095	0.00586109
CREM	2.8277898	4.685E-48	RP11-231D20.	-3.28194134	3.7557E-06

PPP1R3B	2.80208571	5.4797E-10	RP1-265C24.8	-3.27648912	0.00842621
SYTL3	2.79795411	6.4604E-24	KCNJ11	-3.26892221	0.0375317
NRARP	2.7878998	4.9934E-25	PDK4	-3.24836933	1.0949E-56
EREG	2.78464601	3.3758E-19	ZNF133	-3.24813316	0.00171326
TMEM217	2.77864617	8.7195E-06	SMAD6	-3.2428505	0.00355656
DUSP4	2.77145036	5.0653E-21	ZNF624	-3.18099793	7.4421E-08
ZNRF1	2.75394566	9.1198E-07	CCDC36	-3.17552589	0.01690324
MIR3945HG	2.74037185	1.4722E-08	ZNF239	-3.17540496	0.00785546
XKR8	2.7393029	1.1704E-14	COL4A2-AS2	-3.15072832	2.9625E-06
LYPD3	2.72932352	1.3752E-13	RP1-206D15.6	-3.13751788	1.9201E-08
MAP4K4	2.72872485	5.9459E-17	GAL3ST4	-3.13665218	2.7313E-06
SLC35E4	2.72775618	4.5878E-06	IGSF21	-3.12433685	0.00135913
LY6K	2.72168222	5.3799E-05	FCAMR	-3.12373222	0.01740147
AP1S3	2.71636696	4.7085E-12	RP5-1148A21.	-3.12072191	9.6523E-05
LINC01315	2.71134036	0.00013765	NKILA	-3.11989105	0.04184451
TMEM158	2.70094245	2.2487E-06	RP11-477D19.	-3.09391029	0.03179252
RP11-79H23.3	2.68740141	4.2396E-05	GHET1	-3.09292045	0.00339296
CES1	2.68372458	0.00121327	PDIK1L	-3.08784713	7.2024E-08
NRIP3	2.68089238	4.3493E-09	EGR2	-3.07502451	8.9503E-21
CST7	2.68089112	5.0333E-20	RP11-489O18.	-3.07435125	2.6032E-11
GPRC5A	2.6636438	0.00027588	LINC01554	-3.07224616	6.2068E-06
MCEMP1	2.6592382	2.9245E-09	LINC00891	-3.05550767	0.00894249
SLC16A6	2.637604	1.992E-07	FRS3	-3.05120544	0.00035682
MIR22HG	2.62563084	3.5049E-14	ZNF563	-3.04491812	0.00023651
C15orf48	2.61561997	1.9035E-09	RP1-28H20.3	-3.03607098	0.0031302
GALR2	2.60703947	1.4744E-07	SHE	-3.0248776	1.2513E-07
PHLDB1	2.60157454	4.4078E-07	ATP6V0E2-AS1	-3.01345563	0.03719308
GYPC	2.60100265	1.5431E-16	ZBED8	-3.00988767	0.02101464
RHCG	2.59717647	0.00132112	ZNF594	-2.99525995	0.01597342
MALT1	2.59441475	1.9778E-33	RP11-37C7.3	-2.99252303	0.00141666
AC058791.1	2.58958695	5.1584E-14	RP11-474I16.8	-2.98510187	0.00014346
EZR	2.58651194	5.7174E-59	TMEM26	-2.96720695	0.01773618
ZC3H12A	2.58276587	5.9788E-17	RP11-393I2.4	-2.96014358	2.46E-05
AC016831.7	2.57921342	6.4278E-15	RP11-412D9.4	-2.94805091	0.03722377
TOP2A	2.57325462	0.00089543	CABP4	-2.94669882	0.00176779
DNAAF1	2.57238409	1.5676E-08	LRRC69	-2.92726093	0.00088236
NCR3LG1	2.55209623	1.4105E-09	PDP2	-2.92284896	0.00493446
GCH1	2.55064756	3.5213E-13	CTD-2302E22.	-2.9172237	0.00060616
IFFO2	2.54508231	6.9014E-08	RP3-510H16.3	-2.9114061	0.02069092
HPGD	2.53086222	0.00105901	C9orf106	-2.90942266	0.02401011
SLC7A11	2.52038243	0.00070447	MSS51	-2.90700623	0.04708234
C14orf80	2.51030316	3.0992E-06	TXNIP	-2.9033927	8.0575E-43
CDC42EP2	2.50224893	1.9158E-13	RP11-289H16.	-2.89933313	3.1137E-07
ANKRD33B	2.49192201	0.00033556	DHFRL1	-2.89148418	0.00057538
UPK3A	2.48995648	1.4834E-07	RP11-596C23.	-2.89020338	2.8039E-17
METRNL	2.47264857	5.5563E-37	RP11-152N13.	-2.88647551	6.1856E-05

SLAMF1	2.46357224	0.00647439	MYH7B	-2.88637339	0.03374822
BCL2L14	2.46247329	0.00102465	RP11-758H9.2	-2.88626531	1.8023E-26
RGCC	2.44845674	1.8255E-13	ZNF658	-2.87843379	9.6211E-05
HAPLN3	2.44534444	4.4718E-09	CTDSPL	-2.87783445	2.904E-06
TNFAIP6	2.44373966	0.00032607	CD163L1	-2.87774873	3.9323E-15
SLCO4A1	2.43458529	1.0827E-06	RP11-196G18.	-2.8762065	0.0014248
VDR	2.43152476	9.5289E-13	CTD-3193K9.4	-2.87512025	0.00330279
SYN1	2.41458552	0.00090658	AC145124.2	-2.87154403	0.01861766
MTMR11	2.41435894	9.5078E-06	CTD-2376I4.2	-2.86959602	0.00047218
CCL22	2.41227347	0.00895057	LTBP2	-2.85236835	0.01074385
S100A12	2.41161681	0.00162017	RP11-624L4.2	-2.84357152	1.5405E-14
BCL3	2.40799591	5.2405E-20	RP11-967K21.	-2.83771034	0.00062102
TNFRSF9	2.40093226	0.00024977	MZF1-AS1	-2.83656238	1.5598E-10
TYMS	2.4004761	0.00019746	RP11-739B23.	-2.83398869	2.2868E-08
PDE2A	2.36513416	6.0428E-05	AATBC	-2.83106057	0.00080541
PIM3	2.35894697	8.6007E-19	ZNF485	-2.82585598	6.0691E-05
IL1R2	2.35141476	2.5672E-23	RP11-553A21.	-2.82347654	0.01382342
CHST2	2.35102813	1.8247E-10	RP11-422P24.	-2.81315023	2.5798E-05
AGO2	2.35082746	1.5277E-24	AC006116.17	-2.80909205	0.02105831
MIR3142HG	2.32295633	0.00078006	RP11-57G10.8	-2.80897372	0.00268683
AKAP12	2.31526692	0.02987826	FRMD4A	-2.80010076	7.2745E-17
GK	2.30348803	1.5587E-17	HIST1H4I	-2.79329676	0.01554125
PDE4B	2.29271151	1.5987E-36	CIART	-2.78639375	0.00174693
MFSD2A	2.29197425	3.8534E-06	RP1-102E24.8	-2.77708274	0.00729098
PIM2	2.29070431	4.3612E-14	LILRB5	-2.77388194	5.1625E-12
BASP1	2.28649148	9.6417E-17	PTOV1-AS1	-2.77032451	0.00058249
AGPAT4	2.27686273	3.7493E-08	HIST1H2BG	-2.75145911	1.0193E-09
ZBTB43	2.27304123	3.9276E-25	ADORA3	-2.74960873	5.453E-32
IGLON5	2.25318372	0.004928	ZNF737	-2.74326819	0.0315642
CD2	2.24733671	0.00036044	RP11-977B10.	-2.74018251	1.726E-08
SLC2A6	2.23532423	2.3805E-05	GNAL	-2.7323796	7.1914E-05
FOSL1	2.23520546	6.6825E-06	SOWAHD	-2.72533956	4.4046E-08
WDR59	2.2345272	2.8448E-12	RP11-297N6.4	-2.71019771	9.7455E-06
TMEM8B	2.2200273	3.8548E-05	RP11-362F19.	-2.70904058	0.00429659
GPR146	2.21895966	1.8955E-05	RP11-422P24.	-2.69872653	0.01098698
C6orf223	2.20876839	0.01413714	BBS12	-2.69416455	0.00022996
RALA	2.20814551	8.8429E-22	SLC41A1	-2.69104011	0.00142372
ISG20	2.20764758	3.6911E-35	RP11-471B22.	-2.68316104	0.00146961
AKR1C3	2.19568209	0.00513842	KIF3C	-2.68189056	0.04850823
CD300E	2.18022478	3.0367E-13	POLN	-2.67619231	0.04845018
PTPN1	2.17836709	4.0877E-30	PPFIA3	-2.67413808	0.00017863
DUOX1	2.17422186	3.4248E-05	RP11-480C16.	-2.67239686	0.01806405
SAMSN1	2.17223729	4.893E-82	RBMS3	-2.66668809	0.01224172
RP11-598F7.3	2.16723946	9.9821E-05	SLC44A4	-2.65884343	0.04788266
GPR35	2.14730456	1.1364E-13	RP11-567M16	-2.65818167	2.7394E-08
RIPK2	2.14147279	3.9166E-51	RP11-370I10.1	-2.65601619	6.0636E-05

STX11	2.13497878	2.1665E-47	JUN	-2.65515033	1.9097E-18
RP11-147L13.	2.12317259	5.0801E-05	TMEM79	-2.64526532	0.00019746
CD274	2.12271568	0.00113587	WDR5B	-2.64289088	1.5747E-06
IRF4	2.1171449	1.5095E-16	ZNF366	-2.64005965	6.1075E-12
IL23A	2.11611959	0.00013615	GIMAP8	-2.64004658	5.0824E-37
LAYN	2.11230612	0.00250934	NYNRIN	-2.63937474	0.01714774
ATP1B3	2.10546955	1.0864E-33	COL4A4	-2.62821305	0.01430755
SPHK1	2.09806439	4.1323E-06	ZNF382	-2.62777507	0.01868926
HES4	2.09172545	3.3642E-14	ZNF691	-2.62549048	0.00028197
ELL2	2.09038666	3.0194E-17	KCNMB3	-2.61649917	0.0023579
FURIN	2.0901852	1.2084E-09	C1orf109	-2.61364703	2.8686E-13
KCNN4	2.08892804	3.2251E-05	CXCL12	-2.60367077	5.1775E-12
KBTBD11	2.08360986	4.7644E-05	ZNF253	-2.59294626	2.1467E-06
NKX3-1	2.07666807	0.01761828	NAPSA	-2.59181649	0.00080126
SHB	2.07374586	4.2527E-07	ZNF605	-2.5917649	2.9112E-08
NR4A3	2.07056168	3.9479E-17	C3orf18	-2.59095228	0.01355607
TLE1	2.06962391	8.9348E-05	FAM53A	-2.58940033	0.01341917
PLCXD1	2.068328	3.6746E-07	ZNF442	-2.58747302	0.03596191
LIPN	2.06618335	0.04754149	RP11-63L7.5	-2.58484517	0.03713959
TNFRSF4	2.05788543	0.00210446	LPAR5	-2.58474255	0.00245074
HACD1	2.04376954	0.00477895	LINC00996	-2.58408029	8.8429E-22
RILPL2	2.04331685	1.2775E-40	SLCO2B1	-2.58367163	8.2086E-21
NFKB1	2.04174843	3.2685E-20	TMEM37	-2.57471238	1.8506E-05
CLCF1	2.04023363	0.02877006	RP11-325N19.	-2.56581387	0.04874389
MCOLN2	2.0389633	4.9334E-07	HIST1H3A	-2.56482463	0.01650911
TNFSF14	2.03788022	8.782E-05	HIST3H2BB	-2.55946739	0.00519553
NOCT	2.03361706	0.00012401	GIMAP5	-2.5591987	5.206E-12
TREM1	2.03249973	2.8584E-16	C19orf35	-2.55730042	4.5828E-06
TUBB2A	2.02796542	5.1976E-09	APOC2	-2.54019534	0.00323745
EHD1	2.02794674	3.1601E-08	ADAMTS6	-2.53872896	0.00402557
AURKB	2.02542287	0.02096646	RP11-596C23.	-2.53825021	0.04746782
FADS3	2.0222753	1.6304E-05	RP11-573D15.	-2.53577159	0.00059996
NEU4	2.01514115	8.1207E-05	CCDC121	-2.53451787	3.1174E-07
DENND4A	2.00855379	5.1578E-14	CMKLR1	-2.53367812	3.3703E-27
PPIF	2.00792166	1.2515E-23	RP11-390F4.3	-2.52826364	6.8518E-05
SLC35F2	2.00124335	0.00115851	RP11-123K3.9	-2.52001332	0.03936427
BCL2A1	2.00086986	4.7312E-09	ATP8B1	-2.51712294	0.01691943
FAM134B	1.99796809	0.00319605	ZNF420	-2.51422377	2.6857E-05
RHOU	1.9970277	1.8065E-06	ZNF343	-2.50997591	0.01426652
MXD1	1.98432894	1.0517E-36	ZNF57	-2.50965927	0.00353286
CXXC5	1.97738601	2.783E-06	RP4-742C19.1	-2.50647163	0.03458435
NOV	1.9757616	0.00583125	ZNF500	-2.50120441	1.0646E-08
SERPINB9	1.96035843	1.0769E-32	CTD-2516F10.	-2.49992599	0.03914249
TMEM120B	1.9542311	0.00013475	ZNF619	-2.49212417	0.00353803
CCNE2	1.95278632	0.02509626	HIST1H2BN	-2.48849408	2.1019E-13
NINJ1	1.95021485	8.6895E-17	CCL24	-2.48742723	0.00749285

FYN	1.9408526	3.7493E-08	RP11-359K18.	-2.48625686	0.04974249
KIF23	1.93810155	0.01850706	AP000769.7	-2.48424123	0.01993146
CD55	1.93672742	9.0685E-24	SEPP1	-2.47979309	1.029E-10
FAM65C	1.92980786	0.03331434	ZKSCAN4	-2.46104267	7.4961E-06
SLC7A1	1.92851408	1.4587E-07	BCAM	-2.45934354	0.00017331
USP12	1.92671665	4.997E-10	RP11-679C8.2	-2.45068873	0.04846975
UBE2C	1.92450952	0.04498839	FABP1	-2.44276338	3.1674E-05
ARNTL2	1.91363627	0.02086213	RP1-191J18.6(	-2.44247377	0.00095519
RAB7B	1.912421	0.00039468	ZNF501	-2.441285	0.00291739
IL1RN	1.90482241	0.00011561	KIAA0753	-2.44095611	3.1795E-06
FOSL2	1.89967912	1.2107E-44	PLXDC1	-2.43844272	0.00235885
TUBB4B	1.89119634	4.5136E-20	RP11-77P6.2	-2.43778012	0.0360778
IL4I1	1.88170954	2.0236E-06	ZNF775	-2.43716266	7.9312E-06
ZNF267	1.88106648	2.6918E-33	GPR82	-2.43665259	3.6471E-22
NEURL3	1.87893796	0.01235849	RP11-48B3.5	-2.43471203	4.4519E-08
AUNIP	1.87791662	0.02703627	ZBTB37	-2.42153985	2.1094E-15
FAM222A	1.87651147	0.02163681	RP11-486O12.	-2.4124424	0.01614752
IDO1	1.87295932	5.2725E-06	ZNF571-AS1	-2.40949842	0.03449164
TRMT6	1.87280416	1.7054E-08	EDA	-2.40794693	0.01426652
SOD2	1.8696279	1.5394E-24	RP13-582O9.7	-2.40579542	0.04250583
FAM46C	1.86870893	0.0030668	RP11-158H5.8	-2.40514934	0.04672584
ANKRD28	1.86842232	4.1178E-10	HCG27	-2.40436103	0.045604
SELK	1.86391333	5.8029E-58	BRIP1	-2.39512604	0.02391851
LMNA	1.86287904	9.5083E-16	PHGR1	-2.39323947	1.3828E-08
HS3ST3B1	1.86167863	1.4877E-05	RP11-379K17.	-2.39269731	0.04941542
CTB-61M7.2	1.86039505	0.00019663	ZNF441	-2.39257738	0.00039857
COL4A2	1.85958731	0.01452838	AP001372.2	-2.38917033	0.02119649
MPP7	1.85951731	0.00119949	RP11-11M20.4	-2.38730417	0.00372776
KIFC1	1.85917446	0.00295671	C21orf58	-2.38705848	0.00043507
FBXO34	1.85575849	6.522E-12	MBLAC2	-2.38185479	0.00047236
UBASH3B	1.85415269	1.5738E-07	RGS5.1	-2.37211667	0.01263668
ICAM1	1.85053076	9.267E-16	AC142528.1	-2.37028304	0.00028984
ETV7	1.84884898	0.00335037	NRP1	-2.36763696	1.2658E-13
SLC22A4	1.84724083	0.00274732	LGALS4	-2.36645536	0.00021829
PTX3	1.84421703	0.02556504	ABCG2	-2.3639216	2.2347E-05
MARCO	1.84375706	0.00261314	OSGEPL1	-2.36259017	2.148E-07
VEGFA	1.8432684	4.9907E-23	AC062029.1	-2.36147812	2.0266E-05
THBS1	1.84322429	6.7994E-17	ZNF792	-2.36109601	0.02283579
INHBA	1.84174467	0.00200845	GIMAP1	-2.36014223	2.4499E-27
DNMBP	1.82400723	0.00023499	GPR55	-2.35152371	3.5666E-06
FAM49A	1.81994904	2.4499E-27	NKAPL	-2.34947317	0.00062102
GNA15	1.81799291	7.3296E-16	PLA2G2D	-2.33856447	0.01480535
RP11-126O1.5	1.81470036	0.02786957	HECTD2	-2.33451931	7.6387E-05
CHRNE	1.81228406	7.9404E-09	PLEKHH3	-2.31977673	0.00017701
SHF	1.8088493	1.886E-05	APCDD1	-2.31664603	0.02345493
NFKB2	1.8085516	5.8517E-17	STAB1	-2.31424684	7.0102E-48

GBP5	1.80613197	3.284E-07	CYTL1	-2.31342836	0.01031801
TMEM88	1.80517601	1.8198E-08	C10orf95	-2.31175936	0.00019915
IL3RA	1.80189477	3.2604E-09	RP11-307C12.	-2.30902258	0.03932077
SQLE	1.8016837	1.7934E-08	PLS1	-2.30549143	0.04820878
EPOR	1.79982788	0.00140143	CTA-228A9.4	-2.2997072	4.078E-06
ARSG	1.79591384	1.8859E-06	ABCB9	-2.29923756	0.0071922
ADRA2B	1.79006957	0.00648896	ZNF354C	-2.29200712	0.02559553
SMAD3	1.78105198	0.00171388	MANEA-AS1	-2.29064058	0.0001169
SYAP1	1.7807216	2.0278E-41	ARMC7	-2.28977215	1.1858E-10
AVPI1	1.77874933	7.5598E-15	PLA2G15	-2.28344076	4.2045E-11
RP11-138A9.2	1.77540912	4.5015E-16	ARRDC4	-2.27786714	5.1133E-12
CD44	1.7752374	2.2783E-30	RP11-467L13.	-2.27715782	2.6644E-14
CTD-2006K23.	1.77311013	0.0001615	RP11-182J1.1	-2.27255556	0.01078222
FGFR1	1.77310519	0.02889697	BNC2	-2.26900316	0.00140705
PDE4A	1.76914777	6.3712E-09	GDPD3	-2.26868294	0.04426943
THBD	1.76762328	2.7249E-07	TRMT5	-2.26761014	1.0194E-05
GPR132	1.76639888	1.3194E-10	SPSB2	-2.26731306	3.7662E-07
ZNF674-AS1	1.76557099	0.00096726	RP11-693J15.	-2.26360627	8.0066E-05
KIF18A	1.76518015	0.04206738	RP11-1017G2	-2.26350557	1.2116E-08
TPRA1	1.75761418	1.4268E-08	HSPA1L	-2.2583212	0.03321111
RP11-468E2.4	1.75704549	2.8677E-06	KCNE1	-2.25742469	6.1906E-10
ETV3	1.75439417	5.6247E-18	YAE1D1	-2.25546615	4.4223E-08
B4GALT5	1.75312456	4.3553E-09	WLS	-2.25540589	3.9793E-07
SELL	1.75258928	3.3975E-08	CTD-2547L16.	-2.25539199	0.0072043
RELB	1.75191352	3.603E-09	LRP2BP	-2.2536394	3.7434E-05
MAP2K3	1.74908377	3.9387E-10	ILF3-AS1	-2.25269016	3.0324E-36
CHRNA10	1.73670833	0.02883738	RP11-212I21.	-2.25181469	7.621E-08
VCAN	1.73397195	6.4669E-06	BBS10	-2.25005005	3.0539E-07
DNAJB5	1.73076191	0.00280471	HSPA1B	-2.24994852	7.0546E-08
SPIB	1.72863006	2.0744E-05	DISP1	-2.24619714	0.00162057
SDPR	1.71904906	3.3466E-05	ZSCAN9	-2.24612008	5.5408E-07
ENPP4	1.71887568	0.00075605	RP11-45A17.2	-2.23973354	0.0217566
RP11-372K14.	1.71317648	0.03255616	RP3-522D1.1	-2.23791396	0.00149969
RFX2	1.71110921	0.00039444	DNASE1	-2.23733786	0.00030831
CYTH3	1.70936293	0.00038351	ZNF816	-2.23672784	0.00131848
RABGEF1	1.70912002	1.0215E-05	ZNF345	-2.23617762	0.00246083
BIRC5	1.70743162	0.02388939	PLAG1	-2.23033737	0.01931117
AIM1	1.70666014	8.9858E-18	RP11-823E8.3	-2.22064856	0.00225951
INSIG1	1.70407009	6.6776E-34	L3MBTL1	-2.21750838	0.00093516
REL	1.70279255	2.4957E-25	RP11-318A15.	-2.21736443	0.00572275
KLHL25	1.70013904	0.03768407	RP11-793H13.	-2.21723501	4.9259E-06
SNN	1.69831576	5.105E-14	RP11-154H23.	-2.21701021	0.0008484
DUSP5	1.69756172	1.815E-21	MTBP	-2.21511688	0.01518876
FPR2	1.69127468	0.02707429	HSPA6	-2.21218386	0.00011897
MSANTD3	1.68873661	0.00608134	CTC-232P5.3	-2.21046205	0.03549346
CCDC107	1.68659093	5.8427E-28	TAF1A	-2.20985266	4.1716E-05

HIVEP1	1.68640413	4.9761E-06	LEKR1	-2.20746228	0.01687691
BZRAP1	1.68485301	0.01283007	RP11-365P13.	-2.19826727	0.00057538
AREG	1.6843343	2.1486E-26	RP11-588H23.	-2.19651577	0.0311631
STK26	1.68394489	2.3926E-05	FAM174B	-2.19216304	0.01566972
GRASP	1.68359799	2.1119E-10	CACNA1A	-2.1864013	0.04747866
RASSF5	1.68296013	3.0171E-23	STAM-AS1	-2.18357819	0.00161176
NEGR1	1.6828044	0.01088244	RP11-379F4.9	-2.18211574	0.00043759
CD7	1.6799004	0.00020252	GDF15	-2.18119203	0.00958501
MT2A	1.67430863	7.1295E-17	NRAV	-2.18012678	0.0217741
VIM	1.67430836	3.7289E-40	CABYR	-2.17951968	0.03575729
TRAF5	1.67075766	1.7817E-09	PTPRM	-2.17364933	0.01354987
RP11-737O24.	1.66655053	0.00268683	RP11-1E4.1	-2.17295782	0.02370473
NDEL1	1.66096303	2.1088E-22	GIN1	-2.17189014	1.9391E-06
PTTG1	1.65807542	1.1205E-11	FAAP24	-2.16998597	7.9423E-05
NLRP3	1.65665807	7.4109E-11	GIMAP6	-2.16959107	2.7939E-14
TP53BP2	1.65048537	1.5448E-18	RP11-293M10	-2.16956445	0.0014126
REV3L	1.64784204	5.5026E-15	RP11-511P7.5	-2.16439256	0.000825
LILRA5	1.64662513	6.4462E-11	COQ7	-2.15731719	2.4552E-25
ITPRIP	1.64502697	1.8923E-11	SLC47A1	-2.15528302	0.03365597
DFNB31	1.64158878	0.00452602	HES1	-2.15474719	7.8097E-28
SPRED2	1.63772053	6.1129E-05	MAF	-2.15069016	2.8584E-16
CTD-3203P2.3	1.63168602	0.02348455	ZNF324B	-2.14972823	0.00166985
ACSL1	1.62898062	1.3059E-20	PAQR8	-2.14601462	5.6276E-12
PKP2	1.62462869	2.7898E-13	GPAM	-2.14477122	0.00206323
EPHB6	1.62395408	0.03546591	ADGRG6	-2.14118868	1.8932E-07
TESK1	1.62114818	1.9339E-07	ITGA9	-2.13923231	4.3131E-08
KDM6B	1.61988419	3.1991E-30	LOH12CR2	-2.13843627	3.5689E-06
SH2B2	1.61659332	0.00016677	RP11-18H7.1	-2.13800164	0.02289125
BID	1.61021941	1.4814E-27	ZNF19	-2.13709143	0.01159284
GBP1	1.60797962	4.9877E-15	ZNF234	-2.13587012	0.00043659
SOCS3	1.60721652	2.405E-38	B3GNT8	-2.13520557	2.2475E-05
RAB11FIP1	1.6036853	2.8603E-25	ABCC6	-2.13396626	0.01287576
RAB20	1.60052475	1.4596E-27	INHBA-AS1	-2.13301184	7.8068E-06
C12orf75	1.59779926	0.00011222	TIGD7	-2.13155227	0.03748617
S100A9	1.59779214	0.00067647	ZNF502	-2.1300498	0.02069092
ADAM8	1.59724562	3.9842E-09	UBE3D	-2.12956623	0.00090266
PIM1	1.5950706	8.4408E-08	KIAA1211L	-2.11956326	0.00119635
CRY2	1.58502291	3.2776E-05	MYLK-AS1	-2.1171502	0.03903919
CFLAR	1.58357053	2.1348E-18	ZNF671	-2.11535452	9.2243E-05
SEMA6B	1.581011	2.6809E-16	ZNF567	-2.1120971	3.8172E-10
CXCL14	1.58026845	0.02167633	RP11-120K19.	-2.10806655	0.02374826
CCRL2	1.57990338	2.3345E-08	HIST1H2AG	-2.10612644	0.00176689
TUBB6	1.5793828	1.9966E-14	EGR1	-2.10412464	5.2146E-10
GPR84	1.57884793	1.005E-05	RP11-66B24.4	-2.10146631	0.01149425
SATB1	1.57842938	1.84E-10	PLPP6	-2.10036221	0.0009596
AKAP13	1.57536997	1.4518E-27	ZNF799	-2.09776346	0.00635203

PRR7	1.57369531	0.00194589	RP11-400F19.	-2.09613677	2.2347E-05
EMP3	1.57166265	5.1511E-28	FHAD1	-2.09129181	2.1321E-05
SEMA7A	1.56996483	0.00082103	GPR155	-2.08732882	5.5139E-20
RHOF	1.56955608	9.563E-05	TRIM68	-2.08207308	0.01843438
SLC2A3	1.56554321	1.3859E-14	DAAM2	-2.0789965	0.03851553
SNAI1	1.56406733	3.2874E-05	RDH13	-2.0787202	0.00844925
ITGA5	1.56290067	0.00021742	PACERR	-2.07659808	3.8354E-05
SH3BP4	1.5604913	0.00015752	POC5	-2.07633018	3.128E-09
MLF1	1.55870873	0.0004676	WNT5B	-2.07141863	1.0942E-06
SULT1B1	1.54504143	0.04495723	PEG3	-2.06744972	0.04083615
GPER1	1.53642547	0.00092208	ZNF616	-2.0656321	0.00097787
SIK3	1.53390223	2.643E-08	RP1-187B23.1	-2.06184306	0.04642665
PRC1	1.52860808	0.00037099	RWDD2A	-2.06008428	0.00736686
FAM225A	1.52786467	0.03807032	C17orf80	-2.05779848	7.1746E-05
GFOD1	1.52685275	4.5837E-05	CTD-2506P8.6	-2.05775688	0.00284914
BTG3	1.52231599	3.805E-07	FCGR3A	-2.05520743	1.5676E-08
ATP13A3	1.52208068	6.7722E-13	SLC35G2	-2.05339287	0.02415594
FPR1	1.52145844	1.9437E-07	CISH	-2.05059975	3.0429E-06
PHLDA2	1.51986342	9.571E-10	FOS	-2.04903363	3.6578E-26
PIGA	1.5189114	9.163E-06	RP11-727F15.	-2.04494959	0.00220719
MPHOSPH6	1.51609593	1.8255E-13	USP49	-2.0430625	0.00036867
SFT2D3	1.51465083	4.612E-06	GPR34	-2.03999001	5.0416E-17
OXCT2	1.51323216	6.4475E-06	RP11-727F15.	-2.03963379	0.01328638
CEP68	1.51072447	2.5878E-13	LAG3	-2.03810366	4.1184E-05
KLHL21	1.50269936	0.00466365	IER5L	-2.03773031	1.5834E-12
JARID2	1.50245946	1.8495E-17	KCTD21	-2.03693458	0.03559778
SERPINB9P1	1.50190076	0.02739717	RP11-191L17.	-2.03565616	0.00267129
AAED1	1.50151205	6.2698E-22	CCR2	-2.03496238	3.6363E-10
LUCAT1	1.50103617	0.01558793	ACVR2B	-2.03173809	0.01159284
DYRK3	1.50083434	0.03855308	SNAI3	-2.02998569	2.9462E-12
GTF2B	1.49822448	5.0896E-21	ZSCAN12	-2.02919463	0.00420301
PHF1	1.49471144	1.7141E-05	ZNF174	-2.02899434	0.00049293
PDLIM1	1.49360306	0.00079592	NBEA	-2.02658357	0.02446795
OCIAD2	1.49037885	0.01193806	EPHB2	-2.0226251	5.4299E-06
CD52	1.49033287	4.3472E-09	ZNF559	-2.02111496	1.5296E-06
RP11-76E17.3	1.48592201	5.4724E-07	SLC7A8	-2.01700544	1.7115E-06
PLEK	1.4843097	6.2278E-14	RP11-110I1.12	-2.01688982	0.02794964
PTGIR	1.47761175	0.00341217	SYP	-2.01682871	0.00941177
CDKN1A	1.47662075	1.2927E-20	PLEKHG3	-2.01621233	0.00145576
NFE2L2	1.47462571	6.9562E-28	LIPT1	-2.01107203	1.6304E-05
HGSNAT	1.47276706	1.4432E-25	DAB2	-2.01083374	2.8231E-17
FAM65B	1.47240499	0.00096818	POU5F2	-2.01012895	0.01216644
CD1C	1.46975406	7.3512E-10	RP11-73K9.2	-2.00286769	0.00894227
KIAA0101	1.46638171	0.00167432	SIGLEC12	-1.99949297	3.4691E-09
EIF2AK3	1.46565764	7.7566E-06	RP11-182J1.3	-1.99898166	0.04522956
SMCR5	1.45825643	0.00578646	C10orf2	-1.99790347	0.03180997

MAP3K8	1.45453705	9.8813E-12	MERTK	-1.99772795	1.626E-09
ARL4C	1.45073716	5.7464E-23	TSPAN10	-1.99586816	0.00063758
PMEPA1	1.4506231	0.00062209	RP11-474G23.	-1.99302913	3.8339E-07
OASL	1.44916228	0.00232584	RN7SL832P	-1.99190667	0.00285555
GNA12	1.44698629	4.2334E-14	ZNF546	-1.9904714	0.00044957
ARFGAP3	1.44648695	3.041E-27	ZNF436	-1.99036973	0.0484523
ICAM3	1.44371289	5.7055E-07	GSTT2B	-1.98383149	0.01891977
SESN2	1.4422503	2.513E-07	ZNF891	-1.98316059	0.00076673
SNX9	1.43624842	4.8458E-10	STK36	-1.98254131	0.00078267
MEI1	1.43583168	0.03412313	RP11-297D21.	-1.98098266	6.2116E-07
MB21D2	1.4314195	1.3499E-05	RP11-707G18.	-1.98096702	4.86E-10
SLC38A1	1.42959623	1.0546E-09	ALKBH4	-1.97992617	1.6648E-05
TRAF4	1.42933329	0.00036695	GS1-124K5.3	-1.97992443	0.0057479
SERPINB1	1.42790809	1.0667E-19	SLA	-1.97938196	2.1665E-47
GEMIN4	1.42350999	0.00268347	TNS1	-1.97583975	0.00711995
TNFRSF10B	1.42342181	1.5337E-16	RP3-466P17.1	-1.97530007	0.02714399
EZH2	1.42227658	2.5897E-06	NHLRC2	-1.97430907	4.7987E-16
HIVEP2	1.4218007	0.00073292	PHF23	-1.97225875	1.9452E-11
MMP19	1.42119476	9.7742E-05	LINC01220	-1.96825771	0.00051115
RP11-138A9.1	1.42089307	6.2654E-10	RP1-151F17.2	-1.96643758	0.03071301
CDC42EP4	1.41782218	0.00538974	ABCA6	-1.96625997	0.01524345
UTF1	1.41710949	5.2123E-05	ZNF66	-1.96575556	0.02558336
PDE4DIP	1.4170209	2.4917E-06	NEK8	-1.96466844	0.0178626
PFKFB3	1.41477685	2.4422E-19	ARL4D	-1.9645554	0.00044
C5AR1	1.41341061	1.0667E-19	PRRG2	-1.96380853	0.00583699
BEX2	1.41334202	0.0164472	METAP1D	-1.95876608	0.04806636
ATP1B1	1.40922852	1.0946E-08	KRBA2	-1.95507448	0.02809666
ARL8B	1.40601795	9.7409E-12	ZNF525	-1.95336437	0.00047731
SDC2	1.40530067	0.00027687	CAGE1	-1.94976082	0.01427539
BCL11A	1.40507044	0.00021556	CTTNBP2	-1.94376226	3.4369E-14
AEN	1.40483252	6.0372E-05	CCDC102A	-1.94099763	0.00452621
LINC00528	1.40171142	2.9804E-07	CSRP2BP	-1.93837542	8.2196E-05
RRAD	1.40084345	0.00015313	ZNF397	-1.93530423	4.6828E-10
BTG1	1.39798192	2.2386E-39	DCBLD2	-1.93496747	0.00083376
SLC39A8	1.39764724	0.00570858	ME1	-1.93425064	2.806E-05
WTAP	1.39615001	5.6156E-23	ZNF77	-1.93279149	0.01727962
HOXA5	1.38792433	1.8622E-05	C2	-1.93167227	5.6736E-06
REC8	1.38636803	5.1494E-05	RP11-597D13.	-1.93074393	9.423E-05
TK1	1.38551512	0.04908803	CD200R1	-1.92703308	5.6443E-05
SLC25A51	1.38381508	0.00012297	SLC40A1	-1.92016397	3.1019E-09
GPR183	1.38224212	2.9666E-15	ZNF564	-1.91902522	0.03380255
RASGRP3	1.38057614	0.01201145	PGBD2	-1.91789211	3.4921E-05
TNIP2	1.37549489	1.289E-10	RBM26-AS1	-1.91735716	0.04481529
GADD45A	1.37491109	1.9045E-06	RP11-731C17.	-1.91687804	0.00492999
EAF1	1.37355594	6.2777E-07	SLC46A1	-1.91644372	6.4203E-08
POLR3E	1.37346258	1.4748E-06	ZNF223	-1.91589665	0.00461668

CALCRL	1.37345452	0.02153748	LINC01569	-1.91221348	0.03229128
PTK2B	1.37193559	3.4139E-16	RP11-884K10.	-1.91141387	0.01058365
NFKBIE	1.36873257	2.3256E-09	LINC01126	-1.90922566	0.02590737
RUNX3	1.36835895	5.0515E-21	FLJ21408	-1.90921447	9.322E-07
ZC3HAV1	1.3680029	1.1523E-24	RP11-138I1.2	-1.90877562	0.0055901
XBP1	1.36765053	2.3414E-34	CCDC77	-1.90861842	4.2707E-05
C17orf107	1.36755916	0.0011977	BLNK	-1.90762447	6.0612E-08
PNP	1.36617857	0.00031843	PTPDC1	-1.90477149	0.0109267
PLEKHG2	1.36064221	7.7566E-06	GCNT1	-1.90269122	6.4458E-19
ACKR3	1.3584736	0.02756654	ZSCAN21	-1.90129423	0.00283767
GRAMD3	1.356461	0.00037594	MMP14	-1.90107354	2.5949E-07
KYNU	1.35392462	8.0694E-07	LRFN1	-1.8990217	0.00302491
NFAT5	1.35345583	2.0543E-05	DOK3	-1.89547501	2.029E-09
TRAF3	1.3519115	1.3442E-05	PAFAH2	-1.89348264	0.000126
MEF2D	1.34961766	9.6849E-09	LRTOMT	-1.89223115	0.00721229
PFKP	1.34899218	0.00016966	RP11-162G10.	-1.8909742	0.00058892
LDLRAD3	1.34552988	0.02530761	ZNF584	-1.89037199	0.00888724
TCTN3	1.34541017	9.9944E-09	LL22NC03-2H8	-1.88865117	0.00023332
MOB3A	1.34371309	1.873E-12	TLR6	-1.88855142	2.1791E-11
JMY	1.33977201	1.1893E-07	PIK3R3	-1.88531308	5.069E-05
CRIP1	1.33903237	7.8372E-14	ZNF396	-1.88393329	0.00982797
SGMS2	1.33894949	0.00243708	RP11-179B2.2	-1.88225343	0.03051576
LILRA2	1.33298564	1.7962E-06	NFIA	-1.8797041	7.9824E-07
TAF4B	1.33256434	0.00036334	TBX19	-1.87943155	0.00019746
SPON2	1.33253987	4.0903E-05	TCEANC2	-1.87346307	4.2863E-05
CD58	1.33253427	5.4081E-09	L3MBTL4-AS1	-1.87318101	0.00715932
CASP9	1.33251254	0.00015895	ADO	-1.87153028	9.3495E-10
RBBP8	1.33193408	8.5452E-05	IGF1	-1.86930676	3.4075E-12
BANK1	1.3304651	0.02069092	TCTEX1D1	-1.86679307	0.00130901
MTMR9	1.32656171	6.2643E-08	ZNF680	-1.86440008	1.2427E-07
SNHG15	1.32622341	1.8287E-10	HAL	-1.86069289	0.04961118
ST18	1.32533539	0.02688072	RP11-259N19.	-1.85838126	0.02197078
GMEB2	1.32513433	0.00997562	ETAA1	-1.84387355	0.00107941
PPP1R16A	1.32511979	0.00250953	ZSCAN26	-1.84264969	0.00046882
SIPA1L1	1.32429133	5.6443E-05	RBM43	-1.84037696	3.9479E-08
LAMP3	1.32171958	0.00728052	MTUS1	-1.83934026	0.00041741
FSCN1	1.32121238	0.00118066	AC004069.2	-1.83923249	0.00103209
RAPGEF2	1.31969399	0.00051672	SNX11	-1.83873823	1.1941E-18
CD80	1.31902542	0.02405988	ZNF763	-1.83846024	0.03732055
TNFAIP2	1.31781201	1.8565E-07	RP1-34B20.21	-1.83700179	1.2348E-05
ESYT2	1.31744845	2.1161E-06	ZNF287	-1.83483568	0.03681268
ARID5A	1.31668652	5.4007E-17	DZIP1L	-1.83330181	2.2568E-06
ANKLE2	1.31484295	4.0019E-08	ZNF212	-1.82369139	0.00047249
ENTPD7	1.31456359	0.00257265	HMGN3-AS1	-1.82341288	0.01421534
DNAJB9	1.3138553	1.199E-15	WWP1	-1.8212155	1.0519E-16
MGST1	1.31227401	0.00066998	LINC00294	-1.82051464	2.7041E-05

TANK	1.31223904	1.1205E-11	RP11-134G8.5	-1.81994643	0.00095181
GALNT3	1.30852447	0.00329025	GPT2	-1.81980872	0.00121638
BHLHE40	1.30735133	5.2351E-07	PPP1R13L	-1.81514571	0.04095488
MAFG	1.30563122	1.978E-08	ORC5	-1.81279674	3.2377E-05
C16orf54	1.30549826	0.01783035	C2orf81	-1.81258122	0.03967822
RAPGEF1	1.30274898	2.2243E-10	ZNF595	-1.81170273	0.00280554
WARS	1.3013226	2.6312E-14	PPIP5K1	-1.81169641	0.00158246
SDF2L1	1.30053061	8.2072E-23	ZNF764	-1.80551789	0.00057489
SYTL1	1.29450587	0.03420658	ADRB1	-1.80543347	0.0001223
PSMA6	1.29321292	3.6086E-08	AC093627.10	-1.8042417	3.2935E-07
PAPD5	1.29269011	5.6965E-06	NAPEPLD	-1.80350215	0.00053832
RIN2	1.29082121	5.2611E-09	CRHBP	-1.803001	0.00011792
LINC01588	1.28956135	0.012568	ZNF777	-1.8029705	0.00222176
TRAF3IP2	1.28909291	0.02128728	ZNF248	-1.79401411	0.00020054
BCL7A	1.28883501	0.00029306	ZNF320	-1.78811146	0.01986912
FAM177A1	1.28766302	2.9918E-17	PPARGC1B	-1.78764969	1.688E-08
ACAT2	1.28741722	7.3712E-08	HNRNPU-AS1	-1.78596305	2.8049E-17
LY9	1.28652394	0.00020546	ZNRD1-AS1	-1.78555801	0.00028719
RPGR	1.28607443	1.5268E-06	FAXDC2	-1.78502693	4.6235E-05
TUBA1C	1.28547494	2.4914E-17	C8orf58	-1.78362982	0.00279824
CTDP1	1.28057588	1.5914E-06	PPIC	-1.77945788	0.02099656
ITGAX	1.27829696	6.1733E-14	GINS3	-1.77888734	0.01150463
PDCL3	1.27743096	6.5346E-22	ACSM5	-1.77823951	0.02986034
TGIF1	1.27734428	1.1333E-20	PFKFB2	-1.77528883	0.00097264
IL18R1	1.27625149	0.00109964	RP11-634H22.	-1.77450288	0.02797716
C14orf169	1.275589	0.00575373	LCAT	-1.77231237	0.0143424
SCO2	1.27460957	1.4533E-13	METTL18	-1.76716452	0.00019663
MYLPF	1.27355378	0.00611213	GIMAP4	-1.75723988	8.2447E-22
PDZD8	1.27246038	7.4191E-10	SLC38A6	-1.75494452	1.7983E-10
SRGN	1.27105223	2.5541E-59	RP11-452L6.5	-1.75377896	2.4331E-05
CDC42SE1	1.26988745	2.131E-25	ZNF439	-1.75008269	8.9097E-08
RP5-1136G13.	1.26980745	0.01896798	MMACHC	-1.7498279	0.02057127
UAP1	1.26672963	9.1195E-05	AC005253.2	-1.74909845	0.00373492
SMOX	1.26513422	0.00114529	SEPSECS	-1.74862793	1.4185E-05
EDEM1	1.26463468	2.3166E-08	THAP8	-1.74812598	0.01134773
LRRFIP1	1.26335502	2.1617E-29	ZNF527	-1.74776851	0.01286469
IL1R1	1.26006466	7.5388E-06	CTD-2313J17.(	-1.74625393	2.0984E-05
DOLK	1.25743982	0.03468779	SIGLEC1	-1.74541658	8.7721E-13
C1orf56	1.25396555	3.7637E-10	TNFAIP8L2	-1.74457715	2.8244E-23
TSHZ1	1.2536712	6.5198E-06	PRR5L	-1.7429597	0.00015164
MARCKSL1	1.25336726	2.7553E-11	PYGO2	-1.7407824	9.5989E-06
FNIP2	1.25177022	3.5107E-16	DCP1B	-1.74026408	0.01892913
WEE1	1.24718056	0.01566972	CD3EAP	-1.73872693	3.0107E-07
IFT57	1.24452852	9.8141E-06	ZNF322	-1.73832573	5.7126E-14
TIMP1	1.24336112	4.0804E-20	CTD-2562J15.(	-1.73669717	0.01918839
MAP2K1	1.24128577	1.9016E-12	GAREM1	-1.73551067	0.00155371

TNFAIP8	1.23839917	5.5652E-06	CCL8	-1.73476015	0.0140091
BORCS5	1.23823204	5.9458E-05	RP11-258C19.	-1.73368965	0.01594378
S100A10	1.23767493	4.0882E-23	TEFM	-1.7329649	1.791E-08
OSER1	1.23528435	2.1632E-12	CTD-2135D7.5	-1.73280571	0.00101258
IRF1	1.23277421	1.965E-10	LACC1	-1.7309731	1.4589E-22
MANBA	1.22875476	8.9526E-09	CARD6	-1.73082562	1.1137E-07
ETF1	1.22775867	4.4986E-20	ZNF623	-1.72780108	0.00815406
KPNA2	1.22695389	2.2061E-11	RP11-109G23.	-1.72581388	1.277E-05
NMB	1.22685422	0.02835116	LMAN2L	-1.72470877	0.00153064
SNX8	1.22502677	6.5105E-12	RP11-333E1.1	-1.72160078	0.00055309
GLIPR2	1.22375692	2.2271E-12	HIST1H2BE	-1.72141752	0.00274749
RASSF3	1.22329119	7.435E-10	RP11-467D6.1	-1.72081589	0.00339225
BIRC3	1.22155233	3.5082E-16	PPP1R3D	-1.71787832	5.8391E-06
SDS	1.22041958	0.00210627	OLFML2B	-1.7164409	0.000213
AFF3	1.21646774	6.4183E-07	SUCNR1	-1.71393563	0.00059187
NAMPT	1.21635343	4.8251E-30	SIGLEC11	-1.71298964	0.00028612
FASTKD5	1.21541522	0.00348919	NHLRC4	-1.71262367	0.04301634
CSRNP1	1.21262894	3.2513E-11	ZNF678	-1.70661072	0.00232993
MMP9	1.21066422	0.00337567	NLRP12	-1.70347519	0.03764944
GPCPD1	1.20973039	2.1132E-09	GTF2E1	-1.70333709	2.3805E-05
ITPKB	1.20911961	0.01029529	WDR27	-1.70313302	1.7913E-06
OSM	1.20806137	0.01566972	RP11-117D22.	-1.70187188	0.02541444
RNF19B	1.20490887	3.9009E-05	AIM2	-1.69952218	1.6416E-11
CTB-58E17.1	1.19838694	0.01104769	ANKS3	-1.6979712	0.00075703
EIF2AK4	1.19482092	3.9698E-12	SLC29A3	-1.69611047	1.9349E-06
DGKD	1.19257693	5.6443E-05	RP11-332H14.	-1.69479089	9.7179E-07
RAB21	1.19111553	2.3345E-08	HSD17B14	-1.69129867	0.0039466
STARD8	1.19085139	0.00059387	RP11-426C22.	-1.69070818	0.02715336
CXorf40B	1.19032953	5.3799E-05	GOLGA8R	-1.68361409	0.02651474
MYADM	1.18741347	7.6197E-14	LINC00900	-1.68253155	0.03205505
ANKRD37	1.18683141	2.802E-09	HIST1H2BH	-1.68207922	0.01544987
FAIM	1.18471562	0.03717014	ZNF260	-1.68090334	5.0848E-05
IL1B	1.18391222	0.00027836	ALKBH8	-1.67988573	8.7049E-05
LPCAT1	1.18209588	3.3608E-07	RP11-102N12.	-1.67747787	0.00019026
CTNNB1	1.18149689	1.1073E-12	MED20	-1.67732669	0.00171439
ATF5	1.17754588	1.075E-05	ZNF718	-1.6752349	0.00749337
CD109	1.17365572	0.00330279	NUDT17	-1.67504997	0.01693989
FBXO33	1.17342577	7.4911E-07	RP5-821D11.7	-1.67498042	9.6129E-05
FCN1	1.17298302	0.01650323	ARHGAP11B	-1.67465562	0.00037532
SKIL	1.17072417	2.1495E-13	RP11-407N17.	-1.67413911	0.03967941
MEFV	1.17068207	1.5986E-06	CLCN4	-1.66876445	0.00132994
ATP2B1	1.16831475	5.2671E-14	DNAJC3-AS1	-1.66811596	0.00276321
MESDC1	1.16795635	1.6173E-07	ACAD11	-1.65928327	0.02707429
PLAGL2	1.16776153	0.01357894	ZNF34	-1.65843179	0.00051672
TUBA1A	1.16593923	4.8411E-12	CCL3	-1.6580062	6.2133E-05
SRSF5	1.16511072	6.5346E-22	RP11-214N9.1	-1.65754407	0.00706474

MAPK6	1.16144231	6.0289E-05	RP11-1143G9.	-1.65732708	0.00923877
PADI2	1.16056709	0.04137075	RP5-827C21.4	-1.65560078	6.6807E-07
OLR1	1.16005106	4.8872E-09	HOXB2	-1.65364517	0.0108624
NPIPB4	1.15988126	0.00702594	C1QC	-1.65325965	3.2105E-06
CHMP4B	1.15958293	2.8885E-11	ZNF347	-1.65223739	0.00030575
RNF144B	1.15603361	1.5262E-10	ZNF566	-1.64834467	0.00148591
DDX21	1.15435179	3.2484E-39	GFRA2	-1.64672541	5.8067E-15
DOT1L	1.15363753	0.00268593	SLC15A2	-1.64549257	0.01396966
SEMA4A	1.15333725	0.00031557	ZNF155	-1.64142477	0.0329494
ACSL5	1.15218455	1.9148E-05	IFIT5	-1.64010999	3.5819E-05
CBX6	1.15041175	4.5193E-09	PIK3IP1	-1.63940229	3.5317E-14
PLP2	1.15026215	6.3649E-12	ARMCX2	-1.63509767	5.1639E-05
ZNF487	1.14802623	4.4525E-05	RP11-1000B6.	-1.63456501	1.6425E-06
TES	1.14610763	2.0778E-14	FBXO16	-1.63275243	0.01792205
TWISTNB	1.1453784	6.009E-07	ZNF771	-1.63261898	0.03963616
CXorf40A	1.14306296	2.6961E-05	KLF2	-1.63209905	3.7465E-11
CD207	1.14066745	0.01484427	NEIL1	-1.63185005	0.0052942
TMEM154	1.13894591	3.4926E-05	SESN1	-1.6308885	6.6935E-17
CAPN2	1.13662178	2.9712E-06	GADD45G	-1.62904993	8.5157E-06
MED14OS	1.13628609	0.00367159	FIGNL1	-1.62801987	0.00013511
TP53INP2	1.13622191	0.00103282	MFAP3	-1.62754049	0.04582268
RP11-73E17.2	1.12914891	0.0101483	AC004540.5	-1.62695121	0.04908803
IFI30	1.12906233	2.1477E-06	DOCK4	-1.623759	4.5666E-10
PPP1CB	1.12470325	1.1601E-16	CTSD	-1.62321952	9.1005E-13
TFDP1	1.1207844	8.0628E-08	PTCD2	-1.62312054	0.00040648
TSC22D1	1.12030751	3.8878E-05	HMGCL	-1.62273856	1.4581E-19
RNF139	1.11672038	3.4832E-07	FBXL18	-1.6222623	0.04321855
NFKBIA	1.1165755	0.00047167	ZNF486	-1.62200217	0.02423303
PTS	1.11626786	1.4587E-07	UFSP1	-1.62141035	0.04801713
FLNA	1.11552019	3.6757E-06	DOK1	-1.61875051	7.1443E-19
HSPA5	1.11537108	3.9332E-10	CCDC136	-1.61831719	0.02631258
MYO1G	1.11230988	1.9437E-07	ZNF484	-1.61603385	0.01496824
DUSP2	1.11068631	1.3114E-06	RNU12	-1.61491026	0.00019746
GABARAPL1	1.10638252	7.0982E-15	HYPK	-1.61457988	2.0829E-08
AMPD2	1.10623597	1.7906E-12	ZNF514	-1.61453108	0.00029233
PNMA1	1.10434093	0.01460046	THEM6	-1.6128752	0.00034026
ACOT9	1.10255026	7.2549E-10	KBTBD7	-1.61267665	2.19E-05
TET2	1.097043	2.4488E-13	PLEK2	-1.61082173	0.02476864
MTHFD2	1.09664118	2.3896E-24	SPRED1	-1.60923785	1.0898E-12
SH2B3	1.09401742	3.0948E-17	FRMD3	-1.6084678	0.00517868
HOTAIRM1	1.0936696	9.9475E-07	FBF1	-1.60645011	0.04903587
SNX20	1.09230857	1.8302E-05	DNAJC28	-1.60612429	0.04475899
PNPLA8	1.09073294	4.0461E-09	INPP4B	-1.60378317	0.00597666
PANX1	1.0897926	0.01690132	HKR1	-1.60306785	0.00071793
MX2	1.08829897	1.5059E-08	RFXAP	-1.60122461	0.00232834
SPAG9	1.08761422	9.7417E-09	ZNF230	-1.60078653	1.7564E-05

TXN	1.08599212	9.5289E-13	ZNF549	-1.59990451	0.02082779
DUSP3	1.08598799	1.5154E-06	DBR1	-1.59641301	8.3342E-06
SPATA2L	1.08550519	0.00158758	KCTD11	-1.59636867	0.01141577
USP36	1.08232906	9.3396E-08	CTA-384D8.35	-1.59632253	0.00309544
TICAM1	1.08100625	0.00058944	CCDC30	-1.59497356	0.0051009
MANF	1.0804396	1.8774E-15	GUCY1A3	-1.59411111	3.757E-06
B4GALT1	1.07609807	3.7794E-12	DUSP6	-1.59393607	1.302E-09
SLC25A19	1.07553008	2.8494E-10	ZMYM1	-1.59382847	0.0002037
TRIP10	1.07483905	0.0091302	ZBTB34	-1.59210506	9.6397E-05
PLK2	1.07419412	8.7033E-05	TTC23	-1.59193469	0.02699624
IFITM1	1.0726531	0.0494015	SCAMP1-AS1	-1.59152928	9.0039E-06
LATS2	1.07155608	2.8852E-07	FOLR2	-1.5911798	1.2806E-06
LINC00884	1.07050556	0.04221961	AMMECR1	-1.58843559	0.00842586
CHMP1B	1.06962627	3.4103E-26	LINC01473	-1.58814958	0.0311287
YBX3	1.06886498	7.4295E-13	PAXBP1-AS1	-1.58724589	0.00018974
DUSP10	1.06782493	0.0008697	ZDHHC23	-1.58711586	0.0096918
KCTD20	1.0658606	2.0181E-09	CNNM3	-1.58443656	0.02912788
TAP1	1.0651752	5.8058E-08	SERPINH1	-1.58409953	0.00110496
AGAP3	1.06516824	0.00014645	ALS2	-1.58222914	0.01098569
IFITM2	1.06516396	9.6304E-17	SCYL3	-1.58097968	1.9964E-05
HSH2D	1.06364496	0.006577	KLHDC8B	-1.58047518	0.00117918
KIF9	1.06330827	0.01332444	WDR19	-1.57980707	0.03341683
FGR	1.06245454	1.302E-09	ZNF765	-1.5785329	0.00027996
LITAF	1.06112196	9.1589E-22	YEATS2	-1.57828453	1.6665E-06
HMGB3	1.06014834	3.8354E-05	ZSCAN32	-1.57635684	7.873E-05
PLSCR1	1.05976151	1.6684E-23	DNAJA4	-1.57515517	2.4734E-05
LHFP	1.05905568	8.7623E-05	CBX7	-1.57446097	0.00371312
LINC01272	1.05719575	0.00558932	RP11-252A24.	-1.57419708	0.00165913
IZUMO4	1.05387652	0.03884431	INTU	-1.57368905	0.02589373
DRAM1	1.05207352	0.00433564	CAPRIN2	-1.57345236	1.0182E-05
ZNF703	1.05073232	0.00066362	CASP8AP2	-1.57126118	4.4743E-07
TOB1	1.04839108	0.00016456	CCDC142	-1.56897904	0.00616828
BZRAP1-AS1	1.04342356	0.03630193	EGFL7	-1.56726889	0.04875114
SIAH1	1.04173594	4.5432E-06	LINC01094	-1.56433255	0.02154994
FTH1	1.04057297	8.8139E-18	A2M	-1.56407735	3.4238E-12
BZW1	1.03833781	9.5442E-20	RP9	-1.5638338	5.3047E-09
ARL5B	1.03757997	4.6846E-08	PLEKHA7	-1.56366058	0.00065624
OXSR1	1.03349997	8.2208E-06	PGBD4	-1.56339266	0.04136694
RARA	1.03131137	8.5456E-07	ARRDC5	-1.56270352	0.00222176
TUBA4A	1.03131117	0.00049284	CBY1	-1.56180489	0.01566972
IL4R	1.03102576	0.00026081	CD276	-1.55991552	0.00032115
SMG1	1.03005037	3.4023E-08	RP11-708J19.1	-1.55947285	0.00365026
ADA	1.02968467	0.00160335	CACNB4	-1.55701919	0.00015972
SESTD1	1.02917514	4.1184E-05	C10orf88	-1.55396401	0.01445205
CSNK1D	1.02723715	4.6363E-14	EFEMP2	-1.55344085	0.02636854
OAT	1.02604291	4.8978E-10	AMN1	-1.55306436	0.00026284

FMNL1	1.02503655	9.1E-07	RP1-168L15.5	-1.55306354	0.04121471
SMPD2	1.0248478	0.00729594	MAFB	-1.55131732	5.9739E-30
ICOSLG	1.02479862	1.5037E-06	BOLA1	-1.54966763	0.00031655
THEMIS2	1.02302784	1.8487E-15	AC108488.4	-1.54931431	0.04348793
LACTB	1.02010517	8.1088E-11	DLEU7	-1.5470673	0.00028612
DCUN1D3	1.01947069	0.02016288	NISCH	-1.54416717	5.8802E-19
PPARD	1.01695721	0.01687445	NR1H3	-1.53993958	4.0293E-06
NUP98	1.01511565	1.802E-05	ZNF589	-1.53801239	0.00190484
RNF145	1.01304825	3.284E-07	TLR7	-1.53565898	2.9104E-06
CHD1	1.01184323	6.1671E-10	ZNF585A	-1.53305408	0.01258601
SPATA13	1.01176288	0.01400941	MARS2	-1.53236123	0.01902327
RP3-395M20.1	1.01027606	0.00794388	ZNF700	-1.53184409	4.2648E-07
ALG2	1.00928632	1.6336E-06	C12orf65	-1.53096454	6.9914E-10
PAF1	1.00900695	0.00058722	USPL1	-1.53000651	1.1956E-08
MT1F	1.00557659	0.0123139	STON2	-1.52810627	9.7191E-06
BCL2L11	1.00452815	1.8835E-07	ZNF512	-1.52785841	0.00020252
NFKBID	1.00362741	6.2738E-08	ZNF611	-1.52590658	0.00060705
SEPT6	1.00014143	1.0217E-07	TSEN2	-1.52530233	0.00863718
ZMIZ1	0.99944268	1.2413E-05	IPCEF1	-1.52473438	1.8086E-07
CDK2AP1	0.99890338	3.475E-08	CENPBD1	-1.52432262	5.4316E-05
RRP12	0.99585869	0.00226867	HOMER3	-1.52414322	5.5564E-05
RP5-1171I10.5	0.99577531	1.0556E-13	BRD8	-1.52293532	7.0899E-07
STK17A	0.99523166	3.3965E-07	PYCARD-AS1	-1.52214512	0.00055968
BANP	0.99475671	0.00058341	TMEM186	-1.52137751	0.00039141
FAM110A	0.99457056	9.0488E-07	PIGM	-1.52001712	3.5897E-05
SYNGR1	0.99319607	0.00494508	SGK223	-1.51811992	0.00156495
CHSY1	0.991979	0.00208617	GPNMB	-1.51575564	7.8944E-05
SLC9A8	0.99107423	0.01129654	HHEX	-1.51352227	7.4787E-14
RAB8B	0.99028613	1.6471E-11	BRF2	-1.51215475	0.00069765
CYTH1	0.98991782	9.2129E-08	CNRIP1	-1.51143357	0.00017897
OAS3	0.98981844	0.04016191	RP11-629O1.2	-1.51056231	0.00846515
GNA13	0.98886108	6.1763E-13	CCL3L3	-1.50986113	4.7549E-05
S100A6	0.98864046	9.1323E-14	NHS	-1.50954242	0.01537424
CD40	0.98854043	1.0539E-05	ZNF510	-1.50934438	0.02119016
PTGER2	0.9884135	1.4939E-05	LINC01006	-1.50791386	0.02992403
ELOVL5	0.98807254	2.3524E-08	IFFO1	-1.50670859	6.6039E-09
ABCA5	0.98746483	0.03893269	ALG9	-1.50525484	0.01934453
ELK3	0.98698194	0.00108157	RP13-638C3.4	-1.50414246	0.00034406
STARD4	0.98697781	0.00210803	RP11-4B16.4	-1.50403435	0.00290737
B3GNT5	0.98508618	2.5979E-05	ARHGAP25	-1.50373047	5.1032E-14
YWHAZ	0.98342858	2.405E-38	THAP6	-1.5022533	2.8808E-09
BCL7B	0.9828821	1.1303E-07	RPP25	-1.50144683	2.3352E-05
NAPA	0.98237181	3.0083E-12	AC009506.1	-1.50105308	0.00046098
MIDN	0.97917647	7.1389E-06	N6AMT1	-1.50053373	0.00190484
CD86	0.97866718	3.1394E-15	P2RY11	-1.4999721	0.00915237
UBE2B	0.97782624	3.7987E-18	RP11-458J1.1	-1.49897477	0.04806636

PNRC1	0.97690242	3.2158E-11	ITPKC	-1.4960442	0.00093968
DYNLT1	0.97671073	2.1019E-13	PLCL1	-1.49500096	0.00491157
CCNH	0.97602163	9.0651E-13	SDC3	-1.49464631	3.4658E-05
ABCF1	0.97415557	5.3141E-12	CCDC180	-1.49460514	0.03946072
CYB5D1	0.97408887	0.01188166	ADGRG5	-1.49387699	5.8893E-06
VIM-AS1	0.97201071	0.01149952	SWSAP1	-1.4932682	9.278E-06
IRF9	0.97020513	2.3733E-06	RAD52	-1.4922014	0.0234438
BCR	0.96950603	0.0443694	C1QA	-1.49087193	1.5603E-10
BCAT1	0.96945246	5.5309E-05	NAPB	-1.49047893	0.00824814
IRF7	0.96686283	8.7195E-06	SLC12A5	-1.49008999	0.00826621
DLGAP3	0.96617862	0.00643802	RP11-536K7.3	-1.48800947	0.03682864
GPR65	0.96482694	1.8258E-08	ECT2	-1.48758278	0.01023756
ANPEP	0.96200239	0.00578646	SASH1	-1.4864193	1.3897E-06
CHRAC1	0.96080353	6.4935E-08	SLC22A15	-1.48604213	0.00641727
CD83	0.96016633	3.7852E-06	ZNF768	-1.4854959	1.8182E-05
LRG1	0.95937895	0.00928947	LPAR6	-1.48494537	4.4221E-29
DHX34	0.9587813	0.01737827	ITPRIPL1	-1.48354573	0.00441012
ANKRD9	0.95375045	0.02461919	ZNF490	-1.48297206	0.02780843
REXO1	0.95235878	0.00208537	TMEM116	-1.48185555	0.00092828
PAPSS2	0.9505026	0.00010586	C1QB	-1.47965507	1.1917E-05
CYLD	0.94871527	5.6909E-12	MTRNR2L12	-1.47926638	0.0004615
SLC36A4	0.94701837	0.00012892	CCDC51	-1.47857652	0.00636492
PPA1	0.94504744	1.0752E-19	NMNAT1	-1.47606762	0.00023137
PCIF1	0.9448448	0.000208	NPL	-1.47467188	0.00041851
THUMPD3-AS	0.94444148	5.8595E-07	PLTP	-1.47441412	5.3125E-09
ALG13	0.94278908	4.834E-09	ZNF606	-1.4743335	0.01689254
TAF13	0.94161165	3.2616E-05	ZNF232	-1.47399299	0.01192388
GBP2	0.93895939	2.1621E-08	WDR24	-1.47341905	0.00742325
SAV1	0.93843481	0.01785674	BIVM	-1.47245471	0.00346726
CYTIP	0.93801411	2.0126E-13	MTHFSD	-1.47113221	0.00476524
ADGRE3	0.93671072	0.02608315	DCLRE1A	-1.47112784	0.03277746
PIK3R5	0.93526983	0.00111483	CLEC1A	-1.47073198	0.00104883
TPCN2	0.93482211	0.03429842	MSANTD4	-1.46965686	1.0406E-05
RAP1B	0.93309485	1.72E-15	MMS22L	-1.46824763	0.00227862
SDE2	0.93011225	2.1174E-06	RP11-872J21.3	-1.46764851	0.00556246
CNOT10	0.93002806	0.00267657	RP11-350N15.	-1.46743614	0.00546045
ZCCHC3	0.92999632	0.00135044	BCDIN3D	-1.46649405	0.00039037
POGLUT1	0.92930333	0.00042798	MKL2	-1.4656115	4.4761E-05
CXCL3	0.92777742	0.00125868	ZNF816-ZNF32	-1.46477139	0.03718879
RNF168	0.92766441	0.00020164	ZNF823	-1.46277494	0.01829086
RRN3	0.92722693	0.00116162	NCK1-AS1	-1.46167365	1.1478E-05
FRMD8	0.92658004	0.00020211	ANKRD20A4	-1.45848749	0.03430565
WDR45B	0.92635659	1.9489E-07	RP11-490O6.2	-1.45830964	0.00492256
H3F3A	0.92596704	1.0531E-30	FTSJ2	-1.45694762	6.9944E-17
UBTD2	0.92490145	0.00727298	DYNC2H1	-1.45657254	0.04037422
HAUS8	0.92478307	0.00616149	GOLPH3L	-1.45630135	0.00041926

COQ10B	0.9241268	1.0537E-11	CLHC1	-1.45611365	0.02063032
SPTLC2	0.92344034	1.0536E-10	ZNF844	-1.45402539	7.1132E-05
ARL4A	0.9224232	2.904E-06	CHRNB1	-1.45280804	5.1308E-05
GBE1	0.92213277	0.01582868	RP11-379K17.	-1.45155488	0.00960347
IPMK	0.9208992	0.00024283	GRAP	-1.45139554	0.02741019
HSPA13	0.91965111	0.00051093	ZNF182	-1.45005976	0.01425068
ANXA1	0.91900254	8.4457E-27	UTP14C	-1.44981356	0.00209137
RAB1A	0.9188645	1.4006E-09	ARSK	-1.44912671	0.01506896
NFKBIB	0.91845584	0.00024901	ERCC4	-1.44730715	2.3584E-07
COL9A2	0.91591973	0.01110755	FAM135A	-1.44724664	1.8267E-06
CMTM6	0.91375167	4.0433E-22	DNAJB1	-1.44716398	0.00129963
FAM102B	0.91217398	1.6973E-05	TRIM66	-1.44602632	0.01518477
BRCA2	0.91108485	0.00107109	ST5	-1.44584962	0.002623
CHST11	0.91030001	4.4519E-08	ADAMDEC1	-1.44463813	0.0001169
FCER1A	0.90956169	9.2678E-06	KBTBD6	-1.44456069	0.02752088
EMD	0.90684821	4.4546E-07	MDK	-1.44395988	0.03653315
ASGR2	0.90610435	0.00134863	TRIB3	-1.44276548	0.04386776
SLC31A2	0.90597777	1.688E-08	NEU1	-1.44265366	8.1311E-07
RGS13	0.90518592	0.02252279	RP11-401F2.3	-1.44171199	0.04293931
SLC25A33	0.90324469	9.4658E-05	CBX8	-1.44118481	0.04092127
OPN3	0.90186667	2.0372E-05	CCDC86	-1.44009123	2.254E-06
SPNS3	0.89990857	0.00158233	FCHO2	-1.43955485	1.2E-07
CFP	0.89914661	6.0267E-08	TMEM204	-1.43503684	0.00459355
NUAK2	0.89875207	0.00884839	ACBD4	-1.43412189	0.00116187
RFTN1	0.89611282	0.00013599	FAM109B	-1.43218694	0.01050931
LDHA	0.89548953	4.7661E-14	RP11-532F6.3	-1.43044324	0.02031423
ADGRE5	0.89490058	1.2211E-09	KIAA1841	-1.42961523	0.01043471
CCDC9	0.89439118	0.00589011	ZNF550	-1.42525866	0.04301634
HIP1	0.89431122	0.00135961	C1orf112	-1.42271903	0.03963751
N4BP1	0.89429834	0.00132056	NSUN5	-1.41915737	2.3789E-11
ZBTB21	0.89293618	0.00117557	HTR7	-1.41880928	6.1171E-05
CHAF1A	0.89244825	0.0050438	SNX33	-1.41851903	0.01141177
PER2	0.89149559	0.00238279	PROS1	-1.41595056	0.03967941
EXOSC6	0.89088112	1.4545E-07	BORA	-1.41476336	0.04523587
SKI	0.89060868	0.0018361	COPZ2	-1.41372066	0.0093687
FLT3	0.88905428	0.00085364	SETDB2	-1.4115543	6.1763E-13
PLAC8	0.88869634	1.5021E-05	ZNF568	-1.41079962	0.02069092
ZNF331	0.8870093	3.9777E-07	NFXL1	-1.40856002	3.8608E-05
IRF8	0.88685238	3.9941E-06	ITGB5	-1.40821698	3.5572E-07
BAIAP2	0.88479272	1.5992E-06	S100PBP	-1.40776131	4.976E-05
MKNK2	0.88336487	1.3238E-09	ARL11	-1.40733961	5.7432E-09
RIPK1	0.88292011	0.00551166	AP001059.7	-1.40483985	0.04663888
LDLRAD4	0.88176425	1.4489E-05	SEPHS2	-1.40334497	0.00854195
MASTL	0.88027973	0.01115432	FNTB	-1.4031695	0.04475518
HIF1A	0.87841813	3.8583E-11	ARHGEF12	-1.40281449	5.5026E-05
SIK1	0.87829208	0.01384129	TRAFD1	-1.40212738	2.9591E-06

HSPA9	0.87585894	1.1482E-12	ATM	-1.40112836	5.4694E-31
B3GALT6	0.87519447	0.0001409	LHFPL2	-1.40091512	3.5542E-06
CHMP2B	0.87454347	1.6118E-08	C21orf33	-1.39989851	0.00163768
SQSTM1	0.87368695	1.1137E-07	C19orf68	-1.39879164	0.01724541
MAPK1IP1L	0.8734111	4.5826E-12	PIF1	-1.39806795	0.03227156
CXCL2	0.87300094	0.00981256	CCDC7	-1.39764024	0.04257386
CYP2S1	0.8715632	2.0495E-05	FAM50B	-1.3970665	0.00307341
C15orf39	0.86931627	0.01113153	RMI1	-1.39413375	3.3656E-06
RBM15B	0.8685752	0.00012943	PMS1	-1.39410424	1.2664E-07
RELL1	0.86495451	0.02551624	ZNF707	-1.39259674	0.01925481
ST8SIA4	0.86480507	1.4367E-07	RCN3	-1.39257304	0.00057563
DNAJB11	0.86474208	5.3779E-08	POLG2	-1.39129653	7.8441E-07
STK4	0.86421904	2.6605E-08	RP11-326I11.3	-1.38988273	7.8906E-05
SEC23B	0.86312466	0.00010596	ACP2	-1.38926582	1.1856E-08
LINC-PINT	0.85920815	0.00603005	RP11-644F5.1	-1.38855637	0.00366924
DNAJB6	0.85829803	5.6634E-11	LYL1	-1.38827583	2.5021E-17
FAM43A	0.85731377	0.03370472	UNC5B	-1.3880937	0.00645371
ABL2	0.85719248	0.00728052	NNT-AS1	-1.38543811	0.00089393
GPBP1	0.85609693	3.4867E-14	CDPF1	-1.38502791	0.00268834
NETO2	0.8546559	0.00068964	MEGF8	-1.38420595	0.00320725
MGAT2	0.85379562	0.00024008	SH3BP5L	-1.38340404	0.0058986
ID2	0.85260403	0.01133914	MLYCD	-1.38100726	3.4149E-05
TIPARP	0.84927121	5.2707E-06	RNASE1	-1.38096422	0.00090867
SERPINB8	0.84696121	2.6646E-05	MRPS31	-1.38082228	2.6091E-14
HYOU1	0.84675285	0.00227342	NUDT12	-1.37975785	0.00410078
ANXA2	0.84646869	4.903E-14	C6orf203	-1.3751167	8.784E-07
FOXO1	0.84329316	1.0095E-05	FAM160B2	-1.37440753	0.00060816
USP16	0.84284887	9.5502E-08	RIC8B	-1.37372633	0.00712864
STRAP	0.84140897	6.5665E-15	CTD-2017F17.	-1.37320557	0.02532144
PSMD12	0.84086737	5.2301E-07	PIBF1	-1.37051761	2.3863E-06
SMAP2	0.83972392	1.1604E-16	ZSCAN29	-1.36863065	0.00182561
ZBTB1	0.83968579	7.0347E-05	LETM2	-1.36756013	0.02211902
PON2	0.83604265	0.0047596	ZNF513	-1.36680301	0.00056174
YRDC	0.83551335	0.01199938	ZNF573	-1.36394164	0.00574077
GPAT4	0.83253511	3.0559E-05	RUNDC1	-1.36303738	1.2475E-05
CDKN2D	0.83240506	0.01738238	TRMU	-1.36273339	0.00036331
BCAS2	0.8313669	1.992E-07	B3GAT2	-1.3625648	0.02819537
CREB5	0.82821626	0.00054079	LYVE1	-1.36198008	0.04237843
FAM107B	0.82810061	1.9435E-07	ZNF84	-1.36192649	1.3208E-06
TMEM184B	0.82803326	0.04418817	BICD1	-1.36022628	0.0051009
DNAJC3	0.82646148	6.8281E-09	FBXO44	-1.36007639	0.00423517
JOSD1	0.82545867	2.2016E-09	CPT2	-1.35850486	0.0002121
IL2RG	0.82540549	1.2842E-06	GPANK1	-1.35768091	4.7406E-10
ELF1	0.82102085	4.8901E-17	TUBD1	-1.35737268	0.00417483
TTYH2	0.82079391	0.01022128	RGS1	-1.35690155	4.6751E-15
MIR181A1HG	0.82075312	3.1277E-12	TMEM260	-1.35518877	0.00013938

VPS26A	0.82057814	2.4252E-10	GOPC	-1.35459053	1.1402E-09
LMNB1	0.81714898	0.00031436	KNOP1	-1.35393042	3.0181E-07
SLC19A2	0.81710914	0.00700934	ZNF202	-1.35313658	0.04811829
SAR1A	0.81422835	3.5093E-06	PRR4	-1.35284489	0.00093979
BZW2	0.81390633	6.8265E-09	PER3	-1.35283262	0.00047236
S100A4	0.81370015	3.3244E-06	TSC22D3	-1.35276851	1.1487E-22
EIF3C	0.81067149	0.01646701	RP11-834C11.	-1.34854392	0.00074727
TMX4	0.81062231	1.6621E-05	LINC01480	-1.34782944	0.00110009
ACBD3	0.81053794	2.6021E-05	CDKL3	-1.34739845	0.04816464
TNFRSF1B	0.80799304	6.3851E-15	NEURL4	-1.34727396	0.01212648
PLEC	0.80790261	0.00044371	DBP	-1.34402311	3.9228E-07
AZIN1	0.80573657	2.029E-09	LIN7B	-1.34127118	0.00999811
NRAS	0.80472355	0.00090206	AC011899.9	-1.3410061	0.00060977
TMEM38B	0.80366636	0.00857374	SLC16A1-AS1	-1.34082796	0.00013717
AKIRIN2	0.80281882	3.805E-07	C22orf46	-1.3393252	6.1133E-05
ZNF503	0.80256606	0.00077889	POLR1B	-1.33915146	0.00625322
GPN1	0.80106809	0.00079739	TADA2A	-1.33873232	7.6558E-06
TLE3	0.79978816	0.00335222	ZNF444	-1.3385301	0.00481624
AC013461.1	0.79920481	1.4155E-05	AC002310.12	-1.33842051	0.04363811
SMIM3	0.7978568	0.00070947	DBT	-1.33702608	0.00050974
PHF20	0.79707003	1.9489E-07	TGDS	-1.33597335	0.00025631
GNG2	0.79648422	1.5284E-05	MGME1	-1.33569952	2.1214E-15
BRD4	0.79605409	2.1495E-13	AKAP10	-1.33425786	5.9642E-06
GSPT1	0.79522397	3.9048E-08	FKBPL	-1.33404082	0.02101464
HNRNPA0	0.79519197	2.6319E-12	CRAMP1	-1.33279358	0.00766447
ABL1	0.79472123	0.03189743	ADAP2	-1.33036143	6.3673E-23
UGCG	0.79456495	0.00078491	ZNF211	-1.32991487	0.00070693
IGFBP7	0.79403492	0.02511631	ZBTB41	-1.32937382	1.3529E-05
PDIA3	0.79401042	6.4459E-17	ABHD10	-1.32918301	6.845E-06
EMILIN2	0.79008302	2.9346E-14	ZRSR2	-1.32896765	3.4472E-11
LRRFIP2	0.78769434	4.5393E-05	CITED2	-1.32843728	2.5324E-18
NRP2	0.78508819	0.03217858	ZNF283	-1.32804123	0.0307696
GTF3C6	0.78468375	1.1326E-08	ZBTB33	-1.32798034	1.1546E-05
PVRL2	0.78443757	0.00078996	GPATCH1	-1.32794646	0.00089142
PSEN1	0.78245195	0.00019035	INTS6-AS1	-1.32679271	0.00678504
TRIM25	0.78146711	0.02741019	C5orf56	-1.32000953	2.7149E-11
PTP4A2	0.78089418	2.7891E-17	HIST1H2BD	-1.31954962	0.03855995
CHML	0.78036821	0.00047249	GEMIN6	-1.31909468	2.3782E-06
PBDC1	0.78010148	5.737E-08	PDE1B	-1.31799905	0.00439024
BCL2	0.77887183	1.7618E-05	TNF	-1.31779239	0.00881199
RBM7	0.77884891	5.2695E-06	C16orf70	-1.31693672	9.9656E-05
MECP2	0.77797128	1.0664E-05	TRIM23	-1.31539557	0.00176779
NUDT15	0.77695642	0.00829948	CTC-428G20.6	-1.3129969	0.04746782
YPEL5	0.77631436	2.9519E-09	CASP2	-1.31278188	1.1437E-06
NEK1	0.77569193	0.0112314	LINC01011	-1.31098554	0.02255846
ADD3	0.77545681	0.00051076	TRMT1L	-1.31054594	6.7708E-05

ARID3A	0.77385409	0.0036857	NLK	-1.31050324	0.00041406
ST3GAL1	0.77101006	0.00041461	ZNF528	-1.30977811	0.02119649
SLC25A3	0.77015115	1.0476E-11	SLC38A7	-1.30848015	0.01828412
PLK3	0.76993352	0.00959656	MLH3	-1.30701355	3.3594E-06
MAPK8	0.7692379	0.00698412	FBXL20	-1.30617185	4.3341E-08
PHF10	0.76899681	4.3614E-05	CCDC170	-1.30455364	4.9169E-08
IRAK3	0.76800138	1.2054E-07	TRMT13	-1.30439899	4.1176E-05
PRELID3B	0.76773196	5.4249E-06	AC025171.1	-1.30336493	0.01020446
FBP1	0.76717066	5.1094E-06	TMEM86A	-1.30255187	0.00051624
LPIN1	0.76715205	0.00078753	TAF4	-1.30164689	0.00177437
CHPT1	0.76699041	2.2616E-10	TIGD2	-1.30039532	0.02010089
PABPC4	0.76672537	7.1018E-07	MICA	-1.2983068	0.00049877
TOMM34	0.76640809	0.00646342	RPAP1	-1.29815676	0.01468763
ANTXR2	0.7639801	0.00105761	PPP1R3E	-1.2966243	0.01150463
CENPN	0.76389089	0.0477053	RPAP2	-1.29659528	3.9462E-07
STARD3NL	0.76281927	8.7709E-05	MRPS24	-1.2959476	0.02525479
CCND2	0.76167781	8.7195E-06	TMEM223	-1.29558866	6.1314E-10
DDX27	0.75959036	1.1323E-07	C5orf22	-1.29497762	0.00098622
STK10	0.75928305	0.00826574	CFH	-1.29381323	0.00197198
BAZ1A	0.75496178	1.2219E-05	ORC2	-1.29282508	0.00056174
CYCS	0.7548779	6.2328E-11	TRIM16	-1.29253197	0.01484056
PXDC1	0.75231998	0.00546045	TRIM73	-1.29250401	0.00163529
CH25H	0.75175811	0.00392625	TMEM99	-1.29174425	0.00328244
HMGA1	0.75076041	2.3899E-05	FAM72B	-1.291605	0.02164682
ZNFX1	0.74904605	0.00101228	C11orf68	-1.29036521	8.9494E-05
ISOC1	0.74723969	0.03963616	FUT10	-1.28976567	0.02242307
LINC00877	0.7433558	0.01259461	PTPRO	-1.28913901	0.03327582
NIPSNAP3A	0.74231166	7.5119E-07	RHOB	-1.28798485	1.8766E-07
JUND	0.74228618	8.9637E-07	SULT1A3	-1.28778824	0.011494
RAB22A	0.74204911	4.7909E-05	PIGBOS1	-1.28762166	1.1273E-08
CD48	0.74121866	3.7342E-11	SGPP1	-1.28733924	4.5944E-08
TRMT1	0.74112875	4.6774E-05	ERI2	-1.28648529	0.00256935
NR1H2	0.74103756	9.163E-06	KCNMA1	-1.28593544	5.0154E-05
HGS	0.74074477	0.00370454	CSTF1	-1.28430493	2.2772E-05
JMJD1C	0.74063901	3.3E-06	RP5-882C2.2	-1.2839328	0.00079259
FBRSL1	0.73999393	0.00616828	POLR3B	-1.28302311	0.01749098
METTL1	0.73993844	0.04543905	POLR3A	-1.28234042	0.00578618
CREB3	0.73895842	0.00338586	SCRN3	-1.28212815	0.00171256
TEX30	0.73872092	0.03174541	ACVR2A	-1.28198644	0.02892267
UBC	0.73868587	8.8581E-33	PIP5K1B	-1.28081612	0.00925654
RHOQ	0.73800294	8.6309E-06	RP11-242D8.1	-1.28074084	0.00242367
BACH1	0.73735017	1.2488E-06	ZNF227	-1.28065429	0.0049706
EIF1	0.73661927	9.2917E-21	ABI3	-1.28031998	3.1991E-30
CARHSP1	0.73658605	0.00271107	NQO1	-1.27990882	0.04842878
VIMP	0.73559668	2.539E-08	AC025335.1	-1.27928724	0.01170084
IL16	0.73545501	2.0588E-05	SNX18	-1.27914666	7.0518E-13

ISG15	0.73532419	1.6304E-05	NIFK-AS1	-1.27873444	0.00339296
RAB27A	0.73514443	0.0323126	PSMD5-AS1	-1.27781399	0.00013218
HECTD1	0.73496903	0.00052179	FBXL4	-1.27569311	0.00094286
TFRC	0.7342146	0.00395847	LENG8-AS1	-1.27529728	0.01521331
SARAF	0.73405121	4.7774E-19	TMEM176B	-1.27507705	0.00070656
VDAC1	0.73262377	1.1442E-11	ALKBH2	-1.27467108	2.734E-05
AHNAK	0.73136175	9.5487E-07	DTX2	-1.27260201	3.1844E-05
LFNG	0.73110644	0.00229384	ABHD11	-1.27226904	2.0533E-06
RP11-437B10.	0.73054972	0.0483306	SHPRH	-1.26818288	2.1192E-09
CCDC109B	0.73052784	0.0017067	RP11-119B16.	-1.26757985	0.00587852
OGDH	0.73029879	0.00888444	NOD1	-1.26616053	0.01986912
ISCA1	0.730184	0.00021155	TRIM14	-1.26458321	5.6135E-10
SH3KBP1	0.72917891	5.5168E-09	CCNG2	-1.2633094	1.4434E-11
MAFF	0.72866256	0.00285555	RNF219	-1.26316211	4.9833E-07
AP1S2	0.72842342	4.4659E-08	NUDT6	-1.26228959	0.00512916
HNRNPDL	0.72736488	3.055E-12	AC074289.1	-1.26217183	3.3127E-06
TNFRSF21	0.72689359	0.00219556	ZNF570	-1.26045797	0.02573876
DENND5A	0.72685656	0.03775078	VPS50	-1.26021723	8.5178E-06
PQLC1	0.72625648	3.6403E-08	MBNL1-AS1	-1.25889149	0.03120608
SRSF2	0.72546664	3.3106E-09	RNF135	-1.25865917	3.115E-11
UBE2J1	0.72408928	1.731E-06	PAQR4	-1.25855953	0.01426652
TPPP3	0.7237428	0.04153854	LPAR2	-1.25847911	0.01011523
FOXK1	0.72332161	0.0254417	SOCS5	-1.25718639	0.00057298
PPRC1	0.72324579	0.03575729	PUS10	-1.25713715	0.00232834
TXNDC11	0.72288692	0.00410418	FAM46A	-1.25675953	1.1521E-10
SLC35B1	0.72283471	0.00028023	SEC22A	-1.25556306	0.00176797
RBM8A	0.72205184	6.5542E-13	P2RY6	-1.25520262	4.1325E-12
ARMCX1	0.72132344	0.01286203	SMPDL3A	-1.25340284	0.00629983
ZFAND5	0.72091552	5.105E-14	KEAP1	-1.25265833	0.00016218
SLCO3A1	0.72036784	0.00018041	ZNF189	-1.25204058	9.6751E-05
UBALD2	0.71844324	1.3499E-05	SDR42E2	-1.25180812	0.01943946
TXLNG	0.71796379	0.01516438	SLC2A8	-1.25172007	0.00072226
SLU7	0.71765008	0.00015695	ZFP90	-1.25060541	0.00497201
RAP2B	0.71613701	1.1304E-07	PTCH2	-1.24779113	0.02712363
UBE2D3	0.71550338	3.684E-12	WBP5	-1.24722648	0.00093053
TTC7A	0.71491261	0.00813716	PLD3	-1.24284193	1.0556E-13
VCL	0.7149063	0.01018681	TNRC6C	-1.24201934	0.02961967
PQBP1	0.7139147	5.0951E-07	TAMM41	-1.24101825	0.00017897
LSP1	0.71297261	4.9537E-07	P2RY1	-1.24082403	0.01786544
LPXN	0.71174158	0.0011912	SRP14-AS1	-1.23844445	0.0108624
WIPI1	0.71170618	0.01972733	TCAF1	-1.23773699	0.00055109
CSNK1A1	0.71103028	3.4269E-11	RGS12	-1.23753038	1.883E-08
TOP1MT	0.70880943	0.01224731	PCYOX1	-1.23454805	0.00045634
CXCR4	0.70870077	0.00275688	ZNF577	-1.23439066	0.01714774
AKIRIN1	0.70666964	3.755E-05	MICB	-1.23330419	0.00653396
POR	0.70586493	0.00010586	MFSD13A	-1.23292444	5.8266E-05

LRRC59	0.7039444	0.00023041	TACO1	-1.23219042	0.00108683
DDX6	0.70383215	6.659E-08	SEC31B	-1.23196169	0.02158028
RCOR1	0.70380819	7.9423E-05	ASB7	-1.23035683	0.0046552
SBNO1	0.70352848	0.00069704	ATAD5	-1.22995695	0.00046483
SRRT	0.70291931	0.00585566	LAMB2	-1.22992505	0.02672695
SEC22C	0.70262038	0.00574077	KPNA5	-1.22968647	0.00059353
BNIP3L	0.70151859	2.1615E-06	TMEM63A	-1.22955806	6.4074E-05
MAP4K3	0.70070387	0.02370836	ZFP36L1	-1.22943494	1.0309E-11
IQSEC1	0.6996356	0.00667678	CTB-113D17.1	-1.22921655	0.03709364
DDHD1	0.69900165	3.5246E-05	HPSE	-1.22870818	0.00268683
UBR4	0.69889099	0.00484068	TSPAN15	-1.22865968	1.4453E-05
NDUFC1	0.69755082	5.4108E-06	ZNF354B	-1.22846167	0.00067661
N4BP2	0.69695706	0.00032711	MKS1	-1.2282789	0.01676591
SH3BP5	0.69623741	2.98E-05	RELL2	-1.22808724	0.00095412
CSTA	0.6949001	2.4488E-13	ZNF302	-1.22672622	0.00101917
TOM1	0.69295778	0.0234241	TMEM176A	-1.22641315	1.3381E-05
TSPO	0.69233074	1.3415E-07	PTGR2	-1.2263547	0.00030586
RCC2	0.69176423	3.5979E-05	ZNF717	-1.22627574	0.00224029
DBI	0.69141289	7.298E-11	CARF	-1.22572765	0.02330732
USP11	0.69116168	0.01445205	NPAT	-1.22467831	3.6454E-05
DDX24	0.69061117	4.7837E-14	SLFN12	-1.22466022	1.1309E-05
TMEM2	0.689575	0.04893551	AC240274.1	-1.22425813	0.00103296
PABPC1	0.68929308	1.0779E-17	VIPAS39	-1.22417235	6.5081E-05
UBL3	0.68929051	7.039E-06	CIPC	-1.22412424	0.0042066
EIF4A3	0.68891523	0.00292435	PPP1R35	-1.223212	4.3124E-08
SRSF6	0.68843595	1.4825E-07	IPO11	-1.22275465	0.02045914
TOP3A	0.6882145	0.01704216	POMZP3	-1.22108026	0.00625621
MRAS	0.68809754	0.03189449	HFE	-1.22087598	0.0040306
CD47	0.68780706	5.5709E-10	MED22	-1.21979082	0.00227102
TFG	0.6869909	2.4078E-05	PALB2	-1.21959099	0.00761017
PSPC1	0.68622119	0.00258191	MAVS	-1.21927006	4.5044E-09
STK40	0.68562308	0.01758083	AC109826.1	-1.21916018	4.4711E-05
GTPBP4	0.6856221	0.00014736	C9orf114	-1.21901409	7.4558E-05
ZC3H18	0.68549228	6.4393E-05	ZNF839	-1.21858188	0.02391296
INSR	0.68540498	0.0005906	ZC3H6	-1.21782509	1.7286E-06
FOXO3	0.68493173	1.4506E-07	LZTR1	-1.21765912	0.00419853
ELF2	0.68472821	1.681E-05	IFT80	-1.21710744	0.00642038
ACSL3	0.68426799	5.0801E-05	TAB1	-1.21162247	0.01542953
NLRC5	0.68286179	0.04803723	RP11-110G21.	-1.21148794	0.02413308
H3F3B	0.68209255	8.3559E-10	ZFP14	-1.21128313	0.00890699
HMGXB4	0.68105634	7.9429E-05	RGS18	-1.21092982	1.357E-21
DCP1A	0.68052011	0.0008201	GCSAM	-1.21080344	0.01831312
STAU1	0.67963257	3.6331E-06	CEP162	-1.21020888	0.00182851
COPA	0.67950694	4.5672E-06	FO538757.2	-1.21012438	0.00010714
TMEM120A	0.67946441	0.00402557	ZNF761	-1.21011556	0.00578646
TIMM17A	0.67762385	7.2509E-06	NVL	-1.20886828	0.00056233

SUCO	0.67532156	0.0133684	HGF	-1.20881946	0.03939278
RBM3	0.67459624	3.2349E-14	CEBPA	-1.20829499	1.9867E-09
ANKHD1	0.67401114	0.03964546	KIAA0319L	-1.20765763	7.469E-07
TGIF2	0.67395721	0.00495124	WDR81	-1.20657977	7.5444E-06
TMEM18	0.6735076	0.00078593	RBL1	-1.20651456	0.04843537
FABP5	0.67302061	5.291E-08	CENPJ	-1.20619384	0.00020407
ARID5B	0.67230873	0.04820878	AP006222.2	-1.20452187	0.00073264
CSF2RA	0.67120269	1.0575E-08	TMEM67	-1.20444373	0.04581758
TOR3A	0.67004635	0.00285555	RP11-334C17.	-1.20434436	0.00266903
TAF9	0.66951577	5.1451E-06	KLF6	-1.20276507	1.1702E-06
MAT2A	0.66853866	6.2847E-06	TM7SF2	-1.20258096	0.01354926
TMCC3	0.6682973	0.00116889	CRACR2B	-1.20208934	0.02073417
UCKL1	0.66787472	0.04936811	RSBN1L	-1.20101512	4.3675E-18
ARF4	0.66703171	7.7773E-06	BBS4	-1.20055464	0.00786549
RPL36AL	0.66672661	5.462E-17	IGFBP4	-1.20047161	0.00104364
CORO1A	0.66657871	5.1038E-07	DIP2A	-1.19949152	2.3815E-09
LILRB2	0.66122369	1.0857E-06	CX3CR1	-1.19889486	0.00821921
UBE2A	0.66039077	2.5049E-08	ZNF641	-1.19751927	6.0193E-07
FARP2	0.65997611	0.02114092	CLDN23	-1.19413296	0.04904623
SEC61B	0.65991201	6.1839E-21	TRPM2	-1.19233805	1.8859E-06
DCPS	0.65905099	0.01489496	SLC9A9	-1.19221574	2.2959E-08
CPM	0.6587314	1.8859E-06	ALG10B	-1.19221387	0.02128728
ANXA5	0.65775892	8.1579E-11	ZNF562	-1.1919761	1.1616E-09
CASP10	0.65760987	0.00786145	ZBTB48	-1.19032707	1.075E-05
GHITM	0.65752324	2.8835E-15	EGR3	-1.18808082	0.00642038
C9orf72	0.6561863	0.00014905	CCND1	-1.18746805	0.01128406
SRA1	0.65549386	5.0585E-09	ZNF646	-1.18668698	0.01642825
TDG	0.65455456	6.1238E-05	MYC	-1.18317199	0.02485971
RAB5A	0.6536589	1.672E-06	CD180	-1.18273215	7.0131E-09
IFIH1	0.65268634	0.01569056	NICN1	-1.1824553	0.0235736
CEP83	0.65248027	0.01128695	NBPF1	-1.18204658	0.00730676
HBEGF	0.65168776	0.00794388	ZNF629	-1.18131017	0.03658201
RHBDF2	0.65116472	0.0002676	FAM114A1	-1.18083337	0.00278574
KMO	0.65115223	0.0283665	AHSA2	-1.1805802	1.4789E-10
PCGF5	0.65027225	6.6941E-05	FUCA1	-1.17987948	0.00028237
TBC1D8	0.65013034	0.03431212	RNF8	-1.17953078	4.4335E-06
USP37	0.64986513	0.00965521	KCNMB4	-1.17851172	0.00207227
XYLT1	0.64983187	0.0178626	QPRT	-1.17783441	7.873E-05
ZNF511	0.64871245	0.00129392	KIAA0895L	-1.17753427	0.02076239
HNRNPM	0.6479867	8.1688E-15	TLN2	-1.17689483	0.00010778
ARIH1	0.64769768	0.00086952	FAM13A-AS1	-1.17554184	0.0386528
MED13L	0.64714808	1.1741E-05	ZNF548	-1.17534709	0.0009038
NQO2	0.64714747	0.00186307	STOML1	-1.17457817	0.01623438
ATXN1L	0.64657015	0.03719834	MTERF1	-1.17454043	0.02934148
NEDD9	0.6454714	0.00040007	EP400NL	-1.17290782	0.00096329
ATF6	0.64484558	6.3468E-06	IRF2BPL	-1.17158349	0.00078753

NGDN	0.64483586	0.00810848	DPY19L3	-1.1692013	0.01994645
N4BP2L1	0.6446944	0.00010655	LINC01410	-1.16699696	0.03138767
ATG2A	0.64455793	0.00089233	FIZ1	-1.16696466	0.04143334
CD37	0.64320261	3.4357E-10	SPHK2	-1.16667836	0.00826064
CHCHD2	0.64317819	1.4703E-19	MAPKAPK5-AS	-1.16640007	3.7978E-09
POMP	0.64184578	4.4677E-20	HEXIM2	-1.16558403	0.00695245
MRPS22	0.64119412	0.00306157	C12orf73	-1.16528252	0.00093968
EIF2A	0.64089272	2.8039E-06	SAP25	-1.16518449	0.00037091
FAM129A	0.64084389	0.01353146	TCEB3-AS1	-1.16499821	0.03812738
GAS6	0.64042541	0.00425508	FKTN	-1.16493403	0.02981033
WDR55	0.64020021	0.03832073	HN1L	-1.16486269	0.00135469
MORF4L2	0.64004402	1.1758E-06	RHOBTB3	-1.16221575	2.7269E-05
MTA1	0.63962367	0.01886851	TBCK	-1.16105641	0.00348174
ELMSAN1	0.63955913	0.00032449	SMYD4	-1.15953503	2.1626E-05
H2AFZ	0.63894566	2.324E-06	TP53INP1	-1.15929227	0.00028528
CCDC93	0.6388166	0.02184712	HELQ	-1.15921295	5.4352E-05
RHEB	0.63862104	2.7406E-07	CDC42EP1	-1.15907507	0.00424672
C11orf1	0.63513128	0.03429842	TFB1M	-1.15781895	0.00020596
OPA3	0.63471645	0.03533466	PRKD2	-1.15770488	0.03403851
ATP6V1G1	0.63470655	9.1151E-20	NAIP	-1.15731211	1.8264E-17
ZBTB16	0.63110441	0.04776071	KIAA0226L	-1.15725966	0.00353728
RANBP2	0.6310713	0.0002121	SMAD1	-1.15717736	0.03328281
FBXW7	0.63029186	0.00152032	GCNT2	-1.15622753	0.0071725
PAFAH1B2	0.62908643	0.00012668	ANKAR	-1.15568383	0.01525807
MT-ND6	0.62869576	0.00029419	AMBRA1	-1.15560998	0.03687033
PRRC2C	0.62757985	4.119E-13	RP11-556E13.	-1.15517659	0.0215833
GABPB1	0.62731989	0.00042327	HGH1	-1.1536567	0.00378144
ATP1A1	0.6272736	6.6833E-05	LRRC25	-1.1532648	9.3271E-13
DYNC1H1	0.62668737	1.3642E-05	ZFP36L2	-1.15236075	2.2564E-08
LAPTM4A	0.62567534	1.4093E-08	AARS2	-1.15230962	0.04148321
C16orf72	0.62553324	0.00018726	SPATA5	-1.15138384	0.00512573
CNOT2	0.62499088	1.9919E-06	ALDH1A1	-1.15097595	3.5729E-08
POLR2A	0.62443979	0.00102464	TRIM65	-1.15024901	0.01516438
STAT1	0.62321702	0.00126819	BORCS6	-1.15015239	5.085E-05
TMEM189	0.62320344	0.0138421	GALM	-1.14920533	1.7385E-11
MOB4	0.6229519	0.00216013	PROCA1	-1.14902181	0.00888444
ZFYVE16	0.62274683	0.00166755	RNF25	-1.14800402	0.01286136
HMGB2	0.62250331	8.7194E-06	ZNF740	-1.14755728	0.03179252
MTHFD1L	0.62173366	0.03561779	IRF5	-1.14726604	1.2179E-06
VPS37B	0.62158145	0.00695245	DHX57	-1.14719823	0.02756654
RNF149	0.62072702	1.3238E-09	HDX	-1.14687127	0.00978754
MIS12	0.61785402	0.0300279	PPAT	-1.14665486	0.0062333
EIF4A1	0.61754031	8.3006E-14	FAM13A	-1.14610553	9.219E-07
CSGALNACT2	0.61738633	1.8859E-06	NCKAP5	-1.14591806	0.00928947
HPRT1	0.61636151	6.8568E-07	ZNF708	-1.14499209	0.00400976
LDLR	0.61563801	0.00743205	COLGALT1	-1.14489858	3.2818E-13

OLA1	0.61546328	3.5217E-06	SAC3D1	-1.14425619	8.2196E-05
RHOH	0.61533844	0.02528335	FGD3	-1.14243929	4.7217E-09
SFXN3	0.61439647	0.01572415	KLHDC10	-1.14153652	0.00098467
CWC25	0.61420296	0.00271107	CD320	-1.14145992	0.00234448
SNRPB	0.61418473	7.0168E-14	GSPT2	-1.14137481	0.04219729
QPCT	0.61374006	0.02679243	CTU1	-1.14080086	0.00338469
DBF4	0.6134221	0.00368264	MTX3	-1.14017197	0.04016191
GAPDH	0.61334961	9.1589E-22	TIMM21	-1.1395554	0.00164235
DCTN6	0.61312296	1.57E-05	ZNF383	-1.13861871	0.02665307
ENOSF1	0.6126065	0.01237371	ZNF180	-1.13854855	0.04842878
PURB	0.61234364	0.0014248	RBM41	-1.13759112	0.00345942
HINT1	0.61215936	1.3752E-13	TCHP	-1.13565684	3.0595E-05
CCDC12	0.61055166	1.4314E-05	SLC37A4	-1.13547746	0.00563567
SUB1	0.60989602	2.227E-26	MTERF3	-1.13519687	0.01172206
NEAT1	0.60895551	7.1602E-09	CCDC152	-1.13518479	0.0452175
SNRK	0.60800656	0.03327582	BMP2K	-1.13396001	2.931E-09
MCL1	0.60747862	1.5906E-08	DNAJB4	-1.13352101	0.00218997
CDK16	0.6063336	0.01092746	CYP2U1	-1.13292346	0.00275317
RBM39	0.60603347	9.671E-13	MAP1S	-1.13227135	1.6411E-06
MTHFD2L	0.60537143	0.00588655	AC022182.3	-1.13206984	0.03640145
RNF7	0.60506364	2.4272E-09	C17orf67	-1.13195946	0.00654744
KHDRBS1	0.60395301	5.8761E-11	ARHGEF9	-1.13166336	8.2619E-05
SERINC5	0.60295668	0.0044687	PRKAB2	-1.12906384	0.00418478
UBAP1	0.6026924	0.01531087	P2RY13	-1.12694022	1.7035E-11
RP2	0.60213808	0.0005682	ZNF576	-1.12648494	0.00358968
DNAJB12	0.6002019	0.00222088	CENPL	-1.12593048	0.02059424
LIMS1	0.59978511	9.8898E-09	FBXW8	-1.12454929	0.03442508
ZNF787	0.59969011	0.01396869	ZNF438	-1.12368252	0.04088279
ANKRD11	0.59931159	2.6998E-06	DIEXF	-1.12366299	0.03179252
R3HDM4	0.59911437	0.00730102	LRRC14	-1.12292027	0.01354081
MTHFS	0.5980809	0.00809417	RP5-832C2.5	-1.12228975	0.0008562
TMEM41B	0.59699024	0.03126089	ZNF414	-1.12047959	0.00033716
SBNO2	0.59681889	0.03964543	USP51	-1.12000799	0.0385401
YWHAQ	0.59637583	1.5001E-05	MCM9	-1.11962701	0.00417903
NFIL3	0.59574923	0.00134261	GAB3	-1.11924973	1.5865E-05
RALGDS	0.59511094	0.04600513	EFNA4	-1.11772972	0.0297834
CRELD2	0.59406601	0.00390065	POLA2	-1.11628027	0.00391392
PTPRE	0.59391179	2.1441E-10	TTI2	-1.1161525	0.00945016
HEXIM1	0.59344709	0.01460333	CLDN7	-1.11591895	0.00017055
IL18BP	0.59262966	0.01566972	CLEC9A	-1.1154272	7.3259E-06
SNRPG	0.5924032	1.5262E-10	ZNF865	-1.11530737	0.00996692
PITPNB	0.59228271	0.00167806	TMEM144	-1.11515941	0.00050888
ADGRE2	0.59162976	0.00048596	NEU3	-1.11383096	0.00205745
PNRC2	0.59135369	1.4446E-08	CSF1R	-1.11327435	6.3158E-39
SPTY2D1	0.58953858	0.00108079	PFKFB4	-1.11219064	0.04110587
RPL4	0.58953284	4.5911E-27	PPP1R37	-1.11205501	0.02495441

METTL21A	0.58875497	0.00746076	TRIM56	-1.11171211	4.7217E-09
ATXN2L	0.5871021	0.02598634	TTC7B	-1.1115976	0.03967941
GPI	0.58657533	0.00235262	LYPLAL1	-1.11105779	2.3364E-06
COMMD5	0.58482775	0.00376239	SGOL2	-1.11060565	0.03305856
LAP3	0.58469921	1.5179E-09	SEMA4C	-1.11045781	0.00104616
RPS16	0.58425094	2.8863E-23	ABCC3	-1.11013382	0.02436231
ILF2	0.58390922	1.9933E-09	WBSCR27	-1.10886045	0.00371151
CARD19	0.58332126	0.00168895	IDH1	-1.10871938	1.5575E-08
CHD2	0.5828816	0.00194064	SFMBT1	-1.10852466	0.01210224
USE1	0.58278545	0.00357106	THAP5	-1.1081046	5.5859E-07
SDCBP	0.58242186	6.812E-07	AGGF1	-1.10805013	4.0628E-11
ARF5	0.58056917	0.00057319	KRBOX4	-1.10787472	0.0011912
SLC35E1	0.58056067	0.00138408	GEMIN5	-1.10727033	0.04097454
HIST1H1C	0.58040117	0.00614295	NRBP2	-1.10708453	0.04108067
HNRNPA1	0.578353	6.1356E-29	DGUOK-AS1	-1.10687099	0.00158427
SLC3A2	0.57827262	0.00638411	CBL	-1.10618787	2.1128E-14
UQCRFS1	0.57646954	2.2254E-08	KIAA1551	-1.10570579	7.3512E-10
CDC42SE2	0.5763397	1.7113E-10	CEP76	-1.1054043	0.02895945
CSTB	0.5757452	1.2474E-13	ZYG11B	-1.10535097	6.5218E-06
VDAC2	0.57517484	6.6303E-08	KCTD6	-1.1041738	0.00064978
EIF1AX	0.57439879	5.5515E-09	NME6	-1.10298918	0.00019663
CCNY	0.572956	0.0012435	ZMYM3	-1.10177619	0.00119949
SNRNP25	0.57284756	0.00688367	DNASE2	-1.10124431	1.7137E-05
RABIF	0.57265461	0.00763346	ZDHHC21	-1.10058661	0.01384286
GMPPB	0.57024184	0.03026324	ANKZF1	-1.10057856	3.3135E-05
PDXK	0.56976534	9.3042E-05	RECQL5	-1.10053069	0.01610896
SEPT11	0.56975687	0.03874107	KLHL8	-1.09975872	2.035E-06
ILF3	0.56943836	0.00011864	PARP2	-1.09902419	0.00688553
IL6R	0.56742399	0.01022128	IER5	-1.09837987	4.4114E-12
CCZ1	0.56709951	0.00789357	NSUN3	-1.0982563	0.00807841
SMARCE1	0.56656939	8.8053E-05	MIB1	-1.09642548	0.00017892
GOLGB1	0.5664227	3.3106E-09	SLC25A30	-1.09640265	0.04182634
ASMTL	0.56629644	0.00807841	NOL8	-1.09534601	5.2105E-12
EWSR1	0.56611622	4.101E-06	SH2B1	-1.09326861	0.000208
CCT2	0.56546507	5.4089E-06	RHOBTB1	-1.09311083	0.01240062
OSBPL8	0.56531497	1.1123E-06	OXLD1	-1.09219803	1.0571E-07
CD72	0.56529237	9.1602E-05	XPO7	-1.09190913	9.2043E-05
SERPINA1	0.5652617	9.9797E-05	KRCC1	-1.09170597	2.12E-08
ARHGAP26	0.56416479	0.00809417	RNF146	-1.0904483	4.1574E-06
AUP1	0.56373752	1.982E-06	GNAI1	-1.08994057	0.00115072
GNG7	0.56255963	0.02821579	ZBED5	-1.08992713	0.0001298
RLIM	0.56190934	0.00552067	ZNF93	-1.08975421	0.01956546
DIMT1	0.5617991	0.04770616	NOL6	-1.08907864	0.0328257
SRC	0.561261	0.02421153	DPH7	-1.08893693	0.00125006
PCBP2	0.56040878	7.4084E-16	SNHG12	-1.0886784	4.5378E-07
NOP14	0.5593323	0.04601462	OXNAD1	-1.08863583	0.02928018

ALYREF	0.55721942	0.00144079	NLRC4	-1.08719819	0.00230228
PHLDA1	0.55710412	0.0026279	RAB39A	-1.08702838	0.00271107
C1QBP	0.55667825	5.3916E-09	DCUN1D4	-1.08633552	0.00741265
RPL23	0.55571412	2.4914E-17	BLOC1S3	-1.08623524	0.01401842
FAF1	0.55547128	0.0211714	GIMAP2	-1.08586746	1.5497E-08
ABT1	0.55373462	0.02243221	SLC46A2	-1.08461058	0.03074682
DNTTIP2	0.55350803	0.00252381	HEATR5A	-1.08455245	0.00012658
AP3S1	0.55331586	7.9312E-06	FLJ37453	-1.08407157	9.4896E-05
NOP58	0.55294241	0.00011897	ZNF785	-1.08311987	0.00928947
SFPQ	0.55221909	2.0894E-06	NARFL	-1.08239325	0.00150627
KAT6B	0.55220777	0.03214366	CAD	-1.08233895	0.02009641
ARL6IP1	0.55003076	0.00014364	MMP25	-1.08067212	0.00035374
PNO1	0.54950358	0.00272511	UHRF1BP1	-1.08002922	0.01345894
ATP6V0D1	0.54895006	1.7194E-07	KCTD5	-1.07944393	9.052E-07
TOP1	0.54841048	8.9625E-08	RCCD1	-1.07919272	0.00587852
MRPL18	0.54841035	0.01170813	RP5-899E9.1	-1.07913047	0.03413417
HSP90B1	0.54829699	5.3546E-08	RBMS2	-1.07873677	0.00057563
EPB41L3	0.54829357	0.00577621	MOSPD2	-1.07826186	6.0198E-07
SEC13	0.5482016	0.00012463	MARCH1	-1.0778895	7.7971E-12
FOXP1	0.54785129	0.00310501	GNPDA1	-1.07784128	2.029E-09
FOXN3	0.54782942	2.9625E-06	ETV5	-1.07647768	7.5161E-05
FAM210A	0.54692264	0.02422091	ENTHD2	-1.07634012	0.01605154
CAST	0.54663719	7.1468E-05	KCTD3	-1.07410518	0.01400518
PRCP	0.54614658	2.6032E-11	SLC1A3	-1.07395445	3.0934E-06
CUL1	0.54524125	0.02437079	CAMSAP2	-1.0737226	0.00199919
HIST1H4C	0.54469866	0.0054356	KBTBD3	-1.07351723	0.00778211
GNAI3	0.54425572	0.00034394	OXSM	-1.07251752	0.00103282
RPL3	0.54422487	9.6301E-23	CPNE2	-1.0720483	0.00572275
TCEB1	0.54419983	8.5452E-05	PHC3	-1.07131242	7.3703E-06
JAK1	0.54419463	3.242E-06	QSER1	-1.07097504	1.2475E-05
OTUD4	0.54406584	0.02439096	L3HYPDH	-1.07053017	0.0279261
PCBP1	0.54393781	5.043E-11	IPO8	-1.07046902	6.3162E-05
MTHFR	0.54366114	0.00744097	DNAJC16	-1.06928995	0.00070693
PRMT1	0.54361924	3.3866E-07	CEP290	-1.06903376	6.6856E-07
MFSD14A	0.54326555	0.02259934	RP11-347P5.1	-1.06762109	0.00158427
UBE2S	0.54251984	0.00405727	PHC2	-1.06727468	1.5761E-11
LYN	0.54229812	1.7552E-06	ARHGAP6	-1.0668453	0.03056366
RPS11	0.54181192	1.8058E-15	FMNL2	-1.06581769	0.00857938
MSN	0.54165152	9.108E-08	AP4E1	-1.06552189	0.00056504
TAGLN2	0.54131474	2.0739E-07	CTSF	-1.06533154	0.01566972
CD99	0.54075432	0.00152395	RB1	-1.06374059	1.8087E-13
UBE2R2	0.54027881	6.3218E-07	WDR3	-1.06349185	0.00179846
NOTCH2	0.54004074	0.01611126	AASDH	-1.0623456	6.5561E-05
RQCD1	0.53952297	0.00319184	SLC16A5	-1.06140944	0.01749098
DAZAP1	0.53838604	6.23E-05	ZBTB14	-1.06088702	0.01800678
ATP5B	0.53772458	5.8374E-16	PGPEP1	-1.06019316	7.8371E-06

UBE2F	0.53763687	0.00038569	TEX2	-1.05986401	0.00974825
CTNNBL1	0.53758949	6.4419E-05	SASS6	-1.05985823	0.01710876
PSMD2	0.53692448	2.9262E-06	GMNN	-1.05951334	0.00044933
WHAMM	0.5368414	0.00658612	CROT	-1.05920638	0.00077871
SH3TC1	0.53668282	0.00078417	PRPF39	-1.05671831	0.00047114
HNRNPF	0.53548444	9.1327E-09	TFAP4	-1.05587807	0.03025523
CENPW	0.53496488	0.02034973	HS1BP3	-1.05583502	0.00090434
PDHA1	0.53389213	0.00017287	SAMD9	-1.05552115	0.00011721
CDK2AP2	0.53320315	2.7738E-05	UAP1L1	-1.05441728	0.00167577
PAFAH1B1	0.5331445	4.5733E-05	RANBP10	-1.05434087	0.02697492
MBD2	0.53301535	5.2562E-07	RASSF1	-1.05430403	2.4062E-06
VASP	0.53217047	3.5333E-06	MTO1	-1.05396803	0.00012954
HDAC1	0.53201798	0.0011912	TRA2B	-1.05352725	5.67E-14
RPL5	0.53114	1.5396E-17	NAA40	-1.05300003	0.00156369
OTUD5	0.53106246	0.01807594	SHQ1	-1.05299822	0.01494115
NACA	0.53069742	9.5658E-21	SNAPC3	-1.05253892	0.00183009
CDC37	0.53020787	1.1153E-07	KLHL9	-1.05250584	6.6858E-05
APP	0.52976927	0.00933287	ZNF25	-1.05221185	0.0130635
KCNAB2	0.5296436	0.00635432	MS4A14	-1.05207438	0.01856467
FAM3C	0.52938199	0.00724352	RAD51D	-1.05130218	0.02093059
MED10	0.52835857	0.00019307	FBXO46	-1.05129033	0.03741265
COPS2	0.52753696	0.00095181	CPOX	-1.05118747	0.00438457
CRK	0.52714943	0.04412381	XXbac-BPG299	-1.0509568	0.04475518
RAD51C	0.52688337	0.02616219	SPIRE1	-1.04983521	0.03905503
MRPS6	0.52674244	0.01544987	CEP250	-1.04903789	0.00176664
DTD1	0.52666237	0.01800286	FAM221A	-1.04902574	0.04397872
TRABD	0.52650625	0.00406569	CCL4L2	-1.04887066	0.00994366
CTNNBIP1	0.52615522	0.00736686	WDR92	-1.04819487	0.03546591
WAS	0.52614499	3.4056E-08	NPHP3	-1.04810427	0.00766452
AMZ2	0.52507955	0.01679778	ZNF224	-1.04777568	4.1491E-06
RPS13	0.52499888	1.037E-18	HYAL2	-1.04715928	0.00219963
SLC25A5	0.52428482	6.8446E-12	PUS7L	-1.04668031	2.1396E-08
GNL3	0.52422156	0.00052257	ITPR2	-1.04475933	7.4833E-15
NPM1	0.52403071	2.7788E-20	CYTH4	-1.04420777	8.4906E-12
RASGEF1B	0.52359878	0.02565164	CCP110	-1.04338309	0.00025931
HTATSF1	0.52357566	0.01687202	CAPS	-1.04300057	0.02713368
NET1	0.52204952	0.02063029	MAP3K14	-1.04267205	0.00729098
PTPN2	0.5219045	5.7978E-05	ABCC4	-1.04160958	2.94E-06
HMGN1	0.52175054	6.9139E-10	HS3ST1	-1.04147541	0.04059442
EIF3E	0.52162502	6.257E-14	DDX58	-1.04074124	0.00148367
MORF4L1	0.52130313	4.0098E-12	CCDC112	-1.04012533	1.0017E-05
LTA4H	0.52119122	0.00101258	ELAVL4	-1.039954	0.00049387
HSPD1	0.52104335	0.02065384	TGFBR2	-1.03901818	2.8334E-10
AKAP8	0.52099147	0.01401842	FAHD1	-1.03895743	1.7476E-06
PSMD9	0.52003267	4.882E-05	ITFG2	-1.0373577	2.4378E-05
RPS7	0.5199219	1.7309E-24	FAM134C	-1.03726105	0.00031796

KDM4B	0.51970534	0.02476204	TLR1	-1.03696627	3.2604E-09
ADSL	0.51914114	0.00067354	DHX35	-1.035842	0.04974249
MZT1	0.51869071	0.01351879	ACAD10	-1.03462272	0.0168435
APOBEC3G	0.51730635	0.04395174	GKAP1	-1.03420119	0.00973716
WDR33	0.5169903	0.02063011	MTF2	-1.03315898	2.1315E-13
EIF3J	0.51682924	2.4433E-05	AP3M2	-1.03264984	0.00286008
IFNGR2	0.51678794	0.00341219	ZNF37A	-1.03185626	0.0001795
PELI1	0.515549	0.01182206	KIN	-1.03100044	3.498E-10
NCL	0.51550635	1.1023E-08	JRKL	-1.03070596	0.00616736
BTF3	0.51462981	9.3592E-17	ABCC5	-1.03066932	0.00761612
PGK1	0.51428758	8.323E-11	HERC4	-1.03008566	0.00047885
ARF1	0.5142831	3.5031E-07	RTP4	-1.02981898	0.00957028
MCFD2	0.51343777	0.00232254	RAD51B	-1.02898223	0.04936811
SMCHD1	0.51263643	0.00396013	TRUB1	-1.02819368	0.01471547
TCP1	0.51249058	0.00826753	POP7	-1.02786975	1.4318E-06
NR1D2	0.51179958	0.02330732	TMCC1-AS1	-1.02681571	0.04029668
RPS4X	0.5110232	1.0519E-16	ASUN	-1.02554601	0.00406342
EIF3L	0.51068252	7.686E-10	CCDC66	-1.02340445	5.5312E-07
DDX39A	0.51039842	0.00165514	CWF19L2	-1.02246215	8.2865E-08
USP3	0.51025659	0.00197318	RP11-192H23.	-1.02235514	0.04113064
PTGER4	0.50990288	8.5323E-05	RICTOR	-1.02227374	1.2518E-07
ALDH2	0.50975187	0.00046275	ACYP1	-1.02029518	0.01390686
RASSF2	0.50872864	0.00729031	RP11-568N6.1	-1.01932944	0.00693757
BRK1	0.50867019	9.351E-14	RCBTB2	-1.01883131	2.1562E-09
ALCAM	0.50860721	0.03786787	NUP43	-1.01842753	0.00186042
CALR	0.50849265	0.00038569	AXL	-1.01782738	1.4545E-07
DERA	0.50844519	0.00604792	ZNF766	-1.01723506	0.00059367
BLOC1S1	0.50791233	4.2973E-06	CALCOCO1	-1.01304168	0.0001401
GAS7	0.5077913	0.01409229	TMEM51	-1.01152586	1.6713E-06
HM13	0.5075634	0.00075703	NIT1	-1.01137077	3.5911E-08
TAP2	0.50741306	0.00339296	MED7	-1.01135767	2.0001E-05
METAP2	0.50720801	4.0476E-07	SCD	-1.01124938	0.01361794
MAP4K1	0.50557136	0.04027869	RBM15	-1.0110633	0.00434538
RPL18A	0.50515284	5.4007E-17	METTL3	-1.01068829	0.03427015
EIF3I	0.50492089	1.6095E-06	RP1-313I6.12	-1.01049112	0.0010298
DPH3	0.50486738	0.01116302	FAN1	-1.0093368	0.01460333
DECR1	0.50416753	0.00070693	FOXN2	-1.00820622	2.7701E-11
ATF4	0.50388837	0.00208617	ESF1	-1.00799953	3.9637E-10
RPS5	0.5033579	3.6657E-21	ZMYM4	-1.00716492	2.9761E-06
NOLC1	0.50248502	0.02713368	VPS8	-1.00671988	7.9502E-06
RAB9A	0.50218046	0.0046656	RRS1	-1.00625201	0.00136217
TCF25	0.50127601	4.5498E-12	GORASP1	-1.00582693	0.00328134
FDFT1	0.50107762	0.02411072	PEX1	-1.00547975	0.00362772
DERL2	0.50089736	0.0015222	OTUD6B-AS1	-1.00456847	7.9947E-14
BIN1	0.50063867	0.0047596	SCAPER	-1.00448688	1.3553E-05
ATP6V1H	0.50016757	0.02119649	TMEM106B	-1.0042038	5.1548E-05

GNB2L1	0.49963651	1.3327E-18	BTBD10	-1.00350174	0.00040529
GLRX3	0.49834179	5.0343E-05	GATAD1	-1.00316814	3.0931E-08
HEBP2	0.49823587	0.00343799	AC004951.6	-1.0007864	0.0140091
EIF3H	0.49762874	3.8193E-13	ZNF780B	-1.00039136	0.01265051
ENO1	0.49717269	2.3551E-09	FAM193B	-1.00006152	0.02366617
DPM1	0.49713982	0.00096712	KCNJ2	-0.99827234	0.04120044
ZNF277	0.49659077	0.01277406	SDHAF1	-0.99784747	0.00023186
MRPS15	0.49583177	8.5997E-05	NFE2L1	-0.99763053	0.000216
RTN4	0.49506353	8.4671E-06	ZNF493	-0.99739671	0.01614456
CCDC59	0.49496008	5.2873E-05	ZNF561	-0.99714354	0.00146961
CYTH2	0.49466871	0.0254417	CBLB	-0.99683997	0.00509641
MAPKAPK2	0.4936508	0.02885032	VPS72	-0.99604095	2.7814E-06
ATP6V1F	0.49255929	2.0917E-06	ZNF638	-0.99603998	6.532E-15
SNU13	0.49172191	6.0518E-10	HMG20A	-0.9953028	0.000534
RPL14	0.49091327	1.6674E-17	ZNF101	-0.99514299	0.03605971
C1orf162	0.49038655	0.00056064	ACP5	-0.99486367	3.3825E-06
XRCC6	0.49019283	1.1094E-06	KCNE3	-0.99485358	0.0001085
PSTPIP2	0.4901541	0.00091831	EDEM3	-0.99477624	3.6735E-07
GNG5	0.48961653	2.3858E-11	C9orf66	-0.99439999	0.04409522
METTL9	0.4893103	0.00024283	B3GALNT1	-0.99434152	0.04029359
RPS8	0.48878615	1.5692E-24	ARFIP1	-0.99392331	6.8534E-08
BCLAF1	0.48815055	4.8651E-06	TLR8	-0.9936079	4.6488E-05
PER1	0.48797451	0.00010778	ZNF217	-0.9921618	8.4018E-06
RPL8	0.48789968	1.8028E-15	CEPT1	-0.99173504	7.1279E-05
RAN	0.48714785	1.1473E-11	ABHD18	-0.99134614	0.00746076
LILRB3	0.48647257	0.00615031	UBXN7	-0.98997665	6.05E-07
SRSF3	0.48546599	8.3561E-05	ENTPD5	-0.98981177	0.01693691
HIPK1	0.48524877	0.00720262	EZH1	-0.98958943	6.4134E-05
IAH1	0.48507383	0.00022308	KRIT1	-0.988578	7.5287E-06
MBOAT7	0.48498732	0.01438589	FAM213A	-0.98811645	9.6774E-06
CCDC6	0.48461504	0.01471547	CD209	-0.98692079	2.5649E-07
SBDS	0.4845187	0.00964501	TRIP11	-0.98630354	3.603E-09
CFL1	0.48442278	1.1512E-21	TMEM106A	-0.98592405	0.00911477
HSD17B10	0.4834714	0.00027009	RP5-1065J22.8	-0.9841743	0.03332228
EIF3A	0.48323942	3.2838E-09	BTN2A1	-0.9837846	2.8298E-05
ZFP91	0.482977	0.01566953	ASB3	-0.98328742	0.03460234
NUP58	0.48260733	0.04133172	GMDS-AS1	-0.98328229	0.00704932
FXYD5	0.48204266	5.9642E-06	EHBP1	-0.98252557	0.00710145
RPS24	0.48198906	9.6642E-17	LIG4	-0.9821031	0.00585566
RPSA	0.48133678	5.8857E-15	ANKMY1	-0.98162781	0.04608045
ALDOA	0.48060125	3.4726E-09	UBL7-AS1	-0.98150026	0.00074154
EMC8	0.48036495	0.02962354	MUM1	-0.98128846	3.2055E-05
TMEM57	0.48019275	0.04206714	LATS1	-0.98025075	0.00011792
RPL19	0.47973874	1.414E-13	ALKBH1	-0.98008196	0.02987235
EIF3M	0.47926381	1.4295E-10	GATM	-0.97892154	1.1224E-06
SH3BGRL3	0.4790522	3.6859E-12	DDI2	-0.97856935	1.6185E-05

RPS9	0.47876774	3.5042E-18	FANCF	-0.97749011	0.04846328
CLIC1	0.47805733	7.5543E-11	FAM78A	-0.97598687	0.00052835
SNRPF	0.47781938	9.7725E-06	RAD9A	-0.97553025	6.1849E-05
DLD	0.47780557	0.00885979	ZNF143	-0.97552753	0.00084369
NAPG	0.47766951	0.03976118	ATG4C	-0.97474946	6.2866E-07
NDUFV1	0.47686934	2.2046E-05	ANKFY1	-0.97471849	0.00012327
SLC25A6	0.47666498	7.908E-22	C5orf45	-0.97471295	0.04526201
GLTP	0.47577693	0.0457956	UACA	-0.97469265	0.03381616
RPL32	0.47545166	2.1988E-20	DTWD2	-0.97330964	0.02345755
GOLPH3	0.47524615	0.01036734	NEK9	-0.97310843	0.00058892
CERS6	0.47413222	0.01632679	CXorf21	-0.97265646	0.00015804
CREBBP	0.47298242	0.01892702	C2CD3	-0.97259339	0.04868118
TUBGCP2	0.47196599	0.00054625	TANGO6	-0.97236663	0.04600513
WDR43	0.47102804	0.00339225	LEMD3	-0.9704987	0.00390704
NDUFV2	0.47097946	5.0464E-07	CHAMP1	-0.96998426	0.02476204
SRPRA	0.47083608	0.00702594	FAM104A	-0.96882069	0.00095912
EEF1A1	0.47033426	3.8328E-18	DHRS9	-0.96856424	0.00291762
ERGIC1	0.46905632	0.02089108	PCGF6	-0.9681157	0.01939083
TOMM22	0.46904475	7.3674E-05	SLC20A1	-0.96786341	2.1581E-17
COX17	0.4690377	2.7307E-06	ZMYND8	-0.9676316	7.1086E-05
ENSA	0.46855299	2.3364E-06	DISC1	-0.96727674	0.0028131
RPL11	0.46785946	3.496E-14	EXOSC4	-0.96712518	4.1435E-06
RPS3A	0.46762549	1.4987E-17	PTGR1	-0.96680225	0.00387681
RPS23	0.46517632	3.2533E-13	MACC1	-0.96677939	0.03987727
PTRHD1	0.46495612	9.1106E-05	PRR14L	-0.9666126	1.4116E-07
TARS	0.4646787	0.01444371	LRRC8D	-0.96624964	0.00013495
RPL34	0.46462827	1.2444E-09	AKAP11	-0.96587038	0.00011471
TRAPPC10	0.4641503	0.02986034	AP2A2	-0.96576156	1.89E-06
BCL10	0.46369911	0.02531993	NUP35	-0.96512764	0.04875496
CYSTM1	0.46300544	0.00631305	ATP8B4	-0.96371327	4.2371E-11
SERBP1	0.46213726	6.9385E-09	UMPS	-0.96336348	0.00222503
PRKAR2B	0.46117994	0.03976118	PLA2G4A	-0.9632968	0.0050223
LONP2	0.46109584	0.00575074	SUPT20H	-0.96312879	6.272E-06
PRDM2	0.46093307	0.00388639	NOC3L	-0.9624933	0.00017541
AFMID	0.46066055	0.0336633	PECR	-0.96237871	0.03306207
FDX1	0.46065502	0.02382367	RP11-61J19.5	-0.96147956	0.00259878
CNIH1	0.46042915	0.00148637	C7orf43	-0.96098337	0.01371011
FH	0.45986511	0.03395816	SERPINF1	-0.96049353	7.873E-05
KLF3	0.45985899	0.00669714	SYNJ1	-0.9604158	0.0158919
SRSF9	0.45979589	4.3715E-05	TFCP2	-0.96040214	1.3402E-05
TMEM167A	0.45970948	6.9376E-06	APOC1	-0.95982704	0.02038381
RBM4	0.4595334	0.01263117	UEVLD	-0.95935356	0.00391639
MFSD12	0.45930911	0.00699563	KIAA1586	-0.95825772	0.0051014
RPL37	0.45909234	5.6174E-14	FUK	-0.95666934	0.047344
CDV3	0.459092	0.00165514	FPGT	-0.95518218	0.01687101
IVNS1ABP	0.45851096	0.03079405	SAMD9L	-0.95493486	6.726E-05

PSME2	0.45749553	5.8797E-07	UBL4A	-0.9548394	0.01011523
UBA52	0.45701891	3.7987E-18	C9orf64	-0.95363098	0.012642
SLIRP	0.45690366	2.2287E-05	C6orf47	-0.9534567	0.00149714
DNAJA2	0.4557072	0.00313236	RCAN1	-0.95230289	0.00056674
RPS15A	0.45557568	9.8592E-17	TMEM161B-A	-0.950976	0.02105909
UBE2D2	0.45484295	1.0554E-06	POLI	-0.94900462	0.0014146
ALG5	0.45450832	0.0162771	CYP2R1	-0.94843319	0.0321421
PPP1R2	0.45399589	5.4404E-06	PIP4K2B	-0.94806205	0.00574864
HNRNPA3	0.45375549	1.3415E-07	PREPL	-0.9475635	0.0015683
ZFC3H1	0.45371662	0.01157864	DUSP1	-0.94519134	8.9584E-10
ARHGAP21	0.45346487	0.00838038	CHORDC1	-0.9451066	3.5057E-06
TMC6	0.45282081	0.02817261	ZNF148	-0.94404618	2.3278E-08
RPS18	0.4523016	1.5033E-13	CLEC4F	-0.94390403	0.00071237
VTI1A	0.45153644	0.03575692	MGAT4A	-0.94327955	1.5882E-09
RPL10	0.45096424	3.5582E-19	TMEM129	-0.94300041	0.00730861
ANXA7	0.45089823	0.00037915	ATP10D	-0.9419411	2.3155E-05
RPL18	0.45063142	3.5054E-15	MBD6	-0.94150049	7.7188E-05
C16orf13	0.45060473	0.00535456	XRRA1	-0.94146893	0.01943946
DDX3X	0.45049714	0.00193504	TRMT10B	-0.94066769	0.0013408
SERP1	0.45015676	3.0868E-14	LDAH	-0.94055353	0.03231475
CEBPG	0.44869088	0.03179252	RAB33A	-0.94005222	0.03025716
SMARCB1	0.44839485	0.04385355	IER2	-0.93971505	1.018E-06
BLOC1S6	0.44836297	0.00041797	ATP6V0A1	-0.93967175	2.7025E-11
LYRM4	0.44734456	0.01275654	C17orf62	-0.93954852	4.5037E-15
OGFRL1	0.44708428	0.00014404	ZC2HC1A	-0.9392757	0.01137977
RPS14	0.44672588	1.978E-08	NBPF12	-0.93925261	0.00032597
TTC19	0.44640086	0.02694754	ZNF654	-0.9388955	0.00162278
RPL9	0.44630186	8.4395E-17	ATXN2	-0.93833429	1.2238E-07
ARL5A	0.44617968	5.9101E-05	TMEM60	-0.93715514	2.4692E-06
FNBP1	0.44614525	0.00073837	KLF12	-0.93685189	0.02118283
RNH1	0.44589925	4.4085E-12	RNF14	-0.93667768	0.00011475
RPL10A	0.44551849	8.0523E-15	RNF169	-0.93658195	7.6998E-06
RPL7A	0.44519323	9.0804E-17	RHBDD3	-0.93645419	0.01918421
ASAH1	0.44471861	0.00227349	NOTCH1	-0.93590909	0.00189428
RPL35A	0.44471112	7.066E-11	TMEM53	-0.93590699	0.00202225
RBBP6	0.44440459	0.02952211	HMOX1	-0.93522149	2.0181E-09
VAPA	0.44408896	1.1643E-05	CC2D1A	-0.93493503	0.00786145
TKT	0.44203626	6.0758E-09	FZD2	-0.93479176	0.01337614
DENND3	0.44180151	0.03060305	GABPB2	-0.93398586	0.00028237
MPC2	0.44168245	0.00037099	CLEC2D	-0.93387338	0.01518477
HNRNPAB	0.44139431	2.6692E-06	DOPEY1	-0.93383627	0.03713959
RPS3	0.44120347	5.9876E-16	ELMOD3	-0.93284054	0.00706474
PPP2CA	0.44092773	0.00142172	EXTL2	-0.93065071	0.02858153
RYBP	0.44071204	0.00097692	LAIR1	-0.92991084	5.8408E-08
JMJD6	0.44037125	0.01319244	C12orf4	-0.92986604	0.00092895
YBX1	0.44000081	2.89E-10	H1F0	-0.92914579	0.00010635

HNRNPC	0.43972009	0.00050162	TRG-AS1	-0.92820059	0.01983944
MCTP1	0.43959337	0.0076888	KIAA1468	-0.92781731	0.01608315
IGBP1	0.43886393	0.00071267	ADAT2	-0.92754503	0.02577345
LDHB	0.43875996	6.6245E-06	RP11-140K17.	-0.92682076	0.0030972
HADHA	0.43854267	1.1018E-06	TBP	-0.92604791	0.01420821
CHCHD3	0.43795156	0.01396966	PLEKHA1	-0.92505221	9.5785E-08
RPL15	0.43756395	1.2769E-17	BICD2	-0.92477045	7.8526E-11
NUDT1	0.43729629	0.0044687	TCF12	-0.92452466	0.00020252
SUPT4H1	0.4371652	2.0044E-05	ENG	-0.92397253	3.0931E-08
RPS2	0.43646728	3.603E-09	ATRN	-0.92376726	0.01603532
PSMB4	0.43602088	3.2047E-05	OTUD6B	-0.92362248	0.00022428
ATP5L	0.43549026	3.8793E-11	NIF3L1	-0.92359961	0.02754166
UCHL3	0.43506789	0.00578567	FAM217B	-0.92320188	0.00695245
FUS	0.43503259	1.0799E-06	SURF6	-0.92227146	0.00089973
RPL31	0.43487891	1.225E-07	ZNHIT6	-0.92210438	0.00043292
DCTN3	0.43482559	0.0098873	SUFU	-0.92196755	0.0106875
CCNI	0.434664	1.8362E-07	APC	-0.92116939	0.00011986
RPLP0	0.43436194	4.1273E-21	SLC23A2	-0.92043136	8.523E-05
PSMB3	0.43393158	4.5341E-07	KIAA1147	-0.92042478	0.02437079
IDI1	0.43374211	0.01294303	TSPYL4	-0.91985281	0.02674204
EIF3F	0.43371088	4.5728E-06	FADD	-0.91963637	0.01505297
H2AFY	0.43363815	2.5295E-05	ARHGAP22	-0.91959169	0.00202712
RNMT	0.43303114	1.3409E-05	ANAPC7	-0.91958064	0.00078267
UBE2Q1	0.43224923	0.04045082	IGHMBP2	-0.91917893	0.00884791
MTMR6	0.43201976	0.04153854	WDR75	-0.91874763	2.3708E-05
CXCL16	0.43172845	0.00112593	AGL	-0.91801806	0.00166465
NUDC	0.43135109	0.00642038	ASB8	-0.91748884	6.1116E-06
CLEC4E	0.43126901	0.00181409	CLUAP1	-0.91709725	0.01937752
NDUFA6	0.43068818	1.3119E-05	RNF217	-0.91638777	0.0008414
FAU	0.43060794	2.5989E-16	FAM73A	-0.91596199	0.00010518
KMT2E	0.43044954	3.8725E-05	TSPAN4	-0.91489358	1.1588E-07
TSSC4	0.43036676	0.01833048	ASCL2	-0.9146214	9.5353E-07
MAP2K2	0.43021579	0.00136554	FCHSD1	-0.91430103	0.04074088
MYH9	0.4298294	0.00242367	SLC27A1	-0.91390242	0.00035617
UQCRH	0.42931644	4.9081E-13	PTER	-0.91318726	0.01558734
ACO2	0.42921099	0.00841328	CYP4V2	-0.91224566	0.00485412
EIF2S3	0.42869817	7.2162E-06	LINC00662	-0.91156635	0.03449164
NBN	0.42791553	0.01194639	NLN	-0.91125281	0.00883035
UBE2H	0.42698357	0.0164109	MANEA	-0.91121872	0.01835361
ARHGDIA	0.42677719	4.1023E-05	RP11-88E10.5	-0.91097935	0.03595785
RAB18	0.42651478	0.01304991	ANAPC10	-0.91094076	0.00011864
PSMD6	0.42636686	0.00125041	CCL4	-0.90959564	0.02069092
SNRPD1	0.4263508	0.00026328	ZNF532	-0.90955694	0.04423635
NDUFA7	0.42581608	0.01011523	HLX	-0.9095506	0.00802751
WIPI2	0.4257223	0.00907128	ZNF83	-0.90948791	0.0001026
RPL24	0.42522001	3.058E-12	SERPINF2	-0.9091033	0.00057563

GPAT3	0.4249442	0.00986394	PRMT9	-0.90889397	1.6816E-07
PCNP	0.42464189	0.00018175	MGA	-0.90857196	0.00075574
TMED10	0.42337346	2.3615E-06	FAM198B	-0.90824711	7.2271E-09
COX7A2	0.42323095	1.1791E-09	TARS2	-0.90771824	0.00490023
MTPN	0.4227217	6.0938E-05	POLK	-0.90652005	0.00016547
RPL6	0.42222755	3.7229E-16	INTS3	-0.90581094	0.00018201
RPL30	0.42180123	2.8564E-14	GART	-0.905549	0.00046323
IRS2	0.42171903	0.00193155	ZNF117	-0.90549266	0.01050931
PSMA2	0.42155121	9.0252E-08	RFWD3	-0.9054678	0.04183442
UBE2I	0.42149634	2.7572E-06	PIGV	-0.90507474	0.01904755
RPL29	0.42123362	2.0159E-10	DGKH	-0.90502669	0.00390704
RPL21	0.42042164	3.9899E-14	NUP85	-0.9050147	0.00189428
RPS17	0.4199405	1.1783E-15	WDR73	-0.9044959	0.00014242
UXT	0.41975486	1.6429E-05	NSUN6	-0.90382368	0.00210446
RPS27	0.41934525	3.6141E-10	DDX55	-0.90378879	0.00027214
DNAJC15	0.41920195	2.6963E-08	MEF2A	-0.9037113	5.5312E-08
PLBD1	0.41916554	0.02480418	FCER2	-0.90352577	0.00744097
MPC1	0.4188267	0.00038819	FAM65A	-0.90348065	0.00057606
MRPL47	0.41877633	0.00232254	AGAP6	-0.90334278	0.03505207
ZNF394	0.41860522	0.01323458	TMEM203	-0.90313981	1.164E-06
HLA-DQB1	0.41851286	2.3307E-05	ZNF714	-0.9030431	0.00546719
EIF3K	0.41833112	2.4589E-10	FAM160B1	-0.90235964	0.01794861
SPCS3	0.41815121	3.8725E-05	HSD17B7	-0.90132848	0.0015518
PSMB7	0.41786984	0.00017177	CDC23	-0.90108252	0.01034583
CSDE1	0.41747475	1.9118E-07	DDX20	-0.90017189	0.01524345
PARVB	0.41724264	0.00494508	ZNF146	-0.89967833	0.00058622
IMPDH2	0.41701055	0.00035385	MIER2	-0.89948483	0.00789798
FAM162A	0.4166272	0.00070206	LSM14B	-0.89914466	0.00084978
ZCCHC10	0.41652445	0.02097067	RNF34	-0.89912199	0.00212955
GUK1	0.41638739	6.505E-06	ATF3	-0.89781051	7.7249E-09
PMPCB	0.41636695	0.00303087	CTSC	-0.89705085	3.849E-08
PKM	0.41632726	1.8073E-05	SAP30L	-0.89655315	0.00625388
EFHD2	0.41595323	1.8666E-07	MUS81	-0.89597637	0.00074591
RPS15	0.41540225	1.2915E-12	RP11-796E2.4	-0.89591145	0.00160219
DDX5	0.41483193	8.7186E-06	NFATC3	-0.89554259	0.00030436
CALM1	0.41466322	1.7277E-05	SFXN5	-0.89457268	0.00703822
SNRPA1	0.41384952	0.00566896	RNF111	-0.89442325	0.00167726
TMEM123	0.41358674	0.00046488	ZCCHC6	-0.89365461	6.962E-09
ING3	0.41332044	0.02210082	TERF2	-0.89200915	0.00078491
RPL36	0.41319446	8.8728E-10	WWC2	-0.89114978	0.02034272
GRPEL1	0.41311005	0.00509432	TTI1	-0.89081463	0.02463809
PPP4R3A	0.41291705	0.00061327	CPEB3	-0.88981556	0.03536825
RPL37A	0.41291267	7.5188E-12	LARP4	-0.88863499	2.1534E-05
BRE	0.41282559	0.02320887	LINC00847	-0.88860796	0.00497557
PRDX6	0.41267354	1.0799E-06	CEP57L1	-0.88816071	0.02305266
NOL7	0.41252494	0.00063525	MKNK1	-0.88744328	2.513E-07

PDCD4	0.41252081	0.00013675	RBP7	-0.88694925	0.03687819
EEF2	0.41221892	3.0661E-15	DOCK7	-0.88675576	0.00030668
NAA10	0.41181498	0.0028298	ZNF652	-0.88516226	6.0691E-05
PAPOLA	0.41166834	1.0298E-07	KANSL1	-0.88429812	2.5076E-06
DHRS7	0.41104711	0.00518449	THUMPD2	-0.88399174	0.00353803
TSG101	0.41058242	0.00658388	FAM98B	-0.88359476	0.0001295
PARVG	0.41035525	0.00226717	IFI44	-0.88327912	3.0966E-05
RPL7	0.40952156	3.486E-17	UHRF2	-0.88253118	0.00045684
SET	0.40885244	1.4643E-09	CPNE8	-0.88248371	2.7034E-06
ATP5F1	0.40843909	3.2059E-05	PARP10	-0.88208331	2.213E-05
CKLF	0.40835614	8.6754E-05	TRIT1	-0.88167959	0.0005386
DERL1	0.40811459	0.0118689	FDX1L	-0.880903	0.00829433
RAB11A	0.40796685	0.04407802	NUP155	-0.88060823	0.02436231
EMC3	0.4078197	0.00116739	LINC00909	-0.88010728	0.00200845
IKZF1	0.40759853	0.01375759	LENG9	-0.87903268	0.01423424
MYL6	0.40747294	1.2045E-10	DCAF17	-0.87778885	0.04772677
ARF6	0.40666047	2.7738E-05	VASH1	-0.87750488	0.00132827
NRBP1	0.4065762	0.01835251	ISCA2	-0.87569912	8.9758E-05
HIGD2A	0.40587324	1.2244E-08	LTC4S	-0.87554563	0.00493094
DHX9	0.40521388	0.01174931	QRSL1	-0.87531685	0.0039466
RPLP2	0.40483559	1.2663E-11	LIAS	-0.87437432	0.00070754
TOMM20	0.40470545	5.9744E-08	DTWD1	-0.87436413	1.7329E-05
PPDPF	0.40457724	0.01346949	FPR3	-0.87407094	1.1604E-17
PSMB6	0.40428377	5.8112E-06	CYB5D2	-0.87275943	0.00031745
FKBP2	0.40404014	1.5269E-05	SLC38A9	-0.87153859	0.03680664
RBM17	0.40365469	0.01260784	C12orf66	-0.87118894	0.04638511
RPS10	0.40351518	2.6232E-09	LIPA	-0.8709262	1.0348E-06
IRAK1	0.403185	0.04794459	DYRK2	-0.8707242	0.00231792
PFDN1	0.40291097	0.01524345	KCTD12	-0.8700043	4.3775E-09
RPL41	0.40289492	4.9576E-11	RP11-798M19	-0.86990659	0.01819446
RPL39	0.40266768	1.9311E-10	CEP192	-0.86946525	0.00469935
FXR1	0.40243418	0.00123955	TMEM87B	-0.86876974	0.00020364
MIF	0.40226294	0.00576743	RPP38	-0.86857096	0.00095289
SLK	0.40215633	0.01884366	MRPL35	-0.86850903	9.0992E-07
RRAGC	0.4021511	0.04907798	TUSC1	-0.86820071	0.04439574
RPL22	0.4019772	2.1019E-13	COQ6	-0.8680634	0.01468152
GNAQ	0.40171253	4.9953E-05	CREBZF	-0.86805034	3.0745E-05
LIMD2	0.40120951	0.00144939	CCR1	-0.86801634	5.4089E-06
RPS12	0.40119095	2.5567E-10	NEK6	-0.86781013	0.00605685
POLR1D	0.40106853	3.2586E-06	FOXRED2	-0.86634455	0.03167591
RPS21	0.40093366	6.6039E-09	INTS6	-0.86570026	2.1556E-07
RPL26	0.4005624	1.775E-11	UTP3	-0.86553895	9.8334E-06
HBP1	0.40052799	0.0062919	ERAP2	-0.86551766	0.00278974
UFM1	0.40003473	0.01560188	METTL13	-0.86439944	0.02059424
HSP90AB1	0.399068	0.02145772	PLOD1	-0.86410822	0.00316044
TPM3	0.39895687	8.5941E-09	ZNF701	-0.86385933	0.04515108

HNRNPH2	0.39881129	0.00258136	HLTF	-0.86379831	0.00063139
DSTN	0.39858715	0.01131145	ERCC5	-0.86311875	0.00032888
RAB7A	0.39775194	4.955E-06	PEX14	-0.86224436	0.04946307
DEK	0.39771222	3.3655E-06	BYSL	-0.86184558	0.0350363
TMSB10	0.39747074	5.4211E-07	SLC46A3	-0.86156621	4.0753E-06
CDC42	0.39677868	3.4101E-07	ARHGAP24	-0.86130151	0.02742592
RPS29	0.39609753	5.045E-07	STAM2	-0.86108272	0.00233885
GNB1	0.39523567	2.816E-05	ZNF808	-0.8609866	0.03920919
DDAH2	0.39390289	0.03348783	OMA1	-0.86079773	0.00158643
GPX4	0.39389309	1.1363E-05	CHD9	-0.86049168	3.8309E-10
FIBP	0.39365087	0.00037205	ULK2	-0.86040186	0.01813211
PUF60	0.39358069	0.00206729	PTGFRN	-0.85964394	0.0350769
EEF1B2	0.39318962	2.1132E-09	RNF125	-0.85880177	5.5471E-05
RPL27	0.39207714	1.0213E-09	VCPIP1	-0.85805861	8.4836E-08
CLN3	0.39146386	0.04214984	DNASE1L3	-0.85743292	8.7979E-08
ZEB2	0.39080655	0.002353	RDH14	-0.85522967	0.00031515
TRAPPC1	0.39079492	0.00011565	IRF3	-0.85446943	2.5891E-07
EIF3B	0.39078442	0.02070245	KRI1	-0.85314411	0.01756111
MZT2B	0.39052257	0.00058622	RTN2	-0.85289759	0.00222922
EPC1	0.39040213	0.02914899	EPHX1	-0.85289189	0.02102604
EIF5	0.3902201	0.00590938	GSKIP	-0.85235742	0.01046952
ANXA6	0.3901267	0.02425118	E4F1	-0.85216772	0.03331871
PSMB1	0.38974765	2.0119E-10	CDC27	-0.85200123	4.5786E-06
GTF3A	0.38950399	0.00032686	INPPL1	-0.85075568	0.00031835
G3BP2	0.38869589	0.01728322	WDSUB1	-0.85025566	0.01954281
TUFM	0.38859563	0.00014429	ACTR6	-0.84995069	0.00017287
MAN1A1	0.38840473	0.01302488	CLPX	-0.84975092	1.054E-05
COX5A	0.38831728	1.9339E-07	METTL12	-0.84928163	7.0857E-05
RPL28	0.38830037	2.2388E-13	RP11-395A13.	-0.84892477	0.0021507
USF2	0.38749051	0.01454598	TMCC1	-0.84787296	0.02667586
NR3C1	0.38670129	0.01162674	CHMP7	-0.84771751	0.01954281
PSMB9	0.38668572	0.00050061	RPS6KA1	-0.84678645	4.4417E-11
EIF4H	0.38555724	0.01621662	GLI4	-0.8462848	0.01082328
SRGAP2C	0.38543047	0.0385682	COX18	-0.8460626	0.00936106
IKBIP	0.38513489	0.03903919	RASAL2	-0.84561432	0.01544987
TMEM170A	0.38384966	0.01946562	PDRG1	-0.845189	0.00012613
HNRNPR	0.38372979	0.00012087	WDR36	-0.84452612	0.01090797
RPS26	0.38336015	9.6511E-12	C2orf74	-0.84394208	1.6733E-05
ZBTB7A	0.38306626	0.01602384	GGA3	-0.84363943	0.00148594
SPAG7	0.38296663	0.01009255	ZNF516	-0.84351186	0.00028872
TPM4	0.38275373	0.02168499	CHST15	-0.84315343	0.00277976
MRPL11	0.38147847	0.00288205	UVSSA	-0.8398551	0.01287972
HNRNPUL1	0.38137173	0.00281715	SCARB2	-0.83922778	5.0881E-06
RELT	0.38100474	0.03348263	CD14	-0.8389388	0.00147084
PSMA1	0.38094841	3.1795E-06	CLN6	-0.83889569	7.3507E-05
TMEM50B	0.38079464	0.02031423	EHF	-0.83784358	0.04518264

TAF10	0.38017138	0.0030131	SCN4B	-0.83719662	0.012472
UQCRB	0.38000041	1.0536E-10	ZNF384	-0.83693427	0.02059323
RPL23A	0.37915071	4.3775E-09	HOMER1	-0.83691543	0.04294715
MALAT1	0.37904021	0.00013489	DCAF12	-0.83684495	0.0020872
RPL27A	0.37871685	5.2423E-10	AMPD3	-0.83641274	7.2302E-05
UBA2	0.37854983	0.01984312	CH17-189H20	-0.83632368	2.46E-05
ATP5A1	0.37834252	2.5137E-06	TTBK2	-0.83592683	0.00046483
C19orf38	0.3775681	0.03278718	IQCG	-0.83549839	0.03435591
ANXA11	0.37731803	0.00026176	RNF170	-0.83523678	0.00264002
ARPC3	0.37665042	1.4301E-10	RGL1	-0.83513768	0.00056274
ACAA1	0.37613201	0.01542095	FAM122A	-0.83477147	0.00082537
ACTN4	0.37606351	0.01116302	EVI5	-0.83417613	5.062E-08
SNX3	0.37606022	0.00120575	GON4L	-0.83312297	1.4742E-07
PPP1R7	0.37602145	0.00794388	DICER1	-0.83184227	3.5991E-08
CISD2	0.37594486	0.01804345	C2CD2	-0.83168547	0.0481025
ETS2	0.37583414	0.01210222	SRBD1	-0.83114303	0.00601389
NDUFS6	0.37540081	0.00012587	PKD2	-0.83094414	0.01960866
NGLY1	0.37462513	0.03768407	FBXO30	-0.83081071	0.02588434
GALNT1	0.37450777	0.00505506	MS4A4A	-0.83000033	0.00173625
PMF1	0.37445797	0.04352917	TMEM222	-0.82906418	0.01008235
COTL1	0.37352627	8.8053E-05	ZNF44	-0.82860352	0.00491075
EEF1D	0.37318346	3.6247E-09	APPL2	-0.82799178	0.00981256
ARIH2	0.37262369	0.03938763	PALD1	-0.82780933	2.068E-05
YWHAE	0.37185676	1.7667E-05	DCAF15	-0.82752757	0.0049706
PSMC3	0.37183472	0.00340673	SEC24C	-0.82743661	0.04412381
CSF3R	0.37171026	0.00907032	KIF17	-0.82736867	0.00849382
GABARAPL2	0.3707856	0.00059176	ZNF226	-0.82589674	0.00030922
ORMDL2	0.37037124	0.01035252	FFAR3	-0.82565479	0.00922041
MYL12A	0.37022156	7.5796E-07	CTNND1	-0.82560094	4.786E-08
TUBA1B	0.36971136	0.00036394	FAM118B	-0.82543957	0.03596191
ARPP19	0.36949337	0.00250934	SLC35B4	-0.82502033	0.00312102
RRBP1	0.3677038	0.00550252	PDGFC	-0.82447022	0.00012926
ABRACL	0.36747391	1.1266E-06	PHF14	-0.82404358	1.4545E-07
CYC1	0.36723136	0.01971041	CKAP5	-0.82363276	0.00097072
CPNE1	0.36700236	0.00573029	C14orf159	-0.82354619	6.7621E-06
EIF4G2	0.36679396	0.00057783	TNKS	-0.8230762	0.00366929
PPP6C	0.36664816	0.04219729	ELAC1	-0.82274789	0.01461343
TYMP	0.36618099	0.00278724	AASDHPPT	-0.82167221	1.7451E-06
MAGOH	0.36602463	0.00057158	BRIX1	-0.82062148	0.00032605
LINC00152	0.3658458	0.01440318	SPECC1L	-0.82055821	0.017139
GBP4	0.36580925	0.02057127	FAM63B	-0.81945857	0.0055628
EIF3G	0.36520186	5.5114E-05	SLC7A6OS	-0.81939156	6.1133E-05
ATXN10	0.36465973	0.00880676	CBR4	-0.81923396	0.01123566
MGAT1	0.36463441	0.02070198	RRP8	-0.81887791	0.00052392
RPS6	0.36440483	9.4864E-10	AP5S1	-0.81849879	0.03946072
ARPC1B	0.36356302	1.0099E-05	R3HCC1L	-0.81815633	0.00592667

CCT6A	0.36349402	0.00041071	CASD1	-0.81763108	0.00705269
TAPBP	0.36251991	0.00020054	ANAPC4	-0.81750769	0.02966849
NANS	0.36148765	0.0002466	CCDC146	-0.81747366	0.02100303
CEBPZ	0.3610201	0.03196375	JMJD4	-0.81639266	0.04648345
SUPT5H	0.3606807	0.02800002	SAYSD1	-0.81616541	0.00320427
MED4	0.36033545	0.02539625	FBXL15	-0.8161252	1.3114E-06
APRT	0.36025689	0.00044	VPS54	-0.81507889	0.0046656
SEC61G	0.3602067	0.00013244	PPM1K	-0.81500127	2.4433E-05
OSTC	0.35984387	0.00062102	XAF1	-0.81494461	2.3424E-08
PSMC5	0.3591873	0.00017075	LAMP5	-0.81431422	0.00731646
ETFA	0.35879222	0.00125277	CEP295	-0.81267155	0.02302881
LSM7	0.35852231	4.0903E-05	PRPF38B	-0.81256255	5.6836E-17
SAT1	0.35814577	0.00026312	LOXL3	-0.81233797	0.03547841
COX6A1	0.35784525	7.3011E-06	RP11-472N13.	-0.81190873	0.00073891
DDT	0.35749024	0.00021	ARHGAP12	-0.81182868	0.04971025
TXNDC17	0.35735985	0.01993499	ANXA2R	-0.81083083	0.04422899
UPP1	0.35728876	0.02466651	CTBP1-AS2	-0.81065009	0.03406724
EMC7	0.35677147	0.0063097	TNPO3	-0.81048274	0.00178324
UFC1	0.35608596	1.9788E-05	TDP2	-0.81029507	0.00093883
PLEKHB2	0.35569518	0.00600038	SIRT6	-0.81009932	0.00073735
PPIA	0.35557205	1.5115E-10	BAG4	-0.81003765	0.00870336
MRPL54	0.35490532	0.00380966	TET3	-0.80998005	1.9564E-06
PPP1R14B	0.35479169	0.01400241	DCLRE1C	-0.80992143	0.01345894
ANP32B	0.35464176	1.3959E-05	ERV3-1	-0.80971694	0.00077089
PSMA7	0.35437181	2.0808E-10	SACM1L	-0.80930841	0.00038281
IDH3G	0.35371468	0.00319184	SMARCAD1	-0.80900324	0.01172432
RNF115	0.3537014	0.04300228	MCPH1	-0.80785628	0.00492999
UBE2N	0.3535332	0.00125473	C16orf52	-0.80773292	0.03935735
SAP18	0.3534295	1.4302E-05	ZNF43	-0.8076757	0.03204864
RBM47	0.35295284	0.03781406	WRB	-0.80688918	0.00314427
GALK1	0.35275544	0.04556163	PIK3C2A	-0.80589096	5.632E-07
CALM2	0.35154678	1.5393E-08	RASA1	-0.80444485	0.01589441
COPE	0.35122234	0.00013852	MRI1	-0.80421702	0.00329091
MRPL21	0.3505196	0.00806948	HDAC6	-0.8034202	0.01237371
NDUFB6	0.35048842	0.01107618	KIAA0586	-0.80328607	0.01954281
GTPBP6	0.3499266	0.04539189	PRKAB1	-0.80327241	0.01682356
PSMB10	0.34899982	0.02167966	HERC2	-0.80286709	0.0002466
VCP	0.34881126	0.00414625	UBR2	-0.80216554	2.0418E-05
RTFDC1	0.34865879	0.0007723	PLEKHO2	-0.80087146	0.00047236
MED28	0.34836569	0.00540538	ATE1	-0.80051099	0.00907018
ITGB1BP1	0.34830006	0.03073611	TEX10	-0.80030943	0.02645502
DARS	0.34768922	0.04183442	IRF2	-0.80000815	5.0325E-06
HLA-DRA	0.34702011	1.3276E-08	TLR5	-0.79857974	0.0007953
C7orf73	0.34680268	0.00097034	CARKD	-0.79790668	0.0319813
ARHGEF1	0.34668009	0.03211826	CCDC130	-0.79781746	0.00115837
IDS	0.34633404	0.00295481	ABCB4	-0.79768641	0.0111907

MED29	0.34611405	0.04659067	LINC00339	-0.79709076	0.04413715
CCNK	0.345877	0.03972071	IFRD1	-0.79677609	4.5982E-09
MRPS36	0.34538691	0.01192388	WASH1	-0.79663589	0.0010212
PDCD5	0.34529098	0.00635393	TSPAN31	-0.79657751	3.7122E-05
ERH	0.34498952	1.7978E-05	TBRG4	-0.79642225	0.00034999
NSMCE3	0.34455372	0.04898119	IFT88	-0.7962967	0.02634829
EP300	0.34445399	0.04287316	WDFY3	-0.79568145	0.00078379
EDF1	0.34330018	8.7871E-06	HPS4	-0.79502542	0.00129764
PSMB2	0.34319725	0.00101228	ELAC2	-0.79488659	0.00167577
SYF2	0.34253901	0.00026469	INPP5D	-0.79441844	2.0548E-07
C19orf43	0.34219047	0.01452838	YOD1	-0.79408638	0.04010581
COPB1	0.34204915	0.03478674	ALG12	-0.7936764	0.02345755
RPS25	0.34199175	6.4314E-09	CD84	-0.79331591	6.482E-06
ZNF207	0.34187341	0.00597154	ASXL2	-0.79284569	0.00022308
UQCRC1	0.341319	0.00291724	EXT1	-0.79244232	0.02069092
COX7C	0.3406533	6.907E-10	TTC38	-0.79228288	0.00847803
SPCS1	0.34062815	8.809E-07	USP28	-0.79171989	0.02440986
HNRNPL	0.33978785	0.03118938	RP6-159A1.4	-0.79109768	0.01277406
DDOST	0.33972033	0.01050163	SYNJ2BP	-0.79020221	6.6193E-05
WBP11	0.33966206	0.01712328	DSC2	-0.78832707	1.5314E-05
TMED5	0.33924073	0.03260979	AP3S2	-0.78831507	0.00697575
GLTSCR2	0.33923539	0.00351707	CCM2	-0.7882235	0.00198496
SERINC3	0.33883507	0.03191776	RFC4	-0.78767852	0.04475518
RPL17	0.33801098	0.04347212	ARAP1	-0.78752798	6.3247E-07
RPS19	0.33680289	1.8278E-08	ARIH2OS	-0.78749668	0.03285309
PSME1	0.33664767	9.938E-06	CTCF	-0.78732416	8.7623E-05
PPM1G	0.33629195	0.01425068	PHF12	-0.78692081	0.00318555
GSTP1	0.33619496	6.5751E-06	ADCK1	-0.78657575	0.04936811
STAG2	0.33569162	0.01234513	SYNRG	-0.78627194	0.00705664
SELT	0.33555342	0.00041797	CTNS	-0.7846935	0.02197078
MTDH	0.3354075	8.2805E-07	VPS39	-0.78450532	0.01804345
C9orf78	0.33444441	0.00337567	MTIF2	-0.78414744	6.5218E-06
FBXO7	0.33420108	0.03782278	FAM208A	-0.78387809	5.9128E-05
CCT8	0.33398931	7.1279E-05	ENPP2	-0.78372934	0.00839849
CIB1	0.33369814	0.0012359	HENMT1	-0.78198996	0.01468763
WBSCR22	0.33303673	0.01617954	TFEC	-0.78180718	1.0539E-05
TROVE2	0.33295137	0.03189729	SLF1	-0.78093808	0.00578646
PRKDC	0.33251793	0.01846481	UPF1	-0.78072527	0.00041797
SF3B1	0.33194324	0.00326702	FAM126B	-0.78042394	0.0090108
SF3B2	0.3316478	0.00090556	HECTD4	-0.78026967	0.02437079
XRN2	0.33123407	0.00042904	MPHOSPH8	-0.77918137	8.1249E-13
NDUFA10	0.33076723	0.00238559	KDM4A	-0.77859096	0.03197376
SKP1	0.33021246	5.3086E-05	RP11-67L2.2	-0.77634499	0.02342182
USP7	0.3296804	0.02872232	F13A1	-0.77619273	0.00199562
ADSS	0.32946253	0.04732184	ERCC6L2	-0.77593839	0.00712864
POLR2L	0.32924566	0.00022366	DIS3L	-0.77482056	0.00642533

PFN1	0.32902245	2.6774E-06	LUC7L3	-0.77470512	5.6049E-09
CCT5	0.3289324	0.00474515	IKBKB	-0.77429842	0.00059028
ADRM1	0.32885789	0.00918954	MTAP	-0.77429345	0.00040362
RSL24D1	0.32867244	0.00027511	PCED1A	-0.77408063	0.02128747
HNRNPU	0.32823427	0.00069609	ST6GAL1	-0.77384312	0.00069758
PSMD7	0.32821139	0.00101228	RRAGB	-0.77245585	0.01258601
RPL13	0.32778334	4.6419E-05	SMAD5	-0.7723497	0.0133684
PARK7	0.32735474	9.0735E-06	TDRD3	-0.77205655	0.00112593
CLEC10A	0.32692549	0.00115486	NLRP1	-0.77176776	0.00147806
FAM133B	0.32659465	0.00173094	GATC	-0.77162756	0.00608134
RPL36A	0.32586374	0.00014821	NCOA7	-0.76958576	0.00016313
FDPS	0.3252	0.01120286	PPP2R4	-0.76929168	4.2627E-06
LAMTOR5	0.32483589	0.00020097	WDR7	-0.76860703	0.01036734
PRDX2	0.32476829	0.0133537	RENBP	-0.76815137	0.00706759
PSMC4	0.3239514	0.00343787	HELZ	-0.76798407	7.5796E-07
STXBP2	0.32391044	0.00722248	OAS1	-0.76793662	0.00592139
ATG3	0.32305524	0.01434971	ENGASE	-0.76727897	0.00559346
FBL	0.32246336	0.01195171	TMOD2	-0.76601025	0.04133172
UBE2L3	0.32245868	0.00114261	SPRYD7	-0.76586613	0.04150097
HNRNPK	0.32033956	4.6016E-09	NFRKB	-0.76554191	0.03281822
TALDO1	0.32001925	0.00048434	MRPL46	-0.7652465	0.00630426
COX4I1	0.32000969	1.6713E-06	CETN3	-0.76399573	0.02485971
NSA2	0.31890297	0.00034359	PIAS2	-0.76391207	0.00145054
IK	0.31874475	0.00167251	CNST	-0.76347785	0.01341883
EIF2S2	0.31860183	7.3674E-05	CCDC18	-0.76217408	0.01835361
NDUFB11	0.31852127	4.0191E-05	RBM12B	-0.76121982	0.01235574
GMFG	0.31847609	0.00059996	BET1	-0.76109273	0.00118348
GSN	0.3183298	0.00783229	TMEM91	-0.7609956	0.03813451
AURKAIP1	0.31820104	0.00140255	CEP78	-0.76038326	0.02000483
RPS27A	0.31761776	9.5161E-10	TIFAB	-0.75932533	0.00652368
PGLS	0.31747631	0.02634386	LGALS3BP	-0.75877476	0.00869843
FAM96B	0.31728276	0.00252153	ERF	-0.75763357	0.00842045
NDUFB8	0.31694598	0.00025512	ZDHHC7	-0.75745707	1.9036E-06
ERGIC3	0.31686565	0.02424696	CDK19	-0.75745531	0.00031436
SMDT1	0.3168408	0.00370445	OSBPL1A	-0.75645387	0.00022259
CAT	0.31675898	0.00064488	FLI1	-0.75616408	0.00017509
TBCA	0.31612051	1.207E-06	MTR	-0.75573166	0.00013852
RNPS1	0.3160681	0.00685527	ORC4	-0.75566031	0.01296688
HEXB	0.3158519	0.00678545	THAP3	-0.75522287	0.04219729
PFDN5	0.31539763	1.6211E-08	PDCD7	-0.75500287	1.2758E-05
CMPK1	0.31538128	0.01815471	QTRTD1	-0.75494581	2.1195E-05
MAP3K2	0.31533714	0.00869843	SLC33A1	-0.75409194	0.00717054
DAP3	0.31532699	0.02276446	PIGC	-0.7537414	0.00215992
SRRM1	0.31525771	2.2917E-07	POLD3	-0.7536981	0.00196362
STAT3	0.31503716	0.02395591	LTN1	-0.75346373	0.00980298
SMARCC1	0.31485438	0.02479972	POLR2J3	-0.75294936	1.5921E-06

PSMD8	0.31473631	0.00967395	SLF2	-0.75207583	0.00596342
COX7B	0.31446328	6.7463E-06	SENP7	-0.75014753	0.00761017
PTP4A1	0.31399189	0.02324999	SUMF1	-0.750025	0.00042984
DNAJC7	0.31382382	0.01043827	DHRS4-AS1	-0.74965401	0.00732974
FAAP20	0.31353584	0.01332444	SNX19	-0.7496178	0.01002383
RPL38	0.31219249	1.091E-05	POU2F1	-0.74945896	0.01240062
RPL12	0.31194677	6.9548E-08	ING4	-0.74934889	0.00012478
C1orf43	0.31186786	0.00168483	ABHD5	-0.74902057	0.00757352
NUDT21	0.31182629	0.04679569	CMTR2	-0.74837392	0.04010368
LGALS2	0.31142739	0.02798357	ALPK1	-0.74815682	0.04308317
RANBP1	0.31113455	0.00412869	SESN3	-0.7466188	0.00036356
ATP5C1	0.31105084	1.2475E-05	ORC3	-0.74618872	0.00698228
ARF3	0.31096981	0.0467023	CDKAL1	-0.74618408	0.01700491
PTPRC	0.31088704	1.4563E-05	USF1	-0.74442622	0.00759284
ZC3H15	0.3100352	0.00261169	ANKRA2	-0.74417821	0.03672
MRPL14	0.30989434	0.02314206	RNF113A	-0.7438962	0.00064463
ARPC2	0.30928549	1.2968E-05	LINC00324	-0.7438672	0.01248366
LSM12	0.30898721	0.03704	SOS1	-0.7437978	0.00253956
COMMD6	0.30829959	3.8088E-05	RCHY1	-0.74346135	0.00025627
COX5B	0.30766241	0.00056674	HCFC2	-0.74337666	0.04992309
ZNF706	0.30765997	0.00146323	C11orf54	-0.74313046	0.00689033
THRAP3	0.30626807	0.00995585	TNFSF10	-0.7428512	3.9383E-05
UBXN1	0.30509793	5.6193E-05	NUDCD3	-0.74227294	0.00717054
UQCRC2	0.30488049	0.00822553	ST7L	-0.74139175	0.01076421
RPN2	0.30453715	0.02275761	BISPR	-0.74129241	0.00432624
NDUFB4	0.30434318	0.00056426	BTN3A1	-0.74110411	0.00792875
BLOC1S2	0.30409424	0.02095656	ZBTB7B	-0.74110197	0.01274463
COX7A2L	0.30405096	0.00484532	ALAS1	-0.74083624	0.00326415
CPQ	0.30401756	0.02741019	SLC39A10	-0.73983484	3.563E-05
ATP5J2	0.30363408	0.00024847	ZZZ3	-0.73965779	0.02714399
CCNDBP1	0.30263972	0.02235926	NELFA	-0.73927901	0.02421153
NDUFS5	0.30186572	5.5541E-05	GCC2	-0.73890689	2.0091E-07
SRP14	0.30177304	7.1691E-08	CREG1	-0.7387256	5.7506E-06
TMA7	0.3014789	7.5846E-05	TRIM27	-0.7386331	8.9758E-05
GADD45GIP1	0.30143917	0.02178788	ADPRHL2	-0.73862306	0.00024107
PDIA6	0.29997775	0.00107274	TTC1	-0.73786439	2.1559E-08
SRP72	0.29940047	0.00578646	PSMG3	-0.73718733	5.1908E-05
ERP44	0.29895548	0.00222176	ARPIN	-0.7366659	1.1423E-08
HNRNPD	0.29800231	0.01018681	MRFAP1L1	-0.73645562	0.02363197
RPL13A	0.29780799	2.0298E-08	TANGO2	-0.73622009	0.0017742
ATP5G2	0.29722945	1.3303E-10	DNAJA3	-0.73605211	0.04361993
MYL12B	0.29690684	2.7271E-05	ZCCHC11	-0.7358968	0.00212955
SCAND1	0.29388514	0.01475972	ELP3	-0.73584983	0.00585566
MRPL23	0.29375894	0.04287316	METTL7A	-0.73393451	8.0929E-08
RPS28	0.2937191	1.5007E-07	ANKRD54	-0.73294597	0.04999313
LSM3	0.29355217	0.00306612	HRH2	-0.73292259	0.00122496

THOC7	0.29327853	0.00347935	DCAF5	-0.73242818	0.00187373
TNFSF13B	0.29127206	0.00021412	TAF1	-0.73240174	0.00058696
RPA3	0.29104328	0.03236221	PRR14	-0.73177012	0.00292029
TXNL4A	0.29091043	0.03132795	TRIM13	-0.73104954	0.0027308
ACTB	0.29020769	4.8878E-05	ZNF22	-0.7309428	3.5786E-05
EIF6	0.28923195	0.00320976	SVIL-AS1	-0.73035957	0.03073611
CSNK2B	0.2886713	0.00229837	APOL2	-0.73012836	0.00731343
PAIP2	0.28857985	0.00980942	AGO4	-0.72977428	0.00023644
TMED2	0.28848061	0.01500218	OSBPL11	-0.72924618	0.00029463
GSTO1	0.28809891	0.00011122	ATHL1	-0.72912516	0.02197078
NDUFS8	0.2879836	0.00309164	MLLT10	-0.72856887	5.5114E-05
LGALS1	0.28727373	0.0223238	ITM2C	-0.72838763	5.7392E-06
IQGAP1	0.28629929	0.00741387	TMEM187	-0.728126	0.02823836
SRRM2	0.2858964	0.00316228	SLC31A1	-0.72799617	0.00033407
WAC	0.28527784	0.00679097	NSL1	-0.72786607	1.2811E-05
ATP5G3	0.28442728	1.5375E-05	C9orf91	-0.72764395	0.01839862
CMIP	0.28298107	0.04663888	LPIN2	-0.72733907	9.6403E-05
GNAS	0.28281522	0.00076673	CMB9-22P13.:	-0.72715062	0.04495723
SNRPB2	0.28281132	0.00194766	MAML2	-0.72613751	0.00718054
PRPF40A	0.28234648	0.00117557	NCF4	-0.72598942	7.4696E-13
SSR2	0.28229258	0.00125105	NAGK	-0.72568918	4.316E-09
MINOS1	0.28186463	0.00089629	TMEM168	-0.72560667	0.0194793
NCF2	0.2814696	0.02034973	ZDHHC14	-0.72545861	0.01156362
SUMO1	0.27928919	0.01927072	PAPD4	-0.72530914	9.6013E-06
LCP2	0.27818482	0.04912408	METTL17	-0.72443574	0.0068058
FAM50A	0.27734882	0.04600513	ALG8	-0.72423663	0.00077284
BAX	0.27656278	0.00294766	CPED1	-0.72327532	6.9985E-05
SULF2	0.27655932	0.02163681	COIL	-0.72306141	0.0022188
RPS20	0.2748553	8.0098E-08	TMEM206	-0.72138381	0.00371312
USMG5	0.27381294	8.6485E-05	TRUB2	-0.72133242	0.01168871
NDUFA11	0.27372411	0.00016456	NHLRC3	-0.72092185	0.0006928
MDH2	0.27332938	0.00294637	LACTB2	-0.72056854	0.01950644
SIVA1	0.2717074	0.04326272	NAA25	-0.71982828	0.00338893
COX8A	0.27165283	0.00255271	TRAPPC11	-0.71948981	0.02456063
OST4	0.27151848	0.00018603	PRIMPOL	-0.71823455	0.04357229
MAP3K13	0.27118409	0.04425899	ZNF33A	-0.71755656	0.00017756
RPLP1	0.27116914	1.6991E-07	GLE1	-0.71640839	0.04515108
MRPL3	0.27080143	0.02475389	ZNF720	-0.71492941	0.00888444
CAPZA1	0.26989565	0.00367756	C19orf52	-0.71469819	0.02147164
BNIP2	0.26985031	0.03814976	C16orf87	-0.71374699	0.00048448
RPL35	0.26924534	1.5255E-06	LTBR	-0.71328564	1.5473E-08
FAM49B	0.26924275	0.00521188	TBL3	-0.71327657	0.01643247
SEP15	0.26922863	0.00210567	ARHGAP27	-0.71270412	0.00098681
ARPC5	0.26896052	0.00046582	MR1	-0.71156346	0.00028446
H2AFV	0.26829054	0.04095282	HAX1	-0.71155383	0.0002177
C14orf166	0.26749129	0.0048654	S100A13	-0.71144188	0.00361426

HMGN3	0.26720394	0.02197078	NAT9	-0.71087341	0.00967395
ZFAS1	0.26712491	0.00171538	KIF1C	-0.71076078	0.01747697
PRKAR1A	0.26653697	0.0106213	PCMTD2	-0.71036877	0.01538632
LSM5	0.26599509	0.02781933	TIA1	-0.70946223	0.00040546
IDH3B	0.26575142	0.04057111	LYRM7	-0.70900806	0.00113099
ST13	0.26263237	0.0002605	GATAD2B	-0.70893848	0.00176779
GRB2	0.26225466	0.00093968	CYB5R1	-0.70884218	3.6679E-06
RAB31	0.2615705	0.00389284	COQ9	-0.70870484	0.00741571
SON	0.26130052	0.00389184	TPD52	-0.70831702	0.03405341
IQGAP2	0.26122453	0.04024916	SMIM8	-0.70799652	0.0164109
PTMA	0.26114011	3.1746E-05	GCLC	-0.7069222	0.00029645
C8orf59	0.25925148	0.01302976	EPS8	-0.70684517	0.04418817
OXA1L	0.25915169	0.02450532	ZMAT5	-0.70605454	0.00092701
SUMO2	0.25902378	0.00017403	PAAF1	-0.70583993	0.02107542
CASP1	0.25826961	0.03028697	KIAA0430	-0.70572717	0.00047249
SSR3	0.25802354	0.00344424	ADCK3	-0.7054895	0.02387087
COX6C	0.25743401	0.00049804	BTN2A2	-0.70375729	0.00162133
PILRA	0.25518857	0.04557617	ABCG1	-0.70116741	0.0175239
QKI	0.25511788	0.02685859	FUNDC1	-0.70077447	0.00258181
SPCS2	0.25476128	0.00329025	C3AR1	-0.70063352	2.9303E-07
PRELID1	0.25194324	0.01472523	HS2ST1	-0.7006108	0.03726013
B2M	0.25126807	0.00100177	AAMDC	-0.70042337	0.00563327
SNF8	0.25124667	0.04976735	BUD13	-0.69997433	0.04227896
TBCB	0.25119663	0.01884366	CRLF3	-0.69987263	0.0018231
S100A11	0.25116103	0.00014417	METTL14	-0.69959738	0.01314535
ROCK1	0.25046712	0.01388655	ARHGAP17	-0.69885191	0.00973609
ATP5E	0.24992684	1.7506E-05	NCEH1	-0.69855435	0.00878258
GLIPR1	0.24928622	0.0333554	REXO4	-0.69832117	0.00226709
SYNGR2	0.24926944	0.0139127	RXRB	-0.69816768	0.01906048
C9orf16	0.24919359	0.03920919	COA6	-0.69686507	1.1516E-06
FCER1G	0.24729527	0.00236927	SLC25A38	-0.69665352	0.00801928
CHMP2A	0.24643428	0.03374822	USP46	-0.69648394	0.03658201
RAB5C	0.24624695	0.00882136	MB21D1	-0.69645462	0.0041172
ATP5G1	0.24193183	0.01916349	ZMYM5	-0.69623474	0.00056748
TPI1	0.23854587	0.00133249	LNPEP	-0.69540564	1.9063E-05
IL13RA1	0.23792787	0.03464043	TRIP4	-0.69502565	0.01017158
CLEC2B	0.23417801	0.03046121	RP11-352M15	-0.69371662	0.04928163
PPIB	0.23378607	0.00080375	RFC2	-0.69318189	4.7755E-06
SH3BGRL	0.23378388	0.00381231	MED11	-0.69271718	0.00067633
GSTK1	0.23377317	0.01835361	PURA	-0.69150542	0.00034024
SNRPE	0.2333378	0.02574782	THUMPD3	-0.69138677	0.00047019
EIF3D	0.23187878	0.00802546	KMT2E-AS1	-0.69091482	0.03919247
RSL1D1	0.2309608	0.00994366	USP33	-0.69033369	5.0154E-05
EIF5A	0.23073826	0.02440651	ASB2	-0.69014826	0.02469142
SYNCRIP	0.2303397	0.01691943	NUDCD1	-0.69005424	0.02145772
SNRPD3	0.22924161	0.00903983	NCOA6	-0.69002025	0.04680959

ATP5I	0.22872025	0.00284094	CSE1L	-0.68970813	0.03719834
KIF5B	0.22835669	0.03975635	CFAP97	-0.68967381	0.00078742
TMEM258	0.22690481	0.01792432	TCF4	-0.68929675	0.00016189
DNAJC8	0.22623265	0.01497732	CARD8	-0.68919803	2.5537E-06
MT-CO2	0.22529376	0.01035016	SLC4A7	-0.68912206	4.438E-05
SRSF7	0.22482493	0.01115432	MRPL30	-0.68884926	0.00202911
PSMA4	0.22364336	0.02979786	NEK3	-0.68883262	0.00148594
CAMTA1	0.22183481	0.04974249	SLC37A2	-0.68860113	0.04068971
SSR1	0.2206127	0.03197376	GDE1	-0.68802392	5.6063E-05
ATP5O	0.2195535	0.01712105	KCNK6	-0.68740246	4.8776E-05
FKBP1A	0.21823674	0.01712105	NBR1	-0.68723657	0.00789357
HLA-DPB1	0.21560234	0.00250391	RGS19	-0.68688868	1.5913E-06
MOB1A	0.21462027	0.00407962	SCO1	-0.68677355	0.00317041
NDUFA12	0.21226347	0.03546591	MAPK8IP3	-0.68628623	0.04202985
SSR4	0.20957441	0.00981256	RUFY2	-0.68521326	0.01904168
OAZ1	0.20817646	9.8076E-05	RAE1	-0.68414059	4.8521E-08
HLA-B	0.20686167	0.0111274	LIMD1	-0.68395388	0.03533466
C14orf2	0.20586597	0.01887689	DFFA	-0.68283053	0.00088922
NDUFA13	0.20534487	0.01345731	ICT1	-0.68269376	0.0004198
SNHG8	0.20391425	0.00819219	HINT3	-0.68251996	0.00673299
RAP1A	0.20330735	0.01043471	MRPS23	-0.68085724	0.00091458
NDUFA2	0.20148854	0.04936811	ZNF195	-0.68045847	0.02406253
ATP5H	0.19936148	0.0229405	TSC22D4	-0.68017248	0.00025568
ACTR2	0.19692601	0.01619247	TSFM	-0.67979418	0.00391977
SERF2	0.19625339	0.00889434	PGGT1B	-0.67933727	0.00103882
PPP1CA	0.19342876	0.03032067	C1orf131	-0.67817582	0.00483668
DRAP1	0.19337093	0.02934148	ITGAV	-0.67804445	0.01496824
NDUFA1	0.19333629	0.02730289	FGD2	-0.6774621	1.9541E-05
HCLS1	0.19124022	0.03152768	CBR1	-0.67730244	3.8558E-06
NDUFC2	0.19059055	0.02306437	PPP1R21	-0.67699384	0.00134465
ENY2	0.18847596	0.03247137	PHF21A	-0.67663552	0.01618559
RNF130	0.18779026	0.01650911	ATR	-0.67631842	0.02273603
ACTG1	0.18614323	0.02918364	GOLGA1	-0.67621868	0.03341683
MT-ND1	0.18532365	0.02128728	PHIP	-0.67617175	2.0181E-09
GNAI2	0.18082001	0.00524916	SLC39A7	-0.67548578	0.00114239
TPT1	0.17004851	0.00457198	PREB	-0.67544045	0.02475889
YWHAB	0.16865839	0.01659018	MLST8	-0.67494973	0.00791636
NDUFB2	0.16638768	0.03768407	DOPEY2	-0.67424835	0.04452894
COX6B1	0.14798698	0.03109044	TBC1D14	-0.67299711	0.00059511
HMGB1	0.13233015	0.01673387	MADD	-0.67269878	0.02728022
			BTN3A2	-0.67264728	0.00514344
			KIAA0355	-0.67227956	0.04540601
			GIT2	-0.67201158	0.00047249
			LRRC47	-0.67172884	0.01584633
			SPOP	-0.67164749	1.7974E-05
			SRGAP3	-0.67145532	0.00250934

CD151	-0.67128128	0.00090556
ZNF280D	-0.67104267	0.00397887
NCF1	-0.67088673	8.5323E-05
AARS	-0.66995403	0.01454598
PHF3	-0.66975939	1.036E-07
CTA-292E10.6	-0.66955349	0.01582138
MYSM1	-0.66906795	0.0026414
PPIL2	-0.6685761	0.00319605
TMEM69	-0.66813129	0.00982008
NUPL2	-0.66793932	0.00015325
GABPA	-0.66726341	0.01007989
ADCY7	-0.66708979	0.0011309
SLC11A2	-0.66684356	0.0248361
MSR1	-0.66607991	0.00022074
ICE2	-0.66561152	0.00049685
ARL14EP	-0.66554427	0.00012954
PUM3	-0.66382557	0.00043354
KDM5A	-0.66377757	9.8391E-07
RGL2	-0.66343499	0.0071725
DMXL2	-0.66337254	0.00078808
CLCN7	-0.66237069	0.00108799
HAUS3	-0.66225146	0.02818364
CH17-340M24	-0.66145505	0.00019618
MTFMT	-0.66138339	0.00384978
RNF40	-0.6599784	0.03673881
OSTM1	-0.65952653	0.00161716
PSAP	-0.65889111	1.0193E-11
ABCD4	-0.6580345	0.00166319
CTR9	-0.65779268	0.00162771
EXOSC3	-0.65696227	0.00856655
LCORL	-0.65638532	0.03004422
CYB561A3	-0.65596953	0.00031333
CDK10	-0.65536788	0.00746076
TTC9C	-0.65523543	0.03116533
ZNF675	-0.65522974	0.00903983
TMEM192	-0.65519024	0.0085636
CBX1	-0.65452902	0.00118168
FAM105A	-0.65398081	3.8152E-07
ABCD1	-0.65378423	0.02068012
MRPS10	-0.65341587	1.3649E-05
MYO9A	-0.65316341	0.02203976
HSPB1	-0.65306686	0.00057156
PMAIP1	-0.65292944	0.00480503
MEMO1	-0.65216631	0.04465748
ORAI3	-0.65187995	0.00055309
TTF1	-0.65094601	0.0404266
C3orf38	-0.65087037	0.00105517
GBA	-0.65079017	0.02333546
RFC1	-0.65073789	0.0008414

SMAD4	-0.65052523	0.00549033
MRRF	-0.65020805	0.00583699
SORL1	-0.64970173	0.00083434
RCOR3	-0.64957281	0.0031004
LRP1	-0.64940664	1.4379E-05
KIF20B	-0.64868409	0.00273806
HSBP1L1	-0.64819281	0.0164683
NAGLU	-0.64708266	0.03168269
C6orf120	-0.64502278	0.00696526
ACVRL1	-0.64453855	0.01140301
C16orf58	-0.64381099	0.03150937
DPP9	-0.64376701	0.01015241
TCEAL1	-0.643474	0.01861766
BRWD3	-0.64337039	0.00915237
NPRL2	-0.64254251	0.02877603
UTRN	-0.64240819	5.8747E-08
CENPT	-0.64238177	0.03807237
MOB1B	-0.64226798	0.00128105
XPC	-0.64206052	0.0038703
LIMA1	-0.6411308	0.00565156
NFATC2	-0.64108244	0.00371151
MDM4	-0.64083995	7.6723E-07
TRNT1	-0.63968343	0.02352036
G2E3	-0.63966655	0.0113218
IFI16	-0.63958557	2.3237E-11
PARP8	-0.63926929	0.00497201
GOLGA8A	-0.63906279	0.03559778
ARL3	-0.6385202	0.00018431
GPATCH4	-0.63835947	0.00488941
TRAPPC8	-0.63770631	0.01226032
CD68	-0.63704215	7.1389E-06
TMEM9	-0.63667562	0.00371116
PMVK	-0.63635302	1.265E-09
ZNF445	-0.6362006	0.02469142
MBD4	-0.63585266	0.00095953
UTP6	-0.63571311	5.3816E-05
RHNO1	-0.63537447	0.0299154
C3orf62	-0.63367633	0.02189677
STAMBP	-0.63299554	0.00047697
NXPE3	-0.63277188	0.02069092
ZNF160	-0.63026605	0.03327101
DPP8	-0.62964066	0.00155223
ZNF106	-0.62947409	8.6908E-09
BLCAP	-0.62916007	0.00972533
BAZ1B	-0.62881828	0.00147859
SMIM7	-0.62824697	7.428E-06
PLXND1	-0.62780239	0.01626207
ZBTB4	-0.62730012	0.02484637
DIP2B	-0.62726847	0.01242111
C20orf194	-0.62646484	0.04362621

SMIM15	-0.6262957	0.00367353
ZDHHC17	-0.62617221	0.01998216
CRYBG3	-0.62533807	0.01529412
GGA2	-0.62492123	8.5605E-05
EXOC6	-0.62475542	0.00707771
GADD45B	-0.62470677	0.00073706
MRPS18B	-0.62218599	0.000293
PINK1	-0.6218724	0.0153717
GAA	-0.61963124	1.4418E-05
GDAP2	-0.6189727	0.00340992
YWHAH	-0.61786374	0.00030586
SP1	-0.61724901	0.03719834
BMPR2	-0.61695605	0.00984962
CHMP6	-0.6168119	0.01794861
RNF214	-0.61644826	0.03197376
SREK1IP1	-0.6148607	5.4507E-05
ATF7IP	-0.61459844	0.01566953
WDR11	-0.61415988	0.00045857
TGFBI	-0.61390135	2.2169E-07
EHMT2	-0.61386578	0.04095282
RAB14	-0.61367394	1.9764E-07
IFT74	-0.61133276	0.04172198
SASH3	-0.61042005	0.01760958
TFEB	-0.6097494	0.00069796
NCKAP5L	-0.60913721	0.03071301
CSNK1G3	-0.6084242	0.00976713
MFNG	-0.60685182	3.4397E-05
CTSB	-0.60635123	7.2528E-09
ACSF3	-0.60485057	0.01287689
NUP50	-0.60387573	0.01357894
PHKB	-0.60339788	0.00148988
SEC22B	-0.60242703	0.00254364
RCSD1	-0.60223977	4.7551E-10
RYK	-0.60201361	0.0093687
HEATR6	-0.60140816	0.03307479
CNTRL	-0.60110335	0.00028775
MPEG1	-0.59939413	7.8204E-16
TNRC6A	-0.59939335	0.0055628
NSF	-0.59910356	0.00695245
PANK2	-0.59895984	7.253E-05
CDC16	-0.5986555	0.02484637
SULT1A1	-0.59785468	0.00407962
REPIN1	-0.59623929	0.00779886
PUM1	-0.59568689	0.00018431
RAB11FIP2	-0.59566343	0.02781598
RAB3GAP2	-0.59484804	0.03054434
TRIM52	-0.59472942	0.0287372
JADE1	-0.59450595	0.03920307
CEP170	-0.59437224	2.0042E-07
DCP2	-0.59429496	7.3379E-07

ELP6	-0.59420045	0.01503061
WDFY2	-0.59412524	0.00204932
CREB3L2	-0.59273693	0.00309204
PEX13	-0.59256792	0.04293931
C4orf27	-0.5924772	0.01258468
TUBGCP3	-0.59237032	0.04326539
LIMK1	-0.5917414	0.01005196
ZNF710	-0.59155395	0.04697179
ANKRD49	-0.59127944	0.01730501
ERLIN2	-0.59108717	0.02750878
CAMK2D	-0.59093017	6.3826E-05
MEF2C	-0.5897835	0.00012301
MRPL1	-0.58935488	0.01852232
KIFAP3	-0.58931528	0.04475518
FKBP15	-0.58908934	0.00437509
MED23	-0.58810585	0.04357229
C1orf54	-0.58713468	5.2976E-05
MARVELD1	-0.58650876	0.01362631
LENG1	-0.58646113	0.00487872
EXOC1	-0.58607718	0.00453081
ZNF91	-0.58601938	0.00695245
FASTKD1	-0.58587481	0.03154816
KIAA2026	-0.58515198	0.00198572
TOMM70A	-0.58503007	0.01616743
ZMAT3	-0.58410496	0.04301928
DHRS3	-0.58383494	0.00064463
SPATS2	-0.58161313	0.01916349
MRPS5	-0.58136865	3.8548E-05
MRPL17	-0.58126455	0.01438589
NECAP2	-0.58091953	1.8159E-05
NUDT3	-0.58052731	1.289E-10
SMARCD2	-0.58042266	0.00315074
MORC3	-0.57974668	0.00162278
EXOSC9	-0.57972887	0.01716852
DPM2	-0.5792395	0.00176689
PLEKHO1	-0.57830896	0.00083942
AGO1	-0.57760765	0.0254748
MAPK14	-0.57752711	0.01171019
LAMP1	-0.57741368	0.00075605
SRPRB	-0.57718462	0.00167251
PIGF	-0.5767591	0.00222176
ELMOD2	-0.57647812	0.01672373
SRSF1	-0.57642478	0.00065516
CCDC137	-0.57637944	0.03399034
CEP95	-0.57612222	0.03393822
ZNF431	-0.57524606	0.01423424
MPZL1	-0.57503027	0.00922041
PECAM1	-0.57448261	3.2026E-05
AKIP1	-0.57420171	0.00269116
C9orf85	-0.57334737	0.03795453

CFD	-0.57230494	0.00991454
ANKRD26	-0.57228604	0.01764001
RUBCN	-0.57223927	0.01744025
HNRNPLL	-0.57146621	0.02515035
RIOK2	-0.57021409	0.02836215
PRKAG1	-0.56991384	0.00026492
MCM3AP	-0.56961174	0.03076802
GGPS1	-0.56946426	0.0041007
XPO1	-0.56891115	1.2546E-05
FAM72A	-0.56890424	0.04582132
ITPRIPL2	-0.56780312	0.01196677
ARID4A	-0.56640408	8.2457E-05
YPEL2	-0.56605356	0.00265461
SIGLEC10	-0.56538219	6.0617E-06
SLC8A1	-0.56470051	2.9006E-07
MALSU1	-0.56468274	0.01833097
SUGP2	-0.56462566	0.00125041
ZCCHC9	-0.56457716	0.007317
FRMD4B	-0.56319897	9.36E-06
EMC9	-0.56307707	0.04611777
SUZ12	-0.56274408	0.00032888
APOOL	-0.56177008	0.03893269
MILR1	-0.56131287	0.00077725
MRPS18C	-0.56070811	2.4974E-05
CELF1	-0.55991251	0.01413833
PIAS1	-0.55950054	9.4104E-05
FAR1	-0.55842994	0.00017818
METAP1	-0.55839381	0.01566669
APPBP2	-0.55688085	0.03066392
TMX3	-0.55685124	0.00044792
SCAF11	-0.55622066	7.196E-09
FHOD1	-0.55608446	0.02714399
HNRNPUL2	-0.5558892	0.00231693
JKAMP	-0.55450231	0.00362772
PDPR	-0.55399439	0.03852325
PKN2	-0.55384518	0.00078796
NXF1	-0.55276514	0.00314565
SNX29	-0.55275188	0.00315898
RAD50	-0.5527512	0.00037915
DNAJB14	-0.55052788	0.0005181
FAM91A1	-0.55010201	0.00267657
NKTR	-0.54987478	2.4305E-07
ARMCX3	-0.54963843	0.00697794
CPSF2	-0.54901095	0.01938401
LRMP	-0.54680055	0.00499839
TRMT2A	-0.54644218	0.01906048
CTA-384D8.34	-0.54640281	0.04144719
CARM1	-0.5460252	0.03393822
MYLIP	-0.54590929	0.02077147
PPP1R9B	-0.54571959	0.02197078

PPWD1	-0.54564513	0.04088279
ITSN1	-0.54508043	0.0251321
TARBP1	-0.54429861	0.01760578
GTF2A1	-0.543846	0.0223238
CBLL1	-0.54175004	0.03630193
TLK2	-0.54173551	0.01185982
TATDN3	-0.54138474	0.03526107
CD81	-0.54037507	0.00659613
RABGAP1	-0.54023155	0.02811191
RAP2A	-0.54011323	0.01610629
IPO9	-0.53990234	0.04842878
STX16	-0.53990191	0.00216119
CDC40	-0.53976813	0.00047835
SETD5	-0.53972798	0.02256995
RBM26	-0.53971808	0.00024058
RGS14	-0.53830935	0.033825
ATP5SL	-0.53688177	0.01614752
HACD2	-0.53673625	0.0098426
AP1B1	-0.5361674	0.00017897
MRPS26	-0.53587732	0.02798178
PLXNC1	-0.53546966	0.01160351
DAPK1	-0.53543193	0.00149119
YBEY	-0.53492775	0.01566953
CD36	-0.53463063	0.0002403
RFNG	-0.53382807	0.01476104
CHM	-0.53222529	0.03819112
TK2	-0.53187833	0.00900142
TMEM242	-0.53125424	0.01115432
COG2	-0.53090735	0.04086477
MPZL2	-0.5306803	0.01140817
SAE1	-0.53027656	0.01285433
CCDC186	-0.52896453	0.01532771
RNPC3	-0.52826277	0.00635432
SPIN1	-0.52752604	0.00746076
OSCAR	-0.52698101	0.00575553
ZMYND11	-0.52688762	0.04136703
PLXNB2	-0.5268731	0.01248504
AGO3	-0.52624279	0.00679986
TM2D2	-0.52480938	0.00346749
SDCCAG8	-0.52391526	0.00039432
DENND4B	-0.52379446	0.03748617
RP11-108M9.4	-0.52319533	0.00197231
DNAJC13	-0.52312426	0.01098742
LINC00998	-0.52282031	3.5771E-05
HNMT	-0.52249621	1.6796E-06
ABHD3	-0.52025583	0.0193333
DHX29	-0.51971049	0.00373406
ZC3H13	-0.51912117	0.00014242
NCKAP1L	-0.5178998	1.1233E-08
ALDH3B1	-0.51787885	0.01185246

SELM	-0.51609158	0.01619227
NPEPPS	-0.51576189	0.00136217
NFIC	-0.51549541	0.0041911
MSL1	-0.51474325	0.02743265
STK38	-0.5146513	0.028464
ZCCHC7	-0.51390854	0.00929139
CLEC12A	-0.51346874	0.01956639
ALDH9A1	-0.51342767	0.02781214
ARID1A	-0.5124621	0.00056052
MEGF9	-0.51195273	0.04088279
TAF3	-0.51184928	0.02063651
PCF11	-0.5109588	0.00021674
CNOT4	-0.51086889	0.00319605
GALNT7	-0.51056829	0.033825
MAX	-0.51005366	7.2302E-05
STRN	-0.50942337	0.03985238
ARRDC2	-0.50909293	0.02401011
DCAF8	-0.50894686	0.0375317
COMMD3	-0.50827596	0.00028107
GABPB1-AS1	-0.50801638	0.01627673
KIAA1033	-0.50660958	0.00176647
POM121C	-0.5064192	0.01494115
ZRANB2	-0.50582849	9.5579E-05
KLHL24	-0.50499757	0.00453812
APOL3	-0.50492508	0.00724352
PLCB2	-0.50487245	0.00468112
PDZD11	-0.50479002	0.03521913
SNX30	-0.50471041	0.03221554
RP11-488L18.	-0.50446232	0.04029668
TPM1	-0.50401753	0.03059026
CCNL2	-0.50335182	0.00576525
ADAM9	-0.50244504	0.01984312
SDAD1	-0.50204928	0.00912983
TMEM107	-0.5015229	0.00583632
ARHGAP9	-0.5011582	0.00270922
TOR1AIP1	-0.50054613	0.00158427
MED1	-0.50052956	0.03708699
FHL3	-0.50015819	0.03703882
TNRC6B	-0.49965343	0.00084135
SNRNP48	-0.49904325	0.03782278
C14orf119	-0.49885253	0.00194211
AAK1	-0.49862762	0.00771786
ZBTB11	-0.4979566	0.0302476
ANKRD36C	-0.49771996	0.03511007
COPS7A	-0.49698641	0.01282578
MAPK3	-0.49595092	0.0105991
SCRN1	-0.49441831	0.04323907
PYCR2	-0.49409731	0.02617525
ATP11C	-0.49384792	0.04801713
ANKRD10	-0.49352654	0.00654486

LGALS8	-0.49236512	0.0007152
ACOT13	-0.49209354	0.01341917
TSPAN14	-0.49150109	0.00226717
POGZ	-0.4914889	0.02654214
ZNF770	-0.49145391	0.01657622
TMED9	-0.48981684	2.8879E-05
C6orf62	-0.48956308	0.00023089
MTIF3	-0.48880096	6.6879E-05
PRKCA	-0.48876599	0.04633876
SMC3	-0.48850186	0.00702594
RPP25L	-0.48791394	0.01995556
ANKRD17	-0.48766025	0.0133537
RPAP3	-0.48503298	0.02732808
SCOC	-0.4849245	0.02158485
YAF2	-0.484326	0.00084545
FNDC3A	-0.48415045	0.02530681
PUM2	-0.4839797	0.02045914
AHR	-0.48348315	2.5484E-05
PDGFB	-0.48344855	0.04517582
FEZ2	-0.4827843	0.00016252
SNX6	-0.48274769	0.000412
ASGR1	-0.48239515	0.0147363
STARD7	-0.48238468	0.01015701
CWC22	-0.48163425	0.01807669
YLPM1	-0.48159181	0.02257202
TPCN1	-0.48125876	0.04261031
SDSL	-0.48100231	0.0010158
TMEM173	-0.48097648	0.01141577
MYCL	-0.4802674	0.01978938
LARP7	-0.48003825	0.00013938
CISD1	-0.4797531	0.00455049
GSAP	-0.47916085	0.03051576
GPALPP1	-0.47895998	0.0368424
WDR77	-0.47787022	0.02627669
EIF4A2	-0.47783423	4.7057E-06
BRD3	-0.47724452	0.03580057
PTGS1	-0.47718517	0.01695635
UTP18	-0.47659793	0.00690235
BLVRA	-0.47494289	0.00040007
FKBP4	-0.47465498	0.03847198
C19orf25	-0.47461574	0.04622194
ARHGAP18	-0.47459864	0.00254636
NRBF2	-0.47406818	0.00295356
EML4	-0.47351119	0.00194211
NIFK	-0.47248984	0.0002037
YIPF4	-0.47181643	0.00197203
KIAA0141	-0.47091847	0.03196375
VMP1	-0.47046934	0.00274379
CHTF8	-0.47038618	0.02867675
DCTPP1	-0.4702918	0.00261525

MAP3K11	-0.47019361	0.00417483
NUDT16L1	-0.47003279	0.02227611
SLC35A4	-0.4692747	0.03458435
C11orf57	-0.46747132	0.04475518
PRKD3	-0.46687655	0.01548672
ZBTB38	-0.4665634	0.02386533
SLMAP	-0.46644422	0.04672584
UBLCP1	-0.46597524	0.01058365
TYK2	-0.46457643	0.01892913
BAZ2B	-0.46370814	0.00704932
TMEM30A	-0.46216998	0.0124763
ACBD5	-0.46200026	0.04697179
SMIM20	-0.46043486	0.00046836
ZNF524	-0.46030592	0.02068012
KIDINS220	-0.4599682	0.00204693
HEXDC	-0.45783246	0.01387015
STX6	-0.45736865	0.00247748
PLRG1	-0.456639	0.0257765
RAB13	-0.45636613	0.00202145
KIAA0930	-0.45632388	0.00673299
TBC1D22A	-0.45576689	5.6443E-05
CEBPZOS	-0.45506348	0.00646749
HSPBAP1	-0.45480905	0.0375317
UBE2W	-0.45442335	0.00597534
RNF213	-0.45432667	0.00032888
VSIG4	-0.45409552	0.00074245
TPST2	-0.45377957	0.04495723
CCPG1	-0.45285062	0.0311287
SP140L	-0.45258049	0.03074682
USP53	-0.45229061	0.01375259
SIRT7	-0.45227997	0.0175239
GTF2H1	-0.45194044	0.0477053
NUB1	-0.45083427	0.00280401
BROX	-0.45080562	0.00754334
FRYL	-0.4503787	0.01371694
TAOK1	-0.45037496	0.02025107
ACAP2	-0.45032319	0.00812023
USP1	-0.44976911	0.02975497
BRWD1	-0.44908795	0.00950433
DYNC1LI2	-0.44831563	0.0108624
UQCC3	-0.44754858	0.02227611
MAGOHB	-0.44475078	0.02509626
CISD3	-0.44462193	0.00425145
PPIP5K2	-0.44447311	0.0095379
ELK4	-0.44435105	0.00940085
PLEKHJ1	-0.44421574	0.00332036
DOCK11	-0.4442101	0.0166586
TRIM38	-0.44382029	0.00058669
CEP63	-0.44376042	0.02069092
TBL1XR1	-0.44364943	0.00750768

COA3	-0.44351346	0.0033364
PEX2	-0.44306117	0.01658998
SETX	-0.44259041	0.00091481
TSNAX	-0.44196952	0.03189743
TMEM126B	-0.44049777	0.02036253
FBXL5	-0.43999448	0.00438457
FARSA	-0.43994917	0.02206223
LPP	-0.43849947	0.00077871
PLA2G16	-0.43841011	0.00778211
SNX13	-0.43836079	0.03987727
DR1	-0.43813753	0.02045214
IL6ST	-0.43639094	0.0207936
SEC23IP	-0.43633772	0.04106723
SCIMP	-0.43606596	0.00744097
CYSLTR1	-0.4346814	0.01965915
BIN2	-0.43445191	0.01127923
TMEM251	-0.43309606	0.02679807
LSM10	-0.43300447	0.00035956
XPA	-0.43250304	0.01169043
PPCS	-0.43228729	4.9466E-05
SLC25A36	-0.43219048	0.00906456
CRYL1	-0.43083188	0.03231906
PPP1R18	-0.42975567	0.0002632
PSMB8-AS1	-0.42957569	0.03521913
TCEAL8	-0.42898398	0.00237518
STAC3	-0.42799308	0.01884013
SRFBP1	-0.42764503	0.01151419
PAK1IP1	-0.42756292	0.03777176
GTF2I	-0.42728046	0.00484732
METTL23	-0.42597706	0.00567331
HLA-DOA	-0.42576329	7.8606E-06
EMG1	-0.42461462	0.01747697
FAM111A	-0.42414694	0.04452894
AGPAT5	-0.42382491	0.04998185
KMT2A	-0.42250899	0.01134168
SIPA1	-0.42096775	0.00332983
NUDT16	-0.42043519	0.00321854
PIK3R1	-0.41986363	0.00126834
SYK	-0.41936839	0.00242907
REST	-0.41885145	0.0035227
MRPL34	-0.41744328	0.00239288
LRRC8C	-0.41605956	0.0343151
SPG11	-0.41579663	0.04388139
EXOSC8	-0.41574037	0.03025523
ST3GAL6	-0.41474587	0.04492055
STK25	-0.41186887	0.04820878
CCDC28A	-0.40985999	0.02031423
TFPT	-0.40984325	0.00690235
EEA1	-0.40732238	0.02089318
TSPAN33	-0.40681821	0.00656124

CIITA	-0.4052593	0.02625786
CUL5	-0.4018642	0.00733739
EVA1B	-0.40079205	0.00116226
NKAP	-0.40044265	0.02005706
RABEP1	-0.40043298	0.03961127
TNFRSF1A	-0.39907466	0.02359825
CREB1	-0.3979419	0.01765054
FTL	-0.39660916	0.00014132
UNC93B1	-0.396436	0.00337567
RBMX2	-0.39631429	0.02595279
MAT2B	-0.39615475	0.00726062
GRSF1	-0.39575666	0.01848121
CLK1	-0.39352913	0.03532716
CMC1	-0.39293228	0.03033878
URM1	-0.39196043	0.03011681
ARHGAP30	-0.39126635	0.004253
TIGAR	-0.39040905	0.02375095
N4BP2L2	-0.39016446	4.4707E-05
IVD	-0.38848477	0.02495441
ZFHX3	-0.38817195	0.00816142
U2AF1	-0.38815645	0.02753649
TMUB2	-0.38616273	0.02616219
ERICH1	-0.38613512	0.00788385
KLHDC3	-0.38409997	0.02299492
MRPS28	-0.38295331	0.02636782
MYO1F	-0.38168456	0.00067823
ITM2B	-0.38031705	0.00158233
MRC1	-0.37873361	7.9243E-06
CDK4	-0.37785364	0.01800286
SLC7A7	-0.37781716	0.04141972
EBLN3	-0.37491888	0.03171666
GNB4	-0.37472099	0.02751704
PAXBP1	-0.37329666	0.03731485
RPA2	-0.37141739	0.01978938
EMC10	-0.36960331	0.00826381
KPNB1	-0.36755323	0.00082268
MLXIP	-0.36711096	0.02224462
GNS	-0.36644621	0.02365799
SUMF2	-0.36606644	0.01431909
MARCH7	-0.36448657	0.03329828
PTTG1IP	-0.3641761	0.00766452
NAP1L4	-0.36304362	0.02729217
LINC00116	-0.36273353	0.02165032
TIMM9	-0.36221264	0.03663214
TCEAL4	-0.36202535	0.00053578
CD4	-0.36202087	2.7358E-05
RASSF4	-0.35934121	0.00652573
STX7	-0.35893091	0.00206323
HEXA	-0.35768342	0.00041437
RGS2	-0.35731437	0.00298657

PRKACB	-0.3563594	0.03168269
PPIG	-0.35525857	1.1394E-05
UPF3A	-0.35320839	0.01822582
UHMK1	-0.35098522	0.01040974
TPP1	-0.35069	1.4322E-05
WSB1	-0.35068878	0.00390065
TRIM44	-0.35061055	0.01822582
CLTC	-0.34996384	0.0191206
FYB	-0.34909607	0.00485895
LPCAT2	-0.34800479	0.01395159
RSRC2	-0.3466857	0.00256682
PID1	-0.34650556	0.00673691
PCM1	-0.34636198	0.02054771
PHACTR2	-0.34136902	0.04715894
PSMB5	-0.34123555	0.00918757
USO1	-0.34069931	0.02912788
MSL3	-0.33934643	0.03773872
CIR1	-0.33807053	0.01408649
CEP350	-0.336447	0.00506014
TXN2	-0.33608474	0.01311188
AMD1	-0.3353516	0.00770556
MRPS34	-0.33507709	0.00334628
ETNK1	-0.33409652	0.04020317
SPOPL	-0.33358929	0.02962344
TNPO1	-0.33305138	0.0336633
YME1L1	-0.3316763	0.04280411
POP5	-0.32774348	0.04624748
NUCB1	-0.32612478	0.00640869
CAMK1	-0.32469451	0.00446694
SNX5	-0.32385178	0.00815234
ITGB7	-0.32321837	0.04301634
MRPS7	-0.32221379	0.01458465
MED8	-0.32158713	0.04301634
PDLIM2	-0.32121871	0.04874389
COMMD9	-0.32114197	0.02470282
MRPL16	-0.32069762	0.04907798
PCMTD1	-0.31853927	0.04687675
OAZ2	-0.31714799	0.01096978
RPL7L1	-0.31497386	0.04250583
CD93	-0.31303912	0.01463573
CCDC115	-0.31114128	0.02313841
BST2	-0.30974516	0.00043998
FAM192A	-0.30966423	0.04029668
NPC2	-0.30736208	0.00049798
EVL	-0.30616922	0.01701918
MS4A6A	-0.30605893	0.00060603
FAM195B	-0.3058773	0.04357229
CD1D	-0.30267562	0.0109352
ARPC4	-0.2989058	0.00351244
MRPS16	-0.29872526	0.04495723

CTSA	-0.29828782	0.03855995
HEBP1	-0.2968449	0.04707397
HLA-E	-0.29230692	0.01931117
FGL2	-0.29151172	0.00411507
TIMP2	-0.29128953	0.03148922
DCAF7	-0.29044491	0.03565223
RSF1	-0.28999273	0.04497372
SOAT1	-0.28810049	0.03978308
MBNL1	-0.28675813	0.01831171
SFT2D2	-0.2864207	0.02701194
PRMT2	-0.28251511	0.02970408
CREBL2	-0.2707502	0.01923095
WASF2	-0.26544062	0.01741955
ITGA4	-0.26347263	0.02779629
SNAP23	-0.25733196	0.0236601
CNDP2	-0.25258266	0.00964295
PRPF4B	-0.25161753	0.0113943
LAMTOR1	-0.24545815	0.03247137
BOD1L1	-0.24209767	0.02074116
VAMP8	-0.24181791	0.00019248
NIPBL	-0.236444	0.04315368
RPL22L1	-0.23407482	0.03016715
UBE2L6	-0.23320474	0.02237973
RGS10	-0.22967193	0.0086329
C10orf54	-0.22514447	0.00362772
FCGRT	-0.2150993	0.00529821
AIF1	-0.21261469	0.00187076
DYNLL1	-0.20280583	0.04656373
CD302	-0.20208588	0.02279355
CAPZB	-0.17296997	0.01521476
HMGN2	-0.16690938	0.04206714

cDC3 vs M7					
**cDC3 DEGs**	**avg_logFC**	**adj. p-value**	**M7 DEGs**	**avg_logFC**	**adj. p-value**
CD2	4.96518946	1.6986E-12	PLEKHG5	-6.20551737	2.0712E-19
CXCL11	4.78849733	1.0031E-14	MAMDC2	-5.7551685	5.7356E-14
UPK3A	4.56674695	1.3089E-16	MYO7A	-5.46266836	2.8341E-14
SYTL1	4.49050971	6.588E-12	GAL3ST4	-4.98586172	3.9241E-34
CES1	4.44371823	8.2521E-08	PCDH12	-4.90209675	2.246E-14
CD1C	4.31896196	6.1304E-20	FOLR2	-4.78525323	2.0857E-68
MPP7	4.22740727	7.6305E-10	CYTL1	-4.74459741	4.3593E-18
FCN1	4.14121598	1.3621E-13	BCAM	-4.71343095	4.3816E-32
HSH2D	4.08453699	8.1319E-19	LYVE1	-4.6685564	1.3643E-33
NRGN	4.05584501	3.3973E-10	IGSF21	-4.66040124	8.3926E-15
CXCL9	4.01905089	1.4295E-18	PLXDC1	-4.61564727	4.2208E-34
APOBEC3A	4.01846947	1.1524E-12	CD163L1	-4.48382606	2.7867E-47
S100A12	4.01195145	1.082E-07	CCL24	-4.45546833	1.497E-19
TSPAN32	3.9591901	6.6415E-10	LILRB5	-4.44158385	4.6822E-29
FYN	3.82438755	6.4198E-23	TNS1	-4.36460859	2.4658E-18
CD52	3.7496068	3.7489E-23	TDRD10	-4.30093828	2.7466E-11
VCAN	3.68237965	3.5823E-21	CRYBB1	-4.2213881	7.7509E-09
CD207	3.64249036	1.4063E-07	RNASE1	-4.21475801	1.1531E-13
CD1E	3.63251625	5.8436E-11	LINC00891	-4.16621695	4.9499E-09
PFKP	3.61739102	5.8868E-15	LTBP2	-4.15728744	2.5232E-08
FAM65B	3.53568691	2.2543E-08	SIGLEC8	-4.09927513	1.8147E-15
IDO1	3.49518372	8.9398E-27	RP11-733O18.	-4.09745758	5.9185E-05
LIPN	3.47245354	0.00020794	SEPP1	-4.09065016	1.1317E-28
ADAM8	3.45696427	3.7907E-36	TMEM37	-4.05311328	6.0557E-30
PLAC8	3.44959854	3.3194E-29	ABCG2	-4.05192968	2.3825E-28
SLC38A1	3.44124443	1.06E-20	EDA	-4.0436655	1.7044E-11
CTD-2319I12.:	3.43726461	7.5057E-07	MMP2	-4.01703841	2.3666E-07
MTMR11	3.38831374	4.1002E-10	ETV1	-4.00856794	2.2598E-05
TMEM8B	3.348527	7.5168E-08	RAB3IL1	-4.00132005	3.4163E-23
UBD	3.31773978	3.9016E-07	MERTK	-3.9492843	8.2127E-50
ENHO	3.29598932	1.0056E-09	SCAMP5	-3.94334853	3.2121E-07
ARNTL2	3.2801177	3.0392E-05	SMARCA1	-3.94331528	3.1116E-05
ICAM3	3.26757826	7.213E-28	TNFRSF11A	-3.93717121	1.5707E-14
HACD1	3.25815166	7.319E-06	GSDMA	-3.92799644	4.2191E-10
CD1B	3.22877559	3.1495E-10	SHE	-3.9267026	9.5557E-19
QPCT	3.21453515	5.2854E-25	DIP2C	-3.89796603	4.5333E-06
TMEM154	3.21143306	1.7946E-19	NRP1	-3.895634	1.0252E-43
C16orf74	3.20684	0.00012256	TDRD1	-3.87191486	2.4038E-08
CCR6	3.12925905	4.4077E-08	CXCL12	-3.82880371	3.7107E-31
CRLF2	3.11957413	3.3264E-06	CPAMD8	-3.82580087	4.9392E-06
FLT3	3.11180913	5.1297E-14	WLS	-3.80649823	1.0673E-28
TRNP1	3.10329519	0.001604	RHPN2	-3.79959214	0.00039934
CCR7	3.09139305	2.7444E-06	BNC2	-3.77482389	9.6758E-18

GPR18	3.07541035	2.9488E-06	CCL14	-3.74488107	0.00241169
ADORA2B	3.05964641	4.9734E-08	ZNF812P	-3.71722266	9.1733E-08
IL18R1	3.05362907	1.867E-10	SLC7A8	-3.70257548	5.4308E-34
ABHD17C	3.02760319	7.5568E-08	ADAMTS12	-3.69754122	5.5395E-07
PARM1	3.00729419	4.7596E-06	TMEM26	-3.69280097	2.2199E-07
HOMER2	2.98814837	2.4496E-07	HECTD2	-3.59307932	3.9376E-21
FAM134B	2.97780519	1.6914E-05	LINC01133	-3.59125974	0.00170054
CXCR3	2.97770291	1.5752E-07	PDK4	-3.57806604	2.2662E-41
GNG4	2.97764962	0.00687197	BACE2	-3.56084555	2.187E-05
CD1A	2.94896727	1.257E-06	CABP4	-3.55346763	1.6589E-07
CST7	2.90904839	1.0627E-22	NYNRIN	-3.55231011	7.4228E-07
IDO2	2.86381434	2.353E-05	TRIM47	-3.54690705	2.3087E-10
PADI2	2.85780772	3.1814E-07	F2RL3	-3.5424116	8.9737E-10
LA16c-444G7.	2.83105713	9.5674E-06	MAF	-3.54193094	4.6636E-41
SLC22A4	2.79402546	2.4872E-07	RGMB	-3.54188069	6.8268E-05
PPP1R16B	2.77051802	0.00038599	TNFRSF25	-3.53658355	6.203E-09
MGST1	2.76184694	2.2375E-09	BBS5	-3.53601631	2.8187E-06
SH3BP4	2.73507064	1.2092E-13	VCAM1	-3.53568153	3.1132E-21
FCER1A	2.72431163	4.1521E-08	SLCO2B1	-3.52788751	4.0994E-34
SLAMF9	2.71355155	6.2006E-16	PLTP	-3.51991145	2.8767E-73
SULT1B1	2.67269085	0.00018413	F13A1	-3.49379121	2.4603E-32
NEGR1	2.64034476	2.961E-06	CCL3	-3.47604375	2.8882E-52
TRH	2.6245137	0.00690896	CCL4	-3.44756552	4.1034E-63
LAYN	2.62257057	0.00165364	FAM177B	-3.44610501	4.8403E-05
KBTBD11	2.61759119	1.3523E-05	LINC00996	-3.43796958	6.9482E-30
ANGPT1	2.61336677	0.00313439	EGFL7	-3.42983357	6.678E-07
ETV7	2.56304506	3.8706E-06	SEZ6L2	-3.42909512	2.7973E-06
BCL11A	2.55711383	5.0309E-13	STK32B	-3.40742877	0.00082992
ATP2A3	2.54230881	8.1059E-05	ME1	-3.40034623	4.3221E-26
TFPI	2.53781041	9.2577E-07	TNIK	-3.3977328	3.1448E-13
CTD-2006K23.	2.51898858	2.9478E-09	WFS1	-3.38846254	5.7173E-09
IL7R	2.51487896	0.00254024	CCL18	-3.37295003	7.9237E-26
TNNT1	2.50601974	0.00313733	SNCAIP	-3.35042729	0.0058163
VDR	2.49534234	2.5869E-21	DMKN	-3.34442173	0.03911434
APOBEC3H	2.48369116	0.00734697	TRPV4	-3.34178878	9.348E-05
SPIB	2.48130314	2.2E-16	ADGRG6	-3.33709083	9.8169E-21
PLCB1	2.47852731	0.00071262	FRMD4A	-3.32941071	2.2499E-25
AUNIP	2.47681112	0.00022294	DAB2	-3.3227665	5.0948E-35
FGR	2.47407959	2.3562E-14	HSD17B14	-3.3126719	6.1179E-17
ACAP1	2.46769264	6.4718E-13	SLC12A5	-3.29972223	9.677E-16
GPR157	2.46615169	7.2391E-14	LINC01356	-3.29390793	2.9845E-06
IL1R2	2.46195989	1.3268E-13	ZG16	-3.29188642	0.03565881
BASP1	2.44525977	1.3394E-06	SMPDL3A	-3.28119785	4.5553E-23
LMNB1	2.44336819	1.6553E-16	ITGA9	-3.28098776	1.5655E-21
AC079630.4	2.44307486	0.00090515	CNRIP1	-3.27270383	5.7255E-29
SELL	2.43436012	2.0118E-18	CCDC36	-3.2715393	0.00030262

S100A6	2.42650302	2.3952E-13	CYP26B1	-3.27117192	0.0025747
CFP	2.42580716	1.2465E-25	IGFBP4	-3.26854204	2.4967E-35
RAMP1	2.42135842	6.5754E-07	ZNF133	-3.26846928	3.3745E-05
TYMS	2.41310211	1.5645E-05	RP11-489O18.	-3.25126866	4.3136E-19
S100A8	2.41129866	4.5677E-05	ROR2	-3.23921802	3.7632E-05
ACOX2	2.38594181	0.00792281	PTPRM	-3.23906748	3.6652E-08
GALNT3	2.37299805	2.0159E-09	LPAR5	-3.22633354	5.7433E-07
TUBA4A	2.35265475	2.797E-14	GAREM1	-3.22212567	3.1764E-16
TRABD2A	2.34904451	2.6878E-11	PLS1	-3.21940646	3.8448E-05
CHST2	2.34151504	1.7496E-12	GAPLINC	-3.2046956	0.00016231
RAB11FIP4	2.33767862	9.9275E-05	EGR1	-3.20247969	1.0868E-35
ELFN1-AS1	2.33199212	0.02925655	SLC41A1	-3.18909439	5.8343E-08
TNFSF14	2.32440548	4.3769E-06	EPHB2	-3.18774424	1.2273E-21
WEE1	2.32155949	1.69E-07	IKZF2	-3.18688928	0.00325261
C11orf21	2.31669548	0.00516586	FABP1	-3.18462767	1.6308E-09
MYO5C	2.31388294	0.00418873	YES1	-3.17719836	3.0131E-07
TMEM217	2.29159182	4.402E-06	FAM174B	-3.17206486	2.9245E-07
GPER1	2.2905823	4.6775E-08	TRAPPC3L	-3.16857627	0.0041942
MCEMP1	2.28892354	4.7307E-12	AC069363.1	-3.16727504	3.9876E-11
SLC9B2	2.27927925	0.00034746	EML1	-3.1384776	1.7729E-05
LAMP3	2.27540135	1.4912E-07	APOE	-3.13502055	5.1777E-08
ST18	2.2536502	2.1495E-05	RP3-522D1.1	-3.12724204	1.5194E-08
TOP1MT	2.25027478	4.7868E-13	OLFML2B	-3.12222723	3.9868E-19
C12orf42	2.24204534	0.00346087	JUN	-3.11480096	6.1823E-36
KIF23	2.2306491	0.00152334	NR3C2	-3.08598564	0.00467524
CD244	2.22879961	2.4848E-08	HOXB7	-3.08224421	0.00692657
LILRA2	2.21094439	8.8506E-10	FAM198A	-3.06855845	1.3658E-06
SLC46A2	2.20785791	0.00146717	GPNMB	-3.06601472	2.6013E-23
HPGD	2.20776016	0.00094873	NEK10	-3.06530218	0.00872957
LINC01262	2.20613664	0.04653251	IGF1	-3.0648545	1.0512E-32
LSP1	2.20326114	1.507E-15	LAG3	-3.05115914	4.2481E-19
ANPEP	2.19754761	3.1532E-10	TFAP2C	-3.05063406	0.02746261
SMAD3	2.19353434	1.197E-06	HOMER3	-3.04735738	1.1133E-30
FRY-AS1	2.19220667	0.00521839	MTUS1	-3.0463501	1.1872E-11
BEND5	2.18938374	0.02559986	CRHBP	-3.04325173	1.7499E-21
RGL4	2.18660654	0.00966565	C2	-3.03840118	4.5614E-18
NCS1	2.17991034	0.01614319	KANK2	-3.03486421	4.7065E-08
S1PR4	2.17463461	7.5656E-06	ANTXR1	-3.03101217	8.2189E-10
CCDC28B	2.17311475	3.0433E-06	GIMAP5	-3.0306856	5.1558E-20
P2RY10	2.16688829	1.1567E-07	STAB1	-3.01401559	9.8116E-29
PLP2	2.16421217	9.9492E-21	RNF150	-3.00506019	0.00190967
TRIB2	2.15855465	0.00738753	NBL1	-3.00384938	3.1774E-21
DRGX	2.15403175	0.04228336	LPIN3	-2.98901527	0.00214873
NRG1	2.14881632	4.4188E-05	C11orf45	-2.97283825	1.754E-05
PTGER3	2.14499283	0.00533626	TSHZ3	-2.97022116	7.1278E-11
UBXN10-AS1	2.13997311	0.04012492	PIK3CD-AS2	-2.96176628	0.02394473

CEACAM4	2.13719514	8.0067E-16	DYSF	-2.95837921	2.7949E-07
HAS1	2.13192483	2.6194E-06	PRELID3A	-2.94920888	0.00065512
ACPP	2.12907408	6.9037E-06	PPIC	-2.94704131	7.7086E-10
SLC35F2	2.11125235	0.00013199	FN3K	-2.9446316	4.2481E-14
G0S2	2.09984187	1.551E-20	CCDC40	-2.94219118	0.00042736
LGALS2	2.08858721	6.0763E-08	SMTNL2	-2.93950422	0.00290091
GPAT3	2.08018826	2.8482E-24	CXCL1	-2.93198305	6.4718E-13
CLEC5A	2.07626566	0.00554882	TNFSF18	-2.91264714	0.01142889
FRY	2.07499456	0.00272814	KIF3C	-2.91048451	0.00905245
ZNRF1	2.07289471	3.2899E-08	CLDN4	-2.90828856	9.2147E-05
RALA	2.06858282	6.865E-17	CCL4L2	-2.90642865	3.6489E-34
ASGR2	2.06847684	9.4355E-12	AP000439.1	-2.89439005	0.02144411
FAS	2.06374018	4.1563E-07	STMN2	-2.89430134	0.0025591
RP11-1055B8.	2.06210955	0.01368998	HSPA1B	-2.88068394	1.3497E-15
IFITM1	2.04923593	8.0847E-07	PAK6	-2.88065723	0.0020304
DAPK2	2.0482917	0.00202036	HES1	-2.87747829	1.4618E-19
GGT5	2.04450803	0.00024393	LGALS4	-2.87738531	1.4568E-08
IGF2BP2	2.04156437	1.7541E-06	FUCA1	-2.86450573	3.2246E-23
AC013461.1	2.03848087	5.5493E-24	CCDC152	-2.85578233	2.0178E-10
HTRA4	2.02878609	0.04249002	RAB42	-2.85263177	1.0142E-06
WARS	2.02675535	1.59E-26	PRR5	-2.84882865	1.5969E-09
DSG2	2.02531216	0.00991155	PLEKHH3	-2.83136152	1.9126E-08
ADGRE3	2.02408387	3.6513E-08	CMKLR1	-2.83027401	2.5735E-31
NOV	2.02290781	0.00152513	HRH1	-2.82433155	1.6342E-10
GIPR	2.01946846	0.00616047	NFIA	-2.82177014	1.799E-17
UTF1	2.01868173	1.1881E-08	ABLIM3	-2.81803849	0.00594339
MEI1	2.01516524	0.00050711	GPR34	-2.8141444	2.6376E-28
SLCO4A1	2.01476331	1.6363E-06	CCL13	-2.81341067	1.0234E-09
NT5DC3	2.01040297	0.00037919	CCL3L3	-2.80915034	2.5699E-36
NUP210	2.004134	1.675E-07	MICU3	-2.80053421	0.00995019
FLNA	1.99548044	7.5828E-16	WWP1	-2.79614585	1.7381E-37
SDPR	1.99506506	3.5868E-11	GARNL3	-2.79374804	0.00015948
CCL22	1.99400131	0.0002831	MAOA	-2.77239274	0.0104227
PPA1	1.9935452	4.2253E-37	DPEP3	-2.76152071	6.4515E-07
CTLA4	1.99124063	6.7063E-06	OLFML3	-2.75463714	1.076E-08
ABTB2	1.98872614	0.02999181	IGLL5	-2.74578918	0.01445794
TRMT6	1.98737124	1.6911E-14	GSTT2B	-2.73610932	2.5316E-07
ITGAX	1.98728271	1.0405E-11	A2M	-2.73257867	1.4736E-32
MCOLN2	1.98511182	7.3361E-08	CCK	-2.719836	1.9467E-05
SNTB1	1.9815221	0.01278541	NPL	-2.71974036	3.8005E-24
ADORA2A-AS1	1.9809766	0.0330021	IL2RA	-2.71790469	9.7778E-08
NKX3-1	1.9777131	0.0054657	ADORA3	-2.71008755	8.9619E-35
SYN1	1.97135218	0.00197294	LTC4S	-2.70433136	1.2093E-25
LMOD3	1.96475721	0.03109723	LGMN	-2.69870677	3.7182E-33
CTD-2371O3.2	1.96092686	0.02015747	ITGB5	-2.6986847	3.7236E-38
TESPA1	1.95984137	0.00162366	GDPD1	-2.69173185	0.00556978

PRDM8	1.95929523	3.0467E-07	COLEC12	-2.6899968	0.00088996
CD300E	1.95926534	7.2278E-10	RP11-881M11	-2.68872678	0.03191412
AIM1	1.95837866	3.4455E-46	IL20	-2.68619112	6.0497E-07
LIMD2	1.95001074	4.2215E-22	CCL11	-2.68392747	6.3936E-05
GNAZ	1.94904001	0.04204236	IGLC3	-2.68289493	0.00028007
NUDT11	1.94394382	0.0293505	KRT20	-2.67640646	0.02296819
SGMS2	1.93741217	1.6492E-08	ACTN2	-2.67460634	0.00866451
TTLL9	1.9372124	0.01858284	TMEM117	-2.67070535	0.00101019
LINC00694	1.93691929	0.00724925	ALDH1A1	-2.6698697	1.9183E-30
CD109	1.93462153	1.07E-06	AK1	-2.66961062	2.4619E-15
RHOF	1.93273623	8.3247E-10	ADAMDEC1	-2.66588093	2.9965E-25
UBE2C	1.92832773	0.02582585	PGBD5	-2.66133672	0.01510295
KIFC1	1.92386728	0.00277505	SASH1	-2.6603206	6.514E-21
S100A4	1.91530598	3.2218E-14	MLXIPL	-2.65793114	0.04109529
AKR1C1	1.91270205	0.00712993	CCDC80	-2.65151602	0.01883409
SH2D4A	1.91181364	0.02403766	ZKSCAN4	-2.64792965	1.0326E-08
IL2RG	1.91159026	5.7025E-26	PROS1	-2.63449659	5.3632E-12
LOR	1.90942518	0.03979986	FAM20A	-2.63336624	1.8877E-06
TREM1	1.90727312	1.3415E-28	ESR1	-2.63282041	0.0009775
PSTPIP1	1.90699225	8.2801E-11	SLC40A1	-2.63086447	5.7281E-23
TRAF3IP3	1.90625325	2.6706E-15	SPINK4	-2.62945108	0.02286038
GBP5	1.90496189	5.3823E-11	CCL8	-2.62918438	0.00259784
FAM111B	1.90286465	0.0425885	GPR82	-2.62624612	8.5922E-22
STK39	1.89952432	0.00055484	PLD6	-2.62061408	0.00204937
C12orf75	1.89866736	7.473E-05	RP11-588H23.	-2.61964792	5.6745E-05
CTC-529I10.2	1.89609232	0.00101588	PLA2G15	-2.61066687	1.1742E-25
GJB2	1.89434659	0.00010557	PIK3R3	-2.60875824	2.337E-15
SH2D3A	1.89113911	0.00085767	CD276	-2.59303835	8.5056E-20
SGOL1	1.89075749	0.0015977	RFPL1S	-2.58673256	0.00015941
ST3GAL4	1.88809751	0.00033466	ALDOB	-2.58282783	0.00090754
ASPM	1.88280825	0.03452343	CABLES1	-2.57701256	7.7635E-13
GINS2	1.87974324	0.00419026	APCDD1	-2.57700418	0.00078886
LAMB3	1.87719573	3.1984E-10	KLF2	-2.57027542	2.4054E-24
FLT1	1.86898122	4.402E-06	FCHO2	-2.56859806	2.5735E-31
RP3-394A18.1	1.86436744	0.00182776	MS4A4A	-2.55490847	5.3262E-33
PDE4D	1.85811489	2.0584E-10	CIART	-2.55478283	0.000203
TUBA1A	1.85103257	1.6664E-28	MYO6	-2.55265113	0.02350201
EREG	1.8494335	4.2659E-08	ZNRF3	-2.55262891	0.0020046
S100A9	1.84713981	1.9885E-05	CAV2	-2.54501711	0.01309751
EPB42	1.84472942	0.03072753	FAM166B	-2.5444154	0.0013383
AKAP12	1.84265283	0.00177464	EMILIN1	-2.5418895	7.4238E-13
MYLPF	1.83302424	9.2613E-06	LMTK3	-2.53673924	0.00561992
FUT7	1.83035405	0.00460793	RARRES1	-2.53157248	0.02552803
PPM1J	1.82792517	0.03648665	FCGR3A	-2.53128188	3.4029E-07
ADAM19	1.82787955	0.0001691	ZNF662	-2.51804621	0.01320898
SNX20	1.82618283	3.1263E-08	PDCD1LG2	-2.51524422	1.5777E-05

KIF18A	1.82463494	0.01013136	C1QB	-2.51495201	2.0262E-25
GLIPR2	1.8199861	5.7067E-12	SPIRE1	-2.50825572	3.7699E-18
LINC01272	1.81762375	1.0825E-11	PHGR1	-2.507554	1.9436E-09
NKG7	1.81475944	4.5663E-05	PDP2	-2.50482686	0.00815704
NREP	1.80097273	6.2651E-07	BLNK	-2.50361091	3.4764E-17
ZNF408	1.80000871	0.00022215	TRIM32	-2.50085516	0.00134317
ANKRD22	1.79942279	0.00239784	ABLIM2	-2.4999757	0.02061684
XKR8	1.79880156	1.8261E-06	LACC1	-2.49535929	6.0223E-49
LINC01315	1.79733793	0.00041732	DISP1	-2.49196675	5.6911E-05
UHRF1	1.77691028	0.01464576	SPIRE2	-2.48529282	0.03211599
FCAR	1.77518563	0.02641851	STAP2	-2.48087401	0.04190832
LINC01588	1.77273284	9.3757E-05	NISCH	-2.47021507	1.7466E-34
BANK1	1.77256353	0.00736175	ICAM5	-2.46565532	4.8855E-05
KCNN4	1.7700591	0.00358217	COL3A1	-2.46453396	0.00023218
AREG	1.76864022	8.7368E-15	FMNL2	-2.46131473	2.8691E-19
CLMN	1.76847167	0.00020565	MMP14	-2.45209726	3.5432E-15
HAPLN3	1.76757052	6.0845E-07	SIGLEC1	-2.44715379	1.4016E-27
KIAA0101	1.76647538	8.0345E-08	CCND1	-2.44670217	1.2528E-15
PKP2	1.76530796	1.9458E-17	FGD5	-2.4449154	4.9579E-07
BCL2L14	1.76462284	0.00153241	C15orf65	-2.44002881	0.040583
MAP4K4	1.76124315	1.2063E-08	SDC3	-2.43922205	3.4141E-24
PRKAG2-AS1	1.76090378	5.4363E-15	TSPAN8	-2.43879567	0.00289575
CENPM	1.75822735	0.00366262	EPN2	-2.43770002	1.6986E-08
INHBA	1.75606164	5.3977E-06	AURKC	-2.43205615	2.3814E-05
GAPT	1.75357747	2.2251E-09	EGR2	-2.43113924	1.1163E-13
SLC4A3	1.7509566	0.02205916	PRUNE2	-2.42240438	1.8047E-07
COL9A2	1.75020803	2.1109E-05	GDF15	-2.42115442	0.00265017
AKR1C3	1.74913842	0.01091803	RTKN	-2.41508818	0.00616371
TK1	1.74478866	0.00084151	ACSM5	-2.4149139	0.00056153
IRF4	1.74242218	9.2917E-08	INPP4B	-2.41214888	4.0312E-08
HLA-DOB	1.73680912	4.1401E-07	LRFN1	-2.40251806	5.7433E-07
MN1	1.73381543	0.02329636	ZDHHC19	-2.39877965	3.0835E-11
BZRAP1-AS1	1.73293086	4.8225E-05	DNAJB4	-2.39753621	1.7887E-27
MYBL2	1.73167045	0.03492845	CMBL	-2.39583761	0.02335887
FAAH	1.73075376	0.00060893	LHFPL2	-2.39541744	7.8445E-33
CDK14	1.73013248	0.00037724	IFITM10	-2.39094306	1.3208E-14
LILRA5	1.72819879	3.2193E-11	CTSL	-2.38323154	3.4868E-23
AC010883.5	1.72687303	0.01688049	PLAU	-2.3822214	9.317E-21
ITGB8	1.72554151	0.03282591	PLEKHG3	-2.37868355	3.7232E-06
E2F1	1.72128942	0.03945452	DCBLD2	-2.37861852	1.4677E-08
SLC16A6	1.71809052	4.3279E-05	DCN	-2.36732908	0.0003577
AFF3	1.71788999	8.7315E-07	TREM2	-2.36354157	1.2263E-07
SPNS3	1.71384744	2.1705E-09	ZKSCAN3	-2.3569946	0.01278541
CEBPE	1.70066873	0.01511537	SLC25A23	-2.35371761	0.00686378
FCGR2B	1.69899036	6.3792E-05	FAM212A	-2.35097154	0.04927131
VCL	1.68601333	1.5769E-12	NEURL2	-2.34986357	2.2199E-07

RP11-598F7.3	1.68061448	0.0016608	CTSF	-2.34171557	4.9267E-19
LINC00484	1.67893199	0.03960064	WBP5	-2.3392853	7.3223E-23
SVIL	1.67877024	6.0085E-07	NPHP1	-2.31834201	0.0142469
ALPK2	1.67867891	0.00295904	GNG12	-2.31713208	0.00364456
ANKRD28	1.67448773	2.772E-28	WBSCR27	-2.30789471	1.327E-18
CAPN2	1.66838636	4.0128E-15	SGMS1	-2.30448494	7.1088E-25
STK26	1.6673089	2.5924E-06	FARP1	-2.30403059	2.6597E-08
RP11-1151B14	1.66558388	0.00811843	RP11-474I16.8	-2.29944085	4.8882E-05
LINC01010	1.66187593	0.03586413	MAPK12	-2.29873384	7.1771E-05
CITED4	1.66152974	4.4938E-10	FOS	-2.2971608	3.8967E-25
CSTA	1.65833713	7.9681E-17	DTNB	-2.29591886	5.404E-09
RP11-850A17.	1.65773909	0.02379951	C1QA	-2.29428845	7.3223E-23
CDH17	1.65523157	0.000506	DOCK4	-2.29239609	1.3907E-15
TIGD3	1.65354897	0.01883409	STON2	-2.28379003	4.7882E-17
AK4	1.65246423	0.04651045	UNC5B	-2.28332738	6.0006E-10
OCIAD2	1.65047269	0.00030748	C1QC	-2.27958012	1.3915E-12
ZEB1	1.64923568	0.00567041	NR1H3	-2.27841282	1.6987E-20
LYST	1.64635028	4.6917E-14	ZNF354C	-2.27819089	0.00277253
IL1R1	1.64105422	1.0278E-08	ACE	-2.26773219	2.9874E-05
PROSER2	1.63974952	0.02603675	DNAJA4	-2.26770352	3.1557E-16
RP11-147L13.	1.63683216	5.3683E-07	COPZ2	-2.26723278	1.9727E-09
C15orf48	1.62881079	4.017E-09	QPRT	-2.26631271	6.4961E-40
CDKN2D	1.62877469	0.00010424	PLCG1	-2.26431233	0.03563554
CYFIP2	1.62147224	0.01280664	TMEM79	-2.26093619	0.00035875
P2RX1	1.61730981	0.00022508	TNF	-2.25607092	3.8758E-07
TLR10	1.60847042	0.00879087	ZNF704	-2.25336245	2.9632E-08
FAM129A	1.60843971	3.4121E-11	NFIB	-2.24532191	0.00691782
SLC2A1	1.60758113	0.0103297	SCD5	-2.2447149	0.02083446
PPP1R3B	1.60676379	5.3824E-07	NHLRC4	-2.24312333	0.00043664
GPR65	1.60478656	8.2707E-26	RP11-536K7.3	-2.23074765	3.0169E-05
SPON2	1.59386486	5.6311E-07	SPRED1	-2.22391835	6.1306E-37
PKIB	1.59384075	0.00016141	SLC46A1	-2.22262591	2.2102E-14
RP11-446N19.	1.58760236	0.00386937	FKBP9	-2.21831862	1.4577E-06
PLPPR2	1.58576937	0.01779348	GIMAP8	-2.21752361	3.782E-18
SH2B2	1.58161374	4.2362E-08	PRAP1	-2.21721632	0.03700211
CORO1A	1.57741634	4.8204E-43	RP3-486I3.7	-2.21468877	0.0379904
PVT1	1.5762144	0.00651138	NEIL1	-2.21430702	8.5566E-07
LILRA1	1.5761833	5.7442E-07	AMY2B	-2.20766834	8.0931E-06
GPR146	1.57549272	1.9354E-06	SIGLEC11	-2.20010653	1.781E-09
SERPINA1	1.57264914	4.4349E-10	CDH13	-2.19791215	0.03764394
PRAM1	1.57236976	1.8563E-07	PCAT19	-2.19693032	0.02305256
C1orf228	1.56936628	0.01633537	STARD13	-2.19288169	6.6172E-14
BEX2	1.56749185	0.00068828	HPGDS	-2.19156895	0.00042448
CCND2	1.56736055	7.9507E-24	CTSD	-2.19084305	6.3625E-19
TCAF2	1.56672797	0.01977265	MALL	-2.18841104	0.04101471
CACNA2D3	1.56597002	1.497E-19	MRVI1	-2.18827021	0.00550808

AP1S3	1.56580813	2.6518E-07	COL23A1	-2.1749058	0.00026583
MIR4435-2HG	1.564368	8.7114E-18	L3MBTL4-AS1	-2.17242581	2.5011E-05
ASB2	1.56088152	0.00455595	CCDC102A	-2.17104957	9.1698E-05
SYNJ2	1.56034512	0.00733478	LINC01094	-2.16783623	3.896E-06
CALCRL	1.55945991	0.00261114	COX7A1	-2.16773791	8.9555E-07
MTHFS	1.55379377	1.2727E-15	SEC16B	-2.16669065	0.04033056
KCTD15	1.55314839	0.00389049	SLC45A4	-2.16191142	1.7612E-22
LTB	1.54867111	6.2647E-10	ACVR2B	-2.1562819	0.00017109
CHAF1A	1.54847014	2.8256E-07	DNAJB1	-2.15307636	8.7948E-15
SYTL3	1.54805976	3.1091E-18	SLC38A7	-2.15134972	5.977E-11
ANXA6	1.54551218	1.4421E-15	FBXO16	-2.15028559	1.4215E-05
CTD-2547G23	1.54166833	0.00060775	APOC1	-2.14461797	1.6329E-06
LYPD3	1.54056111	2.7561E-08	SMO	-2.14454532	0.00241936
MAP4K1	1.53776797	4.965E-14	MYZAP	-2.14404516	0.00100187
IGLON5	1.53699997	0.01181067	GCNT1	-2.13995893	2.3764E-21
CASP9	1.53588085	7.4719E-11	ARHGEF12	-2.13499974	1.1855E-21
C1RL-AS1	1.53327091	0.01769852	SIGLEC12	-2.13270989	1.005E-09
MYB	1.53251215	0.00444949	CCL23	-2.12988784	0.002569
DUSP4	1.5320658	2.2057E-20	PLD3	-2.12986429	7.4812E-35
ST20-AS1	1.53044379	0.01983958	KRT19	-2.12980939	0.0232804
OXER1	1.52557232	0.02401455	MCC	-2.12793026	0.00164056
GPRC5A	1.52391654	0.00306934	ADGRA2	-2.12559102	0.00432118
SLC2A6	1.52317899	0.00166906	S100A14	-2.11926949	0.01714537
RNF24	1.52250409	4.9475E-06	RP11-605B16.	-2.11723744	0.01612559
TCTN3	1.52184977	5.1745E-16	ANKRD29	-2.10422716	0.0361296
PRKCE	1.52152772	9.9358E-06	PDGFB	-2.1011597	6.5725E-27
S100A10	1.52151449	2.6703E-20	CD200R1	-2.09748011	7.2633E-10
TAP1	1.52131364	1.2789E-21	GATM	-2.09548868	7.5967E-55
MARCO	1.51736743	0.01398364	GNA11	-2.09086211	8.9415E-06
PC	1.5170529	0.02008134	ZNF485	-2.08916063	0.00307763
CD300LB	1.51575636	1.4433E-11	SEPT10	-2.08626001	8.7315E-07
SPN	1.51472021	3.0248E-06	PER3	-2.0841286	9.4903E-20
CHRNE	1.51451312	4.8721E-06	TTC28	-2.08162232	1.1231E-05
TMEM71	1.51251774	9.5871E-10	ARHGAP6	-2.08052936	1.305E-05
RP11-544A12.	1.51001493	0.00114948	PAQR8	-2.07966921	4.7283E-13
RP11-796E2.4	1.50611001	6.0155E-07	CLCN4	-2.07494022	4.3956E-08
PLCD1	1.50142328	0.00016751	PAX8	-2.07419334	0.00037004
AC002456.2	1.49915162	0.0062748	ABCC3	-2.07149791	3.4247E-09
NBEAL2	1.49499367	4.5499E-06	TSPAN15	-2.07147326	3.9327E-20
TPPP3	1.49472826	1.7345E-06	CTDSPL	-2.06763959	0.00346169
RP11-416N2.4	1.49223531	0.02332414	WNT5B	-2.06314728	2.5395E-09
WDR59	1.49186349	3.3723E-10	HSPA1L	-2.05843855	0.00422026
FMNL1	1.49154582	1.7753E-13	PLXNA2	-2.05548822	1.6479E-06
TIMP1	1.49108578	6.9296E-17	SLC38A6	-2.05238566	1.9473E-17
SYCP2L	1.49073602	0.02441755	NGFRAP1	-2.04956554	1.1094E-25
FPR1	1.48734685	5.1518E-09	RHOBTB3	-2.04776628	8.5168E-23

TMEM158	1.48383734	0.0005013	EPHX1	-2.04560002	1.7883E-17
RP13-270P17.	1.47929437	0.00068533	KDM8	-2.04427297	0.0356959
RAB3D	1.47881582	0.02796475	GRIN2D	-2.04105171	0.00429326
LY9	1.47876558	4.3651E-06	SLC25A44	-2.03333298	1.5649E-13
RAB11FIP1	1.47676628	3.9577E-25	CTTNBP2	-2.03209955	2.0474E-21
RAB27A	1.47608765	1.5569E-06	CD320	-2.03173549	1.209E-14
RETN	1.47548427	0.03329428	KIAA1683	-2.03048681	2.03E-07
RP6-91H8.3	1.4724781	1.2053E-07	GIMAP6	-2.02695573	1.1965E-15
NOD2	1.47207968	4.2015E-06	TBC1D16	-2.025852	1.2237E-11
RP11-467L13.	1.47094783	0.00025707	PPP1R26	-2.02350027	0.00626907
NAP1L5	1.47062695	0.00036939	STK36	-2.02124569	0.00022638
TACSTD2	1.46985775	0.0367188	RND3	-2.01788881	2.0838E-13
SLC7A11	1.46945143	0.0068135	PCGF2	-2.01727615	0.01201256
RAB33A	1.46917262	0.00508382	SRPX	-2.01016199	0.01315483
DHRS9	1.46838965	0.00025153	B4GAT1	-2.00940373	0.00363634
DAPP1	1.46807525	1.2339E-09	EPAS1	-2.00880663	4.2318E-10
MYO1G	1.46454983	3.1174E-12	LRRC4	-2.00606241	0.0006846
SYNGR1	1.46422371	3.619E-05	RP11-202G18.	-2.00290453	0.00422026
C16orf54	1.46366894	0.00034005	NUPR1	-2.00063401	0.00011779
ITGB2-AS1	1.46205653	3.6148E-14	DCHS1	-1.99703297	0.00273366
TMEM120B	1.4593404	8.7043E-06	LAMB2	-1.9969789	5.0832E-06
TRAF1	1.45879276	0.00022816	USP51	-1.99327187	6.9832E-06
SP140	1.45850122	0.00020297	AP2A2	-1.99246128	7.9125E-35
CCDC88C	1.45519926	0.00056715	TTC23	-1.99229439	0.00047407
FBP1	1.45457368	1.1026E-18	LINC01358	-1.98076485	0.04043309
AF064858.8	1.45444695	0.01097864	MAN1C1	-1.97909352	1.1774E-08
SH3BGRL3	1.44854686	5.7145E-36	CD14	-1.97740111	5.16E-23
PON2	1.44570488	1.5767E-08	FABP3	-1.97410064	0.02893124
RP11-404O13.	1.44357999	4.9642E-05	KCNMB4	-1.96949116	8.1588E-10
TNNI2	1.43764809	5.4849E-08	ATF3	-1.96865082	2.7898E-21
IL1RN	1.43752694	0.00656506	TNFRSF13C	-1.96622282	1.0266E-05
GEMIN4	1.43508971	0.00045044	RP11-401F2.3	-1.9656138	8.1077E-05
TNIP2	1.43067776	1.9829E-10	MRC2	-1.96268298	1.8668E-09
CBX6	1.42955798	8.6075E-21	TNRC6C	-1.96080137	2.8708E-05
CD101	1.42942847	9.3667E-06	PIR	-1.95969819	0.00341763
TBC1D10C	1.4282963	0.00133589	DYNC2H1	-1.95609552	0.00095557
SLC11A1	1.42754565	4.8762E-05	RHOBTB1	-1.95535931	6.9143E-16
RP11-176H8.1	1.42720734	0.04020002	LINC01554	-1.95479377	0.00557151
PPP1R14A	1.42712976	0.00091959	RCN3	-1.95399149	1.2661E-10
TRAF3IP2	1.42681732	0.00225133	RP11-57H14.2	-1.95125303	0.03331242
AC093673.5	1.42637302	0.00023429	ARRDC4	-1.95114516	5.575E-10
BCL7A	1.4234811	2.1251E-06	PTGS2	-1.94736801	5.3077E-32
KLHL25	1.42022344	0.0164655	RP11-556E13.	-1.94383954	3.9625E-09
MALT1	1.41875293	2.4599E-43	ZNF420	-1.93730357	0.00040045
LDLRAD3	1.39922206	0.0033845	ACP2	-1.93717639	1.7163E-23
NRARP	1.3963952	3.4021E-10	KLF6	-1.93536975	4.6973E-20

GPR171	1.39439031	8.7315E-07	AC240274.1	-1.93525608	2.0727E-12
ARSG	1.39120075	2.6756E-07	IL6	-1.93497705	7.0549E-06
P2RY2	1.39012919	0.01789338	RBP7	-1.93118236	2.3207E-07
SIRPB1	1.38981529	0.00033201	PART1	-1.93021247	0.0320337
PDLIM1	1.38945001	0.00015825	MITF	-1.92734395	8.9295E-23
CDK2AP1	1.38575193	2.129E-08	MAFB	-1.92729334	5.7823E-31
TSPO	1.38529735	6.6948E-09	PLCL1	-1.92216988	4.1741E-06
TRIM25	1.38485937	5.6386E-07	ZSCAN26	-1.92138492	8.7364E-05
PTX3	1.38227642	0.000437	KLHDC8B	-1.92008759	2.1661E-06
PTGIR	1.3767529	9.2632E-05	ENO2	-1.91976087	0.03367723
HOTAIRM1	1.37211842	3.2301E-13	RP3-369A17.4	-1.91755987	0.04271236
YDJC	1.37153302	4.3457E-11	TRMT5	-1.91552735	0.00053322
CPVL	1.37113112	9.4097E-13	SDCBP2	-1.91235627	0.00867298
SUV39H1	1.3706133	0.03911391	DMXL2	-1.91206617	4.994E-41
IL4I1	1.37040715	3.5634E-07	LINC00346	-1.9063622	2.1924E-06
TNFRSF18	1.35950106	0.04173131	GPR155	-1.90578595	8.1722E-15
CD48	1.35585543	4.4336E-21	ZNF619	-1.9021606	0.03991261
RHOC	1.35476492	5.1855E-28	IGHA2	-1.90115416	0.00283326
MPZL3	1.35245482	0.01148275	ST5	-1.90042211	2.6749E-08
CDH23	1.35233643	0.00011098	RP11-701P16.	-1.89986174	0.00563969
FBLN2	1.34971294	0.010021	SAA1	-1.8995417	0.00613606
DGAT2	1.34276737	0.00722962	MAPK13	-1.89883033	0.00071872
CA5B	1.34188292	5.6675E-05	DHFRL1	-1.89605802	0.01320005
POC1A	1.34155151	0.033829	GULP1	-1.89319998	0.01343265
APITD1	1.33788507	0.01317538	RGL1	-1.8916616	6.2065E-26
RAB3A	1.33384182	0.00853708	GPR161	-1.88708976	0.01348907
GK	1.33194399	2.7829E-11	CCL21	-1.8857298	0.03779357
LDHA	1.33017151	2.5318E-25	AMACR	-1.88390394	0.04381685
ISG20	1.32184675	2.3473E-15	RP11-731C17.	-1.87751231	0.00155485
MNDA	1.31773106	8.9254E-15	TLR7	-1.87286793	1.2584E-09
ALOX15	1.31647344	0.04986883	TEX14	-1.87273763	0.00043389
SMCR5	1.31623676	0.00014353	RP5-899E9.1	-1.87153455	8.2484E-08
CERK	1.31545449	1.1531E-10	ZNF613	-1.87143948	0.01708173
RP11-568N6.1	1.31537142	0.00930663	FAXDC2	-1.87066837	3.2899E-08
RAB39B	1.31355027	0.0021477	TCF12	-1.87047117	1.1158E-19
PRICKLE4	1.31350817	0.00390994	BAG3	-1.8686048	3.2102E-06
GFOD1	1.31188062	2.197E-05	DOK3	-1.86841447	4.3271E-15
IMPA2	1.31168553	3.5021E-13	COL6A2	-1.86816354	0.03287193
CXXC5	1.30902291	0.00076266	SLC2A5	-1.86771424	0.01406428
AGO2	1.30852828	5.4369E-13	KIAA1211L	-1.8666403	0.00168742
KIAA0040	1.30229081	1.3008E-05	AHDC1	-1.86648158	1.0274E-06
STK4	1.29846545	5.0371E-21	APPL2	-1.86518913	2.1482E-13
FPR2	1.29720543	0.0150239	CD209	-1.86137391	4.0952E-23
RP11-1143G9.	1.29331741	2.2426E-08	INTU	-1.86130281	0.00146831
KRT18	1.29308342	0.00291177	ABCC5	-1.85969865	2.2897E-15
SPP1	1.28948342	0.04568416	GFRA2	-1.85488692	1.7729E-23

PSKH1	1.28879326	0.02706583	NHS	-1.85010088	5.1642E-05
CA2	1.28857439	0.00106131	ZNF57	-1.84536194	0.01925127
TTC39C	1.28656083	5.1563E-06	TMEM176B	-1.84480426	8.4423E-08
CTD-2020K17.	1.28443613	0.00018892	CTSC	-1.8446451	2.118E-40
FABP5	1.28394496	7.6531E-14	HPSE	-1.84377622	1.992E-06
SLC9A7	1.28238234	0.00060646	CITED2	-1.84309868	1.1824E-29
LINC00152	1.28074148	6.5248E-12	HSPA1A	-1.83953409	1.3052E-05
CD37	1.27656277	6.1272E-22	ZNF792	-1.83783917	0.04458912
GHRL	1.27628429	1.9286E-06	TMEM176A	-1.83767869	4.3184E-11
FSD1L	1.27552475	0.00490044	ADAP2	-1.83604009	3.0153E-42
ACAA1	1.27535233	6.2006E-16	SNHG12	-1.83477983	9.5363E-22
PSME2	1.27035483	1.5514E-25	FAM71A	-1.83438095	0.00676157
DTNA	1.27011154	0.01207635	RGS1	-1.83239359	6.5865E-26
PDCL3	1.26939925	9.7209E-23	ARHGAP12	-1.82425265	1.1595E-10
QSOX2	1.26793018	0.00415504	NEU1	-1.8234368	1.5686E-17
RP4-728D4.2	1.26621214	0.03848365	GIMAP7	-1.82277587	4.0284E-18
SMCO4	1.26494056	2.5394E-09	SLC25A34	-1.82147949	0.00657745
CAPN10-AS1	1.26058856	0.01232476	BMP2K	-1.81929942	4.0785E-40
AVPI1	1.2595049	1.1255E-08	FXYD3	-1.81620721	0.00915765
BORCS5	1.25938848	1.3111E-08	FAM13A	-1.81213121	1.1174E-12
CD79B	1.25838737	0.03565789	ASB9	-1.81184267	1.3597E-09
RP6-159A1.4	1.25675899	0.00065908	SGPP1	-1.81151533	3.9914E-16
PDE3B	1.25631313	4.3678E-06	HYAL2	-1.81039352	1.1312E-14
IRF9	1.25359134	1.0878E-16	LINC01136	-1.81008324	0.03230904
BID	1.24745743	1.0829E-22	SLC9A9	-1.8055977	1.3191E-23
FAM118A	1.24611791	2.0399E-09	PPFIA3	-1.80524057	0.01576031
EMP1	1.24416419	0.00010595	BMP2	-1.80355664	0.00116355
CD274	1.24397699	0.03551578	C3	-1.79837963	0.00247075
CLEC12A	1.24154724	6.1494E-06	CACNB4	-1.79727299	1.0751E-08
CTPS1	1.23653493	0.0003609	NOTCH1	-1.79145384	1.3311E-16
KCNMB1	1.23474076	6.9715E-08	SLC25A4	-1.78855534	0.00643001
CKLF	1.23471287	3.2377E-34	RHOB	-1.78737023	1.8457E-09
SCRG1	1.23363414	0.00427743	LRRC6	-1.78541037	0.00374927
EXOG	1.2292537	2.7391E-05	CBY1	-1.78483556	1.959E-05
HMGA1	1.22816564	2.7315E-12	HGF	-1.78322879	0.00051422
PPP1R16A	1.22583876	0.00035454	L3MBTL4	-1.77997145	3.7515E-05
CTSW	1.22532404	0.04359801	PIP5K1B	-1.7793952	6.472E-07
CLEC4F	1.22375458	6.3828E-05	CHADL	-1.77806681	0.01518426
STRAP	1.21935232	4.9869E-27	C1S	-1.77262464	0.01186808
CLEC10A	1.21579748	3.4176E-11	COBLL1	-1.77235424	0.00633853
IGFBP7	1.21434534	0.00011355	GIMAP1	-1.76963083	1.6247E-11
TCTEX1D2	1.21408259	0.04145504	FAM21A	-1.76819105	4.8792E-20
SEPT6	1.21383432	4.065E-20	TFF3	-1.7677328	0.00509695
SYNE3	1.21331846	0.000864	CXCL3	-1.76590811	6.0963E-24
MYOF	1.21282863	1.0646E-06	LRP3	-1.76230639	0.00195805
MSRB1	1.21199439	1.5164E-05	IL10	-1.76072614	4.3184E-11

MCM8	1.21024337	0.02582379	NMRK1	-1.76029092	6.4194E-15
SGK494	1.21007655	0.00059011	HFE	-1.75970613	1.3233E-07
LYZ	1.20921942	5.1431E-07	IER2	-1.75969042	1.2749E-16
R3HDM4	1.20888705	3.6823E-10	ZNF554	-1.75876615	0.03891718
MX2	1.20537028	2.5618E-11	EFCAB7	-1.75289693	0.03120629
PARVG	1.20376239	2.2127E-30	CTD-2369P2.8	-1.74996024	0.00226483
CD1D	1.19940211	1.0377E-06	PFKFB2	-1.74990997	8.7364E-05
LTA4H	1.19822256	1.3303E-17	FHAD1	-1.74973203	5.236E-06
SQLE	1.19591719	1.6852E-05	ZNF624	-1.74882885	0.01977265
SATB1	1.19354284	1.5836E-12	KLF7	-1.7476208	8.9903E-34
ASH1L-AS1	1.19144367	0.01545831	KCNG2	-1.74686827	0.02029066
C1orf162	1.18783004	2.4194E-13	JAM2	-1.74269412	0.0132579
NDST1	1.18736079	0.0177943	RP11-343H5.6	-1.74157594	9.7012E-06
NEURL1	1.18730206	0.00301698	CDKN1C	-1.73949654	1.0385E-05
TRAF4	1.18643665	0.00017748	DDIT3	-1.73921335	6.1304E-20
CENPH	1.18620727	0.00064767	RP1-30M3.5	-1.73812722	0.02881525
PSMB9	1.18561218	1.4418E-29	RPP25	-1.73387369	2.4814E-11
CENPW	1.18087068	3.9223E-05	ETV5	-1.73369276	2.3055E-15
RP5-1171I10.5	1.18070955	4.0923E-19	BRSK1	-1.72497462	0.03891718
PDE2A	1.17482045	0.00624248	SLC22A23	-1.7246601	1.9114E-06
CCR2	1.17474076	0.04000015	PRTFDC1	-1.71911441	0.01113058
GMPPB	1.17372515	4.2332E-06	SPATA7	-1.71624624	2.0381E-06
RAB3IP	1.17196352	0.02344822	CYB5D1	-1.71185476	1.4702E-13
PDSS1	1.17081456	0.00578514	AC074289.1	-1.70737921	4.3243E-13
PTK2B	1.16689345	4.8248E-11	RBP1	-1.70239803	8.3088E-05
OBFC1	1.16372369	2.8047E-07	FBXO32	-1.70221659	0.02871754
RIPK2	1.16328277	1.2234E-07	EFEMP2	-1.69828572	0.00262149
APOBR	1.16046225	9.8115E-09	ZNF189	-1.69688244	9.0201E-08
ECE2	1.15896319	0.00084172	ZFP36L1	-1.69629642	6.1256E-20
PTTG1	1.157159	1.1939E-08	ITPR2	-1.69627015	2.8773E-22
HELLS	1.1570182	0.00449746	PNPLA7	-1.6944794	0.00246926
SLC2A3	1.14771368	4.1635E-11	RBM43	-1.69302898	2.3202E-06
ENTPD7	1.14706495	0.0005214	SNTB2	-1.69128767	9.6146E-08
CES4A	1.14689398	0.02800375	MMP1	-1.6901658	0.00149038
AP003774.6	1.14684031	0.00101743	FAM213A	-1.68943992	2.5264E-18
VIM-AS1	1.14675106	9.1434E-07	KREMEN1	-1.68419956	0.00744055
S100Z	1.14440124	0.00031983	ULK2	-1.6841441	1.6758E-12
CD226	1.14340996	0.00109868	CIPC	-1.68374981	1.1032E-08
PLD4	1.14302694	4.6553E-07	SOCS2	-1.68287236	5.6546E-07
OAF	1.14096921	0.00077612	CAMSAP2	-1.68226506	1.3208E-14
FAM184B	1.13973563	0.04608795	C17orf80	-1.6752615	0.00182317
CRADD	1.13946706	0.01740869	TEF	-1.67396209	0.02119212
DLEU1	1.13585127	0.00602126	CREG1	-1.66485308	6.2969E-56
RP11-347P5.1	1.12768021	5.1204E-05	SERPING1	-1.66301914	3.2616E-12
HDAC1	1.12676399	1.7401E-12	USP54	-1.66047346	0.03768573
IL3RA	1.1267058	1.3588E-05	RNF208	-1.66007494	0.00223075

JHDM1D-AS1	1.12435716	0.00297235	AC009404.2	-1.6583674	2.3335E-05
LRRFIP1	1.12241998	9.6745E-27	KB-1460A1.5	-1.6578235	0.01948607
POLR1C	1.12238579	0.00192343	DTX2	-1.65638745	2.7783E-14
PKM	1.12077247	2.0346E-25	CARD11	-1.65589528	0.00991126
GCHFR	1.12070713	1.4423E-05	RP11-624L4.2	-1.65549957	6.86E-05
CLEC4D	1.12070451	0.00418348	LCAT	-1.65368928	0.00153177
LY6K	1.11897114	0.01910024	BHLHE41	-1.65305001	0.00159685
ENO1	1.11686582	1.4486E-22	TCTEX1D1	-1.65103795	0.0004435
GBP1	1.11686552	6.7954E-06	EGR3	-1.64907523	2.3861E-08
CEP83	1.11508542	1.4337E-07	NKAPL	-1.64798995	0.0258834
FLOT2	1.11312598	3.9397E-09	DDR1	-1.64672075	0.0057868
BZRAP1	1.11286387	0.03593472	KIF1B	-1.64427813	6.1021E-48
NPFFR1	1.11159655	0.03348681	RP11-4B16.4	-1.6430198	6.276E-05
OSCAR	1.11067533	0.00016045	RP11-365P13.	-1.64168632	0.00212745
COTL1	1.1097911	1.5193E-14	B3GNT8	-1.6403682	0.00092923
CTDP1	1.1091617	9.3533E-08	TP73-AS1	-1.63948939	0.00012071
TET2	1.1075503	2.6976E-15	NCKAP5	-1.63314865	1.3859E-05
PRELID1	1.10401063	1.9065E-20	MS4A7	-1.63269013	1.1627E-30
TIMM17B	1.10310104	8.532E-13	LGR4	-1.63171275	0.02582585
C8orf44	1.09990453	0.03634325	ZC3H12D	-1.63156193	1.4596E-09
NDRG2	1.09773329	8.5606E-10	ICAM4	-1.63036755	9.0964E-12
PGAM1	1.09748996	2.2645E-26	ALS2	-1.63028799	0.00139786
SLC16A10	1.09744303	0.00281929	DPY19L3	-1.62798252	3.9446E-05
SPTLC2	1.09409813	7.0261E-14	LGALS3BP	-1.62789751	2.6609E-15
KIF22	1.09360267	6.5827E-09	WDR81	-1.62702443	9.3064E-13
NME1	1.09352059	1.2514E-22	PLPP3	-1.62662654	0.02246296
NDEL1	1.09222665	1.1626E-17	ENPP2	-1.62536031	2.407E-17
TVP23C	1.08985315	0.03240789	PDGFC	-1.62398535	3.6808E-14
PHF19	1.08955891	0.00017414	RNU12	-1.62229375	3.4799E-06
ACSL5	1.0867074	1.1401E-07	SUCNR1	-1.61986641	0.00220122
LHFP	1.08609238	8.2376E-07	YAE1D1	-1.61786905	5.4329E-05
MT2A	1.08216432	1.0159E-06	GRK5	-1.61524328	9.2606E-07
SNRNP25	1.08168511	6.2326E-15	DUSP6	-1.61336533	1.5261E-12
CBWD1	1.08125686	9.755E-15	SCARB2	-1.61210798	1.8049E-21
RNF139-AS1	1.07963509	0.01155451	TNFAIP3	-1.60971906	3.2217E-29
EHF	1.07902189	0.02111397	CA4	-1.6067048	0.02706611
CPPED1	1.07788458	1.2093E-18	SERPINH1	-1.60616757	1.5645E-05
GPSM2	1.0754634	0.02339124	MGP	-1.60308274	0.03071843
OPN3	1.07543458	1.4844E-10	MMP10	-1.60066522	0.00612003
ZBTB11-AS1	1.07533164	0.01941938	SH3PXD2A	-1.60025487	1.4303E-13
SLC25A19	1.0750776	4.9869E-09	CNNM3	-1.60013678	0.00713113
SUMO4	1.07400853	0.04777878	KCNMA1	-1.59964079	3.8246E-06
FAM229B	1.07249587	0.02307421	NRP2	-1.59771315	2.3691E-15
MYD88	1.07237383	6.6684E-12	DEPDC7	-1.59616451	0.00520374
CCND3	1.07166932	9.0068E-11	FOXRED2	-1.59419341	1.703E-09
PID1	1.0684313	2.8787E-08	GLMP	-1.59393743	3.7378E-16

AC016831.7	1.06825503	1.1196E-05	LUM	-1.58517123	0.02013825
CENPN	1.06692956	0.00014749	CFH	-1.58355707	4.2916E-05
CYTIP	1.06440325	3.1477E-12	IDH1	-1.58354682	4.4153E-26
OAS3	1.06258245	0.0019766	PLPP6	-1.5827047	0.01153975
SLC7A1	1.0596723	2.3191E-05	IFFO1	-1.5814858	1.5041E-11
PAK1	1.05356742	5.7266E-17	SOWAHC	-1.58135314	0.01965817
SRM	1.05333157	3.1218E-14	HS3ST1	-1.57741104	8.2337E-07
EID2B	1.05249891	0.0098958	NBPF1	-1.57596717	2.1852E-06
LRRK2	1.05030507	1.6522E-05	TXNIP	-1.57435355	3.4008E-12
REV3L	1.05000054	3.362E-11	HMOX1	-1.56768982	8.126E-22
PDE7A	1.04950045	3.2566E-05	CTB-119C2.1	-1.56616358	0.01125374
MYCBP	1.04679292	1.0381E-08	ZNF844	-1.56491478	6.3019E-10
CBFA2T3	1.04677905	0.00316002	DMD	-1.55909478	0.00900153
MBOAT7	1.04657418	9.7144E-12	TSPAN4	-1.55851001	3.9864E-27
SIPA1L1	1.04495006	0.00010858	LMAN2L	-1.55480597	0.00147709
RAC2	1.04366591	9.1323E-21	TCN2	-1.55337732	6.5969E-19
VNN1	1.04350503	0.02267263	KIAA0226L	-1.55038125	3.6736E-09
NLRC5	1.04290091	0.00039057	RASSF8-AS1	-1.54883625	0.01252671
HOXA5	1.03909484	0.00054602	SGK223	-1.54554295	7.476E-05
FGGY	1.03865797	0.00183182	FOSB	-1.54514302	4.2816E-24
LINC00528	1.03702299	8.168E-07	RP6-99M1.3	-1.54347661	0.01684617
SACS	1.0364938	8.3042E-05	SLC37A4	-1.53745927	1.9973E-06
EIF2AK4	1.03423164	1.3278E-14	MT1G	-1.53583966	4.6934E-10
METTL1	1.03348082	0.00010557	PRDM1	-1.53574267	1.4821E-19
FAM215B	1.02964272	0.00026874	ZNF319	-1.53571545	0.02822597
IFITM2	1.02943854	7.6245E-21	ITGAV	-1.53548337	2.4466E-19
AC137932.4	1.02848418	0.04339777	NATD1	-1.53482219	0.00164769
BEND6	1.02830755	0.04389785	ETAA1	-1.53222682	0.00485601
G6PD	1.02765804	1.4165E-09	ZNF799	-1.53219393	0.0237312
FANCA	1.02681068	0.00302086	MYO5A	-1.52736313	3.657E-25
CBWD5	1.02680968	9.1811E-07	OSR2	-1.52673054	0.00029713
HGSNAT	1.02455101	7.948E-12	C1R	-1.52456043	0.00746692
SDC2	1.01746503	0.00130766	LPAR1	-1.52343187	0.00111321
LILRB3	1.01736988	1.5776E-08	DUSP1	-1.52172803	4.3593E-18
ZNF487	1.01648507	1.6154E-05	SLC1A3	-1.5195987	5.2177E-08
CTD-3184A7.4	1.01641786	0.0344324	IGHA1	-1.51641798	0.00128487
IFT57	1.01621634	3.774E-07	SLC2A8	-1.51554708	1.9713E-09
TGM2	1.0131503	0.02595499	SLA	-1.51543222	7.9853E-24
TAP2	1.01182816	8.7207E-10	MMD	-1.51533964	7.5396E-12
C7orf50	1.00980444	8.2386E-14	SCN1B	-1.51447301	4.4921E-05
ITPR1	1.00965023	8.1458E-09	ARMC9	-1.51122303	0.03855348
UCHL3	1.00914268	2.3935E-09	THRA	-1.51024397	0.00013328
CCDC69	1.00908018	8.2174E-07	ZFP64	-1.50662297	0.00547434
AFMID	1.00699874	6.4963E-09	STXBP4	-1.50613573	0.03002637
FANCD2	1.00578613	0.01958874	ABCA1	-1.50561127	5.9943E-25
MAD2L1	1.00574895	0.03355832	TBC1D4	-1.50498436	2.3532E-08

HYAL3	1.00374378	0.04315565	IER3	-1.50373665	8.6685E-21
MCF2L2	1.00290671	0.00343241	IL1A	-1.503514	1.2261E-05
GSTP1	0.99912051	5.6154E-14	OSGEPL1	-1.50308341	0.00113553
INSR	0.99416923	5.6362E-09	TBC1D8B	-1.50278231	0.01965929
MAN2A2	0.9930569	7.0189E-06	RP11-350G8.5	-1.50212463	0.02494245
TOMM34	0.9924527	1.1024E-06	ALS2CL	-1.5006425	0.01471892
RP11-231C14.	0.99241709	0.0081432	LBX2-AS1	-1.5003828	0.00891357
MAD1L1	0.99212795	0.00011748	HIPK1-AS1	-1.49979581	0.00090843
RCAN3	0.98904507	4.1001E-06	CTGF	-1.4993118	0.01445398
CCDC12	0.98859637	3.8629E-18	GIN1	-1.49385265	0.00162954
TMEM238	0.98762852	0.01091363	ISYNA1	-1.48916588	1.6949E-07
EXOSC7	0.98671598	1.2902E-08	RP11-44N11.2	-1.48913372	0.02604911
BST1	0.98639271	0.00062649	OPLAH	-1.48719936	0.00016663
TRAPPC1	0.98602029	1.3926E-20	C8orf58	-1.48407932	0.0018491
SERPINB1	0.98473136	6.8677E-10	DAAM1	-1.48394626	2.0553E-07
LRRFIP2	0.98380944	2.6646E-08	ABL2	-1.48331972	9.7232E-26
SNRPF	0.98353432	2.1486E-32	CXCL2	-1.48182455	2.2442E-14
RP11-382A20.	0.98301838	0.00829399	WDFY3	-1.48011204	1.1936E-19
MFSD6	0.9824972	0.00620545	MYLIP	-1.47883586	1.4974E-16
TTC19	0.98242812	1.948E-08	WWC2	-1.47830045	9.1238E-09
UCK2	0.98084186	0.00010305	DNM3	-1.47770354	0.01857712
PHLPP1	0.98016005	0.00035614	RECQL5	-1.47726129	2.4681E-05
CRIP1	0.97782003	1.7355E-07	MAFF	-1.47600862	4.2079E-19
JAK3	0.97747732	0.00020565	ZNF777	-1.4752228	0.00886154
PSTPIP2	0.97716316	5.7346E-08	WDR36	-1.47501589	2.1883E-11
SLC12A6	0.97549229	1.5537E-05	LRRC32	-1.47487569	3.795E-06
CSNK1G2	0.97435564	2.1074E-08	CCDC136	-1.47351528	0.02673101
PSMB8	0.9743407	1.0481E-17	F3	-1.4734582	0.00645045
FAM129B	0.97385631	0.01984746	KCP	-1.47316007	0.01154093
TES	0.97259821	3.5198E-11	TSPAN10	-1.46642848	0.01222025
DFNB31	0.97187619	0.03227728	EMP2	-1.46597308	4.4939E-07
HDAC9	0.97185684	0.00625414	PWP2	-1.4657684	0.00136629
CREM	0.97142738	3.8536E-09	RP13-638C3.4	-1.46538151	1.1478E-05
LUCAT1	0.96905045	0.00886154	CARD6	-1.46312968	2.9615E-07
LYAR	0.96846139	3.3226E-09	TDRKH	-1.46157855	0.01327902
RASGRP2	0.96772907	1.0689E-06	ARMC2	-1.46113584	0.03956667
RFTN1	0.96568873	6.1116E-09	NCEH1	-1.46068747	2.8318E-14
GAPDH	0.96483567	5.2105E-23	NLN	-1.45882097	4.133E-10
HLA-DQB2	0.96483563	0.01243166	DLC1	-1.45852176	0.00202545
HLA-DQB1	0.96283149	3.9587E-12	CCDC170	-1.45656091	1.735E-10
RAB24	0.96208135	0.00013161	MFHAS1	-1.45416392	5.3086E-06
HPS6	0.96134208	0.00741361	C20orf194	-1.451281	6.4422E-10
SPATA13	0.96101317	0.00317448	BOLA1	-1.45033091	4.7752E-05
C14orf80	0.95910272	0.00058407	SOWAHD	-1.44873259	0.00226483
OSM	0.95866013	0.00482966	COLGALT1	-1.44748568	9.1323E-21
IL16	0.95829073	2.3387E-09	EPS8	-1.44371899	6.96E-08

ZC3HAV1	0.9572503	3.9223E-10	HK3	-1.44360082	0.00015905
PRKCH	0.95716574	0.00107957	ZFP90	-1.44348362	6.9991E-05
IL27RA	0.95696625	1.8756E-05	TMEM150B	-1.44299288	0.00010036
LRR1	0.95605603	2.8429E-05	CAPRIN2	-1.44164241	1.8821E-05
MINCR	0.95546723	0.0107646	ANKH	-1.44134176	8.765E-22
PRMT1	0.95522221	1.3945E-23	C1orf112	-1.43625646	0.00531641
GNG7	0.95490601	3.415E-06	GSTM3	-1.43331054	8.3443E-16
SLC25A37	0.95223292	5.2233E-09	GRAP	-1.43035507	0.01248635
ALYREF	0.94892522	1.2186E-12	SLC36A1	-1.4234984	1.1792E-07
ZNF281	0.94718355	2.1398E-07	RP11-196G11.	-1.42310204	0.00170789
ROGDI	0.94484443	1.0095E-05	TMEM51	-1.42229038	3.0079E-14
ACTN1	0.94478771	2.0216E-10	RP11-196G11.	-1.42193909	0.00125716
STX10	0.94372658	1.8963E-14	OSBPL1A	-1.42020451	3.8714E-19
CCDC6	0.94339291	6.2408E-07	ACO1	-1.42011824	1.2114E-08
CSF3R	0.94282256	4.7862E-09	GADD45G	-1.41777948	8.8463E-07
NIPSNAP3A	0.94237259	1.6094E-08	SFXN2	-1.41678822	0.00800541
MAP2K1	0.94182863	7.6539E-21	ID3	-1.41626061	4.1101E-07
IMPDH2	0.94113321	3.4419E-16	RENBP	-1.41497732	1.3089E-09
SRSF8	0.9395936	6.3005E-10	NCK1-AS1	-1.41372988	2.8294E-06
TMEM216	0.93794326	0.00582074	ZFP3	-1.41096814	0.04519911
CD86	0.93632106	5.1331E-14	JCHAIN	-1.40731552	4.0878E-05
LINC00115	0.9352755	0.02502482	PTPDC1	-1.40634932	0.0253748
TLE1	0.93458926	0.02902253	EHHADH	-1.4053824	0.04096918
NAA10	0.93450568	1.2433E-14	ICA1	-1.40456275	3.4614E-06
SLC25A51	0.93385328	0.00097462	SFMBT1	-1.40113496	3.745E-05
PDCD5	0.93266511	3.4635E-17	RP11-54O7.17	-1.39991081	3.9016E-07
CIB1	0.93096183	4.8694E-14	CDKL3	-1.39914294	0.0084187
MTMR9	0.92904966	1.4906E-07	PPP1R3G	-1.39741385	0.00082859
CD55	0.92792334	2.2411E-07	LIPT1	-1.39588878	0.00042588
CBWD2	0.92688327	3.7539E-06	ZNF329	-1.3934346	0.02306058
RP11-325F22.	0.92283718	0.02599136	MSR1	-1.39330952	1.4665E-12
GABBR1	0.9211275	0.00582074	CISH	-1.39271088	0.00253903
LTB4R	0.9199943	0.00053682	ZNF234	-1.38735777	0.03296395
PIGR	0.91800488	0.04324174	CSRP2BP	-1.38662906	0.0056402
GALE	0.91714936	0.0276388	KB-1507C5.4	-1.38568098	3.5891E-06
SFT2D3	0.91694298	7.8138E-06	C19orf68	-1.38452029	0.00273732
MIR181A1HG	0.91606087	8.7715E-14	NR4A2	-1.38431078	9.2651E-15
HSD17B10	0.91580053	4.5009E-11	LMLN	-1.38070118	0.0164655
SRD5A1	0.91427071	0.00296831	CFD	-1.38058771	4.6333E-09
FDPS	0.91362829	1.5047E-18	PIK3IP1	-1.38050757	1.1924E-12
HEBP2	0.91340729	1.9983E-08	ENG	-1.37959925	4.7763E-24
TRABD	0.91207772	3.6455E-10	LSS	-1.37779033	0.00058611
TAF10	0.91131051	3.9464E-15	TCF4	-1.37696421	2.7972E-16
URGCP	0.91124765	0.02552106	SNX33	-1.37612432	0.0006428
CTC-524C5.2	0.9100552	0.01672166	AMN1	-1.37514535	0.00025763
RP4-605O3.4	0.90873237	0.03563554	NPHP3	-1.37413886	2.0399E-06

KYNU	0.90851127	5.2034E-06	ZNF438	-1.37348273	0.00129115
SEMA7A	0.90829074	0.01143512	RP11-48B3.4	-1.3716079	0.03292242
GLO1	0.9073841	2.78E-20	PRSS36	-1.37113392	0.00292353
REC8	0.9061089	9.3757E-05	ZCCHC11	-1.37094374	3.6727E-16
PSMB10	0.90530511	7.9093E-10	P4HA2	-1.37062832	0.04485005
SAMM50	0.90437076	2.7841E-07	YEATS2	-1.36977736	3.2811E-05
RBM34	0.90425354	0.02269524	CCDC146	-1.3695751	7.0571E-07
PAICS	0.90391953	1.1967E-05	KLF10	-1.36766966	2.0842E-23
IL15	0.9026302	9.3283E-05	CXCL8	-1.3669581	2.3716E-07
GNB5	0.90187873	1.5881E-05	RTN2	-1.3606716	1.6254E-10
ZNF296	0.90151748	0.03532068	HSPA6	-1.36027853	0.00840805
ISG15	0.90120905	6.0016E-09	CCL2	-1.36010088	1.3598E-05
NSMCE1	0.90105679	9.1634E-11	MYO1C	-1.35846405	2.5421E-06
MSTO1	0.90053961	0.02603776	GNG11	-1.35832104	0.00537902
POMC	0.90053178	0.00236669	RP11-1017G2:	-1.35828722	0.00022367
RP11-126K1.6	0.90033422	0.00012221	TMEM106B	-1.35639441	1.8752E-09
TRDMT1	0.899451	0.03793676	CPEB3	-1.356389	1.0793E-06
C1QBP	0.89925083	2.2939E-14	CCDC186	-1.35531537	1.4586E-05
CNBD2	0.89924567	0.0010063	APBA1	-1.35128914	0.00108611
RPL5	0.89556439	9.4754E-30	BBS4	-1.34850404	8.5738E-05
DYNLT1	0.89480745	2.3387E-09	ZNF546	-1.34582816	0.01786831
SAMSN1	0.89434674	1.6837E-14	CRAMP1	-1.3451753	0.00153613
ARF5	0.89412662	6.7804E-14	ZFP41	-1.34490229	0.04477754
SLC41A2	0.89391269	0.00662838	PLXND1	-1.34344726	1.4188E-06
PTRHD1	0.89358089	4.2545E-20	DCAF12	-1.34343916	5.8261E-13
NME2	0.8935361	7.3045E-05	RASA1	-1.34340536	9.1288E-07
EMP3	0.89279832	9.0163E-19	ZFHX3	-1.34226921	8.8156E-39
RPL10A	0.89229289	7.038E-27	ADRA2B	-1.34219084	0.00078291
CD7	0.89162524	0.00127954	ZNF585B	-1.34119924	0.02659374
RPL4	0.8914777	3.6214E-39	NXN	-1.34100852	0.03331242
DPP3	0.89060816	1.8172E-05	TMEM255B	-1.33969113	3.4464E-06
HMGN1	0.88989273	5.7295E-14	TMEM86A	-1.33891182	4.0614E-06
ANXA2	0.88981068	3.3827E-09	SGPL1	-1.33680379	7.5341E-10
RP5-832C2.5	0.88966287	0.02458836	FRMD4B	-1.33675657	2.9977E-21
FAM89B	0.88767713	1.4132E-08	TP53I11	-1.33655166	0.00040457
GABARAPL2	0.88590642	5.6935E-15	PLEKHO2	-1.33474205	2.0784E-17
EXOSC5	0.88357238	5.2112E-10	KIAA0753	-1.33421134	0.02108706
VIM	0.88330427	2.1173E-07	POU2F1	-1.33414071	1.3994E-10
CNN2	0.88320476	3.1092E-14	FAM135A	-1.33145717	4.2131E-06
FCHO1	0.8828269	0.01713089	C10orf88	-1.33048547	0.02929348
JARID2	0.88274513	9.0917E-09	ARMCX2	-1.32976153	0.00047356
GHITM	0.88269269	1.0775E-21	HSPH1	-1.3293494	2.8854E-07
NHP2	0.88260949	1.8493E-20	FAM198B	-1.3274306	1.0999E-13
DCPS	0.88211927	6.81E-06	CD163	-1.32621145	7.4282E-11
MIR3945HG	0.88132755	0.00821074	POC5	-1.32565345	0.00013779
EIF4E3	0.8805493	0.00739428	ZNF398	-1.32563515	0.01312296

EEF1E1	0.8794782	0.00010271	GCLC	-1.32515582	9.3174E-14
EIF5A	0.8791125	1.3072E-21	ZNF527	-1.32512083	0.02308178
ECHDC1	0.87898866	4.5278E-09	RP11-160E2.6	-1.32436625	0.00440843
MX1	0.87884669	5.6851E-05	C5AR2	-1.32405776	7.5473E-14
XPNPEP1	0.87823843	4.974E-07	KLF4	-1.32402043	7.9929E-09
CLIC1	0.87769619	6.1973E-19	LDLR	-1.32270691	9.1286E-16
RPL18A	0.87742885	4.6358E-25	PGM2L1	-1.3218939	4.5663E-05
CATSPER1	0.87596181	0.02882108	PXN	-1.32028123	4.8825E-09
ADA	0.87580312	0.00015589	SERPINF1	-1.31851743	1.4372E-14
SFI1	0.87546847	0.02940553	H1F0	-1.31650277	8.8029E-17
TMSB10	0.87446247	5.7899E-23	TRPM2	-1.31471746	1.029E-06
DNMBP	0.87428306	0.00015948	TGFBI	-1.31462514	4.0265E-35
NCF2	0.87415908	1.7116E-15	RASA3	-1.31375382	1.4643E-07
NELFCD	0.87382143	2.1414E-06	LIPA	-1.31306267	9.1437E-22
B3GALNT2	0.8732101	0.0018289	ZNF205	-1.31194829	0.00150386
PDE4A	0.87298885	2.9097E-06	CTD-3088G3.8	-1.31172385	0.03380927
NRM	0.87266258	0.00353981	ITM2C	-1.31083526	5.154E-37
RPL8	0.87117279	5.2086E-30	ITPKC	-1.31029502	0.00114948
ILF2	0.87070009	7.9937E-24	HNRNPU-AS1	-1.30991958	1.6986E-08
SSX2IP	0.87043665	0.02188295	SIPA1L2	-1.30900863	1.3147E-09
C11orf1	0.8693928	0.00018892	AKAP10	-1.30335289	1.3322E-06
EIF6	0.86922537	2.7779E-22	MICA	-1.30323732	0.00017982
GABPB1-AS1	0.86865656	9.2422E-09	FRS2	-1.30264501	1.7006E-06
RPS7	0.86457362	1.592E-27	SMTN	-1.30121877	0.0422572
RPL6	0.86421075	8.8268E-36	TPD52	-1.30053403	1.0041E-06
LINC01089	0.86368612	0.03314598	IRAK1BP1	-1.300526	0.02431433
C17orf49	0.86363596	0.00100199	UHRF1BP1	-1.29914592	7.7386E-07
ZNF580	0.86320551	0.00010633	CBLB	-1.29702024	1.3888E-06
DGKE	0.86251896	0.04219554	UACA	-1.29619679	9.482E-06
BCR	0.86188192	0.01772456	PLIN2	-1.29465712	2.6302E-25
WDR54	0.86184316	0.00131801	ZBTB10	-1.29390487	9.4826E-09
ASGR1	0.86154974	0.00247861	PLEKHA1	-1.29276937	4.2328E-17
PNMA1	0.86136797	0.02065962	AP1B1	-1.28973684	1.7457E-22
AEN	0.86047037	0.00011119	PEAK1	-1.28933977	1.3831E-08
PGP	0.86025811	1.0782E-09	TRIM14	-1.28710272	2.7207E-14
SBNO1	0.85945085	2.2375E-09	SLITRK4	-1.2864951	0.02538236
UIMC1	0.85880195	0.00012071	BTG2	-1.28601139	1.0031E-14
APOM	0.85876063	0.03914895	ALDH7A1	-1.28451038	8.8493E-06
NFAT5	0.85631435	0.0055239	EXTL2	-1.28405806	7.2231E-05
FXYD5	0.85576263	8.2788E-14	TMEM106A	-1.28365152	7.8611E-15
KCTD20	0.85546532	1.1263E-08	ACVRL1	-1.28304218	4.8725E-15
CARD16	0.85534874	1.5167E-25	ZNF623	-1.28035985	0.04517842
ALDH2	0.85514425	2.4753E-07	ACVR2A	-1.27947567	0.00537273
LMNB2	0.85473794	0.00357913	ZNF260	-1.27660449	0.00085808
PTS	0.85454616	9.3317E-08	ADM	-1.2748083	8.7904E-12
COMMD8	0.85453023	5.2151E-11	RNF122	-1.27284395	1.7043E-05

ST20	0.85405748	0.02422574	PTGFRN	-1.27220081	1.5563E-06
EIF3I	0.85341813	1.0918E-18	PDIK1L	-1.27206436	0.04358175
EEF2KMT	0.8529047	0.0441289	SNX24	-1.27154312	4.2861E-05
ZBTB43	0.85165408	2.5729E-06	POLR3A	-1.2707623	0.00115123
SNRPB	0.85140699	1.2745E-25	ARHGAP35	-1.27031366	7.1669E-06
DCAF13	0.850918	2.1787E-07	CD84	-1.2701577	5.5726E-10
RP11-473I1.5	0.85040194	0.03394861	SESN1	-1.26999723	5.309E-09
SAMHD1	0.84977048	6.6197E-17	ZMYM1	-1.26959146	0.00011829
HAAO	0.84823699	0.00056057	C5orf42	-1.26907169	1.9377E-05
PSMD2	0.84813318	8.4935E-17	CPNE2	-1.26890839	1.4247E-05
GBA2	0.84676759	0.00242781	NFKBIZ	-1.2687497	3.5167E-20
PARK7	0.84631552	7.4505E-24	IQCG	-1.26836505	7.0154E-06
PTPN22	0.84588727	0.00038497	WDR19	-1.26754693	0.04887469
ATP5G1	0.84409097	1.1853E-19	OTOA	-1.2657934	0.00435828
FDFT1	0.84217475	7.0178E-09	BMPR2	-1.2651504	2.8467E-16
CLEC4A	0.84207029	1.8762E-14	ZNF510	-1.26512207	0.03364583
RNASEH1	0.84152205	2.878E-07	LRP5	-1.26479765	0.00017068
GBP4	0.84128393	1.1566E-11	TFCP2L1	-1.26206581	0.04909366
LSM7	0.83888407	3.1595E-32	NR1D1	-1.26199583	0.00038169
TMEM170B	0.83824765	0.00072153	AP006222.2	-1.25994365	1.1024E-06
EML2	0.83814844	0.0026188	AXL	-1.25926557	5.4367E-18
GALR2	0.83789067	0.00727908	THNSL2	-1.25693489	0.00401511
HDAC3	0.83682002	1.6904E-09	CSF1R	-1.25555264	2.9053E-31
TRERF1	0.83681988	0.04038133	TMIGD3	-1.25437974	5.2739E-06
LINC01116	0.83632359	0.00285723	FAM193B	-1.25379859	0.00016336
PTP4A2	0.8362586	4.5944E-29	GPD1L	-1.25238952	0.0005978
DIAPH1	0.83582424	2.8218E-10	KRBA1	-1.25128006	0.01834833
ACTB	0.83560914	8.427E-26	MARCKS	-1.25038649	9.0606E-30
RPS18	0.83538662	2.2676E-18	P3H2	-1.24902739	0.00933636
TNFAIP8	0.83285419	0.00119771	FAM72D	-1.24900202	0.00280257
DGKA	0.83258889	0.00025504	MANEA	-1.24829828	4.0789E-06
RP11-138A9.2	0.83252117	3.2912E-05	NR4A1	-1.24736992	1.6387E-11
WDR43	0.8322044	8.1329E-11	ZNF571	-1.2463032	0.03109723
CCNB1IP1	0.83215251	9.0751E-08	CTD-2350C19.	-1.24564003	0.02232313
PTPN1	0.83087525	1.5235E-05	ZNF775	-1.24332258	0.04294558
ADSS	0.8306344	1.514E-09	SCD	-1.24331143	0.00214873
ATIC	0.83053347	1.0924E-06	CCDC149	-1.24324656	6.9197E-05
C16orf91	0.83016678	3.6764E-05	PELI1	-1.24041294	6.0314E-12
CLP1	0.82943999	0.01045998	PLA2G4A	-1.23901491	8.3903E-05
MOB3B	0.82915799	1.9598E-05	ZCCHC24	-1.23880095	4.9841E-05
PSMA2	0.82913575	3.5547E-22	TLR5	-1.23783687	1.6752E-12
VBP1	0.8289873	1.2122E-07	PAFAH2	-1.23597091	0.00693918
MPHOSPH6	0.82750227	1.9155E-07	ZBTB4	-1.23342484	4.9946E-09
EPB41	0.82726103	0.00122763	BACE1	-1.23256592	3.9741E-06
TXNL4A	0.82674938	6.4878E-12	DLEU7	-1.23115553	0.00042161
MARCKSL1	0.82630603	2.4862E-06	RBMS2	-1.23113172	4.8864E-09

YRDC	0.82529247	0.0013383	CD93	-1.23012398	1.8675E-18
MYL12A	0.82354455	7.3376E-15	SNX18	-1.22811128	7.2085E-09
ACOT9	0.82248347	1.2258E-13	CD81	-1.22804734	1.5983E-13
METRNL	0.82207082	4.2375E-07	C16orf70	-1.22668782	2.0323E-05
DDX28	0.82140071	0.00665555	P2RY1	-1.22664162	0.00928719
FBL	0.82092428	6.0535E-14	TMCC1	-1.22602515	3.7686E-05
AMPD2	0.81916863	6.7882E-13	KIAA1147	-1.22583035	0.00025565
EIF3L	0.81884979	2.0161E-19	IGHG1	-1.22577319	0.04640646
BANP	0.81831492	0.00033104	C15orf41	-1.22530609	0.02569122
GLRX3	0.81743559	1.4904E-09	GCLM	-1.22434926	2.9518E-05
MOB3A	0.81709962	5.1031E-07	CLEC1A	-1.2231193	0.00708314
OXSR1	0.81663118	0.00010638	IER5	-1.22188068	5.8937E-13
GNA12	0.81610732	8.0302E-08	B3GNT7	-1.22092753	5.315E-06
PLSCR1	0.81597977	6.5039E-13	WDR24	-1.21947254	0.0207942
CPD	0.81393175	0.00488602	RP11-379F4.9	-1.2180499	0.03238486
DGKD	0.81363917	0.00060457	PPFIBP2	-1.21691954	2.3861E-08
C19orf38	0.81356232	7.5758E-10	CALML4	-1.21667185	0.00691782
PSME1	0.81321045	4.5553E-23	CRACR2B	-1.21547196	0.00280943
RAB4B	0.8130969	6.7919E-08	ZNF185	-1.21431955	0.00084263
RCE1	0.81261463	0.00734697	TTYH3	-1.21285956	6.6134E-13
ANAPC15	0.81243731	1.1792E-07	TMEM135	-1.21091393	0.00012908
ZNF703	0.81160653	0.00975412	SLC2A13	-1.21013656	1.7468E-07
SNAI1	0.81147781	0.00111616	CELA1	-1.20705684	0.04754005
RP11-356I2.4	0.8105831	0.00044324	ABCG1	-1.20643018	7.5099E-09
MAN1B1-AS1	0.81013604	0.01747808	BIVM	-1.20618839	0.02083446
GPN1	0.80981252	2.6388E-06	TTC7B	-1.20430663	0.00653853
CDC42EP4	0.80908529	0.02067611	DZIP1L	-1.20420734	0.00495137
RP11-405M12	0.80866256	0.04965638	C3AR1	-1.20296697	8.8849E-19
NACC2	0.80813469	0.01248723	DOPEY1	-1.20166828	0.00028549
IFT20	0.80738721	2.8086E-09	ICAM1	-1.20162043	4.1013E-12
FURIN	0.80600416	0.00056917	LXN	-1.20067263	0.00146233
CREB3	0.80376199	2.641E-06	HSPA2	-1.20011614	1.439E-05
NDUFS8	0.8034721	4.2983E-20	AC109826.1	-1.19913857	4.7714E-06
FRAT2	0.80290754	1.2747E-06	DNASE1	-1.1946653	0.04120183
FEN1	0.80131826	0.01662548	NMNAT3	-1.19376662	0.00366467
DUSP22	0.80070684	3.5856E-09	MKNK1	-1.19352148	4.7303E-16
GBP2	0.80038913	9.2317E-06	ULK1	-1.19275324	1.765E-05
DDX49	0.79986329	2.0215E-05	ZSCAN9	-1.19004861	0.01365953
ATP5A1	0.79947752	5.5252E-24	NFE2L1	-1.18935956	4.2083E-12
RPL3	0.79910588	5.2111E-22	PALD1	-1.18919291	3.5081E-08
PRKAG2	0.79899598	9.6174E-09	KIFC3	-1.18686855	0.01034889
GNB2L1	0.79869441	5.7676E-20	ZFP36L2	-1.18663403	5.6873E-08
ADSL	0.79783618	3.7021E-07	FLYWCH1	-1.18662803	0.00010849
BZW2	0.79740649	3.8617E-09	PYGO2	-1.18647918	0.00178635
AP1G2	0.79680494	0.01458277	ODF2L	-1.18570234	8.2461E-09
CARD19	0.79636302	3.654E-08	TBCK	-1.18505805	0.00012617

DESI1	0.79529429	1.9473E-05	AC005082.12	-1.18333496	0.04082446
NOC2L	0.79511735	1.713E-07	MACC1	-1.18315052	0.00133347
GTF2E2	0.79451887	0.00034487	FBXL4	-1.18103094	0.00038401
CCSER1	0.79428325	0.02974197	FBXL20	-1.17922337	8.2659E-07
LAP3	0.79377944	4.4441E-19	KCTD12	-1.17839984	4.0061E-15
PPIH	0.79262587	2.9164E-09	ADIRF	-1.17831026	0.0105332
RUNX3	0.79212304	5.3243E-06	GBGT1	-1.17757249	5.9631E-07
APRT	0.79149478	1.537E-22	MRC1	-1.17745121	1.8328E-23
TBCC	0.79125117	0.000576	VAT1	-1.1769099	5.6628E-05
SUPT4H1	0.79073583	5.4973E-16	MGAT5	-1.17491531	1.7536E-06
TP53BP2	0.7903496	5.6964E-06	SLC29A3	-1.17458617	0.00025123
ABT1	0.7901683	2.5453E-05	S100A13	-1.17413895	4.0741E-10
METTL22	0.78998889	0.00055746	FUT10	-1.17322391	0.01383954
TBC1D7	0.7899127	0.00059169	GKAP1	-1.17152936	2.4942E-05
NDUFA7	0.78927045	2.2916E-08	MYL9	-1.17130507	0.02047438
SNX10	0.78884239	4.0291E-05	SP4	-1.17013025	0.00624288
GTPBP4	0.78880327	1.3006E-10	HKR1	-1.16753521	0.00730369
FIBP	0.78783298	7.8487E-17	EBI3	-1.16626658	1.4384E-08
CEP68	0.78765487	2.7375E-07	POMT1	-1.16518306	0.00074265
RPS5	0.78731636	2.5687E-22	CPEB4	-1.16498609	4.1298E-16
TEX30	0.7869373	0.00263767	TMEM236	-1.16470705	2.4144E-05
PLCXD1	0.78678103	0.00176081	TLR6	-1.16424677	6.9598E-05
SHKBP1	0.78569593	1.6492E-08	ADRB2	-1.16287815	1.3095E-06
LY6E	0.7852563	2.9452E-12	MAST3	-1.16279736	5.4832E-05
RPSA	0.78516429	3.0072E-27	ADAM9	-1.16277054	3.4559E-11
TRIAP1	0.7842294	2.5105E-05	HTR7	-1.16135761	0.00029133
SORT1	0.78377392	0.00268128	SMARCAD1	-1.16025892	2.1251E-06
RP11-140K17.	0.78305768	0.014869	PINK1	-1.15988306	8.591E-12
CYP2S1	0.78269947	1.3674E-05	NPAT	-1.15975491	1.0822E-06
RP11-295G20.	0.78233276	0.00357698	MED22	-1.15933724	0.00122737
ADD3	0.78229165	0.00026274	TXLNB	-1.15748294	0.01326113
KCNAB2	0.78183271	8.1079E-05	RIC8B	-1.15469343	0.01700946
UBE2I	0.78168267	4.1328E-15	GRPEL2	-1.1538279	0.02250714
DUOX1	0.78146729	0.04434375	FAM114A1	-1.15169513	0.00243518
RPL29	0.78133618	4.3841E-19	ARHGAP18	-1.15128301	5.6432E-16
SDE2	0.77995952	8.3247E-10	SNX6	-1.15024433	8.6174E-21
FBXO34	0.77961756	1.1973E-05	USPL1	-1.14914579	8.3088E-05
TKT	0.77932737	2.2186E-13	LOXL3	-1.14872789	0.0002447
EIF3M	0.77924822	3.4219E-27	SORBS3	-1.14768612	1.0003E-11
PFN1	0.7788248	3.4859E-21	EIF4A3	-1.147118	1.1471E-17
HINT1	0.77840878	5.2199E-10	MS4A4E	-1.14663731	0.02762763
DECR1	0.77797713	7.5541E-10	BBS10	-1.14562247	0.02182231
MDH2	0.77785659	7.538E-19	TIMP2	-1.1433209	5.2357E-15
IDH3A	0.77581932	2.6389E-05	CHST15	-1.14079109	9.2838E-07
ZNF791	0.7750743	1.3849E-05	RCAN1	-1.13996869	1.7153E-08
RPL32	0.77505773	9.0405E-22	SLC19A2	-1.1395504	1.739E-12

MIF	0.7749363	4.4953E-07	ZSCAN21	-1.13681638	0.030017
CD58	0.77327929	1.6131E-05	MAGEH1	-1.13679488	4.7533E-05
MRPL47	0.77295734	1.8036E-09	RB1	-1.13671065	5.7321E-28
CDKN2AIPNL	0.77255721	0.00083303	CHST7	-1.13455841	0.00425862
CCT7	0.77093828	2.1495E-14	NCF4	-1.13201885	3.3333E-38
DENND1B	0.77065938	2.4418E-07	TGFBR2	-1.13091001	1.9012E-12
SHB	0.76964462	0.0006607	TMEM91	-1.13037584	0.00123933
PSMB6	0.7695182	1.108E-20	SLC11A2	-1.1302437	4.0588E-11
ARL17A	0.76902432	0.00702236	MMP25	-1.12951222	0.00013104
NCBP2-AS2	0.76793091	2.7506E-05	MEGF8	-1.12852911	0.00313439
COMMD6	0.76657053	7.9309E-16	TP53INP2	-1.1278427	3.1383E-10
TALDO1	0.76652858	5.6875E-15	ZBTB38	-1.1277128	5.8598E-15
TMEM38B	0.7657918	0.00060505	TRMT1L	-1.12755412	5.0774E-06
DUSP14	0.7656058	0.03764394	DAG1	-1.12690262	0.00013984
PGK1	0.76507328	1.8171E-20	ZNF347	-1.12657607	0.00806012
SIK3	0.76470121	2.9339E-05	PDE1B	-1.12599862	0.00822624
NDUFA6	0.76376924	2.069E-12	TSC22D3	-1.12494565	6.7412E-14
UBALD1	0.76324971	0.0082689	TULP3	-1.12365788	0.00630731
RAN	0.76316073	1.6672E-27	RHBDD3	-1.12352891	0.00090327
RPL31	0.76314859	1.3222E-17	DENND4C	-1.12337373	1.0041E-07
RPL11	0.76225456	6.1734E-25	LPAR6	-1.12330971	7.1252E-23
RP11-182L21.(	0.76222491	0.0074798	GHDC	-1.12293259	6.7588E-08
MAML3	0.76213278	4.6747E-06	UBE3D	-1.12187232	0.04740586
NUDT1	0.76138506	3.2079E-09	IFT46	-1.12166768	0.00958784
LY75	0.76072675	0.0006747	NNT-AS1	-1.12008529	0.00656059
RPL37	0.75947539	5.3845E-33	RP1-34B20.21	-1.11995674	0.03943469
ATP5B	0.7574774	1.7863E-19	PAQR7	-1.11978156	0.00049589
MTCH2	0.75495262	1.9466E-08	CTSB	-1.11943378	1.3109E-22
RNH1	0.75471986	1.0554E-11	RAB11FIP3	-1.11926289	0.02199891
UBE2F	0.75415889	6.8567E-10	FBXO31	-1.11854621	0.02009744
GTPBP8	0.75415558	0.0011082	HS1BP3	-1.11831431	0.00011153
FAM49A	0.7538321	2.3304E-05	UAP1L1	-1.11802848	1.6448E-05
PSMA4	0.75382732	1.0902E-14	HIST1H2BG	-1.11794971	0.03921262
HMBS	0.75375015	0.00294349	CD3EAP	-1.11781488	0.00032914
ANP32A	0.75349662	7.1039E-13	CD59	-1.1170281	3.5355E-08
CTSH	0.75297674	6.2336E-10	FAM65A	-1.1163597	1.6319E-07
ARPC1B	0.75295988	4.5325E-15	ZNF559	-1.11610329	0.00783649
CTD-2186M15	0.75266378	0.04678188	SEMA4C	-1.11445171	1.2506E-05
AGTPBP1	0.75249965	5.3833E-05	PRNP	-1.11345678	2.0474E-21
CCT8	0.75230874	8.3085E-25	DBP	-1.11294172	7.8672E-06
BAK1	0.75226057	0.00010825	ARID3B	-1.11137636	5.6666E-05
PNPLA4	0.75194322	6.5443E-05	NHLRC2	-1.11099	2.5131E-06
TOMM40	0.75172685	7.8655E-08	MICB	-1.1103848	0.00276829
TSHZ1	0.75151823	1.6844E-05	ABHD11	-1.1095932	7.6735E-05
TFDP1	0.75137843	1.2255E-06	SCAMP1-AS1	-1.10872111	0.00157162
RPIA	0.75115254	5.6344E-06	MOB3C	-1.10849075	1.3279E-05

ALCAM	0.74923611	6.9911E-06	PACERR	-1.10703208	0.04984747
CDC37	0.74810465	3.5264E-09	CH507-9B2.5	-1.10647689	0.00367009
HADHA	0.74727831	3.1641E-22	TCF7L2	-1.10617426	3.8216E-12
NIPA2	0.74668504	6.7333E-06	CYFIP1	-1.10557368	6.7601E-14
RP11-83A24.2	0.74663239	0.03700211	BCL2L1	-1.10501871	0.00865566
EXOSC6	0.74648667	7.5434E-10	NFATC1	-1.10450639	1.6475E-11
SMDT1	0.7458439	7.2857E-16	GNPDA1	-1.10398445	2.113E-15
EIF3K	0.74505662	1.7875E-23	TNFRSF1A	-1.10186964	2.2839E-08
NUTM2B-AS1	0.74481086	3.4673E-05	SEPSECS	-1.10136836	0.00979464
BRK1	0.7441641	9.1361E-24	CCR1	-1.10101573	4.5213E-09
RAD51C	0.74397783	1.342E-06	HSPA5	-1.10068829	4.8234E-12
SPINT1	0.7438993	0.00089149	EIF5A2	-1.10055031	0.00821708
MSI2	0.74360817	0.00270809	CPT2	-1.0993851	0.00149403
USP3	0.74352854	1.4518E-09	CTA-228A9.4	-1.0993387	0.04761666
LSM5	0.74337036	1.9901E-15	ZCCHC14	-1.09874847	3.3875E-05
SLC25A5	0.74290937	5.2687E-24	GADD45B	-1.0985167	3.2616E-12
RPL19	0.74011971	1.4487E-22	DAPK1	-1.09636471	3.8608E-24
RPS14	0.74008527	3.7937E-14	ANKMY2	-1.09618052	0.01439406
RBM8A	0.73960853	2.7792E-18	CHST13	-1.09534183	0.03238486
PRKAR2B	0.73906714	0.00029772	TRA2B	-1.09507523	7.6211E-10
RPS2	0.73883091	5.4325E-12	ITSN1	-1.09498205	1.2514E-07
TSTD1	0.73802079	3.6943E-05	DCUN1D4	-1.09430136	0.00660681
FAM43A	0.73734504	0.00035901	MMP9	-1.09409979	5.7461E-06
TRAP1	0.73712372	3.4246E-05	NAIP	-1.09288482	1.2525E-22
OLA1	0.7368648	2.5341E-07	TEFM	-1.09165182	7.4852E-05
RNASEH2A	0.73598736	0.00021109	ZNF28	-1.09089566	0.01123755
NSMCE3	0.73594496	4.5399E-05	SCARB1	-1.09005272	2.0192E-08
DNAJC4	0.73593218	7.83E-10	MT1M	-1.08990301	0.03956293
UQCRFS1	0.73577964	3.8412E-15	P2RY11	-1.08989719	0.04777994
RNF126	0.73568583	1.6314E-05	RBM41	-1.08946755	7.7546E-05
TBC1D8	0.73559019	0.00135337	CASP8AP2	-1.08908275	0.00034279
RNF7	0.73441245	2.561E-12	PRKD2	-1.08794391	0.04065972
KLF13	0.73407052	0.00019248	EXOC6B	-1.08690237	0.0001029
RPLP0	0.73393153	2.9762E-18	DSC2	-1.08645703	2.4487E-09
RPL7A	0.73386944	8.2657E-19	FGD3	-1.08556886	9.4614E-10
SPINT2	0.73324891	3.4138E-12	F8	-1.08536318	0.00081875
GPR35	0.73320237	0.00208897	ZNF821	-1.08286152	0.00257417
SPRED2	0.73266012	0.03321753	PMP22	-1.08199498	1.0888E-17
RANBP1	0.73249295	2.9895E-13	ZSWIM6	-1.08139651	8.6858E-11
ALDOA	0.73230857	5.8097E-17	ZBTB37	-1.08105801	0.00257693
SNX17	0.73221956	1.0607E-13	P2RY12	-1.08014067	0.01167258
NACA	0.73136147	2.4922E-26	SLC43A1	-1.0776387	0.02237899
AKNA	0.7313064	0.00012032	ZC3H6	-1.07643095	3.1786E-07
RPS21	0.73105903	9.8583E-33	NCOA7	-1.07452157	7.8185E-08
MLLT4	0.73094	0.00098754	ATOH8	-1.07421216	0.03831818
DAZAP1	0.73058472	5.6785E-09	DIP2A	-1.07370396	1.9394E-08

SPRTN	0.73012115	0.00057962	STX2	-1.07308286	2.1007E-06
RP11-37B2.1	0.72999236	0.02487659	MGAT4A	-1.07304208	1.6017E-12
EEF1B2	0.72858755	8.6922E-22	NTAN1	-1.07245788	7.5485E-18
FKBP1A	0.72814025	1.0688E-14	ZNF227	-1.07095398	0.00604971
PFDN6	0.72752606	5.1821E-09	KLHL26	-1.07051728	0.02966567
RPS19	0.72731361	2.8136E-18	EEF2K	-1.0703033	2.7165E-07
NUSAP1	0.72686211	0.01516198	MAP1LC3A	-1.06970866	0.00156518
RPS4X	0.72661963	4.9305E-17	CLDN23	-1.06875606	0.04571347
C20orf27	0.72493696	1.2266E-17	SPATA5	-1.06797164	0.00176602
RABIF	0.72453206	1.7186E-05	TIPARP	-1.06797092	2.1688E-11
BLOC1S2	0.72390611	4.1878E-09	IL6ST	-1.06766822	2.7207E-14
CAPG	0.72361545	0.00050398	ARMC5	-1.0665361	4.1691E-06
EIF4E2	0.72310833	1.2234E-07	DNMBP-AS1	-1.06594723	0.00846285
HNRNPAB	0.72301199	9.5869E-12	DNAJC16	-1.06578973	5.2896E-05
ISOC1	0.72270735	0.00348303	CPED1	-1.0655334	4.899E-10
H3F3A	0.72210815	5.4097E-12	ZNF577	-1.06539165	0.01108434
HPRT1	0.72176651	6.0614E-11	TGFBRAP1	-1.06536555	9.1119E-05
TYMP	0.72097271	3.6437E-19	DYRK3	-1.0646884	0.00285971
SNRPA	0.72096393	1.1444E-05	NFIC	-1.06466108	1.7496E-12
AP1M1	0.71976202	9.8853E-05	DGKH	-1.06098813	0.00028596
PSMC3	0.71931479	9.0281E-10	ZNF765	-1.06077445	0.02623357
EDF1	0.71911776	2.0258E-28	TNS3	-1.06064141	3.1364E-09
KIAA0922	0.71900853	0.01173696	FMN1	-1.06045717	2.5202E-10
SKP2	0.71894865	0.03427693	UNC13B	-1.0601008	0.00916582
CCNDBP1	0.71862549	1.0812E-09	CH25H	-1.05964648	0.00016293
VARS	0.71821509	0.00063672	PLCB3	-1.05886589	8.655E-06
GNA15	0.71757163	4.264E-07	AC083843.1	-1.05815407	0.0121457
RPL14	0.71712223	2.8913E-24	TMEM263	-1.05807413	0.00018242
C10orf128	0.7169105	8.761E-05	TNFRSF21	-1.05736608	3.8747E-13
PIP4K2A	0.71601997	4.301E-09	MPP5	-1.05413961	0.00026164
ATP6V1B2	0.71588084	1.4863E-07	ZFAND5	-1.05364361	4.5553E-23
SNRPD1	0.71574869	1.5863E-11	TPM1	-1.05246687	2.2755E-09
ZNF830	0.71530347	0.00014817	HELQ	-1.05089251	3.9415E-06
CTNNBIP1	0.71527811	0.00039599	KIAA0355	-1.05032008	9.4993E-07
NOCT	0.71494768	0.02339124	TPST2	-1.04978286	6.5221E-10
CLNS1A	0.71485429	1.2192E-10	ZNF18	-1.04873143	0.03907298
SERPINB8	0.71475183	0.00060958	TERF2	-1.04770993	2.2631E-05
CFL1	0.71422729	6.7509E-21	PIBF1	-1.04682291	9.1119E-05
TIMM10	0.71416749	4.2808E-07	ZFYVE1	-1.04586478	0.00161394
XRCC6	0.71353364	1.4404E-16	PTPRO	-1.04381112	0.02242831
POR	0.71331489	6.0392E-08	IFT74	-1.04351663	0.00016784
ABCE1	0.71297464	1.4357E-07	TANC2	-1.04077955	0.01301257
PSMB2	0.71281397	1.9336E-09	ZDHHC21	-1.04028584	0.00012376
EEF1G	0.71096618	0.01026811	CAMKK1	-1.03900553	0.00017509
TMEM164	0.70929324	0.0337887	ZNF444	-1.0372967	0.01830224
TRAPPC6A	0.70892727	3.8924E-08	CYP2U1	-1.03711046	0.00051598

MYO1E	0.70794031	0.0251102	ERRFI1	-1.03659887	0.01877324
PNO1	0.70774439	7.6676E-07	APC	-1.03647191	9.6579E-07
FAM60A	0.70770442	6.3518E-06	KLHL15	-1.03633005	2.1932E-05
MTHFD2L	0.70753558	2.6442E-05	FAM188A	-1.03575681	0.00732033
CHPT1	0.70751617	2.3581E-07	PPARGC1B	-1.03398036	0.00261204
PDHB	0.7074605	4.5647E-05	AKAP11	-1.03389784	1.0298E-05
POLR2B	0.70707596	7.7442E-07	CDC42EP1	-1.03278651	0.03725371
PTPRC	0.70684645	4.3144E-27	HINT3	-1.03251627	8.3105E-07
TBCB	0.70680299	2.0519E-10	ST6GAL1	-1.03172324	1.0669E-08
MICAL1	0.70672539	7.4575E-06	KIAA1033	-1.03077617	2.3984E-13
MCM7	0.70650298	0.01458843	SMAD1	-1.0304288	0.0255827
RPS8	0.70621166	6.9143E-16	RPH3AL	-1.02971158	0.04475543
APOBEC3G	0.70531099	0.00602414	CMB9-22P13.	-1.02891669	0.00129157
SRRT	0.70467364	0.00220122	SAP130	-1.0284056	0.02519793
PPP1R14B	0.70438929	2.2188E-08	CYTH4	-1.0277415	1.0363E-08
MSN	0.70412716	3.877E-10	CHORDC1	-1.02674987	2.0519E-10
CHCHD2	0.70395009	8.8918E-23	PLEKHA7	-1.02674813	0.01856638
CPNE1	0.70325558	2.9283E-10	ALKBH8	-1.02658007	0.02710122
PHPT1	0.70322427	4.9844E-09	TMEM204	-1.02655022	0.03250987
AP2A1	0.7030872	3.3679E-05	GOPC	-1.02638219	0.0001056
MVP	0.70261761	3.4464E-10	BRD8	-1.02483791	4.5839E-05
ATP5G3	0.70250355	3.1049E-13	CREBRF	-1.02471353	8.8058E-18
EZR	0.70166304	9.4614E-10	HSPB1	-1.02441468	1.1052E-07
SNHG21	0.70081174	0.03271938	PGM3	-1.02411122	0.00016867
H2AFY	0.70074115	1.2331E-10	NUDT12	-1.02384548	0.0121864
PIM2	0.70040314	9.2836E-06	FAM134C	-1.02370434	0.00012893
ERI1	0.70037585	0.02882541	TEX10	-1.02335292	1.666E-05
PSMB3	0.70008867	1.0688E-14	ZYG11B	-1.02264494	3.2134E-06
INSIG1	0.69882965	1.7644E-07	TECPR1	-1.02256306	0.00010706
SEC61B	0.69859922	5.2393E-15	NLK	-1.02221804	0.00209171
HINT2	0.69849311	5.2823E-06	SLC26A11	-1.02188728	6.0218E-05
THBD	0.69848246	0.04445668	HSD3B7	-1.02155221	0.03783547
TOR2A	0.69780596	3.8829E-05	TCEAL3	-1.0214804	4.4699E-09
RPL23A	0.69777683	1.3443E-26	TM7SF2	-1.02113988	0.02947189
TAPBPL	0.69709787	0.00146048	MAML2	-1.02083644	1.0424E-08
CCT6A	0.69692606	8.7715E-14	RGL2	-1.02075877	3.2756E-10
RBM7	0.69668135	3.9792E-09	FBXO44	-1.02053834	0.02625695
MEX3C	0.69654496	0.01355806	FLJ31104	-1.02011872	0.03912802
CDCA7L	0.69636566	0.02552555	CEP57L1	-1.01984917	0.00075101
NSMF	0.69600452	0.01721818	FEZ2	-1.01894256	1.829E-25
MED19	0.6956902	0.00012781	INPPL1	-1.01787145	5.4972E-09
FAM58A	0.69517093	0.00154086	ADGRG5	-1.01659266	0.0002336
EIF3C	0.69492502	0.00900153	SRGAP3	-1.01568623	0.00052432
HIVEP1	0.694412	0.03252391	ID1	-1.01487285	0.00538698
CCT5	0.69399104	4.5609E-11	RASGEF1B	-1.0147607	5.8261E-13
PRKCB	0.69384526	3.0026E-06	AGAP4	-1.0147336	0.02814853

FBXO33	0.69380928	9.6433E-05	ZNF611	-1.01471162	0.01698372
ZNF511	0.69261609	2.6395E-05	ZNF322	-1.01392842	0.00013618
NPM3	0.69243079	1.8636E-05	RALGDS	-1.01378261	8.4623E-09
UBFD1	0.69189374	0.02585053	GBA	-1.01337602	1.6749E-05
MZT2A	0.6916745	1.9966E-05	TBC1D14	-1.01313723	2.2092E-07
BTF3	0.69100162	8.4659E-28	IGF2R	-1.01257484	2.502E-05
CDC42SE2	0.69086446	5.9856E-12	PTGR1	-1.01206557	6.4381E-05
DCTPP1	0.69074511	1.7117E-05	IRF2BPL	-1.01074051	0.00018819
ACTR1B	0.69041668	0.00233975	ATG4C	-1.01051107	4.0058E-08
PSMB1	0.69017539	9.1162E-20	BBS9	-1.00746181	0.04664566
RPL36A	0.68987071	2.5564E-18	UEVLD	-1.0061212	0.00017237
CCDC109B	0.68969225	0.00010557	RAB39A	-1.00434109	0.00213752
TPI1	0.68947658	3.3221E-18	DNMT3A	-1.00355414	1.129E-05
RPL35A	0.68943411	2.5364E-21	PTER	-1.00273938	1.3239E-05
PLBD1	0.68915618	1.0646E-05	FAM89A	-1.00268568	0.03093691
RPS24	0.688858	3.8446E-18	AMPD3	-1.00259741	3.94E-09
PRKDC	0.68883181	6.9631E-07	RRAGD	-1.00255671	4.6919E-07
CSTF3	0.68796208	0.00351906	SNX11	-1.00070799	4.3471E-05
ETHE1	0.68722243	4.9419E-05	IFI44	-0.99918289	1.274E-06
PSMA5	0.68720331	9.1219E-08	LINC01480	-0.99897505	0.00014779
LDHB	0.68677343	6.5089E-15	L3HYPDH	-0.99879943	0.01350415
NIPSNAP1	0.68649683	0.01344807	IRF5	-0.99756283	1.3943E-06
MFSD3	0.68578933	0.03965664	SAMD4A	-0.99726978	0.000168
PPA2	0.68573823	2.8745E-12	TNFSF9	-0.99700421	0.0013357
NDUFA9	0.68556141	1.6372E-06	ZNF358	-0.99642476	2.0752E-06
PSMA1	0.6852557	3.8724E-18	AF127936.9	-0.99633249	0.03866614
VCPKMT	0.68469104	0.02012505	ATP7A	-0.99566569	7.7094E-05
FBXO6	0.68432313	0.01107709	FANCL	-0.99559037	0.02660591
MYL6	0.68414652	5.4203E-19	ZNF567	-0.99467283	0.02209525
RAB8A	0.68403373	6.4989E-11	SULT1A1	-0.99464719	5.9892E-12
TMEM11	0.68374608	8.5711E-05	ZNF646	-0.99319445	0.02758973
GPSM3	0.68295	2.0634E-16	RAPH1	-0.99283472	0.00732049
LST1	0.68284076	7.3917E-11	VPS50	-0.99235756	0.00010557
RPS10	0.68273633	1.047E-22	NUMA1	-0.99160382	1.002E-07
FTCDNL1	0.68239435	0.03105611	AAK1	-0.99037618	7.8758E-16
EXOSC1	0.68238125	1.0377E-06	ZNF141	-0.99027121	6.3996E-05
SMIM13	0.68153396	0.0171983	ZFP36	-0.98853154	3.2088E-13
GAN	0.6812293	0.03427693	PPP1R15B	-0.98797279	4.1008E-10
SLC35B1	0.68101657	2.5419E-05	UTP14C	-0.9873197	0.01138532
STAT1	0.68054603	2.7508E-05	DNASE2	-0.98585585	1.3683E-05
SLC25A6	0.68021905	1.7621E-15	ZFYVE26	-0.98578862	0.01013277
RPS15	0.67940644	1.2054E-17	LRRC27	-0.98505117	0.01057573
DIABLO	0.67915383	0.00916874	ZRSR2	-0.98444036	2.3917E-09
RPL15	0.67912814	8.9327E-20	SMYD4	-0.98442428	6.6556E-06
RP11-468E2.4	0.67893262	0.00306701	FBXO8	-0.98335913	3.7118E-05
PRCP	0.67859613	5.1229E-13	AHRR	-0.98263631	0.01097397

RPL37A	0.67721166	1.1801E-20	HSP90AA1	-0.98140501	2.0971E-06
TLE3	0.67703698	0.02508329	LAMP2	-0.98046846	1.6451E-22
RPL27	0.67662906	8.4325E-24	FILIP1L	-0.97979066	1.8209E-16
RPS12	0.6765711	3.0779E-15	FGFR1	-0.97815641	0.00146717
BOP1	0.67640923	0.00085409	ZNF516	-0.97785181	2.4942E-07
PIM1	0.67582881	0.02728669	TAF1C	-0.97781618	0.00204111
ACTG1	0.67568231	2.2825E-10	PPP1R15A	-0.9771185	3.1195E-16
SNRPG	0.67510427	5.1997E-12	ANKRD9	-0.97598479	3.9036E-05
SNRPE	0.67475888	5.4097E-12	JUNB	-0.97496344	9.9293E-07
PGS1	0.67446237	0.0082279	ASAP2	-0.97478011	0.02461571
RPS15A	0.67434102	1.8002E-20	ZNF891	-0.9737355	0.01214999
VRK2	0.67424055	2.8357E-05	PARP8	-0.97226334	6.1321E-09
DCXR	0.6742262	1.2254E-08	OPHN1	-0.97149073	0.00887044
LPXN	0.67384591	2.5287E-06	SLC25A30	-0.97095168	0.0125744
RPS3	0.67380284	1.4318E-20	TMEM53	-0.97066538	3.9777E-05
EIF3F	0.67347902	1.1766E-11	GLUL	-0.96980514	2.2872E-16
STAT4	0.67329262	0.0044012	ULK3	-0.96950266	0.010021
GTF3A	0.67327059	1.4812E-13	ZC3HC1	-0.96949047	0.02025423
RPS23	0.67242221	2.441E-17	OCRL	-0.96918831	0.03683378
HIP1	0.67238101	0.00482051	CYP4V2	-0.96880818	0.00017544
PDCD2	0.6716684	2.5421E-06	MCOLN1	-0.96789106	4.0704E-05
CCDC57	0.67143745	0.00031891	MLF1	-0.96750353	0.00146736
MRPL33	0.67130003	2.6196E-11	DBR1	-0.96601235	0.0055123
MAPKAPK3	0.67064718	6.1554E-06	PLCL2	-0.96526405	9.3667E-06
MED4	0.66891948	7.0296E-08	CR1	-0.96464931	0.00171145
RPS29	0.66802481	3.3789E-17	CEP162	-0.96436276	0.00624369
RPL26	0.66798922	6.4995E-15	BBC3	-0.96209195	0.0069072
TRADD	0.66780802	0.00140421	COL8A2	-0.96083115	0.00298754
TSTA3	0.66749234	0.001335	SLC4A7	-0.96026612	1.2648E-07
CAMK2G	0.66687558	0.0058163	GAA	-0.95995421	2.5311E-18
ZNF92	0.6668417	0.00146151	TMEM138	-0.95976378	7.698E-07
APOL3	0.66635635	0.0011012	ZBTB41	-0.95925619	0.00110199
RPL18	0.66604689	1.75E-17	ANXA2R	-0.95808458	0.00167194
COX5A	0.66552592	5.46E-13	ZMYND8	-0.95748997	4.816E-10
C20orf24	0.66498513	8.7618E-07	ZNF629	-0.95663697	0.03372807
MTPN	0.66434045	2.2275E-11	ZZEF1	-0.95582158	2.5341E-07
CCZ1	0.6637378	5.4874E-05	LZTS2	-0.95514942	0.01709901
RNPS1	0.66370828	3.5804E-15	PGAP3	-0.9549221	0.0237731
TYW5	0.66211383	0.02822511	PTGS1	-0.95456466	2.4984E-09
SLC25A3	0.66190844	2.7812E-09	LRIG2	-0.95402496	0.01374483
MTHFD1L	0.66171163	0.00316472	CRTC1	-0.95298938	0.03636303
RNF139	0.66121191	1.046E-05	ANGEL1	-0.952716	0.03849399
RPS16	0.66107383	4.4363E-23	TNFRSF10D	-0.95170492	0.00011709
CHML	0.660078	0.00015016	KRBOX4	-0.95130807	0.00087946
PSMD7	0.65894646	3.1678E-17	SIAH2	-0.95127186	0.000659
NUDT19	0.65891976	0.04114481	STAM2	-0.95083126	2.0412E-06

MED27	0.65889914	0.00994384	FPR3	-0.95075854	5.1283E-14
UNC119	0.65886984	5.7702E-05	TFEC	-0.95048774	2.2733E-12
ACTR3	0.65838385	3.2642E-08	ATP13A2	-0.95038651	0.0167019
HPS5	0.65837899	2.8359E-06	CLIC4	-0.94929234	2.4003E-08
MTRF1L	0.65797736	0.00395676	NFATC2	-0.94912323	1.0373E-06
NIT2	0.65772864	2.2459E-05	IFRD1	-0.94866683	1.7501E-15
ERH	0.65749176	2.9068E-11	PRKACB	-0.94683534	1.0635E-16
SMIM14	0.65691782	1.6079E-08	SLC38A9	-0.94669613	0.00221445
UBN2	0.65536845	0.03623388	SCRN3	-0.94564009	0.00649759
KPNA2	0.65489906	1.0775E-12	FLI1	-0.94457671	2.3977E-07
CERS5	0.65377782	0.01064314	TMEM87B	-0.94443309	3.1644E-05
CYSTM1	0.65375703	5.2633E-06	ALKBH4	-0.94388316	0.03500138
RPL28	0.65352781	9.928E-16	CYB561A3	-0.94375063	1.5285E-13
ATP5J2	0.65235581	4.3322E-12	PLXNA3	-0.94367189	0.01151556
GALK1	0.65172811	8.9508E-05	CD68	-0.94335783	1.5567E-13
BLOC1S4	0.65134049	0.00479783	CLIP2	-0.94327053	0.0002628
CPSF3	0.65112972	0.00049007	FZD1	-0.94289387	0.00051598
FADS3	0.65075783	0.01063181	RASAL2	-0.94239332	0.00016856
RPS11	0.65026051	1.0847E-13	DOC2A	-0.9420895	0.01559742
PCSK7	0.64945793	1.1301E-05	DYRK2	-0.941924	1.0132E-05
RPS3A	0.64941215	2.1487E-18	ARMCX1	-0.94114256	3.443E-08
RAB22A	0.64876644	0.00067627	KIAA0319L	-0.94104915	8.7204E-05
ATP5F1	0.64865749	3.8909E-16	ITM2B	-0.93933285	3.5342E-10
SMARCC1	0.64825327	4.1049E-07	CDR2	-0.93896229	0.01319533
MAD2L2	0.64808108	0.00172244	DOCK7	-0.93876474	0.00019148
NPIPB4	0.64787193	0.03272228	EMB	-0.93844187	5.2723E-06
THOC7	0.64757015	2.3367E-11	GIMAP2	-0.93809136	4.1642E-08
RCC2	0.64752059	2.2874E-06	HELZ	-0.93731556	1.2455E-12
TMA7	0.64741127	1.0204E-15	RFX7	-0.93707494	0.00190792
ARPC5	0.64642699	8.1722E-15	AMMECR1L	-0.9370064	0.00316654
CBX5	0.64637295	0.00013409	WDR45B	-0.93660948	4.1266E-08
SNHG15	0.64604305	1.4315E-05	LAIR1	-0.93621242	7.2086E-10
SNRPD2	0.64599787	9.1927E-14	METTL7A	-0.93500069	1.3854E-11
TUFM	0.64576457	4.4349E-10	TMEM94	-0.93490689	0.00624718
NOP16	0.64547125	0.00159877	TPRG1L	-0.93262605	2.9294E-05
DCTN3	0.64510415	5.244E-07	TMEM99	-0.93240527	0.04271293
RBFA	0.64461035	0.00846025	RP11-707G18.	-0.93192283	0.00051738
ENDOV	0.64460738	0.00063744	KCTD5	-0.93069512	1.1431E-08
DHRS4L2	0.64355183	4.0739E-06	RDX	-0.92996707	6.423E-08
UBE2N	0.64330258	1.675E-07	LILRA6	-0.92902064	0.02316647
LCP1	0.64186055	2.7168E-21	TCAF1	-0.92901372	0.00076868
RPS27	0.64153832	1.2446E-26	FAM120B	-0.92869845	0.0044362
WAC-AS1	0.6415333	8.4758E-05	ATXN1	-0.92863709	7.0189E-06
ZNF880	0.64052951	0.01070352	TNFSF15	-0.92809945	0.0492292
STXBP2	0.64041548	1.1896E-08	SLC12A7	-0.92623928	1.3954E-06
ENSA	0.64007311	2.6627E-11	ZSCAN29	-0.92488918	0.00828257

FHL3	0.63931008	0.000701	NEDD4L	-0.92427088	6.2378E-07
PCIF1	0.63858756	0.00025747	DEXI	-0.92379393	0.03650002
ZDHHC18	0.63817847	0.00585903	PIK3R4	-0.92356842	0.02665911
RPL10	0.63782317	6.0216E-16	PHC1	-0.92086482	0.01795075
SDHB	0.63778166	4.19E-12	DIP2B	-0.92056908	4.7192E-09
IDH3G	0.6376379	1.2801E-07	HIVEP3	-0.91943454	1.0244E-05
SET	0.63630372	4.2754E-23	BTN2A1	-0.9194335	1.8785E-05
RFX2	0.63594682	0.04230091	GOLPH3L	-0.91911173	0.00567233
RPS17	0.63530104	1.5736E-17	RP11-758H9.2	-0.91882111	0.00649052
TPM3	0.63494182	1.0283E-25	FMNL3	-0.91837122	1.6674E-06
WAS	0.63445642	7.4257E-10	FUT8	-0.91819439	0.00351906
COX7B	0.63408548	6.8662E-17	LZTR1	-0.91705655	0.00900153
CTA-384D8.34	0.63369454	0.00916582	CEBPA	-0.91638547	1.4697E-06
RASSF5	0.63337371	1.3859E-05	RANBP10	-0.91497	0.01454779
MZT1	0.63325727	0.00071269	TAGLN	-0.91457463	0.01306554
ATG3	0.63324757	2.0146E-14	IL1RAP	-0.91386172	4.4278E-07
LSM4	0.63323439	4.5415E-07	MORN2	-0.91274761	0.00367504
SEPT7	0.63315056	1.0295E-14	MFSD5	-0.91221423	0.0086221
PLEKHA5	0.63294505	0.04579835	CTA-292E10.6	-0.91122018	3.4013E-05
UBE2L3	0.63278076	9.8403E-11	CEBPB	-0.91008293	3.8431E-05
ACTR1A	0.63265275	2.3637E-07	TANGO6	-0.90976191	0.02232313
SEC13	0.63261462	4.9135E-08	DHX34	-0.90913849	0.00077862
SNRPA1	0.63240415	8.1457E-08	TRAFD1	-0.90814632	0.00105506
LSM6	0.63222037	4.4073E-09	CEP290	-0.9079669	0.0002336
OSTC	0.63204298	2.8078E-09	MEF2A	-0.90777564	6.4718E-13
PPHLN1	0.63203889	3.3109E-09	LPAR2	-0.90666599	0.01398606
CKS1B	0.63179963	0.00021875	TNPO2	-0.90633712	0.00909161
RPL36	0.63142873	1.1656E-19	GAS2L3	-0.90601488	5.9422E-05
PRPF19	0.63123773	0.00014461	TFRC	-0.90522916	0.00040919
RAP1GDS1	0.63123462	0.00015516	STAG3	-0.90453346	0.0339946
DDHD1	0.63119805	2.4865E-05	MFSD13A	-0.90452522	0.00034543
PTGES2	0.63108049	5.047E-06	LATS1	-0.90377135	1.8107E-05
MRPS22	0.63086538	0.00094568	PECAM1	-0.90156189	6.4406E-16
PELI2	0.63085593	0.0333983	ARL5B	-0.9006241	4.5241E-15
TIMM23B	0.62944534	0.01469944	AHR	-0.90010127	1.9182E-23
CASP8	0.62875563	0.00560421	TUSC1	-0.89861526	0.00497377
STK17A	0.62830624	0.01243166	ANKFY1	-0.89657083	0.00040244
MBD2	0.62812731	1.3032E-06	ZNF468	-0.89617011	0.02774985
ZNF394	0.62768393	3.7723E-06	S1PR2	-0.89615239	0.0068135
AKIRIN1	0.62565121	2.4322E-05	TRG-AS1	-0.89573396	0.00432025
HBS1L	0.62563488	0.00446227	DUSP16	-0.89509608	0.00422026
AKAP13	0.62513459	9.5076E-07	EGLN3	-0.8941016	0.03072753
RAP1B	0.62501582	3.0457E-08	HS2ST1	-0.89307372	0.00022954
HNRNPA0	0.62481327	1.4371E-11	RRAGB	-0.89284145	4.0453E-05
MED28	0.62423683	2.7106E-12	ACP5	-0.89216148	0.00013496
EIF3E	0.62348753	1.3477E-10	VAV1	-0.89148125	1.9286E-07

PAFAH1B3	0.62287824	0.0014931	ZNF148	-0.89029127	3.0946E-08
ASL	0.62188063	0.00118576	BTN3A3	-0.89002296	9.1589E-05
SYAP1	0.6214095	3.4011E-09	LMBRD2	-0.88978497	0.01593392
RPL41	0.62134156	2.836E-21	TGDS	-0.88966977	0.01015484
CASP1	0.62125722	4.2439E-14	ZNF354B	-0.88933412	0.04836148
PDAP1	0.62109027	8.8695E-08	PRMT7	-0.88910808	0.00465839
SMG1	0.62094214	7.1669E-06	HMG20A	-0.88904823	1.6624E-05
RPS6	0.62066177	1.8328E-23	SWAP70	-0.88823596	8.6369E-12
RNF4	0.62051318	0.00044803	FZD5	-0.88742717	0.02017702
ITGB1BP1	0.620163	1.6952E-06	TTLL5	-0.88706315	0.0125358
RPL30	0.61973963	3.5817E-22	RP11-876N24.	-0.8861481	0.01672166
PCCB	0.61968794	0.00301698	PMS1	-0.88591732	0.00020209
PPP1CA	0.6191752	3.8968E-13	LINC01451	-0.88449635	0.00510754
PFDN1	0.61839308	4.449E-05	SLC9A3R2	-0.88409479	0.04137418
RCN1	0.61811383	0.00039599	GGA3	-0.88364851	0.00021585
PPM1G	0.61695514	1.15E-07	HMGCL	-0.88215805	3.219E-07
LYPLA1	0.61665535	2.4442E-10	PLXNB2	-0.88215022	5.4556E-10
HAUS7	0.61659689	0.0002503	FSTL3	-0.88177298	0.03793221
EID2	0.61646906	0.00897206	TET3	-0.88140211	1.0522E-09
BATF3	0.61634765	1.1018E-06	USP24	-0.88116282	1.3782E-06
GSTZ1	0.61603744	0.01185229	FLCN	-0.88102566	7.7442E-07
CD44	0.61585103	0.00012766	P2RX7	-0.88047156	9.6785E-06
PAPD5	0.61565885	9.4247E-05	MAP3K14	-0.87893166	0.01431411
TPRKB	0.61444201	1.8314E-08	SLC46A3	-0.87864646	4.6077E-07
PSMD9	0.61427272	1.6255E-09	PUM1	-0.87831805	3.1428E-13
COX7A2L	0.61399523	3.8747E-13	ZNF266	-0.87676766	0.0024517
SNF8	0.61385107	4.91E-08	DHRS3	-0.87630375	8.4246E-15
HCG18	0.61354758	0.03601618	SMURF2	-0.87597337	1.1563E-06
NUTF2	0.61208492	1.9531E-06	METTL25	-0.8759484	0.00822624
RPLP2	0.61192312	3.689E-17	SIK1	-0.8745679	0.00344827
CPT1A	0.61121521	0.00085409	INTS6	-0.87454164	5.236E-06
MRPL11	0.61062881	1.1525E-07	SMAD7	-0.87279349	0.00855559
RPS6KB2	0.61040294	0.00134679	C3orf62	-0.87263828	2.0718E-06
TOP3A	0.60972858	0.00415409	GIMAP4	-0.87255058	4.1615E-08
RPS9	0.60958796	2.4887E-18	SELM	-0.87244596	4.301E-07
C2orf68	0.60958544	0.03449644	TPCN1	-0.87122075	1.425E-05
NOP58	0.60878652	4.0614E-06	C9orf91	-0.87054	0.00059994
PSMD13	0.60803394	1.1444E-11	SYNRG	-0.87044773	2.8085E-06
FAM162A	0.60789095	2.7883E-10	KCNE3	-0.86927899	2.557E-06
WBSCR22	0.60733044	9.1928E-08	TBC1D15	-0.86822386	4.2171E-14
ARF3	0.60682559	5.041E-09	ARL15	-0.86793425	0.00783492
YIF1B	0.60636595	1.3151E-07	PAN2	-0.86621368	0.017868
FH	0.60573444	0.00057541	ARHGAP10	-0.86418295	1.4023E-06
COPE	0.60567569	6.3101E-12	KCTD3	-0.8638333	0.03104521
CHCHD3	0.60547074	5.3381E-05	FAM50B	-0.863556	0.02571202
GSTK1	0.60522507	5.417E-08	DOCK11	-0.86203238	8.7329E-09

DDX21	0.60422708	1.1401E-20	S100PBP	-0.86122769	0.00140161
ARID5A	0.6037947	3.5658E-06	ALDH9A1	-0.86091515	1.5009E-09
FCMR	0.6035006	0.04944476	MLLT6	-0.86027815	4.4697E-07
SNRPD3	0.60339798	1.2109E-11	DGCR2	-0.86022257	0.03341347
CDC123	0.60299541	9.1317E-05	CBFA2T2	-0.85924184	0.00472288
ANAPC5	0.60187476	5.7043E-07	DOCK1	-0.85800589	0.02958126
BRI3BP	0.60152218	0.00209743	TMEM60	-0.85796228	1.3609E-06
AP1S2	0.6014163	3.7718E-05	PDE4DIP	-0.85738041	1.2693E-05
CAPNS1	0.6009667	3.0156E-13	RNF135	-0.85732749	1.443E-07
GMDS	0.60086855	0.02092054	MKL2	-0.8544076	0.00760037
ACAT2	0.60051944	0.02305723	RP11-472N13.	-0.85428001	5.4874E-05
POLR3H	0.60045299	0.04419859	QSER1	-0.85393018	2.3862E-05
UBA52	0.60020116	2.1073E-14	ARHGEF11	-0.85298501	0.03552524
PLK2	0.59971051	0.00186747	NMNAT1	-0.8524588	0.03451192
TOMM22	0.59941319	1.0237E-05	PPARA	-0.85225009	9.7781E-05
LDLRAD4	0.5994073	0.00163832	ARAP1	-0.85031067	1.7829E-10
GSPT1	0.59928376	9.5639E-08	MLLT11	-0.84826928	0.00016651
TMEM167A	0.59923284	4.4748E-07	CCNG2	-0.84796831	1.8377E-07
COX7A2	0.59872232	7.2222E-13	ELL2	-0.84783967	8.8212E-08
CTNNBL1	0.59826857	3.2834E-05	IL1B	-0.84764789	2.3744E-05
ALG3	0.59821879	8.3896E-05	STMN1	-0.84750482	1.402E-05
FOSL1	0.59816599	0.04645639	C2CD5	-0.84612075	0.00026874
ATXN10	0.59783302	6.7954E-06	CCDC102B	-0.84417418	0.03299913
PRPF4B	0.59737511	2.4773E-05	HMGN5	-0.84414749	0.00273881
LSM12	0.59719094	2.7701E-06	MPP1	-0.84381106	5.6356E-15
ARPC3	0.59705342	8.0826E-14	FOXO3	-0.84373574	7.8111E-16
SMIM12	0.59702025	9.3004E-06	CTNND1	-0.8435884	1.6428E-07
GTF2A2	0.595587	1.6942E-07	NEIL2	-0.84301421	0.00125979
PPDPF	0.59556332	0.00010424	METTL13	-0.84258271	0.00288172
EMD	0.59550245	1.3359E-06	HNMT	-0.84127894	1.4859E-26
NDUFV2	0.59455107	6.2324E-08	RCOR3	-0.84127764	7.604E-05
NUDT15	0.59428775	0.01945483	ERCC4	-0.84086691	0.00058703
KCNK6	0.59401926	0.00186257	MARCH3	-0.83963219	0.04339777
PSMD12	0.59361316	3.7326E-08	P4HA1	-0.8373487	3.8858E-10
HM13	0.59314581	6.2284E-10	DBT	-0.83568008	0.03848365
FOXK1	0.59303443	0.01666885	ZDHHC14	-0.83512904	0.00010893
MRPS15	0.59193157	1.6508E-08	RETSAT	-0.83511744	0.02731744
CACNA2D4	0.59186964	0.00276964	NSUN5	-0.83465952	4.7458E-05
LSM3	0.59184474	1.0242E-12	ZNF654	-0.83455573	0.00199134
SLC27A3	0.59171824	0.00578965	KCNQ1OT1	-0.83412971	0.00213752
VTI1A	0.59167841	0.00024083	FAM217B	-0.83252637	0.02244111
PYURF	0.5916516	8.2235E-06	FAR2	-0.83246183	0.00699944
MRPL36	0.59155637	0.00064643	PIK3C2A	-0.83161352	1.8793E-10
GSN	0.59115497	1.6749E-05	AAMDC	-0.82908968	4.8391E-06
DDX39B	0.59070769	0.00152082	LZTFL1	-0.82905677	0.03049728
USP5	0.59057129	0.01997484	ARHGAP24	-0.82864213	0.03289282

TTF2	0.59042011	0.0366434	MYC	-0.82835616	0.04219554
C8orf33	0.59010686	0.00160277	RGS12	-0.82817531	5.3699E-05
ELOF1	0.58967579	5.5468E-05	SIN3B	-0.82800084	5.6031E-05
RPL24	0.58951854	2.2421E-21	RP9	-0.82793831	0.00148756
GRPEL1	0.58909187	3.1188E-05	GAB1	-0.82789986	0.00882197
PTPN7	0.58895281	0.02363171	ATP11C	-0.8278008	5.3227E-06
ATP2B1	0.58873993	7.7399E-06	TIMM21	-0.82582769	0.0080787
ARF6	0.58843943	1.0567E-13	ADAM17	-0.82464834	1.7035E-19
BZW1	0.5880468	3.1411E-14	NEK3	-0.82455765	1.174E-05
DHPS	0.58799102	0.00048166	WDR75	-0.82396025	0.0107175
VRK1	0.58783843	0.00053838	PHC2	-0.82348093	2.2615E-09
FLOT1	0.58779742	3.4289E-07	NFKBIA	-0.8233585	4.0482E-05
HIGD1A	0.58681951	0.00266494	ABI3	-0.82252742	3.7699E-18
RPL34	0.58654378	3.1927E-11	SPARC	-0.82222459	0.02664121
LPIN1	0.58645393	0.00043283	MCM9	-0.82210966	0.03468153
ODF2	0.58551275	0.032094	ZNF808	-0.82199234	0.033829
ANKRD11	0.58536132	1.7969E-06	KHK	-0.82196569	0.00234511
MAD2L1BP	0.58506075	0.01388257	LCORL	-0.82127731	0.0001017
YIF1A	0.58504653	1.6003E-06	RFWD3	-0.82063315	0.01360991
MPC2	0.5846721	3.9939E-07	METTL15	-0.82052081	0.03476179
EIF3G	0.58461154	9.7614E-18	SLC9A6	-0.82010886	0.03752613
PMPCB	0.58403707	4.065E-07	CDKN2AIP	-0.81992509	0.00082964
HNRNPR	0.58386165	1.4554E-14	PLOD1	-0.81980923	0.01198502
PSMB4	0.5830608	1.863E-10	HSBP1L1	-0.81938268	5.7231E-05
OXCT2	0.58287299	0.00973152	CHD7	-0.8192941	0.0004833
POLR2C	0.58287099	0.00074373	MMP19	-0.81901062	0.00012259
POLD2	0.58286302	0.00039303	ATHL1	-0.81878023	0.00446494
CHST12	0.58270982	0.01084188	PXDC1	-0.81875921	4.0339E-06
LILRB2	0.58193895	5.7404E-05	ZNF317	-0.81850598	5.6675E-05
USB1	0.58141661	0.00189021	GOLIM4	-0.81843172	6.4082E-11
C7orf73	0.58109063	5.4335E-06	ITGAM	-0.81833656	0.01087086
CIDEB	0.58000022	0.01644599	HSF2	-0.81681298	0.01403
NOB1	0.57969154	0.00022656	MAP1S	-0.81602114	0.00010613
PSMA7	0.57964603	1.9064E-17	RNF111	-0.81601541	4.7048E-05
COMMD9	0.57930491	1.7281E-07	SAV1	-0.81591868	0.00904064
MKL1	0.57900132	0.03452343	ZNF236	-0.81576686	0.01654241
ARPC2	0.57865807	7.4509E-18	TBC1D9	-0.81364587	1.6377E-07
ARHGAP30	0.5783591	1.0942E-05	ZBTB48	-0.81301898	0.00046518
PSMD8	0.57826291	1.1143E-05	ATE1	-0.81259738	0.00155726
CCZ1B	0.57826009	0.01435479	SFMBT2	-0.81235803	4.1647E-11
UVRAG	0.57783942	6.7532E-06	FOXO1	-0.8120764	1.698E-09
PSMC5	0.57727644	7.8514E-09	ZNF44	-0.81175223	0.00042328
ADRM1	0.57702388	1.2499E-07	ZNF331	-0.81147986	1.8083E-06
SNU13	0.57603565	1.1821E-12	LRCH1	-0.81102831	0.00035515
CYC1	0.57519314	1.6957E-06	SREBF1	-0.81095839	0.0002552
COX6A1	0.57490714	5.0995E-15	SLC25A25	-0.81069982	0.00250044

B4GALT5	0.57477685	0.00284874	HECTD3	-0.81069578	0.04964489
PPP1R7	0.57444115	1.2922E-06	ABCC4	-0.81019634	0.00034711
ETFA	0.57376014	5.0126E-08	TAB2	-0.80977994	2.2236E-06
VDAC1	0.57311259	1.1863E-06	FO538757.2	-0.80973879	0.00361277
TOLLIP	0.57250618	0.00384428	UPF1	-0.8079192	7.726E-06
ORMDL2	0.57214614	1.0858E-06	OTUD1	-0.80691217	7.1906E-07
PCGF5	0.57148964	2.0916E-05	SLC27A1	-0.80647479	3.8428E-05
EDEM1	0.57132099	9.6466E-05	SLC48A1	-0.80560945	0.00601048
SUB1	0.5710135	8.236E-13	APBB3	-0.80453115	0.0058396
LGALS1	0.57067778	0.00079065	GM2A	-0.80386075	2.7011E-09
EEF1A1	0.57066689	5.6077E-20	NBPF12	-0.80279336	0.00028265
WDR1	0.56984807	9.4831E-10	ARRDC2	-0.80219776	6.89E-06
ORAI2	0.56969067	0.00359658	TEX2	-0.80115683	0.0337887
PHF5A	0.56931679	0.00011334	ZDHHC4	-0.80056087	3.1881E-07
JAML	0.56927242	0.00065831	KANSL1L	-0.80051601	0.0050609
ATF5	0.5692681	0.0074065	ELL	-0.80022103	3.4503E-06
LSM1	0.56919027	2.8703E-06	FNDC3A	-0.79962378	6.8478E-08
APEH	0.56904628	0.00116355	RND1	-0.79903956	0.03355832
GPBP1	0.56738858	4.2499E-07	SYNJ1	-0.79875317	0.03035423
POLR2H	0.5667092	1.8008E-05	RCBTB2	-0.79775121	1.0782E-09
TRAPPC10	0.56643116	0.00094584	ANKRD42	-0.79688289	0.00846285
XRN2	0.56635005	5.9974E-13	BLVRB	-0.79670702	2.6344E-14
HLA-DPB1	0.56606075	1.3375E-07	PLEKHM2	-0.79653342	1.8091E-09
EIF5AL1	0.56588995	0.01568349	FGL2	-0.79633511	1.1094E-25
UBE2V2	0.56583792	4.5849E-05	MPEG1	-0.79629009	8.8268E-36
RBM3	0.56494149	2.2116E-11	ERF	-0.79617514	0.00016489
RNF2	0.56478168	0.00576109	ATP2A2	-0.79611621	8.1884E-05
GUK1	0.56472937	1.0265E-08	BTBD19	-0.79512163	0.01465872
TMEM120A	0.56465553	0.00075991	CWF19L2	-0.79484862	1.7154E-07
EFHD2	0.56465409	3.1083E-07	PPP1R21	-0.79351009	3.3085E-05
ZBTB8OS	0.56463534	5.1801E-05	ZNF395	-0.79236506	0.00171291
SDHAF3	0.56414085	0.00419918	PHLDA1	-0.79213666	1.1739E-08
MRPS2	0.56343183	0.02363171	PLXNA1	-0.79180722	0.0204417
ZSWIM7	0.56304212	1.3897E-05	LCP2	-0.79166262	4.1993E-15
RUVBL1	0.56265975	0.00191334	KLHL9	-0.78896765	0.00027772
SMC1A	0.56259136	6.8499E-05	PGPEP1	-0.78786922	0.00012805
VPS29	0.5622496	4.5588E-07	AMDHD2	-0.7872107	2.0437E-06
AGPAT3	0.56213027	0.00323904	NEK6	-0.78712348	0.00312548
MRPL52	0.5620978	2.3214E-11	GPX3	-0.78709357	0.03711408
TFEB	0.56207739	0.00110717	MIER2	-0.78689273	0.00125213
KIAA0907	0.56150063	0.0171983	DCLRE1B	-0.78677911	0.00125979
NAP1L1	0.56116299	7.8398E-06	ABHD10	-0.78531793	0.0118526
SSNA1	0.56093918	4.5432E-07	MED26	-0.78463929	0.00131295
RILPL2	0.56024076	1.6357E-07	SLC29A1	-0.7844134	0.00184884
C8orf59	0.55999774	1.0968E-08	PPM1L	-0.78336303	0.01693782
N4BP2	0.5598543	0.00055531	TMEM220	-0.78291029	0.00143706

RPPH1	0.55963048	0.04807984	PPP2R5B	-0.78216372	0.03253424
PXK	0.55934915	0.00304741	IFT88	-0.78156605	0.03059185
COMMD5	0.55931472	5.9102E-05	RAB13	-0.78100346	5.1422E-06
BAX	0.5591905	1.4412E-11	PCMTD2	-0.78095997	0.00024442
SRP9	0.55857707	3.9592E-06	ATM	-0.78050349	1.4269E-10
GSTO1	0.55821802	1.5752E-07	CCNL1	-0.7804639	1.5661E-10
ACP1	0.55818101	1.2258E-05	ZNF140	-0.77991307	0.02692234
AURKAIP1	0.55813144	3.9524E-09	TRUB1	-0.77951889	0.03405669
RPL35	0.55788102	5.6733E-16	ZNF330	-0.77915116	6.7555E-08
HNRNPM	0.55749302	1.8328E-19	SLC16A1	-0.77912205	0.00350438
ATP5L	0.55745925	5.7683E-12	CLEC11A	-0.77737416	2.7928E-05
OTUB1	0.55711523	2.6416E-06	NCOA3	-0.7771901	3.4606E-10
SRC	0.55709678	0.00563695	SUFU	-0.77654461	0.02005429
THAP1	0.55706447	0.0422572	POLR3C	-0.77585703	0.00095837
MRTO4	0.55704929	0.00318884	TMEM260	-0.77582943	0.0296884
GGCX	0.55697385	0.00063539	CREB3L2	-0.77527205	3.9203E-07
C14orf2	0.55694878	6.2989E-09	CTD-3138B18.	-0.77456457	0.03089311
DEF6	0.55690938	0.00152082	FTL	-0.77394953	9.4614E-10
NEDD9	0.55640711	0.00280792	SLC35F6	-0.77371882	3.6233E-06
NDUFS6	0.55617269	1.6255E-09	SEMA4A	-0.77282615	0.00991126
DDOST	0.55580382	1.2833E-05	HLA-E	-0.77270795	1.0688E-14
SLC36A4	0.55564572	0.00091706	KLF11	-0.77227948	0.00049128
GPRIN3	0.55534862	0.00286102	IER5L	-0.77175951	0.02999379
MRPL48	0.55433925	0.00020765	EHBP1	-0.77156638	0.01258397
UROS	0.55413345	0.0055589	ARFGAP1	-0.77128255	0.00451579
TUBGCP2	0.55402396	1.7255E-05	IRS2	-0.7712607	2.7593E-07
MRPL4	0.5539952	6.6053E-06	ZNF10	-0.77065932	0.04263924
C12orf45	0.55370119	0.0009557	SLC38A2	-0.7706424	1.6883E-09
MRPL3	0.5530307	1.2747E-06	MYO9A	-0.77056991	0.00106993
PILRA	0.55295967	1.6759E-05	FGD6	-0.77054382	0.00292271
EIF2A	0.55289943	6.8951E-10	RMDN3	-0.76937029	9.4866E-06
CCT3	0.5525687	1.0157E-08	PLVAP	-0.7690412	0.03660993
UBL7	0.55178744	0.00018831	NPC2	-0.76887405	1.5769E-12
STRA13	0.55121147	5.5838E-05	LY96	-0.76859747	2.5063E-18
MARS	0.55088663	0.01679343	RASSF1	-0.76780586	1.5954E-05
MYL12B	0.55072293	1.5871E-08	SENP7	-0.76670654	0.00034279
GMFG	0.55071424	2.2251E-09	ZNF513	-0.76653817	0.03455564
RPL13	0.55028746	1.1499E-07	SYNE1	-0.76637902	0.01508951
ZPR1	0.54994001	0.00077612	MTMR2	-0.76589589	0.02228177
GABARAPL1	0.54987656	9.5166E-05	ZNF316	-0.76577692	0.00120208
TSN	0.54975145	0.00011748	KAT2A	-0.76523413	0.0028424
DHRS1	0.54958168	0.02298211	GAMT	-0.76476331	0.04411182
CYB5R3	0.54933706	6.4706E-06	SLC7A7	-0.76467545	2.9244E-06
MCM5	0.5493367	0.00040045	SNAPC1	-0.76420404	0.00029627
UBE2L6	0.54899369	5.9053E-08	NBR1	-0.76418175	1.2601E-05
INPP5B	0.54869838	0.04667835	MBD6	-0.76417815	0.00240919

DGUOK	0.54851965	8.0883E-05	PDK2	-0.76328252	0.00209142
UFC1	0.54838261	9.4192E-10	ERBB2IP	-0.76285638	8.4173E-12
STK10	0.5481157	0.01061415	LYPLAL1	-0.76258161	0.00030222
IWS1	0.54793123	0.00262149	MAN1A1	-0.76220729	1.0033E-05
IFNGR1	0.54791753	9.84E-06	CPNE8	-0.76106384	2.2975E-05
CIITA	0.54757258	0.00045829	LMNA	-0.75991117	3.5221E-11
SMARCE1	0.54713071	6.9623E-07	NAGLU	-0.75988872	0.0003577
TCIRG1	0.54709539	8.2888E-08	ATXN2	-0.75891762	1.0106E-06
BCL2	0.54704267	0.00084573	ARL3	-0.75839773	3.379E-07
PPP4C	0.546927	3.4521E-06	DENND2D	-0.75807856	0.02943771
ZADH2	0.54670219	0.01072958	PUM2	-0.75627603	2.4767E-05
FAU	0.54617358	8.7114E-18	MROH6	-0.75619681	0.04776612
SORL1	0.54583265	0.03327326	P2RY13	-0.75566199	3.9675E-06
NUDC	0.54523601	6.9536E-05	NFATC3	-0.75445851	0.00015149
TRAF5	0.54488577	0.00385984	ZNF426	-0.75420168	0.0335023
MFSD10	0.54479313	0.00134348	SCAF1	-0.75303127	0.02180406
GABPB1	0.54422708	0.00021476	GDE1	-0.75295146	4.0637E-08
FLAD1	0.5438383	0.03455564	GADD45A	-0.75281385	0.00040323
AIMP2	0.54376693	0.00403406	TRIM36	-0.75239978	0.04645652
DNAJC15	0.54358407	5.3907E-11	ZSWIM8	-0.75181539	0.00127765
CSNK1D	0.54323299	7.4684E-05	ATG14	-0.75167031	0.00143431
PITPNA	0.5431207	0.00011977	TNRC18	-0.75059368	1.8949E-05
SLC25A29	0.54310096	0.04954491	MDC1	-0.75058715	0.02582585
AKR7A2	0.54299987	0.00122197	KANSL1	-0.74908938	2.0525E-06
RPS25	0.5427655	6.549E-18	SLC20A1	-0.74819155	1.8457E-07
SULF2	0.54256365	0.00209171	TCEAL1	-0.74549858	8.3923E-05
AK2	0.54191125	0.00010654	CHD9	-0.74343218	3.0716E-09
PSMB7	0.54169567	1.8392E-07	ERCC3	-0.74283838	0.00153069
RPL39	0.54132727	3.9925E-14	ZFAND2A	-0.74239734	0.01270328
ZNF385A	0.54113586	0.00011244	PPARD	-0.74213947	0.00189798
RALY	0.54111811	8.6184E-06	LYL1	-0.74177022	2.1704E-06
COPS3	0.54098524	4.9311E-08	ARFIP1	-0.74085972	2.3197E-06
HECTD1	0.54086219	0.00081884	MGA	-0.74082768	0.00953431
PDCD6	0.54052919	7.2728E-06	ZNF25	-0.74067893	0.03654537
BTG1	0.5403622	0.0006654	TNRC6A	-0.74057866	3.3216E-06
SOX4	0.54034111	0.00065653	NSL1	-0.74013345	9.9422E-09
NDUFA12	0.54030173	9.8115E-09	USP40	-0.73896658	0.0198639
ACO2	0.5402462	6.056E-05	KIAA1468	-0.73893977	0.0148611
MDP1	0.54022785	0.02335887	KDM3A	-0.73889378	0.00331329
CRELD2	0.5401837	0.00615705	ERCC6L2	-0.7382132	0.001025
BCL7C	0.54009333	0.00023732	WDR26	-0.73778053	1.2195E-07
ACTN4	0.53996532	4.6253E-06	NUDT4	-0.73772319	0.00010686
GLIPR1	0.53940663	4.436E-08	CPM	-0.73747881	1.6769E-13
POLR2E	0.53878273	4.1218E-07	CEP44	-0.73664236	0.02265774
NAA38	0.53849889	2.0762E-08	AFF4	-0.73588061	9.7038E-09
LYRM1	0.53844888	0.03199924	EPB41L2	-0.73531199	0.00226696

SRA1	0.53766912	2.0034E-08	RXRB	-0.73525731	0.00122913
JAZF1	0.53733414	0.02726583	ZNF37A	-0.73496107	0.00744568
TIMM17A	0.53732123	1.4861E-06	RP11-214O1.3	-0.73430476	0.0060824
KIF2A	0.53719524	0.00028462	ANKDD1A	-0.73430133	0.00736912
COX4I1	0.53717399	2.3691E-15	DCAF5	-0.73428518	0.00039311
ATP6V1G1	0.53704104	8.0826E-14	MBIP	-0.73416868	0.000952
HDDC3	0.53701456	0.00080988	ZNF746	-0.7340607	0.04817152
BLOC1S1	0.53652985	3.8925E-08	NT5DC2	-0.73367274	0.00037792
GPS2	0.53612935	0.0107175	ACADSB	-0.73285844	0.03857157
GTPBP1	0.53611722	0.01631087	CLN5	-0.73217555	1.1965E-06
SEC61G	0.53592485	1.833E-07	ERMAP	-0.72954415	0.00680056
UBXN2A	0.53589519	0.02564975	TMEM173	-0.72895017	1.226E-10
IARS	0.53585902	0.00792999	LARP4	-0.72839205	0.00086224
IDH3B	0.53545536	0.00033454	ZMYM3	-0.7282184	0.01934773
RPS13	0.53502518	4.8725E-15	PHF23	-0.72735448	0.03606003
ZNF557	0.53454423	0.04111141	PI4K2A	-0.72724512	0.00203125
NFE2L3	0.53442841	0.00084428	FAM20C	-0.72706471	6.8146E-05
U2AF1L4	0.5343174	0.00074639	KRIT1	-0.72646324	1.5194E-05
NETO2	0.5343021	0.00473584	IQCE	-0.7262435	0.02263699
PTPRE	0.53385338	8.1034E-09	FAM221A	-0.72583455	0.0401971
ACSF2	0.53373825	0.01309067	SPECC1L	-0.72548172	0.02033321
CNBP	0.53331621	1.1285E-09	LMF1	-0.72484603	0.02531644
ATP5C1	0.53307465	7.2625E-09	NCKIPSD	-0.7234081	0.02349082
KLHDC4	0.53291485	0.03402899	FAM105A	-0.72289334	3.485E-10
MRPL21	0.53227613	7.0833E-07	CRYL1	-0.72288115	8.3903E-05
GTF3C6	0.5322086	2.2922E-06	MAU2	-0.7227037	0.00114306
RPF1	0.53205657	0.00787577	INPP5D	-0.72239423	1.5037E-06
PHB	0.531932	4.1595E-09	ZMYND11	-0.72235288	0.00016691
SUCLG1	0.53191056	7.882E-09	RABGAP1	-0.72210709	0.00016416
ODF3B	0.53157835	0.0005835	PATZ1	-0.72197513	0.01773954
EIF3H	0.53146567	2.706E-22	USP53	-0.72182608	6.1437E-05
INPP5K	0.53142493	0.01795328	RP11-334C17.	-0.72163678	0.04895465
ITGB2	0.53075724	2.0894E-06	VMO1	-0.72153755	0.00086817
ARHGDIB	0.53024076	3.1295E-11	SHMT1	-0.72150618	0.00019326
MRS2	0.52985119	0.02330383	CKAP5	-0.72104449	0.00018778
TMEM189	0.52951152	0.00721455	ANKRD50	-0.72075536	0.00783996
B3GALT6	0.52882075	0.00358215	ASB1	-0.72060768	0.03252983
PLRG1	0.52880608	0.01152459	FEM1B	-0.72016871	1.1748E-05
POLR2D	0.52869201	0.0099042	HACD3	-0.71972416	2.6979E-05
MRPS33	0.52861915	8.7813E-07	RAMP2	-0.71971539	0.0449293
SUMO1	0.52855107	1.0165E-05	ZBTB20	-0.71971129	0.00076478
ATXN1L	0.52791472	0.02268862	TRIP4	-0.71946303	0.00085851
C9orf142	0.5274099	8.0965E-06	TRIP11	-0.71934221	3.3399E-05
HAX1	0.52733497	2.2872E-07	KIN	-0.71908284	5.1057E-07
AP1S1	0.52726876	0.00060592	PNRC1	-0.71838755	4.702E-09
PHYKPL	0.52622177	8.0592E-05	PARP10	-0.71832908	0.00012269

RAB29	0.52618841	0.04043633	ALAS1	-0.71815224	0.00182673
UBE2E2	0.52592955	3.9196E-05	RNF169	-0.71799918	0.00022713
SERF2	0.52562275	9.5037E-11	ARID1A	-0.71779055	7.6509E-06
C12orf10	0.52521554	0.00076487	GABPA	-0.71762833	0.00056862
CSF2RA	0.52517712	1.9467E-05	ICE2	-0.71728988	0.00021706
EMC8	0.52513273	0.00039482	SAP25	-0.71712837	0.03622044
ANKLE2	0.52485269	0.00285658	FRA10AC1	-0.71705433	8.4632E-05
IFI30	0.52469886	4.9718E-05	ATP6V1C1	-0.71654837	4.2528E-08
OFD1	0.52405123	4.0049E-06	DHX33	-0.71653692	0.03495912
PTPN2	0.52387896	3.6182E-06	MAN2B2	-0.71633032	0.00068537
PRPF4	0.52360908	0.00714452	ZMYM4	-0.7161977	0.00039303
CHRAC1	0.52355323	0.00095828	CEP250	-0.71495542	0.03452343
GNG5	0.52325275	4.3184E-11	DNAJA1	-0.7147745	1.6085E-05
PRELID3B	0.52289657	5.794E-05	RNF217	-0.71454895	0.00503562
RIOK1	0.52209354	0.00019148	PIP4K2B	-0.71388872	0.01173917
SVIP	0.52201713	0.00836958	SH3PXD2B	-0.71376658	5.3004E-05
NDUFA2	0.52191883	7.5168E-08	TMEM104	-0.712667	0.01100761
ATAD2B	0.52183931	0.01662988	UTRN	-0.71237046	1.4373E-10
PSMD3	0.52163223	0.00011302	MRPS31	-0.71232239	0.00010393
ACSL1	0.52129255	0.01786831	GABPB2	-0.71218577	0.0019708
PEA15	0.52105215	0.00149403	STOM	-0.71165792	5.4239E-08
PABPN1	0.52105052	1.673E-05	MT1X	-0.71156952	0.00010724
C22orf39	0.52094704	0.00483211	TMEM222	-0.71135472	0.00960553
UXT	0.52080621	8.54E-12	FNDC3B	-0.7102079	2.5141E-05
NDUFB10	0.52069504	2.8057E-09	PHYH	-0.70934449	4.9491E-05
C12orf49	0.52058204	0.033829	FAM179B	-0.70916974	0.00291493
DDB2	0.52050141	0.01131188	CLUAP1	-0.70916016	0.04083844
CPNE3	0.51995173	0.00011748	ARHGAP17	-0.70900754	0.00268187
RBBP4	0.51993886	4.2499E-07	PEX1	-0.70752477	0.0292902
COA1	0.51845637	0.00255592	FDX1L	-0.70695149	0.00431847
SSSCA1	0.51823622	0.00137712	ING1	-0.70655327	0.00023793
NDUFB4	0.51817617	5.4147E-09	P2RY6	-0.7064797	6.0218E-05
EMC6	0.51800714	9.9743E-05	RP11-390P2.4	-0.70597759	0.00156911
RASSF3	0.51740526	0.00056435	SPIN1	-0.70575989	8.4669E-06
RBX1	0.51694777	1.0034E-07	MUM1	-0.7052976	0.00144986
FBXW7	0.51689417	0.00054161	PREPL	-0.70388452	0.00573652
EBNA1BP2	0.51674499	0.0002308	HERPUD1	-0.70358944	0.00063
NRROS	0.51651681	0.04915936	C7orf26	-0.70263652	0.0204417
OAT	0.51632707	7.863E-05	FAM208A	-0.70221231	2.4894E-07
BANF1	0.51631416	5.7638E-10	MFN2	-0.70204898	0.00424845
CIAPIN1	0.51583908	0.00526466	CD83	-0.70167862	1.3375E-07
PSMD6	0.51537618	1.571E-07	KRCC1	-0.7014864	0.00019065
HPCAL1	0.51536867	0.00221835	PHKB	-0.70072641	2.696E-06
IPO5	0.51533666	0.00223452	ILF3-AS1	-0.70059237	0.00432025
MTHFR	0.51523146	0.00527162	AP5Z1	-0.70012786	0.00147422
HMGB2	0.51521501	5.2807E-06	FAM63B	-0.6997445	0.00337599

RUFY3	0.51517756	0.00680165	FCGRT	-0.69907419	9.0259E-13
SSBP1	0.51428303	1.4105E-09	ZNF414	-0.69839637	0.01706564
CYB5R4	0.51427337	0.0001449	DISC1	-0.69800676	0.00963171
RPL22	0.51423187	9.0495E-21	GNS	-0.69708232	0.00012129
UFD1L	0.51360196	0.0003693	KLF9	-0.69655787	1.1709E-06
RPL26L1	0.51351991	0.00013469	ZMAT3	-0.69606411	0.0013446
MAP3K8	0.51350989	0.00053457	CYB5R1	-0.69476912	8.7933E-08
UCKL1	0.51324481	0.03200295	GPD2	-0.69454285	0.02751603
CSNK2B	0.51319428	7.5912E-09	HSP90B1	-0.69395738	5.8761E-23
RPL7	0.51318564	4.6944E-10	FAM53C	-0.69365851	0.00027948
ESD	0.51307695	1.1774E-06	PIAS1	-0.69345273	4.2388E-10
RSL1D1	0.51292589	1.0087E-14	IFT22	-0.69339739	0.024166
FAF2	0.51282141	0.0115108	GPR162	-0.69332523	0.03749212
EPM2AIP1	0.51277269	0.00126478	ORC3	-0.69326385	0.00118884
IMPDH1	0.51222073	0.02873814	MAP3K4	-0.69316074	0.00336865
SMARCB1	0.51221088	0.00685356	CDC25B	-0.69216786	0.04106986
SNRNP35	0.51201658	0.0478808	ZSCAN18	-0.69166675	0.01318917
UBE2D2	0.5116635	5.9631E-07	C7orf60	-0.69143844	0.02330383
PSMA6	0.51150926	0.0437125	ATRN	-0.69070393	0.03508948
RPL17	0.51127124	0.0006089	DDX3Y	-0.69044359	0.00157805
SRP72	0.51122165	1.1919E-09	IDI1	-0.69015761	1.5624E-07
LEPROTL1	0.51046758	4.5802E-07	NEK9	-0.69008237	0.00166639
CDK2AP2	0.51020768	0.00016508	MLLT1	-0.68932485	0.00796428
POLDIP3	0.51011807	0.01607742	SLC22A18AS	-0.68919573	0.0179643
DDT	0.5100679	1.2956E-07	FUT11	-0.688767	0.00824118
PQBP1	0.50975792	1.3415E-06	HIRA	-0.6886262	0.02671247
ISOC2	0.50883127	0.0014387	CYBB	-0.68860648	6.9841E-10
RUVBL2	0.50830907	0.00927493	LAMP1	-0.68860471	0.00167091
ITGAE	0.50827139	0.00026818	GEMIN2	-0.68812899	0.03563554
MGAT2	0.50825908	0.00067916	CLK1	-0.68774327	4.1002E-10
NSFL1C	0.50800335	1.1417E-05	SPRY2	-0.68744957	0.0144252
LAS1L	0.50791941	0.00703471	NUP43	-0.6863133	0.0110255
CLTB	0.50743625	0.00346734	C1orf54	-0.68622451	5.8262E-07
TOB1	0.50699585	0.00485635	CD69	-0.68591078	0.0170669
DERA	0.50699228	0.00565426	ZNF768	-0.68575887	0.03811385
SF3B6	0.50654122	0.00011673	NHSL1	-0.68556767	0.02593676
RPS26	0.50621227	4.5222E-21	HHLA3	-0.68386374	0.03378441
BRE	0.5057989	0.00026676	CEP120	-0.68371427	0.03270878
DEK	0.50461438	4.9234E-10	PSAP	-0.68334279	3.8819E-11
PHB2	0.50459506	8.7624E-07	ZNF609	-0.68303805	1.7541E-05
RP11-76E17.3	0.50423656	0.02065083	SESN3	-0.68236053	0.00023519
CHD1L	0.50415805	0.01607742	LRRC25	-0.68176306	2.2297E-06
RAPGEF1	0.50410689	0.00677202	DNAJB6	-0.67766683	2.427E-17
RNF41	0.50402417	0.00358215	CTR9	-0.67704934	0.00030739
VHL	0.5039711	0.00063602	CYSLTR1	-0.67689857	0.00030178
MSRA	0.50362965	0.00024785	ZNF496	-0.67671758	0.02166796

C9orf72	0.50331398	7.6967E-06	NELFA	-0.67469262	0.01406217
EMILIN2	0.5030555	1.2441E-05	PGRMC1	-0.67460093	8.1326E-07
BACH1	0.50303581	0.0008597	NUDCD3	-0.67448562	0.00513817
NCAPD3	0.50289404	0.0425885	TATDN3	-0.67426703	0.00067001
MRPL15	0.50279169	0.00231282	RPS6KA5	-0.6738858	0.00259327
POP5	0.50277459	0.00029171	TLR1	-0.67376815	0.00012419
ZFAND1	0.50266355	0.00023933	SCAF8	-0.67366905	0.00194724
COMMD7	0.50239486	1.3012E-05	DOPEY2	-0.67351872	0.02491053
ABCF2	0.50233171	0.04182894	EPS15	-0.67302768	0.00030223
JAK1	0.50229074	2.6972E-06	RBBP8	-0.67261254	0.00391469
MRPS25	0.50201833	0.00082964	CFAP97	-0.67261028	0.00040068
COX8A	0.50185308	5.597E-10	ST7	-0.6716285	0.03671984
LAMTOR5	0.50181938	5.6769E-11	FAM214A	-0.67148727	0.00941368
ZDHHC12	0.50146895	0.00011779	CCP110	-0.67093884	0.01697172
SOCS4	0.50142927	0.01279539	MOSPD1	-0.66862458	0.0154222
UPF2	0.50053943	0.00497195	ABHD18	-0.66854743	0.0339946
PIN4	0.5003994	0.00017855	SOCS6	-0.66829444	0.03095528
MAP2K2	0.50025544	9.8644E-05	PRKAB1	-0.66789857	0.01354468
NDUFAB1	0.50018456	6.4919E-07	CETN3	-0.66715198	0.00932084
TAF15	0.49980373	0.00018451	TJP2	-0.66701627	0.00218584
SCNM1	0.49962266	0.00028694	LPIN2	-0.66641734	1.8464E-05
CHST11	0.49945431	0.00019572	VPS13B	-0.66618046	0.00097095
CDK5	0.49874989	0.02877755	MAP3K5	-0.66616357	0.00320383
POLE4	0.49853512	0.00012015	TM6SF1	-0.66502215	7.9075E-05
ANP32B	0.49833072	1.8554E-07	PIAS4	-0.664245	0.03109723
ENTPD4	0.49791615	0.03348607	AZI2	-0.66406563	9.0738E-05
RPL27A	0.49736998	3.1219E-10	SIRT1	-0.6635411	1.2261E-05
GNL3	0.49729643	4.1094E-05	SRGAP1	-0.66227019	0.0335023
DNAJA2	0.49691751	1.8777E-06	GPR137B	-0.66217709	0.00127755
TRAF7	0.49668975	0.00946493	SCAPER	-0.66212823	0.00202545
SEPW1	0.4963987	0.00172787	KLHL8	-0.66141898	0.00154086
PMPCA	0.49610863	0.03227728	DCTN4	-0.66132041	1.3175E-06
ROMO1	0.49554842	6.9719E-08	PRIMPOL	-0.66097778	0.01672166
FAM102B	0.49506657	0.00660596	EXOC1	-0.66043128	1.4257E-06
GLRX5	0.49433087	7.6343E-05	MILR1	-0.65964225	1.0275E-06
RPL36AL	0.4942922	4.0075E-10	SLC25A46	-0.65900794	0.00175447
RBBP7	0.49427259	0.00029517	MFSD7	-0.65844652	0.00624718
C14orf166	0.49394556	2.5107E-07	HES4	-0.65839722	4.0695E-05
PNN	0.49388947	3.1128E-07	ZZZ3	-0.65831601	0.01815223
DPM1	0.49384807	0.00095599	EIF4A2	-0.65756467	2.8E-10
C19orf53	0.49366191	8.1239E-13	LTBP3	-0.65737051	0.00109913
TXNDC9	0.49363601	0.01509453	TRIB1	-0.65721241	0.000211
NDUFC2	0.49341429	6.848E-07	ERVK3-1	-0.65681678	0.00084654
MRPL51	0.49306021	3.76E-07	CELF1	-0.65660742	7.78E-08
TMEM205	0.49297512	0.00023276	ST3GAL5	-0.6562047	0.00216604
ELOVL5	0.49296721	0.00209743	CALU	-0.65570219	1.8759E-07

CSK	0.49279095	0.00053533	APPBP2	-0.6552749	0.00533807
CD53	0.49234179	3.1363E-09	ASTE1	-0.65496509	0.01983484
HNRNPA1	0.49231293	7.4366E-12	ARID1B	-0.65486001	2.7832E-06
GPAT4	0.49135193	0.0018953	CCDC112	-0.65469673	0.00030886
SNX8	0.49103178	0.00037058	PPM1N	-0.65386044	0.01263207
SLCO3A1	0.49102561	0.00745161	BHLHE40	-0.65372519	9.3848E-05
TOMM5	0.49095819	0.00107595	VASH1	-0.65361257	0.00619964
DUS1L	0.49084771	0.00210309	SOGA1	-0.65255206	0.02668069
HARS	0.49037711	0.0058396	EVA1B	-0.65229042	2.846E-05
NUDT5	0.49030622	1.8369E-05	CC2D1A	-0.65129789	0.02070386
RAB11A	0.49001695	0.00014424	LRP1	-0.65006198	0.00158832
PSMG4	0.48941772	0.01319533	GORASP1	-0.64971434	0.02934442
MZT2B	0.48896735	1.1195E-05	MGLL	-0.64921348	0.00825275
TMA16	0.48889791	0.00059079	MDFIC	-0.64869598	0.00090335
GLTP	0.48777523	0.00133769	CLTC	-0.64829258	8.7789E-07
UBE2A	0.4875577	3.0184E-08	OAS1	-0.64822015	0.0055135
DNAJB12	0.48709661	0.00019487	DUSP2	-0.64795715	0.00068639
DRAP1	0.48706979	9.2559E-10	MVB12B	-0.64757638	0.00242032
SPCS1	0.48702476	1.1831E-07	FAM174A	-0.64753648	0.01787011
PRKAR2A	0.48633501	0.00203943	TBP	-0.64692268	0.04885116
CXorf21	0.48607671	0.0462224	ARL11	-0.64605133	0.00612729
RNF168	0.48570551	0.00651251	NBPF11	-0.64487947	0.04777878
NOLC1	0.48561817	0.01155451	BAIAP2	-0.64484576	0.00014194
HLA-DQA1	0.4855256	0.00069785	TMEM65	-0.6443942	0.00036631
CNOT6L	0.4851802	0.00153664	C16orf52	-0.64336188	0.033829
TNFSF13B	0.48467754	1.3136E-10	TADA2B	-0.64323464	0.04040057
NUP93	0.4841394	0.00313516	KNOP1	-0.64267252	0.01290119
SLC25A11	0.48377844	4.0006E-05	ARHGAP25	-0.64245249	0.00912026
WDR18	0.4832459	0.00973559	BLMH	-0.64136941	0.00082064
SKA2	0.48323787	0.00457297	MOSPD2	-0.64088394	0.0049759
PSMC4	0.48313045	7.2208E-05	RNF146	-0.64081805	0.00378067
SETD9	0.48277554	0.01431411	C11orf68	-0.6404384	0.03370413
MAGOH	0.48276269	3.6849E-09	GTPBP3	-0.63999531	0.04109573
HIGD2A	0.48219604	8.4881E-08	SUSD6	-0.63899419	7.7283E-05
SMAP2	0.482137	1.2683E-08	ZFYVE21	-0.63865896	0.00559066
PSMB8-AS1	0.48190776	0.00303732	SPRYD7	-0.63830626	0.02017534
SLC31A2	0.48154018	2.9158E-05	CTD-3252C9.4	-0.63825954	0.0030922
ICAM2	0.4812456	0.00680056	ZNF638	-0.63681906	6.6266E-07
CHMP4B	0.48122551	0.00509704	NUP85	-0.63665618	0.02250714
STOML2	0.48093521	2.1838E-06	C11orf54	-0.63628779	0.00359793
TMEM230	0.48035058	2.283E-08	SIPA1	-0.63610383	1.2874E-07
ERCC1	0.48027052	4.9945E-07	RBMS1	-0.63534875	3.7212E-06
SNRNP40	0.47995571	0.00075609	ANKIB1	-0.63454328	0.00267937
MALAT1	0.4797167	1.5915E-05	PTPN9	-0.63435305	0.00019565
SS18L2	0.47936396	8.1884E-05	TPP1	-0.63407753	7.9121E-16
AIFM1	0.4793281	0.00458085	BCOR	-0.63396482	0.02158205

RAB32	0.47888803	0.00493367	ABCD4	-0.63278544	0.00123767
CNOT11	0.47850473	0.01685912	OSBPL3	-0.63169965	0.01253677
MFSD14A	0.47842374	0.02045369	NCOA6	-0.63106235	0.00625571
CHMP2A	0.47822985	9.4313E-05	PLEKHA2	-0.62976824	8.1526E-07
ASNA1	0.47804288	0.00033757	CKS2	-0.62953485	0.00262149
CD47	0.47747239	5.7961E-05	CNST	-0.62903962	0.01999431
RPS6KA4	0.47676918	0.00019339	TBC1D12	-0.6288007	0.00240543
MRPL13	0.47657374	0.00044661	RFNG	-0.627106	0.0023894
GCA	0.47649313	4.5499E-06	DNASE1L3	-0.62694671	0.00364142
NMI	0.47623386	0.00074994	FAM168A	-0.62611854	0.04038783
ZNF277	0.4761871	0.00604308	MSRB2	-0.62440056	0.00020765
PITPNC1	0.47578358	0.04083239	PDIA4	-0.62371687	3.2067E-05
SNRPB2	0.47577633	8.0762E-08	QKI	-0.62367757	1.2111E-09
CDC26	0.47552391	0.00563575	RP11-69E11.4	-0.62294935	0.04110095
STK32C	0.47544316	0.01188726	GOLGA1	-0.62272636	0.02350201
GRK6	0.4753438	0.00422026	EOGT	-0.62266573	0.02794969
DDX41	0.47483034	0.01096168	FCGR2A	-0.62194682	4.5899E-08
THG1L	0.47474736	0.01285727	MRPL46	-0.62118914	0.01350162
NDUFB3	0.47448451	3.795E-06	TXNDC16	-0.61971465	0.00013094
DCTN2	0.47448327	9.8086E-05	SMAD4	-0.61922539	0.00282454
RRP7A	0.47433557	1.9654E-05	COQ7	-0.61917262	0.00227251
SEC11C	0.47425751	0.00071875	MTMR10	-0.61912548	0.01281287
CRIPT	0.4739955	0.00547178	SH3YL1	-0.61871737	0.00236865
PLAUR	0.473862	0.00714013	JADE1	-0.61825163	0.00889807
ATP5S	0.47368611	0.00295904	CSTF1	-0.61764292	0.03994006
CDK16	0.47271417	0.01280762	TRAK2	-0.61748494	0.0056554
S100A11	0.47222682	0.00055253	PHF3	-0.61669949	8.7602E-10
MTHFD2	0.47200425	2.8734E-10	ARNT	-0.61605039	0.00954816
EEF2	0.47199949	1.1567E-16	ERCC5	-0.61510992	0.02037951
TMEM50B	0.47175292	0.00165085	NXF1	-0.61411407	0.00102574
UAP1	0.47151385	0.01265352	CLCN7	-0.61390133	0.00010062
PSMA3	0.4715086	0.00012811	PSME4	-0.61348346	0.0004234
VPS25	0.47134673	0.00234176	DEDD2	-0.6133387	0.01011554
ZNF721	0.4713431	0.01886053	RRAD	-0.61332079	0.02160368
LRRC59	0.47105016	0.00069549	TMEM69	-0.61328719	0.00231618
SRPRA	0.47097605	6.6101E-05	ZCCHC2	-0.6125137	0.00260071
ABRACL	0.47089243	2.2348E-05	FER	-0.61178095	0.00797007
DOCK5	0.47021335	0.00035454	SERINC1	-0.61158236	1.1732E-09
PGLS	0.47003718	5.5738E-05	ANKRD17	-0.61099419	1.3528E-05
SIVA1	0.46999813	3.4007E-07	IRF2	-0.6109012	7.2125E-07
FBXO21	0.46972164	0.04379339	SP1	-0.61068919	0.00907269
WHAMM	0.46960391	0.00136386	TMEM107	-0.60988229	0.00053488
MRPL9	0.46949227	0.01548284	PAPD4	-0.60953444	2.0978E-05
RPL9	0.46945282	1.3108E-09	DOK1	-0.60901367	0.00490728
EIF2D	0.46913825	0.01816726	ENGASE	-0.60867488	0.00914841
PNPLA8	0.46753963	0.00036939	SLC35A5	-0.60744154	0.02740218

DUSP12	0.46731033	0.01984811	PER1	-0.60737453	2.132E-06
NIP7	0.46720415	0.02201403	MIB1	-0.60731614	0.04777878
TTC14	0.46697224	0.0002505	CYBRD1	-0.607178	0.00011388
NRDC	0.46686718	0.00050139	CWC22	-0.60682277	8.9613E-06
C17orf89	0.46684922	0.00114001	FKBP4	-0.60655147	0.00364456
SLC25A28	0.46676994	0.01223178	BAZ2B	-0.60626129	8.2212E-08
SRSF9	0.46649112	0.00107411	CDC27	-0.60566145	0.00069311
INPP5F	0.4658474	0.03849942	CFAP36	-0.6056122	0.01920415
EIF4EBP2	0.46575061	0.00035327	PHC3	-0.60559882	0.00351031
TMEM258	0.46533394	2.4137E-08	NVL	-0.6054096	0.02957998
GID8	0.46513449	0.00174512	MEPCE	-0.60531296	0.00085789
HACL1	0.46505523	0.03026372	SECISBP2L	-0.60400256	0.00044686
KXD1	0.4650376	0.00032469	BDH2	-0.60399573	0.02316652
RP11-426C22.	0.46466836	0.04145504	NCK1	-0.60393923	2.5015E-05
KARS	0.46457758	0.00012152	CTCF	-0.60358759	0.00153876
PFDN5	0.46444681	3.6888E-10	SGK1	-0.60278019	0.00046211
MRPS12	0.46381131	0.00530582	NLRP1	-0.6026471	0.00319901
CARHSP1	0.46308138	0.0175486	CD36	-0.60160387	8.2259E-07
VDAC3	0.4621697	7.8737E-05	TMEM2	-0.60067672	0.02104466
TIMM50	0.46209897	0.00658687	IL12RB1	-0.60064287	0.0023327
COX14	0.46119508	1.96E-06	MLLT10	-0.60034702	7.1511E-05
IKBIP	0.46106077	0.00577891	CDK5RAP2	-0.59994397	0.00221716
COX17	0.46001665	1.5881E-08	CBX1	-0.59953481	0.00082064
PSME3	0.45928719	0.00799125	VGLL4	-0.59912067	0.02031622
SYF2	0.45905812	1.3146E-07	SNX19	-0.59906494	0.01685912
MLF2	0.45898271	1.0799E-05	SETDB2	-0.59796714	0.00269333
C19orf70	0.4588113	1.6727E-09	GPANK1	-0.59771066	0.00102304
UBA2	0.45866677	0.00266786	ZNF280D	-0.59652826	0.00073847
SSR2	0.45853062	2.7315E-10	PCF11	-0.5957261	4.0955E-06
TRIM22	0.45827993	2.5376E-06	TMEM192	-0.59483507	0.00745161
HLA-F	0.45801376	0.00049299	ASXL2	-0.59464375	0.00097459
MAZ	0.45799332	0.00686378	SKI	-0.59429223	0.00424339
SEH1L	0.45764764	0.01247016	CLN6	-0.59379003	0.0043266
CRK	0.45738444	0.0365139	CTSA	-0.5936512	1.3214E-09
SSR3	0.45735164	4.3149E-05	RBM26	-0.59256325	2.9244E-06
NDUFAF2	0.45730078	0.00557151	SLC17A5	-0.59253593	0.01143896
MRPL23	0.45711621	0.00357698	KLHL12	-0.59239668	0.04429171
CPQ	0.45641369	0.00419896	FAM127B	-0.59237831	0.02024526
TMEM183A	0.45602931	0.00084412	NINJ2	-0.59232586	0.00179106
CUX1	0.45577686	0.00015816	CNTLN	-0.59227326	0.00024904
MAP3K13	0.45570403	0.00012234	PBXIP1	-0.59165839	0.0041569
MRPL24	0.45525887	0.00431847	VPS54	-0.58931533	0.02068353
TIMM8B	0.45513641	5.4021E-05	ZBED3	-0.5890518	0.04909684
SP110	0.45484487	0.04002912	FAM73A	-0.58879269	0.00978693
GARS	0.45461333	0.00275799	SLC37A2	-0.58809282	0.02751065
MPND	0.45446668	0.03456817	ANKS1A	-0.58801688	3.0072E-05

PPIA	0.45431573	4.9194E-16	TMEM63A	-0.58749368	0.04645652
SLIRP	0.45416574	1.8738E-05	NBPF10	-0.58740118	0.02000535
RSL24D1	0.4541238	7.5102E-09	TBC1D9B	-0.58725673	0.00267835
RPL13A	0.45400551	6.6682E-14	GAB2	-0.58674206	0.00085808
LAMTOR1	0.45393849	7.3802E-07	ARID4A	-0.58624866	8.0345E-08
TXNDC17	0.45339139	7.1433E-05	PAG1	-0.58573047	9.3202E-06
ALG5	0.45338917	0.00025769	STAG1	-0.58453185	1.7963E-05
PGM2	0.45330189	0.0009575	ATP6V0A1	-0.58423773	0.00223281
ATP5E	0.45292377	4.6067E-11	CHD8	-0.58418132	0.00434308
HLA-DRB1	0.45273198	0.03843644	BST2	-0.58296651	1.1979E-16
RPL12	0.4525934	5.1179E-13	THAP6	-0.58260036	0.01730072
POLR2J	0.45236909	1.645E-06	KDM5B	-0.58155662	0.03449644
PEF1	0.45233227	0.00465708	GALK2	-0.58131322	0.00620472
PCNP	0.45192678	5.7025E-07	GIGYF1	-0.58042102	0.01280664
CHCHD5	0.45181938	3.0121E-05	PET117	-0.58040935	0.03116042
COX6B1	0.4516719	2.9288E-15	NBPF19	-0.5800484	3.4163E-06
TNFRSF10B	0.45165226	5.9037E-05	MTIF2	-0.57987154	0.00367504
TRNAU1AP	0.45103716	0.00794822	LSM14B	-0.57960494	0.01777502
OXA1L	0.45084648	6.8498E-07	SGTB	-0.57941919	0.01568873
ISCA1	0.45056244	0.000243	RNF40	-0.57912988	0.0232438
RTN1	0.44967546	0.01257794	LINC00847	-0.57911942	0.01278102
LGALS9	0.44943032	3.7569E-07	NUP58	-0.57792855	0.00013242
RPL38	0.44928152	1.0642E-14	KIF20B	-0.5770781	0.00313914
HNRNPD	0.4490401	3.731E-07	PIGC	-0.57699132	0.00280741
COPS8	0.4489985	0.01079565	KIAA1191	-0.57656209	0.00275411
PNISR	0.44855932	1.2775E-05	TMEM187	-0.57601329	0.04189682
CCDC107	0.44830284	0.00078426	NRIP1	-0.5757529	0.00033232
POLR3E	0.44778683	0.03956667	PPP1R9B	-0.57528685	0.00754557
HNRNPH2	0.44727304	1.5904E-06	AHSA2	-0.57524961	0.001156
GPI	0.44722755	0.00685356	MAML1	-0.575064	0.02941188
EBP	0.44676457	0.01014853	FAM126B	-0.57463029	0.02582379
PRKRA	0.44656633	0.00426266	CEP170	-0.57447683	1.0685E-08
RBCK1	0.4464921	0.00029112	SNX2	-0.57446424	1.9915E-05
ATP5J	0.44599787	1.2761E-09	DYRK1A	-0.5737783	9.9743E-05
VDAC2	0.44596912	1.8507E-06	CCDC117	-0.57363898	0.01969311
NDUFB8	0.44590996	2.2328E-05	ZNF136	-0.57347704	0.00279352
DPH3	0.44566163	0.03018397	AGTRAP	-0.57345154	0.00054063
CCT2	0.44537903	0.00017884	FAM46A	-0.57329968	0.00152722
SHFM1	0.44529718	4.6553E-07	RUFY2	-0.57313716	0.01609228
RCN2	0.44430753	0.00135833	SMIM15	-0.5726929	0.00177374
ARRDC1	0.44422198	0.00013641	GALM	-0.57256267	0.00116355
NOP14	0.44417373	0.03072753	SLC15A3	-0.57235307	9.6236E-06
TNIP1	0.44408374	0.03944093	RP11-452F19.	-0.5717647	0.03461811
HDGF	0.44404309	8.104E-05	GATAD1	-0.57141253	0.00010793
PRR13	0.44361995	3.8617E-09	ATF6	-0.57100486	6.2327E-05
PIGX	0.44326266	0.02572406	LGALS8	-0.57087094	7.1612E-05

STAT3	0.44311027	0.00047939	CD2AP	-0.57076776	4.2349E-05
GDI2	0.44309434	2.4846E-10	UNC93B1	-0.57062818	3.372E-05
MRPL43	0.4425068	7.7669E-05	ARHGEF40	-0.56896545	0.01131107
RPLP1	0.44182754	6.4419E-10	IRF8	-0.56851616	9.7135E-05
OST4	0.44149255	6.2806E-11	KAT6A	-0.56841802	3.1231E-06
ARPC5L	0.44138392	0.00270809	YWHAH	-0.56619004	3.2266E-05
RFK	0.44081212	0.02882108	SNX13	-0.56615132	0.00795662
KLHL5	0.44041192	0.00312029	RCHY1	-0.56599019	0.00139772
HADHB	0.4397304	2.3375E-05	SAYSD1	-0.56518821	0.04607719
ATXN2L	0.43776044	0.0140437	PIK3R1	-0.56486015	2.9147E-05
RPL21	0.4375056	2.9797E-15	ATF2	-0.56480917	0.00489602
PPP2R2D	0.4369642	0.02382343	RNF38	-0.56375682	0.02365575
CARS2	0.43679003	0.00559997	SLC31A1	-0.56355036	8.2235E-06
TUBA1B	0.43676339	1.7596E-05	ICOSLG	-0.56293999	0.0011411
FAM49B	0.43612099	4.9775E-10	TAGAP	-0.56287947	1.4691E-06
EIF4G3	0.43597746	0.0022696	PIK3AP1	-0.5627504	0.00119771
PRPF31	0.43594907	9.0595E-05	TBL2	-0.56233433	0.00413545
CINP	0.43582547	0.00318791	SLC5A3	-0.56222319	0.04781364
RPS27A	0.43515881	3.8643E-10	ZRANB1	-0.5617177	0.00023442
GPATCH11	0.43498997	0.02110074	CREBL2	-0.56149343	1.4069E-08
FIS1	0.43496747	6.8498E-07	USP9X	-0.56137862	1.4998E-08
PPP1CB	0.43489485	2.0814E-05	UBQLN2	-0.56119375	0.0022026
QTRT1	0.43444139	0.01771358	SLC33A1	-0.56013413	0.01950026
TINF2	0.43343919	0.01045927	HELB	-0.5588559	0.01857712
RBM15B	0.43322737	0.02425501	C16orf58	-0.5587006	0.02287568
SERBP1	0.4330697	1.3242E-17	ABHD12	-0.55795121	3.1634E-06
EIF4EBP1	0.43263603	0.00489636	CYB5A	-0.55791073	0.02044428
STK38L	0.43183859	0.00097095	TDP2	-0.55754505	0.00649272
TM2D3	0.43130552	0.001364	ZNF217	-0.55754334	0.00216511
WDR83OS	0.43127799	3.0184E-08	MRFAP1L1	-0.55659573	0.03500138
SERP1	0.43090172	6.7063E-06	ALPK1	-0.55641636	0.0257124
GNL2	0.43088977	0.01880945	SLC19A1	-0.55637537	0.03183392
BCL2L13	0.4300516	0.00608457	TNFAIP2	-0.55583567	0.02350201
EID1	0.42974237	3.157E-13	KLHL24	-0.5558312	0.0001988
MRPS36	0.42972169	8.3732E-05	RAB11FIP2	-0.55548674	0.01131947
SPECC1	0.42964207	0.04295246	LRMP	-0.55538593	0.00195582
LETM1	0.42945877	0.04777878	C6orf62	-0.55406672	3.3127E-06
PA2G4	0.42944398	7.5535E-11	GSAP	-0.55294153	0.0045438
PHLDB3	0.42840723	0.02838564	ADCY7	-0.55261681	0.01265021
BBIP1	0.42840388	0.04062929	PRR14	-0.55210591	0.01243023
COX20	0.42818824	0.00097459	MSH6	-0.5520789	0.01997484
HMGXB4	0.42759621	0.00823971	LPCAT1	-0.55151097	0.00091271
SARAF	0.42749644	0.00061412	MCPH1	-0.5513671	0.03461811
PMM2	0.42735859	0.02671012	CD151	-0.55097666	0.00620545
BCL2L11	0.4271985	0.01384507	BLVRA	-0.55093131	2.0098E-09
MED8	0.42715425	0.00527618	NR1D2	-0.55062217	0.00024083

RNF145	0.42696609	0.00186858	RP11-473M20	-0.55044635	0.03879267
TRMT10C	0.42641876	0.01186142	ATP8B4	-0.55015711	0.00045339
KCMF1	0.4263043	0.01290119	EVI5	-0.54915943	0.00011636
AP3S1	0.42587345	3.9446E-05	ARHGAP4	-0.54844595	9.419E-07
CDC42	0.42575032	9.4862E-10	EHMT2	-0.54798754	0.02747753
EIF3J	0.42559807	1.7051E-07	TSPAN14	-0.54728463	0.00089046
SIGIRR	0.42550956	0.0007361	TRIM8	-0.54657899	0.02403766
C11orf31	0.42504124	8.3486E-08	CLTA	-0.54515988	7.1925E-06
SSU72	0.42499049	0.00017391	HNRNPU	-0.54433925	1.7933E-09
PMM1	0.42426525	0.02987658	SUPT20H	-0.54422279	0.00484248
ECH1	0.42395641	1.0354E-06	ARHGEF7	-0.54356793	0.0027075
MFSD14B	0.42364796	0.03897691	PANX1	-0.5433367	0.02777333
SIKE1	0.42335722	0.01413998	SLC35B2	-0.54319427	0.00423457
NUP98	0.42318467	0.04224381	SNX5	-0.54288797	6.9639E-10
COX5B	0.42306327	1.3415E-06	TMEM159	-0.54260874	0.03093989
MRPL41	0.42284945	0.00033553	METRN	-0.54211427	0.02582379
PET100	0.42224747	2.3656E-06	PIGBOS1	-0.54199324	0.0152953
SURF2	0.42221401	0.04300154	PLOD3	-0.54190101	0.00298754
EIF2S2	0.42215844	6.7546E-08	MFSD1	-0.54188542	2.2008E-05
TMEM256	0.42173923	6.2108E-06	RGS18	-0.54185832	1.7616E-05
LYRM4	0.42169524	0.01228075	APH1B	-0.54185441	0.00026419
NDUFA10	0.42130946	4.7434E-07	JUND	-0.54176226	0.00122555
HTATSF1	0.42074766	0.00406917	RFX1	-0.54010276	0.00641303
PSMD4	0.42053407	1.8258E-05	SYK	-0.53878198	1.4278E-05
ATP5H	0.42040546	3.1501E-09	POLRMT	-0.53820572	0.0337734
CD72	0.42016389	0.00843948	NDRG3	-0.53724352	0.01028631
PSMD14	0.42016115	7.753E-05	WASL	-0.53718362	0.01288761
UBAP2	0.41983464	0.01425309	PPP2R4	-0.53596531	0.0009914
ARL5A	0.41937775	0.00019854	RASGRP4	-0.53586026	0.01242221
POLE3	0.41888508	0.00236033	SLF2	-0.53519257	0.01101607
COPS6	0.41880383	0.00011086	TMEM223	-0.53508595	0.00485635
NOL7	0.41878206	0.00055992	TFE3	-0.53442334	0.0163646
STK16	0.41867551	0.02838564	SLC30A7	-0.53438788	0.00348303
ATPAF2	0.41859254	0.01125863	RBM47	-0.53412797	2.2535E-06
QARS	0.41854118	0.00153941	CCDC28A	-0.53204952	0.00065718
TRIM69	0.41814502	0.04190291	CBR1	-0.53114737	0.00075075
C16orf13	0.41814415	0.0021388	SREK1IP1	-0.53112323	4.8271E-07
BABAM1	0.41793209	0.00084573	ARHGAP21	-0.53111143	0.00152428
DDX46	0.41791532	4.0898E-06	TNFAIP8L2	-0.53061215	0.00038948
VCP	0.41787478	1.0324E-05	HYI	-0.52993123	0.01493097
SUGP2	0.41774786	0.02115535	ZC3H11A	-0.5285102	1.0326E-08
MRPL20	0.41768186	1.1074E-06	MS4A6A	-0.52823262	6.1671E-10
CCDC167	0.41762812	0.02379951	HHEX	-0.52746084	0.02377778
SH3BGRL	0.41755871	5.6457E-06	IPO8	-0.5274437	0.03760065
DR1	0.41687119	0.0059526	GRINA	-0.52689197	0.00013045
ATF6B	0.41656604	0.00561468	AASDH	-0.52689146	0.02092217
VRK3	0.41635361	0.04873853	MAVS	-0.52684011	0.01204293
ALG2	0.41621141	0.02874433	PDK3	-0.52596988	0.04649014
HSPB11	0.41615094	0.00038959	FOXJ3	-0.5250245	0.00844429

NKTR	0.41614067	0.0002156	DIRC2	-0.52416802	0.01625615
DDX6	0.41613489	3.0363E-06	PLEKHG2	-0.52377478	0.01569046
ST8SIA4	0.41605543	0.00195627	MB21D1	-0.523097	0.00981471
FAM32A	0.41584077	0.00264958	RPRD2	-0.52294084	0.01644813
CAT	0.4156555	2.1961E-05	RCSD1	-0.52198914	2.0978E-07
CALM1	0.41527653	1.0043E-10	SDCCAG8	-0.52193595	1.0266E-05
APEX1	0.41491536	2.7076E-07	SEPT11	-0.52187618	0.00812104
PBDC1	0.41487427	0.00049312	ASB8	-0.52184843	0.00534033
SHISA5	0.4143697	0.00722743	EIF5	-0.52026113	0.00013832
DNTTIP2	0.41422134	0.00353981	VPS72	-0.51783869	0.01125374
MPG	0.41378846	0.03569124	IKBKB	-0.51741931	0.00346734
LAMTOR2	0.41334038	2.1922E-07	FAM21C	-0.51682837	0.00229732
UQCRC2	0.41327025	2.0752E-06	CCNYL1	-0.5155802	0.0033201
PPIB	0.41290929	6.5584E-07	CLASP2	-0.5153702	0.00353981
BOLA3	0.41270478	0.02228177	TLR8	-0.5146293	0.03099197
ERI3	0.41258757	0.0297708	POGZ	-0.5145859	0.00367879
PRKD3	0.41205731	0.02947189	WDTC1	-0.51444976	0.0446338
NDUFB11	0.41191213	1.7909E-09	HECTD4	-0.51378512	0.04637376
TMED3	0.41176179	0.02666215	LMAN1	-0.51343445	0.00033201
SLU7	0.41166375	0.004284	MAP1LC3B	-0.51294663	9.6175E-07
CPSF6	0.41143993	0.00964051	BIN2	-0.51289482	0.0001132
PCID2	0.41116998	0.02958864	NUFIP1	-0.51283673	0.03177423
RAB34	0.4106771	0.00126863	ERO1B	-0.51228787	0.00020672
PFDN4	0.41034617	0.00042866	SOS1	-0.51192393	0.02897788
SPCS3	0.41020913	6.7208E-05	RGS10	-0.51184074	2.2563E-09
MXD1	0.40933151	0.00045054	TOMM70A	-0.51177867	0.01685912
TGIF2	0.40911927	0.02206558	ADNP2	-0.51151481	0.0091332
PHF20	0.40904924	0.00037634	NUP214	-0.51070399	3.6231E-05
ELAVL1	0.40888615	0.00304789	PLCB2	-0.50950272	7.3266E-05
FKBP3	0.40852005	0.00044866	TMEM251	-0.50921327	0.00280792
USP15	0.40845082	3.731E-07	UBALD2	-0.50891355	0.00014852
TUBB4B	0.40835159	1.0146E-05	PDPR	-0.50780306	0.01728113
POLR2L	0.4081292	8.846E-06	REPS1	-0.50772835	0.03960064
COX7C	0.40785966	1.3919E-10	DICER1	-0.50769928	0.00010234
DCTN6	0.40781042	0.00268413	ZMYM2	-0.50682948	0.00196069
TUBA1C	0.40719344	0.00013538	SLFN11	-0.50572829	0.0021276
SEL1L3	0.40690951	0.0367312	IL10RB-AS1	-0.50494498	0.00729111
ATP5G2	0.40563575	7.5835E-15	USP37	-0.50470346	0.01419563
MAPKAP1	0.40555833	0.01435479	PLK3	-0.50410485	0.0019234
MECP2	0.40544217	0.00688163	TMEM88	-0.50310656	0.00769407
SCIMP	0.40493001	0.00683931	PLA2G12A	-0.50271209	0.01839509
REL	0.40457891	0.00159146	HEXA	-0.50228963	5.8557E-09
SF3B2	0.40444755	7.5191E-06	SMIM20	-0.50209205	6.3835E-06
PRRC2C	0.40376959	1.1728E-05	CD302	-0.50193338	1.3989E-11
WDR33	0.40364859	0.00602276	THBS1	-0.50113564	0.00656741
LPGAT1	0.40347975	0.00434809	CD4	-0.50084366	2.6518E-07
SIL1	0.40333587	0.0001029	MTSS1	-0.5005739	0.01164525
RGCC	0.40296826	0.01890491	HNRNPUL2	-0.50029281	0.00163109
CPSF3L	0.40295455	0.00354782	OSTM1	-0.50021448	0.00811279
HLA-DRA	0.40241924	0.00171727	RIC1	-0.49941703	0.01661345

IFITM3	0.40240927	0.00134797	ABHD3	-0.49931019	0.00907375
SRRM1	0.40231058	5.3166E-12	VIMP	-0.49880715	3.297E-05
SLC30A5	0.40115121	0.04517842	ENTPD6	-0.49848619	0.00248052
SLC39A3	0.40100414	0.0422572	OSBPL11	-0.49808839	0.00395676
HAVCR2	0.40070624	0.00612003	MED14	-0.49806958	0.01700019
ARGLU1	0.40051657	1.3286E-06	RRAGC	-0.49754757	0.00039187
MRPS7	0.40023606	0.00026333	AMD1	-0.49729444	1.2234E-07
PAIP2	0.39957908	2.9845E-06	PTPRJ	-0.49697193	0.00745161
UQCRH	0.39903849	1.637E-08	VEGFB	-0.49620883	0.00811119
ACTR10	0.39888172	0.01367288	PHF2	-0.49481165	0.04973803
NDUFB2	0.39824646	1.5097E-06	CEPT1	-0.49422358	0.01692034
TMX2	0.39814933	0.02613499	ATF7IP	-0.49380238	0.01300418
MESDC2	0.39801539	0.00541832	SERTAD1	-0.493441	2.4972E-05
UQCRC1	0.3971206	0.00015879	SLC22A18	-0.49339413	0.00205622
JOSD1	0.39703451	3.3875E-05	ANKRD26	-0.49312914	0.02065962
ATP5O	0.39654407	2.9996E-05	ERC1	-0.49263106	0.01378723
SNX3	0.39651855	0.00010405	IRGQ	-0.49186118	0.01496676
NRBP1	0.39628738	0.00385984	CLDN7	-0.4915961	0.04713616
NDUFA13	0.39622428	2.6395E-05	IFI16	-0.49142258	1.0676E-08
PRDX6	0.39581589	7.9851E-09	SEC22B	-0.49075689	0.00166984
GPCPD1	0.3955015	1.8607E-05	EXOSC4	-0.4906093	0.01744741
SEC23B	0.39492557	0.00576819	ARHGAP27	-0.49005172	0.04553734
CSGALNACT2	0.39463107	0.00037438	GLA	-0.48927458	5.9399E-05
FUBP1	0.3943298	0.00426133	LACTB2	-0.48887226	0.02346751
NDUFV1	0.39399025	5.5174E-05	SLC43A3	-0.48879751	0.00709417
CHD1	0.3939281	0.00326612	FAM210A	-0.48855946	0.00214216
CNPY3	0.39369119	2.1383E-07	FAM134A	-0.48815177	0.01537705
MED10	0.39361803	0.00204498	TSPAN31	-0.4880815	0.00223853
IGBP1	0.39343033	8.7054E-05	RAP2A	-0.48763193	0.02468618
LARP1B	0.39321607	0.02526489	PRDX1	-0.48716439	3.8296E-07
RPA2	0.39319405	0.01145122	CH17-340M24	-0.48694653	0.00447478
COPZ1	0.393083	0.00022451	SPATS2	-0.48620131	0.02130689
CD33	0.39301004	7.7823E-06	PPP3CA	-0.48560153	0.00017736
SLC12A9	0.39236605	0.03452343	ACBD5	-0.48544177	0.00148886
SMIM10L1	0.39145649	0.04394785	GGA2	-0.48483281	0.00021109
CMSS1	0.39105272	0.02033321	SETX	-0.48428223	2.6641E-05
SRSF2	0.39094289	4.4882E-06	RBM38	-0.48373221	0.02147935
DTYMK	0.39065114	0.02977151	C12orf57	-0.48356809	0.00888487
ETV6	0.3903722	0.00836958	SUMF2	-0.48338067	5.4965E-06
TPM4	0.38944495	0.00050087	APLP2	-0.48328578	1.4128E-07
AP2B1	0.3892539	0.00460165	CTSZ	-0.48321548	0.00023658
TPMT	0.38923896	0.00330798	HIBCH	-0.48281403	0.01845851
ARF1	0.38907282	1.578E-05	FAM91A1	-0.48277008	0.01009905
EMC3	0.38893771	7.2704E-05	ATP10D	-0.48260315	0.00775108
TMEM109	0.38878593	0.00528517	CREB1	-0.4817884	0.00164769
YBX1	0.38843889	4.4372E-09	RASSF4	-0.48152404	3.1295E-11
MCCC1	0.38829784	0.04506332	FBXO18	-0.48060105	0.04172762
POLR1D	0.38824751	3.7138E-06	HECA	-0.48059163	9.9135E-05
TROVE2	0.3880498	0.00072828	SCML1	-0.48047764	0.00017706
SRI	0.38741965	0.00014	GAB3	-0.48025387	0.01003764

SDHC	0.3873806	2.5731E-05	PPFIA1	-0.48006808	0.00166931
MPC1	0.38575175	0.01108434	C2CD2L	-0.48004527	0.04182894
OSBPL8	0.38542879	0.0004425	YY1AP1	-0.4796387	0.00693565
LZIC	0.38501511	0.01006253	MGME1	-0.47894627	0.0267246
SCLT1	0.38488752	0.01518183	CSRNP1	-0.4788136	0.00039051
FBXO9	0.38485575	0.00581727	C5orf56	-0.47866619	0.04140088
GPR108	0.38474146	0.01521425	HMGCS1	-0.47840508	0.00833569
MGRN1	0.38459985	0.03410237	SUCLG2	-0.47833567	9.3636E-05
FAM103A1	0.38427552	0.01808385	COA6	-0.47798676	0.00011388
FLII	0.38409222	0.00079065	C1GALT1	-0.47758575	0.00335658
FAM177A1	0.38383088	0.00535237	ARIH1	-0.47695339	0.00019859
MRPL28	0.38352586	0.00301207	ACOX1	-0.4769122	0.00573918
SCAND1	0.38346743	0.00013922	XIAP	-0.47553069	0.000454
CNPY2	0.38325975	0.00032817	IRF2BP2	-0.47458699	0.00038401
LYSMD2	0.38316232	0.02244111	PKNOX1	-0.47448761	0.01959361
ECHS1	0.38316064	9.1119E-05	PLEKHM1	-0.4737167	0.02110074
TWF2	0.38308567	0.00269728	ATP2C1	-0.47354766	0.0003884
FAM136A	0.38295115	0.00393849	REPIN1	-0.47349479	0.00364023
AUP1	0.38275409	8.8576E-06	EEA1	-0.47325131	0.00084532
SMNDC1	0.38273678	0.0058163	CD164	-0.47194258	3.3183E-07
PRPF38A	0.38033089	0.01469414	SRBD1	-0.47117488	0.04730623
TCERG1	0.38004289	0.00572372	MBD3	-0.47097514	0.00796539
ZYX	0.37975847	0.00050711	LTN1	-0.47095503	0.03217368
UFM1	0.37962504	0.00019331	HSP90AB1	-0.47002397	0.00173026
CSTB	0.37957934	0.00019619	NEAT1	-0.46875849	2.5899E-06
ANP32E	0.37923039	0.00507062	PIK3CA	-0.46834724	0.04719289
NDUFB9	0.37901563	4.7995E-06	ARSA	-0.4682855	0.00478215
SUPT16H	0.37873531	0.00099041	RNF125	-0.46787542	0.02065962
IFT27	0.37808341	0.04809825	PTTG1IP	-0.46756732	3.4041E-09
EIF2AK1	0.37725342	0.00419847	REV1	-0.46646371	0.00397723
GRHPR	0.37681266	0.00100518	PURA	-0.46601703	0.01124293
COMMD10	0.37640385	0.00097459	PLXNC1	-0.46568383	0.00326603
ERICH1	0.37616163	0.01281561	YBX3	-0.46517826	0.0007499
URI1	0.37590387	0.00471579	MKLN1	-0.46488709	0.00171683
DCAF11	0.37547354	0.03511228	ERAP2	-0.46419505	0.04001209
CLN3	0.37496134	0.02339124	SLC4A1AP	-0.46302732	0.01293754
DDX27	0.37493402	0.00171947	STX12	-0.46302498	0.0008459
THRAP3	0.37490607	1.9686E-05	KIAA1109	-0.46293559	0.00615334
POLR2G	0.37490172	0.00021598	ESF1	-0.46280575	0.003905
OTUD5	0.37446307	0.02724839	WSB1	-0.46275228	5.857E-07
C19orf43	0.37365178	0.00058647	LYRM5	-0.46256054	0.00563584
ENY2	0.37334844	4.7284E-06	RERE	-0.4622954	0.00057127
PRPF8	0.3727704	0.00543398	JKAMP	-0.4622394	0.00320599
NR1H2	0.37266029	0.0016316	MAPKAPK2	-0.461965	0.0014829
ZC3H15	0.37258232	7.8311E-07	PTPN12	-0.46173442	0.00127783
PGD	0.37254833	0.0298244	P2RX4	-0.46165883	0.00101526
APOA1BP	0.37225641	0.01133069	CCPG1	-0.46164235	0.00015685
COPS4	0.37210163	0.03405669	NOC3L	-0.46096571	0.02831167
KPNA1	0.37197878	0.04754005	SLC39A1	-0.46054491	3.2432E-05
ZMAT2	0.37181202	0.00015908	MAPKAPK5-AS	-0.46041677	0.00649272

SF3B4	0.37179797	0.03024843	DNPH1	-0.45934827	3.1644E-05
EIF2S3	0.37162369	1.8614E-07	CCDC47	-0.45928474	1.1813E-09
TCF25	0.37127994	1.0426E-06	KDM3B	-0.45777556	0.01218584
CCDC115	0.37125456	0.00315863	LENG1	-0.45651542	0.02349275
PSIP1	0.37095183	0.03622734	C4orf27	-0.45636647	0.01136102
CASP4	0.37008913	0.00064189	TM2D2	-0.45629063	0.00557151
ANAPC11	0.36996163	2.5899E-06	SOAT1	-0.45615666	0.00141891
IFI35	0.3694542	0.00814812	CCDC88B	-0.45594063	0.03199207
NDUFB1	0.36910577	0.00029061	VSIG4	-0.45552963	0.00632535
PIN1	0.36887788	0.00447478	PDXK	-0.45481343	0.00080254
ANAPC16	0.36869499	1.3197E-06	AFF1	-0.45466191	0.02621567
STX8	0.36835951	0.00446227	ZNF33A	-0.45326826	0.01518468
TAX1BP3	0.36759165	0.01884434	NECAP2	-0.45249696	2.1385E-05
SPAG7	0.36746359	0.00168809	KDM6A	-0.45071855	0.0171983
KLHDC3	0.36745262	0.03452343	B4GALT7	-0.44997067	0.0065178
VMA21	0.36728245	0.00048011	SGK3	-0.449866	0.00885612
CNIH1	0.3668749	0.00036939	CORO7	-0.44942376	2.5016E-05
GLTSCR2	0.36681965	0.00079469	TFPT	-0.44939597	0.0001702
FAM204A	0.36639709	0.00031262	FRYL	-0.44927545	0.00255405
EIF3D	0.36547596	3.4201E-05	STRN3	-0.44888001	0.04354326
DBF4	0.3653972	0.02726583	LIMA1	-0.44874744	0.01401041
SFT2D2	0.36519922	0.00683341	CEP350	-0.44757398	9.697E-05
SARS	0.36509398	0.01208446	AIG1	-0.4468746	0.00018167
PRDX5	0.3650185	2.8721E-06	ATG7	-0.44683694	0.02287568
THEMIS2	0.36472389	0.00181854	PHACTR4	-0.44535916	0.00151315
PYM1	0.36469845	0.00411913	PRMT9	-0.44507555	0.01465815
IMPAD1	0.36386013	0.03611963	KIF1BP	-0.44416628	0.04978453
VPS35	0.36356546	1.849E-05	EMC2	-0.44135766	0.04463831
BBX	0.3635195	0.01744741	FHIT	-0.43996094	0.04033056
ANXA11	0.36307965	8.3903E-05	CHMP3	-0.43987531	6.8051E-06
APAF1	0.36267962	0.03325646	MAFK	-0.43850763	0.04534756
ZSCAN16-AS1	0.36254342	0.02412459	PPP2CB	-0.43829807	0.00386937
TCEB2	0.36214682	2.5106E-05	TECR	-0.43794731	0.0007364
ABHD14B	0.36201671	0.00821708	NUFIP2	-0.43763176	3.2184E-05
C9orf78	0.36164342	6.8346E-05	DRAM2	-0.43716982	1.6204E-06
ATXN3	0.36109369	0.03850009	LINC00998	-0.43685088	9.5366E-05
HCST	0.36106338	0.03428566	TDRD3	-0.43682439	0.0352843
URM1	0.36090439	0.02541713	SRFBP1	-0.43556351	0.01924945
YY1	0.3606781	6.0218E-05	PLCG2	-0.43510404	0.01753586
SSRP1	0.36050552	0.01133069	NME4	-0.43339842	0.04759915
SLC35E1	0.35968559	0.04730486	PJA2	-0.43334018	9.2472E-06
RHOT2	0.35956235	0.03545604	REXO2	-0.43221407	0.00483383
CHP1	0.35930913	0.00297066	MR1	-0.43152871	0.01083253
TOR1A	0.35817351	0.04139922	ST3GAL6	-0.43068608	0.00114586
MAN1A2	0.35816972	0.01356327	RUBCN	-0.42999553	0.0175141
ERGIC2	0.35771916	0.01970539	TRAK1	-0.42874835	0.00529355
ORMDL1	0.35689068	0.02012197	CCDC97	-0.42866206	0.04485005
RPA3	0.35676667	0.00482966	PICALM	-0.42863295	2.0971E-06
PPP1R2	0.35549033	5.2325E-05	GIPC1	-0.42851773	0.04439989
TMEM126A	0.35526757	0.00576819	MARCH7	-0.42799929	0.00107494

SSB	0.35505136	0.00027316	POLK	-0.42748677	0.01730981
UCP2	0.35484731	6.1522E-05	FUBP3	-0.42681754	0.02277044
PACSIN2	0.35410827	0.00221252	UBB	-0.42651889	0.00095249
CLDND1	0.35400766	0.02782637	TMBIM1	-0.42638756	0.02270783
DLD	0.35394829	0.00822624	SNX29	-0.42605637	0.00195388
DNAJC3	0.35345342	0.0012527	RXRA	-0.42491818	0.02458104
FES	0.3530002	0.00447039	DCAF8	-0.42453366	0.01532372
MAPK1IP1L	0.35298221	0.00018843	ACAP2	-0.42405565	0.00094696
FAM107B	0.35280661	0.03455564	CD63	-0.42383621	0.00018009
NSD1	0.35231025	0.00833138	MYL6B	-0.42379528	0.02057693
TSEN34	0.35224262	0.02770251	DPEP2	-0.42369159	0.00353981
YWHAB	0.35209698	1.9409E-07	DYNC1LI2	-0.42321594	0.0037787
TIGAR	0.35188391	0.02160843	MARCH1	-0.42320234	0.00522485
DNAJC7	0.35183389	0.00183546	ARL14EP	-0.42249631	0.01730887
IL6R	0.35170253	0.01920415	TMEM9	-0.42201761	0.03058912
C1orf52	0.35151823	0.01931315	TANK	-0.42136943	0.00712211
PRKAR1A	0.35106745	1.7499E-05	AZIN1	-0.42063698	1.2852E-06
BUD31	0.35073952	0.00125315	DTX3L	-0.42039821	0.0131442
TXN2	0.35055853	0.0002171	PPP6R3	-0.42026506	0.02081027
CAPN1	0.35000258	0.04189682	CHIC2	-0.42022591	0.04747319
EEF1D	0.34986766	2.2689E-10	C17orf62	-0.41954002	0.00027118
OSGEP	0.34958783	0.00744392	MAX	-0.41634187	0.000124
SMCHD1	0.34855187	0.0022103	MYO9B	-0.41625505	0.00176972
COPS5	0.34850969	0.00516586	KDM6B	-0.41622113	0.00065572
FAM207A	0.34847892	0.03960064	MBTPS1	-0.41601253	0.01406428
CBX3	0.34759924	0.00011465	FKBP15	-0.41463	0.01706564
RAP1A	0.34757842	1.2397E-08	BRWD1	-0.41341418	0.02579589
PIH1D1	0.34743904	0.04773872	PDPK1	-0.41267115	0.00249809
DENR	0.34683037	0.00833443	RTN3	-0.41259859	0.00012847
SSBP4	0.34639712	0.01671461	GAS7	-0.41250501	0.00206712
C4orf3	0.34611747	1.8172E-05	MAP3K3	-0.41214564	0.01634706
MRPL54	0.34594557	6.9673E-05	DPP7	-0.41185082	6.3977E-05
BCL7B	0.3458027	0.0163009	SLC8B1	-0.41142014	0.01992614
SUMO3	0.34570058	0.00028377	ERN1	-0.41090192	0.00336513
KHDRBS1	0.34554817	2.5899E-06	MOB1B	-0.41076368	0.03292242
CAPZA1	0.34548642	6.8816E-06	BNIP3L	-0.41046021	0.0001025
COQ10B	0.34516511	0.01918273	PAPSS2	-0.40971423	0.0091332
RNF10	0.34509703	0.01703261	WASF2	-0.40954261	0.0006519
PNKP	0.34476694	0.03476326	VPS37C	-0.40851027	0.0493395
IK	0.34471016	0.00011506	RNASET2	-0.40850656	0.00117796
DERL2	0.34456791	0.01614319	FOXN2	-0.40818617	0.01669638
C1orf56	0.34371267	0.00961813	NCKAP1L	-0.40722404	0.00019802
ZNF787	0.34366943	0.04647787	BTN3A2	-0.40633079	0.03118085
NEDD8	0.34362637	1.2255E-06	FAM53B	-0.40603454	0.03325646
SRP19	0.34351572	0.02941963	DENND4B	-0.40507847	0.033271
MAF1	0.34341574	0.00698294	CCDC91	-0.40438341	0.01317035
COPS2	0.34276368	0.00124592	AK3	-0.40401414	0.0088987
PAPSS1	0.34262295	0.0491452	NOL8	-0.40364045	0.00641115
HMGB1	0.34260354	1.1781E-12	AKIP1	-0.40291622	0.01516637
ILF3	0.34253442	0.0001901	GAK	-0.40289933	0.00526466

TRMT1	0.34247321	0.01989311	MBNL2	-0.40278116	0.04927131
MSL3	0.34215606	0.01202146	CASC4	-0.40252768	0.01480913
RAB5C	0.34176436	1.1538E-05	YME1L1	-0.40212281	0.00189021
RSU1	0.34145071	0.0032729	SMS	-0.40130627	0.00083131
HNRNPK	0.34122922	2.6651E-08	YAF2	-0.40097702	0.0003371
USP48	0.34103174	0.0349844	KIF13B	-0.40044236	0.01248734
UBL5	0.3406815	0.00045054	CHKA	-0.39957672	0.01424643
CTBP1	0.34063505	0.00736381	RPF2	-0.3992958	0.00509112
PDIA3	0.34055966	0.00026941	ROCK2	-0.39882782	0.04552803
ERGIC1	0.3404803	0.04220675	PANK2	-0.39790926	0.00682216
VASP	0.34035252	0.0082961	TTC1	-0.39720654	0.01348907
TATDN1	0.33997266	0.01508951	FNIP2	-0.39687769	0.00126333
AIMP1	0.33988003	4.1787E-05	HSDL2	-0.39681494	0.00811092
CMTM6	0.33975148	0.00089046	RBPJ	-0.39500861	0.00415861
RTFDC1	0.33972628	0.00018663	LASP1	-0.39311322	0.00337313
H2AFZ	0.33966541	0.00341763	GAPVD1	-0.39260488	0.01957842
BRMS1	0.33935401	0.01790882	VPS37A	-0.39208113	0.04810906
IAH1	0.33915596	0.00132252	CPSF2	-0.391537	0.02672502
COA4	0.33856944	0.00214873	TAF1	-0.39057664	0.04948187
DAP	0.33836422	0.04830646	ZC3H7A	-0.39054997	0.00624288
RQCD1	0.33831952	0.02690042	TAOK1	-0.39049675	0.00125194
USMG5	0.33827692	0.00035824	AGGF1	-0.38946366	0.02448864
PFKL	0.33826334	0.00423599	RABEP1	-0.3892236	0.00688163
TIMMDC1	0.33815302	0.00295853	JMY	-0.38867705	0.0171983
DDX39A	0.3376952	0.02437448	TPD52L2	-0.38865481	0.0089612
PRMT2	0.33653439	0.00048665	MACF1	-0.38800418	0.00020415
TAF9	0.33593991	0.00626133	IST1	-0.38724061	0.0010248
TM9SF2	0.33592689	0.01728796	DPYSL2	-0.38718109	2.2157E-07
NIFK	0.33531496	0.01693778	IL18	-0.38618693	0.0013521
ZNF655	0.33530103	0.00491488	SDCBP	-0.38603095	0.000211
BCCIP	0.33522614	0.03917757	CLN8	-0.3852562	0.00279905
WDR45	0.33503093	0.03362184	MON2	-0.38475822	0.04257572
EIF2B1	0.33479615	0.04727679	DRAM1	-0.38454046	0.02219428
FBXW5	0.33457149	0.02745354	MOCS2	-0.38453688	0.02221868
POLR2F	0.33446684	0.00616714	JDP2	-0.38450141	0.02144369
AATF	0.33342142	0.04563811	MTR	-0.38403977	0.01839279
ELP5	0.33318897	0.02288657	SIN3A	-0.38374777	0.02531644
SNRPC	0.33255401	0.00098974	CTTNBP2NL	-0.38346571	0.01913913
AAMP	0.33225806	0.02030255	TACC1	-0.38330739	3.7303E-07
MIF4GD	0.33188596	0.00594612	YIPF6	-0.38295008	0.01061415
DDA1	0.33135288	0.04294558	WNK1	-0.3825576	0.00010557
RNF5	0.33127131	0.01280802	NCOA1	-0.38251846	0.02384645
ADK	0.33061711	0.01345475	XPO6	-0.38156753	0.04129997
RBM4	0.33048952	0.02975943	EFCAB14	-0.3815291	0.00203476
SCO2	0.3289864	0.01920908	SERINC5	-0.37997495	0.02957998
DERL1	0.32897794	0.00975982	GTF2I	-0.37966128	0.00325944
MRPL14	0.32835172	0.00517377	TMEM30A	-0.37910777	0.01044391
RBM25	0.32792232	0.00035826	LAMTOR3	-0.37860693	0.00988756
NUDT21	0.32786938	0.00919159	FAM35A	-0.37855215	0.02491053
TMEM87A	0.32770905	0.0110255	UBAP2L	-0.37842638	0.00913698

CCNH	0.32699662	0.01567222	SLC25A24	-0.37645308	0.01860144
JPX	0.32657792	0.02363171	CARD8	-0.37630483	0.010021
XRCC5	0.32570409	4.5287E-06	MGAT1	-0.37511735	4.0554E-06
MCTS1	0.32519373	0.00403406	PHIP	-0.37505692	0.0072942
DNAJC21	0.32499039	0.00133164	RBM22	-0.37422854	0.00044398
IGSF6	0.32467949	0.01133069	HSPBAP1	-0.37182778	0.02033321
GGCT	0.324566	0.00513021	JMJD6	-0.371749	0.03941883
UTP11L	0.3245247	0.0426042	NENF	-0.37060494	0.00088982
DENND6B	0.32422379	0.01766835	PRRC2B	-0.36952057	0.01792936
TARS	0.32408623	0.00794138	SMAD2	-0.36946276	0.02674652
SDHA	0.32390331	0.02111397	WBP2	-0.36818957	0.00953431
PUF60	0.3236245	0.00026172	RRP12	-0.36784741	0.01459313
MORF4L1	0.32361587	7.5099E-09	PREX1	-0.36761885	0.01025019
PYCARD	0.32336565	0.00162177	CAB39	-0.36759874	0.01883409
IDH2	0.32335708	0.02859257	KLC1	-0.36659585	0.04233362
RNASEH2C	0.32317931	0.00203484	GNPTAB	-0.36615391	0.0320337
PTPN6	0.32317059	0.00270809	TWF1	-0.36510974	0.02541646
LINC01420	0.32297153	0.00780377	RNF216	-0.36504509	0.02514649
FYTTD1	0.3225503	0.00191334	KIDINS220	-0.36431173	0.00284874
KLF3	0.32221326	0.01609228	DSE	-0.36313716	0.00010004
HLA-C	0.32221058	0.00175816	UTP18	-0.36272383	0.02042781
FAM45A	0.32192555	0.04109529	LEPROT	-0.36252373	0.001887
MYEOV2	0.3214589	0.00087538	SELPLG	-0.36158823	0.04615919
ANKRD44	0.32107949	0.04877119	FCHSD2	-0.35898365	0.00751374
TMED2	0.32057128	0.00026768	XAF1	-0.35774792	0.02301875
OAZ1	0.32035598	2.755E-06	SIGLEC10	-0.35632066	0.00196842
EIF3A	0.32030826	0.00010305	CHID1	-0.35624296	0.03526171
NDUFS7	0.32006396	0.00224462	BAD	-0.35514185	0.03475605
TBCA	0.31936566	0.00011282	PPM1K	-0.35253849	0.02816838
FRG1	0.31929828	0.00174368	SNX9	-0.35195486	0.03736977
NDUFS2	0.31894925	0.00106665	PHACTR2	-0.35187137	0.00756762
CNOT7	0.31869088	0.01788823	VEZF1	-0.35176491	0.04130278
PDHA1	0.31868825	0.00930663	ZNF22	-0.35171325	0.01467109
BRD7	0.31787462	0.0164655	BAZ1B	-0.35094124	0.03872666
ARHGEF1	0.31766805	0.02328652	ZNF518A	-0.35074009	0.02065962
SF3B5	0.31755147	3.3981E-05	ABI1	-0.34963614	0.00043221
LACTB	0.31735925	0.00527016	TNPO1	-0.34918543	0.00617982
CLEC2B	0.31675699	0.02235982	IMP3	-0.3473239	0.04578267
COX6C	0.31669493	5.2031E-05	ZBTB1	-0.34603329	0.02764332
NDUFS5	0.3163771	7.1669E-06	PHTF2	-0.34554222	0.03261126
NPM1	0.31633932	1.4442E-09	ACIN1	-0.34553782	0.02859385
TERF2IP	0.31606774	0.0049862	MT-ND4L	-0.34406976	0.00080277
AAED1	0.31549988	0.00961652	POLR2A	-0.34360116	0.01686205
TNFRSF1B	0.31478517	0.00015238	YTHDF3	-0.34336391	0.00749973
CCNY	0.31474821	0.00593199	SCAMP2	-0.34290941	8.0883E-05
MKRN1	0.31426467	0.03360343	ZNF644	-0.34270985	0.00574481
KMT2E	0.31402783	0.017482	LARP4B	-0.34232286	0.03149896
UQCC2	0.31389606	0.03502226	NR3C1	-0.34163772	0.00122188
NSA2	0.31334947	1.6771E-05	XBP1	-0.33838373	0.00029837
AP2M1	0.31245482	0.00027633	STX4	-0.33647258	0.00187451

TMEM141	0.31231537	0.02672502	C16orf87	-0.33584809	0.04980551
UQCR11	0.31211675	1.4137E-05	HDLBP	-0.33577982	0.00011677
LBR	0.3120369	0.02668069	CAPZB	-0.33575154	1.7789E-05
ATP1B1	0.31183948	0.00034222	STAT2	-0.33556478	0.0123644
TMEM14B	0.31085857	0.00086718	ZFX	-0.3352634	0.00771117
MRPL55	0.31044847	0.03630803	MT-ND2	-0.33517287	0.00036121
COMMD2	0.31027176	0.04578755	ZFYVE16	-0.33381655	0.00031488
IFI27L2	0.31003156	0.00336513	DDX1	-0.33228567	0.03162079
PHF10	0.30912867	0.03444608	FBXO11	-0.33182631	0.00943313
MPV17	0.30820259	0.03526642	CISD2	-0.33159405	0.00096249
DNAJC8	0.30748661	0.00016182	LRRC8C	-0.33139394	0.03011983
ELF2	0.30736669	0.00167429	NTPCR	-0.33032716	0.04517842
SH3KBP1	0.30724643	0.00467134	UNC50	-0.32940658	0.02670935
IMP4	0.30723682	0.00560748	TNFRSF14	-0.32786618	0.00107454
NDUFA1	0.30721201	6.9673E-05	NAMPT	-0.32768204	8.0046E-06
DPM3	0.30677717	0.01377386	EVL	-0.32593681	0.00072828
CCNI	0.30647428	0.00032524	MGST2	-0.32543746	0.00325046
SRP14	0.30591764	1.7332E-05	TCEAL4	-0.32408937	5.9191E-05
ARL2BP	0.30506226	0.03325646	U2AF1	-0.32123098	0.04757471
MRPS11	0.30470689	0.04581067	B4GALT1	-0.32073091	0.00561964
JMJD1C	0.30446831	0.01226739	DSTN	-0.31781098	0.00161504
HCLS1	0.30434406	0.0001417	GNB4	-0.31708355	0.02163147
TCEB1	0.30422366	0.00068551	ITCH	-0.31659595	0.04757471
ARL6IP4	0.30374602	5.8513E-05	MAT2B	-0.31622119	0.00836184
ITPA	0.30335	0.01290177	ARL4C	-0.31581562	0.03444501
PCBP1	0.30306723	1.7101E-07	BRD2	-0.31468065	0.01575632
GPX4	0.30277395	0.00167307	TOR1AIP1	-0.3145238	0.02382343
PLIN3	0.30275981	0.01466921	SNRPN	-0.31402131	0.02270147
NDUFC1	0.30269338	0.02668069	IFNAR1	-0.31303961	0.00055934
DHX9	0.302436	0.02161369	PTMS	-0.31260766	0.00767707
LSM8	0.30118496	0.00095599	UBAC2	-0.31218781	0.00595913
SPTSSA	0.30099612	0.01299652	CTBS	-0.31106801	0.02180406
ZNHIT1	0.30076802	0.00601375	LNPEP	-0.31074719	0.03469699
POP4	0.30065019	0.0450425	TUG1	-0.30930214	0.03961675
PSMG2	0.30048289	0.00567604	HSD17B12	-0.30904376	0.04830646
FAM96B	0.29886624	0.01304456	RAB31	-0.30546374	4.24E-05
FAM110A	0.29862743	0.03620165	TBXAS1	-0.30471683	0.00052097
JOSD2	0.29851762	0.02228177	PCBD1	-0.30238366	0.01210209
COPA	0.29814887	0.01542528	MCL1	-0.3023672	0.00209028
BECN1	0.29774505	0.02411597	PAIP1	-0.30204607	0.04341406
NAPA	0.29697844	0.00500643	FMR1	-0.3017969	0.04518062
TMEM14C	0.29680264	0.00056669	ARL6IP1	-0.3014164	0.00040068
UQCRQ	0.29677505	0.00060958	RTCA	-0.30065414	0.03877762
ARPC4	0.29661653	0.00216316	EPN1	-0.29939062	0.02957998
NDUFA11	0.29657451	5.3887E-05	RAC1	-0.29731014	6.3936E-05
WDR61	0.29622217	0.02603776	DDX5	-0.29649021	3.4431E-05
HMGN3	0.29575679	0.00147551	RNF13	-0.2960199	0.00910014
PFDN2	0.29516666	0.00061907	DNAJC1	-0.29493813	0.03951315
EWSR1	0.29514601	0.00328623	RPL22L1	-0.29307738	0.01264359
REEP5	0.29486286	0.00022131	RSRC2	-0.28927119	0.01674054

LINC00493	0.29477539	0.00201477	MTIF3	-0.28645268	0.02870024
IER3IP1	0.29475891	0.01479442	SUZ12	-0.28630076	0.0321877
ALKBH7	0.29430337	0.00366298	TMBIM6	-0.28616906	9.9308E-06
CCDC59	0.29415525	0.00114101	CISD3	-0.28401856	0.03725371
CSNK1A1	0.29404336	0.00025565	PFKFB3	-0.28400209	0.04381685
PPT1	0.29368837	0.00545958	UGP2	-0.28216306	0.01839279
PSMD1	0.29320015	0.03254098	TPP2	-0.28173408	0.04656987
GNB1	0.29275265	0.00028031	RAB14	-0.28136887	0.00581433
RPL23	0.2923265	4.598E-08	TCEAL8	-0.27701437	0.0192845
TMEM179B	0.29136099	0.00269018	NAGA	-0.27654476	0.0236248
SAP18	0.29104798	0.00020834	MDM4	-0.27612062	0.01832856
NAPRT	0.29081017	0.02684527	DYNC1I2	-0.27502019	0.00494929
PCBP2	0.28991023	1.2859E-06	PTGER4	-0.27497097	0.01758203
HNRNPA3	0.28970398	0.00039422	LARP7	-0.27115642	0.01397841
RNPEP	0.2892133	0.04062929	G3BP1	-0.27113062	0.00243014
NDUFA4	0.28907	0.01324398	DDX3X	-0.26790027	0.00211581
CAP1	0.28842611	1.2201E-06	MT-ND1	-0.26721996	0.01077717
PRDX3	0.28834886	0.02787859	ZNF706	-0.26694244	0.00244818
C9orf16	0.28777495	0.01635914	CAMK1	-0.26629983	0.04036412
ACTR2	0.28739762	7.0675E-06	FYB	-0.26604153	0.02121191
NDUFAF3	0.28646318	0.00392434	SAT1	-0.26398316	0.00021983
IKZF1	0.2857122	0.02693343	BCAP31	-0.2607839	0.01358894
ZCRB1	0.28442351	0.0121864	MPHOSPH8	-0.2605769	0.01790882
AOAH	0.28440645	0.01390051	GINM1	-0.25864087	0.02158386
ERP44	0.28405647	0.00104891	TEX264	-0.25817495	0.02414969
NFATC2IP	0.28355483	0.04029505	SH2B3	-0.25811574	0.00232568
LINC-PINT	0.28324491	0.04775311	DDX17	-0.2570768	0.01907329
STUB1	0.2825187	0.0113039	NCOA4	-0.25622296	0.01576031
PSMD11	0.28242567	0.03809166	PTBP1	-0.25607632	0.04347062
MTDH	0.28171565	8.2235E-06	KMT2C	-0.25595542	0.02005744
NCOR1	0.28102597	0.02377191	TYROBP	-0.25528583	5.6671E-06
ANXA7	0.27910818	0.01335551	ASAH1	-0.25441726	0.02452761
SRSF6	0.27907659	0.01474572	OAZ2	-0.25228624	0.01796517
ARIH2	0.27885137	0.03784793	VOPP1	-0.25211204	0.00845839
UBE2R2	0.2773613	0.001025	CHMP1B	-0.24913465	0.02842042
PRPF40A	0.27708045	0.00013027	WIPF1	-0.24840673	0.013328
MMP24-AS1	0.27695153	0.01843244	AKR1B1	-0.24734285	0.02684527
UBE2G2	0.27667782	0.0276388	SELK	-0.24641342	0.01769852
ACAA2	0.27647809	0.04503043	ADAM28	-0.24283333	0.02959767
BAG1	0.27615473	0.00573505	SEC62	-0.24230316	0.00037634
SUMO2	0.27560142	0.00055024	CD46	-0.2408972	0.04777878
SND1	0.27536002	0.01003099	MT-ND3	-0.23920632	0.02221037
VPS28	0.27515675	9.3757E-05	GCC2	-0.23910339	0.02064993
NCBP2	0.27477857	0.04039384	GTF2B	-0.23907577	0.043307
BRD4	0.27447131	0.00256678	SLC8A1	-0.23767732	0.00366233
SAR1A	0.27397008	0.0389461	ARCN1	-0.23440745	0.04653251
ERGIC3	0.27349696	0.03059185	ADIPOR1	-0.23261866	0.01745825
NUDCD2	0.27349145	0.04647103	BRI3	-0.22629701	0.01087086
SYNCRIP	0.27313992	0.00221716	U2SURP	-0.22303809	0.01721818
COMMD1	0.27293824	0.01437699	SCAF11	-0.2123773	0.02579347

CST3	0.27258205	0.02878552	NUCB1	-0.21204412	0.0380611
ERP29	0.27160806	0.00018687	SOD1	-0.20375681	0.00922161
DNPEP	0.271187	0.04450714	CANX	-0.20259704	0.00620451
EPRS	0.27104806	0.04929842	SERPINB6	-0.19379563	0.03562653
ITGA4	0.27052178	0.02012116	MT-CO3	-0.19372427	0.01780854
GGNBP2	0.2704602	0.04442513	LAPTM5	-0.19076875	0.03176752
ATP6V0D1	0.26923715	0.01758994	PRPF38B	-0.17893151	0.02713437
SSR1	0.26776819	0.02329636	ARRB2	-0.16405238	0.02396265
RBM39	0.26757222	1.8121E-05	IQGAP1	-0.15966106	0.02095961
MOB1A	0.26627378	1.944E-05	SRGN	-0.15042418	0.02728758
RBMX	0.26556007	0.00874322			
TFG	0.26509512	0.03679851			
TIMM13	0.26496769	0.00691982			
RPS20	0.26426327	0.00013891			
PAPOLA	0.26399894	3.3039E-05			
SCP2	0.26343125	0.0025999			
PPIF	0.26278822	0.01745823			
GNAS	0.26250864	0.00264461			
CD300A	0.26239604	0.0359317			
KRR1	0.26185822	0.03645413			
HNRNPDL	0.26119265	0.0006821			
CLEC7A	0.26099195	0.03523237			
H2AFV	0.26086229	0.03730253			
ANXA5	0.26065537	0.04238729			
NUMB	0.26006683	0.02749485			
SSR4	0.2590904	0.00539058			
TGIF1	0.25897948	0.02471342			
PSMF1	0.25895565	0.0121864			
RNASEH2B	0.25797397	0.04796061			
ARPP19	0.25677798	0.01419459			
SRSF4	0.25617063	0.0104163			
SF1	0.255711	0.00586947			
CCDC124	0.25442761	0.0327166			
NDUFA5	0.25306065	0.035183			
TMEM170A	0.25224533	0.02269524			
CWC15	0.25040263	0.0365744			
TMEM243	0.24983764	0.04640646			
HMGN2	0.24861086	0.00501728			
CUEDC2	0.24820694	0.02112634			
RBM23	0.2479341	0.03741142			
PHF20L1	0.24793185	0.03520286			
ELOVL1	0.24700105	0.0491452			
MRPS21	0.24668689	0.00501428			
RAB7A	0.24647494	0.01151556			
MAP3K2	0.24578525	0.00305993			
TMEM147	0.24528374	0.01255604			
TMED10	0.24522805	0.00351525			
DUSP23	0.24518752	0.04573837			
ELMO1	0.24513125	0.01152421			
TRAPPC3	0.24432049	0.04263924			

EIF4H	0.24412935	0.00225992
PSMC1	0.24297798	0.02668069
EVI2A	0.24214646	0.02222632
GPX1	0.24134534	0.0033602
SKP1	0.2389635	0.00143078
SPI1	0.23866325	0.01317035
SRSF10	0.23753978	0.04809825
ROCK1	0.23734367	0.02683904
RAB8B	0.23586252	0.03956293
NCL	0.23579331	8.646E-05
TAPBP	0.23410233	0.03136592
FKBP8	0.23315008	0.00256482
RPS28	0.23240242	1.9966E-06
RAD23A	0.23229178	0.00408857
C1orf43	0.23024946	0.03847269
BCLAF1	0.23008815	0.02305723
TCEA1	0.22987149	0.0133132
HLA-DPA1	0.22825799	0.03055898
AES	0.2267493	0.02564901
YWHAE	0.22671912	0.00467524
MINOS1	0.22598629	0.0487709
ETFB	0.22578699	0.02582918
TMSB4X	0.22457168	0.01596774
RAB1A	0.22350437	0.03475605
TOMM20	0.22324497	0.00102985
PABPC1	0.22184973	0.02646851
DOK2	0.22096599	0.04604832
DBNL	0.21929811	0.01997484
SEC11A	0.21866111	0.0271107
UBXN1	0.21838229	0.02874433
MTCH1	0.21343268	0.03785604
PEBP1	0.21333858	0.00795571
UQCR10	0.21214515	0.03427693
CMPK1	0.21192881	0.02450843
UBE2B	0.21060779	0.00644327
HNRNPH3	0.20899169	0.0398832
GNAI2	0.20663228	0.00050639
PTMA	0.20577097	4.3326E-05
RHOG	0.20441082	0.0186528
BOD1L1	0.20215545	0.04324174
GRB2	0.20143098	0.00250473
ATP5I	0.19926536	0.00652126
SEP15	0.19604215	0.0192851
MT-CO2	0.19276141	0.02360238
RHOA	0.19207127	0.00037316
RNMT	0.18959887	0.01606912
CD74	0.18319533	0.00729363
UQCRB	0.18243324	0.00031576
NDUFA3	0.18122042	0.03579528
ARHGDIA	0.18120942	0.02030255

C11orf58	0.17811799	0.02021869
TRMT112	0.16875984	0.0204417
LY86	0.16697174	0.01172398
SON	0.16105282	0.03751173
POMP	0.15862766	0.033271
TOMM7	0.15196055	0.03832766
TOP1	0.14391216	0.03725371
EIF1	0.13483603	0.00866451

		cDC3 vs M8			
cDC3 DEGs	avg_logFC	adj. p-value	M8 DEGs	avg_logFC	adj. p-value
FCN1	4.47771548	4.9383E-12	RAB42	-5.25573143	1.1633E-31
TMEM8B	4.23793736	1.3929E-14	MT1H	-4.38382637	3.0577E-07
UPK3A	4.17858156	1.1187E-19	GAL3ST4	-4.37605673	3.0141E-27
TSPAN32	4.07851101	6.9868E-11	GPNMB	-4.32706122	2.4694E-31
CCL17	3.62365638	2.6161E-05	TMEM37	-4.29547291	3.1182E-39
AFF3	3.52691798	3.8258E-27	MYO7A	-4.25745329	2.9645E-10
MPP7	3.44182064	5.6807E-09	MT1G	-4.24935202	2.8711E-24
C16orf74	3.42095233	4.3699E-05	BCAM	-4.05170582	6.9863E-25
LAYN	3.33364489	7.8527E-07	HSD17B14	-4.00927431	1.0079E-32
APOBEC3A	3.30682538	5.0119E-13	GDF15	-3.99826226	5.2532E-10
CD2	3.30122772	2.9645E-10	PPIC	-3.97218157	3.0789E-20
FAM65B	3.24916346	1.2312E-12	FUCA1	-3.94530909	2.3716E-25
CRIP3	3.10995946	0.00076772	LAG3	-3.93626718	6.7995E-28
P2RX1	3.09840829	1.0385E-07	CD163L1	-3.92295487	1.9751E-35
SYTL1	3.05355757	3.5583E-08	APOE	-3.86742921	3.3223E-10
ABHD17C	3.04576198	2.7868E-07	CCL24	-3.81669807	5.4575E-06
SMAD3	3.00849181	2.9611E-12	OLFML3	-3.79557318	1.916E-15
MYB	2.94659178	4.3367E-06	TDRD10	-3.755817	9.292E-08
CXCL11	2.91355553	2.0608E-09	ADAMDEC1	-3.74992155	1.7683E-45
CD1C	2.90155419	2.2033E-20	SLC7A8	-3.71380384	2.9697E-42
ANGPT1	2.84201356	0.00173965	ATP6V0D2	-3.70382322	5.742E-26
TRH	2.83113178	0.00151003	APOC1	-3.67195561	1.6496E-22
GPRC5A	2.75786615	5.7927E-07	TMEM26	-3.62997254	2.7466E-06
ANKRD22	2.7397326	0.00010921	YES1	-3.61934482	3.5756E-11
CTD-2006K23.	2.71912513	1.7853E-08	IGSF21	-3.61627114	1.5136E-07
SELL	2.71382379	1.6787E-26	ABCG2	-3.59812391	1.3019E-26
LINC00694	2.70531998	0.00054273	LILRB5	-3.58971313	3.8829E-17
FYN	2.69321353	4.129E-16	FAM174B	-3.55875359	3.756E-09
S100A12	2.6818668	1.7522E-06	RAB3IL1	-3.55786655	1.0634E-17
DRGX	2.66500923	0.01418671	GSDMA	-3.54276132	4.3906E-07
HACD1	2.66456105	1.4431E-05	CYP26B1	-3.52957981	0.0009393
ENHO	2.60763494	8.3007E-09	MMP12	-3.51911914	3.6024E-17
IL1R2	2.60125934	9.1852E-15	CRYBB1	-3.51659534	3.2777E-05
HSH2D	2.59395892	4.9925E-10	NUPR1	-3.50793381	1.4734E-31
SH2D4A	2.58299549	0.00398718	PGBD5	-3.5002116	8.4526E-07
NRGN	2.57582073	1.2823E-06	SEPP1	-3.49720028	1.0618E-22
SPNS3	2.55572543	5.8925E-20	ME1	-3.49563512	2.9583E-34
AUTS2	2.54550448	0.00040274	NRP1	-3.49233473	1.2053E-63
VCAN	2.53384048	2.0063E-12	PLEKHG5	-3.48361917	0.0013295
CRLF2	2.51995242	0.00011234	TRIM47	-3.45167327	4.6363E-10
SDPR	2.51087892	1.2926E-21	ADAMTS12	-3.42720353	3.8993E-05
C11orf21	2.50178233	0.00292349	SHE	-3.39933937	4.3397E-13
FAM134B	2.50055695	0.00002657	TNFSF18	-3.37899845	0.00497533

FCER1A	2.46993248	1.7031E-09	C2	-3.37796794	6.6431E-29
ELFN1-AS1	2.46394168	0.02484023	ABLIM3	-3.36805282	0.00017758
MCEMP1	2.46098237	1.6652E-14	CXCL12	-3.35690727	3.5102E-21
SNTB1	2.45827197	0.0006587	RNASE1	-3.34989691	4.9005E-11
SLC35G1	2.44427968	0.00956369	RP11-733O18.	-3.33868338	0.02667313
KBTBD11	2.44400831	2.1548E-07	MTUS1	-3.31631732	4.0508E-20
MTMR11	2.43526539	1.6327E-10	POLN	-3.31454012	0.00017928
PLPPR2	2.42099946	0.00076819	TNIK	-3.31204573	6.0303E-12
PTGIR	2.38946959	5.4189E-12	OTOA	-3.30548463	1.842E-24
S1PR4	2.38387438	2.7844E-10	PGA5	-3.30457064	0.02861907
MYBL2	2.36594143	0.01222726	CABP4	-3.29984862	1.7811E-05
UTF1	2.34272906	4.3576E-11	PIK3CD-AS2	-3.28589474	0.00418316
ACY3	2.33428874	0.0027103	ZG16	-3.26245585	0.02642147
CFP	2.3336002	3.6797E-24	SIGLEC8	-3.25800966	3.5423E-08
LIPN	2.32198195	0.00144176	BNC2	-3.24519102	5.0308E-14
AKR1C3	2.3213654	0.00148119	NEK10	-3.23334677	0.01879193
KLHL25	2.30990896	0.00058894	PIR	-3.22927985	1.4836E-09
MN1	2.3086583	0.00350017	TFAP2C	-3.22756226	0.01405027
VDR	2.30828404	2.3132E-25	EPHB2	-3.19513231	1.2545E-15
CD52	2.30458915	1.4316E-10	CTSD	-3.19503895	1.7129E-38
FCAR	2.30096372	8.4885E-05	NPL	-3.18855596	2.001E-34
IL18R1	2.30034709	8.2243E-08	MERTK	-3.1583442	1.0352E-29
IGF2BP2	2.29882578	1.8544E-08	TREM2	-3.15520928	1.1103E-14
TMEM217	2.29518755	9.3085E-06	HECTD2	-3.15204087	1.5439E-16
NREP	2.2916268	4.3363E-09	GUCA2A	-3.13968436	0.01649449
TACSTD2	2.29069842	0.00227231	RBP1	-3.1165628	9.8463E-20
HAS1	2.28936604	1.0439E-06	WFS1	-3.11577719	8.6622E-07
SH3BP4	2.27510901	9.9502E-09	PCGF2	-3.10825744	6.7747E-06
PTGER3	2.27278127	0.00241545	BBS5	-3.09919629	0.00011743
SGOL1	2.26791399	0.00093534	RP3-522D1.1	-3.09276084	2.2763E-07
PSTPIP1	2.25841749	2.158E-16	FOLR2	-3.08312498	9.3649E-27
FLT3	2.25106777	8.7959E-22	METTL7B	-3.07512331	0.03777908
SFTPD	2.22965992	0.03897412	MACC1	-3.06114518	1.7711E-18
ZBTB16	2.21536831	3.1999E-09	AOC1	-3.06064247	0.00078092
CDCA7	2.21419506	0.03021732	RARRES1	-3.05458626	0.0001032
SLC38A1	2.21172759	4.4869E-08	CCDC80	-3.05286789	0.01991815
ICAM3	2.20035399	3.1328E-24	AP000439.1	-3.04274042	0.01898138
C6orf223	2.20023142	0.00423038	LINC01356	-3.03263953	0.00055075
IDO1	2.19736809	2.6076E-09	LINC00996	-3.03186748	2.6835E-27
UBD	2.18933144	0.03407542	ITGA9	-3.02553454	2.2295E-17
IDO2	2.18346476	0.00038512	LINC00891	-3.02079723	0.00142763
TMOD1	2.17963289	0.0061465	CRHBP	-3.01969997	3.0326E-20
SLC46A2	2.17631895	0.00414027	PLTP	-3.01405302	3.2305E-60
CCR7	2.15932354	0.00011743	SMTNL2	-3.01374001	0.00159569
RAB3D	2.15351757	0.00156966	SLC16A9	-3.001371	0.00733227
CD207	2.13254325	0.00198581	SMARCA1	-2.98830592	0.03895796

ACOX2	2.12940056	0.01424487	TNS1	-2.98152254	1.7031E-09
NT5DC3	2.1249315	0.00012156	ZNF133	-2.98064591	0.00116621
CD300E	2.11559968	6.4627E-18	FABP3	-2.97405472	2.842E-05
FAS	2.10465343	1.6621E-07	CCL18	-2.97139585	3.9621E-12
CXCR3	2.10094488	7.2646E-05	SNCAIP	-2.96981993	0.04525826
CTB-61M7.2	2.0872788	1.5655E-05	SYT15	-2.95630929	7.5806E-08
C12orf75	2.08613904	1.2195E-10	ROR2	-2.95466395	0.00128654
COL9A2	2.08213158	9.2323E-08	MMP2	-2.93769725	0.01058968
AC013461.1	2.07886977	1.3019E-20	RHPN2	-2.93359527	0.03038431
HOMER2	2.06669922	0.00023715	SLC12A5	-2.92936296	2.6628E-09
ZNRF1	2.05399433	2.1468E-05	APOC4-APOC2	-2.92468949	0.00727024
GPER1	2.04371695	4.4576E-09	AK1	-2.92217702	8.0632E-17
SULT1B1	2.04359302	0.0007102	ALDH1A1	-2.89869132	9.589E-23
GNG4	2.03565791	0.03596911	LPAR5	-2.89703706	1.4614E-06
CD79B	2.01421037	0.00184707	RP11-38M8.1	-2.89127622	0.01656743
PKP2	2.01242291	8.3276E-27	BACE2	-2.88573624	0.01917566
MAP4K4	2.0119326	1.7814E-16	L3MBTL4-AS1	-2.87908139	2.3455E-10
NOV	1.99885281	0.00113728	SMPDL3A	-2.87802703	2.4729E-21
PLAC8	1.99879531	3.3345E-17	CLDN4	-2.87680842	0.00126871
CTD-2319I12.2	1.99318509	0.00016587	EDA	-2.87577597	2.5201E-05
LIMD2	1.98115714	7.79E-35	OLFM2	-2.87209562	0.00412175
KIF18A	1.97986611	0.00763039	RGMB	-2.8712931	0.03985482
TRNP1	1.97668848	0.0264375	RP11-489O18.	-2.86625717	1.5914E-13
TFPI	1.9764425	2.7168E-05	PDPN	-2.8647496	0.03488556
GPR157	1.97130228	5.947E-11	AURKC	-2.85746299	3.4833E-07
TRAF3IP3	1.96782825	2.042E-10	MAPK12	-2.83404873	1.1466E-07
SYNGR1	1.96774297	5.1653E-06	DOK5	-2.82822791	2.9555E-05
SLC22A4	1.96767733	3.4836E-05	FRMD4A	-2.81162494	4.1878E-21
PTPRN2	1.96132536	0.00480828	VCAM1	-2.80640608	3.2381E-13
GZMB	1.9569302	0.04772289	MYO6	-2.79599154	0.00231531
LHFP	1.95518371	2.0598E-17	RP11-597D13.	-2.79083409	0.00999958
C1orf228	1.95418534	0.00557058	RP11-557H15.	-2.78779652	0.00391121
VNN1	1.95132869	2.9717E-05	SLCO2B1	-2.78495839	3.181E-21
NEGR1	1.94645974	7.5001E-05	PLXDC1	-2.78257099	2.116E-06
SLC24A4	1.93822072	0.00132739	MMP14	-2.76569666	2.8526E-17
EPHB6	1.93350529	0.0019264	NPHP1	-2.75069802	0.00115453
AQP9	1.93140386	0.00389189	CXCL1	-2.74953949	9.634E-14
PDE4D	1.92906352	4.2815E-14	SRPX	-2.74898717	0.00010599
BEND5	1.92799454	0.03823496	CTSL	-2.74591687	1.0987E-32
FGR	1.9129816	9.091E-24	CMKLR1	-2.71854335	6.9857E-31
TRMT6	1.91214643	5.2743E-17	RP11-556E13.	-2.71549018	6.538E-15
CCL22	1.91041785	0.00882857	PCDH12	-2.7029762	0.00672094
CBFA2T3	1.89692961	3.439E-09	NYNRIN	-2.70109536	0.0018089
SPIB	1.89666715	1.6037E-12	WLS	-2.69909388	2.7634E-19
ABTB2	1.89576704	0.0438651	IGF1	-2.69494119	5.7065E-27
RP11-126O1.5	1.89348387	0.01007919	PLEKHH3	-2.69457787	8.4036E-07

FBLN2	1.88740981	0.0016293	KLHDC8B	-2.69411019	2.3226E-12
SYN1	1.8826688	0.00078099	PDK4	-2.66194483	8.7392E-27
ADAM8	1.86745408	8.6372E-17	APOC2	-2.65834391	0.00028673
LAMB3	1.86176392	1.3257E-08	MMP1	-2.64963611	1.0079E-09
GPR18	1.85232399	0.00019002	SEZ6L2	-2.64631619	0.00412873
BCL2L14	1.85104932	0.0022012	FGD5	-2.64380338	2.209E-08
PDLIM1	1.84995817	1.3502E-07	FABP1	-2.63917723	3.0265E-06
RP11-542M13	1.84814327	0.01135478	HOXB7	-2.63847675	0.02891829
PTX3	1.84004087	1.6974E-06	INPP4B	-2.6333239	5.2945E-09
AP003774.1	1.83213674	0.02319884	MT1M	-2.63065547	0.00053925
NKX3-1	1.82321043	0.00735799	FMNL2	-2.61699583	7.6646E-27
FLT1	1.8187843	6.1301E-07	TNFRSF11A	-2.60677071	6.7551E-05
FAM110B	1.81430358	0.03874839	ACE	-2.60524026	9.3545E-07
SMCR5	1.80752913	4.0086E-06	SCAMP5	-2.59518762	0.01595967
LILRA2	1.80572091	2.6198E-17	ADORA3	-2.59190536	3.8116E-34
MARCO	1.80015836	0.00074879	FABP4	-2.5890615	9.6898E-05
BANK1	1.79818452	0.00162701	GAREM1	-2.58905656	4.0047E-09
ETV7	1.7962092	0.00293809	LINC01094	-2.58203443	2.3378E-07
CES1	1.7847548	0.0155387	HS3ST1	-2.58068552	4.0047E-14
AIM1	1.78099541	1.7817E-40	KIF3C	-2.57645914	0.02081186
RAMP1	1.78075028	0.00241545	PLS1	-2.57108045	0.00178888
TPPP3	1.77721744	1.6864E-08	PRAP1	-2.5689124	0.00877767
MFSD14C	1.77341506	0.03943303	MICU3	-2.56860976	0.02310603
FRY-AS1	1.7654313	0.03314175	ACP5	-2.56749841	6.0862E-14
HPGD	1.76132913	0.00024889	MAF	-2.56503911	1.1873E-29
VENTX	1.74666532	2.4988E-06	CD276	-2.55798746	1.1537E-15
SLAMF9	1.74563746	8.7291E-10	FAM178B	-2.55711367	0.00146506
ADGRE3	1.74384684	8.8285E-07	RP11-522I20.3	-2.556892	0.0219027
AKAP12	1.73818663	0.00209789	SPIRE1	-2.55333002	1.4562E-23
ARNTL2	1.72936895	0.01049924	DAB2	-2.55084626	8.8019E-33
TYMS	1.71557961	0.00076991	PLA2G2D	-2.55077881	0.00290565
RP13-270P17.	1.71378111	0.00014645	SIGLEC12	-2.54489861	1.6997E-14
TREM1	1.71098288	1.1819E-26	SCD5	-2.53185561	0.00065445
BCL11A	1.70793253	8.3841E-10	ZFP92	-2.53067312	0.04049594
LA16c-444G7.	1.70595169	0.00358783	TFCP2L1	-2.52850358	9.5278E-10
INSR	1.70120817	1.6616E-32	MYZAP	-2.52479782	0.00013602
TOP1MT	1.69624854	5.9874E-09	C1QB	-2.52426455	6.3642E-19
MGST1	1.69077553	4.1823E-07	LGMN	-2.52304539	5.2803E-41
AC002456.2	1.68592096	0.00352889	IL6	-2.5228255	7.0775E-12
S100A6	1.68271758	1.7241E-07	CD28	-2.51691516	0.01899689
NFIX	1.67149977	0.01597123	NBL1	-2.5166219	9.407E-12
GAPT	1.66951474	1.737E-14	SASH1	-2.50475284	1.7672E-19
ASGR2	1.66812136	2.0921E-11	CCDC40	-2.49667125	0.01060268
S100A4	1.66768476	3.3708E-07	IFITM10	-2.48791617	6.7728E-28
EREG	1.65993668	9.9213E-14	LILRA4	-2.48000465	2.004E-14
LYST	1.65660292	6.1574E-26	NQO1	-2.4740117	2.8529E-10

SYCP2L	1.65386051	0.00419867	DHFRL1	-2.46318429	0.00016704
MPZL3	1.65382902	0.00287518	RP11-1008C2:	-2.45409233	0.00677355
LYPD3	1.65110715	1.5439E-16	CYTL1	-2.44664306	0.00072424
PROSER2	1.64972766	0.03893821	ENO2	-2.43440949	0.0030141
IGFBP7	1.64542874	6.24E-07	COL6A2	-2.42378046	0.00172763
SNX20	1.64333968	5.5814E-09	PRELID3A	-2.42200131	0.00662686
SYNE3	1.64217634	6.3002E-06	LACC1	-2.41601368	2.2002E-29
ACPP	1.64188374	0.0001125	TRPV4	-2.40454674	0.01459839
AC005264.2	1.62580282	0.03636937	LPIN3	-2.40022309	0.0464804
EFNB1	1.6221357	0.00431437	PHGR1	-2.39924257	1.0439E-06
WDR66	1.615249	0.04126656	IL2RA	-2.39580471	3.8308E-07
CAPN2	1.5984019	2.7363E-12	RP11-588H23.	-2.39567879	0.00075146
CDH23	1.59006972	4.5173E-05	C1QC	-2.3943329	2.4019E-13
INHBA-AS1	1.58787659	0.04875857	CCDC152	-2.3843154	3.296E-12
SIRPB1	1.58617181	0.00035176	A2M	-2.38139607	2.9748E-24
PLP2	1.58315443	5.9069E-29	HOMER3	-2.38046939	3.5426E-17
CD1E	1.57556155	3.3911E-05	NR1H3	-2.38019609	2.8758E-16
CEACAM4	1.57438274	1.3744E-09	PLA2G7	-2.35863934	7.5367E-12
CD37	1.5732547	3.7204E-57	COL6A1	-2.35697982	0.0185305
CTD-2020K17.	1.57218984	2.6696E-05	PTGDS	-2.35665907	0.0006397
TMEM154	1.57070661	4.2734E-13	ZNF354C	-2.34837874	0.00351174
ADHFE1	1.56883494	0.02242526	LHFPL2	-2.34528556	1.5269E-30
AREG	1.56855114	1.0637E-30	PROS1	-2.33432021	2.8728E-09
OXER1	1.5672692	0.02366515	COPZ2	-2.31541092	7.0033E-09
STEAP4	1.56594348	0.04817238	CNRIP1	-2.31458472	4.3576E-11
C9orf139	1.56223797	0.01892512	GSTT2B	-2.30365267	0.00021427
AKR1C1	1.55987548	0.02851513	ZNF382	-2.27893552	0.02177184
MCM8	1.55794293	0.00928207	CERCAM	-2.27510462	0.00017115
TLE1	1.55377413	6.5355E-05	SUCNR1	-2.27340916	9.1631E-09
CLCF1	1.55108991	0.04226056	TROAP	-2.2671617	0.00507006
DKFZP434I071	1.54976501	0.02676799	MT1X	-2.26438464	5.8306E-19
DAPP1	1.54940808	5.9252E-10	PLD3	-2.2617548	2.9697E-42
RAB27A	1.54461615	5.8798E-08	ESR1	-2.25700698	0.00633995
IFITM2	1.54335629	3.398E-22	FN3K	-2.25382188	7.131E-09
CCNE2	1.54300797	0.00276387	CCL3	-2.24750529	1.7551E-25
PVT1	1.53826596	0.00869204	CCL4	-2.24328817	1.8092E-26
XKR8	1.53324643	4.2154E-10	VAT1	-2.2377395	3.2556E-17
PRDM8	1.52998795	3.878E-07	CCND1	-2.23289895	9.9548E-15
FRY	1.52169601	0.02193014	ZKSCAN4	-2.23144739	1.0854E-05
DTNA	1.51441381	0.00118034	LRFN1	-2.22781817	3.1745E-06
G0S2	1.51276237	4.9194E-18	GUCA2B	-2.21407512	0.00257299
SLC35F2	1.50370651	0.00129405	EML1	-2.21291632	0.0063171
ZDHHC1	1.49729684	0.000559	PLD6	-2.21244589	0.03920715
AP1S3	1.49383916	1.428E-06	HIST1H2BJ	-2.20802729	0.00421203
AL592183.1	1.48758057	0.00414919	MMP15	-2.20681873	0.00326364
IFITM1	1.48685329	5.9069E-05	HSPA1B	-2.20096459	1.4901E-06

C1orf162	1.48651871	1.6711E-23	TMEM176B	-2.19975676	2.0142E-10
CLEC4D	1.48090354	0.00012996	CYP27A1	-2.1965547	3.3911E-05
INHBA	1.4726453	2.36E-05	DOCK4	-2.19482896	7.4451E-23
RHOF	1.47235304	4.7287E-07	FARP1	-2.18665977	8.8583E-05
GDPD5	1.47021435	0.01769007	UNC5B	-2.18184267	1.4793E-08
ST3GAL4	1.46431765	0.00200437	PDP2	-2.16720989	0.04232238
SPTLC2	1.46153431	1.6119E-25	CIART	-2.16104368	0.01741942
S100A9	1.4470689	0.00213189	LIPA	-2.15731288	1.2649E-46
LGALS2	1.44064362	2.3866E-05	ARHGEF12	-2.15639267	3.4408E-20
LUCAT1	1.43881209	5.7007E-05	DNASE2	-2.15633646	5.4009E-20
CITED4	1.43766351	1.9956E-08	ZNF57	-2.15545675	0.00555814
BZRAP1-AS1	1.43510579	0.00202119	ACP2	-2.15448086	2.7978E-28
ATP2A3	1.43193688	0.0032335	KIAA1211L	-2.1483676	1.9615E-05
CD55	1.42947663	4.4962E-22	AATK	-2.14741609	0.00097515
ZNF703	1.42926655	1.6911E-05	COL23A1	-2.14381505	0.00024649
SLC11A1	1.4268528	2.4931E-06	DYSF	-2.14305953	0.00130686
LMNB1	1.42075759	9.6295E-10	TMEM163	-2.13995733	1.1711E-05
LINC01272	1.42062809	5.1204E-05	PGBD1	-2.13403315	0.00204383
GALNT3	1.41957468	1.428E-05	TMEM176A	-2.12846228	1.8996E-15
CYFIP2	1.41674712	0.04119908	OLFML2B	-2.12422521	3.399E-06
FPR1	1.41467998	3.3003E-08	ITGB5	-2.11350773	4.8075E-30
MIR3945HG	1.41274413	4.6657E-06	FAM198A	-2.11074095	0.00017646
SVIL	1.41069258	3.6453E-05	PTCRA	-2.1076751	0.03303034
TSPO	1.41047462	3.7231E-11	TLE6	-2.10710795	0.02398015
KCTD15	1.40834919	0.01313178	HPSE	-2.10511008	2.3965E-07
SLC9B2	1.40788737	0.03396524	GLMP	-2.10144148	2.9675E-28
INPP5A	1.40524573	0.0290566	GPR34	-2.10048453	1.5912E-15
FAAH	1.40292264	0.00624773	SLC25A23	-2.09595577	0.03376335
PPP1R3B	1.40274001	1.67E-07	FCGR3A	-2.09530484	4.8509E-09
KIAA0101	1.40114273	0.00021604	EGFL7	-2.08960022	0.00027262
PSTPIP2	1.39552898	3.0061E-25	SLC46A1	-2.08042887	2.4017E-12
SH2D3A	1.39551645	0.00216869	ASIP	-2.07911113	0.02834095
KIAA0922	1.38755337	1.1311E-05	C3orf18	-2.07839118	0.04158943
ANPEP	1.38416823	2.0025E-05	C1QA	-2.0770858	1.9041E-19
TMEM71	1.38127864	8.8669E-07	ENPP2	-2.07222558	1.0104E-15
ADORA2B	1.38126229	0.00094475	CCL4L2	-2.06360746	3.4963E-19
AF064858.8	1.37627123	0.01495809	AC069363.1	-2.05924134	0.00012369
GJB2	1.37602256	0.00091186	EGR2	-2.05172154	3.3923E-09
ENPP4	1.37426006	0.00160987	SLC38A7	-2.04842833	2.604E-12
KCNMB1	1.374221	3.6947E-08	SLC38A6	-2.03700648	2.6029E-12
CSF3R	1.37369122	5.6965E-26	P4HA2	-2.03326335	0.00150053
MAP4K1	1.36990976	5.7734E-10	SPSB1	-2.03011196	2.7901E-05
SLCO4A1	1.36810662	2.8651E-05	PIK3R3	-2.02509173	9.7039E-07
RAB11FIP4	1.36381918	0.007388	RPP25	-2.02035174	8.3493E-10
CIDEB	1.36288385	1.5936E-05	PLCL1	-2.01592722	5.9148E-07
CTSW	1.36237509	0.02526954	F2RL3	-2.01340386	0.01670686

SERPINA1	1.35604386	5.7327E-11	A4GALT	-2.01111991	1.3595E-06
CHAF1A	1.35210603	3.2849E-07	KCNMA1	-2.0086202	2.4962E-15
FAM118A	1.35204876	2.547E-11	MITF	-2.00149541	5.8956E-20
CA5B	1.34732992	5.8168E-05	ZDHHC19	-2.00143649	2.4153E-09
CCR6	1.34704541	0.0034417	STAB1	-2.00121954	3.0385E-18
RP6-159A1.4	1.34676169	0.00041387	SCD	-1.99902829	1.3976E-13
TESPA1	1.3447035	0.02182697	PDCD1LG2	-1.99366312	0.00140547
FAM65C	1.34398351	0.02793167	HRH1	-1.98862432	7.0155E-05
P2RY14	1.34368167	2.2523E-14	HMOX1	-1.98624223	2.2865E-46
RIPK2	1.34178562	1.6037E-21	FCHO2	-1.98029904	1.9309E-12
AC093495.4	1.33355074	0.03600708	WWP1	-1.98018405	4.4618E-25
TRAF1	1.33147802	0.00689781	PLA2G15	-1.967885	9.6195E-11
GBP5	1.32970095	4.7162E-06	LGALS4	-1.96774042	0.00404395
TTC39C	1.32933165	1.0136E-06	DTNB	-1.9675655	2.2747E-06
TCTN3	1.31912794	1.3322E-07	SLC36A1	-1.96729695	8.7503E-12
ALPK2	1.31812697	0.0193363	P2RY11	-1.96530522	2.2963E-05
RETN	1.316677	0.03332049	SERINC2	-1.96081344	0.00415086
AC137932.4	1.31467585	0.00844774	PPFIA3	-1.96062067	0.01167522
TUBA4A	1.31440833	9.3398E-07	ADGRG6	-1.96013244	3.0449E-07
BASP1	1.31246327	0.00438278	GPR82	-1.95532654	7.6954E-12
PRKAG2-AS1	1.31074874	1.925E-10	CCK	-1.95432272	0.0271645
GK	1.30505273	1.5145E-10	ATAD3C	-1.9503574	0.03159776
PLEKHA5	1.30205811	3.4278E-06	LYVE1	-1.94711315	2.6582E-06
CACNA2D3	1.296885	2.0153E-12	CADM1	-1.9421233	0.00014008
S100Z	1.29533243	0.00011902	B3GNT8	-1.9392918	1.2574E-05
PDE2A	1.29514566	0.00162276	MS4A4A	-1.93905335	2.993E-23
FMNL1	1.29221114	3.931E-17	CLCN4	-1.93568198	3.4846E-05
EPOR	1.29016503	0.0089238	PKD2L1	-1.93557624	0.00869928
ANKRD28	1.28795945	2.3637E-17	HES1	-1.92897813	1.3753E-15
SYTL3	1.28582347	2.3192E-08	FKBP9	-1.91288167	6.3957E-05
ZNF250	1.2855999	0.00443367	ISYNA1	-1.91021807	2.8942E-09
LINC01588	1.28506248	0.00535302	ARRDC4	-1.90626799	1.8798E-11
RP11-796E2.4	1.2816638	1.3508E-06	EMILIN1	-1.90297981	4.1536E-05
CCND2	1.28160613	2.3342E-07	GSTA4	-1.89979725	0.00807438
GHRL	1.27945881	5.9388E-07	SDC3	-1.89656954	2.366E-17
SGMS2	1.27700429	2.533E-05	GPR161	-1.8916103	0.03274379
DGKD	1.27581653	5.1419E-09	ZNF613	-1.88943025	0.01671066
RP11-147L13.	1.27521927	5.0544E-05	TSPAN15	-1.88744845	2.2187E-14
SLC25A37	1.27509337	3.7006E-17	TRMT5	-1.88139756	0.00123638
CXCL9	1.27256273	0.02224898	GIMAP5	-1.88063811	2.7982E-06
ACAP1	1.26726232	6.3541E-06	NRAV	-1.88024149	0.03577855
MAML3	1.26701599	2.4526E-10	SERPINF1	-1.87806029	6.4422E-34
DGKA	1.26427016	2.5887E-06	PER3	-1.87753344	2.2578E-15
LILRA5	1.26365355	2.6881E-14	RND3	-1.87553172	1.2976E-10
JAK3	1.26343636	2.5597E-06	JUN	-1.87049374	4.0047E-09
CDKN2D	1.26156217	6.4339E-06	TBC1D16	-1.86950905	2.0606E-09

LINC00528	1.26124327	8.5303E-12	ZNF485	-1.86902869	0.02016341
FAM129A	1.26098806	1.1485E-08	HSD3B7	-1.86831221	4.6943E-08
MFSD6	1.26089496	0.00076857	CTD-2376I4.2	-1.8664402	0.04155543
SP140	1.25945944	0.00384701	EPN2	-1.86590658	6.2503E-06
RYR1	1.25907683	6.7445E-06	SLC40A1	-1.86047227	2.4153E-09
CTC-529I10.2	1.25575347	0.02496582	NGFRAP1	-1.85873916	2.3835E-14
HOTAIRM1	1.25518891	3.8972E-10	P3H3	-1.85696139	0.01958561
PLCXD1	1.25459366	3.2655E-07	ABCC5	-1.85682545	7.397E-09
HDAC9	1.25176066	2.8803E-06	EPHX1	-1.8567746	1.0587E-16
ELK3	1.24932375	1.8346E-05	MAPK13	-1.85291442	0.00661491
HAPLN3	1.24838027	0.00107157	NAT8L	-1.85232321	0.0391008
FAM46C	1.24555145	0.04252883	QPRT	-1.85206196	1.3798E-21
PRKCE	1.24349739	1.8374E-05	ARL6	-1.85021614	0.04365353
C16orf54	1.24045236	0.0031356	TSHZ3	-1.84953295	0.00115587
RP11-37B2.1	1.23861789	0.00031353	IGFBP4	-1.84934601	4.3599E-14
CTD-2547G23	1.23850155	0.00447534	SOWAHD	-1.84268468	0.00043473
RP11-405M12	1.2372422	0.00495524	THAP8	-1.83573353	0.00058382
IL1RL2	1.23529629	0.00964493	CABLES1	-1.83489584	1.7077E-05
PRKCH	1.23399361	9.7398E-06	KCNMB4	-1.82850097	2.9079E-07
NBEAL2	1.23085152	8.6885E-05	TCN2	-1.82703639	3.7896E-20
ITGB2-AS1	1.2264988	1.1621E-08	FHAD1	-1.82379056	1.8155E-05
C19orf38	1.22303152	3.2486E-18	SNHG12	-1.82280826	9.6452E-37
AP003774.6	1.22296104	0.00747823	EFEMP2	-1.82109488	0.00168032
CCSER1	1.21802913	0.0006805	PLEK2	-1.81923304	0.01068756
SPON2	1.21561387	7.6319E-07	MRC2	-1.81773722	2.5591E-09
MYLPF	1.21505155	0.00018832	STXBP1	-1.81731	0.00016831
STK26	1.21459152	8.0831E-06	GCLC	-1.81676929	6.0171E-27
CDK2AP1	1.21361147	1.9341E-19	CCL3L3	-1.81320923	1.2043E-12
PID1	1.21299038	9.6963E-18	WBP5	-1.81308208	1.5253E-11
PDE3B	1.21144888	1.1191E-06	DCBLD2	-1.81299083	0.00038311
TMEM120B	1.21144692	0.00041204	GRIN2D	-1.80824962	0.02016664
EIF2AK4	1.21072368	1.3662E-24	NISCH	-1.80719142	8.253E-19
REV3L	1.21011667	1.7165E-13	TTC28	-1.80670466	0.00114023
GGT5	1.20906233	0.03503446	PLAU	-1.79987559	6.7455E-12
CD1A	1.20714472	0.00676461	CREG1	-1.7969368	5.6095E-58
MYO1G	1.20621723	4.841E-08	RASAL1	-1.79174054	0.01068205
CST7	1.20296135	0.00116714	CCDC102A	-1.78607204	0.00164316
KIFC1	1.19889425	0.00111343	FAM20A	-1.78561792	0.01004252
ISG20	1.19659135	5.0866E-21	ARHGAP6	-1.78495748	7.203E-08
FLOT2	1.19448785	1.4677E-08	LBX2-AS1	-1.78158361	0.00106997
RP11-231C14.	1.19407729	0.00187537	SDCBP2	-1.77652658	0.04534639
WDR59	1.19307157	1.9956E-08	TSPAN13	-1.77579959	0.00047046
SLC2A3	1.18596796	7.015E-12	HAGHL	-1.77485949	0.00929323
TNFSF14	1.18306693	0.00362408	SIGLEC11	-1.77445404	2.0224E-05
FCGR2B	1.18283148	0.00803652	ZNF571	-1.77345931	0.0004009
CSTA	1.18076696	2.0221E-24	IDH1	-1.76903175	2.339E-26

IRF4	1.18016878	1.9176E-09	FAM127C	-1.76857878	0.00706903
ADD3	1.17992921	4.3831E-12	HSPA6	-1.76842926	0.00213062
CLEC10A	1.17984669	1.7661E-12	DYNC2H1	-1.76304123	0.00182662
CREM	1.17393851	2.0664E-10	MCC	-1.76294695	0.02258067
WEE1	1.17260701	0.0008895	ST5	-1.76074359	1.5803E-06
CALCRL	1.16888401	0.00017378	NFIA	-1.75646125	5.7591E-08
FGGY	1.16523079	0.00038987	COX7A1	-1.75498989	0.00067521
EMP1	1.16316168	3.042E-06	PRTFDC1	-1.75489106	0.0132119
NUDT7	1.15978262	0.0400315	GIMAP8	-1.74766199	1.8628E-08
CXCL10	1.15479896	0.03453441	ZNF691	-1.74546068	0.006796
PON2	1.14603598	3.1511E-06	ZNF624	-1.73707114	0.01339053
ZNF674-AS1	1.14501041	0.00613085	LGALS3BP	-1.73632391	1.0837E-19
TMEM158	1.13836915	0.00328693	TMIGD3	-1.7359064	9.1637E-10
ERV3-1	1.13704414	5.5764E-05	MMP25	-1.73415993	2.4074E-10
PIDD1	1.1357453	0.02799193	KIFC3	-1.73355236	3.2817E-05
CD244	1.13399472	0.00018441	RP11-172H24.	-1.73186548	0.02801753
PRAM1	1.1313077	1.0246E-06	WBSCR27	-1.73072974	1.9956E-08
TMEM170B	1.13103046	4.9005E-11	CTSC	-1.72966965	1.1065E-28
GFOD1	1.12689232	4.1125E-05	CELA1	-1.72937505	0.00473827
IL1R1	1.12159476	9.1315E-06	C1orf21	-1.72935657	0.00022607
GPX7	1.12038913	0.0185305	F3	-1.72822185	2.74E-07
HGSNAT	1.11846527	2.3354E-10	KREMEN1	-1.72246096	0.0020692
BEX2	1.11798046	0.00982189	SLC37A4	-1.72063973	5.8548E-10
ITGAX	1.11474972	2.553E-11	LGALS3	-1.71580026	3.2209E-14
RASGRP2	1.11215083	6.3932E-07	TCTEX1D1	-1.71190864	0.00246246
TCTEX1D2	1.11015337	0.04395584	TMEM86A	-1.71178618	6.6052E-07
TNFSF8	1.10836635	0.00556506	SNX24	-1.71024516	2.1056E-12
NFAT5	1.10587746	5.5695E-07	PIF1	-1.70716654	0.00119445
TIPARP-AS1	1.10515661	0.0182165	SEPT10	-1.69164815	0.00824207
SLC2A6	1.10412675	0.00202119	USP54	-1.69011016	0.03333419
RAB39B	1.1038552	0.0271645	GCNT1	-1.68830593	4.5773E-11
RP11-666O2.2	1.10207404	0.02851513	CDAN1	-1.68382807	0.03532677
AC093673.5	1.09965888	0.00019465	BLNK	-1.68031914	1.1524E-07
TK1	1.09166391	0.04461353	CD320	-1.67445179	2.2695E-08
RP11-568N6.1	1.08916418	0.03121418	IGF2R	-1.66839227	3.4278E-12
SLC7A1	1.08908988	3.5411E-07	NOTCH1	-1.66650612	2.3723E-15
TET2	1.08854689	4.3209E-17	ZNF420	-1.6630733	0.00779805
RNF139-AS1	1.08737597	0.0116253	SLC2A5	-1.66215509	0.04868751
WARS	1.08572817	1.0451E-11	CIPC	-1.65928018	1.93E-08
TUBA1A	1.08548826	5.2026E-10	SERPINH1	-1.65912457	2.2704E-05
GBP1	1.08543489	4.3396E-06	RP11-536K7.3	-1.65714556	0.00593855
MALT1	1.08538033	3.574E-11	FUT10	-1.64899426	8.0419E-06
MGC16275	1.08529712	0.00121753	FBXO16	-1.64838537	0.00278255
BCR	1.08314871	0.0027657	LAMB2	-1.63860343	1.5583E-05
RP11-126K1.6	1.08266577	5.5009E-05	EGR1	-1.63804287	8.9839E-09
PPA1	1.08088642	9.7896E-13	CFH	-1.63491976	2.6892E-05

RP5-1136G13.	1.0777442	0.01432992	MT1E	-1.63288968	0.00332283
NUP210	1.07649148	0.00087119	SPATA7	-1.63188668	3.538E-06
LINC00869	1.07584468	5.1814E-05	CTTNBP2	-1.63031076	5.9105E-11
LINC00877	1.07581945	2.77E-05	DNAJC5B	-1.62731686	2.8106E-05
CHRNE	1.07454778	2.3957E-05	SCARB2	-1.62218544	2.2569E-18
VCL	1.0737744	2.9167E-05	USP51	-1.62206648	1.8506E-06
RP3-394A18.1	1.07303576	0.02282097	ARMC9	-1.6138293	0.02146991
RP11-443B7.1	1.0720355	0.00120162	CTSF	-1.61336081	2.8529E-10
PKIB	1.06821838	1.4049E-05	DNASE1L3	-1.61144286	1.1738E-27
MX2	1.06739952	1.0002E-12	APPL2	-1.61071155	2.525E-13
OCIAD2	1.06656898	0.00965279	RENBP	-1.60482987	1.4621E-13
RAB11FIP1	1.06550434	6.8556E-15	APBA1	-1.60318177	0.0001469
NRARP	1.06506533	2.4677E-06	BAG3	-1.60199174	0.0003055
PLK2	1.06422071	1.6974E-05	TM7SF2	-1.60150388	0.00018325
CLEC4E	1.06394192	9.8239E-10	OPTN	-1.59870717	0.00084775
CASP9	1.06293765	5.7996E-08	DEXI	-1.59534324	1.0854E-05
RILPL2	1.05986934	4.771E-19	FAM213A	-1.59449085	1.3689E-17
HELLS	1.0598341	0.00674917	TEF	-1.58677559	0.04056918
UBR5-AS1	1.05847715	0.04617566	CD9	-1.58594296	0.00046174
GEMIN4	1.05707076	0.01754792	RRAGD	-1.57600306	1.0964E-19
BCL7A	1.05641389	5.9494E-06	PAQR8	-1.57208908	2.1377E-07
RP11-347P5.1	1.04951123	7.2987E-05	NMRK1	-1.57106372	7.8447E-15
LY6K	1.04785795	0.04299547	NEIL1	-1.57053032	0.0038028
HMGA1	1.04327485	2.5553E-09	SGMS1	-1.56975978	2.7338E-12
AGO2	1.04232679	8.053E-09	TPD52	-1.56942993	9.6676E-12
TRMT1	1.04230248	7.2735E-07	TMEM51	-1.56748105	3.0165E-17
LSP1	1.04070775	2.7001E-05	CFB	-1.56619129	0.03620649
CBX6	1.0392884	1.3555E-14	CD209	-1.5653859	1.4954E-17
MYOF	1.03820463	7.9198E-06	MMD	-1.563321	3.4195E-09
STK4	1.03630729	3.0648E-13	NEURL2	-1.56210612	0.01074296
EPB41	1.03542693	5.3678E-07	TMEM138	-1.56071994	4.6895E-28
PLD4	1.0346866	4.1048E-09	ITM2C	-1.56032839	4.8799E-35
HIVEP2	1.02704654	0.01629241	RP11-624C23.	-1.5523682	0.00812138
ATP8A1	1.02425271	0.02091825	AC005082.12	-1.55169666	0.00713094
PHF19	1.02159777	4.7432E-06	GNPDA1	-1.55115229	4.6306E-19
CPVL	1.02151005	1.6708E-20	RASL10A	-1.55025262	0.00362747
GPR146	1.01953039	0.00118034	IER5L	-1.55006308	2.3661E-08
HAAO	1.01721638	9.9518E-05	EGLN3	-1.54807135	0.00026196
SIDT1	1.01579952	0.00081979	LRP5	-1.54788943	6.967E-07
GINS4	1.01353369	0.0037213	BHLHE41	-1.54552902	0.00107651
CERK	1.01187782	5.8967E-07	DMXL2	-1.54521147	1.425E-15
POU2F2	1.01119441	6.1708E-07	TSPAN10	-1.54399976	0.01948067
CORO1A	1.00575457	1.6603E-19	PEX11G	-1.54316308	3.6967E-05
CD1B	1.00425696	0.00392785	SNX25	-1.53102411	0.00823784
LTA4H	1.00418801	1.4759E-13	PAX8	-1.52725629	0.03593172
MINCR	1.00172793	0.01012187	ABCC3	-1.52614979	1.1488E-05

GABPB1-AS1	0.99957301	3.9326E-09	DOK3	-1.52453054	1.6981E-08
IL16	0.99911203	2.2062E-14	TTC23	-1.5224881	0.02332417
BORCS5	0.99842855	1.0883E-08	SIGLEC1	-1.5223692	5.7706E-09
MNDA	0.99820251	1.3976E-13	SPRED1	-1.52085737	9.6512E-14
CEP83	0.99745777	1.5047E-07	EMP2	-1.51945041	1.3264E-07
AMPD2	0.99712932	2.3078E-11	RP11-347C12.	-1.51693342	0.02856999
PLB1	0.99651929	0.00010569	GATM	-1.51527699	6.2901E-25
ZNF487	0.9955305	2.2834E-06	SLC2A8	-1.50949743	1.571E-09
MAP2K1	0.99486914	4.0782E-18	GIMAP6	-1.5084383	5.3249E-09
GPR65	0.99433606	1.1092E-12	STON2	-1.50643201	1.9677E-05
GAN	0.99218753	0.00302883	CORO2A	-1.50335422	9.7184E-05
MBD5	0.98643194	0.00513027	HK3	-1.4966769	7.8664E-05
TRIM25	0.98458114	8.5818E-05	EPAS1	-1.49513254	1.2089E-07
RP3-508I15.2	0.98445386	0.03761777	ETV5	-1.49489531	9.4737E-18
RP5-1171I10.5	0.9843208	2.0916E-10	ARMC7	-1.49261006	0.00033926
TRAF3IP2	0.98416284	0.02642147	LSS	-1.49258158	4.4967E-05
NOD2	0.98166992	0.0015563	S1PR2	-1.49108915	6.1704E-07
MYD88	0.98161195	1.2199E-15	SOCS2	-1.48603848	2.8829E-05
NLRC5	0.98131114	0.00066523	OSGIN1	-1.48342414	0.0011827
LINC00115	0.98067675	0.03356864	ACSM5	-1.48266051	0.03912439
RTN1	0.97955963	1.4004E-06	CLDN7	-1.4795037	4.2174E-12
KCNN4	0.9790346	0.00126264	ZSCAN26	-1.4793306	0.00699916
TNFAIP6	0.97797899	0.04452966	DNAJB1	-1.47931989	0.00029489
LDLRAD4	0.97778925	1.3903E-08	CD59	-1.47929119	7.7687E-15
PARVG	0.9762229	5.1444E-28	UAP1L1	-1.47808869	1.5047E-08
PDCL3	0.97229194	8.5432E-17	SGPP1	-1.47768577	6.6794E-15
THBD	0.97096899	4.3699E-05	TDRKH	-1.47611063	0.01628263
TAP1	0.9705177	1.5137E-09	GBA	-1.47524275	1.8427E-15
SLC16A10	0.9697769	0.00954504	CHPF	-1.4745404	0.00928867
CD1D	0.96886848	1.6927E-10	GPC4	-1.47339912	0.00577102
CRIP1	0.96725009	4.79E-14	SGPL1	-1.47242767	2.6123E-14
NACC2	0.96670812	0.00501179	GPAM	-1.47092786	0.03035873
DNMBP	0.96522194	0.00050049	GADD45G	-1.46790747	5.9773E-06
RP11-473I1.5	0.96140807	0.02310603	OPLAH	-1.46273572	0.00048815
METRNL	0.95810201	6.4245E-14	CBY1	-1.46240929	0.00345208
EIF4E3	0.95795108	0.01355419	ADAP2	-1.46154592	1.2698E-34
SORL1	0.95679452	3.0625E-07	IQCG	-1.45979825	3.8497E-05
CHST2	0.95517973	0.00204383	DNAJB4	-1.45788182	3.7302E-07
CCDC6	0.95507939	4.6265E-09	ZNF570	-1.45224933	0.0003532
DEPTOR	0.95456373	0.00644421	PRDX1	-1.45224261	1.3933E-73
LTB	0.95281684	0.00056779	CYB561A3	-1.44973568	1.0344E-16
AC016831.7	0.95211816	6.7408E-06	ANKH	-1.44532425	4.6515E-15
RPPH1	0.9516438	0.00045936	AP2A2	-1.44290085	8.5612E-21
TIMM17B	0.95132591	1.8708E-08	WNT5B	-1.44110669	9.9518E-05
AP1S2	0.94859286	8.9195E-12	TMEM204	-1.43441873	0.00080535
OLR1	0.94661651	7.9381E-08	CD14	-1.43082009	9.7754E-14

STK10	0.94579714	1.2668E-05	LMAN2L	-1.42873986	0.00291495
SNHG9	0.94551042	4.9483E-06	RP11-356J5.1:	-1.428474	0.01205406
KCNC3	0.94401721	0.02479002	SLC45A4	-1.42658724	5.8405E-07
AC144652.1	0.94369845	0.00674917	SEMA4C	-1.42652457	2.18E-08
OPN3	0.93878408	4.1845E-08	DYX1C1	-1.42374463	0.0492902
CHPT1	0.93841619	3.3028E-09	GIMAP7	-1.42015639	3.6579E-09
TP53BP2	0.93825112	4.0949E-12	HYAL2	-1.41490583	1.2963E-07
TPCN2	0.93683631	0.00350195	DEPDC7	-1.41450697	0.02018171
FANCD2	0.93629631	0.02891829	RHOBTB3	-1.41235154	8.4963E-08
QPCT	0.93584325	7.0568E-05	CTSK	-1.4122042	0.04418675
PELI2	0.93493661	0.00093451	PLPP6	-1.41025876	0.04100703
TCTE3	0.93456806	0.02995337	TRG-AS1	-1.41015513	5.8967E-07
C14orf80	0.93391296	0.01218411	TLR7	-1.4095417	5.5695E-07
IL27RA	0.93362095	7.1624E-07	TNFRSF13C	-1.40899572	0.00583545
SMAP2	0.93293757	3.5497E-21	PDLIM4	-1.40884347	0.04573661
SUN1	0.92890644	0.01664019	S100A13	-1.40285362	3.5595E-19
HLA-DOB	0.92657408	0.00884853	F12	-1.40063961	0.02110486
ITGAL	0.92574504	0.00567818	MMP9	-1.40056685	0.00025398
MBOAT2	0.92560962	0.03365001	SH3PXD2A	-1.39098591	1.0512E-09
PRKCB	0.92520985	1.5043E-06	NLN	-1.39097499	2.6911E-08
RP11-798K3.3	0.92489317	0.03398375	ANGPTL6	-1.3891631	0.0248322
PTK2B	0.92432166	1.7771E-12	TMEM44	-1.3891014	0.00101454
OAF	0.92087557	0.00410188	LYSMD4	-1.38868823	3.7849E-05
LRRFIP1	0.91855997	2.7677E-12	SLC22A23	-1.38538246	0.00048654
CSGALNACT1	0.91595739	0.00933978	CD81	-1.38356835	9.3649E-16
DUSP4	0.91471225	0.00001093	TUBG2	-1.38252559	0.02020604
C9orf72	0.91290148	1.6839E-11	LRP12	-1.38250386	3.3573E-05
CCDC88C	0.91106528	0.02508317	SDS	-1.37924875	0.00017115
SATB1	0.91087543	5.6286E-05	RTN2	-1.37855585	7.9423E-10
ZNF445	0.9094478	0.00478673	EIF5A2	-1.37797209	0.00046493
CLEC12A	0.90835787	2.4006E-05	SLC26A11	-1.37514489	3.7522E-10
NLRP3	0.90707027	0.00012823	CTD-2350C19.	-1.37488202	0.03160115
NDEL1	0.90670192	6.3272E-09	RASAL2	-1.37202058	1.0055E-06
LINC00937	0.90458793	0.03929165	DDR1	-1.37094244	0.03095614
THAP2	0.90404403	0.00020112	MFSD13A	-1.37038298	4.9067E-07
BTG1	0.90391574	1.4514E-13	PTRF	-1.36494058	0.03577855
SERPINB1	0.90312203	1.7083E-09	BMP2K	-1.36356047	1.1809E-25
TES	0.90203336	4.6966E-13	DNAJA4	-1.36316912	0.0004596
ZNF281	0.90031125	1.3178E-07	FAM50B	-1.36228213	4.3699E-05
RP11-138A9.2	0.89913521	4.4846E-09	MAFB	-1.36221982	5.4737E-16
SNHG21	0.89859114	0.01313823	TTC7B	-1.36186087	0.00204383
RPL18A	0.89850516	1.582E-36	TSPAN4	-1.36032554	2.4355E-23
PHLDB3	0.89711627	8.6538E-06	ZNF623	-1.35866997	0.0205841
ERCC8	0.89682222	0.0169299	MFHAS1	-1.35789808	6.5832E-06
GPAT3	0.89576591	6.4201E-08	GRAP	-1.35747352	0.01547717
FAM117B	0.89525586	0.00355653	NCEH1	-1.35411977	6.8013E-11

MPHOSPH6	0.89501517	1.8549E-09	PGM2L1	-1.35281377	0.00010773
OBFC1	0.89354699	0.00021604	PTPRO	-1.35267843	0.00612701
RP11-332H14.	0.8923962	0.04872385	PDGFB	-1.35254191	7.3316E-13
RALA	0.8859757	0.00090114	SPSB2	-1.35167254	0.00212516
SYAP1	0.88501136	5.4737E-16	ABCA1	-1.34886264	1.1047E-16
LILRB3	0.88496516	6.6568E-13	PPP1R3G	-1.3474506	0.00195342
LINC01315	0.88307084	0.04226642	DPY19L3	-1.34510071	0.00047762
LRRK2	0.88147334	0.00068139	SWT1	-1.34293469	0.00743858
DDIT4	0.87993264	3.243E-08	CTSB	-1.34133014	2.3709E-20
ZFAND1	0.87885678	2.1006E-08	GALM	-1.33308906	1.413E-07
ZCCHC3	0.8780687	0.00025009	GM2A	-1.33252438	4.1457E-25
SH2B2	0.87739204	0.0008455	PTGFRN	-1.33221566	3.6013E-06
SERPINF2	0.87506226	0.00186292	HSPA1A	-1.33071738	0.01161268
JARID2	0.87439613	4.7977E-08	RP5-899E9.1	-1.33024552	0.00479562
ATP2B4	0.87419589	0.00258837	STARD13	-1.326169	8.7373E-05
BBX	0.873557	6.6052E-07	SLC29A3	-1.3253794	6.6719E-06
CDK14	0.87319451	0.04547386	TNF	-1.32536261	0.00387797
AKAP13	0.87294423	8.443E-10	CRACR2B	-1.32514852	0.00024006
LINC00936	0.87148819	0.00024164	ACOT11	-1.3242275	0.00108404
FBP1	0.87129951	1.6365E-08	ITGAV	-1.32250775	9.9213E-14
PCCA	0.87111413	0.01977591	THRA	-1.32060876	0.00088191
S100A10	0.86983394	1.2398E-10	NEU1	-1.31812307	2.0563E-20
MTHFD2L	0.86837169	6.7878E-07	BCL2L1	-1.31149913	3.8977E-06
CD7	0.86787207	0.03201407	C17orf80	-1.3075478	0.01866311
ZNF467	0.86760319	0.00025625	SFMBT1	-1.30744831	0.00087467
AFMID	0.86741973	3.2213E-07	BBS4	-1.30730672	3.1392E-05
MSI2	0.86711698	0.00157377	NBPF1	-1.30549585	0.0002017
HOXA5	0.86683682	0.01860814	TRIM14	-1.30468845	2.9993E-14
PFKP	0.86629637	0.03528959	NEK6	-1.30392652	6.6985E-09
RP11-670E13.	0.86223474	0.01206075	NT5DC2	-1.30387	9.4689E-08
MTHFS	0.8616677	4.1553E-05	OSBPL1A	-1.30242632	5.0273E-17
KIAA0040	0.85871871	0.00499072	SERPING1	-1.29330154	5.8967E-07
EIF3E	0.85847708	3.2931E-40	AC109826.1	-1.29244858	2.5461E-06
JHDM1D-AS1	0.85618682	0.00850312	ADAM9	-1.2917215	1.8363E-17
CKLF	0.85581484	1.6327E-10	SERHL2	-1.29171323	0.00125674
PIM2	0.85439766	2.9043E-06	PDE1B	-1.29093693	0.00296242
JAZF1	0.85034552	0.00028055	GPR137B	-1.28651058	3.2527E-14
KYNU	0.84987515	6.7352E-06	ZNF438	-1.28049399	0.00185012
EIF3L	0.84982634	2.3285E-28	ZNF205	-1.27990705	0.00306381
IL1RN	0.84953899	0.04116029	CDR2L	-1.27860234	0.02996948
MTMR9	0.84855562	1.6339E-05	GCLM	-1.27560175	9.3545E-07
IFT20	0.84784482	1.7414E-13	C1orf109	-1.27424228	0.00476593
RPL5	0.84673543	9.0372E-32	CTSA	-1.27391407	1.6031E-29
GLIPR2	0.84634574	4.7927E-11	F13A1	-1.27268335	0.0067931
PGS1	0.84398446	0.00118684	CD68	-1.26911166	5.7276E-22
RPL6	0.84297862	2.8121E-51	ENG	-1.26892357	1.5453E-17

SIPA1L1	0.84293555	0.00300249	NTAN1	-1.26514677	6.5479E-25
RUNX1	0.84044295	1.4712E-06	MKNK1	-1.26504747	2.6757E-16
GBA2	0.83725838	0.00443854	PLEKHG3	-1.26450159	0.04131371
SSBP2	0.83701207	0.01651393	ID3	-1.26364266	5.7007E-05
RARA	0.83575975	9.3357E-05	RCN3	-1.26333509	0.00063482
SAMSN1	0.83505021	2.8251E-14	RP11-54O7.17	-1.26200808	1.3244E-06
FOSL1	0.83454808	0.00212968	TRPM2	-1.2609964	4.6556E-08
MEGF9	0.83391889	0.00039485	ARMCX2	-1.25910133	0.00085431
GABBR1	0.83376101	0.01294857	ZNF823	-1.25818399	0.01325164
PLCD1	0.83206419	0.01698397	PWP2	-1.25667475	0.00531254
EMP3	0.83047352	5.4321E-17	PPM1N	-1.25635588	7.3616E-07
AGTPBP1	0.82880543	7.231E-05	TMEM53	-1.25610137	2.0863E-06
ZEB2-AS1	0.82758283	0.00475523	MORN2	-1.25366516	1.3409E-06
C15orf48	0.8266182	0.00072801	AXL	-1.25208867	1.6901E-17
CTC-428H11.2	0.82635393	0.03095614	GIN1	-1.25137848	0.00789894
TRIM22	0.8251657	3.6026E-16	CDKN1C	-1.24988346	0.00056958
PRKAG2	0.82417631	2.6953E-09	IFIT3	-1.24863045	0.00024486
MEX3C	0.82117702	0.00116621	ZNF366	-1.24521952	0.00632832
CTDP1	0.81979456	8.2497E-05	ZFYVE26	-1.24507149	0.00093957
TNFRSF10B	0.81825546	3.0711E-10	F8	-1.24054908	4.7047E-05
MYCBP	0.81786806	1.3006E-05	HSPB1	-1.2377102	2.29E-08
FAM215B	0.81777676	0.00214331	RHBDD3	-1.23522557	3.8103E-05
BATF3	0.81709486	1.723E-10	MPP1	-1.23406053	7.7758E-22
RP11-658F2.8	0.81672605	0.02971205	KIAA0226L	-1.23382297	3.1424E-05
RP11-356I2.4	0.81474288	0.00010372	TTYH3	-1.22977898	2.1241E-16
LILRA1	0.81465296	0.02206778	IL32	-1.22970477	0.00609634
ALDH2	0.81379393	3.1548E-07	MSR1	-1.22969351	2.84E-07
MBOAT7	0.8137545	4.6338E-09	KIF1B	-1.21679521	3.5167E-24
EZR	0.81288817	1.395E-10	RGS1	-1.20970013	5.7943E-22
FCHO1	0.80854581	0.02795037	TBC1D2	-1.20853912	0.00243886
OXSR1	0.80310601	3.2911E-05	LPAR1	-1.20595381	0.02946338
RP6-91H8.3	0.80305062	0.00049165	DTX2	-1.20577371	7.203E-08
LTB4R	0.80234801	0.00118684	EBI3	-1.20455451	1.2483E-09
SAMHD1	0.80122089	3.7949E-18	ANKMY2	-1.20409109	0.00086512
TESC	0.79980222	0.00279194	TBC1D4	-1.19869491	4.6185E-06
VIM-AS1	0.79885488	0.00059073	EEPD1	-1.1951719	5.675E-05
VIM	0.79636027	5.4604E-10	MGLL	-1.1941069	3.1014E-07
RPL31	0.79487713	5.6895E-26	SDSL	-1.19155097	6.8247E-12
NUDT8	0.79109737	0.04294742	KLF2	-1.18940777	1.3769E-05
NEDD9	0.79065621	3.3721E-05	CCDC102B	-1.18690381	0.00129111
SMIM14	0.79029544	2.6012E-09	LXN	-1.18633062	0.00084139
TRAF4	0.79029402	0.0091572	FRS2	-1.18495411	6.2879E-05
SMCHD1	0.78996272	5.2838E-10	AMY2B	-1.18387632	0.04644061
RPL32	0.78970892	2.6665E-31	OSR2	-1.18016665	0.03484966
ZNF846	0.78960922	0.00011051	ZNF280C	-1.17972388	0.04184541
BST1	0.78956177	0.00317438	SMAD1	-1.17680781	0.00755765

TFDP1	0.78942129	5.5913E-07	CYFIP1	-1.17553263	2.4751E-17
CASP10	0.78773552	0.00218754	COL8A2	-1.17474935	9.3907E-06
RPS8	0.78760894	3.7159E-30	RCAN1	-1.17391919	9.0926E-12
NSUN6	0.78694996	0.00060757	PLIN2	-1.17372835	7.4524E-27
ZC3HAV1	0.78667979	3.8314E-12	GIMAP1	-1.17354359	0.00012242
RP5-906C1.1	0.78665519	0.01102807	CD200R1	-1.17243033	0.01509939
RPS7	0.78584486	1.2514E-34	H1F0	-1.1719375	1.7131E-09
RP11-156K23.	0.78546447	0.04875857	SCARB1	-1.1705213	1.7054E-07
CCDC57	0.78261627	7.0225E-07	MATK	-1.1677408	0.02043208
FANCA	0.78077124	0.01566583	MT1F	-1.16753112	0.0003055
PNP	0.77970907	0.00138484	ADM	-1.16707415	1.0057E-06
EIF3F	0.77955131	2.008E-28	ARHGAP12	-1.16611252	0.00011385
DUSP22	0.77547634	3.8949E-07	PMP22	-1.15919099	2.8191E-18
CHML	0.77531907	2.7865E-07	ZNF184	-1.15907394	0.00111605
LST1	0.77409444	1.5379E-20	UNC13B	-1.15518009	0.00526061
UVRAG	0.77408404	9.8268E-08	MYO5A	-1.1531343	2.5906E-14
FAM43A	0.77236221	0.00338303	ZNF844	-1.15029095	0.00035117
CYTIP	0.77198518	1.1187E-15	METTL18	-1.15019577	0.01336548
RPS23	0.7703537	8.7328E-35	FOXRED2	-1.14955966	1.0257E-05
GPR171	0.76990433	0.00534371	KRBA1	-1.14926269	0.04054495
MCOLN2	0.76917834	0.02210372	TNFSF15	-1.14874618	0.00051022
PSMB9	0.76864251	3.1394E-10	TMEM144	-1.14407139	7.035E-09
RARA-AS1	0.7683617	0.00651476	CHST15	-1.14213136	5.8034E-08
NDRG2	0.76826576	3.4201E-06	TIMP2	-1.1410481	5.6248E-32
PHLPP1	0.76786551	0.0062943	NCKAP5	-1.14063315	0.00647823
RPS24	0.76774992	1.0405E-31	AMDHD2	-1.13593843	1.3228E-11
NOTCH2	0.76769109	0.00019744	PIK3IP1	-1.13525601	2.449E-09
DENND5A	0.7667077	0.00078008	GPR155	-1.13222295	5.0836E-05
GABPB1	0.76653086	9.31E-09	PSAP	-1.13173245	1.6446E-17
ATP2B1	0.76646582	7.4646E-07	NRP2	-1.13122963	2.889E-06
ACAA1	0.76362493	2.7643E-08	RHOBTB1	-1.1306112	5.4915E-05
PDE7A	0.7628576	3.3936E-05	LINC00346	-1.12891479	0.02940817
RPL30	0.76144331	9.2465E-39	LAMP2	-1.12772893	2.6281E-30
NELFCD	0.76125089	5.5046E-05	SFXN2	-1.12738947	0.04599442
FAM49A	0.76008834	7.9528E-06	CDKL3	-1.12639887	0.04879298
RPL34	0.7595802	9.4102E-28	PTGR1	-1.12512088	2.6912E-05
RPL10A	0.75924677	1.1097E-35	ZNF398	-1.12424108	0.03565289
CD48	0.75865577	2.9608E-10	CCR10	-1.12331848	0.04937792
RPS4X	0.75735472	4.6351E-22	LILRA6	-1.12308339	0.00204157
PTRHD1	0.75675699	2.4251E-14	TMEM14A	-1.12255204	0.00011555
RAC2	0.75668134	7.2213E-10	DDIT3	-1.1176319	8.1474E-09
CD44	0.7554042	1.274E-10	CITED2	-1.11459412	2.004E-14
RUFY3	0.7553198	0.00023223	GFRA2	-1.11417292	6.24E-07
CEP68	0.7550132	5.3134E-09	AC240274.1	-1.11334169	0.00149867
KIF22	0.75484925	0.00044742	AP1B1	-1.11161552	2.3624E-18
DLGAP3	0.75304595	0.03464299	DBP	-1.11151032	1.3077E-06

RPL4	0.7504136	2.2601E-25	FTL	-1.11088251	1.0744E-23
BRAF	0.74964072	0.01046273	UACA	-1.10751759	0.00027011
HS6ST1	0.74955703	0.04918384	TCF12	-1.10411939	3.7385E-08
SNX10	0.74855072	1.2914E-05	MYO1C	-1.10399783	4.8856E-05
SPRED2	0.74769862	0.03648983	RP11-4B16.4	-1.10384033	0.02028103
SERPINB9	0.7469551	4.429E-06	ZBTB4	-1.10279366	2.6953E-09
HIPK2	0.74556501	2.2747E-06	C16orf70	-1.10058666	0.00072453
RPL11	0.74542898	1.3725E-27	PLEKHO2	-1.09990679	1.4515E-10
ZBTB18	0.74414088	0.04654637	VMO1	-1.09829572	4.456E-08
KCTD20	0.74375248	7.3287E-07	ABI3	-1.09818048	9.0372E-32
CYP2S1	0.74332403	1.0047E-06	CSRP2BP	-1.09673169	0.0472675
NIPSNAP3A	0.74226117	9.1862E-07	FAM20C	-1.09333296	1.0936E-09
SH3BGRL3	0.74068025	3.8049E-08	TCEAL3	-1.09319939	2.4655E-09
STX10	0.73999628	3.1893E-06	FDX1L	-1.09183294	1.6245E-07
PIP4K2A	0.73994627	3.1518E-08	IFI27	-1.09087202	0.00161309
RPS18	0.73767861	2.2472E-17	YWHAH	-1.08951014	2.6807E-16
ACSL5	0.7374532	9.6718E-05	FBXO31	-1.0892758	0.02984258
FLNA	0.73717842	0.00398692	YAE1D1	-1.08783625	0.02065337
RPS27	0.73649277	1.1065E-28	ZFP90	-1.08755618	0.00613155
FES	0.73632206	4.2023E-05	RGS12	-1.08672722	1.4608E-09
SEPT1	0.73527054	0.01848685	UHRF1BP1	-1.08518417	0.00022761
AEN	0.73417462	0.00012229	FPR3	-1.08065431	2.1149E-20
GAS6	0.73353725	0.00025652	CAMSAP2	-1.08060594	0.00191166
ZFC3H1	0.73086103	2.3754E-06	COLGALT1	-1.07975468	1.3655E-18
RPL14	0.73056541	4.2137E-32	L3HYPDH	-1.07782967	0.00597545
RPS14	0.72996603	1.0581E-17	ACVRL1	-1.07622578	1.274E-10
TLR2	0.72944979	8.3615E-11	ZNF468	-1.07613081	0.00575399
HIVEP1	0.72918156	0.03203058	TEFM	-1.07469002	0.00023081
SPN	0.72854904	0.00049419	SYNJ1	-1.07209488	0.00018021
RPS15A	0.72729378	2.1262E-34	GPD2	-1.07189706	0.00030447
MNAT1	0.72704935	0.03033487	ZNF776	-1.07173158	0.04875857
NFE2L3	0.72679961	8.5368E-06	ALDH7A1	-1.07094227	0.00021104
RASA4	0.72643009	0.01132737	CD80	-1.07036073	0.0003592
RPL26	0.72569729	1.3725E-27	HIST1H2BG	-1.06960136	0.03618426
TNFAIP8	0.72378621	0.00138057	ZMYM1	-1.06732965	0.00411396
ARF5	0.72321137	5.3518E-07	ORC5	-1.06667602	0.01582522
PWWP2B	0.72274567	0.0027842	AHRR	-1.06573696	0.00612643
RPS3A	0.72239797	2.5556E-30	SELM	-1.0646448	5.7585E-12
RPL35A	0.72233188	3.7208E-20	TIMM21	-1.06429417	0.00014708
ATAD2B	0.72177578	0.00035441	ABHD12	-1.06317225	1.0806E-26
PRC1	0.72166599	0.04956619	C3AR1	-1.06237501	8.2937E-13
BRCA2	0.72103099	0.00576279	MCOLN1	-1.06206106	5.1672E-07
SERPINB8	0.7205517	2.6483E-06	LAMP1	-1.06152256	6.7628E-06
DDX39B	0.7204504	4.8198E-05	CCDC186	-1.06048234	0.00025321
DDX27	0.71901017	1.5706E-11	RAB39A	-1.06033923	0.00011651
RPL36A	0.7187946	9.1369E-21	C2CD3	-1.0584752	0.00354882

LRG1	0.71794527	0.02421376	ARID3B	-1.05691624	0.00030747
GYPC	0.71597358	0.00011651	ABHD11	-1.05638944	1.5117E-06
TTC19	0.71375663	2.9927E-08	MOSPD2	-1.05607291	6.1432E-09
RPL37	0.7136472	3.2879E-25	NCK1-AS1	-1.05487134	0.00049633
PPP1CB	0.71354179	5.8818E-15	MS4A7	-1.05017211	4.5843E-11
GNB2L1	0.71198346	9.3668E-21	WDR81	-1.05006047	8.4478E-05
GNA15	0.71151001	1.6076E-06	CDR2	-1.04977301	0.0014733
PILRA	0.71145304	7.9423E-10	FAM114A1	-1.04970584	0.00241545
COMMD6	0.71139101	1.9825E-18	GAS2L3	-1.0476173	1.8773E-05
BTF3	0.71104661	9.3345E-41	ZNF891	-1.04608582	0.01192149
PLAUR	0.70591825	3.1068E-08	ZZEF1	-1.04323641	1.3049E-07
RPS12	0.70587498	7.1373E-24	SLC35F6	-1.04308147	3.4976E-08
SRGAP2	0.70469979	3.4907E-06	KLF6	-1.04044756	3.6436E-07
SFT2D3	0.70340231	0.00372897	SWAP70	-1.04030485	3.6194E-16
NUAK2	0.7030392	0.00362052	RP11-644F5.1	-1.03888594	0.02276978
USP3	0.70283174	7.6871E-10	BBC3	-1.03872062	0.00183966
RP11-182L21.(	0.70256275	0.02507239	B3GNT7	-1.03685114	5.659E-05
TOB1	0.70211832	9.4439E-06	EXTL2	-1.03555167	0.0109479
C15orf61	0.70207995	0.00201652	MANEA	-1.03526661	0.00082881
UBXN2A	0.70191041	0.00156966	FOS	-1.03517949	5.0276E-06
CCND3	0.7014992	1.313E-06	YEATS2	-1.0343578	0.00274117
RPL10	0.70142353	1.1062E-27	DAPK1	-1.032758	6.0388E-13
EFCAB2	0.7004181	0.03846091	AMPD3	-1.03273466	3.1514E-10
BTBD2	0.70027704	0.00832521	IFFO1	-1.03228766	0.00034773
RPL3	0.6989902	2.3473E-28	TTC33	-1.03136191	0.00566988
CNPY3	0.69887009	1.2764E-18	MGAT4A	-1.03111376	3.274E-14
MIR22HG	0.6986423	1.1755E-05	POLR3A	-1.02780753	0.00857754
RPS13	0.69856159	1.7757E-41	INTS7	-1.02692728	0.04978142
APP	0.69820924	0.02057818	TNFRSF12A	-1.02644922	0.00357962
WDR54	0.69757374	0.00147184	PIP4K2C	-1.02637108	0.01268443
CCDC109B	0.69754003	4.7565E-06	MFSD7	-1.02311558	1.6974E-06
NCK2	0.69723516	0.03965795	AMN1	-1.02215764	0.0124576
GLTSCR2	0.69581027	4.8359E-11	MAP1LC3A	-1.0220938	0.00088082
RPS5	0.69514968	5.5536E-24	IL10	-1.02194128	0.00048237
B4GALT5	0.69474528	0.00116709	ACO1	-1.02153531	1.8382E-05
ANKRD44	0.69399575	5.3774E-05	CAPRIN2	-1.02083452	0.00590271
NUTM2A-AS1	0.69399544	0.00233294	TANC2	-1.0200345	0.00078936
EIF4G3	0.69221737	3.0472E-06	CACNB4	-1.02002598	0.01491308
NUTM2B-AS1	0.69170082	6.8617E-05	CEBPA	-1.01990367	9.2026E-06
ACSL1	0.69131523	2.1385E-07	FLYWCH1	-1.01968163	0.00207269
RHOC	0.69009151	7.8153E-05	ZFP36L1	-1.01863377	2.1153E-09
BID	0.68702684	3.825E-06	ARSK	-1.01851683	0.02895241
RPL19	0.68691971	3.3836E-23	ARHGAP35	-1.01642793	7.1823E-05
OFD1	0.68667748	1.0029E-07	DGKH	-1.01636497	0.00053925
RPL29	0.68609783	3.8734E-20	FKBP4	-1.0153187	1.2489E-06
LINC01003	0.68556427	0.00244082	TJP2	-1.01516236	2.174E-06

IRF9	0.68424955	0.00013386	CD84	-1.01492996	7.8875E-08
RPS29	0.68342557	4.5577E-17	KEAP1	-1.01364165	4.908E-05
RPS9	0.68301268	2.7425E-20	POU2F1	-1.01307252	1.9962E-06
ATP1B3	0.68048614	2.2371E-12	NEU3	-1.01297751	0.00151003
RPL37A	0.67924941	3.3072E-23	SLC48A1	-1.01155755	0.00016111
RPS2	0.67889008	2.3053E-16	TMEM150B	-1.01103851	0.03047108
ZNF557	0.67878017	0.00869204	SLC31A1	-1.01013012	7.2213E-10
ITPR1	0.67863447	0.00075333	CNPY4	-1.00995497	0.00825666
PDE4A	0.67714632	1.6686E-05	ANXA2R	-1.00864613	0.00100151
SSH2	0.67639264	0.00033302	ZNF189	-1.00694429	0.00076008
RPS11	0.67603908	3.3377E-19	KNOP1	-1.00664202	5.5672E-05
SLC7A5	0.67564666	0.00041649	AC074289.1	-1.00621617	0.00080198
GABARAPL1	0.67541243	6.8081E-13	RGL1	-1.00606277	6.2457E-09
RPL8	0.67538814	5.5224E-23	MARCKS	-1.00454263	3.4456E-14
TIMP1	0.6752346	0.00833484	SGTB	-1.00374399	6.6062E-06
PSMB8-AS1	0.67442892	2.14E-05	ARHGAP18	-1.00239522	1.7234E-15
RASSF5	0.67403847	1.0829E-06	STAG3	-1.00179993	0.00293809
KIAA0513	0.67355434	0.00143232	WDR36	-0.99708635	0.00013157
HCG18	0.67349228	0.00897779	CUL9	-0.99658464	0.04670517
RPL17	0.67345158	1.8506E-06	ASUN	-0.99613971	0.00086398
HIP1	0.67220041	0.01205406	TIFAB	-0.99548558	1.1889E-05
OLA1	0.67172002	4.9375E-11	PLXNA3	-0.99478038	0.00411396
CDKN2AIPNL	0.67109675	0.00456688	TMEM263	-0.99440042	0.00020266
NACA	0.67077356	1.6458E-20	TGFBI	-0.99420922	1.2869E-17
PNISR	0.67065212	1.1207E-08	C5AR2	-0.99125221	2.2834E-06
PGLS	0.67024769	3.0475E-08	SRGAP3	-0.98815696	5.6509E-07
GNG7	0.67017992	0.00293465	MAGEH1	-0.98807324	0.00039901
PTPN1	0.66991369	4.2803E-06	ERRFI1	-0.98606287	0.01359837
RPL23A	0.66986073	4.7233E-24	ALAS1	-0.98600032	4.0516E-05
ARGLU1	0.66912516	3.9644E-13	ATP13A2	-0.98296431	0.02278498
CHRAC1	0.66580519	2.5435E-06	CLIP2	-0.98085212	1.4757E-06
RP11-404O13.	0.66577704	0.04074822	ABCB1	-0.98079932	0.03735848
CCNH	0.66576904	6.4913E-08	CD63	-0.97399347	7.9167E-31
ADA	0.66516988	0.00179942	STOML1	-0.97391767	0.02874076
AC058791.1	0.66494695	0.0007102	FXYD6	-0.97358066	0.01277808
FAM122B	0.66409931	0.01538658	SLC2A13	-0.97272758	8.4446E-05
GPBP1	0.6639198	2.0252E-09	LINC01451	-0.97209725	0.00309379
IMPA2	0.66384382	2.467E-05	TMEM251	-0.97045662	2.5361E-09
LMNB2	0.66383811	0.03490176	ACOT7	-0.96952186	0.00285135
C8orf59	0.66361954	5.0434E-13	CBLB	-0.9679019	0.00911015
NCF2	0.66341221	3.5506E-12	CFD	-0.96758428	0.00010587
IFT57	0.66338552	0.00019016	MICB	-0.96724475	0.02727912
CLP1	0.6628679	0.03060027	ZNF444	-0.96651612	0.01899689
RPL41	0.66281718	1.0936E-29	AC093627.10	-0.96541319	0.01009014
DDX21	0.66257363	1.4995E-15	DAGLA	-0.96277257	0.03519007
EEF1A1	0.66200117	5.3496E-39	ABL2	-0.96270898	2.1503E-09

MKL1	0.66023273	0.00682404	CPNE2	-0.96209485	0.00261029
SQLE	0.66010284	0.00087593	VEGFB	-0.9619009	2.1702E-09
ASL	0.6596564	0.00297912	METTL13	-0.96092028	0.00038024
TMEM164	0.65885182	0.0320493	PLOD1	-0.9606737	2.2711E-06
YDJC	0.65734143	0.00070684	ATF3	-0.96067153	3.8147E-07
DHFR	0.65681206	0.04709415	NAGLU	-0.96040137	2.9435E-06
RPS15	0.6562619	9.4351E-27	ITPR2	-0.95988312	8.9645E-12
CCDC69	0.6553659	0.00086339	LPAR6	-0.95900555	3.6532E-20
UBXN11	0.65394509	0.00153148	ZNF440	-0.95849566	0.04174285
SMARCE1	0.65315977	2.6012E-09	CBR1	-0.95836548	4.1437E-09
ARHGEF1	0.65295343	3.9499E-05	NUDT12	-0.95814486	0.01892512
DHPS	0.65261077	1.1472E-05	LINC01503	-0.95761459	0.00831466
RPL7A	0.65142938	3.608E-22	SIPA1L2	-0.95718241	0.00017241
RPL18	0.65126009	1.5896E-24	NFE2L1	-0.95687946	2.1377E-07
DNAJC4	0.65009768	1.9463E-06	RNF185	-0.95659185	0.00655186
PABPC1	0.64957475	1.8281E-24	TMEM87B	-0.95648591	2.4666E-07
RSL24D1	0.64912766	1.0173E-22	TMEM99	-0.95587355	0.01615757
KLF3	0.64878573	0.00069814	LTC4S	-0.95543207	0.00365718
R3HDM4	0.6483398	0.00030384	ZC3HC1	-0.95409268	0.02494752
LYAR	0.64797086	1.8506E-06	ARHGAP10	-0.95349796	3.5458E-07
RPS16	0.64746094	9.5956E-22	PTGS2	-0.95340033	1.0402E-09
RPL13	0.64732194	1.2183E-15	TSC22D1	-0.95339309	9.3545E-07
ZNF33B	0.64674721	0.03345374	HECTD3	-0.9530256	0.00872284
EIF3M	0.64617347	4.6736E-21	KCTD5	-0.95154784	2.494E-07
EIF3H	0.64571421	4.9115E-25	ZNF649	-0.94732193	0.02116684
RP11-76E17.3	0.64535024	0.00982889	CCR1	-0.94706213	2.2281E-11
RPSA	0.64483567	2.0142E-29	POMT1	-0.94705431	0.0109479
CXCR4	0.64436691	8.4446E-05	RB1	-0.94613512	4.2376E-17
PLSCR1	0.64383134	5.7996E-08	TFRC	-0.94588928	3.8648E-05
JOSD1	0.64326949	2.7402E-07	TP53I11	-0.94200393	0.02895241
SUPT4H1	0.6419919	1.6652E-14	PXN	-0.93982766	0.00034385
RNF24	0.6418509	0.03038431	PDLIM7	-0.9397117	0.0004114
GPSM3	0.64184679	8.202E-18	FLCN	-0.93927777	1.7791E-06
JAML	0.64178831	2.4468E-08	ALKBH4	-0.93513207	0.04670517
PAK1	0.64152685	7.1613E-09	GADD45A	-0.93423224	3.2128E-08
FADS3	0.64144076	0.00769525	TMEM135	-0.93135796	0.00624773
SLC25A51	0.64126597	0.02276978	IFT74	-0.93122268	0.00137377
CHD1	0.64091586	3.0778E-13	RHOB	-0.9311811	0.01758588
ECHDC1	0.64069886	6.5147E-06	ODF2L	-0.92911608	0.00021838
AKNA	0.64015414	1.9031E-08	AC011899.9	-0.92787731	0.00832521
TIMM23B	0.63936397	0.00670986	ITGB7	-0.92726456	2.379E-27
APAF1	0.63910625	4.7622E-06	SLC9A9	-0.92701013	1.3352E-07
RPL15	0.6367048	6.9468E-26	ELAVL4	-0.92595902	8.7569E-05
ENDOV	0.63505865	0.00209866	MFSD5	-0.92570382	0.004625
LRRC75A	0.6340763	0.00263012	PECR	-0.92338851	0.01995728
DCPS	0.63387449	0.00325727	DBT	-0.92332336	0.03047108

SH3KBP1	0.63368283	2.9926E-05	RASA3	-0.92295818	0.00012155
HMBOX1	0.63227061	0.00793597	PLPP1	-0.92204126	0.00448304
ARHGAP26	0.63162244	4.851E-06	NMNAT3	-0.92077861	0.03311292
C10orf128	0.62997937	0.00022451	WDFY3	-0.92042708	7.1624E-07
RPL24	0.62965429	6.9124E-20	SPR	-0.9197734	0.02483745
RPS21	0.62959711	5.305E-16	RP13-638C3.4	-0.91711953	0.0472752
RPS3	0.629502	2.8711E-24	TGDS	-0.91404524	0.01093766
SLC25A6	0.62938094	8.168E-27	CCL5	-0.91293647	0.0390771
CDC42SE2	0.62935873	1.3615E-11	C1orf54	-0.91232751	1.136E-15
RPS19	0.62808285	1.9965E-12	ZNF768	-0.91220282	0.0043465
SP110	0.62659593	0.00323005	RP11-334C17.	-0.91210167	0.01015881
C17orf107	0.62646248	0.04123872	PDGFC	-0.91137494	2.8087E-05
IL13RA1	0.62643718	9.6295E-10	CYP4V2	-0.91133934	0.00464664
CD58	0.62535636	0.00020015	AP006222.2	-0.91111506	0.00129355
LRR1	0.62489325	0.00699614	ATOX1	-0.90896737	1.5439E-16
DENND4A	0.6248647	0.00787976	FAM188A	-0.90794477	0.00867373
DHRS4L2	0.6247398	2.8782E-07	TMEM140	-0.90583254	0.00484909
RPL7	0.62403918	7.6129E-35	HS1BP3	-0.90581382	0.00141459
RPS6	0.62346391	2.993E-31	PDIA4	-0.90525458	2.3716E-15
TGIF2	0.62294654	0.0007043	RAB7B	-0.90507339	1.8326E-05
MCCC2	0.62272428	0.0251238	SCAMP1-AS1	-0.90463868	0.01526266
ZNF200	0.62199855	0.03697077	TACO1	-0.90087177	0.00212516
HPS5	0.62198756	6.9449E-07	MOSPD1	-0.90022477	0.00093384
LONRF1	0.62141303	0.04315662	AKAP11	-0.89983595	1.1232E-05
STRAP	0.6212633	9.0323E-09	BLVRA	-0.89975887	3.3972E-15
C11orf1	0.62107974	0.00378961	KCNK13	-0.89845587	0.00017584
ROBO3	0.62084696	0.04113347	ASAP2	-0.89810732	0.00161703
TMX4	0.62078768	0.00018069	ICA1	-0.89518002	0.00331892
SLC9A7	0.61995909	0.00748112	PRMT7	-0.89464571	0.01844639
SCLT1	0.61983682	0.00043593	IQCE	-0.89421377	0.00823389
DEF6	0.61974885	0.00107157	MEN1	-0.8933505	0.03311292
RNF139	0.61927331	2.9649E-07	RDX	-0.8932624	1.4779E-07
RSL1D1	0.61871113	3.7661E-15	FAM13A	-0.89303761	0.00048654
EDEM1	0.61856689	8.0718E-06	ARHGAP17	-0.8911069	0.000147
AHNAK	0.61854231	5.4953E-08	EXD2	-0.89015474	0.02891901
RPS10	0.61788542	1.1883E-21	GIPC1	-0.88945956	1.8971E-05
CUL1	0.61771801	0.00328537	INF2	-0.88877869	0.00060437
CD33	0.61769847	4.6716E-07	C17orf96	-0.88840497	0.02276978
MOB3A	0.61700047	0.00048237	FAM198B	-0.88836849	1.4299E-07
CPNE1	0.61687964	4.0757E-07	RELL2	-0.88826807	0.01325164
USP12	0.61660779	0.00573391	DTWD2	-0.88812529	0.00514424
SIGIRR	0.61645907	9.6958E-06	TMEM106B	-0.88516778	0.00017906
ABHD14B	0.61630884	2.7327E-05	NCF4	-0.88502411	1.1834E-13
RPS17	0.61603473	3.6813E-28	ANKRD9	-0.88420694	6.1716E-06
CBX5	0.61592486	0.00011982	MEGF8	-0.88409771	0.03816775
ASCC1	0.61590153	0.0058886	C12orf4	-0.88366002	8.8263E-05

RPL39	0.61568217	3.0053E-27	SNX18	-0.88226616	7.11E-07
FKBP5	0.61550054	1.9414E-15	TNFRSF21	-0.88116324	2.9344E-13
SETD9	0.61496308	0.00117373	CEP162	-0.88088981	0.03118478
HRH2	0.61452161	0.01780571	UBXN8	-0.8799444	0.00300191
FAU	0.61400673	4.4528E-26	SH3PXD2B	-0.87875117	6.6463E-06
RPL21	0.61349601	1.8274E-33	CALU	-0.87762115	1.1676E-08
SLC36A4	0.6133088	0.0012145	TCF4	-0.87720046	8.8669E-07
CD86	0.61288241	3.2439E-06	UTP14C	-0.87646485	0.03376335
MAN2A2	0.61231366	0.00045193	PLA2G4A	-0.87482952	0.00769195
SEPT6	0.61190226	2.2972E-06	PTMS	-0.87440499	1.8329E-14
PTPRE	0.61162354	5.6466E-21	PPFIBP2	-0.87284115	0.0003536
TAF15	0.61121033	6.4757E-07	ATG4C	-0.87240792	2.0453E-07
TARBP1	0.61114962	0.00415554	MRPL46	-0.87164487	1.6022E-05
KCNAB2	0.61082091	0.00052889	SLA	-0.87072261	6.8052E-07
FBL	0.61075769	9.0076E-09	ABCG1	-0.87048362	8.9019E-07
MED13L	0.61063725	7.2213E-10	CSTF1	-0.86948668	0.00162918
EIF2D	0.61029823	0.00239978	CD36	-0.86940517	4.9796E-12
MGRN1	0.60897659	0.00236894	SLC9A3R2	-0.86858928	0.04072707
HDAC1	0.60875003	2.1527E-06	PLXNC1	-0.86816275	3.6952E-12
TMEM120A	0.60809477	0.00022451	NLK	-0.86647424	0.02064349
IFFO2	0.60747612	0.0054828	SLC16A5	-0.86596398	0.01128124
RP11-468E2.4	0.60690439	0.0356274	CD151	-0.86569067	4.9483E-06
SRSF8	0.60459683	1.0275E-05	ZNF227	-0.86548817	0.03365001
ERLIN1	0.60397933	0.03949802	RBP7	-0.86537319	0.00988824
GTPBP4	0.60361695	2.9963E-08	STMN1	-0.86462522	1.5565E-10
MTPN	0.60325077	1.667E-09	ATP6V1C1	-0.86435136	1.8427E-15
ZNF721	0.60306526	0.00552232	COMMD4	-0.86299386	9.0076E-09
FXYD5	0.60263037	1.5104E-17	SLC7A7	-0.86256317	7.6925E-06
AP1G2	0.60213966	0.02994335	ARL15	-0.86221327	0.00687502
CSF2RA	0.60023201	2.1488E-07	REXO2	-0.86036368	2.2791E-10
TBC1D7	0.60010542	0.00757461	TNFRSF1A	-0.85816177	1.2426E-06
NLRP1	0.59915186	0.03089745	FAM65A	-0.85699404	0.00016605
RPLP0	0.59870553	1.8177E-25	CCNG2	-0.85674495	3.0981E-06
RPL22	0.59844119	3.4914E-27	PDE4DIP	-0.8565672	5.1148E-06
ISOC1	0.59743044	0.01420595	TDP2	-0.85513356	5.8305E-05
CUX1	0.59726637	1.2051E-07	PGAP3	-0.85505828	0.03376335
USP15	0.59665905	1.4124E-10	PEX1	-0.85456058	0.00454871
RPL36	0.59601252	1.3425E-20	CARD6	-0.85183372	0.03840922
JMJD1C	0.59471077	3.8135E-08	PLXND1	-0.8517827	0.00070671
CFLAR	0.59466811	0.00019385	NR1D1	-0.84899072	0.01279461
RPL28	0.59421286	2.0884E-26	PHLDA1	-0.84813145	3.2798E-06
MCCC1	0.59406702	0.0065684	TMEM60	-0.84593213	1.6449E-05
GMFG	0.59368012	9.2311E-09	PSEN2	-0.84470262	0.00580216
PTPN2	0.5930923	8.5203E-09	TXNIP	-0.84408967	0.0219027
SOX4	0.59272788	0.00621789	SLC37A2	-0.84232626	0.00179942
IL15	0.59241311	0.01285553	SAMD4A	-0.83931825	0.00345985

TNIP2	0.59238001	0.00028391	GLUL	-0.83877005	4.9031E-09
EIF3K	0.59200113	3.1892E-21	CH17-340M24	-0.83773057	7.8242E-07
SLC12A6	0.59124481	0.00997154	PLXNA1	-0.83693145	0.00565496
REC8	0.59074204	0.01966349	MAST3	-0.83567031	0.01182854
GPAT4	0.58941647	0.00012747	DNPH1	-0.83535941	3.2213E-11
FCGR1B	0.58931975	0.00763412	CAMKK1	-0.83381525	0.00411396
MOB3B	0.58881888	0.00420097	TFEC	-0.83334965	2.6652E-10
ANP32B	0.58800639	1.7114E-10	RBMS2	-0.83247192	0.00039228
TTC14	0.58794925	0.00026635	PPAT	-0.83214066	0.04860152
RBM39	0.58763521	1.357E-26	NPHP3	-0.83083797	0.00989145
RNF7	0.5873431	4.9005E-11	SULT1A1	-0.83075649	1.1808E-08
EEF1B2	0.58715043	7.728E-17	TRAFD1	-0.82912496	0.00601609
CCDC12	0.58708351	9.1854E-06	ADGRG5	-0.82896653	0.00165472
POMC	0.58706009	0.02417936	SNX11	-0.82870337	0.00018069
LDHA	0.58703017	1.2323E-07	ATP2C1	-0.82687838	3.1462E-07
CASP8	0.58694783	0.00123585	SPRYD7	-0.82665027	0.00072801
RCC2	0.58665609	1.074E-05	BMPR2	-0.82343089	1.7559E-05
NT5DC1	0.58641737	0.00829117	ATP6V0A1	-0.82280218	1.7447E-05
SPECC1	0.58639145	0.00057569	PGM3	-0.82228826	0.00328537
LSM7	0.58623394	3.9476E-13	PLEKHA1	-0.82215454	3.6967E-05
CDYL	0.58562942	0.00326368	ZDHHC4	-0.8204608	1.3047E-09
RPL9	0.5854707	5.8891E-27	JMJD4	-0.82000116	0.03304244
BZW1	0.58539652	6.5767E-15	NINJ2	-0.81885739	2.119E-05
MEF2C	0.58487633	0.00038538	BACE1	-0.81879576	0.00724927
TMSB10	0.58422928	2.4149E-11	HSPH1	-0.81873341	0.01714031
MIR181A1HG	0.58354395	2.0403E-09	C7orf43	-0.81741951	0.02529703
PSME1	0.58326217	1.5448E-09	SH3RF1	-0.81714842	0.00088082
REL	0.58301243	0.00025613	PLVAP	-0.8147939	0.0128917
PRKAR2B	0.58180392	0.01222135	PRDM1	-0.81430964	3.5725E-05
SARAF	0.58078085	1.1489E-07	ZNF10	-0.81414579	0.00873861
TPRKB	0.58045027	1.5452E-06	S100B	-0.81375008	0.00120393
RSRP1	0.58024497	2.9876E-06	TPRG1L	-0.81111671	0.00060757
LPGAT1	0.58007091	5.1204E-05	LARP4	-0.8109846	1.1132E-05
FAM76B	0.57977499	0.00519721	GAA	-0.81001749	9.0813E-12
HADHA	0.57861574	4.6982E-11	STX2	-0.80926301	0.0004215
SLC25A19	0.5785653	2.9167E-05	MICA	-0.80910393	0.0412532
RNF144B	0.57785491	0.00036875	APOL1	-0.80464487	0.02630787
RFTN1	0.57675538	0.00766149	NPDC1	-0.80316455	0.01948067
SNHG15	0.57640222	0.00043718	CYB5A	-0.80195768	2.6912E-05
FURIN	0.57555818	0.0227014	LRRC25	-0.80133327	1.67E-07
NIPSNAP1	0.57534741	0.02603873	CPEB4	-0.80100811	3.2484E-08
ACOT9	0.57532259	6.8787E-12	TRIM36	-0.80051522	0.04292905
POLR3E	0.5750167	0.00325514	RALGDS	-0.79883664	4.2823E-06
TBC1D8	0.57467531	0.01382822	CXCL3	-0.79833238	0.00162302
TKT	0.57466473	1.5097E-07	TMEM62	-0.79724003	0.01539371
WAC-AS1	0.57344637	0.00010181	PINK1	-0.796581	2.8248E-05

CPD	0.57327877	0.04621079	CEP250	-0.79557296	0.02956783
TGIF1	0.57298817	5.4477E-05	EGR3	-0.79528242	0.00740531
IGBP1	0.5729417	1.8544E-08	DOPEY2	-0.79377018	0.00202726
SHTN1	0.57233061	8.6602E-08	ZDHHC21	-0.79295738	0.02428958
CTA-384D8.34	0.57191802	0.01012187	ZNF84	-0.79235238	0.00351599
IRF7	0.57137776	5.8356E-05	HIRA	-0.79155102	0.01205285
RPL27	0.57132267	9.1804E-17	GNS	-0.79007967	5.113E-08
IL2RG	0.57123684	0.00767643	RMDN3	-0.78857946	3.2958E-06
PSMB8	0.57123408	3.3919E-07	MBTPS2	-0.7874655	0.03047108
FBXW7	0.57073886	0.00032687	IKBKE	-0.78644322	0.01541336
C20orf27	0.57024875	1.9573E-09	HCFC2	-0.78624063	0.00704922
BCL6	0.57022949	0.0318537	ARHGAP20	-0.78462927	0.00683596
PNPLA8	0.56804568	1.8067E-06	LYPLAL1	-0.78438209	0.00058448
CLEC4A	0.56694125	0.00014995	FAM134C	-0.78412255	0.0013758
HLA-DQB1	0.56651846	1.5098E-09	KHK	-0.78306823	0.00946263
RPL13A	0.5647146	7.6946E-29	SLC11A2	-0.7817688	2.9166E-05
ZNF880	0.56414042	0.02989509	PI4K2A	-0.78113885	0.00053347
MDP1	0.56409575	0.01973115	IL12RB1	-0.78042895	3.3999E-05
RPS25	0.56338477	4.8139E-26	DOCK1	-0.77913536	0.01457888
TRAPPC6A	0.56202726	1.9956E-08	LTBP3	-0.77882713	3.9902E-06
NUP107	0.56195497	0.03047108	TCF7L2	-0.77880348	8.0386E-08
HCLS1	0.56147758	9.6195E-11	SMIM15	-0.77711406	2.3466E-06
GBP4	0.56131044	0.00084619	PUS10	-0.77676259	0.04483584
SRSF6	0.56064077	7.2136E-08	CSF1R	-0.77626269	1.2299E-13
CCNDBP1	0.56061604	1.4569E-09	PLOD3	-0.77578683	1.3457E-05
NETO2	0.56060115	0.04545971	LZTS2	-0.77553126	0.03528959
FAM175A	0.5602006	0.02883306	SLC16A1-AS1	-0.7745897	0.01247858
SYF2	0.56014817	8.0794E-10	PHF23	-0.77201174	0.01664019
CLSTN1	0.56007221	0.04373193	TFPT	-0.77141256	7.5013E-13
CARD16	0.55902702	8.4512E-12	SMURF2	-0.76995762	5.3035E-07
ALYREF	0.55810342	0.00132193	KLHL9	-0.76934396	0.0006521
NFKB2	0.55760035	0.00078099	ZFAND2A	-0.76840898	0.01048752
PHYKPL	0.55682505	0.00014825	PFKM	-0.76729399	0.03897412
CHN2	0.55560533	0.00713094	TM2D2	-0.76713538	5.9603E-06
CTNNBIP1	0.55520223	0.00037981	GIMAP4	-0.76704483	0.00082211
RBCK1	0.55490116	8.1303E-07	C5orf28	-0.76699419	0.0274766
RAB4B	0.5548361	2.6386E-05	CRISPLD2	-0.76666376	0.04853499
IMPDH2	0.55479476	9.7336E-07	FAM21A	-0.76445163	5.1506E-06
PRKDC	0.55446353	6.3723E-09	ZNF761	-0.76342432	0.04340676
MRPL33	0.55439901	3.8758E-10	GSTM3	-0.76338079	0.02076395
STK17B	0.55428639	1.4546E-12	SLC46A3	-0.76329679	1.5806E-05
PQBP1	0.55347227	7.4342E-05	MPP5	-0.76256916	0.00315469
ARHGAP30	0.55333453	4.2607E-05	LY96	-0.76134753	2.6097E-24
CCNB1IP1	0.55262179	0.00378961	AP5B1	-0.76030751	0.00910111
C1orf56	0.55236319	0.00018848	CCDC106	-0.75960022	0.00231531
DOCK5	0.55218598	1.0275E-05	ALG12	-0.75803624	0.01572025

B3GNT5	0.55208189	0.00047926	PLBD2	-0.75727706	6.6637E-07
ANKRD11	0.55192914	6.8802E-06	HES4	-0.75618893	5.2326E-13
MYADM	0.55189441	1.3636E-05	IER5	-0.75521235	0.00016279
ATXN7	0.55189244	0.00235784	CTSZ	-0.75487199	1.7918E-22
CNN2	0.55184525	2.347E-07	RCBTB2	-0.75417625	1.3497E-06
SIK3	0.55153517	0.00420097	CLUAP1	-0.75382239	0.0108013
NSA2	0.55117869	7.4922E-17	DAAM1	-0.75303681	0.00558054
KIAA0907	0.55094669	0.01681736	GPX3	-0.75244691	0.01681736
ZBTB43	0.55059402	3.1105E-05	ARHGAP25	-0.75061144	0.00019612
TRABD	0.55051456	1.1937E-05	NAIP	-0.74928178	1.8256E-14
SRGAP2C	0.55000216	0.00204383	NPAT	-0.74925583	0.00307834
ELMO1	0.54974114	1.2976E-05	CLN6	-0.7491099	1.4202E-05
ANAPC16	0.54937526	1.5788E-11	TRMT1L	-0.74893009	0.01327113
KLHL6	0.54917357	0.00080664	PXDC1	-0.7482012	2.2185E-05
ATXN2L	0.54914806	0.00248549	LOXL3	-0.74756765	0.01769007
THBS1	0.54906111	0.04332211	CD82	-0.74745369	0.00923246
H3F3A	0.54829287	1.8109E-14	CNNM4	-0.74674222	0.0417002
PSMB10	0.54811326	8.4481E-07	SLC1A3	-0.74555442	0.01184451
PAPD7	0.54805566	0.03024397	FUT8	-0.74513169	0.040274
GCA	0.54789158	2.2185E-05	LINS1	-0.74476257	0.015964
RPS27A	0.54787859	1.6273E-22	CRAT	-0.74352967	0.00972129
MAP2K3	0.54728209	0.00412873	CLN5	-0.74326304	1.0469E-05
TAF11	0.54723191	5.3408E-05	ZNF576	-0.74282504	0.04580837
BCL3	0.54711093	0.01811466	MIR4458HG	-0.74212341	0.00135117
CSNK1D	0.54696392	2.29E-08	ZFHX3	-0.74165569	2.9749E-11
EPM2AIP1	0.54612949	0.00098774	ARSA	-0.74149412	1.1749E-07
MPG	0.54612297	0.0004539	ZMYND8	-0.74100505	7.9939E-05
SERP1	0.54610079	6.8787E-12	FBXL4	-0.74082322	0.04925884
PCTP	0.54542977	0.04480605	SLC17A5	-0.74056395	0.00188926
OTUD5	0.54503587	0.00448245	ARFIP1	-0.74004417	7.6107E-06
GNA12	0.54428573	0.00010606	CASP8AP2	-0.73978294	0.02398688
UBA52	0.54422946	1.8269E-20	SLC29A1	-0.73914424	0.01113376
TMEM80	0.54391556	0.02754539	MYLIP	-0.73914152	0.00043718
RBL2	0.54354811	0.00383967	MAP1S	-0.73881772	0.00057672
RPLP2	0.54346369	3.1709E-12	EOGT	-0.73781965	0.01058968
AP001055.6	0.54290389	0.00019171	IRF2BPL	-0.73671851	0.0130081
ELK4	0.5409368	0.00135834	LZTR1	-0.73668264	0.04360396
ADAP1	0.54072429	0.00389936	PHC2	-0.73638392	1.1794E-05
RBM8A	0.54040788	1.1661E-10	CD300LF	-0.73625289	0.00019451
EIF3G	0.54019593	8.8333E-15	IER2	-0.73475705	6.7109E-05
TRIO	0.54013174	0.00550757	CCDC112	-0.73447799	0.00010128
BAZ1A	0.5395593	0.00619488	SNX6	-0.73392139	6.5259E-09
ETV3	0.53942827	0.00074168	SUFU	-0.73367609	0.01118577
DENND6B	0.53936585	6.4257E-05	HIVEP3	-0.73235578	0.00722911
SLC25A3	0.53848092	1.9862E-08	SLC9A6	-0.73185472	0.04974092
CRTAP	0.53830224	0.00023886	SMS	-0.73001907	9.3758E-08

HNRNPA0	0.5381891	2.3949E-09	GGA2	-0.72841772	2.2972E-08
AES	0.53809655	0.00047145	ZNF322	-0.72778542	0.00612643
PRCP	0.53770559	4.3831E-12	LAMP5	-0.72760168	0.00035038
RIN3	0.53766638	2.5033E-05	ITM2B	-0.72739611	2.1676E-09
GLIPR1	0.53740128	1.4295E-08	P2RY6	-0.72661031	2.2206E-06
PDZD8	0.53734986	0.00759633	SLC12A7	-0.72543938	2.0461E-05
SNRPF	0.53722821	3.8804E-09	FAM217B	-0.7252818	0.02197963
SBNO1	0.53719226	0.00024127	NEDD4L	-0.7239762	0.00084197
LINC-PINT	0.53686755	0.0026443	TUSC1	-0.72384762	0.04440551
CCZ1B	0.53653695	0.00946263	CTNS	-0.72290458	0.01535256
NBPF14	0.53458173	0.01064422	SLC25A25	-0.72286073	0.03149443
EEF1D	0.53435834	1.8456E-17	CHD9	-0.72162899	1.0908E-11
GABARAPL2	0.53346774	9.2039E-07	LIMA1	-0.72149307	1.0357E-05
QTRT1	0.53320092	0.00095329	PTTG1IP	-0.72023149	1.4774E-18
ARHGEF6	0.53168188	9.9237E-06	FAM135A	-0.71959742	0.04872385
COTL1	0.53040935	7.4782E-08	GUCY1A3	-0.71955577	0.02957714
RP11-1094M1	0.53025851	0.04790438	NFIC	-0.71936763	1.1808E-08
PTS	0.5290049	0.00199775	CREBRF	-0.71437023	2.6099E-10
IKZF1	0.52895374	0.00023778	HMGCL	-0.71373452	0.0001731
PSIP1	0.52886654	0.00237131	DUSP6	-0.71206694	0.00038231
HMGN1	0.5287322	1.2334E-07	ARL11	-0.71205212	0.00638874
CIITA	0.5284638	2.9148E-05	KIAA0319L	-0.71161152	0.00379897
MTSS1	0.5276962	0.00897921	NSUN5	-0.71151984	0.00068722
RPL27A	0.52755434	7.7261E-19	NCOA7	-0.71073063	1.2302E-05
SLC31A2	0.52705341	2.9539E-05	CYB5R1	-0.70933819	7.3431E-08
MAP3K8	0.52698489	4.9959E-05	GNGT2	-0.70914944	8.8532E-05
ELOVL5	0.5266306	0.00024517	ADRB2	-0.70858697	0.00354245
DEK	0.52654511	1.1162E-07	PELI1	-0.70831606	0.00018679
KIAA1143	0.52594831	0.00020736	TNFSF9	-0.70812063	0.00010311
FBXO21	0.52571702	0.02777248	CLTA	-0.70760798	1.3925E-10
RHOU	0.52521384	0.03577855	METRN	-0.70720216	0.00548844
MED19	0.52442757	0.00256868	ADGRE1	-0.70664399	0.04711848
INO80B	0.52381535	0.04653528	GSTM4	-0.70606932	0.0003885
CLIC1	0.52358614	4.1234E-10	PARP12	-0.70567779	0.00829949
LRRFIP2	0.52338225	6.0145E-05	CXCL8	-0.70552424	0.04642979
APBB1IP	0.52313789	0.00090405	PPP1R21	-0.70544415	0.000265
SORT1	0.52245787	0.04038358	MUM1	-0.70483975	0.00131973
CXorf40B	0.52244475	0.0210199	NPC2	-0.70245976	1.3541E-16
MAPK8	0.52220447	0.01014155	EVA1B	-0.70202201	2.907E-06
SET	0.52086003	3.4553E-21	IL1A	-0.70169956	0.04902839
MYCBP2	0.52042756	7.5565E-10	TERF2	-0.70167008	0.00195388
HBS1L	0.52010347	0.01662407	RNF121	-0.70131063	0.02846228
HEBP2	0.51994243	0.00181511	TMEM222	-0.69919292	0.00473773
KDM4B	0.51962224	0.00459232	COPRS	-0.69896869	1.5036E-06
LEPROTL1	0.51895971	5.0429E-07	GPR160	-0.6987247	4.5745E-10
JAK2	0.51884994	0.04036188	AAK1	-0.69682124	2.3119E-08

FGD4	0.5182321	7.9429E-07	AKAP10	-0.69623825	0.00837169
POLE4	0.51821286	4.9026E-08	ZMAT3	-0.69535776	0.00293467
SRGAP2B	0.51807122	0.00109085	ARL3	-0.69365147	4.0253E-08
C16orf13	0.51778872	0.00025534	TRIP4	-0.69310605	0.00435372
RAD51C	0.51725473	0.00109299	KCTD10	-0.69202654	0.01066732
RPL38	0.51715673	2.1272E-17	SLAMF7	-0.69201159	9.6173E-06
TCOF1	0.51713244	0.03550303	UBR7	-0.69177963	0.00348301
MZT2A	0.51646529	0.00027921	ALDH9A1	-0.69163392	1.4167E-05
SMARCC1	0.51635884	1.5008E-06	HMGN5	-0.69144081	0.01084776
RBBP4	0.51597976	8.6538E-06	HN1	-0.69126574	3.3732E-06
ZNF394	0.51566547	0.00012956	MPZL1	-0.69125411	5.8211E-05
C2orf68	0.51552304	0.01899689	TMEM159	-0.69044749	0.00307804
DYM	0.51411853	0.00499072	GEMIN6	-0.69006493	0.01266696
ZC3H12A	0.51354528	0.00169121	PHYH	-0.68835367	0.00070602
CSNK1G2	0.51199377	0.00879971	DNAJC16	-0.67986861	0.02694327
MRPL3	0.51190385	8.5105E-05	FZD1	-0.67925526	0.01751559
SMDT1	0.51019798	4.4869E-08	HSP90AA1	-0.67767002	0.00956287
IRAK3	0.50986779	2.1355E-06	ZBED3	-0.67743459	0.02630679
CXorf21	0.5097232	0.04878985	MINPP1	-0.67628905	0.01748836
COPS3	0.50940984	3.8135E-08	NCK1	-0.67623928	1.474E-06
MXD1	0.50896728	2.5361E-09	CPEB3	-0.67547758	0.02810564
GSTP1	0.50874053	5.9867E-08	MUC12	-0.67544601	0.04895225
WAS	0.5086598	3.4994E-07	RASA1	-0.67347795	0.02146991
CBWD5	0.50850276	0.01328682	MTFR1	-0.6731464	0.01780571
XRCC6	0.50843795	1.0548E-05	LINC00998	-0.67241865	2.6013E-08
UPF2	0.50798522	6.4696E-05	SLC23A2	-0.67170102	0.01218411
SMC1A	0.50776862	0.00038244	ZNF330	-0.67008122	2.4587E-07
SNRK	0.50704057	0.00180451	DNMT3A	-0.66637145	0.00885404
PTP4A2	0.50675494	1.2574E-07	HS2ST1	-0.66633532	0.02203949
ODF3B	0.5049436	0.00120726	CLIC4	-0.66595168	0.00056257
SMCO4	0.5041688	0.04367453	ATP6V1A	-0.66555524	5.2501E-07
NSMCE4A	0.50396229	0.00314609	SCPEP1	-0.66513989	1.9551E-05
CLNS1A	0.50279806	1.7763E-06	PLCB3	-0.66510863	0.00656159
VRK2	0.50273331	0.00154981	NME4	-0.66400724	0.00029998
POR	0.50148887	8.5105E-05	KLF11	-0.66373921	0.00922896
IMPDH1	0.50073042	0.00733227	MGAT5	-0.6636504	0.00897779
USE1	0.50057604	0.00328601	MPEG1	-0.66220486	5.0162E-17
RP11-138A9.1	0.50036929	0.00084197	SLC15A3	-0.66198247	6.7538E-07
SLCO3A1	0.50035647	0.00837169	PCYOX1	-0.66116886	0.01387594
PTPRC	0.50004679	7.2923E-09	HEXA	-0.66060029	1.9537E-21
ATG2A	0.49910752	0.00066335	ADCY3	-0.66049542	0.01243278
CHD1L	0.49907313	0.01572493	VASH1	-0.66045802	0.00047092
APOBEC3C	0.49881134	0.00184618	CLEC11A	-0.65947508	6.274E-05
ARF6	0.49866971	1.42E-08	POP7	-0.65942653	0.00323071
STK38	0.49794481	0.02642147	SMPD1	-0.65929121	0.02446181
RAP1GDS1	0.49784521	0.00806606	NADK	-0.65881313	6.4141E-08

COX7A2L	0.49783278	1.567E-11	PCBD1	-0.65878313	4.187E-11
MRPS27	0.49753472	0.02720322	SLC25A44	-0.6582446	0.0012497
REXO1	0.4970148	0.01742208	TRIP6	-0.6578875	0.02445019
DENND1B	0.49687495	0.00030894	ZFYVE21	-0.65705444	0.00068317
ZNF511	0.49626055	0.00661443	ZNF37A	-0.65657763	0.03609051
ATP5A1	0.4961088	1.4358E-12	PALD1	-0.65639687	0.00075556
ANP32A	0.4957854	2.1034E-05	SLC39A7	-0.65630522	4.3056E-05
PMPCB	0.49572443	2.8104E-05	STAM2	-0.65514165	0.0096727
BIN1	0.49569423	0.00668316	RAB9A	-0.65504618	1.5041E-05
LUC7L	0.49548181	0.00098919	ZNF141	-0.65497269	0.04191149
CHST11	0.49529613	0.00162825	KLF10	-0.6542481	2.5737E-06
NME2	0.49510277	0.00653224	KIAA0196	-0.6539302	0.00106897
ZC3H18	0.49507233	0.00259742	AAMDC	-0.65390007	0.00015344
RBMX	0.49457185	5.5374E-09	PQLC2	-0.65275359	0.04804885
MAT2A	0.49435847	3.6014E-06	TNS3	-0.65273246	0.00093305
KPNA2	0.49396519	2.836E-06	INTS12	-0.65243561	0.01468136
CNBP	0.49349928	7.9034E-09	TGFBRAP1	-0.65230789	0.03390456
HNRNPA1	0.49283126	5.6311E-21	KIFAP3	-0.6522917	0.00954504
PDE4B	0.49270968	3.7036E-05	ZBTB48	-0.65210142	0.00803326
HCST	0.49218827	0.00082881	SNAPC3	-0.65060993	0.04708752
RBM3	0.49147676	5.9166E-12	ZFYVE19	-0.64966098	0.02645936
BACH1	0.49055432	0.00061515	RAD17	-0.64829914	0.03312741
NUP98	0.4904948	0.02086469	CLCN7	-0.64826862	4.6284E-05
TRIM5	0.49042418	0.04872726	ELL	-0.64785304	0.00100035
C7orf73	0.49015357	6.9345E-09	EXOSC4	-0.64771733	0.00020415
OXA1L	0.49001857	1.2353E-08	PDXK	-0.64688892	3.9534E-08
AKAP8	0.48967357	0.00244082	CISD2	-0.64636658	1.5072E-11
TNFRSF1B	0.48965911	7.5416E-06	C20orf194	-0.64633773	0.00979809
CHD3	0.48957256	0.00626956	NFATC2	-0.64443404	0.00028218
HSD17B10	0.48944651	0.00036883	MRPL17	-0.64395049	0.00107381
ACSF2	0.48916876	0.00599004	FNDC3B	-0.64388539	0.00123432
POGLUT1	0.4884269	0.01268443	SLC43A3	-0.64296985	0.00035169
CEP135	0.48821739	0.04885262	TMEM106A	-0.64210621	0.00038936
EIF2A	0.48813709	6.5302E-06	HEATR5A	-0.64184801	0.03216067
NOP58	0.48785266	1.1367E-06	BBS7	-0.64067787	0.04573661
TRAPPC10	0.48766311	0.00691206	RBM38	-0.64057245	0.00030457
GNAQ	0.48753707	2.5193E-06	BRD8	-0.63919543	0.02706604
HUS1	0.48738565	0.00877767	SLC25A24	-0.63900556	8.7392E-06
EEF2	0.4870909	1.9201E-17	NELFA	-0.63895611	0.01421808
POLD2	0.48672902	0.00242229	LINC00847	-0.63840375	0.00374777
NET1	0.48668774	0.00370176	ATXN1	-0.63828801	0.00055539
DAZAP1	0.48635966	0.00023167	IGHMBP2	-0.63765646	0.04149681
BRI3BP	0.4860947	0.00372897	ZSWIM6	-0.63750518	0.00049633
STAT3	0.48468767	1.52E-05	ATP11C	-0.63733459	0.00041915
SLC25A28	0.48462533	0.00623594	TMEM192	-0.63644485	0.00039058
RNASEH2A	0.48407371	0.02095964	KCNQ1OT1	-0.63545256	0.01165836

SAMM50	0.48322079	0.00280453	ZBTB38	-0.63404895	0.00123915
BRD1	0.48303495	0.04563248	SIAH2	-0.63355057	0.02645222
PCSK7	0.48266388	0.00075333	SOCS6	-0.63318759	0.04184541
IDH3A	0.48262733	0.00257299	ZC3H12D	-0.63260417	0.03869773
ERCC1	0.48188422	2.5231E-05	FEZ2	-0.63220072	4.744E-07
RPS6KA3	0.481667	1.8742E-06	SPATS2	-0.63179285	0.0006397
FBXO34	0.48144255	0.00445646	DHRS3	-0.62923658	2.9038E-05
CENPW	0.48106955	0.03316862	SLC22A18	-0.629202	8.1311E-06
PHF20L1	0.48050301	4.5365E-05	CKAP5	-0.62907707	0.01647016
RABGEF1	0.4803122	0.02910383	ERCC4	-0.6261582	0.02591521
INTS10	0.4791967	0.00634321	OTUD1	-0.6255617	1.1192E-05
ATP5G1	0.47919466	2.9209E-11	GOPC	-0.6250147	0.0188366
TMA7	0.4784416	1.2168E-11	TSPAN14	-0.62481984	0.00012071
THEMIS2	0.47701135	1.1139E-06	CCDC170	-0.62461872	0.00781237
EIF1	0.47638295	1.8001E-23	RAB13	-0.62460212	2.8106E-05
FAM60A	0.47587062	0.00311696	TBL2	-0.62400255	0.0043508
ANKLE2	0.47554328	0.00224391	DYRK2	-0.62236817	0.02916635
PRPF19	0.47534312	0.00498268	SLC3A2	-0.62090774	1.6076E-14
ROCK1	0.47454675	1.5686E-11	PSMB5	-0.61984106	4.9114E-07
ABT1	0.47435652	0.0122594	TNFAIP8L2	-0.61892305	3.0788E-05
CD72	0.47429745	0.00567811	SLC16A3	-0.61763433	0.00529773
ALG13	0.47426608	0.00131844	RNF14	-0.61639751	0.00631657
AKIRIN1	0.47420273	4.2635E-05	SMARCAD1	-0.61522631	0.01785559
CMTM6	0.47328471	7.5777E-09	HSBP1L1	-0.61178098	0.00697398
RPL35	0.47326165	1.916E-15	NSDHL	-0.61051909	0.03816326
ATXN10	0.47297069	0.00034385	RDH14	-0.60911667	0.00234156
SCNM1	0.47297066	6.7443E-05	DPP9	-0.6071688	0.00188831
MAN1A2	0.47269476	0.00087593	KCTD12	-0.60697944	3.0835E-05
C9orf142	0.47259427	8.8393E-05	PDK3	-0.60644397	0.00644023
MAP3K3	0.47247875	0.03033034	TFE3	-0.6046481	0.00109365
TAP2	0.47200794	0.0010679	SLC25A46	-0.60320159	0.00715936
PIGA	0.47200085	0.01184451	ORAI1	-0.60269755	3.4577E-06
KLHL5	0.4719239	0.00051854	SNAP29	-0.60237763	1.2195E-05
IKBKG	0.47171513	0.01257868	MAFF	-0.6018551	0.00226543
SARNP	0.47143297	0.00728492	ZDHHC14	-0.60182511	0.01715343
METTL22	0.47141569	0.03822325	MIER2	-0.60166585	0.02933668
PHF20	0.47137234	3.9387E-06	DENND4C	-0.60103529	0.01457888
TUBB4B	0.47089476	2.7434E-09	HACD3	-0.59901506	0.00102432
RPS6KA4	0.47070771	0.00150594	ARAP1	-0.59866449	5.4083E-05
MSN	0.4706644	6.6563E-07	BLVRB	-0.59792661	4.7463E-09
RNPS1	0.47055028	8.9144E-08	QRSL1	-0.59714018	0.01898333
MAD1L1	0.47048184	0.03110667	AKR1A1	-0.59579622	2.1265E-13
MTHFR	0.47035178	0.00593591	ASAH1	-0.59573117	3.6121E-11
UQCRFS1	0.47033616	1.1676E-08	TLR1	-0.5956094	0.00057565
TLE4	0.46950077	0.00271635	HLA-E	-0.59527908	7.2213E-10
DNTTIP2	0.46928446	0.00014844	ACBD5	-0.59483301	0.00089792

SPOPL	0.46836958	0.01016609	AARS	-0.59466825	0.00963174
DDX6	0.46834188	7.0664E-06	ARID5B	-0.59395416	0.00320011
NDUFA10	0.46826914	1.163E-07	PGPEP1	-0.59389321	0.01113534
STARD4	0.46814857	0.00888484	TMEM70	-0.5921071	2.4786E-06
OGDH	0.46804038	0.01636419	DOCK7	-0.59126128	0.01334163
VHL	0.46789637	0.00128802	CEP290	-0.59067081	0.0060196
PPIH	0.46709427	0.00095012	KLHL21	-0.58951504	0.03016112
RNF145	0.46697248	0.00090225	DLAT	-0.58950426	0.02806316
ZNF385A	0.46677332	1.0322E-06	AKR1B1	-0.58916956	3.1518E-08
DDB2	0.4667182	0.0239567	CTNND1	-0.58843891	0.0032698
CHD2	0.46641983	0.00182662	CAPG	-0.58841889	0.00143375
PHPT1	0.46564339	6.7562E-07	ACOT13	-0.58710548	0.0013758
ECI1	0.46536746	0.00385377	ATG7	-0.5866368	0.00072514
VBP1	0.46507305	0.00316163	ANKRD39	-0.58602123	0.00529663
VPS51	0.46506974	0.00046174	PLEKHM2	-0.58590951	0.00019495
HIGD2A	0.46464513	7.0513E-10	INPPL1	-0.58538379	0.00344769
TTC17	0.46458567	0.00243438	HNMT	-0.58537673	2.224E-10
POLR2B	0.4643267	0.00082868	CDS2	-0.58502107	0.00049847
ADSS	0.46429379	0.00106897	DDI2	-0.58410787	0.00612032
METTL21A	0.46347502	0.0302948	RNF135	-0.58405268	0.00671852
CD47	0.46341224	2.1727E-08	ACOT2	-0.58403793	0.03253613
BCL7B	0.46314265	0.00114254	CPSF4	-0.5839884	0.01526196
NADSYN1	0.46306905	0.02408776	TRIP11	-0.58361343	7.0021E-05
COX4I1	0.463053	7.3465E-14	SAYSD1	-0.58291727	0.02473808
LILRB2	0.46275021	0.00125755	FGD3	-0.58169881	0.00202082
USP5	0.46246845	0.04676876	FMN1	-0.581484	0.00041915
RQCD1	0.46239892	0.00066545	CPNE8	-0.58112947	0.00026334
ERICH1	0.46238336	0.00031874	C7orf49	-0.57802067	0.0202523
SMG1	0.46224851	0.00159115	TXNRD1	-0.57799067	6.265E-07
MALAT1	0.46224363	6.5908E-06	MRAS	-0.57774201	0.0024357
MARCH6	0.46195537	0.00124885	EML4	-0.57757337	0.00010788
MED4	0.46184827	0.00052125	PEX11B	-0.57652808	0.03937938
STK38L	0.46154623	0.01880546	LCORL	-0.57631672	0.02541857
ATP5G2	0.46149487	1.433E-25	MAN2B2	-0.57513984	0.01407731
SRSF5	0.46138759	6.4148E-07	DOHH	-0.57476418	0.03095614
SDE2	0.46118593	0.00038125	ZCCHC11	-0.57377108	0.01988147
RIOK1	0.46096862	0.00389189	MAPK6	-0.57313375	0.00306888
SNX30	0.46066212	0.04878298	ZNF44	-0.57276471	0.03593172
CKS1B	0.46054452	0.00786764	KLHL7	-0.57275103	0.00989998
R3HDM1	0.46052992	0.01279492	FAM63B	-0.57230113	0.02801121
RPL12	0.46032602	4.3092E-29	FCGRT	-0.57186378	2.145E-08
ILF3	0.45994039	1.3779E-05	SSH1	-0.56953449	0.00946496
ROGDI	0.45992327	0.02702878	BCAP31	-0.56904472	2.2429E-14
GSTK1	0.45987208	1.1667E-05	NUDCD3	-0.56848969	0.01711834
TMEM109	0.45983597	0.00599004	DUSP1	-0.56833138	0.00165386
NOB1	0.4598144	0.01152421	GAB2	-0.56820759	0.00107588

RPS26	0.4593143	2.5309E-09	NFATC1	-0.56721643	0.00055231
HSD17B11	0.45764606	3.2085E-05	ELMOD2	-0.56692617	0.00449523
ETV6	0.45708914	0.00822942	RFC2	-0.5666701	2.3271E-05
EDF1	0.45617479	1.6133E-11	DHDDS	-0.56553904	0.04615238
PHF10	0.45602096	0.00661773	HINT3	-0.56466987	0.02657799
ZNF652	0.45504292	0.04171223	NCOA6	-0.56402364	0.02702258
PCIF1	0.45502766	0.01107254	NBR1	-0.5631969	0.00292349
NSMCE1	0.45502093	0.00051301	PDZD11	-0.56267882	0.00087593
BIRC3	0.45438268	3.3911E-05	CDK4	-0.56223668	3.5595E-05
PPIF	0.45404662	3.2958E-06	CHORDC1	-0.56172964	0.01227509
MIS18BP1	0.45305268	0.04483584	LILRB4	-0.56152832	0.0009663
ABCE1	0.4528964	0.00594709	NOC3L	-0.55987543	0.00175271
NFKBID	0.45216589	0.02260761	ADH5	-0.55891197	2.6251E-06
APRT	0.45174457	5.982E-09	TMEM185B	-0.5576849	0.01727494
LYPLA1	0.45099964	2.6003E-05	DFFA	-0.55610202	0.00169274
CBWD2	0.45088055	0.01629241	GDE1	-0.55508815	0.00044612
ZBED6	0.45086662	0.0368302	OAZ2	-0.55446325	2.7347E-09
EIF4B	0.45076992	7.5225E-11	ZNF688	-0.55446176	0.01848984
IVNS1ABP	0.45027192	0.00556548	ZFP36L2	-0.55275452	0.00113989
KCNQ1	0.44990465	0.0372608	MGST3	-0.55256598	4.8074E-08
HINT2	0.44952219	0.00338118	GTF2A1	-0.55135084	0.00317073
EMD	0.44927051	0.00040713	BIN2	-0.54948821	6.6394E-05
HACD4	0.44892522	0.00010002	SLC20A1	-0.54901928	0.00085152
XPNPEP1	0.44891351	0.01894701	CPED1	-0.54725218	0.00101203
FDFT1	0.44888777	0.02578973	FAM168A	-0.54670736	0.03208823
N4BP2	0.4485358	0.00128251	FAM179B	-0.5461907	0.04700526
HMGB2	0.44821416	5.3641E-05	CLN8	-0.54618132	1.7768E-08
EIF2S3	0.44818082	1.0514E-07	POLR3C	-0.5458568	0.04170376
CNOT6L	0.44793173	0.00282469	GIGYF1	-0.54555398	0.02499781
CTDSP1	0.44787234	0.01461516	MPI	-0.54554408	0.01003763
PSME2	0.44748873	0.00018122	RDH11	-0.54533359	0.00032224
PFDN5	0.44743593	1.7462E-14	SEC22B	-0.5421938	0.00023196
TMLHE	0.44720313	0.03430956	TCEAL8	-0.54155217	4.4776E-08
BRK1	0.44649306	8.6707E-13	TUSC2	-0.54135174	0.0008708
FRAT2	0.44649041	0.01814712	ISCA2	-0.54124843	0.02056461
SNRPA	0.44609589	0.00097515	ING1	-0.54048355	0.0005874
TOMM22	0.44606101	0.00014832	B3GNT2	-0.54039702	0.04374623
ZNF791	0.44598991	0.02282097	PRKACB	-0.54016874	5.1485E-05
CELF2	0.44546311	4.1377E-08	FRMD4B	-0.54000489	8.5347E-07
MED28	0.44487126	2.8659E-07	C9orf91	-0.53937075	0.01819172
RBM15B	0.44408836	0.04052451	NOL8	-0.53912272	0.00020534
NR1H2	0.44406102	0.00065919	GOLIM4	-0.53796003	4.348E-06
PHB2	0.44360334	1.2958E-06	CCPG1	-0.53736354	4.7463E-05
UBE2D2	0.44284957	4.7837E-08	ATP6V0E1	-0.53661306	1.5507E-14
GTF2A2	0.44278351	4.0889E-06	FAM126B	-0.5361009	0.01629241
SRGN	0.44247934	8.0355E-10	TTC1	-0.53516471	9.8251E-06

DECR1	0.44200269	0.00062717	SGSH	-0.53487297	0.02045881
LONP2	0.44176082	0.00083322	SNX5	-0.53381433	5.7992E-11
MBD2	0.4410655	9.3567E-05	TMEM223	-0.53378824	0.01058155
PSMA3-AS1	0.44091503	0.00081175	DSC2	-0.53377083	0.00667899
TRIM38	0.44015322	0.00486016	DEDD2	-0.5336544	0.00948092
PCGF5	0.43966478	0.00105381	PTRH1	-0.53252324	0.02421376
AP2A1	0.43909496	0.01664019	FTSJ2	-0.53230361	0.00294402
PNN	0.43900085	5.8615E-05	SIGLEC10	-0.53105222	8.5105E-05
EIF4E2	0.43838277	0.00129262	EMC2	-0.53045188	0.02543424
CCNI	0.43831285	6.4141E-08	TMBIM6	-0.53040057	2.8518E-20
C1QBP	0.43813431	4.5229E-05	TP53RK	-0.53006496	0.03047108
HNRNPA2B1	0.43788375	2.6911E-08	NUCB2	-0.52993154	0.00013393
ILF2	0.43753869	1.1338E-07	CISD3	-0.52922667	4.3631E-05
SLC25A5	0.43622137	1.1385E-08	CD93	-0.52892873	2.9614E-07
CCNY	0.43619704	4.6284E-05	PPP2R4	-0.52850998	0.0010995
INSIG1	0.43616623	0.00014038	FOXO1	-0.52761649	0.00207012
BRE	0.43590974	0.00883084	RHNO1	-0.52686139	0.0217086
CASP4	0.43540384	0.00011124	CGRRF1	-0.52682989	0.04446576
TAF7	0.43520698	0.0005274	EPB41L2	-0.52620112	0.015623
NHP2	0.43473207	1.1144E-05	SNX2	-0.52534245	4.4924E-05
TOP3A	0.43453466	0.0355775	CAPZB	-0.52427245	9.3403E-13
PAN3	0.4342934	0.00631657	TRAF3	-0.52340296	0.00874451
GPCPD1	0.43347914	0.00012944	FAM134A	-0.52151135	0.02970686
DUS1L	0.43125193	0.00104285	CHCHD1	-0.52103729	2.8686E-05
NSMAF	0.43121333	0.01700076	CFAP97	-0.52089312	0.00389189
HNRNPR	0.43082716	4.4837E-10	CDC27	-0.52018765	0.00240014
MECP2	0.4306264	0.00180964	CRYL1	-0.51919184	0.00695926
COPS8	0.43048407	0.01280798	CD2AP	-0.51886712	2.1513E-05
ZNF830	0.42941652	0.03657041	LAIR1	-0.51771294	0.00019385
LSM5	0.42854149	0.00038987	WRB	-0.51684572	0.0332312
C7orf50	0.42842799	0.0020701	CSTB	-0.51607565	6.0445E-11
GAPDH	0.42837195	3.5869E-10	P4HA1	-0.51606653	1.8171E-05
RPL23	0.42819025	5.0178E-21	RRAS	-0.51587783	0.03159776
PPM1F	0.42782031	0.04563941	ATP2A2	-0.51567263	0.00122765
BRD3	0.42740718	0.04122761	ABCC4	-0.51559077	0.04164189
NR4A3	0.42715272	0.01065501	C18orf8	-0.51396687	0.02183232
NDUFAF2	0.42698023	0.00097303	RCOR3	-0.51384885	0.01881805
NSMCE3	0.426341	0.01279492	GALK2	-0.51381934	0.03149443
BDP1	0.42615364	0.00200419	KLF7	-0.5132637	0.02245254
RPS6KB2	0.4260788	0.00343683	ZNF358	-0.51296407	0.04676637
WDR43	0.42501725	0.0007258	CEP95	-0.51263751	0.03613
HNRNPL	0.42489856	0.00023905	TMEM206	-0.51151673	0.01103746
CPPED1	0.42488526	0.00019635	ANKFY1	-0.50931878	0.04851503
TBL1X	0.42478817	0.04204293	MRPL35	-0.50872894	0.00475562
ITGAE	0.42457095	0.00176129	AP5Z1	-0.50816674	0.01058968
PAIP2	0.42431764	5.5311E-10	HELZ	-0.50616769	0.00071483

CHD6	0.42431477	0.00212968	OSTM1	-0.5054276	0.00047274
FOXN3	0.42412184	0.00008257	RASGEF1B	-0.50509398	0.00042605
ATP5L	0.42383948	7.2533E-09	MED26	-0.50504787	0.03306853
TAGLN2	0.42307001	3.4725E-06	VIMP	-0.50463565	0.00038535
RBM4	0.42295587	0.0054828	TPD52L2	-0.50411165	0.00043634
UBA2	0.42245662	0.00088656	HSPA5	-0.50364492	5.2182E-06
CCRL2	0.4223667	0.01801077	ZSWIM8	-0.50310746	0.01228252
NAA10	0.42211283	8.1664E-05	CLTC	-0.50171714	1.7507E-07
ZNF800	0.42198344	0.04149681	LGALS8	-0.5006567	0.00012669
SNRPD2	0.4214477	1.0112E-07	IL18BP	-0.49906073	0.00047013
VRK1	0.42142179	0.0127877	LDLR	-0.49901757	9.2037E-05
EVI2B	0.42109562	0.00148949	ATP6V1H	-0.49698441	1.5964E-05
CNOT7	0.42073654	0.00178974	TGFBR2	-0.49682201	0.00073041
QARS	0.42066043	0.00019061	KIAA1033	-0.49669053	0.00095384
HAUS7	0.42061097	0.0243521	RAD9A	-0.49643865	0.02229757
CAMK1D	0.42048972	0.00929323	SLF2	-0.49569084	0.0279031
C4orf33	0.41993978	0.02593568	FAM76A	-0.4954172	0.01656743
GHITM	0.41921876	8.3711E-08	NFKBIZ	-0.49476667	0.00361728
ARL5A	0.41915317	3.9493E-08	KAT5	-0.49380248	0.00487091
DDHD1	0.41910856	0.00427809	CD163	-0.49351555	0.0111287
PSMA6	0.41816734	0.03999393	ARMT1	-0.49350308	0.01509461
HINT1	0.41775462	1.67E-07	DPP7	-0.49308124	8.8375E-11
HNRNPD	0.41765555	1.6646E-06	MGME1	-0.4927925	0.01085105
IRAK1	0.41757731	0.01080206	FAHD1	-0.4924074	0.006988
CSNK1A1	0.41751132	1.5535E-05	ZNF331	-0.49054673	5.3594E-05
PCBP2	0.41704583	1.5454E-10	DSTN	-0.49024128	6.4302E-10
BUB3	0.41680167	0.00211367	KIN	-0.49000208	0.00464383
ESD	0.41558691	3.6582E-05	DIRC2	-0.48980183	0.0177091
XRN2	0.41516514	4.1048E-09	FNDC3A	-0.48955043	0.00562112
CACNA2D4	0.41478939	0.03793832	ABCD4	-0.48879408	0.00967192
UBE2N	0.41467369	4.428E-06	ATP6AP2	-0.48861382	3.0196E-06
PDCD4	0.41445593	0.00038724	GLA	-0.48737533	1.4049E-05
DPYD	0.41422662	0.00263012	SUCLG2	-0.48677515	2.7454E-05
SMC5	0.41397699	0.01579284	SMIM4	-0.48660868	0.00350075
CASP1	0.41396809	5.1421E-05	SETDB2	-0.48608346	0.00949621
HIPK3	0.41323582	0.00048646	APOO	-0.48594411	0.03598612
FAAP20	0.41316628	5.5329E-05	LMAN1	-0.48428106	0.00033801
NKTR	0.41277521	0.00124196	HPS4	-0.48384335	0.04412113
PYURF	0.41276514	0.0005921	CLPTM1	-0.48322047	0.00760791
GTF3A	0.41239345	2.0975E-05	MYCL	-0.48314463	0.02567073
HNRNPAB	0.41239095	3.1573E-05	MFSD12	-0.48290851	1.0218E-05
LAP3	0.41196075	0.00018773	SRPRB	-0.48231824	0.00322549
ARMC8	0.41165679	0.03095614	HPS3	-0.48199849	0.00145253
PABPN1	0.41161461	9.2124E-05	TM6SF1	-0.48199024	0.0029151
ADSL	0.41145224	0.00235188	SOAT1	-0.48192748	0.0004114
BCL2L13	0.41139448	0.00373819	PPARA	-0.48184978	0.04875312

DDX3X	0.41132049	1.6665E-07	CHMP3	-0.48063184	3.8441E-06
UBL7	0.41129625	0.00476593	UPF1	-0.48023122	0.01814417
CDK16	0.41045913	0.02091805	GNB4	-0.47987469	0.00075717
C3orf58	0.41012392	0.00583319	GADD45B	-0.47957611	0.00713907
USP34	0.40950782	0.00346247	CHCHD10	-0.47929862	1.4726E-05
ELF1	0.40920491	4.4621E-07	GRINA	-0.47908507	0.00031565
TMEM205	0.40908698	0.00126871	FAM46A	-0.47908377	0.02469108
USP25	0.40902227	0.0464804	DPF2	-0.47820657	0.02135546
TRIM44	0.40890872	0.00018441	TIAM1	-0.47790793	0.01132781
VEGFA	0.40824445	0.00135834	ATP6V1D	-0.4766667	0.00087768
USP48	0.40807213	0.0031766	MRPS31	-0.47635943	0.0121033
CCT6A	0.40715498	1.7091E-05	ATP10D	-0.47485742	0.01939969
ARL6IP6	0.40707598	0.04644061	HCFC1R1	-0.47333761	0.00224213
ADAM15	0.40696491	0.03999872	C11orf71	-0.47288777	0.04889702
UBE2F	0.40695967	0.00600404	TNFRSF14	-0.47262579	3.5171E-06
MDH2	0.4068877	1.6002E-08	WDR5	-0.47247284	0.04670517
JAK1	0.40656446	0.00058449	TECR	-0.47239849	2.6386E-05
CBWD1	0.4062749	0.00180793	P2RX4	-0.47219367	0.00231531
CAT	0.40591611	0.00167297	STOM	-0.47196865	0.00819131
FLOT1	0.40564641	0.00134201	BRI3	-0.47181644	2.3793E-10
NOC2L	0.40501723	0.02960416	BST2	-0.47066639	6.8336E-10
PYM1	0.4047845	0.00655187	TPP1	-0.47029794	1.1456E-11
ARL16	0.40445433	0.0224196	FUBP3	-0.47012324	0.01907256
ARF3	0.40436067	0.00057693	PPFIA1	-0.46862214	0.00124341
TSSC4	0.40379782	0.01054923	C5orf56	-0.46683142	0.04491446
USP22	0.40362547	0.04657027	SESN3	-0.46654032	0.01476509
ARRDC1	0.40234655	0.00202119	C3orf62	-0.4664471	0.04850133
PCID2	0.40193735	0.0485933	TMEM199	-0.46605561	0.01113534
MYL12A	0.40105134	1.2166E-05	REPIN1	-0.46600832	0.00612643
CLEC7A	0.40069301	4.9223E-07	WBP2	-0.46313792	9.1104E-05
FBXO9	0.40060147	0.0203465	PIK3R1	-0.46218314	0.00218672
LINC00493	0.40043035	1.0384E-05	NECAP2	-0.46173854	1.4821E-05
NUDT1	0.4003389	0.00146607	CCDC43	-0.46152544	0.00642809
POLE3	0.40017364	0.00117216	KCNE3	-0.46065501	0.04594484
CDC37	0.39955009	0.00060757	CACYBP	-0.45980744	0.00116976
SLC12A9	0.39925279	0.01272298	AK3	-0.45974317	0.0016293
EIF3I	0.39901235	6.2879E-05	GPANK1	-0.45967917	0.03085088
UXT	0.39875576	0.00012072	SUPT20H	-0.45795749	0.02691057
HSCB	0.3982713	0.02398392	SCOC	-0.45757541	0.00640602
RPS20	0.39785667	1.8839E-19	SLC4A7	-0.45552956	0.00283053
EIF3D	0.39778689	2.6386E-05	ABHD3	-0.45544091	0.02209056
PPA2	0.39754732	0.00066803	BAIAP2	-0.45541702	0.0096727
NCOR1	0.39737395	0.00045489	GMPPA	-0.45516348	0.02461424
RBM7	0.39728867	0.0005274	LAGE3	-0.45451526	0.00074107
JPX	0.39714555	0.00683429	PUM1	-0.45424976	0.00323883
PTTG1	0.39711979	0.01459839	UTP18	-0.45327343	0.00065049

PNO1	0.39572389	0.00941964	PRDX4	-0.45317034	0.00023161
RNF168	0.3951807	0.02186956	ATP6V1F	-0.45241124	3.1665E-07
CEBPG	0.39516688	0.01969451	AKIP1	-0.45149519	0.00451351
POLR1D	0.39514059	7.7738E-07	FARSA	-0.45135044	0.00252003
HNRNPH1	0.39501474	5.9069E-05	CEBPB	-0.45072703	0.00199788
SLC35E1	0.39497954	0.00401873	NAGK	-0.44995562	1.4556E-05
STX16	0.39491648	0.04769294	ICT1	-0.44980162	0.02081186
RABGAP1L	0.39472497	0.04126656	SPNS1	-0.4494617	0.00437956
DR1	0.39418539	0.01114323	ST3GAL6	-0.44889734	0.00201398
SRP72	0.39411687	5.2172E-05	HDLBP	-0.44865243	8.1297E-05
ACTN4	0.39361044	0.00089282	MVD	-0.44854634	0.01714633
CDK11A	0.39354736	0.04704022	SUMF2	-0.44795018	0.00022678
FOSL2	0.39314981	0.00082211	AHR	-0.44781998	1.1441E-05
OGFRL1	0.39308286	0.00011386	FGL2	-0.44710487	1.6655E-07
GALK1	0.39254638	0.00880663	MTR	-0.44685657	0.00407201
STXBP2	0.39250866	4.7463E-05	NPTN	-0.44627198	0.00144409
PHF5A	0.39219503	0.00569997	TSPAN31	-0.44506197	0.00665507
RPGR	0.39180625	0.02817708	DICER1	-0.44495868	0.00018871
GDI2	0.39175732	9.011E-10	HSDL2	-0.44455672	0.01367375
TSHZ1	0.39172033	0.03853121	C2orf74	-0.44260692	0.0217026
EVI2A	0.39166571	0.00034757	USP9X	-0.44168573	3.1916E-05
SNRPB	0.39089972	1.5063E-05	PANK2	-0.44148255	0.00237131
PPP1R10	0.39032173	0.01238867	ARL1	-0.4410006	0.01056819
CARS2	0.39017364	0.01344596	COPG1	-0.44029343	0.01622446
SRRM1	0.39003302	8.7735E-12	HNRNPUL2	-0.43935882	0.00445033
GBP2	0.38956865	0.02950368	CD38	-0.43902418	0.0219027
MSL3	0.38933725	0.00739755	AGGF1	-0.43900174	0.01212947
SUGP2	0.38888629	0.03312741	CTR9	-0.43892928	0.01898333
SH3BGRL	0.38886383	2.3423E-07	TMED9	-0.43833511	6.9509E-06
PITPNB	0.38886369	0.02326365	GIMAP2	-0.43494984	0.02406322
IPO5	0.38884745	0.01269379	SPIN1	-0.43493639	0.00499072
SLC50A1	0.38828148	0.01174912	JKAMP	-0.4331977	0.00345138
DERA	0.38782043	0.04593353	EIF4A3	-0.43280569	0.00027262
RPLP1	0.38766688	3.0648E-14	C6orf1	-0.43197938	0.02891829
WAC	0.38757687	0.00034751	TNFAIP3	-0.43186272	0.00746735
MFSD14B	0.38737133	0.02623172	SNX13	-0.43107871	0.01923182
ETF1	0.38734247	2.2912E-05	EPSTI1	-0.43024458	0.00655186
CNOT1	0.3873075	0.01569478	WDR26	-0.4301391	0.00131743
SFPQ	0.38725055	6.9279E-12	PEPD	-0.42965072	8.2239E-06
RBM23	0.38678942	0.00293983	DCTN4	-0.42942455	0.00049074
THUMPD3-AS	0.38652682	9.1019E-05	H2AFJ	-0.42732906	6.6909E-06
UBE2I	0.38646634	5.4152E-05	TIPARP	-0.4266789	0.00659405
FDPS	0.38609942	0.00019691	GAS7	-0.42526629	0.00717584
SNRPD3	0.38592122	1.8366E-06	HEXB	-0.42456338	6.3029E-07
CSK	0.38287223	0.00027744	DPAGT1	-0.42400425	0.02444173
LSM8	0.3828672	1.0322E-06	RABEP1	-0.42326392	0.01073646

SNRNP25	0.38275945	0.00220926	HNRNPLL	-0.42318881	0.03086472
ISG20L2	0.38269369	0.02639572	RRAGC	-0.42309951	0.00072209
CSGALNACT2	0.38257261	0.00110995	GUSB	-0.42206651	2.5231E-05
TRMT10C	0.38235158	0.00687502	PSMC2	-0.421625	0.00063234
METTL9	0.38219232	0.00084216	NAGA	-0.42156479	0.00012155
LSM6	0.38181453	2.4564E-05	NR4A1	-0.42086159	0.04514642
SF1	0.3816749	2.0514E-05	CCM2	-0.4205005	0.01082366
CWC25	0.38166991	0.00254808	SLC15A4	-0.42037606	0.02479002
EIF3J	0.38075465	4.2875E-07	HIC1	-0.41858298	0.0192214
PAICS	0.38069747	0.03695087	STX12	-0.41768001	0.00454551
THOC7	0.38042296	3.6276E-06	C14orf119	-0.41669827	0.00409377
STRA13	0.3802395	0.01294857	IFRD1	-0.41654305	0.01294857
HNRNPM	0.3791735	4.5946E-09	RGS10	-0.41580926	5.1477E-15
KBTBD8	0.37808106	0.03948803	FAM91A1	-0.41579667	0.00612643
SLU7	0.37692345	0.01058968	CLPX	-0.41563095	0.04927394
EXOSC6	0.37654991	0.00813335	MTIF2	-0.41552621	0.01509208
FAM200B	0.37616809	0.01610195	RRP1	-0.41338889	0.03378535
DDX46	0.37585455	2.2834E-06	MRPL27	-0.41279063	0.000143
PPHLN1	0.37572299	0.00013157	COA6	-0.41225415	0.02603873
BAG5	0.37425695	0.04902839	RCHY1	-0.4116936	0.02172124
CMPK1	0.373977	0.00037924	IER3	-0.41105119	0.02806316
GGNBP2	0.3738157	0.00110689	BTG2	-0.41096024	0.01752021
STAG2	0.37361654	0.00016975	PRNP	-0.4103321	0.00527238
BRD4	0.37337184	0.00010206	PPP2CB	-0.41026621	0.01526858
AK2	0.37320711	0.00458069	CALR	-0.40950701	2.0094E-05
KMT2E	0.37300218	0.00162701	NSL1	-0.40846471	0.00988036
ZNF277	0.37293167	0.00818831	DNAJC5	-0.40816059	0.01240822
TAF10	0.37287049	0.00567542	DNAJC13	-0.40789301	0.01985478
OSBPL9	0.37272886	0.03842243	CREB3L2	-0.40622013	0.02524394
CCZ1	0.37235447	0.03058841	DDB1	-0.40619353	0.00394227
BCL2	0.37147296	0.01513702	FMNL3	-0.40604269	0.03814939
SRSF2	0.371407	1.7268E-07	NAA20	-0.40595035	0.00010204
FOXP1	0.37088449	0.03115448	PECAM1	-0.40486174	0.00204431
HLA-DPB1	0.3707715	1.4652E-06	HTATIP2	-0.40389019	0.00498434
DPM1	0.36991131	0.00644966	IFI6	-0.40385914	0.00686513
ARPC1B	0.36970939	7.7271E-05	PAK1IP1	-0.4022171	0.02172124
RUNX3	0.36921403	0.03306853	USP37	-0.40109898	0.01771247
HPRT1	0.36908379	8.8645E-05	UTP23	-0.39930693	0.02562006
YPEL5	0.3690479	0.00527262	TCEAL1	-0.39885004	0.0418848
ELF2	0.36855894	0.00048133	CTCF	-0.39843259	0.03629405
ACTB	0.36778812	3.5632E-09	NENF	-0.39678752	6.3703E-05
STX11	0.36758748	0.01059587	GRN	-0.39668097	1.382E-06
NFKB1	0.36739277	0.04455062	PGD	-0.39653633	0.02624389
ARFGEF1	0.36715522	0.04907089	IMP3	-0.39592286	0.03039995
PRRC2C	0.36696759	8.1332E-05	C4orf48	-0.39552283	0.00098646
APEX1	0.36676384	1.9253E-06	LPCAT1	-0.39409006	0.01113564

RASSF3	0.36670657	0.04933772	SNAPC5	-0.39349208	0.04374338
RAP1B	0.36596294	0.00074388	ABI1	-0.39321907	4.7047E-05
ADGRE5	0.36473845	0.00173965	RAC1	-0.39247678	1.396E-12
PCNP	0.36455004	6.2879E-05	MFSD1	-0.3918169	0.0002434
ZSWIM7	0.36428586	0.0009513	ATP6V1E1	-0.39174424	5.3122E-05
C19orf43	0.36425552	0.00082881	C6orf89	-0.39170547	0.04670517
TCF25	0.36399537	2.9321E-06	LPCAT3	-0.39112132	0.04755834
ETHE1	0.36375749	0.01329357	RALB	-0.39058325	0.00291205
MTHFD2	0.36371377	4.7413E-07	TRAPPC2L	-0.39032705	0.00168651
ARID5A	0.36303912	0.00021468	PDIA6	-0.38939305	1.418E-05
CCT7	0.36281777	0.00080603	C16orf87	-0.38930909	0.02631674
PSEN1	0.36248994	0.03087696	FDX1	-0.38789363	0.01107254
NDUFA7	0.36239284	0.01308675	VKORC1	-0.38669036	8.5818E-05
PSPC1	0.36228867	0.04927394	PTGER2	-0.38657912	0.04563941
CXorf40A	0.36217505	0.03573561	CASC4	-0.38588731	0.00929905
IDH3B	0.36197278	0.00061696	LTBR	-0.38402728	0.00924298
GPI	0.36174358	0.01734943	CCDC47	-0.38365419	2.0431E-06
ARHGAP5	0.36140717	0.03356864	SMIM20	-0.38330253	0.00012527
CRK	0.36123604	0.02541093	ARID4A	-0.38269603	0.00095329
EMILIN2	0.36071811	7.3047E-07	GMFB	-0.38177825	0.00877767
PRELID3B	0.36056573	0.00321698	PHTF2	-0.38176185	0.01988147
FAM136A	0.36056088	0.03285158	MYDGF	-0.38165274	0.00124547
PEX16	0.36022019	0.04669715	MRPS18C	-0.38090008	0.00353971
CHCHD3	0.35982364	0.01322632	TIGAR	-0.38026313	0.00496906
CBX3	0.35907643	6.6463E-06	DTNBP1	-0.38019246	0.02353777
CAPNS1	0.35888467	1.0275E-05	WDR55	-0.37949761	0.04366007
PAPD5	0.358805	0.00821938	DOCK11	-0.37810595	0.01975364
TNFSF13B	0.35851191	0.00884853	TRA2B	-0.37795421	0.00854673
YPEL3	0.35819905	0.02570052	M6PR	-0.3779457	5.0929E-05
IWS1	0.35810319	0.03401174	PRDX2	-0.3763638	0.02887058
TOR2A	0.35728069	0.03490005	ANXA4	-0.37592535	0.00944524
BCAS2	0.35676015	0.01243278	IFI44	-0.37475119	0.04900413
ENY2	0.35638489	4.6634E-09	CAMK1	-0.37429439	0.00016593
RAN	0.35603537	1.1164E-08	PGRMC1	-0.3733528	0.01872735
PHACTR1	0.35600389	0.00613593	SAE1	-0.37278407	0.03574905
PAFAH1B3	0.35590517	0.04481695	UTRN	-0.37261901	0.00956287
SNX17	0.35443229	0.0001988	CYTH4	-0.37226002	0.01105709
ETS2	0.35443221	0.0032941	TWF1	-0.37085718	0.03205239
CEBPD	0.35381563	0.00124658	FAM105A	-0.36975757	0.00111221
TUFM	0.35324596	0.00019315	PIAS1	-0.36865547	0.00437686
ORMDL1	0.35286438	0.00059367	GAK	-0.36841535	0.01239373
URI1	0.35246184	0.01294857	MACF1	-0.36840397	0.00043718
MPLKIP	0.35221733	0.01057518	AIG1	-0.3682559	0.00834438
TCERG1	0.35207426	0.00364116	RAB35	-0.36806646	0.03305024
SETD2	0.35200839	0.04774855	TMEM30A	-0.36795861	0.03479881
SPAG9	0.35192945	0.00691206	RABAC1	-0.36766772	0.00095384

UFC1	0.35173127	0.00070869	RRP36	-0.36737566	0.03942966
CTNNB1	0.35125051	0.00946263	MILR1	-0.36698786	0.01942663
CCT8	0.35055142	9.2124E-05	ATP6AP1	-0.36676341	2.1183E-05
CPSF3L	0.35051576	0.00671113	IQGAP2	-0.36663759	0.0012328
CHP1	0.35046474	0.00117216	MEF2A	-0.36541256	0.00608694
IDH3G	0.3502215	0.00056673	RSBN1L	-0.36463016	0.01741942
PRMT1	0.34976569	0.00317269	SOD1	-0.36438963	1.1889E-05
DNAJB12	0.34947315	0.01415392	AHCY	-0.36365282	0.00832521
SRM	0.34885278	0.0454458	XPO6	-0.36354453	0.04533985
EID1	0.34864689	7.5389E-08	RBM47	-0.36346797	0.01575559
LUC7L3	0.34821994	0.00227453	RRAGA	-0.36252312	0.00387134
GPR183	0.34781059	0.00655269	DNASE1L1	-0.36184828	0.04613847
COPS2	0.34737456	0.00210305	P4HB	-0.35835561	5.2742E-06
SESTD1	0.34733821	0.04152492	RPS27L	-0.35531484	9.7836E-06
SNRPE	0.34729563	0.00438164	SLC25A39	-0.3552676	0.00016291
PSMA2	0.34719673	0.00025672	MVB12A	-0.35515376	0.02648255
EWSR1	0.34636742	0.00066962	SGK1	-0.35500654	0.00611115
SHKBP1	0.34620018	0.00107588	NUCB1	-0.35476086	0.00023167
DDT	0.34593978	2.3396E-05	ORAI3	-0.3538661	0.03948803
IK	0.34593565	0.00912579	COA3	-0.35283422	0.00322449
BCL2L11	0.3454046	0.0383285	CISD1	-0.35198115	0.01372682
ALKBH7	0.34430711	0.00243365	SLC39A1	-0.35003536	0.00461187
SERINC3	0.34393674	0.02695572	C1GALT1	-0.34976187	0.0390549
CTBP1	0.34383107	0.00816271	PIK3C2A	-0.34855725	0.02538283
EXOSC5	0.34262437	0.03674262	CCDC28A	-0.34850544	0.03060027
CCDC88A	0.34244392	0.00107025	TBC1D9B	-0.34810584	0.0487327
NSD1	0.34230719	0.00921436	GSAP	-0.34768205	0.03262265
LYRM4	0.3420881	0.01714777	TMEM208	-0.34629268	0.0031943
CDC42	0.34208564	1.8569E-10	MAX	-0.34626116	0.00689319
RPS28	0.34193303	8.5318E-11	LMNA	-0.3454736	0.0068418
TPM3	0.3416555	7.7115E-08	PRKCA	-0.34505983	0.03504203
ARPC3	0.34148406	1.2192E-05	LINC00116	-0.3448021	0.040591
ANAPC5	0.34109806	0.00258142	TCEAL4	-0.3444956	0.00020863
TMEM131	0.34046197	0.00871886	TSC22D3	-0.34426839	0.02507239
CDKN1A	0.3404611	0.02210372	CYBB	-0.34302652	0.00294648
HMGB1	0.34035682	1.0088E-08	RTN3	-0.34217902	0.00061696
DOCK8	0.34008897	0.01591703	SFT2D1	-0.34201131	0.00021529
YY1	0.33992593	0.00032257	MRPL40	-0.34191522	0.00071929
PDCD2	0.33892364	0.0104367	DYNLL1	-0.34164287	0.01354441
VPS13C	0.33880668	0.00844373	RHBDD2	-0.34142263	0.03374005
DCTN3	0.3385713	0.01308577	SDCCAG8	-0.34067742	0.01365814
NDUFS6	0.33817402	0.00017846	SFMBT2	-0.33865525	0.0067931
VASP	0.33786773	0.01421165	DAD1	-0.33806876	6.154E-09
MSRA	0.33745258	0.01502239	ACAT1	-0.33671338	0.01916722
DIAPH1	0.33581124	0.01107254	TNPO1	-0.33662628	0.0051826
ZNF655	0.3358065	0.00666226	RPF2	-0.33652302	0.03570883

EIF4EBP2	0.3347551	0.00859709	FBXL15	-0.33595156	0.03490005
DUT	0.33452428	0.01068683	STARD3	-0.33574293	0.02068966
POLR2J3	0.33438742	0.00915863	DNMT1	-0.33384586	0.02507239
ITPRIP	0.33385081	0.03203912	DPM2	-0.33261427	0.03761216
TRAPPC1	0.33344561	0.00165143	SDCBP	-0.33244753	0.00062101
PSMD9	0.33316064	0.00368195	LMAN2	-0.33152829	1.4342E-05
STX8	0.33275278	0.00159612	MRPS10	-0.33057519	0.01414236
NDUFV1	0.33245798	9.5623E-05	IL10RB	-0.33041775	0.00510991
USP16	0.33227273	0.00438193	ASMTL	-0.33001491	0.03021732
CPSF6	0.33202936	0.03208823	HSP90B1	-0.32925689	1.4448E-06
SPINT2	0.33197917	0.00720647	CYSTM1	-0.32916854	0.00037201
TUBGCP2	0.3317267	0.01275928	MAP1LC3B	-0.32808432	0.00086453
RALY	0.33152897	0.0015308	RPN1	-0.32772884	0.00026769
PDHB	0.33103582	0.00794645	LITAF	-0.32631623	0.01343878
BORCS7	0.33067445	0.0220395	GNPTG	-0.32526467	0.01893977
MAPKAPK3	0.33054134	0.01792236	ESF1	-0.32498643	0.03584384
MAP2K2	0.33042	0.0079517	FAM110A	-0.3238487	0.00575612
MRPL11	0.33038788	0.00192485	IRF2	-0.32368804	0.03330806
RCN1	0.32985885	0.04483757	EDEM2	-0.32321322	0.02363791
TATDN1	0.32934774	0.01666162	MRPL34	-0.32320042	0.01185088
GLRX3	0.32900106	0.00592893	PAPD4	-0.32197331	0.0458489
WTAP	0.32890145	0.00227951	TEX264	-0.32132772	0.00310955
CHCHD2	0.32867386	8.5247E-08	PLEKHA2	-0.32054179	0.02639363
LINC00152	0.32864273	0.03163448	NFKBIE	-0.31986784	0.01147791
ATF6B	0.32846292	0.04849741	RPS19BP1	-0.31951546	0.00641501
FBXO33	0.3277936	0.02940817	SMIM7	-0.31934217	0.00714356
HNRNPK	0.32776896	2.8289E-08	ARL8A	-0.31870373	0.00842689
NDUFA6	0.32760446	5.3774E-05	HCK	-0.31744437	3.3208E-05
C10orf54	0.32655092	0.00100817	C17orf62	-0.31632723	0.0057598
FKBP1A	0.32566596	0.00010768	RAB10	-0.31621115	0.00216477
RPL36AL	0.32563407	4.8159E-05	SCAMP2	-0.31430727	0.00120717
ADRBK1	0.32551345	0.02842723	TGFBR1	-0.31321092	0.03690374
SNRPD1	0.3254076	0.0024357	GTF2H5	-0.31199224	0.00284233
FUBP1	0.32527714	0.01468817	CALM3	-0.31171145	0.02115155
PPIA	0.32514266	6.4101E-14	LCP2	-0.31126171	0.00662544
SRSF3	0.32465674	1.5535E-05	RPN2	-0.3110439	0.00333501
TMEM87A	0.32372421	0.04036188	MYO9B	-0.31087849	0.02043446
CDC26	0.32229225	0.01415392	RTCB	-0.30883114	0.00736057
SEPT7	0.32203176	0.00011015	VOPP1	-0.30840987	0.00036875
NDUFS8	0.32201817	0.00055689	PHACTR2	-0.30830878	0.02260761
FIBP	0.32120548	0.00118034	LGALS1	-0.30652979	0.00825666
ZEB2	0.32103135	0.01062211	DNAJB6	-0.30621782	1.9677E-05
PRPF38A	0.32099925	0.01851852	CD164	-0.30434346	0.0011111
EIF2AK1	0.3208271	0.01873546	TYROBP	-0.30406043	1.2561E-06
RNMT	0.32078554	1.6635E-05	ZNF22	-0.30378967	0.03912439
YWHAZ	0.3205304	8.9576E-07	FKBP2	-0.30370224	0.00032203

KHDRBS1	0.32024792	5.0389E-05	FUOM	-0.30287495	0.00027782
HUWE1	0.31962742	0.02719096	DEGS1	-0.30238887	0.0033354
BZW2	0.31954063	0.01303256	USO1	-0.30185206	0.02599871
KDM6B	0.31939971	0.01421808	NARS	-0.30172292	0.01228252
LDHB	0.3189977	0.00137177	RHEB	-0.30062463	0.00044918
ITGB2	0.3187848	0.0007531	SERTAD1	-0.29925718	0.00124506
RNASEH2B	0.3185079	0.01132737	SEC61A1	-0.29864405	0.01911844
PFDN4	0.31815793	0.00631657	LRPAP1	-0.29792445	0.00837327
TMA16	0.31805952	0.02158735	UCP2	-0.29759262	0.01634644
ZSCAN16-AS1	0.31713522	0.04497818	FUCA2	-0.29721653	0.00995563
FAM162A	0.31705213	0.00199938	KDELR2	-0.29471967	0.04925674
GOLGB1	0.31645803	0.04115575	REEP3	-0.29448796	0.03236303
HNRNPH3	0.31643253	0.00268892	RAB14	-0.29279914	0.00276477
CAST	0.31633985	0.00533415	LASP1	-0.29259456	0.04276325
CST3	0.31625437	8.4965E-05	CNDP2	-0.29257876	0.00051147
MZT2B	0.31619477	0.00438461	ACAA2	-0.29022519	0.03538272
TROVE2	0.31581195	0.0062724	EIF2S1	-0.29004914	0.01682308
HSPB11	0.31359654	0.02135546	SPG20	-0.28958712	0.02028103
IAH1	0.31340123	0.00166371	NOP10	-0.28911313	0.00167304
ATP5F1	0.31322715	1.7315E-05	DYNLT3	-0.28503785	0.03132756
SON	0.31316415	7.0716E-06	DDRGK1	-0.28381255	0.01734675
CCT2	0.31315963	0.00335445	MAGT1	-0.28362141	0.01268443
CCDC107	0.3127712	0.00393738	LAPTM5	-0.28186872	8.158E-05
RAB8B	0.31255379	0.00392292	SDF2L1	-0.27997604	0.01385899
TSPAN3	0.31239172	0.01872735	SQSTM1	-0.27923461	0.01948067
EIF4H	0.31165845	0.00051656	OSTF1	-0.27849875	0.00325689
NDUFB4	0.31160962	0.0001466	UNC93B1	-0.27844246	0.04440551
AOAH	0.31156565	0.00069559	CD4	-0.27675886	0.00045708
PSMA4	0.31094587	0.00208695	CEP170	-0.27487667	0.02822192
ARL4A	0.31083345	0.04505146	G3BP1	-0.27370474	0.0028064
AKR7A2	0.31066873	0.0289628	RNASET2	-0.27286762	0.03095614
FAM204A	0.31014291	0.01652888	AMD1	-0.27175469	0.00545222
ATP5B	0.31011077	0.00046174	GALNT1	-0.26728619	0.02029233
MDM4	0.30998049	0.00452892	UQCR10	-0.26483601	0.00286019
ENSA	0.30971523	0.00044877	PHF3	-0.26482612	0.02028905
ARID4B	0.30857256	0.01002816	EVL	-0.26441987	0.02057842
HDGF	0.30818163	0.01948067	FAM50A	-0.26275985	0.02766062
MAPK1IP1L	0.30809983	0.00024127	LAMTOR3	-0.2624332	0.03743532
PRELID1	0.30779639	0.02115155	C6orf62	-0.26240128	0.00863778
SRRM2	0.30751721	2.1189E-05	DPY30	-0.2621915	0.0243521
TBC1D5	0.30740862	0.02682993	FCGR2A	-0.26083207	0.02694094
SNRPA1	0.30708933	0.03759367	HSBP1	-0.25893637	0.02444496
RBM5	0.30681332	0.04161901	TPM4	-0.25516994	0.007388
RBM25	0.3064693	2.1664E-06	IDI1	-0.25512323	0.02759048
CPNE3	0.3054626	0.01941882	PTGES3	-0.25414902	0.00644452
SH3TC1	0.30541902	0.03777425	MGST2	-0.25046183	0.0309046

SNHG8	0.30520794	7.6787E-05	SERINC1	-0.24934979	0.02802013
TMEM50B	0.30513112	0.04435395	ZNF706	-0.24818437	0.00053978
EIF5A	0.30513064	0.00097925	FNIP2	-0.24811471	0.03077789
IFITM3	0.30496362	0.01847281	ACADVL	-0.24402966	0.0192214
SNU13	0.30462519	5.736E-05	ARL6IP1	-0.2407854	0.00114499
PARP14	0.30461578	0.01082562	WIPF1	-0.23486206	0.00857899
C14orf2	0.30442257	5.1485E-05	CANX	-0.23160568	0.0017719
HNRNPH2	0.30334494	0.00354658	YWHAG	-0.23154377	0.03170464
SIVA1	0.30328333	0.00155747	BSG	-0.22993086	0.0131298
ATG3	0.30325229	0.01617914	NCKAP1L	-0.22941154	0.02232097
CDC42SE1	0.30305715	0.0206453	PMVK	-0.22851927	0.04047104
NCL	0.30302928	5.0795E-08	ADAM17	-0.22824132	0.04676876
PRPF4B	0.30273458	0.01393276	MRPL57	-0.22715907	0.02562006
DCXR	0.30261565	0.03319594	TGOLN2	-0.22622554	0.03794636
ENO1	0.30180478	0.0089751	SPCS2	-0.22383428	0.0005274
BCLAF1	0.30158437	2.3537E-05	STX7	-0.22341311	0.04960081
SSR1	0.29958361	0.00017742	S100A11	-0.22280128	0.01296684
TOMM7	0.2991079	1.8732E-06	ADIPOR1	-0.2196328	0.03949903
UBE2V2	0.29881176	0.01891636	GLRX	-0.21955367	0.03306853
YWHAQ	0.29876694	0.00813335	QKI	-0.21676296	0.0373833
DYNLT1	0.29819305	0.00525717	B2M	-0.21409631	0.00171354
C4orf3	0.29806684	3.119E-05	MDH1	-0.2113656	0.0091707
PPP1R7	0.29803569	0.00305871	TACC1	-0.20868268	0.0486043
ACTG1	0.29802847	0.00022414	CTSS	-0.2050297	0.03794636
FNBP4	0.29779719	0.0132119	NDFIP1	-0.20501698	0.00512839
SLC6A6	0.29708758	0.03101123	DBNL	-0.2026928	0.02891901
RBM6	0.29694827	0.02570506	ATP6V0B	-0.20010423	0.00863529
WDR83OS	0.29576075	2.2576E-05	SYNGR2	-0.19266285	0.0484141
TBCB	0.29544081	0.00364191	PFDN2	-0.19209049	0.0433786
AKAP9	0.29539369	0.02525607	GSTO1	-0.19186347	0.02507239
SMNDC1	0.29502225	0.03331524	DYNLRB1	-0.19056945	0.02066099
RAB22A	0.29474289	0.04210535	SEC62	-0.18416276	0.00240875
PACSIN2	0.29343983	0.04835388	CYBA	-0.17690028	0.01564898
MRPL23	0.29335758	0.00729869	RSRC2	-0.16452291	0.02837968
PKM	0.29296033	0.00550555	VAMP8	-0.15974901	0.00137703
PSMD2	0.2927393	0.00156593	RHOA	-0.12497473	0.04592671
HSPA9	0.2924638	0.00058774			
SELK	0.29226935	0.0004542			
YWHAB	0.2921393	1.5993E-07			
TIMM17A	0.29139588	0.01986103			
MAGOH	0.29087	0.00291861			
RNASE6	0.28977007	0.01377964			
H3F3B	0.28973592	5.0389E-05			
OTUB1	0.2889905	0.03024111			
H2AFY	0.28882589	0.01509939			
CNIH1	0.28802372	0.00622719			

ISG15	0.28757642	0.04580837
PARK7	0.28739033	0.00110812
HNRNPDL	0.28734609	5.9574E-08
TRA2A	0.28711093	0.00141518
SNRPB2	0.28663437	5.3356E-05
WDR61	0.28647914	0.02622149
NDUFA11	0.28610404	2.626E-05
DHX9	0.28560262	0.02625228
KATNBL1	0.28473727	0.03846091
VPS36	0.28435114	0.02276978
NRDC	0.28398588	0.03249783
FLII	0.28204637	0.0167914
COMMD8	0.28143199	0.02778868
NONO	0.28107457	0.00048987
RAB5A	0.28018133	0.01688948
HLA-F	0.27999884	0.03708349
TMEM167A	0.27990707	0.00680914
TAF9	0.27970723	0.03446719
EIF1B	0.27969016	0.00459557
AAED1	0.27908923	0.04607781
COMMD7	0.27881714	0.01789325
SRA1	0.27870775	0.0023623
COX7C	0.27830481	3.5744E-08
PYCARD	0.27685534	0.00928207
GNAI3	0.27583114	0.00204526
NAA38	0.27509878	0.01344281
C14orf166	0.27495226	0.00095199
ARPC5	0.27481352	0.00074956
HMGN3	0.27466235	0.0061126
TMEM243	0.27432793	0.01496206
ERH	0.27384832	0.00046533
YTHDC1	0.2736392	0.01572493
XIST	0.27317308	0.01258279
HLA-DRA	0.27312112	0.00584268
PSMD12	0.27225248	0.01131937
DNAJC15	0.27156097	0.00111448
RBBP7	0.27044559	0.04801693
NDUFB11	0.27041929	0.00019772
PSMG2	0.26967178	0.00467636
PRDM2	0.26932513	0.03758727
TPR	0.26924556	0.00072453
OSBPL8	0.26915096	0.02274048
ZC3H15	0.26897831	0.00061669
USF2	0.26895713	0.04006776
ATP5J2	0.26804146	0.00235784
DNAJA2	0.26790311	0.00336117

PRKAR1A	0.26733436	0.01075762
USP36	0.26680433	0.0219493
BLOC1S6	0.26677744	0.00320011
ATRX	0.26637131	0.0111793
UBE2R2	0.26596587	0.01415392
UBE2A	0.26590963	0.00916433
CCDC59	0.26519576	0.00104805
BOD1L1	0.26477681	0.00482027
SMU1	0.26474755	0.03335881
NDUFB1	0.26422469	0.00112421
UQCRH	0.26255506	2.4454E-06
COPE	0.26163271	0.00915863
SRSF10	0.26132087	0.01035671
RAB4A	0.26118135	0.0242304
PDAP1	0.26067833	0.01365905
PSMA1	0.26034871	0.00252266
PGAM1	0.26018927	0.02312338
SEC11A	0.26015062	0.00084619
PNRC2	0.25975949	0.00231531
NAMPT	0.25936313	0.03017442
GLRX5	0.25894146	0.03725538
PDCD6	0.25871528	0.02678615
PFN1	0.25788818	0.00021317
AIMP1	0.25762468	0.01059587
SYNCRIP	0.2575941	0.00283053
UQCRB	0.25715496	2.526E-08
NDUFB10	0.25675486	0.00588837
NUMB	0.25613704	0.01904028
ACTR2	0.2561042	1.702E-07
MPC2	0.25609568	0.02720322
TMEM256	0.25543506	0.00094548
LSM1	0.25439723	0.02900027
RANBP1	0.25364532	0.03389203
PRPF40A	0.25276314	0.00132705
UBE2E2	0.25250019	0.03047108
FAM133B	0.25218161	0.0003794
CIB1	0.25192034	0.03033487
APPL1	0.25085707	0.0484785
XBP1	0.24989321	0.00539787
C11orf73	0.24951593	0.01986103
WBSCR22	0.24912037	0.02697486
LSM14A	0.24880088	0.0219493
ACP1	0.24776767	0.01583958
GLO1	0.24769919	0.04045054
SERBP1	0.24766224	0.00033153
MRPS36	0.24758519	0.03746501

RAB8A	0.24641134	0.00443367
SRP9	0.24576374	0.02953675
DGUOK	0.24354497	0.03577855
VDAC2	0.24335463	0.02310217
TBCA	0.24311362	0.00836034
MRPS15	0.24302721	0.02679833
TPI1	0.24301509	0.00107316
ZFAS1	0.24162755	0.02417636
HNRNPUL1	0.24135576	0.00220144
VMP1	0.24125062	0.0312387
LSM3	0.24076523	0.00195468
ARHGDIB	0.24046161	0.00490247
GNB1	0.24042412	0.0185305
SUB1	0.23896559	0.00149506
C9orf78	0.23848932	0.00813335
SPCS1	0.23808502	0.00161703
MKNK2	0.23754382	0.04158943
ATP5E	0.23712089	6.5607E-05
CFL1	0.23709491	0.00063605
COX17	0.23695534	0.0067931
AP3S1	0.23617172	0.02197963
VPS29	0.23557241	0.00813335
MORF4L1	0.23549839	1.1404E-05
NDUFS4	0.23497808	0.02565036
SCO2	0.23345714	0.03266226
NOL7	0.23322691	0.04347713
POLR2J	0.23304307	0.03453371
PGK1	0.23259851	0.00728573
EIF3A	0.23163675	0.00019562
TOMM20	0.23111248	0.00568429
SRPRA	0.23059238	0.04990912
MRPL14	0.23003141	0.03091015
ATP1A1	0.22999555	0.01058968
IFNGR1	0.22998092	0.03798567
TPT1	0.22965175	5.9409E-05
UBE2D3	0.22949492	0.00355164
TOP1	0.2288679	0.00234496
CLEC2B	0.22863957	0.04170719
TXNL4A	0.22851122	0.04535975
UBXN1	0.22702442	0.00499509
SNRPG	0.22664361	0.00853305
COQ10B	0.22651761	0.04851503
PAPOLA	0.22645791	0.0006397
GPX1	0.2261259	0.00529663
CD53	0.22494902	0.00502931
SEC13	0.22460353	0.04593486

NDUFA2	0.22353711	0.00258837
PPP1CA	0.22338688	0.03382868
SAR1A	0.22309988	0.04513799
DHX36	0.22253751	0.03181337
PSMB3	0.22213841	0.01216845
TMEM258	0.222006	0.00336258
PCBP1	0.22163838	0.00135205
OSTC	0.22105595	0.02642147
NDUFA12	0.21964037	0.00729869
CSDE1	0.21738569	0.00946263
SKP1	0.21719627	0.00202726
PSMD4	0.21478127	0.04439514
CTDNEP1	0.21378718	0.02479002
NPM1	0.21257517	7.0239E-06
ARPP19	0.21233616	0.04726751
NDUFB5	0.21202023	0.00811994
ETFA	0.21124974	0.01849725
TAPBP	0.20900469	0.00647032
PPP1R2	0.20858709	0.01624783
FKBP8	0.20843287	0.01667779
HADHB	0.20763444	0.04164189
FYTTD1	0.20690148	0.04966702
DDX5	0.20652776	0.0460127
ARF1	0.20540644	0.00502931
MBNL1	0.20458515	0.02317203
ATP5C1	0.20425657	0.01630627
VDAC1	0.20331238	0.01152816
UQCRC1	0.20324801	0.04295042
MAP3K2	0.20251398	0.02541107
CYCS	0.2019373	0.0247732
DNAJC7	0.20167228	0.01844639
PTMA	0.19993686	9.3635E-06
PA2G4	0.19896314	0.0187585
HNRNPA3	0.1980637	0.00696338
FIS1	0.19364564	0.02276485
UBE2L3	0.19193226	0.03334046
CALM2	0.19192509	0.00022215
MYL6	0.19128188	0.00624747
RNH1	0.18764244	0.02630787
XRCC5	0.18721207	0.01232287
GNG5	0.18712455	0.0236605
COX7B	0.18607936	0.01404677
COX6B1	0.18358627	0.00182837
ATP5G3	0.18053363	0.00958165
SSR2	0.18029177	0.04439702
CAPZA1	0.17982928	0.01445784

SRSF11	0.1795517	0.04363726
ATP5O	0.17940214	0.01578499
RTFDC1	0.1753133	0.04358853
COX7A2	0.17265989	0.04226056
PSMB1	0.17248385	0.02541107
RHOQ	0.17149073	0.04511311
YWHAE	0.17021494	0.00681428
OST4	0.16848499	0.02520646
SEC61B	0.16771997	0.04850133
SF3B1	0.16592643	0.04772289
CIRBP	0.16331109	0.00432826
MTDH	0.1631911	0.02676799
GNAS	0.16286726	0.03793832
YBX1	0.16214748	0.00628713
PSMA7	0.1561795	0.03006006
ARPC2	0.15346195	0.02207565
SAP18	0.13048065	0.04885262

List of shared cDC3 DEGs
CD2
CXCL11
UPK3A
SYTL1
CES1
CD1C
MPP7
FCN1
HSH2D
CXCL9
APOBEC3A
S100A12
FYN
CD52
VCAN
CD207
PFKP
FAM65B
IDO1
LIPN
ADAM8
PLAC8
SLC38A1
MTMR11
TMEM8B
UBD
ENHO
ARNTL2
ICAM3
HACD1
QPCT
TMEM154
CRLF2
FLT3
CCR7
IL18R1
ABHD17C
FAM134B
CXCR3
CST7
SLC22A4
MGST1
SH3BP4

FCER1A
SLAMF9
SULT1B1
NEGR1
LAYN
KBTBD11
ETV7
BCL11A
TFPI
CTD-2006K23.1
VDR
SPIB
FGR
GPR157
IL1R2
BASP1
LMNB1
SELL
S100A6
CFP
RAMP1
TYMS
GALNT3
TUBA4A
CHST2
TNFSF14
WEE1
TMEM217
GPER1
MCEMP1
TOP1MT
LILRA2
HPGD
LSP1
ANPEP
SMAD3
PLP2
HAS1
SLC35F2
G0S2
LGALS2
GPAT3
ZNRF1
RALA
ASGR2
FAS

IFITM1
AC013461.1
WARS
ADGRE3
NOV
UTF1
SLCO4A1
FLNA
SDPR
CCL22
PPA1
TRMT6
ITGAX
MCOLN2
NKX3-1
SYN1
PRDM8
CD300E
AIM1
LIMD2
SGMS2
RHOF
KIFC1
S100A4
IL2RG
TREM1
GBP5
C12orf75
GJB2
SH2D3A
LAMB3
FLT1
PDE4D
TUBA1A
EREG
S100A9
AKAP12
MYLPF
SNX20
KIF18A
GLIPR2
LINC01272
XKR8
LINC01315
LINC01588
BANK1

KCNN4
AREG
HAPLN3
KIAA0101
PKP2
BCL2L14
MAP4K4
INHBA
COL9A2
AKR1C3
TK1
IRF4
BZRAP1-AS1
LILRA5
AFF3
SPNS3
VCL
ANKRD28
CAPN2
STK26
CSTA
OCIAD2
IL1R1
RP11-147L13.15
C15orf48
CDKN2D
FAM129A
PPP1R3B
GPR65
SPON2
SH2B2
CORO1A
GPR146
SERPINA1
BEX2
CCND2
AP1S3
CALCRL
MTHFS
CHAF1A
SYTL3
LYPD3
MAP4K1
CASP9
DUSP4
GPRC5A

SLC2A6
TCTN3
S100A10
TAP1
MARCO
CHRNE
AC002456.2
TPPP3
WDR59
FMNL1
TIMP1
FPR1
TMEM158
RAB11FIP1
RAB27A
MYO1G
SYNGR1
C16orf54
TMEM120B
TRAF1
FBP1
SH3BGRL3
PON2
IL1RN
GEMIN4
TNIP2
CBX6
TRAF3IP2
BCL7A
KLHL25
MALT1
NRARP
PDLIM1
CDK2AP1
TSPO
TRIM25
PTX3
PTGIR
HOTAIRM1
CD48
GK
LDHA
ISG20
SMCR5
GFOD1
AGO2

STK4
LINC00152
CD37
ACAA1
PSME2
PDCL3
BORCS5
IRF9
BID
EMP1
CKLF
HMGA1
STRAP
CLEC10A
IGFBP7
SEPT6
R3HDM4
MX2
PARVG
LTA4H
SQLE
SATB1
C1orf162
TRAF4
PSMB9
CENPW
RP5-1171I10.5
PDE2A
PTK2B
RIPK2
PTTG1
SLC2A3
VIM-AS1
HDAC1
LRRFIP1
PKM
LY6K
ENO1
GBP1
CEP83
COTL1
CTDP1
TET2
PRELID1
SLC16A10
SPTLC2

NDEL1
ACSL5
LHFP
SNRNP25
OPN3
SLC25A19
AC016831.7
CYTIP
SLC7A1
REV3L
MBOAT7
SIPA1L1
NLRC5
HOXA5
LINC00528
EIF2AK4
IFITM2
HGSNAT
LILRB3
ZNF487
IFT57
TAP2
AFMID
GSTP1
INSR
CCDC12
TRAPPC1
SERPINB1
LRRFIP2
SNRPF
TTC19
CRIP1
PSTPIP2
TES
CREM
LUCAT1
RFTN1
GAPDH
HLA-DQB1
C14orf80
IL16
ZC3HAV1
PRMT1
GNG7
ALYREF
CCDC6

CSF3R
NIPSNAP3A
MAP2K1
IMPDH2
CD86
TLE1
NAA10
SLC25A51
CIB1
MTMR9
CD55
SFT2D3
MIR181A1HG
HSD17B10
FDPS
HEBP2
TRABD
TAF10
KYNU
REC8
PSMB10
ISG15
C1QBP
RPL5
DYNLT1
SAMSN1
ARF5
PTRHD1
EMP3
RPL10A
CD7
RPL4
HMGN1
GABARAPL2
VIM
JARID2
GHITM
DCPS
MIR3945HG
EIF5A
CLIC1
RPL18A
ADA
TMSB10
DNMBP
NCF2

PDE4A
RPL8
ILF2
C11orf1
RPS7
RPL6
BCR
AEN
SBNO1
NFAT5
FXYD5
KCTD20
ALDH2
PTS
EIF3I
ZBTB43
SNRPB
PSMD2
PARK7
ATP5G1
FDFT1
GBP4
LSM7
PTP4A2
ACTB
RPS18
TNFAIP8
RP11-138A9.2
WDR43
PTPN1
ADSS
PSMA2
MPHOSPH6
TXNL4A
MYL12A
ACOT9
METRNL
FBL
AMPD2
EIF3L
GLRX3
MOB3A
OXSR1
GNA12
PLSCR1
DGKD

C19orf38
PSME1
ZNF703
FURIN
NDUFS8
GBP2
ATP5A1
RPL3
GNB2L1
ADSL
BZW2
LAP3
RUNX3
APRT
SUPT4H1
TP53BP2
ABT1
NDUFA7
GTPBP4
FIBP
CEP68
RPS5
PLCXD1
RPSA
CYP2S1
ADD3
KCNAB2
UBE2I
RPL29
SDE2
FBXO34
TKT
EIF3M
PFN1
HINT1
DECR1
MDH2
RPL32
CD58
COMMD6
PGK1
SIK3
NDUFA6
RAN
RPL31
RPL11

NUDT1
RPL37
ATP5B
RNH1
UBE2F
FAM49A
PSMA4
ARPC1B
CCT8
TSHZ1
TFDP1
CDC37
HADHA
EXOSC6
SMDT1
EIF3K
BRK1
RAD51C
USP3
LSM5
SLC25A5
RPL19
RPS14
RBM8A
PRKAR2B
RPS2
FAM43A
OLA1
NSMCE3
UQCRFS1
TBC1D8
RNF7
RPLP0
RPL7A
SPRED2
RANBP1
NACA
RPS21
DAZAP1
EEF1B2
FKBP1A
RPS19
RPS4X
HNRNPAB
ISOC1
H3F3A

HPRT1
EDF1
CCNDBP1
GNA15
RPL14
SNRPD1
CTNNBIP1
SERPINB8
CFL1
XRCC6
POR
PNO1
MTHFD2L
CHPT1
PTPRC
TBCB
RPS8
MSN
CHCHD2
CPNE1
ATP5G3
EZR
H2AFY
PIM2
PSMB3
INSIG1
SEC61B
THBD
RPL23A
CCT6A
RBM7
HIVEP1
FBXO33
ZNF511
BTF3
CDC42SE2
PSMB1
RPL36A
CCDC109B
TPI1
RPL35A
RPS24
PRKDC
LDHB
PSMA1
MYL6

RPS10
SLC25A6
RPS15
RPL15
RP11-468E2.4
PRCP
RPL37A
RPL27
RPS12
ACTG1
SNRPG
SNRPE
RPS15A
RPS3
EIF3F
GTF3A
RPS23
HIP1
MED4
RPS29
RPL26
RPL18
MTPN
CCZ1
RNPS1
SLC25A3
RNF139
RPS16
CHML
ERH
KPNA2
RPL28
ATP5J2
GALK1
FADS3
RPS11
RPS3A
RAB22A
ATP5F1
SMARCC1
THOC7
RCC2
TMA7
ARPC5
SNHG15
TUFM

DCTN3
UBE2N
RPS27
STXBP2
ENSA
PCIF1
RPL10
IDH3G
SET
RPS17
TPM3
WAS
COX7B
RASSF5
ATG3
UBE2L3
SEC13
SNRPA1
OSTC
RPL36
DDHD1
MBD2
ZNF394
AKIRIN1
AKAP13
RAP1B
HNRNPA0
MED28
EIF3E
SYAP1
RPL41
CASP1
SMG1
RPS6
RPL30
PPP1CA
CD44
PAPD5
PSMD9
COX7A2L
RPLP2
MRPL11
TOP3A
RPS9
NOP58
FAM162A

WBSCR22
ARF3
COPE
CHCHD3
GSTK1
DDX21
ARID5A
SNRPD3
AP1S2
UBA52
PLK2
TOMM22
LDLRAD4
TMEM167A
COX7A2
FOSL1
ATXN10
ARPC3
EMD
PSMD12
MRPS15
LSM3
RPL24
ATP2B1
ARF6
BZW1
RPL34
ANKRD11
MPC2
EIF3G
PMPCB
HNRNPR
LILRB2
C7orf73
PSMA7
ARPC2
SNU13
B4GALT5
PPP1R7
ETFA
VDAC1
PCGF5
EDEM1
SUB1
EEF1A1
GPBP1

TRAPPC10
XRN2
HLA-DPB1
RBM3
TMEM120A
RILPL2
C8orf59
N4BP2
RPL35
HNRNPM
ATP5L
C14orf2
NEDD9
NDUFS6
SLC36A4
TUBGCP2
MRPL3
PILRA
EIF2A
GMFG
RPL13
GABARAPL1
UFC1
STK10
SMARCE1
BCL2
FAU
GABPB1
DNAJC15
CSNK1D
RPS25
RPL39
BTG1
NDUFA12
ACTN4
GLIPR1
SRA1
TIMM17A
COX4I1
IDH3B
RPS13
NETO2
PTPRE
ATP5C1
EIF3H
CSF2RA

ANKLE2
PTPN2
CHRAC1
GNG5
PRELID3B
NDUFA2
ACSL1
UXT
NDUFB4
RASSF3
FBXW7
MTHFR
HMGB2
RPL22
MAP3K8
RPL7
RSL1D1
UBE2D2
PSMA6
RPL17
SRP72
DDT
PQBP1
TOB1
DERA
RPS26
BRE
DEK
RP11-76E17.3
C9orf72
EMILIN2
BACH1
JAK1
MAP2K2
CHST11
ANP32B
RPL27A
DNAJA2
RPL36AL
C14orf166
DPM1
ELOVL5
HNRNPA1
GPAT4
SLCO3A1
MZT2B

UBE2A
DNAJB12
SPCS1
RNF168
TNFSF13B
MAGOH
HIGD2A
SMAP2
SLC31A2
MALAT1
CD47
ZNF277
SNRPB2
PLAUR
CDK16
MTHFD2
EEF2
TMEM50B
SRPRA
PGLS
SIVA1
RPL9
PNPLA8
TMEM258
PFDN5
COX17
SYF2
UBA2
SSR2
CRK
MRPL23
PPIA
RSL24D1
RPL13A
ATP5E
RPL12
PCNP
COX6B1
TNFRSF10B
OXA1L
RPL38
HNRNPD
CCDC107
POLR3E
HNRNPH2
GPI

VDAC2
CCT2
STAT3
RPLP1
OST4
ATXN2L
RPL21
RPS27A
PPP1CB
RBM15B
SERBP1
SERP1
MRPS36
SARAF
BCL2L11
RNF145
AP3S1
CDC42
EIF3J
NUP98
LYRM4
NDUFA10
CD72
ARL5A
NOL7
C16orf13
SH3BGRL
DDX6
CAT
DNTTIP2
NDUFB11
SLU7
MXD1
TGIF2
PHF20
TUBB4B
COX7C
ATP5G2
MECP2
REL
PRRC2C
HLA-DRA
SRRM1
PAIP2
UQCRH
UQCRC1

JOSD1
ATP5O
GPCPD1
CSGALNACT2
NDUFV1
CHD1
IGBP1
SRSF2
ARF1
YBX1
POLR1D
TROVE2
OSBPL8
DDX27
OTUD5
C19orf43
ENY2
NR1H2
ZC3H15
EIF2S3
TCF25
CNIH1
GLTSCR2
EIF3D
THEMIS2
C9orf78
SLC35E1
PPP1R2
MAPK1IP1L
YWHAB
DNAJC7
PRKAR1A
EEF1D
SMCHD1
BCL7B
KHDRBS1
CAPZA1
COQ10B
IK
C1orf56
COPS2
HMGB1
ILF3
TRMT1
HNRNPK
VASP

CMTM6
RTFDC1
IAH1
RQCD1
TAF9
RBM4
SCO2
MRPL14
CCNH
MORF4L1
KLF3
EIF3A
TBCA
ARHGEF1
CLEC2B
NPM1
AAED1
TNFRSF1B
CCNY
KMT2E
NSA2
PHF10
ELF2
SH3KBP1
CCNI
JMJD1C
HCLS1
PCBP1
DHX9
NDUFA11
HMGN3
EWSR1
CCDC59
CSNK1A1
GNB1
RPL23
SAP18
PCBP2
HNRNPA3
ACTR2
IKZF1
LINC-PINT
MTDH
SRSF6
UBE2R2
PRPF40A

BRD4
SAR1A
SYNCRIP
SSR1
RBM39
RPS20
PAPOLA
PPIF
GNAS
HNRNPDL
TGIF1
ARPP19
MAP3K2
EIF4H
SKP1
ROCK1
RAB8B
NCL
TAPBP
RPS28
BCLAF1
YWHAE
TOMM20
PABPC1
UBXN1
CMPK1
PTMA
RNMT
UQCRB
SON
TOP1
EIF1

Table S7

			cDC1		
**Ileum DEGs**	**avg_logFC**	**adj. p-value**	**Colon DEGs**	**avg_logFC**	**adj. p-value**
MSMO1	2.08915352	0.02683111	NID1	-3.65508945	0.01183912
CFI	1.77855742	0.00314152	CCR7	-2.71200123	1.2348E-05
TSPAN6	1.65943151	0.0252546	FKBP4	-2.38383859	0.01460376
CD1D	1.58109881	0.0019547	IGLC3	-2.17367978	0.0298789
RNASE6	1.47657878	2.6778E-10	S100B	-1.89094491	0.01521225
SQLE	1.34769668	0.00073408	ABCA1	-1.85757783	0.00118051
PLPP3	1.07128837	0.04797198	TSPO	-1.84687847	8.3623E-06
PLA2G16	1.05475629	0.02940561	MARCKSL1	-1.3141754	1.0029E-07
VMO1	0.99610585	2.6459E-05	FSCN1	-1.21236607	0.03005491
FDFT1	0.95221223	0.00040422	TNFAIP8	-1.21043483	2.6778E-10
IDI1	0.90005452	0.00121884	PDE4DIP	-1.08836664	0.00108363
DUSP5	0.8899376	0.00047077	ZNF267	-1.06924544	0.04132701
TACSTD2	0.87832761	0.04121752	GADD45B	-1.01027285	6.4259E-09
NLRP3	0.75971745	0.04663296	HMOX1	-0.9608054	0.00040422
CDH17	0.75722064	0.01251288	S100A6	-0.94798839	2.5153E-06
PLPP1	0.74592712	0.0200394	EMP3	-0.92651414	0.01537813
TUBA1A	0.67625506	0.01183912	CDC42EP3	-0.91719559	0.00163486
XCR1	0.66510661	0.01537813	TIMP1	-0.9004271	0.03928812
AIF1	0.56700016	0.00011471	CACYBP	-0.87936132	0.03206243
CKLF	0.53413499	0.00249969	TCP1	-0.87652492	0.01629979
ATPIF1	0.52454001	0.04061111	RUNX3	-0.79369988	0.00022862
CST3	0.51330255	0.00121884	LGALS3	-0.76554554	0.00227934
HERPUD1	0.49984546	0.00064602	STMN1	-0.74050158	0.02967229
GPR183	0.49367535	0.01183912	ARL6IP5	-0.71597449	0.00048921
C10orf54	0.48915354	0.04061111	PTGES3	-0.69724826	0.00379213
RNASET2	0.39324273	0.03672774	NOP58	-0.69242669	0.02061684
			SOD2	-0.59894399	0.00618799
			LYZ	-0.59068142	2.3404E-08
			GPBP1	-0.56011138	0.01251288
			S100A10	-0.51731642	0.01629979
			SH3BGRL3	-0.46736655	0.00047057
			H2AFZ	-0.45313221	0.02630947

cDC2					
Ileum DEGs	avg_logFC	adj. p-value	Colon DEGs	avg_logFC	adj. p-value
GATA1	3.24794274	0.01149065	HSPA6	-4.59698752	0.00015977
CFI	2.95132131	0.00010954	CSF1	-4.56354672	0.01103294
SYT2	2.5594171	0.00194676	KCNN4	-4.34224799	0.01583994
ELOVL6	2.41304648	0.01694935	UBD	-3.66095001	0.00025141
LAMP5	2.31441083	2.208E-09	SERPINE1	-3.56647907	0.01179511
DHCR24	2.13220573	0.00235304	FSD1L	-3.46941569	0.02346705
MSMO1	2.05807739	6.6607E-08	HSPA1A	-3.43239183	0.00010531
CEACAM1	2.03773983	6.2869E-07	FUT7	-3.2864369	0.04381358
PPP1R3G	1.89173769	0.04381358	CCL17	-3.28238729	0.00337269
DHCR7	1.6894252	0.0090825	PLA1A	-3.27115127	0.01718615
ATP10A	1.6610204	0.00030941	ENPP4	-3.23645114	0.03230381
LDLR	1.65114957	1.9748E-09	CRYAB	-3.23214891	0.00121015
FADS2	1.64067729	0.03004883	NEURL3	-3.13293542	0.02047642
CLEC11A	1.63031865	1.2577E-09	HSPA1B	-3.12410602	0.00161554
RAPGEF4	1.44415399	2.208E-09	NT5DC2	-2.98929968	0.00464917
PALLD	1.39010783	3.6031E-08	SERPINA1	-2.90394042	6.5728E-14
DAPK2	1.33468876	0.00136392	SLC7A11	-2.8682276	5.9228E-09
GPR162	1.26770848	0.04542368	ULBP2	-2.82904074	0.04468376
PPP1R12B	1.2514457	0.04096815	BAG3	-2.80086498	0.02405
CYSLTR1	1.23546448	0.00028906	LUCAT1	-2.76449897	0.0456382
GAPT	1.16655745	4.2185E-05	PTGIR	-2.63293966	0.00130932
LAIR1	1.14706056	5.5215E-08	S100A9	-2.61330705	0.00165252
FDFT1	1.13513086	7.7219E-13	PTX3	-2.58852709	9.565E-05
C14orf1	1.11387616	1.8238E-05	SMAD3	-2.58784879	0.01868174
HMGCR	1.08763104	0.01647511	SDC4	-2.54043624	0.00288003
POR	1.08433164	1.4698E-06	C17orf96	-2.53740694	0.04708663
TMEM97	1.0807675	0.00035544	IER3	-2.4337373	0.01537253
MYEF2	1.05433799	0.04747104	IL1RN	-2.36914602	4.3158E-08
CD38	1.05051176	0.00898846	TNFRSF9	-2.36275341	0.0246927
AGPAT5	1.03872855	0.00902975	DNAJB1	-2.36175722	0.04096815
KRT18	1.03730839	0.01568863	IL4I1	-2.35373346	1.3472E-09
MPRIP	1.03276148	0.00323431	TSPO	-2.33971455	6.7747E-19
RAB33A	1.03067275	0.01411759	GCNT2	-2.32490702	0.00355292
CD207	1.01177428	1.5442E-19	NRP2	-2.29904642	0.00522824
GSTM4	0.99584764	6.6683E-05	C16orf54	-2.25554979	0.00016575
P2RY8	0.97955369	0.01474397	MUC12	-2.13318631	0.04045246
ADGRE3	0.97668818	0.03980747	FCN1	-2.11271583	0.00024308
STARD4	0.96743273	0.01474397	VCAN	-2.10124692	0.04542368
RP11-545E17.	0.96047967	0.03723978	TRAF1	-2.06968466	2.7492E-09
SQLE	0.95937797	0.0001766	MTSS1	-2.06077285	0.00110076
SLC30A1	0.95293754	0.00695178	MIR155HG	-2.05766637	2.4384E-08
CHRNE	0.95048816	0.00322454	CD300E	-2.03265957	0.04118216
CDH17	0.94807375	0.00442622	DNAJB4	-2.01613314	0.0007501
ABCB1	0.94797284	0.04565019	CCL2	-2.00213395	0.0371824

SREBF2	0.93852518	0.04542368	ADRB2	-1.98968776	0.02021665
TOR2A	0.91681999	0.00112846	SEMA4A	-1.984681	6.7424E-09
IDI1	0.91036247	1.6581E-06	TIFAB	-1.92375931	0.00152567
SC5D	0.87845541	0.02880194	APOE	-1.90992845	0.01814344
LGALS3BP	0.86984064	0.00464917	MFSD2A	-1.90192498	0.04465632
IMPA2	0.85874368	0.00011526	A4GALT	-1.88948606	0.00141714
HIPK2	0.85824986	0.00114052	TNFRSF12A	-1.8815056	0.01140988
DSC2	0.85568986	0.0235693	TNF	-1.87984165	0.00320261
PRAM1	0.85401262	0.01474397	RRAD	-1.84782067	3.5736E-06
TNFSF9	0.83696807	0.02380165	MACC1	-1.83139818	0.02953213
MCCC1	0.81986283	0.00902975	DNAJA4	-1.83019899	0.0296332
ITSN1	0.81618356	0.00480862	RAB13	-1.82451328	0.00212217
PLD4	0.81419348	6.6897E-06	HMOX1	-1.79213138	9.4422E-10
HMGCS1	0.8080807	0.00453195	GPR157	-1.75294511	0.0001435
NUDT19	0.78293885	0.03895917	C15orf48	-1.74437304	6.5728E-14
USF2	0.76192983	0.00031257	ZC3H12C	-1.72851651	0.01537253
CD69	0.75212884	0.01321884	FSCN1	-1.72278471	6.4256E-08
STAC3	0.7436096	0.03813548	GPR35	-1.68909004	3.0616E-08
AFF3	0.73608188	0.01000355	TFEC	-1.68163612	0.0024013
AKNA	0.73366101	0.00015012	TCHH	-1.67216668	0.01583994
VAMP2	0.72156755	0.00209808	HIVEP1	-1.66386496	0.00775962
KCNMB1	0.71723234	0.0149498	S100A4	-1.63953225	0.00379682
RP11-347P5.1	0.71472184	0.00132747	CCRL2	-1.63326672	1.87E-10
GCLC	0.71254215	0.00902975	NDE1	-1.62481697	0.01516028
GNB5	0.70883592	0.02659947	ITGA5	-1.59314872	0.00176442
GFOD1	0.70202421	0.01747156	CXCL8	-1.57694327	0.01149065
CREBBP	0.680231	0.01583994	SELL	-1.57467544	0.00442622
ARHGAP9	0.67940888	0.02176494	CTB-58E17.1	-1.56270573	0.04565019
CD1D	0.67764732	0.00028906	CREB5	-1.54216491	1.3752E-06
NET1	0.67063312	0.00010954	HSP90AA1	-1.53162951	0.04757844
SPNS3	0.66697283	0.02346705	DUOX1	-1.5301934	0.00254922
RARRES3	0.66591135	0.00236472	CD1B	-1.49268907	0.01582653
CD33	0.65826605	0.00407828	GPM6B	-1.48917486	0.01765564
SEPP1	0.64738394	0.03971083	TXN	-1.48583853	8.4999E-09
SMCO4	0.63900404	0.00254922	CD9	-1.48368184	3.8742E-05
KLF13	0.6302354	0.03286425	CDC42EP3	-1.48013139	3.0447E-07
PRKAR2B	0.62997083	0.03290315	CD80	-1.47916242	0.02772805
CEBPB	0.62892141	9.1914E-05	PMP22	-1.47174764	4.3747E-08
EXOC3	0.62768905	0.01676474	BIRC3	-1.45805654	2.402E-06
COMMD5	0.61655976	0.00265091	GNA15	-1.45529051	3.1324E-08
RNASE6	0.61283886	0.00054887	IL1B	-1.42423349	4.2527E-17
TUBA1A	0.59501569	0.00484564	KIF23	-1.423587	0.04045246
DNAJC4	0.59423494	0.00161554	CAPG	-1.42057752	5.4224E-07
FCER1A	0.58472921	0.00098936	EMP3	-1.41762678	6.8462E-23
ANAPC16	0.5805168	0.00021342	TUBB2A	-1.41661291	0.00442622
RAC2	0.57395748	0.00522824	ZEB2-AS1	-1.40954689	0.03663064
FBXO34	0.5704613	0.04045246	PRDX1	-1.40177093	2.7419E-17
CYB5R4	0.56286229	0.02496303	HSPB1	-1.38087198	0.00017173
SEL1L3	0.55390574	0.04237618	MARCKSL1	-1.35007689	5.5987E-06
ATF6B	0.54768422	0.04058326	MMP12	-1.34712923	0.00110076

RNF7	0.5472612	0.00136059	ABCA1	-1.33872602	0.00036811
PLAC8	0.54704674	0.00030738	HLA-DQA2	-1.32278693	1.3472E-09
INSIG1	0.54704548	0.02561254	LYZ	-1.31619186	7.7219E-13
GUSB	0.54658836	0.01594601	MAP2K3	-1.31473607	7.1208E-06
MRC1	0.54602208	0.02032922	ID3	-1.31122408	0.001256
RPS6KA1	0.54186757	0.04014184	G0S2	-1.30271411	2.5976E-05
MS4A6A	0.54042875	0.00027961	LAMP3	-1.29663922	0.01887169
TLN1	0.53462512	0.00022317	RAB29	-1.28713943	6.9422E-06
ARL5A	0.52477796	0.00787033	LYL1	-1.28462735	0.04542368
PKIB	0.52283087	0.00480862	ALOX5AP	-1.28258159	1.4156E-06
TBXAS1	0.50452746	0.02382374	ID2	-1.26807463	6.5176E-12
PTMS	0.49679223	0.00442622	CHAF1A	-1.26089288	3.2778E-05
RP5-1171I10.5	0.49324015	0.01206047	LGALS3	-1.25322509	3.453E-06
RIPK2	0.48655873	0.01537253	KLHL21	-1.2486581	0.01478411
HERPUD1	0.48445575	0.00280911	FAS	-1.24646748	0.03971083
STK17B	0.47939508	0.00661302	PDE4DIP	-1.23984186	0.0003311
FAM26F	0.46712249	0.00407828	ICAM3	-1.23966176	4.4886E-07
EVI2B	0.4659234	0.00618646	GADD45B	-1.23273252	2.2885E-10
CNPY3	0.45699196	0.02561254	TNFAIP2	-1.23216579	1.428E-11
AIF1	0.45693538	2.2769E-07	FPR3	-1.23041379	0.00018363
ATPIF1	0.4549232	0.00100282	INF2	-1.22590926	0.04554486
RNF149	0.440894	0.04700079	TNFAIP8	-1.22127792	3.1234E-09
SPATS2L	0.43242825	0.03395608	ABCG1	-1.21530124	0.02560777
RNASET2	0.42611519	0.01140988	TVP23A	-1.20463888	0.02151577
ERP29	0.42326253	0.00049902	FKBP4	-1.19477837	0.01342097
C10orf54	0.419542	0.04237618	LILRA2	-1.19358845	0.00290084
AREG	0.41731123	0.04589129	PHLDA2	-1.19349425	1.2359E-05
CELF2	0.41627245	0.01068349	SLC43A3	-1.1822562	0.04978459
PYCARD	0.41497087	0.00737932	PPM1N	-1.17682832	0.01894545
GPSM3	0.41071583	0.01321884	NFKBIA	-1.17650183	4.8933E-07
CEBPD	0.39022339	0.01894545	ANXA2	-1.17413922	7.7219E-13
LEPROTL1	0.38996496	0.02613519	ZC3H12A	-1.16544757	9.7508E-07
MCL1	0.37552084	0.00312915	BLVRB	-1.16121706	1.701E-08
UCP2	0.36415151	0.02277407	CCDC28B	-1.15185521	0.04574134
SPINT2	0.34722865	0.00752967	TSPAN4	-1.14319091	0.01099607
RNF130	0.33913762	0.04348746	HLA-DQB2	-1.142585	0.02369789
NPC2	0.32718677	0.01474397	MARCKS	-1.13966571	5.7987E-07
ATP1B3	0.32361118	0.04348746	SFT2D3	-1.11237268	0.00632545
JAML	0.31870696	0.04747104	TWISTNB	-1.10674846	6.7018E-11
HMGN2	0.31191008	0.0296332	HSP90AB1	-1.10594723	3.7722E-05
			RAB9A	-1.10129453	6.9697E-07
			CAPN2	-1.0888242	0.02523181
			SDPR	-1.08218433	0.00085292
			SOD2	-1.07605056	1.3472E-09
			ZBTB10	-1.06329064	0.00430729
			TRIP10	-1.06289579	0.01894545
			SELPLG	-1.05970169	0.0051235
			MAP4K4	-1.04965966	0.01537253
			PLEKHO2	-1.03785327	0.04014184
			MYO1G	-1.03519057	0.00610335

DNAJA1	-1.03368403	0.00524753
FLNA	-1.03066633	0.00054887
S100A10	-1.02647153	9.4422E-10
LMNA	-1.02413598	3.1935E-14
ATF5	-1.02392776	0.00410718
PNP	-1.01461107	0.00312915
CCND1	-1.01385611	0.00355232
SERPINF1	-1.00149535	0.02517021
ARRDC3	-1.00141101	0.00603547
AFMID	-0.99417773	0.02941337
FAM65B	-0.98787781	0.04646273
TMEM120B	-0.98170715	0.01916049
TRMT6	-0.97941668	0.01716251
RASSF4	-0.97893016	8.1556E-07
STMN1	-0.97377352	6.2978E-06
PRKCB	-0.97264769	0.00261433
CD99	-0.97140531	0.00522824
PPIF	-0.97007418	2.9523E-08
CCL4	-0.96492842	0.04663954
POGLUT1	-0.96311582	0.00049902
TPRA1	-0.96262775	0.00788349
ADAM19	-0.96153828	0.00364288
S100B	-0.95284527	0.02482092
FPR1	-0.94995089	0.03798485
EIF4A3	-0.94458453	5.5215E-08
CRIP1	-0.93689468	3.0341E-06
USP12	-0.93373965	0.01972552
CDKN1A	-0.93036115	7.2759E-07
MRPL18	-0.92933733	0.00074502
CD44	-0.92444489	7.0229E-05
KLF10	-0.92383239	0.00096508
SQSTM1	-0.92255674	9.6352E-08
CD83	-0.92197916	3.1234E-09
PPID	-0.92080106	0.00500421
AHNAK	-0.91848093	0.03358109
ABL2	-0.90302938	0.01284774
CCL4L2	-0.89991082	0.00054887
GPR132	-0.89875237	0.03671858
NINJ1	-0.89662268	0.00057964
TIMP1	-0.89489182	5.5215E-08
TANK	-0.88547516	3.6287E-05
MAD1L1	-0.87902791	0.01174459
IFIH1	-0.86975304	0.04495733
TUBB	-0.86885527	8.4285E-11
VAMP5	-0.86432439	0.00555931
PHLDA1	-0.86403135	0.00176442
SLC25A19	-0.85518397	0.0007501
TRAF3	-0.84650037	0.04825021
ARFGAP3	-0.83946712	1.2935E-05
MAFF	-0.83542438	9.1371E-05

THBD	-0.82929514	0.01537253
BHLHE40	-0.82907036	0.00307045
STIP1	-0.82746779	0.0044132
TXNIP	-0.82676735	0.02176494
NFAT5	-0.82368936	0.03430769
IL1R2	-0.82346566	1.0063E-06
IL18BP	-0.81429962	0.00105447
N4BP1	-0.80429023	0.04015368
SIAH2	-0.7997016	0.04258669
DUSP10	-0.79086874	0.0296332
GLIPR2	-0.79012137	0.00049902
FCGR2A	-0.78389471	8.1864E-05
PEA15	-0.77492964	0.00376246
SEMA6B	-0.76875139	0.00333187
DENND4A	-0.76803215	0.00161554
PIKFYVE	-0.76678649	0.04118216
ALDH2	-0.75566288	0.00033625
TSPAN33	-0.754337	0.00453195
OXCT2	-0.75079509	0.02021665
NFKB1	-0.74782878	0.00110076
NQO2	-0.74745057	0.02953213
ARL5B	-0.74396106	0.00280911
TNFSF13B	-0.74023979	0.00030738
IL10RA	-0.73831117	0.00254922
CLIC2	-0.73731587	0.00107994
CCND2	-0.72936995	0.0032795
DDX39A	-0.72563124	0.00065798
TSTD1	-0.72264812	0.00105692
S100A6	-0.72090785	0.02075135
AXL	-0.7077905	0.01682166
PLK3	-0.70157945	0.04122277
SLAMF7	-0.70039032	0.03559467
UGCG	-0.69911526	0.02772805
ASAP1	-0.6937313	0.01174459
GSN	-0.69260961	0.0012675
CFLAR	-0.68466673	3.597E-05
ATF7IP2	-0.68335815	0.04124346
PLEK	-0.67320892	0.001256
MNDA	-0.66994373	0.03955901
DDAH2	-0.66961944	0.00110076
IER5	-0.66871967	0.00050254
TNFSF10	-0.65472883	0.03088703
LACTB	-0.65114838	0.0042338
THAP2	-0.64763384	0.03671858
CORO1C	-0.63951806	0.0456382
YBX3	-0.63557061	0.00017173
REL	-0.63243436	0.01279846
FAM46A	-0.6270804	0.03723978
CD58	-0.62707666	0.04920177
GPX4	-0.62334735	2.8751E-06

CSTA	-0.61913683	0.00323431
MXD1	-0.61791777	0.03812973
IRF8	-0.61670432	0.01882168
SUGT1	-0.61529597	0.01583994
RHOF	-0.61514525	0.04348746
TCP1	-0.61281016	0.01710904
LGALS1	-0.5994824	1.3752E-06
ZNF267	-0.58829853	0.00028252
STX4	-0.58364432	0.04950065
SERPINB1	-0.55246608	0.00433465
RGCC	-0.55131619	0.00028252
H2AFZ	-0.53595889	0.00407828
ARL6IP5	-0.52751786	0.00269183
RAB31	-0.5146799	0.02700721
AHR	-0.5135105	0.02620876
RAB10	-0.51332613	0.03004883
NOP58	-0.5056715	0.0105379
RAB11FIP1	-0.50337609	0.00720228
MT2A	-0.49628949	0.03668116
TUBB4B	-0.49542147	0.00210508
NEAT1	-0.49225564	0.01016431
PHACTR1	-0.48529522	0.00061756
NFE2L2	-0.48202142	0.0005064
CCT4	-0.48153869	0.02134208
TUBA1C	-0.47952176	0.02047642
CLK1	-0.47532474	0.04697985
CDC37	-0.47317393	0.04542368
ATOX1	-0.47074963	0.00464917
PSAP	-0.44926737	0.00176442
GPBP1	-0.43872358	0.0090365
BAZ1A	-0.43492686	0.02277407
ZFAND5	-0.42441551	0.01342097
RPL22L1	-0.41978366	0.03154029
MGAT1	-0.41348186	0.03429631
CD63	-0.37611849	0.00105447
PSMA4	-0.34548321	0.03230381
S100A11	-0.32591541	0.00194676
SEC61B	-0.31903905	0.02843297
CALM2	-0.31146657	0.01103294
SH3BGRL3	-0.30850563	0.01103294
MT-ATP6	-0.30149457	0.04042129
CTSZ	-0.28902747	0.04646273
HLA-DMA	-0.26681268	0.04542368

		cDC3			
**Ileum DEGs**	**avg_logFC**	**adj. p-value**	**Colon DEGs**	**avg_logFC**	**adj. p-value**
FABP6	2.74382323	0.02150171	CRYAB	-4.49038261	0.00015142
ADAMDEC1	2.48415538	3.5725E-05	KCNN4	-3.29787901	0.00015896
RP11-556E13.	2.46568599	0.00832481	HSPA1A	-3.04350518	0.03331522
CXCL12	2.3967424	0.00814333	CCL17	-2.92126245	0.01123932
DNAJC5B	2.37357411	0.02318429	LAYN	-2.79541516	0.01916729
LINC00996	2.23885136	0.01123932	CCR7	-2.70390306	0.03045192
ITGA9	2.04835282	0.0246098	S100A8	-2.62481634	0.01119258
MMP25	1.99919242	0.01123932	DTNA	-2.41479648	0.02150171
WLS	1.9462882	0.03739005	HOMER2	-2.4080342	0.0246098
RAMP2	1.87861595	0.04782058	SCN1B	-2.4062559	0.03852786
LAMP5	1.75842158	0.00045827	S100A9	-2.4000999	0.00748243
CD163L1	1.7154239	0.0157905	SLC35E4	-2.23148925	0.02166858
ALOX15B	1.7060419	0.00049105	LTC4S	-2.16237716	0.0001631
SIGLEC1	1.50727971	0.03289543	MARCO	-2.07093047	0.04934
TSPAN15	1.49630362	0.04839507	UBD	-2.05393032	0.04455462
IGF1	1.4855444	0.00205164	S100A12	-2.03437898	0.02407313
TMEM176A	1.42837966	0.0246098	C16orf54	-1.96318892	0.01497699
SLCO2B1	1.29201963	0.04646611	RAPH1	-1.94780451	0.03739005
PALLD	1.25702717	4.4324E-05	CTD-2006K23.	-1.94223288	0.01338709
CLEC11A	1.24948416	0.00215306	IL1RN	-1.85103459	0.03015421
NINJ2	1.19254304	0.01239592	CCL2	-1.84368576	0.01885809
GPR34	1.1915812	0.01536674	FAM65B	-1.84160183	0.00461351
SLC40A1	1.18908177	0.00265035	CXXC5	-1.82383395	0.01123932
FUCA1	1.17864942	0.03622187	APOBEC3A	-1.79715873	0.0034291
TSC22D1	1.15110044	0.01493477	DNAJA4	-1.76084617	0.02166858
CD38	1.13980765	0.0034291	MFSD2A	-1.67834449	0.02407313
GCLC	1.13533382	0.0034291	PTGIR	-1.65681971	0.01430899
NUDT19	1.12292706	0.04647528	EMP1	-1.61906394	0.00429033
GNB5	1.11551781	0.03590968	SLC2A6	-1.5962593	0.03402276
SCN4B	1.09000155	0.04934	MTMR11	-1.57590654	0.01383932
C1QA	1.08042501	0.00473651	SMAD3	-1.57095586	0.02407313
MAF	1.0796548	0.04782058	RHOF	-1.54906902	0.00225937
DHRS3	1.06696829	0.0044356	LILRA2	-1.51240332	0.00016053
CD209	1.05259135	0.00635247	RNASE1	-1.50923696	0.03618637
ITM2C	1.02847709	0.0003435	FSCN1	-1.50036474	0.02046875
CTSC	0.99468058	0.00060763	ITGA5	-1.44440545	0.01831252
CD101	0.95902895	0.04485972	IL4I1	-1.37936266	0.00035764
LAIR1	0.89489695	0.00158951	CD109	-1.34072892	0.03931053
STAB1	0.89197326	0.0246098	SLC5A3	-1.3366415	0.01430899
SESN2	0.88647187	0.01541153	CD300E	-1.30741274	0.00017499
HBEGF	0.84119762	0.02210639	NRIP3	-1.27380175	0.01119258
LDLR	0.81661734	0.0190677	SLAMF9	-1.20867174	0.0246098
ITGB7	0.79982686	0.00735586	SEMA4A	-1.19166021	0.0044356
ALOX5	0.78389835	0.02407313	TSPO	-1.18378866	8.0252E-10

VSIG4	0.77660162	0.00017499	TRMT6	-1.1811925	0.00215306
FAM105A	0.75358307	0.00305356	S100A4	-1.14475553	0.00019363
PLD4	0.73534423	0.01908237	ICAM3	-1.11665732	0.01430899
AKR1B1	0.71509553	0.00205164	SERPINA1	-1.11322527	3.1674E-08
SIGLEC10	0.68123821	0.01920669	HSPE1	-1.11166394	0.0157905
HN1	0.66741017	0.01573737	CAPN2	-1.0971011	0.00954688
CD163	0.64941199	0.00748243	TNFAIP8	-1.0841302	0.0295702
IDI1	0.62574642	0.02481991	IQSEC1	-1.0816242	0.04143633
CD1D	0.60659737	0.02407313	S100A10	-1.07103604	2.9223E-05
NPC2	0.59600423	0.0034291	S100A6	-1.06876426	0.00013198
ZNF331	0.5589049	0.03852786	CHORDC1	-1.04859561	0.00735586
VEGFA	0.50246677	0.03139093	SDPR	-1.04426405	0.02272514
			DNAJA1	-1.03089947	0.00215306
			MAP2K3	-1.01853728	0.0034291
			PNP	-1.0150251	0.00404271
			CDC42EP3	-1.00674039	0.0375567
			C15orf48	-1.00531503	0.04753026
			GNA15	-1.00435323	0.00748243
			MARCKSL1	-0.99386725	0.00068224
			MYO1G	-0.9935983	0.01924377
			CCRL2	-0.9914568	0.00017499
			EMP3	-0.98754085	3.7498E-07
			STK10	-0.97540625	0.02500397
			BCAT1	-0.96753362	0.02166858
			RALGDS	-0.96648639	0.03739005
			CD52	-0.95357155	0.0246098
			G0S2	-0.94420654	0.02150171
			HMOX1	-0.92676889	0.01803598
			PLP2	-0.91789254	0.0034291
			ATF5	-0.91784515	0.0044356
			DUSP10	-0.91292768	0.02046875
			LHFP	-0.90339234	0.03015421
			IL1B	-0.87252294	0.03739005
			STIP1	-0.87232423	0.02426327
			ADAM8	-0.86640262	0.04455462
			CCND2	-0.86072291	0.00404271
			CAPG	-0.84493748	0.00748243
			CD99	-0.84163143	0.00205164
			HSP90AB1	-0.82810886	0.01127902
			PLEK	-0.81400297	0.01916729
			HSPB1	-0.79969367	0.03820834
			PEA15	-0.79325111	0.01916729
			SH3BGRL3	-0.77519048	0.00016053
			PHLDA2	-0.76791586	0.00433472
			ANXA2	-0.76720454	0.00148321
			IER3	-0.75321748	0.04618617
			GLIPR2	-0.74480287	0.0044013
			LMNA	-0.72809516	0.0001631
			CRIP1	-0.71387361	0.01536674
			RHOC	-0.71005958	0.01698594

CFLAR	-0.7074847	0.03479865
LGALS1	-0.69998418	0.00265035
CDKN1A	-0.66839425	0.03334612
CD83	-0.66767726	0.03200632
FCGR2A	-0.65305218	0.00616638
PPIF	-0.64787343	0.00807385
GSN	-0.62952965	0.04618617
TWISTNB	-0.62370749	0.02307843
C1orf162	-0.60894385	0.03117431
DNAJB6	-0.58799725	0.00265035
AP1S2	-0.58646108	0.03852786
BLVRB	-0.58377108	0.03739005
PHACTR1	-0.56008723	0.02500397
EIF4A3	-0.53393002	0.02665372
ARL4C	-0.53217785	0.00404271
NEAT1	-0.52737284	0.02407313
ANXA1	-0.49808672	0.02307843

Table S8

Patient	Age	Sex	Diagnosis	Site of SI resection
1	25-29	M	CD	Ileum
2	50-54	F	CD	Ileum
3	60-64	F	CD	Ileum
4	65-69	M	CD	Ileum
5	25-29	M	CD	Ileum
6	30-34	M	CD	Ileum

CD = Crohn's Disease. PNR = Primary Non-response. LOR = Loss of response

Treatment	D'Haens score for inflamed area	D'Haens score for uninflamed area
None in last year	9/13	0/13
None	1/13	0/13
Golimumab (PNR)	11/13	6/13
Ustekinumab (PNR)	4/13	0/13
Infliximab (LOR)	5/13	0/13
Thiopurines, Steroids	10/13	6/13

Genes upregulated in CCR7+ cDC

Gene	avg_logFC	adj. p-value
NCCRP1	9.22196784	3.4373E-16
AOC1	8.91255364	2.5456E-26
LAD1	8.63038869	1.4913E-89
CXCL11	7.92780281	1.0783E-31
LINC03025	7.81129596	1.5415E-31
TREML1	7.44147217	0.00055181
CCL19	7.06052302	1.1147E-53
TMEM150C	7.05793257	1.5813E-27
ENTHD1	6.93311431	6.0737E-17
SYNPO	6.84595738	3.5853E-33
MSC	6.83868355	4.1525E-10
LINC00158	6.79716751	2.1334E-11
S1PR1	6.70769025	2.1323E-30
GPC5	6.68468393	7.756E-137
ADAM12	6.6761059	2.1337E-32
ENSG0000022	6.61660785	2.691E-19
TNNT2	6.45198914	1.6531E-16
UBD	6.39569313	9.528E-28
NOS1AP	6.39424284	1.63E-25
LIMCH1	6.38362765	1.7364E-62
KCNN1	6.34611908	4.2121E-57
CCDC68	6.28805553	3.2635E-22
ACHE	6.27548282	7.0996E-45
TMCC2	6.25828475	1.6251E-15
CD200	6.23899885	7.5345E-46
IL32	6.17459517	1.3275E-63
HMSD	6.11035553	3.4085E-33
ITIH1	6.03346212	5.3337E-09
ANXA3	6.01136139	6.8263E-13
MSC-AS1	6.00930697	8.6355E-18
IL12B	5.92634641	0.00358828
UPB1	5.85833965	1.0983E-39
EDARADD	5.83968976	8.0158E-80
IL7R	5.795827	1.3672E-84
CGN	5.74927521	2.7867E-11
SLC1A2	5.65805628	8.4997E-24
CCR7	5.61150238	1.6008E-64
NRP2	5.55428189	5.3099E-91
ST8SIA1	5.53349521	9.0869E-20
SLC8A3	5.49712543	4.7228E-09
ENSG0000024	5.46389116	2.3646E-08
ENSG0000022	5.46330839	3.2222E-36
MIA	5.42504207	1.0901E-13
CDHR3	5.42491771	5.8729E-15
TMEM178B	5.40827707	3.5956E-05
EYA4	5.38251306	1.5601E-16

GO terms enriched in CCR7+ DCs

GO term	Adjusted p-v	Genes
Regulation O	1.2067E-09	CD40;TFRC;GC
Cytokine-Med	1.6205E-07	CXCL9;CXCL8;I
Defense Resp	2.1816E-07	IFITM3;IFITM1
Cellular Resp	2.6515E-07	GSK3B;CD40;C
Defense Respo	3.9291E-07	IFITM3;IFITM1
Positive Regul	2.7917E-06	CD40;GOLT1B;
Receptor Sign	2.8057E-06	STAT5A;STAT5
Response To C	7.1719E-06	IFITM3;APP;CD
Receptor Signa	4.1722E-05	STAT5A;STAT5
Small GTPase	6.2683E-05	RB1;CDKN1A;F
Regulation Of	6.2683E-05	APP;CD81;CD8
Positive Regul	0.00010201	APP;CD40;CDK
Ras Protein Sig	0.00013456	RB1;CDKN1A;F
Regulation Of	0.00019545	DAPK1;GATA6
Apoptotic Pro	0.00021985	APP;TRADD;PF
Positive Regu	0.00021985	CD86;CD274;C
Positive Regul	0.00025134	CD86;APP;TFR
Response To	0.00027926	CD86;OTUD5;
Response To	0.00029564	IFITM3;CD40;
Positive Regu	0.00029564	CD86;CD274;T
Positive Regul	0.00050939	APP;ANKLE2;C
Regulation Of	0.00051611	CGAS;TNFAIP3
Negative Regu	0.00051611	IFITM3;HEXIM
Negative Regu	0.00057573	AVEN;GSK3B;A
Positive Regul	0.00057573	CD40;CD81;CD
Regulation O	0.00067819	MACF1;ZRANB
Positive Regul	0.00070516	TNFAIP8;TRAD
Positive Regul	0.00102252	CD86;APP;ITK;
Cellular Respo	0.00102252	CD40;CXCL8;Z
Cellular Respo	0.00102252	CD86;CD274;C
Cellular Respo	0.00102517	CD86;GSK3B;C
Microvillus O	0.00104866	RAP1B;RAP2C;
Protein Modif	0.00106082	APP;GMFB;PR
Negative Regu	0.00153195	IFITM3;IFITM1
Regulation Of	0.00155254	ANKLE2;TNFRS
Negative Regu	0.00155254	RB1;ANKLE2;T
Cellular Respo	0.00169672	FER;IL6;SBNO2
Regulation Of	0.00192608	IFITM3;IFITM1
Tumor Necros	0.00200056	STAT1;TRADD;
Microvillus As	0.00201456	RAP1B;RAP2C;
Regulation Of	0.00201456	CD86;APP;IFIT
Positive Regul	0.00213916	APP;GSK3B;DT
Neuron Devel	0.00213916	RB1;APP;GSK3
Positive Regul	0.00220964	APP;GSK3B;FC
Positive Regul	0.00225292	MDC1;APP;CC
Regulation Of	0.00283392	CD40;BTG1;CX

TNFRSF11A	5.34716245	1.277E-43	Establishment	0.0028605	PPP1R16B;RD)
PDCD1LG2	5.31210763	3.2085E-37	Positive Regu	0.0028605	IL6;RSAD2;CD8
ENSG0000028	5.29571877	4.9135E-08	Cellular Respo	0.0028605	CD86;CD274;C
NRXN2	5.27807695	1.8235E-28	NIK/NF-kappa	0.00312377	CREBBP;TRAF2
LINC01169	5.26304639	0.0232035	Regulation Of	0.00317393	TNFSF4;TRAF6
DLGAP2	5.25846027	4.4788E-14	Phosphorylati	0.00317393	APP;GSK3B;SM
SLC24A3	5.2531841	1.7054E-19	Regulation Of	0.00323088	LMBR1L;OTUD
NXPH3	5.22801638	7.7218E-12	Cellular Respo	0.00340023	STAT5A;IL2RA;
PDPN	5.21538944	0.02982915	Growth Horm	0.00340023	LYN;STAT5A;P
ENSG0000023	5.19693175	1.5698E-08	Interleukin-2-I	0.00340023	STAT5A;IL2RA;
ZFPM2	5.17231938	8.0466E-17	Negative Regu	0.00380969	WWC2;PLEK;C
CCL22	5.17079295	1.5994E-31	Interleukin-27	0.00380969	OAS1;OAS2;ST
CYP27B1	5.12352496	2.8582E-09	Regulation Of	0.00380969	OAS1;OAS2;O
OR2I1P	5.08071142	8.1353E-10	I-kappaB kinas	0.00384225	RIPK2;TANK;R
CNN3	5.07199302	7.9147E-27	Positive Regul	0.00388678	IL23R;HLA-B;H
LAMP3	5.01717371	7.359E-117	Regulation Of	0.00394955	APP;RASGRP1;
ENSG0000024	5.01689934	0.04720973	Positive Regul	0.00459751	APP;GADD45B
FSCN1	4.99375228	2.3352E-68	Regulation Of	0.00459751	MORC3;RIPK2;
WNT5B	4.99342651	4.6535E-17	Positive Regul	0.00480336	CD86;APP;CD8
SMOC1	4.98870331	8.498E-25	Plasma Memb	0.00497672	RB1;APP;GSK3
SYNPO2	4.97782083	1.9896E-23	Regulation Of	0.00497672	ILRUN;APP;CD
C1QTNF1	4.97533619	1.7753E-10	Cellular Resp	0.00497672	STAT5A;IL15R
HOPX	4.97047575	5.0086E-26	Interleukin-15	0.00497672	STAT5A;IL15R
MREG	4.96422898	5.9995E-62	Regulation Of	0.00497672	SEMA7A;GATA
PDGFRA	4.92857535	2.1596E-13	Positive Regul	0.00500387	APP;CD84;WN
C17orf99	4.91405623	9.6999E-15	Negative Regu	0.00500387	APP;BTG3;IFIT
PIEZO2	4.9115977	7.315E-10	Regulation Of	0.00500387	GSK3B;HSPB1;
ACVR1C	4.89807993	1.3724E-09	Antigen Proc	0.00500387	HLA-B;HLA-C;H
LINC01388	4.87176929	5.3763E-05	Antigen Proce	0.00500387	HLA-B;HLA-C;H
NPR1	4.86814431	1.0198E-10	Antigen Proce	0.00500387	HLA-B;HLA-C;H
INSM1	4.85246702	1.1502E-22	Antigen Proce	0.00500387	HLA-B;HLA-C;H
ADORA2A	4.83786913	1.0989E-43	Positive Regu	0.00500387	IL23R;CLEC6A;
EXOC3L4	4.82557664	6.9221E-05	Cytosolic Patt	0.00514575	IFIH1;CYLD;ITC
CARD18	4.81340836	9.2809E-09	Protein Phosp	0.00526242	APP;GMFB;PR
ENSG0000028	4.77068359	0.0001579	Positive Regul	0.00545201	DDR1;APP;CD4
BICDL1	4.76632221	9.2852E-20	Regulation Of	0.00565906	LYN;CD40;IL15
AXIN2	4.76389631	0.00016452	Positive Regul	0.00568779	IL23R;HLA-B;H
MAPK13	4.76229698	1.1507E-17	Positive Regul	0.00583924	APP;MAML1;G
LNCTAM34A	4.75232119	4.4846E-08	Positive Regul	0.00583924	CD40;BTG1;CX
DLL4	4.75114035	1.0555E-06	Regulation Of	0.00583924	GSK3B;RNF34;
ADTRP	4.72406619	1.5165E-20	Negative Regu	0.00583924	GSK3B;RNF34;
CLDN1	4.71942171	0.00081943	Positive Regul	0.00587564	CD40;BTG1;CX
TNFRSF9	4.71728883	7.1006E-47	Positive Regul	0.00595013	CD40;TFRC;CD
ENSG0000023	4.70742394	0.00015888	Myeloid Leuk	0.00712912	FARP2;BATF2;
GRIK4	4.70531079	9.3619E-09	Positive Regul	0.00736044	CD81;TNFSF4;
CD274	4.67652736	9.753E-34	Positive Regul	0.00736044	NFAT5;IL6;FUT
ENSG0000029	4.67188908	0.00140032	Regulation Of	0.00864854	SEMA7A;GATA
NAV2	4.67114782	9.5148E-27	Regulation Of	0.00880847	TBX21;NFKBIZ
DEPP1	4.66976833	1.8769E-14	Response To N	0.00880847	EGLN1;CDK2;C
TBC1D4	4.65544756	4.8077E-71	Regulation Of	0.00880847	CD86;CD274;T

NRG2	4.65469548	7.3881E-15	Antigen Proce	0.00924379	HLA-B;TAP2;H
LINC02605	4.63473019	4.8023E-30	Actin Filament	0.00985495	WIPF1;SH3KBI
GJA1	4.61442813	0.00016035	Regulation Of	0.0103706	ILRUN;OTUD5,
SOX5	4.60994438	2.3872E-08	Response To T	0.01052337	CD40;CXCL8;Z
LINC01170	4.60644603	0.00391775	Ubiquitin-Dep	0.01052337	GSK3B;RNF11;
LINC02541	4.59510269	6.0235E-14	Positive Regul	0.01096679	RAB2B;TICAM
CNKSR3	4.59325213	1.2086E-20	Cellular Respo	0.01106043	CD86;GSK3B;C
GRIN1	4.54752067	1.0527E-20	Regulation Of	0.01106043	RB1;CDKN1A;(
LINC01358	4.53739496	1.4418E-13	Positive Regul	0.01106043	TICAM2;TYROI
CAMK1G	4.53625775	3.3871E-13	Cellular Respo	0.01106043	STAT5A;STAT1
MIR34AHG	4.50683183	1.0061E-16	Interleukin-9-I	0.01106043	STAT5A;STAT1
LORICRIN	4.50577428	7.513E-12	Regulation Of	0.01106043	OAS1;OAS2;O
NES	4.50498355	0.00625486	Regulation Of	0.01126547	RB1;APP;TNFA
PSD3	4.50218509	3.097E-68	Positive Regul	0.01168381	BCAS3;GSN;CF
TNNI1	4.4761579	0.00136867	Positive Regul	0.01235985	APP;GSK3B;BN
ANKRD33B	4.46037408	1.6797E-39	Positive Regul	0.01258512	CD86;CD274;T
AK8	4.45305407	0.00016479	Fc-epsilon Rec	0.01264993	LYN;MAP2K4;I
DNAH11	4.44650926	2.8338E-18	Positive Regul	0.01264993	RAP2C;RAP2A,
CREB3L1	4.44561521	1.8684E-06	Platelet-Deriv	0.01312378	PDGFRA;PTPN
STEAP1B	4.44328954	4.6275E-32	Positive Regul	0.01313996	APP;RASGRP1;
ST3GAL6-AS1	4.43971266	3.6746E-11	Regulation Of	0.01314911	PPP1R16B;AT(
PTGIR	4.40939767	2.5608E-28	T Cell Recepto	0.01335151	ITK;KHDRBS1;I
KCNK5	4.39300375	1.0813E-12	Calcium-Medi	0.01335151	PRKAA1;CXCL8
EBI3	4.38686615	1.6004E-85	Protein Polyul	0.01434793	UBE2D3;RNF1
STRIP2	4.35796096	5.2405E-28	Cellular Respo	0.01436693	GBP5;CCL22;D
EDIL3	4.35106813	1.016E-06	Positive Regul	0.01436693	CD86;CD80;TN
SLAMF1	4.34109457	1.511E-45	Regulation Of	0.01436693	ILRUN;GSK3B;
ENSG0000028	4.29285745	3.1137E-15	Type II Interfe	0.01436693	STAT1;IFNGR2
CXCL10	4.29211561	1.8341E-18	Regulation Of	0.01455037	GSK3B;POGLU
PALM	4.28884055	0.00256176	Positive Regul	0.01575403	MACF1;SEMA
NOSTRIN	4.2700672	4.138E-09	Regulation Of	0.0163519	ADAMDEC1;C)
SSTR2	4.25835876	8.6913E-05	Negative Regu	0.01722072	GSK3B;RNF34;
GATA3	4.24786274	3.9004E-05	Positive Regul	0.01722072	CAMK1D;CXCL
SLCO5A1	4.2347908	1.0129E-40	Regulation Of	0.01722072	LYN;MFHAS1;
LINC03062	4.23085557	3.0019E-18	Regulation Of	0.01724996	MOB1B;RAP2(
PTPRF	4.23075	1.7814E-09	Regulation Of	0.01874264	RB1;APP;TFRC
SOCS2	4.21376318	2.4365E-39	Protein Autop	0.01928892	DDR1;GSK3B;S
LIPG	4.21178456	0.00011726	Regulation Of	0.02004643	APP;CD40;CDK
LINC00504	4.18115182	2.3335E-06	Positive Regul	0.02019337	APP;GSK3B;AE
ENSG0000025	4.15975671	2.4422E-08	Regulation Of	0.02138217	STAT5A;CD40;
TBX21	4.15602048	3.1026E-09	Positive Regul	0.02185429	SEMA5B;BMPI
COLEC12	4.15422181	3.6677E-07	T Cell Mediate	0.02297813	JAG1;CD70;CR
LINC02227	4.14726126	1.5548E-09	Protein Locali:	0.02365529	RAB3A;CD81;F
FSTL3	4.14537084	4.9253E-21	Positive Regul	0.02365529	APOBEC3F;AP(
TNFRSF18	4.1366383	2.2389E-18	Sterol Biosynt	0.02365529	SQLE;HMGCS1
NLGN4Y	4.12827477	1.3986E-08	Regulation Of	0.02365529	APP;GBP5;SER
SLC22A23	4.12459709	8.4072E-40	Interleukin-6-I	0.02366837	FER;IL6;STAT3
RIMS2	4.11333423	9.0051E-07	Positive Regul	0.02461335	ITK;APP;GSK3E
BMAL2	4.11154767	1.5723E-53	Positive Regul	0.02478464	LYN;CD40;IL15
NTN1	4.10466444	5.1143E-18	T-helper 17 Ce	0.02478464	SOCS3;IL6;IRF4

RELN	4.09200992	0.00021461	Positive Regul	0.02478464	IL23R;IL12B;TY
CEACAM19	4.09046206	1.4593E-05	Tryptophan Ca	0.02478464	IL4I1;KYNU;ID
GPR153	4.08979128	2.3511E-25	Tryptophan M	0.02478464	IL4I1;KYNU;ID
PDZD2	4.08888831	8.3616E-15	Negative Regu	0.02478464	RB1;APP;ADAI
PVR	4.08117973	3.7027E-35	Regulation Of	0.02478464	CAV1;SIAH2;TI
CCDC81	4.07780815	3.3713E-14	Rap Protein Si	0.02478464	RAP1B;RAP2C;
CDKN2B	4.07503047	9.031E-17	T-helper Cell L	0.02478464	SOCS3;IL6;IRF
MIR155HG	4.06592865	9.4662E-45	Establishment	0.02478464	RAP2C;RAP2B;
CYP7B1	4.03831201	1.1335E-14	Negative Regu	0.02478464	TBX21;TNFSF4
CCN2	4.03536554	1.6833E-06	Regulation Of	0.02478464	CYLD;ITCH;PEL
ENSG0000025	4.03279236	0.0048675	Positive Regul	0.02478464	NSF;ANXA2;CD
HEY1	4.01605344	1.5842E-06	Regulation Of	0.02478464	APOBEC3F;AP
BMAL2-AS1	4.00892137	1.3969E-11	Positive Regul	0.02478464	GSK3B;CD40;R
UBASH3A	3.99726998	0.00812902	Antiviral Innat	0.02478464	MORC3;USP2
IGSF3	3.98218098	2.0061E-10	Regulation Of	0.02478857	GSK3B;MACF1
SCN11A	3.97203225	2.6332E-23	Response To I	0.02478857	APP;CD40;CXC
GPR39	3.9646517	2.6542E-06	Protein K48-li	0.0249883	RNF34;DTX3L;
CD247	3.9596084	2.0025E-08	Positive Regul	0.02618514	TNFAIP8;BTG1
ITGB8	3.94403056	1.2032E-23	Growth Horm	0.02675211	LYN;STAT5A;S
TNFAIP6	3.9309364	6.5974E-19	Positive Regul	0.02716807	RB1;APP;CRAC
RARRES2	3.9294427	5.719E-08	Negative Regu	0.02726676	USP20;STAT1;
CCL17	3.92865533	3.5567E-06	Regulation Of	0.02726676	CSF1;TNFAIP6;
ENSG0000028	3.91819437	4.7935E-06	Positive Regul	0.02729715	CDKN1A;MOR
XKR4	3.91809092	0.00010794	Positive Regul	0.02800906	GSK3B;IFITM1
LINC02154	3.91409783	0.0357361	Proteasome-M	0.02802724	GSK3B;UBXN2
ACOD1	3.90656986	5.382E-06	Inflammatory	0.02802724	CD40;CIITA;CX
CASC15	3.89965905	3.8131E-13	Cellular Respo	0.02802724	CD40;CXCL8;C
AFF2	3.88716629	1.8584E-08	Macrophage D	0.02802724	CDC42;SOCS1;
EPB41L4A	3.87718865	2.0764E-06	Cholesterol Bi	0.02812056	HMGCS1;GLB1
RGS6	3.87234711	9.3957E-13	Protein Autou	0.02812056	RNF11;DTX3L;
ERICH6B	3.86286996	0.03824376	Regulation Of	0.02861777	APP;GSK3B;CD
KCNH3	3.85506577	1.1771E-08	Regulation Of	0.02861777	SMARCE1;PBR
TRGC2	3.84347617	0.00011224	Negative Regu	0.02909711	ILRUN;OTUD5,
DUSP5-DT	3.83046316	9.0014E-20	Positive Regul	0.02909711	MACF1;SEMA
ENSG0000028	3.81955477	1.1348E-38	Regulation Of	0.03009977	GSK3B;CD40;R
CXCL9	3.80163501	9.9897E-20	Negative Regu	0.03009977	CGAS;TNFAIP3
LINC01094	3.79136281	1.0168E-06	Regulation Of	0.03227353	LGALS3;DUSP
DAB1	3.7889882	7.0823E-26	Extrinsic Apop	0.03250723	GSK3B;BCL2A1
L3MBTL4	3.78590829	1.1885E-41	Cellular Respo	0.03500656	MAP2K4;CD40
CD70	3.77475541	2.3661E-11	Regulation Of	0.03500656	EGLN1;IL6;PLX
TNFSF4	3.76723789	2.1945E-10	Positive Regul	0.03500656	MACF1;LRRK1
CCDC73	3.7415107	1.8603E-11	Negative Regu	0.03559963	RB1;PTPN1;CD
PLCL1	3.73880565	9.6754E-39	Actin Cytoskel	0.03610812	FARP2;PTPN1;
S1PR1-DT	3.73450869	0.0090696	Regulation Of	0.03610812	APP;PTPN1;M
CEACAM22P	3.72933048	0.00970523	Positive Regul	0.03610812	CAMK1D;CXCL
FAM171A1	3.72452068	1.8437E-06	Fc Receptor Si	0.03610812	LYN;MAP2K4;
ENSG0000028	3.72316321	0.02104588	Protein Locali	0.03610812	RAB3A;CD81;
SYT17	3.71364786	2.367E-05	Cellular Respo	0.03610812	CDKN1A;PRKA
TNFRSF4	3.71272062	2.9312E-28	Dendritic Cell	0.03610812	LYN;BATF2;CA
TC2N	3.71203446	2.2856E-11	Negative Regu	0.03610812	ILRUN;GSK3B;

C3orf52	3.69987625	1.9564E-21
HSD11B1	3.69673804	1.4041E-05
TNFRSF6B	3.69165416	5.5424E-12
CNGA1	3.68475448	0.04027885
MT1M	3.68280322	0.00029748
NACAD	3.66800319	0.01480285
CXCL13	3.66027037	2.0836E-05
PPP1R1C	3.66001593	0.00016278
SLC6A12	3.65679904	9.3938E-34
SEMA5B	3.64973633	0.01007324
MGLL	3.63891984	2.1958E-56
IL2RA	3.61968734	7.6257E-11
ADORA2A-AS1	3.61868202	4.5068E-16
SLC9C1	3.60904465	1.9585E-05
CLCF1	3.60544862	8.8926E-16
PPP1R14C	3.60214609	0.00043797
BAALC	3.58761431	0.0008018
L1CAM	3.58289495	0.04882391
MAPK11	3.56910213	4.9042E-19
INHBA	3.56472975	4.7601E-19
GP1BA	3.55545279	1.2701E-06
CPNE4	3.5479144	0.0006905
CRLF2	3.54712536	3.0562E-13
ETS1	3.52136563	2.5836E-37
CFB	3.52009918	1.4519E-15
RNF150	3.50116937	2.8157E-09
ART3	3.50068214	7.5317E-06
BIRC3	3.48972742	1.6008E-64
HSD11B1-AS1	3.48767461	1.1607E-16
MYH11	3.48662589	1.9102E-18
COL7A1	3.48461038	0.00023751
RNF180	3.48399293	0.03886633
EGOT	3.47030525	7.8829E-08
OPTN	3.45973461	7.1991E-14
RAPGEF5	3.45773244	0.01431754
EPOP	3.45683466	1.772E-19
NMRK1	3.45262644	1.6106E-41
CHST7	3.44924367	1.4658E-49
NOL3	3.42206927	4.2893E-09
EBF1	3.41086464	4.4982E-11
MAOA	3.40801695	0.00056763
ABTB2	3.4078005	1.3318E-35
FAM78B	3.40325292	0.04295514
MARCKS	3.39502582	9.7034E-62
LINC03040	3.3858321	4.9952E-12
MCF2	3.36972947	8.7014E-08
ENSG0000029	3.3526748	3.028E-11
CIT	3.34406299	6.7168E-12
CEACAM16-AS	3.33133375	3.7558E-27
CD80	3.32893919	8.7762E-27

B Cell Chemot	0.03610812	HSD3B7;CXCL1
Interleukin-4-I	0.03610812	STAT5A;IL4R;J
Negative Regu	0.03610812	HYOU1;TMBIN
Negative Regu	0.03610812	LYN;MFHAS1;
Protection Fro	0.03610812	HLA-B;SERPINI
Regulation Of	0.03610812	TNIP1;ZC3H12
Lymphocyte C	0.03750465	HSD3B7;CCL22
Negative Regu	0.03750465	SELENOS;IRF7;
Negative Regu	0.03985934	RNF34;ARL6IP
T Cell Activatio	0.041037	CD86;LMBR1L
T Cell Mediate	0.041037	PRF1;CTSH;EM
T-helper 17 Ce	0.041037	SOCS3;IL6;IRF4
Kynurenine M	0.041037	KYNU;KYAT3;II
Regulation Of	0.041037	CXCL10;GJA1;I
Regulation Of	0.041037	FLOT1;TREM2
Tyrosine Phos	0.041037	IL15;PTK6;JAK
Positive Regul	0.041037	SMARCE1;PBR
Response To M	0.041037	PEF1;TRPC1;C
Positive Regul	0.04132081	LGALS3;IL6;FU
Regulation Of	0.04133399	PTPN1;SPHK1;
Positive Regul	0.04406117	CSNK1G3;JUP;
Positive Regul	0.04406117	GSK3B;PRKAA
Positive Regul	0.04406322	CDKN1A;CSF1;
Negative Regu	0.04406322	IL6;GDI1;DYNL
Proteasomal P	0.04406322	GSK3B;RNF34;
Defense Respo	0.04406322	BATF2;CD40;IF
Epiboly Involv	0.04406322	DDR1;PDPN;M
Ganglioside Bi	0.04406322	ST8SIA4;ST3G
Antigen Recep	0.04424653	KHDRBS1;ITK;I
Negative Regu	0.0442537	RB1;ILRUN;TN
Positive Regul	0.04563081	IFIH1;TBK1;OA
Regulation Of	0.04596093	RAP1B;RAP2A
Regulation Of	0.04644861	CDK17;RB1;CD
Modification-I	0.04812568	RNF11;RNF34;
Protein Locali	0.04812568	RAB3A;CD81;N
Regulation Of	0.049037	RB1;APP;ZNF2
Protein Depho	0.04909874	PPP1R15B;PTP

DYNC1I1	3.31949837	4.656E-09
PARD3B	3.30869028	1.3525E-05
ADAM22	3.30480516	0.00016036
MPPED2	3.29743354	6.2391E-13
SLC7A11-AS1	3.29190767	0.0398232
ENSG0000028	3.29188099	1.7824E-21
SCD5	3.29120824	3.6807E-05
EFCAB6-DT	3.28531415	0.01360619
SPC25	3.26131817	0.00263912
CAV1	3.24403021	6.8994E-05
EPDR1	3.23324041	1.4043E-05
GBP1P1	3.230003	3.1452E-14
ARNT2	3.22941529	1.5035E-09
PAPLN	3.21423713	3.8149E-05
HPN	3.19817509	0.00547969
KIF26B	3.19323109	9.3101E-12
IKZF2	3.18975083	4.1453E-12
ARAP2	3.17977504	3.125E-130
ENSG0000028	3.17916723	5.9472E-15
FSTL1	3.17908171	0.00023194
SLC7A11	3.17618697	3.4866E-19
LMCD1	3.17606383	1.3543E-07
ENSG0000022	3.15582278	1.7753E-10
C9orf152	3.15004871	0.0011213
NR6A1	3.14965255	3.7417E-71
PRF1	3.14771381	0.00068999
CACNA2D1	3.14302839	0.0426136
IL31RA	3.13312751	0.00034649
CCL5	3.13061818	1.3714E-09
ARHGAP10	3.12651148	2.1454E-38
LINC02384	3.12416576	7.198E-14
COL6A1	3.12309851	0.00078396
ENSG0000022	3.11058852	0.00615788
ANKRD22	3.1099526	2.9449E-13
HTR1F	3.10453499	0.00073947
LPAR1	3.10324885	1.1309E-31
PLEKHG1	3.10226773	1.5961E-18
MIR210HG	3.09726681	0.00368843
MT1A	3.09477027	0.04064894
SLC4A4	3.09370382	0.00031223
ENSG0000022	3.09325996	1.1663E-06
AK4	3.09263926	5.8871E-11
ENSG0000028	3.08839064	0.0045797
ENSG0000023	3.07105163	0.01852379
LINC01857	3.07102754	3.1939E-09
TVP23A	3.06752788	4.017E-43
IL15	3.063582	7.6736E-37
ADGRD1	3.06239214	1.4679E-19
PPM1K-DT	3.05770446	0.00642375
CELSR1	3.05095713	8.7161E-10

TNFRSF25	3.04552804	1.1335E-06
TRAF1	3.04009817	2.7077E-49
HILPDA	3.03439514	1.8729E-17
SSPN	3.03255155	3.0837E-13
ROR1-AS1	3.031718	0.04280678
CDC20B	3.03056317	0.00059901
SRSF12	3.02235338	3.638E-13
EPX	3.01923455	0.04511828
RASGRP1	3.01679522	0.0005865
SUCNR1	3.0131076	2.1361E-09
CAMK4	3.00948884	0.0013897
NDE1	3.00718258	3.8923E-36
USP54	3.00623065	8.4986E-13
ETV3L	3.00612174	0.00100149
ETV7	3.0061049	5.1123E-22
TPST1	2.99797494	3.6776E-17
TMEM72-AS1	2.99264682	1.746E-09
ENSG0000022	2.98526434	0.01944466
ENSG0000023	2.97802359	0.00109261
ENSG0000028	2.97742846	3.833E-07
ENSG0000024	2.97434505	0.02963472
JADE3	2.97258385	9.2852E-20
PRRG4	2.96900751	3.0127E-16
CSRP2	2.96648175	1.9761E-07
ENOX1	2.95752752	4.9089E-17
ENSG0000029	2.95712506	1.8854E-07
ULK4P3	2.9541351	0.00690871
GNG11	2.9423809	3.295E-07
MCC	2.9411138	1.084E-09
RBPMS	2.93300939	8.5364E-08
SH3PXD2A	2.93196641	4.1796E-23
C2CD4B	2.93185606	0.0051879
ANKRD18DP	2.93182631	1.2103E-09
TMTC1	2.92957851	0.00023538
LINC02362	2.92935251	5.9167E-08
TCF7L1	2.92513883	9.6959E-08
CDYL2	2.91465191	2.9312E-28
TMEM176A	2.90832407	8.4825E-63
CERS6	2.90780867	2.2995E-47
CD5	2.90432997	6.8831E-13
ENSG0000028	2.90377628	8.8476E-31
CFAP70	2.90024024	1.0331E-06
ENSG0000027	2.89523194	0.00469975
EFCAB6	2.89254399	0.00239352
ENSG0000025	2.89170753	0.00012133
ELOVL7	2.89054526	1.3329E-17
MARCKSL1	2.88972928	3.85E-40
GBP1	2.88701398	1.0089E-32
TRIP10	2.87399131	5.1356E-29
KLHDC7B-DT	2.87396394	7.1757E-12

CASZ1	2.86326834	7.1835E-32
MARVELD2	2.86013991	0.00014443
MIR548A1HG	2.86011014	1.8466E-05
GOLGA8H	2.85415422	0.00241334
POGLUT1	2.85233428	1.3458E-64
PIWIL4-AS1	2.85060215	1.4476E-12
H6PD	2.84124116	4.1237E-23
GBP5	2.83626014	1.8628E-21
ENSG0000026	2.82906711	4.0252E-05
ENSG0000022	2.82829996	1.8949E-07
SOCS2-AS1	2.81510361	4.4185E-05
ENSG0000028	2.81327911	0.0056265
EPHX2	2.81143229	1.7606E-06
AARD	2.81108287	0.03449524
UNC5C	2.80479514	3.4916E-07
ENSG0000026	2.80365508	0.00303002
NUB1	2.79740542	1.8819E-43
KDM2B-DT	2.79321668	0.0016918
ENSG0000029	2.79198	4.2906E-09
IL4I1	2.79099402	1.6332E-33
ICOS	2.78755283	1.2814E-05
EEF1A1-AS1	2.78699741	0.00941965
GPR157	2.78685724	4.2108E-25
ENSG0000022	2.78651118	0.00042859
BCL2L14	2.77648983	4.0397E-19
ACKR3	2.7752277	6.6043E-13
CRIM1	2.76962034	1.5607E-12
STEAP4	2.7661399	6.5155E-06
ENSG0000025	2.76602735	0.01894603
CA12	2.76243933	0.00016278
DOK4	2.7619921	0.03267585
ENSG0000026	2.76094172	0.03442589
LINC00964	2.75977415	0.00481858
PC	2.75645692	1.9671E-08
ENSG0000020	2.75416387	0.00521788
C3orf18	2.74500794	1.041E-06
LTBP1	2.74356128	0.0013991
HHIP-AS1	2.74240695	0.00646904
SDC4	2.7325679	2.5365E-23
ENSG0000028	2.72529331	1.7159E-10
RHOV	2.72510317	0.03701023
ENSG0000021	2.72432824	0.02485521
ENSG0000022	2.72312176	0.00106493
SIAH2-AS1	2.72129523	0.00051202
SDK2	2.72007663	1.9772E-07
WLS	2.71527625	0.00115237
KDM2B	2.71370429	2.6107E-59
CLDN3	2.71026647	0.03739761
ADAMDEC1	2.70864413	1.4017E-15
CERS6-AS1	2.70136249	1.5885E-07

OCLN	2.69921994	6.9353E-06
RGMA	2.687542	0.00070998
ENSG0000023	2.68158009	0.04138756
LINC02102	2.68008396	0.04291675
RASSF6	2.67987266	0.00149699
DNM1	2.67461932	3.1966E-06
MACROH2A2	2.67341276	4.233E-35
MERTK	2.67153104	4.9112E-07
TNS1	2.6666201	3.6532E-05
PBX4	2.66616635	4.035E-08
EMP2	2.66536163	3.3299E-08
PTK6	2.65976666	0.04686298
ENSG0000025	2.65739304	0.00751795
MBOAT2	2.63632033	9.5075E-13
PALM2AKAP2	2.63275492	4.8472E-34
GCNT2	2.62267017	1.1229E-15
SYNGR3	2.61411689	6.4971E-07
PDGFA	2.61242548	7.8361E-06
GATA6	2.61084643	0.03914901
MMP7	2.60539795	0.012971
LINC02202	2.60361906	0.0252967
GADD45G	2.59763159	4.3133E-18
LINC02865	2.5972769	0.04312579
IFIT2	2.59472908	1.1419E-13
FBXO2	2.59341821	0.0002205
NIPAL1	2.59333662	0.00082129
TREM2	2.59241974	9.9093E-08
PXYLP1	2.59106326	0.00014414
MMD	2.58925677	1.0729E-34
GSN-AS1	2.58918402	0.00737124
LINC02236	2.5880576	0.00257047
OTUD7B	2.58402806	0.01163313
PTPRK	2.57826811	8.3917E-05
ANGPTL4	2.57736362	0.00164275
ENSG0000023	2.57587914	0.00359043
IFIT1	2.57408394	7.7901E-07
ZNF608	2.57044803	8.9908E-10
TNFAIP8L3	2.56840926	2.8252E-05
MYO5B	2.56369361	0.04471704
SCG3	2.56100561	0.04815059
LINC02934	2.55966615	8.8836E-05
HAPLN3	2.55787349	3.6968E-21
ACVR2A	2.5564899	9.2364E-15
POU6F1	2.55113865	7.9743E-10
PDLIM4	2.54957075	0.0012881
SEMA3D	2.54908473	0.00099667
MIR503HG	2.54600296	0.00319721
ICAM5	2.54403856	2.7992E-09
ADAM7-AS1	2.54397271	4.1501E-13
ABCA6	2.53505548	0.00083264

PDE9A	2.52963737	0.00253805
IL15RA	2.52837656	1.9338E-21
ESPL1	2.52129814	0.00058706
MUSK	2.51503396	0.00916695
MLLT11	2.51198889	2.8677E-05
PBX1	2.51071223	0.0020312
LAMC1	2.50727418	0.00014665
ENSG0000028	2.50617419	0.00043894
USP12-DT	2.50006837	0.00031352
MAGI2-AS3	2.50004693	0.00087263
STAP2	2.49120669	0.00098681
IL1RN	2.48609102	2.0022E-12
LINC00243	2.47835087	5.0189E-06
FXYD2	2.47447197	1.1335E-10
CDKN2B-AS1	2.47413782	1.0913E-09
ENSG0000025	2.46646706	0.00011573
TNFAIP2	2.4631746	1.5852E-48
LINC00861	2.46214566	0.03738877
FGF12	2.45912601	0.01276592
ENSG0000026	2.45562614	0.00143503
CIB3	2.45368313	0.03661107
SERINC2	2.45362283	0.00177603
ZNG1F	2.45131943	5.8871E-11
PTP4A3	2.45130331	1.0491E-06
RRAGC-DT	2.45097332	1.179E-20
KCNA3	2.45071082	2.4881E-07
TRAFD1	2.44949176	2.5962E-26
MTSS1	2.44848699	0.00023614
FZD7	2.43827561	0.00112821
DSG2	2.43310597	6.9423E-15
MAML2	2.42611144	1.6005E-22
CR1L	2.42241582	0.00047874
RCAN2	2.42226816	0.04980567
CLVS1	2.42150438	0.02189524
DTNB-AS1	2.41952077	0.00507223
ZFTA	2.41398165	1.1771E-08
USP18	2.41131278	5.6317E-06
CCDC28B	2.40660845	1.0963E-19
FGF9	2.406264	0.00571253
NOL4L	2.40488575	1.4541E-23
AQP9	2.40394464	5.252E-07
ANO5	2.40360922	4.5219E-06
ENSG0000027	2.40359071	1.0609E-07
DSCAML1	2.39964482	3.6893E-06
H1-10-AS1	2.39618661	7.9445E-06
TXN	2.39017711	7.7056E-16
RASSF4	2.38964406	1.0558E-42
JAG1	2.38961936	6.8684E-11
TMEM176B	2.38787669	2.7328E-34
IL6	2.38730339	3.2184E-08

ANK2	2.38395767	0.00050229
FPR2	2.38211319	8.4986E-13
SLCO5A1-AS1	2.38097093	0.00181529
LINC01013	2.3775934	0.00297103
RAB9A	2.37696414	8.1348E-44
MIR193BHG	2.37605122	0.00100558
USP12	2.36461735	2.1132E-52
SPEF2	2.3631532	0.0002914
RASGRF1	2.36133245	9.7696E-20
NRTN	2.34986485	0.0214351
AGBL4	2.34621767	0.00090073
LOXL1	2.34549201	0.01572834
ZBED3-AS1	2.34383515	7.7061E-06
GSDMB	2.33988374	0.00039393
C15orf48	2.33843588	1.8365E-14
ENSG0000028	2.33549521	0.00259075
LINC02986	2.33427026	7.0126E-06
MELK	2.32358511	0.01778981
SCUBE2	2.32096691	2.6898E-08
LRP12	2.3183312	7.0056E-12
CD40	2.31312107	1.0667E-25
ENSG0000028	2.31288657	0.03780976
KLF7-IT1	2.31034139	0.04192154
CDK2	2.31009448	1.6505E-18
FBN1	2.30264664	0.00282484
MIR9-1HG	2.30205921	3.33E-08
PDE4C	2.30168007	0.00980922
TIAM1	2.29137714	3.8819E-11
BMP2K	2.2899543	1.6654E-29
SLC6A13	2.28554112	0.03904091
DNAI3	2.28490524	2.7876E-14
LINC02397	2.28357946	4.3087E-06
TP53I11	2.27860547	5.3392E-12
OSBPL6	2.27629628	0.00522444
ENSG0000028	2.26934192	0.02896058
NUAK2	2.2649425	2.5041E-22
CLU	2.25458369	0.00533592
ENSG0000028	2.25150167	0.0390975
RSAD2	2.24963882	1.7432E-06
IL21R	2.24705539	5.2655E-15
AZIN2	2.24550698	0.0361585
SOCS1	2.24180875	1.2562E-40
NUP107-DT	2.24161852	0.01516992
IL27	2.24109304	1.5791E-07
KCNMA1	2.23937382	2.8204E-08
CYB5A	2.23251758	8.49E-15
SGPP2	2.22964743	0.02531411
ENSG0000025	2.22923138	0.00426029
SLC2A5	2.22719038	2.2617E-08
CRACD	2.22499689	5.4447E-11

LINC00173	2.22240408	6.7995E-08
IGHG2	2.22188753	0.01851807
IFIT3	2.21895573	1.7793E-08
MIR181A2HG	2.21689383	8.0883E-08
BAIAP2L1	2.21554371	1.0103E-08
ENSG0000028	2.21518122	0.03588523
ENSG0000027	2.21466759	4.1695E-07
NANOS1	2.21379455	0.00736545
SAMD14	2.21191821	0.00551625
JMJD1C-AS1	2.2101473	6.2952E-05
SGCB	2.20871119	0.00796661
LY75	2.20770988	3.6649E-21
KCNE5	2.20127989	0.03867987
ENSG0000026	2.20069011	5.0709E-05
SPRED2	2.19341327	1.0694E-26
MUC1	2.19098207	0.00041163
FGGY-DT	2.18837783	0.00525215
ENSG0000028	2.18747599	0.00249991
APOBEC3H	2.18641579	0.00339962
MYOCOS	2.18636996	0.01353679
MT1E	2.18529143	0.00010872
HSF4	2.17943853	0.00071131
ENSG0000025	2.17933255	0.00837999
SLC4A11	2.1792162	0.00149313
ALS2CL	2.17757319	0.00059419
NT5DC4	2.1756948	0.00646904
LKAAEAR1	2.17503548	0.00105253
SPATC1	2.17169079	0.00159751
LINC01619	2.16496939	7.1478E-25
SLC30A4-AS1	2.16484405	6.0737E-17
ENSG0000029	2.1640639	0.00021026
AGBL3	2.16339295	2.6574E-08
AKT3	2.16190104	7.4718E-15
CD8B2	2.16028957	0.02420871
CLEC6A	2.15945436	6.0552E-07
PERP	2.15742262	0.00014602
MMP10	2.15492425	0.01707528
IGLV7-46	2.15485377	0.0464255
NXN	2.15416935	0.00597687
IL23R	2.15136183	0.00027383
RAB13	2.1483357	2.1872E-09
DHX58	2.1460234	5.7464E-18
CDC42BPG	2.144813	0.03728518
ENSG0000028	2.14216455	0.02147243
ENSG0000028	2.13609302	1.1851E-08
LINC00589	2.13509299	0.04475811
IMPG2	2.13247717	0.03355616
CBLB	2.12661375	5.7397E-18
SYNGAP1	2.12479193	1.2155E-11
MYO1G	2.12270289	2.8784E-13

LINC02432	2.12242427	0.0326359
PLXND1	2.1209572	4.898E-21
IGFBP4	2.11603296	1.3182E-10
TMEM54	2.11587132	0.0366571
IGF2BP3	2.10384354	0.00691
ENSG0000028	2.10381923	9.53E-05
ENSG0000028	2.10352642	4.5047E-06
TSPAN13	2.10265662	2.3962E-06
GALNT18	2.10159649	0.00793144
PIK3CA	2.10081816	2.377E-12
LNCAROD	2.10062964	0.00022763
BCL2L1-AS1	2.10027491	0.03735857
ENSG0000028	2.0974961	1.6683E-05
LINC01088	2.08844817	0.00930291
ENSG0000025	2.08748671	0.00010917
CERS4	2.08730234	1.8061E-10
RPP38-DT	2.0824237	0.03975725
ENSG0000022	2.08225992	0.00470323
CCNA1	2.08202318	4.0091E-05
E2F7	2.07885913	8.4053E-05
MCOLN3	2.07499186	0.03814918
EPS15-AS1	2.07458312	0.00620928
ENSG0000029	2.07441802	0.02999232
GIPC2	2.07241202	0.00954557
COL1A2	2.06868871	0.04280102
ENSG0000026	2.06859307	8.1519E-07
PLEKHA6	2.06833984	0.00012041
LINC00621	2.06706621	0.00082657
SOWAHC	2.06233786	0.03337638
LSS	2.06212996	8.6934E-14
LINC00540	2.06190949	6.069E-08
IDO1	2.06169263	2.3564E-15
ZBTB10	2.05946607	2.0467E-33
ENSG0000028	2.05628582	4.2088E-07
SWAP70	2.05312315	1.8226E-24
LRRC61	2.05305996	8.8705E-12
FAS	2.05305898	1.7187E-18
GADD45A	2.05040107	8.9822E-18
DSCAM	2.04991119	0.03429456
EFNB2	2.04775333	3.3186E-07
PLS3	2.04413756	3.2822E-05
AMIGO2	2.03947312	0.00951898
MBNL2	2.037216	6.4637E-16
ACTR3C	2.03543086	9.0358E-11
WDR86	2.03155347	0.00308218
THBS4	2.02617315	0.02222114
DIXDC1	2.02439778	0.00029006
MGAT3	2.02030086	0.01379507
RHD	2.0187982	0.02139486
NEXMIF	2.01753202	1.7372E-07

PIK3CD-AS2	2.01748609	0.03281029
SLC2A6	2.01541204	2.2233E-32
SLC12A3	2.01397308	9.9406E-08
FOXP1-IT1	2.01393728	0.01123425
ENSG0000028	2.01384212	0.04291644
DRAM1	2.01334053	5.773E-30
LINC02954	2.00866641	0.00680295
PTPRN2	2.0066777	1.8455E-09
ENSG0000028	2.0059319	2.1707E-06
EFNA5	1.99737589	0.03328363
TLCD3A	1.9924368	1.142E-08
PGM5P2	1.99165728	0.00317454
ABCC4	1.98970433	3.8263E-13
PSMC3IP	1.98820385	0.00377724
IL7	1.98756662	5.6978E-09
ENSG0000029	1.98751456	2.8502E-07
LRIG1	1.98673236	5.8813E-10
ENSG0000028	1.98548658	0.00255057
ENSG0000029	1.98254153	0.00158939
DUBR	1.98227781	0.04507663
PDGFB	1.98207614	1.4017E-06
SIMALR	1.98170008	0.00516018
ADARB1	1.97905646	0.0001063
LINC01814	1.97244735	1.7668E-06
SGTB	1.96831779	4.2572E-22
IGF1R	1.96467605	2.1742E-06
RAMP1	1.96316927	2.161E-09
RHEBL1	1.96288783	1.3596E-14
KLF7	1.95865694	9.0952E-17
LRP11	1.95832603	0.00072109
RNF19B	1.95483581	4.3953E-31
SLC5A6	1.95462824	7.7117E-17
ANO9	1.95254892	0.0001852
FSD1L	1.95240767	1.7582E-13
ENPP4	1.95153251	8.5648E-06
MAP3K14	1.95134575	7.5516E-18
KIF2A	1.95085244	3.3354E-24
GJA9	1.95052374	0.02166642
VWA3B	1.94284106	1.2283E-05
ZNG1C	1.93923874	2.0025E-08
DMGDH	1.93759786	6.2911E-06
ENSG0000027	1.93456803	0.00188572
IKZF4	1.93454715	2.0016E-06
TIFAB	1.93430674	9.5607E-18
MICB-DT	1.93417561	0.01289264
ENSG0000027	1.93289658	0.00197628
DGKH	1.9284969	5.048E-20
ENSG0000025	1.9282233	0.00214174
GRAMD1A-AS	1.92666499	0.00446729
GBP4	1.92373161	4.5171E-26

AP3B2	1.91901928	0.03560844
ENSG0000027	1.91867201	0.04533644
CDC14B	1.91591618	0.00328434
HESX1	1.91440187	1.662E-05
ITK	1.91329557	0.0226593
LINC00612	1.91059357	0.03014229
DLEU7	1.9091938	0.00053517
ENSG0000022	1.90914796	0.02560419
NCS1	1.90852982	0.00150318
IQCG	1.90245421	3.2138E-14
ULK4P2	1.90112758	0.00095845
RIPOR3	1.90060913	1.2401E-08
FOXC1	1.89979223	0.00836244
TMPRSS13	1.89897469	0.00905893
ENSG0000028	1.89786702	1.028E-05
KIF21A	1.89779643	0.00142252
NEK6	1.89622798	1.8524E-29
ZNF620	1.8949672	0.00954471
ENSG0000027	1.89443391	1.2822E-05
CENPI	1.89429113	0.03935436
POT1-AS1	1.89385144	0.02830376
SIAH2	1.89225554	5.288E-19
ROBO1	1.88986603	4.4962E-08
PPP1R15B-AS1	1.88405965	1.6661E-07
GRSF1	1.88243249	4.9976E-11
TTN-AS1	1.88232756	9.6281E-10
EBF4	1.88056822	6.667E-09
ENSG0000029	1.87791516	0.00065697
MXI1	1.87786367	3.6872E-08
TTN	1.87717325	5.8111E-06
DAPP1	1.87589037	7.2345E-36
TEX29	1.87362508	0.01662995
ENSG0000025	1.87218177	0.04624151
TBC1D13	1.87066659	5.0685E-09
MAP3K6	1.86816675	6.9463E-08
SLIT1	1.86681457	0.00521756
NPFFR1	1.86631978	1.3373E-10
FXYD6	1.86612628	0.0011659
TMEM63B	1.86358492	7.0558E-28
MAP4K4	1.86347239	6.0732E-13
ISCU	1.86321686	2.0763E-61
NEO1	1.86106724	0.02958394
HOMER2	1.86057185	2.4397E-14
NAA25	1.85978304	4.4228E-17
LRP8	1.85976356	5.269E-08
PHLDB2	1.8595358	0.01020755
TMEM132A	1.85881608	0.02619672
RORA	1.85773707	0.00010191
STMN3	1.85747751	0.01479893
ITGB8-AS1	1.85624121	0.03027581

ST3GAL6	1.85121613	3.0372E-13
ENSG0000028	1.85021432	0.03739761
CDKN1A	1.84952769	4.0186E-22
AGK	1.84942923	3.696E-36
PPP2R3A	1.84859836	3.2935E-05
ENSG0000028	1.84802887	0.03599558
ENSG0000028	1.84798921	0.01053009
MEIS1	1.84735117	0.00876273
GRB10	1.84627525	9.6628E-06
RAB23	1.84318488	0.01700101
SNX11	1.83912637	9.7273E-10
ABALON	1.83899681	0.04810898
GAB1	1.83138436	1.0835E-09
PDE4DIP	1.83123636	1.9422E-32
SLC44A1	1.83037192	1.8712E-18
NEURL3	1.82961934	0.00188793
SEPTIN10	1.82940515	0.00294461
TSPAN15	1.82677229	2.6815E-12
LCA5	1.82631286	0.01400868
TSPAN33	1.82609404	4.1652E-40
NBL1	1.82589029	0.00025874
PNRC1-DT	1.8236877	0.02811097
MMP25	1.82340057	6.4017E-16
MOB1B	1.82277979	5.6045E-13
LSAMP	1.8220277	0.02873644
DUSP5	1.81898243	1.8439E-18
TRPC1	1.81867889	0.0013763
CASR	1.8183399	0.00023753
ENSG0000028	1.81378624	0.01979117
GJB2	1.81093468	0.00108665
ENSG0000028	1.80937127	0.00078797
TFPI	1.80826591	5.296E-05
ATF5	1.8079519	7.8788E-19
CALN1	1.80760543	0.00033264
ENSG0000024	1.8060164	0.00513415
ADAMTS14	1.80571707	0.0239926
RABGAP1L-DT	1.80421252	0.00106777
CAVIN3	1.8018682	4.6718E-05
ACOT4	1.8007412	0.02223996
CLPTM1	1.80070417	3.7627E-34
BCL2L1	1.79734539	5.8256E-17
AIFM2	1.79572175	2.9214E-05
GPC4	1.79459325	1.7681E-06
ENSG0000023	1.79257003	5.5806E-05
RGS16	1.79105366	0.002716
CABYR	1.79083428	0.04928708
RAB30	1.78958581	3.3452E-13
RIPK2-DT	1.78818517	1.2115E-33
PIWIL4	1.78699761	1.5827E-05
RHBDL2	1.78589436	0.00267183

ENSG0000025	1.78434318	0.00045963
HELZ2	1.78310661	1.0946E-07
ABCA3	1.78219943	0.00018801
AFDN	1.78193557	1.0944E-29
LINC02908	1.77617205	0.00503524
CLEC4D	1.77610024	0.01178939
TMEM45A	1.77574456	0.01420957
RELB	1.77197562	6.4408E-28
DCAF6	1.77050908	1.5114E-11
CENPS	1.76951664	1.7753E-10
C8orf88	1.76805847	0.04262014
SPPL2A	1.76746465	6.7752E-28
CASP5	1.76559301	0.00800867
SAMD4A	1.76527542	7.4942E-06
ARHGEF3	1.76287566	1.5474E-20
ADAM19	1.76214371	1.1118E-12
ANTXR1	1.76126853	0.04820955
SLFN5	1.75942444	2.8684E-13
ALCAM	1.75703186	5.4627E-32
PHEX	1.75608916	0.03908232
NECTIN2	1.75493209	2.3449E-25
C12orf60	1.754709	0.02549375
BACH2	1.75419634	5.6398E-14
TNFAIP8	1.75391751	2.0411E-41
SMC5-DT	1.75308272	3.7412E-09
LNCRNA-IUR	1.75206676	0.00026106
BRPF3-AS1	1.75085582	0.00308406
BIRC2	1.74898587	2.2363E-13
FAM20A	1.74235273	2.367E-05
IDO2	1.74131046	6.3709E-25
NAALADL2	1.74040054	0.0024353
P2RX7	1.73424014	1.589E-05
SMG1-DT	1.73304701	0.00116628
ENSG0000028	1.73086327	0.00689403
ENSG0000028	1.73080992	0.00569846
TBC1D8	1.7272351	4.206E-23
IFIH1	1.72457142	2.6681E-39
GLS	1.72395905	1.8948E-20
ENSG0000026	1.72362303	0.03935436
ACOXL	1.72264594	0.00519732
N4BP2L1	1.72244003	6.8104E-83
CHD3	1.72203816	1.0857E-17
NIBAN1	1.72170872	2.8973E-19
ENSG0000025	1.72108883	0.00063132
RHPN2	1.72101019	0.00377724
APOC1	1.72100156	3.2346E-09
BCL9L	1.71680104	1.1265E-05
SYCE3	1.7127374	0.00905964
N4BP3	1.71191145	0.02293264
MIR17HG	1.71172789	1.5366E-06

NIM1K	1.71042545	0.00516018
LINC02964	1.7047205	0.01317973
ENSG0000022	1.70466926	0.00033566
IGHV3-48	1.70456654	0.04567534
GPR137B	1.70291978	1.4184E-19
COCH	1.70079207	0.01726463
LINC01301	1.69976056	0.00377724
ANKRD55	1.69975602	0.0007623
HERC5	1.69721124	7.259E-17
MDK	1.69575571	0.00047764
CD83	1.69548418	1.4109E-43
LINC03004	1.69516879	0.02753153
SHBG	1.69367749	0.04154844
LINC02100	1.69323	0.00202099
HIVEP1	1.69283324	1.8001E-13
BCAS1	1.69068692	0.038272
FMNL1-DT	1.68769393	1.1787E-14
BRPF3	1.68659308	8.0886E-08
CFAP91	1.68607285	0.01198191
RAB5B	1.68554557	2.0782E-19
ARHGAP22	1.68497245	3.4064E-12
DOK5	1.68491692	0.02364562
CD84	1.68221295	1.1963E-23
TLCD5	1.68162197	0.00040021
NQO1-DT	1.67875376	0.02184092
ENSG0000028	1.67823589	0.00072078
DNAAF1	1.6780578	0.00056433
ENSG0000025	1.67765295	5.3941E-09
GBP2	1.6757243	8.677E-15
OLFM2	1.67434972	0.01133756
RNF213-AS1	1.6736793	6.0653E-07
PLCG1	1.6703984	0.01251014
PLA2G4C	1.67027324	2.5633E-08
KREMEN1	1.66608005	0.00509672
ZMIZ1-AS1	1.66566682	0.01810736
TUBB6	1.66098759	1.46E-15
ZC3H7B	1.66079384	8.2785E-17
TIAM2	1.66021538	5.4559E-09
CSF2RB	1.65868321	1.7465E-16
ZNF260	1.65739292	2.6223E-05
TRAF2	1.65685406	1.4304E-09
ENSG0000025	1.65561181	3.72E-05
ZDHHC13	1.65317067	1.3699E-05
EPSTI1	1.65239135	3.1122E-49
ACTA2	1.65054497	0.00058426
GRAMD1A	1.65013752	2.1641E-15
USP12-AS1	1.64876545	9.8666E-06
LINC01762	1.64802621	0.00114384
CLCN5	1.6463015	8.1573E-18
F3	1.6434244	0.02710808

RAB44	1.63919975	0.02200525
ENSG0000022	1.63865635	0.04212659
CASP7	1.63506687	1.0667E-25
CKAP4	1.63439526	4.4849E-05
CLEC16A	1.63056061	1.0659E-12
ENSG0000028	1.62561589	0.0496689
KLF5	1.62319572	1.7341E-06
CACNA1F	1.62252534	0.00791013
ENSG0000028	1.61952932	2.4855E-06
SAMD9L	1.61785513	4.9439E-15
SLC15A4	1.6143147	1.1442E-23
SH2D3A	1.61409256	4.3387E-05
P2RX4	1.61246001	7.0665E-19
ST3GAL5	1.60652098	2.1564E-16
CYTH1	1.60651168	3.7627E-34
IGHV3-23	1.60584967	0.04364379
CALCRL	1.60310964	1.5928E-18
CEP135	1.60189152	3.522E-17
CSF1	1.59900368	0.00075842
LINC00641	1.59819674	2.0016E-06
ENSG0000025	1.59689959	0.00839736
RIMS3	1.59623001	0.04471704
VASN	1.59173709	0.00199463
ENSG0000027	1.59070779	0.01032723
CCNG2	1.58953492	1.7537E-09
HMGCS1	1.58938287	7.6731E-13
ENSG0000026	1.58895672	0.03571217
TMEM150B	1.58724839	0.00533626
ERICH1	1.58648862	1.2977E-14
SINHCAF	1.5841338	2.9523E-23
ENSG0000027	1.58353139	0.0052996
ENSG0000025	1.5823155	0.00230158
ENSG0000028	1.57957038	0.0029543
LRCH3	1.57245097	3.4503E-16
ENSG0000022	1.57168328	0.00989134
USP2-AS1	1.5715843	0.0020591
ENSG0000025	1.56998931	0.04013732
PSEN2	1.56831492	5.1127E-14
SOD2	1.56286223	2.6275E-12
PAPLN-AS1	1.56245391	0.00573255
PRRT1B	1.56210277	0.01629891
CDK18	1.56205327	0.01000834
TNIP2	1.5605245	3.1956E-28
MSANTD3	1.55754129	6.9812E-06
ENSG0000028	1.55686028	0.04507663
ZNF436	1.55643415	0.01221508
SLC41A2	1.55611362	2.6848E-21
LINC01191	1.55451086	2.6908E-07
ENSG0000022	1.55416007	2.0945E-11
NPIPA9	1.55319759	0.01605115

ENSG0000028	1.55249571	1.6225E-05
ID2	1.55177892	1.1772E-09
PLXNA1	1.55119489	1.6243E-07
ATP11C	1.55094448	1.8099E-09
ENSG0000028	1.54972853	0.00822131
GRINA	1.54944811	8.0668E-29
ABCA10	1.54926482	0.01491987
ENSG0000029	1.54863893	1.2108E-05
KYNU	1.54712908	3.3119E-20
HELLS	1.5456912	3.5708E-06
RRAD	1.54425765	0.00027961
IGKC	1.54323753	0.00295852
GGT5	1.54286102	0.00013727
ZHX2	1.5394281	9.2982E-20
AQP3	1.53887109	0.01170811
GNG4	1.53774349	0.00137571
NR3C2	1.53660402	0.03778641
TEDC1	1.53624415	9.2605E-07
ENSG0000028	1.53469797	0.01519177
ENSG0000028	1.53449248	0.03660286
AVIL	1.53442498	0.00060625
HLA-G	1.53434039	0.03767433
BTG1-DT	1.53285805	1.6861E-06
CRIP1	1.53211215	1.7383E-13
GPR55	1.53138733	0.00345395
FNDC3B	1.53129815	2.3449E-25
TTLL9	1.53014923	0.00015795
RNF145	1.53003197	9.0304E-25
FGF18	1.5297459	0.00551153
DLEU1	1.52952102	8.0221E-14
LYST	1.52860226	3.161E-14
ENSG0000029	1.52741601	0.00244869
PDGFC	1.52662453	0.00097823
ABCD2	1.52402078	0.00470323
ENSG0000025	1.52242027	0.00380584
RFTN1	1.52201218	2.9085E-15
CBX7	1.52080143	0.00521433
PPM1K	1.5199119	2.9767E-25
NRP1	1.51588249	1.4611E-08
KCNQ5	1.51539256	0.04990975
RBBP9	1.51505531	5.0397E-10
KLF12	1.51481235	3.6008E-09
CDCA4	1.51284301	1.3497E-08
THAP3	1.51277705	7.1163E-11
SPTLC3	1.50624948	0.00046076
PLAG1	1.50494249	0.00318208
FMNL3	1.50268848	6.5275E-16
PDE3A	1.50256922	0.01348302
SEMA3C	1.50086114	1.2738E-06
SEMA7A	1.50059943	2.6869E-09

ENSG0000028	1.49924711	0.04639102
NFKB2	1.4986934	2.4185E-25
MMP25-AS1	1.49754373	2.2669E-06
FUT7	1.49395697	0.00405703
TNKS1BP1	1.49217888	3.2499E-05
FOXO1	1.49169046	2.9652E-09
LRRFIP2	1.4910655	1.5601E-16
CFLAR	1.4896534	2.7309E-27
TUBB	1.48936005	7.3222E-28
NAV1	1.48875304	3.8245E-09
ENSG0000027	1.4885914	0.04388509
HSPB1	1.48699411	3.0665E-06
ALPK2	1.48608212	2.7045E-05
ENSG0000027	1.48559945	0.03629894
ENSG0000028	1.48514537	7.2399E-06
PAQR3	1.48256462	9.337E-09
PTPN1	1.4816172	4.1018E-30
FTX	1.48019645	1.7105E-10
CDT1	1.48001562	0.01438685
TCEANC	1.4782886	0.00038477
GTDC1	1.4778451	1.3282E-14
MYOSLID	1.47747205	0.00804535
TMOD2	1.47717299	3.4447E-05
SESN3	1.47614641	5.1333E-08
TULP3	1.47465353	0.00034681
LINC00189	1.47373828	0.01948505
ARL17A	1.47237944	1.4496E-08
MYO1B	1.47200197	0.00651302
TUBB2A	1.47106425	6.2387E-05
ARMC9	1.4707508	0.00032706
ENSG0000027	1.47026206	0.01415423
SMAD7	1.46900341	1.529E-07
NID1	1.46806938	1.3205E-10
POU2F1	1.46549487	1.4697E-07
ENSG0000029	1.46444907	0.02141646
ATP8A1	1.46278202	1.3387E-10
DDX59-AS1	1.46174142	0.03007501
RUBCN	1.46142758	2.427E-12
SPINDOC	1.45954922	9.0122E-09
EPAS1	1.45857249	0.00041681
RAP2A	1.45759095	1.8277E-12
ST3GAL2	1.4568488	2.3918E-11
ENSG0000023	1.45613528	0.02463741
ZSCAN2	1.45247207	2.9272E-05
LINC01694	1.4519897	0.0393352
BOLA3-DT	1.45178068	0.01350299
RAB12	1.44916177	6.9169E-11
RAD18	1.44735215	1.0749E-09
PARP14	1.44597362	8.1556E-23
ASAP1-IT2	1.44511993	0.038272

ANK3	1.44496832	2.1136E-05
ZNRF1	1.44399982	1.705E-10
ENSG0000027	1.44324193	0.00070676
CDC42EP4	1.44148144	1.1839E-05
ARID5B	1.43558438	5.4531E-08
IZUMO4	1.43546063	0.00022535
GPR107	1.43280308	7.0023E-23
L3MBTL2-AS1	1.43200006	0.03135733
HROB	1.43095091	0.00356145
CLIC4	1.43030556	1.5608E-09
TDRD7	1.42952769	1.4955E-08
LMCD1-AS1	1.42501306	0.02024316
NEMP1	1.42463479	1.009E-08
CUL1	1.42214971	2.3095E-25
ZER1	1.42211811	7.3751E-07
TMEM131L	1.42210039	2.1569E-09
ORMDL3	1.41642269	0.00030931
PACS1	1.4146738	1.2876E-08
MINK1	1.41297199	4.4402E-12
PDE5A	1.41163867	0.02216307
NABP1	1.41026545	8.3729E-16
C4orf46	1.40986296	0.00029312
PRDM2	1.40681004	5.1278E-12
INSL6	1.40657245	0.00467674
EEF1AKMT3	1.40483035	0.00823389
PEX14	1.40313527	1.0998E-06
IRF4	1.40161899	6.6417E-09
LINC01948	1.40035063	0.0446123
CST7	1.39878756	3.587E-11
ENSG0000028	1.397544	0.00858325
ENSG0000028	1.39717879	1.706E-05
IPO9-AS1	1.39684135	1.0602E-06
BHLHE41	1.39544661	0.04160904
ENSG0000029	1.39508209	0.01206831
IER3	1.3942895	6.8368E-10
ZEB1	1.38873081	7.4669E-11
NRBP2	1.38869892	0.00145546
CDK6-AS1	1.38571709	0.00785026
C17orf58	1.38561238	1.1198E-05
ATP8B2	1.38558572	0.01289264
BRIP1	1.38376683	0.03015651
GPR132	1.38248217	3.6002E-16
FXYD6-AS1	1.37977077	0.04559912
VAV2	1.3784521	1.1924E-07
APOE	1.37624598	0.02529338
ENSG0000023	1.37544683	0.04823415
ST8SIA4	1.37373399	4.6687E-17
ATXN7L1	1.37323859	3.4353E-05
SPSB1	1.37280309	0.00021811
CRISPLD2	1.37267942	0.00019919

NETO2	1.37148402	2.2558E-08
ANKLE2	1.3699998	1.2656E-10
ENSG0000027	1.3699447	1.3381E-08
TBC1D32	1.36947568	5.3497E-10
PRAG1	1.36889091	0.00264688
TRIM69	1.36806808	1.3061E-11
UGCG	1.36806553	1.6251E-15
TMEM140	1.36586259	3.6861E-07
MT2A	1.3652823	2.1347E-09
PPFIBP1	1.36488781	0.0001316
CLIP2	1.36398271	2.5765E-07
WNT5A	1.36329452	3.7008E-11
STAT4	1.36285351	4.5838E-15
ENSG0000027	1.36243293	0.03433578
ENO3	1.36084178	0.00161504
IFI44L	1.36019758	0.00035102
CPEB4	1.35689395	9.9707E-15
ZBTB38	1.35674782	1.1141E-06
TOGARAM1	1.35554705	8.562E-06
CD58	1.35549488	1.7227E-26
TMEM268	1.35407573	8.8461E-13
LYPD3	1.35405051	2.2368E-06
DDR1	1.35383511	0.03716791
MED13L	1.35345637	2.6585E-28
TMEM39A	1.35266444	7.0299E-17
RNF115	1.35033611	1.6639E-26
ENSG0000028	1.35010237	0.01266013
CCDC102A	1.34941647	0.01810736
SPHK1	1.3490606	1.3311E-07
NPIPA1	1.34827369	0.00055839
HDGFL3	1.34800919	4.7885E-25
BCAT1	1.34696427	7.7313E-12
AGRN	1.34305708	2.5949E-05
CXXC5	1.34086974	2.3492E-08
SERPING1	1.33631851	1.9155E-05
GCSAM	1.33469918	0.00247221
CEP350	1.33440583	4.3559E-19
NFKB1	1.33275697	1.4046E-25
GEN1	1.33231682	6.1868E-05
ENSG0000026	1.33173469	0.01412637
MARCHF9	1.3308142	0.0001363
PPP1R16B	1.33055907	1.1971E-07
TTF2	1.32634429	5.3074E-11
NPIPB3	1.32627218	0.00076301
RDX	1.32359871	5.258E-15
SYNGR2	1.32332872	1.0273E-45
PLD2	1.32316321	9.1926E-10
PHLDA3	1.3204371	0.00591571
ZBTB5	1.31961232	6.4036E-08
GPX4	1.31842044	1.3347E-27

FLJ20021	1.31781096	0.01420957
CLIC2	1.31738479	4.8823E-21
KCNJ2	1.31722535	0.03521757
CEP19	1.31718688	5.5827E-05
GABPB1	1.3158806	8.0928E-17
ENSG0000026	1.31496409	0.0361585
TBC1D2B	1.31470367	3.0587E-18
TGM2	1.31393915	0.00035504
BATF2	1.3132573	0.00382924
SQLE	1.31223546	9.0022E-10
ENSG0000027	1.31193489	4.9734E-05
HMMR	1.31130192	0.02333694
PIKFYVE	1.31024183	1.4723E-10
ENPP2	1.31007055	5.8001E-09
CCL20	1.30625062	0.02152502
RND1	1.3056481	0.00011956
CHST2	1.30432613	3.6154E-10
KLF8	1.29889637	0.01270379
CBLN3	1.29828555	0.02307935
A2M	1.29668315	0.00028786
MIR3142HG	1.29431265	0.00650113
RAPH1	1.29367923	0.00106609
TFEC	1.29283637	8.6764E-06
CD1B	1.2922397	0.00782908
MGC16275	1.29170285	0.00010482
EZH2	1.28994647	4.5549E-13
ENSG0000022	1.28987949	0.00537029
TTC7B	1.28968293	0.00092957
CSF2RA	1.28929164	3.0801E-16
TMEM237	1.28814989	0.00899337
ENSG0000027	1.28740734	0.01861182
MRTFA	1.28657227	1.3435E-09
BHLHE40-AS1	1.28382688	2.6071E-05
PLGRKT	1.28337047	2.6939E-17
NECTIN1	1.28317265	0.00028786
PEAK1	1.28280947	3.2906E-17
CLEC2D	1.28005576	0.0032301
GBP3	1.27875335	6.4429E-07
PPP1R15B	1.27708372	4.1419E-19
SEC61A2	1.27213266	0.00640919
TNFAIP1	1.26886668	9.1038E-06
TCF7	1.26723054	0.00652231
S100A13	1.26536561	0.01084456
MTHFR	1.26389587	3.6604E-14
MVP-DT	1.26306982	0.00059867
TMEM131	1.26292396	9.9108E-22
DENND5A	1.26227187	1.4261E-15
ENSG0000024	1.26079571	0.00713161
EPHB3	1.25934493	0.00447411
HSD3B7	1.25914464	2.4769E-06

RCL1	1.25758158	5.0766E-07
ANXA7	1.25649457	9.4153E-19
SMNDC1	1.25546754	4.5997E-17
PHF13	1.25542264	0.00178253
ARHGAP25	1.25463001	2.8633E-10
APBB2	1.25176513	0.00880432
FADS2	1.25109321	3.7897E-07
ENSG0000022	1.25078161	0.01503397
RAPGEF2	1.24780851	5.0007E-08
SRD5A1	1.24767313	1.5463E-07
BAZ1A-AS1	1.24681657	0.00012533
NINJ1	1.24548375	5.0761E-11
DIPK1A	1.24324963	1.1113E-09
CLSPN	1.2406008	0.00675148
FARP1	1.23950587	0.00318927
STK4	1.23931875	5.3054E-16
EHD1	1.2383979	4.2314E-18
ZNG1E	1.23834168	2.3269E-10
DOCK4	1.23823886	2.0362E-05
ZDHHC18	1.23477937	4.0021E-11
SBNO2	1.23271315	3.421E-12
UBE2E2	1.23092565	2.9837E-29
TFRC	1.23061956	1.9984E-08
CASTOR1	1.23036741	2.2024E-06
ENSG0000023	1.23014529	0.04995209
C17orf114	1.22991089	0.0047879
TCHH	1.22958447	0.00860651
TMEM231	1.2287483	0.00096951
WWC2	1.22873009	6.4726E-05
NAPB	1.22831502	0.00035255
BTN2A2	1.22825927	9.604E-13
NEK4	1.22793748	1.1173E-08
ATOX1	1.22729544	4.0046E-48
ENSG0000023	1.22649548	1.0067E-07
RHOF	1.22560582	5.1116E-09
LMNB1	1.22446895	1.2891E-10
ENSG0000027	1.22392116	0.03902288
JPT2	1.22386513	1.8295E-06
ACSM3	1.22356376	0.02097001
ASAP2	1.22294887	2.0063E-10
EIF5A2	1.22290137	0.02619496
IGSF8	1.22213391	1.0732E-08
G0S2	1.22148038	8.138E-16
GPBP1	1.22139616	8.1445E-29
EXO5	1.22072001	0.00654268
TBCEL	1.22037756	0.00024536
RAB29	1.21853954	1.4662E-10
FER	1.21640698	6.4783E-09
ENSG0000028	1.21277847	0.00511459
CREBRF	1.21222376	9.6819E-16

BLVRA	1.21122958	1.0812E-10
GTPBP1	1.21076069	3.0748E-11
TRADD	1.21024883	5.5007E-21
RAB8B	1.20846958	2.1826E-15
TBC1D8-AS1	1.20734579	0.0349893
PDC-AS1	1.20684914	0.0466078
ENSG0000023	1.20683205	0.00649005
ERVK13-1	1.20655426	1.0263E-05
ZNF438	1.20599308	0.0013457
PLAGL1	1.20532842	6.8363E-17
AUH	1.20529011	0.00066454
STAMBPL1	1.20508818	8.5897E-06
SLC35F2	1.20460171	0.0001955
BCL2A1	1.20339732	6.8984E-21
EPM2A	1.2014481	8.2667E-05
ENSG0000028	1.20061439	3.4571E-05
TUBA1C	1.200138	4.6635E-14
DCLRE1B	1.19723915	1.4085E-05
CYRIA	1.19715712	7.5714E-30
RNF207	1.19550685	0.00105253
MYO1C	1.19537367	0.00015992
CHROMR	1.19527043	0.00104285
FNBP1	1.19523091	2.1816E-15
FZD6	1.19522239	0.03705012
GRK3	1.19437981	2.0008E-17
COMMD1	1.19428319	3.4558E-19
SDC3	1.194155	0.00664967
TICAM2	1.19276903	2.5201E-09
MEI1	1.19246173	0.00198798
ARID2	1.18988558	1.6878E-07
PPA1	1.18897379	2.8897E-13
NECAP2	1.18877227	2.1693E-21
PRKAR1B-AS1	1.18767483	0.00463455
CEP120	1.18765821	0.00132393
BMF	1.18615527	2.3302E-08
ETV3	1.18613835	3.2962E-11
ALDH1A3-AS1	1.18569858	0.04611077
PLA1A	1.18555113	0.00011671
MLLT6	1.18399626	7.5536E-06
SLC15A3	1.18316503	1.6679E-22
KPNB1-DT	1.18218984	0.00071031
SLC39A13	1.18080075	3.5071E-06
IL1A	1.18051726	0.00400539
TANK	1.18017961	3.8359E-50
IL2RG	1.17991177	8.8001E-25
PCNX1	1.17796718	5.7054E-11
MMP14	1.17653589	0.00157809
PELI1	1.17629015	1.4801E-10
HIVEP2-DT	1.17494993	0.0380437
NFKBIA	1.17394907	7.4718E-15

ISG20	1.17394821	3.796E-08
DLGAP4	1.17294297	2.414E-15
ENSG0000027	1.17216056	2.2011E-05
TRABD2A	1.17203588	3.34E-09
RASL11A	1.17089491	0.00345631
PSMD14-DT	1.170265	0.01317973
TTL	1.16977274	3.7385E-08
SSH1	1.16965534	1.2215E-10
SPG11	1.16956163	3.0641E-10
TRIM2	1.16955732	0.03336228
TENT4A	1.16936017	2.5502E-07
CNTLN	1.16915826	3.2214E-18
CKB	1.16889919	0.00141402
LMLN	1.16824787	0.02181577
ZFHX2	1.16815277	0.03475289
SLC1A3	1.16681637	0.0045797
MCOLN2	1.16568618	1.9398E-09
GSN	1.16551506	2.4647E-26
SNN	1.16490211	5.3424E-16
MFHAS1	1.16223532	6.8079E-05
ARL17B	1.16193643	0.00095565
IRF1-AS1	1.16091561	5.0525E-14
MAP3K1	1.16045475	1.6794E-08
OASL	1.15987478	0.00061293
REL	1.15736263	4.7414E-39
TDP2	1.15696865	1.9035E-09
ENSG0000029	1.1563375	0.00081381
TFCP2	1.15537094	1.0605E-09
CPEB2	1.15356598	5.7927E-06
ST8SIA6	1.15333199	0.00148928
DLGAP1-AS1	1.15299532	0.00137799
DANT2	1.15276771	0.00573255
DHCR7	1.15199312	0.00034971
AMBRA1	1.15140843	1.1283E-07
ENSG0000028	1.15130929	0.03032467
CDYL	1.14981305	1.8016E-09
DNAJB4	1.14844675	0.00055275
FBRSL1	1.14832254	4.4126E-11
MOV10	1.14639014	1.0867E-06
RYK	1.14396471	1.9058E-05
TGFA	1.14245743	0.00747772
DLEU2	1.14128592	1.1418E-08
PNRC1	1.14082133	2.2441E-16
RAP1B	1.13913948	1.7411E-29
WARS1	1.13879727	3.241E-05
FHAD1	1.13815805	0.00723879
ZC3H7A	1.13604902	5.4783E-20
ENSG0000023	1.13475852	0.04349893
BHLHE40	1.13430192	1.9348E-14
ZWILCH	1.13417199	0.0130657

GGH	1.12935637	0.00279072
SP4	1.12926623	0.00074164
DHCR24	1.12829912	0.00029543
CDC14A	1.1277175	9.6887E-07
ALPK1	1.12587132	5.2728E-11
ZMAT3	1.12545887	1.0445E-05
MTA3	1.12538938	8.5094E-05
OGA	1.12167867	1.1874E-06
HAUS5	1.12121555	7.425E-06
BRCA2	1.12039675	4.0433E-08
STAT5A	1.11480718	8.5394E-10
ZSWIM4	1.11236532	0.00409437
DMXL2	1.11215573	0.00103259
ARID1B	1.11096104	9.3656E-09
MSMO1	1.10904497	0.00073673
MAP3K13	1.1073665	1.3029E-09
RGL1	1.10677148	2.8259E-07
RIGI	1.10593386	1.9012E-06
NXT2	1.10498032	0.00072433
IL13RA1	1.10404533	1.5919E-10
FAM107B	1.10163426	9.6027E-14
TNFSF13B	1.10123737	9.0774E-09
TBL1X	1.10077721	1.257E-11
RUFY3	1.10045934	9.0786E-16
ABCA5	1.09956692	0.00020176
DTNB	1.09878616	0.00029361
LINC00598	1.09820731	0.02262321
PITPNC1	1.09793993	7.3713E-09
DENND11	1.09682861	0.00084435
KATNAL1	1.09598941	0.01540843
PIM1	1.09561574	2.4E-10
IGF2BP2	1.09402366	5.4044E-05
MTMR4	1.09372144	2.0076E-07
RAB3IP	1.0933668	0.01032126
N4BP1	1.0922935	5.3664E-11
CALCRL-AS1	1.09160032	1.4787E-07
ARHGEF7	1.09068798	5.8388E-10
ANKRD10	1.08608113	1.92E-14
DAAM1	1.08602348	2.1613E-06
FUT4	1.08469499	2.2051E-07
NPIPB5	1.08361117	1.0632E-07
SMG1	1.08325113	3.7265E-18
FRS2	1.08262608	1.5985E-10
B4GALT5	1.08207326	5.9503E-22
ATF6	1.08189323	5.2282E-09
ZBTB17	1.08006906	1.3992E-06
ENSG0000028	1.08003074	0.02400655
APBA3	1.07974843	3.7229E-08
IL4R	1.07810842	7.6283E-06
ZFAND4	1.07750247	1.8795E-06

ENSG0000029	1.07684036	4.6201E-07
APOL1	1.07677414	1.1916E-05
LYSMD2	1.07641612	8.7357E-14
VOPP1	1.07635061	1.4196E-37
LUZP1	1.0754729	1.3E-07
MAST4	1.07542042	0.0091225
TNIP1	1.07517904	7.2659E-10
PDLIM5	1.07284888	1.2617E-23
MAP4	1.07269406	3.0782E-16
ZNF684	1.0710169	0.00376634
RNF213	1.07068476	7.1211E-09
ILRUN	1.07014953	1.6884E-16
ENSG0000028	1.06853098	0.0010109
DENND4A	1.06842943	6.9921E-12
DNAJB6	1.06764275	7.4304E-16
MED12	1.06628883	1.5578E-06
LAMB3	1.06586554	2.2708E-05
NBN	1.06517907	4.2012E-19
RPS27L	1.06510593	1.1243E-05
VPS13A	1.06501003	6.4082E-07
ACAT2	1.06435758	2.6121E-13
TBC1D31	1.06412237	0.0009824
RRAGC	1.06145161	3.6998E-12
RIF1	1.06107297	7.5974E-17
ST20-AS1	1.0596547	0.00381599
STAT1	1.05892235	9.2347E-07
JAK1	1.05834046	1.6595E-15
IFFO2	1.05820132	1.0017E-06
ORAI1	1.05777403	4.3013E-17
WSB2	1.05723478	6.9655E-15
COBLL1	1.0565925	0.03165404
SWT1	1.05644852	0.0051879
PXDC1	1.05485963	7.4407E-05
SLC37A1	1.05347235	7.8954E-11
TESMIN	1.0522983	0.03526182
TSC22D1	1.0513387	0.00977582
ROR1	1.05129727	0.00255817
NUP107	1.05077694	7.0174E-06
ST20	1.04989645	8.8732E-10
SPRED1	1.04858648	0.01586163
CFL2	1.04845663	0.00140181
ZNF821	1.04795893	0.00012348
RAP2B	1.04724463	3.1415E-11
LRRK1	1.04575348	4.4653E-12
APOBEC3F	1.04557578	0.0005225
HDAC2-AS2	1.04499513	0.00021729
P2RY10	1.04381258	3.5532E-08
CAMSAP2	1.04059899	0.0308006
ABCB10	1.03944827	0.00111376
RBMXL1	1.03774694	3.3808E-06

DRGX	1.03600727	0.03860933
RHOH	1.03558214	6.0307E-08
ZFP69B	1.03537615	0.04428589
WAKMAR2	1.03394075	1.0075E-07
ENSG0000023	1.0327734	0.03209117
LINC02798	1.0318632	0.00217979
ENSG0000028	1.03131757	0.03744486
DLGAP4-AS1	1.03094835	0.04649804
NOTCH2NLC	1.0307198	5.2269E-05
BCL2	1.02905666	3.0561E-09
PIAS1	1.02828492	1.2896E-16
TNFSF10	1.02651684	0.00031549
C19orf12	1.02602134	0.00013303
ENSG0000024	1.02599616	0.0100856
ARHGEF2	1.02596858	1.4672E-15
PEA15	1.02586148	4.8481E-08
ARHGEF10L	1.02439498	0.03041915
SLC9A9	1.02432502	8.4428E-07
IMMP2L	1.02192827	0.00029392
BBC3	1.02129946	0.00014504
IRF1	1.0198139	2.6342E-10
ABHD17C	1.017921	0.00782908
SREBF2	1.01790721	8.0921E-11
C2CD2L	1.01538839	0.00052674
CGAS	1.01416101	2.1962E-05
BTG3	1.01387358	4.0021E-11
SERPINB9P1	1.01333051	2.5808E-06
TRAF6	1.01153864	0.00022103
KHDRBS3	1.01091851	0.00476485
ENSG0000027	1.00998717	6.9111E-05
RNF144B	1.00824577	8.7587E-17
CCSER2	1.00728218	2.3788E-11
PLEKHA5	1.00707001	0.00043538
MALT1	1.00637567	6.929E-10
HYMAI	1.00635956	0.00447258
PIAS3	1.00566883	4.1087E-05
ALAS1	1.00529514	8.6372E-13
C11orf80	1.0051056	0.00394889
DAPK1	1.00398584	6.5889E-10
KLC2	1.00371972	0.00068757
MOSPD2	1.00210047	1.0678E-12
CTC1	0.99961876	5.8319E-05
FAM53B	0.99950196	1.803E-09
VMO1	0.99943627	0.01323921
F11R	0.99823034	7.9556E-11
JAK3	0.99817859	6.3285E-05
PARP12	0.99805471	1.4404E-08
RAP2C-AS1	0.99743166	0.00187836
POPDC2	0.99581657	0.01473905
MVD	0.99433318	1.1349E-09

ADAMTSL4	0.99360264	3.2982E-05
CDK14	0.99326835	1.9548E-09
DPP9	0.99294033	1.4746E-09
RBMS1	0.99202149	9.7273E-10
APOBEC3G	0.98936796	6.7352E-07
GALNT4	0.98752832	0.00521028
REPIN1	0.98564825	6.6644E-08
ENSG0000029	0.98479968	0.00037343
TAP1	0.98306738	7.1165E-06
ACOT13	0.98144939	9.7269E-10
STAT5B	0.98066694	1.1389E-05
PLXNC1	0.9801645	3.4761E-09
NEU3	0.97941249	0.00014414
MITF	0.97907641	0.02025144
USP24	0.97885866	7.5189E-06
HLA-L	0.97741594	5.9891E-07
TMCC3	0.97503706	1.5141E-05
PIM3	0.97470747	9.1974E-06
THAP2	0.97376508	1.9619E-05
IRAK2	0.97216682	0.00016529
ENSG0000028	0.97136422	0.00171032
PIAS2	0.9685581	2.2215E-06
SMYD4	0.96851443	0.00089901
PDRG1	0.96704031	1.3429E-10
EPS15	0.96691147	3.689E-06
ABL2	0.96642291	1.0975E-07
LINC00862	0.96413789	0.02401159
TMEM51	0.96269355	1.8575E-08
TFG	0.96259566	2.7057E-13
KLHL17	0.96238213	0.03794685
LANCL2	0.96223649	2.9682E-05
PANX1	0.96183574	2.9636E-07
ANTXR2	0.96173175	8.4966E-09
CREB5	0.96113567	4.6758E-13
ST3GAL1	0.96096244	2.5324E-07
STARD4	0.95804904	8.8829E-06
SFMBT1	0.957977	0.00020301
UVRAG	0.95753311	8.0243E-10
PKD2	0.95696058	0.00091526
FOXP1	0.95652881	7.4268E-08
RABGAP1	0.9552385	2.049E-09
HABP4	0.95423317	0.02982727
VAMP5	0.95402347	1.5067E-08
ENSG0000028	0.95396433	0.02198121
ZNFX1	0.95364639	7.4268E-08
TMTC2	0.95316705	0.00010286
APOL2	0.9526456	0.00143214
CREG1	0.95219979	0.00033285
RRAS	0.95174534	7.2759E-05
TMEM164	0.95066806	8.6785E-05

NUDT14	0.94953844	0.00012026
PM20D2	0.94929011	0.03501409
GORAB	0.94853069	0.0003677
PPP3CA	0.94815911	0.00273199
TK1	0.94735814	0.01246338
ZNF775	0.94581494	0.01036329
ZNF532	0.94545205	2.0782E-05
TBC1D30	0.94399833	0.00035479
ATXN1	0.9433797	4.2759E-05
DDB2	0.9423261	0.0001803
PELI2	0.94147667	0.0019733
BLTP3A	0.94039436	2.9972E-06
GALC	0.94017007	2.1019E-08
SPECC1L	0.94008465	0.00046878
KLHDC7B	0.93860589	0.00025945
RIPK2	0.93805962	1.9488E-33
RFFL	0.93802366	0.00050773
XKR8	0.93801011	0.00436955
PTPRO	0.93710138	0.02364562
ZNG1B	0.93578616	1.4795E-14
ARL6IP5	0.93575823	3.6475E-28
NCR3LG1	0.93574704	0.0047879
GLIPR2	0.93454831	3.7518E-13
FYTTD1	0.93453492	3.403E-24
HEATR6-DT	0.93201373	7.0173E-05
NPIPB13	0.9308918	0.00106296
ASMTL	0.93039016	5.4258E-11
SIRPA	0.93005738	0.00023006
PIK3R5-DT	0.92916871	0.02209732
STK3	0.92882657	1.1879E-07
FDPS	0.92881825	1.9454E-11
SQOR	0.92849537	2.451E-09
DUSP8	0.92817926	0.00378694
PKD2L2-DT	0.9269924	0.00796661
PUS10	0.92627625	0.00395674
WDR59	0.92595135	0.00026214
EPM2AIP1	0.92583903	5.0555E-09
SPECC1	0.92500611	0.00019848
TRAF3	0.92495698	6.0858E-15
SC5D	0.92346957	3.5691E-06
GOLM2	0.92238909	1.0198E-10
FBXL8	0.92193919	0.00093515
BAZ1A	0.92165971	3.5743E-25
DERL1	0.92066824	1.6294E-17
C17orf49	0.91971841	7.1921E-23
MAK	0.91939295	0.01373674
APC	0.91861018	6.7685E-09
CATSPER2P1	0.91826759	0.00817236
ZNF43	0.91502311	0.00032855
H2AZ1	0.91449334	2.5764E-07

ECE1	0.91360768	0.02884395
KDM5B	0.91345446	2.3596E-06
FBXO6	0.91326974	7.291E-07
CSNK1G3	0.91259057	0.00124224
KSR1	0.91221362	2.3222E-06
ZFAND5	0.91190572	3.1244E-09
USP20	0.91182154	0.00010299
SENP1	0.91164914	2.7242E-06
MB21D2	0.91035922	0.00078843
ENSG0000027	0.91025971	0.00239971
FANCL	0.90994779	0.00128444
SCIRT	0.90991148	0.00425088
CCM2	0.90783417	2.5754E-08
RALA	0.90674322	4.1982E-07
B3GALT4	0.90668447	0.00020314
SLC35E3	0.90608181	0.00090173
AEBP2	0.90598825	2.7085E-08
ARL8B	0.90547732	4.2265E-19
CAMSAP1-DT	0.9051531	0.02970047
PPP3CC	0.9044105	9.1323E-07
BCL2L11	0.90332044	3.8434E-09
ZNF767P	0.90305345	0.00058579
LIG4	0.90276908	0.00074507
TMEM120B	0.9023374	0.00089652
BTG1	0.89988389	3.4166E-07
NPIPB4	0.89872118	0.00555873
GADD45B	0.89783412	3.1667E-13
TOMM34	0.89777504	5.1162E-07
PATL1	0.89617737	5.4869E-09
NFE2L1	0.89547124	0.00025428
HLA-F	0.89526435	1.0296E-17
UBE2F	0.89500178	5.3682E-07
BNIP3	0.89451823	0.00405703
BZW1	0.89404016	1.5423E-06
PRR14	0.89339835	2.4727E-08
APOL4	0.89316251	0.00198998
SNX24	0.89277855	0.00794637
HELB	0.892742	0.00047305
ARPP19	0.89142307	1.6819E-07
SERPINB9	0.89050965	3.0012E-06
TMC8	0.88975115	8.7789E-07
ENSG0000029	0.889355	0.03087167
SIAH1	0.88871119	1.6282E-05
ENSG0000025	0.88753021	0.02017597
ZNF136	0.88749421	0.00121209
YWHAQ	0.8872196	2.161E-09
LRFN4	0.8866658	0.0045196
ZNF267	0.8845318	9.7626E-09
PLEKHA1	0.88383231	0.00183033
WBP4	0.88310914	1.9479E-07

ASAP1	0.88237777	4.0521E-10
ACAD11	0.88163281	0.00295284
CDC42EP1	0.88067249	0.0117287
PHF21A	0.87915047	3.2305E-05
PIGX	0.87841827	6.8506E-08
ZMYND15	0.87775748	0.0005373
CSNK1G1	0.87693087	5.4092E-05
MARF1	0.87685088	1.3319E-05
NSUN7	0.87625064	0.00397512
TANC1	0.87618925	0.03992451
MAP1LC3A	0.87512641	0.00018928
IL3RA	0.87504254	0.00048629
FGGY	0.87444673	0.00013632
GABARAPL2	0.87421403	3.454E-17
SELENOS	0.87419742	3.1452E-14
TAB2	0.87394658	4.4961E-07
ULBP2	0.87371954	0.04903158
MRAS	0.87118945	2.964E-05
MALAT1	0.8707105	4.34E-13
CEP68	0.8700385	0.00020301
RABGAP1L	0.86876434	3.9897E-05
UBE2E1	0.86708652	1.1053E-20
PLEKHF2	0.86638883	1.1329E-07
PLEC	0.8648196	2.9604E-05
ARHGAP31	0.86443995	4.878E-05
TMEM185A	0.86437481	0.0079641
HLA-DOB	0.86354219	0.00166393
SAMD8	0.86346739	1.038E-06
OAS3	0.86313811	0.00455456
STARD4-AS1	0.86313542	0.03825544
GPAT4-AS1	0.86215449	0.01084049
ENSG0000029	0.86090117	1.9027E-07
DEXI	0.86073099	0.00383739
CMTM6	0.86022793	3.0562E-13
TCF4	0.85984251	0.01038402
JUP	0.85978787	0.0100204
AKT1S1	0.85943096	6.8651E-05
MYO1E	0.8579864	0.00012516
FBXO30	0.8558426	0.00130299
CAMSAP1	0.85567572	1.2704E-06
ZNF778	0.85545324	0.03227745
ANXA6	0.85449395	0.00021375
ENSG0000027	0.85387868	0.0234904
CCNYL1	0.85341429	1.81E-05
RAB21	0.85165018	1.4684E-15
ENSG0000027	0.8514185	0.03650723
PITPNB	0.85125026	5.2924E-07
ENSG0000028	0.85110679	0.01571522
CCDC66	0.85032226	0.01638605
NRAS	0.85024242	1.5791E-07

SCYL3	0.84982121	0.00534007
PIGA	0.84846988	4.6302E-06
SMC5	0.84831596	4.4491E-10
DSC2	0.84773897	0.00034197
CCDC50	0.84579035	0.00030806
RASSF2	0.8451067	2.9588E-08
ALDH2	0.84486079	8.6344E-09
CHD6	0.84463321	9.7741E-11
FMNL2	0.84460426	0.0319073
LAP3	0.84457575	4.2656E-08
ZNF280C	0.84455275	0.04493567
SEPTIN11	0.84422973	1.8819E-08
GCH1	0.84415834	0.00010852
TTYH2	0.84296537	1.4342E-06
SLC25A25	0.84276865	0.00021188
NIPA1	0.84078113	0.00783929
CPNE2	0.83930167	0.00797399
TRPM4	0.83900035	0.0003506
HUWE1	0.83835518	0.00010877
SPPL3	0.83800605	9.7839E-09
WHAMM	0.83785972	2.6055E-09
PML	0.83743319	1.4342E-07
IL1R1	0.83581243	0.0076353
BASP1	0.8347112	2.1538E-09
MELTF	0.83367117	0.00657441
JAK2	0.83351323	3.6831E-15
KMO	0.83102759	0.0011697
RAPGEF6	0.83024715	2.7496E-06
PLAAT3	0.82970615	0.03519834
MIR22HG	0.82866937	7.5613E-05
MAP2K4	0.82785628	1.4598E-07
MIR3945HG	0.82758726	0.02326138
DOCK10	0.82610188	1.9628E-07
FAM118A	0.82481702	1.7905E-08
DUSP4	0.82454869	0.00077738
MTMR9	0.82355078	0.00043528
CROCC	0.8233211	0.04612479
WTAP	0.82272211	2.0663E-09
LIMK2	0.82187811	0.00048867
XIST	0.82115675	0.02556109
AARS2	0.8200409	0.03087179
NCAPG2	0.81857604	0.038272
SAMSN1	0.81756173	5.6437E-05
ILDR1	0.81735894	0.03328363
TYMP	0.81692728	2.0072E-08
MPZL1	0.81620941	0.01165618
SBF2	0.81599154	1.1389E-07
CYP51A1	0.81563667	1.8719E-06
ABHD17B	0.81376662	0.00011213
TSC22D2	0.81130777	1.9226E-12

ANAPC10	0.81096242	5.3629E-05
DHRS7B	0.8103962	0.02145122
HIVEP2	0.80877744	1.2819E-10
TSFM	0.80780074	0.00317076
SHFL	0.80763841	1.4732E-07
UHRF2	0.80605847	0.0001066
ARL5B	0.80576816	3.739E-06
RHBDF2	0.80505735	1.0112E-05
ANKRD6	0.80471412	0.01693182
ZFYVE1	0.8042148	0.01087561
ZEB2	0.8041027	4.8674E-08
SUPT3H	0.8034045	0.00010085
SLC2A1	0.8034007	0.00171026
APOLD1	0.80309218	0.03825682
RIC1	0.80136967	9.3789E-09
CD86	0.79831434	3.0152E-10
CFLAR-AS1	0.79802917	0.00053059
PARP9	0.79760911	0.00021088
IL10RA	0.79751868	9.8919E-12
GK	0.79695523	3.5246E-08
ABCA7	0.79686781	0.00126307
ABCC1	0.79478869	7.4497E-07
IKZF1	0.79478561	2.0606E-08
ACOT9	0.79401805	9.9647E-07
ICAM1	0.7939996	6.3285E-05
ZNF296	0.7935686	0.00680132
MDM1	0.79249155	0.03464662
SIL1	0.79223963	4.4678E-11
FILIP1L	0.79110763	1.1561E-05
SLCO3A1	0.79045563	3.1494E-05
DNAJC18	0.78912345	0.00806658
KPNB1	0.78896676	2.3728E-09
TBC1D17	0.78864162	0.00032692
NCK2	0.78814771	0.00159153
PTTG1	0.78743784	0.00062125
ZBED6	0.78721258	0.00030838
OSTF1	0.78653704	2.0088E-09
RAPGEF1	0.78563592	3.7221E-07
ARL6IP6	0.78503478	0.00011231
SNHG15	0.78473148	9.5387E-14
RRAGD	0.78363739	0.00377513
ASH1L	0.78362662	2.2344E-16
ZFP36L1	0.78292542	1.0957E-05
PI4K2B	0.78234069	0.00024043
CLCN6	0.7811459	2.2625E-07
CTLA4	0.78050944	0.01278875
FPR1	0.78010202	0.00696831
ECPAS	0.77881448	2.0616E-06
FXR2	0.77800492	0.00084314
PRKAG2	0.77771133	6.7295E-05

SERPINB1	0.77722755	2.3926E-05
TPD52	0.77720607	0.00011995
CDK17	0.77440463	0.00037581
USP49	0.77359848	0.03702028
FBXL3	0.77322714	0.00011933
VPS37C	0.77193704	2.8458E-05
OAS2	0.76908516	0.00033308
KLF11	0.76880386	0.00049675
DTWD2	0.76867144	0.00259234
SUZ12	0.76856339	0.00022838
SMURF1	0.76738374	0.00012504
ITFG1	0.76737095	0.00028311
TCP11L1	0.76701341	0.00108215
NUP62	0.76588112	1.5555E-06
TMEM41B	0.7651842	5.3861E-06
SECISBP2L	0.76508118	5.3114E-08
SPIB	0.76385593	2.9119E-07
LINC00938	0.76356708	0.00643909
MFSD2A	0.76324001	0.00770515
TOM1L2	0.76235294	0.01793195
LMNB2	0.76201358	5.8111E-06
DCP1A	0.76131248	7.6012E-08
C5orf15	0.76087638	1.7333E-07
RAB2B	0.76085947	0.00017612
CDKAL1	0.76008924	2.8471E-05
HSDL1	0.75990977	0.04720973
MAFF	0.75920345	5.0117E-10
RELA	0.75893764	1.8061E-10
ST6GALNAC6	0.75730023	0.00411446
MAPK8	0.75632696	0.00028658
CCDC82	0.7553629	0.00095901
ZNF407	0.75529911	1.3773E-05
COA1	0.75420329	6.2208E-06
SEC61B	0.75378037	3.1771E-10
TAP2	0.75280462	4.1695E-07
SESN1	0.75109998	0.00019524
HDX	0.74961274	0.02018128
ZC3H12A	0.7479739	5.8352E-05
TXNDC11	0.74750765	4.8925E-08
KDM4C	0.74719401	3.5714E-13
TCFL5	0.74566342	0.01348096
MIER3	0.74500143	0.01507799
BMAL1	0.7446754	0.00015145
RNF103	0.74465639	0.00142272
PTGES2	0.74385268	1.257E-11
PRDM1	0.74354845	0.00015304
IFI30	0.74323109	5.4996E-07
ZNF281	0.74291309	0.00076536
BCL3	0.74026046	0.00070095
DNAI2	0.74020764	0.0003972

LONRF1	0.73996968	2.1742E-06
VPS8	0.73964492	0.00353179
UBE2Z	0.73927268	6.3269E-12
TP53BP2	0.7376244	3.8019E-08
NBPF19	0.7358292	0.00088998
TSTD1	0.7354977	0.0102658
CDK6	0.73465744	0.00034506
CMPK2	0.73427226	0.00954471
RAB3A	0.73400139	0.03578275
SLC9B2	0.73366872	0.00495999
PLD1	0.73332532	0.02983737
CASP10	0.73260483	0.01874776
ZFY	0.73063159	0.00014816
ATP6V0A2	0.72917877	0.00282261
RMI2	0.72818164	0.03048252
OAS1	0.72632284	0.03548761
ZFYVE28	0.72555293	0.04870765
CDC42SE1	0.72507911	1.8054E-07
PAN3	0.72496503	7.626E-05
TANK-AS1	0.72415139	0.00133279
FCHSD2	0.72393059	3.7962E-08
HLX	0.72380137	0.00184743
MX1	0.72371412	0.01528345
CREBL2	0.72207055	1.3647E-05
PPDPF	0.7219928	0.00027962
TLE3	0.72142126	0.00010622
FAM13A	0.72137903	0.01690279
ZNG1A	0.72136448	0.0012276
ZBTB18	0.72057755	0.00016386
UBQLN2	0.72003747	1.4614E-05
BTBD3	0.71948446	0.01397392
MMP9	0.71890819	0.01571463
ARHGEF11	0.71827752	0.02401336
UNKL	0.71810761	0.03985621
WDR44	0.71664411	2.1752E-06
MTHFS	0.71644528	3.1681E-05
ENSG0000026	0.71615735	0.00777024
SLC7A1	0.71473445	0.00024851
ZBTB24	0.71461177	5.9824E-05
RAP2C	0.71398735	0.00071077
KYAT3	0.71329311	6.5178E-05
ENSG0000027	0.71310364	0.04455585
IRF2	0.71307732	1.9909E-05
CD82	0.71289355	0.01458428
XRN1	0.71271184	2.1179E-06
TPRA1	0.71262762	9.7138E-10
CTSH	0.71220007	1.7217E-11
NPEPPSP1	0.71148001	0.00067117
SSBP3	0.71074588	6.1738E-06
RNF19A	0.7101341	2.1126E-07

GFPT1	0.70810331	5.6995E-05
ZNF292	0.70727405	1.257E-11
TRAF5	0.70715546	0.00065244
CAMKK2	0.70585174	4.0586E-05
HERC6	0.70451719	0.04120673
GNPTAB	0.7044644	3.3665E-07
NPLOC4	0.70443757	1.0642E-05
PMAIP1	0.70220648	0.00843448
FBXW5	0.70159572	1.6825E-14
PTPRM	0.69995897	0.0093626
FBXL14	0.69974627	0.02894592
CLINT1	0.69971116	5.0007E-08
POLD1	0.69892486	0.00175128
ZFAS1	0.69830452	2.2488E-06
GAS6	0.69437981	1.0589E-06
BMERB1	0.69419909	0.0440591
RAB10	0.69416032	1.1443E-10
LILRB4	0.69304291	0.00467674
ISG15	0.69251433	0.00274054
MARK4	0.69053232	0.00014829
AVEN	0.6894418	3.877E-05
TET3	0.68885337	8.5595E-05
PHLPP2	0.68859028	0.04011319
WDFY1	0.68832419	0.00024424
TBC1D22B	0.68695455	0.01216106
DCAKD	0.68658604	0.00150438
PARP15	0.68647281	0.02526504
ZMIZ2	0.6857614	5.3898E-10
GRAMD1B	0.68385072	0.00585467
UPRT	0.68372957	0.00052776
ABI1	0.68354442	1.2812E-07
CHFR	0.68263651	8.5208E-06
PHKB	0.681193	0.00155538
FTH1	0.67884895	0.00066752
ENSG0000029	0.67768948	8.0439E-06
KIFBP	0.6773144	0.00211441
LRIF1	0.67718968	0.00504382
IFITM1	0.67717131	0.01003182
CYBRD1	0.67713264	0.03473511
CHMP4B	0.67708384	1.4427E-05
ATF7IP2	0.67510579	0.00027143
JADE2	0.67399911	0.00636351
NFAT5	0.6721474	0.00047112
MTR	0.67183649	0.00489333
PI4K2A	0.6716494	0.01474336
EPB41L3	0.67042006	5.4002E-05
ERGIC1	0.66969055	8.8829E-06
RCN1	0.66933628	1.1597E-09
SLC30A7	0.66912949	0.00115087
RAB30-DT	0.66899923	0.00021665

NUP98	0.66706878	4.7955E-07
KIAA1191	0.66706243	0.00132351
IFITM3	0.66673256	0.00051244
RRN3	0.66457817	0.00857346
MED13	0.66384653	5.1056E-05
AAK1	0.66380218	0.00293452
SUCO	0.66371749	5.893E-06
LGALS3	0.66351548	0.00115086
ZNF618	0.66336537	0.0100856
SELENOM	0.66253364	0.00350877
ARID4B	0.66179824	1.4281E-09
TAPBP	0.66147743	4.1947E-09
RUNX2	0.661471	3.2626E-05
PTK2B	0.66096539	0.00046062
DUSP3	0.66084646	0.00013739
YEATS2	0.66067365	0.00171562
IFI6	0.65999888	0.00120234
ALG2	0.65932203	2.9699E-06
PFKP	0.65913474	0.00030854
LIMASI	0.65893492	0.01204773
STARD7	0.65775674	7.649E-08
CD44	0.65724894	0.00030119
BET1	0.65721646	0.00946126
ZNF701	0.65721439	0.02011392
BCOR	0.6564926	0.00634223
MALSU1	0.65562332	0.00987141
AMMECR1L	0.65538483	0.01760757
UBXN2A	0.65482772	0.00016788
KCTD10	0.65424432	0.00471761
ATF7IP	0.65408023	0.00041555
MYL5	0.65397658	0.00758388
KCMF1	0.65226598	3.1257E-06
MAD2L2	0.65187615	1.0062E-07
KIFAP3	0.65169195	0.0090696
LINC-PINT	0.65157453	5.6401E-07
TRIM24	0.65094295	6.1721E-08
ENSG0000028	0.65089768	0.03632163
PTPN2	0.64894685	7.3713E-09
STX2	0.6481808	0.03558407
ATL2	0.64754725	4.4273E-06
TBC1D10A	0.64738487	0.00274394
EHD4	0.64708087	0.00197628
SZRD1	0.64696813	6.3782E-07
SLC9A8	0.64623451	0.00027093
ANKRD13A	0.64389314	1.8596E-05
CDC42SE2	0.64354636	8.6411E-12
DHRS12	0.64346404	0.02105327
SELENOI	0.64245043	0.00183865
DENND3	0.64139986	0.00070676
TMEM50A	0.64121516	9.6898E-09

ABHD12	0.64067516	4.06E-08
SLC30A4	0.6400397	0.03639289
ABHD3	0.63902057	0.005032
SMC6	0.63855232	0.00796661
PAK4	0.63820139	0.04131773
SLC2A13	0.63681464	0.0011486
N4BP2L2	0.63604597	1.7662E-08
NSF	0.63469302	0.00065271
MAILR	0.63338381	0.00546389
NR4A3	0.63322011	0.00067301
BCL11A	0.63245464	0.00076507
KLHL28	0.63228735	0.00951747
MARCHF7	0.63197785	7.5067E-08
CARD16	0.63196352	2.6415E-05
HSPA13	0.63186003	0.0006007
B2M	0.63182091	3.4899E-09
STK35	0.63116152	0.04969931
ZNF76	0.63106097	0.00085502
PLEK	0.63031614	0.00184476
CNOT4	0.63028351	1.7368E-05
CXCL8	0.63004631	0.00558923
KRAS	0.63004134	1.8416E-07
ETV6	0.62921892	3.165E-08
RAB11A	0.62921845	9.8435E-11
AHCTF1	0.62810185	0.00021026
PLAU	0.62808244	0.01962694
G3BP1	0.62796558	1.1825E-12
PATL2	0.62792449	0.00352564
ENSG0000025	0.6274023	0.04985258
ACSL4	0.62733028	0.0027133
TOR1AIP2	0.6269732	0.00032248
IGHMBP2	0.62585504	0.00034635
ANKRD36C	0.62543024	0.00388692
ZC3H12D	0.62524161	0.00921512
LRRC57	0.62523409	0.01300035
KLHL21	0.62506845	0.02768861
ENSG0000024	0.62327906	0.0140764
BCAS3	0.62255276	0.01083145
NOCT	0.62233805	9.5556E-05
ZFAND3	0.62205044	2.2215E-06
CAMK1D	0.62168742	2.5265E-07
HMOX1	0.62145576	0.02333933
APPL1	0.62107964	3.8662E-08
HYOU1	0.62061207	0.00599749
ANXA2	0.61899586	0.00027809
IPPK	0.61896393	0.01930463
ORC3	0.61812546	0.00834163
INTS3	0.61791811	0.000446
SH3KBP1	0.61760108	0.00273342
KCNQ1OT1	0.6175103	0.03301972

ZNF274	0.61648704	0.00773079
MDC1	0.61640912	0.01382311
CAPG	0.61578274	0.02609001
ENSG0000028	0.61466255	0.00096951
ENSG0000028	0.61447945	0.00835776
LAMP2	0.61424891	1.1891E-10
ISOC1	0.61364654	0.00248
PHF1	0.61336793	0.00331071
CLSTN1	0.61319267	0.00542403
DOT1L	0.61314875	0.0064819
MICA	0.61290904	0.02932697
C6orf62	0.61122408	0.00047952
JMY	0.61109089	0.01768967
DHX34	0.60963555	0.01762469
FLNA	0.60927097	0.00758388
WNT2B	0.60910748	0.03151171
SVIP	0.60882671	0.03317038
CHSY1	0.60844276	0.00633274
MMAA	0.6072377	0.01499216
PRICKLE3	0.60692812	0.02662433
STX17	0.60665408	0.00489055
UBR4	0.6066237	0.00234961
LRRC28	0.60443108	0.01542793
PANK3	0.60406554	0.00454386
TSPOAP1-AS1	0.60395798	0.00099738
MSH6	0.60360756	0.00277377
CTBP2	0.60280903	0.00020379
GDI1	0.6024111	0.00027685
LACTB	0.60089711	7.718E-09
ADCY9	0.60052226	0.00690825
RPS6KA2	0.60037587	0.04138756
CLK4	0.60014717	0.00052447
SRGAP2C	0.59956438	0.00156929
IFNGR2	0.59925314	3.3452E-13
CYTIP	0.59760196	7.6786E-05
STAT3	0.59759393	2.2946E-05
STIMATE	0.59736119	0.00810039
TALAM1	0.59668498	0.0010314
RIPK1	0.5965181	0.00052643
ACTN4	0.59628955	0.0013516
PLAAT4	0.5960843	0.00051287
PDPK1	0.59560268	1.1585E-06
TAF5L	0.59490911	0.00432544
KHNYN	0.59457156	0.00077569
ARID3A	0.59326049	0.02497026
ASXL1	0.59317217	0.00047242
DUSP16	0.59283192	0.00619059
GPR137	0.59219189	0.00046985
DUSP22	0.59120443	4.9527E-05
PPP1R2	0.58875699	6.9505E-11

ANKRD11	0.58837362	2.6226E-06
ADAM8	0.58795644	1.3201E-06
MYO5A	0.58775994	0.0098911
IRF7	0.58763364	0.00156433
GOSR1	0.58745863	2.3331E-06
ZNF513	0.58721003	0.01337193
APH1B	0.58704977	0.00721865
SELPLG	0.58480787	0.00426002
SLC6A6	0.58254936	0.00180497
FAM222B	0.58180573	0.04120971
HLA-A	0.5814139	1.393E-23
TRIM21	0.58050108	0.03199909
SLC25A43	0.58040151	0.00450317
NOP58	0.57996137	2.0139E-05
RFXAP	0.57960013	0.03889931
INF2	0.57946789	0.04324859
PAPSS1	0.57930276	2.5271E-06
CTNNA1	0.57859642	3.6355E-05
SERPINF1	0.57855704	0.022336
UBXN7	0.57816794	0.00040892
KMT2A	0.57787629	0.00108957
GPAT4	0.57780537	2.2946E-05
COMMD2	0.57720845	0.00573748
WDTC1	0.57717976	0.0202284
WDR48	0.5768514	6.4188E-06
NELFB	0.57651117	9.6754E-07
ZSWIM8	0.57649165	0.00785143
WDR33	0.57629701	5.862E-12
VEGFA	0.57571398	0.02047152
DYNC1I2	0.57453596	0.00150026
TBK1	0.57448057	1.3468E-07
GXYLT1	0.57405468	0.00295467
ANKMY2	0.57353092	0.04058901
NFKBIZ	0.57305354	0.00041985
MED21	0.57272317	0.00387291
PPP1R18	0.57256285	3.9046E-09
HCP5	0.57232794	0.00494833
RNF114	0.57175422	3.6338E-05
ATG13	0.57056273	0.00238285
VRK2	0.57012486	1.686E-05
RTL8A	0.56780539	0.03839233
TNFAIP3	0.56771758	1.399E-05
TYW1B	0.56731246	0.01572918
JOSD1	0.56574871	0.000199
TRPM7	0.56565863	0.00249075
TAOK3	0.56505883	1.8696E-05
ERO1B	0.56406451	0.00015585
TYW5	0.56399779	0.03526182
CD63	0.56340264	7.8889E-08
DNAJA1	0.56326625	0.00043489

FAM117A	0.56290014	0.01271456
APMAP	0.5628102	7.2484E-07
CEBPG	0.56280732	0.00084183
PAX8-AS1	0.56256293	0.02899988
PCGF3-AS1	0.56119243	0.01676681
TRIP4	0.56047593	0.01960723
KLF6	0.55883627	0.00021479
ATXN7	0.55879944	6.7855E-05
RFX2	0.55877478	0.027324
HECTD4	0.55875335	0.00559253
TRAPPC10	0.55790496	3.6291E-06
MBD5	0.55599312	0.00144377
BTBD7	0.55590346	2.4838E-06
RAD1	0.55486245	0.01630836
POMGNT1	0.55420808	0.01995044
CD81	0.55311259	0.00036341
MTHFD2	0.55215874	0.0010297
PFKL	0.55130144	8.9723E-07
TES	0.5512826	2.3124E-06
DDX6	0.55102465	4.5752E-06
NFKBIE	0.54839999	0.00034156
USP45	0.54817824	0.03590228
TMCO3	0.54796566	0.02989549
DMXL1	0.54788702	0.00618645
TJP2	0.54672856	0.0231247
KBTBD2	0.54647861	5.9113E-05
MACF1	0.54585068	5.7629E-05
CCND2	0.54549246	0.00184743
PRXL2C	0.54507308	0.00078926
PPTC7	0.5445962	0.00247221
STRN3	0.54447386	0.02196767
SLC20A1	0.5428657	0.00021371
RFX5	0.54281587	0.00024098
TPCN2	0.54235901	0.03072092
TOR3A	0.54189547	0.00186377
RGS1	0.54174734	0.00018776
DHDDS	0.54124839	0.0045494
CRYBG1	0.54055959	9.7608E-06
MVP	0.54054874	4.3946E-07
WASHC2A	0.53990915	0.0361585
ODF3B	0.53975489	9.7403E-10
AP1S3	0.53919232	0.00867922
FAM241A	0.53913791	0.00079658
HBP1	0.53784644	2.6121E-05
RBM19	0.53783037	0.00241847
RAB27A	0.53767258	0.03151902
DTNBP1	0.53737356	2.3124E-06
WASL	0.53706119	0.03213412
ENSG0000029	0.53613611	0.02249508
R3HCC1L	0.53542234	0.0016936

SLC39A1	0.534254	3.0348E-05
EXOC2	0.53020345	0.00220718
PIM2	0.52942874	0.01817791
MAML1	0.52912968	0.00542323
IST1	0.52905444	1.4183E-07
IKBKG	0.52860558	1.6514E-05
LDHA	0.52843531	0.00031223
DNAJB14	0.52781336	2.0125E-06
OSGIN2	0.52691042	0.00669003
SERTAD2	0.52676036	0.00207289
BAZ2A	0.52633967	6.2896E-05
KHDC4	0.52623645	0.00768924
LAMTOR3	0.52618148	0.00027709
FMNL1	0.52617529	0.00937317
KDM6A	0.52542798	0.00172907
TRNAU1AP	0.52520333	6.2659E-05
HTATIP2	0.52257466	0.00150333
CLCC1	0.5225399	0.00987625
UXS1	0.52128187	0.01995044
WIPF1	0.52121909	1.2184E-05
IL6ST	0.5212041	9.7229E-06
SMIM3	0.5203698	0.00307692
ATF3	0.52008219	0.01136746
DLD	0.51953482	4.4252E-06
TAF4B	0.51945635	0.0417746
EED	0.51926817	0.00216304
ZNF746	0.51860747	0.00824023
BMP6	0.51849202	0.0360296
USP53	0.51796782	0.00548367
SOCS3	0.5169201	0.00865639
FXR1	0.51683167	0.01400056
GNG5	0.51543132	2.5723E-07
ARRB2	0.51525662	6.0761E-08
BTBD6	0.51421469	0.04839586
PHIP	0.51413635	0.00055048
PARP11	0.51375376	0.00881527
ZC3H4	0.51365919	0.00512664
ENTPD7	0.51308811	0.03757226
RRN3P3	0.51293217	0.02315396
IER5	0.51261351	0.00188328
PNKD	0.51259436	0.01124515
LPIN2	0.51250595	2.6506E-05
GNB4	0.51237129	0.01089643
LSP1	0.51234146	9.7839E-09
ATP2C1	0.51207343	0.0020792
BMPR2	0.51180425	0.00858325
SOS1	0.51158287	0.0001852
KCTD13	0.51112487	0.00691036
DTX3L	0.5109477	0.01095982
EMC2	0.51043167	0.01634989

STOM	0.50987129	0.0053396
CC2D1B	0.50969531	0.02656398
MCU	0.5087844	0.02442146
SLK	0.50617747	0.00016575
ATAD2B	0.50574811	3.4446E-05
NUP54	0.50566415	0.00050551
NFKBID	0.50484508	0.00779401
TRIP12	0.50430213	1.9761E-07
SMARCE1	0.50399334	1.7927E-06
IL6R	0.50386261	0.00060976
PSMD5	0.50292284	0.03757226
DYNC1LI1	0.50226021	1.3331E-07
RASGEF1B	0.50183479	0.0454474
RIT1	0.50150727	0.00777849
ACTN1	0.50064172	0.00358755
CDC42	0.50057991	1.3182E-10
PSMA2	0.49884589	5.7629E-05
MCM5	0.49849595	0.00031549
CNP	0.49800688	0.03144515
GALNT11	0.49775014	0.00868497
CHST11	0.49750032	0.00104631
PDCD5	0.49740379	3.4447E-05
LPCAT1	0.49596615	0.00656841
RASGRP3	0.49479227	0.01482904
PTGR3	0.49442676	0.00926049
RNF11	0.49414817	0.00166878
ACOT7	0.49187369	0.00934319
SH3GLB1	0.49175814	9.777E-07
SLC36A4	0.49148758	0.01155925
SCAF8	0.49122882	0.00230158
CLN5	0.49066719	0.01761078
TRIM56	0.48812036	0.02074356
ATP6V1B2	0.48798748	0.0256122
PTPRJ	0.48761376	0.00568247
TNS3	0.48759984	0.00353179
FBXO22	0.4874592	0.04265502
AMDHD2	0.48721217	0.00372502
PTBP2	0.48676857	0.04072904
ZFC3H1	0.4859048	1.5344E-05
ILKAP	0.48586893	2.7688E-06
SLC25A53	0.48572631	0.03904837
DNAJC1	0.48446122	0.00015728
INO80D	0.48358864	0.03728518
ENSG0000023	0.48350434	0.02018123
MED16	0.48302543	0.00473039
MTRFR	0.48141252	0.04865514
OTULIN	0.48001524	0.00235858
UNK	0.47977635	0.04144024
ACP2	0.47921513	0.03485456
HEXIM1	0.4791961	0.01203892

REEP3	0.47910923	0.00601307
MR1	0.47902873	0.00102632
ADIPOR2	0.47800741	0.00082887
HMGCR	0.47784965	0.02259353
RAD21	0.47776132	0.00274365
TRA2A	0.47741107	0.00084183
KATNBL1	0.47673647	0.00215194
APOL6	0.47662586	0.01184698
TMBIM1	0.47622743	0.00020495
SLC25A24	0.47510117	2.8864E-05
GATAD2B	0.47473677	0.00105693
PDCL3	0.47409496	0.03739761
H2AC6	0.47349914	0.00795144
DDHD2	0.47316564	0.0447462
PPP1R12A	0.47235261	1.9531E-06
ABHD13	0.47198521	0.01965448
CD38	0.47192384	0.00174322
CERS5	0.47126519	0.0040623
FOXJ3	0.47080984	0.00191067
LINC01473	0.47067381	0.02312566
LYN	0.46985801	2.0916E-07
LCP1	0.46913749	0.00016146
FLOT1	0.46888765	5.0623E-07
SRP54	0.46868468	0.00017166
PITPNA	0.46853688	0.00011446
USP11	0.46784914	0.00161095
SPART	0.46681908	1.1481E-06
NUP188	0.46679088	0.03571217
MFSD14A	0.46643974	0.00400806
DPH3	0.46633737	0.00420507
RANGAP1	0.46564717	0.00283775
PIK3C3	0.46537202	0.03985785
ARL6IP1	0.46401249	0.00019723
MIB1	0.46363538	0.01573061
KIF1C	0.46308259	0.04407873
SLC43A2	0.46285286	0.00389241
APP	0.46279172	0.0135979
MICAL1	0.46213475	0.00906077
DDX59	0.46207892	0.00881625
VPS13B	0.46206479	0.00067861
RHEB	0.46169385	1.4953E-05
NR2C2	0.46145646	0.00250988
MAGED2	0.46116201	0.0293013
ADNP2	0.46112752	0.03560802
DHX40	0.46097543	0.01902353
DCAF15	0.46083308	0.02114854
RNF121	0.46078531	0.03473511
TUBB4B	0.4605889	0.00641902
ANKRD44	0.46020563	0.00140072
SP2	0.45960971	0.00425378

TSPAN14	0.45947407	0.02467595
FARP2	0.45943211	0.01087372
PSME2	0.458697	0.00124957
GUCD1	0.45801572	0.01614504
PPP2R3C	0.45790464	0.00051037
LITAF	0.45713532	0.00273557
GPR183	0.45481266	0.0001615
SIPA1L1	0.45478243	0.00958934
LY6E	0.45451488	0.0026733
SBDS	0.45437123	0.00101071
ENSG0000027	0.45420833	0.02014647
GPR141	0.45417469	0.00502375
HAUS2	0.45318188	0.00407326
RRBP1	0.45295388	1.1908E-09
KDM2A	0.45284899	0.00010363
EGLN1	0.45222163	0.01153269
CYSTM1	0.45221126	0.00072518
STK26	0.45139655	0.02183526
MRPL14	0.4513831	5.8842E-06
VPS53	0.45080925	1.6101E-05
GTF2E2	0.45022424	9.0635E-05
KLF13	0.44993305	2.1569E-05
GNA15	0.44973175	0.04545342
USO1	0.44769733	4.4065E-08
GALNS	0.4472366	0.04221853
VTI1A	0.44685064	0.00073683
CNOT1	0.44671794	0.00158445
NADK	0.44643669	0.01006651
CDC73	0.44628412	0.00012924
VEZT	0.44614384	0.01156138
ORMDL2	0.44475746	0.00013935
FAM177A1	0.44455572	0.00206632
CALM3	0.44433759	0.0006122
IFI35	0.4421437	0.02144974
FAM98B	0.4421107	0.04150639
MLLT10	0.44177057	0.00059493
MECP2	0.44122428	0.01245553
EDEM1	0.44099738	0.01221508
CIITA	0.44027984	0.00066165
ATL3	0.44020042	0.0106389
KPNA1	0.43973341	0.00104101
MGRN1	0.43934196	0.00148194
RAB18	0.43908381	0.00119925
USP9X	0.43904355	9.0864E-06
HBS1L	0.43851393	0.00157874
SBNO1	0.43816683	1.1197E-06
MOGS	0.43712724	0.00258803
CTSC	0.43691413	0.00245763
PPRC1	0.43665814	0.03985785
ITPRIPL2	0.4363638	0.01986576

CXCL16	0.43558924	0.02609108
PRKCE	0.43558327	0.02295526
LARP4	0.43487885	0.00022517
ENSG0000028	0.43473311	0.01645304
RP2	0.43420434	0.00163776
HIF1A	0.43346628	0.02095431
FOXK1	0.43297965	0.01399733
CHM	0.43282509	0.0295208
ATG7	0.43272223	0.03263154
HIPK1	0.43074692	0.00024617
BACH1	0.43032715	0.00231779
ITPRID2	0.43030455	7.4672E-05
PRKAA1	0.43026473	0.03856789
CELF2	0.42923921	0.00057765
IPO9	0.42797367	0.01141732
PPP1R9B	0.42644829	0.01087372
PPP2CB	0.42610696	0.0032342
LINC00513	0.42469945	0.02982915
KAT7	0.42455653	0.03253215
MED14	0.42398586	0.01160783
FAM135A	0.42366181	0.04598418
RASSF3	0.4234352	0.00509265
HLA-B	0.42321841	6.3535E-09
ITGAV	0.42313048	0.0291981
FZR1	0.4228183	0.0489633
DYNLT1	0.42239049	5.8319E-05
SNHG12	0.42158897	0.01625404
ZRANB1	0.42072141	0.03356864
CLCN7	0.42057612	0.00210068
BUD13	0.42011161	0.03825544
RC3H2	0.41987815	0.01458428
ACTR10	0.41891148	0.00430283
TMOD3	0.41786901	0.00012924
SQSTM1	0.41667282	1.3715E-05
OSER1	0.41626871	0.00661905
FKBP5	0.41503335	0.0047782
DAPK3	0.41413434	0.03058447
SLAMF7	0.41364911	0.04138137
JARID2	0.41300469	0.00026766
RAB35	0.41116307	0.00200885
CAND1	0.41102191	0.00273075
ELOA	0.41058081	0.00381647
ZMYM4	0.41039043	0.00926074
PPM1B	0.41037751	0.01414308
WASHC2C	0.40977065	0.00908489
ELOVL1	0.40937457	0.00019701
ERC1	0.40871121	0.00850407
MMGT1	0.40847565	0.0145887
LCOR	0.40766779	0.01001066
KLHL6	0.40753361	0.00685811

NRF1	0.40708078	0.01445181
RARS2	0.40700113	0.01597845
SAMD4B	0.40602433	0.00152537
TXNL1	0.40570756	2.9147E-06
PDS5B	0.40471078	0.01658887
WBP11	0.40363749	0.00235928
POGZ	0.40355849	0.00509053
UBR5	0.40234399	0.00286557
RBM47	0.40218563	0.00580003
RAB6A	0.40217727	0.00788848
RPS6KC1	0.40183258	0.0009014
PTPN9	0.40163188	0.00441117
C3orf38	0.40044873	0.00696831
CYLD	0.39959572	0.00038961
CHMP1A	0.39942015	0.01272538
TMEM30A	0.39902831	0.00853079
RALGAPB	0.39884837	0.03075885
SRI	0.39872737	0.00019143
SIN3A	0.3985523	0.02003298
WNK1	0.39745712	0.02425131
NCOR2	0.39732217	0.00052286
ERAL1	0.39674494	0.00514013
PEF1	0.39672905	0.00015989
RFC2	0.39600939	0.01031861
SNX6	0.39425587	0.00029256
SELENOT	0.39377523	4.266E-06
HOOK3	0.39307003	2.6745E-06
ATG5	0.3928267	0.00183678
UBA6	0.39282667	0.00179588
ARL8A	0.39135048	0.00060625
KHDRBS1	0.38998681	4.1183E-07
ANKRD28	0.38961818	0.02242407
TMSB10	0.38936913	3.9455E-08
PBX3	0.38890677	0.04673318
SRSF11	0.38874345	5.2681E-06
CCNT2	0.38866205	0.00380584
ANKRD12	0.38704367	0.00113942
ESYT2	0.38657134	0.01095982
SNX14	0.38585801	0.03003414
GOLGA7	0.38555549	0.00088859
YKT6	0.38506355	0.01656912
PSPC1	0.38477463	0.01513881
TASOR2	0.384557	0.0217787
MBD1	0.3841309	0.03470906
NIT2	0.38280341	0.02699816
PNISR	0.38277811	0.00378694
TNFRSF14	0.3822878	0.00600778
PDE4B	0.38191507	0.01857287
PBRM1	0.38175733	0.00128444
EIF4G2	0.38164649	4.247E-09

PPP4R2	0.38162685	7.599E-05
XPO4	0.3812065	0.02301347
ARHGAP45	0.38116325	0.00037581
PIP5K1A	0.38003501	0.04042021
MYO9A	0.37946471	0.03055146
NUBP1	0.37932054	0.00026127
MBD2	0.37901789	0.0003636
ZNF410	0.37880104	0.01134535
GNB1	0.37823642	1.6809E-05
STX11	0.37709913	0.00018458
ADAR	0.37625551	0.02609108
NCOA5	0.37624519	0.02403407
ATP6V1G1	0.37534944	0.00065227
CALHM6	0.37510419	0.04370347
MED15	0.37491065	0.00040018
AGPAT3	0.37433626	0.00259603
UBB	0.37429614	0.0003511
TBL1XR1	0.37389303	0.00034832
SNX8	0.37345765	0.02135878
TGIF2	0.37301893	0.00727023
TAOK1	0.37257173	0.04755378
PDXDC1	0.37245324	0.0217394
RNF34	0.37197367	0.01228029
RNF2	0.37119573	0.03442375
RNF216P1	0.36962442	0.02223996
SMARCA5	0.36876794	0.00050825
MAP2K1	0.36850384	0.0129078
ASCC3	0.36809223	0.01120838
UBE2A	0.36779564	0.00147448
IDI1	0.36714344	0.03851333
CHMP5	0.36611973	0.00161993
TAMALIN	0.36606139	0.01476266
ELF2	0.36556243	0.00151906
VAPA	0.36550299	0.00071134
MAN2A1	0.36545798	0.03606909
SLC17A5	0.36541772	0.03475329
GOLT1B	0.3649706	0.00546802
UBE3A	0.36310359	0.00038561
SELENOW	0.36309437	0.00242819
ZFAND6	0.36276716	0.00076301
DEDD	0.36225995	0.00839366
STK11	0.36211347	0.01733965
PRRC2C	0.36115866	9.2357E-07
TMEM87A	0.36091301	0.00784661
NPEPPS	0.36037628	0.00231779
NFE2L2	0.36017722	0.0059328
MLH1	0.35947363	0.0440591
RPA2	0.35941517	0.01728983
PTMS	0.35931441	0.01662575
FRMD8	0.3593021	0.02425654

EIF1	0.35758958	0.00020846
HAX1	0.35757566	0.00851954
CREBBP	0.35720188	0.00083732
CIRBP	0.35717212	0.00179984
USP42	0.35563367	0.02360614
PGD	0.35550695	0.00358903
ABRAXAS2	0.35525991	0.02091722
STXBP3	0.35514931	0.03068505
DOCK1	0.35512216	0.03442375
KLHL24	0.35504033	0.02254245
KLC1	0.35446076	0.02869525
NSUN2	0.35328125	0.00260593
GNAQ	0.35262837	0.00962577
NCOA3	0.35240918	0.01868744
UBA3	0.35077736	0.00692741
HLA-C	0.35055149	2.1728E-07
CHD2	0.35042554	0.00018468
IGFLR1	0.35023134	0.0063591
CASP4	0.35015088	0.01410877
NCBP2	0.34940694	0.0109111
RSU1	0.34898168	0.00197628
UBTF	0.34890894	0.00677969
ATP1B1	0.34853578	0.02510493
NBEAL1	0.34852865	0.00358903
SAT2	0.34555807	0.00578108
COPB1	0.3438377	0.00081107
ACTR1B	0.34365898	0.03011617
MICU1	0.34273129	0.04649804
GGA1	0.34258929	0.00929292
HNRNPC	0.34249469	4.7561E-07
NRIP1	0.34195881	0.00234806
NFKBIB	0.34151806	0.00207991
PDIA3	0.33928138	0.0267268
PHLDB3	0.33656693	0.03939955
SLC35F5	0.33628037	0.04906606
PTBP3	0.33574199	0.00285672
VPS41	0.33355176	0.00010902
PRMT2	0.33340748	0.00026901
UBE2W	0.33231546	0.00349489
GMFB	0.33209346	0.02894851
C4orf3	0.33194103	0.00495512
TYK2	0.33162707	0.01048086
UGP2	0.33153247	0.03960737
C8orf76	0.33079576	0.0136024
TMEM123	0.33050915	0.00597504
TBCB	0.33023449	0.0095353
CDC27	0.33020358	0.04726349
POLR2A	0.33014822	0.02501546
GET4	0.33000803	0.01520396
CTTNBP2NL	0.32991621	0.01246338

ACTR3	0.3293453	0.00556762
NEAT1	0.32920951	0.00176369
BZW2	0.32911454	0.00411446
MOB1A	0.32790744	0.00464593
COX17	0.32756318	0.00503971
LIX1L	0.32684141	0.0212575
FAM50A	0.32648244	4.9789E-05
TCF12	0.32648149	0.04115222
MED10	0.32632673	0.00894425
EDF1	0.32621787	0.0040285
WDR1	0.3254702	0.00811454
HDGF	0.32523213	0.02982956
TIMM10B	0.32375168	0.03192385
TRIM26	0.32367139	0.01869459
ACTB	0.3222879	0.00184312
AGO2	0.32160267	0.01810502
RAB3GAP1	0.31998434	0.0009982
ITCH	0.31726121	0.02603843
UBE2B	0.31706092	0.01318072
DCTN2	0.31686504	0.02326138
PSMC1	0.31671059	0.00235858
PAFAH1B1	0.31651833	6.4665E-05
DNAJC21	0.31643813	0.02473554
VDAC2	0.31621604	0.01648481
TLK2	0.31621551	0.01326355
RHOC	0.31605583	0.03186339
ATXN2L	0.31482638	0.02694921
SDF4	0.31478899	0.00354414
SMARCA2	0.31456909	0.02116323
SEL1L3	0.31319788	0.03260421
TOB1	0.31315635	0.03940456
DDA1	0.31297958	0.00977821
ANP32E	0.31272076	0.00405703
IFNAR1	0.31239689	0.00027773
ARMH3	0.31213583	0.04969931
PDE7A	0.3118741	0.02821316
ARHGDIA	0.31150347	0.01097381
EHBP1L1	0.30922814	0.02864086
HERC4	0.30905308	0.04031085
DBI	0.3088677	0.00054905
THUMPD3-AS	0.30808535	0.04369414
IQGAP1	0.30740576	0.03780929
SERINC1	0.30606466	0.00532892
EXOC5	0.30532738	0.02139486
SDCBP	0.30490977	0.00150333
CSNK1D	0.3034378	0.00770338
LMBR1L	0.30195262	0.02953437
SLC31A2	0.29890149	0.02442416
AZIN1	0.29732633	0.02382905
UBE2E3	0.29719949	0.00926049

LARP1	0.29644577	0.02445885
GOLGA4	0.2949924	0.01998649
RUFY1	0.29398394	0.04543915
HSH2D	0.29259423	0.03868534
MORC3	0.29207164	0.01743232
PKM	0.2909439	0.0024285
MMP24OS	0.29029404	0.0243737
FGD5-AS1	0.29013998	0.04596714
TYROBP	0.28905225	0.01025768
YWHAZ	0.28882104	0.04114243
STAG1	0.28794746	0.0242886
SMAP2	0.28714199	0.03576797
PCGF5	0.2870045	0.01701452
ATF7	0.28546522	0.04724254
STX5	0.28498895	0.01427475
AKAP13	0.2828862	0.00238213
SMC3	0.28267333	0.02753153
NF1	0.28263324	0.01444313
MORF4L1	0.28143745	0.0024681
EPB41L2	0.28046613	0.01459451
TUBA1B	0.27992898	0.03983313
UPP1	0.27945496	0.01002257
PRCC	0.27897668	0.03528887
PLEKHM2	0.27791887	0.01922929
AFTPH	0.27668645	0.03192385
GGNBP2	0.27665877	0.03412638
AGFG1	0.27557988	0.00430576
THEMIS2	0.27428637	0.03025425
ADD1	0.27341903	0.00925194
MYL6	0.2722089	0.02263818
PUM1	0.27148155	0.00864641
XPO1	0.27049885	0.01960723
ATP6V0E1	0.27035565	0.00210133
TOX4	0.27022586	0.00967319
ELOVL5	0.27000054	0.02480229
NT5C2	0.26970738	0.02067686
RB1	0.26952504	0.0130657
ZFR	0.26720921	0.02816294
VDAC1	0.26713933	0.00483513
SUB1	0.26713593	0.01003672
FNBP4	0.26559367	0.00392098
HLA-E	0.26430357	0.0014829
CDC34	0.26202944	0.04888072
IER2	0.25995839	0.03317038
EIF4H	0.25922279	0.01566847
PSMB4	0.25814274	0.00726909
ATG3	0.25777684	0.02598483
SPAG7	0.25747009	0.00752317
AMZ2	0.25658948	0.03454299
POLR2E	0.25650293	0.01124219

HINT1	0.25555676	0.04969931
PPP2CA	0.25549737	0.01553257
OTUD5	0.25547207	0.04232911
SEC24B	0.25492917	0.04762016
ENO1	0.25462017	0.01121771
PSMD4	0.25310763	0.00129768
TRIM33	0.25274714	0.02242407
PFN1	0.24969792	0.0013554
BRD4	0.24955999	0.0266096
WBP2	0.24856016	0.01793857
TPM3	0.24790177	0.00110139
ATP6V1E1	0.24766482	0.02970047
HNRNPH2	0.2450414	0.01399733
ACTR1A	0.244987	0.04985258
RSF1	0.24479858	0.04986174
MAPRE1	0.24440543	0.0157259
GSK3B	0.24365354	0.0459218
ZDHHC12	0.24361531	0.04168015
KPNA2	0.24290371	0.03350488
USP34	0.24277122	0.03720092
PSMD1	0.24264937	0.04266837
ILK	0.24246301	0.01313863
SRRM2	0.24177552	0.00195864
SND1	0.24055299	0.02063082
PRPF40A	0.23911391	0.00639888
GLB1	0.23748714	0.02212739
RALY	0.23614031	0.00847822
CSDE1	0.23589616	0.01171973
CD55	0.23429645	0.03015651
GSPT1	0.23323279	0.04344708
MT-ND4	0.23045344	0.01504144
RSRP1	0.23000504	0.03477935
MAP3K20	0.22825589	0.02452005
TMEM165	0.22497537	0.02287945
RABGGTB	0.22047283	0.03128032
CHASERR	0.21999704	0.02309366
HDLBP	0.21280528	0.02023601
UBE2D3	0.20595268	0.00978805
STIP1	0.2022741	0.02818085
PAK2	0.20053039	0.00112378
HSPA4	0.19968837	0.04969931
ARPC4	0.19918722	0.00744462
TAX1BP1	0.1964694	0.00669868
EIF4E2	0.19553239	0.03447903
UBXN4	0.18771444	0.00933616
UBC	0.18614812	0.01238836
THRAP3	0.1815479	0.01500486
ZNF207	0.17253569	0.04986174
MT-CO1	0.17232283	0.00024901
CNBP	0.16773855	0.01544925

TMBIM6	0.14154131	0.04394897

OLT1B;ZFAND6;TRADD;OTUD7B;HSPB1;TNFAIP3;RORA;TREM2;ARRB2;LITAF;AKAP13;GJA1;TBK1;TFG;C
L23R;TRADD;ILK;PTPRJ;CXCL13;OASL;IL12B;JAK2;JAK3;IL6R;APPL1;JAK1;IL13RA1;IL15RA;IL4R;IL1R1;IL1
.;CD40;PRF1;CGAS;IFIT1;LYST;IFI44L;IFIT3;IFIT2;OASL;IFIH1;TBK1;DHX58;TRIM26;TRIM21;GBP5;RAB2B
CXCL8;IL23R;ZFAND6;TRADD;PTPRJ;IFIT1;MT2A;ZC3H12A;IL12B;SIRPA;CCR7;JAK2;JAK3;IL6R;JAK1;IL13R
.;CD40;PRF1;CGAS;IFIT1;LYST;IFI44L;IFIT3;IFIT2;OASL;IFIH1;TBK1;GBP5;RAB2B;RSAD2;ACOD1;OAS1;OA
;TFRC;TRADD;LITAF;AKAP13;GJA1;TBK1;TFG;CASP10;NUP62;TNFSF10;CCR7;PIM2;IKBKG;TRIM21;WLS;
B;IL15;STAT1;STAT3;IL31RA;CSF2RB;PTK6;TYK2;PKD2;CSF2RA;PIAS1;FER;MYO1C;CLCF1;STAT4;JAK2;JA
D274;NFAT5;IFITM1;CD40;CIITA;IL23R;ADAR;TNFRSF11A;RELA;CXCL16;RELB;CYP27B1;NUB1;IRAK2;LAN
B;STAT1;STAT3;IL31RA;CSF2RB;TYK2;PKD2;CSF2RA;PIAS1;MYO1C;CLCF1;STAT4;JAK2;JAK3;JAK1;IFNAR
RALA;RRAD;RASGRF1;PLD1;RASGRP1;NTN1;PLD2;RASGRP3;NRAS;RRAS;RASGEF1B;ORC3;G3BP1;KSR1;
30;IL23R;PDGFB;PDGFA;PTPRJ;TREM2;EFNA5;LRP8;SAMSN1;THBS4;RELN;ENPP2;IL12B;PTK2B;TNFRSF1
KN1A;BMPR2;TFRC;CSF1;CD81;CD80;PRKAG2;ILK;PTPRJ;TREM2;RASSF2;TBK1;CCND2;FGF9;ENPP2;FLO
RALA;GDI1;RASGRF1;CELSR1;PLD1;RASGRP1;NTN1;RASGRP3;RAP1B;NRAS;RRAS;RASGEF1B;ARHGDIA;
;CGAS;TREM2;LRP8;FOXP1;HERC5;TRAF3;IRF1;DHX58;CD55;N4BP1;BIRC2;APPL1;BIRC3;NFE2L2
RF1;TNFAIP1;IFIT2;BBC3;STK11;CASP7;CASP5;AIFM2;CASP10;CASP4;C19ORF12;IKBKG;JAK2;IER3;DAPK
D40;TFRC;CD81;CD80;IL23R;SLC7A1;TNFSF13B;CCL5;NCK2;CD38;IL12B;CCL19;JAK2;TNFRSF4;IL15;CD7
C;GOLT1B;CD81;IFI35;IGF1R;NR3C2;GJA1;TBK1;TFG;TNFSF10;IL12B;PIM2;TRIM21;WLS;TGM2;PDGFRA
CD274;CXCL9;CXCL8;CD80;IL23R;TNFAIP3;TNFRSF11A;CXCL13;CDC73;RELA;CYP27B1;SBNO2;CASP7;M
CIITA;IFITM1;IL23R;CXCL16;CYP27B1;NUB1;KYNU;CCL5;UBD;SIRPA;CCL19;CD58;GBP2;TRIM21;CCL17;
TFRC;CD81;CD80;IL23R;GATA3;SLC7A1;ACTB;MDK;CCL5;NCK2;IL12B;SIRPA;ABL2;CCR7;CCL19;ARID2;JA
D40;CDKN1A;TFRC;ILK;TREM2;SPPL3;CCND2;FGF9;FLOT1;PPP1R16B;MUSK;LIMCH1;CSNK1D;AXIN2;M
;TREM2;IFI35;FPR2;LRP8;ZDHHC18;CCL5;DHX58;TRIM21;SLC15A4;N4BP1;APPL1;ATG5;GBP5;PTPN1;T
1;IFITM1;APOBEC3F;RSAD2;APOBEC3G;APOBEC3H;STAT1;MX1;ISG15;IFIT1;OASL;IFIH1;ISG20;ZNFX1;
ANKLE2;TNFAIP8;TNFRSF6B;ARL6IP1;TFRC;ZFAND6;HSPB1;TREM2;SLC7A11;UBE2Z;IFIT3;PTMS;IGF1R;
D80;IL23R;PDGFB;PTPRJ;TREM2;EFNA5;LRP8;THBS4;RELN;CCL5;ENPP2;IL12B;PTK2B;TNFRSF14;JAK2;IL
31;EFNA5;NOL3;LARP4;MYO9A;CAMSAP1;MOV10;CLDN3;FMNL2;ZMYM4;PDPN;PHIP;CD44;MFSD2A
D;PDCD5;PRF1;UBE2Z;CLU;IFIT2;RASSF2;ADAMTSL4;AIFM2;RPS6KA2;ARL6IP5;TNFSF10;CTLA4;HTATIP
CD40;IL23R;CD80;HILPDA;CGAS;RORA;IFIH1;TBK1;MDK;DHX58;FLOT1;PDE4B;IL12B;CCR7;JAK2;IL6R;A
FAND6;TRADD;ILK;GATA3;TNFRSF11A;TANK;RELA;ZFP36L1;CCL5;ZC3H12A;PTK2B;RIPK1;CCL19;CD58;
CXCL9;CXCL8;CD80;TNFAIP3;CXCL13;CDC73;RELA;SBNO2;CASP7;MAPK8;CCL5;ZC3H12A;PDE4B;SIRPA;
CLIC4;CXCL9;CXCL8;CPNE4;CD80;AQP9;TNFAIP3;RORA;MTR;PTPRK;CXCL13;PKD2;CLU;CDC73;ACTB;CA

AG1;MAML1;UBE2D3;JADE3;JADE2;FBXO22;LOXL1;CDC14B;STK11;TBK1;PPP4R2;AKT3;PIM1;PIM3;KA
.;APOBEC3F;RSAD2;APOBEC3G;APOBEC3H;MX1;ISG15;IFIT1;OASL;IFIH1;ISG20;ZNFX1;OAS1;TNIP1;OA
SF6B;TFRC;ARL6IP1;ZFAND6;PDCD5;UBE2Z;CLU;ACTB;IFIT3;IFIT2;IGF1R;RASSF2;CCND2;RPS6KA2;ARH
NFRSF6B;TFRC;ARL6IP1;ZFAND6;UBE2Z;CLU;IFIT3;IGF1R;NRBP2;CCND2;LAMP3;ARHGDIA;ZC3H12A;PI

.;CXCL8;APOBEC3F;RSAD2;APOBEC3G;MX1;ISG15;ADARB1;IFIT1;OASL;IFIH1;ISG20;LARP1;ZNFX1;OAS1
;ILK;TRAF2;TNFRSF11A;TRAF1;RELA;NFKBIA;TRAF3;TRAF6;TRAF5;PTK2B;RIPK1;JAK2;BIRC2;BIRC3

M1;TES;TFRC;CSF1;CD81;PDCD5;ADARB1;IFI35;CLU;ETS1;ACTB;IFIT3;IGF1R;STK11;CCND2;FGF9;FTH1;
TX3L;RIPK2;USP9X;CAV1;DERL1;STK4;SPPL3;MMP9;ADD1;STK3;PPP2CA;TIAM1;PPP2CB;SUB1;FLOT1;R
B;RASGRF1;STMN3;BICDL1;PTPRM;CAMSAP1;PPP1R9B;CLN5;UGCG;SAMD14;UNK;NRTN;APOE;MAP4
DXC1;CEBPG;DERL1;TXN;STK4;SPPL3;CLN5;STK3;DPH3;FLOT1;APOE;CLIC2;B2M;TRIM21;CDT1;DTX3L;R
NT2;CD81;MAML1;RORA;PRDM2;GABPB1;ETS1;DCAF6;SPIB;IL4I1;TBK1;ZMIZ2;ZC3H12A;IER5;IER2;ZB
CL8;PLXND1;PTPRM;HSPB1;TNFAIP3;CXCL13;ETS1;MECP2;SPRED1;ZC3H12A;CTSH;ITGB8;HTATIP2;PP

;F11R;ICAM1;RAP1B;RAP2C;CLDN3;RAP2B;MARVELD2;RAPGEF1;RAPGEF2;RAPGEF6;TJP2

CXCL9;CXCL8;CD80;TNFAIP3;CXCL13;CDC73;RELA;SBNO2;CASP7;MAPK8;ZC3H12A;PDE4B;SIRPA;GBP2;

G1;GMFB;BMPR2;PANK3;PRAG1;MAML1;LRRK1;PRKAG2;ILK;STK11;TBK1;GNPTAB;AKT3;PIM1;TLK2;
5;GSK3B;PTPRO;LRRK1;USP34;ILK;TMEM131L;STK11;MDK;FAM53B;WLS;ANKRD6;CSNK1G3;TLE3;

TUD7B;TNFAIP3;PTPRJ;RORA;TREM2;IFI35;ARRB2;DUSP16;IGF1R;SPRED2;SPRED1;ZC3H12A;PHLDA3;

LA;NFKB1;RELB;NFKB2;NFKBIA;TBK1;IRAK2;TRAF6;BCL3;REL;ERC1;IKBKG;MAP3K14;BIRC2;BIRC3

;IGF1R;SDCBP;RASSF2;SIRPA;PTK2B;RIPK1;CCR7;CCN2;CCL19;SLAMF1;ANKRD6;MAP4K4;DUSP3;
;GADD45A;FZD7;MINK1;TRAF2;TRAF1;RASGRP1;GADD45G;SDCBP;MFHAS1;RASSF2;TAOK3;TRAF3;
;ISG15;RELB;IFIH1;PPM1B;TBK1;OAS1;OAS2;TRAF3;IRF1;OAS3;FLOT1;IRF7;REL;SIRPA;TRIM56;RIGI
4;HSPB1;PTPRJ;CLU;RASGRP1;IFIH1;IL12B;RIPK1;CCL19;JAK2;WNT5A;STAT3;HLA-E;IL1A;IL6;TYROBP;
B;PLEK;RASGRF1;STMN3;BICDL1;PTPRM;PKD2;CAMSAP1;PPP1R9B;SAMD14;NRTN;CCR7;APOE;MAP4;
84;HSPB1;TNFAIP3;PTPRJ;CLU;RASGRP1;FXR1;IFIH1;ZC3H12A;IL12B;SIRPA;RIPK1;CCL19;JAK2;SLAMF1;

6;CGAS;TREM2;LRP8;USP18;RELA;HERC5;SBNO2;DHX58;CASP4;APOE;JAK2;N4BP1;APPL1;BRD4;IL1R1
T5A;STAT3;HSPB1;PTPRJ;CLU;RASGRP1;HLA-E;IFIH1;IL1A;IL6;TYROBP;OAS1;FRMD8;OAS2;OAS3;IL12B;
M1;CDKN1A;TES;BTG1;BMPR2;CXCL8;PDCD5;TNFAIP3;PTPRJ;TREM2;ADARB1;IFI35;PTPRK;PKD2;ETS1
TREM2;STK11;TBK1;LAMP3;ZC3H12A;TLK2;GPR137;PIM2;ATP6V1E1;TRIM21;SH3GLB1;RUBCN;ATP6V

AG1;MAML1;IGF1R;STK11;TBK1;AKT3;PIM1;PIM3;PIM2;MAP3K6;EPHB3;CSNK1G3;PDGFRA;MAP2K1;
40;CDKN1A;TFRC;ILK;TREM2;IGF1R;CCND2;FGF9;FLOT1;NBN;PDGFRA;MUSK;DSCAM;LIMCH1;CSNK1D;
;IL23R;TNFRSF18;IL31RA;PTK6;PARP9;PARP14;VEGFA;PPP2CA;SOCS3;IL6;SOCS1;CCL5;CLCF1;IL12B;
LA-C;TRAF2;HLA-A;HLA-F;CD1B;HLA-G;MALT1;HLA-E;TNFSF4;TRAF6;IL12B;ULBP2;CD55;MICA
ABPB1;ACTB;DCAF6;SPIB;IL4I1;TBK1;ZMIZ2;ZC3H12A;KAT7;IER5;IER2;ZNF281;MRTFA;EBF4;RUNX2;
CL8;GATA6;HSPB1;ETS1;HIF1A;RRAS;MDK;ZC3H12A;HMOX1;CTSH;ITGB8;PLCG1;JAK1;PAK4;JUP;

SIAH2;EYA4;IFI6;TNFAIP3;CFLAR;NOL3;RELA;ICAM1;LGALS3;IL1A;PEA15;TMBIM1;BCL2;HMOX1;ITGAV
CL8;GATA6;HSPB1;ETS1;HIF1A;RRAS;ZC3H12A;HMOX1;CTSH;ITGB8;PLCG1;PAK4;JUP;ANXA3;PDPK1;
81;SH3KBP1;TNFSF13B;MAD2L2;INSL6;MMP14;IL6;PPP2R3C;IL7;TNIP2;CLCF1;GPR183;TNFSF4;BCL2;
CSF1;IL15;SRP54;IL31RA;TREM2;GATA3;TNFRSF11A;MMP9;FOXP1;VEGFA;CDC42;SBNO2;TYROBP;SOC

6;CGAS;TREM2;LRP8;USP18;RELA;HERC5;EFNB2;SBNO2;DHX58;CASP4;JAK2;N4BP1;CD55;APPL1;

FRC;SDC4;IL15;CD70;CD80;IL23R;PDCD1LG2;TYK2;HLA-G;SLC7A1;LILRB4;IL4I1;LGALS3;IL6;CCL5;NCK2;

P1;AVIL;CAPG;WASL;CNN3;PPP1R9B;RND1;TTN;CDC42;SAMD14;BAIAP2L1;PLS3;FLNA;WHAMM;MARC
;RAB2B;TICAM2;CGAS;TANK;CYLD;ITCH;TBK1;TYROBP;NPLOC4;TRAF3;IRF1;DHX58;G3BP1;IRF7;TRIM2[illegible]
FAND6;TRADD;GATA3;TNFRSF11A;TANK;RELA;CXCL16;ZFP36L1;NUB1;UBD;CCL5;ZC3H12A;RIPK1;CCL1
UBXN2A;UBE2D3;OTUD7B;RNF19B;TNFAIP3;ARRB2;TNFAIP1;FBXO22;TNFSF13B;HERC5;HERC4;NUB1;
2;RIPK2;STAT1;CGAS;ISG15;TANK;IFIH1;TBK1;OAS1;OAS2;TRAF3;IRF1;OAS3;DHX58;G3BP1;FLOT1;IRF7
CXCL9;CXCL8;CD80;TNFAIP3;RORA;TREM2;CXCL13;CDC73;CASP7;ZC3H12A;PDE4B;SIRPA;TICAM2;WNT
CSF1;HSPB1;PRKAG2;IQGAP1;CAMKK2;TTN;SPRED1;RASSF2;RELN;ORC3;PTK2B;CCR7;CCL19;PAK2;UVR

AIP6;TRADD;TNFAIP3;RORA;IFI35;ETS1;MDK;CASP4;NFKBIZ;IL12B;SIRPA;JAK2;IL1R1;IL15;SPHK1;WNT5[illegible]
RACD;PRKCE;SYNPO2;PLEK;MTSS1;P2RX7;FER;BAIAP2L1;FCHSD2;KATNBL1;CFL2;CDC42EP4;NUP62;NC[illegible]
MPR2;CXCL8;TFRC;CSF1;PDCD5;ECE1;TREM2;CLU;ETS1;ROBO1;GJA1;C1QTNF1;FGF9;LAMP3;ZC3H12A;
TFRC;IL15;CD70;CD80;IL23R;EBI3;PDCD1LG2;TYK2;SLC7A1;PPP3CA;IL6;CCL5;TNFSF4;NCK2;IL12B;CCL1[illegible]

;SDCBP;RASSF2;ZC3H12A;ARL6IP5;PTK2B;RIPK1;CCR7;CCN2;CCL19;SLAMF1;ANKRD6;GADD45B;GADD[illegible]
G3;RABGAP1;CAV1;RDX;TBC1D8;TBC1D2B;PLD1;CLU;CDC42;FXR1;CYLD;FER;FXR2;P2RX4;FSCN1;TBC1[illegible]
PTPRJ;GATA3;SPPL3;MALT1;ORC3;NFKBIZ;ZC3H12A;PDE4B;CTLA4;IKBKG;PLCG1;TRGC2;BTN2A2;RIPK2
3;PDPK1;CCL20;SPHK1;STIMATE;PTPRJ;ANK2;FPR2;ATP1B1;CLU;TPCN2;PPP1R9B;CAMKK2;P2RX7;PPP3
9B;TNFAIP3;UBE3A;FBXO22;DDA1;TNFSF13B;NPEPPS;RNF115;RNF114;TRIM2;RNF19A;CDC27;TRIM21
DAPK1;CCL20;STAT1;IFNGR2;DAPK3;WNT5A;TYK2;IRF1;CCL5;SIRPA;CCL19;CD58;JAK2;GBP2;CCL17;GBP

CREBBP;TFRC;DTX3L;LIMK2;OTUD7B;TYK2;PARP9;YWHAZ;LILRB4;MFHAS1;SIN3A;ORMDL3;CTNNA1;K[illegible]

T1;PLXND1;TNFAIP3;CXCL13;EFNA5;NOL3;ETS1;CAMSAP1;SIPA1L1;ZMYM4;HMOX1;PDLIM5;PAK2;EP[illegible]

XCL8;PLXND1;ADAM22;C2CD4B;PTPRJ;LAMC1;EFNA5;NID1;RND1;PPP2CA;PPP3CA;FUT7;PLAU;MDK;C[illegible]

;BACH1;GJA1;CREB3L1;GPBP1;ZC3H12A;IL12B;ADNP2;KAT7;MBNL2;MUSK;ZNF281;DAPK3;ANK2;ANK[illegible]
SMG1;STK4;IGF1R;CAMKK2;STK11;PIM1;MAP3K20;PTK2B;PIM3;RIPK1;PIM2;JAK2;PAK2;EPHB3;HTATIP
KN1A;TFRC;HSPB1;ILK;TREM2;CCND2;FGF9;ZC3H12A;FLOT1;SIRPA;MUSK;LIMCH1;PAQR3;CSNK1D;AXI[illegible]
BCD2;PRKAA1;AMBRA1;SLC4A4;HIF1A;FOXO1;STK11;TBK1;ZC3H12A;ORMDL3;ZSWIM8;PIM2;APOE;TR[illegible]
JUP;PDPK1;GADD45A;PDGFB;HSPB1;ETS1;HIF1A;VEGFA;MECP2;P2RX4;AKT3;FGF18;NF1;APOE;PLCG1
R2;CSF1;TRADD;CLDN1;CXCL16;IGF1R;SRGAP2C;PPP3CA;GLIPR2;PLAU;MDK;ENPP2;CTSH;PDGFRA;MM

F11R;TSPAN33;EPB41L3;FLOT1;RAB29;PIP5K1A;FLNA;RAB8B;JUP;SMURF1;CAV1;RDX;TTC7B;MYO5A;A[illegible]

PINB1;RIPK2;WNT5A;STAT3;HSPB1;TNFAIP3;LILRB4;MALT1;FOXP1;PANX1;P2RX7;IL6;TYROBP;ZC3H12

3;BMPR2;CXCL8;TFRC;CSF1;PHF1;PDCD5;HILPDA;TREM2;TENT4A;CLU;ETS1;ROBO1;GJA1;C1QTNF1;FG
5;IL23R;TNFRSF18;IL31RA;PTK6;PARP9;PARP14;VEGFA;IL6;CCL5;CLCF1;IL12B;JAK2;IL6ST;IL6R

MDEC1;IFITM1;TES;PLXND1;PDCD5;ADARB1;ENO1;IFI35;CLU;ETS1;ACTB;IFIT3;GJA1;STK11;FTH1;RPS6K
MC8;G0S2;TRAF2;TRAF1;CFLAR;NOL3;RELA;PML;LGALS3;CYLD;TNFSF10;ITGAV;PHIP;RIPK1;PAK2;BCL2I

RASGRF1;CXCL13;DOCK10;SIPA1L1;RGS1;RASGEF1B;TBC1D30;CCR7;TBC1D10A;TBC1D22B;RGS6;TGM2

;DUSP3;JAG1;CSF1;JUP;LIMCH1;NEXMIF;DAPK3;MINK1;EMP2;EFNA5;CDK6;PLAU;PEAK1;NF1;PTK2B;C
L8;CCL22;IL1R1;CCL20;TNFRSF11A;HIF1A;TANK;RELA;NFKB1;MAPK13;MAPK11;IRAK2;TRAF6;CCL5;ZC3
UBE2B;UBE2E3;UBE2D3;UBE2E1;CUL1;TNFAIP3;UBE2E2;UBE3A;UBE2A;NT5C2;ZNRF1;RNF115;ITCH;CD
;TRADD;PDCD5;UBE2Z;CLU;IFIT2;RASSF2;ADAMTSL4;AIFM2;RPS6KA2;ZC3H12A;ARL6IP5;TNFSF10;CTL

D;SYNPO2;PLEK;CLU;BAIAP2L1;CFL2;NCK2;PTK2B;TOGARAM1;CCR7;APOE;WHAMM;SYNPO;GSN;PRK
OTUD7B;TNFAIP3;RHOH;RORA;TREM2;ARRB2;TANK;LILRB4;PPM1B;TNIP1;ZC3H12A;RIPK1
;IL23R;GPR55;TNFRSF11A;LILRB4;GPR137B;FSTL3;PPP3CA;CAMK4;TRAF6;GPR137;IL12B;FBN1
C3;CXCL8;MUSK;GADD45A;PRKCE;HPN;PDGFB;CGAS;ACOD1;GATA3;HLA-G;CCL5;ZC3H12A;RIPK1;B2M
;BTG1;BMPR2;CSF1;LAMC1;ACTB;PPP3CA;GJA1;GLIPR2;MDK;SIN3A;ZC3H12A;CCN2;ARID2;IL6R;SOX5;
A;UBE2D3;ARRB2;TNFAIP1;TNFSF13B;PSMD4;TRIM2;CDC27;PSMD1;UBXN7;FBXO6;SEC61B;UBXN4;FB
CL9;CXCL8;TNFAIP6;LY75;F11R;CXCL13;TBK1;CCR7;IKBKG;TNFRSF4;PTGIR;RIPK2;WNT5A;PLA2G4C;IL1
CL22;IL1R1;CCL20;HIF1A;TANK;RELA;NFKB1;MAPK13;MAPK11;IRAK2;TNIP2;TRAF6;CCL5;ZC3H12A;SIR

UHRF2;UBE2B;UBE2D3;RNF19B;UBE3A;TRAF2;KLHL24;UBE2A;BRCA2;DDB2;CNOT4;RNF115;ITCH;RNF
KN1A;PDGFB;DERL1;IFIT1;STK4;SPPL3;ADD1;IFIT2;STK3;PPP2CA;MAPK8;FLOT1;B2M;TRIM21;DTX3L;R
M1;TCF7;RHOH;GATA3;PRDM1;ARID1B;SMARCA2;ACTB;MDK;IL7;TBX21;CAMK4;CLPTM1;ARID2

7A;PLXND1;L1CAM;NTN1;ROBO1;TIAM2;TIAM1;GOLGA4;RUFY3;PLXNA1;PLXNC1;MAP3K13
RABGAP1;PLXND1;RGS16;RASGRF1;STMN3;EFNA5;RASGRP1;RASGRP3;DOCK10;SIPA1L1;ALS2CL;RGS1;
;ACOD1;TRAFD1;ISG15;YWHAZ;USP18;ZDHHC18;MYO1C;OAS1;OAS3;DHX58;SERPING1;TRIM21;PTPN

L;PDPK1;CD70;TRADD;G0S2;INHBA;HIPK1;MLLT11;P2RX7;CASP10;BCL2;FAS;ITGAV;RIPK1;JAK2;IL6R;DE
;MAP3K1;GADD45A;BNIP3;F11R;TMEM150C;NFKB1;MAPK8;CASP5;IRF1;HABP4;PIEZO2;FAS;MAP3K1

;USP34;ILK;TNFAIP3;CDC73;UBR5;FAM53B;GPC5;TBL1X;WLS;TRPM4;CSNK1G3;ZRANB1;WNT5B;JUP;U
KN1A;DUSP3;PDPK1;GADD45A;MVP;CAV1;PTPRO;DTNBP1;HSPB1;PRKAG2;ADAR;PTK6;ADARB1;LILRE
CDC42BPG;PLEK;MINK1;DTNBP1;ATP2C1;RAP2A;FER;SIPA1L1;S1PR1;FLNA;PIP5K1A;WHAMM;PDLIM4

11R;TSPAN33;EPB41L3;FLOT1;PIP5K1A;FLNA;RAB8B;JUP;SMURF1;RDX;TTC7B;MYO5A;ANK2;EMP2;AN
A1;BMPR2;CLEC16A;ZFYVE1;FOXO1;MAPK8;NUAK2;MAP1LC3A;SESN3;SESN1;LAMP2;ZC3H12A;PMAI

2;CCL20;CXCL13;CYP7B1;CXCL16;CXCL10;CXCL11;KLF5;CCL5;GPR183;CCL19;GAS6;CCL17

1;SIAH2;IFI6;DHCR24;NOL3;MMP9;VEGFA;DNAJB6;LAMP3;CARD16;GAS6;RFFL;PAK2;CD44
;ITK;CD84;CD80;GATA3;RASGRP1;DLL4;PPP3CA;MDK;ORC3;NCK2;RAB29;SLAMF7;CCR7;CCL19;TNFRSF

M1;IL23R;RHOH;AMBRA1;GATA3;ARID1B;HLA-G;LILRB4;SMARCA2;IL4I1;SOCS1;MDK;IL7;TNFSF4;IL12E
AV1;SLC30A4;AQP3;CRIP1;HIF1A;TTN;PPP2CA;PPP3CA;IL1A;PPP2CB;TNNT2;KCNMA1;BCL2;TESMIN;CA

UBE2B;CAV1;LRRK1;USP34;DAPK3;ILK;CSNK1D;NFKB1;PPM1B;ZEB2;TBL1XR1;WNK1;UBR5;FAM53B;GI
1;CREBBP;CEP135;TFRC;DTX3L;LIMK2;CEP120;WNT5A;TREM2;TYK2;PARP9;GPR137B;PTP4A3;TYROBP;
;IL23R;PDGFB;PRKAG2;IQGAP1;CLU;LRP8;CAMKK2;PPP2CA;STK11;RASSF2;RELN;ORC3;CCL5;IL12B;PTK

UBXN2A;UBE2D3;DERL1;CUL1;ARRB2;TNFAIP1;AMBRA1;TNFSF13B;ZNRF1;PSMB4;PSMD4;TRIM2;TBX

PTPRJ;GATA3;SPPL3;MALT1;IGHG2;IGKC;ORC3;ZC3H12A;NFKBIZ;PDE4B;CD38;CTLA4;PLCG1;IKBKG;TRC
FAIP6;CGAS;TNFAIP3;RORA;FPR2;GATA3;FXR1;ZDHHC18;DHX58;SIRPA;APOE;TRIM21;ATG5;IL10RA;ST

UBA6;UBE2D3;UBR4;RNF19B;FBXO22;TNFSF13B;HERC5;HERC4;NUB1;RNF114;RNF213;UBB;RNF19A;U
NRXN2;F11R;TSPAN33;ARL5B;EPB41L3;FLOT1;RAB29;PIP5K1A;FLNA;RAB6A;RAB8B;SH3GLB1;JUP;SMUI
96;MAML1;ZNF292;IKZF1;ENO1;IKZF2;GABPB1;BACH1;ACTB;IKZF4;BACH2;DCAF6;SPIB;IL4I1;TBK1;ZNF
PRO;DAPP1;PTPRM;PTPRJ;PTPRK;PPM1K;MTMR4;DUSP16;PTPRF;CDC14B;PPP2CA;PPP3CA;PPP2CB;ST

ASP10;ZC3H12A;NUP62;TNFSF10;CCR7;IKBKG;PIM2;TRIM21;WLS;TICAM2;RIPK2;PDPK1;WNT5A;RHO

L5;TRAF2;ACTN4;TYK2;TRAF1;IL1A;OAS1;OAS2;TRAF3;TRAF6;IL3RA;TRAF5;IL6ST;SOS1;BIRC2;BIRC3;FO

;MORC3;RSAD2;ACOD1;OAS1;OAS2;TRAF3;IRF1;OAS3;TRAF6;IRF2;IRF7;TRIM56;IFI6;TANK;USP18;REL

A1;GBP5;IL4R;DAPK1;DAPK3;WNT5A;TYK2;IL1A;CDC34;HAX1;OAS1;IRF1;TRAF6;IL3RA;ARHGEF2;GAS6

S2;TRAF3;IRF1;OAS3;IRF2;IRF7;TRIM56;IFI6;TANK;RELA;G3BP1;PMAIP1;GBP2;ATG7;MICA;APOBEC3F;

TICAM2;RIPK2;ZDHHC13;TRAF2;RHOC;TRAF6;TRAF5;PELI1;ROR1;TNFRSF25;CARD16;ALPK1;BIRC2;BIR

MP3;UBD;KYNU;RIPK1;CCR7;JAK2;IL6R;TRIM21;JAK3;GCH1;IL1R1;STAT1;SPHK1;MX1;ISG15;NFKB1;PM

SIAH2;TIAM2;TIAM1;ARHGAP31;RIT1;MRAS;DOK4;RRAGC;DOK5;CDK2;GNB1;NF1;RAPGEF1;RAB18;RA

L4;JAK2;IL6R;PAK2;IL15;RIPK2;BMP6;VEGFA;RAP2C;PDCL3;IL6;HAX1;RAP2B;CLEC6A;ARHGEF2;CD44

T1;TNKS1BP1;CCR7;NBN;JAK2;IL6R;MUSK;LIMCH1;IL15;MMD;RIPK2;SH2D3A;SPHK1;CSNK1D;AXIN2;M

ORC3;G3BP1;PDPN;ARHGEF11;FARP2;KSR1;RAP2C;TIAM1;RAP2A;RIT1;COL1A2;MRAS;RAP2B;DOK4;DC

1;RIPK2;PRKCE;DAPK3;KREMEN1;LIG4;EMP2;CIT;IL1A;MELK;RRAGC;ADORA2A;ESPL1;PIK3CA;IRF1;VDA

;TICAM2;MUSK;WNT5A;DOK4;DOK5;RRAGD;ADAM8;ROR1;CARD16;ALPK1;PDGFB;LPAR1;PDGFA;GAT

APK8;NOCT;ZC3H12A;PDE4B;SIRPA;JAK2;GBP2;GBP3;TICAM2;GCH1;WNT5A;PDCD1LG2;NFKB1;FOXP1

K2;B2M;HLA-DOB;SMARCE1;PBRM1;IL15;CD70;RHOH;HLA-A;PDCD1LG2;TYK2;SMARCA2;ARID1B;IL6;I

MP9;RAP2A;CLDN3;NOS1AP;FAM20A;ADTRP;GAS6;TRABD2A;PELI2;BAZ2A;FPR2;TXN;AMBRA1;EFNA5

CND2;AIFM2;ARHGDIA;NUP62;PIM1;CD38;CTSH;PIM3;PIM2;IER3;HTATIP2;TSC22D1;GPX4;SPHK1;WN

6R;PAK2;LYN;PTPN1;IL15;RIPK2;TNFRSF18;IL31RA;PTK6;PARP14;PARP9;BMP6;VEGFA;IL6;HAX1;CLEC6

2;PHLDA3;TGM2;GBP5;KLF11;PRMT2;DAPK1;DAPK3;MELK;KCNMA1;ARHGEF7;ATF6;SQSTM1;BCL2A1

PPL1;GBP5;RAB2B;TICAM2;IL1R1;IL15;RIPK2;SPHK1;WNT5A;TRAF2;HLA-A;TYK2;HLA-G;HLA-E;FOXP1;IL

AK2;GBP2;CCL17;GBP1;GBP3;CCL22;CCL20;STAT1;TRAF2;ACTN4;TRAF1;NFKBIA;TRAF3;TRAF6;TRAF5;A

GBP2;GBP3;LYN;TICAM2;PRKCE;WNT5A;PDCD1LG2;NFKB1;CXCL10;IL1A;CXCL11;IL6;SELENOS;TNIP1;TN

P7;ZC3H12A;PDE4B;SIRPA;CPNE2;TGM2;PDGFRA;TICAM2;WNT5B;DAPK1;NCOA3;WNT5A;ACOD1;PD

T7;PIM2;MAP3K6;EPHB3;TGM2;CSNK1G3;MAP2K1;MORC3;PPTC7;DAPK1;PRKCE;FBXO2;DAPK3;CSNK1

DIA;PIM1;ARL6IP5;TNFSF10;TRIM24;PIM3;PIM2;IER3;PHLDA3;TGM2;DAPK1;ACTN1;DAPK3;WNT5A;A

M1;PIM3;PIM2;IER3;KDM2B;WNT5A;LIG4;ADAM8;PHIP;VDAC1;CARD16;ATF5;IL6ST;HPN;GATA6;PSEN

RPS6KA2;PIM2;ARID2;IER5;IL6R;PDGFRA;MAP2K1;H2AC6;NCCRP1;ATG13;PIAS1;TIAM1;CLDN3;PHIP;A

;RAB6A;LYN;RYK;WNT5A;PBX3;DTNBP1;VPS13B;PBX4;L1CAM;PBX1;RAB11A;EHD1;IL6;VAPA;TDP2;WN

TB38;WNT5A;UBE2E1;MRTFA;MITF;EBF4;RUNX2;THAP3;TBL1XR1;RFX5;ATF7;PHIP;ATF5;ZFPM2;ATF6;

P1R16B;ANXA3;PDPK1;SERPINF1;SPHK1;WNT5A;GAB1;EMP2;HLA-G;IL1A;NINJ1;ANGPTL4;FOXC1;NPR

GBP3;TICAM2;WNT5A;PDCD1LG2;NFKB1;CXCL10;IL1A;CXCL11;IL6;SELENOS;TNIP1;TNIP2;TNFSF4;TRAF

PIM3;PIM2;JAK2;MAP3K6;JAK3;JAK1;EPHB3;MAP2K1;MORC3;DAPK1;PDPK1;PRKCE;SPHK1;DAPK3;MIN
3P4;DAPK3;WNT5A;KREMEN1;CSNK1D;AXIN2;ZEB2;PPM1B;FRMD8;TBL1XR1;CSNK1G1;STK4;STK3;UBF

DUSP4;EPM2A;DUSP5;DUSP3;RHOH;DUSP8;HLA-G;MMP9;CIT;PPM1B;CDC34;AKT1S1;PRKAA1;RNF34;

45B;GADD45A;FZD7;MINK1;TRAF2;TRAF1;GADD45G;CYLD;MFHAS1;RAP2A;ITCH;TAOK3;TRAF3;TRAF6;

KIFAP3;MARK4;RAB6A;LYN;PLEKHA1;RYK;DTNBP1;VPS13B;L1CAM;IQCG;RAB11A;EHD1;IL6;VAPA;RAB
;WNT5A;STAT3;LILRB4;FOXP1;HLA-E;IL1A;IL6;TYROBP;SELENOS;OAS1;FRMD8;OAS2;OAS3;ARHGEF2;LC

;IFIT3;CDC73;STK11;FTH1;RPS6KA2;NUP62;NCK2;IL12B;PIM2;JAK2;ARID2;B2M;KLF11;PBRM1;MAP2K1
1G1;ATP6V0E1;DAPK1;DAPK3;ATG13;SREBF2;HAX1;RRAGC;RRAGD;ATP6V1B2;VDAC1;PIK3C3;ATF6;SC

MORC3;MUSK;DAPK1;PRKCE;DAPK3;CSNK1D;VRK2;ATG13;MELK;MAK;CSNK1G1;PDGFB;CSF2RB;STK4;C
AXIN2;MMP9;RAP2A;CLDN3;ROR1;FAM20A;ADTRP;GAS6;PELI2;FPR2;EFNA5;STK4;RASGRP1;CAMKK2;

HAP3;RFX5;ATF7;ATF5;ZFPM2;ATF6;ATF3;CASZ1;CTBP2;EPAS1;GATA6;PDGFB;GATA3;HIF1A;HELZ2;CYP
A3;PDPK1;SPHK1;WNT5A;GAB1;EMP2;HIPK1;VEGFA;IL1A;PDCL3;PKM;NINJ1;AGO2;FGF18;ANGPTL4

4;PDGFRA;IL1R1;CAV1;WNT5A;GP1BA;FOXP1;CYLD;P2RX4;TRAF3;TNFSF4;IRF1;CARD16;BIRC2;MGLL;N

KSL1;GSN;TPM3;ACTN1;MYO5A;ARPC4;EMP2;RHOF;ARHGAP25;MAD2L2;MYO1E;MARCKS;MYO1B;M

9;CD58;JAK2;GBP2;CCL17;GBP1;GBP3;CCL22;GCH1;CCL20;SPHK1;NFKBIA;ARHGEF2;NFE2L2
RNF115;RNF114;PSMD4;TRIM2;RNF19A;CHMP1A;PSMD1;UBXN7;RNF150;HERC6;GET4;FBXW5;DTX3L

5B;NCOA3;WNT5A;ACOD1;PDCD1LG2;SSTR2;IL1A;SELENOS;TRAF6;LCOR;ADTRP;CARD16;LCP1;CHMP5
AG;MMD;PDPK1;DTNBP1;AXIN2;WARS1;RAP2C;RAP2B;GNAQ;AKT1S1;NF1;RAPGEF2;CCNYL1;TAB2;G

;ACOD1;MMP9;FOXP1;SELENOS;NINJ1;CARD16;LCP1;MGLL;BIRC2;BIRC3;SEMA7A;FPR2;GATA3;USP1

CTSH;ITGB8;NSF;MAP2K1;MUSK;ANXA2;DTX3L;WNT5A;ANK2;MITF;ANK3;ADAM19;ACTA2;IL1A;CLDN

45A;FZD7;SPHK1;MINK1;TRAF2;TRAF1;GADD45G;VEGFA;MFHAS1;TAOK3;TRAF3;TRAF6;TAOK1;TRAF5

;WSB2;DTX3L;UBE2E3;UBE2E1;UBE2E2;TRAF2;TRAF1;RC3H2;DDB2;RNF144B;CDC34;TRAF3;TRAF6;TR

HB3;BCL9L;HTATIP2;EGLN1;ZRANB1;EMP2;LARP4;WARS1;IL6;RAP2A;CLDN3;INF2;FMNL2;RUFY3;NF1;P

CL5;IL12B;ABL2;PLXNA1;PTK2B;CCR7;ITGAV;PLXNC1;RSU1;CYTIP;CYTH1;EPHB3;TGM2;LYN;PPP1R12A;

;MARF1;CLDN3;ATF7;ALPK2;ATF5;ZNF274;HPN;PDGFB;BAZ2A;GATA3;HIF1A;CYP27B1;LARP1;APOE;L
2;LYN;PDGFRA;MUSK;DAPK1;PDPK1;NEK6;DAPK3;MINK1;PTK6;VRK2;ATG13;FER;PIKFYVE;MELK;TAOK
2;MMP9;RAP2A;CLDN3;PHIP;FAM20A;ADTRP;GAS6;PPP1R15B;PELI2;PDGFA;FPR2;EFNA5;STK4;RASG
M21;SH3GLB1;PTPN1;DTX3L;USP20;DAPK1;BNIP3;ATG13;GTPBP1;SVIP;P2RX7;MOV10;ITCH;CNOT1;P

IP7;WNT5B;PRKCE;SPHK1;TNFRSF18;GAB1;ACTN4;RHOC;TIAM1;MMP14;CLDN3;RUFY3;RAPGEF2;ARH

NK2;EMP2;ANK3;ATP1B1;RAB11A;EHD1;RAB10;TSPAN15;RAP2A;TSPAN14;EHD4;PACS1;RAB12;RAB13

F9;LAMP3;ZC3H12A;HABP4;FLOT1;ITGB8;CTSH;KAT7;MAP2K1;MUSK;IL15;WNT5A;CIRBP;MITF;ANK2;

KA2;PIM2;PLXNC1;CCN2;ARID2;MAP2K1;DSCAM;H2AC6;ATG13;CLDN3;ATF5;CTBP2;NPR1;GATA3;PDS5

;BCAS3;TBC1D8;TBC1D2B;RANGAP1;ARHGAP45;TIAM2;TIAM1;RALGAPB;TBC1D4;RAPGEF1;RAPGEF2;A

A4;CTSH;PHLDA3;TGM2;KLF11;PRMT2;DAPK1;DAPK3;MELK;KCNMA1;ARHGEF7;ATF6;SQSTM1;BCL2A1

PBRM1;DNAI3;MMD;AXIN2;ARID1B;RUNX2;TIAM1;ZEB1;RAPGEF2;ADAM8;ARHGEF2;LCP1;IL6ST;NBL1

XW5;FBXO2;AXIN2;NCCRP1;CDC20B;CDC34;SELENOS;KCTD10;PSMA2;TBL1XR1;KCTD13;PELI1;BIRC2;R

A;TPST1;ADORA2A;CARD18;ADAM8;CD44;MGLL;FPR1;FPR2;GATA3;RELA;RELB;FUT7;IRAK2;ORC3;CCL5

RASGEF1B;CCL5;TBC1D30;PLXNA1;RSU1;CCL19;PLXNC1;CCL17;RGS6;EPHB3;MAP4K4;TGM2;BCAS3;CC

NK3;ATP1B1;RAB11A;EHD1;RAB10;TSPAN15;RAP2A;TSPAN14;EHD4;PACS1;RAB12;RAB13;RAPGEF2;GA

P1;ATG7;MFSD2A;ATG5;SH3GLB1;GABARAPL2;AOC1;WDR59;SREBF2;RRAGC;STK26;RRAGD;JMY;VPS41

2B;CCR7;CLSPN;CCL19;IL6R;PTPN1;MAP2K1;MMD;EMP2;AXIN2;VEGFA;TRAF6;RAPGEF2;CALM3;TAB2;

21;ZSWIM8;PSMD1;UBXN7;PMAIP1;TBL1X;RFFL;TRIM21;FBXW5;UBE2B;SMURF1;SIAH2;SIAH1;UBE2A;

GC2;BTN2A2;PLEKHA1;RIPK2;RFTN1;HLA-A;RC3H2;NCOR2;PIK3CA;THEMIS2;WNK1;TRAF6;BCL2;CD247

JBD;RNF150;RFFL;HERC6;GET4;UBE2B;DTX3L;SMURF1;SIAH2;SIAH1;UBE2E1;ISG15;UBE2A;FBXL14;RC3
RF1;CAV1;RDX;RFTN1;TTC7B;MYO5A;ANK2;EMP2;ANK3;ATP1B1;RAB11A;EHD1;RAB10;TSPAN15;RAP2
F608;ZMIZ2;CREB3L1;ZC3H12A;IER5;IER2;SOX5;ZNF281;RFX2;EBF1;MRTFA;EBF4;RUNX2;THAP3;SUB1;F
K11;PPP3CC;ILKAP;DUSP5;PTPN1;DUSP3;PPP1R12A;PPTC7;PPP2R3A;SSH1;CTTNBP2NL;DUSP22;PPM1

;ZDHHC13;TRAF2;TRAF1;RHOC;PPM1B;TRAF3;TRAF6;TRAF5;PELI1;ROR1;TNFRSF25;CARD16;ALPK1;SC
XC1;EBI3;PDGFB;CSF2RB;TNFRSF11A;IL2RG;ADIPOR2;CSF2RA;LRP8;RELA;UGCG;SOCS1;IRAK2;CCL5;STA
A;G3BP1;PMAIP1;GBP2;ATG7;MICA;APOBEC3F;APOBEC3G;USP20;APOBEC3H;STAT1;BNIP3;MX1;ISG15
;IL6ST;SOS1;IFNAR1;NFAT5;FOXC1;SELPLG;EBI3;TCF7;CSF2RB;GATA3;IL2RG;HIF1A;TANK;CSF2RA;LRP8
APOBEC3G;APOBEC3H;STAT1;BNIP3;MX1;ISG15;ISG20;MOV10;AGBL4;IL6;ZNFX1;BCL2;MAP3K14;SHFL
C3;SLC20A1;PELI2;LPAR1;ATP2C1;CXXC5;MALT1;RELA;UBD;S100A13;HMOX1;FLNA;RIPK1;CCL19;APOL

MP9;RAP2C;RAP2A;CLDN3;HAX1;RAPGEF2;ARHGEF2;FAM20A;ADTRP;GAS6;CD44;PELI2;PDGFB;PDGF
K5;CDK2;GNB1;NF1;ARHGEF3;RAPGEF1;RAPGEF2;TNFRSF25;KRAS;RGL1;RAPGEF5;SOS1;RAPGEF6;KP

C1;TNFRSF25;IFI6;EFNA5;STK4;FOXO1;STK3;FXR1;PPP2CA;BCL2L11;PLAGL1;PMAIP1;PTK2B;BMF;RIPK

A3;IQGAP1;TXN;UBD;PDGFC;S100A13;HMOX1;PMAIP1;RIPK1;HEXIM1;LYN;WDR59;RBPMS;NEK6;CAV
;CXCL10;IL1A;CXCL11;FER;IL6;SELENOS;TNIP1;TNIP2;TNFSF4;TRAF6;PELI1;TAB2;CARD16;IFNAR1;NFKB

;STK4;RASGRP1;CAMKK2;FXR1;FXR2;RELN;RIPK1;ATG7;LYN;NIBAN1;RARRES2;LIMK2;SMARCA5;SVIP;A

T5A;SERPINB9;MMP9;YWHAZ;RNF144B;HAX1;TAX1BP1;VDAC1;PHIP;TMBIM6;ANGPTL4;ATF5;GAS6;IL

;STK4;TMEM164;FOXO1;STK3;C3ORF38;MAPK8;BCL2L11;ACVR1C;UBD;MAP3K20;PMAIP1;BMF;RIPK1;
1A;TYROBP;IRF4;TRAF3;IRF1;TRAF6;IRF7;ARHGEF2;LCP1;IL6ST;CD274;EBI3;CEBPG;GATA3;HIF1A;TANK

CD1LG2;SSTR2;MMP9;IL1A;RRAGC;SELENOS;TRAF6;RRAGD;RAPGEF1;RAPGEF2;LCOR;LCP1;CARD16;SC

LD;VRK2;ATG13;MELK;CDC34;MAK;LORICRIN;PDGFB;DAPP1;CSF2RB;MTMR4;STK4;CSF2RA;FUT4;STK3

CTN4;MORF4L1;MELK;PHIP;VDAC1;ALPK2;ATF5;IL6ST;ATF6;SQSTM1;HPN;GATA6;PSEN2;STK4;FOXO1;
2;FOXO1;SOCS2;SOCS3;NUAK2;RIPK1;APOE;SNX6;SIAH2;CAV1;IL31RA;DHCR24;NFKB1;IL6;IL7;BCL3;BC

TF5;IL6ST;CTBP2;COX17;PDGFB;PDGFA;TGFA;GATA3;TNFRSF11A;PDS5B;ZFP36L1;EFNB2;CYP27B1;SDC

SQSTM1;ATF3;CASZ1;KMT2A;CTBP2;EPAS1;GATA6;GATA3;HIF1A;FOXO1;HELZ2;CYP27B1;SBNO2;HIVE
1;GATA6;HIF1A;THBS4;RRAS;HMOX1;PLCG1;PAK4;EGLN1;JUP;STAT1;HIPK1;PML;WARS1;VEGFA;PDCL3

IK1;CSNK1D;VRK2;ATG13;YWHAZ;MELK;PIK3CA;PEAK1;MAK;PIK3C3;FAM20A;GAS6;DGKH;NIM1K;PRK
R5;GPC5;APOE;TBL1X;TRPM4;JUP;UBE2B;FZD7;SIAH2;CAV1;FZD6;MCC;NFKB1;MAD2L2;CYLD;APC;WN

MGRN1;NOL3;TANK;RELA;PPP2CA;NUAK2;RIPK1;RFFL;LYN;PLEKHA1;UBE2B;USP20;STAT1;CAV1;LILRB

;MORC3;PTGIR;H2AC6;KDM2B;WNT5A;AXIN2;ATG13;PLAAT4;MTSS1;IL1A;CLDN3;IRF1;RAPGEF2;ATF5
STM1;OPTN;PRKAA1;MTMR9;FOXK1;AMBRA1;MTMR4;FOXO1;DEPP1;MAPK8;DRAM1;ORMDL3;ATP6

CSF2RA;STK3;GRK3;NUAK2;MAP3K20;ABL2;RIPK1;CLSPN;MARK4;CDK17;LYN;CDK18;UVRAG;NEK4;NEK
FXR1;SDCBP;FXR2;RELN;DHX34;CCL5;RIPK1;LYN;NIBAN1;RYK;FZD7;RARRES2;LIMK2;MERTK;ACVR2A;V

27B1;HIVEP1;APOE;STAT5A;STAT5B;CREBBP;JAG1;JUP;FZD7;IL31RA;SMARCA5;TRIP4;PBX4;SMARCA2;

;UBE2E1;VPS37C;AXIN2;RC3H2;RNF144B;CDC34;KCTD10;PSMA2;TBL1XR1;KCTD13;PELI1;SQSTM1;BIR

;CD274;TFPI;RELA;SBNO2;MAPK8;NRIP1;ABL2;PTK2B;GBP2;GBP3;LYN;H2AZ1;FZD7;PTK6;NFKB1;CXCL

8;RELA;FXR1;SBNO2;CCL5;RIPK1;APOE;SNX6;BRD4;IL10RA;STAT3;MAPK13;NFKBIA;CYLD;MFHAS1;IL6;

;TYROBP;RNF207;NOS1AP;ADTRP;GAS6;PRKAA1;SLC24A3;HPN;PDGFB;HIF1A;RELA;TTN;DLL4;CYP27B

AF5;PELI1;BIRC2;TRIM56;RNF34;PELI2;CUL1;NT5C2;ANAPC10;DCAF15;ZNRF1;FZR1;RNF213;UBR5;RFFL

N;CREBBP;FZD7;RDX;SMARCA5;RAB27A;TRIP4;LARP4;PARP14;PUM1;SMARCA2;PARP15;OCLN;ID2;RE

RP1;CAMKK2;TTN;FXR1;PPP2CA;FXR2;RELN;SOCS1;RIPK1;LYN;NIBAN1;RARRES2;LIMK2;ACVR2A;VEGF

GEF2;PFN1;ARHGEF7;DOCK1;SEMA7A;SEMA3C;SEMA3D;PDGFB;PDGFA;HIF1A;CDC42;SDCBP;CCL5;PD

NK3;ARID1B;ADAM19;ACTA2;IL1A;CLDN3;TYROBP;RNF207;NOS1AP;ADTRP;GAS6;PRKAA1;SLC24A3;K

;B;CYP27B1;TP53BP2;PLXNA1;LYN;ZBTB17;CDT1;APOBEC3F;APOBEC3G;APOBEC3H;OSGIN2;INHBA;SM

ARHGEF7;RAPGEF5;SOS1;RAPGEF6;RGS16;RASGRP1;RASGRP3;ARHGAP22;CCL5;TBC1D13;PIP5K1A;RSU

1;STK4;FOXO1;STK3;C3ORF38;MAPK8;BCL2L11;ACVR1C;UBD;TP53BP2;MAP3K20;PMAIP1;BMF;RIPK1;A

.;FOXC1;CREBL2;NID1;ZFP36L1;SDCBP;RRAS;NOCT;PDPN;GCNT2;TRPM4;BCL9L;SMARCE1;RARRES2;SU
RNF121;RNF34;DERL1;CUL1;AMBRA1;ANAPC10;ECPAS;ZNRF1;PSMB4;FZR1;TBX21;ZSWIM8;TBL1X;ASC
5;CCL19;CCL17;APOL2;GGT5;CCL22;CCL20;STAT3;NFKB1;NFKB2;CXCL10;MFHAS1;ITCH;CXCL11;IL6;TNF

L22;RIC1;CCL20;RDX;GPR137B;VAV2;WNK1;RAPGEF1;NF1;RAPGEF2;RGL1;ARHGEF7;RAPGEF5;AGRN;S

;AXIN2;PML;UBE2W;ITCH;SPSB1;CDC34;APC;KCTD10;PSMA2;TBL1XR1;PSMC1;KCTD13;PELI1;TRIP12;A

[illegible]A;TSPAN14;EHD4;PACS1;RAB12;RAB13;RAPGEF2;NUP54;GAS6;AGRN;RAPGEF6;RAMP1

[illegible]RFX5;ATF7;ATF5;ZFPM2;ATF6;ATF3;CASZ1;ZNF274;CTBP2;EPAS1;GATA6;GATA3;ZBTB5;HIF1A;HELZ2;C[illegible]

QSTM1;OPTN;BIRC2;BIRC3;SLC20A1;PELI2;LPAR1;ATP2C1;CXXC5;TANK;RELA;MALT1;UBD;S100A13;HM
AT4;IL21R;PTK2B;RIPK1;CCL19;CCL17;LYN;STAT5A;STAT5B;CCL22;CCL20;STAT1;STAT3;MX1;IL31RA;LILI
;PML;ISG20;CXCL10;MOV10;UBE2W;AGBL4;IL6;ZNFX1;BCL2;MAP3K14;SHFL;BCL2L1;RIGI
;RELA;ZFP36L1;SBNO2;SOCS1;CCL5;STAT4;IL21R;ST3GAL6;RIPK1;CCL19;CD58;GBP2;CCL17;GBP1;GBP4

A;CREBL2;FPR2;IQGAP1;TXN;EFNA5;STK4;RASGRP1;LRP8;THBS4;CAMKK2;FXR1;SDCBP;FXR2;RELN;ORC

1;BCL2L14;PAK4;GADD45A;BNIP3;SIAH1;MX1;DHCR24;CFLAR;NFKBIA;IL2RA;BCL2;FAS;BCL2L1

1;SIAH1;BMP6;MFHAS1;IL6;CLEC6A;REL;TAB2;MAP3K14;DDR1;CD40;BMPR2;TRADD;ILK;TREM2;PTPRJ

ACVR2A;VEGFA;MOB1B;MOB1A;SLCO3A1;TAOK1;FGF18;FAS;CALM3;KRAS;BMAL1;RAMP1;RAB3GAP1

.6ST;CD44;BIRC2;BIRC3;PRKAA1;BCL2A1;HPN;AMIGO2;GATA6;IFI6;PSEN2;NOL3;FSTL1;RELA;FOXO1;M

;MARK4;GBP2;ATG7;GBP1;GBP3;GADD45B;GADD45A;BNIP3;SIAH1;CFLAR;GADD45G;MLLT11;DNAJA1;
K;RASGRP1;RELA;MALT1;G3BP1;S100A13;HMOX1;RIPK1;TNFRSF14;CCL19;CD58;SLAMF1;RFTN1;STAT3

QSTM1;CHMP5;CD274;PRKAA1;PDGFB;IQGAP1;FOXO1;RELA;ADD1;CAMKK2;ZFP36L1;RAP1B;SBNO2;M

;UGCG;GRK3;NUAK2;FUT7;MAN2A1;MAP3K20;ST8SIA4;ST3GAL5;ABL2;ST3GAL6;ST8SIA6;MARK4;ATG

STK3;SOCS2;SOCS3;BCL2L11;NUAK2;UBB;UBD;MAP3K20;ABL2;PMAIP1;BMF;RIPK1;ATG7;GADD45B;G
L2;CPEB4;BCL2L1;AVEN;GSK3B;TNFAIP8;HSPB1;TREM2;PTMS;MDK;NUP62;CD38;CTSH;HTATIP2;TSC22

CBP;PDGFC;S100A13;SLAMF1;TRPM4;LYN;STAT5A;ZBTB17;STAT5B;JAG1;JUP;TNFRSF9;IL31RA;INHBA;S

P1;RIPK1;BCL9L;ZBTB18;STAT5A;ZBTB17;STAT5B;MIR9-1HG;CREBBP;JAG1;POU2F1;JUP;SMARCA5;INHI

AA1;PDGFB;CSF2RB;STK4;CSF2RA;CAMKK2;STK3;GRK3;MAPK8;FGGY;NUAK2;ACVR1C;MAP3K20;PTK2E

;CTBP2;HPN;GATA6;GATA3;PDS5B;TOB1;RNF2;DLL4;CYP27B1;ACVR1C;PDPN;HMOX1;PTK2B;APOE;TP
V0A2;ABL2;BMF;SNX6;UBQLN2;UVRAG;USP20;IL10RA;BNIP3;STAT3;SVIP;GPR137B;TPCN2;BCL2;TAB2

6;PTK6;CLK4;SMAD7;FER;CDK6;WNK1;STK26;CDK2;TAF4B;MAP3K13;CDK14;DDR1;GSK3B;SMG1;BMPI

PBX1;MLLT11;MLLT10;ID2;AGO2;REL;CREBRF;CD40;RSF1;WASL;MED16;BRCA2;LITAF;PTMS;MED12;PI

C2;CHMP5;RNF34;UBA6;CUL1;UBR4;AMBRA1;ZNRF1;PSMB4;RNF213;UBD;TBX21;ZSWIM8;TBL1X;RFFI

1;MAPK8;UBR5;RAB29;RIPK1;PTPN1;GSN;EPHX2;RDX;STAT3;DTNBP1;RAB27A;INHBA;BMP6;WARS1;V

L;EZH2;BTG1;PHF1;ATF7IP2;RSF1;BRCA2;LITAF;ROBO1;MECP2;HINT1;C1QTNF1;ZNF280C;CHMP1A;CT[illegible]

GFC;PDPN;GCNT2;PTK2B;PLCG1;MAP4K4;CAV1;RDX;STAT3;SOD2;RAB11A;VEGFA;FER;MYO1C;APC;AG

MT2A;WBP2;HPN;PDGFB;RPS27L;PLD1;HIF1A;RELA;TTN;FXR1;DLL4;CYP27B1;MAPK8;LARP1;FXR2;POL

IARCA2;HOPX;IL6;CDK6;ST20;CD200;BTG3;CDKN1A;PIWIL4;BTG1;BMPR2;CXCL8;TREM2;PTPRJ;PTPRK;

J1;CCL19;TBC1D17;CCL17;MAP4K4;CCL22;RIC1;CCL20;ARHGAP25;NF1;RGL1;AGRN;RAB3GAP1;EZH2

ATG7;GADD45B;GADD45A;BNIP3;SIAH1;CFLAR;GADD45G;MLLT11;DNAJA1;IL6;APC;JMY;BCL2;NF1;FAS

CO;SMARCA2;BMP6;NFKB1;ACVR2A;XKR8;SLC6A6;MAPK11;IL6;ID2;TCF4;INSM1;BMAL1;EZH2
C3;RFFL;UBQLN2;UBE2B;SMURF1;EDEM1;SIAH2;SIAH1;TRIP4;UBE2A;FBXL14;PML;FBXL8;UBE2W;ITCH

YP27B1;MIER3;RAD21;HESX1;HIVEP1;ZNF821;HIVEP2;ZBED6;HEXIM1;STAT5A;STAT5B;CREBBP;JAG1;JU

IOX1;FLNA;RIPK1;CCL19;APOL2;BRD4;USP20;STAT1;NEK6;CFLAR;LILRB4;TNIP1;VAPA;TNIP2;CLEC6A;RE

-;GBP3;STAT5A;STAT5B;CCL22;CCL20;STAT1;STAT3;IL31RA;LILRB4;NFKB1;MAPK13;NFKBIA;MAPK11;FE

C3;PDGFC;PTK2B;RIPK1;TNFRSF14;CCL19;PAK2;LYN;NIBAN1;RBPMS;CAV1;RARRES2;LIMK2;INHBA;BMF

I;LITAF;BBC3;AKAP13;CASP10;NUP62;ITGAV;CCR7;TNFAIP8L3;IKBKG;JAK2;KSR1;RIPK2;SPHK1;ZDHHC1

ALT1;SOCS2;SOCS3;MAPK8;NUAK2;DPP9;PDPN;FLNA;PTK2B;RIPK1;PAK2;ATG5;MAP4K4;SIAH2;BNIP3;

APK8;NRIP1;ABL2;PTK2B;GBP2;SLC25A24;GBP3;LYN;H2AZ1;AOC1;FZD7;PTK6;NFKB1;PEX14;SYT17;SSH

7;ST3GAL1;ST3GAL2;ATG5;CDK17;LYN;UBE2F;CDK18;UVRAG;NEK4;ST8SIA1;NEK6;OGA;ABCA7;PTK6;SS

ADD45A;SIAH2;SIAH1;IL31RA;DHCR24;NFKB1;GADD45G;MLLT11;CXCL10;IL6;IL7;STK26;BCL3;JMY;BCL
2D1;SPHK1;SERPINB9;MMP9;YWHAZ;RNF144B;HAX1;PIK3CA;TMBIM1;TAX1BP1;TMBIM6;ANGPTL4;GA

MARCA2;BMP6;SQLE;CXCL10;CXCL11;FER;IL6;CDK6;NR4A3;IL7;TNFSF4;ID2;CDK2;FGF18;BCL2;MELTF;I

BA;SMARCA2;PBX1;NFKB1;BMP6;NFKB2;NFKBIA;MLLT10;CXCL10;IL6;TFCP2;NR4A3;AGO2;BCL3;MAFF;

3;MARK4;PAK2;NADK;MAP4K4;PAK4;CDK17;LYN;CDK18;UVRAG;MAP3K1;NEK4;RYK;NEK6;CDC42BPG;L

53I11;E2F7;LYN;ZBTB17;CDKN2B;INHBA;MCC;SOD2;SMARCA2;PML;WARS1;IL6;CDK6;MYO1C;APC;NF1

R2;MAST4;LRRK1;PRKAG2;ILK;TLK2;ERC1;JAK2;JAK3;JAK1;HTATIP2;CAMK1D;PDPK1;MINK1;YWHAZ;PE

PP3CA;MED14;MED13;HEY1;MED10;CD38;IKBKG;HTATIP2;ANXA2;PRMT2;RIPK2;NCOA3;TCF12;ZFY;PA

L;ZRANB1;UBE2B;USP9X;SMURF1;SIAH2;SIAH1;UBE2A;FBXL14;CHFR;PML;MIB1;CNOT4;UBE2W;ITCH;S

EGFA;NFKBIA;P2RX7;PDCL3;MAPK11;OCLN;ITCH;IL6;ID2;FGF18;FAM98B;KRAS;NFE2L2;RIGI

SH;ANXA7;RHOH;BAZ1A;ASH1L;PARP9;SUPT3H;IL1A;HNRNPH2;GAS6;FOXC1;CRIP1;RELA;PPP2CA;PPP2

.R2A;UBR5;S100A13;RIPK1;NIBAN1;GSN;EPHX2;RDX;STAT3;SAMD4A;DTNBP1;RAB27A;INHBA;LARP4;B

PKD2;CDC73;PPP1R9B;RND1;MDK;CHMP1A;NUP62;ANXA6;NCK2;JAK2;KLF11;PBRM1;AXIN2;PLAAT4;[illegible]

JP;BCL11A;GADD45A;PBX3;SMARCA5;PBX4;SMARCA2;PARP15;PBX1;POU6F1;MLLT10;ID2;AGO2;REL;(

6;ACVR2A;VEGFA;MAD2L2;MOB1B;MOB1A;IL6;SLCO3A1;CLEC6A;FGF18;FAS;CALM3;TAB2;KRAS

;TRAF2;RHOC;PTP4A3;HAX1;PIK3CA;TRAF6;NOS1AP;TRAF5;PELI1;RAPGEF2;TNFRSF25;GAS6;BIRC2;BI

1;P2RX7;CXCL10;CXCL11;FER;IL6;P2RX4;NR4A3;TNIP1;TNIP2;TNFSF4;CDK2;GNB1;PDE3A;LAMTOR3;C

H1;FER;CDK6;WNK1;STK26;CDK2;UBA3;TAF4B;MAP3K13;CDK14;GSK3B;SMG1;BMPR2;LRRK1;PTPRO;

2;NF1;ANP32E;BCL2L1;AVEN;GSK3B;TNFAIP8;TRADD;HSPB1;PTMS;ADAMTSL4;AIFM2;TRIM2;NUP62;C
S6;BIRC2;CD44;BIRC3;PRKAA1;BCL2A1;IFI6;NOL3;FSTL1;MALT1;RELA;MAPK8;DNAJB6;PDPN;FLNA;PT

L7R;CD200;EZH2;BTG3;CDKN1A;CXCL9;BTG1;BMPR2;CXCL8;HILPDA;PTPRJ;PTPRK;PKD2;CDC73;PPP1R

RFXAP;REL;CREBRF;TAF4B;TCF4;MELTF;NFE2L1;ZNF410;NFE2L2;RIGI;CIITA;CD40;BMPR2;CHD6;WASL;

LIMK2;STAT3;PTK6;HIPK1;ACVR2A;FER;CDK6;TAOK3;WNK1;TAOK1;STK26;CAMK4;CDK2;TAF4B;MAP3K

AK1;PIK3C3;FAM20A;GAS6;CAMK1G;NIM1K;PRKAA1;CAMKK2;MAPK8;RELN;ACVR1C;PTK2B;PAK2;MA

ARP9;PPRC1;SREBF2;SUPT3H;RGMA;ZEB2;MED21;TET3;TFEC;ARHGEF2;FOXC1;DOT1L;RELA;FSTL3;CAM

?CB;GCNT2;APBB2;ARNT2;GSN;STAT3;VEGFA;PDCL3;BMAL2;BMAL1;CSF1;PDCD5;WWC2;RORA;JADE3;

MP6;WARS1;VEGFA;P2RX7;PDCL3;MAPK11;OCLN;IL6;CLEC6A;ID2;AGO2;BCL3;FGF18;FAM98B;KRAS;N

CTTNBP2NL;IL1A;IRF1;RAPGEF2;SINHCAF;CHMP5;ADAM22;MALSU1;TOB1;RNF2;PPP2CA;DLL4;SERTAD

CREBRF;MAGED2;HBP1;ZNF136;EZH2;CC2D1B;CD40;PHF1;BHLHE41;OTUD7B;WASL;MED16;LITAF;N4B

RC3;TRIM56;NFAT5;SLC20A1;PELI2;CXXC5;ATP2C1;MALT1;RELA;RELN;CCL5;GCNT2;FLNA;CCL19;APOL[illegible]

PRKAG2;PTPRM;ILK;PTPRJ;PTPRK;DUSP16;PHEX;PPP3CA;PPP3CC;TLK2;JAK2;JAK3;JAK1;ILKAP;DUSP5;P[illegible]

TLA4;CD38;CTSH;JAK3;HTATIP2;KLF11;PRMT2;TSC22D1;USP42;SPHK1;SERPINB9;TRAF2;MMP9;YWHA[illegible]

9B;MECP2;PPP3CA;NUP62;NCK2;CTSH;ITGAV;JAK2;KLF11;PBRM1;KSR1;TSC22D1;IL15;TBC1D8;EMP2;[illegible]

MED16;PKD2;LITAF;MED15;CDC73;PTMS;MED12;PPP3CA;MED14;MED13;HEY1;SIN3A;MED10;NCK2;[illegible]

P4K4;PAK4;MAP3K1;RYK;CDC42BPG;LIMK2;HIPK1;ACVR2A;MAPK13;TAOK3;TAOK1;CAMK4

IKK2;RELB;SCAF8;PLAGL1;STAT4;APBB2;TBL1X;BRD4;ARNT2;STAT1;PCGF5;MBD2;STAT3;ZNF76;GATAD

;PRDM2;JADE2;ETS1;CLU;FGF9;LAMP3;SIRPA;NANOS1;MAP2K1;ZBTB38;WNT5A;MITF;ATP1B1;FOXP1;

[illegible]2;GNG4;PDPN;SLIT1;PTK2B;TP53I11;E2F7;SUZ12;CDKN2B;SOD2;PML;WARS1;MYO1C;APC;INSM1;PTP[illegible]

P2L2;PTMS;MECP2;MED12;PPP3CA;MED14;MED13;HEY1;MED10;ELOA;IKBKG;HTATIP2;BATF2;PBRM[illegible]

'PP1R12A;PDPK1;MINK1;RABGGTB;YWHAZ;CTTNBP2NL;PPM1B;PEAK1;PIK3C3;FAM20A;GAS6;B4GALT

Z;RNF144B;HAX1;KCNMA1;AKT1S1;TAX1BP1;TMBIM6;TNFRSF25;ANGPTL4;GAS6;ARHGEF7;BIRC2;CD4

AXIN2;PLAAT4;IL1A;IRF1;RAPGEF2;MVD;SINHCAF;ARHGEF2;SOS1;BIRC2;DOT1L;TOB1;RELA;RNF2;DLL4

KBKG;HTATIP2;ACTR3;BCAS3;PPP1R12A;ANXA2;RIPK2;NCBP2;NCOA3;TCF12;ZFY;PPRC1;SREBF2;RGM/

[illegible]2B;GRIN1;VEGFA;MAD2L2;CAMK4;BMAL2;AGRN;BMAL1;CD86;CCNT2;CD81;CD80;RASL11A;RORA;PR[illegible]

ADAM19;TYROBP;PSPC1;ADTRP;TCF7;ILDR1;CREBL2;TAF5L;ZFP36L1;SBNO1;ATXN7;UBR5;RIPK1;MSC;

1;ZHX2;NCOA5;ANXA2;RIPK2;NCOA3;TCF12;BAZ1A;PARP9;PPRC1;ZFY;SREBF2;SUPT3H;RGMA;TOX4;N(

5;NIM1K;PRKAA1;TTL;RNF2;CAMKK2;PPP2CA;PPP2CB;MAPK8;ACVR1C;GCNT2;PTK2B;PAK2;MAP4K4;P

4;BIRC3;PRKAA1;BCL2A1;IFI6;NOL3;FSTL1;LRP8;MALT1;RELA;C3ORF38;MAPK8;ACVR1C;PDPN;FLNA;PT

;PDPN;GCNT2;STAT4;MARCHF7;PTK2B;TP53I11;E2F7;TCFL5;CDKN2B;KDM4C;STAT1;CNBP;STAT3;SOD

A;SENP1;ZEB2;HAX1;IRF4;THRAP3;MED21;IRF1;TRAF6;IRF2;TET3;TFEC;IRF7;ARHGEF2;NFAT5;FOXC1;PL

DM2;ETS1;PIM2;MAP2K1;ZBTB38;WNT5A;UBE2E1;MITF;MORF4L1;TBL1XR1;PHIP;SQSTM1;INO80D;K[illegible]

ZBTB17;BTN2A2;BRPF3;DENND4A;EPHX2;TDRD7;INHBA;CLK4;NFKB1;BMP6;NFKB2;IL6;CDK6;YEATS2;B

COR2;ZEB2;ZEB1;AEBP2;MED21;TET3;TFEC;ARHGEF2;SINHCAF;FOXC1;DOT1L;RELA;RELB;FSTL3;PLAGL1

[illegible]AK4;PTPN1;MAP3K1;RYK;CDC42BPG;LIMK2;PPP2R3A;HIPK1;ACVR2A;TAOK3;TAOK1;CAMK4;PTPN9

[illegible]AG1;WBP2;DOT1L;CEBPG;NR2C2;RELA;FSTL3;RELB;ATAD2B;MUC1;PLAGL1;TBX21;NRIP1;STAT4;ZNF7[illegible]

MT2A;CREBL2;FOXO1;TAF5L;SBNO2;ATXN7;RIPK1;BCL9L;ZBTB18;ZBTB17;MIR9-1HG;POU2F1;NEK4;INH

HLHE40;FGF18;CDK2;GRSF1;NFKBID;ZNF410;NFE2L2;RIGI;GSK3B;BMPR2;CXCL8;CHD6;TREM2;CHD3;A

L;CREG1;STAT4;HSF4;APBB2;LINC-PINT;TBL1X;HDX;BRD4;ARNT2;PCGF5;STAT1;USP9X;MBD2;STAT3;ZN

46;APBB2;TBL1X;E2F7;BRD4;H2AZ1;ARNT2;STAT1;PCGF5;CNBP;STAT3;FOXJ3;ZNF76;ACVR2A;GRIN1;V[illegible]

HBA;NFKB1;BMP6;NFKB2;NFKBIA;CXCL10;IL6;TFCP2;NR4A3;BCL3;MAFF;RFXAP;TCF4;MELTF;NFE2L1;ZN

AFF2;ZC3H7A;ZC3H7B;ZNF407;NFKBIZ;ITGB8;ERC1;KDM6A;TGIF2;EDF1;PTBP2;TAPBP;PTBP3;ACTA2;PT[illegible]

ıF76;GATAD2B;GRIN1;VEGFA;MAD2L2;NR6A1;HDGF;BMAL2;AGRN;BMAL1;CCNT2;ZBTB24;CD81;UBE2

IF410;NFE2L2;RIGI;CIITA;BMPR2;CHD6;ILK;CHD3;PKD2;CDC73;SIN3A;NUP62;NCK2;UBTF;ACTR3

P4A3;TRAF3;RNF207;IRF2;NOS1AP;TESMIN;ZNF513;PRKAA1;SLC24A3;PLAG1;TULP3;NR2C2;TOB1;RN

D3;WWC2;PRDM2;RORA;JADE3;ARID4B;PRDM1;JADE2;ETS1;NR3C2;ARID2;ZNF684;PIAS3;MORC3;

Table S10

CCR7+cDC cluster 1			CCR7+ cDC cluster 2		
gene	avg_logFC	adj. p-value	gene	avg_logFC	adj. p-value
FCGR2B	-6.20794266	1.9849E-39	ADAM12	7.20494718	2.6255E-22
SLAMF8	-6.10037582	4.072E-72	LIMCH1	6.87317954	6.9325E-45
CD207	-5.75821966	9.4343E-33	ENOX1	6.80239833	1.5096E-28
P2RY6	-5.65524461	4.9834E-23	NCCRP1	6.32966727	8.2339E-11
CPVL	-5.58448661	1.6863E-68	ST8SIA1	5.9116864	4.7886E-17
C1orf162	-5.48253713	1.3738E-19	DLGAP2	5.80979379	4.0253E-23
TNFSF10	-5.21600414	1.2494E-45	TMEM150C	5.540036	1.2376E-14
HCAR2	-5.21509775	3.5709E-23	BICDL1	5.36458352	3.2855E-16
RAB24	-5.09695017	2.3682E-65	NRXN2	5.33950614	8.5444E-18
RNASE6	-5.09333877	1.4005E-30	WNT5B	5.31481145	1.9407E-15
ASB2	-5.07503111	4.7968E-21	PDZD2	5.30998672	2.295E-16
MS4A6A	-5.06519749	3.1133E-59	SMOC1	5.30607357	2.2927E-16
SIGLEC10	-4.97773592	3.1763E-40	KIF26B	5.21541867	3.7808E-13
TMEM273	-4.97245025	1.1558E-23	S1PR1	5.1666533	7.0203E-26
LILRA1	-4.96132723	6.8924E-26	CD200	5.15736162	1.3891E-42
CKLF	-4.94743217	3.7541E-30	ENTHD1	5.08305662	1.8826E-18
ABI3	-4.74755373	1.0717E-22	SLC22A23	4.8990218	8.6461E-47
FCER1A	-4.73472368	5.2614E-10	NPR1	4.89228353	2.3053E-08
CSF1R	-4.64677016	1.99E-23	DAB1	4.84558342	6.4544E-11
TAGAP	-4.61622631	1.239E-41	SYNPO	4.82177839	7.0423E-28
TGFBI	-4.59676018	3.1052E-56	ANO9	4.78493322	0.00417011
ASGR2	-4.59323053	2.3367E-17	AOC1	4.71257696	5.1702E-18
FCGR1A	-4.52308092	1.5666E-25	ENSG0000022	4.67987025	1.6043E-18
CLEC4A	-4.48858167	1.9838E-33	CDHR3	4.67486025	3.3774E-06
LAIR1	-4.47626046	3.5944E-31	NRG2	4.65053362	2.8889E-06
PLAC8	-4.44907736	1.4796E-41	SLC24A3	4.59682192	5.1052E-19
OSM	-4.41565655	6.1952E-08	RNF150	4.53355257	0.00019754
MPEG1	-4.37171219	1.1042E-18	PLEKHG1	4.52575802	1.2128E-29
THBD	-4.37087092	1.2477E-51	FBXO27	4.39273456	6.2901E-13
FCGR2C	-4.36836108	5.382E-32	SH3PXD2A	4.3900692	8.8275E-34
RAC2	-4.34383485	1.1807E-22	SYNPO2	4.37853654	9.2778E-20
PYCARD	-4.3305085	8.6779E-68	KCNH3	4.36888611	0.00107384
MEFV	-4.28056333	9.8583E-30	ACHE	4.35538447	2.12E-32
CCR1	-4.27446386	1.0121E-13	FBN1	4.30536686	9.8532E-07
MRC1	-4.26990795	1.0112E-30	TPST1	4.26913167	2.1878E-27
FCER1G	-4.24386067	1.6139E-21	GPC5	4.26439917	2.3172E-28
C1QB	-4.21693015	1.1031E-14	SLCO5A1	4.26206877	6.2438E-33
RGCC	-4.20735069	5.2453E-50	NLGN4Y	4.23427397	0.0129788
SAMHD1	-4.2061849	2.0648E-54	LINC03025	4.20368142	5.5234E-13
UTF1	-4.19594794	1.2491E-15	LINC02362	4.19951769	4.5131E-11
CD300A	-4.18919398	2.4721E-34	ADTRP	4.19682424	4.9797E-16
IFITM1	-4.18569334	8.5025E-29	MCF2	4.18827212	5.1722E-12

FBP1	-4.17983113	2.2694E-21	NXPH3	4.17941196	5.0384E-06
CYBB	-4.17144286	2.7845E-28	TMCC2	4.16027221	3.6814E-08
PALD1	-4.16719441	1.1761E-10	SCN11A	4.14559097	4.1545E-15
FPR1	-4.11718473	5.9442E-27	GRIN1	4.14170707	4.4494E-06
CLEC10A	-4.09294426	4.5375E-22	HOPX	4.12515784	9.194E-17
CD300C	-4.09219524	7.5548E-22	GCSAM	4.11306429	6.256E-24
FGL2	-4.08058711	2.2672E-30	CELSR1	4.10227378	1.9822E-05
IL1R2	-4.06173582	1.4343E-10	ARNT2	4.08899954	1.0067E-06
HIC1	-4.05495806	1.0174E-17	CASC15	4.07695464	8.3528E-15
GPAT3	-4.00236263	3.832E-32	INSM1	4.06632717	2.3708E-08
SCIMP	-3.97672729	8.6959E-27	NOS1AP	4.05015065	5.0365E-23
ST14	-3.9692576	3.3743E-13	FSTL3	4.02964318	3.532E-10
LTB	-3.95266859	7.9555E-23	DNM1	4.01123516	9.0297E-10
SIGLEC7	-3.94952983	1.6147E-11	TBC1D4	3.95956919	8.8635E-29
IGSF6	-3.94938142	1.6761E-43	LINC01358	3.95043511	1.5041E-17
CD33	-3.92376122	4.9787E-14	KCNN1	3.94703763	3.8474E-16
C1QA	-3.87671347	3.8527E-13	USP54	3.93598318	2.9723E-16
IL1RN	-3.87576535	8.2506E-20	IKZF2	3.88694948	1.161E-07
IMPA2	-3.86821947	2.9283E-15	ITIH1	3.86275459	0.00362452
PTPRE	-3.86132404	4.1864E-39	UPB1	3.85982628	3.3843E-07
GAPT	-3.8432059	2.0343E-13	DEPP1	3.83817549	2.0324E-12
CD14	-3.84218165	5.1179E-12	ROBO1	3.83668075	2.471E-20
PDLIM2	-3.82461697	6.1535E-06	ANKRD18DP	3.83400669	0.00010984
FCGRT	-3.81845094	5.0835E-32	TNFRSF11A	3.83001317	2.4242E-16
MPZL2	-3.80087104	1.9241E-13	MERTK	3.815026	7.8629E-25
IL1B	-3.78081977	2.6539E-58	EDARADD	3.78749718	1.835E-25
HBEGF	-3.7574935	3.1822E-06	CCDC68	3.78623138	4.2382E-14
CACNA2D3	-3.75533188	1.364E-08	TC2N	3.77974259	0.00011052
CMTM3	-3.74924217	2.2954E-17	NTN1	3.7619673	7.2579E-18
ITGB7	-3.74701177	6.1192E-15	EYA4	3.7575051	1.8596E-15
HRH2	-3.74487373	1.0952E-27	TTN	3.75200534	1.769E-06
SLC2A3	-3.73787753	1.541E-39	PSD3	3.73084603	8.32E-30
MYOF	-3.73384249	3.0053E-32	NMRK1	3.71692472	6.772E-22
LRRC25	-3.72858402	8.2022E-10	ZFPM2	3.71573761	1.1277E-21
EVI2A	-3.72521527	1.2132E-28	CNN3	3.71006271	2.7667E-12
LILRA2	-3.71724839	2.478E-12	ENO3	3.70424736	1.2099E-08
SIGLEC9	-3.70388046	1.2142E-05	EDIL3	3.66335117	0.0003611
ARHGDIB	-3.69412545	4.8255E-54	ACVR1C	3.64867798	0.01660189
C1QC	-3.69118269	4.6727E-35	CIT	3.63387301	0.00013624
MMP12	-3.67690145	6.2323E-06	RGS6	3.62564091	2.905E-10
CD93	-3.6763007	2.1895E-20	WLS	3.6232822	0.0057316
ANXA1	-3.67112066	9.1447E-27	SCD5	3.60745195	0.01752365
PLAUR	-3.66597075	4.9021E-27	TLCD5	3.59641956	1.4428E-05
PID1	-3.66338798	2.0455E-17	ABCD2	3.59391719	2.5991E-07
ADA2	-3.65326706	1.7358E-21	APOE	3.58606872	0.01298287
GASK1B	-3.63804522	0.03984576	ANKRD33B	3.57348291	2.951E-27

CORO1A	-3.6332451	9.3013E-39	ROR1	3.54583421	1.0563E-09
SPNS3	-3.62532952	0.0102218	ENSG0000028	3.53057967	0.00719865
FPR3	-3.60500849	3.3793E-25	CCL19	3.52464793	1.357E-21
AIF1	-3.60091437	1.4044E-38	GPR39	3.49598617	0.00258629
GPER1	-3.5972131	1.3035E-05	C17orf99	3.49112435	4.1378E-10
LY86	-3.58332803	5.8503E-18	HEY1	3.47753672	0.00075055
DUSP6	-3.56134278	1.4407E-16	EPB41L4A	3.46964027	1.4073E-08
CAMK1	-3.55806141	3.1531E-12	L3MBTL4	3.46499427	6.2488E-28
THBS1	-3.55047154	4.4949E-23	SPOCK2	3.45516531	0.03371737
PTPN6	-3.53024728	1.046E-46	ZBED3-AS1	3.44938167	0.00011504
CYSLTR1	-3.51773298	2.8544E-07	NAV2	3.44568183	4.9078E-13
ADGRE3	-3.51568569	1.2234E-11	CCDC81	3.44248089	8.3536E-08
SLC8A1-AS1	-3.50522146	7.2765E-09	CRACD	3.44104185	2.0029E-11
VSIG4	-3.50234711	4.9822E-09	DNAH11	3.44016582	1.3678E-09
ITGAX	-3.49002836	1.7867E-28	MIA	3.42694033	1.6596E-05
IFIT2	-3.48774453	4.2493E-05	GRIK4	3.42357531	0.0352687
S100A4	-3.48673221	1.4804E-19	TSPAN13	3.42239978	1.3685E-18
MARCHF1	-3.48483055	4.1241E-17	LINC01094	3.41930964	0.01584064
PLD4	-3.46340327	9.5593E-23	TNFAIP8L3	3.41337218	1.3438E-07
LCP2	-3.44196184	7.4549E-29	LY75	3.41076053	3.7663E-65
CEBPB	-3.4391948	6.5641E-27	FRMD4A	3.40902691	1.5655E-08
CD101	-3.43876844	2.4261E-06	MARVELD2	3.40818412	0.02082778
CD300LB	-3.43252254	2.1038E-08	PC	3.40341713	5.5839E-12
CAMKK1	-3.42875933	0.00370197	NEXMIF	3.38696558	2.0429E-10
IL16	-3.41316183	4.9221E-31	PARD3B	3.382267	0.00032882
NDRG2	-3.41152338	1.9597E-22	CKB	3.38039015	7.7756E-12
NCF2	-3.40066684	3.5758E-31	DMGDH	3.36919146	6.7441E-10
IFIT3	-3.40064964	9.3265E-14	MSC	3.35035495	0.03410055
CLEC2B	-3.38765334	4.1545E-43	MEIS1	3.34814626	0.00057322
MS4A4A	-3.38443581	8.6634E-15	ATOH8	3.34669812	0.00352736
FCGR2A	-3.38429112	3.8978E-17	HMSD	3.33774017	1.6422E-21
PRELID1	-3.38413359	1.1803E-78	ENSG0000028	3.33733098	4.2291E-06
GIMAP4	-3.38028989	5.5538E-23	ZFP69B	3.3304572	0.02507619
HAS1	-3.36551012	1.3644E-05	LINC02384	3.31907062	3.6329E-10
GHRL	-3.35838951	2.1114E-05	CD70	3.31506192	1.4969E-15
LILRA5	-3.35129638	4.5375E-22	ANXA3	3.29786364	2.1122E-16
RSAD2	-3.34858646	0.00279081	HELLS	3.2907	7.3186E-14
RNASET2	-3.34762429	4.8414E-30	KCNK5	3.26240633	3.3506E-05
CD163	-3.34674437	4.1039E-10	PVR	3.25855441	2.1005E-14
CMPK2	-3.3459926	1.0055E-12	MBOAT2	3.25501267	7.2335E-40
PELATON	-3.34398834	8.1196E-13	IL32	3.25383734	9.6503E-21
CIDEB	-3.34275091	0.00931365	MIR34AHG	3.24784523	4.1068E-12
ENSG0000026	-3.34150869	3.2203E-07	ABCC4	3.24046346	1.0019E-47
CHN2	-3.33044392	3.2111E-15	MYH11	3.23895809	2.2975E-06
FCGR1BP	-3.32997937	1.3704E-05	TCF7	3.23578197	1.3019E-08
PTGS1	-3.31248063	6.6289E-06	ENSG0000022	3.23015161	0.01016482

LINC02207	-3.31195627	0.00056086	ZNF260	3.22439034	4.0681E-10
APOBR	-3.30551619	1.0587E-12	STRIP2	3.20886643	1.1989E-12
CCR5	-3.30219458	0.00929091	EBF1	3.20329531	5.8896E-06
NLRP3	-3.2990315	6.4008E-22	MAPK13	3.20119573	6.1363E-09
RAB20	-3.28002229	4.0506E-49	CDYL2	3.19691387	2.7015E-20
IFITM2	-3.25785964	5.3767E-51	LNCTAM34A	3.1888265	0.04663487
CD1D	-3.25356816	3.0995E-15	JAG1	3.18834172	7.4156E-08
RARA	-3.24724883	1.2916E-17	ST3GAL6-AS1	3.18156768	0.00089372
OLR1	-3.23282927	2.3302E-12	KIF2A	3.17961959	5.2633E-77
CXCL10	-3.23226067	6.0971E-16	RASGRF1	3.17912955	8.1058E-20
FGR	-3.2301386	5.1185E-69	FSTL1	3.163847	0.02819887
PTAFR	-3.22547313	1.1785E-20	APOBEC3H	3.16204516	0.03838497
P2RY13	-3.2184421	7.8756E-18	DLEU7	3.15400757	6.6594E-05
CD69	-3.21699068	8.15E-28	DYNC1I1	3.14602231	0.00001484
MS4A4E	-3.21399	0.00411535	ENSG0000022	3.14316057	1.2356E-12
CD48	-3.19284773	8.7484E-48	PLAG1	3.14000848	0.03321849
TRPM2	-3.18653699	4.1952E-06	LMCD1	3.1360147	4.0619E-05
APOBEC3A	-3.18360149	0.00168103	CBLB	3.13485614	4.2077E-35
ALOX5AP	-3.18219036	1.9719E-33	ARHGAP10	3.13285876	7.2292E-42
LILRB1	-3.17832504	8.7422E-20	ADORA2A	3.12925461	1.0838E-13
TREM1	-3.13619442	2.7288E-07	FBXO2	3.11685835	0.01766034
SATB1	-3.13194205	1.4099E-27	CEACAM16-AS	3.09781658	1.144E-11
CLEC4F	-3.12194221	0.00475632	SOX5	3.09330555	6.9435E-12
EML4-AS1	-3.11835097	0.04142053	NRP2	3.08735011	2.0745E-07
MGST2	-3.10013171	6.5474E-19	MACROH2A2	3.08029109	1.729E-40
KCNK6	-3.08985174	1.564E-25	NAALADL2	3.0796654	0.00111087
PRAM1	-3.08475468	1.6854E-06	CCL22	3.07357097	1.2923E-07
TUBA4A	-3.08462005	1.6605E-06	ENSG0000022	3.07309944	7.579E-05
CD53	-3.0734027	7.2037E-43	LAD1	3.06469914	5.7247E-18
LY9	-3.07310376	1.1564E-05	NDE1	3.06406755	1.3487E-24
MTFMT	-3.05805513	2.121E-05	CCDC73	3.048324	0.00314953
ARHGAP18	-3.03482041	1.6901E-10	LINC01301	3.04622505	0.00292388
SCARF1	-3.03167663	7.1775E-20	ST3GAL6	3.02300044	4.2298E-22
HCAR3	-3.01872454	1.5136E-10	MPPED2	3.02078166	0.00291531
CEACAM4	-3.01422303	5.8955E-18	CHRFAM7A	3.01711192	0.00384402
SLC40A1	-3.01187268	2.2949E-09	ACVR2A	2.99726222	3.5521E-07
NUDT16	-3.01057772	4.704E-37	MTSS1	2.99293834	6.6468E-16
TBXAS1	-3.00732423	1.682E-33	MCC	2.990918	1.9679E-11
ADISSP	-3.00635967	8.1231E-18	IL23R	2.98787612	0.0006028
SPN	-2.9988677	2.0666E-13	PTPRK	2.97066622	0.01264145
MIR223HG	-2.99472858	0.00016902	DSCAML1	2.95987648	1.5229E-12
GBP5	-2.98372702	2.8549E-18	BTLA	2.95852923	6.9781E-10
SUSD3	-2.97777611	1.8296E-08	SSPN	2.95314935	0.00026145
CLEC4E	-2.97696499	8.4368E-21	LINC02227	2.94191586	5.0722E-05
B3GNT5	-2.9767236	3.1942E-07	BMP2K	2.93993105	2.0201E-42
HACD4	-2.97652279	1.3076E-26	BIRC3	2.92947938	2.0641E-32

PTGER2	-2.97646591	0.0001233	ENSG0000028	2.92862302	2.4413E-05
PLCB2	-2.97559812	6.2374E-10	LAMC1	2.91811333	0.00875089
CDH17	-2.97159189	1.6486E-05	ZNF608	2.90952731	9.042E-05
GPR82	-2.95990833	7.2581E-05	ENSG0000022	2.90886938	7.4189E-10
CALHM6	-2.95301097	8.8284E-26	TNFSF4	2.90719948	1.2722E-08
MYD88	-2.94995132	1.2666E-31	ENSG0000029	2.90547209	2.7995E-06
TNFRSF21	-2.94991911	3.3323E-17	ADAM7-AS1	2.89413682	2.4103E-15
C3AR1	-2.94127889	1.4296E-07	SDK1	2.8854542	0.0001445
CD1C	-2.93978019	2.0116E-06	CCR7	2.88357675	2.6577E-10
ASGR1	-2.93699019	9.2603E-06	MGLL	2.88302656	8.0519E-16
TIMP1	-2.93658166	2.492E-21	SLC9A9	2.83925226	5.2943E-38
FPR2	-2.9350554	7.4125E-09	HSD11B1-AS1	2.83855354	2.0223E-10
EVL	-2.93191098	7.4183E-11	CRLF2	2.83592776	1.1044E-16
TMEM8B	-2.93086349	0.00040741	ZFTA	2.83468449	0.00135646
DOK3	-2.92198547	5.2432E-05	CDKN2B-AS1	2.82984707	0.00031848
ADAM15	-2.90756091	1.8184E-08	LINC00598	2.81989466	0.02092334
MAP3K5-AS2	-2.89911135	1.6926E-05	PLEKHA6	2.81688759	1.6926E-05
SLC8A1	-2.89881873	4.0311E-46	MBNL2	2.81360666	1.8876E-26
MAP4K1	-2.88073731	1.5751E-14	KDM2B	2.80877661	5.6081E-32
ENSG0000026	-2.88035235	0.04388081	ICAM5	2.80718745	3.9473E-08
IL18R1	-2.8655125	6.7456E-12	CD247	2.79768767	0.02193444
CLEC17A	-2.86527983	0.00947978	CGN	2.79682561	0.00492934
LST1	-2.86490461	1.1535E-26	CYP7B1	2.79650091	5.2925E-11
SELL	-2.86123125	8.5909E-13	RBPMS	2.7936325	0.00014788
SH3BP5	-2.85907984	5.5298E-16	DAAM1	2.79051028	7.512E-19
ARHGAP4	-2.85553124	1.023E-13	TMEM72-AS1	2.78640211	1.9311E-07
RGS14	-2.84849541	0.00912788	LINC00158	2.78270985	0.00064239
AXL	-2.84180886	2.7134E-15	ZEB1	2.77741973	4.7411E-27
VASP	-2.83883321	1.2152E-21	FSCN1	2.76186287	2.5816E-18
CD300LF	-2.8310399	2.8963E-10	SPEF2	2.75689495	0.00052169
EIF4G3	-2.82870091	9.8084E-44	MTA3	2.74729719	5.6898E-16
SEMA6B	-2.828597	2.5645E-29	GNG11	2.7381072	1.8212E-05
GALM	-2.81433579	7.1921E-08	C3orf52	2.73176638	1.7574E-11
CLEC6A	-2.80977257	1.6853E-09	NOSTRIN	2.72756162	0.00010677
ATP8B4	-2.8074607	4.0842E-17	ENSG0000026	2.72733592	0.02631909
ARL4C	-2.80582575	4.789E-43	PHLDA3	2.71453943	4.7194E-07
MFNG	-2.79447516	1.4313E-13	RABGAP1L-DT	2.71130407	0.04358887
DUSP23	-2.79128246	7.5346E-20	ABCA3	2.70522306	0.00018041
CLEC7A	-2.7882877	2.9422E-28	ADGRD1	2.70042395	1.137E-10
VPS9D1	-2.77595501	5.7768E-08	ANKRD55	2.69709285	8.0597E-07
GPR35	-2.77336517	2.0638E-15	NAA25	2.6890754	5.289E-18
SH3PXD2B	-2.75820989	1.0213E-14	KLF5	2.68637389	3.4706E-09
ENSG0000023	-2.75035506	0.00039194	PLAAT3	2.67817579	4.1441E-09
CD52	-2.74785417	2.0796E-32	TVP23A	2.67148825	7.1775E-20
ZFP36L2	-2.74507966	4.4758E-11	IGKC	2.66449023	2.7693E-10
PLB1	-2.74454529	2.9672E-06	RAMP1	2.65785194	6.5576E-15

CFI	-2.74044355	0.0160193	EFNB2	2.6564486	1.9976E-06
CYTH4	-2.74034963	3.0763E-15	MSC-AS1	2.65145958	2.2121E-08
OTULINL	-2.73887952	1.1006E-20	PDCD1LG2	2.64601694	3.9137E-13
ARHGAP20	-2.73836298	7.4568E-07	H6PD	2.6423629	1.3357E-16
ATP2A3	-2.73315573	7.6917E-07	AUTS2	2.63257549	1.3341E-29
ACP5	-2.73127109	4.6207E-13	TNNT2	2.63224303	1.365E-06
TCTN3	-2.72673863	1.2803E-22	TSPAN15	2.62361034	8.1116E-25
PHACTR1	-2.72172162	4.4668E-11	ENSG0000029	2.62129149	0.03562121
TNFSF13	-2.71726449	2.5483E-09	ENSG0000028	2.61083777	2.5361E-06
ARHGAP9	-2.71294736	2.0696E-11	LINC02397	2.59583744	0.0065059
ADGRE2	-2.69966635	1.5229E-24	ENSG0000028	2.59467729	0.00347749
FCMR	-2.69566656	0.00018298	STEAP1B	2.59249618	7.419E-06
GMFG	-2.69161984	2.8484E-29	SNX11	2.59236912	1.9277E-16
TM6SF1	-2.68916444	5.3109E-06	GRSF1	2.59144818	3.6261E-48
SECTM1	-2.68879806	5.294E-25	CNKSR3	2.58878543	2.5924E-08
GPRIN3	-2.68605998	2.8556E-22	ABTB2	2.58270337	3.0465E-15
CST3	-2.68537528	2.4451E-28	PERP	2.57627625	0.00588915
CLEC12A	-2.68027545	0.04844951	PSEN2	2.5707505	1.0191E-21
GIMAP8	-2.67936657	0.00069857	PLCL1	2.56641204	1.4873E-16
PTGS2	-2.67787229	6.114E-10	CDT1	2.56626918	0.01121666
EVI2B	-2.67215567	8.9223E-22	SCUBE2	2.56409856	4.8658E-05
AQP3	-2.66439937	0.01034854	SDK2	2.56384206	0.03083956
IL1RAP	-2.66432702	3.7615E-21	RRAGC-DT	2.5620059	4.749E-11
MS4A7	-2.66419768	3.1579E-09	MAML2	2.56156238	7.283E-14
GNG10	-2.66270101	8.655E-30	IGF1R	2.55789467	1.1451E-13
FSIP1	-2.65648663	0.00047776	KLF12	2.55307307	7.6424E-25
PARVG	-2.65348783	7.6085E-23	RCL1	2.55273643	3.2053E-19
ABCB4	-2.65046811	0.00088044	LPAR1	2.54800321	1.4113E-12
TTC39C	-2.63916902	0.00011074	ERICH1	2.5475877	5.1185E-69
AREG	-2.63771157	1.5476E-29	LINC01814	2.54416674	0.00014046
YIF1B	-2.63591285	5.5538E-23	GAB1	2.53821936	7.839E-21
GNGT2	-2.63221095	8.9471E-09	ALPK2	2.53410979	1.7533E-18
GPR84	-2.62621263	0.00507837	TTC28	2.53179177	0.00026078
PLA2G7	-2.62571046	1.6432E-10	POGLUT1	2.53047299	1.2251E-31
ACSL5	-2.62412226	1.5237E-11	FMNL1-DT	2.52416364	1.6482E-22
KCNAB2	-2.62336489	3.4739E-22	CLU	2.52325619	0.02607103
PMP22	-2.62209897	2.1595E-13	CERS6	2.52289742	9.8221E-23
MIR3945HG	-2.61849546	2.4353E-09	EVA1C	2.51966771	1.0666E-06
SH3BP5-AS1	-2.61587169	2.4149E-06	LRIG1	2.5139938	1.5495E-07
CASP1	-2.61510317	5.7563E-33	ATP8A1	2.50687204	1.7144E-18
PLXDC2	-2.61048463	4.5046E-17	LRFN4	2.49130488	3.5898E-05
NEDD9	-2.60931095	2.0337E-21	GPR55	2.48763944	3.0682E-05
FLT1	-2.60874015	8.6798E-25	DMD	2.48506006	1.7376E-05
XAF1	-2.60552181	1.0305E-19	MXI1	2.48461609	3.0602E-09
LGALS8	-2.60094996	1.3909E-20	PDGFC	2.48352844	1.731E-16
MLKL	-2.59948483	1.274E-07	LINC01857	2.48138716	4.4283E-08

FYB1	-2.58821113	2.0395E-23	AGBL3	2.48003877	2.7563E-05
CD300H	-2.58612326	0.01146183	ENSG0000028	2.47923699	3.9116E-13
DAB2	-2.58362772	8.5359E-08	MLLT11	2.46772333	0.00020848
EREG	-2.58330561	5.1052E-19	ZBED3	2.4656105	4.7733E-05
SKAP2	-2.57578329	1.1982E-16	ORMDL3	2.46207623	1.1098E-11
HOXA5	-2.57285471	8.1203E-07	ACKR3	2.46085503	2.0259E-08
ENSG0000026	-2.57036606	1.1582E-09	PACS1	2.45740964	3.63E-24
SDS	-2.56727333	1.1617E-12	GPR153	2.45500576	7.3097E-18
CCDC26	-2.56355304	0.00040501	DLEU1	2.45499898	3.4002E-58
SLCO4A1	-2.55405889	2.9816E-05	ENSG0000028	2.45229916	0.00312691
GALNT6	-2.55015553	0.00465964	LINC03062	2.4479656	1.0951E-07
NEURL1	-2.54924671	1.9772E-06	MMD	2.44774721	4.049E-18
IFITM3	-2.54392637	2.7075E-26	SRSF12	2.44762034	2.9017E-07
ENSG0000022	-2.53800445	0.01058202	CHD3	2.43608779	1.0363E-39
ENSG0000028	-2.53266635	6.596E-11	MIR193BHG	2.43422782	0.00188088
SNAI1	-2.53120973	7.3441E-21	GCNT2	2.43110774	1.4799E-09
PLIN2	-2.52961936	7.0435E-25	LINC02541	2.42823413	6.3129E-05
SLC37A2	-2.52785967	5.3218E-08	TREM2	2.42759198	0.01158331
LMNA	-2.52538497	1.4691E-12	TRIP10	2.42743209	2.7634E-13
TGFB1	-2.52089783	6.6194E-23	MDK	2.41979159	0.00062803
TLR2	-2.51971747	1.026E-14	PRAG1	2.41780122	2.1708E-06
KCTD12	-2.51721933	1.5275E-20	TFPI	2.41636589	2.8065E-07
OAS3	-2.515729	3.5396E-07	ERVK13-1	2.40806179	1.5553E-07
EIF4EBP1	-2.51524851	1.1366E-26	JADE3	2.40693597	4.3487E-15
LILRB2	-2.51072131	4.0609E-11	ADORA2A-AS1	2.40635152	0.02524481
FCN1	-2.50962805	0.0026275	ATP11C	2.40471741	1.1375E-22
CEACAM3	-2.50806604	6.9815E-05	DUSP5-DT	2.40471211	2.1958E-09
SLC12A6	-2.49829107	1.6856E-08	MIR548A1HG	2.40322278	5.741E-05
MMP19	-2.49617515	1.0296E-10	FUT10	2.40276585	1.7816E-07
LILRB3	-2.48955058	5.0393E-13	PRRG4	2.40011832	1.138E-25
TLR10	-2.48883704	9.1093E-05	CXCL13	2.39984157	0.04086012
KDM7A-DT	-2.4877603	3.9217E-05	ENSG0000026	2.39893156	0.00709305
CARD9	-2.4816488	5.419E-09	TTN-AS1	2.39873642	7.9068E-12
NAIP	-2.48143763	3.1678E-07	LINC02954	2.39815566	0.04358887
FHIP1A	-2.47798319	2.9909E-13	XYLT2	2.39489813	0.00149712
METRNL	-2.47793324	4.1267E-30	LRP8	2.39327565	8.3046E-07
FAM110A	-2.47489859	1.001E-19	FTX	2.39272685	5.2461E-15
NIBAN2	-2.47359763	2.7948E-08	SGO1-AS1	2.39251773	1.0131E-07
JAML	-2.47146069	1.4568E-09	CCDC102A	2.39027394	0.00763545
MSR1	-2.46917129	0.00025525	PAWR	2.38833545	0.00845857
KIFC3	-2.46300452	0.01973717	AUH	2.38618976	1.2658E-12
GPX3	-2.45573522	0.00015591	IL15	2.38515233	8.3926E-40
CD300E	-2.45358171	0.01310283	MOB1B	2.38305015	1.1401E-25
DCAF12	-2.4523149	0.00469223	ENSG0000028	2.37749079	0.02154316
RGS18	-2.45131	6.2018E-07	DOCK3	2.35856116	0.00810307
EXOSC5	-2.44658366	8.8154E-14	KLF7	2.357805	1.487E-09

CD244	-2.44574387	0.00271698	GIPC2	2.35720403	0.03187554
DDX60	-2.44533739	0.0002479	RANBP17	2.34872567	0.03039335
VAV3	-2.43441465	1.9265E-08	ZNG1F	2.34343289	0.00061963
GLRX	-2.43232444	6.9273E-11	ABCB10	2.3432037	3.0405E-21
ENSG0000028	-2.43107518	0.00381733	LMLN	2.34230936	0.00305319
STAB1	-2.42576383	0.00975837	ETS1	2.3370043	4.8606E-22
QPCT	-2.42386581	1.6496E-16	BMAL2	2.331744	1.255E-16
RYR1	-2.41870599	0.01297368	ENSG0000027	2.33128666	0.0241263
FAH	-2.41759666	0.00022358	ENSG0000024	2.3296749	0.01942418
RUFY4	-2.41752855	0.00012903	ENSG0000029	2.3295079	0.012536
MCEMP1	-2.41385191	0.00029238	PDGFA	2.32805703	0.00023605
TNFAIP8L2	-2.39755344	2.5311E-11	P2RY10	2.31746368	2.5089E-14
PIK3AP1	-2.39214549	1.7889E-34	CCDC66	2.31657194	1.4108E-07
ENSG0000025	-2.3899434	2.0959E-07	ENSG0000027	2.3162879	0.00704436
MAML3	-2.38739481	9.0508E-08	NAV1	2.30723857	6.3025E-21
CLIC1	-2.38625818	2.8363E-25	PTGIR	2.30591856	4.9418E-08
SPRY2	-2.38553982	0.00183203	PAQR3	2.30382693	9.6994E-18
VASH1	-2.38518294	3.0499E-13	SLC5A6	2.30033997	3.9388E-17
TLR4	-2.38485247	1.341E-07	SLC8A3	2.29398457	0.03842699
LAPTM5	-2.3810965	1.6617E-27	ZDHHC13	2.290125	8.4308E-09
CSF3R	-2.37843501	3.6163E-17	SPPL3	2.28978459	1.7114E-28
CALCOCO2	-2.37758364	1.1546E-53	ENSG0000026	2.28941154	0.03791153
PRADC1	-2.37255669	2.1718E-05	CCDC28B	2.28540941	5.0708E-11
ICAM3	-2.37160191	1.0008E-10	NBL1	2.28224292	0.02601322
CLEC11A	-2.36899884	1.0094E-10	TCEANC	2.2801381	0.00019473
SAP30	-2.3687499	1.3564E-12	ENSG0000027	2.27738838	0.00286615
CD72	-2.36178996	3.9085E-15	POU6F1	2.27487387	0.00011572
SERPING1	-2.36089715	5.5697E-10	SLC1A2	2.2717682	7.2692E-05
NUBPL	-2.35543054	8.6109E-10	MINK1	2.26953184	1.3831E-19
APBB1IP	-2.35484407	6.1695E-18	ITPR3	2.26629558	0.02223108
LPXN	-2.35410813	1.5041E-19	COLEC12	2.26070907	0.03764901
MCUB	-2.34905202	7.4199E-21	ANXA6	2.2594491	2.073E-42
EEF1AKMT4	-2.34521583	0.0193665	PCED1B	2.2568576	0.02291275
HAVCR2	-2.34457249	2.3943E-17	C3orf18	2.25340005	0.00163243
GLIPR1	-2.33835283	5.4152E-18	CHST7	2.25194316	2.0131E-12
OAS1	-2.33335367	7.0729E-07	CRIP1	2.24513779	3.2857E-21
GZMB	-2.33183472	0.00192522	NQO1	2.24297654	0.03317272
ZNF331	-2.32703865	6.4912E-11	NPIPA9	2.24207381	0.00665498
ITGB2	-2.32540445	1.3804E-18	CLCF1	2.24149839	3.6055E-06
CEACAM21	-2.3219805	0.00481137	TUBB6	2.23829487	3.3852E-20
STARD3	-2.32021372	3.144E-11	ID2	2.23584295	9.9985E-17
AP1B1	-2.31983325	5.1748E-22	RAPH1	2.23507429	2.3311E-15
ANXA2R	-2.31253234	0.01841247	MIR17HG	2.23482183	5.6831E-05
DEF6	-2.31218637	3.4988E-21	APOC1	2.23322099	4.346E-30
IL18BP	-2.30691935	3.2477E-22	RAB30	2.23203389	7.6085E-23
GAS7	-2.30601893	1.0826E-16	TBC1D32	2.23099042	4.312E-09

GBP1P1	-2.30484125	3.5396E-07	DCAF6	2.22952483	5.7844E-22
CYSLTR2	-2.30468494	0.00045438	RPS27L	2.22858533	2.1042E-59
CD1A	-2.30080502	0.00941898	NR6A1	2.22001488	1.797E-16
CALHM2	-2.30008192	4.8607E-06	LINC00299	2.21569837	0.00133421
RNF166	-2.29711512	1.0198E-10	FMNL2	2.20999194	1.2035E-09
ADGRE5	-2.29418228	1.8854E-21	TNFRSF9	2.20824431	6.5714E-24
CYTOR	-2.29388055	3.2027E-13	PITPNC1	2.19991377	6.0266E-12
WAS	-2.29131961	9.2779E-42	ENSG0000029	2.19960476	3.4611E-10
AOAH	-2.28996267	8.0975E-21	RHBDL2	2.19742298	0.02403004
HMBS	-2.28571602	7.0392E-06	FLNB	2.19736094	9.0777E-15
GIMAP7	-2.28262952	0.00014455	MAPK11	2.19632818	5.354E-05
EMP1	-2.28021253	0.02752721	PIK3CA	2.19206425	5.9294E-19
CD109	-2.27972374	2.4746E-10	H1-10-AS1	2.18501313	0.04975869
ZNF469	-2.27786176	0.00030979	SESN3	2.18077566	4.2316E-19
BIN1	-2.2744185	2.7231E-11	SMC5-DT	2.17654868	3.0512E-06
LOXL3	-2.27384712	0.00084546	CASTOR1	2.17174733	5.9969E-08
ITPRIP	-2.27246434	1.0849E-06	SNX33	2.16543062	0.0224318
LIMD2	-2.26597939	4.9424E-21	ZSCAN2	2.1589328	0.00011731
NOD2	-2.25925882	0.00021204	ENSG0000028	2.15874122	2.6268E-07
HLA-DMB	-2.25872341	5.7694E-55	BIRC2	2.14983642	2.0217E-19
PILRA	-2.25801857	1.9596E-15	ANK3	2.14839024	2.8039E-30
TMEM154	-2.25706473	6.8929E-05	LZTFL1	2.14772021	0.00030642
RASGRP4	-2.25541285	0.00071271	RYK	2.14762669	6.5505E-06
TK2	-2.25516717	0.00021994	IL7R	2.14714109	0.0003758
B3GNTL1	-2.25445431	1.2515E-06	HILPDA	2.14680753	1.5598E-05
LYZ	-2.25141697	4.1131E-21	SLC12A3	2.14579801	1.7982E-05
TAGAP-AS1	-2.24854173	0.00210422	SYNGAP1-AS1	2.14003729	0.02761145
ST18	-2.24800171	2.3913E-11	CEP120	2.13780686	3.4812E-06
CCNH	-2.247284	2.0677E-31	CLEC16A	2.1371654	6.7832E-17
PLAAT4	-2.2415228	2.295E-16	PIWIL4	2.13686886	6.673E-06
IFI16	-2.24149187	6.799E-12	CERS4	2.12437938	1.9116E-08
MILR1	-2.23898344	4.0964E-06	ABLIM1	2.12114659	2.2706E-05
PTPN22	-2.23777439	8.7702E-07	C11orf80	2.11598719	0.01295131
SLA	-2.23030191	5.7786E-09	AIFM2	2.10847022	0.00116409
SNX10	-2.2299848	4.6785E-20	ZBTB10	2.10158188	2.1924E-12
KIAA0930	-2.2292643	6.0005E-11	TBCEL	2.09684869	9.5284E-13
WDR46	-2.22610837	2.2135E-06	PANK1	2.0955903	0.00706418
SNAI3	-2.22369091	0.00514353	NAPB	2.09379391	0.00011494
RGS19	-2.22067264	2.0761E-33	ZNF280C	2.09313644	4.6022E-05
MX1	-2.21961817	1.075E-11	ENSG0000025	2.09241834	0.03896936
MSRB2	-2.21739611	1.2112E-10	MYB	2.08980613	0.00032556
SESN2	-2.21616723	3.4003E-12	NETO2	2.08755409	1.4515E-24
CCL4L2	-2.21456896	1.2731E-05	PRDM2	2.08611667	2.1727E-25
LRP1	-2.21165146	0.01521188	TIAM1	2.08446046	8.2286E-10
WARS1	-2.21006137	2.5687E-22	TRIM62	2.08385219	0.00705524
ECHDC1	-2.20979948	4.8767E-20	TRIM35	2.08304404	0.00163756

FAM151B-DT	-2.20682423	2.5972E-08	TOGARAM1	2.07384705	1.6553E-09
ACP3	-2.20462547	0.00034473	RALGPS1	2.07149181	0.04120522
CD2	-2.20432604	0.00318614	CD84	2.07147013	2.934E-19
MKNK1	-2.19838468	3.1569E-08	NRBP2	2.06587736	0.02754656
SYTL1	-2.19509402	0.01012779	ENSG0000027	2.06204234	0.00110031
AGTRAP	-2.19245394	4.4145E-07	RABGAP1L	2.05507149	9.7589E-53
FES	-2.19229795	1.2232E-13	CCDC191	2.05257033	0.00182544
HK2	-2.18526824	2.0491E-08	EBI3	2.05166002	8.1041E-10
MX2	-2.17967969	1.5269E-12	IGSF3	2.05151882	0.01353393
LIPA	-2.17896546	7.0535E-13	PALM2AKAP2	2.05107351	6.501E-15
FHAD1	-2.17809553	0.00113883	PECR	2.04935847	0.03310086
RNF125	-2.17642998	9.8556E-05	TMEM231	2.04822015	4.061E-05
NPM3	-2.17050224	8.9246E-05	GP1BA	2.04751361	0.04233622
AMPD2	-2.17009559	1.6842E-15	TSPAN2	2.04250073	0.02916306
FRAT1	-2.16997929	2.3172E-13	NEK6	2.03776288	6.2211E-12
MGAT4A	-2.16950745	0.00010806	ENSG0000027	2.03648227	0.00055871
MTHFD1	-2.16806146	7.1177E-08	PDGFRA	2.02357773	0.00070916
HCST	-2.16718243	9.4471E-25	PIK3R3	2.02300102	2.7584E-05
SERPINA1	-2.16629603	6.3741E-18	ENSG0000026	2.02156595	0.04069119
ENHO	-2.16519796	0.00875089	DANT2	2.02051706	9.1724E-05
GZF1	-2.16330153	1.3313E-12	TBL1X	2.01583431	6.747E-12
ITGAM	-2.154226	0.01574448	ALS2CL	2.0114637	0.01330943
SATB1-AS1	-2.15378412	2.2695E-06	FOXO1	2.00860375	1.9526E-11
LYL1	-2.15074738	2.6286E-06	S100A13	2.00573586	6.9396E-05
CIB1	-2.14430738	4.8873E-37	SPINDOC	2.00381025	1.6932E-13
AKR1B1	-2.13651737	2.2274E-20	CTC1	2.00046155	1.8771E-08
HEXA	-2.13610518	1.8467E-15	MYEF2	1.99901344	6.0768E-06
FKBP15	-2.13261829	1.6212E-15	TRIM16	1.99824629	0.02144476
SLC31A1	-2.12745658	6.8113E-10	BTG1-DT	1.99539009	3.9909E-07
LPCAT2	-2.11865712	1.1708E-17	MARCKSL1	1.99318281	4.9383E-11
FHL3	-2.11198351	4.7318E-09	ENSG0000028	1.99110459	9.2456E-05
RTN1	-2.10596877	2.1548E-10	MIR155HG	1.9890754	0.00024386
PTPN7	-2.10529667	1.0994E-05	NPIPA1	1.98817461	0.00053519
DTD2	-2.10407879	9.0392E-05	CASZ1	1.98405138	3.9778E-12
APOL3	-2.10402563	1.5229E-12	TRAF1	1.97861479	9.6731E-12
ARPIN	-2.10077977	1.4314E-10	SEC61A2	1.97635108	0.0017519
ELAVL4	-2.09741149	0.00808772	ARHGAP22	1.97595001	1.6842E-15
MIR99AHG	-2.09633704	0.00869542	ARID1B	1.97570606	4.6338E-23
RIPOR2	-2.09258815	2.7942E-08	RASSF4	1.97165749	6.7443E-19
CCL3L3	-2.09010001	0.00012637	TBC1D13	1.9688372	6.1926E-07
IFI6	-2.08890513	5.1068E-05	SPECC1	1.96877507	2.1173E-29
CHRNE	-2.08714954	5.1573E-07	CDK18	1.96729074	0.02516651
TMEM156	-2.08626777	0.00474326	LAMP3	1.96677701	5.4344E-11
SRPRB	-2.08529603	3.2668E-06	CLPTM1	1.96634684	6.0665E-29
C17orf107	-2.08292575	1.3644E-05	BACH2	1.96202132	5.1425E-18
RTP4	-2.08269758	0.03402161	BAIAP2L1	1.96059642	0.00286615

RAB33A	-2.07862826	0.00420279
TLR1	-2.0749549	4.2635E-08
LINC00963	-2.0740774	1.9215E-07
PLSCR1	-2.0698725	2.1602E-34
ABRACL	-2.06905985	9.4473E-11
MYO1D	-2.06888114	5.3756E-08
ZNF791	-2.06058362	2.1762E-05
PRMT9	-2.05609293	0.00135321
LONRF3	-2.0534199	0.00833943
LILRA6	-2.0526309	0.00010948
OASL	-2.05010294	2.3326E-06
CEBPD	-2.04796028	9.5022E-20
CD209	-2.04752225	0.00132333
PSTPIP2	-2.04634993	5.0474E-12
TRMT6	-2.04173083	7.0313E-12
IFI44	-2.03402876	0.01743627
RARA-AS1	-2.03277721	0.0140149
GNAO1	-2.02885515	0.00520385
CCDC86	-2.02668053	0.00537922
NAPSB	-2.02442786	1.5958E-23
MRPS24	-2.02291044	1.023E-13
CD68	-2.01316793	1.3903E-20
PTPN18	-2.01298065	1.977E-23
HM13	-2.01280348	4.2169E-15
CDK5	-2.00977127	0.00334685
S100B	-2.00933163	0.00134791
SLC43A3	-2.00724516	5.2314E-06
SP110	-2.00660217	8.8943E-26
LRG1	-2.00499979	0.00089184
SH3BP4	-2.00146048	2.033E-09
DISC1	-1.99826775	5.5152E-06
ENSG0000028	-1.99286675	0.00365131
TOMM40L	-1.99123361	0.00927667
TRIM25	-1.98574834	4.5852E-07
LIPN	-1.9844643	0.0063988
EEIG2	-1.98050574	6.1218E-08
NAGK	-1.9801491	1.6502E-16
SP140	-1.97926024	2.0428E-09
HHEX	-1.97654968	0.00361067
ALDH3B1	-1.97525077	0.00028957
MT2A	-1.97516149	1.7358E-21
PLEKHA7	-1.96993349	0.00023848
NUCB2	-1.96867413	5.2696E-06
ETV7	-1.96441317	1.9917E-05
RFLNB	-1.96296724	0.00394304
CD36	-1.96221464	9.4375E-09
ID1	1.95892081	0.02368429
NUAK2	1.95811035	2.6804E-27
MAP3K14	1.95798482	3.6219E-23
TSPAN33	1.95739056	2.1323E-12
CLIP2	1.94626967	6.222E-17
TCF7L1	1.9449284	0.0011286
SPG11	1.94487217	4.0798E-18
RASL11A	1.9441813	0.00015787
ZNF398	1.94353732	0.00014226
PHLDB2	1.94005212	0.00964365
VAMP1	1.93952682	0.041075
ICAM4	1.93708524	0.00026901
RNF32-DT	1.93601766	0.01213147
ENSG0000029	1.93489823	1.2652E-05
ADAMDEC1	1.93456622	0.00072742
ATG16L1	1.93337069	2.7675E-07
PPP1R16B	1.93201423	2.3595E-14
KIF21B	1.93062377	0.0001097
HAUS5	1.92759636	3.6244E-06
MARCKS	1.92469619	3.535E-14
SWAP70	1.92341878	1.0835E-22
TMTC2	1.92150686	3.2691E-10
ENSG0000023	1.92058455	1.7424E-06
LINC00540	1.91899171	5.9463E-05
NIPA1	1.9177314	0.00013914
TNFRSF25	1.91754021	0.02842334
DCLK2	1.91658781	0.00028008
TIAM2	1.91658644	1.7033E-06
PKD2L2-DT	1.91594952	0.01040307
PAQR7	1.91422454	0.00434232
DTNB	1.91278422	5.7193E-06
COQ8A	1.91177555	9.7587E-07
HABP4	1.9109065	0.00314041
GTDC1	1.90914485	4.6672E-25
RORA	1.9048707	0.01153444
PRRT3	1.9019104	0.03647985
CYB5A	1.90089111	1.5624E-13
ARAP2	1.89961828	6.1938E-21
ZNG1C	1.89660936	7.0467E-05
ABCA5	1.89599893	4.5508E-06
CLEC4G	1.89444943	0.00253894
AKT3	1.89179806	1.5731E-16
AGBL4	1.89145685	0.03093819
FAM174B	1.89059346	1.3644E-05
ATXN7L1	1.88665737	3.535E-05
TMEM268	1.88570914	1.4714E-16

IFI35	-1.95927941	1.6717E-17	STMN1	1.88556397	1.031E-06
LINC03002	-1.95851426	1.9822E-05	MFHAS1	1.87736869	2.2447E-05
EIF2AK2	-1.9582476	2.2053E-09	ENSG0000027	1.86535126	0.02563482
SDF2L1	-1.95661863	1.2633E-21	ENSG0000024	1.86338958	0.01046068
ENOSF1	-1.95625999	9.439E-09	MTERF2	1.86038011	0.03290585
IFI44L	-1.94993841	0.00618342	NIBAN1	1.85485725	8.1173E-12
EEF1E1	-1.94983968	3.4962E-09	RAP2A	1.85141605	3.0091E-33
TMEM163	-1.94895166	1.9031E-09	IPO9-AS1	1.85119675	1.8722E-05
FGD3	-1.94597318	1.0014E-05	CEP350	1.85027072	7.7141E-22
S100A9	-1.94274093	0.00431228	STAG3	1.84868607	0.01173037
P2RY14	-1.94234866	1.4928E-06	UBE2Q2P1	1.84673692	0.02546223
MNDA	-1.94007254	1.5708E-12	IQCG	1.84309195	1.1905E-09
ENSG0000028	-1.9395238	1.5435E-05	DHCR7	1.84229665	3.1961E-05
MFSD1	-1.9392305	8.5051E-23	CAMK1G	1.83860985	0.00048667
SLC12A7	-1.93706066	1.5153E-18	LRCH3	1.83797681	2.7713E-14
LGALS3BP	-1.93597075	3.0274E-07	HMGCS1	1.83251586	2.9123E-16
LINC01128	-1.93506619	1.2268E-08	SLC2A5	1.83216655	0.00020184
DCBLD1	-1.93438269	7.689E-06	TBC1D8	1.83190762	6.0005E-31
C11orf96	-1.93437443	0.00055854	RAB8B	1.83112812	9.5885E-22
ISG15	-1.93167939	6.6729E-11	ENSG0000028	1.82079101	0.00380635
IL18	-1.92877302	9.019E-09	FOXP1	1.82078785	8.3855E-55
MAPKAPK3	-1.92761735	6.1206E-11	KHDRBS3	1.81979255	7.3139E-08
SH2B2	-1.92589797	8.2468E-15	UBL7-DT	1.81716504	0.01592775
LYSET	-1.92509503	0.00044727	DGKH	1.81500312	4.9136E-08
CRYL1	-1.9240884	2.7584E-05	MPZL1	1.8141225	5.907E-16
ERCC1	-1.92247515	1.0147E-20	GPR157	1.81395516	5.0721E-08
ADORA2B	-1.92126906	0.00538485	LINC01619	1.81212285	1.4487E-08
OAS2	-1.92116062	7.9072E-11	TNFRSF8	1.81182412	0.00355267
PXK	-1.92070752	4.8778E-08	SOCS2	1.81109279	1.8237E-18
TOM1	-1.9106135	2.718E-18	ENSG0000027	1.80470516	0.00210695
CH25H	-1.90677097	1.8431E-06	NOL4L	1.80284856	1.9634E-07
ENSG0000029	-1.90444203	9.1242E-16	LYST	1.79883092	9.762E-33
ZNF594-DT	-1.89784713	0.00490619	CRIM1	1.79879609	0.02425174
BIN2	-1.89735256	1.6482E-06	IMMP2L	1.79787035	2.3881E-14
MEF2C	-1.89607917	1.0034E-06	TMEM185A	1.79614037	1.5278E-07
LIMK2	-1.89557302	7.8595E-14	IQCN	1.79347403	0.01298287
SH3BGRL3	-1.88940662	5.3918E-40	DSG2	1.78972458	1.5329E-10
IDH2	-1.88819312	1.0769E-23	GOLGA8B	1.78712116	6.1175E-05
PPP1R14A	-1.88649346	9.8411E-09	NUB1	1.77882299	2.0783E-21
TNFRSF1A	-1.88639872	9.7645E-30	EPM2A-DT	1.77581001	0.01774115
SGK1	-1.88594207	2.0661E-09	MAP3K1	1.77312584	2.1656E-23
MOCS2	-1.88427931	8.4552E-05	CYTH1	1.7721844	2.6451E-17
MYO1F	-1.88008304	2.6207E-24	IL21R	1.77177569	1.1421E-08
PNP	-1.88005685	5.4852E-10	LINC01410	1.77165669	0.00071835
HCLS1	-1.878044	1.9871E-22	ACTR3C	1.77127531	3.7005E-06
GPX7	-1.8776986	0.00091383	BCL9L	1.77100164	1.6414E-06

PPIL1	-1.87652238	0.00199633	TMEM164	1.76862975	2.951E-10
ENSG0000029	-1.87494448	7.624E-09	SPRED2	1.76712993	7.7751E-15
IL27RA	-1.87476608	5.3549E-08	MYO1C	1.76266109	1.0109E-08
AFF3	-1.87418239	0.00071534	FLT3	1.76230308	6.269E-17
SYPL1	-1.87351638	3.519E-09	WHRN	1.76186664	0.0003128
DOCK5	-1.87065818	6.013E-18	AGFG2	1.76086503	0.01212664
SAMD9	-1.86997003	3.1585E-07	LINC02605	1.76079178	0.00038853
PPIF	-1.86864357	1.9674E-18	MRTFA	1.75963359	8.852E-29
TPMT	-1.86687686	1.4996E-12	TMCC3	1.75793901	1.3011E-09
ISOC2	-1.86496847	2.1397E-11	TMEM131L	1.75571697	1.9703E-16
ETV5	-1.86440107	0.00476764	RANBP10	1.75493291	0.00080727
CLN8-AS1	-1.8631481	4.4099E-08	ENSG0000029	1.75072187	0.01044377
GAS2L1	-1.86232307	0.00014909	HIVEP1	1.74909191	9.116E-23
MMP14	-1.85990732	2.1027E-05	SIAH2	1.74802052	3.6617E-17
MOB3C	-1.85828923	0.00730527	PNMA1	1.74760922	7.9917E-08
CARD8-AS1	-1.85637875	0.00031129	ENSG0000025	1.74703432	0.04903206
SLAMF9	-1.85479544	0.00099813	TRAFD1	1.74662665	4.345E-19
APOL6	-1.85264696	6.2211E-12	ENSG0000028	1.74304651	1.1738E-05
SUCNR1	-1.84607261	0.01590903	KIF3A	1.74151961	0.00416939
PPM1F	-1.84476483	0.00030158	PNKD	1.73971588	8.3947E-23
SPG21	-1.83791306	3.1615E-16	RPS6KA2	1.73901926	9.498E-06
IFI27L2	-1.83734185	1.1003E-16	RAB13	1.73477858	1.9759E-08
FAM20C	-1.8348101	1.799E-09	POU2F1	1.72880496	6.7733E-10
CCND3	-1.83424512	3.0456E-20	ZER1	1.72734263	5.4179E-09
MTMR14	-1.8286044	1.0922E-08	SUPT3H	1.7269626	8.7913E-12
NFATC2	-1.82308398	1.0131E-07	PELI2	1.72503376	1.5265E-14
NME1	-1.82305305	8.8652E-08	AMMECR1	1.72367629	0.04481215
HELZ2	-1.82212945	8.4552E-05	NEK4	1.72009892	6.9153E-05
CCDC107	-1.81992729	5.2693E-13	GADD45A	1.71650243	4.4929E-22
TESPA1	-1.8185294	0.00753053	NID1	1.7160775	3.6515E-05
MLEC	-1.81823363	2.2361E-14	STPG4	1.71512638	0.00202361
SSR1	-1.81756916	6.4602E-19	HDAC2-AS2	1.71361861	3.54E-05
BAG1	-1.81662104	2.1946E-10	PCMTD2	1.70603764	9.4566E-08
CLN8	-1.81649658	1.7197E-18	SYNGAP1	1.70553924	0.00019969
NAAA	-1.80863406	3.2111E-15	ENSG0000022	1.7041	0.01503816
ENSG0000028	-1.80415785	0.01934288	SPATA2	1.70211466	0.00652186
FXYD5	-1.80398255	1.9285E-20	USP12	1.70027198	6.1192E-15
ENSG0000025	-1.80180631	1.2142E-05	ZBTB16	1.6991897	8.8422E-09
CAMK2D	-1.79860933	2.5619E-09	COA1	1.69776842	9.4055E-21
RAD51C	-1.79540443	1.6285E-06	ATL2	1.69628576	8.1255E-06
NIPAL2	-1.79328982	0.02304268	ARHGEF7	1.69301538	1.0107E-16
CCL3	-1.79241843	8.115E-06	SLC6A12	1.69058629	5.3657E-09
ATP13A3-DT	-1.79143366	0.00183867	EHBP1	1.68619354	0.00093584
C15orf61	-1.79029136	0.00009077	AHCYL2	1.68321216	6.5285E-17
SEMA4D	-1.79004199	7.4513E-07	IZUMO4	1.68213059	3.3851E-05
CARD8	-1.78856886	0.00095149	RAB9A	1.68195778	6.0467E-28

CD226	-1.78425121	7.7591E-09	SLC38A4-AS1	1.67971416	9.1924E-08
COMMD8	-1.7825622	3.6033E-14	CCNG2	1.67963201	3.96E-12
EML4	-1.78039369	1.6906E-11	GPC4	1.67864736	0.0008436
USP6NL	-1.77980781	1.9409E-13	TFCP2	1.67246069	1.99E-23
PSD4	-1.77891073	0.01422442	POLD1	1.66970447	3.9332E-06
ICAM2	-1.77826059	0.00030896	ATP6V0A2	1.66564359	1.4843E-22
PPP1R3B	-1.77822389	1.2828E-06	TNKS1BP1	1.66373729	0.00730272
CEP250-AS1	-1.77676335	0.00267715	ZC3H7B	1.66185355	4.0161E-10
POLE4	-1.77609268	8.3659E-16	DNAI3	1.66120399	0.00226348
ATP6V1B2	-1.77446188	1.0962E-27	VOPP1	1.6580976	3.1256E-33
CHPT1	-1.77122923	4.5376E-08	TCF4	1.65760859	6.2512E-16
POLD2	-1.76944999	1.3994E-06	BMERB1	1.65684738	8.1255E-06
GABARAPL1	-1.76848204	4.0383E-12	FAM168A	1.65223425	1.6652E-08
ANKRD9	-1.76709563	8.5436E-05	NOL3	1.650171	0.01996213
OSGEP	-1.76528076	1.7574E-11	ZBTB38	1.65005329	1.6987E-13
ARPC3	-1.76470782	1.1996E-50	PM20D2	1.64998942	0.00278929
PLCD1	-1.76357352	0.0007036	CCHCR1	1.64167903	0.03916747
CTSL	-1.76290718	2.4367E-07	TUBA1C	1.64071842	1.6161E-32
ALG3	-1.76269363	1.0431E-07	MTURN	1.64040249	0.00022383
CTSB	-1.75247008	5.8477E-14	LINC03004	1.64033923	0.04616639
IRF2BPL	-1.75208017	0.00055214	EHF	1.6392639	1.2474E-05
PTPRC	-1.75009313	4.4599E-33	NPIPB13	1.63902619	4.2956E-08
FRMD4B	-1.74232552	2.9632E-10	FNBP1	1.63862986	1.1256E-20
UTRN	-1.73719701	1.23E-15	THAP3	1.63857741	7.447E-08
KLF2	-1.73493226	9.2783E-08	MELTF	1.6367013	0.00133857
MAFB	-1.73138857	5.0513E-14	GMDS-DT	1.63613601	5.8456E-11
TNFSF12	-1.73076998	3.6741E-08	ZC3H12C	1.63532056	0.00679183
DCPS	-1.72835089	6.4352E-07	CLCC1	1.63369847	6.9317E-08
RASAL3	-1.72824146	8.186E-05	ENSG0000029	1.63368163	0.00236564
TASL	-1.72200748	3.4933E-06	ENSG0000029	1.63131554	4.4539E-06
PLOD1	-1.72131898	0.00017529	GLS	1.63047505	8.5086E-15
PPT1	-1.72104042	4.4976E-16	TIGD2	1.63046422	0.03908592
TTC38	-1.71711105	0.00107222	RAB39B	1.63020373	0.01338391
RCBTB2	-1.71626033	9.8917E-06	RAD51B	1.62804388	7.4568E-07
UCP2	-1.71593936	1.4916E-15	PBX3	1.62795155	6.5374E-10
PDXK	-1.71551856	6.0573E-16	HES6	1.62414829	0.00244228
ECHDC3	-1.7145401	9.0276E-05	RAPGEF4	1.62413501	0.00549665
MBP	-1.7121535	5.9519E-12	LIN52	1.62396898	0.00076044
ENSG0000027	-1.71166262	0.0276962	PEX14	1.62276253	4.4963E-10
ENSG0000020	-1.70958321	0.00686876	CLEC2D	1.62130407	0.00014922
C15orf48	-1.70854888	5.519E-13	NECTIN1	1.62044231	0.01318426
CCL4	-1.70713459	0.02714072	GRAMD1A	1.61628631	1.4303E-12
FKBP1A	-1.70410124	2.0014E-19	ENSG0000029	1.61504171	0.00730751
GALK1	-1.70394591	5.4508E-06	PHF21A	1.6144894	4.6034E-13
SELENOH	-1.70379353	1.5883E-32	TRAF6	1.61365447	8.3094E-10
ENSG0000029	-1.70261953	0.00542604	LYRM9	1.60956113	0.02665986

CAPN2	-1.70256315	7.6527E-10	ENSG0000028	1.60885147	1.6163E-07
CDC42EP2	-1.70100991	3.8001E-12	TXN	1.6079414	1.2324E-06
SIPA1	-1.6987654	2.4917E-10	LRRC61	1.60734724	8.6837E-05
IKBKE	-1.69862341	0.00314727	MVB12B	1.60504806	2.6105E-10
ARL11	-1.69426297	0.0117283	LIG1	1.60429255	0.02413671
ATP6AP2	-1.69319131	5.5874E-15	ATXN1	1.60240476	2.7942E-08
SCML1	-1.69226195	1.1724E-07	RAB5B	1.60021674	5.017E-17
ZFYVE16	-1.69177278	1.3364E-19	ENSG0000025	1.60008542	0.01514194
HLA-DMA	-1.69022464	1.036E-21	FRS2	1.59875062	8.9015E-12
ABTB3	-1.68829053	0.00023503	WEE1	1.59819938	0.00278716
ARPC1B	-1.68684365	5.4194E-55	SEC22A	1.59543352	0.00042591
DYRK4	-1.68530679	0.00033882	GTPBP1	1.5952113	1.9326E-18
IDH3A	-1.68427175	2.2219E-09	ENSG0000028	1.59183204	4.4614E-06
TIMP2	-1.68297331	5.4648E-09	TUBB2A	1.58620828	1.2234E-06
PHC2	-1.68271718	1.1226E-09	JPT2	1.58583525	5.8188E-06
NAB2	-1.68256565	0.00061377	FNBP1L	1.58525795	0.00145125
ZNF385A	-1.68221378	1.6681E-07	RFTN1	1.58484549	3.0186E-14
RRS1	-1.68149394	0.04032559	MAGI3	1.58387253	0.02401213
PSTPIP1	-1.6713168	0.00025174	DAPP1	1.5806386	3.8053E-14
LSM10	-1.6697298	5.862E-17	SEMA7A	1.58035236	2.9762E-18
CSGALNACT2	-1.66655403	6.6788E-12	CSRP2	1.57908115	0.0121089
FTCDNL1	-1.6648097	0.00357567	ARID2	1.5790174	2.7416E-13
UBXN11	-1.6593338	0.0167368	ST3GAL3	1.57762238	0.00502387
PDLIM7	-1.65773394	0.0020631	MAPK8	1.57610238	5.9669E-18
NDUFAF2	-1.65731573	3.3274E-06	IDO2	1.57564434	2.7034E-09
HDHD5	-1.65670929	1.5441E-05	ABCA7	1.57307362	8.2563E-05
COMMD9	-1.65242397	1.3644E-15	ECE1	1.57172977	8.029E-05
CXCL2	-1.6524177	5.385E-06	ZFP36L1	1.57028736	8.997E-10
TESC	-1.65157891	0.00789193	ECT2	1.56952331	0.0384711
LILRB4	-1.65157037	3.7297E-16	PTPRN2	1.56441563	4.925E-06
ERCC8	-1.65062445	3.3813E-05	SPECC1L	1.56423556	1.5244E-10
GLUL	-1.65009056	2.6264E-09	ENSG0000022	1.56245854	0.00762593
UBE2L6	-1.64779676	3.5198E-35	HOMER2	1.56171363	5.437E-09
LMO2	-1.64623315	3.9724E-07	INTS3	1.55900977	3.0517E-15
CCNJ	-1.64611389	0.01883309	RUBCN	1.55852269	1.1902E-20
ASCL2	-1.64608494	1.2718E-05	DLEU2	1.55712119	3.0053E-12
PSAP	-1.6454437	1.8449E-45	SFMBT1	1.55701234	6.9153E-05
FXN	-1.64499282	0.00802678	RAB11FIP3	1.55452151	3.7381E-05
RBKS	-1.64222117	2.6392E-05	SINHCAF	1.55218543	5.0424E-21
FOSL1	-1.63882267	1.7516E-05	DPP4	1.54952514	9.9186E-05
EXOC6	-1.63824112	4.9842E-06	ZNF296	1.54707689	2.0554E-09
AGPAT1	-1.63728603	2.3166E-07	LINC00342	1.54593425	2.4591E-06
NQO2	-1.63713494	9.5008E-09	MTMR4	1.54305346	1.7576E-08
SRC	-1.63366221	4.7692E-08	VAV2	1.5422314	3.5863E-10
INPP5A	-1.63090006	2.194E-06	AK9	1.54102557	0.04368971
SIRPB1	-1.63045036	0.00203596	NABP1	1.54095127	5.1631E-20

CD4	-1.62846062	1.9594E-21	VPS13A	1.54050545	7.2777E-09
GGT5	-1.62785425	2.2882E-05	ENSG0000028	1.53884692	0.01423826
NANS	-1.62509565	3.5218E-19	BCAS3	1.53712537	3.6848E-10
METRN	-1.61578739	0.01747268	ANKRD10	1.53400198	4.2629E-22
TMEM109	-1.61244486	0.0002229	WDR44	1.5333353	4.8429E-07
RIN3	-1.61101052	1.7458E-17	GRB10	1.53016369	0.00095334
DIAPH1	-1.61085046	9.0923E-10	AGK	1.52943715	5.2137E-07
TRIM22	-1.61020985	1.8826E-18	RGS13	1.52926745	1.6246E-07
STAMBPL1	-1.60955629	7.1036E-07	ZNF438	1.52734213	0.00011768
MAP2K3	-1.60537234	1.3525E-17	LINC00173	1.52720831	0.00783447
BTK	-1.60528991	3.2806E-08	BTBD3	1.52549289	1.5021E-05
SEPTIN9	-1.60525647	3.187E-16	TET3	1.52336626	1.9431E-13
SPINT2	-1.60276121	2.466E-18	PBX4	1.52330342	0.0399766
SMIM3	-1.60189058	1.0335E-07	PRMT3	1.51848541	0.00032478
S1PR4	-1.60089511	8.4517E-05	ARID3B	1.51824953	0.00013335
SLC25A37	-1.59996495	2.241E-05	DHRS13	1.51499505	0.00446621
C1QBP	-1.59905805	1.0386E-18	SH2D3A	1.51475231	0.00134238
LPAR6	-1.59493132	0.00145096	TMC8	1.51241125	2.3172E-05
GNPDA1	-1.59271949	1.6752E-07	CPNE2	1.51031478	0.00847109
DECR1	-1.59265366	1.568E-21	GPR107	1.51013963	8.1785E-13
ETFB	-1.59058179	9.0534E-19	LDAF1	1.51009533	2.7995E-06
FRAT2	-1.58780028	3.4246E-12	SLC15A4	1.50988145	5.519E-13
KRT10	-1.58623441	1.4985E-11	FADS2	1.50967237	2.0775E-07
KCNMB1	-1.5859919	2.5741E-05	POMT1	1.50921056	0.03071162
PA2G4	-1.58041756	5.4996E-14	IGSF8	1.50850586	9.0666E-06
SRM	-1.57972977	9.0457E-09	RNF215	1.50834865	0.00549665
CTNND1	-1.57830961	5.7737E-08	UVRAG	1.50731154	1.5085E-15
SCRN1	-1.57777562	1.5435E-05	PELI1	1.50719846	5.827E-19
EMP3	-1.57711713	1.2696E-15	TMEM176A	1.50578116	4.936E-07
CASS4	-1.57423885	2.377E-05	UHRF2	1.50533286	6.6472E-09
ACAA1	-1.57354869	1.758E-30	KCNQ1OT1	1.50487092	3.7438E-07
WDFY3	-1.57293922	9.0886E-05	MREG	1.50459645	7.2175E-05
ZSWIM7	-1.57132657	5.4344E-11	RASSF2	1.5032015	1.8561E-14
SASH3	-1.56878068	7.0669E-08	CDC14A	1.49953914	1.7202E-14
RBM38	-1.56744898	0.00012215	TNFAIP6	1.49807954	0.00136078
TBC1D16	-1.56570067	0.00791214	DENND4A	1.49750155	1.62E-09
TRMT1	-1.56274665	1.9846E-08	ST7	1.49602848	2.4376E-05
RRP12	-1.56273596	2.4123E-05	RRAGB	1.49473089	0.00066743
TRIM14	-1.56258002	1.4104E-07	JMY	1.48991546	4.6218E-07
ASAH1	-1.56070914	2.7667E-12	MOSPD2	1.48730764	7.3662E-08
CISH	-1.55995094	0.01019841	MAP3K6	1.4870258	3.1665E-05
FUCA1	-1.55941251	0.00045508	TYW1B	1.48632646	8.6837E-05
TMEM144	-1.55921681	0.03438275	LRRK1	1.48616768	1.3903E-15
HDDC2	-1.55779997	0.00181251	DAPK1	1.48399606	1.3805E-10
SNCA	-1.55703755	0.00109102	ELOVL7	1.48117014	3.5546E-07
AKR1A1	-1.55631171	5.5872E-27	KLHDC7B-DT	1.47901697	3.3648E-05

UNC93B1	-1.55600976	1.3639E-08	CLIC2	1.47868863	4.823E-23
PRKACB	-1.55477595	5.318E-05	PBXIP1	1.47861414	5.306E-07
VAMP8	-1.5547171	1.0063E-30	BRPF3	1.47792138	0.00059236
NOP10	-1.55367254	4.4293E-19	GALNT2	1.47763065	3.0791E-09
DENND1C	-1.54999999	0.00030873	MYOSLID	1.47556714	0.02095766
IFT27	-1.547996	0.00017853	SCMH1	1.47472223	8.2141E-05
FMN1	-1.54521084	4.2459E-06	CD1B	1.4745712	0.04856285
HPS3	-1.54505317	3.3019E-13	FGD6	1.47315046	2.0095E-06
ENTPD1	-1.53860133	5.1168E-06	CDC42EP4	1.46940402	9.4967E-06
CLPB	-1.53843977	0.00032741	OSBPL3	1.46913955	1.3771E-09
GM2A	-1.5380455	1.8867E-13	LPCAT1	1.46771727	2.2228E-10
LACTB2	-1.53703547	0.00462443	ZMAT3	1.46738364	8.1521E-09
MBOAT7	-1.53675454	2.5874E-12	PPM1K	1.46713837	2.2219E-21
AP3D1	-1.53608047	1.0257E-13	PLXND1	1.4667406	7.3228E-07
STX3	-1.53588142	4.9962E-06	PDPK1	1.46621992	2.4679E-11
EIF4EBP3	-1.53474501	0.00456173	ENSG0000022	1.46477009	7.3495E-08
CYB5R1	-1.53471767	0.00039669	DIPK1A	1.46457068	6.0015E-08
ATP6V0A1	-1.53318092	0.00042228	DNAJC5B	1.45994051	2.6162E-05
ENSG0000028	-1.53137879	0.0006139	PLXNC1	1.4570967	4.5178E-08
ATP6V1F	-1.53082354	2.5436E-16	RAPGEF6	1.45703636	1.3715E-13
FAM78A	-1.53064373	0.01503816	CEP135	1.45626031	1.1305E-17
INPP1	-1.52941059	0.00025246	CDYL	1.45323572	8.605E-18
SUSD6	-1.5279159	3.3776E-12	BCL2L14	1.45231208	7.5962E-09
OXCT2	-1.52554936	0.01047156	MSMO1	1.45149538	6.4072E-07
RILPL2	-1.52553963	1.2739E-18	SREBF2-AS1	1.45000283	0.02594027
PPP1R16A	-1.52307532	0.00038708	CASP2	1.44976236	0.01852092
RASAL1	-1.52101271	0.03697007	ANKLE2	1.44616664	5.1732E-17
TBC1D22A-DT	-1.52099455	4.4871E-07	PKD2	1.44608194	0.00041698
NAGA	-1.51982362	6.6893E-16	KDM4C	1.44420632	1.8753E-19
CSGALNACT1	-1.51810573	0.0375314	AVEN	1.4420028	3.1781E-10
ZNF358	-1.51667344	0.01704327	RAB3IP	1.43888797	0.00014977
CHCHD7	-1.51651465	2.2572E-08	TSPOAP1-AS1	1.4382705	7.3291E-14
CHCHD4	-1.51517733	0.00426442	PHKB	1.4368692	4.9311E-16
CENPW	-1.51510135	0.00064522	LSR	1.43427348	0.00582075
ALOX5	-1.51331775	9.1621E-08	WAKMAR2	1.43257481	8.2327E-14
ATP6AP1	-1.5123725	2.2255E-14	IRF1-AS1	1.4294016	3.61E-07
HDDC3	-1.51048935	0.01467906	CD274	1.42879869	1.3345E-11
ANKUB1	-1.50883014	0.00010173	TTF2	1.42844184	4.2997E-09
OTUD1	-1.50831811	3.2152E-14	CENPS	1.42764355	0.00209085
GSTM4	-1.50461684	8.6934E-07	C4orf46	1.42181119	0.00032491
PON2	-1.50442405	4.5598E-11	MARF1	1.42088027	1.3443E-09
CXCL3	-1.50359601	6.754E-06	ZC2HC1A	1.41816163	0.03254895
ITPR2	-1.50320315	1.1747E-11	ENSG0000022	1.41496821	0.01122653
CC2D2B	-1.50115132	0.0189856	NFE2L1	1.41398424	8.2012E-09
SRXN1	-1.50009421	0.02912682	COMMD1	1.41373493	7.4519E-16
GON7	-1.49898078	0.00028804	DCAKD	1.41249072	9.7276E-05

IFT74	-1.49864171	0.00910461	ZFYVE19	1.40920703	4.2079E-05
SP140L	-1.49686821	1.4317E-08	ALOX15B	1.4085345	0.00503086
LSM7	-1.49677446	1.4208E-14	MAP4K4	1.40814346	2.7425E-07
PLP2	-1.49519093	2.1129E-10	ATP2B4	1.40769114	0.00029334
PLK3	-1.49486494	3.7037E-08	ANKRD36C	1.40609336	3.0694E-09
SLC1A5	-1.49410745	9.8114E-07	ANTXR2	1.40472281	7.0095E-11
SSBP4	-1.49312436	1.2836E-09	LRP12	1.39966569	0.00063941
POMP	-1.49302274	2.0702E-27	ISCU	1.39892811	1.9373E-11
TOP1MT	-1.49156512	0.03892869	ABCG1	1.39874084	0.00020334
SLFN12	-1.49105097	0.01592094	CDIN1	1.3978702	0.04975457
RHOQ	-1.4903639	1.0645E-15	NPIPB4	1.39715303	0.00016061
SMCO4	-1.48952443	5.5203E-21	NT5DC3	1.39713876	0.01137701
CASP10	-1.4882123	0.01632273	USP24	1.39376062	3.4778E-05
RXRA	-1.4877787	0.02566682	NCK2	1.3928392	1.1984E-08
PIK3R1	-1.48738606	7.4913E-11	PIP5K1B	1.39067908	0.00609907
MTLN	-1.48736411	3.4477E-07	PLAGL1	1.38776161	2.5158E-10
FARSB	-1.48709495	0.0037725	NUP107	1.38734278	1.0414E-06
LINC01010	-1.48550752	0.00519047	PIAS1	1.38673466	1.4713E-13
ATP5IF1	-1.48543471	1.9241E-13	CSNK1G3	1.38648058	2.6849E-08
PRPF19	-1.4829435	1.2154E-05	FAM53B	1.38646058	1.3391E-09
RBMS2	-1.48273826	0.02995394	RELB	1.38573955	6.4317E-18
COMMD4	-1.48246607	3.097E-06	MALSU1	1.38567156	7.5857E-07
TRANK1	-1.48161629	0.02702409	DOCK10	1.38486795	6.799E-12
GRWD1	-1.48142601	0.00683321	ALCAM	1.38404312	1.1356E-08
PRKCB	-1.47897818	4.385E-18	ATF7IP	1.38001902	3.951E-23
FTL	-1.47892246	2.7332E-10	CAMKMT	1.3778328	0.00204601
SHISA5	-1.47723151	7.2869E-10	ODF2L	1.3769663	0.0248462
MFSD3	-1.47632638	0.04055416	APC	1.3769272	3.684E-08
CYB5B	-1.47533362	4.9116E-08	ILRUN	1.37628867	1.9391E-17
TBC1D7	-1.47406673	1.0928E-05	CEP68	1.37589754	7.1821E-19
GFOD1	-1.47286594	0.00016997	GOLM2	1.3755062	1.4695E-14
CDCP1	-1.47182295	0.00013119	C3orf14	1.37453752	0.01522055
ITGB2-AS1	-1.4711851	1.5269E-06	LINC01473	1.37423976	0.00195514
PCTP	-1.46818962	0.012536	FBXO25	1.37022643	0.00421892
DNASE2	-1.46286378	3.3167E-06	EPM2A	1.36980464	0.00563923
NKG7	-1.46196701	0.00963494	SC5D	1.3688611	2.7736E-10
MRPL3	-1.4601773	2.5634E-08	STK35	1.36830678	0.00771853
TMEM147	-1.45930698	5.4648E-09	ANKRD6	1.36770956	0.02988394
RBFA	-1.45907317	9.037E-06	FER	1.36703049	8.1754E-09
CNIH4	-1.45843846	1.5986E-12	KDM5B	1.36550627	2.4341E-05
GSTO1	-1.45796416	3.0341E-15	ENPP4	1.3649955	4.5169E-07
HEXB	-1.45792843	1.5974E-14	APBB2	1.36337544	0.00658203
ATP13A3	-1.45569664	2.0188E-07	F11R	1.36311345	1.8406E-19
ARMCX1	-1.45471762	0.00865816	AMBRA1	1.36294758	3.5856E-12
CAVIN2	-1.45464217	0.01065928	ADCY9	1.36276719	0.0001137
MPP7	-1.45302379	0.00075271	WDSUB1	1.3625323	2.8809E-05

CISD1	-1.45266522	7.0748E-07	FSD1L	1.361954	4.5376E-08
NAPA	-1.45217974	6.3568E-25	BTN2A2	1.36138965	9.0923E-10
MACROH2A1	-1.45203949	5.4433E-35	LDLRAD3	1.3603453	1.5506E-10
PRDX4	-1.45080156	0.02718444	SQLE	1.36033623	1.1902E-20
IL10RB-DT	-1.44928872	0.00107659	HIVEP2-DT	1.35880541	0.04143452
CTSS	-1.44792469	7.7254E-42	PAN3	1.35865725	1.5928E-16
TMEM191A	-1.44776342	0.03637106	ZNF767P	1.35859656	2.0622E-05
HK1	-1.44614012	2.791E-09	FBXO30	1.35839423	1.6658E-05
RGS2	-1.44236429	2.5532E-34	ENSG0000027	1.35751511	0.04641605
DNASE1L3	-1.44170582	1.0112E-05	UBD	1.35503017	0.00058604
PIP4P2	-1.43989028	0.02597942	APOLD1	1.35492431	0.01285233
MPI	-1.43954459	0.00129879	CDKL5	1.35076593	0.02910032
LRRK2	-1.43873085	3.2249E-06	ENSG0000028	1.34985967	2.8074E-06
DCXR	-1.43773196	7.8692E-11	SOCS1	1.34957317	1.0661E-07
NCF1	-1.43764355	7.2175E-05	EPAS1	1.349481	3.7423E-05
ZNF577	-1.43520335	0.02181141	LANCL2	1.34748623	3.7861E-06
DCLRE1C	-1.43507076	0.00023644	CCDC127	1.34694994	0.0140149
GCA	-1.43416027	1.0108E-27	SRGAP1	1.34529554	4.6486E-06
ARFIP1	-1.43377491	4.7561E-05	ENSG0000028	1.34434989	0.03691005
RNF149	-1.43357054	2.6816E-28	USP20	1.34341148	7.8061E-05
PDK4	-1.43343597	0.00223096	MAGEF1	1.33850447	6.0142E-05
APOL4	-1.43315757	0.00015897	SH3KBP1	1.33799442	9.6453E-13
DELE1	-1.42978891	1.7419E-06	CDK6	1.33767501	1.8794E-13
DDX60L	-1.4292184	0.00127119	SESN1	1.33751771	7.7092E-08
IKBIP	-1.42676528	2.431E-11	PIKFYVE	1.33726388	1.0738E-07
TRIM5	-1.42053067	4.3086E-06	ZNG1E	1.33667327	2.2787E-12
MYG1	-1.41883862	7.1692E-05	ZDHHC21	1.33290267	0.02501009
LAMTOR2	-1.41858167	1.0038E-19	ITFG1	1.33196339	2.0538E-06
TCN2	-1.41731501	0.00048284	TRPM7	1.33147522	1.451E-11
ALG8	-1.41686217	0.00266438	MAP2K4	1.33140227	3.1131E-08
PRR5L	-1.41682528	0.03743263	SYNGR3	1.33031749	0.00935738
TTC19	-1.41555358	6.2512E-16	PAK4	1.33006646	0.0220276
RIGI	-1.41552045	3.2185E-05	SP4	1.32923873	0.0005578
TRAF3IP2	-1.41519308	0.0398483	FGGY	1.32908289	4.2929E-06
ENSG0000027	-1.41485621	4.1004E-06	ENSG0000027	1.32741367	0.00011605
RGS12	-1.41351003	5.4559E-05	ACOT13	1.32640072	4.4812E-12
TCIRG1	-1.41086376	3.2483E-17	MYO9A	1.32397237	2.321E-07
NCF1B	-1.41005554	0.00352596	ZNF76	1.32157501	0.00079666
VARS1	-1.40833442	6.4076E-06	KLHL28	1.32058375	8.3545E-07
GCHFR	-1.40825549	0.03852693	TRAF5	1.32043759	1.1959E-12
JDP2	-1.40706619	3.5064E-13	SEPTIN11	1.3192084	1.427E-08
UBALD2	-1.4064855	1.3509E-12	RAPGEF2	1.31837086	0.00012569
ENSG0000025	-1.39989165	0.00057149	DNAJB6	1.31731758	1.0107E-16
HIF1A-AS3	-1.39818104	1.3818E-06	CHD7	1.31662884	0.00011311
SLC7A5	-1.39762152	1.2977E-09	SEMA3C	1.31634427	0.00854336
TRIT1	-1.39565887	0.0048674	HLA-DOB	1.31535522	1.9452E-10

BAK1	-1.39421954	1.3731E-10	ENSG0000028	1.31367345	0.00362617
LINC00937	-1.39256973	0.0273679	RDX	1.31294669	3.9809E-05
GYG1	-1.39196467	3.3776E-12	MAP4	1.30830727	2.7969E-31
SLC12A9	-1.39109749	1.84E-13	ENSG0000027	1.30806511	0.00102564
ANKH	-1.39049558	0.04006382	PCYOX1	1.30712339	0.01496865
RALGDS	-1.38989664	3.5519E-06	STK4	1.30642094	7.3732E-16
C7orf50	-1.3894259	1.5271E-07	ZNRF1	1.3050635	6.4005E-07
FLVCR2	-1.38861411	7.1598E-06	CBX1	1.30427845	3.0152E-09
EPN1	-1.38785577	4.0715E-09	CPEB2	1.30367683	9.808E-07
LIMK1	-1.38781791	1.2471E-05	DSC2	1.30245129	2.4353E-09
METTL22	-1.38628246	0.00024594	TXNDC11	1.30116521	2.1845E-18
ADGRG5	-1.38615707	0.01700464	ZRANB3	1.30095039	0.02530511
CYBA	-1.38473032	2.7524E-28	RAB12	1.30090194	5.8486E-07
DGAT1	-1.38434591	0.00023054	FBXL4	1.30051227	0.03941599
GRAMD4	-1.38407228	0.00072477	B3GALNT2	1.29746319	2.1071E-06
UQCC2	-1.38392539	1.9822E-05	EXO5	1.29649691	0.03356556
REC8	-1.38293527	0.00318062	CDK2	1.29622849	2.905E-10
IQGAP2	-1.38274359	1.1777E-10	APLF	1.29476082	0.01676765
ZNF276	-1.38187448	0.00642891	MMP25	1.29387808	2.1038E-08
TGFA	-1.38043652	0.01421768	ABHD3	1.29289795	5.0773E-09
COQ2	-1.37997816	0.00024183	CREG1	1.29217701	8.1368E-15
SGSH	-1.3759145	0.01284036	AEBP2	1.28955418	2.1768E-12
LRRCC1	-1.37576767	0.01206189	TP53I11	1.28883528	0.00349707
IRAK3	-1.37346776	1.0863E-13	ACVR1	1.28823938	0.00031603
DNAJB11	-1.37243914	6.6869E-15	PPP2R3A	1.28720242	0.01040307
HSBP1	-1.36920137	2.0783E-21	UNKL	1.28539732	0.02015768
AIFM1	-1.36909856	4.4999E-06	EPS15	1.27942574	4.6499E-12
YIPF1	-1.36819229	0.00027826	CLSTN1	1.27828999	8.3476E-10
ETS2	-1.36786863	8.6875E-16	TRAF2	1.27781577	6.1718E-05
TIMM13	-1.36750098	2.9223E-09	FANCL	1.27774007	4.2052E-05
SLC39A8	-1.36297407	0.00018599	ZNF618	1.27743841	0.02265726
GNA15	-1.36167624	7.4914E-12	ENSG0000028	1.27331433	0.00461047
SLC66A2	-1.35894372	1.3379E-17	CRYBB2P1	1.27167102	0.0008479
FERMT3	-1.35851286	1.8867E-20	SLC25A35	1.2704921	0.03316406
S100A11	-1.3564357	8.573E-14	ENSG0000025	1.26633817	0.02968841
SCRN2	-1.35595482	0.02742492	UBE2E1	1.26467503	3.8254E-07
MCF2L2	-1.35433751	0.00297702	EPOP	1.26366889	0.00285736
MIF4GD	-1.35430302	2.0856E-07	OGA	1.26147008	2.119E-20
HLA-DQB1	-1.35312786	1.0475E-11	ARMC9	1.26069161	0.04706595
CRELD2	-1.35201126	2.3396E-06	OPTN	1.2587333	6.7294E-05
TFEC	-1.35151147	0.00026047	C1orf21	1.25801704	0.00223835
SDSL	-1.35004603	9.3726E-05	ZC3H12D	1.25737778	0.00388845
CTDSP1	-1.34961736	4.2303E-06	CAMSAP1	1.25713488	4.9664E-09
SLC22A4	-1.3490797	0.04605279	DENND5A	1.2569377	1.6512E-09
PLBD1	-1.3486414	3.0693E-07	RUFY3	1.25680016	2.9926E-23
SIGLEC1	-1.34853507	0.03021231	CYBRD1	1.25477538	0.00023679

RPN2	-1.34796157	3.3724E-15	GRK3	1.25406812	8.9703E-22
RNF13	-1.34767261	1.6623E-14	TUBB	1.25235072	3.7227E-11
FAM210A	-1.34760416	2.9748E-09	PIWIL4-AS1	1.25034119	0.03722933
HSD17B10	-1.34724023	4.3139E-12	AGL	1.2498758	0.01375227
SPTLC2	-1.34691147	3.4854E-12	NT5DC1	1.2496442	1.0866E-07
VAMP5	-1.34350745	3.6592E-08	TENT4A	1.24892057	8.6827E-13
SYN1	-1.34322693	0.00116586	REPIN1	1.24890663	3.2652E-10
GAS2L3	-1.34201401	0.00751782	CFB	1.24646109	0.00381515
PSMB3	-1.34134246	9.0619E-23	GEN1	1.24544815	0.00162391
ENSG0000029	-1.34122914	0.00408477	ATF6	1.24324132	7.2869E-08
CLTB	-1.34009358	1.7204E-26	SDC3	1.24221539	0.02457035
GIMAP2	-1.33844688	0.04126775	MVP-DT	1.24038649	0.00475025
TXNDC5	-1.33817726	1.8212E-05	USP40	1.23696217	0.02217167
NDUFA12	-1.33728878	1.6678E-13	TMOD2	1.23641096	0.00222763
GSTM2	-1.33612398	0.00044367	C19orf12	1.23506527	3.3648E-05
HSCB	-1.33609112	2.214E-05	ARID3A	1.23466565	2.1408E-05
DDX21	-1.33540886	6.6505E-15	ARHGEF3	1.23455664	2.0747E-05
FOSL2	-1.33452526	2.8633E-11	RAD51D	1.23407826	0.02602068
SEC11C	-1.33386807	1.7266E-07	SENP1	1.23380328	4.4614E-06
RUVBL1	-1.33353946	0.03116311	FAM117A	1.22982734	1.5896E-06
PLA2G4A	-1.33252709	0.000649	RNF115	1.22932924	1.9576E-11
ATAD3A	-1.33113261	0.03198672	JAK1	1.22840727	3.9429E-22
PLEKHO2	-1.32877659	1.0384E-05	FBRSL1	1.22720231	3.647E-08
AAAS	-1.32738808	0.00173659	CD5	1.22678449	0.01271614
NXT1	-1.32717575	8.3595E-12	HSPA2	1.22480638	0.04440329
IDUA	-1.3265633	0.01406501	SIL1	1.22446897	8.1682E-11
NUDT1	-1.32575067	8.7248E-09	ARL17A	1.22405425	0.00017798
DNMT1	-1.32507587	6.3811E-14	RIPOR3	1.22278393	0.02669928
SIRPB2	-1.32485586	0.00865342	FAM118A	1.21997806	3.2404E-09
SLFN11	-1.32294199	0.00020365	RALGAPA2	1.21849886	0.00459254
MAT2A	-1.32270387	1.0986E-06	SMURF1	1.21825372	4.1703E-10
ENSG0000027	-1.32203843	0.00117309	TMEM97	1.216757	0.00564416
ETFRF1	-1.32191907	0.0283199	ULK2	1.21666865	0.0078472
FOS	-1.32142067	0.00578508	ENSG0000028	1.21623662	0.00026641
MIER2	-1.32141997	0.00164314	SLC39A13	1.21580071	0.00016771
FOSL2-AS1	-1.3206995	0.00875089	ZHX2	1.21375682	8.854E-08
ABHD17A	-1.32061221	7.4736E-12	PTGR3	1.21350559	0.00034245
PSMA6	-1.32049793	3.4748E-22	NOD1	1.21272813	0.01823452
SERPINB1	-1.32029884	2.9048E-07	LNCAROD	1.21249233	0.0022589
ZNF706	-1.31724311	8.0685E-09	TP53INP1	1.21201182	0.01871054
AP5B1	-1.31722004	0.00045418	DDX11	1.21052682	0.02181141
METTL7A	-1.3157719	0.01922964	DMXL2	1.20969761	0.00048667
MAX	-1.31527651	1.0628E-16	ZNF821	1.20953606	0.02413512
RNF130	-1.3141858	6.0665E-29	RABGAP1	1.20894444	7.763E-13
PHLDB1	-1.31382184	0.00156857	MYO1G	1.20671588	2.6759E-08
CCNB1IP1	-1.3138021	0.00011074	PPP3CA	1.20603186	4.0164E-06

SHMT2	-1.31363762	1.2342E-06	HSD3B7	1.20183233	0.00623268
INHBA	-1.31350341	1.9415E-05	KIFBP	1.20092899	0.0051056
KIF16B	-1.31174151	8.937E-05	ST3GAL2	1.19975143	5.4907E-06
PDE4D	-1.31159986	0.00090103	CSRNP2	1.19927918	0.0329283
ENY2	-1.31066219	1.9488E-17	MIR646HG	1.19909462	0.04869963
KCNG2	-1.30864002	0.0355453	RHOXF1	1.19823942	0.0267134
BCL7A	-1.30685631	0.00306723	PALLD	1.19704677	1.5428E-10
PRKACA	-1.30498859	7.8986E-08	SREBF2	1.19385921	9.6663E-13
UBA7	-1.3048536	0.0021422	REEP3	1.19376636	7.0361E-10
GTF3A	-1.30348174	4.3202E-07	NRM	1.19185594	1.9261E-05
QSOX1	-1.30223301	1.8489E-14	ALDH4A1	1.19167095	0.00858843
CES2	-1.3018815	0.01591959	RND1	1.19084069	0.00360263
GTF2B	-1.30087963	1.2977E-09	ST3GAL5	1.18982657	2.1411E-05
ERI1	-1.30082421	4.7663E-05	SMNDC1	1.18787038	7.1388E-19
ARFGAP3	-1.30069824	4.4519E-13	JCHAIN	1.18667898	0.0101822
SLC25A5	-1.30053463	3.1479E-40	KATNAL1	1.1866275	0.03935502
ATP5PB	-1.30036542	1.3225E-20	NPIPB5	1.18656905	1.111E-06
THOC6	-1.30020346	5.7601E-07	NHSL1	1.18241122	0.00107289
TMED10	-1.29658953	1.185E-13	CHD6	1.18210899	3.9921E-13
BCKDK	-1.29627538	1.7067E-15	TSTD1	1.18166568	1.8734E-06
STAT2	-1.2961681	1.485E-21	STX2	1.18139002	0.00034496
CDC42EP3	-1.29580432	2.5417E-05	MYOM1	1.18129645	0.01964878
IGLON5	-1.29522089	0.00297231	SLX4IP	1.18050322	0.048579
SCPEP1	-1.29491781	2.1309E-06	TLCD3A	1.17917709	0.00549789
DCTN3	-1.29306713	2.2138E-11	STARD7	1.17748771	5.7195E-14
TFEB	-1.29189336	4.1994E-08	PLEKHA5	1.17723328	0.04805607
ALDH9A1	-1.29142084	0.00037472	FCHSD2	1.17503278	2.0952E-16
TNFRSF10D	-1.28997125	0.04346674	SLC44A1	1.17489596	1.8745E-08
SYVN1	-1.28892047	4.8368E-05	LINC01191	1.17376963	0.04692284
OGFR	-1.28678479	2.9558E-14	BCL2L1	1.17354279	3.6783E-08
MSRA	-1.2866195	1.3167E-09	GGH	1.17288918	0.02516651
ANXA5	-1.28637025	4.0381E-32	ZBTB5	1.17236625	0.00698328
UBE2D1	-1.28606501	7.8756E-18	FAM234A	1.172244	0.00061542
CDC26	-1.28515413	5.4478E-11	ST8SIA4	1.17004863	6.5028E-16
SSB	-1.28342487	1.1223E-12	LUZP1	1.16997082	2.7541E-09
MCTP1	-1.28049629	1.9998E-08	TBC1D10A	1.16975424	8.9851E-09
B4GALT3	-1.2796432	0.00206689	ZC3H7A	1.16754012	1.9538E-21
ZNF787	-1.27926136	2.0546E-05	USP45	1.16515883	0.00144378
MYL12A	-1.27788256	1.3449E-24	PSIP1	1.16129721	1.5629E-09
SPHK1	-1.27740555	5.4371E-05	BASP1	1.15978211	1.8046E-07
DGKD	-1.27640453	0.00038979	DUSP5	1.15973723	2.64E-07
HES1	-1.27626998	0.00081733	MTMR9	1.1590374	0.00023977
PPARD	-1.27484222	1.4303E-08	ZNF281	1.15740551	2.2314E-05
ANPEP	-1.27448702	5.6816E-05	ZDHHC18	1.15700581	1.2836E-09
VPS37B	-1.27440201	1.4431E-05	GXYLT1	1.15584421	4.8165E-06
NFE2L3	-1.27367901	1.9238E-05	NLK	1.15573366	0.01031033

GLA	-1.27082673	9.7967E-09	IQSEC2	1.15519683	0.02657875
SMPD1	-1.2694776	0.03336738	ZDHHC14	1.15513589	0.01189552
UNC119	-1.26894179	3.7833E-07	TRA2A	1.15491876	7.1249E-12
TLR8	-1.26845395	0.00093206	TIFAB	1.15261045	0.00288503
CCT5	-1.26820628	4.533E-13	BBC3	1.15258547	0.01642356
GK-AS1	-1.26793397	0.00074248	SSH1	1.15007918	3.7321E-08
DENND4C	-1.26742423	1.5264E-05	APH1B	1.14931145	6.0411E-05
MRPL12	-1.26691991	9.8876E-07	DDB2	1.1484825	1.4295E-05
SSBP1	-1.26668996	1.3763E-17	MALAT1	1.14635374	8.3777E-06
LY6E	-1.26638321	1.6588E-09	CCSER2	1.14573753	1.9784E-05
CPPED1	-1.26461092	5.008E-12	RIC1	1.14356776	1.6276E-11
TIMM50	-1.26428934	3.9247E-05	RAP2B	1.14284487	5.1742E-14
UNC13D	-1.26356063	0.00279081	SLC41A2	1.14113999	3.1919E-07
CSTF2T	-1.26352492	0.002526	HMOX1	1.1394284	0.0002716
ENSG0000027	-1.26184162	0.04259374	BLVRA	1.13802424	1.4586E-19
TTYH3	-1.26168984	8.0289E-09	CALCRL	1.13718791	0.00011712
SLC7A7	-1.26075962	2.2346E-06	PPP1R15B-AS1	1.13536216	0.02657875
FLT3LG	-1.25992636	0.00376886	FNDC3B	1.13446707	1.2026E-08
MKKS	-1.25844016	2.0853E-05	TYK2	1.13427992	1.4037E-05
DENND4B	-1.25668603	0.00046393	FARP2	1.13188314	5.1901E-06
DNAJC10	-1.25618596	6.7656E-06	ENSG0000024	1.12945695	0.00058582
SPI1	-1.2556731	2.6781E-11	TEX2	1.12861983	0.04142639
CASP4	-1.2544784	3.4467E-11	B4GALT6	1.12557443	0.01798399
CHST14	-1.25436361	0.03008436	MSANTD3	1.12500462	0.02413671
STAT1	-1.25414871	7.3593E-19	NET1	1.12455361	6.473E-05
FZD2	-1.25351745	0.00355468	SLC1A3	1.12238103	0.00305056
CASP8	-1.25021748	3.6515E-05	WASHC2A	1.12209277	5.0847E-07
TMEM9B	-1.25016229	6.5877E-10	NBPF11	1.12182626	0.02092369
PEPD	-1.24919526	4.5089E-08	WNT2B	1.12158348	0.00057636
HAT1	-1.24902582	0.00003349	SAMD8	1.12109076	9.2982E-10
LMTK2	-1.24900668	0.01785684	SMC5	1.12045144	6.3171E-08
HNRNPLL	-1.24861868	3.2758E-05	EMP2	1.11983586	0.00379738
NDUFV3	-1.2484872	1.3669E-06	GPBP1	1.11968399	4.1701E-13
CMC2	-1.24793164	7.4301E-08	SORBS1	1.11879797	0.03852121
ORAI3	-1.24746316	1.4949E-05	EZH1	1.11809018	0.00431005
COX7B	-1.24733716	1.1495E-12	MTMR10	1.11549184	0.00012903
ENSG0000028	-1.24709683	0.03165203	MARCHF9	1.1153696	0.00362105
POR	-1.24498983	5.416E-06	TBC1D9	1.11509515	0.00011547
ASPHD2	-1.24493827	0.00137736	ZFAS1	1.11263751	4.263E-22
SYAP1	-1.24488929	5.6808E-06	ARHGAP26	1.11257786	0.0006358
HDAC4	-1.24486502	1.7103E-05	MYO5A	1.11094423	4.1378E-10
RTF2	-1.24343596	8.1722E-10	CASP6	1.10713878	0.03634793
SH3RF1	-1.24169014	0.00825619	SPPL2A	1.10602041	3.1011E-15
DOK1	-1.24102459	8.1695E-06	SMC6	1.10472677	0.0002662
CDK2AP2	-1.23964458	1.3555E-09	YWHAQ	1.10441986	6.9543E-21
ILVBL	-1.23777656	0.00291323	TOM1L2	1.10413291	0.01524065

PLEKHM1	-1.23698008	0.00039278	JUN	1.10370223	0.01210228
MT1X	-1.23634196	6.1395E-05	UBXN2A	1.10289318	8.5308E-06
DNPEP	-1.23620304	5.2829E-06	PRKCE	1.10259259	0.00024321
VEGFB	-1.23616097	7.1209E-14	IGHA1	1.10087783	0.02524481
C11orf24	-1.23592981	0.00075285	RNF144B	1.09944052	2.4062E-11
PSMG1	-1.23529629	0.00294401	CLASP1	1.0993008	2.7245E-08
MPND	-1.23475383	7.3159E-06	TCP11L1	1.09699923	0.00087277
GATM	-1.23449464	0.00187897	RNF145	1.09515041	4.7062E-08
ALDOA	-1.2344937	3.1632E-12	FILIP1L	1.09306077	4.5874E-07
SNX20	-1.23386847	2.2601E-06	NPFFR1	1.09292079	1.7908E-06
PDIA4	-1.23297578	4.4353E-07	CSF2RA	1.09267369	1.7984E-14
ITGA5	-1.23271242	0.03763179	VPS8	1.09251094	4.0263E-10
EIF4A1	-1.23094436	1.2246E-31	FAM135A	1.09246082	1.5549E-11
PHLDA2	-1.23078615	7.7469E-06	WWC2	1.08872111	9.7854E-05
SIRT2	-1.23046925	1.3833E-09	LRRFIP2	1.08848796	3.7092E-14
CYP4V2	-1.22876058	0.01781918	CDCA4	1.08535103	0.01537518
TIPARP	-1.22824031	1.1984E-08	PGAP2	1.08529495	0.00480488
ERP29	-1.22805269	8.2337E-11	KIFAP3	1.084404	0.00008217
PRDX2	-1.22791399	2.8135E-05	P2RY8	1.0837724	5.3598E-10
HEBP1	-1.22589618	1.9378E-06	ABHD17B	1.08130561	2.2752E-05
C4orf48	-1.22539734	2.0608E-15	TTL	1.08122741	0.0001175
GK	-1.22498649	5.5881E-07	ZNF592	1.08022257	8.6302E-08
MPRIP	-1.22395719	9.4735E-05	TMEM176B	1.07904004	0.00098463
HLA-DRB5	-1.22381192	6.5276E-10	KMT2A	1.07829367	2.922E-13
C1GALT1C1	-1.2234874	0.00033046	EGLN1	1.07769473	7.2303E-05
CENPN	-1.22340556	0.03202342	TRIO	1.07611789	1.3419E-18
EIF3K	-1.22277953	3.5218E-19	FBXL8	1.07538851	0.01616185
ARAP1	-1.22133973	4.5137E-06	ASH1L	1.074963	5.0967E-13
REX1BD	-1.22119435	2.4348E-11	RRN3	1.07422253	5.2027E-05
NHERF1	-1.22068766	1.946E-06	SLC6A6	1.07097616	2.3045E-08
MRPL33	-1.21953806	4.3284E-08	PPP1R15B	1.06958661	5.0862E-08
BORCS5	-1.2195289	7.6398E-05	MTHFR	1.06647714	0.00090121
COMMD5	-1.21936652	6.107E-08	GEMIN8	1.0663918	0.00704741
ATG2A	-1.21895276	0.00036252	LRRC28	1.06518166	0.00037559
CD99	-1.21719025	1.1487E-06	ZBTB20	1.0634028	2.0533E-06
MRTO4	-1.21446161	0.00423395	DHX58	1.06257283	1.6828E-05
SOAT1	-1.21422753	1.4914E-11	PHLPP2	1.06134277	0.00555302
PNPLA6	-1.21362472	3.5331E-05	DYRK2	1.06094143	0.01463819
SNU13	-1.21322458	4.1429E-14	HS6ST1	1.06069889	0.0005029
ATPAF1	-1.2125689	0.01441595	TBC1D31	1.06006838	0.04752645
RASA4	-1.21219143	0.03521482	MED14	1.05983727	1.8222E-06
SH3BP1	-1.20700962	1.9245E-07	TNNI2	1.059211	0.02999216
BLOC1S1	-1.20619243	1.0738E-12	ZRANB1	1.05861791	9.5178E-05
NOP16	-1.2056724	3.8157E-05	ARL17B	1.05593286	0.02578618
FCHO1	-1.20543726	0.01873652	FAM222B	1.05486627	0.00269124
ANAPC13	-1.20155202	0.00021772	IRF4	1.0547312	5.1507E-09

HLA-DRB1	-1.20122939	9.7758E-11
LIMS1	-1.20120728	1.1075E-13
MNT	-1.20039528	0.00377993
POLR2H	-1.20008921	1.861E-05
ABHD16A	-1.20008794	3.2749E-06
BISPR	-1.19957396	0.03537577
CCDC85B	-1.1994788	4.5393E-05
ANKFY1	-1.19929479	0.00262921
LMAN1	-1.19857174	0.00083706
HTR7	-1.19849279	0.0145668
SRA1	-1.19802272	6.2343E-15
SUCLG2	-1.19709033	0.00284907
GPX1	-1.19569651	3.628E-18
BOP1	-1.19454745	0.00388522
PIGF	-1.19360948	0.01269367
IRF7	-1.19355279	6.2343E-15
PUS1	-1.1935463	0.01482665
PPIB	-1.19270582	1.4606E-11
NRARP	-1.19208281	4.5553E-07
ITGAE	-1.19183074	1.7305E-08
ENG	-1.19044758	0.0001263
NEK1	-1.18970001	3.9815E-07
FIBP	-1.18950437	2.5738E-06
DMAC1	-1.18874992	8.2054E-05
CARD19	-1.18742807	2.4291E-08
ADCY7	-1.18564256	4.3972E-06
RAP1A	-1.18551665	4.1616E-18
MRPL47	-1.18426216	0.0008015
CHMP2B	-1.18332472	7.4531E-11
FOSB	-1.18253181	0.00017529
UCHL3	-1.18141171	7.588E-06
ERF	-1.18056278	0.00141294
NDUFS8	-1.18000796	4.029E-19
C15orf39	-1.17837557	0.0001031
CREM	-1.17811658	7.413E-09
NHP2	-1.17753021	1.3999E-09
RPS6KA4	-1.17619017	2.7625E-05
EDEM2	-1.17439261	0.00032656
ATP5PF	-1.17298823	1.7763E-14
DISP1	-1.17232795	0.02265368
ZNF263	-1.17217685	0.00260446
HLA-DPA1	-1.17191803	8.6956E-07
STK32C	-1.17153913	0.02166497
SNRPF	-1.17002665	6.501E-15
LEPROTL1	-1.16880102	1.9592E-12
IFT20	-1.16862139	7.0122E-07
SMS	1.05397981	2.7505E-12
CREBRF	1.05252749	1.7311E-08
TEDC1	1.05014048	0.00070369
CDK17	1.04958678	1.9453E-09
FBXL20	1.04907427	0.00281542
NPEPPSP1	1.04833211	0.00033481
GNG4	1.047633	0.00145282
SMC4	1.04685057	2.0118E-06
ATAD2	1.04574782	0.00244425
DHX34	1.04433296	0.03609494
MRTFB	1.04351057	0.02227156
TDP2	1.04309108	3.0617E-06
CD83	1.04210802	0.00017005
NEU3	1.04197353	0.00859071
OSTF1	1.04188566	7.8471E-15
CTTNBP2NL	1.03976561	4.4017E-06
CELF2	1.03867905	8.2022E-10
ARL3	1.03449425	0.00074428
ENSG0000023	1.03297429	0.0220421
ST20	1.03257052	2.4098E-05
BMF	1.03177655	0.00276476
LRRC57	1.03155346	0.00422257
RIF1	1.03089595	7.8596E-09
SPOP	1.02922091	0.00015388
SAMSN1	1.02560462	6.3362E-05
TMEM39A	1.02538534	3.334E-05
NFYA	1.02409599	0.00881252
CLCN6	1.02237338	1.4898E-05
IFT43	1.02153712	0.00098291
HLA-L	1.0214799	0.00104256
SMYD4	1.02098156	0.00213674
TRIM24	1.02096511	0.00177666
PDE4DIP	1.02093118	0.00022164
FBXL14	1.02029525	0.00186207
TOR1AIP2	1.01860805	2.6658E-05
ZNF43	1.01813011	0.00149301
DLGAP1-AS1	1.0177444	0.03000423
KYAT3	1.01618942	6.8115E-07
ASAP1	1.01567785	4.9527E-08
ENSG0000028	1.01501118	0.008535
CCDC18-AS1	1.01479134	0.00024802
BCLAF3	1.01368885	0.04805607
SLC37A1	1.0129732	2.0622E-05
TULP4	1.01140523	0.00255643
CDKN2C	1.01006472	0.04888282
JADE2	1.00961181	0.00473196

ZNF703	-1.16833636	0.0103671	ENSG0000028	1.00852131	0.00536501
ARID5A	-1.16750048	5.3974E-05	SNX29	1.00708158	2.7257E-05
MYPOP	-1.16640961	0.00170621	INO80D	1.00450628	4.2245E-06
ALG1	-1.16633352	0.04656083	CXXC5	1.00393153	0.00223733
CD180	-1.16444573	0.0004068	HERC1	1.00373846	0.00025788
ATP5MC3	-1.16372133	1.1325E-14	DUSP16	1.00232856	2.64E-07
BTF3L4	-1.1634173	1.31E-06	TBC1D17	1.00140942	0.00167548
VDR	-1.16341297	1.5676E-06	GABARAPL2	1.00101909	0.00011005
NDUFS6	-1.16166963	1.1777E-10	USP53	1.00023213	0.0001577
YWHAH	-1.16079739	2.6207E-24	TTC28-AS1	1.00022404	0.01040347
NDUFA8	-1.16033617	3.8985E-07	ZNF100	0.99800763	0.00706817
SNAP29	-1.16026115	5.7834E-13	FAM107B	0.99761609	2.6852E-05
SLC11A2	-1.15883978	0.01028238	R3HCC1L	0.99709633	0.00029107
PSENEN	-1.15733949	6.3562E-11	ZMAT1	0.99580069	0.02861842
TSEN15	-1.15541559	0.00122032	SVIP	0.99571895	0.00264657
ENSG0000028	-1.15541389	0.04164456	ZNF506	0.99420785	0.00774274
CXCL9	-1.15434573	0.00398682	USP46	0.9935807	0.00176517
ZC3H15	-1.15417048	8.0028E-07	GABBR1	0.99289036	0.01589209
GPSM3	-1.15401786	3.0405E-21	SDC4	0.99259361	0.01256432
ENSG0000023	-1.15314675	0.01354968	GPR132	0.99222626	3.1307E-06
DRAP1	-1.15169835	4.3563E-24	GABPB1	0.99159008	6.668E-16
ENTPD1-AS1	-1.15044712	0.00073437	ENSG0000029	0.99098034	0.03961393
CYTH3	-1.15004712	0.00161102	CRAMP1	0.99079572	0.02816452
NDUFS5	-1.14978265	1.2481E-13	ACAD11	0.99043083	0.01375138
SLC16A3	-1.1494544	1.6911E-11	PEAK1	0.98876501	0.00024834
SH3BP2	-1.14932227	7.0387E-11	C6orf62	0.98849076	1.4652E-07
RPL22L1	-1.14924787	8.485E-20	CIB2	0.98572726	0.00703231
ZMIZ1	-1.14895935	1.7627E-11	SLC35E2B	0.98503524	0.00363358
NOL12	-1.14857505	0.00302618	CYRIA	0.9848367	1.7021E-06
DTWD1	-1.14846264	0.00479123	ZNF611	0.98454024	0.01805162
SELENOK	-1.14797165	5.633E-07	XYLT1	0.98386417	1.6714E-05
GOT1	-1.14742779	0.00835442	BMP6	0.98383714	0.00697067
CISD3	-1.1470883	0.00082823	FHIT	0.98309031	0.00809768
CCSER1	-1.14681414	7.8148E-06	CLINT1	0.98294928	4.5944E-14
LRMDA	-1.14644888	1.3143E-09	MED13L	0.98117527	5.4765E-05
MLF2	-1.1455106	4.1994E-09	HDGFL3	0.97970923	3.3274E-06
PMVK	-1.14518231	7.3375E-06	NOP58	0.97958905	2.8004E-09
DNAJC13	-1.14385299	1.8443E-10	ELP2	0.97923831	0.00036739
SPCS3	-1.14335527	1.8916E-06	ANAPC10	0.97842029	0.00016302
FBXO4	-1.14263008	0.03356886	ERGIC1	0.97722302	4.5461E-05
PICALM	-1.14249747	3.1537E-21	VPS54	0.97640578	0.02668634
SKIC8	-1.14187792	1.283E-06	NHLRC2	0.9744659	0.00137358
AK2	-1.14162597	1.6285E-05	MYL5	0.97291661	0.0247286
PGK1	-1.13996167	4.9984E-16	YEATS2	0.97220373	0.00040491
KLHDC3	-1.13688925	0.00221814	OXNAD1	0.97104499	0.00370895
VPS35	-1.13550565	4.8855E-20	UBTF	0.97002974	2.7914E-07

ETHE1	-1.13511293	1.4194E-09
MRPS34	-1.13430531	3.161E-05
HLA-DRB6	-1.13379909	3.5396E-07
MDH2	-1.13212506	1.1219E-14
LUCAT1	-1.13192613	0.0197178
GBP2	-1.13181516	1.0287E-09
RAB3A	-1.13172524	0.00429809
TIMM44	-1.13167024	0.02565508
ZCCHC3	-1.13139657	0.01766034
OSCAR	-1.13008796	0.0011965
IL17RA	-1.12948517	6.9005E-05
ECHS1	-1.12888168	5.4708E-16
CD63	-1.12857771	4.3932E-13
SMDT1	-1.12851244	2.18E-25
RHOT1	-1.12788881	3.9677E-06
PWWP2B	-1.12667362	0.00145595
CTSA	-1.12635042	1.4815E-12
NAA10	-1.12556074	0.00921741
LINC01003	-1.12527107	0.03019211
EVA1B	-1.12423482	1.0109E-08
COX8A	-1.12401099	3.8787E-15
GGCT	-1.12385307	1.2544E-05
QTRT1	-1.12325738	0.00231664
PSMA4	-1.12217163	9.6362E-13
RAB31	-1.1201839	6.3485E-07
PTPA	-1.11936301	5.0404E-05
TESK1	-1.11926694	0.00012114
COA4	-1.11800813	3.5927E-07
TMEM70	-1.11751781	4.7507E-05
XBP1	-1.11616387	1.0378E-15
PARP9	-1.11527664	6.4164E-07
MRPL55	-1.11479565	1.2745E-06
ATP6AP1-DT	-1.11468632	0.00261914
YIF1A	-1.11463321	2.1763E-07
LAMP5	-1.11450831	0.00170181
MPPE1	-1.11379966	0.00084546
RASSF5	-1.11374684	1.3147E-08
SPNS1	-1.11344224	1.7103E-05
ARHGEF1	-1.11263429	3.0347E-06
PRKX	-1.11262173	0.00154745
NDUFAF4	-1.11228155	0.02962973
RRAGA	-1.1121406	0.00498094
MRPS26	-1.11197697	2.3335E-05
UROS	-1.11187588	0.00037557
TIMM8B	-1.1118587	1.0029E-09
GCH1	-1.11152978	1.1328E-05
AHCTF1	0.96969296	2.0705E-05
ENSG0000029	0.96931438	0.02582398
ITPKB	0.96844398	0.04842463
PTGES2	0.96762086	3.4631E-14
RHOF	0.96744203	2.0979E-06
DNAJC9	0.96708707	0.0009153
LINC-PINT	0.96558995	2.8093E-06
H2BC12	0.9649033	1.2639E-05
MARK4	0.96475209	0.0024862
KLF13	0.96464731	1.3693E-09
KCNMA1	0.96449718	0.00913499
C2CD2L	0.96402761	0.00084212
HIVEP2	0.96392129	0.0008488
GALC	0.96189303	8.2838E-05
BLTP3A	0.96144556	0.00021384
STARD4	0.96090175	0.00046431
COMTD1	0.96044668	0.00023362
MAP1LC3A	0.95950978	0.00857609
PI4K2B	0.95884938	0.00957702
ZNF366	0.95853498	0.00046704
IFFO2	0.95759062	0.0005484
RARS2	0.95561325	0.00640913
RAD18	0.95462263	0.01087431
ZDHHC7	0.95443367	1.2099E-08
MICAL2	0.95358845	0.00113883
SNAPC3	0.95200683	0.0294595
PPP1R9B	0.95156238	0.0004787
PALS1	0.95066823	0.02995431
DPYSL2	0.94998708	1.1981E-06
ZNF639	0.94944815	0.01111798
ASXL1	0.94644922	2.6674E-06
PANK3	0.94588496	6.754E-06
DNAAF9	0.94550075	2.7534E-05
TCFL5	0.94429008	0.01990164
ANXA7	0.93950015	1.1346E-09
NECAP2	0.93943588	2.7214E-14
PI4K2A	0.93910418	4.6094E-05
SCAF8	0.93891502	7.0208E-05
SMG1	0.93875825	4.4978E-09
PDXDC1	0.93840397	3.0889E-05
MCM5	0.93697536	9.5309E-07
ZCCHC7	0.93634532	0.00026209
SGMS1	0.93571879	0.00146445
ZNF407	0.9354311	1.1182E-05
IGF2BP2	0.93494688	3.6194E-07
AGRN	0.93463019	0.01012357

RAB5IF	-1.11125001	7.3188E-15
PAGR1	-1.10912105	3.3805E-07
VCAN	-1.10891341	0.00037472
THADA	-1.10704718	0.00024231
GBP4	-1.10659243	8.0552E-10
OSBPL11	-1.10625913	3.6691E-05
RAB5C	-1.10603253	9.9764E-26
BID	-1.104923	1.942E-12
MYDGF	-1.1048762	4.8892E-10
ATP5MC1	-1.10447471	6.2362E-07
ARFIP2	-1.10412188	0.00028452
FTSJ1	-1.10397678	0.00033488
IFRD2	-1.10353969	0.03066314
VAV1	-1.10336772	4.2003E-05
ATP2B1-AS1	-1.10280401	0.0008499
GRN	-1.10199242	8.2472E-23
BST2	-1.10031915	5.6012E-21
ACAT1	-1.09973547	0.01860477
SDHB	-1.09961288	5.5442E-06
APEX1	-1.09945865	3.5901E-08
TLNRD1	-1.09883911	7.5625E-05
LFNG	-1.09867402	1.1536E-05
TMED2	-1.09827434	1.6573E-07
G6PC3	-1.09805824	0.0008028
ZNF106	-1.09762751	1.2883E-06
MICOS10	-1.09558614	7.3747E-12
SEPHS2	-1.09419274	1.4235E-06
UBXN2B	-1.09243008	0.01423826
TFPT	-1.09205493	9.7153E-08
COLGALT1	-1.08960312	0.00051643
OSTC	-1.08895083	3.8934E-09
DENR	-1.0885113	3.2735E-05
RENBP	-1.08838875	0.00806564
TMCO1	-1.08828977	3.3389E-06
RAB34	-1.08711407	2.3045E-08
COX16	-1.08699437	1.0079E-09
HEBP2	-1.08646681	9.8195E-05
TMEM126A	-1.08628022	2.6972E-06
MIA3	-1.08457751	3.3426E-07
RNPEP	-1.08413031	4.8452E-13
AHCY	-1.08410718	2.1494E-05
VEGFA	-1.08276907	2.5446E-05
NPC2	-1.08272506	5.7124E-13
BLVRB	-1.08270415	5.2454E-09
FABP5	-1.08176488	4.9962E-06
CCR5AS	-1.0817615	0.03129918

CNTLN	0.93387396	1.0067E-06
SLC2A1	0.93317984	0.00019038
MALT1	0.93283175	3.7767E-08
TMEM63B	0.93202522	0.00219323
YEATS4	0.93013636	0.00349038
SNN	0.93012287	5.2287E-12
GADD45G	0.92742213	0.01825589
AFDN	0.92623029	9.0998E-09
FLOT2	0.92571724	2.2278E-07
IL13RA1	0.92556169	7.275E-12
TAF5L	0.92499126	6.3642E-06
GRAMD1B	0.92495085	0.00021557
ZNF532	0.92326737	0.00388672
BMAL1	0.92312606	5.9306E-05
PLEKHM3	0.92264807	0.0148325
MMP2-AS1	0.92141304	0.04611754
KDM6A	0.91941703	1.2831E-08
GTSF1	0.91915314	0.04122745
ZMYM4	0.91912496	1.4727E-08
MRPL46	0.91876112	0.03996971
LINC03040	0.91591864	0.00436888
TALAM1	0.91577111	1.4843E-17
ST3GAL1	0.91344281	5.8445E-09
SLC39A11	0.91336005	0.00172048
B3GALT4	0.9130376	0.00903432
CEP192	0.91153653	0.0003611
MAN1B1	0.91125418	0.00305946
GFM2	0.91097048	0.04249068
CPNE8	0.91068371	0.00268753
TAF4B	0.91048103	0.00069327
FAM98B	0.90881269	0.00036163
TPT1-AS1	0.90776201	0.01369216
HSPB1	0.9063312	1.0161E-05
ARHGEF2	0.9054246	5.6498E-10
ANKRD11	0.90411017	1.2588E-15
AP1S3	0.90361143	0.00088213
CSNK1G1	0.90246801	9.438E-05
BAZ1A-AS1	0.90034974	0.02070311
EOLA1	0.90031751	0.00040501
ARPP19	0.89675036	3.1474E-08
N4BP2L1	0.89632961	1.1864E-05
CALCRL-AS1	0.89515356	0.00079147
PREX1	0.89316916	5.1876E-10
ENSG0000028	0.89210377	0.04680687
SNHG12	0.89100042	2.5546E-07
MTRFR	0.88983316	0.0329283

CAT	-1.08098768	4.158E-08	TTLL3	0.88834362	0.02211206
EBPL	-1.08091432	0.00209244	CASP3	0.88715471	6.3259E-06
HCCS	-1.08019527	0.00193146	PPM1A	0.88706112	0.00129527
GALNT3	-1.08011001	0.00010417	PTPN1	0.88466146	1.5969E-08
PDIA6	-1.07960598	4.9936E-07	NSD2	0.88087953	0.00030123
RCC2	-1.07848162	1.3529E-09	CDKAL1	0.8787694	1.8686E-07
LIMA1	-1.07798515	0.02862775	PHF13	0.87848281	0.04989806
LYPLA1	-1.0779041	5.449E-10	IL6R	0.87754805	2.8375E-06
KDELR2	-1.07750054	5.1882E-09	ADARB1	0.8764883	0.02520122
APAF1	-1.07743403	3.4584E-06	MLLT6	0.87588959	0.00309201
BLCAP	-1.0774205	0.00104256	PLD2	0.87558785	7.8671E-05
PPIH	-1.07651722	3.3997E-06	GALNT11	0.87550264	0.00044727
YIPF2	-1.07596587	0.00024795	MBD5	0.87535703	0.00021257
BORCS7	-1.07501664	6.2657E-05	DYRK1A	0.87402774	8.6427E-08
CNPY3	-1.07274846	9.5036E-13	ADAM19	0.87383043	0.00435533
RNF122	-1.07272509	0.01331675	PIAS3	0.8728032	0.04396303
UBE2J1	-1.07259737	2.1584E-16	NME3	0.87248586	2.0287E-07
ARMCX3	-1.07221685	3.1467E-07	SMARCA2	0.87152161	1.498E-06
TXNDC12	-1.07096732	5.0659E-06	VAC14	0.87123894	0.00016315
POP5	-1.07082708	4.0773E-05	PLEC	0.87065995	0.00363358
FAM117B	-1.07067596	0.00878966	NUP50-DT	0.86854176	0.00689597
MLX	-1.06951259	2.0182E-12	RNF207	0.86773202	0.04935697
OAZ2	-1.06888547	2.4317E-07	MLLT10	0.86731536	8.2302E-07
TCEAL4	-1.06875276	0.0006545	ABCC1	0.86673237	5.1784E-05
INSIG1	-1.06868155	3.2202E-06	MACF1	0.86389657	3.4579E-08
ARF3	-1.06841651	8.6673E-09	STARD10	0.86378551	0.04242096
PYGL	-1.06742495	0.04032499	WNK1	0.8635961	0.00013852
SPATA2L	-1.06701443	0.04651801	TRAF3	0.86336567	4.0288E-09
GNB4	-1.06573841	7.4568E-07	TSHZ1	0.8632225	0.00067141
GSTZ1	-1.06498721	0.03165571	CLCN7	0.86212209	0.00749954
C1orf174	-1.06475767	0.00299283	INTS6	0.86208394	0.00077887
TMEM150A	-1.06387251	0.00156166	ARID4B	0.86144819	4.0844E-08
SEC61G	-1.0614546	2.0983E-07	ARL6IP6	0.86114791	1.3812E-07
TFAM	-1.06135468	9.4703E-06	DCAF8	0.86106117	0.00062471
GPATCH4	-1.06126642	0.03371737	LRSAM1	0.86069102	0.04234387
TSPO	-1.06024537	0.00079672	TOMM34	0.85996659	2.7039E-10
ZNF433-AS1	-1.05988415	0.00050531	CREB1	0.85789523	1.0347E-05
TMEM59	-1.05882225	2.2852E-10	SVIL-AS1	0.85742263	0.01157292
FKBP2	-1.05856702	7.5096E-05	TMEM123	0.85620956	5.9347E-10
FLI1	-1.05841671	0.00121583	DPP9	0.85553372	2.2038E-06
SNX30	-1.05787272	0.00964717	INPP5K	0.85380453	0.0001125
SSR4	-1.05726243	8.404E-24	TPD52	0.85199438	0.00157249
LRRC58	-1.05642251	0.01453783	SYT11	0.85124568	0.03371737
EXOSC7	-1.05555812	0.00325744	PPP1R21	0.85093437	0.00010011
CEBPZ	-1.05554793	0.00020285	LSP1	0.85085794	7.3283E-07
LAP3	-1.05544599	3.0709E-25	GLI4	0.85048759	0.04416259

ANAPC15	-1.05516192	0.00025779	ZBTB24	0.84874323	0.00059727
MRPS35	-1.05407556	0.00157679	SLC7A1	0.8478114	0.00072742
INO80C	-1.05329653	0.00426442	JAK2	0.84622603	1.0161E-06
ABHD14B	-1.05310866	0.00061386	MVD	0.8461371	0.03647985
ROGDI	-1.05134106	0.00113619	FXR1	0.84401467	4.268E-09
LTBR	-1.05091175	9.4547E-13	TJP2	0.84242036	0.00180377
C1orf122	-1.05034224	5.7565E-06	SUCO	0.84093447	1.4954E-09
TOR2A	-1.04990961	1.551E-07	FAM13A	0.83995137	0.00304144
FAM120A	-1.04957634	1.2874E-08	SLC12A2	0.83900062	0.00491795
RSPH3	-1.04760447	0.01437551	STAT5A	0.83854442	6.6807E-07
NDUFA4	-1.04677111	2.9795E-27	H2AZ1	0.83845152	8.6827E-13
XXYLT1	-1.04640207	0.0056311	CLCN5	0.83801505	0.00189893
MAGOH	-1.04617678	9.1141E-09	SYNGR2	0.8370837	3.457E-05
CDIP1	-1.04549849	0.02062735	OTULIN	0.8370564	9.9727E-09
COA5	-1.0454047	0.00017939	FYTTD1	0.8362796	1.2681E-18
PRDX1	-1.04360996	5.5277E-14	NFKB2	0.83541615	3.6121E-08
TGM2	-1.04255563	0.02616245	LPP	0.83453479	1.5393E-06
INPP4A	-1.04248769	0.00075531	GPX4	0.83444302	2.1739E-05
CALR	-1.04228187	1.1399E-20	SOS2	0.83298235	7.9658E-08
HIF1A	-1.04207833	2.0764E-19	STAT5B	0.83279153	1.1518E-06
SYNJ2	-1.04189254	0.00377872	DDHD2	0.83275273	0.00504012
POLR3H	-1.04131538	0.00661883	SELPLG	0.83249108	9.9659E-06
TXNIP	-1.0410255	0.00046437	FOXJ3	0.83034768	0.00034175
GSAP	-1.04026884	2.4123E-05	MTMR12	0.82987623	0.00035077
KLF9	-1.03990454	1.1078E-09	TBL1XR1	0.8291388	9.7809E-06
ARL5A	-1.03928894	6.2957E-09	FARS2	0.82828766	0.001044
ITPK1	-1.03877251	2.1325E-06	SLC15A3	0.82736007	2.5988E-06
HAPLN3	-1.03853541	2.5025E-06	ATRN	0.82652972	0.00580176
NDUFAB1	-1.03845271	6.5644E-08	TAB2	0.82542082	1.7416E-13
ACAA2	-1.0381995	9.9351E-05	TNFAIP2	0.82370533	0.00398357
DDIT4	-1.03795543	6.2929E-06	CTBP2	0.82286882	0.0035624
TRAM1	-1.03720109	1.4799E-09	BTG1	0.82022805	1.8554E-05
TMEM175	-1.03674356	0.00278583	BCL2	0.8201836	0.00240209
MRPL22	-1.03668099	0.00085512	KATNBL1	0.81776596	2.1295E-05
CREB3L2	-1.03661812	0.03943034	ZNF136	0.81752779	0.03339457
WIPI1	-1.0358941	0.00122652	SGTB	0.81726704	0.02230696
HNRNPF	-1.03585429	1.0544E-15	FDPS	0.81675562	0.00463127
CERK	-1.03511168	2.8899E-05	MAP3K13	0.81635532	6.0001E-05
AP1S2	-1.03441179	7.9696E-06	JUP	0.81504582	0.01990375
ENSG0000028	-1.03390843	0.00078554	MYLIP	0.81487444	0.00463127
SNRPB	-1.03362635	7.4308E-11	MIER3	0.81363733	0.01417639
MAD1L1	-1.03360686	1.2923E-07	RAB29	0.81324125	5.459E-05
RHBDD2	-1.03317997	4.7606E-06	CRY2	0.81188809	0.02592177
MMP9	-1.03317445	0.02243614	HTRA2	0.81142385	0.02481616
LMAN2	-1.03238517	1.0725E-10	HMGCR	0.80951572	0.00111827
SNHG16	-1.03207498	2.0668E-08	ASAP2	0.80860083	0.00992178

PPIE	-1.03168847	0.01002841	FCHO2	0.80839753	0.03622163
CYBC1	-1.03143877	3.4116E-07	PTK2B	0.80699042	6.6561E-05
HES4	-1.03139271	7.9438E-06	CCPG1	0.80683965	0.00015092
GRPEL1	-1.03125012	0.00032662	BHLHE40-AS1	0.80660248	0.04066228
MRPS30	-1.03069891	0.00738653	BCAT1	0.8056455	0.00977456
SRFBP1	-1.03015621	0.00261986	TMEM41B	0.80553672	1.1862E-05
UNC50	-1.03008876	0.00206453	SLC35A5	0.80502248	0.00424621
GNL1	-1.02924846	0.0001769	BAZ2B	0.80470661	5.9763E-05
GCLC	-1.0292057	0.00984137	ENSG0000028	0.80394264	0.01451302
KCTD5	-1.02863739	0.01230141	HCP5	0.80372954	0.00060863
PPFIBP2	-1.02821916	0.00344997	TBC1D2B	0.80161785	0.00215225
POMC	-1.02783329	0.0017485	HIPK1	0.80143876	7.8723E-05
AIMP2	-1.02743035	5.5088E-05	EPM2AIP1	0.79906779	0.00044731
CMTM7	-1.02741059	3.3498E-11	USP48	0.79868511	7.4014E-06
PRNP	-1.0269405	1.7092E-07	FASN	0.79813298	0.03999691
MRPL58	-1.02658685	0.00271488	ZEB2	0.79664341	5.6047E-05
RFX5-AS1	-1.02636403	0.01375227	ARGLU1	0.7953524	6.6156E-15
MRPL4	-1.02601368	6.456E-06	SERTAD2	0.7950576	2.2436E-05
MAPRE2	-1.02557889	3.1057E-05	NSUN7	0.79503731	0.00256241
STAC3	-1.02520066	0.00050055	USP47	0.79344737	2.9334E-09
SAT1	-1.02507583	4.1316E-12	CYP51A1	0.79039695	0.00018968
HLA-DRA	-1.02498024	0.00062342	MED13	0.78984364	1.0885E-08
ZDHHC4	-1.02478295	0.01239328	SLC25A29	0.78871733	0.00117179
ARHGAP15	-1.02418565	0.00018221	PITPNB	0.78833627	7.9201E-09
SEC13	-1.02366385	2.593E-12	TLE1	0.78586139	0.00761689
PLPP5	-1.02283338	0.00143772	SLCO3A1	0.78538848	0.00353475
SEC24D	-1.02236025	1.4928E-06	CBFA2T2	0.78396744	0.00286615
MOB3B	-1.0213652	0.00122778	NUP98	0.78381586	4.9845E-09
PSMB10	-1.02114746	2.8112E-15	TTBK2	0.78333863	0.02086784
CMC1	-1.02113589	4.5202E-05	FBXL17	0.78151167	0.00155703
SCNM1	-1.0207948	2.6299E-10	PRPF39	0.78145656	0.00958156
FUNDC2	-1.02033036	2.4495E-06	RAB11FIP1	0.78108648	1.6926E-05
MRPS25	-1.01981398	6.6594E-05	SIRPA	0.78021971	0.00049777
LMO4	-1.01854958	1.9492E-05	HBS1L	0.78018724	0.00128836
LAMTOR1	-1.01851263	2.3887E-15	GFPT1	0.77906365	6.6045E-06
DDIT3	-1.01735089	0.00016993	CENATAC	0.77891873	0.04552563
CSK	-1.01725548	5.6006E-10	NCOA3	0.77827025	2.624E-06
CCDC112	-1.01676731	0.00836809	TDRD7	0.77825777	0.0002738
GPR171	-1.01646638	0.01304057	PKN2	0.77819131	3.8114E-07
MCOLN1	-1.01457353	0.00887668	ABI1	0.77812591	4.4681E-12
TXN2	-1.01448524	5.4743E-12	PBRM1	0.77811952	7.9652E-10
SRSF9	-1.01404494	7.1602E-15	COMMD2	0.77808509	0.00107659
CTSD	-1.01401689	0.00137221	GPHN	0.77647246	0.00068909
FLYWCH2	-1.01369165	0.00091082	PNRC1	0.77613292	0.00015821
MTDH	-1.01358592	3.6216E-17	MOV10	0.77467051	0.00475025
NPHP4	-1.01323946	0.00931561	NAP1L1	0.77300374	1.1438E-13

PARM1	-1.01294813	0.03277543	DHDDS	0.77248519	0.01715637
ABL1	-1.01292168	0.00982891	WHAMM	0.77061041	0.00359213
SNRPE	-1.0127693	1.8239E-06	TMEM131	0.76642069	2.7942E-08
DNMBP	-1.01276409	0.00074133	FAS	0.76517099	3.7386E-05
PAPSS2	-1.01264171	0.00903405	SPIB	0.7640793	0.0283199
MTM1	-1.01260217	0.02196482	BET1	0.76390669	0.00223304
MRPL17	-1.01197969	0.0024564	WTAP	0.76346177	9.5381E-06
ARHGEF6	-1.01138852	2.3662E-05	WBP11	0.7631339	2.8413E-06
COPS6	-1.01028149	2.9397E-08	TTYH2	0.76273375	4.4182E-06
CCT2	-1.00982411	4.6169E-08	ALAS1	0.76228703	0.000941
FUCA2	-1.00953066	2.9424E-06	HLA-F	0.76216823	1.1755E-06
YDJC	-1.00894135	5.6624E-06	NSMCE2	0.76207162	0.00309435
PSMD3	-1.00779051	1.0717E-10	XPO6	0.76201248	0.00651855
CCNT1	-1.00774295	0.0004437	BRCA2	0.76184982	0.00460714
LPIN1	-1.00770458	0.00271488	PIAS2	0.76164872	0.00085292
TEP1	-1.00747159	0.03671723	HACD2	0.75733397	0.00685111
SFXN1	-1.00745271	0.04287896	TRADD	0.75579721	2.721E-05
TOMM40	-1.00738831	2.9816E-05	WASL	0.75568008	0.00079147
NCKAP5L	-1.00584708	0.01122653	MED12L	0.7514345	0.0475066
WDFY2	-1.00570405	0.0061583	SUZ12	0.75133897	0.00431235
HMGB1	-1.00560834	1.1082E-18	CEP128	0.74875908	0.03285747
THAP9-AS1	-1.00467446	0.00124482	NCOA7	0.74858778	0.00024321
UBASH3B	-1.00432182	0.00318156	PLEKHF2	0.7467441	0.00133421
MRPS18C	-1.00425593	5.0351E-05	CD46	0.74595464	1.3113E-05
HS3ST3B1	-1.0042298	0.00378782	PDE7A	0.74482547	2.0965E-07
NMI	-1.00405992	2.2983E-09	NCKAP1L	0.74435555	5.4301E-09
TMEM250	-1.00398683	0.01967969	RGS1	0.74366218	0.00075249
UQCRQ	-1.00377824	3.5309E-11	SOX4	0.74196711	0.00588002
PSMB6	-1.00308393	1.0364E-12	DUSP22	0.74163852	7.3243E-06
IL10RB	-1.00270508	1.5769E-06	EPB41L2	0.7416322	0.00163375
BEST1	-1.002361	0.00018357	MTMR1	0.74161478	0.00206953
NDEL1	-1.00232832	1.2513E-15	HDX	0.74102873	0.01708373
MTPN	-1.00169967	6.1379E-10	GTF2E2	0.74046135	2.1681E-06
EXOC3	-1.00168094	1.2891E-06	CEBPG	0.73984915	1.2112E-05
NECAP1	-1.00167981	0.0027526	SNX14	0.73893533	9.5942E-05
CENPX	-1.00001072	5.6948E-06	ACAT2	0.73794958	0.00867913
RUVBL2	-0.9995007	0.00276551	MARCHF7	0.73555696	4.0744E-07
IFI30	-0.99916378	9.6275E-05	RUNX2	0.73373712	0.01478039
MRPL52	-0.99890234	1.0106E-13	DIDO1	0.73222804	0.00287411
LSM6	-0.99841875	3.1057E-05	KLF6	0.73149116	0.00238162
CPD	-0.99764642	0.00021053	RNF168	0.7314271	9.2523E-07
GSTM1	-0.99744456	0.03609803	EXT1	0.73049856	0.01709517
SH2D3C	-0.99450457	0.03288104	SRD5A1	0.73014763	0.00030726
H3-3B	-0.99421011	1.8045E-17	ENSG0000029	0.73007758	8.7381E-07
LAMTOR4	-0.99312834	1.2263E-20	RAB11A	0.72905474	5.1802E-06
PCK2	-0.99247348	0.02502675	CCDC50	0.72735667	3.0418E-06

SCFD2	-0.99175964	0.00065307	CD80	0.72529655	0.00218438
JUNB	-0.9911285	0.0004613	ENSG0000029	0.72452446	0.00022828
TXNDC17	-0.99069199	1.3279E-07	BZW1	0.72360711	7.1241E-08
GTF2A2	-0.99015077	4.8526E-07	TSC22D2	0.72327367	4.2676E-05
MANF	-0.98965249	1.0571E-07	STRN3	0.72311555	0.00034948
SLC25A40	-0.98931592	0.01376097	SMURF2	0.72299904	0.00038277
AZI2	-0.98912096	0.00081553	BTBD9	0.72293036	7.0514E-06
MIDN	-0.98894306	2.1648E-07	GPR137B	0.72138103	0.00962177
ATP5F1C	-0.98891546	8.7958E-14	ZZEF1	0.72090428	0.00053362
B4GALT7	-0.98835704	0.02714072	RASGRP3	0.71768537	0.00489949
ARMC10	-0.98803008	0.00058653	RBMXL1	0.71697165	0.01861732
TKT	-0.98728827	5.0844E-13	EOLA2	0.71591991	0.00051327
COX5B	-0.98711583	6.4902E-12	KIF13B	0.71522137	9.9636E-05
UQCRFS1	-0.9862491	3.6785E-13	ZNF581	0.71367058	0.00606275
PIK3CG	-0.98618127	0.03809482	RNF111	0.7127911	2.0747E-05
SLC35A4	-0.98589845	0.01053949	KLHL24	0.71175739	0.00027133
MTHFD1L	-0.98560714	0.00328591	ZFAND3	0.71153472	1.1234E-07
CHMP7	-0.98531479	0.01224656	MIIP	0.71132419	0.00192522
GUSB	-0.98519017	5.9447E-06	IKZF1	0.71109995	0.00140618
INPP5D	-0.9850495	1.83E-05	DLGAP4	0.7108999	5.7559E-06
HDAC3	-0.98413494	3.4449E-07	NEMP1	0.71088095	0.04655122
SEL1L	-0.98337982	0.00015063	PPP3CC	0.70938061	1.692E-06
URGCP	-0.98326438	0.04080895	SIAH1	0.70901464	0.0011466
BROX	-0.98293446	1.4341E-06	PPM1B	0.70715326	0.00012256
RAB32	-0.98273553	5.7253E-07	DCP1A	0.70696852	1.0865E-05
SUPT4H1	-0.98262703	8.256E-18	NELFB	0.70534665	0.00012548
NDUFA9	-0.98243253	2.9716E-07	CHML	0.70429815	0.0133382
IMP4	-0.98104468	8.2302E-07	ZNG1B	0.70396128	1.6743E-05
CASP9	-0.98038647	0.00094811	BCL2L11	0.70392046	2.4376E-05
RAB5A	-0.98005666	1.5088E-08	RASGRP2	0.70384513	0.01823158
FURIN	-0.97988808	0.00733089	SUMF1	0.70384323	6.9554E-07
TRABD	-0.97853561	9.5836E-08	WDTC1	0.70342345	0.02879668
PSMB8	-0.97830372	3.4467E-11	APPL1	0.70313887	2.4595E-09
TRIM21	-0.97797108	0.0042944	CTSC	0.70254159	0.00043345
SEC62	-0.97781978	5.9619E-08	CFLAR	0.70174708	1.5582E-05
RGS10	-0.97661852	6.8432E-14	ASMTL	0.70061603	5.0253E-05
ENSG0000027	-0.97648171	0.03552197	NKTR	0.70061085	1.9587E-06
LAT2	-0.97596091	1.6606E-06	LCOR	0.70046308	0.00391935
GPCPD1	-0.97574356	6.1908E-16	ENSG0000025	0.69751626	0.01429081
PARL	-0.97440385	3.1489E-06	RFX5	0.69658266	0.0002211
DUS3L	-0.97418183	0.00065052	RAP1B	0.69530748	1.6359E-05
AHR	-0.97378794	6.1495E-07	FOXK1	0.69508026	0.01349241
RRP9	-0.97294243	0.02464941	POLD3	0.69489843	0.04357943
RRP1B	-0.97219091	0.01582453	C5AR1	0.69440599	0.02865228
PSMB8-AS1	-0.9721182	0.00056583	PPTC7	0.69392935	0.00079725
CIAO2B	-0.97208091	1.8208E-08	CPEB4	0.6928971	0.00172091

NDUFA5	-0.97177294	0.00018663	PTBP2	0.69259926	0.00197817
NDUFC2	-0.97155555	7.0125E-11	BZW2	0.69206933	2.4357E-05
LRRC59	-0.97088368	6.5421E-08	RIPK2-DT	0.69028827	0.00035733
BCCIP	-0.97049238	3.0512E-06	ZBTB17	0.68991272	0.00501168
SNX3	-0.96993182	2.2307E-10	STRADA	0.68989507	0.04287896
EYA3	-0.9699203	3.339E-05	AFTPH	0.68957674	2.2317E-07
CTNS	-0.96931719	0.02161912	KDM4B	0.68567128	0.00275428
M6PR	-0.96900773	8.1233E-08	MED4	0.68535514	9.8556E-05
ZCWPW2	-0.96779376	0.0493698	TBC1D9B	0.68512537	2.0111E-05
UQCC5	-0.96772555	0.00709986	HLX	0.68474912	0.0193665
CCR6	-0.96722138	0.04483681	PPDPF	0.68465189	3.7861E-06
RRP15	-0.96647513	0.04194671	POLR2B	0.68405539	0.00010914
MPHOSPH10	-0.96607707	0.01651822	GRHPR	0.68311122	0.00010244
ISG20	-0.96556103	2.7865E-06	TRAPPC10	0.68303122	1.6864E-06
ENSG0000029	-0.96524941	0.00589976	ETV6	0.68258185	0.00019509
COMMD10	-0.96520936	3.5927E-07	WDR6	0.68150636	0.01735314
NDUFS7	-0.96462137	7.0193E-15	AAK1	0.68143124	0.00400141
HERPUD1	-0.96401274	7.9338E-16	ZFAND6	0.67819432	4.648E-06
ENSG0000026	-0.96384362	0.01959466	SLC5A3	0.67782785	0.02665986
PTPMT1	-0.96151767	1.585E-05	NIT2	0.67727785	0.00030838
CCDC57	-0.96031185	0.0050431	CD40	0.67677117	0.02178699
DNAJC4	-0.95966579	0.00035157	HMGN3	0.67566453	3.6284E-05
SIGIRR	-0.95965997	0.00083735	ULK4	0.67439922	0.02049634
METTL5	-0.95961569	0.00764216	TRIP4	0.67413892	0.02116838
SLC39A3	-0.95860441	0.00238382	PPARA	0.67405223	0.01347588
LSM3	-0.9585303	4.7064E-06	SBF2	0.67396098	4.3309E-05
ANKRD37	-0.95814457	1.209E-07	IL4R	0.67341711	0.00011691
UBE2M	-0.95809595	3.6268E-09	CAB39	0.67339716	1.0778E-05
ENSG0000028	-0.95692539	0.01111865	AMDHD2	0.67295249	0.01593968
LINC02245	-0.95663243	0.001897	GATAD2B	0.6721919	9.2486E-07
ABHD2	-0.9557458	0.00055527	HPCAL1	0.67070561	0.00516029
SLC1A4	-0.9549309	0.00012147	MB21D2	0.66897962	0.00381252
TMBIM4	-0.95395036	2.7713E-14	BCL7C	0.66863578	0.00010557
WDR77	-0.95391386	0.00885944	ABTB1	0.66774634	0.01148648
EEFSEC	-0.95348227	0.0024862	SRPK2	0.66763239	0.00021082
RNF41	-0.95316699	0.00044641	PPP1R2	0.66576237	2.4265E-10
AP2S1	-0.95246803	2.8923E-14	CCM2	0.66570353	9.7791E-06
YWHAB	-0.95234449	1.1146E-13	IPO9	0.66470433	0.04227548
BUD23	-0.95126436	0.00017277	KANSL1L	0.66460034	0.03966457
RPS4Y1	-0.95010958	5.6498E-10	FBXO11	0.66351098	0.00719865
SNRPD1	-0.94900895	1.5271E-07	MTHFS	0.66304508	0.00020366
ATP6V0B	-0.94805587	1.6405E-13	FNTA	0.66281635	7.7763E-06
SNAPC1	-0.9478171	0.04289467	TAOK3	0.66229547	0.000137
TBC1D14	-0.94626181	0.00309435	TNFAIP1	0.66204326	0.04094382
S100A6	-0.94588164	0.00602343	ILKAP	0.66182465	0.00019137
PSMC2	-0.94568428	1.0259E-06	TANK	0.66057129	0.00330634

NR4A2	-0.94363411	0.00475088	VPS37C	0.66025715	0.00666069
HAAO	-0.94342226	0.00710637	TELO2	0.65911814	0.01523188
SNRPG	-0.94340382	2.7314E-17	HSH2D	0.65902509	0.00286438
GLRX5	-0.94286978	8.0796E-06	NCBP1	0.65885624	0.02167453
UQCRC1	-0.94220389	7.236E-09	MED21	0.65844663	0.01252097
COX7A2	-0.94214613	7.6432E-11	RNASEH2B	0.65713099	0.00042242
SDHC	-0.94173955	2.2081E-09	NFKB1	0.65693982	0.00098922
CDK4	-0.94172125	0.00154745	ARHGAP25	0.65652284	0.00280434
ZNF672	-0.9416406	8.1668E-05	ETV3	0.6555461	0.00031301
TMEM51	-0.94163517	0.00027872	RANBP9	0.65385516	0.0257432
SUN2	-0.94160076	0.00010225	ZFAND5	0.65336584	0.00048004
POLR3GL	-0.94075938	0.0010966	ADIPOR2	0.65247957	0.00386661
SLX9	-0.94070211	0.02595419	TMEM87B	0.65202819	0.00576243
RILP	-0.94067009	0.00117824	GINM1	0.65057487	0.00017517
TST	-0.94042613	0.00334603	DDX6	0.65021548	5.3137E-09
IPMK	-0.939553	0.00011564	ANKRD44	0.65001138	0.00194965
ELOF1	-0.9395353	0.00043047	ATG10	0.64956284	0.02092334
GPN3	-0.93887769	0.00573445	CCDC82	0.648919	0.00566506
BCKDHA	-0.93841649	1.4343E-05	RSRC1	0.64837484	0.00149105
USB1	-0.93835374	4.5887E-06	CAMKK2	0.64807359	0.02471468
SCP2	-0.93811328	3.4855E-09	UBN2	0.64805091	0.00334685
NCF4	-0.93721125	5.2363E-05	HSPH1	0.64351062	0.00011712
METTL23	-0.93682766	0.00050246	SMAD2	0.64185356	0.00017879
HMGB2	-0.93667858	3.5239E-07	HECTD4	0.64161864	0.00146445
NDUFB10	-0.93661648	3.5042E-06	TMBIM1	0.64081028	0.02844207
AGPAT2	-0.93626628	0.00672719	KYNU	0.6393964	0.00092461
ATP6V0C	-0.93443158	2.2328E-21	WWP1	0.63833228	0.01026855
IARS1	-0.93395689	0.00149301	TRIM33	0.63570758	0.00073787
DPM3	-0.93388082	9.2302E-06	SLC30A7	0.63534267	0.00658203
PES1	-0.93305768	0.00617647	ATXN2	0.63520892	0.00655306
ASCC2	-0.93217426	4.3504E-06	DHX40	0.63517683	0.04597907
C12orf4	-0.93178403	0.03694428	EED	0.63473312	0.00295627
PTTG1	-0.9316465	9.6436E-06	CD58	0.63422512	0.00302351
MIOS	-0.93110407	0.00449682	RAPGEF1	0.63406461	0.00280496
E2F4	-0.93108656	2.0745E-07	TMPO	0.63363701	0.01876854
DHRS4	-0.93055815	0.00013161	ATAD2B	0.63295792	6.0441E-07
SLC12A2-DT	-0.93049834	0.03353041	RBM19	0.63288288	0.02653897
ENSG0000028	-0.93044832	0.03600948	TM7SF3	0.63197093	0.03429118
STX11	-0.92996866	2.1759E-08	TIMMDC1	0.6318202	0.01524065
EAF1	-0.92970492	0.00221047	PDLIM5	0.63148191	0.0001445
B4GALT1	-0.92937231	1.6768E-09	MAN2A1	0.63070532	0.00818822
PDCD6	-0.92936725	1.2112E-05	PTPRJ	0.63050057	0.01431659
BANF1	-0.92920809	2.1104E-07	ZBTB46	0.63042146	0.0006758
PSMA5	-0.92853319	3.1482E-16	APBA3	0.63016888	0.03437785
MRPL11	-0.92742969	1.5651E-06	WDR59	0.62983849	0.00854223
AURKAIP1	-0.92712272	1.1752E-06	TCF12	0.62944054	0.00025788

MRPS22	-0.92681271	0.00420279	ITPRID2	0.62843663	0.00010363
LINC00667	-0.92644325	0.0051622	TNS3	0.6283181	0.00357217
GMPPA	-0.92593447	0.00173454	WDR33	0.62692865	1.2686E-07
MRPL50	-0.92478992	0.00405582	CHFR	0.62660543	0.00140595
ATP10D	-0.92415653	0.03756814	VPS13B	0.62609125	4.7159E-05
NCOA4	-0.92365307	4.4837E-09	TFRC	0.62574201	0.01080805
ADSL	-0.92346837	0.00019743	EMC2	0.62530112	0.0250831
NKAPD1	-0.92315718	0.02559191	TNIP2	0.62433393	0.00157499
UQCR10	-0.92296761	9.8157E-17	ECPAS	0.62325612	2.9561E-05
MRPL21	-0.92231051	3.7124E-06	LMBRD1	0.62190952	5.0384E-06
LITAF	-0.92210264	1.8332E-10	EPSTI1	0.62070348	3.0991E-05
DENND10	-0.92073041	0.00023977	AMFR	0.62054271	0.00575645
SP100	-0.91991622	1.1442E-09	FAM172A	0.62035553	0.00509546
NBR1	-0.9197707	0.00212727	PDS5A	0.61929365	5.7088E-08
NDUFA2	-0.91892144	7.4183E-11	TP53BP2	0.6186336	0.00169311
RNASEH2C	-0.91863981	2.2529E-05	ARSB	0.61835264	0.00062061
AARSD1	-0.91836758	0.01178567	JOSD1	0.61828597	0.00037478
CD37	-0.91767675	2.2318E-05	HIPK3	0.61622975	0.00111827
ELOC	-0.91762629	2.738E-10	TMCC1	0.61613258	0.02120637
ANKRD22	-0.91742603	3.98E-07	CAMLG	0.61605477	4.7866E-05
SPTSSA	-0.91703069	8.2005E-05	NOA1	0.6159278	0.02940896
SYNJ1	-0.91688033	0.00967113	ATG7	0.61460801	0.02698075
ACADM	-0.91674903	0.0012595	TRIP12	0.61397892	1.5528E-06
SH3GL1	-0.91666817	1.3323E-05	KANSL1	0.61352962	0.00146445
PTEN	-0.91471171	5.6006E-10	TRIM8	0.61291788	0.00927704
USP15	-0.91332007	1.3806E-07	MAPK8IP3	0.61266311	0.01469105
RNF7	-0.9130073	8.0544E-09	WBP1	0.61252109	0.00033242
NTMT1	-0.9129661	0.0002083	CHKB	0.61080981	8.3364E-06
TCERG1	-0.91280147	1.9311E-07	ZSWIM6	0.61079373	0.00020184
REEP5	-0.91272374	5.4689E-07	HERC4	0.60821707	0.00220379
SNHG17	-0.9123202	0.00159256	NR2C1	0.6070241	0.0282785
GNB2	-0.9119484	1.0878E-10	PRR14	0.60669716	0.01474858
RPN1	-0.91155535	6.5644E-08	CDK8	0.60616425	0.01230928
PRDX5	-0.91128373	1.906E-10	ENSG0000028	0.60546851	0.01403117
GNB5	-0.91061338	0.00617621	G3BP1	0.60316144	0.00029656
BSCL2	-0.90959759	0.0030769	RELCH	0.60292385	0.02283859
DDX18	-0.90774573	6.2996E-06	PRKAG2	0.60288351	0.00767538
CD302	-0.90688496	0.01102344	SRSF11	0.60270584	1.4157E-07
SLIRP	-0.90641018	5.7403E-06	TFG	0.60265482	3.516E-06
NOC4L	-0.90537056	0.02117167	TMEM218	0.60244874	0.02105897
ZNF330	-0.90521947	0.0002251	N4BP2L2	0.60184356	1.1015E-05
VPS18	-0.90451392	0.02566682	STK3	0.60170429	0.0083873
C19orf38	-0.90445907	0.00070478	ORAI1	0.59823137	0.00227823
STK16	-0.90437583	0.01769907	RC3H2	0.59698613	0.00134554
PIN4	-0.90429357	5.9706E-05	KCMF1	0.59632133	1.3267E-05
UTP11	-0.90408795	0.00627667	ARID4A	0.59593716	0.00166367

NDUFA6	-0.90376589	8.8161E-08	PAM	0.59398711	0.04069119
NSMF	-0.90361167	0.0022758	USP34	0.59345622	7.3662E-08
DCTPP1	-0.90189045	1.1738E-05	ZZZ3	0.59319959	0.0301333
NAPRT	-0.90139489	5.7853E-05	DDX42	0.59101574	0.00061963
MAMLD1	-0.90127721	0.00076508	BMI1	0.59060663	0.026128
MCTS1	-0.9006649	6.2231E-06	SPG7	0.58964274	0.00169162
ATP1B3	-0.90049925	3.4579E-05	ATL3	0.5888326	0.0213669
COMMD6	-0.90036769	3.9198E-12	ZNG1A	0.58841312	0.02595419
NOC2L	-0.90007866	0.00189304	DMTF1	0.5868084	0.01205684
FAM204A	-0.89988631	0.00020184	NEPRO	0.58652558	0.0023286
SELENOF	-0.89928897	1.5746E-07	LETMD1	0.58622266	0.01846185
PPP1CB	-0.89921506	2.5404E-08	MGAT5	0.58577158	0.01211476
BAG6	-0.89855269	1.5905E-06	DENND5B	0.58541042	0.0191839
MPHOSPH6	-0.89821108	0.00041629	OGT	0.5849752	0.00785628
CDC40	-0.89784528	7.8161E-06	WDR48	0.58382502	0.00157533
SLC35C2	-0.89769524	0.0003611	CLIC4	0.58320591	0.00143864
BRK1	-0.89659168	1.2157E-19	ATF4	0.58133704	1.4133E-07
SNX8	-0.89657748	1.7971E-05	TASOR2	0.58046565	0.01203563
PHF20	-0.89635145	7.9696E-06	STXBP3	0.57959378	0.00909828
BPNT2	-0.89632999	9.9501E-05	KHDC4	0.579497	0.025765
NT5C3B	-0.8957885	0.04014356	TGFBR1	0.57679329	0.00067424
PNPLA8	-0.89482589	2.4299E-05	ATOX1	0.57574183	0.00919499
PPP1R14B	-0.89474937	0.00014151	USP9X	0.57563437	4.1741E-05
TENT5A	-0.89472904	0.00138872	KCTD13	0.57421545	0.04142188
IFT57	-0.89330981	0.03689148	REPS1	0.57395251	0.03384219
ETF1	-0.89290354	8.2337E-11	ENSG0000029	0.57154647	0.00946963
MRPS15	-0.89276334	9.2361E-08	H2AC6	0.57139596	0.03938494
OCEL1	-0.89249623	0.03451146	TMEM14C	0.5701957	1.0813E-05
APH1A	-0.89247686	1.3336E-14	SHTN1	0.56860156	0.00071047
RANBP1	-0.89137658	1.0007E-06	SBNO1	0.56779389	7.902E-06
HSPA9	-0.89129021	3.1582E-11	DMXL1	0.5661605	0.00627367
GHITM	-0.89068131	4.3414E-10	PI4KB	0.56535988	0.00481687
CLCN3	-0.89032413	0.00080221	ZNF654	0.5649644	0.0415174
IK	-0.89004972	2.746E-05	DUSP4	0.56401697	0.00418667
SETD7	-0.88983784	0.00183975	GTF2A1	0.56226821	0.00927704
UST	-0.88955996	0.00025174	TBC1D1	0.55793024	5.8854E-06
PRKAG2-AS1	-0.88949864	0.00074772	SIK3	0.55738479	0.00959842
TAF13	-0.8891831	0.00035936	IST1	0.55492363	0.00018599
DDX39B	-0.8891607	3.7382E-12	TNFAIP8	0.55490302	0.00927932
NUDT22	-0.88901233	0.0015056	DERL1	0.55412579	0.00080339
DDOST	-0.88888616	3.1147E-11	PCMTD1	0.55367304	0.00226988
EXOSC4	-0.88884521	0.00604307	ENOX2	0.55257728	0.04698743
HLA-DPB1	-0.8871732	3.8171E-08	GAS5	0.55139622	0.00534226
APEH	-0.88715988	0.00388845	TTC17	0.55036305	0.00017237
TRIAP1	-0.88591001	0.02605401	SMARCA4	0.55024889	0.0036694
COPS5	-0.88568233	1.9519E-05	CHD2	0.54925718	8.7059E-05

PRCP	-0.88210466	2.9178E-07	TNIP1	0.5488305	0.00026591
ARMCX6	-0.88177405	0.0072552	ZMYM2	0.54882873	0.00632513
SNAP23	-0.88159928	0.00004986	CCDC88A	0.54798079	0.00049637
PLEK	-0.88158723	0.00091708	CBX5	0.5474041	0.02105212
TIGAR	-0.88078461	1.1343E-06	CUX1	0.54698537	0.00816214
GDAP2	-0.88019974	0.01016908	ATRX	0.5465903	2.6824E-07
PGGT1B	-0.88012396	0.0063563	KPNA1	0.5465197	0.00025662
ENSG0000028	-0.87978524	0.01632541	ECI1	0.54643351	0.00359213
ABHD6	-0.87939901	0.00279081	VPS41	0.54572021	0.0014192
SH3TC1	-0.87900308	3.937E-05	ZSWIM8	0.5455276	0.04481215
PIGS	-0.87861942	0.00868089	RAB10	0.5432064	9.0603E-05
PPAN	-0.87729902	0.02944137	PIK3C3	0.54256159	0.03886055
UBAP1	-0.87712687	0.00022582	BMPR2	0.54174191	0.00246507
GUK1	-0.87611759	1.1219E-13	SNHG7	0.5408259	0.01803599
TSPAN3	-0.87561268	5.8854E-06	UBE2H	0.54063264	0.00575645
HADH	-0.87548127	0.03641643	RRAS	0.53843099	0.01521188
HSPA5	-0.87547599	1.1328E-05	LARP4	0.53658794	0.00351493
EIF5A	-0.87493215	4.1363E-14	CDC42SE1	0.53540769	4.2912E-07
TRAF4	-0.8748317	0.00355338	TRABD2A	0.5343069	0.00416765
PDZD11	-0.87455567	0.00322741	WDR7	0.53394876	0.02179987
ABT1	-0.87448088	0.01642356	PNISR	0.53359216	0.0007207
FKBP4	-0.87274728	0.00847342	P2RX4	0.53320578	0.02451899
RPL26L1	-0.87260709	0.00124524	CCNT2	0.53261759	0.00039183
ADCY3	-0.87212942	0.00400825	POLR2A	0.53164847	0.00125517
EIF2S2	-0.87182502	4.5149E-06	CALM3	0.53094251	0.00124857
BLOC1S6	-0.8712889	2.1371E-09	PDS5B	0.53021704	0.00586235
GLRX3	-0.87096307	1.6356E-06	PFKL	0.52931971	0.00580145
P4HB	-0.87068237	1.0669E-08	RPAP3	0.52760641	0.03196191
TWF2	-0.8706772	8.0387E-06	USP33	0.52668388	0.00648309
GMPPB	-0.8703105	0.00179611	SLC35D2	0.52595987	0.02494385
PAK1	-0.86967389	1.9738E-07	ARL6IP1	0.52244387	4.6743E-06
RHEBL1	-0.86955202	0.04727969	CDR2	0.52210547	0.02628864
MON1B	-0.86932104	0.02753327	CCNYL1	0.51949807	0.01437379
BBX	-0.86926612	1.4085E-06	EXOC4	0.51896255	0.00027193
TPST2	-0.86914373	0.02492446	PPP3CB	0.51895019	0.00565078
HLA-DQA2	-0.86891178	4.2767E-07	SPINT1	0.51887143	0.00829139
OSTM1	-0.86826121	0.00037562	SLC25A36	0.51879593	0.00911077
FBXO9	-0.86809007	1.2388E-05	RSU1	0.51681323	0.00514981
GPAA1	-0.86776641	3.7351E-05	VEZT	0.51659179	0.01239328
TLN1	-0.86733819	2.051E-10	B4GALT5	0.51634096	0.00087181
DUSP2	-0.86707999	0.01508085	KPNB1	0.51584183	2.9561E-05
BNIP2	-0.86689483	8.9321E-10	RCOR3	0.5153863	0.04998571
OXR1	-0.8668246	0.00017599	PLEKHM2	0.51493945	0.01234237
SDE2	-0.86678082	2.8586E-06	ADD1	0.51467905	0.00015083
ECI2	-0.86677193	0.01436869	ERGIC2	0.51394926	0.0254464
CHD1	-0.86623279	4.1398E-10	NSF	0.51103734	0.00125507

WDR83OS	-0.86556669	3.9835E-11
GSTK1	-0.86533649	1.6767E-11
MAPK3	-0.86529308	0.00529442
IGFBP7	-0.86391837	0.01633909
ACSL1	-0.86375798	3.4752E-07
SLC4A2	-0.86373538	0.02190735
MSRB1	-0.86305899	0.00371826
ADIPOR1	-0.86293989	2.4367E-07
PSMD8	-0.86277761	1.2874E-08
CNDP2	-0.86275784	1.7012E-10
GNAI2	-0.86229344	4.2746E-28
ABL2	-0.86217714	0.00017663
TFDP1	-0.86173868	1.3857E-05
GDE1	-0.86094023	0.02881152
C9orf72	-0.86083127	1.2165E-05
USF1	-0.85949502	0.00108205
TOMM6	-0.85852889	2.339E-11
TXNL4A	-0.85847702	6.44E-11
ALDH16A1	-0.85843289	0.00020576
ADM	-0.85843029	0.01471121
RNF139	-0.85820519	2.1671E-05
POLR3E	-0.85768854	0.02206725
PUM3	-0.85658112	0.03035586
MAN2B1	-0.8564046	1.5631E-05
LSM5	-0.85633445	1.6163E-07
PSMB9	-0.85560359	3.0417E-12
KDM6B	-0.85450973	7.1137E-11
CSNK2B	-0.85335112	4.6862E-12
AIG1	-0.85132309	0.02167453
SLC35A1	-0.85042514	0.0108852
SIDT2	-0.85025824	0.00760742
CFAP410	-0.85018975	0.01201249
MSN	-0.85013914	5.7253E-07
CBL	-0.84949489	3.6609E-05
NDST2	-0.84946986	0.01321518
RLIM	-0.84858345	3.1099E-06
AKR7A2	-0.8479866	0.00661837
IDH3B	-0.84783732	0.00120752
SLC19A2	-0.84751377	0.00329983
TMEM179B	-0.84646878	3.0605E-09
ENTPD6	-0.84625301	0.00758003
FHOD1	-0.84623859	0.00776652
SGTA	-0.84585392	1.7537E-05
MRPL40	-0.84537545	0.00098299
ZNF580	-0.84474265	5.9763E-05
RHOB	-0.84454253	0.00511251
STX17	0.51045279	0.02616245
PMPCB	0.5103426	0.0048637
FBXL3	0.51007316	0.02478051
GTF2I	0.50923367	0.00515063
RNF114	0.50913183	0.02093532
MCOLN2	0.50729458	0.0168313
NPLOC4	0.50713078	0.00106261
LINC00513	0.50672618	0.04020419
FAM13B	0.50637331	0.00019698
HOOK3	0.50582044	5.5873E-05
BACH1	0.5056138	0.00283744
PARP1	0.50480412	0.01401696
TNRC6A	0.50441669	0.02369195
RBM33	0.50229834	0.00064055
IL10RA	0.50213803	0.00227872
KAT7	0.50123828	0.04024069
ESYT2	0.50105568	0.01726555
IL6ST	0.50020803	0.00392096
APOBEC3G	0.49909114	0.00649861
PHC3	0.49798967	0.02418036
RUFY1	0.49747392	0.00073661
CNOT4	0.49676018	0.00021188
TRAPPC9	0.49513814	0.0113836
GNG7	0.49498759	0.02092369
SELENOT	0.49483318	0.0017645
CAND1	0.49459584	0.00300573
IER5	0.49406301	0.00496924
PPP1R12A	0.49182446	0.00181592
ZNF292	0.49005546	7.0281E-05
SENP5	0.48995662	0.00590141
IRF2	0.48884024	0.00911077
DCTN1	0.48773975	0.01585039
DUSP11	0.48677561	0.04294177
NR3C1	0.48639178	1.7748E-05
EZH2	0.48531819	0.00227823
KDM1A	0.48475208	0.03254895
MFSD14CP	0.4840911	0.01258507
REST	0.48356377	0.02595419
C17orf49	0.48277177	0.00049777
PHIP	0.4827676	0.01349241
TNRC6B	0.48173373	0.01219553
SIK2	0.48135043	0.00064612
IDS	0.48059163	0.00090471
WBP4	0.48043624	0.03580857
CYP20A1	0.47937723	0.01856212
N4BP2	0.47899441	0.01280612

KIF22	-0.84424577	0.00404508	AKAP13	0.47706298	0.00489021
KBTBD8	-0.84410921	0.00358378	NAP1L4	0.47410996	0.03353041
MPLKIP	-0.84384755	1.3738E-05	MGRN1	0.47390634	0.00127052
RERE	-0.84383941	4.0247E-05	UPF2	0.47360108	0.00366729
DDX41	-0.84365703	0.00266599	CDC42SE2	0.47342028	1.2545E-06
ZNF200	-0.84342618	0.02773554	ALDH2	0.47324423	0.02413671
EMC7	-0.84324406	7.651E-07	SEC61B	0.47240191	0.0017519
PNO1	-0.8428723	0.00063087	SLC20A1	0.4712438	0.03423145
PARP11	-0.8426997	0.00095352	LEMD3	0.47039329	0.01765949
SERPINB8	-0.84244683	0.04822094	LMNB1	0.470387	0.00524027
ZNF622	-0.84170992	0.01964878	GGNBP2	0.46940561	0.00312837
AKIRIN2	-0.84130408	4.0271E-11	KIAA2026	0.46851208	0.03462895
PPP6R1	-0.84086122	0.00061487	CDKN1A	0.46769743	0.03086685
L3MBTL2	-0.84011432	0.03999691	KDM3B	0.4676947	0.00970345
DDX49	-0.84006417	0.00014995	ERO1B	0.4672307	0.01020799
STK26	-0.83999119	5.9072E-05	NPEPPS	0.4662505	0.00297231
ALKBH5	-0.83946687	0.00017277	DLST	0.46595069	0.0396948
SLC16A10	-0.83940506	0.04726908	MECP2	0.46578741	0.0068048
HLA-DQA1	-0.83921495	0.00010847	NECTIN2	0.46535504	0.00018316
SAR1B	-0.83918024	0.00010699	TBCB	0.46525564	6.0382E-05
ATP5MJ	-0.8390051	1.0581E-06	USP3	0.46256272	0.0001178
NDUFA13	-0.83888304	1.6253E-09	RNF216	0.46218554	0.03524906
ATIC	-0.83813779	0.01029799	UBE3A	0.46217623	0.0001068
LGALS2	-0.83766664	0.00101766	MARCHF6	0.46209909	0.01496418
NRROS	-0.83617988	0.00084179	CD55	0.46165609	0.00023054
PLIN3	-0.83601454	1.1234E-07	DST	0.4613699	0.01381752
TSEN34	-0.8357975	3.7448E-06	ZNFX1	0.45971088	0.00491795
SAV1	-0.83574965	0.01658724	BLTP1	0.45840824	0.00589734
SEC23B	-0.83476701	2.0261E-08	EBF4	0.45743824	0.02834104
OXCT1	-0.83395318	0.01107773	VTI1A	0.4571991	0.00770753
SKI	-0.8333442	0.04090418	FAM193A	0.45693766	0.00714758
DGCR6L	-0.83287884	0.01117608	FOXO3	0.45427396	0.01006519
CUTA	-0.83193089	7.295E-08	IFRD1	0.4538039	0.04231986
SLC35B1	-0.8311937	8.4122E-06	IKBKG	0.4538024	0.00886548
SNX13	-0.83071737	0.00561059	TAOK1	0.45323021	0.01715568
POLD4	-0.83063386	2.359E-10	SAMD4B	0.45249666	0.01609476
LBR	-0.82959338	0.01273538	WSB2	0.45144647	0.02207667
CAPZA2	-0.82947069	1.6072E-09	CCNG1	0.45066813	0.03760234
NSUN6	-0.82930336	0.01298287	DLG1	0.44899579	0.04826291
ARPC5L	-0.8290767	2.6146E-05	RYBP	0.44861679	0.01778056
ERBIN	-0.82897163	5.4224E-06	RNF19B	0.44855118	0.01300211
MLXIP	-0.82731736	0.00491559	CLASP2	0.44812748	0.00516417
ATP5F1A	-0.82656873	9.7555E-11	RRAGC	0.44697167	0.01437379
GOT2	-0.82648151	0.01375227	CAST	0.44649754	4.4416E-05
TMA7	-0.82647058	5.7247E-18	MOGS	0.44529211	0.04142053
SERP1	-0.82634976	1.0168E-08	ARIH1	0.44298044	0.02367068

FAM89B	-0.82588864	0.0017215	UBR5	0.44265306	0.00045583
PPP1CA	-0.82575383	1.9861E-08	RALGAPA1	0.44246962	0.02722289
FASTKD2	-0.82485543	0.00607478	CYTH2	0.44218077	0.03999136
SCARB2	-0.82480273	0.00023279	DIS3	0.43925811	0.048579
PDP1	-0.82361128	0.00417381	GOSR1	0.43846524	0.02031766
EID2	-0.82304384	0.0178538	FDFT1	0.43802494	0.02847005
LDHA	-0.82279789	4.232E-09	HERPUD2	0.4379471	0.0173912
ZNF593	-0.82255662	0.02909091	RSF1	0.43764309	0.00194743
IRAK1	-0.82232533	0.00050727	R3HDM2	0.43729722	0.00291323
AAMP	-0.82082859	1.8384E-05	LASP1	0.43691237	0.01795707
ACOT8	-0.82031843	0.01922678	NUP153	0.43620605	0.03369112
NBDY	-0.8194716	1.1393E-08	WASHC2C	0.43455026	0.023729
POLR2L	-0.8193945	2.4708E-06	HNRNPH1	0.43420627	0.02099177
CNOT7	-0.8184662	2.6114E-06	STK17A	0.43394827	0.00660846
TCEA2	-0.81750817	0.04346674	H2AX	0.43055479	0.04685319
MCFD2	-0.81736073	0.00056086	MRPS6	0.42869734	0.04242735
TPD52L2	-0.81727515	0.00091708	RIPK1	0.42731792	0.02610465
POLB	-0.81706393	0.0075704	CUL1	0.42684281	0.01251429
RAB1B	-0.81699412	2.7478E-06	CIRBP	0.42591199	6.9059E-06
STARD3NL	-0.81664132	9.1207E-08	SRRM2	0.4252784	2.9432E-07
ARMC8	-0.81652131	0.00068505	ACOT9	0.42336529	0.00010137
GBP1	-0.81646334	9.8431E-09	ATXN7	0.42161826	0.00197795
ARF1	-0.81569126	2.6351E-12	STAG1	0.421121	0.00293988
GNS	-0.81503504	0.00031651	TXNL1	0.4197611	0.03390332
THOC3	-0.81484259	0.0179164	FAF1	0.41904624	0.01239328
TPI1	-0.81426144	3.1548E-11	CYSTM1	0.41900564	0.00510912
MIF	-0.8138592	1.6089E-08	ARHGAP17	0.41863816	0.03075313
IL3RA	-0.81279856	0.00023362	PPP2R5C	0.41846917	0.03194214
MXD1	-0.81268476	1.3959E-05	HEXIM1	0.41803973	0.04434331
ARL1	-0.81224501	0.01131245	CHORDC1	0.41748393	0.03171974
FAM174C	-0.81215982	5.3398E-05	SQSTM1	0.41688984	0.03974163
DTX2	-0.81167037	0.02382352	SNX9	0.41542	0.00424377
PSMA3	-0.81123866	1.3206E-08	PPP6R3	0.41346451	0.00275622
DGLUCY	-0.81106618	0.01630586	TUBB4B	0.4130929	0.039957
HSPBP1	-0.80984376	0.02939116	FBXW5	0.41249586	0.01818419
SPATS2L	-0.80967855	0.00539062	NOTCH2	0.40884047	0.02002693
MPV17	-0.80948386	1.692E-06	BRWD1	0.40873143	0.01627328
DEGS1	-0.80926633	8.0544E-09	EHMT1	0.40758207	0.0251482
TMEM243	-0.80924965	9.1134E-06	VPS13C	0.40654007	0.00086914
SLAMF7	-0.80914409	0.00179412	KLC1	0.40642342	0.0095735
EIF6	-0.80882451	2.7245E-08	RHOC	0.40215102	0.00652639
UBE2V2	-0.80812652	4.8664E-05	H1-10	0.39911622	0.00706275
ENSG0000028	-0.80770324	0.00866629	KRAS	0.39713952	0.0282785
STX17-DT	-0.8073256	0.00012951	KMT2C	0.39489243	0.01798717
NCBP2AS2	-0.80667761	0.00407399	IMPDH1	0.39471769	0.02395211
R3HCC1	-0.80559806	0.03042089	KIDINS220	0.3911451	0.04463999

PSMA7	-0.80464599	7.9755E-07
NSFL1C	-0.80452809	1.2374E-08
PSMB7	-0.80445353	1.6751E-06
MED30	-0.80424487	0.00109289
COA6	-0.80347728	0.00317392
BOLA2B	-0.80336921	0.03073432
TCEAL8	-0.80238635	0.03276442
CDK2AP1	-0.80205322	1.0628E-16
HTATSF1	-0.80174612	0.00064239
CD2AP	-0.80101635	3.9305E-05
PGAM1	-0.80082485	1.7985E-06
HNRNPAB	-0.80074344	1.2054E-06
C2CD5	-0.79941661	0.02907506
COX5A	-0.79883211	6.1784E-11
MED8	-0.79817372	0.00485399
BICD2	-0.7981385	2.3884E-05
TP53I13	-0.79799363	1.6359E-05
ELK4	-0.79723926	0.00219059
ENOPH1	-0.79718908	0.00996866
MRGBP	-0.79646492	0.01971073
SNRPB2	-0.79563703	0.00038216
DNAJC8	-0.79528456	4.6986E-09
PCBD1	-0.79431924	1.7537E-05
ATP5MF	-0.79427732	8.1196E-14
DOCK4	-0.79417168	0.00512005
CHRAC1	-0.79397829	0.00014222
HPS5	-0.79363908	1.9986E-07
ALYREF	-0.79360497	1.7651E-06
ACSL3	-0.7932663	9.7018E-06
TMEM214	-0.79299186	0.01526385
DAD1	-0.79274648	2.5204E-07
GADD45GIP1	-0.79229226	2.0956E-06
PSMC1	-0.79189206	9.1724E-05
ARHGAP27	-0.79103961	0.00621574
PFDN2	-0.79050607	0.00026552
ENSG0000023	-0.78889408	0.02199559
DNTTIP2	-0.78857278	0.00037179
SEC14L1	-0.78842567	1.4562E-08
SEPTIN6	-0.78804614	7.1963E-13
ZBTB7A	-0.78788815	2.4365E-05
HOTAIRM1	-0.78733543	0.00027208
DBF4	-0.78726201	0.03993444
MRPL24	-0.78704361	0.00334626
PIGT	-0.7863306	0.00082429
PARVB	-0.7862722	3.4564E-06
OAZ1	-0.78602126	2.0769E-09

ZNF644	0.39026575	0.0063489
ACTN1	0.3898826	0.0317087
ZFC3H1	0.38979819	0.02198971
AGFG1	0.38957927	0.00856277
PARP12	0.38943471	0.04395431
EIF4A2	0.38899226	0.01345116
RAD21	0.38835766	0.02644653
CLASRP	0.38541589	0.03460469
GAK	0.38536979	0.01820802
ITSN2	0.38153153	0.00703511
KDM2A	0.38054273	0.03066314
PCNX1	0.37940794	0.04787205
OSBPL9	0.37912057	0.04783825
ANKRD12	0.37737766	0.00170546
SF3A1	0.37578793	0.04658382
GSTP1	0.37489492	0.01512638
POGZ	0.37483133	0.04376587
GPATCH8	0.37371344	0.04583852
MED28	0.37366901	0.0252967
SBNO2	0.37135542	0.01682813
H2AJ	0.37043181	0.00625401
SMARCA5	0.36633166	0.00086165
TENT2	0.36607656	0.02991123
ITCH	0.36288652	0.01671592
UBR4	0.36168263	0.00820539
ACTN4	0.35765188	0.00280519
RSRP1	0.35674718	0.00052168
CYTIP	0.35500983	0.0112778
XPO1	0.35176989	0.04676019
SEC24B	0.34935053	0.02799021
HNRNPC	0.34929749	0.00522536
CHP1	0.34837854	0.04691132
PTPRA	0.34809988	0.04969968
ARL6IP5	0.34709556	0.02878384
PUM1	0.34668845	0.02179727
BTBD7	0.3428688	0.00606265
ARFGEF1	0.33411874	0.04479528
PUM2	0.33383019	0.02454003
ARHGAP45	0.33349272	0.01526385
LUC7L3	0.32951193	0.04491132
ZNF638	0.32916486	0.03664221
ELF1	0.32756157	0.0025142
UBE2F	0.32442557	0.03333962
PTMS	0.32271843	0.02433441
SFT2D1	0.32024287	0.00624117
SLK	0.31976933	0.03421037

GTF3C6	-0.78588177	7.6638E-07
GRIPAP1	-0.78568315	9.3777E-07
TIFA	-0.78338786	0.00032986
SCAMP2	-0.78300412	4.4099E-08
APOO	-0.78246965	0.01271614
JTB	-0.78148059	1.423E-06
MGMT	-0.78073838	0.0188697
CYCS	-0.78063334	6.4515E-12
EIF3I	-0.78049384	6.7391E-07
STOM	-0.7804142	0.00028783
BCL7B	-0.77942325	1.6359E-05
GPI	-0.7792614	2.5911E-06
SDCCAG8	-0.77872746	3.3816E-06
PSMD9	-0.77860549	0.00072798
KIAA0040	-0.77817467	0.02715184
GMPR2	-0.77801831	0.00533658
SLC31A2	-0.77783765	1.1021E-06
POLR2J	-0.77708262	0.00061963
INO80E	-0.77673688	0.00866389
PPM1M	-0.77555995	0.00093316
NOPCHAP1	-0.77523512	0.01042461
CAP1	-0.7748083	6.867E-11
SMIM15	-0.77448153	0.0062686
GABARAP	-0.77428952	1.5003E-12
LYSMD2	-0.77411761	5.383E-05
MRPL20	-0.77332454	3.5396E-07
ZNF503	-0.77297568	0.00350209
DDI2	-0.77226383	0.02382142
ERI3	-0.77216971	0.03941599
RRP7A	-0.77211314	4.0269E-05
AHCYL1	-0.77204805	0.00026897
PFDN5	-0.77136131	1.4137E-25
NGLY1	-0.7712151	3.5134E-08
PIP5K1C	-0.77043437	0.0013345
ANKRD13D	-0.77031745	0.01111798
KDM7A	-0.76938123	0.00138967
CHMP1B	-0.76920406	0.00261914
CA2	-0.76903803	0.00313912
PSME2	-0.76807686	3.621E-07
PLCG2	-0.76762318	0.00059397
DAGLB	-0.76650286	0.00959842
FBXO6	-0.76639307	2.4317E-07
TYMP	-0.766386	8.4281E-07
MAGT1	-0.76590126	5.9623E-05
PAFAH1B3	-0.76565698	0.01288724
RRM2B	-0.76536164	0.00460375

NSD1	0.31849692	0.01774619
VDAC2	0.31225789	0.02164931
HINT1	0.30190735	0.00022558
PNRC2	0.30098825	0.03364036
REL	0.29917935	0.02798792
TMEM50A	0.2991335	0.00259351
SELENOS	0.29538747	0.0476051
ACSL4	0.29068027	0.02819061
FMNL1	0.28830991	0.01503816
IQGAP1	0.26620779	0.00258858
PRPF40A	0.25663928	0.04676019
MT-ND2	0.25441808	0.01595445
MT-ND4	0.25252956	0.00990664
MT-ND3	0.25046473	0.01382925
MT-CYB	0.24584025	0.00296681
RAB2A	0.24196167	0.03655171
MT-ND4L	0.19149486	0.02151276

NUCB1	-0.7651103	2.2135E-06
NAMPT	-0.76494952	1.4305E-07
TRPT1	-0.76488652	0.00962887
COPE	-0.7648165	1.1402E-12
RNF187	-0.76432003	5.5406E-05
BOLA3	-0.76369993	0.00855831
RPL36A	-0.76327388	5.0042E-13
SKA2	-0.76325148	0.02257783
MRPL19	-0.76294209	0.04028037
PTTG1IP	-0.76267974	2.9001E-07
PRDX6	-0.761995	7.8641E-07
RABGGTA	-0.76065165	0.01595445
LINC01678	-0.75989635	0.04089051
PEX2	-0.75946492	0.00867336
UQCRH	-0.75920888	1.3938E-09
RNF24	-0.75918681	2.1709E-05
IQSEC1	-0.75867026	0.00081595
GAPDH	-0.75847652	2.7303E-11
ATP5F1B	-0.75815213	3.5677E-07
PREB	-0.75814145	0.00363358
APRT	-0.757597	6.8083E-09
AMZ2P1	-0.75716932	0.00345436
ARF4	-0.75700902	3.016E-08
MRPL36	-0.75670894	0.0112118
MTX1	-0.75621759	0.00447517
TMEM126B	-0.75586902	0.00952656
LEPROT	-0.7547738	0.02151276
SYK	-0.75443464	2.0686E-05
LGALS9	-0.7542196	5.1121E-08
YARS1	-0.75364584	0.00917251
VSIR	-0.75309418	0.00023039
COPS3	-0.75298137	7.3683E-05
DKC1	-0.75249828	0.01466484
NF2	-0.7524228	0.04227548
RPIA	-0.7522559	0.00326349
DEK	-0.75206484	6.9029E-09
H3-3A	-0.75190012	1.2344E-06
MRM2	-0.75123449	0.01009186
PRKAG1	-0.75084862	0.0153256
CHCHD10	-0.75060748	0.00023605
TMX4	-0.7502284	0.00018599
COA3	-0.74993577	0.00023652
CBX3	-0.74933605	8.7372E-07
TFE3	-0.7471293	0.0037589
CHMP2A	-0.74708085	1.0886E-09
SRP68	-0.74607547	0.02808527

CARHSP1	-0.74602464	0.01287419
MPST	-0.74597475	0.01112256
CORO1C	-0.74543253	0.00020474
TOMM22	-0.74486102	2.9762E-06
NDUFAF8	-0.74479563	0.00236677
CLN3	-0.7443029	0.00044641
IFNAR2	-0.74387673	3.3167E-06
PPP1R11	-0.74297937	4.3216E-06
SSRP1	-0.74269314	0.00031189
AKAP7	-0.74263502	0.04415452
POLR2G	-0.74227571	5.4467E-07
TM9SF1	-0.7422414	0.01173037
KRR1	-0.74214442	0.00153357
ACAP1	-0.74133044	0.00134791
EI24	-0.74074432	0.04013036
PHB1	-0.740626	0.00092237
EEF1B2	-0.74057532	1.0573E-07
SMIM30	-0.74021078	0.03053028
PUF60	-0.73996492	0.00011212
MAP3K11	-0.73947226	0.00025257
PFDN1	-0.73944901	2.7463E-05
SARAF	-0.7393331	8.6885E-07
STK17B	-0.73932753	0.00666069
ELP5	-0.73932024	0.00121361
CCT7	-0.73847977	1.024E-05
ATP5PO	-0.73825451	1.5828E-09
LRPAP1	-0.73799706	3.4744E-06
ATP5PD	-0.73794615	3.3978E-10
MFSD12	-0.73764493	4.1952E-06
DENND1A	-0.73655365	9.1271E-07
PSMA1	-0.73628319	8.1722E-10
C1D	-0.73625595	0.00021199
KLF10	-0.73621406	0.00164314
BTG2	-0.73618966	0.0015535
NINJ1	-0.73595085	0.00399314
IDH3G	-0.73545895	5.6097E-07
JMJD6	-0.73519981	0.00167548
FH	-0.73511975	0.00066575
LRR1	-0.73500351	0.01865384
MAZ	-0.73416281	9.2453E-05
PLEKHO1	-0.73413826	4.4358E-08
PLPBP	-0.73315789	0.00354757
ATP5F1D	-0.73287225	1.2805E-06
RIOK1	-0.73233622	0.00242923
RIPOR1	-0.73226759	0.01805474
COASY	-0.73196678	0.00927704

SUCLA2	-0.73152909	0.00790952
NSUN4	-0.73137413	0.02682421
SNF8	-0.7309804	1.1244E-06
ALG5	-0.72991432	0.02880363
DESI1	-0.72978434	1.7049E-05
TMEM50B	-0.72935122	0.00703355
RPS19BP1	-0.72898248	0.00025137
GNAI3	-0.72868313	8.1754E-09
CDC37	-0.72845312	8.3779E-10
AVPI1	-0.72772369	0.00793836
KIAA1143	-0.72748915	0.0239387
PSMC5	-0.72727212	0.00010838
WDR18	-0.7268928	0.00538866
RPL27	-0.72679352	3.3776E-12
IRAG2	-0.72664883	0.04668353
EIF2S1	-0.72597539	2.4775E-05
VPS25	-0.72534125	0.00872274
LAMTOR5	-0.72517698	1.2219E-07
ABCD1	-0.72434758	0.04133839
S100A10	-0.72376703	3.8961E-05
GIT2	-0.7235805	4.3588E-05
RBIS	-0.72328127	7.5942E-05
ARPC5	-0.72293047	1.7625E-10
ARMC6	-0.72220649	0.03410751
CDC123	-0.72173069	9.3679E-06
EBNA1BP2	-0.72133125	0.04584188
CSNK1G2	-0.72103236	1.7034E-06
MAILR	-0.72045915	0.0437653
AGPAT5	-0.71982548	0.04221326
HNRNPUL1	-0.71950231	1.9129E-10
IVNS1ABP	-0.71899594	0.01001444
TAPBPL	-0.71792524	0.0032785
NAXE	-0.71768952	0.00874049
CALM1	-0.71739017	1.1699E-06
CPNE3	-0.71672432	0.00040945
GLRX2	-0.7160786	0.04025381
CALU	-0.71552942	0.01438297
RBM42	-0.71466818	6.111E-05
PSMB1	-0.71443101	8.5277E-14
STX12	-0.71378223	1.6489E-07
RPL37A	-0.71301231	6.1835E-10
MTA2	-0.71286418	0.00288119
ZNHIT1	-0.71261289	1.7919E-05
MRPL23	-0.71258251	0.008134
CCS	-0.71224574	9.809E-05
IER3IP1	-0.71224217	0.00503086

PSMD13	-0.71162421	6.484E-07
STK40	-0.71156562	0.03371737
DDHD1	-0.71154846	0.00330703
XRN2	-0.70998756	4.2929E-07
ISCA1	-0.70981705	0.01721447
HAUS1	-0.70913077	0.03562838
AARS1	-0.70911973	0.02398453
EPS15L1	-0.70911361	0.03992339
ADRM1	-0.70880508	8.0529E-07
GEM	-0.70814209	0.04207615
CERS2	-0.70811074	0.01968465
NDUFS2	-0.70788175	5.1293E-05
YBX1	-0.70747456	6.607E-12
DUS1L	-0.70739347	0.00032715
PTS	-0.70734989	0.00059236
ABCA1	-0.70709135	0.03208602
SFT2D2	-0.7070578	0.00010577
HSD17B11	-0.70615199	0.04674521
LSM1	-0.70595314	0.0008698
POM121C	-0.70567965	0.00629829
ZCCHC10	-0.70501609	0.00779476
MRPS7	-0.70501079	0.00063409
HECTD1	-0.70463583	0.00013103
CNOT8	-0.70423334	0.00197966
NSMAF	-0.7031596	0.00068752
PPA1	-0.70310612	0.00025491
ZNF487	-0.70248614	0.03494848
PDHB	-0.70207717	0.00677126
COX14	-0.7014973	2.4572E-06
MTMR6	-0.70122615	0.02457416
GPS1	-0.70026894	0.00053965
GRB2	-0.70005434	2.2957E-15
EZR	-0.69966644	0.00013367
SYNCRIP	-0.69916551	3.471E-07
UBE2J2	-0.69813791	0.00050572
MPDU1	-0.69783305	0.00048149
FARSA	-0.69767727	0.0119288
RRP1	-0.69729544	0.01920633
STARD8	-0.69705608	0.00460714
IRF9	-0.69683507	0.00072596
AKT1	-0.69627681	0.0012159
POP4	-0.69599218	0.00652493
UBA5	-0.69495921	0.0300636
SGPL1	-0.69449993	0.01851688
GPR65	-0.69445684	1.7885E-05
GTF2H1	-0.69406071	0.00262898

DSE	-0.69367006	0.00454864
HEXD	-0.6928502	0.0105851
RBM8A	-0.6926879	6.1081E-06
MPC1	-0.69253235	5.7285E-08
SMIM26	-0.69214362	0.00011564
RPS11	-0.69200053	2.5669E-20
LINC02256.1	-0.69151417	0.02315414
RPL39	-0.69144247	1.6322E-07
TMED7	-0.69143626	1.4176E-05
ZFP36	-0.69115549	0.01887922
NDUFB1	-0.69092154	0.00020593
SEC61A1	-0.69066457	0.00453803
PDE4A	-0.6906011	0.00400825
TRAPPC2L	-0.69015412	0.01209248
C21orf91	-0.68961432	0.04101715
NDUFB2	-0.68929595	2.9553E-05
PPA2	-0.68914439	2.335E-05
COPS4	-0.68907065	0.04768108
CTDP1	-0.68898335	0.00084641
DNAJC3	-0.68881373	7.4828E-09
NUDT5	-0.68869877	0.00310675
RPS6KB2	-0.68732476	0.00246494
NIPSNAP2	-0.68716553	0.00093014
TMEM230	-0.68690278	5.4522E-07
TMED9	-0.68657183	3.206E-05
CNPY2	-0.68594044	0.00014402
CCDC69	-0.68436946	0.02403605
GRK2	-0.68391445	0.02048767
SMIM7	-0.68343084	0.00058678
MPP1	-0.68339504	0.01851904
SERF2	-0.68308772	6.5264E-07
RAD23A	-0.6830729	9.7431E-06
CREB3	-0.68299492	0.00593238
CHCHD2	-0.68230283	4.5376E-08
EMC4	-0.68151971	0.00013993
G6PD	-0.68145202	0.00161106
TPM4	-0.68134219	3.8311E-07
NSL1	-0.68107063	0.00955993
CD74	-0.68029803	0.01961117
ORMDL2	-0.67985367	1.2665E-05
PSMD6	-0.67877613	1.2822E-05
TM2D2	-0.67802616	0.02471105
BCAS2	-0.67681027	0.00147876
ESF1	-0.67643774	0.02803647
RPP21	-0.67574392	0.03729488
RGL1	-0.67550704	0.00693515

METAP2	-0.67546118	0.00103248
MYCBP	-0.67526096	0.02621201
TPP1	-0.6746661	1.318E-07
WASH6P	-0.67457815	0.0330483
PRKD3	-0.67435336	0.00624755
USP36	-0.67411248	0.00268955
MBD4	-0.67383611	0.00521864
TOLLIP	-0.67351551	0.00034171
LRRFIP1	-0.67349928	1.1818E-06
ATF1	-0.67348882	0.01002841
SMCHD1	-0.67195643	0.00718214
SERTAD1	-0.67189773	0.00147513
STT3A	-0.67157047	0.00253924
PHACTR4	-0.671167	0.00935487
POLR1F	-0.67083328	0.00714238
DNAJC2	-0.67011051	0.00577267
NEK9	-0.66997969	0.01093605
GGTA1	-0.66952652	0.00831944
CORO7	-0.6695069	7.0044E-05
EFCAB14	-0.66856993	0.00057807
TSC22D4	-0.66749221	0.00271764
CA5BP1	-0.66733341	0.03941599
CD164	-0.66727645	1.1508E-08
PRDX3	-0.66713467	2.5984E-05
UQCR11	-0.66709744	8.7208E-11
ARF6	-0.66699057	6.4184E-07
SCAMP3	-0.66698928	0.0018363
SRP19	-0.6657912	0.0005383
METTL26	-0.66560424	0.00128261
NEDD8	-0.66556688	1.8401E-08
DDX27	-0.6651887	0.00263811
HSD17B12	-0.66486647	0.00575645
RCOR1	-0.66478897	1.2916E-05
MICU1	-0.66466128	0.00320292
SS18L2	-0.66448835	0.00065387
PFDN6	-0.6644175	0.00286615
BLOC1S2	-0.66387531	0.00138872
STK10	-0.66342417	0.00015083
HSPE1	-0.66337228	0.00155076
SNRNP40	-0.66331815	0.00284907
CTLA4	-0.66265992	0.04675096
SRP9	-0.66182445	3.4133E-07
LSM12	-0.66166994	0.00089673
DOCK2	-0.66146235	8.5215E-05
SAP18	-0.661314	0.00018804
WSB1	-0.66114283	1.9146E-08

NDUFB11	-0.66094288	3.5469E-11
SMU1	-0.66086521	0.00071835
PTPN11	-0.66010025	0.00133421
EIF4A3	-0.65987204	0.000473
GATD3	-0.65953395	0.00067255
KRTCAP2	-0.65909987	2.5451E-05
RBPJ	-0.65854667	0.00083172
RPA3	-0.65850299	0.00300573
RPS18	-0.65843198	2.1722E-09
PSMA2	-0.65769773	1.3893E-07
PGP	-0.65709133	0.01005081
ZNRD2	-0.65587785	0.0065611
TALDO1	-0.65586072	4.1553E-05
RPL23A	-0.65528223	1.6891E-14
H1-3	-0.65493638	0.019777
UBA2	-0.6547784	0.00246023
SPIDR	-0.65455239	0.00230839
AIDA	-0.65436906	0.00158753
ARHGAP5	-0.65378068	1.7312E-05
VAMP7	-0.65374442	0.01644384
TRAPPC5	-0.65373059	8.8474E-06
WDR43	-0.65366116	0.00758305
COX6C	-0.653611	6.6796E-07
CYP2S1	-0.65333784	0.00044131
SSU72	-0.65271931	5.7636E-06
PTRHD1	-0.65234933	4.6358E-07
RNF181	-0.65228717	3.7901E-10
SPTY2D1	-0.65200978	0.02478051
RWDD4	-0.65188667	0.02528418
OCIAD1	-0.6518242	3.8946E-06
STX10	-0.65159761	0.03074029
HNRNPR	-0.65125078	1.6242E-05
LY96	-0.65043573	0.00156857
GFUS	-0.64962099	0.00652639
RPL35	-0.64900101	5.1146E-08
RPS13	-0.64829441	1.6375E-17
PPP4C	-0.64777576	1.6322E-07
ERLEC1	-0.64757291	0.01132895
MRFAP1	-0.64703283	0.00047144
GLO1	-0.64663675	0.0001558
IRF8	-0.64651599	0.00142655
HDGF	-0.64604778	1.3569E-05
DNPH1	-0.64601792	4.2004E-05
WAC-AS1	-0.64576165	0.02170135
TPRKB	-0.64553781	0.00477856
SNX27	-0.64523645	0.00187488

UCHL5	-0.6448351	0.02205825
PHTF1	-0.64474744	0.00236009
MRPS16	-0.64440296	0.0030588
GSK3A	-0.64434465	0.00314741
ACP1	-0.64416483	3.8396E-05
PSMD7	-0.64312609	1.2923E-07
MRPL54	-0.64264904	0.00026641
RCN2	-0.64198121	0.00286586
UBE2L3	-0.64189329	4.1176E-09
CAPZA1	-0.64172565	2.1282E-07
OTUD4	-0.64170399	0.00965474
MVB12A	-0.64162706	0.03257594
UFM1	-0.64134416	1.3669E-06
HNRNPA3	-0.64114372	8.3436E-08
U2AF1	-0.64114072	1.2749E-08
PSMD2	-0.64042636	3.1158E-05
YRDC	-0.63996716	0.01887288
ARF5	-0.63951386	9.5235E-06
RNMT	-0.63946874	0.00013208
SF3B5	-0.63933787	2.2389E-06
FUOM	-0.63813875	0.00477245
ACTR2	-0.63742476	1.3001E-12
CTSZ	-0.63669905	1.9613E-12
PARP10	-0.63661554	0.01432532
TMEM208	-0.636596	0.00274679
SLC35E1	-0.6356857	0.01345148
FDX1	-0.63524794	0.00025541
SEC24A	-0.6350802	0.0276962
MAP3K8	-0.6346224	0.00763545
TPGS2	-0.63437648	0.01012779
SNRPD3	-0.63432577	3.8141E-05
COPG1	-0.63390154	0.00449105
HINT2	-0.63386309	0.00189893
UBE2D3	-0.63302018	2.7866E-13
PPP4R3A	-0.63280818	2.2882E-05
LSM4	-0.63156083	0.00230091
YTHDF1	-0.63110852	0.03601335
TAX1BP3	-0.63110528	0.00189265
HNRNPA2B1	-0.63085099	7.8454E-13
TMX1	-0.63040961	0.00034296
PSMD12	-0.6302188	0.00048382
SRP72	-0.62934483	2.2903E-06
HPRT1	-0.62926843	0.00025525
PSMC3	-0.62793929	0.00071978
MRPS23	-0.62780068	0.03353425
MYADM	-0.62773957	6.9188E-05

CACUL1	-0.62764206	0.00239075
SURF4	-0.62740758	0.00068505
TRAPPC1	-0.62728022	1.3275E-05
HNRNPK	-0.62695662	1.5933E-07
VPS4A	-0.62654865	0.00018738
PKN1	-0.62618449	0.02912682
CYC1	-0.62613838	0.00024123
CHID1	-0.62578694	0.04209322
SRSF8	-0.62543816	0.04586395
TECR	-0.6252145	0.00672719
MIR4435-2HG	-0.62453447	0.00101766
GDI2	-0.62378511	8.8126E-06
ATP5MG	-0.62344714	1.4287E-07
PGLS	-0.6233826	2.2882E-05
DNAJB9	-0.62334971	0.00023841
SUPT5H	-0.62301539	2.3561E-05
COPA	-0.62288616	4.9842E-06
HCFC1	-0.62178086	0.02463937
HADHB	-0.62164036	0.00144581
DNAJC19	-0.62145146	0.02148488
LDLRAD4	-0.62115959	0.04956551
RPS9	-0.62100792	5.6816E-09
ATP13A1	-0.62082967	0.02380544
NDUFV2	-0.62002054	4.8862E-05
ZNF511	-0.61943153	0.00161464
EIF4G1	-0.61918995	0.00038216
ATG16L2	-0.61769252	0.00604877
MTHFD2	-0.61760842	0.00021579
SPIN1	-0.61751475	0.04133805
RHOG	-0.61666773	2.6669E-07
PPIP5K2	-0.61498731	0.00269984
NACA	-0.61485468	9.1356E-08
MEPCE	-0.61343669	0.03763179
PDIA3	-0.61269485	9.8283E-11
AIMP1	-0.61237676	0.00105615
POLR2F	-0.61233665	0.00366224
SETX	-0.61218975	0.00551413
EPB41L3	-0.61170727	1.8672E-06
TMED5	-0.61075832	9.9863E-07
TARS1	-0.60969326	0.00419166
NDUFB5	-0.60915017	0.00010772
UFC1	-0.60913118	8.9246E-05
MAPKAPK2	-0.60907085	0.00088693
STOML2	-0.60854017	0.01757652
RPRD1A	-0.60842114	0.0390283
PDCD4	-0.60834426	0.00050888

MSL3	-0.60803135	0.00819981
ATP5F1E	-0.60782959	1.1826E-20
BCAP31	-0.60678403	0.00037924
SCO2	-0.60639983	2.5472E-05
BEX4	-0.60615719	0.00102344
OS9	-0.60600135	1.0572E-06
CYB5R4	-0.60595114	0.00096666
PSMC4	-0.6058556	0.00056856
AKAP9	-0.60418801	0.01860239
EIF1AX	-0.60387341	0.01309472
PRPS2	-0.6038197	0.04126775
FRG1	-0.60204249	0.00148745
RING1	-0.60124985	0.03869032
SEC22B	-0.6011146	0.01776362
UQCRB	-0.60060484	0.00025997
UBL3	-0.60004775	5.9742E-05
CHMP6	-0.59971081	0.04405314
MMADHC	-0.59790771	0.00012011
ATP6V1D	-0.59745392	0.00737391
UPP1	-0.59739146	2.9993E-05
ARCN1	-0.59718459	9.0806E-05
SDHD	-0.59658069	5.2874E-06
NIFK	-0.59411521	0.00703355
TRPS1	-0.59378341	0.01968465
LYAR	-0.5935484	0.03576696
PI4KA	-0.59289837	0.00225167
CISD2	-0.59268878	0.00199677
TIMM17A	-0.59199767	6.0645E-05
SERINC3	-0.59162967	0.00086165
RUNX3	-0.59126382	0.01287657
JUND	-0.59124285	1.0812E-09
SNX17	-0.59120379	0.00017944
SEC63	-0.59099872	0.0390838
ARRB1	-0.59054569	0.0250831
HMGA1	-0.59044161	0.01653291
AUP1	-0.59017739	2.5842E-05
PSMD11	-0.58984639	0.00291586
EIF2S3	-0.58980899	1.3828E-07
SH3BGRL	-0.58957881	1.2214E-08
SERTAD3	-0.58934327	0.01210321
NCL	-0.58878596	0.00015692
SDCBP	-0.58792568	3.6386E-05
NDUFB8	-0.58738261	1.9311E-07
DDX56	-0.58714347	0.02072012
RBM22	-0.58713998	0.04300174
ZBTB11	-0.58697669	0.01477108

CHCHD5	-0.58642589	0.02744727
NDUFB6	-0.58564145	0.00022066
SHC1	-0.58562029	0.03392342
ERGIC3	-0.58545178	0.00018848
CYB5R3	-0.58506893	1.6322E-07
PRKAR1A	-0.58500373	6.7875E-05
PRR13	-0.58497875	9.4375E-09
CIAPIN1	-0.58495661	0.03362227
RNF40	-0.5848848	0.02916592
PSMB2	-0.58474852	7.1063E-07
SLC38A10	-0.58453098	0.04289467
CCDC124	-0.58437945	0.00030563
UGP2	-0.58350712	0.00119142
DUSP3	-0.58278791	0.03271939
STXBP2	-0.58212655	1.4947E-05
ARRDC2	-0.582088	0.01288724
CAPZB	-0.58187983	1.536E-10
ZNF655	-0.58182301	0.00059035
CCNL1	-0.58127834	8.7388E-05
HIP1	-0.58094918	0.00142373
RPL32	-0.58042145	4.5331E-13
SKIL	-0.58014715	3.774E-05
SPCS2	-0.58011629	0.00012472
CKS2	-0.57974992	0.00060298
NOSIP	-0.57963628	0.00142514
ADI1	-0.57918473	0.00521315
NIPA2	-0.57854846	0.0071167
CTNNBL1	-0.57846729	0.001037
UFD1	-0.57837582	0.00042242
ASPSCR1	-0.57780936	0.04338535
ATP6V0D1	-0.57780101	3.8093E-06
GATAD2A	-0.57775861	0.04448512
HMGXB4	-0.57705973	0.03887888
MRPL27	-0.57669643	0.00559636
RAB1A	-0.57647613	1.8271E-05
DDX39A	-0.57617575	0.0002667
ERVK3-1	-0.57587227	0.01044157
DAZAP2	-0.57551498	3.1785E-10
NDUFA1	-0.57510547	7.2259E-06
VPS29	-0.57381593	5.4526E-06
CCT6A	-0.5721145	3.3263E-05
DDX3Y	-0.57206044	0.00672055
NFATC1	-0.5710069	0.02165547
GSS	-0.5707091	0.0303692
RPL10A	-0.57067985	2.1417E-21
BLTP2	-0.57065595	0.00173643

USF2	-0.57059237	0.00940603
HPS1	-0.57043997	1.3372E-05
CDV3	-0.57028304	2.7226E-05
TPT1	-0.56957387	7.429E-07
ZHX1	-0.56903777	0.04966567
ZNF428	-0.568072	0.01496418
ARPC2	-0.56798981	3.197E-12
COPS9	-0.56712425	0.00011625
RPS6KA1	-0.56684672	0.0454125
CDK5RAP3	-0.56647544	0.00126007
BCL10	-0.56569382	0.01511846
MFSD11	-0.5653877	0.0207198
PRPF4	-0.56536302	0.01633909
GSDMD	-0.56474919	0.0035981
STRAP	-0.56443067	0.00056986
RPL29	-0.56402	1.5237E-11
SIVA1	-0.56398774	0.00059121
MAD2L2	-0.56294495	0.00104838
SYS1	-0.5628249	0.00484358
SNRPD2	-0.56225193	3.4416E-05
TUFM	-0.56222828	8.0993E-06
RPSA	-0.56186945	8.5092E-08
CSTB	-0.56171373	0.01484685
GTF2F1	-0.5616361	0.00130377
CD59	-0.56150508	0.00912377
ARHGAP30	-0.56092948	0.00251796
NDUFB7	-0.56076321	7.5625E-05
NARS1	-0.56072038	0.02511943
ELOB	-0.55965199	1.7422E-06
C4orf3	-0.55963593	3.5359E-06
DNAJA2	-0.55925688	0.00427796
RIT1	-0.55916506	0.02134782
H4C3	-0.55862831	0.0089786
ENSG0000029	-0.55753859	0.02690716
ARHGAP21	-0.5574459	0.00531319
RABAC1	-0.5571541	0.00448076
SNRPA	-0.55532491	0.00189893
SEM1	-0.55504541	1.1732E-08
ATP5ME	-0.55465872	0.00057686
BABAM1	-0.55331752	0.00201704
KLF4	-0.55323397	0.00796985
RAP2C	-0.55304342	0.00650588
MAP3K2	-0.55260325	6.4649E-07
DOK2	-0.55214857	0.02494385
ATP6V1C1	-0.55175975	0.00623667
NAA20	-0.55043043	0.01158141

IRF2BP2	-0.55031727	7.0882E-05
RPL19	-0.54950932	3.0667E-13
RPS16	-0.54943023	3.0933E-09
EIF2A	-0.54937902	0.00032804
NUDCD2	-0.54921576	0.03328551
SNRPA1	-0.54848701	0.00855831
OXA1L	-0.54821262	0.01138321
SRSF2	-0.54768947	2.0325E-09
EIF3M	-0.54638748	0.00039456
ARHGDIA	-0.54636364	5.0245E-05
CFL1	-0.54628315	8.6837E-05
FAM32A	-0.54472707	0.00226313
ZPR1	-0.54467804	0.04869122
TIMM10	-0.54461894	0.01158141
DIMT1	-0.54437196	0.03835987
SMIM19	-0.54396867	0.02142672
ACYP2	-0.54357987	0.01437551
COX6B1	-0.54246688	1.5872E-06
HADHA	-0.54150673	0.00018357
TAP1	-0.54141206	0.00046614
RPL27A	-0.54094912	3.4984E-10
PHB2	-0.54037936	0.00394645
MGAT1	-0.54025088	6.4114E-05
PACSIN2	-0.53961059	0.00449501
COMT	-0.5390273	0.00216673
NDUFS3	-0.53893551	0.00662931
SCYL1	-0.53873506	0.01140157
GORASP2	-0.53638433	0.01835494
ADSS2	-0.53542582	0.00420765
IFT25	-0.53523509	0.00229183
NAA38	-0.53514288	0.00506913
NOP14	-0.5344894	0.04823211
TMEM33	-0.53412494	0.01436869
PHF20L1	-0.53372317	0.01997558
CDK16	-0.53296299	0.01639827
PRPF38A	-0.53279826	0.03443828
SRSF6	-0.53267869	0.00083199
DENND1B	-0.53251617	0.00062471
PTPN12	-0.53234517	0.01591959
ANAPC11	-0.53217093	0.00073787
ADPRS	-0.53214027	0.02433441
PDHA1	-0.52994624	0.04301059
NDUFA10	-0.52973897	0.00426784
PLAA	-0.52911532	0.04680687
RPL3	-0.52908242	1.9759E-11
RPL8	-0.52899829	7.4125E-09

MDM4	-0.52875783	0.02562056
TCEA1	-0.52767788	0.00012884
NME2	-0.52732824	2.6015E-06
ERO1A	-0.52645839	0.00025682
VHL	-0.52632823	0.00729735
SDF4	-0.52583148	3.081E-06
FBL	-0.52544723	2.8178E-05
C18orf32	-0.52456672	0.00049377
CXorf38	-0.5242522	0.01795027
TBC1D5	-0.52319487	0.0220276
AFMID	-0.52258861	0.02842031
TUSC2	-0.52224912	0.0289535
CCDC59	-0.52180013	0.04015134
XRCC6	-0.52123703	0.00012279
LAGE3	-0.52059883	0.02272206
COMMD7	-0.52018299	0.00048076
MZT2B	-0.51941945	0.00018298
THOC7	-0.5192623	0.00997136
RHOA	-0.51899882	4.5575E-05
MRPS12	-0.51848789	0.00795267
NDUFAF3	-0.51830385	0.00054084
SLC50A1	-0.51756982	0.00458722
COX7C	-0.5170436	0.00030182
EMC6	-0.51678467	0.01671592
COPZ1	-0.51655129	0.01287869
SNX1	-0.51636353	0.00102044
MAN1A2	-0.51600712	0.02644653
TMEM219	-0.51584107	0.00026727
GPR108	-0.51487768	0.01705081
FAU	-0.51346545	1.4124E-14
PSMD14	-0.51269521	0.01421768
GOLIM4	-0.51250724	0.01018043
ABCF1	-0.51244578	0.00116468
RBM3	-0.51223075	3.1341E-07
OTUB1	-0.5110117	0.00237972
SET	-0.51067988	0.00055412
SRSF3	-0.51025152	8.8177E-05
COTL1	-0.51022651	0.01144806
MRPS28	-0.50989622	0.02869389
BTF3	-0.50955832	2.3829E-07
TM9SF2	-0.50929296	1.6803E-05
VAMP3	-0.50879707	0.00286009
ETFA	-0.50710194	0.00168103
SLC38A1	-0.50699581	0.04013829
ERAP2	-0.5067579	0.01951902
GNL2	-0.50631425	0.01343248

ACER3	-0.50623231	0.00158214
YTHDF2	-0.50556439	0.01368994
ITPR1	-0.50554019	0.00185359
NUDT3	-0.50520349	0.02146143
RPL4	-0.5046058	5.3114E-09
DICER1	-0.50453752	0.01951902
UBE2N	-0.50363478	0.01769907
KDSR	-0.50343446	0.02706466
RBCK1	-0.502949	0.00031828
DNM2	-0.50241716	0.00229793
PTP4A2	-0.50237934	3.3888E-06
RPS3	-0.50236914	2.2657E-09
ATG3	-0.50232093	0.00053888
PSMC6	-0.50217788	0.00330634
DRG1	-0.50208937	0.02559353
COX6A1	-0.50206688	1.3763E-06
RPS2	-0.5018893	4.2737E-06
RPL18A	-0.50167341	2.0029E-11
HCFC1R1	-0.50146768	0.03296921
HNRNPD	-0.50061015	7.0401E-05
LACTB	-0.50018216	0.0001531
AP2M1	-0.49948833	0.00017281
ACBD3	-0.49902443	0.01860531
KARS1	-0.49878682	0.01047775
HSPA13	-0.49878218	0.03779051
OSBPL8	-0.49820862	0.01790011
TWF1	-0.49668572	0.03353041
RAB14	-0.49637086	0.00279449
TRPV2	-0.49594635	0.01145533
SSR2	-0.4948841	0.00158898
NDUFB3	-0.494791	0.00110218
EIF3B	-0.49477528	0.00056421
ADAM10	-0.49328229	0.00134433
PURB	-0.49311901	0.02184996
TEX264	-0.49287297	0.04837575
DGUOK	-0.49264397	0.00136157
HSP90AB1	-0.4920437	0.01253864
BRI3	-0.49012809	6.1148E-05
RER1	-0.48993136	0.00201112
RAB22A	-0.48983735	0.00349564
ISY1	-0.48976452	0.01595445
ZBED1	-0.489572	0.03070924
DIAPH2	-0.48887898	0.02370951
MARCHF2	-0.48852885	0.01173037
WBP2	-0.48820458	0.00311591
TOP1	-0.48771261	0.02889312

TM9SF3	-0.48615167	0.00018654
ANXA11	-0.48596263	0.00018828
ETNK1	-0.48464707	0.03718947
CCT4	-0.48458966	0.00323097
ERH	-0.48284284	7.4308E-05
SCAND1	-0.48281389	2.1348E-05
SRRM1	-0.48208077	9.5753E-05
RPLP2	-0.4819974	8.3568E-08
RPLP0	-0.48145096	8.0174E-14
ILF2	-0.48109459	0.00085371
DDT	-0.48042187	0.00015897
MRPL13	-0.48025453	0.02954863
TMEM256	-0.48009559	0.00536628
CCDC47	-0.47999268	0.00146698
MESD	-0.47922807	0.04383322
CHD9	-0.47913748	0.015048
NDUFA3	-0.47810204	0.00079466
TPM3	-0.47791982	4.0096E-07
EIF3J	-0.47738821	0.0042944
COPS7A	-0.47715881	0.04195597
CD81	-0.47667718	0.00010384
NUMB	-0.47656203	0.00057479
EFR3A	-0.47650308	0.00088336
SLC25A3	-0.47621646	2.5322E-05
ME2	-0.47599398	0.01861732
TMEM165	-0.47565625	0.00030642
EEF1G	-0.47555315	2.3746E-06
DRAM2	-0.47488023	0.01019232
RPL6	-0.47362669	5.4128E-07
OSBP	-0.47261995	0.0491703
MAP7D1	-0.47248886	0.00553241
TMED3	-0.47231915	0.042842
ODF3B	-0.47213163	0.00156977
CORO1B	-0.47200592	0.001522
TAF9	-0.47183997	0.02014709
MFF	-0.47171134	0.01502892
RPL23	-0.47137096	7.2308E-06
GYPC	-0.47130755	0.00086086
ARL6IP4	-0.47110428	0.00648884
RPS6	-0.47073951	9.2982E-10
HSP90B1	-0.47022358	0.00211034
PDE4B	-0.46995271	3.1158E-05
SLC25A33	-0.46919668	0.00542081
RPS24	-0.4691546	8.3502E-13
CYRIB	-0.46911001	9.8053E-06
SNRPC	-0.46862529	0.00311591

MTCH2	-0.4683409	0.00129608
CRYBG3	-0.4682686	0.02127337
ZCRB1	-0.46791805	0.03167857
SERBP1	-0.46762948	0.00028008
GMIP	-0.46748387	0.02552761
UHMK1	-0.46551356	0.03576696
FPGS	-0.46513337	0.01661332
ATP6V1E1	-0.46506508	0.00779329
DTX3L	-0.46409266	0.03026722
C11orf58	-0.46367324	2.3265E-05
RPS14	-0.46341648	2.357E-13
CHCHD3	-0.4629892	0.01422442
BLTP3B	-0.46197514	0.0478981
ORMDL1	-0.46174914	0.00328591
KIAA0232	-0.46173602	0.03685441
RANBP2	-0.46170554	0.02286473
RPL38	-0.46093889	0.00018898
EIF3L	-0.46079245	0.000247
TMEM167A	-0.4604172	0.00529639
MCL1	-0.45961425	7.5625E-05
RPL22	-0.45757442	3.2263E-08
RPS4X	-0.45725907	0.0002283
UBE2V1	-0.45593239	0.00168639
ATP2A2	-0.45564458	0.00341045
HCK	-0.45356545	0.00162115
RPL34	-0.45337555	1.318E-07
PTDSS1	-0.45333742	0.00808772
RPS5	-0.45145025	1.1288E-08
FIS1	-0.4513068	0.0071167
RPS3A	-0.45125191	5.7019E-07
MAF1	-0.45105377	0.00304697
RABGEF1	-0.45060475	0.00788126
ABHD12	-0.45038193	0.03216575
GALNT1	-0.45013922	0.01554121
EMD	-0.44992902	0.00063953
USP37	-0.44987602	0.03993444
SURF1	-0.44986561	0.02123686
PSMF1	-0.4486174	0.00031753
CSRNP1	-0.44724452	0.03800248
RUNX1	-0.44719728	0.00118814
CARS2	-0.44717533	0.0372072
USP32	-0.44687325	0.00865342
FKBP3	-0.44603153	0.02835557
UBA52	-0.44425858	9.806E-15
TAF15	-0.44382893	0.00100037
CHSY1	-0.4437991	0.04527773

MORF4L2	-0.44376052	0.00953087
VPS26A	-0.44359394	0.00788125
ZNF24	-0.44150852	0.04844951
COPB2	-0.44120811	0.00311591
PDAP1	-0.44112172	0.00264211
RPS8	-0.44092401	9.512E-11
RNASEK	-0.43988212	2.9075E-05
PPP6C	-0.43919617	0.02173836
STIP1	-0.43903439	0.03022635
YBX3	-0.43897161	0.02445236
MARK2	-0.43896836	0.02865012
MAP4K3	-0.43868341	0.01955767
PPP2R1A	-0.43857932	0.00209555
CPQ	-0.43762182	0.02113976
ESD	-0.437231	0.00475025
RPL13	-0.4370808	1.4419E-09
CANX	-0.43692487	0.00059952
SRRT	-0.43659799	0.04145842
MRPL18	-0.43655271	0.0020079
ORAI2	-0.43620387	0.02454787
ADPGK	-0.43576183	0.00072771
MIR142HG	-0.43451846	0.03694428
NFE2L2	-0.43423818	0.00302351
SMARCB1	-0.43377046	0.01318505
GNG2	-0.43369105	0.02062735
LYPLA2	-0.433628	0.02861724
ROMO1	-0.43339446	0.00012867
RSL24D1	-0.43309603	0.01838045
ERP44	-0.43156144	0.00429139
CWC15	-0.43104402	0.03694327
TOMM5	-0.4310187	0.042879
NONO	-0.43075851	0.01362713
SLC3A2	-0.43006545	0.00292185
CHMP3	-0.42925134	0.02629185
RPS26	-0.42865237	0.00073785
U2AF1L4	-0.42823172	0.03356556
NDUFB4	-0.42809975	9.0392E-05
UBE2D2	-0.4265864	0.00180638
PPP1CC	-0.42610871	0.02231663
PRRC2A	-0.42454265	0.03941599
ATP1A1	-0.42397342	0.00011236
MYL12B	-0.42090948	8.8952E-05
SRGN	-0.42074272	0.01087064
C1orf54	-0.42006592	0.04630154
UTP18	-0.42001825	0.02528418
TACC1	-0.41996333	0.00366729

ENO1	-0.41886264	0.00182901
CLTA	-0.41878079	0.00058874
NDUFA11	-0.41722552	0.00018113
XRN1	-0.41641446	0.02616245
RPS29	-0.41606381	6.8452E-07
NUTF2	-0.41568208	0.03528119
MPG	-0.41545722	0.04199736
FKBP8	-0.41497783	0.00142164
ACAP2	-0.414489	0.00964335
DCTN4	-0.41337679	0.03393598
TRIM38	-0.4114305	0.00425504
RPL11	-0.41115243	2.7471E-07
IGFLR1	-0.41019574	0.03873051
RPS23	-0.4085772	0.00001484
HNRNPH2	-0.40810696	0.01627722
SNHG8	-0.40807476	0.0029745
ATP5MK	-0.40774486	0.00954646
NRBP1	-0.40676702	0.00461359
RPS10	-0.40563798	1.1776E-06
PPM1G	-0.40561861	0.00600081
ATXN10	-0.40530085	0.03847033
NOL7	-0.40473021	0.00145096
CCT3	-0.40468269	0.01695882
YIPF3	-0.40414085	0.01692861
HSPD1	-0.40382581	0.02606275
MEA1	-0.40115097	0.0267417
RPL31	-0.40089746	1.9782E-05
API5	-0.39884411	0.02196482
DBNL	-0.39877604	0.00213674
SNX5	-0.39854595	0.0089744
COQ10B	-0.39652924	0.02641479
RPL30	-0.39647222	6.6767E-08
DNM1L	-0.39516748	0.03524347
AP3S1	-0.39485768	0.03242144
SRP14	-0.39438106	0.00495938
MAP2K2	-0.39409326	0.0017367
TOMM7	-0.39391875	0.00047468
RPL7	-0.39290436	1.3308E-05
TAF10	-0.39231765	0.00041908
ZFP91	-0.39023557	0.01002653
TIMM23	-0.39007629	0.03590319
DUT	-0.38935934	0.02116838
C1orf43	-0.38673043	0.001312
KLHL5	-0.38637546	0.01754682
FLII	-0.38616352	0.00218913
TGOLN2	-0.38598636	0.00765323

PHPT1	-0.38461043	0.02367487
ATP5MC2	-0.38444083	0.0083036
EFHD2	-0.38433826	0.01412078
RPL26	-0.38414304	4.6997E-06
CCT8	-0.38370351	0.01291132
FNIP1	-0.38071952	0.02622399
ITM2B	-0.37910368	0.00212051
RTCB	-0.37877349	0.02262889
CLDND1	-0.37791899	0.01511671
NUDC	-0.37489739	0.02869389
RPL15	-0.37484385	0.00034141
CNOT2	-0.37483013	0.01705081
NDUFV1	-0.37481399	0.02704967
RBM25	-0.3747666	0.01296867
PTBP1	-0.37476424	0.00856267
ILF3	-0.37408663	0.02425178
RPS15	-0.37232873	6.6796E-07
ITGB1	-0.37191006	0.00331099
RNH1	-0.37176319	0.00056019
RPS15A	-0.37173667	0.00101051
RBBP4	-0.37137016	0.0071167
RPL24	-0.37117677	4.5583E-05
PABPN1	-0.37087794	0.00085512
CXCL16	-0.37053245	0.00199633
ACTR3	-0.37030026	0.00154745
TMSB4X	-0.36967789	0.01426412
SQOR	-0.36944666	0.03492108
VPS28	-0.36932336	0.00359021
CTDNEP1	-0.36756969	0.00256258
SFPQ	-0.36750058	0.00174037
RAB7A	-0.36734757	0.01982546
TUBA1B	-0.3670204	0.01402949
TMEM258	-0.36686562	0.00058582
EIF3A	-0.36673688	0.00264971
TMSB10	-0.36540542	3.8961E-05
RNPS1	-0.36367145	0.02397852
RPL18	-0.36358702	5.7331E-05
RWDD1	-0.36245905	0.01658724
PCBP1	-0.36086128	0.00087287
RPS12	-0.36031113	1.7715E-08
TMBIM6	-0.35909095	0.00395309
UXT	-0.35865725	0.00206036
SLC25A24	-0.35865373	0.03510601
ARIH2	-0.35706656	0.02409367
SLC25A6	-0.35655311	0.00387355
COX4I1	-0.35628096	0.00113883

NIPSNAP3A	-0.35602206	0.03251761
NR1H2	-0.35453876	0.00764503
KDELR1	-0.35430496	0.00529442
DTNBP1	-0.35125135	0.03000795
RPL37	-0.35117128	2.3899E-10
PER1	-0.35114775	0.02042971
TXNRD1	-0.35063688	0.02413671
CIAO2A	-0.35030843	0.00769801
RPL9	-0.34934677	6.6577E-06
SRSF5	-0.34929833	0.00119975
RPS21	-0.34735219	1.7424E-06
TRMT112	-0.34641662	0.00346506
CNN2	-0.34505049	0.0281195
CSNK1A1	-0.34426049	0.00140163
WAPL	-0.34361276	0.02633174
UBL5	-0.34301863	0.00654827
BAX	-0.34185563	0.00549665
DYNLT1	-0.34114335	0.00045418
DYNLRB1	-0.34112831	0.01094749
RBMX	-0.33906406	0.02120121
WDR45B	-0.33352512	0.04009897
SUMO1	-0.33308512	0.02238039
SHKBP1	-0.3330773	0.01964339
PSME1	-0.33171872	0.00055365
CS	-0.3277627	0.03847033
PEBP1	-0.32554957	0.03216706
GNAS	-0.32518339	1.3639E-05
HSPA8	-0.32421062	0.02396818
RPL5	-0.32399059	0.00039911
C5orf15	-0.32389146	0.03043673
RPL28	-0.32311765	0.00064038
RAD23B	-0.32309983	0.00376646
EWSR1	-0.32258739	0.00065595
RPS7	-0.32236361	0.00028912
GNA13	-0.32175895	0.0047197
SOCS3	-0.3191707	0.03703149
DDAH2	-0.3182491	0.04009972
PTGES3	-0.31723945	0.04007396
HNRNPA0	-0.31691396	0.03098851
MRPL41	-0.31671592	0.01816776
RBBP6	-0.3155203	0.02842142
VMP1	-0.31393114	0.03685175
EIF3G	-0.31287732	0.0241414
HLA-DOA	-0.31111338	0.01825138
SNHG29	-0.31093356	0.00055854
SRP54	-0.31016824	0.04013829

ICAM1	-0.30850382	0.0195223
FAM50A	-0.30677034	0.01465716
PKM	-0.30647079	0.03226219
PAPOLA	-0.3059451	0.01652533
RPS19	-0.30111547	0.00012798
PPIG	-0.29905874	0.03604796
SNHG5	-0.29899777	0.0157105
RPLP1	-0.29856669	0.01644384
MAP1LC3B	-0.29344151	0.00225423
RPL21	-0.29341892	0.00015287
C19orf53	-0.29089213	0.04785672
EEF1D	-0.28991739	0.01388931
BNIP3L	-0.28830275	0.04772698
KTN1	-0.28736642	0.04019788
EIF3H	-0.28601289	0.03575302
HNRNPL	-0.2832245	0.02690716
RPS27	-0.2821151	0.00350816
HNRNPM	-0.28200455	0.02373837
MOB1A	-0.28058192	0.01314166
RPL17	-0.27944715	0.00710449
SNRNP70	-0.27768361	0.03605017
UBXN4	-0.27434312	0.01644384
HMGN1	-0.27144518	0.03094196
EIF4E2	-0.26927579	0.03231749
RPS17	-0.26553323	0.00230839
ANKHD1	-0.25991704	0.04397397
VDAC1	-0.25873891	0.04096681
RPL7A	-0.24591994	0.03764901
NRDC	-0.24506503	0.03663751
RBM39	-0.2438994	0.0301333
ROCK1	-0.24386253	0.03080254
RPL12	-0.24122151	0.00203393
RTRAF	-0.24118306	0.02790909
DYNLL1	-0.24074253	0.04903206
YWHAE	-0.22919216	0.03254895
SEPTIN7	-0.22789401	0.039675
PPIA	-0.22125264	0.01568223
LYN	-0.21725164	0.04069119
RPL13A	-0.17625517	0.02383631

Table S11

cDC1					
**Inflamed DEGs**	**avg_logFC**	**adj. p-value**	**Uninflamed DE**	**avg_logFC**	**adj. p-value**
CCR7	2.98597341	0.04013044			
HAPLN3	2.05785531	0.04013044			
SELL	1.6008601	0.04013044			
PLEK	0.51646507	0.04013044			

cDC2					
**Inflamed DEGs**	**avg_logFC**	**adj. p-value**	**Uninflamed DE**	**avg_logFC**	**adj. p-value**
S100B	1.83622103	0.00064911	CD207	-1.0439263	0.00064911
MIR155HG	1.63460674	0.02829664			
IFITM1	1.39391475	0.00064911			
LTB	1.33739459	0.04353737			
TRAF1	1.1609381	0.04779759			
IFITM3	1.12100594	0.0021254			
C1QB	1.06877836	0.00854795			
CALCRL	1.01614417	0.04779759			
IDO1	0.990589	0.04779759			
SPIB	0.94939162	0.02028977			
IER3	0.90653053	0.01564235			
IFITM2	0.8719038	0.00499382			
TAP1	0.84570379	0.04353737			
NFKBIA	0.77668851	0.00064911			
SNHG15	0.75435348	0.00064911			
PPP1R14A	0.70575367	0.04655242			
MT-ATP8	0.67500501	0.00201484			
DUSP2	0.6627113	0.04779759			
IRF1	0.58376022	0.00854795			
S100A6	0.5549559	0.04779759			
LGALS2	0.50936012	0.00064911			

cDC3					
**Inflamed DEGs**	**avg_logFC**	**adj. p-value**	**Uninflamed DE**	**avg_logFC**	**adj. p-value**
FCGR3A	2.03694683	0.03969029	IGF1	-2.96581534	2.6919E-05
FPR2	1.69738298	0.02320015	SDS	-1.33268254	0.0038451
IL7R	1.61249581	0.0118191			
IFITM1	1.38076145	0.00161938			
IFITM3	1.17282336	0.00992614			
GLIPR2	0.88319705	0.00470179			
HMOX1	0.84851145	0.0091307			
GBP1	0.72483964	0.0253421			
TYMP	0.71756905	0.02681232			
MT-ATP8	0.65677716	0.0091307			
UPP1	0.53339305	0.02681232			

Ambiguous

Inflamed DEGs	avg_logFC	adj. p-value	Uninflamed DE	avg_logFC	adj. p-value
IFITM1	1.38058901	0.00016289	NET1	-1.2626516	0.00636968
IFITM3	0.94499436	4.8612E-07			

CCR7+ DC

Inflamed DEGs	avg_logFC	adj. p-value	Uninflamed DE	avg_logFC	adj. p-value
SOX5	4.39086751	0.0001772	CD209	-3.09236744	0.01850823
EBF1	4.17066535	0.0001772	ENSG0000017	-3.02478129	0.04355548
ALOX15B	3.97666984	0.00232101	DNASE1L3	-2.48328156	0.00194535
MSC-AS1	3.96396061	0.01367127	HOXA5	-2.47867119	0.01821418
ZFPM2	3.89962423	0.00355682	IL22RA2	-2.29470408	0.01367127
MSC	3.24000497	0.02497269	ASB2	-2.10900832	0.01850823
C1QTNF1	3.15938101	0.03412149	GUCY1A1	-2.04066664	0.03440022
KCNE5	3.14062711	0.02117014	APBA1	-1.89809991	0.04585041
CD70	3.11842069	0.00232101	CD1E	-1.88977219	0.01821418
ENPP4	3.06235636	0.00040571	RGCC	-1.44968079	0.0108428
MAPK13	2.8735925	0.04011435	GPRIN3	-1.44145314	0.04783785
MTSS1	2.84783285	0.00232101	ADCY3	-1.23411133	0.0289572
CASC15	2.77164611	0.03329617	GCLC	-1.22353367	0.04328596
ITGB8	2.7268461	0.01821418	GSAP	-1.09721429	0.03056985
TMEM150C	2.4877068	0.03412149	DDX54	-1.04930634	0.0289572
LDAF1	2.4537714	0.0282071	SDF2L1	-1.02688278	0.00232101
LINC03025	2.4223474	0.01367127	PDIA4	-1.00321119	0.01367127
CRLF2	2.28728945	0.04355548	P2RY14	-0.95294516	0.03304758
CLCF1	2.27455361	0.01850059	LILRA5	-0.93541309	0.02095551
ECE1	2.03880474	0.03412149	IDI1	-0.84979184	0.04951115
PRAG1	2.03790689	0.04355548	PSTPIP2	-0.81960195	0.04011435
RAB13	2.02705565	0.02117014	DNAJB11	-0.70716183	0.048906
ENSG00000256948	1.94563698	0.04355548	IFNGR1	-0.65633928	0.01821418
GCNT2	1.88666272	0.03412149	HSPA5	-0.56946291	0.02825678
TNFRSF11A	1.77222089	0.04011435			
L3MBTL4	1.76461537	0.00960638			
S100A6	1.71750081	6.4492E-05			
MYO1G	1.65697274	0.04328596			
SLC1A3	1.61682752	0.0254737			
RAMP1	1.54503944	0.01999832			
STEAP1B	1.54292351	0.02254532			
IL1R2	1.53675456	0.00843575			
GPC5	1.52969704	0.00694972			
GRSF1	1.50258299	0.04165882			
SERPINA1	1.45391546	0.0108428			
MAP4K4	1.43451137	0.01850059			
CAPG	1.4328141	0.01821418			
PACS1	1.37434253	0.048906			
PPP3CA	1.30749564	0.04011435			
SERPINF1	1.2965913	0.04011435			

ZBTB38	1.27795097	0.04355548
SPECC1L	1.24434269	0.02345401
SPECC1	1.24370776	0.04216701
AHNAK	1.11521614	0.01367127
SOCS1	1.06728024	0.03304758
ELL2	0.99144853	0.03412149
FLT3	0.84340573	0.0254737
PTPN2	0.75915186	0.01531796
MT-ATP8	0.68522781	0.04011435

Table S12

Experiment	Label	Target	Clone
Flow cytometry	BUV737	CD38	HB-7
	BUV393	CD45	HI30
	BV786	CD1c	F10/21A3
	BV711	CD141	1A4
	BV650	CD163	GHI/61
	BV605	CD103	B-Ly7
	BV480	CD1a	HI149
	BV421	CD14	MϕP9
	BB700	CD123	7G3
	BB515	CYTOX	F10/21A3
	PE/Cy7	CD55	JS11
	PE/Cy5	CD206	15-2
	PE/CF594 or AF647	CD3	UCHT1
	PE/CF594 or BV421	CD19	HIB19
	PE	CD5	UCHT2
	APCFire750	CD11a	TS2/4
	AF700	HLADR	G46-6
	APC	CD207	10E2
	BV650	EpCAM	9C4
	7-AAD	viability	viability
	PE-Cy5	CD235ab	HIR2
CITEseq	Totalseq-A	CD1c	L161
	Totalseq-A	CD14	M5E2
	Totalseq-A	CD11a	TS2/4
	Totalseq-A	CD55	JS11
	Totalseq-A	CD5	UCHT2
	Totalseq-A	CD206	15-2
	Totalseq-A	CD209	9E9A8
	Totalseq-A	CD11c	S-HCL-3
Immunohistochemistry	*unlabelled*	LAMP3	EPR24265-8
	BV421	anti-rabbit IgG	*polyclonal*
	FITC	Ki67	REA183
	AF555	CD3	SP162
	AF647	CCR7	EPR23192-57
